# Characterization of porcine simple sequence repeat variation on a population scale with genome resequencing data

**DOI:** 10.1038/s41598-017-02600-8

**Published:** 2017-05-24

**Authors:** Congcong Liu, Yan Liu, Xinyi Zhang, Xuewen Xu, Shuhong Zhao

**Affiliations:** 1 Key Lab of Agricultural Animal Genetics, Breeding, and Reproduction of the Ministry of Education & Key Lab of Swine Genetics and Breeding of the Ministry of Agriculture, College of Animal Science and Technology, Huazhong Agricultural University, Wuhan, 430070 Hubei PR China; 2 The Cooperative Innovation Center for Sustainable Pig Production, Wuhan, 430070 China

## Abstract

Simple sequence repeats (SSRs) are used as polymorphic molecular markers in many species. They contribute very important functional variations in a range of complex traits; however, little is known about the variation of most SSRs in pig populations. Here, using genome resequencing data, we identified ~0.63 million polymorphic SSR loci from more than 100 individuals. Through intensive analysis of this dataset, we found that the SSR motif composition, motif length, total length of alleles and distribution of alleles all contribute to SSR variability. Furthermore, we found that CG-containing SSRs displayed significantly lower polymorphism and higher cross-species conservation. With a rigorous filter procedure, we provided a catalogue of 16,527 high-quality polymorphic SSRs, which displayed reliable results for the analysis of phylogenetic relationships and provided valuable summary statistics for 30 individuals equally selected from eight local Chinese pig breeds, six commercial lean pig breeds and Chinese wild boars. In addition, from the high-quality polymorphic SSR catalogue, we identified four loci with potential loss-of-function alleles. Overall, these analyses provide a valuable catalogue of polymorphic SSRs to the existing pig genetic variation database, and we believe this catalogue could be used for future genome-wide genetic analysis.

## Introduction

Simple sequence repeats (SSRs) are tandem repeats with core motifs of 2 to 6 base pairs (bp), which are widely distributed in both eukaryotic and prokaryotic genomes. Because of their wide distribution, high level of polymorphism and co-dominant characters, SSRs are usually used as molecular markers for genetic mapping, population diversity and evolution studies. However, SSRs do not serve only as molecular markers; they also contribute very important functional variations in both protein coding regions and non-coding regions^1^. SSR variations in coding regions directly produce mutant proteins, of which the most typical cases are human trinucleotide repeat expansions leading to neurological disorders, such as X-linked spinal and bulbar muscular atrophy^2^ and Huntington’s disease^3^. Other functional SSR variations have been identified in 5′-untranslated regions (UTRs), which influence gene expression by modulating transcription or translation^1^. For instance, a GAG trinucleotide-repeat polymorphism identified in the 5′UTR of the human GCLC gene influences its translation and was proven to be associated with lung cancer risk^4^. Furthermore, SSR density and the dominant type of 5′UTR displayed a significant difference in housekeeping and tissue-specific genes, supporting their regulatory function in gene expression^5^. Aside from the 5′UTR, regulatory SSR variations have also been identified in 3′UTRs, promoters and introns. For example, the copy number variation of the “CCG” trinucleotide repeat, located immediately upstream of the transcriptional start site of PLAG1, was identified to be the quantitative trait nucleotide (QTN) influencing bovine stature by serving as nuclear factor binding sites and modulating the expression of PLAG1^6^. Recently, a genome-wide analysis of SSRs and their relationship with the expression level of their adjacent genes identified 2,060 significant expression SSRs (eSSRs), which further highlight the contributions of SSRs to gene expression and complex trait variations^7^.

In the past twenty years, high-throughput identification and characterization of SSRs based on expressed sequence tag (EST) or genome sequencing data have been conducted in various species^8–11^. Benefiting from these advances, cross-species comparisons of SSR distribution revealed the following features: (1) SSRs are non-randomly distributed, and dinucleotide repeats are the most abundant type in the genomes of various species; (2) exons contain more trinucleotide and hexanucleotide SSRs than other kinds of repeats; (3) trinucleotide repeats in exons display different motif preference in different biological kingdoms, e.g., AGC triplets were more abundant in animals, whereas AAG triplets were the dominant motif in some plants^1, 12, 13^. In recent years, next-generation sequencing technologies have developed rapidly, promoting the high-throughput discovery of EST-SSR markers in various plant species, such as adzuki beans^14^ and wheat^15^, based on transcriptome sequencing data. For animals, however, most achievements have been concentrated on single-nucleotide polymorphisms (SNPs)^16^; SSR marker development based on next-generation sequencing has only been reported in a few species, such as Korean water deer^17^, Megalobrama amblycephala^18^ and humans^19^. Until now, to our knowledge, high-throughput and population-scale SSR polymorphism analysis based on next-generation sequencing data has been conducted only in maize^20^ and humans^19^.

The pig was one of the first domesticated animals and has formed numerous diverse breeds around the world due to the long-term artificial and natural selection. The complete sequencing of the female Duroc pig genome is a milestone in pig genetic research^21^. Since then, various genome resequencing or de novo sequencing projects have been conducted in different pig breeds, and related data have been published and made available through GenBank^22–28^. With these data, regions responding to domestication, specific breed characters and introgression were identified in a genome-wide scan based on high-throughput SNP analysis. However, no genome-wide analysis targeting SSRs had been conducted in pigs, except for an SSR scanning report based on porcine ESTs^29^. The aims of the current study were to identify and characterize all possible SSR loci in the pig reference genome and to characterize all polymorphic SSRs based on partial public genome resequencing data. With these polymorphic SSRs, we attempt to analyse some potential functional variations and evaluate their utility in population genetics.

## Results

### Overview of SSRs in the pig reference genome

**Figure 1:**
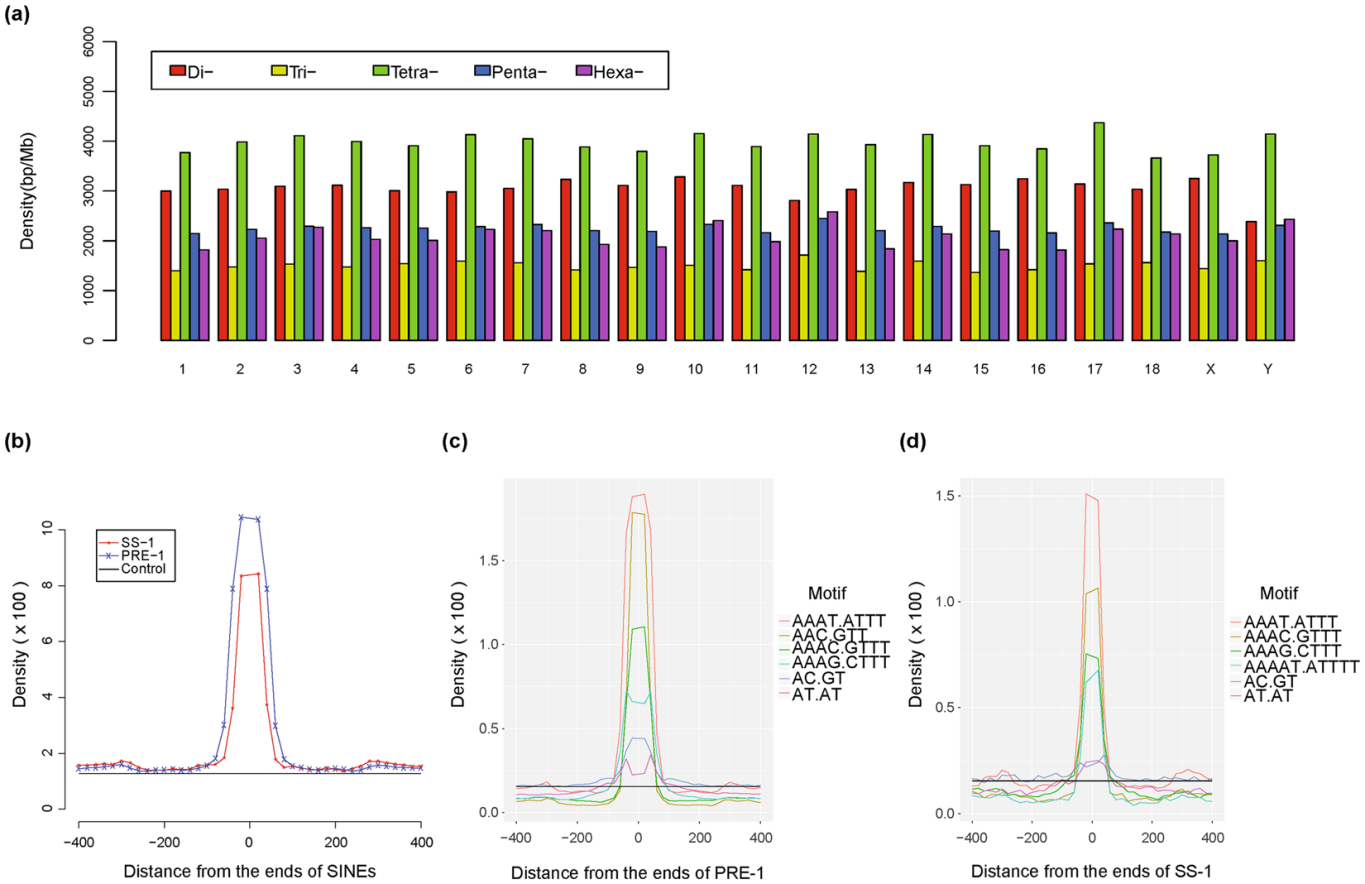
Overall distribution of SSRs in the pig reference genome and their enrichment in the vicinity of SINEs. (**a**) Density of each kind of SSR in different chromosomes. The densities are calculated as the total length of each type of SSR in one specific chromosome divided by the chromosome length. (**b**) Enrichment of all SSRs in the vicinity of two SINEs. “Control” (black line) represents the genome-wide average SSR density (1.28%). (**c**) Top six enriched SSR motifs in the vicinity of PRE-1. (**d**) Top six enriched SSR motifs in the vicinity of SS-1. The black line in (**c**) and (**d**) represents the maximum genome-wide average density of the top six SSR motifs.

Using the Tandem Repeats Finder program, we identified a total of 1,620,469 SSRs, including 395,943 dinucleotide repeats, 209,971 trinucleotide repeats, 507,867 tetranucleotide repeats, 281,380 pentanucleotide repeats and 225,308 hexanucleotide repeats, in 18 well-assembled autosomes, X chromosome and Y chromosome. In total, ~1.28% of the pig reference genome is occupied by SSRs. The average length of different SSRs was not significantly different among autosomes and the X chromosome; however, the dinucleotide repeats density in the Y chromosome seems lower than that in other chromosomes (Fig. 1a, Supplementary Table S1). One common hypothesis is that SSRs tend to be enriched in the vicinity of short interspersed nuclear elements (SINEs); we therefore analysed the SSR density in the vicinity of two identified porcine SINEs: PRE-1 and SS-1. The results revealed that the total SSR density is much higher within 40 bp to the SINE boundary and rapidly diminishes as distance increases (Fig. 1b). The most commonly enriched SSRs for PRE-1 and SS-1 are A/T-rich motifs, including AAAT/ATTT, AAAC/GTTT, AAAG/CTTT and AAAAT/ATTTT (Fig. 1c and d), whereas it seems that AAC/GTT repeats are enriched predominantly in the boundary of PRE-1 (Fig. 1c).

Among dinucleotide repeats, AC/GT (45.91%) repeats are the most abundant motif overall, followed by AT/TA (30.09%) repeats and AG/CT (23.64%) repeats, and CG/GC (0.36%) repeats are the least abundant motif (Supplementary Fig. S1a). For trinucleotide repeats, AAC/GTT (30.46%) repeats are the most abundant type, followed by AAT/ATT (26.91%) repeats and AAG/CTT (11.82%) repeats, and the least abundant motifs are ACG/CGT (0.13%) (Supplementary Fig. S1b). Among tetranucleotide repeats, AAAT/ATTT (27.57%), AAAC/GTTT (17.91%) and AAAG/CTTT (14.68%) are the three main types and occupy more than 60.16% of all identified tetranucleotide repeats, whereas the palindromic motifs, such as ACGT/ACGT, are much lower in abundance (Supplementary Fig. S1c). Similar to tetranucleotide repeats, the top three pentanucleotide repeats are also A/T-rich motifs (Supplementary Fig. S1d); however, for hexanucleotide repeats, the most abundant motif is ACAGCC/GGCTGT (32.33%), followed by A/T-rich motifs (Supplementary Fig. S1e).

For each kind of SSR, the densities in the intergenic region are similar as those in introns, and both are slightly lower than the densities in the defined promoter region (Supplementary Fig. S2a, Supplementary Table S2). As expected, the overall density of SSRs in the coding region is significantly lower than any other region, except for trinucleotide repeats, which have obviously higher densities in the coding sequence (CDS), whereas the most striking fact is that the 5′UTR region has an extremely high density of trinucleotide repeats (Supplementary Fig. S2a, Supplementary Table S2). Furthermore, the most abundant motif of trinucleotide repeats in 5′UTRs is CCG/CGG, which is in agreement with observations in rice and Arabidopsis^30^, and the enriched trinucleotide repeats in the CDS regions include AGC/GCT, CCG/CGG and AGG/CCT (Supplementary Fig. S2b).

### Polymorphic SSR scanning and genotyping quality analysis

To identify polymorphic SSRs (pSSRs), we analysed the next-generation sequencing data of 102 individuals from 13 different domestic pig breeds, Chinese and European wild boars and 2 out-groups (Supplementary Table S3). The sequencing depth of the studied individuals varied from approximately 3.5 to 22.6 times. With a total of 1,620,469 SSRs as the input, we acquired genotype information for 1,343,193 loci in total; 17.11% of SSR loci were missing from the catalogue because they were not suitable for the “allotype” requirement.

**Figure 2:**
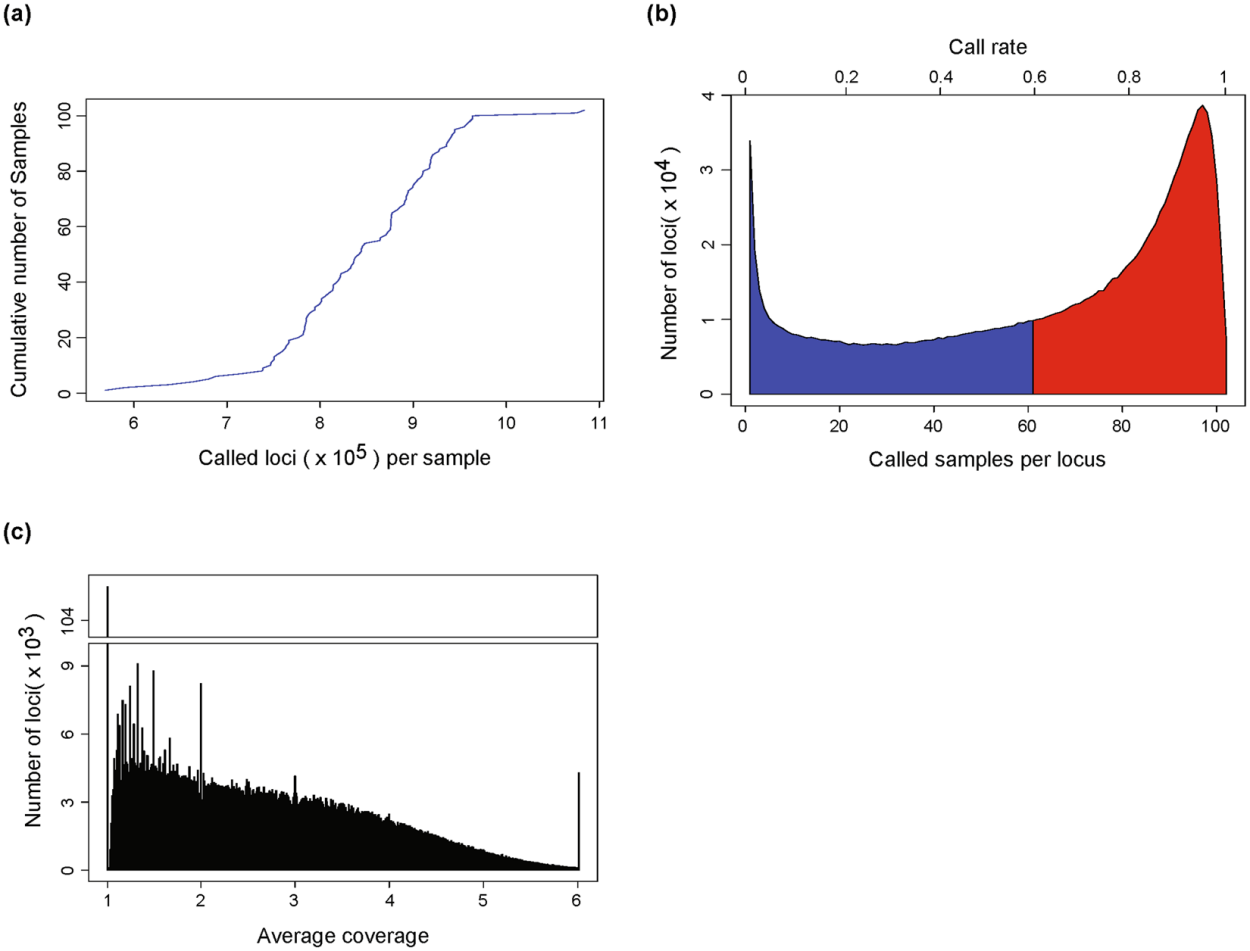
Statistics of genotyping quality. (**a**) Distribution of the number of called loci per sample. The average is 842,707.4 SSRs per sample. (**b**) Distribution of the number of called samples per locus. The average is 63.99 samples per SSR. (**c**) Distribution of average coverage. The average coverage is calculated as the number of reads in a locus divided by the number of called samples.

On average, we obtained genotypes for 842,707.4 SSR loci per individual (Fig. 2a) and 63.99 individuals per locus (Fig. 2b). The density of call rate exhibited a double-peak distribution, indicating that some loci had a very low call rate. In addition, 820,354 SSR loci (61.07%) had a call rate greater than or equal to 0.6 (Fig. 2b). The average read numbers displayed a severely left-skewed distribution and peaked at an average read number of 1.0 (the lowest coverage threshold of the software), and there were 463,793 SSR loci (34.53%) with an average read number greater than or equal to 3.0 (Fig. 2c). We further compared the average read number and call rate per locus across different SSR motifs individually. This comparison revealed that different SSR motifs had dramatically variable average read numbers (Supplementary Fig. S3a,b) and call rates (Supplementary Fig. S3c,d). The general trend was that the motifs containing “CG” had a significantly lower average read number (P < 2.2 × 10E-16) and call rate (P < 2.2 × 10E-16) than non-“CG”-containing motifs (Supplementary Fig. S3e,f).

Based on the genotyping results, we selected all SSR loci with at least two alleles, which yielded 630,906 pSSRs, accounting for 38.93% of overall SSRs. We compared the identified pSSRs with the indel (insertion/deletion) variants included in the Ensembl dbSNP. We found that 350,531 pSSRs had no intersection with any indel, whereas the other 280,375 (44.44%) pSSRs overlapped with at least one indel. Furthermore, we analysed overlap between the identified pSSRs and 887 microsatellite markers with clear genomic coordinates in porcine linkage maps. We found that 736 (82.98%) markers overlapped with pSSRs.

### Variation analysis of pSSRs

**Figure 3:**
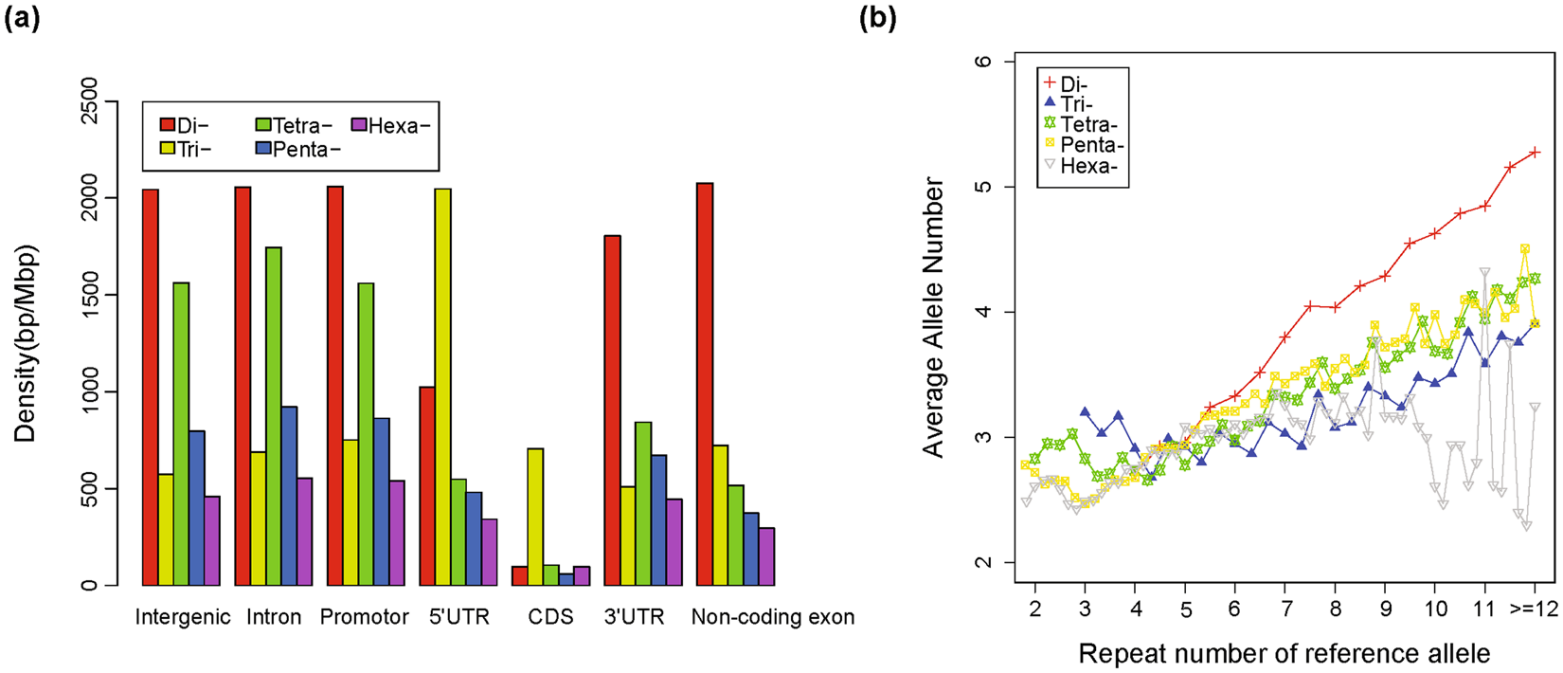
Distribution and polymorphism of pSSRs. (**a**) The distribution of each kind of polymorphic SSR in different genomic regions. (**b**) Average allele numbers of each kind of SSR plotted against the number of reference repeats.

First, we analysed the distribution of pSSRs in different genomic regions (Fig. 3a). In total, 456,035 of them were located in intergenic regions, 201,540 in introns, 5,058 in exonic regions and 14,293 in 2 kb-length defined promoters. The dinucleotide pSSR density within the non-exonic regions was similar and was lower in 3′UTRs, followed by the 5′UTRs, with the lowest density occurring in coding regions. Tetranucleotide, pentanucleotide and hexanucleotide pSSRs displayed a similar distribution to dinucleotide pSSRs. However, trinucleotide pSSRs had much higher density in 5′UTRs.

We then assessed the relationship between average allele number and the repeat number of the allele in assembly 10.2 of the pig reference genome (Fig. 3b). The average allele number generally became larger with increases in the repeat number of the allele in the reference genome, especially for dinucleotide pSSRs, indicating that the average allele number is positively related to SSR length. Compared within the same repeat number, dinucleotide pSSRs had a relative higher average allele number than any other kinds of pSSRs.

**Figure 4:**
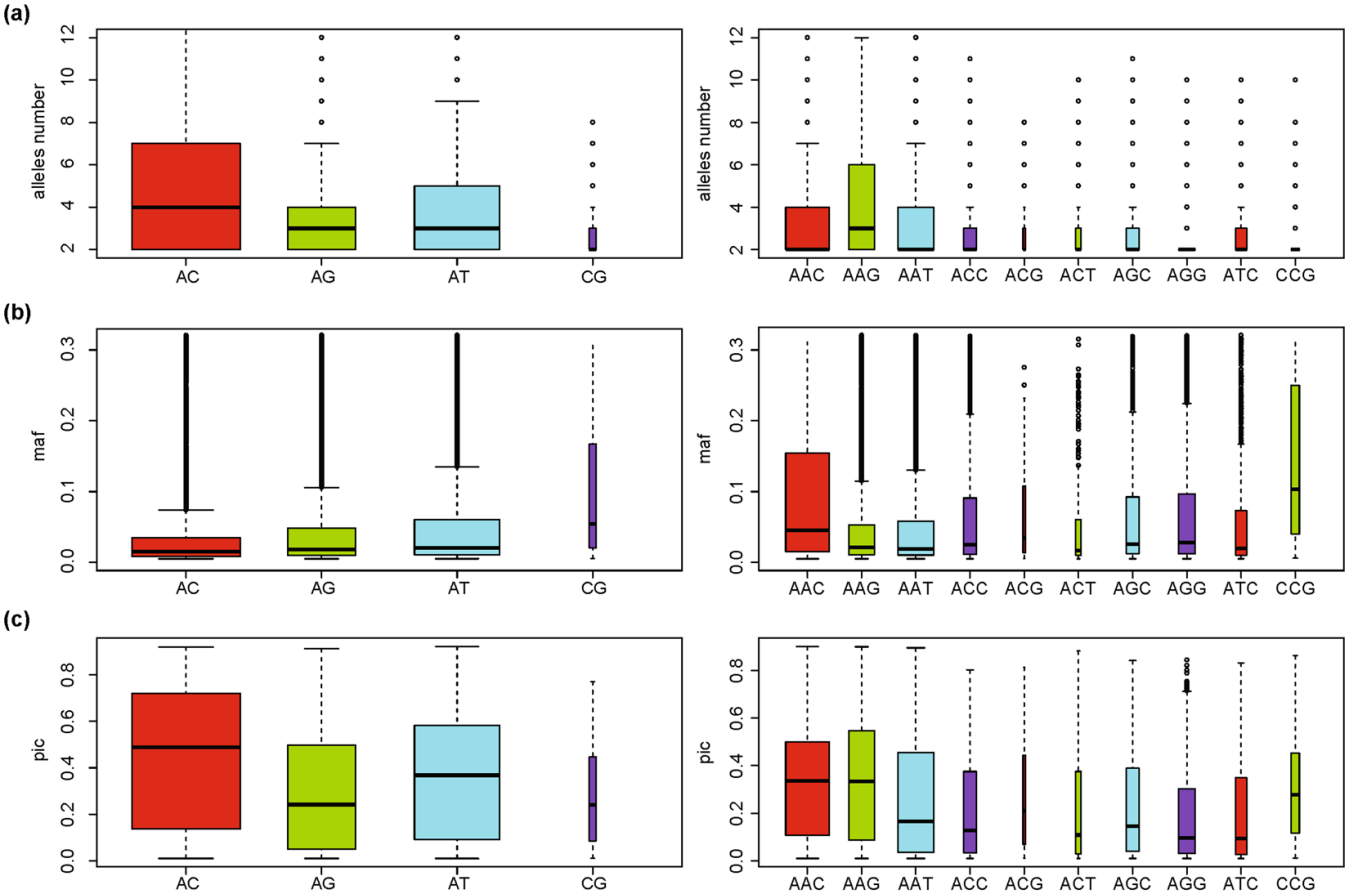
Comparisons of characteristics in distinct motifs. Differences of allele number (**a**), minor allele frequency (**b**) and PIC (**c**) of distinct motifs in dinucleotide (left) and trinucleotide (right) polymorphic SSRs. The CG-enriched motifs, such as CG, CCG, and ACG, usually have a trend of conservation, which is in accordance with the higher allele number, higher PIC, and lower minor allele frequency because of the rarity of opportunities for mutation. The bar width is proportional to the square root of the number of observations in the group.

We further assessed the influence of motif types to pSSR variability. We calculated the proportion of pSSRs in genotyped overall SSRs for different motif types individually. AC/GT and AT/AT dinucleotide SSRs have the highest pSSR proportion, measuring greater than 70%, followed by AG/CT and CG/CG dinucleotide SSRs. A/T-enriched motifs, including AAC/GTT, AAG/CTT and AAT/ATT, had relatively higher pSSR proportions than other types of trinucleotide SSRs, and similarly higher proportions of pSSR preferences for A/T-enriched motifs were also observed in tetra-, penta- and hexanucleotide SSRs. Then, we compared allele number, minor allele frequency (MAF) and polymorphism information content (PIC) across different types of di- and trinucleotide SSR motifs (Fig. 4). We found that AC dinucleotide SSRs had the highest allele numbers and PIC, followed by AT, AG and CG dinucleotide SSRs, whereas for trinucleotide SSRs, AAG, AAC and AAT had higher allele numbers and PIC (Fig. 4a,c). Conversely, it seems that CG dinucleotide SSRs and CCG trinucleotide SSRs have higher MAF than other motif types within the corresponding classes, which probably due to their lower average allele number (Fig. 4b).

### Conservation analysis of pSSRs

**Figure 5:**
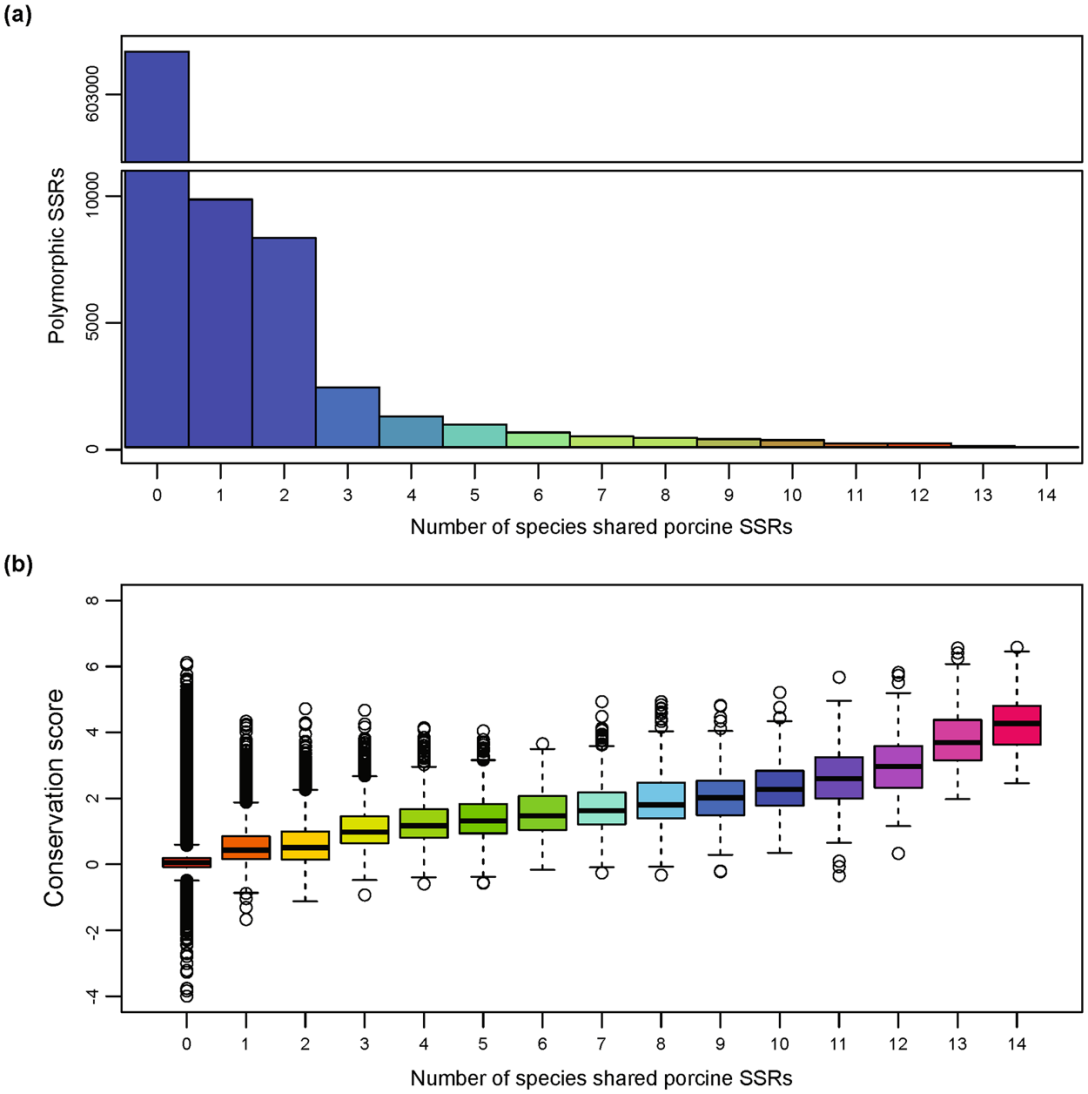
Conservation analysis of pSSRs. (**a**) Distribution of pSSRs according to the number of other species with BLAST hits. All pSSRs were classified into 15 categories: the first category, designated “0”, represents all pig-specific pSSRs, which have no hits in any other genome; the second category, “1”, includes the pSSRs with hits in one genome; the third category, “2”, includes the pSSRs that have hits in two genomes, etc.; finally, the fifteenth category, designated “14”, represents all common pSSRs, which are conserved in all analysed genomes. (**b**) Boxplots of average PhyloP in a locus as the conservation score in the above 15 categories.

We extracted the surrounding sequences (200 bp) of both sides of the identified high-quality pSSRs, then BLASTed them against 14 different species’ genomes. Generally, the number of pSSRs decreased as the number of species that shared porcine pSSRs increased (Fig. 5a). In total, we identified 604,694 pig-specific pSSRs and 102 highly conserved pSSRs (conserved in all species).

Furthermore, we calculated the average PhyloP score for the surrounding sequences on both sides of the high-quality pSSRs. A boxplot revealed that the average PhyloP score of pSSRs increased gradually with increases in the number of species that shared porcine pSSRs (Fig. 5b). We compared the average PhyloP score across different motifs of dinucleotides and trinucleotides SSRs, revealing that the surrounding sequences of CG-containing SSRs have a significantly higher average PhyloP score, indicating a relatively higher degree of conservation (Supplementary Fig. S5).

### Selection and loss-of-function analysis of high-quality pSSRs

To obtain high-quality pSSRs, we conducted a rigorous quality control and minor allele frequency filtering procedure and finally identified 16,527 high-quality pSSRs, including 7,032 dinucleotide, 2,379 trinucleotide, 5,026 tetranucleotide, 1,579 pentanucleotide and 511 hexanucleotide pSSRs (Supplementary Table S4). We found that 13,542 (81.94%) pSSRs overlapped with at least one indel in dbSNP, indicating the high reliability of the identified pSSRs. Among these, 12,137 pSSRs were in intergenic regions; 3,737 pSSRs were associated with one transcript, including 3484 in introns, 186 in 2 kb-length defined promoters, 1 in a 5′UTR, 9 in coding regions, 53 in 3′UTRs and 4 in the exons of non-coding transcripts; and 653 pSSRs loci intersecting with various transcripts (Supplementary Table S4).

For the selected high-quality pSSRs, we sought to identify loci with alleles causing potential loss of function (LoF), such as a frame-shift mutation in the coding regions or interference with splicing. Finally, we identified one SSR with a potential frame-shift allele and three pSSRs with alleles interfering with splicing. The first one was an “AG” repeat in SSC2 that had the non-reference major allele of a 4 bp deletion, overlapping with two dbSNP records (rs695224126 and rs792939048), which caused frame-shift mutation of a novel single-exon gene that probably belonged to ribosomal protein S17 family (ENSSSCT00000015379); however, it probably did not cause loss of function because its corresponding host transcripts (ENSSSCT00000015379) aligned with some pseudogenes with very high sequence identity. The second one was the “TTG” insertion in the acceptor site of the 28th intron of LAMA5 in SSC6, which was supported by two dbSNP records, rs699235883 and rs787272636. According to the multiple alignment results across 18 eutherian mammals, it seems that there is an error in the reference annotation. The correct core acceptor site is probably the “AG” behind the annotated acceptor site “TG”. Therefore, the “TTG” insertion here seems unlikely to produce abnormal splicing. The third one was the “GT” insertion in the donor site of the second intron of ARHGDIB in SSC5 (overlapped with dbSNP records rs696757177 and rs786598512), which was unlikely to affect correct splicing because any single instance of the “GT” dinucleotide motif in the “GT” tandem repeats could serve as a donor site. The last one was the “AAAAAG” deletion in SSCX, which overlapped with the donor sites of intron 36 of DOCK11 (ENSSSCT00000013815) and was evidenced by a dbSNP record (rs792712529).

### Genetic analysis of high-quality pSSRs in Chinese and European pig populations

**Figure 6:**
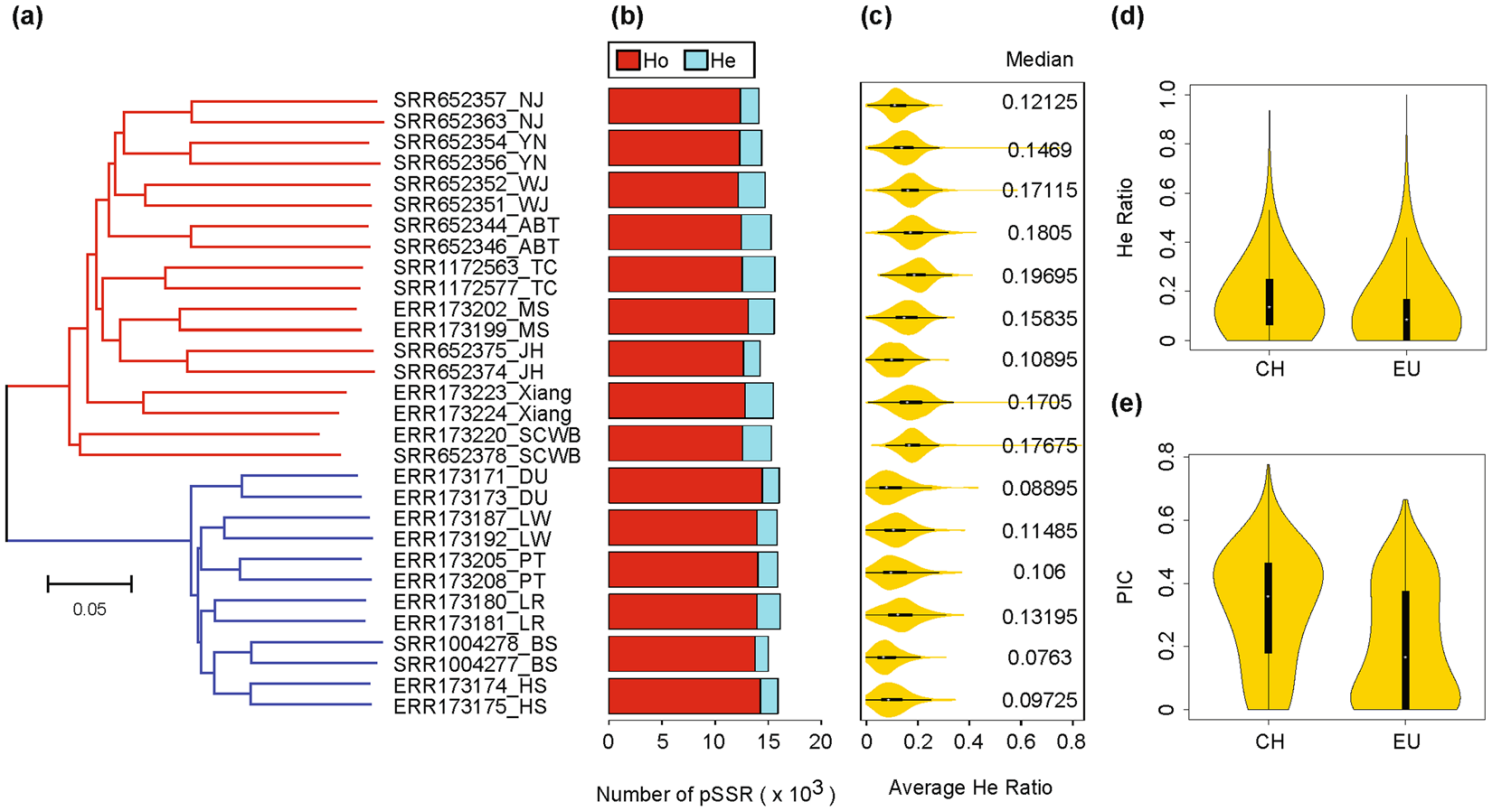
Validation of high-quality pSSRs in population genetics. (**a**) Neighbour-joining phylogenetic tree constructed from 30 samples based on all high-quality pSSRs. The red and blue branches represent Chinese and European breeds, respectively. The scale bar represents the pairwise distance. NJ, Neijiang; YN, Yanan; WJ, Wujin; ABT, Aba Tibetan; TC, Tongcheng; MS, Meishan; JH, Jinhua; SCWB, southern Chinese wild boar; DU, Duroc; LW, Large White; PT, Pietrain; LR, Landrace; BS, Berkshire; HS, Hampshire. (**b**) Average number of homozygous and heterozygous SSRs of two individuals in each breed. (**c**) Average heterozygous ratio of each breed in 10 Mb-length non-overlapping sliding windows. (**d**) Heterozygous ratio and (**e**) PIC of each high-quality pSSR in the “CH” and “EU” subpopulations. The “CH” subpopulation contains all Chinese breeds except for SCWB, and all 14 European breeds are in the “EU” subpopulation.

We constructed a phylogenetic tree according to high-quality pSSRs genotyping results in the 30 individuals. As shown in Fig. 6a, two individuals from each breed were clustered together; all Chinese pigs, including two wild boars, were clustered into one branch, whereas all commercial lean pigs clustered into an independent branch, which is consistent with the knowledge of their geographical distribution and breed history^27, 31^. In the branch of Chinese domestic pigs, pigs from Neijiang, Yanan, Wujin and Tibet were clustered together, representing a branch around the Qinghai-Tibet Plateau in the southwest of China; pigs from Tongcheng, Meishan and Jinhua formed another branch that represents pig breeds along the Yangzi river in central China; and Xiang pigs (mainly located in Guangxi province) represent an independent branch from the south of China. Therefore, the phylogenetic trees based on obtained high-quality pSSRs are in agreement with the relative geographical distribution and diverse climatic environments of the breeds.

We calculated the number of homozygous and heterozygous pSSRs for each breed (Fig. 6b). Generally, the number of heterozygous pSSRs was far less than that of homozygous pSSRs. Furthermore, we calculated the average heterozygous pSSR ratio of two individuals from the same breed in a defined window. We found that Chinese domestic pig breeds have generally higher heterozygous pSSR ratios than the western commercial lean pig breeds (Student’s t-test p value for median is 0.002287) (Fig. 6c), which was further supported by the group comparisons of heterozygous pSSR ratio and PIC between Chinese domestic pigs and western lean pigs (Fig. 6d,e). The finding of relatively lower heterozygous ratios and PIC in western lean pigs was in agreement with the fact that they experienced strong artificial selection as part of modern breeding, which was supported by previous observation^31^. We also noted that Jinhua and Neijiang pigs from the Chinese group had relatively lower heterozygous ratios, which was probably caused by the low coverage of the sequencing data of these two pig breeds.

## Discussion

Benefiting from the rapid technological development and subsequently declining cost of next-generation sequencing, large-scale genome sequencing has made tremendous progress in exploring genetic variation in pig breeds worldwide in the past few years. To date, release 86 of the porcine Ensembl variation database has collected ~55 million SNPs and ~4.8 million insertion and deletions. Here, we scanned all possible SSR loci in assembly 10.2 of the pig reference genome and conducted a population-scale analysis of SSR variation, providing another layer of genetic variation to existing catalogues.

Based on the defined SSR catalogue, population-scale resequencing data analysis yielded ~0.63 million candidate pSSRs, slightly less than the number of pSSR loci discovered from the human 1000 Genomes Project^19^. Within the identified candidate pSSRs, more than 55% are novel variants, indicating that SSRs could contribute a large number of variations. However, these novel variants are not included in dbSNP, probably because of the following reasons: (1) previous variant-calling procedures filtered out low-complexity repeat regions, and (2) popular software or pipelines focused on SNP calling and displayed poor performance in SSR variant calling.

From the catalogue, we noted that GC-containing SSR motifs have significantly lower sequencing coverage, indicating the effect of GC content on fragment yield in next-generation sequencing^32–34^. Furthermore, GC-containing SSR motifs generally displayed a lower proportion of polymorphism, which is consistent with the notion that GC-abundant sequences often correlated with functionality^35^. Perhaps due to the overlapping effect of lower sequencing coverage and proportion of polymorphism, the GC-containing SSR motifs, such as “CG” dinucleotide and “CCG” trinucleotide SSRs, are completely lost from the high-quality pSSR catalogue, which also limited the appearance of functional SSR variants. These observations remind us not only to increase the sequencing depth in future SSR discovery experiments based on next-generation sequencing but also to employ some improved library preparation protocols against GC bias^33^.

Consistent with previous observations^19^, intensive analysis regarding the identified pSSRs provide some biological insights into SSR variability: (1) most of the pSSRs are located outside the coding regions; (2) shorter SSR motifs have relatively higher variability, e.g., dinucleotide pSSRs have the highest variability; (3) pSSRs with longer reference alleles display higher variability. Apart from the above-summarized observations, the current study reveals that base composition also has a large influence on SSR variability. The general trend is that pSSRs with A/T-enriched motifs display higher variation, whereas CG-containing SSR motifs are relatively stable. The higher stability of CG-containing SSR motifs was reflected by their lower allele number and PIC but higher minor allele frequency (Fig. 4), which was consistent with and further supported by the higher average PhyloP score of their surrounding sequences.

Regarding the distribution of pSSR in coding regions, we found that non-triplet coding pSSRs are rarer than triplet coding pSSRs, which is in agreement with the observation in the human genome^19^. In our current study, we identified 654 potential frame-shift mutations (data not shown) in the catalogue of ~0.63 million candidate pSSRs; however, most of them are filtered out and do not appear in the catalogue of high quality pSSR due to their low frequency. The rare occurrence and low frequency of the non-triplet pSSRs in the coding regions indicated that they were not well tolerated and were probably under negative selection^19^. In the high-quality pSSR catalogue, we identified one pSSR with a potential frame-shift allele, although we finally ruled out the possibility that they contained deleterious alleles, but microsatellite/SSR variation-induced frame-shift mutation is well established to be the cause of some diseases, such as TGFB-II-, IGF-II-^36^ and MSH3-induced cancers^37^. Another example is that one copy of a TC deletion in the TC dinucleotide SSR of the first exon of the CCD4 gene resulted in yellow-fleshed peaches^38^. Therefore, these cases remind us to treat the non-triplet coding pSSRs as candidate functional variations in seeking the causative mutations underlying a phenotype. In addition, we found three pSSRs that overlapped with defined splicing sites. Although they probably will not lead to abnormal splicing, we could not exclude their potential influence on splicing efficiency because it has been observed in CFTR^39^ and NOS3^40^. In summary, common SSR polymorphisms rarely result in loss of function, but we should consider the possibility of SSRs as functional variations.

We have provided a catalogue of high-quality pSSRs that can be used to infer the patterns of porcine SSR variations such as allelic spectra, heterozygosity, PIC and population pedigree. However, the catalogue was produced from published genomic resequencing data of 102 individuals, a heterogeneous population consisting of Chinese domestic pigs and western lean-type pigs as well as some wild pigs. In addition, the number of pigs sampled from each breed was different, so the population is not genetically representative. In addition, the ineluctable shortcoming of the catalogue is allelic dropouts due to the low sequencing coverage of some selected individuals. Despite these problems, we screened for the minor allele frequencies greater than 0.1 to ensure the reliability of the pSSRs, which finally provided correct phylogenetic relationships and valuable summary statistics for the selected 30 individuals.

In conclusion, we analysed the overall distribution of SSRs in the pig reference genome and have provided a valuable catalogue of porcine polymorphic SSRs with next-generation sequencing data. To our knowledge, this study is the first to deeply analyse genome-wide SSR variation on a population scale with pig resequencing data. We hope this catalogue will be used for future genome-wide genetic analyses based on high-throughput markers.

## Methods

### Characterization of SSRs in pig genome assembly 10.2

The sequence of pig genome assembly 10.2 was downloaded from ftp://ftp.ensembl.org/pub/release-75/fasta/sus_scrofa/dna/. We employed the well-assembled chromosome 1 to 18 and sex chromosomes X and Y for microsatellite identification with the program Tandem Repeats Finder^41^. The size of the repeat unit of microsatellites in the current study is specified as two to six nucleotides. We set the length of an SSR region to at least 10 bp and the minimum repeat to 3, so the minimum repeats of each type of SSR are 5, 4, 3, 3 and 3 for dinucleotides, trinucleotides, tetranucleotides, pentanucleotides, and hexanucleotides, respectively.

Based on the scanning results from Tandem Repeats Finder, we removed all overlapping SSRs and ambiguous SSRs with non-specified motif length. With the resulting dataset, we analysed the total SSR density in different chromosomes or the whole genome, which was calculated by dividing the total length of SSRs existing in a particular chromosome or whole genome (with bp as the unit) by their corresponding length (with Mb as the unit). Furthermore, we analysed the count distribution of different SSR motifs according to their repeat number in the reference genome. For the distribution analysis, each kind of theoretically possible microsatellite repeat and its corresponding reverse complement, e.g., AC and GT for dinucleotide repeats, were categorized as one type, and for a specific repeat unit of a microsatellite, all related units generated using a one-base moving window were classified as one “motif”. Based on the reference genome annotation, we further analysed the distribution of SSRs in intergenic regions and different functional regions including introns, coding regions, 5′-untranslated regions (5′UTRs), 3′-untranslated regions (3′UTRs) and promoter regions, which was defined as 2 kb upstream of the given transcription start site.

To analyse potential enrichment of SSR in the vicinity of short interspersed elements (SINEs), we downloaded the characterized porcine SINE sequences from SINEBank (http://sines.eimb.ru/banks/SINEs.bnk), which were used for a BLAST search in the reference genome with an identity threshold of 65%. The 200 bp boundaries of SINEs were used for SSR enrichment analysis based on enriched SSR length density in non-overlapping sliding windows of 20 bp. The genome-wide average density of each kind of SSR motif was calculated and used as a control.

### Genotyping of SSRs based on resequencing data

We downloaded the genome resequencing data of 102 individuals from the NCBI SRA database (http://www.ncbi.nlm.nih.gov/sra/); the accession IDs and sample information are presented in Supplementary Table S3. The downloaded SRA data files were converted into FASTQ format with the SRA toolkits^42^, which were aligned to the pig reference genome via Bowtie 2 with default settings^43^. The resulting BAM files were sorted with SAMtools^44, 45^ and then used for SSR profiling with the program lobSTR^46^. For each SSR locus, the average coverage was calculated as the number of total mapped reads divided by the number of samples with available genotypes.

### Genotyping quality control and high-quality pSSR selection

After primary genotyping with lobSTR, all SSRs with only one allele (no polymorphism) were filtered out, and the remaining ones were defined as polymorphic SSRs (pSSRs). The polymorphism of each SSR locus within the 102 individuals was accessed through the number and frequency of alleles number, heterozygosity and polymorphism information content (PIC), and related descriptive statistical analysis was conducted with the R package. According to the recommended standard of lobSTR, high-quality polymorphic SSRs were selected with the following criteria: (1) average coverage greater than 3x; (2) average -log_10_(1-Q) bigger than 0.6, where Q is the likelihood ratio score of allelotype call; (3) at least 60% call rate; and (4) reference allele length less than or equal to 80 bp.

### Conservation analysis of pSSRs

To evaluate the conservation of the identified pSSRs, we extracted the surrounding sequences (200 bp) on each side of the identified pSSRs, then BLASTed them against 14 different species’ genomes (Bos taurus, Canis lupus familiaris, Equus caballus, Felis catus, Gorilla gorilla, Loxodonta africana, Macropus eugenii, Ornithorhynchus anatinus, Oryctolagus cuniculus, Ovis aries, Pan troglodytes, Rattus norvegicus, Mus musculus, Homo sapiens). If both sides of one SSR locus could be aligned co-ordinately with a specific region in the query genome, and the interval between the two sides is less than 300 bp, it was regarded as one hit.

Furthermore, we downloaded the multiple alignment format files of 100 vertebrate species and the corresponding PhyloP score files from http://hgdownload.soe.ucsc.edu/goldenPath/hg38/, which were used to construct the positional relationship between the human and pig genomes with in-house Python scripts. Then, the conservation of each SSR was represented by the average PhyloP score of its surrounding sequences on both sides, transformed from human to pig according to their corresponding positional relationship.

### Population genetics analysis of high-quality polymorphic SSRs

To access the utility of obtained high-quality pSSRs in population genetics, we employed the resequencing data of 30 individuals from eight Chinese domestic pig breeds, Chinese wild boars and six European domestic pig breeds. Two individuals from each pig breed were selected according to their sequencing depth. With MEGA 7.0^47^, the phylogenetic tree was constructed with identified high-quality polymorphic SSRs using the Neighbour-Joining method^48^, where the pairwise distance was defined as: 1 - identical SSRs / (identical SSRs + distinct SSRs)^49^.

To evaluate the diversity of each sample, we calculated binned heterozygous ratios with high-quality polymorphic SSRs, which was calculated as the number of heterozygous pSSRs in each non-overlapping 10 Mb sliding window divided by the total number of pSSRs in the corresponding window. To compare the genetic diversity of Chinese domestic pigs and commercial lean pigs, the heterozygous ratio and PIC for each locus were calculated within groups. The heterozygous ratio for each SSR locus was calculated as the number of heterozygous individuals divided by the number of individuals within the group, and the PIC for each locus was calculated as PIC = 1 − ∑(P_i_)^2^, where P_i_ is the allele frequency.

## Electronic supplementary material

Supplementary files
