# Supplementary material for: Characterization of porcine simple sequence repeat variation on a population scale with genome resequencing data
Source: Sci Rep. 2017 May 24;7:2376. doi: 10.1038/s41598-017-02600-8 (PMC5443785; doi:10.1038/s41598-017-02600-8)
Supplement: Supplementary file 1 — Supplementary files [file 41598_2017_2600_MOESM1_ESM.pdf]

Characterization of porcine simple sequence repeat variation on a population scale with genome resequencing data

Congcong Liu[1], Yan Liu[1], Xinyi Zhang[1], Xuewen Xu[1,2,*], Shuhong Zhao[1,2]

[1]Key Lab of Agricultural Animal Genetics, Breeding, and Reproduction of the Ministry of Education & Key Lab of Swine Genetics and Breeding of the Ministry of Agriculture, College of Animal Science and Technology, Huazhong Agricultural University, Wuhan, 430070 Hubei, PR China

[2]The Cooperative Innovation Center for Sustainable Pig Production, Wuhan 430070, China

*Corresponding author

Contact information
Xuewen Xu
College of Animal Science and Technology,
Huazhong Agricultural University,
Wuhan, 430070 Hubei, China
Tel.: +86 27 87388470
Fax: +86 27 87280408
Email: xuewen_xu@mail.hzau.edu.cn

Supplementary Table S1. Distribution of each kind of SSRs in pig genome.

Chromosome	SSR Type	SSR Count	Count/Mbp	Density(bp/Mbp)
1	Dinucleotide	46900	148.737	2997.076
1	Trinucleotide	24229	76.839	1397.803
1	Tetranucleotide	59311	188.097	3771.753
1	Pentanucleotide	33042	104.788	2147.679
1	Hexanucleotide	24604	78.028	1816.823
2	Dinucleotide	24529	150.883	3033.646
2	Trinucleotide	13209	81.251	1473.586
2	Tetranucleotide	32234	198.278	3981.715
2	Pentanucleotide	17750	109.184	2232.395
2	Hexanucleotide	14319	88.079	2053.246
3	Dinucleotide	22044	152.251	3095.955
3	Trinucleotide	12165	84.02	1533.719
3	Tetranucleotide	29068	200.763	4109.849
3	Pentanucleotide	15995	110.472	2291.319
3	Hexanucleotide	13973	96.507	2270.33
4	Dinucleotide	21996	153.319	3114.725
4	Trinucleotide	11723	81.713	1476.803
4	Tetranucleotide	28391	197.894	3995.345
4	Pentanucleotide	15629	108.939	2261.645
4	Hexanucleotide	12503	87.15	2030.092
5	Dinucleotide	16763	150.332	3005.988
5	Trinucleotide	9472	84.946	1542.53
5	Tetranucleotide	21750	195.056	3908.16
5	Pentanucleotide	12238	109.752	2252.247
5	Hexanucleotide	9637	86.426	2007.274
6	Dinucleotide	23181	146.933	2981.474
6	Trinucleotide	13657	86.565	1593.776
6	Tetranucleotide	31856	201.92	4130.349
6	Pentanucleotide	17490	110.861	2284.522
6	Hexanucleotide	15072	95.534	2230.607
7	Dinucleotide	20227	150.091	3051.575
7	Trinucleotide	11504	85.364	1560.344
7	Tetranucleotide	26943	199.927	4047.757
7	Pentanucleotide	15119	112.188	2326.458
7	Hexanucleotide	12704	94.268	2203.028
8	Dinucleotide	23586	158.837	3233.538
8	Trinucleotide	11493	77.398	1414.933
8	Tetranucleotide	28623	192.758	3883.46
8	Pentanucleotide	15801	106.41	2203.098
8	Hexanucleotide	12250	82.496	1927.756
9	Dinucleotide	23709	154.285	3109.822
9	Trinucleotide	12367	80.478	1466.042
9	Tetranucleotide	29440	191.579	3794.522
9	Pentanucleotide	16485	107.275	2189.501
9	Hexanucleotide	12236	79.625	1875.569
10	Dinucleotide	12618	159.515	3281.798
10	Trinucleotide	6558	82.905	1509.854
10	Tetranucleotide	15955	201.701	4151.961
10	Pentanucleotide	8909	112.626	2330.904
10	Hexanucleotide	8056	101.843	2406.97
11	Dinucleotide	13576	154.817	3111.965
11	Trinucleotide	6827	77.853	1420.837
11	Tetranucleotide	16851	192.164	3893.714

11	Pentanucleotide	9272	105.735	2164.736
11	Hexanucleotide	7488	85.391	1981.148
12	Dinucleotide	9015	141.771	2811.622
12	Trinucleotide	5990	94.199	1714.836
12	Tetranucleotide	13282	208.874	4145.336
12	Pentanucleotide	7586	119.298	2448.144
12	Hexanucleotide	7058	110.995	2583.672
13	Dinucleotide	33032	151.083	3031.648
13	Trinucleotide	16782	76.758	1385.495
13	Tetranucleotide	42751	195.536	3930.711
13	Pentanucleotide	23367	106.877	2207.023
13	Hexanucleotide	17311	79.178	1837.952
14	Dinucleotide	23893	155.299	3171.893
14	Trinucleotide	13315	86.544	1593.122
14	Tetranucleotide	31416	204.196	4136.288
14	Pentanucleotide	17150	111.471	2287.459
14	Hexanucleotide	14146	91.946	2139.394
15	Dinucleotide	24274	153.943	3129.198
15	Trinucleotide	11872	75.291	1363.038
15	Tetranucleotide	30296	192.134	3906.01
15	Pentanucleotide	16773	106.373	2196.255
15	Hexanucleotide	12422	78.779	1821.455
16	Dinucleotide	13865	159.553	3248.197
16	Trinucleotide	6755	77.734	1420.592
16	Tetranucleotide	16625	191.314	3846.443
16	Pentanucleotide	9178	105.617	2158.61
16	Hexanucleotide	6774	77.953	1815.74
17	Dinucleotide	10542	151.245	3141.464
17	Trinucleotide	5778	82.896	1537.713
17	Tetranucleotide	14511	208.188	4371.723
17	Pentanucleotide	7895	113.269	2360.406
17	Hexanucleotide	6642	95.292	2233.45
18	Dinucleotide	9373	153.103	3033.401
18	Trinucleotide	5035	82.244	1561.106
18	Tetranucleotide	11533	188.386	3662.377
18	Pentanucleotide	6529	106.648	2176.214
18	Hexanucleotide	5600	91.473	2135.231
X	Dinucleotide	22613	156.721	3250.66
X	Trinucleotide	11096	76.902	1439.14
X	Tetranucleotide	26716	185.157	3725.703
X	Pentanucleotide	14991	103.896	2135.691
X	Hexanucleotide	12369	85.724	1999.193
Y	Dinucleotide	207	126.396	2383.808
Y	Trinucleotide	144	87.927	1599.789
Y	Tetranucleotide	315	192.341	4142.965
Y	Pentanucleotide	181	110.52	2308.703
Y	Hexanucleotide	144	87.927	2430.824
All	Dinucleotide	395943	152.483	3089.982
All	Trinucleotide	209971	80.863	1477.24
All	Tetranucleotide	507867	195.586	3947.496
All	Pentanucleotide	281380	108.363	2230.945
All	Hexanucleotide	225308	86.769	2024.533

Supplementary Table S2. Distribution of SSRs in different genomic regions.

unit length	feature	count	count/Mb	length	length/Mb
2	3'utr	1971	146.447	37085	2755.445
2	5'utr	354	122.389	6409	2215.788
2	cds	845	26.083	11397	351.794
2	exon	238	122.989	5740	2966.203
2	intron	115445	153.526	2273707	3023.714
2	promoter	8901	159.578	176721	3168.263
2	intergenic	295353	154.093	6046489	3154.604
3	3'utr	1011	75.118	17745	1318.468
3	5'utr	1485	513.41	30788	10644.356
3	cds	6832	210.885	131087	4046.291
3	exon	312	161.229	6371	3292.278
3	intron	65568	87.196	1185662	1576.766
3	promoter	6452	115.672	121658	2181.09
3	intergenic	145963	76.153	2667311	1391.602
4	3'utr	2153	159.97	37031	2751.433
4	5'utr	719	248.58	12413	4291.555
4	cds	1470	45.375	23956	739.455
4	exon	212	109.553	3963	2047.92
4	intron	159129	211.619	3121794	4151.552
4	promoter	12208	218.866	240685	4315.012
4	intergenic	368982	192.507	7533565	3930.448
5	3'utr	1616	120.07	30652	2277.468
5	5'utr	740	255.841	15136	5232.979
5	cds	1333	41.146	23705	731.707
5	exon	183	94.567	3408	1761.118
5	intron	89952	119.624	1835760	2441.306
5	promoter	8091	145.056	164774	2954.076
5	intergenic	200977	104.855	4158150	2169.41
6	3'utr	1046	77.719	23363	1735.889
6	5'utr	392	135.526	9180	3173.807
6	cds	2045	63.123	49450	1526.384
6	exon	145	74.93	3670	1896.509
6	intron	76767	102.089	1775897	2361.696
6	promoter	6082	109.038	142212	2549.584
6	intergenic	157203	82.017	3677799	1918.799
all	3'utr	7797	579.323	145876	10838.702
all	5'utr	3690	1275.746	73926	25558.485
all	cds	12525	386.612	239595	7395.632
all	exon	1090	563.268	23152	11964.029
all	intron	506861	674.055	10192820	13555.034
all	promoter	41734	748.209	846050	15168.025
all	intergenic	1168478	609.624	24083314	12564.864

Supplementary Table S3. Information of 102 individuals.

Run	Bases	Study	BioSample	Breed
SRR1004277	13.4G	SRP030626	SAMN02364505	Berkshire
SRR1004278	11.5G	SRP030626	SAMN02364506	Berkshire
SRR1004279	11.4G	SRP030626	SAMN02364507	Berkshire
ERR173171	23.5G	ERP001813	SAMEA1557419	Duroc
ERR173172	12.3G	ERP001813	SAMEA1557423	Duroc
ERR173173	15.4G	ERP001813	SAMEA1557434	Duroc
ERR173174	23.4G	ERP001813	SAMEA1557391	Hampshire
ERR173175	20.6G	ERP001813	SAMEA1557400	Hampshire
ERR173179	21.4G	ERP001813	SAMEA1557388	Jiangquhai
SRR652374	11.6G	SRP018123	SAMN01894455	Jinhua
SRR652375	12.4G	SRP018123	SAMN01894456	Jinhua
SRR652376	10.6G	SRP018123	SAMN01894457	Jinhua
ERR173180	19G	ERP001813	SAMEA1557405	Landrace
ERR173181	28.1G	ERP001813	SAMEA1557416	Landrace
ERR173182	18.4G	ERP001813	SAMEA1557426	Landrace
ERR173183	15.1G	ERP001813	SAMEA1557436	Landrace
ERR173184	15.5G	ERP001813	SAMEA1557390	Landrace
ERR173185	11.2G	ERP001813	SAMEA1557412	LargeWhite
ERR173186	20.8G	ERP001813	SAMEA1557415	LargeWhite
ERR173187	20.8G	ERP001813	SAMEA1557431	LargeWhite
ERR173188	20.8G	ERP001813	SAMEA1557413	LargeWhite
ERR173189	19.2G	ERP001813	SAMEA1557402	LargeWhite
ERR173191	19.5G	ERP001813	SAMEA1557435	LargeWhite
ERR173192	20.9G	ERP001813	SAMEA1557422	LargeWhite
ERR173193	20.3G	ERP001813	SAMEA1557406	LargeWhite
ERR173194	17.3G	ERP001813	SAMEA1557404	LargeWhite
ERR173195	18G	ERP001813	SAMEA1557383	LargeWhite
ERR173196	19.2G	ERP001813	SAMEA1557425	LargeWhite
ERR173197	17.5G	ERP001813	SAMEA1557427	LargeWhite
ERR173198	17.6G	ERP001813	SAMEA1557399	LargeWhite
SRR1178916	40.9G	SRP039012	SAMN02665306	mangalica
SRR1178923	63.2G	SRP039012	SAMN02665304	mangalica
SRR1178925	41.8G	SRP039012	SAMN02665305	mangalica
ERR173199	18.9G	ERP001813	SAMEA1557420	Meishan
ERR173200	18.8G	ERP001813	SAMEA1557428	Meishan
ERR173201	17.9G	ERP001813	SAMEA1557395	Meishan
ERR173202	20.8G	ERP001813	SAMEA1557410	Meishan
SRR652357	15.7G	SRP018123	SAMN01894447	Neijiang
SRR652363	11.4G	SRP018123	SAMN01894452	Neijiang
SRR652348	11.8G	SRP018123	SAMN01894438	Penzhou
SRR652350	14.1G	SRP018123	SAMN01894440	Penzhou
ERR173204	21.4G	ERP001813	SAMEA1557430	Pietrain
ERR173205	21.7G	ERP001813	SAMEA1557432	Pietrain
ERR173206	17.2G	ERP001813	SAMEA1557392	Pietrain
ERR173207	11.3G	ERP001813	SAMEA1557408	Pietrain
ERR173208	22.7G	ERP001813	SAMEA1557397	Pietrain
ERR173209	20.2G	ERP001813	SAMEA1557409	SusCebifrons
ERR173211	25.9G	ERP001813	SAMEA1557384	SusVerrucosus
SRR652258	12.2G	SRP018123	SAMN01894346	Tibetan
SRR652264	11.8G	SRP018123	SAMN01894369	Tibetan
SRR652343	11.5G	SRP018123	SAMN01894412	TibetanABa
SRR652344	18.6G	SRP018123	SAMN01894434	TibetanABa
SRR652345	14.5G	SRP018123	SAMN01894435	TibetanABa
SRR652346	18.5G	SRP018123	SAMN01894436	TibetanABa
SRR652347	15.1G	SRP018123	SAMN01894437	TibetanABa
SRR652262	16G	SRP018123	SAMN01894367	TibetanDiqing
SRR652265	17.6G	SRP018123	SAMN01894370	TibetanDiqing
SRR652327	15.6G	SRP018123	SAMN01894407	TibetanGannan
SRR652339	12G	SRP018123	SAMN01894408	TibetanGannan
SRR652340	12.9G	SRP018123	SAMN01894409	TibetanGannan
SRR652341	12.5G	SRP018123	SAMN01894410	TibetanGannan
SRR652342	11.7G	SRP018123	SAMN01894411	TibetanGannan

SRR652257	12G	SRP018123	SAMN01894345	TibetanGanzi
SRR652259	10.6G	SRP018123	SAMN01894349	TibetanGanzi
SRR652260	14.3G	SRP018123	SAMN01894360	TibetanGanzi
SRR652261	14.2G	SRP018123	SAMN01894361	TibetanGanzi
SRR652267	9.8G	SRP018123	SAMN01894387	TibetanNyingchi
SRR652268	19G	SRP018123	SAMN01894388	TibetanNyingchi
SRR652269	13.4G	SRP018123	SAMN01894389	TibetanNyingchi
SRR652270	12.2G	SRP018123	SAMN01894390	TibetanNyingchi
SRR652302	17.9G	SRP018123	SAMN01894391	TibetanNyingchi
SRR652303	14.7G	SRP018123	SAMN01894392	TibetanShigatse
SRR652304	11.5G	SRP018123	SAMN01894393	TibetanShigatse
SRR652305	15G	SRP018123	SAMN01894394	TibetanShigatse
SRR652306	12.4G	SRP018123	SAMN01894395	TibetanShigatse
SRR652307	14.9G	SRP018123	SAMN01894406	TibetanShigatse
SRR1172563	22.5G	SRP038221	SAMN02646543	Tongcheng
SRR1172577	24.4G	SRP038221	SAMN02646545	Tongcheng
SRR1216635	17.5G	SRP038221	SAMN02646544	Tongcheng
SRR1216636	16.6G	SRP038221	SAMN02646546	Tongcheng
ERR173217	19.2G	ERP001813	SAMEA1557401	WildBoarFrance
ERR173212	22.5G	ERP001813	SAMEA1557411	WildBoarJapan
ERR173215	10.9G	ERP001813	SAMEA1557394	WildBoarNetherlandsMeinweg
ERR173216	16.3G	ERP001813	SAMEA1557387	WildBoarNetherlandsMeinweg
ERR173213	18.8G	ERP001813	SAMEA1557424	WildBoarNetherlandsVeluwe
ERR173214	23.5G	ERP001813	SAMEA1557433	WildBoarNetherlandsVeluwe
ERR173221	9.8G	ERP001813	SAMEA1557437	WildBoarNorthChina
ERR173222	20.2G	ERP001813	SAMEA1557421	WildBoarNorthChina
ERR173219	10.3G	ERP001813	SAMEA1557386	WildboarSouthChina
ERR173220	20.8G	ERP001813	SAMEA1557396	WildBoarSouthChina
SRR652377	12G	SRP018123	SAMN01894458	WildBoarSouthChina
SRR652378	16.3G	SRP018123	SAMN01894459	WildBoarSouthChina
SRR652379	16.3G	SRP018123	SAMN01894460	WildBoarSouthChina
ERR173218	29.4G	ERP001813	SAMEA1557403	WildBoarSwitzerlandMalcantone
SRR652351	15.9G	SRP018123	SAMN01894441	Wujin
SRR652352	14.2G	SRP018123	SAMN01894442	Wujin
SRR652353	12G	SRP018123	SAMN01894443	Wujin
ERR173223	18.4G	ERP001813	SAMEA1557398	Xiang
ERR173224	18.1G	ERP001813	SAMEA1557385	Xiang
SRR652354	12.1G	SRP018123	SAMN01894444	Yanan
SRR652355	11.1G	SRP018123	SAMN01894445	Yanan
SRR652356	13.1G	SRP018123	SAMN01894446	Yanan

Supplementary Table S4. Information of polymorphic SSRs.

motif	chromosome	start	end	ref repeat	transcript id	gene name	feature	conservation	dbSNP	alleles
AC.GT	1	1084452	1084463	6	ENSSSCT00000004431	WDR27	intron	-0.432	.	3
AC.GT	1	1084452	1084463	6	ENSSSCT00000004434	ERMARD	intron	-0.432	.	3
AC.GT	1	1969878	1969899	11	.	.	intergenic	.	rs788463500	4
AAAAG.CTTTT	1	2874375	2874390	3.2	.	.	intergenic	0.134	502872 rs7888	2
AAAAC.GTTTT	1	3226034	3226057	4.8	.	.	intergenic	-0.132	.	4
AG.CT	1	4062541	4062553	6.5	.	.	intergenic	-0.332	rs791046496	2
AC.GT	1	4336676	4336697	11	.	.	intergenic	-0.303	rs792187070	2
ACAG.CTGT	1	4883530	4883563	8.5	.	.	intergenic	-0.032	rs790720923	2
AC.GT	1	5054068	5054086	9.5	.	.	intergenic	-0.235	rs792589336	2
AAAT.ATTT	1	6199497	6199513	4.25	.	.	intergenic	.	rs790148838	2
AAC.GTT	1	6344042	6344055	4.66667	.	.	intergenic	.	rs706466330	2
AAAT.ATTT	1	6600847	6600862	4	.	.	intergenic	-0.749	rs788323413	2
AAAC.GTTT	1	6603990	6604006	4.25	.	.	intergenic	-0.032	389601 rs7893	2
AG.CT	1	6775793	6775814	11	.	.	intergenic	-0.211	355022 rs7902	4
AT.AT	1	6887154	6887167	7	.	.	intergenic	0.147	750062 rs7863	3
AAAAC.GTTTT	1	6975470	6975501	6.4	.	.	intergenic	0.207	rs788965134	2
AC.GT	1	7131330	7131362	16.5	.	.	intergenic	-0.023	rs702403861	3
AAAC.GTTT	1	7302256	7302284	7.25	ENSSSCT00000004459	PARK2	intron	0.165	943149 rs7871	2
AAAC.GTTT	1	7317717	7317738	5.5	ENSSSCT00000004459	PARK2	intron	-0.268	580533 rs7878	2
AC.GT	1	7494020	7494032	6.5	ENSSSCT00000004459	PARK2	intron	.	rs787554650	3
AAT.ATT	1	7500466	7500483	6	ENSSSCT00000004459	PARK2	intron	0.017	rs789938892	2
AAAT.ATTT	1	7537649	7537666	4.5	ENSSSCT00000004459	PARK2	intron	-0.607	rs790609900	2
AAAC.GTTT	1	7711651	7711665	3.75	ENSSSCT00000004459	PARK2	intron	.	703991 rs7932	2
AG.CT	1	7841421	7841430	5	.	.	intergenic	-0.132	rs697031563	2
AAAAT.ATTTT	1	7966978	7966992	3	.	.	intergenic	.	rs791064598	2
AC.GT	1	7968585	7968610	13	.	.	intergenic	0.018	342666 rs7885	2
AAAAC.GTTTT	1	8390257	8390276	4	ENSSSCT00000004461	AGPAT4	intron	-0.069	rs786341973	2
ACAT.ATGT	1	8410522	8410536	3.75	ENSSSCT00000004461	AGPAT4	intron	-0.036	rs793406062	2
AGC.GCT	1	8517259	8517276	6	ENSSSCT00000004465	.	intron	-0.465	rs793698502	2
AC.GT	1	8829617	8829630	7	ENSSSCT00000004467	SLC22A3	intron	-0.075	rs790865956	2
AC.GT	1	9540541	9540555	7.5	.	.	intergenic	.	rs792066398	2
AAGG.CCTT	1	9555317	9555342	6.5	.	.	intergenic	-0.011	rs793646991	2
AT.AT	1	9656766	9656775	5	.	.	intergenic	-0.004	rs697943185	2
AAAAC.GTTTT	1	9842832	9842857	5.2	ENSSSCT00000004480	FNDC1	intron	-0.125	rs789382973	2
AATG.CATT	1	9931936	9931959	6	.	.	intergenic	-0.174	rs789660373	2
AAAGG.CCTTT	1	9995403	9995419	3.4	.	.	intergenic	-0.107	rs790741907	2
AAAAC.GTTTT	1	10140516	10140533	3.6	ENSSSCT00000004483	RSPH3	intron	.	rs787080539	2
AAAC.GTTT	1	10201654	10201677	6	.	.	intergenic	0.198	981079 rs7896	2
AAAC.GTTT	1	10652559	10652572	3.5	.	.	intergenic	.	rs786371742	2
AAAC.GTTT	1	10762186	10762204	4.75	ENSSSCT00000027733	.	intron	.	.	2
AAG.CTT	1	11460374	11460392	6.33333	.	.	intergenic	-0.223	rs791901651	2
AC.GT	1	11553654	11553668	7.5	.	.	intergenic	-0.039	.	2
AG.CT	1	11671921	11671931	5.5	.	.	intergenic	.	.	2
AAAC.GTTT	1	11713500	11713515	4	ENSSSCT00000004492	.	intron	0.215	rs789509937	2
AAC.GTT	1	11742438	11742451	4.66667	ENSSSCT00000004492	.	intron	.	rs790544711	2
AC.GT	1	11922585	11922604	10	.	.	intergenic	.	.	4
AC.GT	1	12403704	12403725	11	.	.	intergenic	0.31	rs789806900	2
AAG.CTT	1	12611170	12611183	4.66667	.	.	intergenic	-0.028	rs791506100	2
AAAT.ATTT	1	12627427	12627439	3.25	.	.	intergenic	0.048	rs791486872	2
AC.GT	1	12666645	12666680	18	.	.	intergenic	0.164	101909 rs7934	2
AG.CT	1	12960924	12960936	6.5	.	.	intergenic	0.124	rs703979254	5
ATC.GAT	1	13437854	13437869	5.33333	.	.	intergenic	0.185	rs790889517	2
AAGC.GCTT	1	13495642	13495664	5.75	.	.	intergenic	.	066385 rs7894	2
AAAAC.GTTTT	1	13599074	13599091	3	ENSSSCT00000031943	TFB1M	intron	.	590558 rs7917	2
AC.GT	1	13613107	13613119	6.5	ENSSSCT00000031943	TFB1M	intron	-0.067	403028 rs7885	2
AAAAC.GTTTT	1	13697925	13697948	4.8	ENSSSCT00000004494	TIAM2	intron	-0.2	rs787491324	2
AAAAC.GTTTT	1	13697925	13697948	4.8	ENSSSCT00000027128	TIAM2	intron	-0.2	rs787491324	2
AC.GT	1	13700410	13700420	5.5	ENSSSCT00000004494	TIAM2	intron	-0.1	.	3
AC.GT	1	13700410	13700420	5.5	ENSSSCT00000027128	TIAM2	intron	-0.1	.	3
AAAT.ATTT	1	13791250	13791270	5.25	.	.	intergenic	.	.	2
AAAT.ATTT	1	14407955	14407973	4.75	ENSSSCT00000004500	.	intron	0.247	rs788683343	2
AC.GT	1	14600614	14600627	7	ENSSSCT00000004501	.	intron	0	rs793563016	2
AAC.GTT	1	14623134	14623147	4.66667	.	.	intergenic	-0.119	527592 rs7892	5
AAAG.CTTT	1	14896538	14896549	3	.	.	intergenic	-0.021	.	2
AAAC.GTTT	1	14928503	14928516	3.5	.	.	intergenic	0.028	rs792568041	2
AC.GT	1	15405845	15405859	7.5	.	.	intergenic	.	302227 rs7870	2
AAAAC.GTTTT	1	15519417	15519443	4.5	.	.	intergenic	-0.061	rs692048080	2
AT.AT	1	15564918	15564941	12	.	.	intergenic	0.537	465066 rs7899	2
AAAAC.GTTTT	1	15580047	15580070	4.8	.	.	intergenic	0.181	rs791463609	2
AC.GT	1	15730042	15730053	6	.	.	intergenic	.	.	2
AT.AT	1	15811346	15811364	9.5	.	.	intergenic	0.12	rs793636148	2
AT.AT	1	16037898	16037909	6	.	.	intergenic	-0.133	233206 rs7886	2
AAAC.GTTT	1	16043276	16043290	3.75	.	.	intergenic	0.074	545834 rs7894	2
AAT.ATT	1	16106484	16106503	6.66667	ENSSSCT00000004510	.	intron	0.052	rs690406510	2
AG.CT	1	16168442	16168455	7	ENSSSCT00000004510	.	intron	-0.009	994057 rs7025	6
AG.CT	1	16168442	16168455	7	ENSSSCT00000030512	.	intron	-0.009	994057 rs7025	6
AC.GT	1	16243125	16243136	6	.	.	intergenic	0.38	rs699214597	3
AAAAC.GTTTT	1	16407160	16407182	4.6	ENSSSCT00000004511	.	intron	-0.279	765270 rs7924	2
AG.CT	1	16522300	16522309	5	.	.	intergenic	.	rs793887413	2
AT.AT	1	16954338	16954351	7	.	.	intergenic	.	129803 rs7864	2
AAAAG.CTTTT	1	17064141	17064166	4.33333	.	.	intergenic	-0.014	508143 rs7918	2
AC.GT	1	17125793	17125812	10	ENSSSCT00000004518	CCDC170	intron	0.201	.	2
AC.GT	1	17125793	17125812	10	ENSSSCT00000030322	CCDC170	intron	0.201	.	2

AAATG.CATTT	1	17190714	17190734	4.2	ENSSSCT00000004519	C6orf211	3'utr	0.311	474168 rs7910	2
AT.AT	1	17229949	17229964	8	ENSSSCT00000004520	RMND1	intron	0.058	rs707573049	2
AAATG.CATTT	1	17234709	17234727	3.8	ENSSSCT00000004520	RMND1	intron	-0.251	471975 rs7913	2
AC.GT	1	17247561	17247570	5	ENSSSCT00000004520	RMND1	intron	-0.021	rs789218113	2
AAAC.GTTT	1	17252969	17252987	4.75	ENSSSCT00000004520	RMND1	intron	.	rs790570326	2
AAC.GTT	1	17347418	17347431	4.66667	.	.	intergenic	0.251	rs693832947	4
AT.AT	1	17495488	17495500	6.5	.	.	intergenic	-0.187	497810 rs7882	2
AC.GT	1	17796962	17796983	11	.	.	intergenic	.	rs790238141	2
AAAAC.GTTTT	1	17915438	17915457	3.33333	.	.	intergenic	0.231	rs792853253	2
AC.GT	1	17916678	17916689	6	.	.	intergenic	.	rs790410673	2
AAAAT.ATTTT	1	18035005	18035027	4.6	ENSSSCT00000004526	PPP1R14C	intron	-0.083	rs790941110	2
AAAC.GTTT	1	18085891	18085906	4	.	.	intergenic	-0.096	rs793875210	2
AAAT.ATTT	1	18193220	18193234	3.75	.	.	intergenic	.	rs790840692	2
AC.GT	1	18491402	18491426	12.5	ENSSSCT00000004536	LRP11	intron	.	183637 rs7053	2
AC.GT	1	18513426	18513440	7.5	ENSSSCT00000004536	LRP11	intron	0.166	rs786633528	2
AAAAC.GTTTT	1	18580440	18580468	5.8	ENSSSCT00000004540	NUP43	intron	0.13	rs792767079	2
AAAC.GTTT	1	18677125	18677140	4	ENSSSCT00000023948	.	intron	4.141	rs787759203	2
AAAC.GTTT	1	18677125	18677140	4	ENSSSCT00000024741	PPIL4	intron	4.141	rs787759203	2
AAAAC.GTTTT	1	19290976	19290999	4.8	.	.	intergenic	-0.059	rs787645188	2
AAAC.GTTT	1	19333443	19333460	4.5	ENSSSCT00000024450	.	intron	-0.315	.	2
AAAT.ATTT	1	19528921	19528945	6.25	.	.	intergenic	0.109	rs788127697	2
AAAC.GTTT	1	19833214	19833239	6.5	.	.	intergenic	.	123306 rs7937	2
AAAG.CTTT	1	20078640	20078662	5.75	.	.	intergenic	0.149	rs788232476	2
AT.AT	1	20307776	20307807	16	.	.	intergenic	0.083	rs787681869	2
AAAAT.ATTTT	1	20409587	20409601	3	.	.	intergenic	.	rs788275668	2
AAT.ATT	1	20511284	20511298	5	.	.	intergenic	0.027	.	3
AT.AT	1	20619863	20619873	5.5	ENSSSCT00000004548	.	intron	.	rs792095552	2
AT.AT	1	20828987	20829001	7.5	.	.	intergenic	.	rs786808095	3
AAAT.ATTT	1	20921656	20921684	7.25	.	.	intergenic	-0.034	rs788394548	2
AG.CT	1	21268672	21268694	11.5	.	.	intergenic	-0.001	rs792573785	2
AAAC.GTTT	1	21500670	21500688	4.75	ENSSSCT00000004554	GRM1	intron	-0.067	rs790826708	2
AC.GT	1	21532888	21532920	16.5	ENSSSCT00000004554	GRM1	intron	0.332	590770 rs7874	4
AAAC.GTTT	1	21542962	21542978	4.25	ENSSSCT00000004554	GRM1	intron	.	rs792676375	2
AAC.GTT	1	21700070	21700084	5	ENSSSCT00000004554	GRM1	intron	0.084	rs793139146	2
AAAT.ATTT	1	21742006	21742026	5.25	ENSSSCT00000004554	GRM1	intron	0.206	rs787679935	2
AT.AT	1	21778438	21778448	5.5	ENSSSCT00000004554	GRM1	intron	0.068	.	3
AT.AT	1	21913731	21913741	5.5	ENSSSCT00000004555	SHPRH	intron	0.068	.	2
AAC.GTT	1	21951985	21952007	7.66667	.	.	intergenic	.	.	2
AAT.ATT	1	22044955	22044969	5	.	.	intergenic	0.141	rs791863290	2
AG.CT	1	22056323	22056338	8	ENSSSCT00000004557	EPM2A	intron	0.127	rs787195960	2
AC.GT	1	22192928	22192940	6.5	.	.	intergenic	0.006	rs788252448	4
AAAC.GTTT	1	22218097	22218121	6.25	.	.	intergenic	.	428732 rs7927	2
AAC.GTT	1	22359082	22359100	6.33333	.	.	intergenic	0.22	rs792767503	2
AC.GT	1	22974449	22974468	10	.	.	intergenic	0.025	201761 rs7892	2
AAC.GTT	1	22985964	22985980	5.66667	.	.	intergenic	0.065	rs789724657	2
AT.AT	1	23021299	23021309	5.5	ENSSSCT00000004558	.	intron	0.086	rs791010547	2
AC.GT	1	23277319	23277345	13.5	.	.	intergenic	0.007	rs787716774	2
AC.GT	1	23456794	23456806	6.5	.	.	intergenic	.	rs791239132	2
AAAG.CTTT	1	23476616	23476641	6.5	.	.	intergenic	0.14	046967 rs7874	2
AG.CT	1	24048324	24048333	5	.	.	intergenic	.	rs706863033	4
AAAAC.GTTTT	1	24066376	24066394	3.8	.	.	intergenic	.	.	2
AT.AT	1	24086598	24086612	7.5	.	.	intergenic	0.202	926209 rs7902	2
AAAC.GTTT	1	24454377	24454397	5.25	.	.	intergenic	-0.157	rs788971360	2
AT.AT	1	24457904	24457924	10.5	.	.	intergenic	-0.039	rs790840906	2
AAAC.GTTT	1	25210781	25210795	3.75	.	.	intergenic	0.084	rs788312525	2
AAT.ATT	1	25572131	25572151	7	.	.	intergenic	-0.01	rs788738300	2
AAAT.ATTT	1	25814878	25814895	4.5	ENSSSCT00000004576	NMBR	intron	-0.043	.	2
AC.GT	1	26089473	26089501	14.5	.	.	intergenic	.	rs793098374	2
AC.GT	1	26292786	26292796	5.5	.	.	intergenic	0.172	.	2
AC.GT	1	26440793	26440805	6.5	.	.	intergenic	-0.099	538694 rs7912	2
AAAG.CTTT	1	26610706	26610724	4.75	.	.	intergenic	-0.02	rs789844007	2
AT.AT	1	26832123	26832132	5	.	.	intergenic	0.115	.	2
AT.AT	1	26958549	26958582	17	.	.	intergenic	0.067	043435 rs7882	2
AAC.GTT	1	27308104	27308123	6.66667	.	.	intergenic	0.022	.	3
AG.CT	1	27348221	27348231	5.5	.	.	intergenic	0.09	177742 rs7875	3
AT.AT	1	27376830	27376847	9	.	.	intergenic	-0.256	rs786224763	2
AAAC.GTTT	1	27580717	27580745	7.25	.	.	intergenic	-0.137	rs788389829	3
AC.GT	1	27604864	27604877	7	.	.	intergenic	1.286	266483 rs7868	2
AG.CT	1	27705511	27705521	5.5	.	.	intergenic	-0.118	.	2
AT.AT	1	27835615	27835624	5	.	.	intergenic	0.056	rs787806904	2
AG.CT	1	27871328	27871337	5	.	.	intergenic	0.207	rs789872320	2
AAG.CTT	1	27881079	27881096	6	.	.	intergenic	0.174	088913 rs7901	2
ATC.GAT	1	28304800	28304821	7.33333	.	.	intergenic	-0.022	rs693039343	2
AAG.CTT	1	28727401	28727418	6	ENSSSCT00000004583	ECT2L	intron	-0.067	346615 rs7900	2
AAG.CTT	1	28727401	28727418	6	ENSSSCT00000023305	ECT2L	intron	-0.067	346615 rs7900	2
AAAC.GTTT	1	28767210	28767228	4.75	ENSSSCT00000004583	ECT2L	promoter	0.057	rs787937088	2
AT.AT	1	28773510	28773536	13.5	.	.	intergenic	0.003	584548 rs7876	4
AAAC.GTTT	1	28894274	28894292	4.75	ENSSSCT00000004585	NHSL1	intron	0.196	740607 rs7890	4
AGC.GCT	1	28948150	28948167	6	ENSSSCT00000004585	NHSL1	intron	-0.389	587813 rs7880	2
AC.GT	1	29013044	29013054	5.5	ENSSSCT00000004585	NHSL1	intron	0.189	rs791549308	2
AAAT.ATTT	1	29034708	29034731	6	ENSSSCT00000004585	NHSL1	intron	0.028	798660 rs7879	2
AAAG.CTTT	1	29084804	29084831	7	ENSSSCT00000004585	NHSL1	intron	.	235900 rs7876	2
AT.AT	1	29087476	29087513	19	ENSSSCT00000004585	NHSL1	intron	0.154	rs787786393	2
AAC.GTT	1	29097477	29097490	4.66667	ENSSSCT00000004585	NHSL1	intron	0.172	rs792277197	2

AGAT.ATCT	1	29519836	29519854	4.75	.	.	intergenic	.	.	2
AAT.ATT	1	29698715	29698731	5.66667	.	.	intergenic	-0.136	.	3
AG.CT	1	29745805	29745818	7	.	.	intergenic	-0.162	rs791800352	2
AT.AT	1	29812304	29812316	6.5	.	.	intergenic	-0.048	rs786479551	2
AG.CT	1	30174340	30174354	7.5	.	.	intergenic	-0.036	.	3
AG.CT	1	30200631	30200649	9.5	.	.	intergenic	-0.103	rs792468410	3
AT.AT	1	30447306	30447320	7.5	.	.	intergenic	.	503564 rs7927	2
AAT.ATT	1	30811903	30811919	5.66667	ENSSSCT00000025418	.	intron	0.346	rs789892211	2
AAT.ATT	1	30811903	30811919	5.66667	ENSSSCT00000034480	MAP3K5	intron	0.346	rs789892211	2
AAC.GTT	1	30900483	30900497	5	ENSSSCT00000025418	.	intron	.	378393 rs7932	2
AAAT.ATTT	1	31006824	31006839	4	ENSSSCT00000025418	.	intron	0.311	rs789938523	2
AAC.GTT	1	31039621	31039637	5.66667	.	.	intergenic	0.264	rs791577097	2
AG.CT	1	31105306	31105316	5.5	.	.	intergenic	-0.162	585323 rs7882	2
AAAC.GTTT	1	31191487	31191507	5.25	.	.	intergenic	0.033	rs786286157	2
AC.GT	1	31295451	31295462	6	.	.	intergenic	.	rs695309575	2
AC.GT	1	31461209	31461221	6.5	.	.	intergenic	0.318	rs792518567	3
AC.GT	1	31466850	31466862	6.5	.	.	intergenic	0.004	rs691964012	2
AAAAT.ATTTT	1	31617834	31617851	3.6	.	.	intergenic	0.172	rs791663684	3
AC.GT	1	31631155	31631167	6.5	.	.	intergenic	0.096	.	3
AAAG.CTTT	1	31803525	31803551	6.75	.	.	intergenic	0.468	794696 rs7910	3
AAAG.CTTT	1	31826720	31826734	3.75	.	.	intergenic	0.184	rs792727813	2
AC.GT	1	31939315	31939329	7.5	.	.	intergenic	0.184	rs793539951	2
AG.CT	1	31964740	31964755	8	.	.	intergenic	0.095	469178 rs7872	2
AT.AT	1	32037086	32037097	6	.	.	intergenic	0.065	rs791285537	3
AAAT.ATTT	1	32360390	32360412	5.75	.	.	intergenic	0.16	.	2
AAAT.ATTT	1	32402539	32402555	4.25	.	.	intergenic	0.307	rs792721706	2
AC.GT	1	32483080	32483096	8.5	ENSSSCT00000004608	HBS1L	intron	0.1	.	3
AAAC.GTTT	1	32554196	32554222	6.75	ENSSSCT00000029261	.	intron	-0.018	rs788908377	2
AT.AT	1	32870857	32870866	5	.	.	intergenic	0.022	rs789465370	2
AC.GT	1	32938888	32938907	10	.	.	intergenic	0.109	rs793587743	2
AG.CT	1	32972569	32972579	5.5	.	.	intergenic	.	rs789858920	2
AC.GT	1	33044810	33044822	6.5	.	.	intergenic	.	rs789977224	2
AAC.GTT	1	33103392	33103420	9.66667	.	.	intergenic	0.129	rs696764037	2
AC.GT	1	33238752	33238762	5.5	.	.	intergenic	.	955231 rs7924	3
AG.CT	1	33355327	33355337	5.5	ENSSSCT00000004611	SLC2A12	intron	0.165	900187 rs7894	3
AT.AT	1	33448520	33448531	6	.	.	intergenic	0.082	566407 rs6963	3
AAAAC.GTTTT	1	33663032	33663074	8.6	.	.	intergenic	0.122	rs692570749	2
AAAAC.GTTTT	1	33690056	33690076	4.2	.	.	intergenic	0.193	180519 rs7879	2
AT.AT	1	33975908	33975918	5.5	.	.	intergenic	0.025	rs787694904	2
AC.GT	1	34001299	34001311	6.5	.	.	intergenic	-0.218	rs702970519	2
AAC.GTT	1	34169566	34169580	5	.	.	intergenic	0.747	rs792874633	2
AAC.GTT	1	34244945	34244968	8	.	.	intergenic	.	rs791734867	2
AAAG.CTTT	1	34361506	34361523	4.5	.	.	intergenic	0.028	.	2
AAAAAC.GTTTTT	1	34703924	34703943	3.33333	ENSSSCT00000004629	STX7	promoter	.	rs786759960	2
AAAAC.GTTTT	1	34917240	34917259	4	.	.	intergenic	0.136	rs791576210	2
AG.CT	1	35014407	35014428	11	.	.	intergenic	0.098	.	2
AC.GT	1	35037599	35037612	7	.	.	intergenic	-0.124	rs790592337	2
AG.CT	1	35604637	35604648	6	.	.	intergenic	.	rs788086882	2
AAAAT.ATTTT	1	35707441	35707460	4	.	.	intergenic	-0.028	rs790491269	2
AC.GT	1	35781903	35781938	18	.	.	intergenic	0.204	.	3
AC.GT	1	36220332	36220344	6.5	.	.	intergenic	0.205	780242 rs7893	2
AC.GT	1	36257153	36257185	16.5	.	.	intergenic	0.04	rs791413266	2
AG.CT	1	36387088	36387100	6.5	.	.	intergenic	.	096972 rs7933	4
AAAC.GTTT	1	36418105	36418122	4.5	.	.	intergenic	0.432	rs792313104	2
ACCC.GGGT	1	36499250	36499264	3.75	.	.	intergenic	-0.137	rs792523257	2
ACATAT.ATATGT	1	36577899	36577927	4.83333	.	.	intergenic	0.06	rs789959299	2
AAT.ATT	1	36779877	36779900	8	.	.	intergenic	.	rs790741775	2
AG.CT	1	36914123	36914133	5.5	.	.	intergenic	-0.196	.	2
AG.CT	1	36951166	36951175	5	.	.	intergenic	-0.134	.	2
AG.CT	1	36972239	36972248	5	.	.	intergenic	0.419	388982 rs7881	2
AAAC.GTTT	1	36998013	36998048	9	.	.	intergenic	.	rs790793036	3
AT.AT	1	37114618	37114644	13.5	ENSSSCT00000004646	.	intron	-0.136	.	4
AAAAAT.ATTTTT	1	37126147	37126168	3.66667	ENSSSCT00000004646	.	intron	0.187	716399 rs7911	2
AC.GT	1	37469021	37469044	12	ENSSSCT00000004648	.	intron	0.054	rs789237962	2
AC.GT	1	37501715	37501725	5.5	ENSSSCT00000004648	.	intron	0.093	rs789994649	2
AC.GT	1	37979270	37979294	12.5	.	.	intergenic	.	rs790658527	2
ACCT.AGGT	1	38408590	38408616	6.75	ENSSSCT00000004650	PTPRK	intron	0.519	rs793397346	2
AAAAG.CTTTT	1	38665448	38665488	8.2	ENSSSCT00000004650	PTPRK	intron	0.057	rs698167926	2
AT.AT	1	38668295	38668306	6	ENSSSCT00000004650	PTPRK	intron	.	rs789406737	2
AAAC.GTTT	1	38694394	38694430	9.25	ENSSSCT00000004650	PTPRK	intron	0.038	rs793156035	2
AT.AT	1	38731088	38731102	7.5	ENSSSCT00000004650	PTPRK	intron	-0.038	rs789238536	2
AAAAT.ATTTT	1	38790209	38790238	6	ENSSSCT00000004650	PTPRK	intron	4.065	rs792994735	2
AAAC.GTTT	1	38790423	38790444	5.5	ENSSSCT00000004650	PTPRK	intron	3.643	.	2
AC.GT	1	39040859	39040885	13.5	.	.	intergenic	0.018	rs787402741	2
AAAT.ATTT	1	39292573	39292588	4	.	.	intergenic	.	403547 rs7880	2
AG.CT	1	39425336	39425353	9	ENSSSCT00000004657	.	intron	0.027	394044 rs7869	2
AT.AT	1	39625199	39625213	7.5	.	.	intergenic	0.16	rs786232848	4
AATT.AATT	1	39838847	39838872	6.5	.	.	intergenic	-0.046	rs793138401	2
AC.GT	1	39847813	39847843	15.5	.	.	intergenic	0.16	rs790933277	3
AAAT.ATTT	1	39872694	39872717	6	.	.	intergenic	0.095	rs792178259	2
AAT.ATT	1	39873337	39873368	10.6667	.	.	intergenic	0.127	rs789883712	2
AT.AT	1	40017272	40017297	13	.	.	intergenic	0.172	427596 rs7938	2
AAC.GTT	1	40201536	40201555	6.66667	.	.	intergenic	0.128	rs786287949	2
AAAAC.GTTTT	1	40218686	40218710	5	.	.	intergenic	.	rs792066293	2

AAAAC.GTTTT	1	40581538	40581566	5.8	.	.	intergenic	-0.154	rs791198671	2
AC.GT	1	40607227	40607241	7.5	.	.	intergenic	-0.028	rs792296636	3
AC.GT	1	40934783	40934801	9.5	ENSSSCT00000027938	NCOA7	intron	-0.106	rs791588396	2
ACTCT.AGAGT	1	41026982	41026996	3	.	.	intergenic	0.193	rs789251351	3
AAAC.GTTT	1	41479169	41479192	6	.	.	intergenic	-0.083	rs789018301	2
AC.GT	1	42040798	42040807	5	.	.	intergenic	0.247	rs793294116	2
AC.GT	1	42327669	42327679	5.5	.	.	intergenic	-0.124	rs792718851	2
AAC.GTT	1	42448247	42448260	4.66667	.	.	intergenic	0.71	rs791578037	2
AAAAT.ATTTT	1	42714130	42714146	3.4	.	.	intergenic	0.105	rs792758029	2
AAAC.GTTT	1	42874049	42874063	3.75	.	.	intergenic	-0.005	rs789692543	2
ACGC.GCGT	1	42938175	42938193	4.75	.	.	intergenic	-0.18	rs792144167	2
AT.AT	1	42958453	42958462	5	.	.	intergenic	-0.179	rs790256328	2
AAT.ATT	1	43186991	43187014	8	.	.	intergenic	-0.08	.	2
AG.CT	1	43275152	43275161	5	.	.	intergenic	0.275	rs793015065	2
AAAC.GTTT	1	43297277	43297303	6.75	.	.	intergenic	0.192	rs791827495	2
AC.GT	1	43466116	43466134	9.5	.	.	intergenic	0.531	183380 rs7876	2
AAT.ATT	1	43502935	43502948	4.66667	ENSSSCT00000029492	TRDN	intron	-0.221	.	2
AC.GT	1	43566732	43566744	6.5	.	.	intergenic	-0.131	rs790494770	2
AC.GT	1	43616051	43616071	10.5	.	.	intergenic	-0.144	rs787543403	2
AAAT.ATTT	1	43697562	43697579	4.5	.	.	intergenic	.	rs791628028	2
AAAAG.CTTTT	1	43932329	43932353	5	ENSSSCT00000004677	CLVS2	intron	-0.009	167133 rs7894	2
AC.GT	1	43937111	43937124	7	ENSSSCT00000004677	CLVS2	intron	0.044	.	3
AAC.GTT	1	43974992	43975005	4.66667	ENSSSCT00000004677	CLVS2	intron	0.139	rs791614704	2
AAT.ATT	1	44089536	44089552	5.66667	ENSSSCT00000033012	SMPDL3A	intron	-0.154	rs788892991	2
AAT.ATT	1	44089536	44089552	5.66667	ENSSSCT00000034434	SMPDL3A	intron	-0.154	rs788892991	2
AAT.ATT	1	44089536	44089552	5.66667	ENSSSCT00000004678	SMPDL3A	intron	-0.154	rs788892991	2
AAAC.GTTT	1	44159818	44159848	7.75	.	.	intergenic	.	rs789277234	2
AAAC.GTTT	1	44171450	44171472	5.75	.	.	intergenic	0.08	rs786973217	2
AG.CT	1	44736625	44736654	15	.	.	intergenic	-0.029	322824 rs7867	2
AC.GT	1	45030265	45030278	7	.	.	intergenic	-0.049	rs793291164	2
AAAT.ATTT	1	45163763	45163786	6	.	.	intergenic	-0.062	071294 rs7906	2
AAAC.GTTT	1	45175722	45175740	4.75	.	.	intergenic	0.125	rs788602046	2
AT.AT	1	45222384	45222406	11.5	.	.	intergenic	0.009	rs788705357	2
AAC.GTT	1	45252182	45252196	5	.	.	intergenic	0.138	rs786255226	2
AAAC.GTTT	1	45333005	45333018	3.5	.	.	intergenic	0.03	rs787514789	2
AATT.AATT	1	45433128	45433149	5.5	.	.	intergenic	-0.015	rs789200495	2
AG.CT	1	45499676	45499688	6.5	.	.	intergenic	1.35	.	2
AAAT.ATTT	1	45739199	45739213	3.75	ENSSSCT00000004689	TBC1D32	intron	.	rs788758978	2
AAAC.GTTT	1	45784509	45784555	11.75	ENSSSCT00000004689	TBC1D32	intron	0.099	rs788627854	2
AT.AT	1	45792245	45792260	8	ENSSSCT00000004689	TBC1D32	intron	.	rs788964391	2
AAAT.ATTT	1	45807102	45807128	6.75	ENSSSCT00000004689	TBC1D32	intron	.	rs792553793	2
AG.CT	1	45981913	45981925	6.5	.	.	intergenic	0.369	.	4
AAC.GTT	1	45990524	45990539	5.33333	.	.	intergenic	0.068	230982 rs7875	2
AT.AT	1	46224422	46224432	5.5	.	.	intergenic	0.03	rs791374551	2
AAC.GTT	1	46480135	46480157	7.66667	.	.	intergenic	.	rs792875546	2
AC.GT	1	47167554	47167565	6	.	.	intergenic	0.055	.	3
AAAT.ATTT	1	47340700	47340716	4.25	.	.	intergenic	-0.033	531314 rs7915	2
AAAC.GTTT	1	47406087	47406113	6.75	.	.	intergenic	-0.127	367789 rs7884	4
AAC.GTT	1	47496552	47496570	6.33333	.	.	intergenic	0.08	350954 rs7938	2
AAAAC.GTTTT	1	47984615	47984632	3.6	ENSSSCT00000004691	MAN1A1	intron	0.031	.	2
AG.CT	1	48202205	48202235	15.5	ENSSSCT00000004692	FAM184A	intron	0.123	125083 rs7919	2
AT.AT	1	48281554	48281564	5.5	.	.	intergenic	.	rs793280148	2
AAAC.GTTT	1	48333168	48333186	4.75	ENSSSCT00000004693	MCM9	intron	0.134	259831 rs7932	3
AAAC.GTTT	1	48592701	48592720	5	.	.	intergenic	0.285	rs787781254	2
AAG.CTT	1	48819457	48819473	5.66667	.	.	intergenic	0.182	471355 rs7934	2
AATG.CATT	1	48984816	48984847	8	ENSSSCT00000004697	SLC35F1	intron	-0.049	rs793239753	2
AAAAC.GTTTT	1	49028360	49028377	3.6	ENSSSCT00000004697	SLC35F1	intron	-0.14	rs793206570	2
AAAAC.GTTTT	1	49257643	49257666	4.8	.	.	intergenic	0.267	rs789687289	2
AC.GT	1	49312173	49312188	8	.	.	intergenic	0.763	300714 rs7929	2
AT.AT	1	49540762	49540776	7.5	.	.	intergenic	.	rs792540454	4
AATAC.GTATT	1	49565727	49565755	5.8	ENSSSCT00000004699	.	intron	0.192	rs786871772	2
AT.AT	1	49591149	49591158	5	ENSSSCT00000004701	GOPC	intron	0.027	rs791762296	2
AAAG.CTTT	1	49594084	49594097	3.5	ENSSSCT00000004701	GOPC	intron	.	rs788965010	2
AAAAC.GTTTT	1	49723035	49723052	3.6	.	.	intergenic	.	731484 rs7863	2
AAC.GTT	1	49781713	49781736	8	ENSSSCT00000029401	ROS1	intron	1.769	rs793825776	2
AT.AT	1	49814565	49814591	13.5	ENSSSCT00000029401	ROS1	intron	0.236	299273 rs7930	2
AG.CT	1	49816006	49816016	5.5	ENSSSCT00000029401	ROS1	intron	-0.052	rs791713439	3
AG.CT	1	49821229	49821241	6.5	ENSSSCT00000029401	ROS1	intron	0.072	792624 rs7867	2
AAT.ATT	1	49881944	49881973	10	ENSSSCT00000004703	VGLL2	intron	0.237	270292 rs7912	2
AAAC.GTTT	1	50031303	50031325	5.75	.	.	intergenic	0.393	rs792271105	2
AT.AT	1	50090476	50090496	10.5	.	.	intergenic	0.009	rs789890725	2
AT.AT	1	50112443	50112457	7.5	.	.	intergenic	.	rs792997640	2
AAC.GTT	1	50965905	50965928	8	.	.	intergenic	0.115	418470 rs7937	3
AT.AT	1	51030264	51030275	6	.	.	intergenic	-0.286	.	2
AAC.GTT	1	51415020	51415035	5.33333	.	.	intergenic	-0.112	rs787793039	2
AAAT.ATTT	1	51490970	51490987	4.5	.	.	intergenic	.	rs790714614	2
AAAC.GTTT	1	51535089	51535103	3.75	.	.	intergenic	-0.25	rs787218245	2
AT.AT	1	51568236	51568249	7	.	.	intergenic	0.066	326012 rs7891	2
AC.GT	1	51820026	51820040	7.5	.	.	intergenic	.	rs787905262	2
AT.AT	1	51998574	51998585	6	.	.	intergenic	.	rs789870168	3
AAAAT.ATTTT	1	52085844	52085865	4.4	.	.	intergenic	-0.016	278586 rs7906	2
AAAC.GTTT	1	52148276	52148290	3.75	.	.	intergenic	-0.063	rs788204617	2
AC.GT	1	52149954	52149972	9.5	.	.	intergenic	-0.078	.	4
AAAC.GTTT	1	52418691	52418717	6.75	.	.	intergenic	0.499	rs788152693	2

AG.CT	1	52433160	52433172	6.5	.	.	intergenic	0.241	rs792664890	4
AAAC.GTTT	1	52521132	52521150	4.75	.	.	intergenic	0.037	rs786645131	2
AT.AT	1	52602233	52602244	6	.	.	intergenic	-0.011	.	3
AC.GT	1	52609675	52609687	6.5	.	.	intergenic	0.272	rs787436227	4
AG.CT	1	52809987	52810009	11.5	.	.	intergenic	.	rs792994759	2
AAAT.ATTT	1	52961824	52961844	5.25	.	.	intergenic	.	.	2
AAAT.ATTT	1	52981958	52982001	11	.	.	intergenic	-0.151	rs786384731	2
AAT.ATT	1	53063505	53063531	9	.	.	intergenic	.	rs792150368	2
AAAT.ATTT	1	53152663	53152679	4.25	.	.	intergenic	.	rs791317766	2
AG.CT	1	53411464	53411473	5	.	.	intergenic	.	.	3
AAGG.CCTT	1	53665506	53665521	4	.	.	intergenic	-0.053	rs693352739	2
AAC.GTT	1	54007386	54007401	5.33333	.	.	intergenic	.	rs791944749	2
AT.AT	1	54125420	54125431	6	.	.	intergenic	-0.046	)80604 rs7871	2
AT.AT	1	54756818	54756829	6	.	.	intergenic	0.213	rs790436411	2
AAAAC.GTTTT	1	54766526	54766545	4	.	.	intergenic	.	392297 rs7901	2
AC.GT	1	54829662	54829675	7	.	.	intergenic	.	.	3
AAAC.GTTT	1	54853395	54853407	3.25	.	.	intergenic	.	rs787786107	3
AAAT.ATTT	1	55042066	55042088	5.75	.	.	intergenic	.	rs786786479	2
AG.CT	1	55068231	55068243	6.5	.	.	intergenic	0.284	.	3
AT.AT	1	55088385	55088394	5	.	.	intergenic	-0.109	rs788165428	2
AT.AT	1	55292363	55292372	5	.	.	intergenic	0.05	rs789434919	2
AT.AT	1	55388423	55388435	6.5	ENSSSCT00000004719	BAI3	intron	0.021	rs788590703	2
AAAC.GTTT	1	55419931	55419949	4.75	ENSSSCT00000004719	BAI3	intron	.	rs789873526	2
AT.AT	1	55557246	55557257	6	ENSSSCT00000004719	BAI3	intron	.	)93585 rs7936	2
AAAAC.GTTTT	1	55674261	55674289	5.8	ENSSSCT00000004719	BAI3	intron	-0.037	rs788855722	2
AAC.GTT	1	55842785	55842797	4.33333	.	.	intergenic	.	rs793884080	2
AT.AT	1	55949280	55949296	8.5	.	.	intergenic	0.08	rs787560591	2
AAAC.GTTT	1	55978380	55978406	6.75	.	.	intergenic	0.11	rs790761851	2
AAAAAC.GTTTTT	1	56065330	56065352	3.83333	ENSSSCT00000004721	LMBRD1	intron	0.066	rs790238799	2
AG.CT	1	56322836	56322859	12	ENSSSCT00000004722	COL19A1	intron	0.017	rs790666988	2
AAAAC.GTTTT	1	56766781	56766802	4.4	ENSSSCT00000004723	COL9A1	intron	0.301	rs786217443	2
AT.AT	1	56835837	56835846	5	.	.	intergenic	0.153	.	3
AAATAG.CTATTT	1	57020516	57020535	3.33333	.	.	intergenic	0.086	rs786972921	2
AT.AT	1	57284988	57284997	5	ENSSSCT00000024170	B3GAT2	intron	.	.	2
AAAC.GTTT	1	57345496	57345518	5.75	ENSSSCT00000024170	B3GAT2	intron	.	rs791689238	2
ACAT.ATGT	1	57388624	57388644	5.25	.	.	intergenic	.	rs789294864	2
AAAAT.ATTTT	1	57394415	57394430	3.2	.	.	intergenic	.	rs789500911	2
AC.GT	1	57576736	57576746	5.5	.	.	intergenic	0.214	rs793791655	2
AAT.ATT	1	57619904	57619922	6.33333	.	.	intergenic	.	rs789518475	2
ATC.GAT	1	57823485	57823505	7	.	.	intergenic	-0.005	rs787917129	2
AG.CT	1	57857687	57857705	9.5	.	.	intergenic	-0.077	431688 rs7934	2
AAAC.GTTT	1	57899650	57899673	6	.	.	intergenic	.	rs786533512	2
AT.AT	1	57901826	57901844	9.5	.	.	intergenic	-0.019	.	2
AAAAG.CTTTT	1	57939720	57939741	4.4	.	.	intergenic	0.641	rs704974494	2
AG.CT	1	58141913	58141928	8	.	.	intergenic	0.261	rs787229297	4
AG.CT	1	58320254	58320265	6	.	.	intergenic	0.032	)97603 rs7862	2
AC.GT	1	58404370	58404380	5.5	ENSSSCT00000004730	RIMS1	intron	0.053	rs792964788	3
AG.CT	1	58566974	58566993	10	ENSSSCT00000004730	RIMS1	intron	-0.3	rs790102141	2
AAAC.GTTT	1	58636080	58636096	4.25	.	.	intergenic	0.023	rs787043340	2
AAAG.CTTT	1	58671786	58671805	5	.	.	intergenic	0.157	328174 rs7938	2
ACC.GGT	1	58866687	58866703	5.66667	.	.	intergenic	0.023	rs791299335	2
AAAAC.GTTTT	1	59037392	59037421	6	.	.	intergenic	.	rs788144130	2
AAT.ATT	1	59051657	59051668	4	.	.	intergenic	0.088	rs791788582	2
AAAG.CTTT	1	59092158	59092176	4.75	.	.	intergenic	.	rs792035886	2
AAAAC.GTTTT	1	59328526	59328541	3.2	.	.	intergenic	.	rs788688388	2
AT.AT	1	59366349	59366359	5.5	ENSSSCT00000004740	DDX43	intron	-0.184	rs789954417	2
AT.AT	1	59366349	59366359	5.5	ENSSSCT00000023321	CYB5R4	intron	-0.184	rs789954417	2
AAC.GTT	1	59551967	59551979	4.33333	.	.	intergenic	.	.	2
AC.GT	1	59739540	59739552	6.5	ENSSSCT00000004742	KIAA1009	intron	0.109	rs786526874	2
AC.GT	1	60039276	60039302	13.5	.	.	intergenic	0.201	rs787927710	2
AT.AT	1	60214391	60214403	6.5	ENSSSCT00000004744	TBX18	intron	0.38	.	4
AAT.ATT	1	60558740	60558758	6.33333	.	.	intergenic	0.097	.	2
ATCC.GGAT	1	60751412	60751439	7	.	.	intergenic	-0.076	I98232 rs7877	2
AAAAT.ATTTT	1	60889906	60889930	5	.	.	intergenic	0.178	rs792451913	2
AAT.ATT	1	60905397	60905414	6	ENSSSCT00000004747	SNX14	intron	1.934	372845 rs7929	2
AAAT.ATTT	1	61006591	61006617	6.75	ENSSSCT00000004748	SYNCRIP	intron	4.12	)93812 rs7932	2
AAAC.GTTT	1	61539589	61539611	5.75	.	.	intergenic	0.1	rs789077953	2
AC.GT	1	61556464	61556478	7.5	.	.	intergenic	0.124	rs791191714	2
AG.CT	1	62012587	62012603	8.5	.	.	intergenic	0.563	rs792872172	3
AT.AT	1	62024381	62024395	7.5	.	.	intergenic	.	.	3
AAAG.CTTT	1	62062816	62062831	4	.	.	intergenic	0.035	.	2
AAAT.ATTT	1	62379993	62380006	3.5	ENSSSCT00000029112	ZNF292	intron	0.293	rs786818103	2
AAAT.ATTT	1	62407926	62407947	5.5	.	.	intergenic	-0.049	rs786672980	2
AAAC.GTTT	1	62436537	62436556	5	.	.	intergenic	0.166	I46663 rs7917	2
AATT.AATT	1	62440067	62440089	5.75	.	.	intergenic	0.159	rs793147507	2
AAAC.GTTT	1	62790015	62790030	4	ENSSSCT00000004762	AKIRIN2	3'utr	.	573312 rs7928	2
AT.AT	1	62817841	62817866	13	.	.	intergenic	0.2	.	2
AAAAT.ATTTT	1	62900592	62900606	3	.	.	intergenic	0.027	rs792325963	2
AG.CT	1	63071982	63071992	5.5	.	.	intergenic	0.009	.	3
AAAAC.GTTTT	1	63307658	63307681	4.8	.	.	intergenic	0.216	rs789573594	2
AC.GT	1	63374393	63374402	5	.	.	intergenic	-0.222	rs788338558	2
AAG.CTT	1	63383771	63383782	4	.	.	intergenic	0.398	)59288 rs7888	4
AC.GT	1	63447148	63447162	7.5	.	.	intergenic	0.32	rs793252978	2
AAAAC.GTTTT	1	63473963	63473986	4.8	.	.	intergenic	0.182	rs788417584	2

AAAAC.GTTTT	1	63673626	63673642	3.4	ENSSSCT00000029164	.	intron	.	rs789276901	2
AC.GT	1	64362444	64362456	6.5	.	.	intergenic	0.275	rs789515298	2
AT.AT	1	64741617	64741628	6	ENSSSCT00000027915	.	intron	.	rs793485844	3
AT.AT	1	64741617	64741628	6	ENSSSCT00000004785	.	intron	.	rs793485844	3
AAC.GTT	1	64979479	64979491	4.33333	ENSSSCT00000004788	BACH2	intron	0.305	rs786290649	2
AAAAC.GTTTT	1	65192289	65192304	3.2	.	.	intergenic	-0.089	rs791835771	2
AAAAC.GTTTT	1	65224399	65224422	4.8	.	.	intergenic	-0.044	rs792420111	2
AATG.CATT	1	65311927	65311947	5.25	.	.	intergenic	0.031	rs793870556	2
AAAG.CTTT	1	65367475	65367518	11	.	.	intergenic	0.314	rs790552560	2
AG.CT	1	65763250	65763259	5	.	.	intergenic	.	rs793044469	2
AC.GT	1	66048304	66048320	8.5	.	.	intergenic	0.164	)01117 rs7915	4
AT.AT	1	66123484	66123494	5.5	.	.	intergenic	0.151	rs790986049	2
AC.GT	1	66224158	66224190	16.5	.	.	intergenic	0.034	rs793249009	4
AT.AT	1	66265022	66265035	7	.	.	intergenic	0.025	rs793733624	2
AT.AT	1	66445628	66445644	8.5	.	.	intergenic	0.119	rs788273912	2
AC.GT	1	66457452	66457466	7.5	.	.	intergenic	0.537	398088 rs7930	2
AC.GT	1	66744550	66744591	21	.	.	intergenic	-0.15	505125 rs7884	2
AAAT.ATTT	1	67058682	67058696	3.75	.	.	intergenic	.	rs790428743	3
AT.AT	1	67244439	67244450	6	.	.	intergenic	-0.061	.	2
AAAAG.CTTTT	1	67359354	67359369	3.2	.	.	intergenic	-0.025	rs791119865	2
AAG.CTT	1	67549563	67549576	4.66667	.	.	intergenic	.	rs789044493	2
AT.AT	1	68132123	68132147	12.5	.	.	intergenic	-0.081	rs712254206	2
AAT.ATT	1	68874901	68874912	4	.	.	intergenic	0.032	rs786663249	2
AAAC.GTTT	1	68886557	68886575	4.75	.	.	intergenic	.	142729 rs7871	3
AT.AT	1	69105307	69105325	9.5	.	.	intergenic	-0.021	rs790754580	2
AC.GT	1	69598605	69598618	7	.	.	intergenic	-0.018	rs692181455	3
ATC.GAT	1	69610242	69610264	7.66667	.	.	intergenic	-0.151	.	3
AG.CT	1	69642707	69642732	13	.	.	intergenic	0.006	rs791158789	4
AG.CT	1	69807950	69807961	6	.	.	intergenic	.	.	4
AC.GT	1	70032511	70032524	7	.	.	intergenic	-0.03	rs787530510	2
AG.CT	1	70144263	70144297	17.5	.	.	intergenic	0.039	rs789190993	2
AT.AT	1	70192148	70192160	6.5	.	.	intergenic	.	rs793278755	2
AC.GT	1	70374929	70374948	10	.	.	intergenic	-0.024	344603 rs7872	3
AC.GT	1	70908997	70909008	6	.	.	intergenic	0.101	rs791088007	4
AT.AT	1	71100499	71100512	7	.	.	intergenic	0.095	rs791520505	2
AAT.ATT	1	71129560	71129577	6	.	.	intergenic	0.256	rs791957658	2
AG.CT	1	71138631	71138645	7.5	.	.	intergenic	.	rs786552360	2
ACAT.ATGT	1	71193879	71193899	5.25	.	.	intergenic	.	964225 rs7936	2
AT.AT	1	71430084	71430093	5	.	.	intergenic	0.121	rs789459557	2
AT.AT	1	71452036	71452046	5.5	.	.	intergenic	-0.084	rs789796795	2
AT.AT	1	71629379	71629389	5.5	.	.	intergenic	0.142	.	4
AAG.CTT	1	71851218	71851249	10.6667	.	.	intergenic	0.109	rs789240231	2
ACAG.CTGT	1	71947359	71947377	4.75	.	.	intergenic	0.398	rs790216507	2
AC.GT	1	71994527	71994541	7.5	.	.	intergenic	0.083	.	3
AAAC.GTTT	1	72272987	72273000	3.5	.	.	intergenic	0.148	342770 rs7884	2
AC.GT	1	72380406	72380416	5.5	.	.	intergenic	0.551	rs788473725	2
AT.AT	1	72408881	72408894	7	ENSSSCT00000029077	NDUFAF4	intron	0.083	rs787641866	2
AT.AT	1	72408881	72408894	7	ENSSSCT00000004798	NDUFAF4	intron	0.083	rs787641866	2
AT.AT	1	72883454	72883471	9	ENSSSCT00000004802	.	intron	.	.	3
AAAAAC.GTTTTT	1	72917693	72917715	3.83333	.	.	intergenic	.	rs790450543	2
AAAC.GTTT	1	73691003	73691038	9	.	.	intergenic	0.054	rs793281321	2
AAT.ATT	1	73849452	73849471	6.66667	.	.	intergenic	0.086	.	2
AAAAAT.ATTTTT	1	73874251	73874272	3.66667	.	.	intergenic	-0.179	406965 rs7883	2
AAAT.ATTT	1	73895895	73895909	3.75	.	.	intergenic	0.032	.	2
AG.CT	1	73952002	73952013	6	.	.	intergenic	0.137	rs787510403	2
AAAT.ATTT	1	74214254	74214267	3.5	.	.	intergenic	-0.001	rs790450786	2
AG.CT	1	74215940	74215956	8.5	.	.	intergenic	0.314	rs789160924	3
AC.GT	1	74274815	74274825	5.5	.	.	intergenic	0.21	rs789534369	2
AT.AT	1	74330827	74330836	5	.	.	intergenic	0.114	.	3
AAAAC.GTTTT	1	74453887	74453901	3	ENSSSCT00000004804	FBXL4	intron	0.149	.	2
AGG.CCT	1	74926707	74926721	5	.	.	intergenic	0.07	.	2
AG.CT	1	74942943	74942957	7.5	ENSSSCT00000004805	.	intron	0.08	rs791406515	2
AC.GT	1	75177547	75177565	9.5	.	.	intergenic	.	rs789045638	6
AG.CT	1	75460762	75460779	9	.	.	intergenic	0.176	rs702096286	3
AAC.GTT	1	75634314	75634333	6.66667	.	.	intergenic	.	797363 rs7903	2
AT.AT	1	76097926	76097938	6.5	.	.	intergenic	0.151	rs790737973	3
AG.CT	1	76106519	76106530	6	.	.	intergenic	-0.022	rs788078783	2
AAAC.GTTT	1	76555888	76555909	5.5	.	.	intergenic	.	rs790972507	2
AAAC.GTTT	1	76749396	76749415	5	.	.	intergenic	.	rs789418874	2
AC.GT	1	76947261	76947281	10.5	.	.	intergenic	0.368	033003 rs7898	3
AAAT.ATTT	1	76973342	76973362	5.25	.	.	intergenic	-0.019	.	2
AACT.AGTT	1	77101986	77101999	3.5	.	.	intergenic	.	rs789726133	2
AAAC.GTTT	1	77186796	77186822	6.75	ENSSSCT00000032833	GRIK2	intron	-0.086	208126 rs7865	2
AAAC.GTTT	1	77186796	77186822	6.75	ENSSSCT00000004819	GRIK2	intron	-0.086	208126 rs7865	2
AAAG.CTTT	1	77200534	77200561	7	ENSSSCT00000032833	GRIK2	intron	1.065	271576 rs7891	2
AAAG.CTTT	1	77200534	77200561	7	ENSSSCT00000004819	GRIK2	intron	1.065	271576 rs7891	2
AT.AT	1	77206723	77206749	13.5	ENSSSCT00000032833	GRIK2	intron	0.075	.	2
AT.AT	1	77206723	77206749	13.5	ENSSSCT00000004819	GRIK2	intron	0.075	.	2
AAAAC.GTTTT	1	77223155	77223178	4.8	ENSSSCT00000032833	GRIK2	intron	.	rs786385099	2
AAAAC.GTTTT	1	77223155	77223178	4.8	ENSSSCT00000004819	GRIK2	intron	.	rs786385099	2
AAAT.ATTT	1	77345200	77345220	5.25	ENSSSCT00000032833	GRIK2	intron	0.422	rs790811796	2
AAAT.ATTT	1	77345200	77345220	5.25	ENSSSCT00000004819	GRIK2	intron	0.422	rs790811796	2
AAT.ATT	1	77517036	77517062	9	ENSSSCT00000035045	GRIK2	intron	-0.036	rs787870500	2
AAT.ATT	1	77517036	77517062	9	ENSSSCT00000004819	GRIK2	intron	-0.036	rs787870500	2

AC.GT	1	77967026	77967036	5.5	.	.	intergenic	.	rs787761492	2
AT.AT	1	78067448	78067458	5.5	.	.	intergenic	0.169	rs790838868	4
AG.CT	1	78073332	78073345	7	.	.	intergenic	0.019	rs789803196	2
AG.CT	1	78363036	78363061	13	.	.	intergenic	-0.084	rs790316557	2
AG.CT	1	78376790	78376818	14.5	.	.	intergenic	0.461	rs786837778	2
AG.CT	1	78434834	78434848	7.5	.	.	intergenic	0.511	.	4
AAAAT.ATTTT	1	78444928	78444942	3	.	.	intergenic	0.229	.	2
AAAAAC.GTTTTT	1	78559613	78559638	4.33333	.	.	intergenic	0.287	315042 rs7870	2
AC.GT	1	78621379	78621389	5.5	.	.	intergenic	0.4	.	2
AT.AT	1	78871183	78871197	7.5	.	.	intergenic	-0.019	rs789040157	2
AT.AT	1	78956582	78956608	13.5	.	.	intergenic	.	rs793506424	2
AG.CT	1	79026382	79026400	9.5	.	.	intergenic	.	.	4
AG.CT	1	79096147	79096157	5.5	.	.	intergenic	.	.	4
AC.GT	1	79452380	79452390	5.5	.	.	intergenic	0.098	.	2
AAAAAT.ATTTTT	1	79556831	79556856	4.33333	.	.	intergenic	0.232	rs788252706	2
AC.GT	1	79568793	79568804	6	.	.	intergenic	.	rs791259860	3
AG.CT	1	79622915	79622926	6	.	.	intergenic	.	.	4
AAAT.ATTT	1	79919873	79919895	5.75	.	.	intergenic	0.003	rs788944966	2
AAAAAT.ATTTTT	1	79975403	79975430	4.66667	.	.	intergenic	1.101	rs792619341	2
AAAAG.CTTTT	1	80213002	80213035	6.8	.	.	intergenic	0.19	300033 rs7900	2
AC.GT	1	80294279	80294300	11	ENSSSCT00000004823	HACE1	intron	0.373	rs793069219	2
AAAC.GTTT	1	80409159	80409172	3.5	.	.	intergenic	0.266	504889 rs7866	2
AAAC.GTTT	1	80425859	80425888	7.5	.	.	intergenic	0.247	rs786527673	2
AAAT.ATTT	1	80665016	80665036	5.25	ENSSSCT00000004826	BVES	intron	0.266	rs789174196	2
AAAT.ATTT	1	80665016	80665036	5.25	ENSSSCT00000004825	BVES	intron	0.266	rs789174196	2
AAC.GTT	1	80745434	80745448	5	.	.	intergenic	.	.	3
AT.AT	1	80893253	80893263	5.5	ENSSSCT00000004829	PREP	promoter	0.109	.	2
AAAG.CTTT	1	81155518	81155539	5.5	.	.	intergenic	0.073	.	2
AAAAAT.ATTTTT	1	81286437	81286473	6.16667	.	.	intergenic	.	rs787782609	2
AC.GT	1	81386354	81386364	5.5	.	.	intergenic	0.271	rs791121836	2
AAAC.GTTT	1	81388698	81388720	5.75	.	.	intergenic	-0.28	rs789920509	2
AATG.CATT	1	81883838	81883857	5	.	.	intergenic	0.049	rs787241279	2
AAG.CTT	1	82131564	82131581	6	ENSSSCT00000004835	.	intron	0.376	rs786761030	2
AG.CT	1	82169733	82169744	6	.	.	intergenic	.	rs792644708	2
AT.AT	1	82306148	82306162	7.5	.	.	intergenic	.	130261 rs7866	2
AC.GT	1	82428557	82428571	7.5	.	.	intergenic	-0.148	rs791114306	2
AAAT.ATTT	1	82652788	82652806	4.75	.	.	intergenic	0.211	.	2
AAC.GTT	1	82665154	82665173	6.66667	.	.	intergenic	0.081	.	2
AC.GT	1	82719863	82719876	7	.	.	intergenic	.	rs791464634	2
AC.GT	1	82845362	82845373	6	.	.	intergenic	0.784	rs792805843	2
AT.AT	1	83007068	83007078	5.5	.	.	intergenic	.	rs788674184	2
AT.AT	1	83210423	83210433	5.5	.	.	intergenic	0.02	)88642 rs7887	2
AAAC.GTTT	1	83523580	83523601	5.5	.	.	intergenic	0.248	)68876 rs7886	2
AG.CT	1	83618869	83618893	12.5	.	.	intergenic	0.198	rs788534522	3
AAAAAC.GTTTTT	1	83721148	83721169	3.66667	ENSSSCT00000004848	.	intron	.	rs790787126	2
AT.AT	1	83852158	83852168	5.5	.	.	intergenic	.	rs790709432	2
AAAAC.GTTTT	1	83989803	83989840	7.6	ENSSSCT00000004849	FOXO3A	intron	0.107	.	2
AAAAT.ATTTT	1	84027916	84027937	4.4	ENSSSCT00000004849	FOXO3A	intron	.	rs789098840	2
AATG.CATT	1	84175909	84175936	7	.	.	intergenic	-0.023	rs793573133	2
AC.GT	1	84176691	84176703	6.5	.	.	intergenic	.	.	4
AAAG.CTTT	1	84405669	84405699	7.75	.	.	intergenic	0.292	.	2
AT.AT	1	84512121	84512130	5	ENSSSCT00000004854	CEP57L1	intron	.	.	2
AT.AT	1	84512121	84512130	5	ENSSSCT00000029999	CEP57L1	intron	.	.	2
AAAC.GTTT	1	84655744	84655758	3.75	ENSSSCT00000004878	.	intron	.	rs793515583	2
AC.GT	1	84671844	84671856	6.5	ENSSSCT00000004878	.	intron	-0.052	rs790155291	2
AG.CT	1	84802871	84802881	5.5	.	.	intergenic	0.196	.	3
AAAC.GTTT	1	84877908	84877934	6.75	ENSSSCT00000004874	ZBTB24	promoter	.	rs789530094	2
AGG.CCT	1	85162623	85162635	4.33333	.	.	intergenic	0.359	rs792037026	2
AAAAC.GTTTT	1	85186920	85186936	3.4	.	.	intergenic	0.146	rs789988478	2
AAAC.GTTT	1	85199328	85199371	11	.	.	intergenic	0.312	rs789903018	2
AT.AT	1	86000503	86000512	5	.	.	intergenic	.	rs788967357	2
AAC.GTT	1	86046218	86046229	4	ENSSSCT00000004862	SLC22A16	intron	0.2	rs787798329	2
AAC.GTT	1	86076678	86076700	7.66667	.	.	intergenic	-0.002	)01817 rs7890	2
AAC.GTT	1	86320573	86320596	8	ENSSSCT00000004857	RPF2	intron	-0.167	rs793814625	2
AC.GT	1	86353179	86353193	7.5	ENSSSCT00000004856	GTF3C6	3'utr	0.075	rs788608643	2
AAAAC.GTTTT	1	86921610	86921633	4.8	ENSSSCT00000025331	.	intron	0.324	rs790923989	2
AAG.CTT	1	87126065	87126082	6	.	.	intergenic	0.245	169834 rs7868	2
AC.GT	1	87140027	87140039	6.5	.	.	intergenic	1.152	rs694281725	2
AAAAGC.GCTTTT	1	87249168	87249191	4	ENSSSCT00000004886	FYN	intron	1.697	rs789466128	2
AAAAGC.GCTTTT	1	87249168	87249191	4	ENSSSCT00000004884	FYN	intron	1.697	rs789466128	2
AAAAGC.GCTTTT	1	87249168	87249191	4	ENSSSCT00000004885	FYN	intron	1.697	rs789466128	2
AGAT.ATCT	1	87377759	87377786	7	ENSSSCT00000004886	FYN	intron	.	rs791976396	2
AGAT.ATCT	1	87377759	87377786	7	ENSSSCT00000004884	FYN	intron	.	rs791976396	2
AGAT.ATCT	1	87377759	87377786	7	ENSSSCT00000004885	FYN	intron	.	rs791976396	2
AAAG.CTTT	1	87519823	87519835	3.25	.	.	intergenic	-0.045	.	4
AG.CT	1	87648187	87648198	6	.	.	intergenic	.	rs694852576	3
AAAAC.GTTTT	1	87735253	87735275	4.6	.	.	intergenic	.	.	2
AAC.GTT	1	87735353	87735367	5	.	.	intergenic	.	)16768 rs7909	2
AT.AT	1	87759643	87759665	11.5	ENSSSCT00000004890	.	intron	.	rs787062072	2
AC.GT	1	88470564	88470587	12	.	.	intergenic	0.55	rs793871696	2
AT.AT	1	88605136	88605147	6	.	.	intergenic	-0.021	rs790173143	2
AAT.ATT	1	88903340	88903355	5.33333	.	.	intergenic	0.063	rs789223752	2
AAAG.CTTT	1	89168891	89168922	8	.	.	intergenic	-0.009	.	2
AG.CT	1	89171593	89171613	10.5	.	.	intergenic	0.197	rs788834601	2

AAAC.GTTT	1	89413203	89413220	4.5	.	.	intergenic	0.698	rs791435727	2
AAAT.ATTT	1	89745706	89745723	4.5	.	.	intergenic	.	rs793270621	2
AAAG.CTTT	1	89930963	89930976	3.5	ENSSSCT00000004897	HDAC2	intron	.	390355 rs7863	2
AT.AT	1	90128062	90128081	10	.	.	intergenic	0.105	.	4
AC.GT	1	90292671	90292681	5.5	.	.	intergenic	0.343	rs790990254	4
AAAC.GTTT	1	90480271	90480298	7	.	.	intergenic	.	rs793244422	2
AAAG.CTTT	1	90648749	90648763	3.75	.	.	intergenic	0.039	rs791190943	2
AAAT.ATTT	1	90737138	90737156	4.75	.	.	intergenic	-0.271	rs787229695	2
AT.AT	1	90752481	90752492	6	.	.	intergenic	-0.073	rs788909394	2
AAAC.GTTT	1	90903351	90903364	3.5	.	.	intergenic	-0.127	.	3
AAAAT.ATTTT	1	90942753	90942771	3.8	.	.	intergenic	0.088	rs789057237	2
AT.AT	1	90960110	90960123	7	.	.	intergenic	-0.253	rs789817240	2
AAAAC.GTTTT	1	91101568	91101593	5.2	.	.	intergenic	0.579	220656 rs7867	2
AT.AT	1	91118153	91118164	6	.	.	intergenic	0.002	rs792534764	2
AAT.ATT	1	91190574	91190595	7.33333	.	.	intergenic	-0.035	rs700471871	2
AAAT.ATTT	1	91452983	91452996	3.5	.	.	intergenic	0.16	rs790173712	2
AC.GT	1	91703290	91703308	9.5	.	.	intergenic	-0.015	.	4
AAGG.CCTT	1	91851864	91851878	3.75	.	.	intergenic	0.115	rs790550013	3
AAAT.ATTT	1	91860756	91860778	5.75	ENSSSCT00000004900	NT5DC1	promoter	0.084	773771 rs7867	2
AAAT.ATTT	1	91860756	91860778	5.75	ENSSSCT00000035653	NT5DC1	promoter	0.084	773771 rs7867	2
AG.CT	1	91992465	91992475	5.5	ENSSSCT00000035653	NT5DC1	intron	0.187	rs792074774	2
AG.CT	1	91992465	91992475	5.5	ENSSSCT00000004900	NT5DC1	intron	0.187	rs792074774	2
AAAT.ATTT	1	92228771	92228801	7.75	.	.	intergenic	0.355	rs708434469	2
AAAG.CTTT	1	92267185	92267218	8.5	ENSSSCT00000004906	DSE	intron	.	rs790557641	2
AAAAC.GTTTT	1	92519700	92519727	5.6	.	.	intergenic	.	rs792614937	2
AG.CT	1	92842886	92842897	6	ENSSSCT00000004918	SNAP91	intron	-0.047	rs789250221	2
AT.AT	1	93040784	93040795	6	.	.	intergenic	0.513	204508 rs7907	2
AG.CT	1	93044288	93044305	9	.	.	intergenic	.	.	2
AAC.GTT	1	93176165	93176179	5	ENSSSCT00000004920	ME1	intron	.	rs792484034	3
AAAAC.GTTTT	1	93224461	93224499	7.8	ENSSSCT00000004920	ME1	intron	0.058	rs787861998	2
AAAC.GTTT	1	93287068	93287086	4.75	ENSSSCT00000035912	DOPEY1	intron	0.238	rs789532639	2
AAAC.GTTT	1	93287068	93287086	4.75	ENSSSCT00000004923	DOPEY1	intron	0.238	rs789532639	2
AAAC.GTTT	1	93287068	93287086	4.75	ENSSSCT00000022952	DOPEY1	intron	0.238	rs789532639	2
AT.AT	1	93460078	93460101	12	ENSSSCT00000032416	.	intron	0.39	.	2
AAAC.GTTT	1	93508292	93508314	5.75	ENSSSCT00000025129	UBE3D	intron	0.083	rs787501743	2
AC.GT	1	94094725	94094738	7	.	.	intergenic	0.09	rs793085506	2
AT.AT	1	94126276	94126286	5.5	.	.	intergenic	.	.	2
AAAC.GTTT	1	94369344	94369363	5	.	.	intergenic	0.177	918074 rs7923	2
AG.CT	1	94455768	94455780	6.5	.	.	intergenic	0.381	rs792648033	2
AAAT.ATTT	1	94539328	94539357	7.5	ENSSSCT00000004926	IBTK	intron	-0.095	rs793297516	2
AAAT.ATTT	1	94539328	94539357	7.5	ENSSSCT00000021698	SNORD112	promoter	-0.095	rs793297516	2
AAAC.GTTT	1	94614765	94614787	5.75	.	.	intergenic	.	rs786298667	2
AAAT.ATTT	1	94694546	94694573	7	.	.	intergenic	.	rs788118469	2
AATG.CATT	1	94709038	94709055	4.5	.	.	intergenic	0.104	.	2
AC.GT	1	94952399	94952429	15.5	.	.	intergenic	0.252	rs788197600	4
AC.GT	1	95164223	95164237	7.5	.	.	intergenic	0.222	.	2
AAAT.ATTT	1	95379225	95379244	5	.	.	intergenic	0.136	rs793238310	2
AG.CT	1	95562729	95562748	10	.	.	intergenic	.	rs791537104	2
AT.AT	1	95583481	95583493	6.5	.	.	intergenic	-0.317	rs791821044	2
AC.GT	1	95631515	95631530	8	.	.	intergenic	-0.083	rs790223518	2
AT.AT	1	95790417	95790446	15	.	.	intergenic	.	rs789568397	2
AAC.GTT	1	95892966	95892981	5.33333	.	.	intergenic	-0.002	rs790286441	2
AT.AT	1	96056338	96056349	6	.	.	intergenic	.	rs788789194	2
AAAC.GTTT	1	96317126	96317154	7.25	.	.	intergenic	0.022	048889 rs7907	2
AAG.CTT	1	96332481	96332494	4.66667	.	.	intergenic	0.142	rs788172496	2
AAT.ATT	1	96494262	96494293	10.6667	ENSSSCT00000024946	BCKDHB	intron	0.007	rs790914807	2
AC.GT	1	97129394	97129412	9.5	.	.	intergenic	.	rs793306491	3
AAAC.GTTT	1	97184041	97184060	5	.	.	intergenic	-0.201	rs787623495	2
AG.CT	1	97192209	97192225	8.5	.	.	intergenic	-0.053	486374 rs7886	2
AAAC.GTTT	1	97336648	97336675	7	ENSSSCT00000004936	LCA5	intron	0.14	rs787117732	2
AAAAC.GTTTT	1	97546417	97546444	5.6	.	.	intergenic	.	rs789252979	2
AT.AT	1	97829824	97829865	21	.	.	intergenic	-0.094	rs790211222	2
AT.AT	1	97865297	97865308	6	.	.	intergenic	0.187	rs792790144	2
AAAAC.GTTTT	1	97868685	97868709	5	.	.	intergenic	0.099	rs790692992	2
AAAC.GTTT	1	98545862	98545880	4.75	.	.	intergenic	.	971501 rs7914	2
AC.GT	1	98630326	98630345	10	.	.	intergenic	0.079	rs792249373	3
AATG.CATT	1	98631106	98631136	7.75	.	.	intergenic	0.06	711505 rs7899	2
AG.CT	1	98789885	98789895	5.5	.	.	intergenic	.	.	4
AAC.GTT	1	98822636	98822657	7.33333	.	.	intergenic	.	777318 rs7935	2
AC.GT	1	98980696	98980706	5.5	.	.	intergenic	.	.	3
CACCC.GGGTG	1	99156830	99156854	4.16667	.	.	intergenic	0.002	rs792715940	2
AAAC.GTTT	1	99422196	99422222	6.75	.	.	intergenic	.	rs788734692	2
AGG.CCT	1	99440523	99440537	5	.	.	intergenic	.	rs788903210	2
AAT.ATT	1	99509313	99509327	5	.	.	intergenic	0.178	rs793795513	3
AAAC.GTTT	1	99888903	99888929	6.75	.	.	intergenic	.	rs793687577	2
AG.CT	1	99977410	99977430	10.5	.	.	intergenic	.	rs787679454	2
AG.CT	1	100041632	100041646	7.5	.	.	intergenic	.	rs789698475	2
AAAC.GTTT	1	100253088	100253106	4.75	.	.	intergenic	0.251	605251 rs7931	2
AGAT.ATCT	1	100277892	100277903	3	.	.	intergenic	0.165	472777 rs7894	2
AAC.GTT	1	100311826	100311839	4.66667	.	.	intergenic	.	rs791186672	2
AG.CT	1	100362367	100362376	5	.	.	intergenic	.	rs789249129	2
AT.AT	1	100413357	100413371	7.5	.	.	intergenic	.	.	2
AC.GT	1	100520500	100520512	6.5	.	.	intergenic	0.006	rs786946960	3
AC.GT	1	100524315	100524333	9.5	.	.	intergenic	0.036	rs706172646	2

AAAC.GTTT	1	101291815	101291826	3	.	.	intergenic	0.437	781628 rs7909	2
AAGG.CCTT	1	101345322	101345335	3.5	.	.	intergenic	.	rs786302860	2
AAAC.GTTT	1	101501462	101501489	7	.	.	intergenic	-0.025	rs792963588	2
AG.CT	1	101625294	101625306	6.5	ENSSSCT00000004949	FILIP1	intron	-0.049	rs790580784	2
AAAAC.GTTTT	1	102002980	102003004	5	.	.	intergenic	.	rs790302266	2
ACAT.ATGT	1	102165762	102165775	3.5	.	.	intergenic	.	rs792867680	2
AT.AT	1	102449738	102449750	6.5	.	.	intergenic	.	rs793814351	4
AAAC.GTTT	1	102627186	102627230	11.25	.	.	intergenic	0.163	383320 rs7906	2
AAACC.GGTTT	1	102657847	102657863	3.4	.	.	intergenic	.	.	2
AC.GT	1	103175127	103175137	5.5	ENSSSCT00000035901	CD109	intron	-0.302	.	2
AC.GT	1	103175127	103175137	5.5	ENSSSCT00000004956	CD109	intron	-0.302	.	2
AC.GT	1	103175127	103175137	5.5	ENSSSCT00000035127	CD109	intron	-0.302	.	2
AT.AT	1	103363625	103363634	5	ENSSSCT00000004957	SLC17A5	intron	.	.	2
AC.GT	1	103667558	103667568	5.5	.	.	intergenic	.	rs790666734	3
AC.GT	1	103685722	103685732	5.5	.	.	intergenic	.	rs792886333	3
AC.GT	1	104155184	104155193	5	.	.	intergenic	-0.3	.	2
AAC.GTT	1	104675818	104675831	4.66667	.	.	intergenic	.	317624 rs7894	2
AAAAT.ATTTT	1	104937520	104937538	3.8	.	.	intergenic	0.921	rs793464536	2
AC.GT	1	104950580	104950590	5.5	.	.	intergenic	0.617	rs787710023	2
AC.GT	1	105269509	105269529	10.5	.	.	intergenic	0.047	rs787636788	2
AATG.CATT	1	105438596	105438644	12.25	ENSSSCT00000004963	SLC14A1	intron	0.017	389852 rs7937	2
AAAC.GTTT	1	105900826	105900850	6.25	.	.	intergenic	.	rs792765820	2
AT.AT	1	105921955	105921964	5	ENSSSCT00000004969	HAUS1	intron	-0.095	rs793579115	2
AAAT.ATTT	1	105929361	105929376	4	ENSSSCT00000004969	HAUS1	intron	0.11	rs789664012	2
AAG.CTT	1	106097505	106097517	4.33333	.	.	intergenic	.	.	2
AC.GT	1	106147009	106147022	7	.	.	intergenic	-0.039	.	3
AC.GT	1	106761598	106761610	6.5	.	.	intergenic	.	rs788419756	4
AAG.CTT	1	106978525	106978538	4.66667	ENSSSCT00000004977	SMAD2	intron	.	rs792020409	2
AT.AT	1	107009267	107009278	6	.	.	intergenic	.	rs788189960	2
AAAC.GTTT	1	107048037	107048056	5	.	.	intergenic	0.621	rs793249720	2
AG.CT	1	107131892	107131908	8.5	.	.	intergenic	-0.085	rs791501235	2
AG.CT	1	107182157	107182168	6	.	.	intergenic	-0.275	rs791610942	2
AAG.CTT	1	107284891	107284907	5.66667	.	.	intergenic	-0.087	rs792121340	2
AAT.ATT	1	107386051	107386063	4.33333	.	.	intergenic	.	rs789005855	2
AC.GT	1	107450784	107450795	6	.	.	intergenic	0.104	.	2
AAAAC.GTTTT	1	107475981	107476010	5	.	.	intergenic	0.339	rs787397115	2
AATG.CATT	1	107562149	107562166	4.5	.	.	intergenic	0.002	rs789634613	2
AAGG.CCTT	1	107606974	107607023	12.5	.	.	intergenic	.	rs787606811	2
AG.CT	1	107626845	107626865	10.5	.	.	intergenic	0.284	rs790048786	2
AG.CT	1	107677091	107677109	9.5	ENSSSCT00000004978	CTIF	intron	0.144	rs788888514	2
AAAAC.GTTTT	1	107783811	107783831	4.2	ENSSSCT00000004978	CTIF	intron	-0.18	rs786720488	2
AT.AT	1	107926894	107926905	6	ENSSSCT00000004978	CTIF	intron	.	rs790581810	2
AATG.CATT	1	108006850	108006873	6	ENSSSCT00000004978	CTIF	intron	-0.008	rs793349418	2
ATC.GAT	1	108040423	108040434	4	.	.	intergenic	.	.	2
ACC.GGT	1	108062550	108062565	5.33333	.	.	intergenic	-0.281	rs786897073	2
AAAC.GTTT	1	108210151	108210166	4	.	.	intergenic	.	rs787631705	2
AAAC.GTTT	1	108327711	108327732	5.5	ENSSSCT00000004980	.	intron	0.234	rs788830916	2
AAT.ATT	1	108363617	108363635	6.33333	.	.	intergenic	.	472519 rs7929	2
ATC.GAT	1	108380999	108381019	7	.	.	intergenic	0.447	rs786987620	3
AT.AT	1	108763075	108763093	9.5	.	.	intergenic	-0.139	378689 rs7912	2
AAAAT.ATTTT	1	109260733	109260750	3.6	ENSSSCT00000004983	MYO5B	intron	-0.171	rs792007039	2
AGGG.CCCT	1	109672566	109672580	3.75	ENSSSCT00000004990	SKA1	intron	0.307	rs789045417	2
AAAC.GTTT	1	109674731	109674747	4.25	ENSSSCT00000004990	SKA1	intron	-0.049	rs791288558	2
AT.AT	1	109742803	109742812	5	.	.	intergenic	.	rs792216251	2
AAAC.GTTT	1	109815609	109815627	4.75	.	.	intergenic	.	rs787939488	2
AT.AT	1	109931016	109931030	7.5	.	.	intergenic	.	rs793540813	2
AAAAC.GTTTT	1	110428109	110428131	4.6	.	.	intergenic	.	339554 rs7911	2
AC.GT	1	110554404	110554420	8.5	ENSSSCT00000004997	SMAD4	intron	.	rs789540428	2
AAAC.GTTT	1	110567061	110567082	5.5	.	.	intergenic	1.245	rs787805463	2
AC.GT	1	110601493	110601505	6.5	.	.	intergenic	-0.126	581936 rs7873	3
AAAAG.CTTTT	1	110739714	110739733	4	.	.	intergenic	.	rs792222964	2
AAAC.GTTT	1	110979348	110979366	4.75	.	.	intergenic	0.109	rs789912785	2
AAAT.ATTT	1	110989580	110989596	4.25	.	.	intergenic	0.256	rs792405517	2
AT.AT	1	111321430	111321439	5	.	.	intergenic	0.047	.	4
AC.GT	1	111333173	111333198	13	.	.	intergenic	0.088	rs786858880	2
AAC.GTT	1	112151793	112151812	6.66667	.	.	intergenic	-0.077	rs791681359	2
AAAC.GTTT	1	112314873	112314904	8	.	.	intergenic	-0.268	240740 rs7900	2
AC.GT	1	112485332	112485348	8.5	.	.	intergenic	-0.05	.	2
AAAAC.GTTTT	1	112596416	112596438	4.6	.	.	intergenic	-0.096	381147 rs7938	2
AT.AT	1	112649623	112649640	9	.	.	intergenic	-0.048	rs789862355	2
AC.GT	1	112876668	112876681	7	.	.	intergenic	.	rs789398152	4
AC.GT	1	113027818	113027835	9	.	.	intergenic	0.145	rs793145327	2
AAT.ATT	1	113139576	113139606	10.3333	.	.	intergenic	.	508373 rs7924	2
AAAC.GTTT	1	113307237	113307261	6.25	.	.	intergenic	0.106	563540 rs7890	2
AAC.GTT	1	113575862	113575884	7.66667	.	.	intergenic	-0.045	rs790551132	2
AAAAC.GTTTT	1	113879872	113879899	4.66667	.	.	intergenic	0.268	rs792590366	2
AC.GT	1	113903797	113903807	5.5	.	.	intergenic	-0.14	.	2
AAAC.GTTT	1	114102739	114102752	3.5	.	.	intergenic	-0.025	349616 rs7887	2
AT.AT	1	114111969	114111978	5	ENSSSCT00000005003	POLI	intron	.	rs787834152	2
AG.CT	1	114329334	114329344	5.5	.	.	intergenic	0.312	rs791405627	2
AAAAC.GTTTT	1	114560756	114560784	5.8	.	.	intergenic	0.123	482675 rs7873	2
AAGG.CCTT	1	114737550	114737562	3.25	ENSSSCT00000005007	RAB27B	intron	0.031	rs793709834	2
AT.AT	1	115122403	115122413	5.5	ENSSSCT00000036505	TCF4	intron	0.086	.	2
AT.AT	1	115122403	115122413	5.5	ENSSSCT00000036325	TCF4	intron	0.086	.	2

AT.AT	1	115122403	115122413	5.5	ENSSSCT00000005009	TCF4	intron	0.086	.	2
AAT.ATT	1	115178667	115178679	4.33333	ENSSSCT00000036505	TCF4	intron	0.117	.	2
AAT.ATT	1	115178667	115178679	4.33333	ENSSSCT00000036325	TCF4	intron	0.117	.	2
AG.CT	1	115383141	115383152	6	.	.	intergenic	-0.006	rs789809284	2
AAAC.GTTT	1	115932111	115932135	6.25	.	.	intergenic	.	rs793832595	2
AG.CT	1	117491782	117491791	5	ENSSSCT00000005017	NARS	promoter	0.941	746752 rs7863	2
AAAC.GTTT	1	117840938	117840966	7.25	ENSSSCT00000005020	ANKDD1A	intron	0.043	rs790324971	3
AT.AT	1	118140098	118140114	8.5	.	.	intergenic	.	rs786926125	2
ACC.GGT	1	118302572	118302584	4.33333	ENSSSCT00000005027	ZNF609	intron	0.38	rs789740323	3
AAC.GTT	1	118358881	118358894	4.66667	ENSSSCT00000005027	ZNF609	intron	0.677	rs786367323	2
AAAG.CTTT	1	118547522	118547544	5.75	.	.	intergenic	.	362916 rs7923	2
AAG.CTT	1	118713651	118713665	5	ENSSSCT00000005035	.	intron	0.135	rs789783703	2
AC.GT	1	118926227	118926242	8	ENSSSCT00000005037	.	intron	.	rs793876140	2
AC.GT	1	119054849	119054875	13.5	.	.	intergenic	.	065577 rs7931	2
AAGG.CCTT	1	119174347	119174364	4.5	.	.	intergenic	-0.09	rs792695785	2
AGG.CCT	1	120045958	120045971	4.66667	.	.	intergenic	-0.138	rs792798500	2
AAG.CTT	1	120098873	120098884	4	.	.	intergenic	.	rs792332831	2
AT.AT	1	120157050	120157060	5.5	.	.	intergenic	.	rs790021466	2
AAAC.GTTT	1	120284076	120284094	4.75	.	.	intergenic	.	280168 rs7867	2
ACAT.ATGT	1	120302025	120302043	4.75	.	.	intergenic	0.038	rs788842614	3
AG.CT	1	120414585	120414594	5	.	.	intergenic	-0.005	rs786398777	3
AGGG.CCCT	1	120461889	120461906	4.5	ENSSSCT00000031480	.	intron	-0.092	.	2
AT.AT	1	120641380	120641390	5.5	.	.	intergenic	-0.018	rs791821700	2
AT.AT	1	120654633	120654643	5.5	.	.	intergenic	-0.143	rs793540228	5
AT.AT	1	121512667	121512676	5	ENSSSCT00000028902	.	intron	-0.045	rs790829188	3
AC.GT	1	121528720	121528734	7.5	ENSSSCT00000028902	.	3'utr	0.197	rs788729051	2
AAAC.GTTT	1	121633098	121633125	7	.	.	intergenic	-0.006	rs786810350	2
AT.AT	1	121738649	121738660	6	.	.	intergenic	0.099	511292 rs7886	2
AC.GT	1	121807241	121807255	7.5	.	.	intergenic	-0.121	.	3
AC.GT	1	122303330	122303345	8	.	.	intergenic	-0.058	rs786858064	8
AAT.ATT	1	122369258	122369277	6.66667	.	.	intergenic	0.28	rs792523759	2
AGG.CCT	1	122529827	122529847	7	.	.	intergenic	-0.253	rs789947701	2
AT.AT	1	122664146	122664156	5.5	.	.	intergenic	-0.002	rs790856163	3
AG.CT	1	123304883	123304897	7.5	ENSSSCT00000005056	NARG2	intron	.	rs786862388	2
AG.CT	1	123304883	123304897	7.5	ENSSSCT00000025364	NARG2	intron	.	rs786862388	2
AAAAAC.GTTTTT	1	123313980	123314001	3.66667	ENSSSCT00000005056	NARG2	intron	0.202	rs793477378	2
AAAAAC.GTTTTT	1	123313980	123314001	3.66667	ENSSSCT00000025364	NARG2	intron	0.202	rs793477378	2
AC.GT	1	123391935	123391952	9	ENSSSCT00000034155	ANXA2	intron	0.005	233630 rs7887	2
AC.GT	1	123391935	123391952	9	ENSSSCT00000005057	ANXA2	intron	0.005	233630 rs7887	2
AAAT.ATTT	1	123434676	123434696	5.25	.	.	intergenic	-0.097	rs791372657	2
AG.CT	1	123563826	123563837	6	.	.	intergenic	-0.012	rs786910972	2
AAT.ATT	1	124116134	124116151	6	.	.	intergenic	0.125	.	2
AAC.GTT	1	124172764	124172777	4.66667	.	.	intergenic	-0.046	rs790093526	2
AT.AT	1	124532099	124532127	14.5	.	.	intergenic	.	rs788644968	3
AG.CT	1	124578472	124578482	5.5	ENSSSCT00000005065	FAM81A	intron	-0.064	rs793475618	3
AG.CT	1	124614557	124614568	6	ENSSSCT00000005065	FAM81A	intron	0.102	.	2
AAAC.GTTT	1	124660569	124660583	3.75	.	.	intergenic	-0.13	939523 rs7879	2
AAAAT.ATTTT	1	124664976	124664991	3.2	.	.	intergenic	.	.	2
AAAT.ATTT	1	124698307	124698334	7	ENSSSCT00000005066	MYO1E	intron	-0.101	rs792867664	2
AAAC.GTTT	1	124712766	124712788	5.75	ENSSSCT00000005066	MYO1E	intron	0.418	rs793746688	2
AAAT.ATTT	1	124763674	124763691	4.5	ENSSSCT00000005066	MYO1E	intron	-0.199	rs786287793	2
AAAAC.GTTTT	1	124963871	124963894	4.8	ENSSSCT00000005070	RNF111	intron	0.04	269572 rs7862	2
AAAC.GTTT	1	125470996	125471013	4.5	.	.	intergenic	0.074	.	2
AC.GT	1	125614624	125614635	6	ENSSSCT00000005077	.	intron	-0.31	rs793132949	2
ACAG.CTGT	1	125721839	125721859	5.25	.	.	intergenic	-0.05	rs792165466	2
AAAC.GTTT	1	125964933	125964961	7.25	.	.	intergenic	0.165	rs790415115	2
ACAT.ATGT	1	126019389	126019411	5.75	.	.	intergenic	-0.015	581133 rs7917	2
AAC.GTT	1	126097524	126097544	7	ENSSSCT00000027950	ALDH1A2	intron	-0.015	rs790208815	2
AT.AT	1	126160436	126160448	6.5	ENSSSCT00000024465	.	intron	0.67	rs792298211	3
AC.GT	1	126295054	126295082	14.5	.	.	intergenic	0.393	rs792205332	2
AT.AT	1	126403488	126403498	5.5	.	.	intergenic	.	rs787419712	2
AAC.GTT	1	126496513	126496524	4	ENSSSCT00000005079	MYZAP	intron	0.137	rs793097661	2
AAAG.CTTT	1	126553233	126553255	5.75	.	.	intergenic	-0.307	rs792380013	3
AAC.GTT	1	126713586	126713608	7.66667	.	.	intergenic	-0.123	rs786943153	2
AAACC.GGTTT	1	126860915	126860946	6.4	ENSSSCT00000005081	H242-58J12	intron	0.211	rs790049578	2
AAAC.GTTT	1	126929973	126929999	6.75	ENSSSCT00000005081	H242-58J12	intron	.	rs790042938	2
AAAC.GTTT	1	126929973	126929999	6.75	ENSSSCT00000034080	H242-58J12	intron	.	rs790042938	2
AAAC.GTTT	1	126929973	126929999	6.75	ENSSSCT00000035899	H242-58J12	intron	.	rs790042938	2
AAAAT.ATTTT	1	127061154	127061176	4.6	ENSSSCT00000005081	H242-58J12	intron	0.133	rs789517459	2
AAAAAC.GTTTTT	1	127531468	127531487	3.33333	.	.	intergenic	0.135	rs786691686	2
AAAC.GTTT	1	127687551	127687567	4.25	ENSSSCT00000028724	ZNF280D	intron	0.309	rs787186319	2
AAAT.ATTT	1	128141091	128141114	6	.	.	intergenic	.	rs791534032	2
AT.AT	1	128246359	128246369	5.5	ENSSSCT00000029885	.	intron	0.194	154525 rs7879	4
AAAG.CTTT	1	128577153	128577169	4.25	.	.	intergenic	-0.144	.	2
AC.GT	1	128583741	128583753	6.5	.	.	intergenic	-0.163	rs790998674	3
AAAT.ATTT	1	128784833	128784855	5.75	ENSSSCT00000005088	PYGO1	intron	0.09	rs790598978	2
AAC.GTT	1	129119486	129119502	5.66667	ENSSSCT00000005093	RAB27A	intron	.	rs788469444	2
AG.CT	1	129218734	129218746	6.5	.	.	intergenic	0.209	294120 rs7895	3
AAAT.ATTT	1	129278268	129278291	6	.	.	intergenic	.	rs791571327	2
AG.CT	1	129359895	129359904	5	.	.	intergenic	0.579	rs788337842	3
AAGG.CCTT	1	129616778	129616790	3.25	.	.	intergenic	.	.	2
AC.GT	1	129781445	129781455	5.5	.	.	intergenic	0.181	rs792314728	4
AAAC.GTTT	1	129792957	129792969	3.25	ENSSSCT00000005095	UNC13C	intron	-0.032	.	2
AAC.GTT	1	129847071	129847087	5.66667	ENSSSCT00000005095	UNC13C	intron	.	.	2

AC.GT	1	129847543	129847565	11.5	ENSSSCT00000005095	UNC13C	intron	.	552912 rs7934	2
AAAC.GTTT	1	130088643	130088659	4.25	ENSSSCT00000005095	UNC13C	intron	.	rs793329662	2
AAAC.GTTT	1	130088643	130088659	4.25	ENSSSCT00000032068	.	promoter	.	rs793329662	2
AAAC.GTTT	1	130278811	130278830	5	ENSSSCT00000005095	UNC13C	intron	0.091	rs792422066	3
AAAAG.CTTTT	1	130287302	130287316	3	ENSSSCT00000005095	UNC13C	intron	.	.	2
AAAC.GTTT	1	130580645	130580675	7.75	.	.	intergenic	.	rs789914029	2
AC.GT	1	130684716	130684733	9	.	.	intergenic	-0.071	rs793632274	2
AAAC.GTTT	1	130812912	130812931	5	ENSSSCT00000005096	.	intron	-0.002	rs791713190	2
AAC.GTT	1	132018054	132018072	6.33333	ENSSSCT00000005097	ONECUT1	intron	-0.075	rs786478979	2
AAGG.CCTT	1	132157932	132157952	5.25	ENSSSCT00000005098	FAM214A	intron	0.228	.	2
ACAT.ATGT	1	132446344	132446370	6.75	ENSSSCT00000005103	MYO5A	intron	0.009	rs789746199	2
AT.AT	1	132595155	132595165	5.5	ENSSSCT00000005104	MYO5C	intron	-0.156	rs792269429	3
AAC.GTT	1	132835053	132835077	8.33333	ENSSSCT00000005107	MAPK6	intron	0.314	238225 rs7902	4
AAAAC.GTTTT	1	133098178	133098206	5.8	ENSSSCT00000005113	SCG3	intron	0.184	125829 rs7886	4
AAAAC.GTTTT	1	133243899	133243921	4.6	ENSSSCT00000005111	TMOD2	intron	0.282	rs790653184	2
AT.AT	1	133520794	133520803	5	ENSSSCT00000005116	GLDN	intron	.	.	3
AAAC.GTTT	1	134270026	134270039	3.5	.	.	intergenic	.	rs791174336	2
AT.AT	1	134346735	134346747	6.5	.	.	intergenic	.	rs793220282	2
AACC.GGTT	1	134366223	134366234	3	.	.	intergenic	0.286	.	2
AAAAC.GTTTT	1	134424181	134424201	4.2	.	.	intergenic	0.071	.	2
AC.GT	1	134501137	134501150	7	.	.	intergenic	.	rs793452660	2
AC.GT	1	134627686	134627695	5	ENSSSCT00000005121	TRPM7	intron	0.211	.	2
AAAC.GTTT	1	135676894	135676914	5.25	.	.	intergenic	0.124	rs787335075	2
ATC.GAT	1	135912391	135912410	6.66667	.	.	intergenic	.	564520 rs7874	2
AAAC.GTTT	1	135920264	135920294	7.75	.	.	intergenic	0.204	rs788332169	5
AAC.GTT	1	136059356	136059377	7.33333	.	.	intergenic	.	.	2
AAC.GTT	1	136181735	136181753	6.33333	ENSSSCT00000005136	GALK2	intron	-0.022	rs792696986	2
AC.GT	1	136226815	136226840	13	ENSSSCT00000005136	GALK2	intron	0.094	.	2
AC.GT	1	136281060	136281074	7.5	ENSSSCT00000005136	GALK2	intron	1.519	rs791602164	8
AC.GT	1	136589391	136589400	5	ENSSSCT00000005140	SHC4	intron	.	.	3
AAAT.ATTT	1	136754175	136754202	7	ENSSSCT00000029380	CEP152	intron	0.087	531853 rs7889	2
AAAT.ATTT	1	136754175	136754202	7	ENSSSCT00000005142	CEP152	intron	0.087	531853 rs7889	2
AC.GT	1	137280383	137280397	7.5	ENSSSCT00000005143	FBN1	intron	-0.077	rs791275074	3
AAAT.ATTT	1	137616509	137616528	5	.	.	intergenic	0.176	rs786371088	2
AAT.ATT	1	137658629	137658642	4.66667	ENSSSCT00000005147	MYEF2	intron	0.242	rs791703459	2
AAAC.GTTT	1	137874450	137874464	3.75	.	.	intergenic	.	rs786582093	2
AC.GT	1	137904531	137904542	6	.	.	intergenic	.	.	2
AG.CT	1	137934728	137934743	8	.	.	intergenic	0.217	200224 rs7936	2
AATG.CATT	1	137975676	137975706	7.75	.	.	intergenic	0.122	rs786525994	2
AAAAC.GTTTT	1	138261322	138261348	5.4	.	.	intergenic	0.896	rs791359567	2
ATC.GAT	1	138290999	138291016	6	.	.	intergenic	0.227	rs790469347	2
AGG.CCT	1	138362227	138362242	5.33333	.	.	intergenic	0.923	rs791604818	2
AAAC.GTTT	1	138444536	138444550	3.75	.	.	intergenic	0.009	rs788707269	2
AAC.GTT	1	138454916	138454930	5	.	.	intergenic	0.094	rs791243477	2
AT.AT	1	139008614	139008643	15	.	.	intergenic	0.122	rs704391296	2
AC.GT	1	139252838	139252847	5	.	.	intergenic	0.32	.	3
AAAAT.ATTTT	1	139328335	139328362	5.6	.	.	intergenic	0.36	933318 rs7869	2
AG.CT	1	139362732	139362748	8.5	.	.	intergenic	0.275	rs789147906	2
AT.AT	1	139363422	139363440	9.5	.	.	intergenic	0.017	.	2
AG.CT	1	139393253	139393266	7	.	.	intergenic	0.092	.	3
AAAG.CTTT	1	139496192	139496207	4	.	.	intergenic	.	260431 rs7906	2
AAAC.GTTT	1	139672521	139672544	6	.	.	intergenic	.	rs787578220	2
AC.GT	1	139765876	139765885	5	.	.	intergenic	-0.049	.	2
AC.GT	1	139946796	139946809	7	.	.	intergenic	.	123716 rs7889	2
AG.CT	1	140195013	140195022	5	.	.	intergenic	.	.	3
AAC.GTT	1	140250250	140250267	6	.	.	intergenic	.	rs790189511	2
AAAT.ATTT	1	140351942	140351962	5.25	.	.	intergenic	.	rs788051368	2
AAAAC.GTTTT	1	140515798	140515826	5.8	.	.	intergenic	.	rs703663377	2
AAAC.GTTT	1	140529589	140529604	4	.	.	intergenic	.	393023 rs7889	2
AT.AT	1	140898877	140898906	15	ENSSSCT00000005157	SPATA5L1	intron	.	rs789898881	4
AAGC.GCTT	1	141068932	141068954	5.75	.	.	intergenic	.	rs788614685	2
AAAT.ATTT	1	141114594	141114612	4.75	.	.	intergenic	0.057	rs792509535	2
AAAC.GTTT	1	141205239	141205253	3.75	ENSSSCT00000005162	DUOXA2	3'utr	-0.244	174743 rs7932	2
AC.GT	1	141585216	141585226	5.5	.	.	intergenic	.	rs788651008	2
AAAC.GTTT	1	141603737	141603751	3.75	ENSSSCT00000028050	.	intron	-0.083	rs792090377	2
AAAG.CTTT	1	141728541	141728560	5	ENSSSCT00000025826	CASC4	intron	.	rs792611395	2
AAAG.CTTT	1	141728541	141728560	5	ENSSSCT00000005181	EIF3J	intron	.	rs792611395	2
AAAG.CTTT	1	141767356	141767374	4.75	ENSSSCT00000025826	CASC4	intron	0.332	rs793077787	2
AAAG.CTTT	1	141767356	141767374	4.75	ENSSSCT00000005180	CTDSPL2	intron	0.332	rs793077787	2
AAGGG.CCCTT	1	141818305	141818329	5	ENSSSCT00000025826	CASC4	intron	0.066	095256 rs7883	2
AAG.CTT	1	141893949	141893962	4.66667	ENSSSCT00000025826	CASC4	intron	.	rs790172176	2
AAAC.GTTT	1	141996154	141996167	3.5	.	.	intergenic	0.182	rs787182697	2
AAAT.ATTT	1	142242687	142242713	6.75	.	.	intergenic	0.791	779283 rs7889	2
AC.GT	1	142311289	142311303	7.5	.	.	intergenic	.	rs789409078	2
AC.GT	1	142528849	142528859	5.5	ENSSSCT00000005185	WDR76	intron	.	rs790891936	2
AGC.GCT	1	142665492	142665508	5.66667	ENSSSCT00000005193	STRC	cds	0.427	rs786894502	2
AAC.GTT	1	142945665	142945691	9	ENSSSCT00000005201	ZSCAN29	intron	0.44	939312 rs7892	2
AAAT.ATTT	1	142963277	142963299	5.75	ENSSSCT00000005202	ADAL	intron	.	rs787330722	2
AAAAC.GTTTT	1	143054985	143055008	4.8	ENSSSCT00000005205	TGM5	intron	0.194	rs786450617	3
AAC.GTT	1	143123580	143123598	6.33333	ENSSSCT00000005206	EPB42	intron	-0.285	rs789279781	2
AAAG.CTTT	1	143270561	143270582	5.5	ENSSSCT00000032277	UBR1	intron	0.4	rs791433863	2
AAAG.CTTT	1	143270561	143270582	5.5	ENSSSCT00000005209	UBR1	intron	0.4	rs791433863	2
AAAC.GTTT	1	143371157	143371183	6.75	ENSSSCT00000005210	TTBK2	intron	0.302	rs793617146	2
AAAT.ATTT	1	143635070	143635088	4.75	.	.	intergenic	.	rs791408965	2

AAC.GTT	1	143643702	143643730	9.66667	.	.	intergenic	.	rs793631431	2
AAAAC.GTTTT	1	143647975	143648002	4.66667	.	.	intergenic	0.197	rs788085651	2
AC.GT	1	143821806	143821820	7.5	ENSSSCT00000005218	ZNF106	intron	0.253	570280 rs7933	4
AAAT.ATTT	1	143942296	143942310	3.75	ENSSSCT00000005225	GANC	intron	-0.067	rs787790810	2
AAAC.GTTT	1	144152998	144153025	7	ENSSSCT00000005228	PLA2G4F	intron	-0.184	035472 rs7894	2
AAC.GTT	1	144249755	144249772	6	.	.	intergenic	.	rs787067792	2
AAAC.GTTT	1	144510463	144510487	6.25	ENSSSCT00000005234	.	intron	0.329	533439 rs7903	2
AAAC.GTTT	1	144510463	144510487	6.25	ENSSSCT00000005241	.	intron	0.329	533439 rs7903	2
AAAAT.ATTTT	1	144627944	144627959	3.2	ENSSSCT00000022785	EHD4	intron	.	rs786843519	2
AGGG.CCCT	1	145026193	145026207	3.75	.	.	intergenic	0.122	rs788609414	2
AAAG.CTTT	1	146176423	146176445	5.75	.	.	intergenic	.	402095 rs7933	2
GATCC.GGATC	1	146181902	146181923	3.66667	.	.	intergenic	-0.084	127303 rs7937	2
AT.AT	1	146665914	146665926	6.5	ENSSSCT00000005286	GPR176	intron	0.361	308044 rs7109	4
AAT.ATT	1	146773948	146773964	5.66667	.	.	intergenic	0.136	rs793051324	2
AAACT.AGTTT	1	146979366	146979382	3.4	.	.	intergenic	.	rs787257115	2
AAAC.GTTT	1	147033637	147033656	5	.	.	intergenic	0.128	rs790405531	2
AAAAC.GTTTT	1	147142239	147142263	5	.	.	intergenic	-0.164	rs788624398	2
AAAAC.GTTTT	1	147199769	147199789	4.2	.	.	intergenic	0.044	rs789568756	2
AT.AT	1	147331572	147331582	5.5	.	.	intergenic	0.216	rs793015433	2
AAAC.GTTT	1	147366943	147366965	5.75	.	.	intergenic	0.272	rs793063140	2
AAAAC.GTTTT	1	147371381	147371399	3.8	.	.	intergenic	0.322	rs790611037	2
AAT.ATT	1	147375806	147375819	4.66667	.	.	intergenic	.	.	3
AAG.CTT	1	147387886	147387903	6	.	.	intergenic	0.104	rs788860185	2
AATG.CATT	1	147950123	147950150	7	.	.	intergenic	0.009	rs788041245	2
AATG.CATT	1	148493013	148493035	5.75	ENSSSCT00000005292	SPRED1	intron	0.139	rs790577236	2
AT.AT	1	148499712	148499721	5	.	.	intergenic	0.173	.	2
AG.CT	1	148518103	148518119	8.5	.	.	intergenic	.	.	2
AAAAG.CTTTT	1	148597037	148597071	5.83333	.	.	intergenic	0.276	rs786372638	2
AAC.GTT	1	148753162	148753178	5.66667	.	.	intergenic	.	rs791863593	2
AT.AT	1	148819045	148819055	5.5	ENSSSCT00000005293	TMCO5A	intron	0.135	rs789973232	2
AG.CT	1	148888633	148888647	7.5	.	.	intergenic	2.815	.	3
AT.AT	1	148931206	148931233	14	.	.	intergenic	0.234	rs788910442	2
AC.GT	1	148957124	148957140	8.5	.	.	intergenic	3.645	323640 rs7884	2
AT.AT	1	149047892	149047905	7	.	.	intergenic	0.482	rs788309855	2
AT.AT	1	149076683	149076695	6.5	.	.	intergenic	0.169	rs786335864	2
AG.CT	1	149597650	149597659	5	.	.	intergenic	.	.	2
AT.AT	1	149637454	149637467	7	.	.	intergenic	0.2	rs792735777	3
AAAAC.GTTTT	1	149796299	149796321	4.6	.	.	intergenic	0.375	.	2
AC.GT	1	149853167	149853176	5	.	.	intergenic	1.314	.	3
AC.GT	1	150238861	150238873	6.5	.	.	intergenic	1.888	rs793145154	2
AAAT.ATTT	1	150251839	150251854	4	.	.	intergenic	0.621	rs791163837	2
AAAAC.GTTTT	1	150254564	150254587	4.8	.	.	intergenic	0.412	rs787388315	2
AAAAC.GTTTT	1	150293785	150293813	5.8	.	.	intergenic	0.435	rs787846915	2
AAAC.GTTT	1	150308264	150308278	3.75	.	.	intergenic	0.074	rs790636657	2
AAAT.ATTT	1	150620332	150620349	4.5	.	.	intergenic	0.131	rs786997038	2
AAT.ATT	1	151009854	151009867	4.66667	.	.	intergenic	.	.	2
AT.AT	1	151019445	151019478	17	.	.	intergenic	.	.	3
AAC.GTT	1	151143794	151143810	5.66667	.	.	intergenic	0.155	rs790501882	2
AG.CT	1	151325689	151325700	6	.	.	intergenic	.	rs791304498	2
AC.GT	1	151536059	151536076	9	.	.	intergenic	0.19	.	4
AAAAC.GTTTT	1	151791751	151791775	5	ENSSSCT00000029836	DPH6	intron	0.067	134360 rs7862	2
AC.GT	1	151859918	151859928	5.5	.	.	intergenic	.	rs790169309	2
AC.GT	1	151861239	151861248	5	.	.	intergenic	.	.	4
AAC.GTT	1	151885423	151885442	6.66667	.	.	intergenic	0.485	.	3
AG.CT	1	151935641	151935660	10	.	.	intergenic	0.176	rs786736069	2
AAAAAT.ATTTTT	1	152097439	152097461	3.83333	.	.	intergenic	-0.106	rs786658485	2
AAAAAC.GTTTTT	1	152342234	152342272	6.5	ENSSSCT00000005301	.	intron	0.146	rs791149149	2
AC.GT	1	152599168	152599177	5	.	.	intergenic	.	rs789623042	2
AAAT.ATTT	1	152635065	152635082	4.5	ENSSSCT00000005305	RHGAP11	cds	.	rs788980568	2
AAAT.ATTT	1	152635065	152635082	4.5	ENSSSCT00000005305	RHGAP11	intron	.	rs788980568	2
AT.AT	1	152873848	152873857	5	.	.	intergenic	0.359	rs789410452	4
AC.GT	1	153145273	153145296	12	.	.	intergenic	0.442	rs791222687	4
AC.GT	1	153158181	153158196	8	.	.	intergenic	0.126	rs790240910	2
AAAC.GTTT	1	153247867	153247885	4.75	.	.	intergenic	0.134	rs788230104	2
AC.GT	1	153321263	153321282	10	ENSSSCT00000030424	FAM169B	intron	0.068	.	4
AAAAG.CTTTT	1	153386424	153386438	3	.	.	intergenic	.	rs789797939	2
AAAC.GTTT	1	153486451	153486463	3.25	ENSSSCT00000005310	PGPEP1L	intron	.	rs793678263	2
AC.GT	1	153625926	153625939	7	ENSSSCT00000005311	IGF1R	intron	0.067	221873 rs7882	2
AAAC.GTTT	1	153733206	153733225	5	ENSSSCT00000005311	IGF1R	intron	-0.036	rs786556133	2
AC.GT	1	153969007	153969021	7.5	.	.	intergenic	0.007	rs786789619	2
AAAC.GTTT	1	154048315	154048335	5.25	.	.	intergenic	-0.069	018950 rs7902	2
AG.CT	1	154078047	154078058	6	ENSSSCT00000005313	TTC23	intron	-0.056	357375 rs7935	2
AAAC.GTTT	1	154151676	154151699	6	ENSSSCT00000005313	TTC23	intron	.	rs788508336	2
AC.GT	1	154310111	154310124	7	ENSSSCT00000005314	LRRC28	intron	.	rs791650790	2
AT.AT	1	154725207	154725218	6	.	.	intergenic	.	rs790361806	2
AAC.GTT	1	154815235	154815249	5	.	.	intergenic	0.078	rs793613062	2
AAAAG.CTTTT	1	154902756	154902782	5.4	.	.	intergenic	-0.172	rs789912694	2
AAAC.GTTT	1	155027752	155027770	4.75	.	.	intergenic	-0.249	392711 rs7870	2
AT.AT	1	155872821	155872832	6	.	.	intergenic	-0.012	142507 rs7938	3
AAAC.GTTT	1	155879448	155879469	5.5	.	.	intergenic	.	rs793371509	2
AAAC.GTTT	1	155924299	155924311	3.25	.	.	intergenic	-0.007	rs788910619	2
AAAAC.GTTTT	1	155974660	155974683	4.8	ENSSSCT00000005327	CHSY1	intron	-0.001	rs790703459	2
AT.AT	1	156046282	156046292	5.5	.	.	intergenic	.	310590 rs7872	3
AG.CT	1	156445683	156445697	7.5	.	.	intergenic	.	.	2

AAAC.GTTT	1	156538581	156538594	3.5	.	.	intergenic	.	rs786470848	3
AT.AT	1	156577123	156577149	13.5	.	.	intergenic	.	rs791157969	2
AAC.GTT	1	156723902	156723918	5.66667	.	.	intergenic	-0.198	rs789733672	2
AG.CT	1	157336713	157336730	9	.	.	intergenic	0.062	rs790244316	2
AG.CT	1	157562807	157562817	5.5	.	.	intergenic	0.088	rs788438470	2
AG.CT	1	157602843	157602856	7	.	.	intergenic	.	.	2
AGATAT.ATATCT	1	157603016	157603036	3.5	.	.	intergenic	.	546068 rs7897	2
AAAT.ATTT	1	158189442	158189466	6.25	.	.	intergenic	.	rs789332202	2
AC.GT	1	158252861	158252875	7.5	.	.	intergenic	.	.	4
AAAC.GTTT	1	158355549	158355573	6.25	.	.	intergenic	.	rs793429476	2
AT.AT	1	158430195	158430205	5.5	.	.	intergenic	.	rs788057471	2
AC.GT	1	158532975	158532987	6.5	.	.	intergenic	.	rs788731524	2
AAAC.GTTT	1	158802221	158802254	8.5	.	.	intergenic	.	rs790699565	2
AC.GT	1	158826152	158826166	7.5	.	.	intergenic	.	.	7
AAAC.GTTT	1	158953479	158953505	6.75	.	.	intergenic	.	rs790236687	2
AAAAC.GTTTT	1	159078067	159078086	4	.	.	intergenic	.	rs790362289	2
AAAC.GTTT	1	159347439	159347462	6	ENSSSCT00000005342	OTUD7A	intron	.	rs792550873	2
AAAG.CTTT	1	159461779	159461801	5.75	ENSSSCT00000032440	KLF13	intron	.	rs790639506	2
AC.GT	1	160199442	160199452	5.5	ENSSSCT00000005353	APBA2	intron	0.251	rs790568248	3
AC.GT	1	160199442	160199452	5.5	ENSSSCT00000022624	.	promoter	0.251	rs790568248	3
AC.GT	1	160726571	160726587	8.5	.	.	intergenic	-0.137	rs786723885	2
AT.AT	1	161566603	161566618	8	ENSSSCT00000005362	NFATC1	intron	.	rs788854904	2
AAAC.GTTT	1	161601430	161601450	5.25	ENSSSCT00000005362	NFATC1	intron	.	rs790337423	2
AAAC.GTTT	1	161601430	161601450	5.25	ENSSSCT00000005360	ATP9B	intron	.	rs790337423	2
AAAC.GTTT	1	161601430	161601450	5.25	ENSSSCT00000005363	ATP9B	intron	.	rs790337423	2
AAAC.GTTT	1	161672512	161672524	3.25	ENSSSCT00000005360	ATP9B	intron	.	rs791089037	2
AAAC.GTTT	1	161672512	161672524	3.25	ENSSSCT00000005363	ATP9B	intron	.	rs791089037	2
AG.CT	1	161737307	161737317	5.5	ENSSSCT00000005360	ATP9B	intron	.	rs788364538	2
AG.CT	1	161737307	161737317	5.5	ENSSSCT00000005363	ATP9B	intron	.	rs788364538	2
AG.CT	1	162682422	162682440	9.5	.	.	intergenic	.	540763 rs7889	2
AG.CT	1	162690223	162690243	10.5	.	.	intergenic	-0.281	.	3
AAAC.GTTT	1	162937743	162937765	5.75	.	.	intergenic	-0.227	rs788277955	2
AAT.ATT	1	163035112	163035134	7.66667	.	.	intergenic	-0.242	rs791111207	2
AAC.GTT	1	163066837	163066855	6.33333	.	.	intergenic	.	367397 rs7863	2
AAAT.ATTT	1	163287225	163287246	5.5	ENSSSCT00000025136	.	intron	0.012	rs789276387	2
AAAAAT.ATTTTT	1	163389289	163389308	3.33333	.	.	intergenic	.	383864 rs7886	2
AC.GT	1	163439034	163439047	7	.	.	intergenic	-0.045	926020 rs7917	2
AC.GT	1	163571655	163571667	6.5	.	.	intergenic	.	.	3
AAAAGC.GCTTTT	1	163944029	163944055	4.5	ENSSSCT00000005365	ZNF516	intron	-0.519	rs690563562	2
AAAC.GTTT	1	163965180	163965208	7.25	.	.	intergenic	-0.081	732906 rs7905	2
AT.AT	1	164092182	164092191	5	.	.	intergenic	.	.	3
AC.GT	1	164196505	164196516	6	.	.	intergenic	0.062	rs792770799	2
AAAC.GTTT	1	164254714	164254744	7.75	.	.	intergenic	0.926	rs692494150	2
AAAT.ATTT	1	164338445	164338464	5	.	.	intergenic	.	rs793443539	2
AAAC.GTTT	1	164348524	164348550	6.75	.	.	intergenic	0.421	.	2
AT.AT	1	164459041	164459050	5	.	.	intergenic	-0.123	rs787864527	2
AAAC.GTTT	1	164461132	164461146	3.75	.	.	intergenic	.	242823 rs7913	2
ATC.GAT	1	164798142	164798178	12.3333	.	.	intergenic	-0.351	rs790802063	2
AGGG.CCCT	1	165233636	165233653	4.5	.	.	intergenic	-0.203	.	2
AAAC.GTTT	1	165511379	165511401	5.75	.	.	intergenic	-0.211	rs792904995	2
AAAAC.GTTTT	1	165581724	165581751	5.6	ENSSSCT00000005375	CNDP1	intron	-0.064	rs790475319	2
AGG.CCT	1	166065938	166065950	4.33333	ENSSSCT00000005379	FBXO15	intron	-0.347	.	2
AG.CT	1	166159160	166159188	14.5	.	.	intergenic	-0.289	568285 rs7912	2
AAG.CTT	1	166244280	166244310	10.3333	.	.	intergenic	-0.063	rs789603253	2
AC.GT	1	166285160	166285176	8.5	.	.	intergenic	-0.128	.	3
AAAC.GTTT	1	166481782	166481797	4	.	.	intergenic	.	797450 rs7874	2
AAAAT.ATTTT	1	166869375	166869398	4.8	.	.	intergenic	.	rs791842718	2
AATG.CATT	1	167122718	167122741	6	.	.	intergenic	-0.176	rs793483334	2
AT.AT	1	167279724	167279734	5.5	.	.	intergenic	-0.186	.	2
AAAAT.ATTTT	1	167405203	167405229	5.4	.	.	intergenic	.	538381 rs7916	2
AT.AT	1	167882325	167882345	10.5	.	.	intergenic	.	.	2
AC.GT	1	168133223	168133242	10	.	.	intergenic	-0.043	.	4
AAAAT.ATTTT	1	168171992	168172011	4	.	.	intergenic	0.07	047078 rs7936	2
AT.AT	1	168198082	168198094	6.5	.	.	intergenic	0.056	523928 rs7894	3
AAT.ATT	1	168232363	168232380	6	.	.	intergenic	.	rs789925926	2
AG.CT	1	168601575	168601584	5	.	.	intergenic	-0.263	.	3
AC.GT	1	168776246	168776259	7	.	.	intergenic	0.045	981612 rs7035	2
AC.GT	1	169146782	169146793	6	.	.	intergenic	0.371	rs789118399	2
AC.GT	1	169876852	169876866	7.5	.	.	intergenic	-0.131	rs790362646	2
AT.AT	1	170038296	170038308	6.5	.	.	intergenic	-0.314	rs793800538	2
AG.CT	1	170171635	170171651	8.5	.	.	intergenic	-0.039	.	4
AAAC.GTTT	1	170321890	170321916	6.75	.	.	intergenic	0.02	rs791733318	2
AAAAAT.ATTTTT	1	170341859	170341883	4.16667	.	.	intergenic	-0.04	742635 rs7895	2
AAT.ATT	1	170448568	170448597	10	.	.	intergenic	.	rs786603503	2
AG.CT	1	170454081	170454093	6.5	.	.	intergenic	0.294	rs787415394	2
AC.GT	1	170464352	170464367	8	.	.	intergenic	-0.048	028961 rs7888	2
AC.GT	1	170518582	170518591	5	.	.	intergenic	.	rs789951961	2
AGAT.ATCT	1	170528236	170528250	3.75	.	.	intergenic	-0.311	rs788810646	2
AC.GT	1	170565703	170565723	10.5	.	.	intergenic	0.188	rs790139300	2
AAC.GTT	1	170768783	170768798	5.33333	ENSSSCT00000005390	TMX3	intron	0.91	rs791581063	2
AAG.CTT	1	170847703	170847719	5.66667	.	.	intergenic	0.04	rs787435088	3
AT.AT	1	170854504	170854515	6	.	.	intergenic	-0.161	315644 rs7900	2
AT.AT	1	170858462	170858474	6.5	.	.	intergenic	.	598456 rs7889	2
AGG.CCT	1	170865338	170865353	5.33333	.	.	intergenic	.	rs793685331	2

AT.AT	1	171350655	171350666	6	.	.	intergenic	.	.	4
AC.GT	1	171353292	171353305	7	.	.	intergenic	-0.042	.	4
AAAAT.ATTTT	1	171694381	171694401	4.2	.	.	intergenic	.	rs793207176	2
AAAAT.ATTTT	1	171789096	171789119	4.8	.	.	intergenic	-0.038	929074 rs7935	2
AAAT.ATTT	1	171790761	171790789	7.25	.	.	intergenic	.	937287 rs7899	2
AAAC.GTTT	1	171840646	171840657	3	.	.	intergenic	.	rs786880792	2
AG.CT	1	171896511	171896523	6.5	.	.	intergenic	-0.045	573178 rs7865	2
AAAAC.GTTTT	1	172001952	172001969	3.6	.	.	intergenic	.	.	2
AC.GT	1	172507364	172507386	11.5	.	.	intergenic	-0.117	rs788505708	2
AC.GT	1	172968941	172968970	15	ENSSSCT00000005391	CDH19	intron	.	.	2
AAC.GTT	1	173066013	173066026	4.66667	.	.	intergenic	-0.136	rs787918431	2
AT.AT	1	173101498	173101509	6	.	.	intergenic	0.309	rs787999112	3
AAAAC.GTTTT	1	173147904	173147923	4	.	.	intergenic	-0.079	.	2
AT.AT	1	173147987	173147997	5.5	.	.	intergenic	-0.014	rs791475690	2
AAAG.CTTT	1	173159897	173159934	9.5	.	.	intergenic	.	rs788185469	3
AT.AT	1	173221795	173221820	13	.	.	intergenic	0.116	116357 rs7902	2
AAAC.GTTT	1	173232072	173232083	3	.	.	intergenic	.	313978 rs7907	2
AC.GT	1	173307841	173307851	5.5	.	.	intergenic	-0.001	707616 rs7919	2
AAT.ATT	1	173448163	173448174	4	.	.	intergenic	-0.093	rs789704377	2
AAAT.ATTT	1	173574612	173574624	3.25	.	.	intergenic	0.298	rs789574028	2
AAAAC.GTTTT	1	173924154	173924170	3.4	.	.	intergenic	-0.079	.	2
AAAT.ATTT	1	173929750	173929769	5	.	.	intergenic	.	rs791642673	2
AT.AT	1	174292009	174292022	7	.	.	intergenic	.	647431 rs7936	2
AAAC.GTTT	1	174348080	174348103	6	.	.	intergenic	-0.133	746845 rs7897	2
AT.AT	1	174354756	174354766	5.5	.	.	intergenic	-0.054	rs787073884	2
AAAAC.GTTTT	1	174590982	174591009	5.6	.	.	intergenic	.	rs790203361	2
AC.GT	1	174899125	174899154	15	.	.	intergenic	0.093	rs793217450	2
AT.AT	1	174971280	174971291	6	.	.	intergenic	.	904696 rs7928	2
AC.GT	1	174974799	174974809	5.5	.	.	intergenic	-0.121	.	2
AT.AT	1	175084494	175084506	6.5	.	.	intergenic	0.054	675316 rs7869	2
AG.CT	1	175498517	175498526	5	.	.	intergenic	-0.116	rs791138280	2
AAAAC.GTTTT	1	176062518	176062542	5	.	.	intergenic	0.114	rs790528443	2
AAAC.GTTT	1	176071265	176071288	6	.	.	intergenic	0.086	rs791904300	2
AC.GT	1	176254328	176254341	7	.	.	intergenic	0.173	rs791711792	2
AC.GT	1	176450792	176450819	14	.	.	intergenic	.	rs786301829	3
AAAC.GTTT	1	176522062	176522076	3.75	.	.	intergenic	0.238	rs791968880	2
AAAC.GTTT	1	176608228	176608246	4.75	ENSSSCT00000028414	TNFRSF11	intron	0.236	rs786887881	2
AAAC.GTTT	1	176608228	176608246	4.75	ENSSSCT00000005404	TNFRSF11	intron	0.236	rs786887881	2
AC.GT	1	176652757	176652767	5.5	.	.	intergenic	-0.005	rs793039609	3
AT.AT	1	176693494	176693505	6	ENSSSCT00000005405	KIAA1468	intron	0.19	rs787342210	2
AAAC.GTTT	1	176727809	176727827	4.75	ENSSSCT00000005405	KIAA1468	intron	0.199	rs788429704	2
AC.GT	1	176764225	176764243	9.5	ENSSSCT00000005405	KIAA1468	intron	.	790591 rs7871	3
AAAAG.CTTTT	1	177231963	177231988	5.2	.	.	intergenic	0.237	rs786808743	3
AAAAT.ATTTT	1	177287503	177287532	6	.	.	intergenic	.	610110 rs7913	2
AAAAC.GTTTT	1	177740137	177740160	4.8	.	.	intergenic	-0.05	rs787373162	2
AAT.ATT	1	178101164	178101185	7.33333	.	.	intergenic	0.732	.	4
AAT.ATT	1	178202438	178202451	4.66667	.	.	intergenic	0.04	666211 rs7902	2
AG.CT	1	178292479	178292491	6.5	.	.	intergenic	.	rs788486597	2
AAAC.GTTT	1	178309217	178309234	4.5	.	.	intergenic	0.042	505324 rs7916	2
AC.GT	1	178491253	178491271	9.5	.	.	intergenic	0.108	.	3
AC.GT	1	178567203	178567216	7	.	.	intergenic	0.016	rs793326887	2
AAAC.GTTT	1	178571671	178571698	7	.	.	intergenic	-0.008	rs712428425	3
AG.CT	1	178725070	178725089	10	.	.	intergenic	.	298765 rs7913	2
AT.AT	1	178736924	178736936	6.5	.	.	intergenic	0.213	rs786672848	2
AC.GT	1	178996543	178996558	8	ENSSSCT00000005413	CCBE1	intron	0.165	757324 rs7929	2
AC.GT	1	179084651	179084670	10	ENSSSCT00000005413	CCBE1	intron	-0.031	137573 rs7937	3
ACAG.CTGT	1	179187730	179187748	4.75	ENSSSCT00000005415	LMAN1	intron	0.055	.	2
AAAC.GTTT	1	179332717	179332739	5.75	ENSSSCT00000005418	.	intron	-0.114	608942 rs7880	2
AAAC.GTTT	1	179466820	179466851	8	.	.	intergenic	-0.054	rs790535280	2
AAC.GTT	1	179583516	179583532	5.66667	.	.	intergenic	0	495441 rs7863	2
AG.CT	1	179612428	179612440	6.5	.	.	intergenic	.	rs787466048	2
AAAC.GTTT	1	179798167	179798185	4.75	ENSSSCT00000005427	ALPK2	intron	-0.051	rs791566553	2
AAAG.CTTT	1	180110591	180110619	7.25	.	.	intergenic	-0.183	rs793239157	2
AAAC.GTTT	1	180358208	180358234	6.75	.	.	intergenic	-0.008	333479 rs7907	2
AATG.CATT	1	180496477	180496494	4.5	.	.	intergenic	0.082	.	2
AC.GT	1	180529638	180529663	13	.	.	intergenic	-0.008	.	3
AAAC.GTTT	1	180544811	180544836	6.5	.	.	intergenic	.	rs791262304	2
AAAC.GTTT	1	180590683	180590697	3.75	.	.	intergenic	.	rs792732569	2
AAC.GTT	1	180700035	180700054	6.66667	ENSSSCT00000005437	CLPX	intron	1.762	rs786495487	2
AC.GT	1	180895939	180895952	7	ENSSSCT00000005439	PARP16	promoter	0.089	rs791393309	3
AAT.ATT	1	180917186	180917211	8.66667	.	.	intergenic	-0.064	rs791685764	2
AAAAG.CTTTT	1	180947395	180947417	4.6	ENSSSCT00000005442	IGDCC3	intron	-0.198	727224 rs7938	2
AAAT.ATTT	1	180968770	180968787	4.5	.	.	intergenic	.	rs789478509	2
AG.CT	1	180997071	180997086	8	ENSSSCT00000005441	IGDCC4	intron	0.06	rs690634157	2
AG.CT	1	180997071	180997086	8	ENSSSCT00000025179	IGDCC4	intron	0.06	rs690634157	2
AG.CT	1	181104316	181104325	5	ENSSSCT00000025793	PTPLAD1	intron	0.361	rs793449393	2
AG.CT	1	181104316	181104325	5	ENSSSCT00000005446	PTPLAD1	intron	0.361	rs793449393	2
AAC.GTT	1	181190076	181190098	7.66667	.	.	intergenic	.	476732 rs7913	3
AAC.GTT	1	182000106	182000127	7.33333	.	.	intergenic	.	rs788331612	2
AAAT.ATTT	1	183341070	183341088	4.75	ENSSSCT00000024115	MAP2K5	intron	1.841	rs793132451	2
AAAT.ATTT	1	183341070	183341088	4.75	ENSSSCT00000036194	MAP2K5	intron	1.841	rs793132451	2
AAAT.ATTT	1	183350924	183350945	5.5	ENSSSCT00000024115	MAP2K5	intron	.	rs791304572	2
AAAT.ATTT	1	183350924	183350945	5.5	ENSSSCT00000036194	MAP2K5	intron	.	rs791304572	2
AAAAAC.GTTTTT	1	183385859	183385884	4.33333	ENSSSCT00000024115	MAP2K5	intron	0.146	rs790131245	2

AAAAC.GTTTT	1	183385859	183385884	4.33333	ENSSSCT00000036194	MAP2K5	intron	0.146	rs790131245	2
AAC.GTT	1	183408755	183408771	5.66667	ENSSSCT00000024115	MAP2K5	intron	0.149	rs788845914	2
AAC.GTT	1	183408755	183408771	5.66667	ENSSSCT00000036194	MAP2K5	intron	0.149	rs788845914	2
AAAC.GTTT	1	183413325	183413338	3.5	ENSSSCT00000036194	MAP2K5	intron	0.296	rs793250612	2
AC.GT	1	183441453	183441467	7.5	ENSSSCT00000036194	MAP2K5	intron	0.019	rs791711193	2
AC.GT	1	183441453	183441467	7.5	ENSSSCT00000025567	.	intron	0.019	rs791711193	2
AG.CT	1	183536295	183536305	5.5	ENSSSCT00000005468	SKOR1	intron	-0.129	.	3
AAAT.ATTT	1	183570728	183570749	5.5	.	.	intergenic	.	.	2
AAAC.GTTT	1	183620423	183620443	5.25	.	.	intergenic	.	rs699586630	2
AC.GT	1	183668820	183668840	10.5	.	.	intergenic	0.147	rs789935561	2
AAAAC.GTTTT	1	183700977	183700995	3.8	.	.	intergenic	-0.013	rs788031561	2
AAAAT.ATTTT	1	183800756	183800784	5.8	ENSSSCT00000005470	.	intron	.	rs787486974	2
AC.GT	1	183879619	183879637	9.5	ENSSSCT00000005469	CLN6	3'utr	0.962	rs793746344	2
AC.GT	1	183879619	183879637	9.5	ENSSSCT00000005470	.	intron	0.962	rs793746344	2
AT.AT	1	184634317	184634330	7	.	.	intergenic	0.211	.	3
AAG.CTT	1	184660837	184660861	8.33333	.	.	intergenic	.	rs789485578	2
AAAT.ATTT	1	184865624	184865645	5.5	ENSSSCT00000005478	.	intron	-0.34	rs792883147	2
AAAC.GTTT	1	184922219	184922237	4.75	.	.	intergenic	0.069	)43127 rs7888	2
AAC.GTT	1	184991326	184991339	4.66667	ENSSSCT00000022924	GLCE	intron	0.458	rs791768499	2
AAAAC.GTTTT	1	185126532	185126555	4.8	.	.	intergenic	-0.204	rs791784911	2
ACAG.CTGT	1	186422281	186422299	4.75	.	.	intergenic	-0.201	rs786567202	2
AC.GT	1	186455618	186455636	9.5	.	.	intergenic	-0.077	.	3
AT.AT	1	186623585	186623596	6	.	.	intergenic	-0.13	.	4
AAAT.ATTT	1	186714496	186714516	5.25	.	.	intergenic	0.099	rs793443737	2
AG.CT	1	187470242	187470258	8.5	.	.	intergenic	0.002	rs789499584	2
AAAAC.GTTTT	1	187496419	187496435	3.4	ENSSSCT00000023869	.	intron	0.268	rs792005652	2
AAAC.GTTT	1	187706319	187706341	5.75	.	.	intergenic	0.159	rs793027883	2
AAAAC.GTTTT	1	187855960	187855987	5.6	ENSSSCT00000005492	THSD4	intron	0.081	rs790257501	3
AAAAC.GTTTT	1	187855960	187855987	5.6	ENSSSCT00000005493	THSD4	intron	0.081	rs790257501	3
AT.AT	1	187885190	187885199	5	ENSSSCT00000005492	THSD4	intron	.	.	2
AT.AT	1	187885190	187885199	5	ENSSSCT00000005493	THSD4	intron	.	.	2
AT.AT	1	187947406	187947437	16	ENSSSCT00000005492	THSD4	intron	0.167	rs788500093	2
AT.AT	1	187947406	187947437	16	ENSSSCT00000005493	THSD4	intron	0.167	rs788500093	2
AT.AT	1	187947406	187947437	16	ENSSSCT00000026271	.	intron	0.167	rs788500093	2
AAAC.GTTT	1	187977182	187977206	6.25	ENSSSCT00000026271	.	3'utr	1.155	rs789958075	2
AAAC.GTTT	1	187977182	187977206	6.25	ENSSSCT00000005492	THSD4	intron	1.155	rs789958075	2
AAAC.GTTT	1	187977182	187977206	6.25	ENSSSCT00000005493	THSD4	intron	1.155	rs789958075	2
AAAAC.GTTTT	1	188350937	188350959	4.6	ENSSSCT00000005496	.	intron	-0.054	573123 rs7882	2
AAAT.ATTT	1	188392734	188392759	6.5	.	.	intergenic	.	rs792353224	2
AT.AT	1	189035388	189035399	6	.	.	intergenic	0.113	rs792426573	2
AC.GT	1	189379993	189380013	10.5	.	.	intergenic	0.14	436330 rs7863	3
AAC.GTT	1	189441866	189441879	4.66667	.	.	intergenic	.	rs788988144	3
AAC.GTT	1	189506550	189506563	4.66667	.	.	intergenic	.	)33736 rs7917	2
AAT.ATT	1	189560761	189560776	5.33333	.	.	intergenic	0.08	.	2
AC.GT	1	189655007	189655027	10.5	.	.	intergenic	.	.	3
AT.AT	1	190254244	190254253	5	.	.	intergenic	-0.001	rs707784002	3
AGGG.CCCT	1	191191723	191191739	4.25	.	.	intergenic	-0.193	.	2
AAC.GTT	1	191413840	191413853	4.66667	.	.	intergenic	0.061	)54526 rs7873	2
AC.GT	1	191582012	191582037	13	.	.	intergenic	-0.042	rs792561874	2
AAAC.GTTT	1	191740920	191740942	5.75	.	.	intergenic	0.033	rs793338035	2
AC.GT	1	191891408	191891421	7	.	.	intergenic	0.233	)70907 rs7918	2
AAAC.GTTT	1	191893060	191893078	4.75	.	.	intergenic	.	rs789762398	2
AAAC.GTTT	1	191905397	191905416	5	.	.	intergenic	.	rs790644929	2
AT.AT	1	192161413	192161423	5.5	.	.	intergenic	-0.037	.	4
AT.AT	1	192218334	192218354	10.5	.	.	intergenic	.	rs789457805	2
AC.GT	1	192242646	192242657	6	.	.	intergenic	.	.	2
AT.AT	1	192454012	192454021	5	.	.	intergenic	-0.285	.	3
AC.GT	1	192669665	192669678	7	.	.	intergenic	.	381653 rs7928	4
AC.GT	1	192677290	192677301	6	.	.	intergenic	.	133315 rs7918	2
AAAG.CTTT	1	192705621	192705633	3.25	.	.	intergenic	.	rs789996090	2
AGAT.ATCT	1	192721736	192721753	4.5	.	.	intergenic	.	.	4
AT.AT	1	192906274	192906285	6	.	.	intergenic	0.128	rs792005388	2
AAAC.GTTT	1	192915158	192915178	5.25	.	.	intergenic	.	rs788792217	2
AAAG.CTTT	1	192947872	192947886	3.75	.	.	intergenic	.	rs793717397	3
AT.AT	1	192952095	192952109	7.5	.	.	intergenic	.	524459 rs7923	2
AAAC.GTTT	1	193508312	193508339	7	.	.	intergenic	-0.254	rs792092763	2
AAAAC.GTTTT	1	193532521	193532535	3	.	.	intergenic	.	rs789763699	2
AT.AT	1	193583870	193583880	5.5	.	.	intergenic	.	rs787019036	2
AT.AT	1	194192567	194192576	5	.	.	intergenic	.	.	3
AG.CT	1	194488613	194488627	7.5	.	.	intergenic	.	rs787784979	2
AT.AT	1	194719892	194719903	6	.	.	intergenic	.	rs786319888	2
AC.GT	1	194968899	194968929	15.5	ENSSSCT00000005510	KLHL28	intron	0.201	405783 rs7883	3
AAC.GTT	1	195138697	195138716	6.66667	ENSSSCT00000025268	FAM179B	intron	.	rs786512632	2
AT.AT	1	195540292	195540311	10	.	.	intergenic	0.253	rs791625421	2
AT.AT	1	195640969	195640980	6	.	.	intergenic	0.279	rs788880404	3
AC.GT	1	195700583	195700592	5	.	.	intergenic	0.45	.	3
AT.AT	1	195816954	195816963	5	.	.	intergenic	0.337	rs791610709	2
AAAC.GTTT	1	196021226	196021243	4.5	.	.	intergenic	0.241	rs788683265	2
AAAC.GTTT	1	196038262	196038276	3.75	.	.	intergenic	.	rs789677907	3
AAC.GTT	1	196677276	196677292	5.66667	.	.	intergenic	0.003	rs792974690	3
AT.AT	1	196996720	196996736	8.5	ENSSSCT00000005517	.	intron	0.057	.	4
AATG.CATT	1	197863377	197863402	6.5	.	.	intergenic	.	)54171 rs7921	2
AC.GT	1	197931492	197931503	6	.	.	intergenic	-0.053	rs790533878	3
AC.GT	1	197944929	197944941	6.5	.	.	intergenic	-0.064	rs791920090	2

AAAC.GTTT	1	197954676	197954695	5	.	.	intergenic	.	)18262 rs7931	2
AAAC.GTTT	1	198008393	198008423	7.75	.	.	intergenic	.	rs791781768	2
AT.AT	1	198254633	198254644	6	.	.	intergenic	-0.018	361785 rs7889	2
AAAC.GTTT	1	198805835	198805869	8.75	.	.	intergenic	.	592011 rs7911	2
AAT.ATT	1	199762214	199762225	4	.	.	intergenic	.	.	4
AAAC.GTTT	1	199875331	199875346	4	ENSSSCT00000005523	DNAAF2	intron	0.534	rs789743133	2
AAC.GTT	1	200330787	200330809	7.66667	ENSSSCT00000005531	SOS2	intron	.	323261 rs7909	2
AC.GT	1	200602609	200602619	5.5	ENSSSCT00000005536	.	intron	0.187	.	3
AAC.GTT	1	200939159	200939178	6.66667	ENSSSCT00000005539	ABHD12B	intron	0.191	rs789750270	2
AAC.GTT	1	200939159	200939178	6.66667	ENSSSCT00000023698	ABHD12B	intron	0.191	rs789750270	2
AAAC.GTTT	1	201078448	201078470	5.75	ENSSSCT00000005541	TRIM9	intron	0.219	rs788550861	2
AAAC.GTTT	1	201078448	201078470	5.75	ENSSSCT00000022907	TRIM9	intron	0.219	rs788550861	2
AAT.ATT	1	201225055	201225068	4.66667	ENSSSCT00000028405	TMX1	intron	0.121	rs790530994	2
AC.GT	1	201247526	201247538	6.5	.	.	intergenic	.	.	3
AG.CT	1	201352926	201352936	5.5	.	.	intergenic	0.023	.	3
AGC.GCT	1	201470442	201470453	4	.	.	intergenic	0.088	.	2
AC.GT	1	201606825	201606848	12	.	.	intergenic	0.153	rs793833170	2
AAAAT.ATTTT	1	201811107	201811130	4.8	.	.	intergenic	.	rs791088850	2
AG.CT	1	202057531	202057543	6.5	.	.	intergenic	1.153	.	5
AC.GT	1	202215971	202215987	8.5	.	.	intergenic	0.169	rs788024309	3
AAC.GTT	1	202318531	202318551	7	.	.	intergenic	0.08	352276 rs7895	2
AT.AT	1	202462682	202462692	5.5	ENSSSCT00000027583	.	intron	.	rs791431380	2
AT.AT	1	202462682	202462692	5.5	ENSSSCT00000005553	ERO1L	intron	.	rs791431380	2
AAAT.ATTT	1	202481560	202481583	6	ENSSSCT00000027583	.	intron	0.339	rs789361988	2
AAAT.ATTT	1	202481560	202481583	6	ENSSSCT00000005553	ERO1L	intron	0.339	rs789361988	2
AAC.GTT	1	202726988	202727001	4.66667	.	.	intergenic	0.122	rs791353349	2
AAG.CTT	1	202875596	202875621	8.66667	.	.	intergenic	-0.3	rs789440178	4
AAAAC.GTTTT	1	202883727	202883749	4.6	.	.	intergenic	0.419	rs789929565	2
AC.GT	1	202891414	202891436	11.5	.	.	intergenic	0.111	)01869 rs7917	4
AG.CT	1	203043706	203043722	8.5	.	.	intergenic	0.116	)42342 rs7879	3
AG.CT	1	203100535	203100550	8	.	.	intergenic	.	rs691587047	2
AC.GT	1	203706580	203706595	8	.	.	intergenic	0.305	)75065 rs7874	2
AT.AT	1	204166497	204166514	9	.	.	intergenic	0.159	)09110 rs7931	2
AG.CT	1	204447640	204447649	5	ENSSSCT00000024259	SAMD4A	intron	0.161	rs790935450	2
ATC.GAT	1	204511141	204511156	5.33333	ENSSSCT00000024259	SAMD4A	intron	0.076	rs787804043	2
AAAC.GTTT	1	204616524	204616539	4	ENSSSCT00000024259	SAMD4A	intron	-0.1	)26414 rs7922	2
AAAAC.GTTTT	1	204771869	204771889	4.2	.	.	intergenic	0.912	506037 rs7937	2
AT.AT	1	204903892	204903901	5	.	.	intergenic	0.713	.	2
AT.AT	1	204907741	204907754	7	ENSSSCT00000005571	MAPK1IP1L	promoter	.	149046 rs7876	2
AAAC.GTTT	1	204978256	204978278	5.75	.	.	intergenic	0.409	rs787778147	2
AAC.GTT	1	205407371	205407390	6.66667	.	.	intergenic	0.101	rs788870542	2
AAC.GTT	1	205414840	205414855	5.33333	.	.	intergenic	0.108	352414 rs7898	2
AAAC.GTTT	1	205491318	205491334	4.25	ENSSSCT00000005577	KTN1	intron	1.083	rs786498266	2
AGC.GCT	1	205517979	205518000	7.33333	ENSSSCT00000005577	KTN1	intron	0.472	315934 rs7937	2
AG.CT	1	205645061	205645070	5	.	.	intergenic	.	rs789073244	2
AAAAC.GTTTT	1	205713396	205713410	3	.	.	intergenic	.	rs792757370	2
AAAC.GTTT	1	205764701	205764717	4.25	.	.	intergenic	0.317	187259 rs7921	3
AAAAC.GTTTT	1	205929633	205929653	4.2	.	.	intergenic	0.203	rs786536401	2
AAAAC.GTTTT	1	205930807	205930828	4.4	.	.	intergenic	-0.021	rs787544441	2
AC.GT	1	206135419	206135434	8	ENSSSCT00000029724	PELI2	intron	-0.064	.	4
AAC.GTT	1	206244502	206244513	4	.	.	intergenic	.	rs792205585	2
AG.CT	1	206368580	206368593	7	.	.	intergenic	.	rs788263694	2
AAGG.CCTT	1	206459729	206459753	6.25	ENSSSCT00000005579	TMEM260	intron	0.305	117692 rs7895	2
AAG.CTT	1	206519216	206519230	5	.	.	intergenic	0.078	rs790785391	2
AAAT.ATTT	1	206544031	206544053	5.75	.	.	intergenic	0.17	703454 rs7894	2
AAC.GTT	1	206593487	206593504	6	.	.	intergenic	0.047	.	2
AAG.CTT	1	206655708	206655721	4.66667	.	.	intergenic	1.128	561931 rs7879	2
AT.AT	1	206771264	206771276	6.5	.	.	intergenic	0.121	rs790093025	3
AT.AT	1	206862083	206862093	5.5	.	.	intergenic	.	rs789719415	2
AATG.CATT	1	207149186	207149218	8.25	.	.	intergenic	-0.038	704162 rs7906	2
AAT.ATT	1	207218733	207218748	5.33333	.	.	intergenic	0.084	rs792124243	2
AAG.CTT	1	208077876	208077890	5	.	.	intergenic	.	rs792132198	2
AAAT.ATTT	1	208180167	208180190	6	.	.	intergenic	0.11	rs694024205	2
AT.AT	1	208632597	208632610	7	.	.	intergenic	0.512	rs788199673	2
AAT.ATT	1	208710310	208710321	4	.	.	intergenic	0.132	rs787409374	2
AAAG.CTTT	1	208719834	208719848	3.75	.	.	intergenic	.	155505 rs7874	2
AAC.GTT	1	208749320	208749333	4.66667	.	.	intergenic	0.068	508579 rs7914	2
AAAC.GTTT	1	208778179	208778201	5.75	.	.	intergenic	0.123	115876 rs7872	2
AAAT.ATTT	1	209535158	209535192	8.75	.	.	intergenic	-0.024	rs790848758	2
AG.CT	1	209926313	209926325	6.5	ENSSSCT00000022854	RTN1	intron	.	)13079 rs7865	2
AC.GT	1	210116887	210116899	6.5	ENSSSCT00000005600	LRRC9	intron	0.16	rs791548185	2
AG.CT	1	210124832	210124841	5	ENSSSCT00000005600	LRRC9	intron	0.241	.	2
AAAC.GTTT	1	210237958	210237977	5	.	.	intergenic	0.165	rs787661908	2
AATT.AATT	1	210484658	210484674	4.25	.	.	intergenic	0.193	rs793356513	2
AAG.CTT	1	210550666	210550684	6.33333	.	.	intergenic	.	rs787730643	2
AAAT.ATTT	1	210592544	210592577	8.5	.	.	intergenic	.	.	2
AG.CT	1	210708214	210708226	6.5	.	.	intergenic	0.161	.	3
AAT.ATT	1	211165013	211165026	4.66667	.	.	intergenic	.	rs790808553	2
AG.CT	1	211365904	211365933	15	.	.	intergenic	0.1	rs792968484	2
AG.CT	1	211499520	211499530	5.5	.	.	intergenic	-0.233	.	2
AT.AT	1	211782165	211782176	6	ENSSSCT00000005616	PKC	intron	0.268	rs713454280	2
AC.GT	1	212767376	212767394	9.5	.	.	intergenic	0.033	rs791448163	2
AAAAAC.GTTTTT	1	212782017	212782037	3.5	.	.	intergenic	.	314404 rs7937	2
ACCT.AGGT	1	213029287	213029308	5.5	ENSSSCT00000005628	NTRK3	intron	0.035	rs792426465	2

ATC.GAT	1	213044664	213044680	5.66667	ENSSSCT00000005628	NTRK3	intron	0.018	rs790639664	2
AG.CT	1	213076717	213076729	6.5	ENSSSCT00000005628	NTRK3	intron	0.233	.	4
ATC.GAT	1	213120147	213120159	4.33333	ENSSSCT00000005628	NTRK3	intron	0.084	.	4
AAC.GTT	1	213742957	213742972	5.33333	.	.	intergenic	.	.	2
AAC.GTT	1	213743642	213743658	5.66667	.	.	intergenic	.	.	2
AAC.GTT	1	214050188	214050200	4.33333	.	.	intergenic	-0.103	533184 rs7876	2
AAAT.ATTT	1	214201215	214201239	6.25	.	.	intergenic	0.125	281426 rs7888	2
AT.AT	1	214275929	214275938	5	.	.	intergenic	-0.168	.	2
AC.GT	1	214297370	214297382	6.5	.	.	intergenic	-0.06	rs793853763	2
AAAC.GTTT	1	214360158	214360193	9	.	.	intergenic	-0.055	rs788724601	2
AAT.ATT	1	214422808	214422819	4	.	.	intergenic	-0.109	rs791192165	2
AGATAT.ATATCT	1	214636492	214636520	4.83333	.	.	intergenic	-0.042	rs791979364	2
AC.GT	1	214800703	214800715	6.5	.	.	intergenic	-0.006	rs790386629	2
AT.AT	1	214895023	214895037	7.5	.	.	intergenic	0.096	rs793784045	2
AG.CT	1	214941374	214941384	5.5	.	.	intergenic	0.29	.	2
AC.GT	1	215049402	215049416	7.5	.	.	intergenic	0.163	rs787940574	2
AAGG.CCTT	1	215322847	215322874	7	.	.	intergenic	-0.029	374749 rs7913	2
AATC.GATT	1	215413963	215413995	8.25	.	.	intergenic	0.58	rs787057807	2
AAAC.GTTT	1	215501516	215501531	4	.	.	intergenic	-0.168	rs787393159	2
AC.GT	1	215749522	215749535	7	ENSSSCT00000005632	ER2	intron	0.085	rs792130290	4
AAAAC.GTTTT	1	216098066	216098082	3.4	.	.	intergenic	-0.604	rs787045859	2
AAAC.GTTT	1	216108849	216108861	3.25	.	.	intergenic	0.15	rs792767040	2
AAAC.GTTT	1	216130083	216130109	6.75	.	.	intergenic	0.344	385682 rs7865	2
AGG.CCT	1	216952038	216952049	4	.	.	intergenic	0.014	.	2
AT.AT	1	217479920	217479930	5.5	.	.	intergenic	0.379	rs787755021	2
AG.CT	1	217502675	217502685	5.5	.	.	intergenic	-0.062	rs793082133	2
AG.CT	1	217533612	217533624	6.5	.	.	intergenic	0.559	.	4
AC.GT	1	218472319	218472331	6.5	ENSSSCT00000035816	TEK	intron	-0.058	316373 rs7888	3
AC.GT	1	218472319	218472331	6.5	ENSSSCT00000036581	TEK	intron	-0.058	316373 rs7888	3
AC.GT	1	218472319	218472331	6.5	ENSSSCT00000005647	TEK	intron	-0.058	316373 rs7888	3
AC.GT	1	218472319	218472331	6.5	ENSSSCT00000033502	TEK	intron	-0.058	316373 rs7888	3
AAAAT.ATTTT	1	218578907	218578939	6.6	ENSSSCT00000005649	IFT74	intron	0.223	rs787837741	2
AAAAT.ATTTT	1	218578907	218578939	6.6	ENSSSCT00000005650	LRRC19	intron	0.223	rs787837741	2
AAAATT.AATTTT	1	218980835	218980858	4	.	.	intergenic	.	062069 rs7880	2
AAG.CTT	1	219029074	219029099	8.66667	.	.	intergenic	0.09	rs786951786	2
AC.GT	1	219817655	219817666	6	.	.	intergenic	0.097	rs792790458	2
AAAC.GTTT	1	219886165	219886187	5.75	.	.	intergenic	0.194	rs791638553	2
AT.AT	1	219932429	219932449	10.5	.	.	intergenic	0.067	rs792687691	2
AC.GT	1	220253323	220253333	5.5	.	.	intergenic	.	.	2
AT.AT	1	220293658	220293682	12.5	.	.	intergenic	.	071747 rs7902	2
AT.AT	1	220385436	220385459	12	.	.	intergenic	-0.056	164616 rs7886	2
AAAC.GTTT	1	220746731	220746750	5	.	.	intergenic	.	rs788047428	2
AAAC.GTTT	1	220970674	220970692	4.75	.	.	intergenic	-0.047	rs790268891	2
AG.CT	1	221033521	221033537	8.5	.	.	intergenic	.	293362 rs7884	2
AAC.GTT	1	221245641	221245652	4	.	.	intergenic	0.049	rs792424746	4
ACAT.ATGT	1	221489944	221489966	5.75	.	.	intergenic	.	rs786743081	3
AT.AT	1	221495167	221495179	6.5	.	.	intergenic	0.16	369913 rs7865	3
AAAT.ATTT	1	221516759	221516775	4.25	.	.	intergenic	0.327	rs787912607	2
AG.CT	1	221646071	221646084	7	.	.	intergenic	.	487236 rs7923	2
AT.AT	1	221923773	221923783	5.5	.	.	intergenic	0.319	rs790574856	2
AAAT.ATTT	1	222260416	222260434	4.75	.	.	intergenic	0.044	rs790914660	2
AC.GT	1	222477096	222477108	6.5	.	.	intergenic	-0.13	.	2
AC.GT	1	223010462	223010472	5.5	.	.	intergenic	0.556	.	2
AT.AT	1	223694787	223694799	6.5	.	.	intergenic	.	763147 rs7887	2
AG.CT	1	223695304	223695316	6.5	.	.	intergenic	.	rs793684259	2
AT.AT	1	223952510	223952519	5	.	.	intergenic	.	.	2
AATC.GATT	1	224087266	224087286	5.25	ENSSSCT00000005663	.	intron	.	463242 rs7882	2
AAAC.GTTT	1	224138972	224139002	7.75	.	.	intergenic	0.06	.	2
AG.CT	1	224227937	224227948	6	.	.	intergenic	0.003	.	4
AG.CT	1	224371169	224371183	7.5	.	.	intergenic	0.019	510519 rs7925	2
AAT.ATT	1	224389797	224389811	5	.	.	intergenic	0.287	rs791752331	2
AC.GT	1	225582074	225582083	5	.	.	intergenic	.	.	4
AAC.GTT	1	225677718	225677743	8.66667	.	.	intergenic	0.361	rs791536108	2
AAATT.AATTT	1	225712461	225712475	3	ENSSSCT00000028872	.	intron	.	117541 rs7911	2
AG.CT	1	225848812	225848826	7.5	.	.	intergenic	0.26	187877 rs7934	3
AAAC.GTTT	1	226229551	226229565	3.75	ENSSSCT00000005694	MLLT3	intron	0.066	rs790641846	3
AC.GT	1	226666639	226666667	14.5	ENSSSCT00000005695	SLC24A2	intron	0.307	152378 rs7916	2
AC.GT	1	226712261	226712272	6	ENSSSCT00000005695	SLC24A2	intron	-0.038	rs793039930	2
AC.GT	1	227091879	227091889	5.5	.	.	intergenic	.	.	3
AAAAAC.GTTTTT	1	227233477	227233499	3.83333	ENSSSCT00000005700	DENND4C	intron	.	rs792318383	2
AC.GT	1	227425344	227425367	12	.	.	intergenic	.	372215 rs7937	2
AAAAAG.CTTTTT	1	227433448	227433473	4.33333	.	.	intergenic	0.032	rs787814633	2
AAAC.GTTT	1	227581108	227581124	4.25	.	.	intergenic	0.032	rs793671225	2
AG.CT	1	228027148	228027159	6	.	.	intergenic	.	rs789047047	5
AC.GT	1	228066062	228066076	7.5	.	.	intergenic	0.065	533212 rs7031	4
ACCC.GGGT	1	228162439	228162455	4.25	.	.	intergenic	-0.162	.	2
AAAAC.GTTTT	1	228337478	228337507	6	.	.	intergenic	0.65	025188 rs7926	2
AATG.CATT	1	229009264	229009300	9.25	.	.	intergenic	-0.016	345278 rs7895	2
AAAT.ATTT	1	229045143	229045162	5	.	.	intergenic	-0.114	rs787579796	2
AC.GT	1	229849194	229849224	15.5	ENSSSCT00000031467	.	intron	4.063	rs788979451	2
AAAC.GTTT	1	230414008	230414026	4.75	.	.	intergenic	0.001	rs793752163	2
AAAC.GTTT	1	230431837	230431857	5.25	.	.	intergenic	0.005	rs788327913	2
AG.CT	1	230476385	230476395	5.5	.	.	intergenic	0.505	.	2
AT.AT	1	230583643	230583653	5.5	ENSSSCT00000005713	.	intron	0.025	.	2

ACAC.GTGT	1	230649318	230649356	9.75	ENSSSCT00000005713	.	intron	.	rs793171914	3
AAAC.GTTT	1	230706869	230706899	7.75	.	.	intergenic	.	rs787040612	2
AAAT.ATTT	1	231360462	231360478	4.25	.	.	intergenic	0.21	905140 rs7930	2
AC.GT	1	231521164	231521176	6.5	.	.	intergenic	0.104	.	3
AG.CT	1	231839662	231839673	6	.	.	intergenic	0.075	.	2
AAAC.GTTT	1	231871825	231871844	5	.	.	intergenic	.	rs793528775	2
AAC.GTT	1	231879602	231879621	6.66667	.	.	intergenic	0.015	rs786596121	2
AAAT.ATTT	1	232449039	232449061	5.75	.	.	intergenic	0.238	rs791927119	2
AAAAT.ATTTT	1	232526550	232526576	5.4	.	.	intergenic	-0.183	352848 rs7911	2
AC.GT	1	232551618	232551627	5	.	.	intergenic	-0.326	.	2
AAAAT.ATTTT	1	232625268	232625287	4	.	.	intergenic	0.206	rs789525219	2
AAAC.GTTT	1	232787936	232787957	5.5	.	.	intergenic	0.443	rs791601012	2
AG.CT	1	232912746	232912773	14	.	.	intergenic	-0.211	.	4
AAAC.GTTT	1	233156056	233156078	5.75	ENSSSCT00000005723	MPDZ	intron	-0.109	973183 rs7925	2
AAAAT.ATTTT	1	233306639	233306654	3.2	.	.	intergenic	-0.219	rs792299827	2
AC.GT	1	233961246	233961281	18	.	.	intergenic	0.446	rs787775959	2
AT.AT	1	234056948	234056960	6.5	.	.	intergenic	.	rs792552518	2
AT.AT	1	234090450	234090471	11	.	.	intergenic	0.443	.	2
ACAG.CTGT	1	234175813	234175824	3	.	.	intergenic	.	rs789346276	2
AAAC.GTTT	1	234470007	234470033	6.75	.	.	intergenic	0.016	262712 rs7881	2
AT.AT	1	234497374	234497386	6.5	.	.	intergenic	-0.068	.	3
AT.AT	1	235084062	235084081	10	.	.	intergenic	.	163564 rs7925	2
AC.GT	1	235113287	235113299	6.5	.	.	intergenic	.	.	3
AT.AT	1	235792274	235792305	16	.	.	intergenic	.	rs791961786	2
AAAAG.CTTTT	1	235891542	235891567	5.2	.	.	intergenic	.	.	2
AG.CT	1	236044165	236044182	9	.	.	intergenic	0.025	rs792186575	2
AAAC.GTTT	1	237038149	237038167	4.75	.	.	intergenic	-0.052	rs791027208	2
AG.CT	1	237144009	237144023	7.5	.	.	intergenic	0.019	rs787498964	2
AT.AT	1	237250677	237250688	6	.	.	intergenic	.	rs788071634	2
AAAC.GTTT	1	237863323	237863340	4.5	.	.	intergenic	0.836	rs792541702	2
AAAAC.GTTTT	1	238252553	238252575	4.6	.	.	intergenic	.	.	2
AC.GT	1	238351466	238351478	6.5	.	.	intergenic	0.031	.	2
AT.AT	1	239200162	239200186	12.5	.	.	intergenic	-0.211	994070 rs7889	2
AT.AT	1	239774830	239774859	15	.	.	intergenic	.	.	2
AC.GT	1	239986002	239986022	10.5	.	.	intergenic	0.113	.	2
AAG.CTT	1	240474816	240474833	6	.	.	intergenic	0.071	141105 rs7928	2
AAAC.GTTT	1	240601522	240601547	6.5	.	.	intergenic	.	rs792429494	2
AC.GT	1	240802447	240802458	6	ENSSSCT00000005730	.	intron	0.253	.	6
AT.AT	1	240861131	240861142	6	ENSSSCT00000005730	.	intron	2.917	rs786938690	3
AC.GT	1	240880337	240880351	7.5	ENSSSCT00000005730	.	intron	0.378	rs792139077	2
AAAC.GTTT	1	241247951	241247973	5.75	.	.	intergenic	0.13	rs791760118	2
AAAT.ATTT	1	241704933	241704954	5.5	.	.	intergenic	0.044	rs792519218	2
AT.AT	1	241967279	241967299	10.5	ENSSSCT00000005738	MLANA	intron	.	026367 rs7888	2
AC.GT	1	242411760	242411772	6.5	ENSSSCT00000005744	PDL1	intron	0.11	.	3
AAAC.GTTT	1	242566826	242566848	5.75	.	.	intergenic	.	360500 rs7865	2
AAC.GTT	1	242771010	242771023	4.66667	ENSSSCT00000005748	JAK2	intron	0.061	rs790680260	2
ACT.AGT	1	242779629	242779644	5.33333	ENSSSCT00000005748	JAK2	intron	.	.	2
AT.AT	1	242846964	242846980	8.5	.	.	intergenic	0.384	.	2
AT.AT	1	242872912	242872921	5	.	.	intergenic	.	.	3
AAAC.GTTT	1	243100746	243100765	5	ENSSSCT00000005752	CDC37L1	intron	0.19	rs790369434	2
AC.GT	1	243408097	243408111	7.5	.	.	intergenic	-0.045	rs788067360	4
AAG.CTT	1	243431999	243432024	8.66667	.	.	intergenic	0.296	263228 rs7866	3
AAAAC.GTTTT	1	243692888	243692915	4.66667	.	.	intergenic	.	.	2
AG.CT	1	243696491	243696509	9.5	.	.	intergenic	.	rs788325201	3
AC.GT	1	243966142	243966151	5	ENSSSCT00000005757	GLIS3	intron	0.561	rs788442659	2
AC.GT	1	243966142	243966151	5	ENSSSCT00000005758	GLIS3	intron	0.561	rs788442659	2
AC.GT	1	244075335	244075349	7.5	.	.	intergenic	.	rs791346242	2
AT.AT	1	244096162	244096171	5	.	.	intergenic	0.238	.	3
AC.GT	1	244435177	244435219	21.5	.	.	intergenic	0.15	rs787391441	2
AAAC.GTTT	1	244523078	244523093	4	.	.	intergenic	.	rs788551564	2
AGGGG.CCCCT	1	244599002	244599016	3	.	.	intergenic	0.056	rs790844808	2
ACAT.ATGT	1	245532578	245532595	4.5	ENSSSCT00000005766	SMARCA2	intron	0.026	rs793639137	2
AT.AT	1	245701774	245701785	6	.	.	intergenic	0.24	rs788909449	2
AT.AT	1	245711846	245711855	5	.	.	intergenic	0.017	rs786301555	2
AT.AT	1	245745321	245745331	5.5	.	.	intergenic	0.097	rs692217869	2
AAT.ATT	1	246494633	246494644	4	.	.	intergenic	-0.073	rs789546339	2
AAC.GTT	1	246775741	246775758	6	.	.	intergenic	.	rs789522032	2
AC.GT	1	247134011	247134020	5	.	.	intergenic	-0.158	rs699871035	2
AAAAC.GTTTT	1	247424232	247424254	4.6	ENSSSCT00000005769	.	intron	0.007	685949 rs7901	2
AT.AT	1	247457282	247457293	6	ENSSSCT00000005769	.	intron	-0.078	356829 rs7938	3
AC.GT	1	247564684	247564694	5.5	ENSSSCT00000005774	DOCK8	intron	0.102	rs787608350	2
AAAC.GTTT	1	247697358	247697372	3.75	ENSSSCT00000005774	DOCK8	intron	0.206	113728 rs7908	2
AAAAC.GTTTT	1	247829305	247829323	3.8	.	.	intergenic	.	648152 rs7884	2
AAAC.GTTT	1	247878018	247878036	4.75	.	.	intergenic	.	rs790391453	2
AC.GT	1	248031213	248031223	5.5	ENSSSCT00000005777	PGM5	intron	0.138	.	4
AT.AT	1	248318662	248318671	5	.	.	intergenic	.	rs786921956	2
AAAT.ATTT	1	248328596	248328613	4.5	.	.	intergenic	-0.05	rs789370282	2
AT.AT	1	248619841	248619851	5.5	.	.	intergenic	0.058	rs695132593	2
AC.GT	1	248718751	248718762	6	ENSSSCT00000027067	FXN	intron	.	166570 rs7882	2
AC.GT	1	248718751	248718762	6	ENSSSCT00000005781	TJP2	intron	.	166570 rs7882	2
AAAT.ATTT	1	248738522	248738536	3.75	ENSSSCT00000027067	FXN	intron	1.356	rs790489107	3
AAAT.ATTT	1	248738522	248738536	3.75	ENSSSCT00000005781	TJP2	intron	1.356	rs790489107	3
AAAAC.GTTTT	1	248974175	248974201	4.5	ENSSSCT00000005783	FAM189A2	intron	.	rs791108184	2
AAAC.GTTT	1	249128415	249128433	4.75	.	.	intergenic	-0.333	rs790909030	2

AAT.ATT	1	249236709	249236722	4.66667	.	.	intergenic	0.013	rs791701819	2
AC.GT	1	249418897	249418922	13	.	.	intergenic	0.153	.	4
AAAC.GTTT	1	249525150	249525170	5.25	.	.	intergenic	0.097	rs787553139	2
AATG.CATT	1	249528006	249528029	6	.	.	intergenic	0.055	37784 rs7913	2
AG.CT	1	249653827	249653836	5	.	.	intergenic	.	rs788910759	2
AAAAC.GTTTT	1	249877356	249877372	3.4	ENSSSCT00000022526	.	intron	.	.	2
AT.AT	1	250439790	250439802	6.5	ENSSSCT00000005792	.	intron	.	54485 rs7892	2
AC.GT	1	250548949	250548959	5.5	ENSSSCT00000005792	.	intron	0.508	rs696600665	2
AT.AT	1	250652573	250652591	9.5	.	.	intergenic	0.011	.	2
AC.GT	1	250796974	250796990	8.5	.	.	intergenic	-0.16	.	5
AAC.GTT	1	250907551	250907566	5.33333	.	.	intergenic	0.044	00332 rs7921	2
AC.GT	1	250971063	250971073	5.5	.	.	intergenic	0.282	75181 rs7903	4
AC.GT	1	250995438	250995456	9.5	.	.	intergenic	.	rs790124857	2
AT.AT	1	251244818	251244828	5.5	.	.	intergenic	.	rs792453852	2
AAT.ATT	1	251362325	251362342	6	.	.	intergenic	0.196	rs789509170	2
AAGG.CCTT	1	251624335	251624356	5.5	.	.	intergenic	0.095	rs790048187	2
AG.CT	1	251784811	251784820	5	ENSSSCT00000026837	C9orf85	intron	0.185	.	2
AT.AT	1	251895491	251895510	10	.	.	intergenic	.	rs789192313	2
AC.GT	1	251922983	251922997	7.5	.	.	intergenic	.	rs786877099	2
AAAC.GTTT	1	251982959	251982978	5	.	.	intergenic	.	37391 rs7906	2
AC.GT	1	251999913	251999923	5.5	.	.	intergenic	.	.	4
AG.CT	1	252723742	252723752	5.5	.	.	intergenic	0.073	rs789462631	2
AC.GT	1	252776100	252776109	5	.	.	intergenic	-0.093	.	2
AAAAC.GTTTT	1	252914639	252914664	5.2	.	.	intergenic	-0.009	rs792673784	2
AAAT.ATTT	1	253051200	253051212	3.25	.	.	intergenic	-0.003	12016 rs7878	2
AT.AT	1	253111632	253111643	6	.	.	intergenic	.	rs702742460	4
AAT.ATT	1	253113130	253113143	4.66667	.	.	intergenic	.	71066 rs7873	3
AC.GT	1	253593166	253593175	5	.	.	intergenic	-0.02	.	2
AG.CT	1	253600941	253600953	6.5	.	.	intergenic	-0.214	.	3
AT.AT	1	254031336	254031346	5.5	.	.	intergenic	0.338	.	4
AC.GT	1	254159280	254159294	7.5	.	.	intergenic	-0.064	.	3
AAAC.GTTT	1	254566628	254566655	7	.	.	intergenic	-0.209	rs792903289	2
AATG.CATT	1	254620865	254620887	5.75	ENSSSCT00000005803	RORB	intron	0.095	rs790316738	2
AAAAC.GTTTT	1	254671474	254671492	3.8	ENSSSCT00000005803	RORB	intron	0.908	rs788203651	2
AAAAC.GTTTT	1	254750669	254750697	4.83333	ENSSSCT00000005804	TRPM6	intron	0.193	rs711263056	2
AG.CT	1	254789402	254789417	8	ENSSSCT00000005804	TRPM6	intron	-0.009	rs787072607	2
AAAAT.ATTTT	1	254790145	254790169	5	ENSSSCT00000005804	TRPM6	intron	-0.035	rs792209822	2
AATG.CATT	1	255071859	255071872	3.5	ENSSSCT00000005808	OSTF1	intron	0.3	.	2
AAAC.GTTT	1	255330464	255330499	9	.	.	intergenic	0.03	rs786259474	2
AG.CT	1	255483015	255483029	7.5	.	.	intergenic	.	66360 rs7902	2
GATAT.ATATC	1	255539086	255539122	6.16667	.	.	intergenic	.	96306 rs7868	2
AAAC.GTTT	1	255552880	255552899	5	.	.	intergenic	.	rs792175322	2
AG.CT	1	255576468	255576489	11	.	.	intergenic	0.056	71338 rs7925	2
ACAT.ATGT	1	255660920	255660944	6.25	ENSSSCT00000024731	PCSK5	intron	0.345	rs791410765	2
AATC.GATT	1	255833271	255833294	6	.	.	intergenic	.	rs789507914	2
AT.AT	1	256209346	256209361	8	.	.	intergenic	.	rs787640429	2
AC.GT	1	256264906	256264917	6	.	.	intergenic	-0.347	rs789636832	2
AT.AT	1	256281712	256281747	18	.	.	intergenic	.	.	2
AT.AT	1	256290092	256290122	15.5	.	.	intergenic	.	rs792886975	2
AAAAC.GTTTT	1	256351327	256351348	4.4	ENSSSCT00000005813	PRUNE2	intron	0.387	09520 rs7880	2
AAAC.GTTT	1	256705340	256705358	4.75	.	.	intergenic	-0.008	54834 rs7917	2
AAAAC.GTTTT	1	256766231	256766256	5.2	.	.	intergenic	0.363	28187 rs7868	4
AAAC.GTTT	1	257000970	257000992	5.75	ENSSSCT00000005817	VPS13A	intron	0.056	rs792266996	2
AAAAC.GTTTT	1	257037351	257037374	4.8	ENSSSCT00000005817	VPS13A	intron	.	rs786388844	2
AAAT.ATTT	1	257203942	257203960	4.75	ENSSSCT00000005819	GNA14	intron	-0.198	07180 rs7936	2
AC.GT	1	257256139	257256152	7	ENSSSCT00000005819	GNA14	intron	.	00032 rs7919	4
AC.GT	1	257295261	257295272	6	ENSSSCT00000005819	GNA14	intron	0.191	.	2
AAGAG.CTCTT	1	257584258	257584277	4	.	.	intergenic	0.498	13519 rs7891	2
AG.CT	1	257703587	257703599	6.5	.	.	intergenic	0.046	rs791754582	2
TCCCC.GGGGA	1	257818486	257818509	4	ENSSSCT00000005824	PSAT1	intron	2.169	rs789398984	2
AC.GT	1	257820102	257820124	11.5	ENSSSCT00000005824	PSAT1	intron	-0.172	88211 rs7934	2
AAAC.GTTT	1	257840482	257840510	7.25	.	.	intergenic	0.126	rs789096332	2
AAAG.CTTT	1	257942074	257942095	5.5	.	.	intergenic	-0.042	74865 rs7892	2
AT.AT	1	258233761	258233782	11	.	.	intergenic	.	rs789587549	2
AAAT.ATTT	1	258309934	258309952	4.75	.	.	intergenic	.	rs789410528	2
AC.GT	1	258359134	258359150	8.5	.	.	intergenic	0.146	84886 rs7901	2
AAAC.GTTT	1	258446892	258446907	4	.	.	intergenic	0.034	84397 rs7864	2
AC.GT	1	258522622	258522646	12.5	.	.	intergenic	1.472	06470 rs7921	2
AAGG.CCTT	1	258544900	258544920	5.25	.	.	intergenic	0.062	rs786857248	2
AC.GT	1	259032204	259032214	5.5	.	.	intergenic	1.851	rs791440978	2
AAAC.GTTT	1	259407745	259407759	3.75	.	.	intergenic	0.117	rs791402252	2
AAAC.GTTT	1	259959067	259959094	7	.	.	intergenic	-0.061	rs793461800	2
AC.GT	1	260006911	260006931	10.5	.	.	intergenic	0.117	.	2
AAT.ATT	1	260338753	260338770	6	.	.	intergenic	0.353	50525 rs7136	2
AAAC.GTTT	1	260486784	260486804	5.25	.	.	intergenic	.	rs786872240	2
ACC.GGT	1	260584626	260584660	11.6667	.	.	intergenic	-0.015	rs711012884	4
AAAGG.CCTTT	1	260721999	260722017	3.16667	.	.	intergenic	0.047	11333 rs7918	2
AAC.GTT	1	260725490	260725509	6.66667	.	.	intergenic	0.13	rs791670677	2
ACAT.ATGT	1	261736431	261736458	7	.	.	intergenic	0.513	rs787657392	2
AAAC.GTTT	1	261820417	261820444	7	ENSSSCT00000005828	TLE1	intron	0.312	97019 rs7930	2
AAAT.ATTT	1	262055649	262055670	5.5	.	.	intergenic	-0.002	rs787692642	2
ACCT.AGGT	1	262149642	262149664	5.75	.	.	intergenic	0.223	rs791979606	2
AAAG.CTTT	1	262435099	262435113	3.75	.	.	intergenic	0.15	.	2
AAAC.GTTT	1	262443239	262443261	5.75	.	.	intergenic	0.203	rs788174140	2

AG.CT	1	262617368	262617381	7	.	.	intergenic	.	355894 rs7909	2
AC.GT	1	262713548	262713559	6	.	.	intergenic	0.239	.	3
AC.GT	1	263326543	263326552	5	.	.	intergenic	0.163	rs793751484	2
AAC.GTT	1	263364936	263364953	6	.	.	intergenic	.	rs793742188	2
AAAAC.GTTTT	1	263846716	263846745	6	ENSSSCT00000005843	UNC13B	intron	0.233	rs787910726	2
AT.AT	1	263963666	263963677	6	ENSSSCT00000005845	.	intron	.	rs791555299	3
AG.CT	1	264132460	264132470	5.5	ENSSSCT00000005854	TPM2	intron	0.475	rs793794750	2
AAAG.CTTT	1	264205318	264205339	5.5	.	.	intergenic	.	rs792390355	3
AC.GT	1	264309440	264309450	5.5	.	.	intergenic	-0.066	)11413 rs7881	4
AAC.GTT	1	264328482	264328506	8.33333	.	.	intergenic	.	rs786917869	2
AAAT.ATTT	1	264700907	264700928	5.5	ENSSSCT00000005877	RECK	intron	0.302	rs787193157	2
AAC.GTT	1	264737354	264737368	5	.	.	intergenic	.	.	2
AAC.GTT	1	264973757	264973782	8.66667	.	.	intergenic	-0.035	rs787315253	3
AC.GT	1	265001384	265001394	5.5	.	.	intergenic	.	rs786232991	2
AAAAC.GTTTT	1	265003129	265003144	3.2	.	.	intergenic	0.155	)00363 rs7909	2
AAC.GTT	1	265192041	265192053	4.33333	ENSSSCT00000005883	MELK	intron	0.152	rs788533262	2
AG.CT	1	265235969	265235981	6.5	ENSSSCT00000005883	MELK	intron	.	rs790142761	2
\AAAAC.GTTTT	1	265307146	265307166	3.5	.	.	intergenic	0.221	rs788128816	2
AT.AT	1	265352792	265352813	11	.	.	intergenic	0.168	rs790715294	2
AC.GT	1	265392411	265392435	12.5	.	.	intergenic	-0.324	.	2
AAG.CTT	1	265399580	265399592	4.33333	.	.	intergenic	0.227	rs787448143	2
AAG.CTT	1	265405209	265405233	8.33333	.	.	intergenic	0.644	.	2
AAAC.GTTT	1	265418912	265418931	5	.	.	intergenic	0.956	rs790025999	3
AAAC.GTTT	1	265435029	265435043	3.75	.	.	intergenic	0.075	rs788067110	2
AAAC.GTTT	1	265486462	265486479	4.5	ENSSSCT00000005884	PAX5	intron	.	rs788991063	2
AAAT.ATTT	1	265586830	265586851	5.5	ENSSSCT00000005884	PAX5	intron	0.13	rs787423838	2
AATC.GATT	1	266026484	266026495	3	.	.	intergenic	.	.	2
AAAG.CTTT	1	266234926	266234938	3.25	ENSSSCT00000005889	.	intron	.	rs793669994	2
AAAAC.GTTTT	1	266383443	266383457	3	ENSSSCT00000005892	FRMPD1	intron	0.053	rs790531402	2
AC.GT	1	266435026	266435042	8.5	ENSSSCT00000005892	FRMPD1	intron	1.516	rs792541181	2
AAAC.GTTT	1	266677477	266677503	6.75	.	.	intergenic	-0.007	rs787440082	3
ACAG.CTGT	1	266876858	266876879	5.5	.	.	intergenic	0.351	rs792698595	2
AG.CT	1	266962429	266962440	6	.	.	intergenic	-0.059	rs793372049	2
AC.GT	1	267235082	267235098	8.5	.	.	intergenic	-0.141	rs786939698	2
AAC.GTT	1	267261615	267261631	5.66667	ENSSSCT00000027712	CCDC180	intron	.	rs791532007	4
AATG.CATT	1	267310940	267310972	8.25	ENSSSCT00000027712	CCDC180	intron	-0.081	406422 rs7929	2
AG.CT	1	267402394	267402404	5.5	ENSSSCT00000005904	TDRD7	intron	0.021	rs787468622	3
AAAT.ATTT	1	267405219	267405250	8	ENSSSCT00000005904	TDRD7	intron	0.137	.	2
AAAC.GTTT	1	267591764	267591789	6.5	ENSSSCT00000005906	NCBP1	cds	3.377	rs793548265	2
AAAC.GTTT	1	267591764	267591789	6.5	ENSSSCT00000027470	TMOD1	intron	3.377	rs793548265	2
AAAC.GTTT	1	267591764	267591789	6.5	ENSSSCT00000005906	NCBP1	intron	3.377	rs793548265	2
AAAAC.GTTTT	1	267632306	267632328	4.6	ENSSSCT00000005906	NCBP1	intron	-0.113	rs792019976	2
AAAAC.GTTTT	1	267632306	267632328	4.6	ENSSSCT00000027470	TMOD1	intron	-0.113	rs792019976	2
AC.GT	1	267691007	267691023	8.5	ENSSSCT00000005908	XPA	intron	.	rs788452866	2
AAAAC.GTTTT	1	267918577	267918599	4.6	.	.	intergenic	.	.	2
AAAC.GTTT	1	268067250	268067264	3.75	ENSSSCT00000005913	NANS	intron	.	575527 rs7881	2
AT.AT	1	268075050	268075062	6.5	ENSSSCT00000005913	NANS	intron	.	rs792571617	2
AC.GT	1	268077804	268077814	5.5	ENSSSCT00000005913	NANS	intron	0.126	493746 rs7934	2
AC.GT	1	268174386	268174396	5.5	.	.	intergenic	-0.243	rs793646951	2
AG.CT	1	268214730	268214740	5.5	ENSSSCT00000005916	TBC1D2	intron	-0.183	rs788208773	2
AT.AT	1	268223127	268223138	6	.	.	intergenic	0.154	594296 rs7881	2
AC.GT	1	268284288	268284307	10	ENSSSCT00000025444	.	intron	0.021	.	2
AG.CT	1	268315441	268315450	5	ENSSSCT00000025444	.	intron	-0.199	.	2
AAC.GTT	1	268383105	268383118	4.66667	ENSSSCT00000005917	.	intron	0.157	rs788512954	2
AAAAC.GTTTT	1	268388170	268388193	4.8	ENSSSCT00000005917	.	intron	0.061	.	4
AT.AT	1	268444287	268444313	13.5	ENSSSCT00000005917	.	intron	0.355	rs790663100	2
AAAC.GTTT	1	269309624	269309646	5.75	.	.	intergenic	-0.136	593562 rs7927	2
AG.CT	1	269838617	269838630	7	ENSSSCT00000005925	NOR-1	intron	2.63	rs790788355	2
AT.AT	1	270006928	270006938	5.5	.	.	intergenic	0.131	rs790203602	3
AAAAG.CTTTT	1	270248909	270248930	4.4	ENSSSCT00000005931	INVS	intron	.	rs788083919	2
AT.AT	1	270542615	270542626	6	ENSSSCT00000005933	MSANTD3	intron	.	rs788433485	3
AAAC.GTTT	1	270574196	270574223	7	ENSSSCT00000005934	.	promoter	0.31	.	2
AAAT.ATTT	1	270742806	270742827	5.5	.	.	intergenic	-0.228	299017 rs7927	2
ACAT.ATGT	1	270831601	270831628	7	.	.	intergenic	0.016	rs787611906	2
AAC.GTT	1	270983569	270983582	4.66667	.	.	intergenic	.	rs789231471	2
AG.CT	1	270991754	270991763	5	.	.	intergenic	0.135	.	3
\AAAAT.ATTTT	1	271021687	271021712	4.33333	.	.	intergenic	.	rs790976326	2
AAAC.GTTT	1	271180161	271180183	5.75	.	.	intergenic	0.061	rs789856446	3
AT.AT	1	271357851	271357869	9.5	.	.	intergenic	0.823	rs786495799	2
AT.AT	1	271401980	271401992	6.5	.	.	intergenic	.	rs788845460	3
AC.GT	1	271439660	271439671	6	.	.	intergenic	0.016	.	2
AC.GT	1	271498404	271498417	7	ENSSSCT00000005936	.	intron	-0.027	)04455 rs7934	2
AAAG.CTTT	1	271651716	271651730	3.75	.	.	intergenic	0.007	rs790009438	2
AAAT.ATTT	1	271835521	271835542	5.5	.	.	intergenic	.	356295 rs7896	2
AAAC.GTTT	1	271878992	271879012	5.25	ENSSSCT00000005943	GRIN3A	intron	0.192	rs792118443	2
AT.AT	1	271953083	271953115	16.5	ENSSSCT00000005943	GRIN3A	intron	0.157	rs790922929	7
AC.GT	1	272174674	272174689	8	.	.	intergenic	0.078	503970 rs7884	2
AAT.ATT	1	272233128	272233139	4	.	.	intergenic	0.607	.	2
AAAAG.CTTTT	1	272292274	272292290	3.4	.	.	intergenic	0.044	rs789093622	2
AT.AT	1	272746581	272746591	5.5	.	.	intergenic	.	rs788850838	2
AT.AT	1	272758026	272758043	9	.	.	intergenic	.	.	2
AAAAC.GTTTT	1	272996971	272996986	3.2	.	.	intergenic	-0.145	rs790174932	2
AAT.ATT	1	273024467	273024488	7.33333	.	.	intergenic	0.169	rs791537208	2
AC.GT	1	273061239	273061248	5	.	.	intergenic	.	.	4

AAAAC.GTTTT	1	273085229	273085245	3.4	.	.	intergenic	.	rs791563778	2
AAC.GTT	1	273289902	273289913	4	.	.	intergenic	.	rs793754918	2
AT.AT	1	273616322	273616336	7.5	.	.	intergenic	0.072	rs787501189	2
AAAC.GTTT	1	273625203	273625221	4.75	.	.	intergenic	-0.223	rs793874584	2
AT.AT	1	273673546	273673559	7	.	.	intergenic	-0.048	rs786591841	4
AT.AT	1	273680718	273680729	6	.	.	intergenic	0.086	rs788371582	2
AC.GT	1	274669091	274669115	12.5	.	.	intergenic	0.278	rs786270839	2
AAC.GTT	1	274702357	274702372	5.33333	.	.	intergenic	.	rs696427781	3
AAC.GTT	1	275762746	275762759	4.66667	ENSSSCT00000005966	.	intron	0.048	rs787056651	3
AAT.ATT	1	275883692	275883717	8.66667	.	.	intergenic	0.381	rs787438476	2
AAAAC.GTTTT	1	275918634	275918653	4	.	.	intergenic	0.167	rs786940753	2
AAAT.ATTT	1	275934031	275934064	8.5	.	.	intergenic	0.147	rs793853871	2
AT.AT	1	276122793	276122803	5.5	.	.	intergenic	0.099	383203 rs7869	2
AAT.ATT	1	276228672	276228685	4.66667	ENSSSCT00000034909	SLC44A1	intron	0.188	rs786637055	2
AAT.ATT	1	276228672	276228685	4.66667	ENSSSCT00000033541	SLC44A1	intron	0.188	rs786637055	2
AAT.ATT	1	276228672	276228685	4.66667	ENSSSCT00000033835	SLC44A1	intron	0.188	rs786637055	2
AAAT.ATTT	1	276343188	276343204	4.25	ENSSSCT00000034909	SLC44A1	intron	0.071	rs793358438	2
AAAT.ATTT	1	276343188	276343204	4.25	ENSSSCT00000005968	SLC44A1	intron	0.071	rs793358438	2
AAAT.ATTT	1	276343188	276343204	4.25	ENSSSCT00000033541	SLC44A1	intron	0.071	rs793358438	2
AAAT.ATTT	1	276343188	276343204	4.25	ENSSSCT00000033835	SLC44A1	intron	0.071	rs793358438	2
AGAT.ATCT	1	276921343	276921372	7.5	.	.	intergenic	0.249	371417 rs7888	4
AAAC.GTTT	1	277209481	277209508	7	.	.	intergenic	0.083	rs792208659	4
AT.AT	1	278345247	278345257	5.5	.	.	intergenic	0.096	rs787439489	3
AT.AT	1	278652579	278652589	5.5	.	.	intergenic	0.116	rs790065614	2
AAAC.GTTT	1	278696633	278696655	5.75	.	.	intergenic	0.349	404650 rs7933	2
AAAC.GTTT	1	278721639	278721656	4.5	.	.	intergenic	-0.051	379079 rs7881	2
AC.GT	1	278736955	278736969	7.5	.	.	intergenic	0.35	rs786495878	2
AAC.GTT	1	278919031	278919047	5.66667	ENSSSCT00000005980	RAD23B	intron	.	rs791349395	2
AATG.CATT	1	278981242	278981262	5.25	.	.	intergenic	0.124	rs791643113	2
AT.AT	1	279115732	279115748	8.5	.	.	intergenic	.	rs789305394	2
AT.AT	1	279169476	279169486	5.5	.	.	intergenic	-0.083	.	2
AAAT.ATTT	1	279308067	279308094	7	.	.	intergenic	.	rs789897842	2
AC.GT	1	279316784	279316797	7	.	.	intergenic	0.119	rs787084421	2
AG.CT	1	279373943	279373956	7	.	.	intergenic	-0.01	045498 rs7924	2
AT.AT	1	279530627	279530639	6.5	.	.	intergenic	0.075	.	2
ACAT.ATGT	1	279999791	279999816	6.5	.	.	intergenic	0.553	rs786621692	2
AAAT.ATTT	1	280010475	280010512	9.5	.	.	intergenic	0.221	rs790768977	2
AAT.ATT	1	280096310	280096323	4.66667	.	.	intergenic	0.097	rs790737867	3
AAAC.GTTT	1	280216619	280216642	6	.	.	intergenic	-0.002	rs791761796	4
AG.CT	1	280293377	280293394	9	.	.	intergenic	-0.056	.	2
AAG.CTT	1	280319828	280319845	6	.	.	intergenic	0.144	rs793810552	2
AC.GT	1	280323233	280323243	5.5	.	.	intergenic	-0.009	.	2
AAT.ATT	1	280774865	280774878	4.66667	ENSSSCT00000005990	EPB41L4B	intron	.	rs789057314	2
AT.AT	1	281393587	281393598	6	.	.	intergenic	.	rs790322732	3
AC.GT	1	281423614	281423628	7.5	.	.	intergenic	0.333	.	2
AG.CT	1	281829450	281829459	5	.	.	intergenic	0.121	rs792685861	2
AAAG.CTTT	1	281879360	281879388	7.25	.	.	intergenic	0.202	253050 rs7899	3
AAAT.ATTT	1	282041284	282041300	4.25	ENSSSCT00000005998	TXN	intron	.	098121 rs7927	2
AAAT.ATTT	1	282081817	282081846	7.5	.	.	intergenic	0.072	rs793717781	2
AAAAG.CTTTT	1	282103096	282103116	4.2	.	.	intergenic	0.027	364056 rs7925	2
AAAC.GTTT	1	282166986	282167000	3.75	ENSSSCT00000006000	SVEP1	intron	-0.012	rs789545329	2
ATC.GAT	1	282326357	282326371	5	.	.	intergenic	0.231	280614 rs7901	2
AAAT.ATTT	1	282411724	282411746	5.75	.	.	intergenic	.	rs710638899	2
AAAAAT.ATTTTT	1	282421330	282421347	3	.	.	intergenic	.	306559 rs7883	2
AATT.AATT	1	282488774	282488804	7.75	ENSSSCT00000025482	MUSK	intron	.	rs790848194	2
AATT.AATT	1	282488774	282488804	7.75	ENSSSCT00000006001	MUSK	intron	.	rs790848194	2
ACCT.AGGT	1	282567854	282567866	3.25	.	.	intergenic	0.208	.	2
AATG.CATT	1	282577821	282577862	10.5	.	.	intergenic	0.057	304598 rs7895	2
AAAC.GTTT	1	282891846	282891860	3.75	.	.	intergenic	0.013	rs792713268	2
AAT.ATT	1	282954869	282954887	6.33333	.	.	intergenic	0.306	rs787482397	2
AG.CT	1	283090760	283090773	7	ENSSSCT00000006004	KIAA0368	intron	1.526	519986 rs7898	2
AAC.GTT	1	283092347	283092360	4.66667	ENSSSCT00000006004	KIAA0368	intron	.	rs793404741	2
AAAG.CTTT	1	283178294	283178316	5.75	ENSSSCT00000028358	PTGR1	promoter	.	036130 rs7926	2
AAG.CTT	1	283223732	283223750	6.33333	ENSSSCT00000006005	DNAJC25	intron	0.202	rs789806971	2
AAT.ATT	1	283289688	283289702	5	.	.	intergenic	0.295	.	2
AAAC.GTTT	1	283363874	283363897	6	.	.	intergenic	.	rs792781549	2
AT.AT	1	283461861	283461871	5.5	ENSSSCT00000024726	UGCG	intron	0.236	rs786291314	2
AAAC.GTTT	1	283464372	283464388	4.25	ENSSSCT00000024726	UGCG	intron	0.325	362195 rs7874	2
AAAC.GTTT	1	283477321	283477334	3.5	ENSSSCT00000024726	UGCG	3'utr	1.93	rs787158087	2
AT.AT	1	283733410	283733431	11	ENSSSCT00000006011	SUSD1	intron	0.248	rs788429302	2
AAAT.ATTT	1	283869449	283869471	5.75	.	.	intergenic	.	575160 rs7935	2
AT.AT	1	283882974	283882987	7	.	.	intergenic	0.35	930035 rs7919	2
AAAC.GTTT	1	283899942	283899961	5	.	.	intergenic	.	rs789052328	2
AC.GT	1	284342716	284342742	13.5	ENSSSCT00000006017	SNX30	intron	0.054	rs789017796	3
AAC.GTT	1	284360126	284360145	6.66667	.	.	intergenic	-0.018	312570 rs7888	2
AAT.ATT	1	284404144	284404163	6.66667	.	.	intergenic	.	rs792705242	2
AT.AT	1	284474631	284474640	5	.	.	intergenic	0.19	.	2
AT.AT	1	284517141	284517153	6.5	.	.	intergenic	0.167	rs792495533	2
AAAG.CTTT	1	284570995	284571009	3.75	.	.	intergenic	0.174	288560 rs7898	2
AAAT.ATTT	1	284891476	284891496	5.25	ENSSSCT00000006026	.	intron	-0.064	rs792882540	2
AAC.GTT	1	284963048	284963076	9.66667	ENSSSCT00000006026	.	intron	0.088	rs787802400	2
AC.GT	1	285069130	285069139	5	.	.	intergenic	-0.655	.	3
AAC.GTT	1	285487791	285487802	4	.	.	intergenic	0.262	rs791873223	2
AAAC.GTTT	1	285558479	285558497	4.75	.	.	intergenic	0.311	779104 rs7902	2

AAAT.ATTT	1	285580537	285580559	5.75	.	.	intergenic	-0.284	585862 rs7912	2
AC.GT	1	285670647	285670659	6.5	ENSSSCT00000006030	ZNF618	intron	-0.003	rs791785667	2
AT.AT	1	286276872	286276881	5	.	.	intergenic	0.092	rs791057608	2
AAAC.GTTT	1	286288627	286288639	3.25	.	.	intergenic	.	585712 rs7928	2
AAAC.GTTT	1	286326333	286326348	4	.	.	intergenic	0.809	051780 rs7874	2
AAAC.GTTT	1	286519198	286519221	6	.	.	intergenic	0.159	rs792932811	2
AAC.GTT	1	286807205	286807229	8.33333	ENSSSCT00000023943	.	intron	.	584868 rs7866	2
AAC.GTT	1	286807205	286807229	8.33333	ENSSSCT00000006041	.	intron	.	584868 rs7866	2
AAC.GTT	1	286807205	286807229	8.33333	ENSSSCT00000032138	.	intron	.	584868 rs7866	2
AAAC.GTTT	1	286923768	286923782	3.75	.	.	intergenic	.	rs792050705	2
AG.CT	1	287135699	287135712	7	.	.	intergenic	0.303	rs792386815	2
AAC.GTT	1	287171776	287171795	6.66667	.	.	intergenic	0.285	rs792353633	2
AG.CT	1	287189685	287189721	18.5	.	.	intergenic	0.245	303416 rs7921	3
AC.GT	1	287221589	287221601	6.5	.	.	intergenic	-0.177	013579 rs7900	2
ACAT.ATGT	1	287461760	287461779	5	.	.	intergenic	-0.253	rs788480697	2
AT.AT	1	287509281	287509292	6	.	.	intergenic	-0.157	rs694102459	4
AAGG.CCTT	1	287698199	287698212	3.5	.	.	intergenic	0.038	.	2
AG.CT	1	287711404	287711413	5	.	.	intergenic	-0.013	.	3
AC.GT	1	287730480	287730492	6.5	.	.	intergenic	.	rs789526364	2
AAAC.GTTT	1	287795870	287795890	5.25	.	.	intergenic	.	rs788440889	2
AC.GT	1	288029383	288029392	5	ENSSSCT00000006045	PAPPA	intron	-0.02	.	2
AT.AT	1	288148049	288148058	5	.	.	intergenic	.	rs786610255	2
AAAAG.CTTTT	1	288494238	288494262	5	.	.	intergenic	.	312169 rs7891	2
AT.AT	1	288530434	288530457	12	ENSSSCT00000006050	.	intron	-0.167	512343 rs7883	2
ACGC.GCGT	1	288688171	288688182	3	ENSSSCT00000006050	.	intron	.	749062 rs6976	2
AAAC.GTTT	1	288688537	288688563	6.75	ENSSSCT00000006050	.	intron	.	.	2
AC.GT	1	288760297	288760307	5.5	ENSSSCT00000006050	.	intron	0.001	rs790746535	2
ACAC.GTGT	1	288807693	288807734	10.5	ENSSSCT00000006050	.	intron	0.099	rs793220131	2
AC.GT	1	288947099	288947112	7	.	.	intergenic	0.153	rs710188634	2
AG.CT	1	289224476	289224488	6.5	ENSSSCT00000023014	.	intron	-0.273	rs788950590	2
AG.CT	1	289368182	289368191	5	ENSSSCT00000023014	.	intron	0.066	516996 rs7868	2
AAG.CTT	1	289500062	289500078	5.66667	.	.	intergenic	-0.235	440133 rs7905	2
AC.GT	1	289555814	289555836	11.5	.	.	intergenic	-0.098	rs788481234	2
ATC.GAT	1	289556037	289556055	6.33333	.	.	intergenic	-0.121	rs786703233	2
AC.GT	1	289625280	289625293	7	.	.	intergenic	0.271	rs786812479	2
AC.GT	1	289755390	289755409	10	.	.	intergenic	0.599	rs790469535	3
AAAC.GTTT	1	289758880	289758898	4.75	.	.	intergenic	0.044	rs789446321	2
AAC.GTT	1	289865782	289865796	5	.	.	intergenic	.	rs788026427	2
AT.AT	1	289903713	289903723	5.5	.	.	intergenic	-0.189	555432 rs7937	3
AAC.GTT	1	289919959	289919977	6.33333	.	.	intergenic	0.662	102767 rs7882	2
AT.AT	1	290032652	290032663	6	.	.	intergenic	-0.212	553670 rs7888	5
AC.GT	1	290277736	290277747	6	.	.	intergenic	-0.171	rs791923935	6
AAAC.GTTT	1	290497811	290497825	3.75	.	.	intergenic	0.295	rs787120072	2
AC.GT	1	290530984	290531009	13	.	.	intergenic	.	.	4
AC.GT	1	290677590	290677605	8	.	.	intergenic	0.274	rs791323491	2
AAAAC.GTTTT	1	290728920	290728953	6.8	.	.	intergenic	.	rs790543834	2
AG.CT	1	291071081	291071104	12	.	.	intergenic	.	rs793229422	2
AT.AT	1	291125695	291125705	5.5	.	.	intergenic	-0.051	rs789983271	2
AC.GT	1	291134333	291134344	6	.	.	intergenic	.	.	2
AG.CT	1	291197612	291197623	6	.	.	intergenic	-0.052	rs787666624	2
AAAC.GTTT	1	291251807	291251825	4.75	.	.	intergenic	-0.146	377804 rs7887	2
AAAAAC.GTTTTT	1	291298850	291298877	4.66667	.	.	intergenic	.	541807 rs7892	3
AC.GT	1	291669887	291669900	7	.	.	intergenic	-0.031	.	3
AAAAC.GTTTT	1	291677942	291677968	5.4	.	.	intergenic	0.076	920638 rs7926	2
AGG.CCT	1	291690642	291690658	5.66667	.	.	intergenic	0.095	970044 rs7910	2
AAAC.GTTT	1	291718521	291718561	10.25	.	.	intergenic	-0.093	545386 rs7933	2
AT.AT	1	291898230	291898244	7.5	.	.	intergenic	.	rs787185369	3
AC.GT	1	291967518	291967530	6.5	.	.	intergenic	0.022	rs787632424	2
AG.CT	1	291978400	291978412	6.5	.	.	intergenic	-0.061	rs787155767	2
AATC.GATT	1	291984413	291984440	7	.	.	intergenic	-0.091	598359 rs7908	2
AG.CT	1	291993539	291993551	6.5	.	.	intergenic	1.286	rs790537206	3
AGG.CCT	1	292010135	292010165	10.3333	.	.	intergenic	.	rs790249687	2
AATG.CATT	1	292190872	292190900	7.25	.	.	intergenic	-0.235	rs791571219	2
AGG.CCT	1	292677756	292677767	4	ENSSSCT00000025473	.	intron	-0.293	rs788712895	2
AT.AT	1	292761509	292761521	6.5	ENSSSCT00000006055	MEGF9	intron	0.353	155830 rs7896	2
AG.CT	1	292777473	292777487	7.5	ENSSSCT00000006055	MEGF9	intron	0.081	321603 rs7920	2
AATG.CATT	1	292838423	292838455	8.25	.	.	intergenic	0.021	rs697109109	2
AG.CT	1	292846040	292846049	5	.	.	intergenic	-0.048	.	2
AG.CT	1	292864642	292864652	5.5	.	.	intergenic	.	.	3
AAAC.GTTT	1	292953275	292953295	5.25	ENSSSCT00000006057	.	intron	0.152	rs787821196	2
AT.AT	1	293041864	293041875	6	ENSSSCT00000023007	.	intron	0.095	239028 rs7894	2
AT.AT	1	293041864	293041875	6	ENSSSCT00000006061	.	intron	0.095	239028 rs7894	2
AAAT.ATTT	1	293337997	293338011	3.75	.	.	intergenic	0.085	rs790124314	3
AC.GT	1	293339779	293339792	7	.	.	intergenic	0.289	rs793780291	2
AAAAC.GTTTT	1	293392809	293392828	4	.	.	intergenic	-0.02	.	2
AAT.ATT	1	293488217	293488233	5.66667	ENSSSCT00000006064	RAB14	intron	1.56	458347 rs7920	2
AC.GT	1	293759147	293759164	9	.	.	intergenic	-0.149	rs792204132	2
AAAAT.ATTTT	1	294783616	294783635	4	.	.	intergenic	.	466385 rs7877	2
AT.AT	1	294802876	294802889	7	.	.	intergenic	.	152872 rs7929	2
AG.CT	1	295356903	295356915	6.5	.	.	intergenic	.	rs791801771	2
AT.AT	1	295500128	295500144	8.5	.	.	intergenic	.	.	2
AT.AT	1	295601010	295601021	6	.	.	intergenic	.	.	2
CAGGC.GCCTG	1	296004340	296004359	3.33333	.	.	intergenic	.	rs711021477	2
AG.CT	1	296231881	296231893	6.5	.	.	intergenic	.	rs787907250	2

AG.CT	1	296306983	296306993	5.5	.	.	intergenic	.	rs790386439	3
AG.CT	1	296745361	296745371	5.5	.	.	intergenic	.	.	3
AAC.GTT	1	296765741	296765761	7	.	.	intergenic	.	.	2
AC.GT	1	296808879	296808906	14	.	.	intergenic	.	764711 rs7934	2
AAAC.GTTT	1	296844296	296844313	4.5	.	.	intergenic	.	rs786528438	4
AAAC.GTTT	1	297147722	297147740	4.75	.	.	intergenic	.	014732 rs7923	2
AG.CT	1	297181655	297181678	12	.	.	intergenic	.	rs793402115	3
AAAAC.GTTTT	1	297224903	297224917	3	ENSSSCT00000006127	.	promoter	0.21	rs791630919	2
AT.AT	1	297443813	297443824	6	ENSSSCT00000023452	.	promoter	0.114	.	3
AAAG.CTTT	1	297473997	297474021	6.25	ENSSSCT00000023452	.	intron	0.172	rs787634728	2
AG.CT	1	297579969	297579991	11.5	ENSSSCT00000006135	STRBP	intron	0.318	.	3
AC.GT	1	297604061	297604071	5.5	ENSSSCT00000006135	STRBP	intron	0.27	.	2
AAC.GTT	1	297724275	297724294	6.66667	.	.	intergenic	.	rs792626000	2
AAAC.GTTT	1	297734558	297734571	3.5	.	.	intergenic	.	rs790101678	4
AGG.CCT	1	298064435	298064451	5.66667	.	.	intergenic	-0.031	.	2
AAT.ATT	1	298417588	298417600	4.33333	ENSSSCT00000006139	DENND1A	intron	.	rs789979951	2
AAAC.GTTT	1	298643920	298643932	3.25	.	.	intergenic	0.092	rs792727121	2
AAAC.GTTT	1	299018728	299018747	5	.	.	intergenic	.	090514 rs7927	2
AC.GT	1	299069742	299069770	14.5	.	.	intergenic	0.232	.	2
AAAC.GTTT	1	299082218	299082244	6.75	ENSSSCT00000006145	NR6A1	intron	.	.	2
AAAC.GTTT	1	299338165	299338179	3.75	ENSSSCT00000029437	NR6A1	intron	0.486	589655 rs7904	2
AAAAC.GTTTT	1	299377781	299377801	4.2	ENSSSCT00000033935	NR6A1	intron	0.459	rs791455385	2
AAAAC.GTTTT	1	299377781	299377801	4.2	ENSSSCT00000029437	NR6A1	intron	0.459	rs791455385	2
AC.GT	1	299448983	299448993	5.5	.	.	intergenic	0.349	rs793150925	2
AAAT.ATTT	1	299664011	299664036	6.5	.	.	intergenic	-0.054	rs788479754	2
AC.GT	1	299734059	299734070	6	.	.	intergenic	0.25	rs793648857	2
AAC.GTT	1	299755357	299755369	4.33333	ENSSSCT00000006159	HSPA5	intron	4.587	111438 rs7876	2
AC.GT	1	299824668	299824681	7	ENSSSCT00000006160	GAPVD1	intron	-0.069	rs792877127	4
AC.GT	1	299827524	299827545	11	ENSSSCT00000006160	GAPVD1	intron	0.042	rs705994651	4
AC.GT	1	299889118	299889132	7.5	ENSSSCT00000006160	GAPVD1	intron	0.368	rs791340267	4
AAAAC.GTTTT	1	299892976	299892996	4.2	ENSSSCT00000006160	GAPVD1	intron	0.145	rs791521330	2
AAAAAC.GTTTTT	1	299944209	299944233	4.16667	ENSSSCT00000006160	GAPVD1	intron	0.122	rs791040032	2
AAAAAG.CTTTTT	1	300171334	300171354	3.5	ENSSSCT00000027058	.	intron	0.121	rs786869189	3
AAAT.ATTT	1	300178057	300178074	4.5	ENSSSCT00000027058	.	promoter	0.232	rs786859504	2
AT.AT	1	300341631	300341642	6	ENSSSCT00000006162	PBX3	intron	0.252	787970 rs7925	2
AC.GT	1	300363853	300363869	8.5	ENSSSCT00000006162	PBX3	intron	2.358	491117 rs7877	2
AC.GT	1	300388316	300388325	5	ENSSSCT00000006162	PBX3	intron	0.216	582310 rs7876	2
AAAAC.GTTTT	1	300808794	300808812	3.8	.	.	intergenic	0.162	376713 rs7867	3
AAAC.GTTT	1	300827048	300827063	4	.	.	intergenic	1.472	369055 rs7865	2
AG.CT	1	300873670	300873686	8.5	.	.	intergenic	.	rs791464182	2
AAC.GTT	1	300901071	300901087	5.66667	.	.	intergenic	-0.5	.	2
AT.AT	1	301017333	301017344	6	.	.	intergenic	-0.441	rs790576264	4
ATCC.GGAT	1	301185432	301185452	5.25	.	.	intergenic	-0.228	rs787745189	2
AC.GT	1	301197891	301197909	9.5	.	.	intergenic	0.439	rs788722446	2
AAAAT.ATTTT	1	301291059	301291087	5.8	.	.	intergenic	0.261	rs789161390	2
AAGG.CCTT	1	301294104	301294133	7.5	.	.	intergenic	-0.237	.	2
AAAT.ATTT	1	301380062	301380076	3.75	.	.	intergenic	.	rs793314843	2
AAACC.GGTTT	1	301691448	301691474	5.4	.	.	intergenic	0.062	rs789195748	2
AAC.GTT	1	301822455	301822471	5.66667	ENSSSCT00000006168	.	intron	1.286	rs791456528	2
AC.GT	1	302064138	302064148	5.5	ENSSSCT00000006177	STXBP1	intron	0.576	rs788473073	2
AG.CT	1	302391661	302391672	6	.	.	intergenic	-0.008	.	2
AAAC.GTTT	1	302724749	302724766	4.5	.	.	intergenic	.	rs792959720	2
AC.GT	1	302810153	302810163	5.5	ENSSSCT00000006207	.	intron	0.281	rs789003993	2
AAAT.ATTT	1	303185103	303185122	5	ENSSSCT00000006225	C9orf114	promoter	.	rs789065348	2
AAAC.GTTT	1	303448136	303448153	4.5	.	.	intergenic	-0.344	rs793282743	2
ACAG.CTGT	1	303622789	303622806	4.5	.	.	intergenic	-0.333	rs793193415	2
AAAT.ATTT	1	304007127	304007145	4.75	ENSSSCT00000006256	FNBP1	intron	0.148	rs790913661	2
AT.AT	1	304252498	304252510	6.5	.	.	intergenic	0.093	rs788245287	3
AG.CT	1	304508171	304508187	8.5	ENSSSCT00000006270	ASS1	intron	0.048	.	3
ATCC.GGAT	1	304569547	304569588	10.5	.	.	intergenic	.	484992 rs7938	3
AC.GT	1	304577406	304577440	17.5	ENSSSCT00000006271	FUBP3	intron	0.222	rs789082484	4
AGG.CCT	1	304622235	304622249	5	ENSSSCT00000006271	FUBP3	intron	-0.633	rs791878078	2
AAAT.ATTT	1	304990828	304990845	4.5	ENSSSCT00000006279	LAMC3	intron	.	rs791500988	2
AAAT.ATTT	1	304990828	304990845	4.5	ENSSSCT00000006282	LAMC3	intron	.	rs791500988	2
AAAT.ATTT	1	305442780	305442805	6.5	ENSSSCT00000006286	POMT1	intron	-0.29	rs786331726	2
AG.CT	1	306192405	306192423	9.5	ENSSSCT00000006294	SETX	intron	.	rs786801295	2
AAC.GTT	1	306224057	306224069	4.33333	ENSSSCT00000006294	SETX	intron	1.09	rs793595646	2
AAAAC.GTTTT	1	306299923	306299944	4.4	.	.	intergenic	-0.262	487055 rs7889	2
AG.CT	1	306571573	306571600	14	.	.	intergenic	1.652	540137 rs7906	2
AC.GT	1	307007849	307007860	6	ENSSSCT00000022734	ADAMTS13	intron	1.68	rs787239809	2
AAAAAG.CTTTTT	1	307280607	307280625	3.16667	ENSSSCT00000006315	VAV2	intron	0.019	rs786570512	2
AC.GT	1	308233598	308233607	5	.	.	intergenic	-0.307	.	2
AG.CT	1	309977506	309977520	7.5	.	.	intergenic	.	rs786517412	2
AAAAC.GTTTT	1	310072676	310072697	4.4	ENSSSCT00000006349	RBFA	intron	.	rs793227167	2
AAC.GTT	1	310137370	310137387	6	ENSSSCT00000006353	TXNL4A	intron	.	rs792914287	2
AAAT.ATTT	1	310139339	310139356	4.5	ENSSSCT00000006353	TXNL4A	intron	.	rs791733339	2
AG.CT	1	310833055	310833067	6.5	.	.	intergenic	.	rs789655674	2
AGAT.ATCT	1	310847880	310847891	3	.	.	intergenic	.	rs792442818	2
AAAT.ATTT	1	310858723	310858749	6.75	.	.	intergenic	.	rs790857695	2
AAAC.GTTT	1	311076812	311076842	7.75	ENSSSCT00000006357	TARSL2	3'utr	0.484	rs695509989	2
AC.GT	1	311142963	311142977	7.5	.	.	intergenic	.	rs788713628	3
AAAC.GTTT	1	311336146	311336174	7.25	ENSSSCT00000029763	.	promoter	.	907273 rs7929	2
AAAG.CTTT	1	311897732	311897744	3.25	.	.	intergenic	.	rs789177870	2
AAT.ATT	1	311929960	311929988	9.66667	.	.	intergenic	.	rs792376082	2

AC.GT	1	312205548	312205562	7.5	ENSSSCT00000006371	OR10AG1	promoter	.	rs791844213	2
AC.GT	1	312264615	312264635	10.5	ENSSSCT00000006373	.	cds	.	459435 rs7931	2
AAAAT.ATTTT	1	312300085	312300103	3.8	.	.	intergenic	.	.	2
AAAT.ATTT	1	312333791	312333808	4.5	.	.	intergenic	.	rs699408104	2
AC.GT	1	312357739	312357750	6	ENSSSCT00000006377	.	promoter	-0.074	rs793230177	2
AT.AT	1	312361685	312361696	6	.	.	intergenic	.	708862 rs7895	2
AAT.ATT	1	312607522	312607535	4.66667	.	.	intergenic	.	rs790484225	2
AG.CT	1	312826748	312826758	5.5	.	.	intergenic	.	.	4
AT.AT	1	313147182	313147192	5.5	ENSSSCT00000006399	.	promoter	.	rs790166632	2
AC.GT	1	313505206	313505224	9.5	.	.	intergenic	.	rs789508903	3
AAC.GTT	1	314849237	314849248	4	.	.	intergenic	.	312056 rs7900	3
AG.CT	1	315205481	315205500	10	.	.	intergenic	.	543382 rs7907	2
AAAAC.GTTTT	1	315209256	315209277	4.4	.	.	intergenic	.	rs786282086	2
AG.CT	1	315314217	315314240	12	.	.	intergenic	.	.	3
AAAT.ATTT	10	45277	45300	6	.	.	intergenic	.	rs792373976	2
AAAC.GTTT	10	51881	51903	5.75	ENSSSCT00000011811	NDUFAB1	intron	0.098	rs791455171	2
AAAAC.GTTTT	10	74596	74618	4.6	ENSSSCT00000011812	UBFD1	3'utr	4.025	rs786218814	2
AAAC.GTTT	10	183123	183135	3.25	ENSSSCT00000011815	COG7	intron	.	rs786755422	2
AAAG.CTTT	10	208539	208557	4.75	ENSSSCT00000011815	COG7	intron	.	rs789022093	2
AC.GT	10	518496	518526	15.5	.	.	intergenic	.	144473 rs7891	4
AC.GT	10	579486	579496	5.5	.	.	intergenic	.	rs786886041	4
AC.GT	10	861830	861843	7	.	.	intergenic	.	.	2
AAAAC.GTTTT	10	862125	862144	4	.	.	intergenic	.	217621 rs7911	3
ACAT.ATGT	10	876903	876914	3	.	.	intergenic	0.004	.	3
AAAT.ATTT	10	879528	879552	6.25	.	.	intergenic	.	rs791031652	2
AAC.GTT	10	926935	926950	5.33333	.	.	intergenic	-0.153	rs791386715	2
AAAC.GTTT	10	1096889	1096907	4.75	.	.	intergenic	.	910257 rs7928	2
AT.AT	10	1675392	1675405	7	.	.	intergenic	0.051	rs788357198	4
AAAT.ATTT	10	1701587	1701603	4.25	.	.	intergenic	-0.233	rs789265533	2
AT.AT	10	1721260	1721283	12	.	.	intergenic	-0.086	.	4
AT.AT	10	2091846	2091858	6.5	.	.	intergenic	.	rs790664076	2
AAAAAG.CTTTTT	10	2158501	2158534	5.66667	.	.	intergenic	0.169	rs787548172	2
AAAC.GTTT	10	2311719	2311730	3	.	.	intergenic	0.116	rs790076880	2
AG.CT	10	2401047	2401059	6.5	ENSSSCT00000011818	.	intron	0.259	.	3
AG.CT	10	2487658	2487670	6.5	.	.	intergenic	.	rs787028695	2
AAAC.GTTT	10	2690250	2690272	5.75	.	.	intergenic	-0.158	rs789099648	4
AAG.CTT	10	2783087	2783101	5	.	.	intergenic	.	rs792532332	2
AAC.GTT	10	2827044	2827057	4.66667	.	.	intergenic	-0.141	rs790105288	2
AC.GT	10	3276260	3276282	11.5	.	.	intergenic	-0.222	rs788196815	3
AAAT.ATTT	10	3551073	3551096	6	.	.	intergenic	-0.057	586692 rs6958	2
AAAC.GTTT	10	3773778	3773795	4.5	.	.	intergenic	.	rs790238737	2
AAGG.CCTT	10	3830249	3830277	7.25	.	.	intergenic	0.06	.	2
AAC.GTT	10	3950750	3950768	6.33333	.	.	intergenic	-0.328	rs786477753	2
AT.AT	10	4026232	4026241	5	.	.	intergenic	0.166	rs789575508	2
AAAC.GTTT	10	4184355	4184369	3.75	.	.	intergenic	0.086	rs786741293	2
AT.AT	10	4257892	4257906	7.5	.	.	intergenic	.	rs793079454	3
AAAC.GTTT	10	4362296	4362308	3.25	.	.	intergenic	.	rs787121554	2
AC.GT	10	4911952	4911966	7.5	.	.	intergenic	.	rs790773764	4
AG.CT	10	4929648	4929659	6	.	.	intergenic	0.327	rs789468717	3
ACT.AGT	10	4933803	4933819	5.66667	.	.	intergenic	-0.167	.	2
AGG.CCT	10	4933852	4933871	6.66667	.	.	intergenic	-0.207	.	2
AC.GT	10	5108443	5108456	7	ENSSSCT00000011825	BRINP3	intron	-0.107	202663 rs7869	2
ACAT.ATGT	10	5132219	5132237	4.75	ENSSSCT00000011825	BRINP3	intron	0	rs793420717	2
AAAC.GTTT	10	5181949	5181970	5.5	ENSSSCT00000011825	BRINP3	intron	0.335	906470 rs7931	2
AC.GT	10	5204906	5204931	13	.	.	intergenic	-0.162	rs787420647	2
AC.GT	10	5410462	5410474	6.5	.	.	intergenic	-0.099	350471 rs7892	2
AT.AT	10	5838717	5838726	5	.	.	intergenic	-0.207	.	2
AAAC.GTTT	10	5868245	5868260	4	.	.	intergenic	-0.081	329724 rs7920	2
AAC.GTT	10	5874152	5874171	6.66667	.	.	intergenic	-0.139	rs791540704	2
ACAT.ATGT	10	6012990	6013009	5	.	.	intergenic	-0.136	.	3
AAC.GTT	10	6035977	6035993	5.66667	.	.	intergenic	-0.079	102995 rs7928	2
AAG.CTT	10	6051977	6051989	4.33333	.	.	intergenic	0.223	515745 rs7888	2
AG.CT	10	6282297	6282331	17.5	.	.	intergenic	-0.107	340880 rs7874	2
AC.GT	10	6388369	6388379	5.5	.	.	intergenic	-0.175	rs787569450	2
AAAAC.GTTTT	10	7032655	7032673	3.8	.	.	intergenic	-0.088	rs791859959	2
AAC.GTT	10	7033545	7033561	5.66667	.	.	intergenic	-0.192	.	3
AC.GT	10	7118711	7118720	5	.	.	intergenic	.	.	2
AG.CT	10	7145045	7145059	7.5	.	.	intergenic	0.036	050539 rs7913	2
AGG.CCT	10	7178559	7178572	4.66667	.	.	intergenic	-0.125	567829 rs7095	2
AAAC.GTTT	10	7263139	7263161	5.75	.	.	intergenic	-0.009	rs792531132	2
AT.AT	10	7278949	7278966	9	.	.	intergenic	.	.	2
AAC.GTT	10	7300614	7300628	5	.	.	intergenic	-0.185	rs791188810	3
AATC.GATT	10	7320196	7320207	3	.	.	intergenic	-0.163	rs790170023	2
AAAC.GTTT	10	7593401	7593416	4	.	.	intergenic	.	rs790557827	2
AC.GT	10	7878020	7878032	6.5	.	.	intergenic	0.297	rs792715015	2
AG.CT	10	7888261	7888275	7.5	.	.	intergenic	0.043	rs790813199	2
AAC.GTT	10	8012796	8012819	8	.	.	intergenic	.	203712 rs7909	4
AATG.CATT	10	8199005	8199032	7	.	.	intergenic	-0.049	552233 rs7908	2
AAAC.GTTT	10	8317091	8317109	4.75	.	.	intergenic	-0.238	rs790050469	2
AAAAC.GTTTT	10	8407285	8407300	3.2	.	.	intergenic	.	197562 rs7890	2
AC.GT	10	8465879	8465890	6	.	.	intergenic	.	.	2
AAAAT.ATTTT	10	8577080	8577103	4.8	.	.	intergenic	-0.146	rs791053155	2
AAAAC.GTTTT	10	8652363	8652387	5	ENSSSCT00000011830	ESRRG	intron	0.125	rs790156236	2
AC.GT	10	8783517	8783528	6	ENSSSCT00000011830	ESRRG	intron	0.063	rs787869528	2

AAT.ATT	10	8811335	8811349	5	ENSSSCT00000011830	ESRRG	intron	-0.026	rs707590475	2
AAAT.ATTT	10	8885213	8885232	5	.	.	intergenic	-0.18	rs790819958	2
AC.GT	10	8947357	8947369	6.5	.	.	intergenic	0.991	371854 rs7911	2
AGAT.ATCT	10	9090155	9090176	5.5	.	.	intergenic	-0.118	374061 rs7911	4
AT.AT	10	9093616	9093637	11	.	.	intergenic	0.249	rs788783901	4
AAGG.CCTT	10	9155413	9155426	3.5	.	.	intergenic	-0.33	.	4
AG.CT	10	9295064	9295076	6.5	.	.	intergenic	-0.342	rs786767916	2
AG.CT	10	9361545	9361554	5	.	.	intergenic	-0.101	.	2
AT.AT	10	9374394	9374406	6.5	.	.	intergenic	0.083	rs788304655	3
AG.CT	10	9412632	9412642	5.5	.	.	intergenic	-0.2	rs791458282	2
AAT.ATT	10	9443807	9443823	5.66667	.	.	intergenic	0.042	349376 rs7873	2
ACC.GGT	10	9530761	9530785	8.33333	.	.	intergenic	0.069	068934 rs7889	2
AATG.CATT	10	9711455	9711481	6.75	ENSSSCT00000025633	GPATCH2	intron	0.652	rs789479694	2
AT.AT	10	9725585	9725594	5	ENSSSCT00000025633	GPATCH2	intron	-0.036	.	2
AT.AT	10	9733545	9733555	5.5	ENSSSCT00000025633	GPATCH2	intron	1.214	rs793307899	2
AC.GT	10	9772882	9772902	10.5	ENSSSCT00000025633	GPATCH2	intron	-0.225	rs786323567	3
AG.CT	10	9892614	9892627	7	ENSSSCT00000026746	SPATA17	intron	0.146	rs792441139	2
AT.AT	10	9955964	9955988	12.5	ENSSSCT00000026746	SPATA17	intron	-0.136	.	2
AAAG.CTTT	10	10005953	10005969	4.25	.	.	intergenic	1.408	.	2
AAAAC.GTTTT	10	10027488	10027504	3.4	.	.	intergenic	.	rs792739626	2
AG.CT	10	10132674	10132684	5.5	.	.	intergenic	-0.063	.	2
AC.GT	10	10143409	10143425	8.5	.	.	intergenic	.	rs793105625	5
AAAAC.GTTTT	10	10249604	10249625	4.4	.	.	intergenic	.	rs793627903	3
AC.GT	10	10419206	10419217	6	ENSSSCT00000011833	TGFB2	intron	-0.281	rs789976916	3
AAC.GTT	10	10578024	10578040	5.66667	.	.	intergenic	0.688	rs789938844	2
AC.GT	10	10636105	10636115	5.5	.	.	intergenic	-0.202	rs695037902	3
AAAC.GTTT	10	10702368	10702382	3.75	.	.	intergenic	.	rs793265678	2
AAAC.GTTT	10	10982823	10982841	4.75	.	.	intergenic	-0.024	rs793031380	2
AAAC.GTTT	10	11121634	11121653	5	.	.	intergenic	.	rs791078934	2
AG.CT	10	11232724	11232734	5.5	.	.	intergenic	0.103	573643 rs7925	3
AAG.CTT	10	11448812	11448825	4.66667	.	.	intergenic	-0.053	rs790299823	2
AAC.GTT	10	11466193	11466204	4	.	.	intergenic	0.009	rs791721078	2
AC.GT	10	11512468	11512480	6.5	.	.	intergenic	0.248	.	4
AAC.GTT	10	11546640	11546658	6.33333	.	.	intergenic	-0.036	rs791811106	2
AAAAG.CTTTT	10	11982895	11982916	4.4	.	.	intergenic	-0.13	rs792026381	2
AAAAC.GTTTT	10	12000598	12000613	3.2	.	.	intergenic	0.213	.	2
AAAT.ATTT	10	12115803	12115821	4.75	.	.	intergenic	.	320260 rs7864	2
AAAAC.GTTTT	10	12291980	12292002	4.6	ENSSSCT00000011847	1-Mar	intron	.	rs789563382	3
AC.GT	10	12499149	12499180	16	.	.	intergenic	-0.23	rs789824821	4
AAAC.GTTT	10	12747432	12747446	3.75	.	.	intergenic	-0.165	945240 rs7892	2
AAAAT.ATTTT	10	12931770	12931792	4.6	.	.	intergenic	-0.159	rs790836235	2
AAAC.GTTT	10	13043059	13043073	3.75	.	.	intergenic	-0.109	rs787538384	2
AGC.GCT	10	13050995	13051008	4.66667	.	.	intergenic	.	rs789025652	2
AAAG.CTTT	10	13110005	13110020	4	.	.	intergenic	-0.009	042582 rs7928	2
AC.GT	10	13157205	13157215	5.5	.	.	intergenic	-0.203	.	2
AC.GT	10	13214057	13214071	7.5	.	.	intergenic	0.093	399172 rs7929	2
AG.CT	10	13231867	13231878	6	.	.	intergenic	-0.081	.	2
AC.GT	10	13259906	13259917	6	.	.	intergenic	-0.032	.	4
AAAC.GTTT	10	13267265	13267292	7	.	.	intergenic	0.874	715607 rs7894	2
AAAC.GTTT	10	13324777	13324797	5.25	.	.	intergenic	-0.008	rs787490101	2
AC.GT	10	13365961	13365975	7.5	.	.	intergenic	.	331426 rs6945	2
AAAAC.GTTTT	10	13400645	13400669	5	.	.	intergenic	.	531210 rs7910	2
AT.AT	10	13616368	13616379	6	ENSSSCT00000011854	AIDA	intron	0.053	rs793846089	2
AGAT.ATCT	10	13655956	13655969	3.5	.	.	intergenic	0.153	.	2
AAAT.ATTT	10	13660845	13660859	3.75	.	.	intergenic	0.194	rs786287104	2
AAAAT.ATTTT	10	14020510	14020538	5.8	ENSSSCT00000011858	.	intron	0.374	799700 rs7874	2
AATG.CATT	10	14751923	14751941	4.75	.	.	intergenic	-0.113	rs786703479	2
AAAC.GTTT	10	14963667	14963693	6.75	ENSSSCT00000029068	ZNF596	intron	0.159	rs793337367	2
AAAC.GTTT	10	14980771	14980793	5.75	.	.	intergenic	.	rs793834344	2
AT.AT	10	14987802	14987813	6	.	.	intergenic	.	084828 rs7901	3
AAAT.ATTT	10	15045968	15045990	5.75	ENSSSCT00000025010	DEGS1	intron	-0.069	081344 rs7885	2
AC.GT	10	15142079	15142090	6	ENSSSCT00000011871	WDR26	intron	-0.265	340518 rs7873	2
AAT.ATT	10	15164865	15164881	5.66667	ENSSSCT00000011871	WDR26	intron	0.314	rs789333623	2
AAC.GTT	10	15169584	15169598	5	ENSSSCT00000011871	WDR26	intron	0.049	194882 rs7926	3
AAAAC.GTTTT	10	15207380	15207404	5	.	.	intergenic	0.036	rs692419319	2
AAAAT.ATTTT	10	15314950	15314967	3.6	ENSSSCT00000027767	CNIH3	intron	0.015	rs712705666	2
AAC.GTT	10	15319411	15319424	4.66667	ENSSSCT00000027767	CNIH3	intron	0.142	rs787654037	2
AG.CT	10	15633769	15633783	7.5	ENSSSCT00000024654	.	intron	0.275	.	2
AAC.GTT	10	15656879	15656895	5.66667	ENSSSCT00000024654	.	intron	.	916037 rs7909	2
AT.AT	10	15707441	15707451	5.5	ENSSSCT00000029429	.	intron	0.201	rs789508405	2
AAAT.ATTT	10	15899251	15899265	3.75	ENSSSCT00000011880	SDE2	intron	0.014	530922 rs7905	2
AAAT.ATTT	10	15964402	15964426	6.25	.	.	intergenic	.	343949 rs7886	3
AAAC.GTTT	10	16207111	16207125	3.75	.	.	intergenic	0.025	.	2
AAAC.GTTT	10	16403436	16403450	3.75	.	.	intergenic	.	.	2
AAT.ATT	10	16516611	16516630	6.66667	.	.	intergenic	.	rs793058454	2
AC.GT	10	16762421	16762461	20.5	ENSSSCT00000028613	ITPKB	intron	-0.225	rs788617966	2
AAAC.GTTT	10	16828804	16828826	5.75	.	.	intergenic	0.463	rs790876086	2
AG.CT	10	16851009	16851022	7	.	.	intergenic	.	.	4
AAC.GTT	10	16991445	16991458	4.66667	ENSSSCT00000011884	CDC42BPA	intron	1.345	rs696314909	2
AC.GT	10	17766994	17767004	5.5	ENSSSCT00000011891	PLD5	intron	0.043	385695 rs7055	3
AAAC.GTTT	10	17846363	17846377	3.75	.	.	intergenic	0.219	rs790619580	2
AC.GT	10	17970243	17970256	7	ENSSSCT00000011894	AKT3	intron	0.007	rs787597184	2
AC.GT	10	18017369	18017379	5.5	ENSSSCT00000011894	AKT3	intron	0.112	.	2
AAAG.CTTT	10	18343206	18343224	4.75	ENSSSCT00000028157	.	intron	.	550219 rs7866	2

AC.GT	10	19030750	19030775	13	.	.	intergenic	0.082	.	3
AAAAC.GTTTT	10	19060492	19060514	4.6	.	.	intergenic	.	rs792851272	2
AC.GT	10	19083231	19083262	16	.	.	intergenic	.	.	3
AC.GT	10	19113272	19113284	6.5	.	.	intergenic	-0.33	rs787023993	2
AAT.ATT	10	19285308	19285328	7	.	.	intergenic	0.658	.	2
AAGAG.CTCTT	10	19290095	19290127	6.6	.	.	intergenic	0.012	266415 rs7899	2
AAC.GTT	10	19494059	19494079	7	.	.	intergenic	-0.072	rs791604414	2
AAAC.GTTT	10	19721761	19721795	8.75	ENSSSCT00000011898	DESI2	intron	0.286	rs789435300	2
AAAT.ATTT	10	19839670	19839682	3.25	.	.	intergenic	-0.125	.	2
AAAT.ATTT	10	20147358	20147377	5	.	.	intergenic	0.005	rs691945452	2
AAAAAT.ATTTTT	10	20344443	20344461	3.16667	.	.	intergenic	-0.252	rs787630331	2
AC.GT	10	20382343	20382354	6	.	.	intergenic	0.001	rs792471802	3
AAAC.GTTT	10	20415968	20415987	5	ENSSSCT00000011902	KIF26B	intron	0.036	rs787097923	2
AAAC.GTTT	10	20415968	20415987	5	ENSSSCT00000026658	KIF26B	intron	0.036	rs787097923	2
AAAG.CTTT	10	20633614	20633629	4	ENSSSCT00000011902	KIF26B	intron	0.251	rs790802809	2
AAAG.CTTT	10	20633614	20633629	4	ENSSSCT00000026658	KIF26B	intron	0.251	rs790802809	2
AAAG.CTTT	10	20633614	20633629	4	ENSSSCT00000022970	.	promoter	0.251	rs790802809	2
AG.CT	10	20846287	20846308	11	.	.	intergenic	0.475	.	2
AAC.GTT	10	21128379	21128391	4.33333	.	.	intergenic	.	rs790180302	2
AG.CT	10	21320237	21320248	6	.	.	intergenic	.	rs791021197	2
AG.CT	10	21478014	21478026	6.5	ENSSSCT00000032587	.	intron	.	rs789416626	2
AC.GT	10	21544872	21544884	6.5	ENSSSCT00000032587	.	intron	.	.	2
AG.CT	10	21660657	21660668	6	ENSSSCT00000011908	.	intron	.	rs790051654	2
AG.CT	10	21660657	21660668	6	ENSSSCT00000025284	.	intron	.	rs790051654	2
AAAC.GTTT	10	21805698	21805716	4.75	.	.	intergenic	.	rs791237464	3
AAC.GTT	10	21830700	21830719	6.66667	.	.	intergenic	0.104	rs791240903	2
AG.CT	10	22046115	22046128	7	ENSSSCT00000011910	.	intron	0.296	.	4
ACAG.CTGT	10	22056932	22056947	4	.	.	intergenic	-0.16	.	2
AT.AT	10	22076577	22076597	10.5	.	.	intergenic	0.037	.	2
AC.GT	10	23483977	23483989	6.5	.	.	intergenic	.	rs789506852	2
AC.GT	10	23563738	23563748	5.5	.	.	intergenic	.	.	2
AAAAT.ATTTT	10	23575950	23575965	3.2	.	.	intergenic	0.064	rs787339688	2
AT.AT	10	23680614	23680627	7	.	.	intergenic	0.02	332796 rs7913	2
AT.AT	10	23688141	23688151	5.5	.	.	intergenic	.	rs791323503	2
AAAAT.ATTTT	10	24103504	24103521	3.6	.	.	intergenic	-0.096	rs790065989	2
AC.GT	10	24481188	24481198	5.5	ENSSSCT00000011919	TP53BP2	intron	-0.466	rs788311382	3
AC.GT	10	24743814	24743825	6	.	.	intergenic	0.286	rs791515574	2
AAAT.ATTT	10	24954372	24954388	4.25	.	.	intergenic	.	rs791153079	2
AAAT.ATTT	10	25205779	25205811	8.25	.	.	intergenic	-0.007	rs787858034	2
AAGG.CCTT	10	25413041	25413074	8.5	.	.	intergenic	0.399	rs790980759	2
AAAG.CTTT	10	25473766	25473780	3.75	.	.	intergenic	0.006	556464 rs7898	2
AAATT.AATTT	10	25715672	25715708	7.4	ENSSSCT00000011931	NEK7	intron	.	450971 rs7892	2
AC.GT	10	25813640	25813652	6.5	.	.	intergenic	.	rs786834065	2
AT.AT	10	25878822	25878834	6.5	.	.	intergenic	0.022	955283 rs7883	3
AAC.GTT	10	25888851	25888868	6	.	.	intergenic	.	187122 rs7876	2
AC.GT	10	25969868	25969884	8.5	.	.	intergenic	0.009	rs793233399	2
AAAAC.GTTTT	10	26423656	26423679	4.8	.	.	intergenic	-0.078	rs791824894	2
ACCT.AGGT	10	26454479	26454493	3.75	.	.	intergenic	-0.127	.	2
AGG.CCT	10	26572109	26572120	4	.	.	intergenic	-0.208	rs790749820	2
AC.GT	10	26684828	26684849	11	.	.	intergenic	-0.137	597436 rs7905	2
AG.CT	10	26729457	26729470	7	.	.	intergenic	0.05	948074 rs7906	2
AAAAG.CTTTT	10	26732812	26732829	3.6	.	.	intergenic	-0.163	rs792222761	2
AAT.ATT	10	26757359	26757372	4.66667	.	.	intergenic	-0.002	189779 rs7884	2
AT.AT	10	26827090	26827100	5.5	.	.	intergenic	0.227	rs787308746	2
AC.GT	10	26957983	26957994	6	.	.	intergenic	.	rs790813389	2
AAAAAG.CTTTTT	10	27078634	27078651	3	.	.	intergenic	.	rs786428495	2
AAAC.GTTT	10	27087987	27088001	3.75	.	.	intergenic	.	rs787513680	2
AAAAC.GTTTT	10	27104376	27104393	3.6	.	.	intergenic	0.175	.	2
AAAT.ATTT	10	27320475	27320497	5.75	ENSSSCT00000026077	NR5A2	intron	-0.174	rs788467062	2
AAAC.GTTT	10	27546613	27546628	4	.	.	intergenic	0.318	rs792056328	2
AAAAAC.GTTTTT	10	27732636	27732658	3.83333	.	.	intergenic	0.006	rs789571944	2
AAAC.GTTT	10	27747018	27747040	5.75	ENSSSCT00000030669	CAMSAP2	3'utr	1.108	rs786717134	2
AAAG.CTTT	10	27748502	27748520	4.75	ENSSSCT00000030669	CAMSAP2	intron	3.734	104251 rs7899	2
AAAC.GTTT	10	27750930	27750944	3.75	ENSSSCT00000030669	CAMSAP2	intron	-0.141	rs792312322	2
AC.GT	10	28451562	28451578	8.5	.	.	intergenic	-0.28	.	2
AAT.ATT	10	28606450	28606464	5	ENSSSCT00000026899	IPO9	intron	0.116	rs791878670	2
AAAC.GTTT	10	28852543	28852561	4.75	ENSSSCT00000011955	UBE2T	intron	1.786	rs786396032	3
AC.GT	10	29093010	29093038	14.5	ENSSSCT00000011958	SYT2	intron	-0.495	rs791399731	2
GGGGC.GCCCC	10	29170931	29170951	3.5	.	.	intergenic	-0.284	974407 rs7922	2
AAAAC.GTTTT	10	29196193	29196210	3.6	.	.	intergenic	.	rs788005751	2
AAAAG.CTTTT	10	29359330	29359348	3.8	ENSSSCT00000011960	.	intron	.	rs793228739	2
AAC.GTT	10	29574906	29574920	5	ENSSSCT00000030381	CDC14B	intron	.	052026 rs7874	2
AAG.CTT	10	29816101	29816112	4	.	.	intergenic	.	.	2
AAAC.GTTT	10	29944023	29944041	4.75	.	.	intergenic	.	173428 rs7879	2
AT.AT	10	30009603	30009615	6.5	ENSSSCT00000011969	HSD17B3	intron	0.166	rs791408508	3
AATGAT.ATCATT	10	30130645	30130674	5	ENSSSCT00000011969	HSD17B3	intron	0.111	.	2
AC.GT	10	30154518	30154542	12.5	ENSSSCT00000011969	HSD17B3	intron	-0.064	270812 rs7865	4
AC.GT	10	30235194	30235205	6	.	.	intergenic	0.717	rs792392932	2
AAC.GTT	10	30346780	30346793	4.66667	.	.	intergenic	0.138	rs790472787	2
AAC.GTT	10	30392044	30392055	4	ENSSSCT00000011974	ERCC6L2	intron	.	rs792141902	2
AG.CT	10	30596947	30596962	8	.	.	intergenic	-0.102	rs792138543	2
AAAAC.GTTTT	10	30750516	30750530	3	.	.	intergenic	0.043	.	2
AT.AT	10	30941517	30941535	9.5	.	.	intergenic	.	rs790881526	3
AT.AT	10	31046603	31046612	5	.	.	intergenic	.	rs790445697	2

AAC.GTT	10	31050640	31050653	4.66667	.	.	intergenic	.	rs788473766	2
AT.AT	10	31180813	31180823	5.5	.	.	intergenic	.	rs793333513	2
AG.CT	10	31224357	31224375	9.5	.	.	intergenic	.	rs786876858	2
AT.AT	10	31251517	31251532	8	ENSSSCT00000031684	.	promoter	0.044	180529 rs7911	3
AAT.ATT	10	31480790	31480811	7.33333	.	.	intergenic	-0.181	rs793455068	2
AG.CT	10	31692892	31692903	6	.	.	intergenic	0.035	rs791478951	4
AC.GT	10	31802041	31802055	7.5	ENSSSCT00000011979	FBP1	intron	-0.022	rs788895371	5
AAAT.ATTT	10	31851266	31851284	4.75	ENSSSCT00000011982	FBP2	intron	.	999286 rs7898	2
AC.GT	10	31976701	31976720	10	ENSSSCT00000026142	DAPK1	intron	.	rs790915979	2
AAAC.GTTT	10	31996849	31996867	4.75	ENSSSCT00000026142	DAPK1	intron	.	rs792578163	2
AAAC.GTTT	10	31996849	31996867	4.75	ENSSSCT00000011985	.	intron	.	rs792578163	2
AAC.GTT	10	32156204	32156224	7	.	.	intergenic	0.041	rs791813333	2
AC.GT	10	32163926	32163949	12	.	.	intergenic	.	.	4
AT.AT	10	32335382	32335391	5	.	.	intergenic	0.828	.	2
AT.AT	10	32503085	32503094	5	.	.	intergenic	-0.1	rs793617288	2
AAAAT.ATTTT	10	32604334	32604355	4.4	.	.	intergenic	0.003	rs789304099	2
AAC.GTT	10	32765995	32766015	7	.	.	intergenic	-0.312	rs790527237	3
AG.CT	10	32830312	32830323	6	.	.	intergenic	0.17	.	2
ACAT.ATGT	10	32923147	32923162	4	.	.	intergenic	-0.461	532140 rs7898	3
AC.GT	10	32927870	32927881	6	.	.	intergenic	-0.12	rs790392673	2
AC.GT	10	32942866	32942895	15	.	.	intergenic	0.145	rs792398287	2
AAAC.GTTT	10	32958465	32958479	3.75	.	.	intergenic	0.589	rs786812595	2
AAAG.CTTT	10	32966547	32966565	4.75	.	.	intergenic	.	rs793845191	2
AC.GT	10	33011054	33011068	7.5	.	.	intergenic	0.724	rs793569321	3
AAAC.GTTT	10	33195079	33195093	3.75	ENSSSCT00000011991	GOLM1	intron	.	381843 rs7867	2
AAAC.GTTT	10	33199519	33199533	3.75	ENSSSCT00000011991	GOLM1	intron	.	rs787216146	2
AAAC.GTTT	10	33428441	33428457	4.25	.	.	intergenic	.	rs787884701	2
AC.GT	10	33442343	33442365	11.5	.	.	intergenic	.	.	3
AT.AT	10	33471020	33471033	7	.	.	intergenic	.	rs788560314	2
AAAAT.ATTTT	10	33621572	33621588	3.4	ENSSSCT00000011993	.	intron	0.13	395555 rs7913	2
AAAAG.CTTTT	10	33736479	33736502	4.8	.	.	intergenic	-0.227	rs792376736	2
AAT.ATT	10	33781409	33781424	5.33333	.	.	intergenic	.	rs786972405	2
AAAC.GTTT	10	34055560	34055586	6.75	.	.	intergenic	-0.089	rs788715586	2
ACC.GGT	10	34066589	34066610	7.33333	.	.	intergenic	-0.281	rs789839184	2
AAAG.CTTT	10	34097263	34097277	3.75	.	.	intergenic	-0.17	rs788584249	2
AAAT.ATTT	10	34220671	34220692	5.5	.	.	intergenic	-0.064	rs786530455	2
AG.CT	10	34223293	34223303	5.5	.	.	intergenic	0.1	.	2
AAAT.ATTT	10	34264504	34264530	6.75	ENSSSCT00000011996	NTRK2	intron	-0.077	096454 rs7866	2
AAAT.ATTT	10	34264504	34264530	6.75	ENSSSCT00000011995	NTRK2	intron	-0.077	096454 rs7866	2
AAAAC.GTTTT	10	34345284	34345307	4.8	.	.	intergenic	0.746	.	2
AAAAT.ATTTT	10	34417999	34418023	5	.	.	intergenic	.	rs786382970	2
AC.GT	10	34532000	34532012	6.5	.	.	intergenic	-0.029	319025 rs6905	3
AATG.CATT	10	34773258	34773280	5.75	.	.	intergenic	0.211	rs793576689	2
AAG.CTT	10	34839027	34839042	5.33333	.	.	intergenic	0.247	rs788329598	2
AAAAG.CTTTT	10	34909221	34909243	4.6	.	.	intergenic	.	143411 rs7868	2
AT.AT	10	35050049	35050063	7.5	.	.	intergenic	-0.035	156289 rs7899	2
AAAAT.ATTTT	10	35371334	35371353	4	ENSSSCT00000027988	UBQLN1	intron	-0.043	rs786760116	2
AAAAAC.GTTTTT	10	35445376	35445410	5.83333	.	.	intergenic	0.135	rs787078489	2
AG.CT	10	35468402	35468427	13	.	.	intergenic	0.082	045595 rs7867	4
AAAAC.GTTTT	10	35469040	35469067	5.6	.	.	intergenic	0.153	rs791130341	2
AC.GT	10	35490880	35490900	10.5	.	.	intergenic	.	163309 rs7929	2
AT.AT	10	35546911	35546921	5.5	.	.	intergenic	.	rs793622590	2
AC.GT	10	35557876	35557888	6.5	.	.	intergenic	0.157	.	2
AAAC.GTTT	10	35732493	35732511	4.75	ENSSSCT00000011999	FRMD3	intron	-0.189	rs790744873	2
AT.AT	10	35775502	35775521	10	ENSSSCT00000011999	FRMD3	intron	-0.23	.	2
AC.GT	10	35805499	35805510	6	.	.	intergenic	.	rs786465848	4
AC.GT	10	35808865	35808875	5.5	.	.	intergenic	.	297622 rs7918	2
AC.GT	10	36040314	36040340	13.5	.	.	intergenic	0.352	969498 rs7900	2
AAAC.GTTT	10	36068403	36068417	3.75	.	.	intergenic	0.017	rs694323775	3
AT.AT	10	36113255	36113273	9.5	ENSSSCT00000012001	RASEF	intron	0.038	977930 rs7921	2
AAC.GTT	10	36498424	36498439	5.33333	.	.	intergenic	0.232	rs793701877	2
AAAAAC.GTTTTT	10	37107687	37107716	5	ENSSSCT00000012026	UBAP2	intron	-0.231	.	2
AAAAAC.GTTTTT	10	37107687	37107716	5	ENSSSCT00000025202	SNORD121	promoter	-0.231	.	2
AC.GT	10	37208291	37208304	7	.	.	intergenic	.	rs786784548	2
AC.GT	10	37356048	37356063	8	.	.	intergenic	0.336	.	2
AATC.GATT	10	37410805	37410828	6	ENSSSCT00000012035	.	intron	.	rs787755876	2
AAAT.ATTT	10	37477310	37477335	6.5	ENSSSCT00000012036	CHMP5	intron	0.165	rs792920474	2
AT.AT	10	37480574	37480596	11.5	ENSSSCT00000012036	CHMP5	intron	-0.333	rs789806657	2
AAAC.GTTT	10	37655646	37655665	5	ENSSSCT00000012041	SMU1	intron	.	rs787365275	2
AAAC.GTTT	10	37666431	37666449	4.75	ENSSSCT00000012041	SMU1	intron	0.316	787172 rs7875	2
AAAC.GTTT	10	37788592	37788616	6.25	ENSSSCT00000012043	APTX	intron	0.186	rs791910246	2
AG.CT	10	38113167	38113179	6.5	ENSSSCT00000012045	NDUFB6	intron	0.008	rs788084693	2
AC.GT	10	38246547	38246557	5.5	.	.	intergenic	-0.045	rs690988480	3
AAAG.CTTT	10	38248250	38248277	7	.	.	intergenic	.	340732 rs7920	2
AT.AT	10	38362200	38362211	6	.	.	intergenic	0.183	773173 rs7925	2
AT.AT	10	38800439	38800449	5.5	.	.	intergenic	0.103	rs787349895	2
AGGG.CCCT	10	38870894	38870912	4.75	.	.	intergenic	0.138	rs787116052	2
AC.GT	10	39047295	39047318	12	.	.	intergenic	0.151	rs792957186	4
AT.AT	10	39407315	39407329	7.5	.	.	intergenic	0.12	rs787020106	2
AC.GT	10	39409019	39409038	10	.	.	intergenic	0.203	.	4
AC.GT	10	39431874	39431889	8	.	.	intergenic	.	rs786463274	4
AC.GT	10	39488831	39488860	15	.	.	intergenic	-0.245	.	2
AT.AT	10	39513791	39513820	15	.	.	intergenic	0.056	350459 rs7864	2
AG.CT	10	39682266	39682280	7.5	.	.	intergenic	-0.188	rs793433799	2

AAG.CTT	10	39725120	39725135	5.33333	.	.	intergenic	.	rs788722477	2
AC.GT	10	39881437	39881447	5.5	.	.	intergenic	.	rs793397347	2
AT.AT	10	40117962	40117991	15	.	.	intergenic	.	.	2
AAAC.GTTT	10	40255975	40255997	5.75	.	.	intergenic	.	310030 rs7899	2
AAC.GTT	10	40282082	40282099	6	.	.	intergenic	.	rs788220660	2
AAAT.ATTT	10	40379626	40379640	3.75	.	.	intergenic	0.157	.	2
AAAT.ATTT	10	40388506	40388527	5.5	.	.	intergenic	.	rs792137424	2
AAAC.GTTT	10	40394036	40394073	9.5	.	.	intergenic	.	rs791673532	2
AG.CT	10	40414050	40414070	10.5	.	.	intergenic	.	rs788254004	2
AGAT.ATCT	10	40587363	40587404	10.5	.	.	intergenic	0.036	rs788132469	4
AC.GT	10	40628276	40628291	8	.	.	intergenic	0.044	758910 rs7919	3
AT.AT	10	40806803	40806830	14	.	.	intergenic	-0.089	rs788584603	4
AAAG.CTTT	10	40840925	40840939	3.75	.	.	intergenic	.	.	2
AAAT.ATTT	10	40845190	40845207	4.5	.	.	intergenic	.	.	2
AT.AT	10	40878006	40878017	6	.	.	intergenic	-0.131	282637 rs7888	3
AAAT.ATTT	10	41051394	41051423	7.5	.	.	intergenic	0.116	rs788506665	2
AC.GT	10	41103005	41103023	9.5	.	.	intergenic	.	rs790867697	7
AT.AT	10	41197409	41197420	6	.	.	intergenic	-0.042	rs792852727	3
AAT.ATT	10	41390706	41390729	8	.	.	intergenic	.	.	2
AAATT.AATTT	10	41433941	41433955	3	.	.	intergenic	.	071375 rs7894	2
AT.AT	10	41490324	41490333	5	.	.	intergenic	0.174	rs789519210	2
AC.GT	10	41703941	41703954	7	.	.	intergenic	.	rs789643408	5
AT.AT	10	41785739	41785748	5	.	.	intergenic	0.445	702068 rs7917	2
AT.AT	10	41814503	41814517	7.5	.	.	intergenic	0.18	rs792953018	2
AG.CT	10	41971237	41971251	7.5	.	.	intergenic	.	rs791942931	4
AAAAC.GTTTT	10	41984045	41984065	4.2	.	.	intergenic	0.168	rs790168878	2
AAAAG.CTTTT	10	42094548	42094573	5.2	.	.	intergenic	0.113	rs786694622	2
AT.AT	10	42238136	42238147	6	.	.	intergenic	.	rs787338091	2
AAC.GTT	10	42307921	42307936	5.33333	.	.	intergenic	-0.161	rs789324582	2
AAAC.GTTT	10	42572964	42572978	3.75	.	.	intergenic	-0.076	rs792021027	2
AAAT.ATTT	10	42579574	42579594	5.25	.	.	intergenic	-0.05	566930 rs7893	2
AT.AT	10	42710756	42710778	11.5	.	.	intergenic	.	704899 rs7926	2
AAC.GTT	10	42715172	42715188	5.66667	.	.	intergenic	.	rs708403384	2
AG.CT	10	42803133	42803163	15.5	.	.	intergenic	0.069	394620 rs7891	2
AT.AT	10	43057593	43057607	7.5	.	.	intergenic	0.024	.	4
ATCC.GGAT	10	43092041	43092055	3.75	.	.	intergenic	0.142	.	2
AATT.AATT	10	43410121	43410135	3.75	.	.	intergenic	-0.169	rs792797493	2
AT.AT	10	43527201	43527218	9	.	.	intergenic	.	rs789051924	2
AAAG.CTTT	10	43684762	43684777	4	.	.	intergenic	-0.085	370954 rs7907	4
AAAC.GTTT	10	43760330	43760343	3.5	.	.	intergenic	0.242	rs792541828	2
AC.GT	10	43805459	43805471	6.5	.	.	intergenic	.	378933 rs7921	2
AAAAAT.ATTTTT	10	43806696	43806721	4.33333	.	.	intergenic	.	rs790717523	2
AT.AT	10	43913986	43913996	5.5	.	.	intergenic	.	rs792944589	2
AAAC.GTTT	10	43925829	43925847	4.75	.	.	intergenic	.	160805 rs7916	2
AAAG.CTTT	10	44024024	44024044	5.25	.	.	intergenic	0.097	rs793786850	2
AAC.GTT	10	44053925	44053937	4.33333	.	.	intergenic	0.106	503606 rs7926	2
AAAC.GTTT	10	44657796	44657814	4.75	.	.	intergenic	-0.271	rs791141472	2
AGG.CCT	10	44693898	44693916	6.33333	.	.	intergenic	-0.064	.	2
AG.CT	10	44755711	44755722	6	.	.	intergenic	0.009	.	3
AT.AT	10	44826432	44826442	5.5	.	.	intergenic	-0.035	733091 rs7865	2
AAAAAG.CTTTTT	10	45029561	45029588	4.66667	.	.	intergenic	.	rs792891109	2
AAAC.GTTT	10	45118050	45118066	4.25	ENSSSCT00000034425	MAP3K8	intron	0.492	rs793489797	2
AAAC.GTTT	10	45118050	45118066	4.25	ENSSSCT00000033487	MAP3K8	intron	0.492	rs793489797	2
AAAC.GTTT	10	45118050	45118066	4.25	ENSSSCT00000033089	MAP3K8	intron	0.492	rs793489797	2
AC.GT	10	45243640	45243652	6.5	.	.	intergenic	.	268936 rs7911	2
ACTC.GAGT	10	45385967	45385981	3.75	ENSSSCT00000024823	KIAA1462	promoter	-0.17	rs790486082	2
ACTC.GAGT	10	45385967	45385981	3.75	ENSSSCT00000012062	KIAA1462	promoter	-0.17	rs790486082	2
AC.GT	10	45394370	45394384	7.5	ENSSSCT00000024823	KIAA1462	intron	-0.066	.	2
AC.GT	10	45394370	45394384	7.5	ENSSSCT00000012062	KIAA1462	intron	-0.066	.	2
AAC.GTT	10	45424173	45424186	4.66667	ENSSSCT00000024823	KIAA1462	intron	-0.157	rs790099975	2
AAC.GTT	10	45424173	45424186	4.66667	ENSSSCT00000012062	KIAA1462	intron	-0.157	rs790099975	2
AGGG.CCCT	10	45571974	45571994	5.25	.	.	intergenic	.	rs789875897	2
AAAAG.CTTTT	10	45716852	45716868	3.4	.	.	intergenic	-0.082	rs788109735	2
AAC.GTT	10	45917218	45917244	9	ENSSSCT00000012064	SVIL	intron	-0.084	rs791084106	2
AAC.GTT	10	45917218	45917244	9	ENSSSCT00000012066	SVIL	intron	-0.084	rs791084106	2
AC.GT	10	45945136	45945146	5.5	ENSSSCT00000012064	SVIL	intron	-0.653	rs789625042	3
AC.GT	10	45945136	45945146	5.5	ENSSSCT00000012066	SVIL	intron	-0.653	rs789625042	3
AC.GT	10	45984606	45984634	14.5	ENSSSCT00000012064	SVIL	intron	-0.273	rs789703627	2
AC.GT	10	45984606	45984634	14.5	ENSSSCT00000012066	SVIL	intron	-0.273	rs789703627	2
AC.GT	10	46130066	46130084	9.5	.	.	intergenic	.	.	2
AAAT.ATTT	10	46170265	46170287	5.75	.	.	intergenic	0.308	rs790796770	2
AT.AT	10	46301895	46301905	5.5	.	.	intergenic	.	rs789540306	2
AT.AT	10	46482587	46482601	7.5	.	.	intergenic	0.239	.	4
AAC.GTT	10	46576031	46576047	5.66667	ENSSSCT00000035562	ZEB1	intron	0.267	rs792287309	2
AAAC.GTTT	10	46616398	46616410	3.25	ENSSSCT00000035562	ZEB1	intron	0.116	rs793107734	2
AC.GT	10	46947134	46947153	10	.	.	intergenic	.	.	3
AT.AT	10	46957538	46957548	5.5	.	.	intergenic	.	380583 rs7908	2
AT.AT	10	46973893	46973905	6.5	.	.	intergenic	0.141	.	2
AAAG.CTTT	10	47281806	47281824	4.75	ENSSSCT00000012069	KIF5B	intron	0.291	455021 rs7863	2
ACAG.CTGT	10	47282471	47282497	6.75	ENSSSCT00000012069	KIF5B	intron	.	358657 rs7865	2
AAAAT.ATTTT	10	47953291	47953306	3.2	ENSSSCT00000012072	CUBN	intron	0.05	rs787458564	2
AG.CT	10	48014021	48014039	9.5	.	.	intergenic	-0.073	376695 rs7902	2
AC.GT	10	48252393	48252407	7.5	.	.	intergenic	0.215	060711 rs7873	4
AAC.GTT	10	48386158	48386171	4.66667	.	.	intergenic	0.77	rs788364054	2

AAAAG.CTTTT	10	48607761	48607786	4.33333	ENSSSCT00000012078	.	intron	0.015	rs694656015	2
AGAT.ATCT	10	48625461	48625483	5.75	.	.	intergenic	0.16	523197 rs7929	2
AG.CT	10	48730794	48730803	5	.	.	intergenic	-0.129	rs793568983	2
AAC.GTT	10	48758514	48758534	7	ENSSSCT00000012080	.	intron	-0.682	983319 rs7922	2
AC.GT	10	48893453	48893463	5.5	ENSSSCT00000012082	H242-21O2	intron	-0.27	.	3
AAAC.GTTT	10	48901524	48901546	5.75	ENSSSCT00000012082	H242-21O2	intron	-0.831	799126 rs7903	2
AACC.GGTT	10	48970206	48970219	3.5	.	.	intergenic	-0.012	591073 rs7876	2
AAAC.GTTT	10	49086444	49086462	4.75	.	.	intergenic	.	rs790377612	2
AC.GT	10	49231913	49231926	7	.	.	intergenic	-0.092	rs691181848	2
AAAG.CTTT	10	49412392	49412407	4	.	.	intergenic	-0.111	rs786465961	2
AAAT.ATTT	10	49461371	49461387	4.25	.	.	intergenic	.	rs789197907	2
AT.AT	10	49654268	49654279	6	ENSSSCT00000012085	.	intron	.	114463 rs7862	3
AC.GT	10	49665013	49665032	10	ENSSSCT00000012085	.	intron	.	rs791153623	2
AAT.ATT	10	49734174	49734187	4.66667	.	.	intergenic	.	rs789756776	2
AAAG.CTTT	10	49925942	49925954	3.25	.	.	intergenic	0.103	372807 rs7931	2
AAT.ATT	10	50013983	50014000	6	.	.	intergenic	-0.192	977612 rs7869	2
AT.AT	10	50073533	50073544	6	.	.	intergenic	2.941	rs788079614	2
AAAAC.GTTTT	10	50169484	50169502	3.8	ENSSSCT00000026176	.	intron	0.331	rs792572016	2
AAAG.CTTT	10	50174660	50174674	3.75	ENSSSCT00000026176	.	intron	-0.165	rs787673008	2
AC.GT	10	50218384	50218396	6.5	.	.	intergenic	-0.033	529005 rs7862	3
AAAC.GTTT	10	50395728	50395749	5.5	.	.	intergenic	-0.089	163229 rs7862	2
AGATAT.ATATCT	10	50471954	50471986	5.5	.	.	intergenic	-0.157	rs788052573	2
ACATG.CATGT	10	50603290	50603314	5	.	.	intergenic	.	rs791614895	2
AAAT.ATTT	10	50678361	50678378	4.5	ENSSSCT00000012089	FAM188A	intron	0.225	rs790602660	2
AT.AT	10	50738139	50738148	5	ENSSSCT00000012089	FAM188A	intron	.	rs790275020	2
AAAT.ATTT	10	50802875	50802894	5	.	.	intergenic	-0.015	319793 rs7921	2
AC.GT	10	50835571	50835590	10	ENSSSCT00000012090	ITGA8	intron	-0.03	.	3
ATCC.GGAT	10	50986655	50986677	5.75	ENSSSCT00000012090	ITGA8	intron	-0.044	rs791287109	2
AT.AT	10	51182090	51182100	5.5	ENSSSCT00000012091	FAM171A1	intron	0.433	rs792364023	3
AG.CT	10	51212597	51212611	7.5	ENSSSCT00000012091	FAM171A1	intron	0.016	533993 rs7886	2
AGGG.CCCT	10	51455957	51455975	4.75	ENSSSCT00000024077	RPP38	intron	-0.121	599592 rs7899	2
AGG.CCT	10	51595725	51595741	5.66667	ENSSSCT00000012093	DCLRE1C	cds	0.731	rs787346006	2
AGG.CCT	10	51595725	51595741	5.66667	ENSSSCT00000012093	DCLRE1C	intron	0.731	rs787346006	2
AAAT.ATTT	10	51809083	51809098	4	.	.	intergenic	.	rs792459203	2
AC.GT	10	51855840	51855850	5.5	.	.	intergenic	0.079	.	2
ACAG.CTGT	10	52068045	52068071	6.75	.	.	intergenic	0.143	500737 rs7930	2
AG.CT	10	52391721	52391734	7	.	.	intergenic	0.156	204346 rs7883	2
AC.GT	10	52396442	52396463	11	.	.	intergenic	-0.141	rs786860558	2
AT.AT	10	52397734	52397745	6	.	.	intergenic	0.259	rs789271449	2
AG.CT	10	52467943	52467960	9	.	.	intergenic	0.211	789224 rs7893	2
AG.CT	10	52491127	52491144	9	.	.	intergenic	0.894	rs787756435	2
AT.AT	10	52547146	52547157	6	.	.	intergenic	0.356	774432 rs7907	2
AG.CT	10	52550689	52550707	9.5	.	.	intergenic	-0.067	248980 rs7885	2
AC.GT	10	52797217	52797227	5.5	ENSSSCT00000012100	.	intron	.	.	3
AC.GT	10	53122060	53122074	7.5	ENSSSCT00000012114	MPP7	intron	-0.032	rs789666518	2
AAAT.ATTT	10	53357554	53357574	5.25	ENSSSCT00000026458	.	intron	.	387048 rs7887	2
AAAAG.CTTTT	10	53696426	53696452	5.4	.	.	intergenic	0.181	902542 rs7869	2
AT.AT	10	53808752	53808761	5	.	.	intergenic	.	.	4
AAAC.GTTT	10	54023464	54023482	4.75	ENSSSCT00000012110	YME1L1	promoter	0.181	075231 rs7883	2
AAC.GTT	10	54120513	54120532	6.66667	ENSSSCT00000012112	.	intron	0.134	345183 rs7865	2
AAAT.ATTT	10	54560177	54560195	4.75	.	.	intergenic	0.352	714390 rs7902	2
ATC.GAT	10	54565508	54565527	6.66667	.	.	intergenic	0.067	rs790550532	2
AAAT.ATTT	10	54574195	54574212	4.5	.	.	intergenic	0.138	rs790365345	2
AAAT.ATTT	10	54599132	54599146	3.75	.	.	intergenic	0.2	264326 rs7938	2
AAAG.CTTT	10	54725088	54725106	4.75	.	.	intergenic	.	rs793236715	2
AT.AT	10	54812566	54812577	6	ENSSSCT00000012103	MYO3A	intron	0.075	.	2
AAAAAC.GTTTTT	10	54818557	54818575	3.16667	ENSSSCT00000012103	MYO3A	intron	1.311	464480 rs7897	2
AAAT.ATTT	10	54822690	54822712	5.75	ENSSSCT00000012103	MYO3A	intron	0.323	rs788770853	2
AC.GT	10	54926351	54926360	5	.	.	intergenic	0.208	.	3
AAAT.ATTT	10	54942573	54942584	3	.	.	intergenic	0.265	rs786557547	2
AAAAAC.GTTTTT	10	54971915	54971941	4.5	.	.	intergenic	-0.172	rs788357612	2
AG.CT	10	55015962	55015972	5.5	.	.	intergenic	0.146	.	4
AAC.GTT	10	55030349	55030365	5.66667	.	.	intergenic	0.058	307906 rs7891	2
AAAC.GTTT	10	55104315	55104330	4	.	.	intergenic	0.004	rs789420311	2
AT.AT	10	55497997	55498017	10.5	.	.	intergenic	.	183017 rs7877	2
AAG.CTT	10	55566380	55566394	5	.	.	intergenic	.	rs790324247	2
AAAT.ATTT	10	55778706	55778720	3.75	.	.	intergenic	0.022	rs789331382	2
AT.AT	10	55872840	55872850	5.5	ENSSSCT00000012116	.	intron	0.28	rs786577071	3
ACAG.CTGT	10	56063685	56063707	5.75	ENSSSCT00000012118	ARHGAP21	intron	0.125	467272 rs7917	2
ACAG.CTGT	10	56063685	56063707	5.75	ENSSSCT00000029464	ARHGAP21	intron	0.125	467272 rs7917	2
ACAG.CTGT	10	56063685	56063707	5.75	ENSSSCT00000028075	ARHGAP21	intron	0.125	467272 rs7917	2
AC.GT	10	56257227	56257247	10.5	ENSSSCT00000012119	KIAA1217	intron	.	385117 rs7906	2
AAAT.ATTT	10	56422321	56422337	4.25	.	.	intergenic	-0.129	rs787226325	2
AATG.CATT	10	56518488	56518507	5	.	.	intergenic	-0.094	rs789791161	2
AAAT.ATTT	10	57350240	57350254	3.75	.	.	intergenic	.	rs790037726	2
AAC.GTT	10	57578893	57578906	4.66667	.	.	intergenic	-0.085	rs790361585	2
AAC.GTT	10	57650685	57650702	6	ENSSSCT00000012124	ARMC3	intron	-0.069	rs790802988	2
AAAC.GTTT	10	57687456	57687478	5.75	ENSSSCT00000012124	ARMC3	intron	0.223	rs786264790	3
AAT.ATT	10	57691147	57691161	5	ENSSSCT00000012124	ARMC3	intron	.	rs791591205	2
AG.CT	10	57789047	57789064	9	.	.	intergenic	.	rs696633143	4
AAC.GTT	10	58155533	58155549	5.66667	ENSSSCT00000012127	SPAG6	intron	.	rs788912012	2
AG.CT	10	58199022	58199035	7	ENSSSCT00000012127	SPAG6	intron	0.119	rs787436733	3
AAC.GTT	10	58219431	58219450	6.66667	ENSSSCT00000012127	SPAG6	intron	0.1	095354 rs7923	2
AAAAG.CTTTT	10	58325441	58325461	4.2	.	.	intergenic	0.059	790618 rs7923	2

AAAAT.ATTTT	10	58406797	58406819	4.6	.	.	intergenic	.	rs789959223	2
AAAT.ATTT	10	58508637	58508666	7.5	.	.	intergenic	-0.072	.	2
AAAC.GTTT	10	58526593	58526612	5	.	.	intergenic	0.357	rs788295614	2
AAAC.GTTT	10	58745639	58745656	4.5	ENSSSCT00000012132	MLLT10	intron	0.409	rs787896746	2
AAAC.GTTT	10	58853788	58853803	4	ENSSSCT00000012132	MLLT10	intron	0.103	rs793812793	2
AG.CT	10	59146851	59146871	10.5	.	.	intergenic	0.216	.	2
AAAAG.CTTTT	10	59451101	59451115	3	.	.	intergenic	-0.075	rs787115558	2
AAAAC.GTTTT	10	59529835	59529850	3.2	.	.	intergenic	0.3	rs793106034	2
AG.CT	10	59756863	59756884	11	.	.	intergenic	-0.004	rs790861400	3
AAAG.CTTT	10	59791967	59791988	5.5	.	.	intergenic	0.243	rs788438908	2
AC.GT	10	59807604	59807613	5	.	.	intergenic	-0.052	.	2
AAC.GTT	10	59899710	59899725	5.33333	.	.	intergenic	0.367	rs793225345	2
AAAAT.ATTTT	10	60015353	60015368	3.2	.	.	intergenic	.	.	2
AAG.CTT	10	60463494	60463510	5.66667	.	.	intergenic	-0.079	285394 rs7920	2
AAG.CTT	10	60540849	60540861	4.33333	.	.	intergenic	.	rs786290967	2
AAAC.GTTT	10	60665727	60665740	3.5	.	.	intergenic	.	rs792175717	2
AG.CT	10	60842415	60842426	6	.	.	intergenic	-0.098	940956 rs7920	2
AAC.GTT	10	61035875	61035891	5.66667	.	.	intergenic	.	430326 rs7867	2
AAC.GTT	10	61047727	61047750	8	.	.	intergenic	0.208	rs791790437	2
AAC.GTT	10	61151849	61151860	4	.	.	intergenic	0.008	189753 rs7868	2
AAAC.GTTT	10	61388243	61388266	6	.	.	intergenic	.	rs789507038	2
AT.AT	10	61417063	61417072	5	.	.	intergenic	0.299	.	3
AAGG.CCTT	10	61528058	61528082	6.25	.	.	intergenic	0.165	rs792621719	3
AGGGG.CCCCT	10	61544575	61544594	4	.	.	intergenic	-0.063	326061 rs7881	2
AT.AT	10	61759165	61759180	8	ENSSSCT00000033235	NRP1	intron	0.469	rs787042928	2
AT.AT	10	61759165	61759180	8	ENSSSCT00000035993	NRP1	intron	0.469	rs787042928	2
AT.AT	10	61759165	61759180	8	ENSSSCT00000012149	NRP1	intron	0.469	rs787042928	2
AT.AT	10	61759165	61759180	8	ENSSSCT00000034324	NRP1	intron	0.469	rs787042928	2
AT.AT	10	61759165	61759180	8	ENSSSCT00000034108	NRP1	intron	0.469	rs787042928	2
AT.AT	10	61759165	61759180	8	ENSSSCT00000035186	NRP1	intron	0.469	rs787042928	2
AAAC.GTTT	10	62129018	62129044	6.75	.	.	intergenic	1.038	408966 rs7918	2
AAAAAC.GTTTTT	10	62169044	62169073	5	.	.	intergenic	0.159	.	2
AAAAAC.GTTTTT	10	62191755	62191781	4.5	.	.	intergenic	0.058	rs697076905	2
AAAC.GTTT	10	62840569	62840592	6	ENSSSCT00000012150	PARD3	intron	-0.062	.	2
AG.CT	10	62869065	62869075	5.5	ENSSSCT00000012150	PARD3	intron	-0.316	.	3
AC.GT	10	62973016	62973032	8.5	ENSSSCT00000012150	PARD3	intron	0.552	.	2
AAAC.GTTT	10	63094677	63094701	6.25	.	.	intergenic	-0.045	982864 rs7937	2
AC.GT	10	63303295	63303305	5.5	ENSSSCT00000012151	CUL2	3'utr	0.192	.	2
AGG.CCT	10	63349981	63349996	5.33333	.	.	intergenic	.	rs787349103	2
AAAC.GTTT	10	63628210	63628231	5.5	ENSSSCT00000012155	CCNY	intron	0.042	973215 rs7869	2
AC.GT	10	63753703	63753735	16.5	.	.	intergenic	0.201	rs786733782	2
AC.GT	10	64327260	64327274	7.5	.	.	intergenic	-0.09	.	3
AAAC.GTTT	10	64461332	64461350	4.75	.	.	intergenic	-0.104	rs787801440	2
AAAT.ATTT	10	64510426	64510450	6.25	.	.	intergenic	0.041	rs793375663	2
AG.CT	10	64728963	64728973	5.5	.	.	intergenic	.	rs786250222	3
AAAAAG.CTTTTT	10	64754619	64754637	3.16667	.	.	intergenic	0.341	rs792599884	2
AT.AT	10	64755201	64755212	6	.	.	intergenic	0.27	rs789314756	4
AGG.CCT	10	64993474	64993489	5.33333	.	.	intergenic	0.077	325583 rs7938	2
AC.GT	10	65042410	65042423	7	.	.	intergenic	-0.185	736390 rs7892	3
AC.GT	10	65149791	65149806	8	.	.	intergenic	.	.	3
AC.GT	10	65264057	65264070	7	.	.	intergenic	.	rs787257547	2
AC.GT	10	65294551	65294572	11	.	.	intergenic	-0.582	.	2
AC.GT	10	65300508	65300519	6	.	.	intergenic	.	rs788604638	2
AC.GT	10	65359386	65359397	6	.	.	intergenic	.	.	2
AAC.GTT	10	65403253	65403271	6.33333	.	.	intergenic	-0.08	737714 rs7883	2
AAAAC.GTTTT	10	65668150	65668170	4.2	ENSSSCT00000029333	.	intron	0.228	rs787288743	2
AAAC.GTTT	10	65900890	65900910	5.25	.	.	intergenic	.	rs789292636	2
AC.GT	10	66200998	66201008	5.5	.	.	intergenic	0.039	.	2
AC.GT	10	66335186	66335199	7	.	.	intergenic	-0.306	297468 rs7930	2
AAAAC.GTTTT	10	66530778	66530803	5.2	.	.	intergenic	.	rs791009543	2
AAGG.CCTT	10	66676695	66676708	3.5	.	.	intergenic	0.085	020656 rs7931	2
AC.GT	10	66738092	66738105	7	.	.	intergenic	-0.012	rs787018054	2
AATG.CATT	10	66907552	66907571	5	.	.	intergenic	0.076	rs787622682	2
AAAAAT.ATTTTT	10	66985185	66985208	4	.	.	intergenic	.	923300 rs7870	2
ACCT.AGGT	10	67260177	67260198	5.5	.	.	intergenic	-0.184	rs790142788	2
AAC.GTT	10	67803496	67803514	6.33333	.	.	intergenic	0.959	524468 rs7918	2
ACAT.ATGT	10	67929356	67929386	7.75	.	.	intergenic	.	rs792488980	2
AAAT.ATTT	10	68008222	68008236	3.75	.	.	intergenic	0.451	904169 rs7932	2
AAAC.GTTT	10	68193298	68193313	4	.	.	intergenic	0.424	rs787711089	2
AAAG.CTTT	10	68206481	68206508	7	.	.	intergenic	.	.	2
AAAG.CTTT	10	68302056	68302070	3.75	.	.	intergenic	0.045	rs787058390	3
AC.GT	10	68330167	68330179	6.5	.	.	intergenic	0.283	.	2
AAAT.ATTT	10	68336632	68336647	4	.	.	intergenic	.	rs790969819	2
AAC.GTT	10	68416466	68416478	4.33333	.	.	intergenic	.	.	3
AC.GT	10	68639189	68639216	14	.	.	intergenic	-0.045	402155 rs7923	6
AT.AT	10	68782001	68782017	8.5	.	.	intergenic	0.524	.	2
AG.CT	10	68883534	68883546	6.5	.	.	intergenic	.	.	3
AAAT.ATTT	10	68951816	68951830	3.75	.	.	intergenic	-0.036	rs787374607	2
AAAG.CTTT	10	68996931	68996953	5.75	.	.	intergenic	.	564384 rs7894	2
AG.CT	10	69106337	69106353	8.5	.	.	intergenic	0.31	rs786736389	4
AC.GT	10	69139759	69139771	6.5	.	.	intergenic	-0.077	.	4
AC.GT	10	69229467	69229489	11.5	.	.	intergenic	0.047	.	4
AAAAC.GTTTT	10	69232996	69233020	5	.	.	intergenic	0.073	747801 rs7886	2
AAAC.GTTT	10	69394781	69394798	4.5	.	.	intergenic	0.296	rs787423545	2

AACT.AGTT	10	69430469	69430483	3.75	.	.	intergenic	-0.02	)17687 rs7936	2
AC.GT	10	69646208	69646219	6	ENSSSCT00000012178	TAF3	intron	-0.002	rs791090906	2
AAAC.GTTT	10	69759936	69759950	3.75	.	.	intergenic	-0.05	rs789222515	2
AAAAG.CTTTT	10	69779442	69779464	3.83333	ENSSSCT00000012181	.	intron	-0.004	rs789232732	3
AC.GT	10	69889437	69889447	5.5	.	.	intergenic	-0.155	rs786962421	2
AT.AT	10	69919784	69919794	5.5	.	.	intergenic	-0.064	.	2
AC.GT	10	70006509	70006520	6	.	.	intergenic	0.82	rs788105202	2
AAAG.CTTT	10	70291679	70291694	4	.	.	intergenic	-0.136	rs789849736	2
AAG.CTT	10	70390471	70390488	6	.	.	intergenic	0.431	rs790943582	2
AG.CT	10	70582338	70582348	5.5	ENSSSCT00000012183	PRKCQ	intron	.	'65738 rs7865	4
AAAC.GTTT	10	70824252	70824271	5	.	.	intergenic	-0.305	)58095 rs7891	2
AT.AT	10	70826704	70826718	7.5	.	.	intergenic	0.128	rs793280060	2
AAAAC.GTTTT	10	70990863	70990887	5	.	.	intergenic	.	rs787727998	2
AC.GT	10	71470792	71470808	8.5	.	.	intergenic	0.152	rs790157304	3
AAAC.GTTT	10	71700955	71700968	3.5	.	.	intergenic	.	rs789310337	2
AAAAC.GTTTT	10	72158058	72158076	3.8	.	.	intergenic	.	.	2
AAAAG.CTTTT	10	72206739	72206758	4	.	.	intergenic	0.205	187674 rs7870	2
AAAC.GTTT	10	72263403	72263422	5	.	.	intergenic	0.292	315794 rs7937	2
AAAT.ATTT	10	72272502	72272522	5.25	.	.	intergenic	-0.16	rs786863476	2
AAAC.GTTT	10	72446649	72446668	5	.	.	intergenic	0.008	.	3
AC.GT	10	72935764	72935776	6.5	.	.	intergenic	.	rs786588170	2
AAAAT.ATTTT	10	72972121	72972138	3	.	.	intergenic	-0.501	rs793361411	2
AC.GT	10	73123504	73123517	7	.	.	intergenic	-0.251	rs793192092	2
AC.GT	10	74091722	74091732	5.5	.	.	intergenic	-0.159	286392 rs7891	3
AAAGC.GCTTT	10	74098179	74098202	4.8	.	.	intergenic	-0.114	rs787337571	2
AT.AT	10	74468600	74468610	5.5	.	.	intergenic	.	rs789126447	2
AG.CT	10	75274833	75274845	6.5	ENSSSCT00000012222	ADARB2	intron	-0.007	529966 rs7919	3
AC.GT	10	75663532	75663542	5.5	ENSSSCT00000012224	LARP4B	intron	-0.197	rs788506794	2
AT.AT	10	75980130	75980145	8	.	.	intergenic	0.152	rs790993763	2
AAG.CTT	10	76123979	76123990	4	.	.	intergenic	.	rs786493516	2
AAAAG.CTTTT	10	76234735	76234750	3.2	ENSSSCT00000012228	ZYMND11	intron	.	rs788569249	2
AAAT.ATTT	10	76304158	76304180	5.75	ENSSSCT00000012228	ZYMND11	intron	-0.233	rs792192209	2
AAAC.GTTT	10	76416463	76416485	5.75	.	.	intergenic	.	rs793492560	2
AT.AT	10	76603122	76603135	7	.	.	intergenic	.	rs792796894	2
AAG.CTT	10	76845150	76845168	6.33333	.	.	intergenic	.	rs787378782	2
AAAAAC.GTTTT	10	76992808	76992830	3.83333	.	.	intergenic	-0.092	)41328 rs7871	2
AAAG.CTTT	10	77000415	77000433	4.75	ENSSSCT00000012233	CTSL	intron	0.307	531463 rs7892	2
AAAAC.GTTTT	10	77246982	77247005	4.8	.	.	intergenic	.	176478 rs7880	2
AATG.CATT	10	77330262	77330278	4.25	.	.	intergenic	0.186	rs790105139	2
AAAT.ATTT	10	77396937	77396954	4.5	.	.	intergenic	0.073	rs787882693	2
AAAAT.ATTTT	10	77513083	77513099	3.4	.	.	intergenic	0.243	rs788604446	2
AC.GT	10	77692029	77692045	8.5	ENSSSCT00000023307	OPTN	intron	-0.099	581484 rs7920	2
AC.GT	10	78597958	78597971	7	.	.	intergenic	.	rs787085042	3
AGG.CCT	10	78604603	78604623	7	ENSSSCT00000028985	.	exon	.	.	2
AAC.GTT	11	48127	48142	5.33333	ENSSSCT00000010149	GULDH	intron	.	rs791505587	2
AC.GT	11	173765	173778	7	ENSSSCT00000024896	IL17D	intron	.	.	2
AC.GT	11	173765	173778	7	ENSSSCT00000030327	IL17D	intron	.	.	2
AAC.GTT	11	179545	179560	5.33333	ENSSSCT00000024896	IL17D	intron	.	.	2
AAC.GTT	11	179545	179560	5.33333	ENSSSCT00000030327	IL17D	intron	.	.	2
AAAAC.GTTTT	11	526787	526810	4.8	.	.	intergenic	0.114	240979 rs7896	2
AAGAG.CTCTT	11	629980	629997	3.6	.	.	intergenic	0.315	rs792550281	2
AAAAT.ATTTT	11	713097	713117	4.2	ENSSSCT00000010161	FGF9	intron	0.579	rs792382611	2
AAAT.ATTT	11	1161722	1161750	7.25	.	.	intergenic	0.043	rs792859607	2
AAAC.GTTT	11	1220048	1220062	3.75	.	.	intergenic	-0.069	rs791323010	2
AAT.ATT	11	1287713	1287737	8.33333	.	.	intergenic	.	312886 rs7863	2
AC.GT	11	2031634	2031647	7	.	.	intergenic	.	rs787994511	4
AC.GT	11	2074625	2074644	10	ENSSSCT00000010177	.	intron	.	rs786953761	2
AAC.GTT	11	2084873	2084889	5.66667	ENSSSCT00000010177	.	intron	.	rs791918534	2
AAACC.GGTTT	11	2454264	2454284	4.2	.	.	intergenic	-0.27	rs787189329	2
AAAAC.GTTTT	11	2728499	2728514	3.2	.	.	intergenic	-0.128	rs790783683	2
AC.GT	11	2839620	2839640	10.5	ENSSSCT00000031012	.	intron	-0.157	rs788563464	4
AAAC.GTTT	11	3082399	3082410	3	.	.	intergenic	-0.145	)27783 rs7870	2
AAAAT.ATTTT	11	3121848	3121865	3.6	.	.	intergenic	.	rs786298174	2
AT.AT	11	3606511	3606520	5	.	.	intergenic	.	528213 rs7866	2
AC.GT	11	3717410	3717422	6.5	.	.	intergenic	0.032	)69621 rs7877	2
AAAAC.GTTTT	11	3865911	3865940	6	.	.	intergenic	.	rs786657393	2
AAAC.GTTT	11	4073777	4073797	5.25	.	.	intergenic	-0.112	rs690065995	2
AAAG.CTTT	11	4240543	4240559	4.25	.	.	intergenic	-0.247	rs786762049	2
AAAC.GTTT	11	4542370	4542391	5.5	.	.	intergenic	.	rs792330356	2
AC.GT	11	5092717	5092727	5.5	.	.	intergenic	.	rs788819692	2
AC.GT	11	5335328	5335339	6	ENSSSCT00000010212	FLT1	intron	-0.119	rs791393148	2
AAAC.GTTT	11	5409527	5409545	4.75	ENSSSCT00000010212	FLT1	intron	0.085	rs791998111	2
AGC.GCT	11	5497293	5497309	5.66667	.	.	intergenic	0.212	rs788173357	2
AAAC.GTTT	11	5620441	5620459	4.75	.	.	intergenic	0.039	rs789381153	2
AAAAC.GTTTT	11	5915402	5915422	4.2	ENSSSCT00000024217	.	intron	-0.078	rs787130959	2
AAAG.CTTT	11	5924888	5924900	3.25	ENSSSCT00000024217	.	intron	.	)23595 rs7902	2
AC.GT	11	6172173	6172187	7.5	ENSSSCT00000029859	MTUS2	intron	-0.186	rs788825312	2
AC.GT	11	6172173	6172187	7.5	ENSSSCT00000010215	MTUS2	intron	-0.186	rs788825312	2
AC.GT	11	6224898	6224910	6.5	.	.	intergenic	-0.027	rs788871162	2
AC.GT	11	6577039	6577052	7	.	.	intergenic	0.423	rs792182315	2
AT.AT	11	6762968	6762984	8.5	ENSSSCT00000010220	KATNAL1	intron	0.103	rs790535074	2
AC.GT	11	6813037	6813051	7.5	.	.	intergenic	0.176	.	3
AC.GT	11	7113892	7113904	6.5	.	.	intergenic	.	rs786795307	4
AAAC.GTTT	11	7240921	7240947	6.75	.	.	intergenic	-0.2	140471 rs7928	3

AG.CT	11	7261798	7261810	6.5	ENSSSCT00000010224	ALOX5AP	intron	-0.037	367292 rs7912	3
AAAT.ATTT	11	7265500	7265525	6.5	ENSSSCT00000010224	ALOX5AP	intron	.	rs787577610	2
AAT.ATT	11	7327657	7327668	4	.	.	intergenic	0.152	.	2
AAAAC.GTTTT	11	7370972	7370994	3.83333	.	.	intergenic	-0.242	rs789092509	2
AAAAC.GTTTT	11	7761984	7762007	4.8	.	.	intergenic	0.161	rs788713702	2
AT.AT	11	7807629	7807641	6.5	ENSSSCT00000010230	B3GALTL	intron	0.371	rs793226828	2
AT.AT	11	8386484	8386493	5	.	.	intergenic	.	rs790016370	2
AC.GT	11	8495208	8495222	7.5	ENSSSCT00000010234	FRY	intron	1.264	rs793092356	2
AAAC.GTTT	11	8505761	8505780	5	ENSSSCT00000010234	FRY	promoter	1.618	.	2
AG.CT	11	8638241	8638257	8.5	ENSSSCT00000032040	.	intron	-0.042	575220 rs7928	2
AAAC.GTTT	11	8884963	8884986	6	.	.	intergenic	.	rs792037397	2
AG.CT	11	9192876	9192886	5.5	.	.	intergenic	0.059	524280 rs7869	3
AAG.CTT	11	9800493	9800514	7.33333	.	.	intergenic	0.435	rs790766612	2
AT.AT	11	9878160	9878170	5.5	.	.	intergenic	0.073	rs788356695	3
AAAG.CTTT	11	10123165	10123185	5.25	ENSSSCT00000010246	RFC3	intron	0.003	508077 rs7932	2
AT.AT	11	10171645	10171662	9	ENSSSCT00000010246	RFC3	intron	.	.	2
AC.GT	11	10303756	10303771	8	.	.	intergenic	-0.1	.	4
AAAC.GTTT	11	10385783	10385801	4.75	.	.	intergenic	0.1	429296 rs7930	2
ATC.GAT	11	10431280	10431295	5.33333	.	.	intergenic	0.067	.	2
AC.GT	11	10512727	10512737	5.5	.	.	intergenic	0.035	rs789388232	2
ACAT.ATGT	11	11028749	11028764	4	.	.	intergenic	0.036	.	2
AAC.GTT	11	11128046	11128059	4.66667	.	.	intergenic	-0.025	.	3
AAC.GTT	11	11380132	11380145	4.66667	.	.	intergenic	0.125	rs790500902	2
AAAT.ATTT	11	11450710	11450728	4.75	ENSSSCT00000010247	.	intron	-0.104	rs786547237	2
AAAAT.ATTTT	11	11517590	11517608	3.8	ENSSSCT00000010247	.	intron	.	rs790064464	2
AC.GT	11	11527631	11527643	6.5	ENSSSCT00000010247	.	intron	-0.155	rs787336372	2
AAAT.ATTT	11	11558725	11558756	8	ENSSSCT00000010247	.	intron	-0.223	rs789870668	2
AAAC.GTTT	11	11640659	11640673	3.75	ENSSSCT00000010247	.	intron	0.448	rs787251379	2
AAAAT.ATTTT	11	12072180	12072197	3.6	.	.	intergenic	0.101	rs789085784	2
AC.GT	11	12333658	12333672	7.5	.	.	intergenic	-0.053	rs787914685	4
AAAC.GTTT	11	12463676	12463694	4.75	.	.	intergenic	.	194290 rs7888	3
AAAC.GTTT	11	12530159	12530178	5	.	.	intergenic	0.152	rs792622607	2
AAAC.GTTT	11	12670543	12670565	5.75	ENSSSCT00000010254	SMAD9	intron	0.057	353914 rs7938	3
AAG.CTT	11	12718804	12718817	4.66667	.	.	intergenic	0.024	rs791623841	2
AG.CT	11	12770068	12770080	6.5	.	.	intergenic	.	rs792815622	2
AG.CT	11	12949265	12949278	7	.	.	intergenic	0.214	rs791273572	2
AAG.CTT	11	12977745	12977760	5.33333	.	.	intergenic	0.132	.	2
AC.GT	11	13272953	13272967	7.5	.	.	intergenic	-0.142	rs787500556	2
AAAT.ATTT	11	13305521	13305540	5	ENSSSCT00000010259	TRPC4	intron	-0.129	rs789084227	4
AC.GT	11	13429379	13429395	8.5	ENSSSCT00000010259	TRPC4	intron	0.032	415444 rs7916	3
AC.GT	11	13473100	13473118	9.5	ENSSSCT00000010259	TRPC4	intron	.	rs696734017	2
AT.AT	11	13618282	13618291	5	.	.	intergenic	-0.521	rs788121657	2
AC.GT	11	13697316	13697325	5	.	.	intergenic	.	.	2
AAAC.GTTT	11	13832392	13832406	3.75	.	.	intergenic	.	794689 rs7926	2
AAC.GTT	11	13856430	13856446	5.66667	.	.	intergenic	.	.	2
AG.CT	11	13996940	13996972	16.5	ENSSSCT00000010261	FREM2	promoter	0.004	rs708630237	2
AAC.GTT	11	14108541	14108554	4.66667	ENSSSCT00000010261	FREM2	intron	0.063	rs786551136	2
AG.CT	11	14196016	14196030	7.5	ENSSSCT00000010261	FREM2	intron	-0.072	rs793509467	2
AT.AT	11	14293417	14293444	14	ENSSSCT00000010264	.	intron	-0.082	rs789313544	2
AT.AT	11	14293417	14293444	14	ENSSSCT00000024776	.	intron	-0.082	rs789313544	2
AC.GT	11	14680800	14680812	6.5	.	.	intergenic	-0.092	027918 rs7927	3
AAACC.GGTTT	11	14954957	14954971	3	.	.	intergenic	0.135	583008 rs7885	2
AGG.CCT	11	14979844	14979859	5.33333	.	.	intergenic	0.204	rs787098870	2
AC.GT	11	15131694	15131705	6	.	.	intergenic	0.076	.	2
AAC.GTT	11	15358219	15358232	4.66667	.	.	intergenic	0.146	rs792733912	2
CACTC.GAGTG	11	15492873	15492895	3.83333	.	.	intergenic	0.092	rs788445609	2
AC.GT	11	15544903	15544917	7.5	ENSSSCT00000010267	FOXO1	3'utr	1.03	457116 rs7887	3
AAAC.GTTT	11	15685085	15685107	5.75	ENSSSCT00000010267	FOXO1	intron	0.18	976749 rs7917	3
AAAAC.GTTTT	11	15892168	15892191	4.8	ENSSSCT00000010271	.	intron	-0.784	520434 rs7901	2
AT.AT	11	15901059	15901077	9.5	.	.	intergenic	-0.986	.	3
AACC.GGTT	11	16093944	16093960	4.25	.	.	intergenic	.	rs789986051	2
AC.GT	11	16133664	16133676	6.5	ENSSSCT00000010277	NEK5	intron	0.048	280659 rs7864	3
AAAC.GTTT	11	16199636	16199650	3.75	ENSSSCT00000010279	ALG11	intron	0.287	746274 rs7875	2
AAAAC.GTTTT	11	16276011	16276034	4.8	.	.	intergenic	0.111	rs696233964	2
AT.AT	11	16338317	16338326	5	.	.	intergenic	.	rs788637544	3
AG.CT	11	16384835	16384847	6.5	.	.	intergenic	-0.126	rs790796250	2
AG.CT	11	16441365	16441374	5	.	.	intergenic	-0.058	.	2
AAAC.GTTT	11	16450065	16450079	3.75	.	.	intergenic	0.134	rs787115795	2
AAAG.CTTT	11	16564139	16564168	7.5	.	.	intergenic	0.271	.	2
AC.GT	11	16684915	16684931	8.5	.	.	intergenic	0.201	rs789649934	2
AC.GT	11	16740223	16740233	5.5	ENSSSCT00000010282	INTS6	intron	0.347	.	2
AAAAG.CTTTT	11	16758952	16758971	3.33333	ENSSSCT00000010282	INTS6	intron	0.033	rs791808133	2
AT.AT	11	16777069	16777078	5	ENSSSCT00000010282	INTS6	intron	1.368	rs787718591	2
AAAAC.GTTTT	11	16896321	16896335	3	.	.	intergenic	.	rs793238884	2
AAT.ATT	11	17124401	17124414	4.66667	.	.	intergenic	0.297	rs791755326	2
AC.GT	11	17232563	17232576	7	.	.	intergenic	0.048	rs790302347	2
AT.AT	11	18311367	18311380	7	ENSSSCT00000010292	EBPL	intron	0.079	rs788887634	2
AAC.GTT	11	18325102	18325118	5.66667	ENSSSCT00000010292	EBPL	intron	.	rs792964408	2
AAAC.GTTT	11	18478741	18478755	3.75	ENSSSCT00000023143	RCBTB1	intron	-0.224	rs789709781	2
AAAC.GTTT	11	18529356	18529378	5.75	ENSSSCT00000010295	PHF11	intron	0.851	rs711546293	2
AC.GT	11	18564081	18564097	8.5	ENSSSCT00000010295	PHF11	intron	0.141	.	3
AAC.GTT	11	18613504	18613522	6.33333	.	.	intergenic	-0.182	719806 rs7904	2
AAT.ATT	11	19000744	19000761	6	ENSSSCT00000026092	FNDC3A	intron	0.187	rs791535583	2
AG.CT	11	19089767	19089777	5.5	ENSSSCT00000028831	.	intron	.	rs790528832	3

AAAC.GTTT	11	19114449	19114470	5.5	ENSSSCT00000028831	.	intron	.	rs788346310	2
AT.AT	11	19139264	19139274	5.5	ENSSSCT00000028831	.	intron	.	506124 rs7879	2
AAAG.CTTT	11	19253226	19253239	3.5	.	.	intergenic	0.721	rs792397382	2
AAAAC.GTTTT	11	19853189	19853212	4.8	ENSSSCT00000010302	ITM2B	intron	0.085	109866 rs7928	2
AC.GT	11	20183804	20183814	5.5	.	.	intergenic	0.057	.	5
AGC.GCT	11	20446227	20446238	4	.	.	intergenic	-0.072	rs789013524	2
AC.GT	11	20455025	20455053	14.5	.	.	intergenic	0.035	)99580 rs7913	3
AC.GT	11	20640661	20640670	5	.	.	intergenic	-0.085	.	3
AT.AT	11	20749734	20749745	6	.	.	intergenic	.	rs786823539	2
AAAC.GTTT	11	21268858	21268877	5	ENSSSCT00000010309	LRCH1	intron	-0.03	rs788881331	3
AAAC.GTTT	11	21310697	21310715	4.75	.	.	intergenic	0.415	757241 rs7870	2
AAC.GTT	11	21468078	21468092	5	.	.	intergenic	.	389123 rs7930	2
AAG.CTT	11	21469497	21469510	4.66667	.	.	intergenic	-0.089	rs792867354	2
AAAAT.ATTTT	11	21484118	21484140	4.6	.	.	intergenic	0.243	560002 rs7889	2
AAAG.CTTT	11	21689334	21689348	3.75	.	.	intergenic	-0.003	rs793141889	2
AAAAG.CTTTT	11	22082573	22082590	3.6	.	.	intergenic	0.063	rs791741317	2
AAAC.GTTT	11	22337264	22337282	4.75	.	.	intergenic	.	376684 rs7881	2
AC.GT	11	22337353	22337367	7.5	.	.	intergenic	.	)43048 rs7934	5
AAAT.ATTT	11	22461467	22461493	6.75	.	.	intergenic	.	203537 rs7868	2
AAAAT.ATTTT	11	22468046	22468078	6.6	.	.	intergenic	-0.082	529457 rs7873	2
AGAT.ATCT	11	22509881	22509906	6.5	.	.	intergenic	0.11	rs789987469	2
AAC.GTT	11	22660462	22660489	9.33333	.	.	intergenic	.	rs788091861	2
AAAC.GTTT	11	22768463	22768493	7.75	.	.	intergenic	0.389	.	2
AT.AT	11	22806354	22806367	7	.	.	intergenic	.	rs788749462	2
AAAC.GTTT	11	23449346	23449364	4.75	.	.	intergenic	0.075	.	2
AAAC.GTTT	11	23654817	23654839	5.75	.	.	intergenic	0.129	412966 rs7923	2
AAAT.ATTT	11	23755167	23755181	3.75	.	.	intergenic	0.225	rs789236549	2
AATT.AATT	11	23785863	23785882	5	.	.	intergenic	.	rs793537936	2
AAAAT.ATTTT	11	23903742	23903765	4.8	.	.	intergenic	-0.296	rs788816977	2
AC.GT	11	24147051	24147061	5.5	.	.	intergenic	0.177	rs792666490	2
AG.CT	11	24473218	24473227	5	ENSSSCT00000022928	ENOX1	intron	.	rs710177075	2
AC.GT	11	24535137	24535147	5.5	.	.	intergenic	-0.142	)44770 rs7908	3
AG.CT	11	24541251	24541270	10	.	.	intergenic	-0.155	rs793735416	2
AAAT.ATTT	11	24554565	24554586	5.5	.	.	intergenic	0.123	rs792419758	3
AAAAC.GTTTT	11	24663852	24663870	3.8	ENSSSCT00000010334	DNAJC15	intron	0.168	rs788599592	2
AAT.ATT	11	24830001	24830012	4	.	.	intergenic	.	rs793261158	2
AC.GT	11	24877976	24877986	5.5	.	.	intergenic	0.009	.	2
AAT.ATT	11	25005858	25005870	4.33333	.	.	intergenic	0.406	rs793614763	2
AAT.ATT	11	25247825	25247859	11.6667	.	.	intergenic	.	rs790576500	2
AAAT.ATTT	11	25274744	25274765	5.5	.	.	intergenic	0.025	741642 rs7892	2
AG.CT	11	25436112	25436124	6.5	ENSSSCT00000010337	AKAP11	intron	0.229	582247 rs7885	2
AG.CT	11	25914662	25914673	6	ENSSSCT00000026550	.	intron	0.101	rs787756192	2
AC.GT	11	26101626	26101640	7.5	ENSSSCT00000031457	.	intron	0.304	rs791177682	2
AC.GT	11	26185231	26185244	7	.	.	intergenic	0.532	rs793359705	2
AAAC.GTTT	11	26225702	26225724	5.75	.	.	intergenic	.	rs792635502	2
AAC.GTT	11	26589647	26589660	4.66667	ENSSSCT00000010343	MTRF1	intron	1.243	426583 rs7916	2
AC.GT	11	26643171	26643215	22.5	.	.	intergenic	0.977	rs788493465	3
AT.AT	11	26983119	26983134	8	.	.	intergenic	-0.035	.	4
AG.CT	11	27303606	27303616	5.5	.	.	intergenic	.	rs790940477	3
AAAC.GTTT	11	27397440	27397463	6	.	.	intergenic	0.039	rs693251157	2
AC.GT	11	27747312	27747323	6	.	.	intergenic	1.074	986571 rs7905	2
AG.CT	11	27799677	27799716	20	.	.	intergenic	0.007	rs787464622	2
AAAC.GTTT	11	27843783	27843803	5.25	.	.	intergenic	.	rs791395234	2
AGC.GCT	11	27855336	27855349	4.66667	.	.	intergenic	0.117	.	2
AAAC.GTTT	11	28135635	28135651	4.25	.	.	intergenic	-0.005	346219 rs7935	4
AT.AT	11	28341554	28341567	7	.	.	intergenic	.	339519 rs7874	2
\AAAAC.GTTTT	11	28350372	28350390	3.16667	.	.	intergenic	-0.116	rs789440481	2
AC.GT	11	28467898	28467910	6.5	.	.	intergenic	0.008	rs788163006	3
AC.GT	11	28518661	28518684	12	.	.	intergenic	.	)34754 rs7917	2
AAAC.GTTT	11	28546012	28546027	4	.	.	intergenic	0.088	rs793334926	2
AT.AT	11	28565917	28565932	8	.	.	intergenic	-0.119	336808 rs7888	2
AAAC.GTTT	11	28580062	28580076	3.75	.	.	intergenic	.	rs786935309	2
AAAG.CTTT	11	28792035	28792048	3.5	.	.	intergenic	-0.121	rs709145132	2
AAT.ATT	11	28809634	28809650	5.66667	.	.	intergenic	.	227700 rs7905	2
AAAC.GTTT	11	28967218	28967251	8.5	.	.	intergenic	0.059	rs790728000	2
AAAAC.GTTTT	11	28988196	28988222	5.4	.	.	intergenic	-0.075	rs791863553	3
AAC.GTT	11	29091869	29091885	5.66667	.	.	intergenic	1.44	251637 rs7872	2
AT.AT	11	29104105	29104118	7	.	.	intergenic	.	rs792251561	2
AT.AT	11	29211050	29211067	9	.	.	intergenic	0.017	rs788655755	3
AAAT.ATTT	11	29388288	29388310	5.75	.	.	intergenic	.	795049 rs7905	2
AC.GT	11	29436202	29436213	6	.	.	intergenic	0.169	rs792688635	2
AT.AT	11	29475683	29475693	5.5	.	.	intergenic	-0.245	390741 rs7905	2
AT.AT	11	29572459	29572469	5.5	.	.	intergenic	0.065	rs793620402	2
AAAC.GTTT	11	29575436	29575448	3.25	.	.	intergenic	-0.033	rs792652355	2
AAC.GTT	11	29614715	29614735	7	.	.	intergenic	0.279	rs790282037	2
AC.GT	11	29635635	29635644	5	.	.	intergenic	-0.031	rs792745543	3
AC.GT	11	29668971	29668981	5.5	.	.	intergenic	-0.019	rs788952389	2
AT.AT	11	29712914	29712927	7	.	.	intergenic	0.292	.	5
AAAT.ATTT	11	29816187	29816209	5.75	.	.	intergenic	.	rs791466976	2
AAAAC.GTTTT	11	29964172	29964196	5	.	.	intergenic	.	.	2
AG.CT	11	30376827	30376844	9	.	.	intergenic	-0.179	rs791092084	3
AG.CT	11	30640523	30640537	7.5	.	.	intergenic	.	rs792809471	2
AAT.ATT	11	30679625	30679636	4	.	.	intergenic	.	rs786472790	2
AT.AT	11	30861001	30861013	6.5	.	.	intergenic	.	789015 rs7918	2

AAAT.ATTT	11	30969266	30969285	5	.	.	intergenic	-0.047	.	2
AT.AT	11	31292577	31292595	9.5	.	.	intergenic	0.051	.	2
AGGGG.CCCCT	11	31514450	31514466	3.4	.	.	intergenic	.	.	2
AC.GT	11	31691283	31691297	7.5	.	.	intergenic	-0.063	.	2
AAT.ATT	11	31955490	31955509	6.66667	.	.	intergenic	.	182780 rs7937	2
AAAAG.CTTTT	11	32344667	32344688	4.4	.	.	intergenic	-0.111	rs789114717	2
AC.GT	11	32435281	32435301	10.5	.	.	intergenic	.	108267 rs7915	2
AAG.CTT	11	32567683	32567697	5	.	.	intergenic	.	rs792108250	3
AC.GT	11	32722876	32722891	8	.	.	intergenic	0.043	rs788594733	2
AAC.GTT	11	33185590	33185603	4.66667	.	.	intergenic	-0.029	rs787864838	3
AAAAC.GTTTT	11	33548942	33548970	5.8	.	.	intergenic	-0.05	)85462 rs7862	2
AC.GT	11	34495438	34495462	12.5	.	.	intergenic	-0.275	rs787504577	2
AC.GT	11	34576472	34576486	7.5	.	.	intergenic	-0.179	rs792083088	2
AT.AT	11	34658097	34658112	8	ENSSSCT00000010357	.	intron	0.003	rs793469890	2
AATG.CATT	11	34769824	34769848	6.25	.	.	intergenic	0.077	)22008 rs7894	2
AC.GT	11	34817022	34817031	5	.	.	intergenic	0.49	rs791821248	2
AAT.ATT	11	34964720	34964733	4.66667	.	.	intergenic	0.166	257744 rs7915	2
AAAG.CTTT	11	34972418	34972432	3.75	.	.	intergenic	-0.014	rs791606493	2
AAAC.GTTT	11	34978823	34978842	5	.	.	intergenic	.	rs792404293	2
AC.GT	11	35011621	35011634	7	ENSSSCT00000010359	.	intron	.	301514 rs7867	4
AC.GT	11	35510234	35510262	14.5	.	.	intergenic	0.217	543058 rs7921	4
AAAC.GTTT	11	35518972	35518995	6	.	.	intergenic	0.224	rs786229412	3
ATAT.ATAT	11	35809341	35809382	10.5	.	.	intergenic	0.172	rs790830185	2
ACAT.ATGT	11	35900093	35900107	3.75	.	.	intergenic	-0.245	.	2
AT.AT	11	36016472	36016481	5	.	.	intergenic	.	rs793502107	2
AG.CT	11	36029218	36029232	7.5	.	.	intergenic	.	rs691576979	2
AT.AT	11	36179750	36179761	6	.	.	intergenic	.	rs789547208	2
AC.GT	11	36605840	36605872	16.5	.	.	intergenic	0.088	212166 rs7913	3
AAAC.GTTT	11	37129483	37129506	6	.	.	intergenic	1.031	596007 rs7887	2
AAATAT.ATATTT	11	37367079	37367104	4.33333	.	.	intergenic	.	.	2
AGAT.ATCT	11	37975459	37975479	5.25	.	.	intergenic	-0.1	311169 rs7878	2
AT.AT	11	38284630	38284652	11.5	.	.	intergenic	.	rs787350232	2
AAAAC.GTTTT	11	38495732	38495750	3.8	.	.	intergenic	.	427205 rs7911	2
AGAT.ATCT	11	38583636	38583672	9.25	.	.	intergenic	0.568	.	2
AT.AT	11	38996381	38996396	8	.	.	intergenic	-0.306	rs788231268	3
AAG.CTT	11	39582532	39582548	5.66667	.	.	intergenic	0.128	rs791639838	2
AAAT.ATTT	11	39741814	39741829	4	.	.	intergenic	.	rs788040796	2
AAAT.ATTT	11	39844699	39844733	8.75	.	.	intergenic	.	rs698100049	2
AT.AT	11	39909345	39909357	6.5	.	.	intergenic	.	.	2
AAAC.GTTT	11	40122611	40122625	3.75	.	.	intergenic	.	.	4
AG.CT	11	40599107	40599121	7.5	.	.	intergenic	-0.315	rs792043943	2
AAAAT.ATTTT	11	40749454	40749477	4.8	.	.	intergenic	.	987226 rs7866	2
AAAC.GTTT	11	40989959	40989980	5.5	.	.	intergenic	.	956349 rs7913	2
AAT.ATT	11	41048767	41048781	5	.	.	intergenic	0.084	.	2
AG.CT	11	41139631	41139642	6	.	.	intergenic	-0.068	.	2
AT.AT	11	41184854	41184872	9.5	.	.	intergenic	.	166002 rs7932	2
AT.AT	11	41606341	41606351	5.5	.	.	intergenic	-0.065	rs793638147	4
AAAT.ATTT	11	41737912	41737926	3.75	.	.	intergenic	-0.105	rs788686791	2
AC.GT	11	41765641	41765653	6.5	.	.	intergenic	.	.	3
AAAAT.ATTTT	11	41891818	41891846	5.8	.	.	intergenic	-0.031	rs791500176	2
AT.AT	11	42068362	42068385	12	.	.	intergenic	0.029	rs792871787	2
AATAC.GTATT	11	43094348	43094365	3.6	.	.	intergenic	0.714	rs789114243	2
AT.AT	11	43252442	43252458	8.5	.	.	intergenic	.	.	3
AAC.GTT	11	43407566	43407582	5.66667	.	.	intergenic	0.337	.	2
AAT.ATT	11	43420842	43420855	4.66667	.	.	intergenic	-0.071	rs787352257	2
ATC.GAT	11	43452068	43452087	6.66667	.	.	intergenic	0.192	.	2
AGG.CCT	11	43490723	43490735	4.33333	.	.	intergenic	0.411	306342 rs7892	2
AAAC.GTTT	11	43558387	43558419	8.25	.	.	intergenic	.	954101 rs7895	2
AAAG.CTTT	11	43585769	43585781	3.25	.	.	intergenic	-0.02	740128 rs7862	2
AT.AT	11	43886811	43886825	7.5	.	.	intergenic	.	.	2
AT.AT	11	43975755	43975776	11	.	.	intergenic	-0.165	480922 rs7874	2
AC.GT	11	44041750	44041769	10	.	.	intergenic	-0.043	rs703129254	4
AAAAAT.ATTTTT	11	44175417	44175439	3.83333	.	.	intergenic	.	.	2
AATAT.ATATT	11	44401061	44401077	3.4	.	.	intergenic	0.096	.	2
AAAAAC.GTTTTT	11	45005977	45006005	4.83333	.	.	intergenic	-0.055	rs791573445	2
AC.GT	11	45042696	45042709	7	.	.	intergenic	-0.014	)45889 rs7885	2
AAAT.ATTT	11	45054624	45054659	9	.	.	intergenic	.	rs788414690	2
AAAC.GTTT	11	45554345	45554367	5.75	.	.	intergenic	-0.426	rs790907355	2
AAC.GTT	11	45627809	45627828	6.66667	.	.	intergenic	.	737671 rs7871	3
AAAC.GTTT	11	45643444	45643460	4.25	.	.	intergenic	-0.004	986492 rs7863	3
AG.CT	11	46116611	46116621	5.5	.	.	intergenic	0.104	rs787909042	3
AAAAC.GTTTT	11	46141477	46141508	6.4	.	.	intergenic	-0.059	rs792684620	2
AG.CT	11	46149492	46149508	8.5	.	.	intergenic	.	334297 rs7932	4
AT.AT	11	46221614	46221636	11.5	.	.	intergenic	-0.103	252566 rs7920	2
ATCC.GGAT	11	46268482	46268524	10.75	ENSSSCT00000010366	KLHL1	intron	-0.177	.	2
ACT.AGT	11	46287713	46287733	7	ENSSSCT00000010366	KLHL1	intron	0.036	rs793674111	2
AATT.AATT	11	46293142	46293154	3.25	ENSSSCT00000010366	KLHL1	intron	0.069	460961 rs7887	2
AAC.GTT	11	46463108	46463121	4.66667	ENSSSCT00000010366	KLHL1	intron	-0.144	rs792991936	2
AAAC.GTTT	11	46553538	46553562	6.25	.	.	intergenic	.	rs791298226	2
AAT.ATT	11	46672825	46672842	6	.	.	intergenic	-0.116	rs792431749	2
AG.CT	11	46734785	46734796	6	.	.	intergenic	.	rs787390780	2
AAAAT.ATTTT	11	46777723	46777742	4	.	.	intergenic	-0.108	rs787883830	2
AAAT.ATTT	11	47215934	47215956	5.75	.	.	intergenic	.	206494 rs7886	2
AAAAC.GTTTT	11	47548665	47548691	5.4	.	.	intergenic	.	996341 rs7866	2

AT.AT	11	47619016	47619034	9.5	.	.	intergenic	-0.094	.	4
AT.AT	11	47688815	47688839	12.5	.	.	intergenic	0.465	358123 rs7916	3
AG.CT	11	48334572	48334582	5.5	ENSSSCT00000010367	.	intron	0.126	.	2
AAC.GTT	11	48357999	48358019	7	ENSSSCT00000010367	.	intron	0.225	910035 rs7927	4
AC.GT	11	48470428	48470440	6.5	ENSSSCT00000010367	.	intron	0.158	319428 rs7882	3
AAAC.GTTT	11	48641735	48641750	4	.	.	intergenic	0.105	336576 rs7925	2
ATC.GAT	11	48701428	48701443	5.33333	.	.	intergenic	0.03	336282 rs7866	2
AAAG.CTTT	11	48847621	48847649	7.25	.	.	intergenic	0.184	396798 rs7913	2
AAC.GTT	11	49058300	49058314	5	.	.	intergenic	.	rs789915022	2
AT.AT	11	49331813	49331842	15	.	.	intergenic	0.12	rs791672511	2
AAAC.GTTT	11	49545561	49545575	3.75	.	.	intergenic	.	rs791290065	3
AC.GT	11	49605902	49605918	8.5	ENSSSCT00000010370	DIS3	intron	0.245	366436 rs7872	2
AC.GT	11	49707438	49707449	6	ENSSSCT00000025113	.	intron	0.074	rs792514803	2
AACC.GGTT	11	49952386	49952420	8.75	.	.	intergenic	0.35	540418 rs7866	2
AAG.CTT	11	50012522	50012533	4	.	.	intergenic	.	.	4
AG.CT	11	50033239	50033251	6.5	ENSSSCT00000010373	KLF5	promoter	-0.447	383083 rs7917	2
AG.CT	11	50310928	50310943	8	.	.	intergenic	0.126	rs792201362	2
AC.GT	11	50414537	50414548	6	.	.	intergenic	.	.	3
AAC.GTT	11	50476785	50476804	6.66667	.	.	intergenic	0.162	rs789454701	2
AT.AT	11	50597519	50597544	13	.	.	intergenic	-0.048	.	2
AAAC.GTTT	11	50597680	50597692	3.25	.	.	intergenic	0.004	.	2
AC.GT	11	50708521	50708530	5	.	.	intergenic	-0.101	rs786476259	2
AAAC.GTTT	11	50999526	50999544	4.75	.	.	intergenic	0.003	rs789037479	2
AAAT.ATTT	11	51214738	51214752	3.75	.	.	intergenic	0.442	rs790146137	2
ATC.GAT	11	51247168	51247180	4.33333	.	.	intergenic	0.135	rs791451231	2
AC.GT	11	51325981	51325991	5.5	.	.	intergenic	1.054	rs693516300	2
AAC.GTT	11	51456482	51456496	5	.	.	intergenic	.	.	2
AAT.ATT	11	51700830	51700843	4.66667	.	.	intergenic	.	rs788659090	2
AT.AT	11	51717635	51717645	5.5	.	.	intergenic	0.859	297748 rs7868	3
AAAT.ATTT	11	51795711	51795738	7	.	.	intergenic	0.262	rs789639136	2
AAC.GTT	11	51854588	51854599	4	.	.	intergenic	.	.	2
AAC.GTT	11	51893871	51893887	5.66667	.	.	intergenic	.	rs791205663	3
AAAC.GTTT	11	52026323	52026355	8.25	.	.	intergenic	0.302	rs792280597	2
AC.GT	11	52111800	52111809	5	.	.	intergenic	0.046	rs790249605	2
AT.AT	11	52161404	52161426	11.5	ENSSSCT00000010376	.	3'utr	1.223	rs788854435	2
AAC.GTT	11	52303351	52303366	5.33333	.	.	intergenic	0.178	rs787407054	3
AAC.GTT	11	52443163	52443185	7.66667	ENSSSCT00000010377	UCHL3	intron	0.994	rs788554344	3
AC.GT	11	52452549	52452558	5	ENSSSCT00000010377	UCHL3	intron	0.161	581217 rs7930	2
AAAT.ATTT	11	52522101	52522117	4.25	.	.	intergenic	0.225	rs792502294	2
AGC.GCT	11	52803192	52803204	4.33333	.	.	intergenic	0.058	rs791454439	2
AC.GT	11	52834914	52834928	7.5	.	.	intergenic	-0.046	.	4
AAAAT.ATTTT	11	52886072	52886096	5	.	.	intergenic	0.038	.	2
AG.CT	11	52931932	52931943	6	.	.	intergenic	-0.137	rs786357461	3
AAAAC.GTTTT	11	53042330	53042345	3.2	.	.	intergenic	-0.036	rs786937544	2
AAGG.CCTT	11	53105710	53105740	7.75	.	.	intergenic	0.127	rs787443944	2
AC.GT	11	53197847	53197856	5	.	.	intergenic	0.008	.	3
AAAT.ATTT	11	53202335	53202357	5.75	.	.	intergenic	0.165	rs787320216	2
AT.AT	11	53369470	53369482	6.5	.	.	intergenic	0.092	rs786717608	2
AC.GT	11	53589105	53589124	10	.	.	intergenic	0.188	290420 rs7869	2
AAAT.ATTT	11	53611573	53611605	8.25	.	.	intergenic	.	rs786477143	2
AG.CT	11	53635741	53635769	14.5	.	.	intergenic	0.098	rs793689260	3
AG.CT	11	53718369	53718383	7.5	ENSSSCT00000010382	.	intron	0.066	rs789601831	2
AAAT.ATTT	11	53822698	53822725	7	ENSSSCT00000010386	MYCBP2	intron	3.303	rs788500524	2
AACC.GGTT	11	53846986	53847007	5.5	ENSSSCT00000010386	MYCBP2	intron	0.182	.	2
AAC.GTT	11	54142031	54142048	6	.	.	intergenic	0.229	rs791807607	2
AAAC.GTTT	11	54157089	54157101	3.25	.	.	intergenic	.	rs792531550	3
AATG.CATT	11	54211064	54211083	5	.	.	intergenic	-0.024	509688 rs7889	2
AT.AT	11	54259468	54259478	5.5	.	.	intergenic	-0.012	rs791600090	2
AAAC.GTTT	11	54260098	54260117	5	.	.	intergenic	0.014	163856 rs7928	2
AAAC.GTTT	11	54295849	54295863	3.75	.	.	intergenic	-0.105	rs789180678	2
AG.CT	11	54305366	54305376	5.5	ENSSSCT00000010387	SCEL	promoter	0.189	rs791294894	3
AAAGG.CCTTT	11	54312315	54312335	3.5	ENSSSCT00000010387	SCEL	intron	0.044	506143 rs7933	2
AAAT.ATTT	11	54585047	54585066	5	.	.	intergenic	.	559178 rs7923	2
AG.CT	11	54598648	54598658	5.5	.	.	intergenic	0.097	rs791961709	4
AC.GT	11	54675583	54675593	5.5	.	.	intergenic	.	rs790811785	2
AAAAT.ATTTT	11	54707881	54707905	5	ENSSSCT00000010390	EDNRB	intron	0.011	rs793728456	2
AATT.AATT	11	54714515	54714535	5.25	ENSSSCT00000010390	EDNRB	intron	0.253	rs787308011	2
AAAT.ATTT	11	54876345	54876364	5	.	.	intergenic	0.104	rs787373665	2
AC.GT	11	54996153	54996163	5.5	.	.	intergenic	0.52	362625 rs7879	4
AC.GT	11	54998951	54998963	6.5	.	.	intergenic	.	.	3
AAC.GTT	11	55094415	55094435	7	.	.	intergenic	0.015	rs711970894	4
AAAC.GTTT	11	55222290	55222308	4.75	.	.	intergenic	0.068	rs793672930	2
AAAG.CTTT	11	55245598	55245612	3.75	.	.	intergenic	0.031	.	2
AAAC.GTTT	11	55248945	55248963	4.75	.	.	intergenic	0.27	rs789503557	2
AAAG.CTTT	11	55506610	55506623	3.5	.	.	intergenic	0.15	rs790021609	2
AT.AT	11	55964566	55964576	5.5	.	.	intergenic	.	rs788370548	3
AAAC.GTTT	11	56228851	56228869	4.75	.	.	intergenic	.	rs793509576	2
AAAAT.ATTTT	11	56397614	56397634	4.2	.	.	intergenic	.	533393 rs7916	2
AAAC.GTTT	11	56730133	56730147	3.75	.	.	intergenic	0.324	rs788545978	2
AT.AT	11	57088580	57088604	12.5	.	.	intergenic	-0.382	rs789107174	2
AAAC.GTTT	11	57315026	57315048	5.75	.	.	intergenic	0.105	245168 rs7930	2
AC.GT	11	57949716	57949733	9	.	.	intergenic	.	rs786316012	2
AAT.ATT	11	57993939	57993954	5.33333	.	.	intergenic	0.016	rs787313452	2
AAAC.GTTT	11	58121424	58121440	4.25	.	.	intergenic	.	307428 rs7898	2

AT.AT	11	58121878	58121911	17	.	.	intergenic	0.196	314409 rs7877	2
AAAT.ATTT	11	58349296	58349322	6.75	.	.	intergenic	-0.113	rs789532993	2
AC.GT	11	58606794	58606806	6.5	.	.	intergenic	0.08	.	3
AC.GT	11	58673662	58673693	16	.	.	intergenic	.	356959 rs7925	4
ACAT.ATGT	11	58757785	58757800	4	.	.	intergenic	-0.151	rs791184979	2
AC.GT	11	58784694	58784707	7	.	.	intergenic	.	rs691559367	4
AC.GT	11	58908779	58908790	6	.	.	intergenic	-0.192	rs786845773	4
ACAT.ATGT	11	59027556	59027571	4	.	.	intergenic	.	rs788360019	3
AC.GT	11	59107172	59107190	9.5	.	.	intergenic	.	)30628 rs7903	2
ACAT.ATGT	11	59239166	59239178	3.25	.	.	intergenic	.	rs788779542	2
AC.GT	11	59742691	59742703	6.5	.	.	intergenic	.	rs789374600	2
AAT.ATT	11	59865388	59865418	10.3333	.	.	intergenic	0.075	353318 rs7908	2
AAAAAC.GTTTT	11	59880261	59880283	3.83333	.	.	intergenic	.	.	2
AAC.GTT	11	59881878	59881892	5	.	.	intergenic	.	.	2
AAC.GTT	11	59914265	59914279	5	.	.	intergenic	.	rs788778989	3
AGAT.ATCT	11	60002283	60002303	5.25	.	.	intergenic	0.046	497159 rs7934	2
AT.AT	11	60071584	60071604	10.5	.	.	intergenic	.	rs789108428	2
AGATAT.ATATC	11	60273524	60273546	3.83333	.	.	intergenic	.	265274 rs7924	2
AAT.ATT	11	60290366	60290383	6	.	.	intergenic	-0.093	rs789915760	3
AC.GT	11	60954777	60954790	7	.	.	intergenic	-0.043	rs786947221	2
AC.GT	11	61009553	61009572	10	.	.	intergenic	-0.405	594753 rs7896	2
ACT.AGT	11	61317930	61317948	6.33333	.	.	intergenic	-0.045	rs789375038	3
AC.GT	11	61478654	61478669	8	.	.	intergenic	-0.013	269140 rs7935	3
AAC.GTT	11	61863097	61863111	5	.	.	intergenic	-0.001	rs793124347	2
AC.GT	11	62040996	62041008	6.5	.	.	intergenic	-0.119	.	4
AATCAT.ATGAT	11	62057913	62057933	3.5	.	.	intergenic	0.202	rs786219681	2
AAT.ATT	11	62379063	62379078	5.33333	.	.	intergenic	0.14	925893 rs7926	2
AC.GT	11	62407235	62407256	11	.	.	intergenic	.	rs791102912	2
AAAT.ATTT	11	62413126	62413143	4.5	.	.	intergenic	.	.	2
AT.AT	11	62511453	62511470	9	.	.	intergenic	-0.064	)13196 rs7896	3
AT.AT	11	62624152	62624161	5	.	.	intergenic	-0.014	.	2
AG.CT	11	62732136	62732150	7.5	.	.	intergenic	0.004	.	4
AAAC.GTTT	11	62931150	62931164	3.75	.	.	intergenic	.	.	2
AT.AT	11	63053264	63053273	5	.	.	intergenic	.	.	3
AAAC.GTTT	11	63397968	63397990	5.75	.	.	intergenic	-0.167	rs793389302	2
AC.GT	11	63491168	63491190	11.5	.	.	intergenic	-0.124	324493 rs7913	3
AAAC.GTTT	11	63983089	63983111	5.75	.	.	intergenic	.	rs788934121	2
AAAAC.GTTTT	11	63994264	63994287	4.8	.	.	intergenic	.	925198 rs7916	2
AAAC.GTTT	11	64362345	64362379	8.75	.	.	intergenic	-0.227	rs709452796	2
AG.CT	11	64478616	64478638	11.5	.	.	intergenic	0.262	rs704841174	5
AAAC.GTTT	11	64711838	64711853	4	.	.	intergenic	-0.169	503776 rs7875	2
AAAAG.CTTTT	11	64713743	64713761	3.8	.	.	intergenic	-0.206	742676 rs7866	2
AG.CT	11	64726172	64726182	5.5	.	.	intergenic	.	rs786941530	2
AAAT.ATTT	11	64864352	64864373	5.5	.	.	intergenic	-0.277	432501 rs7930	2
AAT.ATT	11	64878754	64878773	6.66667	.	.	intergenic	0.06	.	2
AAAT.ATTT	11	65196714	65196744	7.75	.	.	intergenic	.	.	2
AG.CT	11	65203040	65203057	9	.	.	intergenic	1.02	rs788201781	2
AGAT.ATCT	11	65233463	65233479	4.25	.	.	intergenic	0.052	rs791444215	2
AAAAG.CTTTT	11	65238356	65238374	3.8	.	.	intergenic	0.101	585114 rs7926	2
AAAAT.ATTTT	11	65486311	65486331	4.2	.	.	intergenic	0.138	312239 rs7932	2
AC.GT	11	65519291	65519306	8	.	.	intergenic	0.124	rs791642038	2
AAC.GTT	11	65558343	65558360	6	.	.	intergenic	.	413773 rs7929	2
AT.AT	11	65787088	65787097	5	.	.	intergenic	.	rs790976266	2
AAAT.ATTT	11	66022913	66022928	4	.	.	intergenic	.	rs787815930	2
AAAT.ATTT	11	66129072	66129098	6.75	.	.	intergenic	-0.273	551977 rs7881	2
AT.AT	11	66362320	66362330	5.5	.	.	intergenic	0.256	rs786529796	2
AG.CT	11	66401370	66401382	6.5	.	.	intergenic	0.086	.	2
AAC.GTT	11	66511995	66512007	4.33333	.	.	intergenic	-0.039	rs793801072	3
AAAC.GTTT	11	66575337	66575361	6.25	.	.	intergenic	0.186	rs793716412	2
AT.AT	11	66632955	66632972	9	.	.	intergenic	-0.028	rs791316118	3
AC.GT	11	66784757	66784778	11	.	.	intergenic	0.22	.	2
AGG.CCT	11	67193534	67193552	6.33333	.	.	intergenic	.	.	2
AC.GT	11	67488093	67488103	5.5	.	.	intergenic	-0.176	997547 rs7882	2
AAAT.ATTT	11	67678307	67678321	3.75	.	.	intergenic	-0.152	.	3
AC.GT	11	67749266	67749278	6.5	.	.	intergenic	.	.	2
AAAC.GTTT	11	67851292	67851318	6.75	.	.	intergenic	-0.429	rs791567489	2
AAAAAG.CTTTT	11	67868619	67868637	3.16667	.	.	intergenic	-0.345	.	2
AC.GT	11	67892707	67892727	10.5	.	.	intergenic	.	rs789948154	2
AG.CT	11	67973037	67973048	6	.	.	intergenic	0.012	rs786492754	2
AC.GT	11	67977820	67977848	14.5	.	.	intergenic	.	.	2
AATG.CATT	11	68355150	68355168	4.75	.	.	intergenic	-0.202	411436 rs7867	2
AG.CT	11	68472410	68472424	7.5	.	.	intergenic	0.278	594360 rs7884	2
AT.AT	11	68582233	68582245	6.5	.	.	intergenic	-0.074	.	3
AAAAC.GTTTT	11	68582815	68582837	4.6	.	.	intergenic	0.186	rs787296748	2
AT.AT	11	68737766	68737776	5.5	.	.	intergenic	-0.008	rs707645564	3
AACAC.GTGTT	11	68906543	68906571	5.8	.	.	intergenic	0.448	rs789001271	2
AT.AT	11	68912984	68912994	5.5	.	.	intergenic	0.126	.	2
AAAC.GTTT	11	69169147	69169160	3.5	.	.	intergenic	.	rs786651440	2
AAAC.GTTT	11	69434036	69434064	7.25	.	.	intergenic	0.046	rs790327218	2
AG.CT	11	69477431	69477445	7.5	.	.	intergenic	0.001	514870 rs7882	3
AAAG.CTTT	11	69532691	69532707	4.25	.	.	intergenic	0.281	rs791247442	2
AAAC.GTTT	11	69707678	69707701	6	ENSSSCT00000010405	DCT	intron	-0.255	rs792256572	2
AC.GT	11	69723506	69723516	5.5	.	.	intergenic	-0.002	rs791912599	2
AAAT.ATTT	11	69848910	69848936	6.75	ENSSSCT00000010408	GPR180	intron	0.241	rs787185847	3

AC.GT	11	69869604	69869616	6.5	ENSSSCT00000010407	TGDS	intron	-0.098	rs792281924	2
AG.CT	11	69941011	69941025	7.5	.	.	intergenic	0.044	113412 rs7877	3
AAAAT.ATTTT	11	70175986	70176005	4	ENSSSCT00000032528	.	intron	.	rs789342337	2
AAAAG.CTTTT	11	70314269	70314289	3.5	.	.	intergenic	0.016	.	2
AG.CT	11	70521523	70521534	6	.	.	intergenic	.	rs791325381	2
AAAC.GTTT	11	70529195	70529217	5.75	.	.	intergenic	.	751623 rs7881	2
AT.AT	11	70541994	70542005	6	.	.	intergenic	.	571644 rs7892	2
AAC.GTT	11	71917759	71917780	7.33333	.	.	intergenic	0.045	369937 rs7881	2
AC.GT	11	72017533	72017543	5.5	ENSSSCT00000010418	DZIP1	intron	-0.144	rs788837795	2
AAAAT.ATTTT	11	72139752	72139778	5.4	ENSSSCT00000026790	DNAJC3	intron	.	rs787696529	3
AC.GT	11	72314489	72314500	6	ENSSSCT00000010419	.	intron	-0.111	.	2
AAC.GTT	11	72320921	72320935	5	ENSSSCT00000010419	.	intron	0.187	rs786503617	2
AAAC.GTTT	11	72322321	72322344	6	ENSSSCT00000010419	.	intron	0.275	561972 rs7927	2
AG.CT	11	72768891	72768902	6	.	.	intergenic	0.478	rs786439971	2
AAAT.ATTT	11	72774616	72774635	5	.	.	intergenic	-0.14	.	2
AAT.ATT	11	72814559	72814572	4.66667	.	.	intergenic	0.352	.	3
AAT.ATT	11	72905260	72905271	4	.	.	intergenic	0.323	.	2
AAC.GTT	11	72989873	72989893	7	.	.	intergenic	-0.123	526151 rs7883	2
AT.AT	11	72994098	72994113	8	.	.	intergenic	0.007	.	2
AGC.GCT	11	73193202	73193219	6	.	.	intergenic	0.309	rs792296959	2
AAAC.GTTT	11	73527940	73527961	5.5	ENSSSCT00000010422	MBNL2	intron	0.187	395830 rs7872	2
AAAT.ATTT	11	73770058	73770075	4.5	.	.	intergenic	0.301	rs791595196	2
AC.GT	11	73933915	73933927	6.5	.	.	intergenic	0.224	539533 rs7931	3
AAACC.GGTTT	11	73989425	73989448	4.8	.	.	intergenic	0.188	.	2
AC.GT	11	74021094	74021103	5	.	.	intergenic	0.246	.	3
AG.CT	11	74212264	74212284	10.5	.	.	intergenic	.	.	2
AAAT.ATTT	11	74221469	74221486	4.5	.	.	intergenic	-0.107	rs791219053	2
AAAC.GTTT	11	74230730	74230743	3.5	.	.	intergenic	0.172	231748 rs7890	2
AC.GT	11	75208489	75208505	8.5	.	.	intergenic	0.109	577462 rs7874	2
AAC.GTT	11	75314389	75314401	4.33333	.	.	intergenic	0.074	rs793870879	2
AAT.ATT	11	75377074	75377086	4.33333	.	.	intergenic	0.092	rs710583847	2
AC.GT	11	75390494	75390505	6	ENSSSCT00000010434	UBAC2	intron	-0.037	rs786385243	2
AAAG.CTTT	11	75895940	75895961	5.5	.	.	intergenic	0.051	251452 rs7928	2
AC.GT	11	75953709	75953720	6	.	.	intergenic	0.062	398311 rs7913	2
AAAT.ATTT	11	76143429	76143448	5	.	.	intergenic	.	rs791827958	2
AC.GT	11	76231063	76231072	5	.	.	intergenic	.	.	4
AC.GT	11	76308780	76308794	7.5	.	.	intergenic	-0.094	.	2
AC.GT	11	76381404	76381414	5.5	ENSSSCT00000010440	.	intron	0.056	029591 rs7899	3
AAAAAT.ATTTTT	11	76816230	76816248	3.16667	.	.	intergenic	0.233	.	2
AAAC.GTTT	11	76919733	76919751	4.75	.	.	intergenic	-0.17	769905 rs7877	2
AG.CT	11	77175607	77175616	5	ENSSSCT00000024376	.	intron	.	rs792511496	2
AC.GT	11	77242977	77242987	5.5	.	.	intergenic	0.056	.	4
AAAC.GTTT	11	77251967	77251981	3.75	.	.	intergenic	0.033	rs793818437	2
AATG.CATT	11	77445298	77445313	4	ENSSSCT00000010444	ITGBL1	intron	0.105	rs786896676	2
AC.GT	11	77625537	77625547	5.5	ENSSSCT00000010445	FGF14	intron	0.027	.	2
AC.GT	11	77632003	77632012	5	ENSSSCT00000010445	FGF14	intron	-0.006	.	2
AC.GT	11	77724250	77724261	6	ENSSSCT00000010445	FGF14	intron	0.005	.	3
AAC.GTT	11	77758256	77758269	4.66667	ENSSSCT00000010445	FGF14	intron	.	527079 rs7926	2
AATG.CATT	11	77761670	77761695	6.5	ENSSSCT00000010445	FGF14	intron	-0.128	236497 rs7897	2
AAAAC.GTTTT	11	77901503	77901519	3.4	.	.	intergenic	0.069	rs790677206	2
AG.CT	11	78231661	78231671	5.5	.	.	intergenic	-0.07	.	2
AAG.CTT	11	78299348	78299362	5	ENSSSCT00000010446	.	intron	0.021	rs790753732	2
AAAAAC.GTTTT	11	78313038	78313059	3.66667	ENSSSCT00000010446	.	intron	-0.265	rs793834621	2
AAAAC.GTTTT	11	78331377	78331394	3.6	ENSSSCT00000010447	.	intron	-0.025	rs788387988	2
AAAC.GTTT	11	78420040	78420067	7	ENSSSCT00000026999	.	intron	0.061	rs786275002	2
AAAC.GTTT	11	78420040	78420067	7	ENSSSCT00000010450	.	intron	0.061	rs786275002	2
AT.AT	11	78484023	78484032	5	ENSSSCT00000026999	.	intron	.	rs789775285	2
AT.AT	11	78484023	78484032	5	ENSSSCT00000010450	.	intron	.	rs789775285	2
AAAAC.GTTTT	11	78519624	78519648	5	.	.	intergenic	-0.099	rs788555922	2
AAAG.CTTT	11	78524238	78524252	3.75	.	.	intergenic	-0.318	394180 rs7894	2
ACAT.ATGT	11	78674647	78674661	3.75	.	.	intergenic	.	rs790923508	2
AAAG.CTTT	11	78689232	78689255	6	.	.	intergenic	.	.	2
ATC.GAT	11	79181897	79181910	4.66667	.	.	intergenic	-0.025	301607 rs7889	2
AAAT.ATTT	11	79206302	79206318	4.25	.	.	intergenic	0.056	rs790383575	2
AAAG.CTTT	11	79218586	79218598	3.25	.	.	intergenic	0.055	305972 rs7889	3
AAG.CTT	11	79701328	79701340	4.33333	.	.	intergenic	-0.183	rs792633361	2
AAAC.GTTT	11	79706393	79706430	9.5	.	.	intergenic	0.151	752879 rs7901	2
AAT.ATT	11	79790607	79790622	5.33333	.	.	intergenic	.	.	2
AAAAAC.GTTTT	11	79812053	79812072	3.33333	.	.	intergenic	.	rs792631240	2
AG.CT	11	79813009	79813018	5	.	.	intergenic	-0.098	rs789993801	2
AC.GT	11	79851296	79851322	13.5	.	.	intergenic	.	rs788489213	4
AAAC.GTTT	11	79916107	79916127	5.25	.	.	intergenic	-0.369	rs787942919	2
AAAG.CTTT	11	79938641	79938664	6	.	.	intergenic	-0.619	rs786544994	2
AAC.GTT	11	79952384	79952405	7.33333	.	.	intergenic	0.275	389220 rs7873	3
AG.CT	11	80232406	80232423	9	.	.	intergenic	-0.21	rs791976986	4
AAAC.GTTT	11	80542937	80542959	5.75	.	.	intergenic	-0.199	rs792340603	2
AAAG.CTTT	11	80605644	80605668	6.25	.	.	intergenic	0.082	934138 rs7917	2
AT.AT	11	80620465	80620476	6	.	.	intergenic	0.8	rs786793877	4
AT.AT	11	81012620	81012631	6	.	.	intergenic	.	rs789475347	4
AAC.GTT	11	81237191	81237206	5.33333	.	.	intergenic	0.615	.	2
AT.AT	11	81333534	81333558	12.5	.	.	intergenic	-0.136	rs696997454	2
AC.GT	11	81361726	81361737	6	.	.	intergenic	-0.01	rs787971803	2
AG.CT	11	81380623	81380637	7.5	.	.	intergenic	-0.035	rs793520924	2
AAC.GTT	11	81498183	81498195	4.33333	.	.	intergenic	-0.189	.	2

AAC.GTT	11	81554787	81554800	4.66667	.	.	intergenic	-0.404	rs790140784	2
AG.CT	11	81725018	81725030	6.5	.	.	intergenic	.	rs792236205	2
AC.GT	11	81977432	81977441	5	.	.	intergenic	-0.452	rs789656395	2
AAAC.GTTT	11	82306410	82306430	5.25	.	.	intergenic	-0.068	rs789788399	4
AAAT.ATTT	11	82429193	82429213	5.25	.	.	intergenic	.	100140 rs7873	2
AC.GT	11	82879037	82879047	5.5	.	.	intergenic	-0.236	rs792923445	2
AT.AT	11	82998551	82998561	5.5	.	.	intergenic	.	rs790896202	3
AC.GT	11	83238529	83238551	11.5	.	.	intergenic	0.119	315591 rs7880	2
AG.CT	11	83478416	83478436	10.5	ENSSSCT00000010461	MYO16	intron	0.405	383154 rs7877	2
AC.GT	11	83536413	83536427	7.5	ENSSSCT00000010461	MYO16	intron	0.093	rs793463374	2
AC.GT	11	84377604	84377614	5.5	ENSSSCT00000010462	COL4A1	intron	-0.273	.	3
AC.GT	11	84508348	84508359	6	.	.	intergenic	-0.161	286132 rs7879	2
AAAT.ATTT	11	84714694	84714706	3.25	.	.	intergenic	.	.	2
AC.GT	11	84757422	84757432	5.5	ENSSSCT00000029690	.	intron	.	rs791770753	3
AGGC.GCCT	11	86485271	86485289	4.75	.	.	intergenic	-0.053	rs788141475	2
ACCCT.AGGGT	11	86616589	86616607	3.16667	ENSSSCT00000010486	GAS6	promoter	-0.024	428578 rs7913	2
AC.GT	11	86734704	86734719	8	ENSSSCT00000010488	RASA3	intron	-0.351	.	2
AAAC.GTTT	11	87143341	87143355	3.75	.	.	intergenic	.	362958 rs7915	3
AAAC.GTTT	12	379850	379866	4.25	ENSSSCT00000018654	TBCD	intron	.	rs787646222	2
AC.GT	12	777100	777111	6	.	.	intergenic	.	.	2
AC.GT	12	1234609	1234636	14	ENSSSCT00000027597	.	intron	-0.446	758095 rs7885	2
AC.GT	12	1234609	1234636	14	ENSSSCT00000023942	.	intron	-0.446	758095 rs7885	2
AAAT.ATTT	12	1490750	1490764	3.75	ENSSSCT00000023391	AATK	intron	-0.084	210732 rs7882	2
AAAT.ATTT	12	1490750	1490764	3.75	ENSSSCT00000018660	AATK	intron	-0.084	210732 rs7882	2
AT.AT	12	2494898	2494913	8	ENSSSCT00000018683	TBC1D16	intron	-0.026	.	4
AG.CT	12	2606583	2606592	5	.	.	intergenic	-0.073	rs789782727	2
AC.GT	12	3694988	3694999	6	.	.	intergenic	0.088	.	4
AG.CT	12	3770214	3770224	5.5	.	.	intergenic	-0.252	.	2
AAAG.CTTT	12	4614506	4614525	5	.	.	intergenic	.	rs788589848	2
AC.GT	12	4627442	4627453	6	ENSSSCT00000022358	MFSD11	3'utr	0.046	.	3
AAAAC.GTTTT	12	4768423	4768444	4.4	.	.	intergenic	.	rs791456632	2
AC.GT	12	4770636	4770645	5	.	.	intergenic	.	rs791608894	2
AAAG.CTTT	12	5070979	5070992	3.5	ENSSSCT00000018709	PRPSAP1	intron	.	rs788340760	2
AG.CT	12	5134357	5134367	5.5	ENSSSCT00000018711	RNF157	intron	-0.123	.	4
AAAT.ATTT	12	5409776	5409798	5.75	.	.	intergenic	.	rs790076277	2
AC.GT	12	5501731	5501745	7.5	.	.	intergenic	.	397295 rs7927	2
AAAG.CTTT	12	5552484	5552506	5.75	ENSSSCT00000018726	UNK	intron	-0.09	rs791163156	2
AAAC.GTTT	12	5676979	5676994	4	ENSSSCT00000018730	SAP30BP	intron	0.625	rs790962948	2
AAAC.GTTT	12	5676979	5676994	4	ENSSSCT00000023832	SAP30BP	intron	0.625	rs790962948	2
AAAC.GTTT	12	5676979	5676994	4	ENSSSCT00000025315	RECQL5	promoter	0.625	rs790962948	2
AC.GT	12	6241924	6241936	6.5	ENSSSCT00000018742	KCTD2	intron	-0.235	rs788603062	2
AAC.GTT	12	7032192	7032206	5	.	.	intergenic	.	463279 rs7899	2
AG.CT	12	7787417	7787437	10.5	ENSSSCT00000018773	SDK2	intron	-0.265	341615 rs7903	2
AAAT.ATTT	12	7984803	7984821	4.75	.	.	intergenic	.	330541 rs7921	2
AAC.GTT	12	8385690	8385712	7.66667	ENSSSCT00000018779	SLC39A11	intron	.	722006 rs7938	2
ACAG.CTGT	12	8393053	8393071	4.75	ENSSSCT00000018779	SLC39A11	intron	-0.224	245741 rs7887	2
AATG.CATT	12	8393725	8393740	4	ENSSSCT00000018779	SLC39A11	intron	0.196	.	2
AAAC.GTTT	12	8482615	8482634	5	.	.	intergenic	.	763786 rs7864	2
AG.CT	12	8814490	8814516	13.5	.	.	intergenic	-0.092	rs788320892	2
AAG.CTT	12	8977494	8977513	6.66667	.	.	intergenic	-0.075	rs786527230	2
AAAAG.CTTTT	12	9087997	9088035	7.8	.	.	intergenic	-0.05	392071 rs7903	2
AC.GT	12	9229715	9229729	7.5	.	.	intergenic	0.519	rs789675738	4
AAAAC.GTTTT	12	9436628	9436645	3.6	.	.	intergenic	-0.181	185345 rs7902	2
AG.CT	12	9542873	9542882	5	.	.	intergenic	-0.143	.	3
AAAC.GTTT	12	9774590	9774605	4	.	.	intergenic	0.11	215024 rs7922	2
AAAAC.GTTTT	12	9827241	9827260	3.33333	.	.	intergenic	-0.022	700848 rs7928	2
AC.GT	12	10204451	10204462	6	.	.	intergenic	-0.167	rs791002237	2
AC.GT	12	10244368	10244386	9.5	.	.	intergenic	0.073	.	2
AGG.CCT	12	10369330	10369359	10	.	.	intergenic	-0.325	.	2
AC.GT	12	10391354	10391364	5.5	.	.	intergenic	-0.206	.	3
AAAT.ATTT	12	10443746	10443764	4.75	.	.	intergenic	0.416	738438 rs7906	2
AAAC.GTTT	12	10482611	10482638	7	.	.	intergenic	-0.08	rs786331727	2
AC.GT	12	10730599	10730609	5.5	.	.	intergenic	-0.106	.	3
AAC.GTT	12	10754794	10754817	8	.	.	intergenic	-0.067	rs792444481	2
AG.CT	12	10816149	10816161	6.5	.	.	intergenic	0.055	.	2
AG.CT	12	10910990	10911004	7.5	.	.	intergenic	0.208	.	4
AAAC.GTTT	12	10936176	10936194	4.75	.	.	intergenic	0.304	rs787097161	2
AAAG.CTTT	12	10957759	10957771	3.25	.	.	intergenic	-0.146	377431 rs7895	2
AG.CT	12	10969663	10969674	6	.	.	intergenic	0.05	.	3
AAAT.ATTT	12	11116567	11116584	4.5	.	.	intergenic	-0.136	rs789572496	2
AC.GT	12	11149616	11149632	8.5	ENSSSCT00000035633	MKK6	intron	0.242	rs787496064	2
AC.GT	12	11193889	11193901	6.5	ENSSSCT00000034463	MKK6	intron	.	rs789825134	2
AC.GT	12	11193889	11193901	6.5	ENSSSCT00000018783	MKK6	intron	.	rs789825134	2
AAAT.ATTT	12	11204602	11204617	4	ENSSSCT00000034463	MKK6	intron	0.206	rs791367607	2
AAAT.ATTT	12	11204602	11204617	4	ENSSSCT00000018783	MKK6	intron	0.206	rs791367607	2
AC.GT	12	11246306	11246320	7.5	ENSSSCT00000018783	MKK6	intron	0.233	983186 rs7893	2
AAC.GTT	12	11268388	11268405	6	.	.	intergenic	0.141	365557 rs7925	2
AAGG.CCTT	12	11372101	11372122	5.5	.	.	intergenic	0.1	321510 rs7865	2
AAAC.GTTT	12	11654947	11654963	4.25	.	.	intergenic	0.099	rs792430399	2
AG.CT	12	11904317	11904333	8.5	.	.	intergenic	-0.156	129982 rs7917	2
AAAAC.GTTTT	12	11935854	11935868	3	.	.	intergenic	.	rs792207455	2
AT.AT	12	12019960	12019969	5	.	.	intergenic	.	rs791888597	2
AAAC.GTTT	12	12109421	12109439	4.75	ENSSSCT00000018787	FAM20A	promoter	-0.046	rs788746754	2
AAAC.GTTT	12	12187916	12187932	4.25	ENSSSCT00000018789	WIPI1	intron	0.125	rs792690484	2

AAAC.GTTT	12	12415914	12415928	3.75	ENSSSCT00000018792	GNA13	intron	.	.	2
AAAT.ATTT	12	12838802	12838834	8.25	ENSSSCT00000018795	.	intron	0.2	.	2
AT.AT	12	12873323	12873334	6	ENSSSCT00000018795	.	intron	.	506061 rs7879	4
AT.AT	12	12989539	12989548	5	.	.	intergenic	.	rs787370227	2
AAAC.GTTT	12	12990761	12990787	6.75	.	.	intergenic	.	522623 rs7938	2
AC.GT	12	13042016	13042045	15	ENSSSCT00000027357	.	intron	0.344	.	2
AT.AT	12	13048999	13049009	5.5	ENSSSCT00000027357	.	intron	-0.513	946841 rs7863	2
AC.GT	12	13137379	13137391	6.5	.	.	intergenic	.	.	2
AC.GT	12	13437776	13437787	6	.	.	intergenic	-0.02	rs789466189	2
AAC.GTT	12	13526697	13526737	13.6667	ENSSSCT00000018798	PRKCA	intron	-0.089	rs792679305	2
AT.AT	12	13896725	13896736	6	ENSSSCT00000018802	HELZ	intron	.	358151 rs7904	2
AAATG.CATTT	12	14012826	14012844	3.8	ENSSSCT00000018802	HELZ	intron	.	rs786988064	2
AAAAAC.GTTTT	12	14112774	14112795	3.66667	ENSSSCT00000018804	PITPNC1	intron	0.071	.	2
AT.AT	12	14165378	14165387	5	ENSSSCT00000018804	PITPNC1	intron	0.276	.	4
AAAT.ATTT	12	14184560	14184578	4.75	ENSSSCT00000018804	PITPNC1	intron	0.036	rs789941461	2
AAAG.CTTT	12	14228023	14228046	6	ENSSSCT00000018804	PITPNC1	intron	.	795966 rs7896	3
AAAC.GTTT	12	14262647	14262671	6.25	ENSSSCT00000018804	PITPNC1	intron	-0.113	rs786336558	2
AAAC.GTTT	12	14356137	14356153	4.25	ENSSSCT00000018804	PITPNC1	intron	.	rs790438582	3
AAAC.GTTT	12	14464199	14464211	3.25	.	.	intergenic	.	952708 rs7896	2
AAAAC.GTTTT	12	14511937	14511961	5	.	.	intergenic	0.16	525604 rs7922	3
AT.AT	12	14593979	14593992	7	.	.	intergenic	.	rs793837668	2
AC.GT	12	14837796	14837806	5.5	ENSSSCT00000018810	TEX2	intron	0.039	.	2
AAAAC.GTTTT	12	15353089	15353111	4.6	ENSSSCT00000018827	KCNH6	intron	-0.19	rs793853612	2
AAAAC.GTTTT	12	15353089	15353111	4.6	ENSSSCT00000029983	KCNH6	intron	-0.19	rs793853612	2
AAAAAC.GTTTT	12	15467426	15467453	4.66667	ENSSSCT00000018832	TANC2	intron	2.397	rs790996112	2
AGGAG.CTCCT	12	15475025	15475045	3.5	ENSSSCT00000018832	TANC2	intron	.	.	3
AAAAAG.CTTTT	12	15492197	15492221	4.16667	ENSSSCT00000018832	TANC2	intron	0.257	936077 rs7879	2
AG.CT	12	15583684	15583698	7.5	ENSSSCT00000018832	TANC2	intron	0.096	.	2
AAAG.CTTT	12	15714029	15714055	6.75	.	.	intergenic	.	rs787008094	2
AAAT.ATTT	12	15890017	15890045	7.25	.	.	intergenic	0.066	rs789286792	2
AAAAC.GTTTT	12	16066096	16066124	5.8	ENSSSCT00000018837	TLK2	intron	-0.464	rs787564705	2
AAAC.GTTT	12	16160606	16160621	4	ENSSSCT00000018840	EFCAB3	intron	0.881	rs786826650	2
AC.GT	12	16221175	16221187	6.5	.	.	intergenic	.	rs789299486	3
AG.CT	12	16370338	16370348	5.5	.	.	intergenic	.	rs787371421	2
AC.GT	12	16435610	16435621	6	.	.	intergenic	.	rs792081110	3
AAAAC.GTTTT	12	16472177	16472205	5.8	.	.	intergenic	.	rs789052850	2
AAAG.CTTT	12	16703915	16703938	6	ENSSSCT00000027689	EFCAB13	promoter	0.528	rs790237671	2
AAAG.CTTT	12	16703915	16703938	6	ENSSSCT00000018841	EFCAB13	promoter	0.528	rs790237671	2
AAC.GTT	12	16714611	16714631	7	.	.	intergenic	0.049	rs786740483	2
AAAC.GTTT	12	16780068	16780086	4.75	.	.	intergenic	0.154	785182 rs7892	2
AAAG.CTTT	12	16791109	16791123	3.75	.	.	intergenic	0.652	rs791701690	2
AAC.GTT	12	16852963	16852976	4.66667	ENSSSCT00000018844	CDC27	intron	0.281	rs789868521	2
AAC.GTT	12	17098964	17098981	6	ENSSSCT00000018846	KANSL1	intron	0.076	rs792698204	2
AC.GT	12	17275518	17275529	6	.	.	intergenic	.	345493 rs7862	2
AT.AT	12	17487094	17487105	6	.	.	intergenic	.	766269 rs7937	2
AAAAC.GTTTT	12	17520958	17520980	4.6	.	.	intergenic	.	.	2
AAAC.GTTT	12	18182019	18182031	3.25	ENSSSCT00000018861	PLEKHM1	intron	0.143	rs788009974	2
AC.GT	12	18477473	18477487	7.5	.	.	intergenic	0.218	rs791298802	2
AG.CT	12	18735329	18735339	5.5	ENSSSCT00000018882	EFTUD2	intron	1.442	rs790573875	2
AAC.GTT	12	18991927	18991949	7.66667	.	.	intergenic	.	rs787891582	2
AC.GT	12	19492272	19492284	6.5	.	.	intergenic	-0.11	rs788628203	3
AAAT.ATTT	12	19536391	19536407	4.25	ENSSSCT00000018911	MPP3	intron	-0.565	rs793050085	2
CACTC.GAGTG	12	19581046	19581066	3.5	ENSSSCT00000024088	DUSP3	3'utr	0.004	rs792613631	2
AAAC.GTTT	12	19995727	19995749	5.75	.	.	intergenic	.	rs792697774	2
AAAT.ATTT	12	20074109	20074126	4.5	ENSSSCT00000025436	PIGBRCA1	intron	0.282	rs788880834	2
AAAG.CTTT	12	20163618	20163662	11.25	.	.	intergenic	.	rs790885245	2
AG.CT	12	20283177	20283186	5	ENSSSCT00000018927	WNK4	intron	1.741	.	2
AG.CT	12	20516644	20516653	5	ENSSSCT00000018941	.	intron	-0.011	rs787196663	2
AC.GT	12	21303649	21303663	7.5	.	.	intergenic	-0.731	.	2
AAAC.GTTT	12	21721872	21721893	5.5	.	.	intergenic	.	rs696776831	2
AC.GT	12	21964569	21964579	5.5	.	.	intergenic	.	rs788827895	2
AG.CT	12	21977051	21977068	9	.	.	intergenic	0.149	rs786324393	2
AAAT.ATTT	12	22041665	22041696	8	.	.	intergenic	-0.092	rs791118502	2
AC.GT	12	22137031	22137041	5.5	.	.	intergenic	0.223	rs786777208	2
AC.GT	12	22231850	22231868	9.5	.	.	intergenic	0	rs791826496	4
AT.AT	12	22305306	22305318	6.5	.	.	intergenic	-0.021	rs788671773	2
AC.GT	12	22402562	22402579	9	.	.	intergenic	0.335	590455 rs7877	2
AAAG.CTTT	12	22536644	22536676	8.25	.	.	intergenic	0.114	rs787144341	2
AAAG.CTTT	12	22699744	22699763	5	.	.	intergenic	0.084	rs791737909	2
AG.CT	12	22825786	22825798	6.5	ENSSSCT00000019043	.	intron	0.809	.	4
AAAAT.ATTTT	12	22828490	22828509	4	ENSSSCT00000019043	.	intron	0.768	rs788203953	2
AC.GT	12	22865212	22865226	7.5	.	.	intergenic	0.34	rs788052736	4
AAAT.ATTT	12	22914198	22914215	4.5	ENSSSCT00000019046	ZPBP2	intron	.	.	2
AAAT.ATTT	12	22914198	22914215	4.5	ENSSSCT00000025164	ZPBP2	intron	.	.	2
AT.AT	12	22950810	22950828	9.5	ENSSSCT00000019047	IKZF3	intron	.	.	2
AC.GT	12	23133618	23133637	10	.	.	intergenic	0.175	rs789224789	4
AAC.GTT	12	23194643	23194658	5.33333	.	.	intergenic	0.2	rs790545834	2
AAC.GTT	12	23258603	23258616	4.66667	ENSSSCT00000019057	CDK12	intron	2.584	rs790879186	2
AAAT.ATTT	12	23385555	23385576	5.5	ENSSSCT00000019059	FBXL20	intron	0.025	rs791007275	2
AC.GT	12	24267147	24267170	12	.	.	intergenic	0.151	254262 rs7889	2
AC.GT	12	24640470	24640486	8.5	.	.	intergenic	0.198	rs790325760	2
AAAT.ATTT	12	24656615	24656637	5.75	.	.	intergenic	0.386	rs793742545	2
AG.CT	12	24709947	24709956	5	.	.	intergenic	0.341	rs789729137	2
AAC.GTT	12	24932985	24933000	5.33333	.	.	intergenic	-0.141	rs789750965	2

AC.GT	12	25144760	25144781	11	.	.	intergenic	.	989849 rs7884	2
AC.GT	12	25186685	25186698	7	ENSSSCT00000022825	.	intron	.	.	4
AC.GT	12	25796529	25796539	5.5	.	.	intergenic	0.511	748609 rs7929	2
AAAAC.GTTTT	12	25831965	25831986	4.4	ENSSSCT00000030769	.	intron	0.233	rs786842842	2
AAAT.ATTT	12	25967341	25967358	4.5	.	.	intergenic	0.296	rs790825254	2
AT.AT	12	25984950	25984965	8	ENSSSCT00000019107	KAT7	intron	0.333	rs789977908	2
AGG.CCT	12	26067776	26067790	5	.	.	intergenic	0.012	rs791341717	2
AC.GT	12	27185680	27185693	7	.	.	intergenic	.	357943 rs7895	2
AAAAT.ATTTT	12	27194261	27194281	4.2	.	.	intergenic	-0.154	rs788710853	2
AC.GT	12	27527051	27527060	5	ENSSSCT00000019153	MBTD1	intron	0.08	036363 rs7884	2
AG.CT	12	27860823	27860848	13	.	.	intergenic	.	rs790064443	3
AT.AT	12	28230612	28230626	7.5	.	.	intergenic	.	559157 rs7871	2
AAATG.CATTT	12	28258058	28258101	8.8	.	.	intergenic	.	519409 rs7924	2
AAC.GTT	12	28460147	28460160	4.66667	.	.	intergenic	-0.082	153982 rs7910	2
AC.GT	12	28466762	28466775	7	.	.	intergenic	0.839	rs790559473	2
AG.CT	12	28493260	28493302	21.5	.	.	intergenic	-0.163	.	2
AAAAAC.GTTTTT	12	28506123	28506144	3.66667	.	.	intergenic	0.005	rs788485916	2
AT.AT	12	28537583	28537592	5	.	.	intergenic	0.041	.	2
AG.CT	12	28631466	28631479	7	.	.	intergenic	-0.117	rs789654058	4
AT.AT	12	28638193	28638203	5.5	.	.	intergenic	0.007	rs793270913	2
AG.CT	12	28693427	28693436	5	.	.	intergenic	.	.	4
AAAC.GTTT	12	29249064	29249093	7.5	.	.	intergenic	0.018	597706 rs7871	3
AAAC.GTTT	12	29250638	29250655	4.5	.	.	intergenic	0.146	rs787725711	2
AAG.CTT	12	29608147	29608163	5.66667	.	.	intergenic	.	rs701620606	2
AG.CT	12	29684077	29684089	6.5	.	.	intergenic	.	rs789207399	2
AAC.GTT	12	30203681	30203695	5	.	.	intergenic	.	.	2
AC.GT	12	30765123	30765142	10	.	.	intergenic	-0.171	.	4
AAC.GTT	12	30872794	30872816	7.66667	.	.	intergenic	-0.321	329748 rs7923	2
AT.AT	12	31140567	31140576	5	.	.	intergenic	.	rs789612612	3
AATG.CATT	12	31228906	31228927	5.5	.	.	intergenic	-0.038	rs791181123	2
AC.GT	12	31591223	31591240	9	.	.	intergenic	-0.035	.	3
AT.AT	12	31696961	31696978	9	ENSSSCT00000019162	TOM1L1	intron	0.908	rs792154637	2
AT.AT	12	31696961	31696978	9	ENSSSCT00000031451	TOM1L1	intron	0.908	rs792154637	2
AT.AT	12	31704792	31704812	10.5	ENSSSCT00000019162	TOM1L1	intron	0.069	.	4
AT.AT	12	31704792	31704812	10.5	ENSSSCT00000031451	TOM1L1	intron	0.069	.	4
AAG.CTT	12	31713654	31713667	4.66667	ENSSSCT00000019162	TOM1L1	intron	.	rs789690233	2
AAG.CTT	12	31713654	31713667	4.66667	ENSSSCT00000031451	TOM1L1	intron	.	rs789690233	2
AAAC.GTTT	12	32180681	32180697	4.25	.	.	intergenic	0.489	rs788878701	2
AAAC.GTTT	12	32322834	32322853	5	.	.	intergenic	-0.158	rs791492486	2
AC.GT	12	32514008	32514033	13	.	.	intergenic	0.069	rs792459905	2
AAAAC.GTTTT	12	32598156	32598180	5	.	.	intergenic	.	rs701326797	2
AAG.CTT	12	32618212	32618232	7	.	.	intergenic	0.053	rs712737905	2
AAAAAT.ATTTTT	12	32693149	32693166	3	.	.	intergenic	.	rs786246456	2
AATG.CATT	12	33227193	33227210	4.5	.	.	intergenic	0.07	rs706132842	2
AAT.ATT	12	33295024	33295039	5.33333	.	.	intergenic	0.065	rs791707497	4
AAG.CTT	12	33499875	33499887	4.33333	ENSSSCT00000019171	ANKFN1	intron	.	rs786550046	2
AC.GT	12	33945689	33945703	7.5	ENSSSCT00000034924	C17orf67	intron	-0.227	549169 rs7936	3
AC.GT	12	34001817	34001827	5.5	ENSSSCT00000019179	SCPEP1	intron	0.783	413477 rs7871	3
AAAT.ATTT	12	34112515	34112535	5.25	ENSSSCT00000019177	DGKE	intron	-0.153	rs790167855	2
AGG.CCT	12	34703358	34703372	5	.	.	intergenic	0.251	rs790467068	2
AAAC.GTTT	12	35029108	35029122	3.75	.	.	intergenic	0.47	rs787488879	2
AC.GT	12	35386959	35386972	7	ENSSSCT00000027876	DYNLL2	intron	0.214	.	2
AAGC.GCTT	12	35840318	35840330	3.25	.	.	intergenic	.	rs791503096	2
AT.AT	12	35881938	35881949	6	ENSSSCT00000019208	TEX14	intron	0.273	rs790322415	2
AT.AT	12	35881938	35881949	6	ENSSSCT00000019209	TEX14	intron	0.273	rs790322415	2
AAAC.GTTT	12	36028941	36028959	4.75	ENSSSCT00000035676	4-Sep	promoter	0.403	rs793729884	2
AAAC.GTTT	12	36028941	36028959	4.75	ENSSSCT00000035025	4-Sep	promoter	0.403	rs793729884	2
AAAC.GTTT	12	36545469	36545495	6.75	ENSSSCT00000033129	TRIM37	intron	0.319	rs786418078	2
AAAC.GTTT	12	36545469	36545495	6.75	ENSSSCT00000024586	TRIM37	intron	0.319	rs786418078	2
AAAC.GTTT	12	36684039	36684059	5.25	.	.	intergenic	.	rs793141766	2
AAAT.ATTT	12	36687870	36687884	3.75	.	.	intergenic	0.328	.	4
AAAC.GTTT	12	36725812	36725827	4	.	.	intergenic	0.147	561001 rs7883	2
AAAAC.GTTTT	12	36742404	36742432	5.8	.	.	intergenic	-0.038	rs789014010	2
AAGC.GCTT	12	36934513	36934533	5.25	ENSSSCT00000027408	GDPD1	intron	.	304215 rs7921	2
AAAT.ATTT	12	36938172	36938190	4.75	ENSSSCT00000027408	GDPD1	intron	.	rs792667601	2
AAC.GTT	12	37055851	37055864	4.66667	ENSSSCT00000026532	PRR11	intron	0.161	511715 rs7894	2
AAATT.AATTT	12	37164709	37164732	4.8	.	.	intergenic	.	rs792732175	2
AAAAG.CTTTT	12	37316378	37316405	5.6	ENSSSCT00000019232	ssc-mir-21	intron	0.279	rs786524572	2
AT.AT	12	37331185	37331206	11	ENSSSCT00000019232	ssc-mir-21	intron	.	763270 rs7908	2
AAAAG.CTTTT	12	37361871	37361893	4.6	ENSSSCT00000019233	TUBD1	intron	.	rs790157563	2
AAC.GTT	12	37592850	37592864	5	.	.	intergenic	.	764395 rs7912	2
AT.AT	12	37707120	37707129	5	ENSSSCT00000019236	MED13	intron	.	070560 rs7907	2
AT.AT	12	37707120	37707129	5	ENSSSCT00000024721	MED13	intron	.	070560 rs7907	2
AT.AT	12	37742198	37742207	5	ENSSSCT00000019238	.	intron	2.268	rs791722157	2
AAAC.GTTT	12	37996370	37996390	5.25	.	.	intergenic	.	964869 rs7930	2
AG.CT	12	38451809	38451823	7.5	.	.	intergenic	.	rs793578196	2
AAT.ATT	12	38487306	38487336	10.3333	.	.	intergenic	0.263	583077 rs7862	2
AAAAC.GTTTT	12	38538113	38538144	6.4	.	.	intergenic	0.226	rs787509228	2
AAC.GTT	12	38539045	38539058	4.66667	.	.	intergenic	0.105	.	2
AG.CT	12	38559044	38559053	5	.	.	intergenic	1.567	rs791757850	2
AC.GT	12	38575375	38575393	9.5	.	.	intergenic	-0.092	rs792209293	3
AAC.GTT	12	38582480	38582501	7.33333	.	.	intergenic	1.471	rs793134338	2
AAAT.ATTT	12	38653005	38653032	7	ENSSSCT00000019240	BCAS3	intron	-0.065	rs789984582	4
AT.AT	12	38677341	38677350	5	ENSSSCT00000019240	BCAS3	intron	0.146	rs792459785	2

AAAC.GTTT	12	38741107	38741133	6.75	ENSSSCT00000019240	BCAS3	intron	-0.102	327388 rs7909	2
ACAT.ATGT	12	38752023	38752053	7.75	ENSSSCT00000019240	BCAS3	intron	0.161	117082 rs7870	3
AAAC.GTTT	12	38785982	38786003	5.5	ENSSSCT00000019240	BCAS3	intron	.	rs788279875	2
AT.AT	12	38859892	38859907	8	ENSSSCT00000019240	BCAS3	intron	0.184	rs790975632	2
AAAG.CTTT	12	39051837	39051857	5.25	.	.	intergenic	.	rs789610886	2
AGG.CCT	12	39453175	39453205	10.3333	.	.	intergenic	.	.	2
AAAT.ATTT	12	39590462	39590476	3.75	ENSSSCT00000032517	ZNHIT3	promoter	-0.017	.	2
AAAAC.GTTTT	12	39744686	39744700	3	ENSSSCT00000019255	MRM1	intron	0.224	709855 rs7899	2
AC.GT	12	39799779	39799789	5.5	.	.	intergenic	1.539	rs793104148	4
AATG.CATT	12	39836954	39836974	5.25	.	.	intergenic	0.464	rs790317292	4
AC.GT	12	39843919	39843944	13	.	.	intergenic	1.644	rs791084697	2
AGGC.GCCT	12	39923448	39923464	4.25	.	.	intergenic	-0.023	rs791517462	2
AAAG.CTTT	12	39940428	39940441	3.5	.	.	intergenic	.	rs788954969	2
AAAC.GTTT	12	40149335	40149358	6	ENSSSCT00000019257	AATF	intron	1.132	rs786900806	2
AAT.ATT	12	40176921	40176941	7	ENSSSCT00000019257	AATF	intron	0.174	.	2
AAAC.GTTT	12	40236718	40236732	3.75	ENSSSCT00000019259	ACACA	intron	1.698	302047 rs7914	2
AC.GT	12	40239266	40239277	6	ENSSSCT00000019259	ACACA	intron	0.272	rs790869966	2
AG.CT	12	40287688	40287698	5.5	ENSSSCT00000019259	ACACA	intron	0.642	rs790746766	2
AAAC.GTTT	12	40381313	40381329	4.25	.	.	intergenic	.	746257 rs7896	2
AC.GT	12	40390180	40390192	6.5	.	.	intergenic	0.159	rs704531194	2
AG.CT	12	40610670	40610679	5	ENSSSCT00000029194	DUSP14	intron	.	.	3
AAAT.ATTT	12	40624183	40624204	5.5	ENSSSCT00000028499	SYNRG	intron	.	.	3
AC.GT	12	40882274	40882288	7.5	.	.	intergenic	0.207	328102 rs7910	2
AGG.CCT	12	41052856	41052868	4.33333	ENSSSCT00000019265	.	promoter	-0.202	rs792544423	2
AG.CT	12	41124840	41124852	6.5	.	.	intergenic	.	rs791703721	2
AAAAG.CTTTT	12	41203480	41203502	3.83333	.	.	intergenic	.	rs791339384	2
AAAG.CTTT	12	41249894	41249909	4	ENSSSCT00000019272	C17orf66	intron	0.114	rs788461490	3
AAAAC.GTTTT	12	41288610	41288628	3.8	.	.	intergenic	.	rs792372491	2
AAAC.GTTT	12	41389243	41389274	8	ENSSSCT00000019278	.	intron	0.265	rs792957057	2
AAC.GTT	12	41393894	41393908	5	ENSSSCT00000019278	.	intron	0.383	036759 rs7926	2
AAAC.GTTT	12	41398945	41398957	3.25	ENSSSCT00000019278	.	intron	0.393	rs787885580	2
AAAAC.GTTTT	12	41611702	41611719	3.6	.	.	intergenic	0.347	rs786542148	2
AC.GT	12	41783363	41783377	7.5	ENSSSCT00000027932	.	intron	.	.	2
AAAT.ATTT	12	41867355	41867377	5.75	.	.	intergenic	0.541	371467 rs7889	2
ATC.GAT	12	42198817	42198828	4	.	.	intergenic	-0.127	.	2
AC.GT	12	42217457	42217477	10.5	.	.	intergenic	0.409	rs698586585	2
AAAC.GTTT	12	42421066	42421083	4.5	.	.	intergenic	-0.249	rs786992900	2
AG.CT	12	42671223	42671232	5	.	.	intergenic	-0.132	275417 rs7920	2
AAAC.GTTT	12	42890016	42890042	6.75	.	.	intergenic	0.169	rs791350412	2
AT.AT	12	43011533	43011544	6	.	.	intergenic	.	rs792905070	2
AC.GT	12	43078544	43078567	12	.	.	intergenic	-0.158	215089 rs7876	2
AAAC.GTTT	12	43094947	43094963	4.25	.	.	intergenic	0.41	rs788078807	2
AG.CT	12	43402482	43402500	9.5	.	.	intergenic	0.112	rs791162974	2
AC.GT	12	43464270	43464309	20	.	.	intergenic	0.174	rs793463094	4
AAAC.GTTT	12	43983327	43983339	3.25	ENSSSCT00000019295	MYO1D	intron	-0.082	rs789967368	2
AT.AT	12	44127207	44127217	5.5	ENSSSCT00000019295	MYO1D	intron	0.183	rs698222345	5
AC.GT	12	44138270	44138298	14.5	.	.	intergenic	.	.	4
AG.CT	12	44327467	44327477	5.5	.	.	intergenic	.	rs791026089	2
AG.CT	12	44486173	44486185	6.5	ENSSSCT00000019302	RHOT1	intron	0.384	rs793276948	2
AT.AT	12	44883637	44883652	8	ENSSSCT00000019314	COPRS	intron	0.142	rs788017689	2
AATC.GATT	12	44976045	44976065	5.25	.	.	intergenic	0.204	907136 rs7927	2
AAAC.GTTT	12	44989176	44989193	4.5	.	.	intergenic	-0.132	rs792093192	2
AGG.CCT	12	45011709	45011746	12.6667	.	.	intergenic	.	rs696607077	2
AC.GT	12	45052019	45052029	5.5	.	.	intergenic	0.101	rs786393542	2
AAAAC.GTTTT	12	45062633	45062649	3.4	.	.	intergenic	0.002	rs792811989	2
AAAC.GTTT	12	45110917	45110933	4.25	.	.	intergenic	.	411335 rs7904	4
AAAC.GTTT	12	45121283	45121314	8	.	.	intergenic	0.007	rs787259524	2
AGGGC.GCCCT	12	45150048	45150063	3.2	.	.	intergenic	-0.121	321165 rs7895	2
AACC.GGTT	12	45294222	45294241	5	ENSSSCT00000019316	RAB11FIP4	intron	-0.269	rs793293142	2
AATG.CATT	12	45377650	45377672	5.75	ENSSSCT00000023079	NF1	intron	0.08	rs787406297	3
AATG.CATT	12	45377650	45377672	5.75	ENSSSCT00000019317	NF1	intron	0.08	rs787406297	3
AT.AT	12	45473082	45473093	6	ENSSSCT00000023079	NF1	intron	0.162	.	3
AT.AT	12	45473082	45473093	6	ENSSSCT00000019317	NF1	intron	0.162	.	3
AAAC.GTTT	12	45480355	45480391	9.25	ENSSSCT00000023079	NF1	intron	.	rs791397445	2
AAAC.GTTT	12	45480355	45480391	9.25	ENSSSCT00000019317	NF1	intron	.	rs791397445	2
AAAT.ATTT	12	45546826	45546839	3.5	.	.	intergenic	.	988342 rs7925	2
AAAT.ATTT	12	45607586	45607601	4	.	.	intergenic	.	rs786458511	2
AAAAAC.GTTTTT	12	46172195	46172214	3.33333	.	.	intergenic	0.667	535853 rs7881	2
AAAG.CTTT	12	46247574	46247599	6.5	.	.	intergenic	0.227	rs788360378	2
AC.GT	12	46287149	46287171	11.5	ENSSSCT00000030105	NLK	intron	0.309	497998 rs7926	4
AT.AT	12	46294405	46294415	5.5	ENSSSCT00000030105	NLK	intron	0.33	.	2
AC.GT	12	46401794	46401803	5	ENSSSCT00000030105	NLK	intron	.	rs790209380	2
AAAT.ATTT	12	46831894	46831906	3.25	ENSSSCT00000019329	SPAG5	intron	.	073846 rs7876	2
AAAAAC.GTTTTT	12	47121489	47121522	5.66667	ENSSSCT00000019351	PHF12	intron	0.177	rs793198860	2
AAT.ATT	12	47200394	47200410	5.66667	.	.	intergenic	0.466	rs792511012	2
AAAT.ATTT	12	47458769	47458787	4.75	ENSSSCT00000019357	NUFIP2	promoter	1.779	967108 rs7925	2
AAC.GTT	12	47489387	47489400	4.66667	.	.	intergenic	0.467	.	2
AAAAC.GTTTT	12	47507605	47507635	6.2	.	.	intergenic	.	032058 rs7904	2
AAAC.GTTT	12	47620086	47620101	4	ENSSSCT00000019362	.	intron	.	rs788409646	2
AAAC.GTTT	12	47842712	47842734	5.75	ENSSSCT00000033840	SSH2	intron	1.882	rs793172005	2
AAAC.GTTT	12	47842712	47842734	5.75	ENSSSCT00000019363	SSH2	intron	1.882	rs793172005	2
AAAC.GTTT	12	47842712	47842734	5.75	ENSSSCT00000034147	SSH2	intron	1.882	rs793172005	2
AC.GT	12	48030886	48030899	7	.	.	intergenic	0.29	rs789635737	2
AG.CT	12	48303667	48303677	5.5	ENSSSCT00000019366	EFCAB5	intron	0.416	929311 rs7927	2

ACC.GGT	12	48435141	48435152	4	ENSSSCT00000019366	EFCAB5	intron	0.237	rs790684128	2
AAT.ATT	12	48675911	48675939	9.66667	ENSSSCT00000036665	GOSR1	intron	0.32	485348 rs7905	2
AAT.ATT	12	48675911	48675939	9.66667	ENSSSCT00000030283	GOSR1	intron	0.32	485348 rs7905	2
AC.GT	12	48866081	48866121	20.5	.	.	intergenic	0.123	137784 rs7891	2
AAAC.GTTT	12	49295671	49295690	5	.	.	intergenic	.	rs793286927	2
AAAT.ATTT	12	49431364	49431377	3.5	ENSSSCT00000019386	VPS53	intron	-0.122	rs792616556	2
AAAC.GTTT	12	49964320	49964337	4.5	.	.	intergenic	0.089	rs790891720	2
AC.GT	12	49988786	49988803	9	.	.	intergenic	0.251	362981 rs7905	2
AAAAC.GTTTT	12	50055570	50055591	4.4	ENSSSCT00000019401	SMG6	intron	0.198	rs791273615	2
AAAC.GTTT	12	50151382	50151396	3.75	ENSSSCT00000019401	SMG6	intron	0.25	.	2
AT.AT	12	50181340	50181352	6.5	ENSSSCT00000019401	SMG6	intron	0.228	rs789830421	2
AAAG.CTTT	12	50312479	50312503	6.25	ENSSSCT00000019407	.	intron	0.234	rs789489416	2
AC.GT	12	50380701	50380716	8	.	.	intergenic	.	rs786288043	4
AG.CT	12	50500538	50500548	5.5	ENSSSCT00000019411	PAFAH1B1	intron	1.283	.	2
AAC.GTT	12	50544675	50544691	5.66667	ENSSSCT00000019409	.	intron	.	rs787401046	2
AAC.GTT	12	50544675	50544691	5.66667	ENSSSCT00000025005	.	intron	.	rs787401046	2
AAAT.ATTT	12	50622498	50622521	6	.	.	intergenic	-0.085	420283 rs7933	2
AC.GT	12	50833317	50833333	8.5	ENSSSCT00000019412	RAP1GAP2	intron	1.372	334682 rs7901	2
AAG.CTT	12	50904772	50904783	4	.	.	intergenic	.	rs788884371	2
AC.GT	12	51061205	51061225	10.5	.	.	intergenic	.	rs712730120	3
AG.CT	12	51261121	51261136	8	.	.	intergenic	-0.088	675180 rs7896	2
AGAT.ATCT	12	51284642	51284682	10.25	ENSSSCT00000019432	OR1K1	promoter	0.161	rs710759770	2
AC.GT	12	51345393	51345404	6	.	.	intergenic	0.021	rs791672849	2
AT.AT	12	51489369	51489379	5.5	ENSSSCT00000019437	ASPA	intron	1.886	239033 rs7898	2
AC.GT	12	51495388	51495397	5	.	.	intergenic	0.612	rs790218254	2
AC.GT	12	51807567	51807580	7	ENSSSCT00000035080	ITGAE	intron	-0.085	393147 rs7896	2
AC.GT	12	51807567	51807580	7	ENSSSCT00000034272	ITGAE	intron	-0.085	393147 rs7896	2
AG.CT	12	51974585	51974599	7.5	.	.	intergenic	-0.067	.	2
AATT.AATT	12	52025159	52025178	5	.	.	intergenic	.	.	2
AC.GT	12	52255639	52255650	6	ENSSSCT00000019455	UBE2G1	intron	.	946679 rs7883	2
AAAT.ATTT	12	52298112	52298126	3.75	.	.	intergenic	-0.033	369347 rs7897	2
AC.GT	12	52432100	52432113	7	ENSSSCT00000019457	SPNS3	intron	-0.251	.	2
AAAC.GTTT	12	52533567	52533587	5.25	ENSSSCT00000019461	TEKT1	intron	.	rs788102791	2
AAG.CTT	12	52917085	52917099	5	.	.	intergenic	-0.083	rs788256184	2
AAG.CTT	12	53316721	53316738	6	.	.	intergenic	.	rs791135501	2
AAAG.CTTT	12	53432543	53432557	3.75	.	.	intergenic	.	rs791605492	2
AAC.GTT	12	53540751	53540767	5.66667	.	.	intergenic	0.338	rs789979757	2
AAG.CTT	12	53747620	53747632	4.33333	.	.	intergenic	.	rs789051959	2
AAT.ATT	12	53946738	53946755	6	.	.	intergenic	.	rs788622937	2
AC.GT	12	54066487	54066501	7.5	.	.	intergenic	-0.029	469178 rs7884	2
AAAAT.ATTTT	12	54642280	54642296	3.4	.	.	intergenic	0.006	rs790632275	3
AAAAT.ATTTT	12	55927165	55927180	3.2	ENSSSCT00000027166	CTC1	promoter	0.211	rs789506559	2
AAAAC.GTTTT	12	56530287	56530305	3.8	.	.	intergenic	0.383	rs791171316	2
AAC.GTT	12	56541639	56541651	4.33333	.	.	intergenic	-0.105	rs792689631	2
AAAAC.GTTTT	12	56693943	56693962	4	ENSSSCT00000019577	PIK3R6	intron	0.115	141153 rs7893	2
AG.CT	12	57044577	57044596	10	.	.	intergenic	-0.049	609108 rs7913	2
AC.GT	12	57247691	57247708	9	.	.	intergenic	0.536	155983 rs7882	2
AGGG.CCCT	12	57251861	57251874	3.5	.	.	intergenic	0.124	rs789857361	2
AT.AT	12	57358858	57358876	9.5	ENSSSCT00000019584	USP43	intron	-0.123	393808 rs7872	3
AATG.CATT	12	57572655	57572683	7.25	.	.	intergenic	-0.233	rs786740873	2
AAAT.ATTT	12	57636117	57636130	3.5	.	.	intergenic	-0.237	rs791938622	3
AAAAC.GTTTT	12	57936488	57936507	4	.	.	intergenic	-0.081	.	2
AAC.GTT	12	58025266	58025284	6.33333	.	.	intergenic	.	rs792865829	2
AC.GT	12	58622338	58622350	6.5	ENSSSCT00000031516	.	intron	-0.165	rs790830628	2
AT.AT	12	58651517	58651527	5.5	ENSSSCT00000031516	.	intron	0.234	.	2
AC.GT	12	58922908	58922922	7.5	.	.	intergenic	-0.307	rs789141193	4
AAATT.AATTT	12	59135178	59135192	3	.	.	intergenic	0.161	rs792503636	2
AAAC.GTTT	12	59141572	59141590	4.75	.	.	intergenic	.	235694 rs7904	2
AAC.GTT	12	59154545	59154567	7.66667	.	.	intergenic	1.098	615865 rs7874	3
AAC.GTT	12	59243575	59243592	6	ENSSSCT00000019610	.	intron	0.216	902789 rs7884	2
AAC.GTT	12	59363735	59363746	4	ENSSSCT00000019611	MAP2K4	intron	0.265	rs793334950	2
AAAC.GTTT	12	59598949	59598979	7.75	.	.	intergenic	0.081	581924 rs7908	2
AC.GT	12	59648689	59648699	5.5	.	.	intergenic	.	617766 rs7890	2
AG.CT	12	60441140	60441153	7	.	.	intergenic	0.027	rs792830425	2
AC.GT	12	60473048	60473068	10.5	.	.	intergenic	-0.159	rs791730749	2
AT.AT	12	60535724	60535734	5.5	.	.	intergenic	0.093	rs789907191	2
AAACC.GGTTT	12	60804993	60805014	4.4	.	.	intergenic	-0.095	rs787746681	2
AC.GT	12	60824865	60824888	12	.	.	intergenic	-0.025	907293 rs7903	2
AAC.GTT	12	61567027	61567051	8.33333	.	.	intergenic	0.046	.	2
ACC.GGT	12	61617124	61617140	5.66667	.	.	intergenic	-0.244	rs788355862	2
AAGG.CCTT	12	61764919	61764937	4.75	.	.	intergenic	-0.058	626002 rs7897	2
AAAG.CTTT	12	61993853	61993867	3.75	.	.	intergenic	.	rs792563320	2
AAAAG.CTTTT	12	62134942	62134963	4.4	.	.	intergenic	.	rs788670008	2
AC.GT	12	62457880	62457897	9	ENSSSCT00000019634	NCOR1	intron	-0.082	.	2
AC.GT	12	62532845	62532873	14.5	ENSSSCT00000019634	NCOR1	intron	-0.114	.	2
AC.GT	12	62725893	62725911	9.5	ENSSSCT00000019641	SLC47A1	intron	.	.	2
AAC.GTT	12	62925204	62925218	5	.	.	intergenic	.	rs788281346	2
AC.GT	12	63083567	63083583	8.5	.	.	intergenic	.	794969 rs7885	2
AAAC.GTTT	12	63428133	63428150	4.5	.	.	intergenic	0.004	rs790542815	2
AAC.GTT	13	39146	39170	8.33333	.	.	intergenic	.	rs790008254	4
AAAG.CTTT	13	105570	105584	3.75	.	.	intergenic	.	rs790254515	3
AC.GT	13	500072	500086	7.5	ENSSSCT00000029249	.	intron	.	rs792810317	2
.CACTC.GAGTG	13	518521	518549	4.83333	.	.	intergenic	0.215	943350 rs7900	2
AC.GT	13	599873	599890	9	ENSSSCT00000033415	CPNE4	intron	0.295	.	4

AC.GT	13	599873	599890	9	ENSSSCT00000012241	CPNE4	intron	0.295	.	4
AAAT.ATTT	13	820512	820530	4.75	.	.	intergenic	.	rs793535439	2
AAAAC.GTTTT	13	1129743	1129773	5.16667	ENSSSCT00000012246	ATP2C1	intron	0.032	344126 rs7864	2
AT.AT	13	1203798	1203810	6.5	ENSSSCT00000012246	ATP2C1	intron	.	193548 rs7921	2
AG.CT	13	1258937	1258947	5.5	.	.	intergenic	0.618	.	2
AG.CT	13	1314683	1314702	10	.	.	intergenic	-0.155	rs789654903	3
AG.CT	13	1379723	1379732	5	.	.	intergenic	0.289	rs791633651	3
AAAC.GTTT	13	1551721	1551734	3.5	.	.	intergenic	-0.047	rs791994657	2
AT.AT	13	1844201	1844231	15.5	ENSSSCT00000012249	COL6A5	intron	0.103	594311 rs7913	2
AAAT.ATTT	13	1859405	1859420	4	ENSSSCT00000012249	COL6A5	intron	0.852	rs789354620	2
AC.GT	13	1873879	1873891	6.5	ENSSSCT00000012249	COL6A5	intron	0.087	.	3
AG.CT	13	1885079	1885093	7.5	.	.	intergenic	.	rs790708406	3
AAAT.ATTT	13	2040367	2040386	5	.	.	intergenic	.	rs788789861	2
AAAAT.ATTTT	13	2461221	2461252	6.4	ENSSSCT00000032548	SH3BP5	intron	-0.108	rs787533892	2
AAAC.GTTT	13	2559642	2559655	3.5	.	.	intergenic	.	rs792967134	3
AAAT.ATTT	13	3120540	3120554	3.75	ENSSSCT00000012259	ANKRD28	intron	0.194	rs790320256	2
AAAC.GTTT	13	3139849	3139895	11.75	ENSSSCT00000012259	ANKRD28	intron	0.048	340974 rs7902	2
AAAC.GTTT	13	3147930	3147947	4.5	ENSSSCT00000012259	ANKRD28	intron	0.181	rs793117341	2
AAAAG.CTTTT	13	3168080	3168098	3.8	ENSSSCT00000012259	ANKRD28	intron	.	359180 rs7902	2
AAAAC.GTTTT	13	3263899	3263921	4.6	.	.	intergenic	.	rs788376436	2
AT.AT	13	3266147	3266157	5.5	.	.	intergenic	.	.	4
AATT.AATT	13	3309997	3310011	3.75	.	.	intergenic	.	rs789013397	2
AAAT.ATTT	13	3360831	3360846	4	.	.	intergenic	0.154	394961 rs7935	2
AC.GT	13	3681161	3681171	5.5	.	.	intergenic	0.41	rs791505356	2
AAAAC.GTTTT	13	3884788	3884808	4.2	.	.	intergenic	0.057	rs790731392	2
AAAC.GTTT	13	4222431	4222453	5.75	.	.	intergenic	0.136	218848 rs7909	2
AAAAAT.ATTTTT	13	4350184	4350204	3.5	.	.	intergenic	-0.059	rs793254451	2
AAAC.GTTT	13	4614734	4614748	3.75	ENSSSCT00000012264	TBC1D5	intron	0.408	rs790715038	2
AAAC.GTTT	13	4614734	4614748	3.75	ENSSSCT00000012265	TBC1D5	intron	0.408	rs790715038	2
AAAAC.GTTTT	13	4709792	4709812	4.2	ENSSSCT00000012264	TBC1D5	intron	0.145	rs793804749	2
AAAAC.GTTTT	13	4709792	4709812	4.2	ENSSSCT00000012265	TBC1D5	intron	0.145	rs793804749	2
AAAC.GTTT	13	4767890	4767911	5.5	ENSSSCT00000012264	TBC1D5	intron	0.102	rs787642185	4
AAAC.GTTT	13	4767890	4767911	5.5	ENSSSCT00000012265	TBC1D5	intron	0.102	rs787642185	4
AAAAT.ATTTT	13	5102173	5102188	3.2	.	.	intergenic	0.3	375718 rs7863	2
AAAG.CTTT	13	5507943	5507959	4.25	.	.	intergenic	0.425	976534 rs7874	2
AC.GT	13	5785976	5786008	16.5	.	.	intergenic	0.113	901165 rs7922	3
AT.AT	13	5808278	5808288	5.5	.	.	intergenic	0.471	rs792279375	4
AAT.ATT	13	5909329	5909344	5.33333	.	.	intergenic	.	.	2
AAAT.ATTT	13	6047996	6048010	3.75	ENSSSCT00000012267	SATB1	intron	-0.241	rs792776629	2
AC.GT	13	6148248	6148263	8	.	.	intergenic	0.207	.	2
AT.AT	13	6544587	6544615	14.5	.	.	intergenic	-0.09	272536 rs7894	2
AAAT.ATTT	13	6603564	6603581	4.5	.	.	intergenic	0.416	rs793343215	2
AC.GT	13	6691988	6692000	6.5	.	.	intergenic	-0.229	rs793242763	2
AG.CT	13	6783130	6783139	5	.	.	intergenic	0.51	rs786348939	2
AC.GT	13	6894184	6894198	7.5	.	.	intergenic	-0.033	031236 rs7936	7
AT.AT	13	7122707	7122716	5	.	.	intergenic	-0.108	rs792159334	2
AT.AT	13	7159836	7159854	9.5	.	.	intergenic	.	011652 rs7909	2
AAAC.GTTT	13	7370956	7370970	3.75	.	.	intergenic	-0.087	425800 rs7894	2
AG.CT	13	7373920	7373929	5	.	.	intergenic	0.074	.	3
AAAC.GTTT	13	7446351	7446363	3.25	ENSSSCT00000012270	KCNH8	intron	0.059	rs786779855	2
AT.AT	13	7469347	7469373	13.5	ENSSSCT00000012270	KCNH8	intron	0.148	.	2
AAAT.ATTT	13	7737381	7737398	4.5	ENSSSCT00000012271	EFHB	intron	.	rs787868331	2
AAT.ATT	13	7754691	7754703	4.33333	ENSSSCT00000012271	EFHB	intron	0.181	rs709925234	2
AC.GT	13	7758740	7758751	6	ENSSSCT00000012271	EFHB	intron	0.594	rs787086315	2
ATC.GAT	13	8010418	8010432	5	ENSSSCT00000024917	KAT2B	intron	.	rs793575345	2
AG.CT	13	8033940	8033950	5.5	ENSSSCT00000024917	KAT2B	intron	-0.255	rs789588750	3
AC.GT	13	8140578	8140587	5	.	.	intergenic	.	rs787232243	2
AC.GT	13	8328347	8328357	5.5	.	.	intergenic	0.044	.	4
AC.GT	13	8933057	8933066	5	.	.	intergenic	.	rs791392229	3
AAAC.GTTT	13	8933920	8933938	4.75	.	.	intergenic	.	312116 rs7887	2
AG.CT	13	8935884	8935899	8	.	.	intergenic	-0.13	447951 rs7871	2
ACAG.CTGT	13	8961621	8961637	4.25	.	.	intergenic	-0.202	rs788098360	3
AC.GT	13	9081598	9081608	5.5	.	.	intergenic	.	.	7
AAAT.ATTT	13	9210172	9210196	6.25	.	.	intergenic	0.134	rs793625084	2
AT.AT	13	9443173	9443183	5.5	.	.	intergenic	.	544007 rs7878	2
AG.CT	13	9475381	9475395	7.5	.	.	intergenic	0.367	rs788928957	2
AC.GT	13	9724766	9724776	5.5	ENSSSCT00000012274	ZNF385D	intron	0.205	.	4
AAAAC.GTTTT	13	9857103	9857123	4.2	.	.	intergenic	-0.111	.	2
AT.AT	13	9874269	9874289	10.5	.	.	intergenic	.	rs790582602	2
AAC.GTT	13	9890559	9890577	6.33333	.	.	intergenic	-0.04	041657 rs7937	3
AG.CT	13	10027194	10027204	5.5	.	.	intergenic	-0.186	rs788795698	2
AG.CT	13	10037041	10037057	8.5	.	.	intergenic	.	rs787336068	2
AC.GT	13	10098806	10098833	14	.	.	intergenic	0.038	.	2
AT.AT	13	10292816	10292826	5.5	.	.	intergenic	.	rs790301135	2
AAC.GTT	13	10308360	10308379	6.66667	.	.	intergenic	.	370213 rs7932	2
AAAAC.GTTTT	13	10337845	10337861	3.4	.	.	intergenic	0.042	rs789246528	2
AC.GT	13	10452923	10452935	6.5	.	.	intergenic	-0.087	rs792119252	4
AAAC.GTTT	13	10582141	10582159	4.75	.	.	intergenic	0.07	481239 rs7881	2
AAC.GTT	13	10714072	10714088	5.66667	.	.	intergenic	0.345	rs792440453	2
AC.GT	13	10811241	10811253	6.5	.	.	intergenic	0.081	rs789177049	2
AAAC.GTTT	13	11049814	11049832	4.75	.	.	intergenic	-0.025	.	2
AATG.CATT	13	11164218	11164246	7.25	.	.	intergenic	.	.	2
AAAT.ATTT	13	11225614	11225631	4.5	.	.	intergenic	0.135	rs792929112	2
AAT.ATT	13	11247484	11247495	4	.	.	intergenic	-0.063	.	2

AG.CT	13	11267607	11267618	6	.	.	intergenic	0.336	.	4
AAAT.ATTT	13	11293284	11293302	4.75	.	.	intergenic	0.414	rs786645934	2
AG.CT	13	11338888	11338900	6.5	.	.	intergenic	-0.003	rs787007697	2
AAAAG.CTTTT	13	11432752	11432768	3.4	.	.	intergenic	0.259	rs786903113	2
AAAC.GTTT	13	11475723	11475737	3.75	.	.	intergenic	0.099	765599 rs7923	2
AAAC.GTTT	13	11566215	11566239	6.25	.	.	intergenic	0.356	.	2
AG.CT	13	11649728	11649738	5.5	.	.	intergenic	0.161	.	2
ATC.GAT	13	11653998	11654017	6.66667	.	.	intergenic	-0.003	rs791592517	2
AC.GT	13	11707485	11707494	5	ENSSSCT00000012275	UBE2E2	intron	.	.	2
AC.GT	13	11707485	11707494	5	ENSSSCT00000035075	UBE2E2	intron	.	.	2
AG.CT	13	11863531	11863541	5.5	.	.	intergenic	.	rs791006428	2
AC.GT	13	12188318	12188334	8.5	.	.	intergenic	-0.079	.	4
AC.GT	13	12338607	12338619	6.5	.	.	intergenic	.	rs792620503	3
AAAAGG.CCTTT	13	12436621	12436641	3.5	ENSSSCT00000012277	THRB	intron	0.591	320136 rs7873	2
AC.GT	13	12493057	12493077	10.5	ENSSSCT00000012277	THRB	intron	0.769	148160 rs7921	2
AC.GT	13	12540012	12540030	9.5	.	.	intergenic	0.137	rs793545909	2
AT.AT	13	12659955	12659967	6.5	.	.	intergenic	0.127	168273 rs7888	2
AC.GT	13	12800126	12800143	9	.	.	intergenic	-0.123	413926 rs7863	4
AT.AT	13	12954072	12954099	14	.	.	intergenic	0.165	.	4
AAAC.GTTT	13	13000721	13000739	4.75	.	.	intergenic	.	.	2
AG.CT	13	13042411	13042426	8	.	.	intergenic	0.075	rs791126904	4
AAT.ATT	13	13185781	13185792	4	.	.	intergenic	0.068	rs788261872	2
AGATAT.ATATC	13	13243319	13243350	5.33333	.	.	intergenic	.	rs789131309	2
AAAAT.ATTTT	13	13327848	13327872	5	.	.	intergenic	-0.089	rs789529248	2
AAAAT.ATTTT	13	13346123	13346138	3.2	.	.	intergenic	-0.267	rs789228912	2
AC.GT	13	13360656	13360672	8.5	.	.	intergenic	-0.037	rs786937662	2
AAT.ATT	13	13378917	13378934	6	.	.	intergenic	-0.067	rs791836680	2
AG.CT	13	13474705	13474730	13	.	.	intergenic	0.009	533439 rs7903	2
AG.CT	13	13827964	13827979	8	.	.	intergenic	-0.047	rs787657263	2
AAAG.CTTT	13	13922930	13922949	5	.	.	intergenic	.	rs792412171	2
AT.AT	13	14172457	14172470	7	ENSSSCT00000012279	TOP2B	intron	0.183	915503 rs7893	2
AAAAAC.GTTTT	13	14279431	14279453	3.83333	ENSSSCT00000012280	NGLY1	intron	0.266	rs787035754	2
AAT.ATT	13	14639389	14639401	4.33333	.	.	intergenic	-0.075	rs791581923	2
AG.CT	13	14762658	14762667	5	.	.	intergenic	-0.095	.	2
AAAC.GTTT	13	14819146	14819160	3.75	.	.	intergenic	.	rs789595618	2
AAAT.ATTT	13	14861327	14861347	5.25	.	.	intergenic	0.186	303616 rs7895	2
AAC.GTT	13	14923909	14923936	9.33333	.	.	intergenic	.	rs792869017	2
AAAAAT.ATTTTT	13	15050479	15050502	4	.	.	intergenic	0.188	rs793199945	2
AAC.GTT	13	15120208	15120223	5.33333	.	.	intergenic	-0.075	.	3
AAAC.GTTT	13	15166107	15166125	4.75	.	.	intergenic	0.182	rs789200198	2
AT.AT	13	15166250	15166260	5.5	.	.	intergenic	0.107	rs787484679	5
AAAT.ATTT	13	15264875	15264899	6.25	.	.	intergenic	0.214	rs788616890	2
AATT.AATT	13	15439573	15439594	5.5	ENSSSCT00000012284	NEK10	intron	0.528	rs792253824	2
AATT.AATT	13	15439573	15439594	5.5	ENSSSCT00000012283	NEK10	intron	0.528	rs792253824	2
AAAC.GTTT	13	15470473	15470487	3.75	ENSSSCT00000012284	NEK10	intron	0.117	rs787813529	2
AAAC.GTTT	13	15470473	15470487	3.75	ENSSSCT00000012283	NEK10	intron	0.117	rs787813529	2
AAAG.CTTT	13	15499308	15499325	4.5	ENSSSCT00000012285	SLC4A7	intron	2.153	rs788542756	2
AAAG.CTTT	13	16007952	16007968	4.25	.	.	intergenic	-0.151	rs787614402	2
AT.AT	13	16226120	16226135	8	.	.	intergenic	0.008	rs787375335	2
AAAAC.GTTTT	13	16326862	16326878	3.4	.	.	intergenic	.	509853 rs6900	2
ACAG.CTGT	13	16550752	16550764	3.25	.	.	intergenic	-0.422	rs793381989	2
AAC.GTT	13	16613380	16613394	5	.	.	intergenic	.	.	3
AC.GT	13	17000409	17000421	6.5	.	.	intergenic	-0.021	324500 rs7877	2
AT.AT	13	17249037	17249046	5	.	.	intergenic	.	rs788275271	2
AC.GT	13	17405196	17405207	6	.	.	intergenic	-0.278	.	3
AC.GT	13	17428581	17428595	7.5	.	.	intergenic	-0.264	.	2
AC.GT	13	17444931	17444942	6	.	.	intergenic	0.092	.	2
AC.GT	13	17563590	17563600	5.5	.	.	intergenic	-0.031	rs790212611	2
AAAC.GTTT	13	17701480	17701494	3.75	.	.	intergenic	0.024	rs790221028	2
AC.GT	13	17770440	17770451	6	.	.	intergenic	0.934	.	3
AAT.ATT	13	18466822	18466846	8.33333	.	.	intergenic	0.047	.	2
AC.GT	13	18594917	18594936	10	.	.	intergenic	-0.202	rs789050651	2
AG.CT	13	18694510	18694541	16	.	.	intergenic	-0.173	rs789137709	2
AC.GT	13	18730094	18730112	9.5	.	.	intergenic	0.134	rs790393039	2
AAC.GTT	13	18870291	18870308	6	.	.	intergenic	0.115	088608 rs7915	2
AT.AT	13	19169060	19169070	5.5	.	.	intergenic	0.194	rs789410952	2
AT.AT	13	19415332	19415341	5	.	.	intergenic	0.107	rs786790125	4
AAAAT.ATTTT	13	19453310	19453328	3.8	.	.	intergenic	-0.024	rs792708129	2
AG.CT	13	19503297	19503309	6.5	.	.	intergenic	.	rs789822881	4
AGG.CCT	13	19637750	19637766	5.66667	.	.	intergenic	0.206	rs788980750	2
AC.GT	13	19682684	19682696	6.5	ENSSSCT00000012296	STT3B	intron	0.116	735707 rs7938	2
AAAAC.GTTTT	13	19830763	19830792	6	ENSSSCT00000012297	OSBPL10	intron	-0.063	rs789579719	3
AC.GT	13	19906288	19906299	6	ENSSSCT00000012297	OSBPL10	intron	.	.	2
AAAAAC.GTTTTT	13	19913016	19913036	3.5	ENSSSCT00000012297	OSBPL10	intron	.	rs700450684	2
AC.GT	13	20001883	20001893	5.5	ENSSSCT00000012297	OSBPL10	intron	-0.091	rs792618394	2
AAT.ATT	13	20086769	20086787	6.33333	ENSSSCT00000012297	OSBPL10	intron	0.343	rs792979632	2
AAC.GTT	13	20091838	20091855	6	ENSSSCT00000012297	OSBPL10	intron	-0.004	rs707838022	2
AT.AT	13	20103954	20103964	5.5	ENSSSCT00000012297	OSBPL10	intron	-0.315	rs792862113	2
AAC.GTT	13	20124742	20124754	4.33333	ENSSSCT00000012297	OSBPL10	intron	-0.053	.	2
AT.AT	13	20257325	20257337	6.5	.	.	intergenic	.	223348 rs7878	2
AAAC.GTTT	13	20419022	20419038	4.25	.	.	intergenic	0.095	962779 rs7886	2
AC.GT	13	20490591	20490601	5.5	ENSSSCT00000026794	CMTM8	intron	0.02	rs690275310	2
AC.GT	13	20490591	20490601	5.5	ENSSSCT00000030407	CMTM8	intron	0.02	rs690275310	2
AAAC.GTTT	13	20586775	20586789	3.75	.	.	intergenic	.	rs790077680	2

AT.AT	13	21034786	21034800	7.5	ENSSSCT00000012303	CLASP2	intron	.	rs791571602	2
AT.AT	13	21034786	21034800	7.5	ENSSSCT00000025077	CLASP2	intron	.	rs791571602	2
AAAAC.GTTTT	13	21096735	21096763	4.83333	ENSSSCT00000012303	CLASP2	promoter	-0.318	rs701351268	2
AAAAC.GTTTT	13	21096735	21096763	4.83333	ENSSSCT00000025077	CLASP2	promoter	-0.318	rs701351268	2
AC.GT	13	21403631	21403651	10.5	.	.	intergenic	0.073	rs789289119	2
AT.AT	13	22057288	22057302	7.5	.	.	intergenic	0.191	.	2
AC.GT	13	22129813	22129825	6.5	.	.	intergenic	0.71	.	4
AT.AT	13	22158238	22158255	9	.	.	intergenic	-0.146	.	3
AC.GT	13	22642202	22642221	10	.	.	intergenic	.	72120 rs7893	2
AAAC.GTTT	13	22791856	22791873	4.5	ENSSSCT00000012305	ARPP21	intron	0.004	rs788596871	2
AAAC.GTTT	13	22811373	22811386	3.5	ENSSSCT00000012305	ARPP21	intron	-0.267	92823 rs7932	2
AGAT.ATCT	13	23166871	23166907	9.25	.	.	intergenic	.	63530 rs7866	2
AC.GT	13	23288776	23288788	6.5	.	.	intergenic	.	05065 rs7881	2
AG.CT	13	23499149	23499173	12.5	.	.	intergenic	0.004	.	4
AC.GT	13	23528267	23528277	5.5	.	.	intergenic	.	.	2
AAAC.GTTT	13	23533664	23533684	5.25	.	.	intergenic	0.257	rs789821148	2
AAAAC.GTTTT	13	23556659	23556695	7.4	.	.	intergenic	.	rs790044559	2
AT.AT	13	23636741	23636750	5	ENSSSCT00000027443	MLH1	intron	.	.	4
AG.CT	13	23798289	23798301	6.5	.	.	intergenic	0.397	.	3
AAAC.GTTT	13	23825414	23825428	3.75	.	.	intergenic	.	rs792261865	2
AAAT.ATTT	13	24000568	24000586	4.75	.	.	intergenic	0.226	rs789058024	2
AC.GT	13	24666870	24666892	11.5	ENSSSCT00000030803	.	intron	0.207	.	4
AAAT.ATTT	13	24719539	24719557	4.75	ENSSSCT00000030803	.	intron	2.45	rs787443306	2
AAAAT.ATTTT	13	25473356	25473379	4.8	ENSSSCT00000012326	EXOG	intron	0.188	66845 rs7906	2
AC.GT	13	25597883	25597900	9	ENSSSCT00000012327	SCN5A	intron	-0.039	.	2
AATG.CATT	13	25614771	25614800	7.5	.	.	intergenic	0.095	rs792519025	2
AC.GT	13	25741247	25741256	5	ENSSSCT00000012328	SCN10A	intron	-0.042	60217 rs7111	4
AG.CT	13	25797457	25797479	11.5	.	.	intergenic	0.078	15796 rs7892	2
AC.GT	13	25983994	25984004	5.5	.	.	intergenic	.	rs788203292	2
AAC.GTT	13	26044919	26044930	4	ENSSSCT00000028766	GORASP1	3'utr	1.079	rs791676965	2
AC.GT	13	26073005	26073017	6.5	ENSSSCT00000022750	TTC21A	intron	-0.127	rs788352832	2
AC.GT	13	26073005	26073017	6.5	ENSSSCT00000012331	TTC21A	intron	-0.127	rs788352832	2
AAAC.GTTT	13	26302383	26302398	4	ENSSSCT00000029630	SLC25A38	intron	-0.045	rs791835222	2
AC.GT	13	26449628	26449638	5.5	ENSSSCT00000012335	MOBP	promoter	0.013	rs793000087	2
AAAT.ATTT	13	26583128	26583145	4.5	.	.	intergenic	0.086	rs787080389	2
AC.GT	13	26657314	26657332	9.5	ENSSSCT00000012336	.	intron	-0.077	rs789092451	2
AT.AT	13	26689813	26689823	5.5	ENSSSCT00000012336	.	intron	0.193	rs789364549	3
AAT.ATT	13	27030611	27030631	7	.	.	intergenic	.	rs791501570	2
AAAG.CTTT	13	27391519	27391536	4.5	.	.	intergenic	0.512	rs790338659	2
AT.AT	13	27547545	27547558	7	.	.	intergenic	-0.12	27807 rs7869	2
AT.AT	13	27553434	27553444	5.5	.	.	intergenic	-0.094	.	3
AAAAC.GTTTT	13	27589977	27590000	4	.	.	intergenic	0.045	71176 rs7866	2
ATCC.GGAT	13	27599815	27599841	6.75	.	.	intergenic	.	59229 rs7915	2
AT.AT	13	27634646	27634657	6	ENSSSCT00000012342	CTNNB1	intron	-0.017	rs787241206	2
AAGG.CCTT	13	27687245	27687280	9	.	.	intergenic	.	95207 rs7864	2
AT.AT	13	27783683	27783693	5.5	.	.	intergenic	.	.	3
AAAAC.GTTTT	13	27845862	27845879	3.6	.	.	intergenic	.	rs710615137	2
AAAAT.ATTTT	13	27907846	27907863	3.6	.	.	intergenic	.	.	2
AC.GT	13	27916849	27916858	5	.	.	intergenic	0.065	.	3
AAAG.CTTT	13	28166147	28166165	4.75	.	.	intergenic	0.03	rs790362967	2
AT.AT	13	28175575	28175590	8	.	.	intergenic	-0.019	rs789808395	3
AAAC.GTTT	13	28203800	28203819	5	ENSSSCT00000029576	.	intron	.	81202 rs7906	3
AAAC.GTTT	13	28487920	28487940	5.25	.	.	intergenic	.	22589 rs7903	2
ATCC.GGAT	13	28493356	28493392	9.25	.	.	intergenic	0.477	rs792471742	2
AC.GT	13	28500128	28500140	6.5	.	.	intergenic	-0.203	33455 rs7908	2
AAC.GTT	13	28564660	28564680	7	ENSSSCT00000012347	TRAK1	intron	-0.093	rs793476475	4
AC.GT	13	28603760	28603770	5.5	ENSSSCT00000012347	TRAK1	3'utr	0.473	rs786550572	2
AAAAG.CTTTT	13	28865856	28865881	5.2	.	.	intergenic	.	rs791011066	2
AAC.GTT	13	28949406	28949434	9.66667	ENSSSCT00000012360	HIGD1A	intron	0.171	rs787505788	2
AC.GT	13	29109597	29109607	5.5	.	.	intergenic	0.193	rs790846018	2
AG.CT	13	29708209	29708224	8	ENSSSCT00000012366	ANO10	intron	0.182	14575 rs7894	2
AAAT.ATTT	13	29753039	29753053	3.75	ENSSSCT00000012366	ANO10	intron	0.228	92068 rs7862	2
AAAC.GTTT	13	29930167	29930184	4.5	.	.	intergenic	-0.062	rs792970677	2
AT.AT	13	29955403	29955419	8.5	.	.	intergenic	-0.004	rs786291030	2
AG.CT	13	30055044	30055053	5	.	.	intergenic	-0.068	rs791213493	2
AT.AT	13	30085698	30085707	5	.	.	intergenic	0.183	.	4
AAAC.GTTT	13	30224034	30224059	6.5	.	.	intergenic	-0.044	32060 rs7870	2
AAC.GTT	13	30629480	30629493	4.66667	.	.	intergenic	.	54839 rs7911	2
AC.GT	13	30723803	30723814	6	ENSSSCT00000012375	ZNF197	promoter	0.189	rs790066577	3
AAC.GTT	13	30811178	30811191	4.66667	ENSSSCT00000012379	KIAA1143	promoter	0.162	rs790562658	3
AT.AT	13	30866688	30866714	13.5	ENSSSCT00000012380	KIF15	intron	0.022	rs787287836	2
AAAAC.GTTTT	13	30955083	30955121	7.8	.	.	intergenic	.	rs790020294	3
ACC.GGT	13	31302836	31302849	4.66667	ENSSSCT00000024122	.	intron	-0.124	38420 rs7904	3
AAAG.CTTT	13	31329055	31329073	4.75	.	.	intergenic	0.132	rs790795922	2
AAAG.CTTT	13	31435242	31435260	4.75	.	.	intergenic	-0.236	rs791510199	2
AT.AT	13	31562695	31562706	6	ENSSSCT00000012384	.	intron	0.021	rs788364185	2
ATCAT.ATGAT	13	31581192	31581214	3.83333	ENSSSCT00000012384	.	intron	0.057	rs789956898	3
AG.CT	13	32197752	32197765	7	ENSSSCT00000035018	FYCO1	intron	-0.486	44643 rs7886	2
AG.CT	13	32298166	32298193	14	.	.	intergenic	-0.068	rs792986480	2
AC.GT	13	32542826	32542837	6	.	.	intergenic	-0.23	rs792264969	2
AG.CT	13	32717675	32717701	13.5	.	.	intergenic	-0.153	rs793629680	4
AAAAC.GTTTT	13	33094126	33094149	4.8	ENSSSCT00000025127	.	intron	-0.052	rs788524594	2
AAAAC.GTTTT	13	33094126	33094149	4.8	ENSSSCT00000012405	.	intron	-0.052	rs788524594	2
AAAC.GTTT	13	33310562	33310580	4.75	.	.	intergenic	0.127	rs790291429	2

AC.GT	13	33312926	33312937	6	.	.	intergenic	0.142	rs793592069	2
AAAC.GTTT	13	33437611	33437631	5.25	.	.	intergenic	.	rs791748963	2
AAC.GTT	13	33533443	33533456	4.66667	.	.	intergenic	.	145069 rs7932	2
AATG.CATT	13	33555282	33555316	8.75	.	.	intergenic	.	303525 rs7913	2
AAAT.ATTT	13	33729264	33729287	6	ENSSSCT00000023426	.	intron	.	405113 rs7888	2
AAAT.ATTT	13	33729264	33729287	6	ENSSSCT00000012417	.	intron	.	405113 rs7888	2
AC.GT	13	33798903	33798913	5.5	ENSSSCT00000012417	.	intron	2.621	rs696260336	4
AATC.GATT	13	34006501	34006523	5.75	.	.	intergenic	-0.046	010919 rs7876	2
AC.GT	13	34028286	34028296	5.5	.	.	intergenic	0.248	rs793491676	2
AC.GT	13	34495018	34495030	6.5	ENSSSCT00000012430	PFKFB4	intron	0.877	rs792183956	3
AAAC.GTTT	13	35531933	35531964	8	.	.	intergenic	.	rs790231630	2
AG.CT	13	35814319	35814341	11.5	.	.	intergenic	-0.247	.	4
AG.CT	13	35968380	35968396	8.5	ENSSSCT00000029394	IFRD2	intron	-0.026	.	3
AC.GT	13	36217366	36217381	8	.	.	intergenic	0.436	rs790515932	4
AAAAC.GTTTT	13	36477339	36477362	4.8	.	.	intergenic	0.173	rs789667610	2
AAAC.GTTT	13	36486841	36486856	4	.	.	intergenic	.	rs786308065	2
AAAG.CTTT	13	36872806	36872820	3.75	.	.	intergenic	0.214	rs792150056	2
AAAAC.GTTTT	13	36890017	36890041	5	.	.	intergenic	1.252	555610 rs7886	2
AC.GT	13	37686813	37686828	8	ENSSSCT00000025398	DNAH1	intron	-0.243	rs787490335	2
AAAAT.ATTTT	13	38046664	38046687	4.8	ENSSSCT00000012526	NEK4	intron	0.288	510602 rs7909	2
AG.CT	13	38167587	38167606	10	.	.	intergenic	.	rs712945353	2
AC.GT	13	38648424	38648433	5	.	.	intergenic	-0.691	.	2
AC.GT	13	38685415	38685429	7.5	.	.	intergenic	-0.031	rs789075309	4
AG.CT	13	38744672	38744689	9	.	.	intergenic	-0.086	rs793601963	2
AGCCC.GGGCT	13	38942621	38942643	4.6	ENSSSCT00000012536	.	intron	-0.076	427140 rs7884	2
GGCCC.GGGCC	13	38943801	38943823	3.83333	ENSSSCT00000012536	.	intron	-0.244	rs787102476	2
AAAT.ATTT	13	39233286	39233301	4	.	.	intergenic	.	rs787355941	2
AC.GT	13	39382690	39382716	13.5	.	.	intergenic	0.087	.	2
AAAAC.GTTTT	13	39914601	39914621	4.2	ENSSSCT00000023696	.	intron	-0.057	rs791793745	2
AAAT.ATTT	13	40205123	40205141	4.75	ENSSSCT00000024071	.	intron	-0.065	rs786360471	2
AC.GT	13	40625837	40625858	11	.	.	intergenic	0.564	.	2
AAGG.CCTT	13	40929189	40929208	5	.	.	intergenic	0.086	rs786739210	2
AT.AT	13	41522244	41522263	10	ENSSSCT00000030632	.	intron	0.189	.	2
AG.CT	13	41533755	41533767	6.5	ENSSSCT00000030632	.	intron	0.25	.	3
AAC.GTT	13	41769319	41769338	6.66667	ENSSSCT00000028128	.	intron	0.059	rs791743426	2
ACC.GGT	13	41972450	41972462	4.33333	.	.	intergenic	0.101	rs786313758	2
AAAC.GTTT	13	42178222	42178241	5	.	.	intergenic	.	rs790442654	2
AAAC.GTTT	13	42242870	42242890	5.25	.	.	intergenic	0.469	rs788475631	2
AT.AT	13	42243463	42243474	6	.	.	intergenic	-0.351	rs786719828	2
AAAC.GTTT	13	42348829	42348852	6	ENSSSCT00000028725	FAM208A	intron	0.002	rs790243017	2
AG.CT	13	42412112	42412121	5	.	.	intergenic	.	rs787030650	2
AAAG.CTTT	13	42772852	42772870	4.75	.	.	intergenic	-0.163	rs790003471	2
AAAG.CTTT	13	42856153	42856164	3	.	.	intergenic	0.074	rs788981400	2
AAC.GTT	13	43007976	43007992	5.66667	.	.	intergenic	.	901050 rs7889	4
AAAT.ATTT	13	43331626	43331653	7	ENSSSCT00000012546	PDE12	3'utr	0.461	rs788681578	2
AT.AT	13	43447055	43447067	6.5	.	.	intergenic	0.123	353666 rs7863	2
AG.CT	13	44059671	44059681	5.5	ENSSSCT00000012556	PXK	intron	.	.	3
AAAAAC.GTTTTT	13	44141302	44141329	4.66667	.	.	intergenic	-0.101	rs791912056	2
AAACC.GGTTT	13	44755043	44755063	4.2	ENSSSCT00000012564	C3orf67	intron	.	rs790525899	2
AAACC.GGTTT	13	44755043	44755063	4.2	ENSSSCT00000012565	C3orf67	intron	.	rs790525899	2
AT.AT	13	44943325	44943335	5.5	.	.	intergenic	.	rs786255695	2
ACAT.ATGT	13	45150495	45150510	4	.	.	intergenic	0.381	rs787310034	3
AC.GT	13	45325393	45325404	6	.	.	intergenic	.	383237 rs7867	3
AAC.GTT	13	45406956	45406972	5.66667	.	.	intergenic	1.155	rs790696620	2
AT.AT	13	45567727	45567739	6.5	.	.	intergenic	.	rs788609955	3
AC.GT	13	45882309	45882326	9	.	.	intergenic	0.024	rs791011441	2
AAAC.GTTT	13	45988116	45988145	7.5	.	.	intergenic	0.392	368409 rs7875	2
AAAC.GTTT	13	46017568	46017582	3.75	.	.	intergenic	-0.393	rs789395611	2
AC.GT	13	46130984	46131014	15.5	.	.	intergenic	0.464	rs788772261	2
AT.AT	13	46328112	46328137	13	.	.	intergenic	0.052	rs786746624	2
AC.GT	13	46402002	46402018	8.5	.	.	intergenic	.	.	3
AG.CT	13	46457090	46457100	5.5	.	.	intergenic	1.064	.	2
AAAC.GTTT	13	47291955	47291973	4.75	.	.	intergenic	.	rs789083373	2
AAAT.ATTT	13	47312699	47312718	5	.	.	intergenic	0.977	073343 rs7892	2
AT.AT	13	47318549	47318559	5.5	.	.	intergenic	-0.064	rs787985369	2
AG.CT	13	47656411	47656420	5	.	.	intergenic	-0.126	.	4
AC.GT	13	47714460	47714470	5.5	.	.	intergenic	-0.026	066835 rs7910	3
AAT.ATT	13	47732354	47732365	4	ENSSSCT00000012568	PTPRG	intron	-0.233	rs787181173	3
AAGG.CCTT	13	48212530	48212542	3.25	ENSSSCT00000012572	CADPS	intron	0.073	.	2
AG.CT	13	48755712	48755723	6	.	.	intergenic	-0.094	.	3
AG.CT	13	48853984	48853998	7.5	.	.	intergenic	0.049	rs789117854	2
AC.GT	13	48878673	48878682	5	.	.	intergenic	0.121	.	4
AAT.ATT	13	49325975	49326004	10	.	.	intergenic	0.008	229219 rs7916	2
AC.GT	13	49532102	49532114	6.5	ENSSSCT00000023657	SYNPR	intron	0.061	rs786878749	2
AC.GT	13	49549173	49549183	5.5	ENSSSCT00000023657	SYNPR	intron	0.301	rs786613344	2
AG.CT	13	49571998	49572007	5	ENSSSCT00000023657	SYNPR	intron	0.074	rs692533966	3
AG.CT	13	49679548	49679572	12.5	ENSSSCT00000012575	SNTN	intron	-0.199	rs791347164	2
ATC.GAT	13	49738268	49738282	5	.	.	intergenic	-0.023	rs792101039	2
AAAAC.GTTTT	13	49764492	49764513	4.4	.	.	intergenic	-0.017	138311 rs7921	2
AGG.CCT	13	50084104	50084127	8	.	.	intergenic	0.029	rs787886527	2
AC.GT	13	50101760	50101772	6.5	ENSSSCT00000012578	PRICKLE2	intron	-0.001	rs787582462	2
AC.GT	13	50162419	50162442	12	ENSSSCT00000012578	PRICKLE2	intron	-0.159	.	3
AC.GT	13	50162419	50162442	12	ENSSSCT00000020410	7SK	promoter	-0.159	.	3
AAAC.GTTT	13	50274277	50274291	3.75	.	.	intergenic	.	514076 rs7923	3

AAAC.GTTT	13	50317993	50318020	7	.	.	intergenic	0.355	.	2
AAAC.GTTT	13	50375451	50375477	6.75	.	.	intergenic	-0.13	rs787025699	2
AC.GT	13	50375879	50375899	10.5	.	.	intergenic	.	.	4
AAT.ATT	13	50940234	50940253	6.66667	.	.	intergenic	-0.494	rs787336480	2
AAAC.GTTT	13	51006787	51006805	4.75	.	.	intergenic	0.524	470768 rs7897	3
AT.AT	13	51132467	51132476	5	.	.	intergenic	0.085	rs791389124	2
AAAC.GTTT	13	51390483	51390499	4.25	.	.	intergenic	0.163	rs791270571	2
AAAAC.GTTTT	13	51755788	51755816	4.83333	ENSSSCT00000012581	MAGI1	intron	0.707	rs704150598	2
AAAT.ATTT	13	51855328	51855353	6.5	.	.	intergenic	-0.145	959390 rs7883	2
AAG.CTT	13	51924829	51924841	4.33333	.	.	intergenic	.	330978 rs7938	2
AATG.CATT	13	52020468	52020490	5.75	.	.	intergenic	-0.052	rs792183963	2
AAAAG.CTTTT	13	52048922	52048939	3.6	.	.	intergenic	0.092	.	2
ACAT.ATGT	13	52112916	52112934	4.75	.	.	intergenic	-0.101	.	2
AAAG.CTTT	13	52285023	52285048	6.5	.	.	intergenic	-0.141	rs787159703	2
AAAT.ATTT	13	52444143	52444166	6	.	.	intergenic	.	rs789811949	2
AG.CT	13	52882811	52882825	7.5	.	.	intergenic	-0.399	rs793455553	2
AAAG.CTTT	13	53138151	53138168	4.5	.	.	intergenic	-0.349	578822 rs7892	2
AAAG.CTTT	13	53268364	53268382	4.75	.	.	intergenic	.	.	2
AT.AT	13	53915841	53915850	5	ENSSSCT00000012586	SUCLG2	intron	0.001	rs790885087	2
AAC.GTT	13	54122260	54122273	4.66667	.	.	intergenic	.	rs792541616	4
AG.CT	13	54146399	54146408	5	.	.	intergenic	-0.228	.	3
AAAAT.ATTTT	13	54672903	54672918	3.2	.	.	intergenic	.	rs792250497	2
ACAT.ATGT	13	55172700	55172722	5.75	.	.	intergenic	.	384859 rs7932	2
AACAC.GTGTT	13	55873707	55873730	4.8	ENSSSCT00000012598	FRMD4B	intron	-0.045	352894 rs7898	2
AAAT.ATTT	13	55993370	55993392	5.75	.	.	intergenic	0.098	747772 rs7936	2
AC.GT	13	56055724	56055736	6.5	.	.	intergenic	-0.021	rs792922318	2
AAAAC.GTTTT	13	56068553	56068581	5.8	.	.	intergenic	0.189	rs788508069	2
AAAAC.GTTTT	13	56397756	56397776	4.2	.	.	intergenic	0.085	556492 rs7897	2
AC.GT	13	56439631	56439648	9	.	.	intergenic	0.044	429265 rs7868	2
AAAC.GTTT	13	56728682	56728711	7.5	.	.	intergenic	0.154	754937 rs7890	2
ATC.GAT	13	56813348	56813365	6	.	.	intergenic	0.08	rs786327247	4
AC.GT	13	57191947	57191966	10	.	.	intergenic	0.157	rs701370608	2
AC.GT	13	57380990	57381011	11	.	.	intergenic	-0.195	364485 rs7884	2
AC.GT	13	57773298	57773307	5	.	.	intergenic	-0.091	.	2
AAACC.GGTTT	13	57893084	57893116	6.6	ENSSSCT00000022793	.	intron	-0.192	574033 rs7870	2
AAC.GTT	13	58553913	58553926	4.66667	.	.	intergenic	-0.131	rs791148983	2
AAG.CTT	13	58672860	58672876	5.66667	.	.	intergenic	-0.058	.	2
AAT.ATT	13	58829741	58829765	8.33333	.	.	intergenic	.	rs793496951	2
AAC.GTT	13	58934280	58934312	11	.	.	intergenic	0.149	193924 rs7026	6
AAAC.GTTT	13	58981208	58981232	6.25	.	.	intergenic	0.007	rs789561223	2
AAG.CTT	13	59209195	59209209	5	.	.	intergenic	-0.056	170744 rs7865	3
AATG.CATT	13	59431092	59431124	8.25	.	.	intergenic	-0.21	rs792205396	2
AC.GT	13	59578169	59578189	10.5	.	.	intergenic	.	062378 rs7869	2
AG.CT	13	59681886	59681897	6	.	.	intergenic	-0.098	rs790919278	2
AG.CT	13	59772469	59772481	6.5	ENSSSCT00000012605	SHQ1	intron	0.234	rs793190558	2
AAAC.GTTT	13	59847718	59847748	7.75	ENSSSCT00000012605	SHQ1	intron	0.126	rs790783651	2
AT.AT	13	59945999	59946024	13	ENSSSCT00000024001	GXYLT2	intron	.	196400 rs7916	2
AT.AT	13	59945999	59946024	13	ENSSSCT00000012607	GXYLT2	intron	.	196400 rs7916	2
AT.AT	13	60006780	60006790	5.5	ENSSSCT00000024001	GXYLT2	intron	-0.119	411582 rs7863	2
AT.AT	13	60006780	60006790	5.5	ENSSSCT00000012607	GXYLT2	intron	-0.119	411582 rs7863	2
AT.AT	13	60109083	60109101	9.5	.	.	intergenic	0.145	rs786925911	3
AC.GT	13	60298782	60298792	5.5	.	.	intergenic	-0.037	rs704071348	4
AC.GT	13	60410003	60410026	12	.	.	intergenic	-0.061	rs790172442	2
AC.GT	13	60528516	60528528	6.5	.	.	intergenic	-0.062	rs786278605	2
ACC.GGT	13	60534823	60534840	6	.	.	intergenic	-0.284	rs788710245	2
AAAC.GTTT	13	60538022	60538042	5.25	.	.	intergenic	-0.382	567711 rs7903	2
AAAC.GTTT	13	60612891	60612910	5	.	.	intergenic	-0.09	rs788630418	2
AAAAC.GTTTT	13	60645765	60645783	3.8	.	.	intergenic	-0.165	rs788634207	2
AG.CT	13	61106715	61106732	9	.	.	intergenic	.	527868 rs7871	2
AAAC.GTTT	13	61219753	61219767	3.75	.	.	intergenic	0.007	476763 rs7905	2
AAAC.GTTT	13	61471390	61471406	4.25	.	.	intergenic	.	rs793855624	2
ACAT.ATGT	13	61605868	61605882	3.75	ENSSSCT00000012610	CNTN3	intron	-0.125	.	4
AT.AT	13	61885989	61886004	8	.	.	intergenic	0.028	rs789468932	2
AAAAT.ATTTT	13	61911220	61911243	4.8	.	.	intergenic	0.027	rs792404628	2
AC.GT	13	61965120	61965130	5.5	.	.	intergenic	-0.126	rs786889732	2
AAAT.ATTT	13	62525769	62525783	3.75	.	.	intergenic	.	rs788026106	2
ACAT.ATGT	13	63001840	63001862	5.75	.	.	intergenic	0.11	rs790475416	2
AAAAG.CTTTT	13	63062302	63062319	3	.	.	intergenic	-0.06	rs786961117	2
AAATT.AATTT	13	63243666	63243688	4.6	.	.	intergenic	-0.23	rs790880635	2
AAAC.GTTT	13	63252226	63252240	3.75	.	.	intergenic	-0.105	rs789625145	2
AT.AT	13	63267140	63267151	6	.	.	intergenic	-0.171	rs792413675	2
AC.GT	13	63483337	63483349	6.5	.	.	intergenic	-0.014	.	2
AC.GT	13	63509129	63509140	6	.	.	intergenic	-0.119	220841 rs7932	2
AG.CT	13	63526322	63526336	7.5	.	.	intergenic	.	179272 rs7883	4
AAC.GTT	13	64274214	64274225	4	.	.	intergenic	-0.071	rs789668333	3
AAAT.ATTT	13	64834916	64834939	6	.	.	intergenic	0.098	rs790192565	2
AAAAC.GTTTT	13	64845159	64845181	4.6	.	.	intergenic	-0.164	158096 rs7883	2
AAAG.CTTT	13	65050157	65050171	3.75	.	.	intergenic	0.577	rs792726466	4
AT.AT	13	65141367	65141379	6.5	.	.	intergenic	-0.192	066379 rs7932	2
AAAAG.CTTTT	13	65644426	65644448	4.6	.	.	intergenic	0.416	585191 rs7897	2
AT.AT	13	65740502	65740513	6	.	.	intergenic	0.272	rs787566944	2
AAAG.CTTT	13	65767130	65767148	4.75	.	.	intergenic	0.587	rs788857402	2
AAAC.GTTT	13	65787763	65787781	4.75	.	.	intergenic	-0.078	983522 rs7885	2
AG.CT	13	65819747	65819757	5.5	.	.	intergenic	0.161	rs791993592	2

AAAC.GTTT	13	65896023	65896037	3.75	.	.	intergenic	0.358	rs789252641	2
AC.GT	13	65944338	65944362	12.5	.	.	intergenic	-0.039	rs789834090	2
AAAT.ATTT	13	65995850	65995874	6.25	.	.	intergenic	.	rs793481838	2
AAAAC.GTTTT	13	66169775	66169792	3.6	ENSSSCT00000012620	CRBN	intron	1.182	rs786861271	2
AAAAT.ATTTT	13	66503751	66503771	4.2	.	.	intergenic	-0.041	rs792030496	2
AAAC.GTTT	13	66717698	66717724	6.75	.	.	intergenic	0.075	rs792500441	2
AAC.GTT	13	66868541	66868552	4	.	.	intergenic	.	rs788100655	2
AAT.ATT	13	66896419	66896436	6	.	.	intergenic	0.244	rs790450337	3
AGAGG.CCTCT	13	67337864	67337886	3.83333	ENSSSCT00000012622	SUMF1	intron	0.352	rs787683635	2
AAAC.GTTT	13	67352732	67352754	5.75	.	.	intergenic	.	rs792634699	2
AAAAT.ATTTT	13	67845643	67845668	5.2	.	.	intergenic	0.106	331062 rs7936	2
AAAT.ATTT	13	67982374	67982396	5.75	.	.	intergenic	.	rs788438406	2
AC.GT	13	68170289	68170308	10	.	.	intergenic	0.061	rs792083379	2
AC.GT	13	68183825	68183842	9	.	.	intergenic	-0.086	175907 rs7881	2
AAAC.GTTT	13	68211672	68211690	4.75	.	.	intergenic	-0.151	rs788196845	2
AAAT.ATTT	13	68246111	68246126	4	.	.	intergenic	-0.097	rs789843072	2
AAC.GTT	13	68251274	68251293	6.66667	.	.	intergenic	0.002	rs793547420	2
AAAT.ATTT	13	68526303	68526321	4.75	.	.	intergenic	-0.055	rs789562208	2
AT.AT	13	68824348	68824358	5.5	.	.	intergenic	-0.147	.	5
AG.CT	13	69031669	69031681	6.5	.	.	intergenic	.	rs790513826	2
AT.AT	13	69236359	69236372	7	.	.	intergenic	-0.226	.	2
AC.GT	13	69291988	69292012	12.5	.	.	intergenic	-0.16	rs793185893	2
AAAC.GTTT	13	69462333	69462351	4.75	.	.	intergenic	0.046	.	2
AGAT.ATCT	13	69626060	69626089	7.5	.	.	intergenic	.	.	2
AG.CT	13	69629850	69629861	6	.	.	intergenic	-0.049	.	4
AAG.CTT	13	69665943	69665956	4.66667	.	.	intergenic	0.154	359982 rs7862	2
AAAAC.GTTTT	13	70356290	70356308	3.8	.	.	intergenic	-0.197	391661 rs7911	2
AG.CT	13	70533132	70533142	5.5	.	.	intergenic	0.468	062936 rs7929	4
AAAC.GTTT	13	70595017	70595032	4	ENSSSCT00000031304	GRM7	intron	0.035	rs787703290	2
AAT.ATT	13	70697249	70697273	8.33333	ENSSSCT00000031304	GRM7	intron	0.04	.	2
AAAT.ATTT	13	70754559	70754577	4.75	ENSSSCT00000031304	GRM7	intron	0.561	rs791854810	2
AAAG.CTTT	13	70930557	70930591	8.75	ENSSSCT00000031304	GRM7	intron	-0.117	.	2
ACAT.ATGT	13	71255699	71255713	3.75	.	.	intergenic	-0.08	rs786736555	2
AAAAC.GTTTT	13	71356395	71356418	4.8	.	.	intergenic	-0.289	rs786541371	2
AAT.ATT	13	71402307	71402326	6.66667	.	.	intergenic	-0.119	rs792036414	2
AAAC.GTTT	13	71815706	71815724	4.75	.	.	intergenic	-0.066	rs792777511	2
AGAT.ATCT	13	72192588	72192602	3.75	.	.	intergenic	0.175	rs793810941	2
AAGAG.CTCTT	13	72789793	72789811	3.16667	.	.	intergenic	.	.	3
AAAAT.ATTTT	13	72905961	72905975	3	ENSSSCT00000012630	SETD5	intron	0.084	338028 rs7870	2
AG.CT	13	73210770	73210789	10	ENSSSCT00000012645	RPUSD3	promoter	.	rs791498941	2
AC.GT	13	73588113	73588128	8	ENSSSCT00000013960	ATP2B2	intron	-0.228	037325 rs7906	4
AGG.CCT	13	73863692	73863704	4.33333	.	.	intergenic	.	.	2
AT.AT	13	74409593	74409607	7.5	.	.	intergenic	.	rs786963284	2
AT.AT	13	74415513	74415524	6	ENSSSCT00000027036	.	promoter	.	rs786808150	2
GAGGG.CCCTC	13	74707410	74707435	4.33333	ENSSSCT00000012667	ATG7	intron	0.069	rs790299824	2
AAAC.GTTT	13	74762683	74762699	4.25	ENSSSCT00000012667	ATG7	intron	0.254	rs791868246	2
AAAAC.GTTTT	13	74876573	74876588	3.2	ENSSSCT00000012667	ATG7	intron	0.529	002889 rs7902	2
AAAT.ATTT	13	74968446	74968459	3.5	.	.	intergenic	0.005	309457 rs7901	2
CCCCC.GGGGG	13	74989473	74989489	2.83333	.	.	intergenic	-0.021	981955 rs7933	2
AT.AT	13	75440072	75440081	5	.	.	intergenic	0.091	rs788357200	2
AT.AT	13	75568087	75568096	5	.	.	intergenic	0.284	rs792496486	3
AAAC.GTTT	13	76042237	76042259	5.75	ENSSSCT00000012675	.	intron	-0.059	461243 rs7885	2
AAAC.GTTT	13	76042237	76042259	5.75	ENSSSCT00000031032	.	intron	-0.059	461243 rs7885	2
AAAT.ATTT	13	76835228	76835250	5.75	ENSSSCT00000012690	NR2C2	intron	.	rs793836431	2
AC.GT	13	77154110	77154121	6	.	.	intergenic	.	rs713599403	2
AAAC.GTTT	13	77497413	77497449	9.25	ENSSSCT00000012696	.	intron	.	935243 rs7873	2
AAC.GTT	13	78671702	78671715	4.66667	ENSSSCT00000012707	NUP210	intron	.	.	5
AC.GT	13	78686757	78686768	6	ENSSSCT00000012707	NUP210	intron	-0.256	.	2
AATG.CATT	13	78999136	78999162	6.75	.	.	intergenic	-0.323	rs786658083	2
AAC.GTT	13	79399924	79399940	5.66667	ENSSSCT00000012708	HMCES	intron	.	rs793691100	2
AAAC.GTTT	13	79756435	79756459	6.25	.	.	intergenic	0.108	358739 rs7899	2
AAAC.GTTT	13	79811698	79811715	4.5	ENSSSCT00000012719	KBTBD12	intron	0.739	514511 rs7865	2
AC.GT	13	79873323	79873340	9	.	.	intergenic	.	rs792789795	2
AAC.GTT	13	79892784	79892796	4.33333	ENSSSCT00000012717	.	intron	.	314628 rs7924	3
AAAT.ATTT	13	79907799	79907818	5	ENSSSCT00000012717	.	intron	.	327698 rs7893	2
AAAT.ATTT	13	80948079	80948098	5	.	.	intergenic	.	rs791585417	2
AAT.GTT	13	81054073	81054098	8.66667	.	.	intergenic	.	185624 rs7875	2
AAAC.GTTT	13	81125911	81125922	3	.	.	intergenic	0.18	rs786466500	2
AG.CT	13	81184245	81184255	5.5	ENSSSCT00000012725	DNAJC13	intron	0.275	rs792803571	4
AAAAC.GTTTT	13	81435127	81435148	4.4	ENSSSCT00000012730	.	intron	2.485	.	2
AC.GT	13	81644410	81644422	6.5	.	.	intergenic	0.092	rs792351155	2
AAAC.GTTT	13	81705442	81705461	5	.	.	intergenic	0.14	281174 rs7912	2
AT.AT	13	81751732	81751743	6	.	.	intergenic	.	.	3
AAC.GTT	13	81952558	81952573	5.33333	.	.	intergenic	.	214072 rs7863	2
AAAC.GTTT	13	82041611	82041625	3.75	ENSSSCT00000012735	BFSP2	intron	.	327679 rs7908	2
AAAC.GTTT	13	82335551	82335569	4.75	ENSSSCT00000023415	.	intron	.	rs789738351	2
AAAC.GTTT	13	82420795	82420811	4.25	.	.	intergenic	.	450223 rs7879	2
AC.GT	13	82517483	82517494	6	ENSSSCT00000012738	RAB6B	intron	-0.442	.	4
AAAC.GTTT	13	82881653	82881668	4	ENSSSCT00000012743	RYK	intron	0.153	504892 rs7920	2
AAAG.CTTT	13	82961383	82961405	5.75	.	.	intergenic	-0.018	rs787472562	2
AAT.ATT	13	82970523	82970536	4.66667	.	.	intergenic	0.203	rs792650354	2
AAAC.GTTT	13	83063280	83063298	4.75	.	.	intergenic	-0.146	rs786367340	2
AAAAC.GTTTT	13	83085858	83085881	4.8	.	.	intergenic	.	rs788694178	2
AAAT.ATTT	13	83250505	83250531	6.75	.	.	intergenic	-0.004	574274 rs7921	2

AC.GT	13	83409746	83409758	6.5	.	.	intergenic	-0.048	.	3
AAAAC.GTTTT	13	83446694	83446712	3.8	.	.	intergenic	.	rs792554614	2
AAAC.GTTT	13	83587504	83587522	4.75	ENSSSCT00000012749	.	promoter	0.387	rs792061168	2
AAAC.GTTT	13	83677560	83677586	6.75	.	.	intergenic	0.039	rs792786960	2
AAG.CTT	13	84183182	84183219	12.6667	.	.	intergenic	0.639	.	2
AAAAC.GTTTT	13	84277344	84277362	3.8	.	.	intergenic	0.214	rs791785441	2
AAAAC.GTTTT	13	84582171	84582199	5.8	.	.	intergenic	.	327029 rs7909	2
AAAC.GTTT	13	84621035	84621046	3	ENSSSCT00000012757	PCCB	intron	.	rs788636489	3
AAATC.GATTT	13	84993461	84993479	3.8	ENSSSCT00000012754	.	intron	0.106	rs791218747	2
AG.CT	13	85046287	85046298	6	ENSSSCT00000012754	.	intron	.	.	2
AAAAAC.GTTTTT	13	85059932	85059950	3.16667	ENSSSCT00000012754	.	intron	.	256474 rs7920	2
AT.AT	13	85079479	85079489	5.5	ENSSSCT00000012754	.	intron	0.083	.	2
AAAC.GTTT	13	85225842	85225860	4.75	.	.	intergenic	0.221	373146 rs7912	2
AAAAC.GTTTT	13	85238798	85238816	3.8	.	.	intergenic	0.221	rs789399556	2
AT.AT	13	85242521	85242535	7.5	.	.	intergenic	0.242	.	2
AAAT.ATTT	13	85551837	85551859	5.75	.	.	intergenic	.	rs792965579	2
AC.GT	13	85733328	85733348	10.5	.	.	intergenic	-0.106	rs789941590	2
AAAC.GTTT	13	85753086	85753119	8.5	.	.	intergenic	-0.016	784509 rs7872	2
AAAC.GTTT	13	85849666	85849680	3.75	.	.	intergenic	0.194	rs789473158	2
AAAC.GTTT	13	85955109	85955129	5.25	.	.	intergenic	.	rs787416508	2
AATT.AATT	13	86162027	86162044	4.5	.	.	intergenic	.	rs792607108	2
AAAC.GTTT	13	86164736	86164750	3.75	.	.	intergenic	0.938	rs791295750	2
AAAC.GTTT	13	86317101	86317126	6.5	.	.	intergenic	.	rs789645413	2
AAT.ATT	13	86675562	86675582	7	ENSSSCT00000012762	.	intron	0.223	rs788142371	2
AAAAC.GTTTT	13	86748699	86748715	3.4	.	.	intergenic	0.078	934780 rs7935	2
ACC.GGT	13	86952825	86952840	5.33333	ENSSSCT00000023265	CEP70	intron	1.148	263142 rs7893	2
AT.AT	13	86974857	86974869	6.5	ENSSSCT00000023265	CEP70	intron	0.02	rs791972279	7
AAAT.ATTT	13	87707094	87707113	5	.	.	intergenic	0.156	069714 rs7913	2
AC.GT	13	87750073	87750091	9.5	.	.	intergenic	0.429	.	3
AC.GT	13	88016142	88016158	8.5	.	.	intergenic	0.227	rs698980167	2
AAGG.CCTT	13	88424832	88424858	6.75	.	.	intergenic	0.005	240880 rs7931	2
AC.GT	13	88484285	88484309	12.5	.	.	intergenic	0.101	350340 rs7917	2
AAAC.GTTT	13	88495417	88495430	3.5	.	.	intergenic	0.022	rs791396297	2
AG.CT	13	88531723	88531738	8	.	.	intergenic	-0.057	rs787116124	2
AAAC.GTTT	13	88569210	88569228	4.75	.	.	intergenic	0.05	428928 rs7864	3
AC.GT	13	88660362	88660373	6	.	.	intergenic	0.232	.	4
AATG.CATT	13	88788458	88788486	7.25	.	.	intergenic	-0.015	rs786885058	2
AAAC.GTTT	13	89024381	89024399	4.75	.	.	intergenic	0.444	rs790328009	2
AAAAC.GTTTT	13	89077381	89077396	3.2	.	.	intergenic	0.098	rs790746502	2
AAT.ATT	13	89166103	89166118	5.33333	ENSSSCT00000012771	CLSTN2	intron	.	.	2
AAAC.GTTT	13	89342727	89342741	3.75	.	.	intergenic	.	rs790070956	2
AAAC.GTTT	13	89402502	89402516	3.75	ENSSSCT00000012773	TRIM42	intron	0.089	rs787502755	2
AAAAG.CTTTT	13	89553956	89553991	7.2	.	.	intergenic	.	rs788806531	2
AGC.GCT	13	89565530	89565545	5.33333	.	.	intergenic	-0.102	rs793396417	2
AG.CT	13	89616104	89616114	5.5	ENSSSCT00000030064	SLC25A36	intron	-0.001	.	2
AG.CT	13	89616104	89616114	5.5	ENSSSCT00000012774	SLC25A36	intron	-0.001	.	2
AG.CT	13	89616104	89616114	5.5	ENSSSCT00000021586	snoU13	promoter	-0.001	.	2
AAAAC.GTTTT	13	89685931	89685956	5.2	.	.	intergenic	.	rs787717808	2
ATCC.GGAT	13	89800614	89800642	7.25	.	.	intergenic	0.141	227191 rs7936	3
AAAC.GTTT	13	89932661	89932684	6	.	.	intergenic	0.144	216734 rs7883	2
AAC.GTT	13	89968783	89968804	7.33333	.	.	intergenic	0.681	235713 rs7868	2
AG.CT	13	90035741	90035758	9	.	.	intergenic	0.481	.	2
AG.CT	13	90308822	90308834	6.5	.	.	intergenic	-0.018	rs793498384	3
AAAC.GTTT	13	90361168	90361191	6	.	.	intergenic	.	rs789950470	2
AAC.GTT	13	90371830	90371870	13.6667	.	.	intergenic	0.239	rs793888058	4
AC.GT	13	90499600	90499611	6	ENSSSCT00000012782	.	intron	.	rs789769223	2
ACAT.ATGT	13	90660474	90660489	4	.	.	intergenic	.	.	4
AT.AT	13	90686315	90686324	5	.	.	intergenic	0.933	rs791899573	3
AAAT.ATTT	13	90733627	90733641	3.75	.	.	intergenic	-0.001	rs789090883	2
AAAC.GTTT	13	90910803	90910822	5	ENSSSCT00000012784	XRN1	intron	1.559	rs792428307	2
AT.AT	13	91024055	91024072	9	ENSSSCT00000012786	ATR	intron	0.235	rs789334603	2
AAAC.GTTT	13	91168383	91168397	3.75	ENSSSCT00000012787	PLS1	intron	0.313	.	3
AAC.GTT	13	91263290	91263304	5	ENSSSCT00000012789	TRPC1	intron	.	rs788175362	2
ATC.GAT	13	91361588	91361602	5	ENSSSCT00000012791	PCOLCE2	intron	.	rs790276611	2
AT.AT	13	91408276	91408286	5.5	.	.	intergenic	0.122	rs790799013	2
AAAC.GTTT	13	91476357	91476375	4.75	.	.	intergenic	0.141	rs695024171	2
AC.GT	13	91645543	91645584	21	.	.	intergenic	-0.02	rs792419344	2
AC.GT	13	91651231	91651250	10	.	.	intergenic	-0.052	.	2
AAAT.ATTT	13	91779415	91779433	4.75	ENSSSCT00000024870	.	intron	0.071	.	2
ATC.GAT	13	91950711	91950731	7	ENSSSCT00000024870	.	intron	0.323	rs789042023	2
ACAGT.ACTGT	13	92077128	92077159	5.33333	ENSSSCT00000012794	.	intron	0.237	rs787637315	2
AC.GT	13	92202203	92202217	7.5	ENSSSCT00000012794	.	intron	0.116	rs787506942	2
AAAC.GTTT	13	92232917	92232931	3.75	ENSSSCT00000012794	.	intron	0.073	rs788529157	2
AAT.ATT	13	92439574	92439590	5.66667	.	.	intergenic	.	rs703710880	3
AAAC.GTTT	13	92669253	92669271	4.75	.	.	intergenic	0.201	rs790584829	2
AC.GT	13	92831955	92831965	5.5	.	.	intergenic	-0.085	.	2
AG.CT	13	93063159	93063169	5.5	.	.	intergenic	-0.58	.	3
AC.GT	13	93287819	93287845	13.5	.	.	intergenic	-0.006	rs791429930	3
AAG.CTT	13	93392922	93392934	4.33333	.	.	intergenic	-0.153	rs788978063	2
AAAC.GTTT	13	93427507	93427525	4.75	.	.	intergenic	-0.098	rs789601794	2
AAC.GTT	13	93491659	93491683	8.33333	.	.	intergenic	0.001	rs787392972	2
AT.AT	13	93516600	93516609	5	.	.	intergenic	.	rs786920882	2
AC.GT	13	93668819	93668829	5.5	.	.	intergenic	.	rs790468821	2
AC.GT	13	93686785	93686821	18.5	.	.	intergenic	0.032	.	3

AAG.CTT	13	94223493	94223507	5	ENSSSCT00000012797	PLOD2	intron	-0.004	rs793366082	2
AT.AT	13	94500039	94500053	7.5	.	.	intergenic	0.011	.	4
AC.GT	13	94501685	94501704	10	.	.	intergenic	-0.062	.	5
AT.AT	13	94622522	94622540	9.5	.	.	intergenic	-0.097	rs792811636	2
AC.GT	13	94678460	94678476	8.5	.	.	intergenic	0.07	.	3
ATC.GAT	13	94692366	94692382	5.66667	.	.	intergenic	.	rs787642696	2
ATC.GAT	13	95321166	95321186	7	.	.	intergenic	-0.03	711841 rs7892	2
AC.GT	13	95589370	95589382	6.5	.	.	intergenic	0.707	rs792650624	3
AAAC.GTTT	13	95657831	95657855	6.25	.	.	intergenic	0.056	118219 rs7912	2
AAAC.GTTT	13	95708304	95708324	5.25	.	.	intergenic	-0.091	rs792283550	2
AAAT.ATTT	13	96043730	96043749	5	.	.	intergenic	-0.262	rs793070054	2
AAAC.GTTT	13	96304155	96304189	8.75	.	.	intergenic	.	258533 rs7880	2
AAAC.GTTT	13	96409003	96409017	3.75	.	.	intergenic	-0.02	rs787486474	2
AC.GT	13	96662275	96662298	12	.	.	intergenic	0.381	393334 rs7936	2
AAAAC.GTTTT	13	96861412	96861448	7.4	.	.	intergenic	0.264	795703 rs7886	2
AT.AT	13	96960641	96960656	8	ENSSSCT00000035119	AGTR1	intron	0.276	rs791226358	2
AT.AT	13	96960641	96960656	8	ENSSSCT00000034453	AGTR1	intron	0.276	rs791226358	2
AC.GT	13	98114656	98114672	8.5	ENSSSCT00000022374	RNF13	intron	0.179	.	2
AAAAG.CTTTT	13	98171159	98171187	5.8	ENSSSCT00000022374	RNF13	intron	.	rs789672418	2
AAT.ATT	13	98247217	98247232	5.33333	.	.	intergenic	0.095	rs791052462	2
AGG.CCT	13	98278197	98278211	5	.	.	intergenic	0.027	.	2
AC.GT	13	98597788	98597804	8.5	.	.	intergenic	0.11	rs792157493	3
AT.AT	13	98689160	98689170	5.5	.	.	intergenic	0.334	rs789232657	4
AAAAC.GTTTT	13	98731433	98731460	5.6	ENSSSCT00000030003	TSC22D2	intron	0.253	rs788721684	2
AAAAC.GTTTT	13	98731433	98731460	5.6	ENSSSCT00000012818	TSC22D2	intron	0.253	rs788721684	2
AAC.GTT	13	98943235	98943252	6	.	.	intergenic	.	rs789782396	2
AAGC.GCTT	13	99139451	99139474	6	.	.	intergenic	0.147	783909 rs7932	3
AATG.CATT	13	99317028	99317044	4.25	.	.	intergenic	0.27	rs786637988	2
AAC.GTT	13	99663649	99663668	6.66667	.	.	intergenic	-0.125	rs792463131	2
ACAT.ATGT	13	99711413	99711444	8	.	.	intergenic	-0.218	772259 rs7882	2
AC.GT	13	99720108	99720123	8	.	.	intergenic	-0.026	313252 rs7867	2
AAAAG.CTTTT	13	99864451	99864469	3.8	ENSSSCT00000012827	IGSF10	intron	.	rs790409434	2
AC.GT	13	99931117	99931128	6	.	.	intergenic	.	.	4
AAAT.ATTT	13	99944830	99944843	3.5	.	.	intergenic	0.059	rs790129980	2
AAAT.ATTT	13	99989335	99989350	4	.	.	intergenic	.	rs793820697	3
AAT.ATT	13	100020177	100020190	4.66667	.	.	intergenic	0.089	rs791736690	2
AT.AT	13	100171414	100171424	5.5	.	.	intergenic	.	rs792904633	2
AC.GT	13	100197935	100197947	6.5	.	.	intergenic	-0.013	.	2
AC.GT	13	100211900	100211913	7	.	.	intergenic	.	rs786942442	2
AAAAC.GTTTT	13	100246331	100246359	5.8	.	.	intergenic	.	786858 rs7867	2
AAG.CTT	13	100501796	100501812	5.66667	.	.	intergenic	0.108	rs788028937	2
AAAAC.GTTTT	13	100644662	100644688	5.4	ENSSSCT00000012830	MBNL1	intron	-0.027	069012 rs7925	2
AC.GT	13	100761073	100761092	10	.	.	intergenic	0.061	rs792853589	2
AAC.GTT	13	100968179	100968210	10.6667	.	.	intergenic	-0.275	rs791539892	2
AAAT.ATTT	13	101069996	101070016	5.25	.	.	intergenic	0.005	rs792614520	2
AG.CT	13	101092237	101092246	5	.	.	intergenic	0.096	.	3
AAAC.GTTT	13	101213987	101214009	5.75	.	.	intergenic	.	435571 rs7876	2
AAAC.GTTT	13	101282129	101282143	3.75	.	.	intergenic	.	.	2
AC.GT	13	101336308	101336327	10	ENSSSCT00000012832	RAP2B	intron	.	rs791796784	2
AT.AT	13	101688092	101688103	6	.	.	intergenic	0.025	rs788142846	2
AAAAC.GTTTT	13	101723363	101723390	5.6	.	.	intergenic	-0.038	rs790340187	2
AAG.CTT	13	101930451	101930465	5	.	.	intergenic	.	rs789186517	2
AAAC.GTTT	13	102101672	102101698	6.75	.	.	intergenic	.	rs792934231	2
AAAC.GTTT	13	102374342	102374370	7.25	ENSSSCT00000027576	DHX36	intron	-0.055	rs786751763	2
AAAC.GTTT	13	102374342	102374370	7.25	ENSSSCT00000023601	DHX36	intron	-0.055	rs786751763	2
AAAAC.GTTTT	13	102493612	102493634	4.6	.	.	intergenic	0.307	rs793657473	2
AAAAT.ATTTTT	13	102553299	102553318	3.33333	.	.	intergenic	0.263	rs787460942	2
AAAT.ATTT	13	102661899	102661917	4.75	.	.	intergenic	.	rs792694393	4
AAAAC.GTTTT	13	102669466	102669483	3.6	.	.	intergenic	.	.	2
AAC.GTT	13	102704558	102704578	7	.	.	intergenic	0.144	rs793010317	2
AC.GT	13	102718545	102718555	5.5	.	.	intergenic	0.016	rs786625468	2
AGGG.CCCT	13	102826124	102826140	4.25	.	.	intergenic	.	rs792443788	2
AT.AT	13	102906032	102906042	5.5	.	.	intergenic	-0.219	rs793305148	2
AT.AT	13	102963974	102963987	7	.	.	intergenic	0.147	.	4
AAAT.ATTT	13	103010913	103010938	6.5	.	.	intergenic	0.263	rs789090036	2
AAAC.GTTT	13	103287420	103287439	5	.	.	intergenic	0.417	rs792138217	2
AAAAC.GTTTT	13	103395404	103395431	4.66667	.	.	intergenic	0.261	rs792980948	2
AG.CT	13	103561467	103561478	6	.	.	intergenic	0.001	rs792563150	3
AATT.AATT	13	103741311	103741329	4.75	ENSSSCT00000027200	SLC33A1	intron	0.197	rs790585426	2
AAAT.ATTT	13	103927942	103927969	7	.	.	intergenic	0.251	rs789226357	4
AAAC.GTTT	13	104115063	104115081	4.75	.	.	intergenic	0.58	rs791098528	2
AAAC.GTTT	13	104210745	104210769	6.25	.	.	intergenic	.	rs792656031	2
AT.AT	13	104314401	104314412	6	ENSSSCT00000027152	.	intron	0.018	rs788011014	2
AAAC.GTTT	13	104748272	104748292	5.25	.	.	intergenic	-0.006	rs790809350	2
AAAC.GTTT	13	104776291	104776304	3.5	.	.	intergenic	0.038	rs787171606	2
AG.CT	13	104949441	104949452	6	ENSSSCT00000024993	.	intron	.	rs791614510	2
AG.CT	13	105290920	105290930	5.5	ENSSSCT00000012836	VEPH1	intron	0.247	.	4
AT.AT	13	105303909	105303924	8	ENSSSCT00000012836	VEPH1	intron	0.051	.	2
AAAT.ATTT	13	105353537	105353560	6	ENSSSCT00000012836	VEPH1	intron	.	rs787928575	2
AAAAC.GTTTT	13	106083206	106083233	5.6	ENSSSCT00000025053	SHOX2	intron	0.675	rs792821710	2
AC.GT	13	106394767	106394785	9.5	ENSSSCT00000030951	.	intron	0.465	rs790114922	4
AT.AT	13	106751717	106751728	6	.	.	intergenic	.	rs791720596	2
AATG.CATT	13	106840502	106840525	6	.	.	intergenic	-0.033	rs791213632	2
AG.CT	13	107102539	107102555	8.5	.	.	intergenic	.	rs790112905	3

AAAG.CTTT	13	107121143	107121165	5.75	.	.	intergenic	0.237	rs791464063	2
AG.CT	13	107356280	107356290	5.5	.	.	intergenic	-0.139	.	4
AAAC.GTTT	13	107399680	107399704	6.25	.	.	intergenic	.	rs787084423	2
AAAC.GTTT	13	107498666	107498681	4	.	.	intergenic	-0.094	241643 rs7929	2
AC.GT	13	107536938	107536969	16	.	.	intergenic	-0.107	.	2
AT.AT	13	107756509	107756519	5.5	.	.	intergenic	-0.054	.	2
AAAC.GTTT	13	107820545	107820562	4.5	.	.	intergenic	0.109	rs788623457	2
AAAC.GTTT	13	107955136	107955152	4.25	ENSSSCT00000012839	QCJ-SCHIF	intron	-0.134	.	2
AG.CT	13	107997559	107997569	5.5	.	.	intergenic	.	547214 rs7915	2
AAAT.ATTT	13	108011306	108011323	4.5	.	.	intergenic	0.134	932429 rs7887	2
AAAC.GTTT	13	108017582	108017603	5.5	.	.	intergenic	0.097	rs793807983	2
AAC.GTT	13	108087899	108087912	4.66667	ENSSSCT00000032569	.	intron	-0.07	rs787642274	2
AT.AT	13	108684956	108684965	5	.	.	intergenic	0.174	rs788397120	2
AAT.ATT	13	109179431	109179449	6.33333	.	.	intergenic	.	.	2
AAAT.ATTT	13	109207148	109207167	5	.	.	intergenic	.	rs792303534	2
AAAC.GTTT	13	109250690	109250711	5.5	ENSSSCT00000024911	NMD3	intron	.	rs788276520	2
AT.AT	13	109422859	109422879	10.5	.	.	intergenic	0.078	rs786688734	2
AAAC.GTTT	13	109485440	109485454	3.75	.	.	intergenic	0.256	rs790814299	2
AG.CT	13	109525191	109525203	6.5	.	.	intergenic	.	rs791394089	4
AAAC.GTTT	13	109593400	109593424	6.25	.	.	intergenic	0.19	rs792571797	2
AAAG.CTTT	13	109598521	109598544	6	.	.	intergenic	0.343	rs786911075	2
AT.AT	13	109751279	109751288	5	.	.	intergenic	0.684	rs789324854	3
AAAAC.GTTTT	13	110063107	110063131	5	.	.	intergenic	0.188	rs792269303	2
AT.AT	13	110105536	110105551	8	.	.	intergenic	0.591	506736 rs7877	2
AC.GT	13	110780330	110780349	10	.	.	intergenic	.	rs792036269	2
AAAT.ATTT	13	110848577	110848597	5.25	.	.	intergenic	.	130273 rs7919	2
AT.AT	13	111389934	111389950	8.5	.	.	intergenic	.	rs792574996	2
AT.AT	13	111510711	111510723	6.5	.	.	intergenic	0.055	584116 rs7893	2
AC.GT	13	111584310	111584326	8.5	.	.	intergenic	-0.042	.	3
AG.CT	13	111937897	111937921	12.5	.	.	intergenic	.	742434 rs7895	5
AG.CT	13	111986963	111986973	5.5	.	.	intergenic	.	.	2
AAC.GTT	13	112018107	112018121	5	.	.	intergenic	.	.	4
AATC.GATT	13	112080497	112080519	5.75	.	.	intergenic	0.104	rs787030142	2
AAAC.GTTT	13	112112328	112112340	3.25	.	.	intergenic	-0.042	.	2
AAC.GTT	13	112348479	112348504	8.66667	.	.	intergenic	.	rs792642979	2
AT.AT	13	112473487	112473497	5.5	.	.	intergenic	-0.139	rs786973025	2
AAAC.GTTT	13	112483537	112483567	7.75	.	.	intergenic	.	rs786664261	2
AC.GT	13	112514175	112514189	7.5	.	.	intergenic	-0.026	290704 rs7879	2
AT.AT	13	112679362	112679374	6.5	.	.	intergenic	0.016	.	2
AC.GT	13	112762236	112762246	5.5	ENSSSCT00000032326	.	intron	.	.	3
AC.GT	13	112762236	112762246	5.5	ENSSSCT00000012846	SI	intron	.	.	3
AAAT.ATTT	13	112791651	112791662	3	ENSSSCT00000012846	SI	intron	-0.411	rs786953802	2
AAAAAT.ATTTTT	13	112800426	112800453	4.66667	ENSSSCT00000012846	SI	intron	0.541	039880 rs7924	2
AAAAT.ATTTT	13	113009972	113009993	4.4	.	.	intergenic	-0.139	746755 rs7896	2
AG.CT	13	113057640	113057652	6.5	.	.	intergenic	-0.034	rs790512242	2
AC.GT	13	113096583	113096597	7.5	.	.	intergenic	0.056	rs793189608	2
AT.AT	13	113117345	113117376	16	.	.	intergenic	.	rs787246812	2
AT.AT	13	113246286	113246300	7.5	.	.	intergenic	-0.116	350294 rs7895	2
AC.GT	13	113250244	113250254	5.5	.	.	intergenic	0.06	rs788306182	3
AT.AT	13	113748731	113748741	5.5	.	.	intergenic	-0.155	.	2
AG.CT	13	114048040	114048051	6	.	.	intergenic	-0.216	rs708232354	2
ACTAT.ATAGT	13	114465225	114465251	5.4	.	.	intergenic	-0.001	rs793144495	2
AT.AT	13	114892571	114892584	7	ENSSSCT00000012848	SERPINI2	intron	-0.114	571214 rs7932	2
AAAC.GTTT	13	114904949	114904963	3.75	.	.	intergenic	0.542	rs790929373	2
AC.GT	13	115066551	115066567	8.5	.	.	intergenic	0.122	rs789443203	2
AAAAT.ATTTT	13	115212633	115212655	4.6	.	.	intergenic	0.252	.	2
AT.AT	13	115307415	115307425	5.5	.	.	intergenic	.	rs792665880	3
AAT.ATT	13	115761554	115761571	6	.	.	intergenic	0.062	rs786403961	2
AG.CT	13	116170768	116170785	9	.	.	intergenic	-0.12	rs792756598	2
AT.AT	13	116211173	116211182	5	.	.	intergenic	0.021	rs791701574	2
AAAAC.GTTTT	13	116250305	116250328	4.8	.	.	intergenic	-0.052	rs787147634	2
ACAT.ATGT	13	116370713	116370727	3.75	.	.	intergenic	-0.083	rs792515796	2
AAAC.GTTT	13	116663391	116663408	4.5	.	.	intergenic	-0.056	rs791072884	2
AT.AT	13	116986260	116986279	10	.	.	intergenic	1.03	rs792186808	2
AAAC.GTTT	13	117486241	117486259	4.75	.	.	intergenic	.	291156 rs7938	2
AAC.GTT	13	117577815	117577828	4.66667	ENSSSCT00000027451	PHC3	intron	0.208	rs786912536	2
AAC.GTT	13	117577815	117577828	4.66667	ENSSSCT00000029926	PHC3	intron	0.208	rs786912536	2
AC.GT	13	117581998	117582007	5	ENSSSCT00000027451	PHC3	intron	0.269	.	2
AC.GT	13	117581998	117582007	5	ENSSSCT00000029926	PHC3	intron	0.269	.	2
AAATG.CATTT	13	117695069	117695107	7.8	ENSSSCT00000012856	PKC	promoter	0.333	.	2
AC.GT	13	117813639	117813649	5.5	.	.	intergenic	0.094	rs790356292	2
AAAC.GTTT	13	118425912	118425939	7	.	.	intergenic	0.356	rs792738519	2
AC.GT	13	118570920	118570931	6	.	.	intergenic	0.22	.	2
AATG.CATT	13	118583925	118583946	5.5	.	.	intergenic	0.173	rs696016252	2
AAAAG.CTTTT	13	118935359	118935376	3.6	.	.	intergenic	0.058	rs793627670	2
AAAAC.GTTTT	13	119033297	119033319	4.6	.	.	intergenic	0.093	rs792358020	2
AC.GT	13	119059799	119059813	7.5	.	.	intergenic	.	rs787642771	2
AC.GT	13	119177905	119177916	6	ENSSSCT00000012861	PLD1	intron	.	364904 rs7872	2
AAAAAT.ATTTTT	13	119405015	119405032	3	ENSSSCT00000012862	TMEM212	intron	-0.033	rs790645566	2
AAAC.GTTT	13	119876823	119876846	6	ENSSSCT00000032717	TNFSF10	intron	0.071	393336 rs7886	2
AAAC.GTTT	13	119876823	119876846	6	ENSSSCT00000025711	TNFSF10	intron	0.071	393336 rs7886	2
AAC.GTT	13	119878516	119878532	5.66667	ENSSSCT00000032717	TNFSF10	intron	0.132	rs793024408	2
AAC.GTT	13	119878516	119878532	5.66667	ENSSSCT00000025711	TNFSF10	intron	0.132	rs793024408	2
AAAT.ATTT	13	119942603	119942619	4.25	.	.	intergenic	.	rs788195025	2

AT.AT	13	119975726	119975746	10.5	.	.	intergenic	.	.	2
AT.AT	13	120180940	120180949	5	.	.	intergenic	.	.	3
AAAC.GTTT	13	120261803	120261822	5	.	.	intergenic	.	598737 rs7897	3
AC.GT	13	120639395	120639429	17.5	.	.	intergenic	.	rs793813400	3
AC.GT	13	120686315	120686334	10	.	.	intergenic	-0.153	rs788684324	2
AG.CT	13	120784799	120784808	5	.	.	intergenic	-0.075	.	3
AG.CT	13	120818340	120818349	5	.	.	intergenic	0.028	.	3
AAT.ATT	13	121219370	121219395	8.66667	.	.	intergenic	-0.069	rs790738475	2
AT.AT	13	121295031	121295043	6.5	.	.	intergenic	.	076764 rs7863	2
ACC.GGT	13	121310641	121310654	4.66667	.	.	intergenic	0.01	.	2
AAAAAG.CTTTT	13	121341970	121341991	3.66667	.	.	intergenic	-0.272	rs792584036	2
AAAAAC.GTTTT	13	121497017	121497052	6	.	.	intergenic	0.012	305784 rs7905	2
AAC.GTT	13	121668771	121668793	7.66667	.	.	intergenic	0.695	rs786884213	2
AAAC.GTTT	13	121697134	121697152	4.75	.	.	intergenic	0.141	rs790778976	5
AAC.GTT	13	121883546	121883564	6.33333	.	.	intergenic	0.121	rs787038629	2
AC.GT	13	121896331	121896340	5	.	.	intergenic	0.122	rs791388723	2
AG.CT	13	122027241	122027254	7	.	.	intergenic	.	rs792172764	2
AAAC.GTTT	13	122520749	122520775	6.75	ENSSSCT00000026159	NAALADL2	intron	0.049	rs787512344	2
AT.AT	13	122792084	122792094	5.5	.	.	intergenic	0.027	rs786807495	2
AG.CT	13	122822063	122822075	6.5	.	.	intergenic	0.204	rs792434871	2
AATC.GATT	13	122853256	122853285	7.5	.	.	intergenic	0.213	rs791403288	2
AT.AT	13	122854733	122854746	7	.	.	intergenic	-0.21	rs787327342	3
AAAC.GTTT	13	123114005	123114027	5.75	.	.	intergenic	0.11	rs792705176	2
AAC.GTT	13	123278462	123278489	9.33333	.	.	intergenic	.	rs787130627	4
AAAAAG.CTTTT	13	123285637	123285654	3	.	.	intergenic	-0.002	rs790779402	2
AAC.GTT	13	123352048	123352064	5.66667	.	.	intergenic	.	rs792221827	2
AAAG.CTTT	13	123649881	123649893	3.25	.	.	intergenic	-0.035	rs789359651	4
AC.GT	13	123911817	123911829	6.5	.	.	intergenic	.	rs788518138	4
AAAC.GTTT	13	123927818	123927842	6.25	.	.	intergenic	.	rs790713979	2
AGC.GCT	13	124253148	124253162	5	.	.	intergenic	-0.193	rs790396244	2
AG.CT	13	124504948	124504957	5	.	.	intergenic	-0.005	rs786669639	2
AT.AT	13	124521424	124521435	6	.	.	intergenic	0.023	rs789120604	2
AAAC.GTTT	13	124660839	124660877	9.75	.	.	intergenic	0.095	rs793568017	3
AAAC.GTTT	13	125133918	125133936	4.75	.	.	intergenic	0.141	rs790723897	2
AAAAC.GTTTT	13	125171216	125171249	6.8	.	.	intergenic	.	774173 rs7904	3
AC.GT	13	125366980	125366994	7.5	.	.	intergenic	0.132	500109 rs7869	4
AAC.GTT	13	125524022	125524035	4.66667	.	.	intergenic	.	.	5
AG.CT	13	125904034	125904044	5.5	.	.	intergenic	.	.	3
AT.AT	13	125924036	125924048	6.5	.	.	intergenic	0.037	rs793428464	2
AAC.GTT	13	125996320	125996333	4.66667	.	.	intergenic	0.278	rs788469901	2
AT.AT	13	126007610	126007626	8.5	.	.	intergenic	-0.162	351534 rs7905	3
AAAC.GTTT	13	126206451	126206468	4.5	.	.	intergenic	0.089	rs791468118	2
ACACAT.ATGTG	13	126241107	126241148	7	.	.	intergenic	0.191	rs787176727	2
AT.AT	13	126742251	126742260	5	ENSSSCT00000012874	MRPL47	intron	0.194	rs789124362	2
AT.AT	13	126930210	126930220	5.5	.	.	intergenic	1.591	rs789657565	2
AAG.CTT	13	126963303	126963325	7.66667	ENSSSCT00000012877	PEX5L	intron	.	rs787535207	2
AAG.CTT	13	127163765	127163779	5	.	.	intergenic	0.294	.	2
AAAT.ATTT	13	127213121	127213148	7	.	.	intergenic	.	.	2
AATC.GATT	13	127335764	127335783	5	.	.	intergenic	0.25	rs788930436	2
AAAATC.GATTT	13	127461748	127461767	3.33333	.	.	intergenic	0.285	rs788240153	2
AAAT.ATTT	13	127479934	127479948	3.75	.	.	intergenic	-0.171	rs788574207	2
AT.AT	13	127521978	127521987	5	.	.	intergenic	0.153	rs792663588	2
AAC.GTT	13	127652543	127652571	9.66667	.	.	intergenic	0.356	rs790112653	4
AC.GT	13	127822043	127822059	8.5	ENSSSCT00000012878	TTC14	intron	-0.151	rs787291697	2
AAAT.ATTT	13	127827950	127827979	7.5	.	.	intergenic	0.297	rs791880697	2
AAAT.ATTT	13	128102344	128102358	3.75	ENSSSCT00000027605	FXR1	intron	0.12	rs792271396	2
AAAT.ATTT	13	128102344	128102358	3.75	ENSSSCT00000027021	FXR1	intron	0.12	rs792271396	2
AAAT.ATTT	13	128102344	128102358	3.75	ENSSSCT00000012881	FXR1	intron	0.12	rs792271396	2
AC.GT	13	129037773	129037788	8	.	.	intergenic	0.15	.	4
AAACC.GGTTT	13	129063729	129063766	7.6	.	.	intergenic	0.298	rs791808672	2
AT.AT	13	129486712	129486724	6.5	.	.	intergenic	0.373	rs790052494	6
AT.AT	13	129703643	129703663	10.5	.	.	intergenic	0.221	rs786718245	2
AC.GT	13	129851225	129851235	5.5	.	.	intergenic	.	.	2
AAAT.ATTT	13	130321916	130321944	7.25	.	.	intergenic	.	.	2
AAT.ATT	13	130472086	130472103	6	.	.	intergenic	0.3	rs792273139	2
AAGG.CCTT	13	130554696	130554730	8.75	.	.	intergenic	.	rs793175875	2
AAAAT.ATTTT	13	130966135	130966157	4.6	.	.	intergenic	.	rs789731998	2
AAAAT.ATTTT	13	131034758	131034792	7	ENSSSCT00000012887	KLHL24	intron	.	.	2
AAC.GTT	13	131239859	131239874	5.33333	ENSSSCT00000012891	PARL	intron	.	.	5
AC.GT	13	131279363	131279377	7.5	ENSSSCT00000012892	.	intron	0.209	rs790657095	2
AC.GT	13	131299173	131299185	6.5	ENSSSCT00000012893	.	promoter	-0.11	248674 rs7927	2
AAAAC.GTTTT	13	131555759	131555791	6.6	.	.	intergenic	.	940599 rs7921	2
AC.GT	13	131561974	131561984	5.5	.	.	intergenic	.	rs788915122	2
AC.GT	13	131632998	131633010	6.5	ENSSSCT00000024405	PSMD2	intron	2.544	rs792578807	2
AG.CT	13	131798573	131798589	8.5	.	.	intergenic	.	rs788012927	2
AC.GT	13	131961865	131961887	11.5	ENSSSCT00000023583	EPHB3	intron	0.222	.	2
AC.GT	13	132269857	132269866	5	ENSSSCT00000012900	.	intron	0.121	rs786893416	2
AC.GT	13	132282470	132282481	6	ENSSSCT00000012900	.	intron	0.318	rs787021117	3
AATG.CATT	13	132319820	132319850	7.75	ENSSSCT00000012900	.	intron	1.366	rs792621711	2
AATG.CATT	13	132319820	132319850	7.75	ENSSSCT00000024055	.	intron	1.366	rs792621711	2
AAC.GTT	13	132598580	132598594	5	.	.	intergenic	.	.	3
AAAT.ATTT	13	132681196	132681212	4.25	ENSSSCT00000024873	EHHADH	intron	0.351	rs787772567	2
AAC.GTT	13	133161636	133161651	5.33333	.	.	intergenic	.	rs786338350	2
AC.GT	13	133188493	133188502	5	.	.	intergenic	-0.192	rs787311838	2

AC.GT	13	133459799	133459828	15	ENSSSCT00000024619	DGKG	intron	-0.051	.	2
AG.CT	13	133734300	133734316	8.5	.	.	intergenic	.	rs791765889	2
AC.GT	13	133743610	133743621	6	.	.	intergenic	-0.229	rs792006591	3
AAAC.GTTT	13	134015870	134015892	5.75	.	.	intergenic	-0.035	rs789797566	2
AAAAC.GTTTT	13	134144433	134144452	4	.	.	intergenic	-0.012	rs793858143	2
AATG.CATT	13	134150450	134150480	7.75	.	.	intergenic	0.121	430446 rs7916	2
AT.AT	13	134394341	134394350	5	ENSSSCT00000012917	RTP1	promoter	-0.016	rs786672394	3
AAAAG.CTTTT	13	134409793	134409821	4.83333	.	.	intergenic	.	rs788982483	2
AAC.GTT	13	134456468	134456483	5.33333	.	.	intergenic	.	rs789654620	2
AC.GT	13	134612007	134612016	5	.	.	intergenic	0.058	.	2
AAAC.GTTT	13	134988391	134988408	4.5	ENSSSCT00000026129	.	intron	0.037	rs786337215	2
AAAAC.GTTTT	13	135029061	135029087	5.4	ENSSSCT00000026129	.	intron	.	589136 rs7888	2
AG.CT	13	135127252	135127264	6.5	ENSSSCT00000026129	.	intron	-0.145	rs698227816	3
AT.AT	13	135198265	135198276	6	ENSSSCT00000026129	.	intron	-0.018	rs788135865	2
AG.CT	13	135769869	135769880	6	.	.	intergenic	0.081	rs790652703	3
AG.CT	13	135784246	135784271	13	.	.	intergenic	0.077	rs789471454	2
AC.GT	13	135830244	135830257	7	.	.	intergenic	0.334	.	3
AAAC.GTTT	13	135927635	135927663	7.25	.	.	intergenic	-0.034	rs787611826	4
AT.AT	13	136354209	136354224	8	.	.	intergenic	0.092	.	4
AT.AT	13	136360218	136360227	5	.	.	intergenic	0.85	rs787012219	2
AT.AT	13	136361212	136361227	8	.	.	intergenic	.	rs789903405	2
AAAT.ATTT	13	136373033	136373051	4.75	.	.	intergenic	-0.008	.	2
AAAT.ATTT	13	136756941	136756959	4.75	ENSSSCT00000012925	LEPREL1	intron	.	rs788834472	2
AG.CT	13	136783404	136783416	6.5	ENSSSCT00000012925	LEPREL1	intron	-0.274	rs789974312	2
AC.GT	13	136826938	136826952	7.5	ENSSSCT00000012925	LEPREL1	intron	-0.153	rs791568582	2
AG.CT	13	136864967	136864977	5.5	.	.	intergenic	-0.099	rs792998124	2
AG.CT	13	136900863	136900875	6.5	.	.	intergenic	-0.206	759088 rs7875	3
AAAC.GTTT	13	137527098	137527128	7.75	.	.	intergenic	-0.056	rs787917532	2
AAG.CTT	13	137626597	137626614	6	ENSSSCT00000031033	GMNC	intron	0.467	rs793620339	2
AAG.CTT	13	137626597	137626614	6	ENSSSCT00000027738	GMNC	intron	0.467	rs793620339	2
AAAAT.ATTTT	13	137686253	137686272	4	.	.	intergenic	.	rs791898150	2
AT.AT	13	137714086	137714095	5	.	.	intergenic	.	.	2
AG.CT	13	137771377	137771388	6	.	.	intergenic	.	357577 rs7876	2
AAAT.ATTT	13	137867229	137867248	5	.	.	intergenic	.	rs789619657	2
AC.GT	13	138021424	138021443	10	.	.	intergenic	0.344	rs787678322	5
AAAAC.GTTTT	13	138047026	138047046	4.2	ENSSSCT00000012929	CCDC50	intron	.	.	2
AC.GT	13	138292995	138293012	9	.	.	intergenic	0.308	rs787517496	2
AT.AT	13	138360831	138360841	5.5	.	.	intergenic	-0.185	rs709617218	2
AC.GT	13	138528131	138528142	6	.	.	intergenic	.	rs791816415	4
AT.AT	13	138545691	138545702	6	.	.	intergenic	0.032	.	4
AAAT.ATTT	13	138589260	138589287	7	.	.	intergenic	0.024	rs792402764	2
AG.CT	13	138633750	138633767	9	.	.	intergenic	0.014	rs793518551	2
AC.GT	13	138778830	138778855	13	ENSSSCT00000023418	FGF12	intron	.	268595 rs7865	3
AT.AT	13	138938693	138938708	8	ENSSSCT00000023418	FGF12	intron	-0.127	rs786606950	3
AG.CT	13	139009543	139009554	6	.	.	intergenic	0.119	rs789916304	2
AAAAT.ATTTT	13	139010395	139010414	4	.	.	intergenic	0.073	rs788600753	2
AT.AT	13	139134083	139134092	5	.	.	intergenic	0.051	rs790934105	2
ACAG.CTGT	13	139155024	139155037	3.5	.	.	intergenic	0.027	rs789854648	2
ATC.GAT	13	139687826	139687846	7	.	.	intergenic	.	rs792304406	3
AG.CT	13	139722928	139722941	7	.	.	intergenic	-0.009	rs792628484	2
AG.CT	13	139724373	139724384	6	.	.	intergenic	0.043	.	2
AAC.GTT	13	139732841	139732860	6.66667	.	.	intergenic	0.06	rs786803869	3
AT.AT	13	139893792	139893823	16	ENSSSCT00000031000	ATP13A5	intron	0.404	.	4
AAAT.ATTT	13	139903906	139903923	4.5	ENSSSCT00000031000	ATP13A5	intron	-0.001	rs791164603	2
AC.GT	13	140210010	140210028	9.5	ENSSSCT00000012934	OPA1	intron	0.223	528844 rs7899	3
AAAC.GTTT	13	140215587	140215609	5.75	ENSSSCT00000012934	OPA1	intron	0.073	rs789471023	2
AAGG.CCTT	13	140223436	140223457	5.5	ENSSSCT00000012934	OPA1	intron	0.029	910921 rs7872	2
AAAT.ATTT	13	140536540	140536556	4.25	.	.	intergenic	0.16	rs793068272	2
AAAAC.GTTTT	13	140609956	140609984	5.8	.	.	intergenic	0.192	rs793667527	2
AC.GT	13	140726174	140726185	6	.	.	intergenic	1.113	rs789724059	2
AC.GT	13	140873369	140873386	9	.	.	intergenic	-0.171	rs790801237	2
AAAC.GTTT	13	140994605	140994627	5.75	ENSSSCT00000012937	ATP13A3	intron	0.129	rs788348379	2
AAAAT.ATTTT	13	141144459	141144476	3.6	.	.	intergenic	.	975044 rs7879	2
AC.GT	13	141185431	141185443	6.5	ENSSSCT00000012938	TMEM44	intron	0.123	rs786784052	2
AGAGG.CCTCT	13	141271842	141271865	4.8	.	.	intergenic	0.52	602734 rs7886	2
AAAC.GTTT	13	141279582	141279600	4.75	.	.	intergenic	0.217	rs787324582	2
AT.AT	13	141304003	141304012	5	.	.	intergenic	.	rs791348176	2
AG.CT	13	141355537	141355549	6.5	.	.	intergenic	-0.132	rs787486498	2
AAAC.GTTT	13	141496653	141496676	6	.	.	intergenic	0.12	335428 rs7934	2
AAAAC.GTTTT	13	141644694	141644719	5.2	.	.	intergenic	.	rs790714713	2
AATG.CATT	13	141719591	141719621	7.75	.	.	intergenic	-0.147	650182 rs7928	2
ACAG.CTGT	13	142058289	142058302	3.5	.	.	intergenic	0.248	rs789340117	2
AAAAT.ATTTT	13	142109883	142109907	5	.	.	intergenic	0.554	rs790353432	2
AG.CT	13	142166196	142166210	7.5	.	.	intergenic	0.142	rs789232144	2
AAAAT.ATTTT	13	142178470	142178489	4	.	.	intergenic	0.003	rs787425972	2
AC.GT	13	142192310	142192334	12.5	.	.	intergenic	-0.18	rs788991606	2
AG.CT	13	142312085	142312099	7.5	.	.	intergenic	0.089	rs786911351	2
AAC.GTT	13	142450287	142450303	5.66667	ENSSSCT00000034060	DLG1	intron	.	rs788547417	2
AAC.GTT	13	142450287	142450303	5.66667	ENSSSCT00000012945	DLG1	intron	.	rs788547417	2
AAC.GTT	13	142450287	142450303	5.66667	ENSSSCT00000033279	DLG1	intron	.	rs788547417	2
AAAC.GTTT	13	142692703	142692726	6	.	.	intergenic	.	rs791195127	3
AGG.CCT	13	142791558	142791574	5.66667	.	.	intergenic	.	rs792097994	2
AAC.GTT	13	142853788	142853803	5.33333	ENSSSCT00000012953	SENP5	intron	0.152	rs793863528	2
AC.GT	13	142930813	142930825	6.5	ENSSSCT00000012955	.	intron	0.041	477736 rs7938	2

AAT.ATT	13	143073994	143074008	5	ENSSSCT00000023388	CEP19	3'utr	2.103	rs787893637	3
AAAAC.GTTTT	13	143177138	143177155	3.6	.	.	intergenic	.	rs791771041	2
AC.GT	13	143627339	143627349	5.5	.	.	intergenic	0.25	rs699935854	4
AC.GT	13	143739728	143739741	7	.	.	intergenic	0.08	rs788174837	2
AC.GT	13	143836549	143836558	5	ENSSSCT00000012962	MUC4	intron	-0.164	.	4
AAAAC.GTTTT	13	143997771	143997796	5.2	.	.	intergenic	-0.087	374002 rs7898	2
AGGC.GCCT	13	144104670	144104692	5.75	ENSSSCT00000012966	LRCH3	intron	0.728	rs790765043	2
AAC.GTT	13	144175430	144175448	6.33333	ENSSSCT00000012968	IQCG	intron	.	rs791332749	2
AAAG.CTTT	13	144591312	144591327	4	.	.	intergenic	0.258	rs789586877	3
AAAG.CTTT	13	144678474	144678492	4.75	ENSSSCT00000012973	SLC12A8	intron	0.187	rs793270485	2
AAAAC.GTTTT	13	145055246	145055266	4.2	.	.	intergenic	-0.061	rs787102930	2
AAAAC.GTTTT	13	145129233	145129261	5.8	ENSSSCT00000012978	UMPS	intron	.	rs786225280	2
AAAAC.GTTTT	13	145129233	145129261	5.8	ENSSSCT00000027472	UMPS	intron	.	rs786225280	2
AAAC.GTTT	13	145169844	145169870	6.75	ENSSSCT00000023434	.	intron	.	rs791065965	2
AAAC.GTTT	13	145190513	145190526	3.5	ENSSSCT00000023434	.	intron	0.177	.	2
AG.CT	13	145291425	145291435	5.5	ENSSSCT00000023434	.	intron	.	rs790370032	2
AT.AT	13	145461833	145461842	5	ENSSSCT00000029280	.	intron	0.017	.	2
AT.AT	13	145461833	145461842	5	ENSSSCT00000023434	.	intron	0.017	.	2
AG.CT	13	145946976	145946988	6.5	.	.	intergenic	0.334	rs789325940	4
AAAC.GTTT	13	145960014	145960028	3.75	.	.	intergenic	-0.153	rs786573524	2
AAAT.ATTT	13	146172486	146172498	3.25	.	.	intergenic	0.117	rs792250699	2
AAAT.ATTT	13	146181176	146181196	5.25	.	.	intergenic	.	rs790864816	2
AAAAAG.CTTTTT	13	146655987	146656013	4.5	ENSSSCT00000029344	ADCY5	intron	0.103	rs786979273	2
AAAC.GTTT	13	147250196	147250210	3.75	.	.	intergenic	.	109542 rs7874	2
AAAAC.GTTTT	13	147331504	147331528	5	.	.	intergenic	0.082	rs792631141	2
ATC.GAT	13	147438046	147438060	5	ENSSSCT00000012988	PARP14	intron	.	rs788994819	2
AAAT.ATTT	13	147489070	147489087	4.5	.	.	intergenic	.	336254 rs7908	2
AT.AT	13	147527557	147527566	5	ENSSSCT00000012989	PARP15	intron	-0.22	rs792600023	2
AGG.CCT	13	147621806	147621819	4.66667	.	.	intergenic	0.137	rs790038331	2
AAAAT.ATTTT	13	147766094	147766109	3.2	ENSSSCT00000030693	CCDC58	intron	0.193	rs691169158	2
AAAC.GTTT	13	147774497	147774513	4.25	ENSSSCT00000030693	CCDC58	intron	0.277	rs787959677	2
AAAGG.CCTTT	13	147914053	147914075	4.6	ENSSSCT00000012993	CASR	intron	0.149	rs786937055	2
AT.AT	13	148003591	148003602	6	.	.	intergenic	.	.	4
AT.AT	13	148035260	148035272	6.5	.	.	intergenic	.	rs793053275	2
AC.GT	13	148076877	148076894	9	ENSSSCT00000012992	CD86	intron	0.217	rs791900845	2
AC.GT	13	148076877	148076894	9	ENSSSCT00000036378	CD86	intron	0.217	rs791900845	2
AC.GT	13	148076877	148076894	9	ENSSSCT00000034076	CD86	intron	0.217	rs791900845	2
AC.GT	13	148076877	148076894	9	ENSSSCT00000033968	CD86	intron	0.217	rs791900845	2
AC.GT	13	148076877	148076894	9	ENSSSCT00000012991	CD86	intron	0.217	rs791900845	2
AC.GT	13	148076877	148076894	9	ENSSSCT00000034541	CD86	intron	0.217	rs791900845	2
AAAC.GTTT	13	148083231	148083249	4.75	ENSSSCT00000012992	CD86	intron	0.116	rs788596982	3
AAAC.GTTT	13	148083231	148083249	4.75	ENSSSCT00000036378	CD86	intron	0.116	rs788596982	3
AAAC.GTTT	13	148083231	148083249	4.75	ENSSSCT00000034076	CD86	intron	0.116	rs788596982	3
AAAC.GTTT	13	148083231	148083249	4.75	ENSSSCT00000033968	CD86	intron	0.116	rs788596982	3
AAAC.GTTT	13	148083231	148083249	4.75	ENSSSCT00000012991	CD86	intron	0.116	rs788596982	3
AAAC.GTTT	13	148083231	148083249	4.75	ENSSSCT00000034541	CD86	intron	0.116	rs788596982	3
AAAC.GTTT	13	148083231	148083249	4.75	ENSSSCT00000034788	CD86	intron	0.116	rs788596982	3
AAC.GTT	13	148326723	148326734	4	ENSSSCT00000012997	IQCB1	intron	.	rs790725308	2
AAC.GTT	13	148539457	148539476	6.66667	.	.	intergenic	.	140389 rs7881	2
AT.AT	13	148570084	148570095	6	.	.	intergenic	.	rs787269734	2
AAGG.CCTT	13	149118429	149118443	3.75	.	.	intergenic	0.299	rs791311114	2
AAAC.GTTT	13	149251682	149251705	6	.	.	intergenic	0.249	rs788647036	2
AAC.GTT	13	149313625	149313639	5	ENSSSCT00000013005	GPR156	intron	0.356	rs791623059	2
AAAC.GTTT	13	149329403	149329420	4.5	ENSSSCT00000013005	GPR156	intron	.	327338 rs7895	2
AAG.CTT	13	149373063	149373080	6	.	.	intergenic	0.213	175357 rs7911	2
AAAT.ATTT	13	149475743	149475770	7	ENSSSCT00000013006	GSK3B	intron	.	rs786974269	2
AAC.GTT	13	149894909	149894934	8.66667	.	.	intergenic	.	rs787055834	2
AT.AT	13	150021411	150021423	6.5	.	.	intergenic	0.098	rs792989684	2
AAAC.GTTT	13	150103149	150103167	4.75	ENSSSCT00000013023	TMEM39A	intron	0.047	rs792128773	2
AAC.GTT	13	150361287	150361298	4	.	.	intergenic	.	.	3
AAAC.GTTT	13	150617008	150617023	4	.	.	intergenic	0.017	rs787979579	4
AT.AT	13	150693668	150693678	5.5	.	.	intergenic	0.357	.	2
AT.AT	13	150708276	150708292	8.5	.	.	intergenic	1.38	rs791831087	3
AT.AT	13	150954994	150955004	5.5	.	.	intergenic	.	rs712127282	3
AT.AT	13	151318004	151318014	5.5	.	.	intergenic	0.086	366683 rs7867	3
AAAT.ATTT	13	151396601	151396622	5.5	.	.	intergenic	0.014	rs788454805	2
AC.GT	13	151867762	151867773	6	.	.	intergenic	1.072	.	2
AC.GT	13	152048102	152048112	5.5	.	.	intergenic	0.232	.	2
AC.GT	13	152204542	152204559	9	.	.	intergenic	-0.112	755740 rs7871	3
ACAT.ATGT	13	152797969	152797987	4.75	.	.	intergenic	-0.075	.	4
AT.AT	13	152808638	152808651	7	.	.	intergenic	0.6	rs786280746	2
AAAC.GTTT	13	152961942	152961968	6.75	.	.	intergenic	-0.088	rs792070512	2
AC.GT	13	153239777	153239787	5.5	ENSSSCT00000021219	SNORD12	promoter	-0.197	.	2
AC.GT	13	153308795	153308809	7.5	.	.	intergenic	0.155	.	2
AAAC.GTTT	13	153622839	153622863	6.25	ENSSSCT00000030211	LSAMP	intron	.	.	2
AT.AT	13	153712761	153712772	6	ENSSSCT00000030211	LSAMP	intron	.	rs787696803	2
AC.GT	13	153739483	153739497	7.5	ENSSSCT00000030211	LSAMP	intron	0.339	rs786357110	2
AT.AT	13	153904957	153904983	13.5	.	.	intergenic	.	rs696175118	3
AC.GT	13	153947288	153947317	15	.	.	intergenic	0.143	rs699793355	4
ACAT.ATGT	13	154016772	154016794	5.75	.	.	intergenic	.	rs792635342	2
AATG.CATT	13	154305306	154305334	7.25	.	.	intergenic	0.222	394868 rs7899	2
AAT.ATT	13	154318192	154318211	6.66667	.	.	intergenic	.	rs789434079	2
AG.CT	13	154898781	154898792	6	.	.	intergenic	1.265	rs788641945	2
AAC.GTT	13	154943397	154943413	5.66667	.	.	intergenic	0.393	332922 rs7868	2

AAC.GTT	13	155027825	155027839	5	.	.	intergenic	0.652	rs786823913	2
AAT.ATT	13	155348367	155348386	6.66667	ENSSSCT00000013034	.	intron	.	rs791675414	2
AC.GT	13	155534032	155534048	8.5	ENSSSCT00000013036	.	intron	0.724	rs786744009	3
AAT.ATT	13	155560966	155560979	4.66667	ENSSSCT00000013036	.	intron	.	rs792161369	2
AAAG.CTTT	13	155766722	155766735	3.5	ENSSSCT00000013040	ATP6V1A	intron	0.297	rs788858587	2
AT.AT	13	155769527	155769536	5	ENSSSCT00000013040	ATP6V1A	intron	0.141	rs792942614	2
AAT.ATT	13	156235908	156235924	5.66667	.	.	intergenic	0.009	341520 rs7916	3
AC.GT	13	156934258	156934276	9.5	.	.	intergenic	.	.	2
AC.GT	13	157474396	157474420	12.5	.	.	intergenic	-0.007	176408 rs7916	3
AAC.GTT	13	157637754	157637767	4.66667	.	.	intergenic	0.863	rs793211901	2
AAAC.GTTT	13	157716108	157716119	3	.	.	intergenic	.	469038 rs7934	2
ACC.GGT	13	157771634	157771650	5.66667	.	.	intergenic	.	.	2
AAC.GTT	13	157972944	157972970	9	.	.	intergenic	.	913156 rs7868	2
AAT.ATT	13	158043035	158043061	9	.	.	intergenic	-0.098	rs789535702	2
AAT.ATT	13	158081632	158081662	10.3333	.	.	intergenic	0.063	205113 rs7899	2
AAG.CTT	13	158806181	158806192	4	.	.	intergenic	-0.123	643761 rs7936	2
AC.GT	13	158920709	158920719	5.5	ENSSSCT00000013059	.	intron	-0.042	.	3
AAAAG.CTTTT	13	159002448	159002464	3.4	ENSSSCT00000013058	PLCXD2	intron	0.336	330656 rs7883	2
AC.GT	13	159082620	159082630	5.5	.	.	intergenic	0.113	.	2
AAAAG.CTTTT	13	159275738	159275769	6.4	ENSSSCT00000031200	DPPA4	intron	1.16	rs788466515	2
AAAAG.CTTTT	13	159275738	159275769	6.4	ENSSSCT00000027112	DPPA4	intron	1.16	rs788466515	2
AT.AT	13	159313066	159313079	7	.	.	intergenic	.	rs791748282	4
AG.CT	13	159830276	159830286	5.5	.	.	intergenic	-0.264	rs792844859	2
AAC.GTT	13	159954451	159954466	5.33333	ENSSSCT00000013065	DZIP3	intron	0.36	rs792296243	2
AG.CT	13	160108386	160108402	8.5	ENSSSCT00000027252	MYH15	intron	.	.	4
AAAT.ATTT	13	160110786	160110833	12	ENSSSCT00000027252	MYH15	intron	.	925848 rs7891	2
AAG.CTT	13	160189915	160189947	11	ENSSSCT00000027252	MYH15	intron	.	rs793582277	2
AAAC.GTTT	13	160321123	160321142	5	.	.	intergenic	0.382	rs787184927	2
AAAC.GTTT	13	160409508	160409535	7	.	.	intergenic	0.206	rs788900157	2
AAAAT.ATTTT	13	160513422	160513449	5.6	ENSSSCT00000013068	CD47	intron	0.055	419380 rs7893	2
AG.CT	13	160544654	160544666	6.5	.	.	intergenic	-0.078	493601 rs7917	2
AT.AT	13	160683677	160683689	6.5	.	.	intergenic	0.219	rs787859164	2
AAAT.ATTT	13	160695791	160695816	6.5	.	.	intergenic	.	rs791651928	2
AAAT.ATTT	13	160782423	160782438	4	ENSSSCT00000013069	.	intron	0.316	rs791156694	2
AC.GT	13	160814541	160814555	7.5	ENSSSCT00000013069	.	intron	0.156	.	3
AAAT.ATTT	13	161062984	161063002	4.75	.	.	intergenic	0.325	748752 rs7905	2
AAC.GTT	13	161154826	161154842	5.66667	.	.	intergenic	0.238	rs792185447	2
AC.GT	13	161162491	161162501	5.5	.	.	intergenic	0.113	498190 rs7870	2
AAAG.CTTT	13	161472337	161472353	4.25	.	.	intergenic	.	358713 rs7887	2
AGG.CCT	13	161556855	161556869	5	.	.	intergenic	0.872	rs789205550	2
AAAT.ATTT	13	161559694	161559719	6.5	.	.	intergenic	0.127	rs792272132	2
ACCT.AGGT	13	161897561	161897583	5.75	.	.	intergenic	-0.01	261995 rs7897	2
AT.AT	13	161958460	161958472	6.5	.	.	intergenic	-0.033	368608 rs7934	2
AAAC.GTTT	13	162051542	162051562	5.25	.	.	intergenic	.	rs789681176	2
AAG.CTT	13	162141772	162141786	5	.	.	intergenic	0.224	rs792238671	2
AAAAC.GTTTT	13	162547865	162547892	5.6	.	.	intergenic	0.029	rs787095509	2
AAAC.GTTT	13	162706954	162706969	4	ENSSSCT00000023448	CBLB	intron	0.208	rs789067102	2
AAC.GTT	13	162726972	162726990	6.33333	ENSSSCT00000023448	CBLB	intron	0.211	718077 rs7924	2
AAC.GTT	13	163328634	163328654	7	.	.	intergenic	.	641565 rs7874	2
AAGG.CCTT	13	163443520	163443535	4	.	.	intergenic	0.084	rs790592012	2
AAAC.GTTT	13	163662165	163662187	5.75	.	.	intergenic	-0.193	rs790541607	2
AC.GT	13	163684540	163684549	5	.	.	intergenic	0.095	rs786954588	2
ATC.GAT	13	163853861	163853882	7.33333	.	.	intergenic	-0.093	rs789411713	2
AT.AT	13	163887139	163887150	6	.	.	intergenic	-0.037	.	2
AAAAC.GTTTT	13	163947372	163947398	5.4	.	.	intergenic	0.239	269576 rs7933	3
AAAAG.CTTTT	13	163967058	163967077	3.33333	.	.	intergenic	-0.102	rs787190325	2
AT.AT	13	163975536	163975547	6	.	.	intergenic	.	rs711374830	4
AAGG.CCTT	13	164152034	164152077	11	.	.	intergenic	-0.048	rs792804608	2
AT.AT	13	164179286	164179296	5.5	.	.	intergenic	-0.005	953214 rs7874	2
AT.AT	13	164252760	164252769	5	.	.	intergenic	0.147	rs786300329	2
AAAT.ATTT	13	164269080	164269104	6.25	.	.	intergenic	.	rs791689337	2
AAAT.ATTT	13	164371706	164371721	4	.	.	intergenic	-0.081	rs789621187	2
AAAT.ATTT	13	164541418	164541447	7.5	.	.	intergenic	-0.112	rs788781691	2
AAG.CTT	13	164559987	164560002	5.33333	.	.	intergenic	-0.246	rs787841569	3
AAAC.GTTT	13	164564030	164564048	4.75	.	.	intergenic	0.018	228210 rs7913	2
AGAT.ATCT	13	164566557	164566595	9.75	.	.	intergenic	0.116	rs697092002	2
AAAC.GTTT	13	164601685	164601703	4.75	.	.	intergenic	.	337343 rs7862	2
AAAC.GTTT	13	164611175	164611210	9	.	.	intergenic	.	956820 rs7878	2
AC.GT	13	164872284	164872299	8	.	.	intergenic	-0.158	.	2
AT.AT	13	164996689	164996708	10	.	.	intergenic	-0.794	rs787828684	2
AT.AT	13	165042092	165042103	6	.	.	intergenic	.	rs786714749	2
AGG.CCT	13	165091901	165091919	6.33333	.	.	intergenic	.	rs791749649	2
AC.GT	13	165272797	165272809	6.5	.	.	intergenic	-0.069	rs789846434	2
AT.AT	13	165282781	165282793	6.5	.	.	intergenic	-0.123	.	4
AAAC.GTTT	13	165406862	165406880	4.75	.	.	intergenic	0.027	rs790289507	2
AAT.ATT	13	165411436	165411449	4.66667	.	.	intergenic	0.035	rs793275597	2
AAC.GTT	13	165684375	165684388	4.66667	.	.	intergenic	0.103	.	4
AAC.GTT	13	165881342	165881373	10.6667	.	.	intergenic	0.609	rs788256489	2
AT.AT	13	165913500	165913520	10.5	.	.	intergenic	-0.1	rs787176383	2
AAAC.GTTT	13	165940639	165940661	5.75	.	.	intergenic	0.167	941470 rs7908	2
AAAT.ATTT	13	166234697	166234722	6.5	.	.	intergenic	0.157	rs791926704	2
AC.GT	13	166459971	166459983	6.5	.	.	intergenic	0.179	230905 rs7911	3
AAAAC.GTTTT	13	166740307	166740330	4.8	.	.	intergenic	0.102	rs786817495	2
AC.GT	13	166994230	166994240	5.5	ENSSSCT00000032455	.	intron	.	rs793426165	3

AG.CT	13	166995067	166995078	6	ENSSSCT00000032455	.	intron	0.258	.	2
AG.CT	13	167075180	167075191	6	ENSSSCT00000022387	SENP7	intron	0.342	.	2
AT.AT	13	167113665	167113676	6	ENSSSCT00000022387	SENP7	intron	0.323	311458 rs7916	3
AAAC.GTTT	13	167165438	167165464	6.75	.	.	intergenic	0.377	034131 rs7863	2
AAAT.ATTT	13	167418393	167418420	7	.	.	intergenic	.	rs791737886	2
AAAT.ATTT	13	167428811	167428828	4.5	.	.	intergenic	.	.	2
AT.AT	13	167441552	167441561	5	.	.	intergenic	.	rs787955841	2
AC.GT	13	167554799	167554811	6.5	.	.	intergenic	0.056	920706 rs7864	2
AC.GT	13	167567178	167567192	7.5	.	.	intergenic	.	rs793315119	2
AC.GT	13	167599518	167599546	14.5	.	.	intergenic	-0.077	rs789213025	3
AC.GT	13	167702326	167702337	6	.	.	intergenic	-0.054	740885 rs7922	2
AC.GT	13	167818135	167818155	10.5	ENSSSCT00000013085	.	intron	0.12	721935 rs7929	3
AC.GT	13	168101607	168101637	15.5	ENSSSCT00000013092	.	intron	0.241	.	4
AC.GT	13	168262494	168262507	7	.	.	intergenic	.	rs786433717	2
AT.AT	13	168451131	168451144	7	.	.	intergenic	-0.33	220478 rs7866	2
AAAC.GTTT	13	168490392	168490410	4.75	ENSSSCT00000013098	CMSS1	intron	0.329	.	2
AAAC.GTTT	13	168994126	168994140	3.75	.	.	intergenic	.	rs787751584	2
AC.GT	13	169218953	169218968	8	.	.	intergenic	0.254	049646 rs7880	2
AC.GT	13	169750579	169750591	6.5	.	.	intergenic	.	.	3
AAAG.CTTT	13	169965337	169965369	8.25	.	.	intergenic	.	rs790371034	2
AAAT.ATTT	13	170101700	170101725	6.5	.	.	intergenic	.	787780 rs7889	2
AAC.GTT	13	170116315	170116328	4.66667	.	.	intergenic	.	rs790417242	3
AATT.AATT	13	170326156	170326170	3.75	.	.	intergenic	.	.	2
AT.AT	13	170358493	170358503	5.5	ENSSSCT00000013106	DCBLD2	intron	0.044	rs793258559	2
AC.GT	13	170407659	170407688	15	.	.	intergenic	-0.07	rs791588820	2
AAAAG.CTTTT	13	170488620	170488638	3.8	.	.	intergenic	.	rs788397586	2
AAAC.GTTT	13	170839623	170839651	7.25	.	.	intergenic	.	rs791981861	2
AAAC.GTTT	13	170849056	170849077	5.5	.	.	intergenic	.	rs787926568	3
AAAAT.ATTTT	13	170876561	170876581	4.2	.	.	intergenic	.	rs789840925	2
AAAC.GTTT	13	171009845	171009867	5.75	.	.	intergenic	.	.	2
AAG.CTT	13	172054849	172054864	5.33333	.	.	intergenic	.	.	2
AAAAAT.ATTTTT	13	172085437	172085460	4	.	.	intergenic	.	rs787891574	2
AT.AT	13	172086787	172086797	5.5	.	.	intergenic	.	075525 rs7909	2
AG.CT	13	172117459	172117477	9.5	.	.	intergenic	.	419068 rs7936	2
AAAAG.CTTTT	13	172169401	172169419	3.8	.	.	intergenic	.	rs792176964	2
AT.AT	13	172330565	172330574	5	.	.	intergenic	.	.	2
AAT.ATT	13	172585699	172585719	7	.	.	intergenic	.	rs791397198	2
AAACC.GGTTT	13	172589411	172589433	4.6	.	.	intergenic	.	rs791807898	2
AAAT.ATTT	13	172600913	172600936	6	.	.	intergenic	.	rs787390909	2
AAAC.GTTT	13	172987201	172987223	5.75	.	.	intergenic	.	rs790743569	2
AAAT.ATTT	13	173111418	173111437	5	.	.	intergenic	-0.103	.	2
AAAC.GTTT	13	173397982	173398004	5.75	.	.	intergenic	-0.035	rs790118779	2
AG.CT	13	173439583	173439594	6	.	.	intergenic	-0.207	.	2
AAAG.CTTT	13	173610590	173610608	4.75	.	.	intergenic	.	rs790215600	2
AC.GT	13	173613279	173613288	5	.	.	intergenic	.	.	2
AT.AT	13	173725002	173725016	7.5	.	.	intergenic	0.172	007137 rs7915	2
AG.CT	13	173956726	173956746	10.5	.	.	intergenic	0.041	646186 rs7872	2
AAC.GTT	13	174067340	174067353	4.66667	.	.	intergenic	.	rs792439866	3
AAAAC.GTTTT	13	174079262	174079286	5	.	.	intergenic	.	920788 rs7933	2
AT.AT	13	174176462	174176476	7.5	.	.	intergenic	0.003	.	4
AT.AT	13	174328263	174328276	7	.	.	intergenic	.	rs786853853	2
AC.GT	13	174421629	174421651	11.5	.	.	intergenic	0.081	rs789992706	2
AAAAT.ATTTT	13	174593187	174593205	3.8	.	.	intergenic	.	rs788374711	2
AAGC.GCTT	13	174788714	174788734	5.25	.	.	intergenic	.	rs791433753	3
AC.GT	13	174796616	174796628	6.5	.	.	intergenic	0.205	.	2
AAAG.CTTT	13	175020246	175020264	4.75	.	.	intergenic	-0.42	.	3
AC.GT	13	175027241	175027252	6	.	.	intergenic	.	.	3
AC.GT	13	175086213	175086253	20.5	.	.	intergenic	.	rs788968407	2
AG.CT	13	175324466	175324476	5.5	.	.	intergenic	.	.	2
AG.CT	13	175368524	175368543	10	.	.	intergenic	.	128681 rs7929	2
AAT.ATT	13	175537984	175538001	6	.	.	intergenic	0.031	.	2
AAAT.ATTT	13	175543232	175543253	5.5	.	.	intergenic	0.273	rs787988287	2
AC.GT	13	175580072	175580081	5	.	.	intergenic	.	rs787491961	3
AC.GT	13	175832274	175832289	8	.	.	intergenic	0.004	rs786965035	4
AATG.CATT	13	176089746	176089775	7.5	.	.	intergenic	-0.111	rs788386163	2
AT.AT	13	176704777	176704791	7.5	.	.	intergenic	0.125	.	4
AAAC.GTTT	13	176785169	176785183	3.75	.	.	intergenic	-0.007	444121 rs7864	2
AT.AT	13	177398847	177398858	6	.	.	intergenic	-0.05	rs790870880	2
AACT.AGTT	13	177509216	177509230	3.75	ENSSSCT00000022760	EPHA3	intron	-0.061	rs788167573	2
AAAC.GTTT	13	177700784	177700805	5.5	ENSSSCT00000022760	EPHA3	intron	.	995078 rs7908	2
AC.GT	13	177737271	177737286	8	ENSSSCT00000022760	EPHA3	intron	0.015	.	2
AAAAC.GTTTT	13	178092405	178092427	4.6	.	.	intergenic	-0.047	rs793032312	2
ACAT.ATGT	13	178349598	178349617	5	.	.	intergenic	.	rs793573974	2
AAAT.ATTT	13	178354087	178354106	5	.	.	intergenic	0.664	rs789947326	2
AT.AT	13	178820371	178820394	12	.	.	intergenic	0.173	rs788370904	2
AT.AT	13	179313362	179313371	5	.	.	intergenic	.	.	3
AAAC.GTTT	13	180017939	180017969	7.75	.	.	intergenic	0.1	rs787412785	2
AATT.AATT	13	180029841	180029858	4.5	.	.	intergenic	.	rs791901012	2
AAAC.GTTT	13	180137870	180137892	5.75	.	.	intergenic	0.015	rs791300628	2
AAAC.GTTT	13	180269532	180269563	8	.	.	intergenic	0.01	rs793195554	2
AG.CT	13	180280587	180280602	8	.	.	intergenic	0.359	779359 rs7880	2
AAGG.CCTT	13	180320261	180320277	4.25	.	.	intergenic	-0.118	364231 rs7866	2
AC.GT	13	180385452	180385468	8.5	.	.	intergenic	-0.102	rs786902806	2
AG.CT	13	180419076	180419098	11.5	.	.	intergenic	-0.043	rs792869260	2

AC.GT	13	180663171	180663204	17	.	.	intergenic	0.273	.	2
AT.AT	13	180903079	180903089	5.5	ENSSSCT00000013130	CADM2	intron	-0.257	rs786543319	2
AC.GT	13	180908583	180908594	6	ENSSSCT00000013130	CADM2	intron	0.004	rs788824551	2
AAAAT.ATTTT	13	181204815	181204842	5.6	.	.	intergenic	-0.242	rs787771507	2
ACC.GGT	13	181707449	181707461	4.33333	.	.	intergenic	-0.053	.	2
AC.GT	13	181832325	181832338	7	.	.	intergenic	.	.	2
AC.GT	13	181922151	181922163	6.5	.	.	intergenic	.	rs793740087	3
AT.AT	13	181942523	181942539	8.5	.	.	intergenic	.	.	4
AAAC.GTTT	13	182363857	182363879	5.75	.	.	intergenic	.	326086 rs7871	2
AC.GT	13	182600228	182600247	10	.	.	intergenic	.	.	4
AATG.CATT	13	182777057	182777083	6.75	.	.	intergenic	0.017	rs787425028	2
AAAT.ATTT	13	182811126	182811141	4	.	.	intergenic	.	rs787889767	2
AAAC.GTTT	13	182849847	182849861	3.75	.	.	intergenic	-0.085	)20475 rs7902	4
AC.GT	13	183036252	183036264	6.5	.	.	intergenic	-0.066	.	3
AC.GT	13	183036540	183036550	5.5	.	.	intergenic	-0.24	rs791853220	5
ATC.GAT	13	183077980	183078001	7.33333	.	.	intergenic	.	rs792575305	2
AAAT.ATTT	13	183105168	183105182	3.75	.	.	intergenic	0.268	rs787801344	2
AT.AT	13	183218057	183218077	10.5	.	.	intergenic	.	rs789143462	2
AC.GT	13	183368374	183368400	13.5	.	.	intergenic	.	rs787270395	2
AAAT.ATTT	13	183799448	183799475	7	.	.	intergenic	0.834	rs786996946	2
AAT.ATT	13	183821078	183821093	5.33333	.	.	intergenic	0.032	rs788416194	2
AT.AT	13	183838534	183838545	6	ENSSSCT00000024901	.	intron	.	rs788864876	2
AT.AT	13	184222968	184222978	5.5	ENSSSCT00000031865	.	intron	0.141	rs790648650	2
AT.AT	13	184283269	184283281	6.5	.	.	intergenic	0.417	rs788649801	2
AT.AT	13	184290050	184290073	12	.	.	intergenic	0.056	rs793240004	2
AT.AT	13	184353022	184353049	14	.	.	intergenic	.	rs791978160	3
AT.AT	13	184482449	184482468	10	.	.	intergenic	-0.084	.	3
AGGG.CCCT	13	184506333	184506346	3.5	.	.	intergenic	-0.097	rs788296666	2
AAC.GTT	13	185268220	185268241	7.33333	.	.	intergenic	-0.009	.	2
AT.AT	13	185465502	185465511	5	.	.	intergenic	-0.116	rs791772427	4
AAC.GTT	13	185653203	185653219	5.66667	.	.	intergenic	-0.059	526994 rs7904	2
AAAAAT.ATTTTT	13	185682341	185682373	5.5	.	.	intergenic	0.054	rs791340111	2
AC.GT	13	185722995	185723007	6.5	.	.	intergenic	.	rs787289738	4
AAC.GTT	13	185819861	185819874	4.66667	.	.	intergenic	-0.054	.	2
AAAT.ATTT	13	185917490	185917501	3	.	.	intergenic	0.026	rs791274622	2
AAAT.ATTT	13	185939521	185939535	3.75	.	.	intergenic	-0.121	rs787117823	2
AC.GT	13	186006330	186006346	8.5	.	.	intergenic	.	307256 rs7893	2
AAAC.GTTT	13	186064544	186064562	4.75	.	.	intergenic	-0.095	504833 rs7906	2
AG.CT	13	186232457	186232472	8	.	.	intergenic	.	.	4
AC.GT	13	186316281	186316300	10	.	.	intergenic	0.104	rs786853194	2
AAAAAC.GTTTTT	13	186452254	186452274	3.5	.	.	intergenic	0.008	rs786645395	2
AG.CT	13	186637551	186637560	5	ENSSSCT00000013132	.	intron	-0.102	.	3
AAAAAC.GTTTTT	13	186638095	186638132	6.33333	ENSSSCT00000013132	.	intron	.	rs792286979	2
AAAG.CTTT	13	186643327	186643350	6	ENSSSCT00000013132	.	intron	-0.271	445745 rs7886	2
AGG.CCT	13	186715826	186715842	5.66667	ENSSSCT00000013132	.	intron	0.223	rs787835464	2
AAC.GTT	13	186767983	186768014	10.6667	ENSSSCT00000013132	.	intron	0.153	904951 rs7897	2
AT.AT	13	186804229	186804240	6	ENSSSCT00000013132	.	intron	-0.006	rs793489650	3
AT.AT	13	186957102	186957117	8	.	.	intergenic	-0.12	.	2
AT.AT	13	187005368	187005387	10	.	.	intergenic	.	rs787571072	2
AC.GT	13	187063117	187063132	8	.	.	intergenic	0.461	405705 rs7937	2
AC.GT	13	187120459	187120471	6.5	.	.	intergenic	0.002	.	4
AAAAC.GTTTT	13	187130860	187130880	4.2	.	.	intergenic	0.505	rs789856921	2
AAAT.ATTT	13	187194932	187194963	8	.	.	intergenic	0.58	278228 rs7872	2
ACC.GGT	13	187517857	187517879	7.66667	.	.	intergenic	.	rs788239117	2
ACT.AGT	13	187518006	187518020	5	.	.	intergenic	.	.	2
AT.AT	13	187630196	187630221	13	.	.	intergenic	-0.058	.	2
AC.GT	13	187868628	187868640	6.5	.	.	intergenic	0.045	.	4
AAAC.GTTT	13	187956200	187956222	5.75	.	.	intergenic	-0.055	563464 rs7915	2
AT.AT	13	188092742	188092751	5	.	.	intergenic	0.261	.	2
AAAT.ATTT	13	188185701	188185716	4	.	.	intergenic	0.106	396992 rs7888	2
AAAC.GTTT	13	188408283	188408309	6.75	.	.	intergenic	0.165	549785 rs7913	2
AAAC.GTTT	13	188455871	188455896	6.5	.	.	intergenic	-0.06	rs787546872	2
AAAT.ATTT	13	188677960	188677976	4.25	.	.	intergenic	0.17	281382 rs7902	2
AAC.GTT	13	188690428	188690444	5.66667	.	.	intergenic	0.099	rs786901222	3
AC.GT	13	188691157	188691166	5	.	.	intergenic	-0.023	.	4
AATG.CATT	13	188695629	188695646	4.5	.	.	intergenic	-0.172	755616 rs7889	2
AAAAG.CTTTT	13	188718612	188718630	3.8	.	.	intergenic	.	375903 rs7905	2
AAAAT.ATTTT	13	188787949	188787979	6.2	.	.	intergenic	0.051	339512 rs7875	2
AC.GT	13	188803175	188803194	10	.	.	intergenic	-0.127	.	4
AC.GT	13	188908058	188908068	5.5	.	.	intergenic	-0.107	rs789499107	2
AC.GT	13	188952346	188952356	5.5	.	.	intergenic	-0.212	rs786410731	2
AG.CT	13	189021291	189021300	5	.	.	intergenic	-0.014	rs788455001	2
AC.GT	13	189265792	189265803	6	.	.	intergenic	.	rs786333872	2
AAAT.ATTT	13	189305517	189305536	5	.	.	intergenic	.	rs793119984	2
AT.AT	13	189376683	189376701	9.5	ENSSSCT00000013136	.	intron	0.533	.	2
AAAT.ATTT	13	189529890	189529913	6	ENSSSCT00000013137	.	intron	0.062	478839 rs7880	2
AC.GT	13	189710263	189710282	10	ENSSSCT00000013139	SAMSN1	intron	0.539	rs792869020	3
AC.GT	13	189750950	189750972	11.5	.	.	intergenic	0.101	357129 rs7921	2
AG.CT	13	189758305	189758319	7.5	.	.	intergenic	0.034	058699 rs7915	2
AG.CT	13	190000518	190000528	5.5	.	.	intergenic	0.05	rs786946961	4
AT.AT	13	190389953	190389962	5	.	.	intergenic	0.603	rs790968432	2
AT.AT	13	190429103	190429117	7.5	.	.	intergenic	0.095	.	2
AAGG.CCTT	13	190497012	190497047	9	.	.	intergenic	-0.044	362714 rs7900	2
AT.AT	13	190503808	190503819	6	.	.	intergenic	.	rs787924237	2

AAAT.ATTT	13	190584657	190584673	4.25	.	.	intergenic	-0.077	rs791717364	2
AC.GT	13	190673038	190673048	5.5	.	.	intergenic	-0.024	rs789356326	2
AAC.GTT	13	190784126	190784139	4.66667	.	.	intergenic	0.138	365959 rs7927	2
AC.GT	13	190940456	190940466	5.5	ENSSSCT00000022689	USP25	intron	-0.006	rs790287437	3
AC.GT	13	190940456	190940466	5.5	ENSSSCT00000027672	USP25	intron	-0.006	rs790287437	3
AAG.CTT	13	191111954	191111970	5.66667	.	.	intergenic	.	rs793778958	2
AAAG.CTTT	13	191135786	191135802	4.25	.	.	intergenic	0.092	rs790141548	2
AAAAT.ATTTT	13	191215604	191215618	3	.	.	intergenic	0.168	rs789476969	2
AAC.GTT	13	191311224	191311237	4.66667	.	.	intergenic	1.913	rs792272271	4
AAAC.GTTT	13	191456023	191456044	5.5	.	.	intergenic	-0.021	rs787888337	2
AAC.GTT	13	191485310	191485330	7	.	.	intergenic	0.173	rs788616681	2
AC.GT	13	191704124	191704134	5.5	.	.	intergenic	0.038	.	2
AG.CT	13	191704770	191704790	10.5	.	.	intergenic	.	rs787993722	6
AAC.GTT	13	192191520	192191534	5	.	.	intergenic	.	rs700207044	5
AAT.ATT	13	192232476	192232498	7.66667	.	.	intergenic	.	341077 rs7918	4
AAAC.GTTT	13	192443773	192443785	3.25	.	.	intergenic	.	rs789897803	2
AT.AT	13	192721722	192721731	5	.	.	intergenic	0.128	rs792425644	3
AAAG.CTTT	13	193447154	193447184	7.75	.	.	intergenic	0.053	rs791420001	2
AAAAC.GTTTT	13	193826089	193826112	4.8	.	.	intergenic	0.05	253225 rs7908	2
AC.GT	13	193835521	193835546	13	.	.	intergenic	.	rs787890387	3
AAG.CTT	13	193900001	193900016	5.33333	.	.	intergenic	0.532	.	2
AAC.GTT	13	193948385	193948398	4.66667	.	.	intergenic	-0.228	rs787181926	2
AG.CT	13	194009399	194009413	7.5	.	.	intergenic	-0.023	rs791459050	3
AT.AT	13	194324615	194324631	8.5	.	.	intergenic	0.137	rs793588797	2
AAAAC.GTTTT	13	194637270	194637288	3.8	.	.	intergenic	0.075	rs791091127	2
AC.GT	13	194693330	194693343	7	.	.	intergenic	-0.094	732209 rs7890	2
AAT.ATT	13	194784637	194784649	4.33333	.	.	intergenic	-0.037	rs786525791	2
AAAC.GTTT	13	194806327	194806345	4.75	.	.	intergenic	0.185	rs791412263	2
AAT.ATT	13	194820470	194820504	11.6667	.	.	intergenic	0.063	rs793093181	2
AT.AT	13	194958050	194958059	5	.	.	intergenic	0.072	.	4
AC.GT	13	194981890	194981902	6.5	.	.	intergenic	-0.023	rs786813782	3
AC.GT	13	195248468	195248478	5.5	.	.	intergenic	-0.139	rs790762832	3
AAAC.GTTT	13	195406707	195406727	5.25	ENSSSCT00000013151	.	intron	0.025	rs788320697	2
AATC.GATT	13	195415639	195415660	5.5	ENSSSCT00000013151	.	intron	0.252	141903 rs7925	2
AAT.ATT	13	195597064	195597085	7.33333	.	.	intergenic	0.047	rs792666218	2
AAC.GTT	13	195632254	195632281	9.33333	.	.	intergenic	-0.111	rs787739408	2
AAC.GTT	13	195799932	195799948	5.66667	.	.	intergenic	0.091	148260 rs7921	2
AC.GT	13	196002250	196002273	12	.	.	intergenic	0.14	.	2
AAAAC.GTTTT	13	196146120	196146144	4.16667	.	.	intergenic	.	rs789152540	2
AG.CT	13	196245121	196245132	6	.	.	intergenic	-0.058	.	2
AT.AT	13	196304031	196304058	14	.	.	intergenic	.	rs789851275	2
AAAC.GTTT	13	196305223	196305234	3	.	.	intergenic	0.063	rs790755632	2
AAAT.ATTT	13	196320746	196320758	3.25	.	.	intergenic	0.089	rs792436606	2
AGGAG.CTCCT	13	196465850	196465878	4.83333	.	.	intergenic	.	rs786407122	2
AT.AT	13	196574569	196574579	5.5	.	.	intergenic	-0.082	rs787038350	2
AT.AT	13	196746481	196746492	6	.	.	intergenic	-0.161	240159 rs7868	2
AAAC.GTTT	13	196966995	196967009	3.75	.	.	intergenic	-0.034	rs791415801	2
AT.AT	13	196982628	196982638	5.5	.	.	intergenic	.	rs793398620	3
AAC.GTT	13	197195550	197195561	4	.	.	intergenic	.	rs788973873	3
AAC.GTT	13	197235143	197235156	4.66667	.	.	intergenic	.	.	2
AG.CT	13	197603757	197603769	6.5	.	.	intergenic	-0.312	rs786705444	3
AAAT.ATTT	13	197680317	197680347	7.75	.	.	intergenic	-0.2	rs786292750	2
AC.GT	13	197729230	197729242	6.5	.	.	intergenic	.	rs793810963	3
AAAT.ATTT	13	197844661	197844686	6.5	.	.	intergenic	.	.	2
AC.GT	13	198163399	198163410	6	.	.	intergenic	-0.105	.	3
AG.CT	13	198397962	198397974	6.5	.	.	intergenic	0.097	rs789371945	2
ACACC.GGTGT	13	198437969	198437991	4.6	.	.	intergenic	-0.058	208068 rs7863	2
AAAC.GTTT	13	198656597	198656623	6.75	.	.	intergenic	0.095	rs790791967	2
AG.CT	13	198733550	198733559	5	.	.	intergenic	-0.046	.	2
AG.CT	13	198960852	198960864	6.5	.	.	intergenic	.	rs791948569	2
AC.GT	13	199060709	199060718	5	.	.	intergenic	.	.	3
AC.GT	13	199061623	199061641	9.5	.	.	intergenic	.	404104 rs7929	3
AG.CT	13	199081571	199081581	5.5	.	.	intergenic	0.209	368658 rs7915	2
AAAT.ATTT	13	199160277	199160299	5.75	ENSSSCT00000024945	JAM2	intron	.	rs788922203	2
AAAT.ATTT	13	199160277	199160299	5.75	ENSSSCT00000023520	JAM2	intron	.	rs788922203	2
AAT.ATT	13	199293529	199293542	4.66667	ENSSSCT00000013154	APP	intron	-0.055	401061 rs7935	2
AAT.ATT	13	199293529	199293542	4.66667	ENSSSCT00000013153	APP	intron	-0.055	401061 rs7935	2
AC.GT	13	199341044	199341065	11	ENSSSCT00000013154	APP	intron	0.259	.	5
AC.GT	13	199341044	199341065	11	ENSSSCT00000013153	APP	intron	0.259	.	5
AC.GT	13	199462061	199462076	8	ENSSSCT00000013154	APP	intron	.	.	2
AC.GT	13	199462061	199462076	8	ENSSSCT00000013153	APP	intron	.	.	2
AAAC.GTTT	13	199578943	199578957	3.75	.	.	intergenic	0.02	rs788087639	2
ACATAT.ATATG	13	199583909	199583928	3.33333	.	.	intergenic	.	rs786708689	2
AAC.GTT	13	199708897	199708913	5.66667	.	.	intergenic	0.071	rs791922989	2
AG.CT	13	199778956	199778968	6.5	.	.	intergenic	0.09	rs791652846	2
AAAT.ATTT	13	199935182	199935200	4.75	.	.	intergenic	0.058	rs788779893	4
AG.CT	13	200383482	200383491	5	.	.	intergenic	0.315	rs786883431	4
AC.GT	13	200396760	200396770	5.5	.	.	intergenic	0.113	rs791556412	2
AAAC.GTTT	13	200421551	200421573	5.75	ENSSSCT00000013159	ADAMTS5	intron	-0.095	404227 rs7887	5
AT.AT	13	200495344	200495362	9.5	.	.	intergenic	-0.025	.	4
AC.GT	13	200523610	200523626	8.5	.	.	intergenic	.	.	2
AG.CT	13	200538067	200538076	5	.	.	intergenic	-0.022	rs791242765	2
AAC.GTT	13	200618448	200618460	4.33333	.	.	intergenic	0.032	rs788010440	2
AAAT.ATTT	13	200838629	200838647	4.75	.	.	intergenic	-0.18	rs793553889	2

AT.AT	13	201031400	201031409	5	.	.	intergenic	-0.085	.	2
AAAT.ATTT	13	201109699	201109731	8.25	.	.	intergenic	.	rs786582733	2
AT.AT	13	201113579	201113603	12.5	.	.	intergenic	-0.154	.	4
AAAC.GTTT	13	201165716	201165727	3	.	.	intergenic	-0.171	rs787155108	2
AT.AT	13	201313985	201313998	7	.	.	intergenic	0.026	.	2
AAAC.GTTT	13	201329519	201329537	4.75	.	.	intergenic	.	079034 rs7900	2
AT.AT	13	201336149	201336160	6	.	.	intergenic	0.036	481791 rs7881	2
AC.GT	13	201347467	201347488	11	.	.	intergenic	0.212	336113 rs7902	4
AAC.GTT	13	201589868	201589887	6.66667	.	.	intergenic	0.12	rs792082553	2
AAAC.GTTT	13	201592872	201592888	4.25	.	.	intergenic	.	rs789383768	2
AT.AT	13	201628048	201628065	9	.	.	intergenic	0.037	358266 rs6929	2
AAAG.CTTT	13	201716303	201716327	6.25	.	.	intergenic	-0.041	.	2
AAAAC.GTTTT	13	201865237	201865256	4	.	.	intergenic	-0.127	rs787725045	2
AAAG.CTTT	13	201903525	201903539	3.75	.	.	intergenic	-0.009	rs791460764	2
AC.GT	13	202182805	202182814	5	.	.	intergenic	0.2	.	2
AAAC.GTTT	13	202191391	202191406	4	.	.	intergenic	.	739831 rs7921	2
AAAT.ATTT	13	202248909	202248936	7	.	.	intergenic	-0.056	rs788091367	2
AT.AT	13	202274814	202274828	7.5	.	.	intergenic	0.116	.	2
AAAT.ATTT	13	202492783	202492800	4.5	.	.	intergenic	.	249906 rs7894	2
AAT.ATT	13	202515507	202515520	4.66667	.	.	intergenic	.	.	2
AAAC.GTTT	13	202594350	202594368	4.75	.	.	intergenic	.	206142 rs7881	2
AAAG.CTTT	13	202795869	202795885	4.25	.	.	intergenic	.	rs788349071	2
AC.GT	13	203056723	203056732	5	.	.	intergenic	-0.045	rs786428414	2
AT.AT	13	203074622	203074633	6	.	.	intergenic	0.212	519799 rs7884	2
AAC.GTT	13	203421784	203421799	5.33333	ENSSSCT00000028121	GRIK1	intron	-0.173	rs789008871	2
AC.GT	13	203679867	203679882	8	.	.	intergenic	-0.105	722358 rs7925	4
AAC.GTT	13	203790256	203790277	7.33333	.	.	intergenic	0.054	rs788447758	2
AG.CT	13	204089632	204089646	7.5	.	.	intergenic	-0.017	rs788205154	2
AG.CT	13	204132804	204132825	11	.	.	intergenic	0.03	rs789338498	2
AAAAAT.ATTTTT	13	204219076	204219096	3.5	.	.	intergenic	-0.278	rs793472911	2
AAAAC.GTTTT	13	204232873	204232888	3.2	.	.	intergenic	0.146	444599 rs7912	2
AAAAG.CTTTT	13	204240795	204240817	4.6	.	.	intergenic	-0.043	rs787802660	2
AAAC.GTTT	13	204268035	204268061	6.75	.	.	intergenic	-0.063	.	2
AAAC.GTTT	13	204273839	204273873	8.75	.	.	intergenic	-0.152	362597 rs7919	3
AC.GT	13	204422172	204422182	5.5	.	.	intergenic	-0.177	rs786996933	4
AAC.GTT	13	204487881	204487900	6.66667	.	.	intergenic	.	262280 rs7891	2
AAAC.GTTT	13	204526320	204526336	4.25	.	.	intergenic	.	.	3
AAAG.CTTT	13	204883713	204883725	3.25	.	.	intergenic	0.385	rs786588080	2
AAAAC.GTTTT	13	204949871	204949889	3.8	.	.	intergenic	-0.193	752954 rs7932	2
AATG.CATT	13	204961716	204961738	5.75	.	.	intergenic	-0.01	250519 rs7900	2
AAAAAG.CTTTTT	13	204964003	204964032	5	.	.	intergenic	-0.022	rs786633817	2
AAG.CTT	13	205082796	205082813	6	.	.	intergenic	-0.031	rs792116373	2
AC.GT	13	205138695	205138706	6	.	.	intergenic	-0.066	579667 rs7864	3
AAAAC.GTTTT	13	205296172	205296187	3.2	ENSSSCT00000031132	TIAM1	intron	0.564	rs788361589	2
AAAAC.GTTTT	13	205296172	205296187	3.2	ENSSSCT00000013166	TIAM1	intron	0.564	rs788361589	2
AAAG.CTTT	13	205467442	205467464	5.75	.	.	intergenic	0.199	533711 rs7869	2
AAAC.GTTT	13	205763565	205763593	7.25	ENSSSCT00000020619	U6	promoter	0.307	033865 rs7878	2
AG.CT	13	206107102	206107120	9.5	.	.	intergenic	.	rs791389258	2
AAAG.CTTT	13	206203995	206204013	4.75	.	.	intergenic	.	rs789128252	2
AAG.CTT	13	206530178	206530203	8.66667	.	.	intergenic	-0.118	rs786669695	2
AATC.GATT	13	206630483	206630500	4.5	.	.	intergenic	-0.184	.	2
AG.CT	13	206631644	206631653	5	.	.	intergenic	0.541	.	2
AC.GT	13	206781403	206781417	7.5	.	.	intergenic	.	554738 rs7879	2
AT.AT	13	207022997	207023006	5	ENSSSCT00000030581	CRYZL1	intron	0.148	rs790470125	2
AC.GT	13	207248334	207248346	6.5	ENSSSCT00000027819	ITSN1	intron	.	.	3
AAAG.CTTT	13	207477130	207477161	8	.	.	intergenic	0.161	274108 rs7933	2
AAAT.ATTT	13	207514368	207514381	3.5	.	.	intergenic	.	.	2
AAAT.ATTT	13	207677284	207677300	4.25	.	.	intergenic	0.05	rs793535537	2
AAAG.CTTT	13	207750034	207750050	4.25	.	.	intergenic	0.043	rs791612070	2
AAAAC.GTTTT	13	207901674	207901700	5.4	.	.	intergenic	0.128	166809 rs7889	2
AC.GT	13	207994317	207994328	6	.	.	intergenic	-0.148	.	6
AAC.GTT	13	208028379	208028395	5.66667	.	.	intergenic	0.058	rs791968424	2
AT.AT	13	208106886	208106896	5.5	ENSSSCT00000029780	CLIC6	intron	-0.1	.	2
AC.GT	13	208120666	208120677	6	ENSSSCT00000029780	CLIC6	intron	-0.422	rs791441099	6
AAAC.GTTT	13	208191402	208191422	5.25	.	.	intergenic	.	743090 rs7868	4
AC.GT	13	208251515	208251524	5	ENSSSCT00000013183	RUNX1	intron	-0.173	.	2
AC.GT	13	208427282	208427296	7.5	.	.	intergenic	-0.201	.	6
AC.GT	13	208432676	208432692	8.5	.	.	intergenic	0.307	rs787994677	2
AAC.GTT	13	208725599	208725614	5.33333	.	.	intergenic	0.031	.	4
AAAT.ATTT	13	209069656	209069682	6.75	.	.	intergenic	.	.	2
AAAT.ATTT	13	209111459	209111482	6	.	.	intergenic	.	rs791019842	2
AT.AT	13	209219948	209219969	11	.	.	intergenic	1.342	rs788984758	2
AAAG.CTTT	13	209382692	209382721	7.5	.	.	intergenic	0.351	.	3
AGG.CCT	13	209459003	209459021	6.33333	.	.	intergenic	.	276756 rs7880	3
AAAAAT.ATTTTT	13	209587413	209587445	5.5	.	.	intergenic	.	rs788175848	2
AAAAAG.CTTTTT	13	209603207	209603229	3.83333	.	.	intergenic	0.857	.	2
AT.AT	13	209612569	209612581	6.5	.	.	intergenic	-0.013	329253 rs7907	2
AAAC.GTTT	13	209851008	209851026	4.75	ENSSSCT00000013185	CBR3	intron	0.032	777432 rs7927	2
AAAT.ATTT	13	210092848	210092878	7.75	ENSSSCT00000013188	CHAF1B	intron	.	969543 rs7924	2
AAT.ATT	13	210236980	210237003	8	.	.	intergenic	-0.131	rs786563502	2
AC.GT	13	210306230	210306243	7	.	.	intergenic	0.102	rs789108495	2
AT.AT	13	210328189	210328198	5	.	.	intergenic	.	576826 rs7874	2
AG.CT	13	210419903	210419935	16.5	.	.	intergenic	0.005	rs793792561	2
AG.CT	13	210536423	210536441	9.5	ENSSSCT00000013191	HLCS	intron	.	rs788782271	2

AAAAC.GTTTT	13	210628052	210628077	4.33333	ENSSSCT00000013191	HLCS	intron	.	rs791400967	2
AAAT.ATTT	13	210632449	210632467	4.75	ENSSSCT00000013191	HLCS	intron	0.198	rs793386197	2
AC.GT	13	210690573	210690588	8	.	.	intergenic	0.023	rs793096321	2
AAAAC.GTTTT	13	210785420	210785434	3	ENSSSCT00000013194	.	intron	0.241	.	2
AAAC.GTTT	13	211089086	211089109	6	.	.	intergenic	0.425	rs788773152	2
AAAT.ATTT	13	211294470	211294491	5.5	.	.	intergenic	-0.155	rs792464813	2
AG.CT	13	211510958	211510968	5.5	.	.	intergenic	0.052	.	4
AAAAG.CTTTT	13	211763829	211763847	3.8	.	.	intergenic	.	rs789008293	2
AC.GT	13	211798288	211798298	5.5	.	.	intergenic	-0.022	rs786934131	2
ACAG.CTGT	13	211886647	211886661	3.75	.	.	intergenic	-0.236	.	2
AC.GT	13	212122187	212122197	5.5	ENSSSCT00000023594	ERG	intron	0.026	.	4
AAT.ATT	13	212140928	212140943	5.33333	ENSSSCT00000023594	ERG	intron	-0.017	rs790747085	2
AC.GT	13	212398252	212398263	6	.	.	intergenic	0.244	rs791140140	3
AC.GT	13	212466232	212466242	5.5	.	.	intergenic	.	rs787373127	2
AG.CT	13	212474292	212474313	11	.	.	intergenic	-0.57	.	2
AGAT.ATCT	13	212622917	212622932	4	.	.	intergenic	-0.061	rs791883580	2
AAC.GTT	13	213306279	213306293	5	ENSSSCT00000029140	SH3BGR	intron	-0.169	.	2
AAC.GTT	13	213514380	213514399	6.66667	.	.	intergenic	.	rs787682840	2
AC.GT	13	213552501	213552510	5	.	.	intergenic	0.217	.	2
AAAAC.GTTTT	13	213745621	213745650	6	ENSSSCT00000013204	DSCAM	intron	0.027	rs788468602	2
AC.GT	13	213969582	213969594	6.5	ENSSSCT00000013204	DSCAM	intron	-0.199	rs791766852	2
AAAT.ATTT	13	214189060	214189083	6	.	.	intergenic	-0.117	rs793643402	2
AC.GT	13	214216783	214216801	9.5	.	.	intergenic	0.065	rs793807641	2
AC.GT	13	214370798	214370825	14	.	.	intergenic	-0.135	rs713152239	3
AG.CT	13	214440756	214440765	5	.	.	intergenic	-0.113	.	3
AT.AT	13	214560631	214560642	6	.	.	intergenic	.	506734 rs7884	2
AC.GT	13	214919425	214919442	9	.	.	intergenic	-0.054	.	4
GGGGG.CCCCC	13	214999457	214999475	3.16667	ENSSSCT00000034038	MX1	intron	-0.288	rs791546487	2
GGGGG.CCCCC	13	214999457	214999475	3.16667	ENSSSCT00000034940	MX1	intron	-0.288	rs791546487	2
GGGGG.CCCCC	13	214999457	214999475	3.16667	ENSSSCT00000034771	MX1	intron	-0.288	rs791546487	2
GGGGG.CCCCC	13	214999457	214999475	3.16667	ENSSSCT00000013211	MX1	intron	-0.288	rs791546487	2
GGGGG.CCCCC	13	214999457	214999475	3.16667	ENSSSCT00000035557	MX1	promoter	-0.288	rs791546487	2
AG.CT	13	215023402	215023411	5	.	.	intergenic	.	356985 rs7871	2
AC.GT	13	215073406	215073428	11.5	ENSSSCT00000013208	FAM3B	promoter	.	125581 rs7869	4
AAAC.GTTT	13	215246630	215246648	4.75	.	.	intergenic	.	rs790450637	2
AC.GT	13	215474134	215474156	11.5	ENSSSCT00000013217	RIPK4	intron	-0.429	.	3
AAC.GTT	13	215481237	215481252	5.33333	.	.	intergenic	.	rs788724109	2
AAAC.GTTT	13	215486947	215486962	4	.	.	intergenic	.	rs790000996	2
AC.GT	13	216397426	216397438	6.5	ENSSSCT00000025792	PDE9A	intron	.	.	2
AAAC.GTTT	13	216544105	216544121	4.25	.	.	intergenic	0.221	rs791203542	2
ACACC.GGTGT	13	216636925	216636939	3	ENSSSCT00000028037	RRP1B	intron	-0.338	rs788508458	2
AGC.GCT	13	216744253	216744269	5.66667	.	.	intergenic	.	rs793284669	3
AAAAG.CTTTT	13	217284384	217284410	5.4	ENSSSCT00000025447	.	promoter	.	rs787075361	2
AC.GT	13	218139850	218139860	5.5	.	.	intergenic	.	rs789658886	2
AAAT.ATTT	14	491997	492010	3.5	.	.	intergenic	.	rs792157345	2
AT.AT	14	608566	608582	8.5	.	.	intergenic	.	rs793520912	2
AAAT.ATTT	14	778302	778324	5.75	.	.	intergenic	.	315963 rs7867	2
AC.GT	14	790321	790332	6	.	.	intergenic	.	rs786985176	2
AC.GT	14	1483716	1483727	6	.	.	intergenic	-0.355	516276 rs7881	3
AAAC.GTTT	14	1508227	1508249	5.75	.	.	intergenic	-0.117	289546 rs7897	2
AAAAT.ATTTT	14	1536844	1536881	7.6	.	.	intergenic	-0.212	rs789489002	2
AAAT.ATTT	14	1666663	1666677	3.75	.	.	intergenic	.	223218 rs7884	2
ACC.GGT	14	2018100	2018113	4.66667	.	.	intergenic	.	rs792925621	2
AG.CT	14	2737880	2737898	9.5	.	.	intergenic	.	360680 rs7867	2
AG.CT	14	2949759	2949771	6.5	.	.	intergenic	.	349589 rs7862	2
AC.GT	14	3051851	3051868	9	.	.	intergenic	-0.415	.	2
AC.GT	14	3055809	3055820	6	.	.	intergenic	-0.259	362035 rs7930	3
AAAC.GTTT	14	3183102	3183120	4.75	ENSSSCT00000010511	.	intron	.	rs792282895	2
AG.CT	14	3494595	3494626	16	.	.	intergenic	0.555	.	5
AAC.GTT	14	3538529	3538543	5	.	.	intergenic	-0.097	rs788624018	2
AT.AT	14	3673512	3673523	6	.	.	intergenic	0.148	rs787915505	2
AC.GT	14	3705230	3705246	8.5	.	.	intergenic	0.029	302860 rs6911	4
AGG.CCT	14	3736736	3736756	7	.	.	intergenic	-0.249	rs793085551	2
AAAAT.ATTTT	14	3810693	3810710	3.6	.	.	intergenic	.	.	2
AAAAC.GTTTT	14	3830594	3830612	3.16667	.	.	intergenic	0.714	rs792472184	2
AT.AT	14	3986912	3986923	6	ENSSSCT00000010516	SPTLC1	intron	-0.207	rs789511956	2
AAAAT.ATTTT	14	3992310	3992327	3.6	ENSSSCT00000010516	SPTLC1	intron	.	384429 rs7863	4
AAAC.GTTT	14	4097610	4097628	4.75	.	.	intergenic	.	rs793082015	2
AAAAT.ATTTT	14	4123637	4123655	3.8	.	.	intergenic	.	rs786403505	3
AAAAC.GTTTT	14	4139248	4139270	3.83333	.	.	intergenic	.	366311 rs7875	2
AC.GT	14	4298003	4298014	6	ENSSSCT00000010524	SLC18A1	intron	1.457	rs793824270	3
AATC.GATT	14	4482547	4482565	4.75	ENSSSCT00000010521	.	promoter	-0.047	.	2
AAAG.CTTT	14	4663716	4663744	7.25	ENSSSCT00000010526	.	intron	-0.145	002078 rs7936	2
AAAC.GTTT	14	4884657	4884673	4.25	.	.	intergenic	0.008	457611 rs7882	2
AT.AT	14	5047952	5047962	5.5	.	.	intergenic	-0.119	rs788832415	2
ACC.GGT	14	5073333	5073349	5.66667	.	.	intergenic	.	rs791904975	2
AAAC.GTTT	14	5482203	5482220	4.5	.	.	intergenic	0.168	965302 rs7869	2
AC.GT	14	5566676	5566686	5.5	.	.	intergenic	0.18	rs790193301	2
AG.CT	14	5577092	5577101	5	.	.	intergenic	.	.	2
AG.CT	14	5703411	5703420	5	.	.	intergenic	-0.06	.	2
AC.GT	14	6075191	6075200	5	ENSSSCT00000010528	GFRA2	intron	.	.	2
AATT.AATT	14	6779768	6779786	4.75	ENSSSCT00000010536	DMTN	intron	0.194	rs788835676	2
AAAG.CTTT	14	6868798	6868812	3.75	.	.	intergenic	0.07	349361 rs7896	3
AAC.GTT	14	7149260	7149274	5	.	.	intergenic	.	rs790209760	2

AT.AT	14	7346536	7346560	12.5	ENSSSCT00000010555	CCAR2	intron	0.164	rs786551605	2
AATG.CATT	14	7448191	7448211	5.25	ENSSSCT00000010558	PEBP4	intron	-0.023	rs792639896	2
AG.CT	14	7729173	7729187	7.5	.	.	intergenic	.	944106 rs7888	3
AAC.GTT	14	8409667	8409692	8.66667	.	.	intergenic	-0.317	rs793256105	2
AAAAC.GTTTT	14	8412002	8412031	6	.	.	intergenic	.	rs789983743	2
AG.CT	14	8946417	8946440	12	.	.	intergenic	0.071	rs791342469	4
AC.GT	14	8975508	8975518	5.5	.	.	intergenic	-0.077	.	3
AT.AT	14	9376934	9376944	5.5	.	.	intergenic	-0.032	.	2
AAC.GTT	14	9403020	9403039	6.66667	.	.	intergenic	-0.005	rs792614825	2
AAT.ATT	14	9584228	9584240	4.33333	.	.	intergenic	-0.077	rs787453619	2
AAAC.GTTT	14	9686706	9686720	3.75	.	.	intergenic	.	rs790177688	2
AC.GT	14	9703663	9703681	9.5	.	.	intergenic	0.032	.	2
AGAT.ATCT	14	9730791	9730802	3	.	.	intergenic	0.026	338996 rs7931	3
AT.AT	14	9913438	9913452	7.5	.	.	intergenic	.	.	3
AC.GT	14	10363270	10363282	6.5	.	.	intergenic	.	rs791593380	3
AAATG.CATTT	14	10569334	10569352	3.8	ENSSSCT00000010579	DOCK5	intron	-0.059	rs788392806	2
AT.AT	14	10747817	10747826	5	.	.	intergenic	0.016	rs691686128	3
AC.GT	14	10792086	10792096	5.5	.	.	intergenic	0.245	.	4
AC.GT	14	10816991	10817009	9.5	.	.	intergenic	0.043	rs789879639	5
AG.CT	14	10970817	10970829	6.5	.	.	intergenic	0.269	rs790529710	4
AC.GT	14	11114367	11114377	5.5	ENSSSCT00000010585	.	intron	1.097	.	2
AC.GT	14	11215250	11215261	6	.	.	intergenic	-0.924	984958 rs7879	2
AC.GT	14	11319149	11319161	6.5	.	.	intergenic	0.088	259853 rs7923	2
AAAC.GTTT	14	11456361	11456381	5.25	.	.	intergenic	.	rs792550318	2
ATC.GAT	14	11458887	11458911	8.33333	.	.	intergenic	.	rs788087016	2
AAAC.GTTT	14	11498762	11498776	3.75	ENSSSCT00000010590	BNIP3L	intron	.	rs788977131	2
AAGG.CCTT	14	11588508	11588526	4.75	.	.	intergenic	-0.032	220305 rs7915	2
ATCC.GGAT	14	11858503	11858531	7.25	ENSSSCT00000010593	ALPHA-1A	intron	0.162	.	2
AAAAC.GTTTT	14	11954616	11954636	4.2	.	.	intergenic	0.592	524917 rs7871	2
AAT.ATT	14	12000129	12000141	4.33333	.	.	intergenic	0.202	.	2
AAC.GTT	14	12524230	12524243	4.66667	ENSSSCT00000010601	CLU	intron	0.084	rs791657364	3
AAC.GTT	14	12524230	12524243	4.66667	ENSSSCT00000036649	CLU	intron	0.084	rs791657364	3
AAC.GTT	14	12524230	12524243	4.66667	ENSSSCT00000035611	CLU	intron	0.084	rs791657364	3
AAC.GTT	14	12524230	12524243	4.66667	ENSSSCT00000033441	CLU	intron	0.084	rs791657364	3
AAC.GTT	14	12524230	12524243	4.66667	ENSSSCT00000034107	CLU	intron	0.084	rs791657364	3
AC.GT	14	12570705	12570716	6	ENSSSCT00000025007	SCARA3	intron	.	rs789247999	4
AG.CT	14	12606676	12606687	6	.	.	intergenic	0.109	.	2
AAAT.ATTT	14	12690849	12690872	6	ENSSSCT00000010603	ESCO2	intron	1.388	rs787127579	2
AAAT.ATTT	14	12731791	12731809	4.75	ENSSSCT00000010604	PBK	intron	.	rs787919085	2
AC.GT	14	12800633	12800648	8	.	.	intergenic	.	rs789511203	2
AG.CT	14	13194254	13194266	6.5	.	.	intergenic	-0.144	rs790538808	2
AAAG.CTTT	14	13396395	13396407	3.25	ENSSSCT00000010612	FBXO16	intron	.	rs788573208	4
AC.GT	14	13420516	13420537	11	ENSSSCT00000010612	FBXO16	intron	0.113	rs788584938	2
AAAAC.GTTTT	14	13614573	13614593	4.2	.	.	intergenic	.	374685 rs7906	2
AGC.GCT	14	13620387	13620403	5.66667	.	.	intergenic	0.134	rs786240165	2
AAAAC.GTTTT	14	13637459	13637478	4	.	.	intergenic	0.087	.	2
AAG.CTT	14	13681054	13681066	4.33333	ENSSSCT00000010614	EXTL3	intron	0.031	.	2
AAAT.ATTT	14	13686069	13686099	7.75	ENSSSCT00000010614	EXTL3	intron	.	571316 rs7927	2
AAAC.GTTT	14	13714065	13714085	5.25	.	.	intergenic	-0.014	rs787364606	2
AT.AT	14	14031371	14031389	9.5	ENSSSCT00000010617	HMBOX1	intron	0.187	.	2
AT.AT	14	14031371	14031389	9.5	ENSSSCT00000010616	HMBOX1	intron	0.187	.	2
AC.GT	14	14158960	14158970	5.5	ENSSSCT00000010618	KIF13B	intron	-0.434	rs791671713	2
AC.GT	14	14225686	14225719	17	ENSSSCT00000010618	KIF13B	intron	0.79	241762 rs7869	3
AAAAG.CTTTT	14	14228378	14228394	3.4	ENSSSCT00000010618	KIF13B	intron	-0.064	.	2
AC.GT	14	14244067	14244084	9	ENSSSCT00000010618	KIF13B	intron	0.607	459424 rs7906	2
AG.CT	14	14257015	14257025	5.5	ENSSSCT00000010618	KIF13B	intron	-0.12	rs787173718	2
AC.GT	14	14318289	14318299	5.5	ENSSSCT00000010618	KIF13B	intron	0.134	.	2
AAT.ATT	14	14409639	14409658	6.66667	.	.	intergenic	.	rs787439291	2
AAAG.CTTT	14	14680014	14680035	5.5	ENSSSCT00000010619	MSRA	intron	0.664	rs792994866	2
AAAAT.ATTTT	14	14978734	14978770	7.4	ENSSSCT00000010619	MSRA	intron	0.012	rs786729596	2
ATC.GAT	14	15160949	15160963	5	.	.	intergenic	-0.166	rs790538630	3
AC.GT	14	15193452	15193472	10.5	.	.	intergenic	.	.	2
AAC.GTT	14	15421106	15421122	5.66667	ENSSSCT00000010627	PINX1	intron	-0.206	303208 rs7895	2
AAGG.CCTT	14	15601723	15601740	4.5	.	.	intergenic	0.848	151197 rs7910	2
AAC.GTT	14	15907738	15907754	5.66667	.	.	intergenic	.	rs789981557	3
AG.CT	14	15954581	15954593	6.5	.	.	intergenic	-0.524	.	3
AG.CT	14	15992530	15992544	7.5	.	.	intergenic	-0.067	.	2
AC.GT	14	16385033	16385047	7.5	.	.	intergenic	.	rs788738459	2
AAAAAC.GTTTTT	14	16473012	16473046	5.83333	.	.	intergenic	-0.08	162645 rs7873	2
AAAAC.GTTTT	14	16694142	16694160	3.8	.	.	intergenic	.	rs793771081	2
AAAC.GTTT	14	17074002	17074016	3.75	ENSSSCT00000010637	FBXO8	intron	0.074	rs790870194	2
AC.GT	14	17194365	17194386	11	.	.	intergenic	0.23	rs786685005	4
AAAG.CTTT	14	17331605	17331631	6.75	.	.	intergenic	0.569	254720 rs7875	2
AC.GT	14	17390198	17390210	6.5	.	.	intergenic	-0.048	rs791834067	3
AAAT.ATTT	14	17578013	17578027	3.75	.	.	intergenic	0.378	.	2
AC.GT	14	17785426	17785439	7	.	.	intergenic	.	355858 rs7934	3
AAAC.GTTT	14	17848733	17848749	4.25	.	.	intergenic	.	328175 rs7873	2
AAAC.GTTT	14	17922341	17922370	7.5	ENSSSCT00000010641	GALNT7	intron	-0.212	332852 rs7920	2
AC.GT	14	17941638	17941657	10	.	.	intergenic	-0.289	760526 rs7868	2
AAAG.CTTT	14	18018293	18018311	4.75	.	.	intergenic	0.579	rs694348998	2
AAAC.GTTT	14	18199148	18199168	5.25	.	.	intergenic	.	rs792350932	2
AAAT.ATTT	14	18204844	18204861	4.5	.	.	intergenic	.	rs788864451	2
AAT.ATT	14	18552646	18552659	4.66667	.	.	intergenic	-0.111	rs793629881	2
AAAAT.ATTTT	14	18563196	18563218	4.6	.	.	intergenic	0.609	rs791845355	2

AC.GT	14	18589239	18589251	6.5	.	.	intergenic	-0.011	521859 rs7864	2
AC.GT	14	18653884	18653898	7.5	.	.	intergenic	0.033	rs790245846	2
AAAAG.CTTTT	14	18752222	18752246	5	.	.	intergenic	.	362392 rs7059	2
AAC.GTT	14	18831613	18831635	7.66667	.	.	intergenic	-0.234	rs699130384	3
AATT.AATT	14	18841086	18841100	3.75	.	.	intergenic	-0.061	.	2
AAAC.GTTT	14	19042125	19042150	6.5	.	.	intergenic	-0.135	rs693177946	2
AC.GT	14	19088453	19088463	5.5	.	.	intergenic	0.215	rs692694970	2
AAAC.GTTT	14	19315090	19315115	6.5	.	.	intergenic	-0.226	.	2
AC.GT	14	19356786	19356796	5.5	.	.	intergenic	.	rs793080581	2
AAAC.GTTT	14	19429363	19429377	3.75	.	.	intergenic	0.034	rs789048652	2
AAAC.GTTT	14	19561761	19561777	4.25	.	.	intergenic	.	rs793238902	2
AC.GT	14	19569198	19569228	15.5	.	.	intergenic	.	949804 rs7918	4
AAAAG.CTTTT	14	19577819	19577847	5.8	.	.	intergenic	.	rs700674711	2
AC.GT	14	19758999	19759008	5	.	.	intergenic	.	.	2
AG.CT	14	19830168	19830197	15	.	.	intergenic	-0.136	rs789885277	3
AT.AT	14	20049437	20049453	8.5	.	.	intergenic	-0.041	rs787800268	2
AC.GT	14	20093861	20093871	5.5	.	.	intergenic	-0.268	rs790093673	2
AAGG.CCTT	14	20167717	20167732	4	.	.	intergenic	.	rs787530512	2
AC.GT	14	20319374	20319394	10.5	.	.	intergenic	.	rs787198196	2
AT.AT	14	20629761	20629770	5	.	.	intergenic	-0.222	.	4
AGAT.ATCT	14	20667271	20667290	5	.	.	intergenic	.	.	2
AT.AT	14	20808313	20808324	6	.	.	intergenic	.	rs793849147	2
AAAT.ATTT	14	20820541	20820556	4	.	.	intergenic	0.16	rs788371100	2
AAC.GTT	14	20862779	20862790	4	.	.	intergenic	.	rs792883743	2
AC.GT	14	20964314	20964330	8.5	.	.	intergenic	0.013	.	2
AC.GT	14	21025418	21025427	5	.	.	intergenic	.	rs695436509	4
AAAAC.GTTTT	14	21213633	21213654	3.66667	.	.	intergenic	0.06	799153 rs7898	2
AAAG.CTTT	14	21368830	21368848	4.75	ENSSSCT00000010649	CLCN3	intron	0.267	297380 rs7916	2
AG.CT	14	21412970	21412982	6.5	ENSSSCT00000027721	.	promoter	0.175	.	3
AC.GT	14	21451964	21451974	5.5	ENSSSCT00000010651	NEK1	intron	.	rs793443492	3
AC.GT	14	21451964	21451974	5.5	ENSSSCT00000010650	NEK1	intron	.	rs793443492	3
AAAAC.GTTTT	14	21505393	21505418	5.2	ENSSSCT00000010651	NEK1	intron	0.117	rs787036419	2
AAAAC.GTTTT	14	21505393	21505418	5.2	ENSSSCT00000010650	NEK1	intron	0.117	rs787036419	2
ACAG.CTGT	14	21648148	21648166	4.75	.	.	intergenic	.	rs788813491	2
AAAC.GTTT	14	21823957	21823980	6	ENSSSCT00000010652	SH3RF1	intron	.	rs792986608	2
AATC.GATT	14	21925644	21925660	4.25	ENSSSCT00000010653	CBR4	intron	.	rs792754673	2
AAAAT.ATTTT	14	21953362	21953392	6.2	.	.	intergenic	.	323792 rs7903	2
AC.GT	14	22005038	22005048	5.5	ENSSSCT00000022985	PALLD	intron	0.195	rs791130042	3
AT.AT	14	22280701	22280715	7.5	.	.	intergenic	0.228	.	2
AAC.GTT	14	22367009	22367025	5.66667	ENSSSCT00000010657	DDX60	promoter	0.068	142492 rs7879	4
AC.GT	14	22424991	22425016	13	.	.	intergenic	0.196	.	2
AC.GT	14	22605403	22605416	7	ENSSSCT00000028371	ANXA10	intron	0.133	rs788678528	2
AC.GT	14	22605403	22605416	7	ENSSSCT00000028014	ANXA10	intron	0.133	rs788678528	2
AT.AT	14	22641696	22641721	13	ENSSSCT00000028371	ANXA10	intron	-0.003	rs791995613	2
AT.AT	14	22641696	22641721	13	ENSSSCT00000028014	ANXA10	intron	-0.003	rs791995613	2
AT.AT	14	22741629	22741640	6	.	.	intergenic	0.086	rs790354788	2
AC.GT	14	22978953	22978971	9.5	.	.	intergenic	.	rs709886859	3
AGG.CCT	14	23029128	23029145	6	.	.	intergenic	0.06	491036 rs7926	3
AAAAC.GTTTT	14	23102685	23102706	4.4	ENSSSCT00000010659	SPOCK3	intron	.	275413 rs7875	3
AC.GT	14	23164160	23164176	8.5	ENSSSCT00000010659	SPOCK3	intron	-0.024	.	2
AC.GT	14	23210920	23210966	23.5	ENSSSCT00000010659	SPOCK3	intron	.	rs793855535	2
AC.GT	14	23256471	23256483	6.5	ENSSSCT00000010659	SPOCK3	intron	-0.033	.	3
AAAAAG.CTTTTT	14	23275043	23275065	3.83333	ENSSSCT00000010659	SPOCK3	intron	-0.133	rs789014607	2
AAAC.GTTT	14	23425674	23425692	4.75	ENSSSCT00000010659	SPOCK3	intron	.	rs791960229	2
AAAC.GTTT	14	23426916	23426937	5.5	ENSSSCT00000010659	SPOCK3	intron	.	111232 rs7885	2
AAT.ATT	14	23473095	23473110	5.33333	ENSSSCT00000010659	SPOCK3	intron	.	rs788100199	2
AG.CT	14	23575885	23575895	5.5	.	.	intergenic	.	rs792998908	2
AAAT.ATTT	14	23597428	23597450	5.75	.	.	intergenic	-0.194	070692 rs7893	2
AAAT.ATTT	14	23768268	23768285	4.5	.	.	intergenic	.	rs791902483	2
AAAAG.CTTTT	14	23781906	23781921	3.2	.	.	intergenic	.	948428 rs7879	2
AAC.GTT	14	23851398	23851415	6	.	.	intergenic	.	rs786991550	2
AAAC.GTTT	14	23851723	23851745	5.75	.	.	intergenic	.	071263 rs7916	2
AAAAAG.CTTTTT	14	24102116	24102134	3.16667	ENSSSCT00000010671	.	intron	.	rs705486960	2
AAAC.GTTT	14	24233232	24233254	5.75	.	.	intergenic	.	rs789414433	2
AAAC.GTTT	14	24524288	24524305	4.5	.	.	intergenic	.	rs791166863	2
AATG.CATT	14	24685331	24685353	5.75	ENSSSCT00000010677	GALNT9	intron	.	rs790960525	2
AT.AT	14	25766927	25766942	8	ENSSSCT00000010684	GPR133	intron	-0.529	.	2
AAAAC.GTTTT	14	25996283	25996297	3	.	.	intergenic	-0.094	rs790555062	2
AC.GT	14	26021986	26022000	7.5	.	.	intergenic	.	573898 rs7904	2
AAAT.ATTT	14	26022292	26022312	5.25	.	.	intergenic	.	rs788040466	2
AC.GT	14	26432079	26432098	10	.	.	intergenic	-0.538	rs792377483	3
AAAG.CTTT	14	26448401	26448431	7.75	.	.	intergenic	-0.047	rs786237452	2
CAGAG.CTCTG	14	26724931	26724954	4	.	.	intergenic	.	rs786415066	2
AT.AT	14	26889663	26889672	5	.	.	intergenic	-0.49	rs788859356	4
AC.GT	14	26905253	26905267	7.5	.	.	intergenic	.	529658 rs7882	2
AAGG.CCTT	14	27158328	27158360	8.25	.	.	intergenic	-0.205	rs786768159	2
AG.CT	14	27239740	27239749	5	.	.	intergenic	-0.32	.	3
AAAC.GTTT	14	27256606	27256620	3.75	.	.	intergenic	-0.307	982196 rs7918	2
AC.GT	14	27301601	27301616	8	.	.	intergenic	.	776856 rs7928	2
AT.AT	14	27322624	27322643	10	.	.	intergenic	.	045473 rs7927	2
AAAAC.GTTTT	14	27344249	27344280	6.4	.	.	intergenic	-0.193	rs788653703	2
AAAT.ATTT	14	27378811	27378825	3.75	.	.	intergenic	.	rs789132968	2
AAAC.GTTT	14	27403463	27403481	4.75	.	.	intergenic	-0.306	rs787427900	2
AC.GT	14	27444284	27444298	7.5	.	.	intergenic	-0.328	549421 rs7899	2

AC.GT	14	27794635	27794649	7.5	ENSSSCT00000031300	SLC15A4	intron	-0.284	rs792549984	2
AT.AT	14	27985563	27985573	5.5	ENSSSCT00000010692	.	intron	.	rs786416081	2
AG.CT	14	28111956	28111972	8.5	ENSSSCT00000010692	.	intron	-0.058	.	2
AAAT.ATTT	14	28259852	28259876	6.25	ENSSSCT00000010692	.	intron	-0.347	rs792344317	2
AG.CT	14	28342004	28342016	6.5	.	.	intergenic	-0.344	rs790440171	2
AAAAAC.GTTTTT	14	28362320	28362343	4	.	.	intergenic	.	rs788236202	2
AAAAC.GTTTT	14	28437992	28438020	5.8	.	.	intergenic	.	rs789802100	3
AAAC.GTTT	14	28449894	28449912	4.75	.	.	intergenic	-0.128	rs790890270	2
AAT.ATT	14	28473061	28473077	5.66667	.	.	intergenic	0.188	309163 rs7920	2
AATC.GATT	14	28578047	28578066	5	.	.	intergenic	.	rs786228866	2
AAG.CTT	14	28673450	28673466	5.66667	.	.	intergenic	-0.172	701323 rs7900	2
AAAC.GTTT	14	28689932	28689947	4	.	.	intergenic	-0.259	rs786903841	2
AC.GT	14	28731121	28731152	16	.	.	intergenic	-0.212	rs790154934	5
AAG.CTT	14	28825250	28825275	8.66667	.	.	intergenic	-0.243	224344 rs7919	4
AAG.CTT	14	28889873	28889906	11.3333	.	.	intergenic	-0.245	rs790224340	2
AAC.GTT	14	28963522	28963543	7.33333	.	.	intergenic	-0.348	rs793055253	2
AC.GT	14	29027868	29027878	5.5	.	.	intergenic	-0.01	rs793271609	2
AG.CT	14	29110487	29110503	8.5	.	.	intergenic	.	rs793088220	2
AAAC.GTTT	14	29212623	29212639	4.25	.	.	intergenic	-0.273	rs792247471	2
AAAAC.GTTTT	14	29385614	29385637	4.8	.	.	intergenic	-0.262	041514 rs7925	2
AT.AT	14	29485409	29485418	5	ENSSSCT00000010693	TMEM132E	intron	.	rs793380917	3
AT.AT	14	29492382	29492401	10	ENSSSCT00000010693	TMEM132E	intron	.	351348 rs7933	2
AAAC.GTTT	14	29547350	29547372	5.75	ENSSSCT00000010693	TMEM132E	intron	-0.263	.	2
AC.GT	14	29713365	29713384	10	.	.	intergenic	.	rs789449130	2
AAAAG.CTTTT	14	29792190	29792213	4.8	.	.	intergenic	0.063	rs789507887	2
AC.GT	14	29834340	29834349	5	ENSSSCT00000010695	AACS	intron	-0.873	rs791104563	2
AAAC.GTTT	14	30072056	30072074	4.75	ENSSSCT00000010699	SCARB1	intron	-0.318	rs787983158	2
ACACC.GGTGT	14	30624973	30624987	3	.	.	intergenic	-0.051	rs793553618	2
AT.AT	14	30895586	30895596	5.5	ENSSSCT00000010707	DNAH10	intron	1.273	rs791367805	2
AAAAT.ATTTT	14	31046795	31046810	3.2	ENSSSCT00000010711	GTF2H3	intron	0.024	.	2
AAAAT.ATTTT	14	31046795	31046810	3.2	ENSSSCT00000010712	EIF2B1	promoter	0.024	.	2
AATC.GATT	14	31094434	31094457	6	ENSSSCT00000010714	TMED2	promoter	0.26	rs790856767	2
AAAT.ATTT	14	31472204	31472226	5.75	ENSSSCT00000010723	PITPNM2	intron	-0.19	597962 rs7899	2
AG.CT	14	31497228	31497240	6.5	ENSSSCT00000010723	PITPNM2	intron	-0.374	.	2
AAG.CTT	14	31531640	31531656	5.66667	ENSSSCT00000010723	PITPNM2	intron	-0.15	974442 rs7935	2
AAAC.GTTT	14	31578479	31578493	3.75	ENSSSCT00000010725	OGFOD2	3'utr	0.761	206850 rs7891	2
AAAC.GTTT	14	31578479	31578493	3.75	ENSSSCT00000010724	ARL6IP4	promoter	0.761	206850 rs7891	2
AAC.GTT	14	31767757	31767771	5	ENSSSCT00000010730	DENR	intron	0.783	rs788325933	2
AAC.GTT	14	32459654	32459670	5.66667	.	.	intergenic	.	rs788828337	2
AAAAC.GTTTT	14	32469770	32469790	4.2	.	.	intergenic	1.984	rs789658381	2
AGG.CCT	14	32529921	32529937	5.66667	ENSSSCT00000010744	WDR66	intron	-0.342	rs791556020	2
AC.GT	14	32530718	32530742	12.5	ENSSSCT00000010744	WDR66	intron	-0.047	rs792585074	4
AC.GT	14	32728680	32728694	7.5	ENSSSCT00000010751	TMEM120E	intron	.	rs788492582	4
AC.GT	14	32977113	32977147	17.5	.	.	intergenic	-0.321	176531 rs7934	4
AT.AT	14	33103827	33103849	11.5	.	.	intergenic	.	rs793436139	2
AAT.ATT	14	33275436	33275451	5.33333	.	.	intergenic	-0.13	279469 rs7910	2
AAAT.ATTT	14	33463361	33463387	6.75	.	.	intergenic	.	.	2
AAAT.ATTT	14	33514604	33514637	8.5	.	.	intergenic	-0.134	rs786723958	3
AAAC.GTTT	14	33832291	33832312	5.5	ENSSSCT00000010768	PPTC7	intron	0.464	rs790637939	2
AAAT.ATTT	14	33885094	33885109	4	.	.	intergenic	.	rs790167385	2
AT.AT	14	33955323	33955336	7	.	.	intergenic	-0.15	rs787154267	2
CACGC.GCGTG	14	34054889	34054922	5.66667	.	.	intergenic	-0.028	305490 rs7888	2
AC.GT	14	34086389	34086405	8.5	ENSSSCT00000010773	CCDC63	intron	0.318	rs787190365	5
AAAC.GTTT	14	34095289	34095312	6	ENSSSCT00000010773	CCDC63	intron	-0.156	rs786319378	2
AC.GT	14	34115278	34115290	6.5	ENSSSCT00000010773	CCDC63	intron	-0.229	550690 rs7887	2
AC.GT	14	34319820	34319834	7.5	.	.	intergenic	-0.129	rs791342430	2
AG.CT	14	35014844	35014854	5.5	.	.	intergenic	0.049	507804 rs7915	3
AAC.GTT	14	35034973	35034992	6.66667	ENSSSCT00000010783	.	intron	0.046	rs787110337	3
AAC.GTT	14	35047205	35047226	7.33333	ENSSSCT00000010783	.	intron	0.259	rs787303554	2
AAAC.GTTT	14	35090480	35090502	5.75	ENSSSCT00000032561	.	intron	.	295117 rs7913	2
AAAC.GTTT	14	35090480	35090502	5.75	ENSSSCT00000010783	.	intron	.	295117 rs7913	2
AT.AT	14	35184594	35184604	5.5	.	.	intergenic	-0.021	.	4
AAGG.CCTT	14	35316114	35316135	5.5	ENSSSCT00000010786	CCDC60	intron	.	.	2
AAGG.CCTT	14	35432144	35432166	5.75	.	.	intergenic	-0.083	rs787131861	3
AAAC.GTTT	14	35472678	35472697	5	.	.	intergenic	-0.059	310811 rs7879	2
AAAC.GTTT	14	35606563	35606586	6	ENSSSCT00000010791	.	intron	0.019	rs787153454	2
AAAC.GTTT	14	35636920	35636951	8	.	.	intergenic	-0.18	rs786665708	2
AAAC.GTTT	14	35846325	35846339	3.75	.	.	intergenic	.	305515 rs7884	2
AG.CT	14	36035650	36035659	5	.	.	intergenic	-0.372	740847 rs7929	2
AAAAAT.ATTTTT	14	36067667	36067689	3.83333	.	.	intergenic	0.031	048625 rs7934	2
AAG.CTT	14	36137721	36137742	7.33333	.	.	intergenic	0.224	731484 rs7896	2
AAAAC.GTTTT	14	36162962	36162990	5.8	.	.	intergenic	.	rs786410909	2
AC.GT	14	36253606	36253616	5.5	.	.	intergenic	0.042	.	2
AG.CT	14	36416628	36416645	9	ENSSSCT00000010795	SUDS3	intron	-0.16	rs789423461	2
AAAC.GTTT	14	36468295	36468314	5	ENSSSCT00000010796	TAOK3	intron	0.252	924293 rs7868	2
AAC.GTT	14	36675923	36675947	8.33333	.	.	intergenic	0.135	.	2
AAAC.GTTT	14	36801165	36801183	4.75	ENSSSCT00000010801	RFC5	intron	3.75	787379 rs7937	2
AAAAAC.GTTTTT	14	36880274	36880291	3	.	.	intergenic	.	rs789551600	2
AAAAC.GTTTT	14	36884560	36884583	4.8	.	.	intergenic	0.075	316121 rs7893	2
AAAT.ATTT	14	37041926	37041942	4.25	.	.	intergenic	.	rs786544802	2
AAC.GTT	14	37049149	37049164	5.33333	ENSSSCT00000010802	KSR2	promoter	.	.	2
AG.CT	14	37207730	37207746	8.5	ENSSSCT00000010802	KSR2	intron	0.14	rs786724561	3
ACACAT.ATGTGT	14	37300488	37300508	3.5	ENSSSCT00000010802	KSR2	intron	-0.131	592364 rs7938	2
AC.GT	14	37365171	37365183	6.5	.	.	intergenic	0.098	.	3

AC.GT	14	37708265	37708283	9.5	ENSSSCT00000010808	FBXW8	intron	.	rs789012904	2
AC.GT	14	37796511	37796534	12	ENSSSCT00000010808	FBXW8	promoter	.	rs787879530	4
AAAC.GTTT	14	37865409	37865427	4.75	ENSSSCT00000010810	RNFT2	intron	0.657	rs792049674	2
AAT.ATT	14	37899157	37899173	5.66667	ENSSSCT00000010810	RNFT2	intron	.	rs792810188	2
AAAG.CTTT	14	38104877	38104899	5.75	.	.	intergenic	0.178	rs704686469	3
AAAT.ATTT	14	38190417	38190429	3.25	.	.	intergenic	0.036	.	2
AATG.CATT	14	38226107	38226140	8.5	.	.	intergenic	0.152	448110 rs7914	2
AC.GT	14	38835985	38835996	6	.	.	intergenic	-0.126	351490 rs7925	2
ATC.GAT	14	38990094	38990118	8.33333	.	.	intergenic	-0.036	rs791452340	2
AC.GT	14	39128627	39128643	8.5	.	.	intergenic	-0.135	rs793269571	2
AC.GT	14	39160406	39160417	6	.	.	intergenic	-0.112	rs789341322	2
AC.GT	14	39560869	39560879	5.5	.	.	intergenic	-1.032	080831 rs7891	2
AAAAC.GTTTT	14	39887268	39887296	5.8	.	.	intergenic	-0.182	rs790707045	2
AAG.CTT	14	39942876	39942911	12	.	.	intergenic	.	rs790947642	2
AT.AT	14	40190075	40190086	6	.	.	intergenic	.	143107 rs7928	2
ACC.GGT	14	40204379	40204397	6.33333	.	.	intergenic	-0.386	339528 rs7872	3
AAAC.GTTT	14	40406374	40406393	5	.	.	intergenic	0.07	932376 rs7936	3
AGC.GCT	14	40428174	40428186	4.33333	.	.	intergenic	-0.206	.	2
AC.GT	14	40624618	40624627	5	ENSSSCT00000010816	RBM19	intron	0.223	553828 rs7893	2
AAC.GTT	14	40758863	40758875	4.33333	.	.	intergenic	.	399829 rs7914	2
AAAG.CTTT	14	41140437	41140459	5.75	ENSSSCT00000010826	IQCD	promoter	.	rs792780690	2
AG.CT	14	41178119	41178138	10	ENSSSCT00000028773	.	intron	-0.05	rs702371938	2
AC.GT	14	41197041	41197053	6.5	ENSSSCT00000010831	OAS2	intron	0.011	.	2
AG.CT	14	41396191	41396202	6	.	.	intergenic	-0.162	.	2
AAAG.CTTT	14	41740851	41740875	6.25	ENSSSCT00000030697	PTPN11	intron	.	rs787756673	2
AAAT.ATTT	14	41802040	41802062	5.75	.	.	intergenic	0.233	rs706831724	2
AAAT.ATTT	14	42047711	42047724	3.5	ENSSSCT00000036207	TRAFD1	intron	.	rs793007621	2
AATT.AATT	14	42246914	42246928	3.75	ENSSSCT00000010843	TMEM116	intron	.	rs787524876	2
AATG.CATT	14	42293366	42293396	7.75	ENSSSCT00000010843	TMEM116	3'utr	0.347	rs786443693	2
AC.GT	14	42714187	42714202	8	ENSSSCT00000010848	GCN1L1	3'utr	1.687	rs787251512	4
AC.GT	14	42714187	42714202	8	ENSSSCT00000031208	PLA2G1B	intron	1.687	rs787251512	4
AAAAAG.CTTTTT	14	42859192	42859214	3.83333	.	.	intergenic	.	rs787301223	2
AAG.CTT	14	43256748	43256767	6.66667	ENSSSCT00000010867	SPPL3	intron	0.342	353217 rs7874	2
AAG.CTT	14	43256748	43256767	6.66667	ENSSSCT00000010868	SPPL3	intron	0.342	353217 rs7874	2
AG.CT	14	43907937	43907948	6	ENSSSCT00000010885	UBE3B	intron	-0.162	.	3
AAAAC.GTTTT	14	43915620	43915643	4.8	ENSSSCT00000010885	UBE3B	intron	-0.292	rs792842594	2
AATG.CATT	14	44190440	44190461	5.5	ENSSSCT00000010889	ACACB	intron	-0.214	005232 rs7868	2
AAAC.GTTT	14	44274738	44274757	5	.	.	intergenic	0.033	rs786309900	2
ACAT.ATGT	14	44437688	44437710	5.75	ENSSSCT00000010893	SVOP	intron	.	561055 rs7877	2
AAAC.GTTT	14	44445092	44445111	5	ENSSSCT00000010893	SVOP	intron	-0.137	rs793124446	2
AAG.CTT	14	44469823	44469837	5	.	.	intergenic	0.21	rs788106308	2
AC.GT	14	44529382	44529397	8	ENSSSCT00000010895	SSH1	intron	-0.261	143326 rs7883	2
AAAT.ATTT	14	44619629	44619646	4.5	.	.	intergenic	0.278	rs786693161	2
AAAAC.GTTTT	14	44825679	44825698	4	.	.	intergenic	0.38	172457 rs7894	2
AGG.CCT	14	45178781	45178801	7	ENSSSCT00000025264	.	cds	1.204	.	2
ACATAT.ATATGT	14	45559715	45559733	3.16667	.	.	intergenic	.	079489 rs7888	2
AAAAT.ATTTT	14	45580927	45580948	4.4	ENSSSCT00000010902	PIWIL3	intron	0.252	585741 rs7911	2
AAAAT.ATTTT	14	45580927	45580948	4.4	ENSSSCT00000010901	PIWIL3	intron	0.252	585741 rs7911	2
AG.CT	14	45711196	45711206	5.5	.	.	intergenic	0.193	rs792999089	2
AAT.ATT	14	46087367	46087378	4	.	.	intergenic	0.017	rs791493530	2
AT.AT	14	46120815	46120827	6.5	.	.	intergenic	0.299	rs793335668	2
AGATG.CATCT	14	46291799	46291828	6	ENSSSCT00000010912	MYO18B	promoter	0.389	rs788284879	2
AG.CT	14	46672192	46672207	8	.	.	intergenic	-0.116	.	2
AGGG.CCCT	14	46730717	46730731	3.75	.	.	intergenic	-0.106	rs792914076	2
AG.CT	14	46878423	46878432	5	ENSSSCT00000010913	SEZ6L	intron	0.838	.	2
ACAG.CTGT	14	47089193	47089219	6.75	.	.	intergenic	-0.401	744720 rs7916	2
AG.CT	14	47186504	47186519	8	.	.	intergenic	0.058	rs790713086	2
AAAAC.GTTTT	14	47190251	47190270	4	.	.	intergenic	-0.432	348211 rs7868	2
AAAT.ATTT	14	47242402	47242419	4.5	.	.	intergenic	-0.35	493714 rs7916	2
AG.CT	14	47388628	47388642	7.5	.	.	intergenic	.	909771 rs7877	2
AAAG.CTTT	14	47587267	47587281	3.75	.	.	intergenic	-0.17	rs793127006	2
AC.GT	14	47754732	47754746	7.5	.	.	intergenic	-0.041	232411 rs7937	2
AC.GT	14	48195004	48195015	6	.	.	intergenic	.	080170 rs7893	4
AC.GT	14	48470305	48470323	9.5	ENSSSCT00000010924	TTC28	intron	.	.	2
AAG.CTT	14	48797958	48797976	6.33333	.	.	intergenic	.	rs786797027	2
AAC.GTT	14	48906003	48906019	5.66667	.	.	intergenic	-0.046	rs791950653	2
AT.AT	14	48916516	48916526	5.5	.	.	intergenic	.	.	2
AAAAC.GTTTT	14	49155954	49155976	4.6	.	.	intergenic	.	rs793796845	2
AAAAG.CTTTT	14	49224464	49224481	3.6	ENSSSCT00000010928	KREMEN1	intron	0.918	362256 rs7935	2
AC.GT	14	49252594	49252608	7.5	ENSSSCT00000010928	KREMEN1	intron	0.157	.	2
AAC.GTT	14	49279601	49279617	5.66667	ENSSSCT00000010928	KREMEN1	intron	3.551	rs791359179	2
AG.CT	14	49659653	49659662	5	ENSSSCT00000010945	NIPSNAP1	intron	0.961	.	2
AT.AT	14	50047974	50047994	10.5	ENSSSCT00000010951	MTMR3	intron	.	.	2
AG.CT	14	50049213	50049228	8	ENSSSCT00000010951	MTMR3	intron	0.134	.	3
AAC.GTT	14	50107447	50107460	4.66667	.	.	intergenic	0.327	.	2
AG.CT	14	51166637	51166648	6	.	.	intergenic	0.368	.	2
AAAT.ATTT	14	51300782	51300799	4.5	ENSSSCT00000010986	DRG1	intron	.	rs791167560	2
AAAC.GTTT	14	51733380	51733403	6	ENSSSCT00000010991	DEPDC5	intron	.	200311 rs7923	2
AAAAT.ATTTT	14	52149971	52149987	3.4	.	.	intergenic	0.116	307926 rs7868	2
AAAC.GTTT	14	52263311	52263335	6.25	.	.	intergenic	-0.344	309737 rs7924	2
AC.GT	14	53392097	53392119	11.5	.	.	intergenic	.	rs790645052	2
AAT.ATT	14	53997385	53997401	5.66667	ENSSSCT00000011056	CRKL	intron	0.196	rs792322926	2
AAAAC.GTTTT	14	54660068	54660091	4.8	.	.	intergenic	0.254	709429 rs7913	2
AG.CT	14	55466834	55466843	5	.	.	intergenic	-0.295	.	2

AAAAC.GTTTT	14	55561108	55561129	4.4	.	.	intergenic	-0.019	334216 rs7927	2
AAAG.CTTT	14	55617123	55617141	4.75	.	.	intergenic	-0.397	003832 rs7924	2
AT.AT	14	55629490	55629499	5	.	.	intergenic	0.003	rs786774931	2
AAAT.ATTT	14	55895676	55895691	4	.	.	intergenic	-0.365	011153 rs7889	2
AAC.GTT	14	55949181	55949197	5.66667	.	.	intergenic	-0.158	.	2
AAC.GTT	14	55951230	55951246	5.66667	.	.	intergenic	-0.122	rs793024995	2
AAAC.GTTT	14	56118621	56118637	4.25	.	.	intergenic	0.026	rs787524929	2
AT.AT	14	56261061	56261072	6	.	.	intergenic	-0.145	.	2
AC.GT	14	56382889	56382898	5	.	.	intergenic	-0.167	rs790903453	3
AT.AT	14	56461715	56461732	9	.	.	intergenic	-0.068	rs792627774	2
AAG.CTT	14	56463037	56463050	4.66667	.	.	intergenic	-0.092	rs789512375	2
AAAC.GTTT	14	56604111	56604127	4.25	.	.	intergenic	0.029	rs786657623	2
AAAC.GTTT	14	57373211	57373222	3	.	.	intergenic	.	.	2
AATG.CATT	14	57479662	57479681	5	.	.	intergenic	-0.074	rs786286746	2
AT.AT	14	57607025	57607037	6.5	ENSSSCT00000011103	RYR2	intron	.	963039 rs7937	4
AAC.GTT	14	57736288	57736304	5.66667	ENSSSCT00000011103	RYR2	intron	-0.233	rs787859271	2
AT.AT	14	57785478	57785489	6	ENSSSCT00000011103	RYR2	intron	-0.174	rs790185061	2
AAAAT.ATTTT	14	57793380	57793403	4.8	ENSSSCT00000011103	RYR2	intron	-0.208	731952 rs7930	2
AC.GT	14	57806744	57806758	7.5	ENSSSCT00000011103	RYR2	intron	-0.398	.	4
AC.GT	14	58004232	58004242	5.5	.	.	intergenic	-0.167	rs792604062	2
AT.AT	14	58324508	58324518	5.5	.	.	intergenic	.	rs787725610	2
AAAAT.ATTTT	14	58382575	58382595	4.2	.	.	intergenic	0.133	rs792053458	2
AG.CT	14	58587129	58587147	9.5	ENSSSCT00000011105	MTR	intron	0.347	rs787918567	2
AG.CT	14	58598233	58598243	5.5	ENSSSCT00000011105	MTR	intron	-0.104	rs792269431	2
AAAC.GTTT	14	58629044	58629058	3.75	.	.	intergenic	0.289	525766 rs7878	2
AG.CT	14	58911423	58911443	10.5	ENSSSCT00000030665	EDARADD	intron	0.145	rs788693512	2
ATCC.GGAT	14	58936981	58937003	5.75	ENSSSCT00000030665	EDARADD	intron	0.004	517874 rs7915	2
AAAAG.CTTTT	14	59080753	59080769	3.4	ENSSSCT00000011111	ERO1LB	promoter	0.008	512501 rs7912	2
AAAG.CTTT	14	59264227	59264249	5.75	ENSSSCT00000011112	.	intron	.	786373 rs7862	3
AAAG.CTTT	14	59264227	59264249	5.75	ENSSSCT00000011114	.	intron	.	786373 rs7862	3
AAAC.GTTT	14	59280607	59280618	3	ENSSSCT00000011112	.	intron	.	rs789456907	2
AAAC.GTTT	14	59280607	59280618	3	ENSSSCT00000011114	.	intron	.	rs789456907	2
AAAT.ATTT	14	59292865	59292883	4.75	ENSSSCT00000011112	.	intron	-0.134	rs789541292	2
AAAC.GTTT	14	59526486	59526502	4.25	.	.	intergenic	0.093	rs792408674	2
AC.GT	14	59661432	59661446	7.5	ENSSSCT00000011115	LYST	intron	0.169	582102 rs7932	2
AT.AT	14	59696807	59696817	5.5	.	.	intergenic	-0.097	rs786306465	2
AAG.CTT	14	59808750	59808763	4.66667	.	.	intergenic	.	345200 rs7897	2
AAAC.GTTT	14	59845277	59845306	7.5	.	.	intergenic	-0.007	292281 rs7909	2
AC.GT	14	60014694	60014711	9	.	.	intergenic	.	.	2
AAAT.ATTT	14	60093206	60093224	4.75	ENSSSCT00000011120	ARID4B	intron	1.154	rs787868089	2
AT.AT	14	60642261	60642297	18.5	.	.	intergenic	.	rs792712968	2
AAAG.CTTT	14	60698437	60698453	4.25	.	.	intergenic	0.32	rs789424707	2
AAAAG.CTTTT	14	60796641	60796663	4.6	ENSSSCT00000011124	TARBP1	intron	0.188	959279 rs7913	2
AG.CT	14	60818495	60818507	6.5	.	.	intergenic	0.267	.	2
AT.AT	14	60886581	60886602	11	ENSSSCT00000011127	SLC35F3	intron	.	rs792321760	2
AC.GT	14	60886985	60886997	6.5	ENSSSCT00000011127	SLC35F3	intron	-0.011	rs789536151	2
AAAT.ATTT	14	60922251	60922273	5.75	ENSSSCT00000011127	SLC35F3	intron	-0.136	rs786274974	2
AAAAG.CTTTT	14	60967650	60967674	5	.	.	intergenic	-0.073	rs789105536	2
ACCT.AGGT	14	61088156	61088186	7.75	.	.	intergenic	0.046	799633 rs7897	2
AT.AT	14	61107249	61107259	5.5	.	.	intergenic	-0.257	rs786726686	3
AT.AT	14	61400723	61400734	6	.	.	intergenic	0.15	rs787819139	2
AG.CT	14	61585177	61585193	8.5	.	.	intergenic	.	.	3
AAC.GTT	14	61607126	61607139	4.66667	ENSSSCT00000011130	.	intron	-0.144	rs789533263	3
AC.GT	14	61858659	61858682	12	ENSSSCT00000026272	PCNXL2	intron	.	rs790514914	2
AC.GT	14	61858659	61858682	12	ENSSSCT00000011131	PCNXL2	intron	.	rs790514914	2
AC.GT	14	62053365	62053375	5.5	.	.	intergenic	0.054	.	2
AAAATT.AATTTT	14	62297429	62297459	5.16667	.	.	intergenic	-0.033	.	2
AATT.AATT	14	62299697	62299709	3.25	.	.	intergenic	-0.02	rs791026695	2
AAAC.GTTT	14	63624089	63624112	6	.	.	intergenic	.	rs792424976	2
AAAT.ATTT	14	63908512	63908533	5.5	ENSSSCT00000011142	C1orf131	intron	-0.12	222599 rs7896	2
AC.GT	14	64356815	64356828	7	ENSSSCT00000011149	COG2	intron	-0.132	147338 rs7913	2
AC.GT	14	64514633	64514656	12	.	.	intergenic	0.12	929451 rs7895	2
AAAAC.GTTTT	14	64702165	64702185	4.2	.	.	intergenic	.	rs789287231	2
AC.GT	14	64912818	64912832	7.5	ENSSSCT00000011151	GALNT2	intron	-0.189	rs789489793	2
AAAAC.GTTTT	14	65140502	65140530	5.8	ENSSSCT00000011154	ABCB10	intron	.	rs787373638	2
AC.GT	14	65552838	65552850	6.5	.	.	intergenic	0.116	rs791210534	2
ATC.GAT	14	65565541	65565559	6.33333	.	.	intergenic	.	.	2
AAAG.CTTT	14	65653747	65653761	3.75	ENSSSCT00000023762	ZNF33B	intron	.	rs789057917	2
AAAAAC.GTTTTT	14	65818104	65818129	4.33333	ENSSSCT00000011162	.	intron	.	328826 rs7872	2
AT.AT	14	66111694	66111718	12.5	.	.	intergenic	0.093	195140 rs7883	3
AAAC.GTTT	14	66118693	66118717	6.25	.	.	intergenic	0.167	075810 rs7877	2
AAAC.GTTT	14	66217529	66217548	5	ENSSSCT00000011166	SGALNACT	intron	0.284	964553 rs7874	2
AGAT.ATCT	14	66240465	66240484	5	ENSSSCT00000011166	SGALNACT	intron	0.17	382214 rs7899	2
AAG.CTT	14	66405489	66405512	8	.	.	intergenic	.	rs788739417	4
AAAT.ATTT	14	67031394	67031411	4.5	.	.	intergenic	0.011	.	2
AAC.GTT	14	67463260	67463275	5.33333	.	.	intergenic	.	rs787408063	2
AAAAAC.GTTTTT	14	67465040	67465065	4.33333	.	.	intergenic	0.124	.	2
AAAT.ATTT	14	67497612	67497623	3	.	.	intergenic	0.952	245449 rs7878	2
ATC.GAT	14	67502697	67502712	5.33333	ENSSSCT00000011175	FAM13C	intron	0.186	658724 rs7871	2
AG.CT	14	67702152	67702163	6	.	.	intergenic	-0.13	rs787404107	2
AAAAT.ATTTT	14	67746438	67746465	5.6	.	.	intergenic	.	rs787011245	2
AC.GT	14	68353956	68353968	6.5	.	.	intergenic	.	203044 rs7867	3
ATCC.GGAT	14	68565028	68565055	7	ENSSSCT00000035859	ANK3	intron	1.869	rs793886336	2
ATCC.GGAT	14	68565028	68565055	7	ENSSSCT00000033954	ANK3	intron	1.869	rs793886336	2

ATCC.GGAT	14	68565028	68565055	7	ENSSSCT00000011178	ANK3	intron	1.869	rs793886336	2
ATCC.GGAT	14	68565028	68565055	7	ENSSSCT00000034950	ANK3	intron	1.869	rs793886336	2
AC.GT	14	68576094	68576115	11	ENSSSCT00000035859	ANK3	intron	-0.097	.	4
AC.GT	14	68576094	68576115	11	ENSSSCT00000033954	ANK3	intron	-0.097	.	4
AC.GT	14	68576094	68576115	11	ENSSSCT00000011178	ANK3	intron	-0.097	.	4
AC.GT	14	68576094	68576115	11	ENSSSCT00000034950	ANK3	intron	-0.097	.	4
AAGG.CCTT	14	68842892	68842918	6.75	.	.	intergenic	0.014	305094 rs7886	2
AAC.GTT	14	68922243	68922260	6	.	.	intergenic	0.123	rs792992330	2
AAAAC.GTTTT	14	68991509	68991529	4.2	.	.	intergenic	0.205	rs790618478	2
AAAT.ATTT	14	69033177	69033194	4.5	.	.	intergenic	-0.066	rs786709721	3
AC.GT	14	69034895	69034919	12.5	.	.	intergenic	.	206841 rs7910	3
AAC.GTT	14	69050442	69050455	4.66667	.	.	intergenic	0.244	rs787770105	2
AC.GT	14	69090986	69090996	5.5	.	.	intergenic	.	.	2
AAAAC.GTTTT	14	69537438	69537466	5.8	.	.	intergenic	-0.017	rs786340202	2
AAAT.ATTT	14	69679552	69679569	4.5	.	.	intergenic	-0.09	012970 rs7938	2
AAAAT.ATTTT	14	69814080	69814098	3.8	.	.	intergenic	0.098	720916 rs7902	2
AT.AT	14	69852533	69852543	5.5	.	.	intergenic	0.489	rs791015605	2
AAAT.ATTT	14	69933898	69933918	5.25	.	.	intergenic	-0.104	.	2
AG.CT	14	70234861	70234871	5.5	.	.	intergenic	.	rs789031676	2
AC.GT	14	70253450	70253462	6.5	.	.	intergenic	0.141	rs792916873	3
AC.GT	14	70407149	70407161	6.5	.	.	intergenic	.	965633 rs7934	2
AAG.CTT	14	70573041	70573057	5.66667	.	.	intergenic	-0.134	240374 rs7933	2
AAAC.GTTT	14	70720505	70720525	5.25	.	.	intergenic	.	544143 rs7866	2
AT.AT	14	70930847	70930864	9	.	.	intergenic	.	rs790855693	2
AT.AT	14	70938686	70938696	5.5	ENSSSCT00000011188	ZNF365	intron	0.288	rs787896863	2
AATG.CATT	14	71284906	71284934	7.25	.	.	intergenic	0.04	rs788248513	2
AAAAC.GTTTT	14	71326612	71326645	6.8	.	.	intergenic	0.11	941555 rs7895	2
AT.AT	14	71651129	71651138	5	.	.	intergenic	.	.	2
AC.GT	14	71659411	71659421	5.5	.	.	intergenic	-0.11	rs792978989	2
AAAT.ATTT	14	71743493	71743514	5.5	.	.	intergenic	.	rs790387962	2
AC.GT	14	71828414	71828423	5	.	.	intergenic	0.273	rs790775511	3
AAAT.ATTT	14	71896763	71896787	6.25	ENSSSCT00000011192	JMJD1C	intron	3.567	062750 rs7938	2
AAAAAC.GTTTTT	14	71973501	71973531	5.16667	.	.	intergenic	0.152	003473 rs7862	2
AAAAAC.GTTTTT	14	72022208	72022231	4	.	.	intergenic	.	.	2
AAAC.GTTT	14	72143776	72143804	7.25	.	.	intergenic	.	rs790150268	2
AAAC.GTTT	14	72328157	72328180	6	.	.	intergenic	0.054	rs788443623	2
AC.GT	14	72377526	72377540	7.5	.	.	intergenic	0.355	289795 rs7936	2
AG.CT	14	72589187	72589210	12	.	.	intergenic	0.344	rs791874533	3
AT.AT	14	72623243	72623252	5	.	.	intergenic	.	rs786352749	3
AC.GT	14	72627660	72627672	6.5	.	.	intergenic	0.128	rs788346881	2
AAT.ATT	14	72901475	72901493	6.33333	.	.	intergenic	-0.18	.	2
AC.GT	14	72935348	72935358	5.5	.	.	intergenic	0.02	rs787738198	4
AC.GT	14	72954610	72954623	7	.	.	intergenic	.	rs788446582	2
AATAT.ATATT	14	73028434	73028449	3.2	.	.	intergenic	.	558431 rs7893	2
AAC.GTT	14	73144093	73144105	4.33333	.	.	intergenic	.	.	2
AAAAC.GTTTT	14	73191458	73191500	8.6	.	.	intergenic	.	181302 rs7862	2
AAAC.GTTT	14	73354317	73354334	4.5	.	.	intergenic	0.045	rs790456899	4
AT.AT	14	73472766	73472792	13.5	.	.	intergenic	.	247343 rs7913	2
ACCT.AGGT	14	73511764	73511783	5	.	.	intergenic	0.423	rs789465383	2
AAC.GTT	14	73545303	73545323	7	.	.	intergenic	0.018	384399 rs7915	2
AAAT.ATTT	14	73644145	73644165	5.25	.	.	intergenic	0.06	.	2
AAAT.ATTT	14	73659194	73659212	4.75	.	.	intergenic	.	rs792268086	2
AAAC.GTTT	14	73669069	73669081	3.25	.	.	intergenic	.	378525 rs7928	2
AT.AT	14	73814234	73814244	5.5	.	.	intergenic	0.15	rs790273431	2
AT.AT	14	74224832	74224842	5.5	.	.	intergenic	.	rs789960881	2
ACATAT.ATATGT	14	74301281	74301307	4.5	.	.	intergenic	-0.063	rs791649817	2
AC.GT	14	74988612	74988622	5.5	.	.	intergenic	.	.	2
AC.GT	14	75387469	75387502	17	.	.	intergenic	0.276	.	2
AAAAC.GTTTT	14	75581200	75581218	3.8	.	.	intergenic	0.174	rs788113688	2
AAAAC.GTTTT	14	75634451	75634469	3.8	.	.	intergenic	0.051	rs791234685	2
AG.CT	14	75763495	75763508	7	.	.	intergenic	0.391	.	4
AAAT.ATTT	14	75833610	75833632	5.75	.	.	intergenic	-0.045	153304 rs7873	2
AAAC.GTTT	14	75977367	75977393	6.75	.	.	intergenic	0.006	960376 rs7915	2
AAAC.GTTT	14	76016891	76016905	3.75	.	.	intergenic	0.105	rs791761972	2
AAAT.ATTT	14	76034957	76034976	5	.	.	intergenic	-0.209	rs786710025	2
AG.CT	14	76048170	76048181	6	.	.	intergenic	-0.123	rs792597052	3
AAAAG.CTTTT	14	76280556	76280572	3.4	.	.	intergenic	0.227	rs790739385	2
AT.AT	14	76388256	76388265	5	.	.	intergenic	0.282	rs791383088	2
AT.AT	14	76418829	76418839	5.5	.	.	intergenic	.	.	3
AAAACC.GGTTTT	14	76830991	76831011	3.5	.	.	intergenic	0.167	rs792699909	2
AAC.GTT	14	77057385	77057396	4	ENSSSCT00000011202	SIRT1	intron	0.154	rs791570588	2
AAT.ATT	14	78398506	78398521	5.33333	ENSSSCT00000024082	.	promoter	-0.157	rs786501554	2
AAAT.ATTT	14	78404085	78404103	4.75	ENSSSCT00000024082	.	intron	-0.096	rs791726745	2
AAAAC.GTTTT	14	78434191	78434211	4.2	ENSSSCT00000024082	.	intron	-0.206	rs787640133	3
AC.GT	14	78864023	78864033	5.5	ENSSSCT00000011226	COL13A1	intron	-0.035	rs791780673	2
AAAAT.ATTTT	14	79005244	79005264	4.2	ENSSSCT00000011229	TYSND1	intron	-0.131	rs790493403	2
AAAAG.CTTTT	14	79032162	79032181	4	.	.	intergenic	0.175	446658 rs7901	2
AAATG.CATTT	14	79034210	79034227	3.6	.	.	intergenic	.	rs793203243	2
AT.AT	14	79398856	79398867	6	.	.	intergenic	.	rs787087365	2
AC.GT	14	79712021	79712037	8.5	.	.	intergenic	-0.111	570206 rs7920	3
AT.AT	14	79793432	79793443	6	.	.	intergenic	-0.113	rs791656049	2
AC.GT	14	79811844	79811859	8	.	.	intergenic	-0.164	489485 rs7893	2
AC.GT	14	79840980	79841002	11.5	.	.	intergenic	.	rs791496715	3
AC.GT	14	79915906	79915917	6	.	.	intergenic	-0.157	rs789999213	4

AAAT.ATTT	14	79988084	79988111	7	.	.	intergenic	.	rs791069397	4
AC.GT	14	80073785	80073795	5.5	ENSSSCT00000011248	UNC5B	intron	-0.11	rs692948756	2
AC.GT	14	80707962	80707976	7.5	.	.	intergenic	.	rs790974054	2
AAAC.GTTT	14	80980447	80980474	7	.	.	intergenic	0.092	rs792662472	2
AAT.ATT	14	81297619	81297641	7.66667	ENSSSCT00000011261	MICU1	intron	.	›48308 rs7879	2
AAAAT.ATTTT	14	81327175	81327190	3.2	ENSSSCT00000011261	MICU1	intron	0.169	›01147 rs7889	2
AAAAT.ATTTT	14	81384252	81384270	3.8	ENSSSCT00000011261	MICU1	intron	0.445	rs791916371	2
AC.GT	14	81571230	81571253	12	.	.	intergenic	.	rs793880295	2
AT.AT	14	82266813	82266825	6.5	ENSSSCT00000011271	TTC18	intron	0.321	›56485 rs7914	2
AT.AT	14	82266813	82266825	6.5	ENSSSCT00000022350	TTC18	intron	0.321	›56485 rs7914	2
AAAT.ATTT	14	82382897	82382908	3	ENSSSCT00000036513	PPP3CB	intron	0.744	.	2
AAAT.ATTT	14	82382897	82382908	3	ENSSSCT00000011274	PPP3CB	intron	0.744	.	2
AAAT.ATTT	14	82382897	82382908	3	ENSSSCT00000035011	PPP3CB	intron	0.744	.	2
AAAT.ATTT	14	82382897	82382908	3	ENSSSCT00000035106	PPP3CB	intron	0.744	.	2
AAC.GTT	14	82410716	82410730	5	ENSSSCT00000011274	PPP3CB	intron	0.495	rs709062871	4
AAC.GTT	14	82410716	82410730	5	ENSSSCT00000035011	PPP3CB	intron	0.495	rs709062871	4
AAC.GTT	14	82410716	82410730	5	ENSSSCT00000035106	PPP3CB	intron	0.495	rs709062871	4
AAC.GTT	14	82410716	82410730	5	ENSSSCT00000035374	PPP3CB	intron	0.495	rs709062871	4
AAC.GTT	14	82410716	82410730	5	ENSSSCT00000036513	PPP3CB	intron	0.495	rs709062871	4
AG.CT	14	82436676	82436685	5	ENSSSCT00000011275	USP54	intron	0.199	.	2
AG.CT	14	82772844	82772853	5	.	.	intergenic	.	.	2
AC.GT	14	82867954	82867967	7	ENSSSCT00000011290	VCL	intron	.	.	2
AC.GT	14	82867954	82867967	7	ENSSSCT00000011289	VCL	intron	.	.	2
AAC.GTT	14	83043173	83043188	5.33333	ENSSSCT00000011292	ADK	intron	.	›82633 rs7875	2
AAAAT.ATTTT	14	83119341	83119359	3.8	ENSSSCT00000011292	ADK	intron	.	rs792265150	2
AAAG.CTTT	14	83161330	83161359	7.5	ENSSSCT00000011292	ADK	intron	.	rs789807553	2
AT.AT	14	83462665	83462686	11	.	.	intergenic	.	.	2
AG.CT	14	83580890	83580904	7.5	ENSSSCT00000011293	KAT6B	intron	0.33	rs792704379	4
AG.CT	14	83580890	83580904	7.5	ENSSSCT00000019986	.	promoter	0.33	rs792704379	4
AAT.ATT	14	83954144	83954155	4	.	.	intergenic	.	rs788329588	2
AAAT.ATTT	14	84154335	84154349	3.75	.	.	intergenic	.	rs792859739	2
AAAC.GTTT	14	84181988	84182006	4.75	.	.	intergenic	.	›68371 rs7899	2
AAAT.ATTT	14	84456708	84456725	4.5	.	.	intergenic	0.591	rs793793518	2
AG.CT	14	85430208	85430217	5	.	.	intergenic	.	rs787602118	2
AC.GT	14	85642623	85642635	6.5	.	.	intergenic	-0.234	rs789192341	2
AC.GT	14	85808100	85808115	8	ENSSSCT00000011303	.	intron	.	rs789242773	4
ACCCC.GGGGT	14	85815230	85815244	3	ENSSSCT00000011303	.	intron	-0.066	rs787352680	2
AG.CT	14	85869613	85869622	5	ENSSSCT00000011303	.	intron	0.102	rs789742298	2
AAC.GTT	14	86020799	86020813	5	.	.	intergenic	.	rs793036972	3
AAC.GTT	14	86179042	86179053	4	.	.	intergenic	0.034	rs789643678	2
AT.AT	14	86202923	86202933	5.5	.	.	intergenic	-0.16	rs792994569	2
AAAAC.GTTTT	14	86379296	86379312	3.4	.	.	intergenic	0.097	rs789560223	2
AC.GT	14	86490041	86490053	6.5	.	.	intergenic	-0.077	.	4
AATC.GATT	14	86629343	86629364	5.5	.	.	intergenic	-0.133	rs793681674	2
AG.CT	14	86717625	86717637	6.5	.	.	intergenic	0.366	rs786712708	2
ACAT.ATGT	14	86965671	86965706	9	ENSSSCT00000011305	POLR3A	intron	0.071	›93750 rs7902	3
AATG.CATT	14	87191553	87191594	10.5	.	.	intergenic	-0.142	rs787971159	2
AAAT.ATTT	14	87508699	87508713	3.75	.	.	intergenic	0.709	›93187 rs7917	2
AAAT.ATTT	14	87594018	87594032	3.75	.	.	intergenic	0.123	.	2
AC.GT	14	87806828	87806840	6.5	.	.	intergenic	0.037	rs791710585	2
AAGC.GCTT	14	87927799	87927810	3	.	.	intergenic	-0.267	rs790076155	2
AACC.GGTT	14	87984439	87984450	3	.	.	intergenic	-0.249	.	2
AAAT.ATTT	14	88080627	88080658	8	ENSSSCT00000011307	.	intron	-0.177	rs791491388	2
AT.AT	14	88133434	88133444	5.5	ENSSSCT00000011307	.	intron	-0.182	.	3
AATG.CATT	14	88314988	88315019	8	.	.	intergenic	-0.036	rs788717088	2
AAAC.GTTT	14	88549551	88549565	3.75	ENSSSCT00000028444	TMEM254	intron	0.238	›65662 rs7879	2
AAAC.GTTT	14	88589639	88589655	4.25	.	.	intergenic	.	rs787093511	2
AAAC.GTTT	14	88790306	88790320	3.75	ENSSSCT00000011318	DYDC1	intron	-0.108	›99282 rs7895	2
AAAAT.ATTTT	14	89168391	89168406	3.2	.	.	intergenic	.	rs792119254	2
AAAC.GTTT	14	89182534	89182553	5	.	.	intergenic	.	rs788637539	2
ACT.AGT	14	89649831	89649857	9	.	.	intergenic	.	rs790419937	2
AG.CT	14	89659847	89659859	6.5	.	.	intergenic	0.067	rs789435181	4
AT.AT	14	89889153	89889165	6.5	.	.	intergenic	0.201	rs789609006	2
AAAT.ATTT	14	89936186	89936204	4.75	.	.	intergenic	.	rs791701347	2
AC.GT	14	90150739	90150751	6.5	.	.	intergenic	-0.092	›79665 rs7904	2
AAC.GTT	14	90207129	90207143	5	.	.	intergenic	0.363	rs790494052	3
AC.GT	14	90291486	90291502	8.5	.	.	intergenic	0.1	rs790682706	2
AC.GT	14	90352338	90352349	6	.	.	intergenic	.	›81416 rs7002	4
AT.AT	14	90399958	90399977	10	.	.	intergenic	-0.109	rs787400110	2
AAAAC.GTTTT	14	90410196	90410217	4.4	.	.	intergenic	0.652	.	2
AAGG.CCTT	14	90497879	90497890	3	.	.	intergenic	-0.098	.	2
AC.GT	14	90508999	90509013	7.5	.	.	intergenic	-0.131	rs788573072	2
AAC.GTT	14	90516633	90516651	6.33333	.	.	intergenic	-0.156	rs786842063	2
AC.GT	14	90807495	90807507	6.5	.	.	intergenic	0.013	rs790141700	2
ACAG.CTGT	14	90834316	90834334	4.75	.	.	intergenic	.	rs787856072	2
AC.GT	14	90936658	90936672	7.5	.	.	intergenic	0.016	.	4
AAAC.GTTT	14	90995703	90995721	4.75	.	.	intergenic	0.084	rs790383143	2
AAAC.GTTT	14	91315791	91315804	3.5	.	.	intergenic	0.324	rs790866372	2
AC.GT	14	91354362	91354374	6.5	.	.	intergenic	-0.213	.	4
AAAC.GTTT	14	91462365	91462385	5.25	.	.	intergenic	-0.107	›57598 rs7868	2
AAAT.ATTT	14	92048108	92048124	4.25	.	.	intergenic	.	rs788991539	2
AG.CT	14	92087386	92087401	8	.	.	intergenic	-0.135	rs786212459	2
AC.GT	14	92205970	92205979	5	ENSSSCT00000011325	GHITM	3'utr	0.022	.	2
AAAC.GTTT	14	92396054	92396074	5.25	ENSSSCT00000031701	CCSER2	intron	.	rs786804769	2

AAAC.GTTT	14	92396054	92396074	5.25	ENSSSCT00000011332	CCSER2	intron	.	rs786804769	2
AAAAC.GTTTT	14	92398141	92398165	5	ENSSSCT00000031701	CCSER2	intron	.	rs788469148	2
AAAAC.GTTTT	14	92398141	92398165	5	ENSSSCT00000011332	CCSER2	intron	.	rs788469148	2
AAAAC.GTTTT	14	92485911	92485926	3.2	ENSSSCT00000031701	CCSER2	intron	0.462	rs787290101	2
AAAAC.GTTTT	14	92485911	92485926	3.2	ENSSSCT00000011332	CCSER2	intron	0.462	rs787290101	2
AT.AT	14	92643720	92643729	5	.	.	intergenic	0.083	.	3
AC.GT	14	92719156	92719177	11	.	.	intergenic	-0.16	rs786644501	2
AC.GT	14	92829745	92829755	5.5	.	.	intergenic	0.067	.	6
AT.AT	14	92953164	92953176	6.5	.	.	intergenic	-0.01	rs792209537	2
AT.AT	14	93325443	93325459	8.5	.	.	intergenic	.	458113 rs7921	2
AAAAC.GTTTT	14	93483178	93483202	5	ENSSSCT00000011333	.	intron	-0.057	rs702369505	2
AC.GT	14	93795645	93795674	15	.	.	intergenic	0.049	773179 rs7867	2
AAAC.GTTT	14	93847917	93847931	3.75	.	.	intergenic	0.123	135552 rs7904	2
AAAT.ATTT	14	93988794	93988808	3.75	ENSSSCT00000022896	.	intron	-0.249	rs793086732	2
AT.AT	14	95272150	95272161	6	ENSSSCT00000011342	BMPR1A	intron	-0.146	rs701455741	3
AAAC.GTTT	14	95536449	95536473	6.25	.	.	intergenic	-1.228	rs787861184	2
AC.GT	14	95599054	95599068	7.5	ENSSSCT00000022588	FAM35A	promoter	.	rs791214122	4
AAAG.CTTT	14	95647723	95647748	6.5	.	.	intergenic	.	599004 rs7932	2
ACACAT.ATGTG	14	96308749	96308787	6.5	.	.	intergenic	.	rs791343162	2
AATG.CATT	14	96726165	96726192	7	ENSSSCT00000011362	MAPK8	intron	0.328	181931 rs7919	2
AAAC.GTTT	14	96975533	96975547	3.75	ENSSSCT00000011364	.	intron	-0.166	rs793612040	2
AAG.CTT	14	97199323	97199336	4.66667	ENSSSCT00000026607	WDFY4	intron	-0.032	rs792651729	2
AAG.CTT	14	97199323	97199336	4.66667	ENSSSCT00000011366	WDFY4	intron	-0.032	rs792651729	2
AG.CT	14	97440054	97440065	6	.	.	intergenic	-0.317	164255 rs7888	2
AG.CT	14	97854520	97854531	6	ENSSSCT00000011374	DRGX	intron	.	rs792323172	2
AG.CT	14	98027435	98027445	5.5	ENSSSCT00000011376	CHAT	intron	0.057	rs791707032	2
AAAC.GTTT	14	98044157	98044184	7	ENSSSCT00000011376	CHAT	intron	.	rs786407113	2
AAAC.GTTT	14	98111632	98111652	5.25	.	.	intergenic	0.362	516341 rs7921	2
AC.GT	14	98521325	98521336	6	ENSSSCT00000011380	PARG	intron	-0.191	324687 rs7896	2
AC.GT	14	98521325	98521336	6	ENSSSCT00000023429	.	promoter	-0.191	324687 rs7896	2
AAAAC.GTTTT	14	98592958	98592983	5.2	ENSSSCT00000033578	MSMB	intron	.	384817 rs7885	2
AAT.ATT	14	98633025	98633041	5.66667	ENSSSCT00000011385	.	intron	.	rs793283629	2
AC.GT	14	98730443	98730453	5.5	ENSSSCT00000011386	ZFAND4	intron	0.152	rs793262236	2
AAC.GTT	14	98868066	98868083	6	ENSSSCT00000011387	8-Mar	intron	.	999189 rs6907	2
AAAG.CTTT	14	99670176	99670190	3.75	.	.	intergenic	0.011	rs786338411	2
AAAC.GTTT	14	99857852	99857882	7.75	.	.	intergenic	0.131	rs791447062	2
AT.AT	14	99861498	99861527	15	.	.	intergenic	.	133677 rs7873	3
AT.AT	14	99864545	99864556	6	.	.	intergenic	0.158	rs787349911	2
AT.AT	14	99956006	99956016	5.5	.	.	intergenic	-0.083	084933 rs7879	4
AG.CT	14	100022843	100022869	13.5	.	.	intergenic	.	rs793354657	2
AC.GT	14	100067471	100067485	7.5	.	.	intergenic	-0.477	.	2
AAG.CTT	14	100282168	100282179	4	.	.	intergenic	-0.001	.	2
AG.CT	14	100529761	100529777	8.5	.	.	intergenic	.	rs789087903	2
AAC.GTT	14	100667740	100667753	4.66667	.	.	intergenic	.	300333 rs7919	2
AAAC.GTTT	14	100701532	100701559	7	.	.	intergenic	.	rs789375958	2
AATG.CATT	14	100894636	100894651	4	.	.	intergenic	0.079	rs789490802	2
AC.GT	14	101162196	101162228	16.5	.	.	intergenic	0.017	rs787691380	3
ACC.GGT	14	101496084	101496098	5	.	.	intergenic	.	280305 rs7923	2
AAC.GTT	14	101551959	101551971	4.33333	.	.	intergenic	0.325	rs792074434	3
AAAT.ATTT	14	101581886	101581903	4.5	.	.	intergenic	0.042	rs788126197	2
AAAC.GTTT	14	101664934	101664949	4	.	.	intergenic	.	rs788074911	2
AC.GT	14	101691517	101691534	9	.	.	intergenic	.	rs793882437	2
AT.AT	14	101742021	101742032	6	.	.	intergenic	.	215311 rs7909	2
AAAC.GTTT	14	101792034	101792054	5.25	.	.	intergenic	.	rs788728116	3
AT.AT	14	101895978	101895988	5.5	.	.	intergenic	-0.104	rs697260293	2
AT.AT	14	102542728	102542741	7	.	.	intergenic	0.11	418918 rs7878	2
AAAC.GTTT	14	102551989	102552017	7.25	.	.	intergenic	0.176	.	2
AG.CT	14	102649493	102649516	12	.	.	intergenic	.	rs789833803	2
AGAT.ATCT	14	102733821	102733842	5.5	.	.	intergenic	0.052	rs792076121	2
AT.AT	14	102852359	102852369	5.5	.	.	intergenic	.	rs792431709	2
AAAC.GTTT	14	102982258	102982285	7	.	.	intergenic	.	rs792482719	3
AAAC.GTTT	14	103021225	103021248	6	.	.	intergenic	-0.089	rs789970035	2
AAC.GTT	14	103213483	103213499	5.66667	.	.	intergenic	.	rs698917760	3
AAAT.ATTT	14	103362014	103362035	5.5	.	.	intergenic	.	.	2
AAAC.GTTT	14	103540965	103540986	5.5	.	.	intergenic	.	988414 rs7916	2
AT.AT	14	103677008	103677024	8.5	.	.	intergenic	-0.128	.	2
AG.CT	14	103981747	103981759	6.5	.	.	intergenic	-0.236	.	3
AAG.CTT	14	104036803	104036821	6.33333	.	.	intergenic	-0.068	rs792131669	2
AC.GT	14	104194502	104194513	6	.	.	intergenic	.	rs791946096	2
AAAAAT.ATTTTT	14	104246333	104246353	3.5	.	.	intergenic	.	343644 rs7865	2
AAAAC.GTTTT	14	104295986	104296000	3	.	.	intergenic	.	145957 rs7890	2
AAAT.ATTT	14	104347013	104347031	4.75	.	.	intergenic	.	rs787512565	2
AG.CT	14	104544963	104544972	5	ENSSSCT00000011410	PCDH15	intron	0.035	.	3
AAAAAG.CTTTTT	14	104605831	104605853	3.83333	ENSSSCT00000011410	PCDH15	intron	-0.163	rs790506981	2
AAAC.GTTT	14	104958024	104958038	3.75	.	.	intergenic	0.016	rs789198168	2
AC.GT	14	105019560	105019572	6.5	.	.	intergenic	.	rs788378544	2
AC.GT	14	105037794	105037810	8.5	.	.	intergenic	0.185	.	2
AT.AT	14	105063799	105063819	10.5	.	.	intergenic	0.189	rs791979319	2
AT.AT	14	105069922	105069931	5	.	.	intergenic	0.56	.	2
AAAT.ATTT	14	105145571	105145585	3.75	.	.	intergenic	0.161	rs791063792	2
AC.GT	14	105235331	105235343	6.5	.	.	intergenic	.	rs791178100	3
AAAAT.ATTTT	14	105346500	105346523	4.8	.	.	intergenic	.	rs789046372	2
AT.AT	14	105373119	105373128	5	.	.	intergenic	0.196	.	3
AAAT.ATTT	14	105417987	105418005	4.75	.	.	intergenic	-0.195	.	3

AAAC.GTTT	14	105423019	105423038	5	.	.	intergenic	-0.027	rs789696022	3
ACC.GGT	14	105441814	105441827	4.66667	.	.	intergenic	.	.	2
AC.GT	14	105938690	105938700	5.5	.	.	intergenic	-0.061	l34951 rs7892	3
AAAAT.ATTTT	14	105987158	105987172	3	.	.	intergenic	.	.	2
AG.CT	14	106069457	106069473	8.5	.	.	intergenic	0.043	.	4
AAAT.ATTT	14	106290309	106290336	7	ENSSSCT00000011414	PRKG1	intron	-0.014	?94540 rs7872	2
AATG.CATT	14	106335018	106335045	7	ENSSSCT00000011414	PRKG1	intron	0.03	rs792930340	2
\AAAAC.GTTTT	14	106708929	106708951	3.83333	ENSSSCT00000011414	PRKG1	promoter	-0.05	596042 rs7864	2
AAAC.GTTT	14	106794351	106794378	7	.	.	intergenic	0.179	rs692239853	2
AC.GT	14	106803249	106803259	5.5	.	.	intergenic	-0.153	rs791772307	2
AACC.GGTT	14	106821751	106821773	5.75	.	.	intergenic	0.044	rs793585100	2
AAAAC.GTTTT	14	106865044	106865071	5.6	.	.	intergenic	0.255	rs787784747	2
AAAAC.GTTTT	14	106878055	106878078	4.8	.	.	intergenic	-0.097	.	2
AGC.GCT	14	107457176	107457192	5.66667	.	.	intergenic	0.11	331774 rs7896	2
AAAT.ATTT	14	107491601	107491623	5.75	.	.	intergenic	0.185	rs786423762	2
AT.AT	14	107552754	107552767	7	ENSSSCT00000027836	.	intron	-0.022	rs787378793	3
AC.GT	14	107581331	107581352	11	ENSSSCT00000027836	.	intron	1.253	rs788578249	2
AC.GT	14	107590297	107590308	6	ENSSSCT00000027836	.	intron	.	.	2
AG.CT	14	107593842	107593851	5	ENSSSCT00000027836	.	intron	0.176	'26989 rs7927	2
AAAC.GTTT	14	107608459	107608477	4.75	ENSSSCT00000027836	.	intron	-0.19	rs788935657	2
AATG.CATT	14	107612750	107612772	5.75	ENSSSCT00000027836	.	intron	-0.042	rs786829744	2
AT.AT	14	107650813	107650823	5.5	ENSSSCT00000027836	.	intron	0.123	.	4
AC.GT	14	107728077	107728087	5.5	.	.	intergenic	-0.046	rs789279910	2
AC.GT	14	107770901	107770917	8.5	.	.	intergenic	-0.056	946345 rs7931	2
AC.GT	14	107903001	107903014	7	ENSSSCT00000011417	.	intron	0.437	rs792039466	5
AT.AT	14	108063640	108063649	5	ENSSSCT00000011418	SGMS1	intron	0.021	rs793620830	4
AAAAG.CTTTT	14	108176575	108176596	4.4	.	.	intergenic	-0.095	206631 rs7876	2
AC.GT	14	108308328	108308340	6.5	.	.	intergenic	0.369	rs787840612	2
AAAT.ATTT	14	108323243	108323269	6.75	.	.	intergenic	-0.168	393240 rs7901	2
AC.GT	14	108381254	108381273	10	.	.	intergenic	-0.045	586799 rs7884	2
\AAAAG.CTTTT	14	108408625	108408648	4	.	.	intergenic	0.174	rs792145478	2
AC.GT	14	109378760	109378778	9.5	.	.	intergenic	0.049	.	2
ACAT.ATGT	14	109378828	109378841	3.5	.	.	intergenic	0.032	387274 rs7902	2
AAT.ATT	14	109569967	109569981	5	.	.	intergenic	0.085	rs788381207	2
AT.AT	14	109707091	109707100	5	.	.	intergenic	-0.036	rs787704779	2
\AAAAC.GTTTT	14	109979984	109980006	3.83333	.	.	intergenic	.	.	2
AAC.GTT	14	110000453	110000475	7.66667	.	.	intergenic	0	rs789148338	2
ACC.GGT	14	110024315	110024332	6	.	.	intergenic	-0.114	rs698361527	2
AG.CT	14	110030526	110030538	6.5	.	.	intergenic	-0.031	rs789092955	2
AAAC.GTTT	14	110079098	110079124	6.75	.	.	intergenic	-0.036	)00779 rs7881	2
AC.GT	14	110297591	110297619	14.5	.	.	intergenic	0.308	rs793751433	2
AAAAC.GTTTT	14	110700418	110700441	4.8	.	.	intergenic	0.154	rs700669677	2
AAG.CTT	14	110927387	110927402	5.33333	.	.	intergenic	.	rs706636575	2
AAAC.GTTT	14	111250580	111250596	4.25	.	.	intergenic	.	rs787674820	2
AGAT.ATCT	14	111655911	111655929	4.75	ENSSSCT00000011445	HTR7	intron	.	rs787651446	2
AAAAC.GTTTT	14	111775923	111775941	3.8	.	.	intergenic	.	rs789568607	2
AAAG.CTTT	14	111782852	111782870	4.75	.	.	intergenic	-0.11	rs789250806	3
AT.AT	14	111949889	111949913	12.5	.	.	intergenic	0.098	rs787986359	2
AGG.CCT	14	111987791	111987804	4.66667	.	.	intergenic	-0.009	rs792142342	2
\AAAAC.GTTTT	14	112573831	112573851	3.5	.	.	intergenic	.	rs793203088	2
AAAC.GTTT	14	113079098	113079112	3.75	ENSSSCT00000011455	.	intron	.	rs790417532	2
AAAT.ATTT	14	113095299	113095315	4.25	ENSSSCT00000011455	.	intron	0.266	rs705196049	2
AAGC.GCTT	14	113391327	113391349	5.75	.	.	intergenic	0.057	)40556 rs7908	2
\AAAAC.GTTTT	14	113857294	113857322	4.83333	.	.	intergenic	0.124	l53678 rs7877	2
AAC.GTT	14	113885223	113885235	4.33333	.	.	intergenic	.	rs792646611	3
\AAAAC.GTTTT	14	114093895	114093921	4.5	ENSSSCT00000011461	.	intron	.	'45228 rs7938	2
AT.AT	14	114505493	114505503	5.5	ENSSSCT00000011464	.	intron	1.093	rs791845277	2
AAC.GTT	14	114557261	114557277	5.66667	ENSSSCT00000011464	.	intron	0.241	)41506 rs7884	3
AGG.CCT	14	114574592	114574606	5	ENSSSCT00000011464	.	intron	0.026	rs791316818	2
AAC.GTT	14	114594570	114594583	4.66667	ENSSSCT00000011464	.	intron	0.003	'29927 rs7901	2
AAAC.GTTT	14	114819757	114819774	4.5	ENSSSCT00000011468	PDE6C	intron	.	rs792741937	2
AT.AT	14	114887484	114887502	9.5	ENSSSCT00000031763	FRA10AC1	intron	.	rs793724923	3
AAAC.GTTT	14	114939005	114939019	3.75	.	.	intergenic	0.072	510025 rs7903	2
AT.AT	14	115004717	115004726	5	.	.	intergenic	.	?66687 rs7897	2
AC.GT	14	115491792	115491821	15	ENSSSCT00000011471	PLCE1	intron	0.165	334012 rs7871	4
AC.GT	14	115556913	115556934	11	ENSSSCT00000011471	PLCE1	intron	0.042	rs712757476	4
AT.AT	14	115633931	115633944	7	ENSSSCT00000032028	NOC3L	intron	.	rs790359886	2
AT.AT	14	115633931	115633944	7	ENSSSCT00000011472	NOC3L	intron	.	rs790359886	2
AT.AT	14	115853912	115853925	7	.	.	intergenic	.	rs793798219	2
AC.GT	14	116524317	116524329	6.5	.	.	intergenic	-0.212	308071 rs7938	3
ACC.GGT	14	116687490	116687507	6	.	.	intergenic	0.246	368440 rs7895	2
ACAT.ATGT	14	117030103	117030114	3	.	.	intergenic	0.164	.	2
AT.AT	14	117269925	117269935	5.5	ENSSSCT00000011493	BLNK	intron	.	rs788791413	2
AAAAC.GTTTT	14	117303371	117303397	5.4	ENSSSCT00000011493	BLNK	intron	0.795	l72958 rs7898	2
AAACC.GGTTT	14	117522398	117522416	3.8	.	.	intergenic	1.065	rs793393327	2
ACT.AGT	14	117649306	117649317	4	ENSSSCT00000011498	TM9SF3	intron	0.158	.	2
ACC.GGT	14	118061840	118061855	5.33333	.	.	intergenic	0.495	rs791251930	2
AATG.CATT	14	118260488	118260505	4.5	ENSSSCT00000011504	RRP12	intron	1.016	rs786905432	3
AT.AT	14	118282574	118282587	7	.	.	intergenic	0.358	rs792330472	3
AAAC.GTTT	14	118408972	118408990	4.75	ENSSSCT00000027226	UBTD1	intron	-0.011	rs793471726	2
AAAC.GTTT	14	118408972	118408990	4.75	ENSSSCT00000011511	UBTD1	intron	-0.011	rs793471726	2
AAGG.CCTT	14	118462387	118462404	4.5	ENSSSCT00000011513	HOGA1	promoter	-0.092	rs788623068	2
AAC.GTT	14	118649493	118649508	5.33333	.	.	intergenic	-0.121	.	2
AT.AT	14	118898565	118898575	5.5	ENSSSCT00000011521	R3HCC1L	intron	0.315	rs789009323	2

AC.GT	14	118901816	118901830	7.5	ENSSSCT00000011521	R3HCC1L	intron	0.199	rs789484336	3
AAG.CTT	14	119014723	119014737	5	.	.	intergenic	0.081	342991 rs7866	2
AAAC.GTTT	14	119067026	119067044	4.75	.	.	intergenic	-0.257	)40451 rs7864	2
AAAC.GTTT	14	119239711	119239725	3.75	.	.	intergenic	0.118	rs791835811	2
AAAC.GTTT	14	119322182	119322202	5.25	ENSSSCT00000011525	HPSE2	intron	0.079	)85816 rs7935	3
AG.CT	14	119365646	119365657	6	ENSSSCT00000011525	HPSE2	intron	.	rs790040572	4
AAC.GTT	14	119430253	119430277	8.33333	.	.	intergenic	0.234	rs792849015	2
AT.AT	14	119481458	119481467	5	.	.	intergenic	.	rs788706902	2
AC.GT	14	119624100	119624112	6.5	.	.	intergenic	.	.	2
AG.CT	14	119916725	119916737	6.5	.	.	intergenic	0.513	.	3
AAAT.ATTT	14	120071347	120071366	5	ENSSSCT00000011527	GOT1	intron	0.182	rs792271819	2
AAAC.GTTT	14	120189675	120189695	5.25	.	.	intergenic	0.093	rs791547111	2
AATG.CATT	14	120240636	120240654	4.75	.	.	intergenic	-0.167	rs789116003	2
AAAC.GTTT	14	120367606	120367624	4.75	.	.	intergenic	0.224	252025 rs7862	2
AAAC.GTTT	14	120393238	120393264	6.75	.	.	intergenic	.	rs789117889	2
AATG.CATT	14	120450868	120450888	5.25	ENSSSCT00000011534	ABCC2	intron	.	rs786642206	2
\AAAAC.GTTTTT	14	120466290	120466310	3.5	ENSSSCT00000011534	ABCC2	intron	0.558	I80495 rs7866	2
AAC.GTT	14	120498474	120498492	6.33333	ENSSSCT00000011535	DNMBP	intron	-0.058	rs787518318	2
AG.CT	14	120543982	120543995	7	ENSSSCT00000011535	DNMBP	intron	0.404	rs788707019	2
AG.CT	14	120859741	120859751	5.5	ENSSSCT00000011542	CWF19L1	intron	0.178	.	3
AAT.ATT	14	120891363	120891388	8.66667	ENSSSCT00000011542	CWF19L1	promoter	0.302	rs787918365	2
AAAC.GTTT	14	121302844	121302864	5.25	ENSSSCT00000011547	HIF1AN	intron	.	rs786209295	2
AC.GT	14	121404084	121404103	10	.	.	intergenic	-0.061	rs790191413	2
AAAC.GTTT	14	121445300	121445322	5.75	.	.	intergenic	0.053	rs793370237	2
\AAAAC.GTTTTT	14	121945031	121945059	4.83333	.	.	intergenic	-0.099	rs788392920	2
AAAAC.GTTTT	14	122108588	122108603	3.2	.	.	intergenic	.	.	2
AAAC.GTTT	14	122159299	122159317	4.75	ENSSSCT00000011555	BTRC	intron	0.297	340511 rs7866	2
AT.AT	14	122190322	122190340	9.5	ENSSSCT00000011555	BTRC	intron	0.375	.	2
AGGGG.CCCCT	14	122326979	122326998	4	ENSSSCT00000011555	BTRC	3'utr	1.844	353888 rs7933	2
AAAT.ATTT	14	122405679	122405697	4.75	ENSSSCT00000011558	FBXW4	intron	.	rs793299212	2
AAAAC.GTTTT	14	122406782	122406804	4.6	ENSSSCT00000011558	FBXW4	intron	.	rs786408174	2
AC.GT	14	122714560	122714569	5	ENSSSCT00000011562	KCNIP2	intron	0.18	.	2
AT.AT	14	122833681	122833691	5.5	ENSSSCT00000011564	C10orf76	intron	0.09	.	3
AG.CT	14	122835007	122835019	6.5	ENSSSCT00000011564	C10orf76	intron	0.446	rs791035938	2
AAAAT.ATTTT	14	122906716	122906735	4	ENSSSCT00000011564	C10orf76	intron	.	rs787621523	2
AG.CT	14	123787273	123787282	5	.	.	intergenic	.	.	3
AAC.GTT	14	124554069	124554082	4.66667	.	.	intergenic	-0.064	rs693209044	4
AAAAC.GTTTT	14	124576072	124576094	4.6	.	.	intergenic	.	rs787698717	2
AAAC.GTTT	14	124722826	124722844	4.75	.	.	intergenic	0.063	I56998 rs7915	2
AAACC.GGTTT	14	124802367	124802391	5	.	.	intergenic	0.123	310136 rs7932	2
ATC.GAT	14	124803353	124803370	6	.	.	intergenic	.	581811 rs7924	3
AACAT.ATGTT	14	124834121	124834136	3.2	ENSSSCT00000011602	SLK	intron	0.263	I58110 rs7905	2
AAAG.CTTT	14	124870127	124870149	5.75	ENSSSCT00000011602	SLK	3'utr	0.87	337108 rs7874	2
AAC.GTT	14	124953709	124953727	6.33333	.	.	intergenic	.	rs793202717	2
AAC.GTT	14	125196158	125196177	6.66667	ENSSSCT00000011608	.	intron	.	rs787596396	4
AAAC.GTTT	14	125252589	125252609	5.25	.	.	intergenic	0.379	rs793875581	2
AC.GT	14	125278483	125278494	6	ENSSSCT00000011610	CCDC147	intron	-0.105	rs786474874	2
AG.CT	14	125584945	125584956	6	.	.	intergenic	-0.103	rs792192136	2
AGG.CCT	14	125812228	125812240	4.33333	.	.	intergenic	-0.059	rs787423546	2
AGC.GCT	14	126065383	126065399	5.66667	ENSSSCT00000011613	SORCS3	intron	0.491	.	2
AAAAC.GTTTT	14	126194364	126194378	3	.	.	intergenic	-0.277	rs789628983	2
AAT.ATT	14	126198191	126198206	5.33333	.	.	intergenic	0.019	523765 rs7889	2
AC.GT	14	126285097	126285107	5.5	.	.	intergenic	-0.064	.	2
AAGG.CCTT	14	126290094	126290114	5.25	.	.	intergenic	-0.143	rs791080255	2
AAAC.GTTT	14	126476072	126476094	5.75	.	.	intergenic	-0.035	227797 rs7926	2
AC.GT	14	126480669	126480683	7.5	.	.	intergenic	-0.182	780758 rs7878	4
AT.AT	14	127012927	127012937	5.5	.	.	intergenic	.	rs790267295	3
AAAAC.GTTTT	14	127117986	127118002	3.4	.	.	intergenic	.	rs788813745	2
AAAAC.GTTTT	14	127170584	127170610	5.4	.	.	intergenic	-0.007	718106 rs7871	2
AC.GT	14	127177753	127177768	8	.	.	intergenic	-0.033	rs786395924	2
AT.AT	14	127199743	127199761	9.5	.	.	intergenic	-0.027	rs791260306	3
AC.GT	14	127211656	127211678	11.5	.	.	intergenic	.	rs787195489	2
AC.GT	14	127217902	127217920	9.5	.	.	intergenic	.	.	2
AC.GT	14	127282937	127282955	9.5	.	.	intergenic	-0.091	rs789054436	2
AAC.GTT	14	127283321	127283351	10.3333	.	.	intergenic	-0.33	579303 rs7929	2
AC.GT	14	127370080	127370090	5.5	.	.	intergenic	-0.125	rs701905227	2
AAAG.CTTT	14	127546472	127546486	3.75	.	.	intergenic	.	.	2
AAT.ATT	14	127749028	127749053	8.66667	.	.	intergenic	0.057	596249 rs7934	2
AAAAC.GTTTT	14	127985871	127985889	3.8	ENSSSCT00000033867	SORCS1	intron	-0.079	rs793554040	2
AAAAC.GTTTT	14	127985871	127985889	3.8	ENSSSCT00000034837	SORCS1	intron	-0.079	rs793554040	2
AAAAC.GTTTT	14	127985871	127985889	3.8	ENSSSCT00000034800	SORCS1	intron	-0.079	rs793554040	2
AAAAC.GTTTT	14	127985871	127985889	3.8	ENSSSCT00000032873	SORCS1	intron	-0.079	rs793554040	2
AAAAC.GTTTT	14	127985871	127985889	3.8	ENSSSCT00000026529	SORCS1	intron	-0.079	rs793554040	2
AAAAC.GTTTT	14	127985871	127985889	3.8	ENSSSCT00000034580	SORCS1	intron	-0.079	rs793554040	2
AAAAC.GTTTT	14	127985871	127985889	3.8	ENSSSCT00000035864	SORCS1	intron	-0.079	rs793554040	2
AC.GT	14	128142382	128142403	11	ENSSSCT00000033867	SORCS1	intron	0.758	rs792692119	2
AC.GT	14	128142382	128142403	11	ENSSSCT00000034837	SORCS1	intron	0.758	rs792692119	2
AC.GT	14	128142382	128142403	11	ENSSSCT00000034800	SORCS1	intron	0.758	rs792692119	2
AC.GT	14	128142382	128142403	11	ENSSSCT00000032873	SORCS1	intron	0.758	rs792692119	2
AC.GT	14	128142382	128142403	11	ENSSSCT00000034580	SORCS1	intron	0.758	rs792692119	2
AC.GT	14	128142382	128142403	11	ENSSSCT00000035864	SORCS1	intron	0.758	rs792692119	2
AGG.CCT	14	128173537	128173553	5.66667	ENSSSCT00000033867	SORCS1	intron	-0.11	.	2
AGG.CCT	14	128173537	128173553	5.66667	ENSSSCT00000034837	SORCS1	intron	-0.11	.	2
AGG.CCT	14	128173537	128173553	5.66667	ENSSSCT00000034800	SORCS1	intron	-0.11	.	2

AGG.CCT	14	128173537	128173553	5.66667	ENSSSCT00000032873	SORCS1	intron	-0.11	.	2
AGG.CCT	14	128173537	128173553	5.66667	ENSSSCT00000034580	SORCS1	intron	-0.11	.	2
AGG.CCT	14	128173537	128173553	5.66667	ENSSSCT00000035864	SORCS1	intron	-0.11	.	2
AC.GT	14	128203044	128203058	7.5	ENSSSCT00000033867	SORCS1	intron	-0.32	.	3
AC.GT	14	128203044	128203058	7.5	ENSSSCT00000034837	SORCS1	intron	-0.32	.	3
AC.GT	14	128203044	128203058	7.5	ENSSSCT00000034800	SORCS1	intron	-0.32	.	3
AC.GT	14	128203044	128203058	7.5	ENSSSCT00000032873	SORCS1	intron	-0.32	.	3
AC.GT	14	128203044	128203058	7.5	ENSSSCT00000034580	SORCS1	intron	-0.32	.	3
AC.GT	14	128203044	128203058	7.5	ENSSSCT00000035864	SORCS1	intron	-0.32	.	3
AACC.GGTT	14	128398599	128398618	5	.	.	intergenic	0.359	67089 rs7906	2
AATT.AATT	14	128888242	128888268	6.75	.	.	intergenic	.	rs790250139	2
AG.CT	14	128909642	128909651	5	.	.	intergenic	-0.183	86356 rs7866	3
AC.GT	14	129183649	129183680	16	.	.	intergenic	.	rs694951115	4
AC.GT	14	129427195	129427221	13.5	.	.	intergenic	-0.193	.	4
AC.GT	14	129439839	129439854	8	.	.	intergenic	.	.	2
AG.CT	14	129747941	129747955	7.5	.	.	intergenic	.	rs788911720	2
AAC.GTT	14	129751987	129752011	8.33333	.	.	intergenic	.	02965 rs7890	2
AT.AT	14	129900364	129900374	5.5	.	.	intergenic	0.148	rs694790927	3
AAAC.GTTT	14	129936756	129936771	4	.	.	intergenic	0.223	41259 rs7902	2
AC.GT	14	130089656	130089665	5	.	.	intergenic	.	01527 rs7929	2
AAAT.ATTT	14	130205241	130205273	8.25	.	.	intergenic	.	.	2
AAAC.GTTT	14	130370709	130370727	4.75	.	.	intergenic	.	.	2
AAAC.GTTT	14	130420255	130420269	3.75	.	.	intergenic	.	rs791334539	2
AAAAC.GTTTT	14	130439463	130439480	3.6	.	.	intergenic	.	37042 rs7877	2
AAAAC.GTTTT	14	130513951	130513984	5.66667	.	.	intergenic	0.033	rs792459125	2
AAAC.GTTT	14	130727746	130727758	3.25	.	.	intergenic	-0.134	rs788417306	2
AAAT.ATTT	14	130771078	130771093	4	.	.	intergenic	0.076	rs792037296	2
AAAC.GTTT	14	130978847	130978866	5	.	.	intergenic	0.021	rs790638832	2
AAT.ATT	14	131086391	131086425	11.6667	.	.	intergenic	1.494	rs786944832	2
AC.GT	14	131101101	131101112	6	.	.	intergenic	0.188	.	2
AG.CT	14	131123069	131123078	5	ENSSSCT00000011616	XPNPEP1	intron	-0.176	.	2
AAAC.GTTT	14	131300445	131300457	3.25	ENSSSCT00000011617	ADD3	intron	0.447	rs793717325	2
AC.GT	14	131406600	131406617	9	ENSSSCT00000011617	ADD3	intron	0.147	rs788032262	4
AAC.GTT	14	131437560	131437576	5.66667	.	.	intergenic	0.051	55825 rs7882	4
AC.GT	14	131681037	131681061	12.5	.	.	intergenic	-0.085	rs790244257	2
AAAC.GTTT	14	131731559	131731582	6	.	.	intergenic	-0.234	rs793202512	2
AC.GT	14	131911509	131911521	6.5	.	.	intergenic	0.33	rs792535956	2
AAAT.ATTT	14	131917580	131917601	5.5	.	.	intergenic	.	rs788096666	2
AAAC.GTTT	14	131956028	131956046	4.75	.	.	intergenic	0.007	55875 rs7892	2
AAAAT.ATTTT	14	131994908	131994926	3.8	.	.	intergenic	-0.057	rs787454130	4
ACGC.GCGT	14	132043429	132043446	4.5	.	.	intergenic	0.007	18249 rs7930	2
AGG.CCT	14	132266364	132266379	5.33333	ENSSSCT00000025919	.	intron	0.115	50061 rs7880	2
AGAT.ATCT	14	132509450	132509469	5	.	.	intergenic	0.079	rs789317961	2
AAAC.GTTT	14	132563949	132563963	3.75	.	.	intergenic	-0.334	rs793544187	2
AAAG.CTTT	14	132597529	132597542	3.5	.	.	intergenic	.	rs788770045	2
AAC.GTT	14	132754347	132754360	4.66667	.	.	intergenic	-0.137	rs790406841	2
AC.GT	14	132755146	132755159	7	.	.	intergenic	-0.028	rs787670132	6
AT.AT	14	132834708	132834728	10.5	.	.	intergenic	.	rs792604405	2
AATC.GATT	14	132918427	132918454	7	.	.	intergenic	0.123	91143 rs7916	4
AG.CT	14	133055554	133055564	5.5	.	.	intergenic	-0.117	rs791101010	4
AT.AT	14	133100105	133100114	5	.	.	intergenic	-0.558	rs791229650	2
AAAG.CTTT	14	133119554	133119567	3.5	.	.	intergenic	0.004	.	2
AGGG.CCCT	14	133197342	133197359	4.5	.	.	intergenic	0.093	28305 rs7896	2
AAAAG.CTTTT	14	133262039	133262065	4.5	.	.	intergenic	0.102	19437 rs7039	2
AC.GT	14	133534443	133534453	5.5	.	.	intergenic	.	76447 rs7923	2
AAGAG.CTCTT	14	134243290	134243307	3.6	.	.	intergenic	.	14170 rs7876	2
AC.GT	14	134719860	134719897	19	ENSSSCT00000011635	TCF7L2	intron	0.67	.	3
AT.AT	14	134816149	134816162	7	.	.	intergenic	-0.007	rs789638631	3
AAAC.GTTT	14	134969851	134969865	3.75	.	.	intergenic	-0.143	rs787674942	2
AGC.GCT	14	135058500	135058514	5	.	.	intergenic	0	.	2
AAAG.CTTT	14	135072952	135072977	6.5	.	.	intergenic	0.083	02554 rs7922	2
AAAAC.GTTTT	14	135329922	135329952	6.2	ENSSSCT00000011644	.	intron	.	22100 rs7901	3
ATC.GAT	14	135405515	135405528	4.66667	.	.	intergenic	0.054	rs790925042	2
AG.CT	14	135452710	135452724	7.5	.	.	intergenic	-0.141	.	2
AT.AT	14	135519560	135519581	11	ENSSSCT00000011646	ADRB1	promoter	0.298	21700 rs7878	3
AAC.GTT	14	135558128	135558147	6.66667	.	.	intergenic	0.253	rs792603298	2
AAC.GTT	14	135582756	135582771	5.33333	.	.	intergenic	.	rs792346789	3
AC.GT	14	135640640	135640651	6	ENSSSCT00000011647	C10orf118	intron	0.08	rs787720560	2
AT.AT	14	135984017	135984028	6	ENSSSCT00000011650	ABLIM1	intron	-0.041	rs789983681	2
AT.AT	14	136086450	136086468	9.5	ENSSSCT00000011650	ABLIM1	intron	.	rs788032883	2
AAGG.CCTT	14	136157156	136157189	8.5	.	.	intergenic	0.039	rs788102461	4
AT.AT	14	136585208	136585223	8	ENSSSCT00000032203	ATRNL1	intron	.	79722 rs7917	2
AT.AT	14	136585208	136585223	8	ENSSSCT00000011653	ATRNL1	intron	.	79722 rs7917	2
AAC.GTT	14	136586085	136586101	5.66667	ENSSSCT00000032203	ATRNL1	intron	.	01490 rs7917	2
AAC.GTT	14	136586085	136586101	5.66667	ENSSSCT00000011653	ATRNL1	intron	.	01490 rs7917	2
AAT.ATT	14	136669884	136669895	4	ENSSSCT00000032203	ATRNL1	intron	.	rs793862343	3
AAT.ATT	14	136669884	136669895	4	ENSSSCT00000011653	ATRNL1	intron	.	rs793862343	3
AAAG.CTTT	14	136709472	136709491	5	ENSSSCT00000032203	ATRNL1	intron	0.374	17924 rs7925	2
AAAG.CTTT	14	136709472	136709491	5	ENSSSCT00000011653	ATRNL1	intron	0.374	17924 rs7925	2
AAAAT.ATTTT	14	136846474	136846489	3.2	ENSSSCT00000032203	ATRNL1	intron	.	rs790266147	2
AG.CT	14	136968878	136968890	6.5	.	.	intergenic	.	.	2
AC.GT	14	137019383	137019394	6	.	.	intergenic	-0.048	70599 rs7873	2
AAC.GTT	14	137089312	137089344	11	.	.	intergenic	.	86373 rs7927	2
AT.AT	14	137131150	137131159	5	.	.	intergenic	0.026	rs790812728	2

AAAAC.GTTTT	14	137200199	137200228	6	.	.	intergenic	0.362	rs695589149	2
AC.GT	14	137214977	137214987	5.5	.	.	intergenic	-0.146	rs789674459	2
AC.GT	14	137248721	137248732	6	.	.	intergenic	-0.07	rs702446103	3
AACAC.GTGTT	14	137502657	137502673	3.4	.	.	intergenic	.	458225 rs7883	2
AAT.ATT	14	137547162	137547180	6.33333	ENSSSCT00000011654	.	intron	0.319	rs792827803	2
AAAT.ATTT	14	137585171	137585186	4	ENSSSCT00000011654	.	intron	.	rs793605989	2
AATG.CATT	14	137921886	137921918	8.25	.	.	intergenic	-0.296	.	2
AAC.GTT	14	137945226	137945239	4.66667	.	.	intergenic	0.251	rs791843766	3
AAAT.ATTT	14	137976791	137976804	3.5	.	.	intergenic	0.108	rs790591097	2
AAT.ATT	14	138164546	138164559	4.66667	ENSSSCT00000011663	.	intron	.	rs787547669	2
AAAC.GTTT	14	138412122	138412138	4.25	ENSSSCT00000011666	KIAA1598	intron	-0.125	rs789179311	2
AC.GT	14	138526992	138527009	9	.	.	intergenic	0.645	rs792459179	2
AAAAC.GTTTT	14	138912961	138912979	3.8	.	.	intergenic	0.05	rs789598444	2
AG.CT	14	138921640	138921650	5.5	.	.	intergenic	.	rs789440631	2
AAAT.ATTT	14	138971146	138971162	4.25	.	.	intergenic	0.006	rs791256751	2
AT.AT	14	139085725	139085734	5	.	.	intergenic	0.06	195529 rs7879	3
AAAC.GTTT	14	139095682	139095697	4	.	.	intergenic	-0.176	rs792218735	2
AC.GT	14	139330725	139330739	7.5	.	.	intergenic	0.352	rs787474716	2
AC.GT	14	139590043	139590055	6.5	ENSSSCT00000011673	RAB11FIP2	intron	0.443	703138 rs7877	2
AT.AT	14	139677316	139677332	8.5	.	.	intergenic	0.162	rs793283664	3
AAG.CTT	14	139682159	139682176	6	.	.	intergenic	1.94	rs792531021	2
AT.AT	14	139731415	139731428	7	.	.	intergenic	0.035	.	2
AC.GT	14	139744331	139744342	6	.	.	intergenic	0.11	rs788409927	2
AT.AT	14	139784283	139784298	8	.	.	intergenic	1.294	313304 rs7904	3
ATCC.GGAT	14	139837932	139837949	4.5	.	.	intergenic	0.004	rs790258959	2
ATCC.GGAT	14	139981861	139981880	5	.	.	intergenic	0.139	rs789215315	2
AC.GT	14	140205779	140205803	12.5	ENSSSCT00000023068	.	intron	.	341455 rs7919	2
AG.CT	14	140208359	140208368	5	ENSSSCT00000023068	.	intron	0.053	.	4
AT.AT	14	140217676	140217686	5.5	ENSSSCT00000023068	.	intron	0.103	rs793783198	4
AAGC.GCTT	14	140284624	140284640	4.25	.	.	intergenic	-0.166	585545 rs7924	2
AC.GT	14	140444876	140444885	5	.	.	intergenic	.	rs788377222	2
AAAC.GTTT	14	140670705	140670736	8	ENSSSCT00000011684	GRK5	intron	-0.148	778424 rs7923	2
AAAG.CTTT	14	140701901	140701927	6.75	ENSSSCT00000011684	GRK5	intron	.	594811 rs7924	3
AAAAAC.GTTTTT	14	140703401	140703424	4	ENSSSCT00000011684	GRK5	intron	0.071	234869 rs7902	2
AAAAT.ATTTT	14	140819316	140819341	5.2	ENSSSCT00000011684	GRK5	intron	-0.34	.	2
AAG.CTT	14	140982667	140982681	5	ENSSSCT00000011686	.	intron	0.041	.	3
AAAC.GTTT	14	141055944	141055970	6.75	ENSSSCT00000011687	BAG3	intron	0.154	rs786437605	3
AAAC.GTTT	14	141132515	141132533	4.75	ENSSSCT00000011688	INPP5F	intron	0.305	rs792154506	2
AAAT.ATTT	14	141299420	141299452	8.25	ENSSSCT00000011691	.	intron	.	779960 rs7862	2
AC.GT	14	141535497	141535512	8	.	.	intergenic	.	rs791627779	2
AC.GT	14	141644981	141644997	8.5	.	.	intergenic	-0.165	rs792170167	3
AC.GT	14	141674001	141674013	6.5	.	.	intergenic	-0.003	rs786635185	5
AAAC.GTTT	14	141862139	141862153	3.75	ENSSSCT00000011692	PPAPDC1A	intron	.	136091 rs7923	2
AC.GT	14	142000537	142000549	6.5	.	.	intergenic	-0.024	144030 rs7865	2
AAAC.GTTT	14	142097645	142097656	3	.	.	intergenic	.	rs790643193	2
AGG.CCT	14	142288992	142289007	5.33333	ENSSSCT00000011693	.	intron	-0.217	.	2
AG.CT	14	142376511	142376522	6	.	.	intergenic	0.144	rs787016329	2
AG.CT	14	142424998	142425009	6	.	.	intergenic	-0.198	.	3
AGGG.CCCT	14	142503749	142503762	3.5	ENSSSCT00000036109	FGFR2	intron	-0.077	rs786761694	2
AGGG.CCCT	14	142503749	142503762	3.5	ENSSSCT00000035425	FGFR2	intron	-0.077	rs786761694	2
AGGG.CCCT	14	142503749	142503762	3.5	ENSSSCT00000033576	FGFR2	intron	-0.077	rs786761694	2
AGGG.CCCT	14	142503749	142503762	3.5	ENSSSCT00000033651	FGFR2	intron	-0.077	rs786761694	2
AGGG.CCCT	14	142503749	142503762	3.5	ENSSSCT00000033270	FGFR2	intron	-0.077	rs786761694	2
AGGG.CCCT	14	142503749	142503762	3.5	ENSSSCT00000011702	FGFR2	intron	-0.077	rs786761694	2
AGGG.CCCT	14	142503749	142503762	3.5	ENSSSCT00000032653	FGFR2	intron	-0.077	rs786761694	2
AGGG.CCCT	14	142503749	142503762	3.5	ENSSSCT00000033856	FGFR2	intron	-0.077	rs786761694	2
ATCC.GGAT	14	142535400	142535442	10.75	ENSSSCT00000032653	FGFR2	intron	-0.22	322781 rs7926	2
ATCC.GGAT	14	142535400	142535442	10.75	ENSSSCT00000033856	FGFR2	intron	-0.22	322781 rs7926	2
ATCC.GGAT	14	142535400	142535442	10.75	ENSSSCT00000035980	FGFR2	intron	-0.22	322781 rs7926	2
ATCC.GGAT	14	142535400	142535442	10.75	ENSSSCT00000035159	FGFR2	intron	-0.22	322781 rs7926	2
ATCC.GGAT	14	142535400	142535442	10.75	ENSSSCT00000036109	FGFR2	intron	-0.22	322781 rs7926	2
ATCC.GGAT	14	142535400	142535442	10.75	ENSSSCT00000035425	FGFR2	intron	-0.22	322781 rs7926	2
ATCC.GGAT	14	142535400	142535442	10.75	ENSSSCT00000033270	FGFR2	intron	-0.22	322781 rs7926	2
ATCC.GGAT	14	142535400	142535442	10.75	ENSSSCT00000011702	FGFR2	intron	-0.22	322781 rs7926	2
ATCC.GGAT	14	142535400	142535442	10.75	ENSSSCT00000033576	FGFR2	intron	-0.22	322781 rs7926	2
ATCC.GGAT	14	142535400	142535442	10.75	ENSSSCT00000033651	FGFR2	intron	-0.22	322781 rs7926	2
AAAAC.GTTTT	14	142821353	142821373	4.2	ENSSSCT00000011705	ATE1	intron	0.119	rs786769470	2
AC.GT	14	142835110	142835124	7.5	ENSSSCT00000011705	ATE1	intron	.	570395 rs7899	3
AAAAC.GTTTT	14	142894050	142894073	4.8	.	.	intergenic	.	rs790658812	2
AAAAAC.GTTTTT	14	143519950	143519974	4.16667	ENSSSCT00000011709	HTRA1	intron	-0.402	rs791393909	2
AC.GT	14	143526896	143526909	7	ENSSSCT00000011709	HTRA1	intron	-0.2	rs707819821	2
AAT.ATT	14	144252883	144252898	5.33333	.	.	intergenic	-0.068	rs789390171	2
AAAAGC.GCTTTT	14	144318818	144318837	3.33333	.	.	intergenic	.	rs787779039	3
AT.AT	14	144401311	144401323	6.5	.	.	intergenic	-0.028	.	3
ATCC.GGAT	14	144409808	144409851	11	.	.	intergenic	-0.036	rs791077414	2
AT.AT	14	144702509	144702520	6	.	.	intergenic	.	rs786688565	3
AT.AT	14	144765075	144765085	5.5	.	.	intergenic	.	rs787302541	2
AC.GT	14	144988261	144988283	11.5	ENSSSCT00000011739	CPXM2	intron	0.152	rs793738220	2
AC.GT	14	145784030	145784041	6	.	.	intergenic	0.142	rs791480491	2
AG.CT	14	145789795	145789808	7	.	.	intergenic	-0.09	rs787045842	2
AC.GT	14	145800366	145800388	11.5	.	.	intergenic	-0.032	.	3
AAAT.ATTT	14	146398871	146398886	4	.	.	intergenic	-0.063	.	2
AAC.GTT	14	146492762	146492785	8	.	.	intergenic	0.002	.	4
AC.GT	14	146847678	146847691	7	ENSSSCT00000011757	FANK1	intron	0.159	414173 rs7900	3

AAAAT.ATTTT	14	147098871	147098895	5	.	.	intergenic	-0.041	rs786262518	2
AAAC.GTTT	14	147678990	147679008	4.75	.	.	intergenic	0.517	rs792973326	2
AAC.GTT	14	147796675	147796693	6.33333	.	.	intergenic	0.009	rs787156282	2
AAAC.GTTT	14	147901415	147901429	3.75	.	.	intergenic	0.021	rs791836864	2
AG.CT	14	148006490	148006499	5	.	.	intergenic	-0.166	rs792410233	2
ACTC.GAGT	14	148316495	148316512	4.5	.	.	intergenic	-0.364	rs788276817	2
AAAC.GTTT	14	148360099	148360112	3.5	.	.	intergenic	-0.097	?13644 rs7882	2
AAAC.GTTT	14	148890663	148890681	4.75	.	.	intergenic	-0.085	rs788940214	2
AAAT.ATTT	14	149238217	149238236	5	.	.	intergenic	-0.048	rs791138178	2
AG.CT	14	149570043	149570060	9	.	.	intergenic	0.303	.	4
AAGG.CCTT	14	150784352	150784369	4.5	ENSSSCT00000011768	MGMT	intron	.	329277 rs7925	2
AAC.GTT	14	150836535	150836548	4.66667	ENSSSCT00000011768	MGMT	intron	.	rs789294994	3
AC.GT	14	151168207	151168216	5	.	.	intergenic	-0.302	.	2
AC.GT	14	151409919	151409930	6	.	.	intergenic	-0.086	rs697147015	2
AC.GT	14	151415410	151415420	5.5	.	.	intergenic	.	rs790433291	3
AC.GT	14	151486359	151486372	7	.	.	intergenic	-0.236	rs792011147	3
AG.CT	14	151532287	151532303	8.5	.	.	intergenic	.	I29718 rs7873	3
AAAC.GTTT	14	153137638	153137654	4.25	ENSSSCT00000011784	KNDC1	intron	.	?08650 rs7877	2
AAAC.GTTT	15	161163	161182	5	.	.	intergenic	0.034	rs791935134	2
AAAC.GTTT	15	212488	212502	3.75	.	.	intergenic	.	rs789856867	3
\AAAAC.GTTTT	15	240548	240566	3.16667	.	.	intergenic	-0.015	rs787791742	2
AAAAT.ATTTT	15	790821	790836	3.2	.	.	intergenic	.	rs793766940	2
AT.AT	15	1029658	1029668	5.5	.	.	intergenic	0.478	rs789380752	2
AAAAC.GTTTT	15	1043446	1043470	5	.	.	intergenic	0.039	rs789744071	2
AC.GT	15	1154286	1154301	8	.	.	intergenic	0.297	.	2
AC.GT	15	1365403	1365415	6.5	.	.	intergenic	0.187	rs787129316	4
AACAC.GTGTT	15	1419055	1419070	3.2	.	.	intergenic	-0.048	'81686 rs7869	2
ATC.GAT	15	1807007	1807027	7	.	.	intergenic	0.132	rs793696946	2
AG.CT	15	2429787	2429797	5.5	.	.	intergenic	-0.149	rs793734884	2
AAAT.ATTT	15	2598638	2598655	4.5	.	.	intergenic	0.544	rs793647362	3
AT.AT	15	2622808	2622823	8	.	.	intergenic	0.367	913607 rs7906	2
AG.CT	15	2721470	2721481	6	.	.	intergenic	0.106	.	2
AG.CT	15	2755595	2755606	6	ENSSSCT00000023118	KIF5C	intron	-0.066	319729 rs7911	2
AG.CT	15	2827275	2827285	5.5	ENSSSCT00000023118	KIF5C	intron	1.638	.	3
AC.GT	15	2829785	2829811	13.5	ENSSSCT00000023118	KIF5C	intron	-0.604	rs791744435	2
AT.AT	15	3009046	3009056	5.5	ENSSSCT00000028524	.	intron	0.2	rs707442548	2
AAAT.ATTT	15	3289469	3289484	4	.	.	intergenic	.	rs790750869	2
AAAT.ATTT	15	3331183	3331200	4.5	.	.	intergenic	0.738	rs791678594	2
AAAC.GTTT	15	3558394	3558410	4.25	.	.	intergenic	.	rs791923080	2
AAAT.ATTT	15	3734152	3734167	4	.	.	intergenic	0.249	.	2
AT.AT	15	3744124	3744142	9.5	.	.	intergenic	0.203	rs788458313	4
AT.AT	15	3838006	3838017	6	ENSSSCT00000017067	ACVR2	intron	0.054	rs788413974	2
AC.GT	15	3914338	3914359	11	.	.	intergenic	0.236	rs786494723	2
AT.AT	15	3970620	3970630	5.5	.	.	intergenic	0.458	'54345 rs7886	2
AATG.CATT	15	4436490	4436506	4.25	.	.	intergenic	0.05	rs790933998	3
AAG.CTT	15	4703298	4703309	4	.	.	intergenic	.	rs790644887	2
AC.GT	15	4869811	4869827	8.5	.	.	intergenic	0.06	rs790296310	2
AGAT.ATCT	15	4877175	4877188	3.5	.	.	intergenic	-0.192	rs790258735	2
AAAAC.GTTTT	15	5015885	5015908	4.8	.	.	intergenic	-0.044	rs787723120	2
AAAC.GTTT	15	5402904	5402922	4.75	.	.	intergenic	0.05	?47327 rs7866	3
AAT.ATT	15	5454254	5454279	8.66667	.	.	intergenic	0.014	.	2
AAAC.GTTT	15	5508752	5508766	3.75	.	.	intergenic	0.157	rs788112281	3
AAAT.ATTT	15	5652854	5652872	4.75	.	.	intergenic	-0.041	rs786685259	2
AAAT.ATTT	15	5655216	5655240	6.25	.	.	intergenic	.	316864 rs7876	2
AAAT.ATTT	15	5744516	5744536	5.25	.	.	intergenic	-0.004	rs786431934	2
AAGG.CCTT	15	5854793	5854820	7	.	.	intergenic	-0.244	rs792965161	2
AG.CT	15	5934573	5934582	5	.	.	intergenic	0.054	rs793591991	2
AAC.GTT	15	6140860	6140879	6.66667	.	.	intergenic	-0.129	rs787161988	3
AT.AT	15	6344033	6344043	5.5	.	.	intergenic	0.084	rs792479545	2
AAC.GTT	15	6344195	6344214	6.66667	.	.	intergenic	0.142	rs789287243	2
AG.CT	15	6499505	6499516	6	.	.	intergenic	.	458206 rs7897	3
AT.AT	15	6555027	6555036	5	.	.	intergenic	-0.065	rs786910854	3
AAAC.GTTT	15	6759992	6760007	4	.	.	intergenic	.	593513 rs7910	2
AAAT.ATTT	15	6821870	6821886	4.25	.	.	intergenic	-0.033	580831 rs7925	2
AC.GT	15	6836364	6836382	9.5	.	.	intergenic	0.083	.	2
AC.GT	15	6846291	6846303	6.5	.	.	intergenic	0.075	rs792903518	2
AG.CT	15	6975497	6975507	5.5	.	.	intergenic	0.886	rs787195071	3
AC.GT	15	7121414	7121425	6	.	.	intergenic	-0.021	.	2
\AAAAG.CTTTT	15	7225354	7225374	3.5	.	.	intergenic	0.746	.	3
AT.AT	15	7264731	7264742	6	.	.	intergenic	-0.101	I75406 rs7938	4
AAAC.GTTT	15	7289148	7289169	5.5	.	.	intergenic	0.284	rs787384045	2
AAAAC.GTTTT	15	7322024	7322048	5	.	.	intergenic	0.14	rs787757442	2
AAGG.CCTT	15	7333352	7333367	4	.	.	intergenic	0.64	373375 rs7893	2
AAAAC.GTTTT	15	7335323	7335352	6	.	.	intergenic	.	rs789821232	2
AAC.GTT	15	7485500	7485518	6.33333	.	.	intergenic	0.113	.	4
\AAAAC.GTTTT	15	7527112	7527141	5	.	.	intergenic	0.054	.	2
AG.CT	15	7549362	7549377	8	.	.	intergenic	0.019	.	3
AAAAC.GTTTT	15	7590331	7590359	5.8	.	.	intergenic	0.56	rs791293089	2
AT.AT	15	7664756	7664774	9.5	.	.	intergenic	0.315	rs786723364	2
AAAT.ATTT	15	8563937	8563957	5.25	ENSSSCT00000017069	GTDC1	intron	0.31	rs787514427	2
AT.AT	15	8867917	8867927	5.5	ENSSSCT00000027823	.	intron	1.516	.	3
AG.CT	15	8882348	8882357	5	ENSSSCT00000027823	.	intron	0.73	rs788038404	2
AAAC.GTTT	15	8891178	8891195	4.5	ENSSSCT00000027823	.	intron	1.014	rs786247219	2
AATG.CATT	15	9076648	9076677	7.5	ENSSSCT00000017070	.	intron	-0.043	593333 rs7918	2

AT.AT	15	9096196	9096212	8.5	ENSSSCT00000017070	.	intron	.	.	2
AAAC.GTTT	15	9196573	9196584	3	ENSSSCT00000017070	.	intron	1.308	rs788200412	2
AC.GT	15	9588167	9588190	12	.	.	intergenic	-0.057	.	4
AAAC.GTTT	15	9628528	9628539	3	ENSSSCT00000017072	KYNU	intron	0.069	163914 rs7862	2
AAG.CTT	15	9894703	9894714	4	.	.	intergenic	.	187505 rs7931	2
AT.AT	15	9929113	9929125	6.5	.	.	intergenic	0.019	rs790617741	3
AAAAT.ATTTT	15	10004594	10004608	3	.	.	intergenic	.	rs793127213	2
AC.GT	15	10436658	10436680	11.5	.	.	intergenic	0.092	.	4
AAC.GTT	15	10446699	10446714	5.33333	.	.	intergenic	1.538	486188 rs7928	2
AC.GT	15	10735343	10735365	11.5	.	.	intergenic	.	rs793166489	2
AT.AT	15	10761545	10761564	10	.	.	intergenic	0.122	rs786424310	2
AC.GT	15	11113986	11114002	8.5	.	.	intergenic	-0.125	rs793172882	3
ACAT.ATGT	15	11238617	11238632	4	.	.	intergenic	-0.189	rs793622745	2
AAAC.GTTT	15	11274324	11274363	10	.	.	intergenic	-0.092	rs791507954	2
AC.GT	15	12600292	12600306	7.5	.	.	intergenic	.	408014 rs6992	4
AC.GT	15	12710618	12710627	5	.	.	intergenic	-0.169	.	3
AAAC.GTTT	15	12760774	12760789	4	.	.	intergenic	-0.182	rs793519326	2
AG.CT	15	13002516	13002547	16	ENSSSCT00000017081	.	intron	-0.233	rs792712312	2
AAT.ATT	15	13014687	13014708	7.33333	ENSSSCT00000017081	.	intron	-0.11	rs789218785	2
AT.AT	15	13047546	13047559	7	.	.	intergenic	0.207	rs788810728	4
AAAC.GTTT	15	13064361	13064385	6.25	.	.	intergenic	0.138	rs787913386	2
AG.CT	15	13223439	13223449	5.5	.	.	intergenic	-0.101	rs711487972	3
AC.GT	15	13572389	13572401	6.5	.	.	intergenic	-0.22	rs793418767	2
AT.AT	15	13705298	13705309	6	.	.	intergenic	0.097	rs789386808	3
AAAG.CTTT	15	13753457	13753479	5.75	.	.	intergenic	-0.058	495666 rs7890	2
AAAT.ATTT	15	13817342	13817371	7.5	.	.	intergenic	0.144	rs792267453	2
AC.GT	15	14003157	14003168	6	.	.	intergenic	0.072	rs790352246	2
AG.CT	15	14397991	14398001	5.5	.	.	intergenic	.	rs792386792	2
AC.GT	15	14414818	14414833	8	.	.	intergenic	-0.009	rs788782183	4
AAAAT.ATTTT	15	14451713	14451732	4	.	.	intergenic	0.026	rs787065318	2
AAG.CTT	15	14915434	14915447	4.66667	.	.	intergenic	-0.029	356832 rs7925	2
AC.GT	15	15057337	15057354	9	.	.	intergenic	.	rs791310832	2
AC.GT	15	15124184	15124198	7.5	.	.	intergenic	-0.153	361603 rs7929	2
AT.AT	15	15313580	15313591	6	ENSSSCT00000017083	SPOPL	intron	0.178	rs792737168	2
AAAC.GTTT	15	15364312	15364339	7	.	.	intergenic	0.065	204587 rs7895	2
AAAC.GTTT	15	15913072	15913091	5	.	.	intergenic	0.113	rs787877386	2
AG.CT	15	15924426	15924436	5.5	.	.	intergenic	.	.	2
AAC.GTT	15	16138137	16138157	7	.	.	intergenic	.	rs793742344	2
AC.GT	15	16198155	16198167	6.5	.	.	intergenic	-0.181	.	4
AAAC.GTTT	15	16217770	16217786	4.25	.	.	intergenic	0.138	rs791817720	2
AG.CT	15	16237369	16237386	9	.	.	intergenic	.	957870 rs7922	2
AAC.GTT	15	16450428	16450443	5.33333	.	.	intergenic	0.145	685449 rs7924	2
AAC.GTT	15	16489858	16489871	4.66667	.	.	intergenic	0.459	620542 rs7875	3
AC.GT	15	16507307	16507317	5.5	.	.	intergenic	-0.319	628008 rs7933	2
AG.CT	15	16595245	16595261	8.5	.	.	intergenic	0.077	.	2
AGAT.ATCT	15	16619836	16619863	7	.	.	intergenic	-0.116	056436 rs7918	2
AG.CT	15	16688198	16688207	5	.	.	intergenic	.	.	2
AAAC.GTTT	15	16703889	16703909	5.25	.	.	intergenic	0.108	086328 rs7876	2
AAAC.GTTT	15	16770737	16770759	5.75	.	.	intergenic	0.156	rs793732807	2
AAAC.GTTT	15	16965605	16965624	5	.	.	intergenic	-0.158	307310 rs7869	2
AT.AT	15	17119360	17119370	5.5	.	.	intergenic	.	rs793674413	2
AAT.ATT	15	17161874	17161887	4.66667	.	.	intergenic	0.115	rs788521192	2
AT.AT	15	17395174	17395195	11	.	.	intergenic	0.062	rs791049410	2
AT.AT	15	17520820	17520829	5	.	.	intergenic	-0.004	rs791190860	2
AAAT.ATTT	15	17567963	17567980	4.5	.	.	intergenic	.	rs792425936	2
AAAAC.GTTTT	15	17734467	17734506	8	.	.	intergenic	0.204	679135 rs7896	2
AG.CT	15	18089572	18089581	5	.	.	intergenic	0.077	.	3
AAAAC.GTTTT	15	18206178	18206206	5.8	.	.	intergenic	0.255	rs788316744	2
AT.AT	15	18783143	18783160	9	ENSSSCT00000017092	R3HDM1	intron	0.073	rs788412391	2
AC.GT	15	18956024	18956037	7	.	.	intergenic	0.232	rs789947965	3
AAAAT.ATTTT	15	18969836	18969860	5	.	.	intergenic	0.528	339219 rs7905	2
AAT.ATT	15	18985220	18985231	4	.	.	intergenic	.	rs691986805	3
AAAAC.GTTTT	15	19072232	19072260	5.8	ENSSSCT00000017094	ZRANB3	intron	.	rs793172458	2
AAC.GTT	15	19089584	19089597	4.66667	ENSSSCT00000017094	ZRANB3	intron	0.23	rs787457884	2
AAC.GTT	15	19264470	19264487	6	ENSSSCT00000017095	RAB3GAP1	intron	0.204	rs791037470	2
AAAT.ATTT	15	19359558	19359580	5.75	.	.	intergenic	.	rs790023183	4
AAAC.GTTT	15	19385971	19385995	6.25	ENSSSCT00000017096	MAP3K19	intron	0.494	968349 rs7915	2
AAC.GTT	15	19441378	19441389	4	ENSSSCT00000017097	CCNT2	intron	0.392	rs787591063	2
AAAT.ATTT	15	19466235	19466256	5.5	ENSSSCT00000017097	CCNT2	intron	-0.099	rs793224032	2
AGC.GCT	15	19941803	19941819	5.66667	ENSSSCT00000017100	.	intron	-0.082	rs789684041	2
AGC.GCT	15	19941803	19941819	5.66667	ENSSSCT00000036620	.	intron	-0.082	rs789684041	2
AGC.GCT	15	19941803	19941819	5.66667	ENSSSCT00000034389	.	intron	-0.082	rs789684041	2
AT.AT	15	21315368	21315377	5	.	.	intergenic	-0.021	rs786979248	2
AAAAC.GTTTT	15	21427583	21427608	5.2	ENSSSCT00000031658	.	intron	-0.073	rs793092527	2
AAC.GTT	15	21540971	21540984	4.66667	.	.	intergenic	-0.098	937975 rs7920	2
AAT.ATT	15	21692422	21692450	9.66667	.	.	intergenic	-0.091	.	2
AC.GT	15	21694496	21694507	6	.	.	intergenic	-0.131	rs786492165	2
AC.GT	15	22189143	22189152	5	.	.	intergenic	0.086	.	3
ATC.GAT	15	22344590	22344609	6.66667	.	.	intergenic	-0.031	rs793733994	2
AAAC.GTTT	15	22426468	22426487	5	.	.	intergenic	0.068	rs789863994	2
AAAC.GTTT	15	22649228	22649245	4.5	.	.	intergenic	.	rs792610614	2
AAAG.CTTT	15	22699815	22699832	4.5	.	.	intergenic	0.099	.	2
AAAC.GTTT	15	22815052	22815086	8.75	.	.	intergenic	0.321	rs787300278	3
AAT.ATT	15	22848562	22848575	4.66667	.	.	intergenic	0.071	rs792225614	2

AT.AT	15	22853859	22853868	5	.	.	intergenic	-0.271	.	3
AG.CT	15	22890843	22890856	7	.	.	intergenic	-0.161	rs712694662	3
AC.GT	15	23018730	23018741	6	.	.	intergenic	.	.	2
AAAG.CTTT	15	23141240	23141262	5.75	.	.	intergenic	0.09	rs788325089	2
AATG.CATT	15	23250661	23250676	4	.	.	intergenic	-0.104	rs789892012	2
AAAG.CTTT	15	23271564	23271581	4.5	.	.	intergenic	.	rs786983271	2
AC.GT	15	23460904	23460918	7.5	.	.	intergenic	-0.22	.	3
AC.GT	15	23731154	23731164	5.5	.	.	intergenic	0.277	rs791871003	2
AT.AT	15	24340953	24340962	5	.	.	intergenic	0.066	rs788059585	3
AAAAT.ATTTT	15	24522928	24522944	3.4	.	.	intergenic	0.025	rs787779848	2
AT.AT	15	24540570	24540579	5	.	.	intergenic	-0.066	rs789408810	2
AAAAT.ATTTT	15	24623049	24623063	3	ENSSSCT00000031946	.	intron	0.198	rs789946961	2
AT.AT	15	24647456	24647471	8	ENSSSCT00000031946	.	intron	.	.	2
AAAT.ATTT	15	24647858	24647880	5.75	ENSSSCT00000031946	.	intron	.	rs788046773	2
AC.GT	15	24710157	24710184	14	ENSSSCT00000031946	.	intron	0.069	rs792296320	2
AG.CT	15	24744644	24744657	7	.	.	intergenic	-0.12	rs790991562	2
AC.GT	15	24895452	24895463	6	.	.	intergenic	-0.059	.	3
ACAT.ATGT	15	25095122	25095136	3.75	.	.	intergenic	-0.082	rs789442542	2
AT.AT	15	25181235	25181246	6	.	.	intergenic	.	rs789433787	3
AC.GT	15	25253547	25253558	6	ENSSSCT00000017111	DPP10	intron	-0.088	.	3
CACCC.GGGTG	15	25464762	25464783	3.66667	.	.	intergenic	.	rs789887254	2
AAAC.GTTT	15	25562580	25562593	3.5	.	.	intergenic	.	102900 rs7866	2
AAC.GTT	15	25911971	25911986	5.33333	.	.	intergenic	0.074	rs788380040	2
AC.GT	15	25944999	25945019	10.5	.	.	intergenic	.	.	3
AGAT.ATCT	15	26261522	26261542	5.25	.	.	intergenic	0.296	rs788168321	2
AG.CT	15	26285265	26285278	7	.	.	intergenic	.	.	2
AAATT.AATTT	15	26351946	26351970	5	.	.	intergenic	0.084	rs789425375	2
AAAC.GTTT	15	26365778	26365797	5	.	.	intergenic	-0.063	rs787195877	2
ACAT.ATGT	15	26788373	26788388	4	.	.	intergenic	0.111	89305 rs7913	2
ACAG.CTGT	15	26804286	26804307	5.5	ENSSSCT00000025125	.	promoter	-0.07	62753 rs7875	2
ACAG.CTGT	15	26804286	26804307	5.5	ENSSSCT00000025310	.	promoter	-0.07	62753 rs7875	2
ACAG.CTGT	15	26804286	26804307	5.5	ENSSSCT00000017112	.	promoter	-0.07	62753 rs7875	2
AT.AT	15	26810048	26810065	9	ENSSSCT00000025125	.	intron	-0.039	51234 rs7882	2
AT.AT	15	26810048	26810065	9	ENSSSCT00000025310	.	intron	-0.039	51234 rs7882	2
AT.AT	15	26810048	26810065	9	ENSSSCT00000017112	.	intron	-0.039	51234 rs7882	2
ACC.GGT	15	26936676	26936694	6.33333	ENSSSCT00000017114	CCDC93	intron	0.041	rs789820615	2
AAAG.CTTT	15	27209091	27209105	3.75	.	.	intergenic	0.023	.	2
AT.AT	15	27233535	27233545	5.5	.	.	intergenic	.	rs786638684	2
AAAT.ATTT	15	27686569	27686589	5.25	.	.	intergenic	-0.056	rs786400497	2
AG.CT	15	28145090	28145105	8	ENSSSCT00000034415	MARCO	intron	0.266	rs789921818	2
AG.CT	15	28145090	28145105	8	ENSSSCT00000017116	MARCO	intron	0.266	rs789921818	2
AAAG.CTTT	15	28230882	28230908	6.75	.	.	intergenic	.	rs792062396	2
AGG.CCT	15	28330701	28330714	4.66667	.	.	intergenic	-0.253	rs793740672	2
AAAC.GTTT	15	28530885	28530908	6	ENSSSCT00000017118	C2orf76	intron	0.313	.	2
AAAC.GTTT	15	28539153	28539171	4.75	ENSSSCT00000017118	C2orf76	intron	0.202	rs791125899	2
AAAC.GTTT	15	28597437	28597456	5	.	.	intergenic	-0.207	rs791799615	2
AAT.ATT	15	28715045	28715065	7	.	.	intergenic	0.135	rs787359478	4
AAAAAC.GTTTTT	15	28739359	28739377	3.16667	.	.	intergenic	0.216	rs791502899	2
AAAC.GTTT	15	28797671	28797701	7.75	ENSSSCT00000030086	MAP3K2	intron	.	rs787242923	2
AC.GT	15	28850198	28850208	5.5	ENSSSCT00000022881	MAP3K2	intron	-0.027	.	4
AC.GT	15	28850198	28850208	5.5	ENSSSCT00000030086	MAP3K2	intron	-0.027	.	4
AAAT.ATTT	15	28902746	28902764	4.75	ENSSSCT00000017121	ERCC3	intron	-0.058	34504 rs7927	2
AC.GT	15	28917033	28917053	10.5	.	.	intergenic	.	rs792890847	3
AT.AT	15	28918431	28918443	6.5	.	.	intergenic	-0.072	rs792548042	2
AAT.ATT	15	28980779	28980791	4.33333	.	.	intergenic	-0.188	rs793114068	2
AGC.GCT	15	28983598	28983611	4.66667	.	.	intergenic	-0.038	rs709651006	2
AAC.GTT	15	29233015	29233037	7.66667	.	.	intergenic	.	75472 rs7906	2
AAAT.ATTT	15	29299267	29299286	5	.	.	intergenic	.	94866 rs7877	2
AAAT.ATTT	15	29819983	29820002	5	.	.	intergenic	.	87617 rs7879	3
AC.GT	15	29838158	29838179	11	.	.	intergenic	-0.032	70888 rs7898	2
ATC.GAT	15	29977999	29978017	6.33333	.	.	intergenic	0.016	rs792009258	2
AT.AT	15	29995151	29995162	6	.	.	intergenic	.	rs705197599	2
AC.GT	15	30004710	30004719	5	.	.	intergenic	.	rs789239408	2
AC.GT	15	30202575	30202590	8	.	.	intergenic	-0.257	.	2
AT.AT	15	30244464	30244475	6	.	.	intergenic	.	113731 rs7928	4
AAAC.GTTT	15	30987917	30987933	4.25	.	.	intergenic	-0.285	rs790916410	2
AAAC.GTTT	15	31054854	31054881	7	.	.	intergenic	-0.105	rs793728658	2
AAAAG.CTTTT	15	31060959	31060978	4	.	.	intergenic	-0.219	rs789376407	2
AC.GT	15	31073298	31073310	6.5	.	.	intergenic	.	rs787812618	3
AAAC.GTTT	15	31261978	31261996	4.75	ENSSSCT00000017126	CNTNAP5	intron	0.155	rs786602637	3
AAAAC.GTTTT	15	31692702	31692724	4.6	ENSSSCT00000017126	CNTNAP5	intron	-0.2	rs792579296	2
AAAC.GTTT	15	31778204	31778227	6	ENSSSCT00000017126	CNTNAP5	intron	-0.051	rs787199014	2
AT.AT	15	31789870	31789880	5.5	ENSSSCT00000017126	CNTNAP5	intron	.	rs789752598	2
AAC.GTT	15	31791280	31791297	6	ENSSSCT00000017126	CNTNAP5	intron	.	rs692704124	2
AAC.GTT	15	31799601	31799619	6.33333	ENSSSCT00000017126	CNTNAP5	intron	-0.291	.	2
AG.CT	15	32048796	32048813	9	.	.	intergenic	-0.188	rs790587798	2
AT.AT	15	32246906	32246918	6.5	.	.	intergenic	-0.198	.	4
AAG.CTT	15	32262540	32262562	7.66667	.	.	intergenic	-0.192	rs791287421	2
AG.CT	15	32377213	32377223	5.5	.	.	intergenic	-0.253	rs792528283	2
AAAT.ATTT	15	32474928	32474949	5.5	.	.	intergenic	0.012	79005 rs7884	2
AT.AT	15	32501864	32501875	6	.	.	intergenic	-0.244	81946 rs7889	3
AC.GT	15	32576520	32576530	5.5	.	.	intergenic	0.965	.	3
AC.GT	15	32845492	32845508	8.5	.	.	intergenic	-0.171	19343 rs7873	2
AAAAT.ATTTT	15	32875764	32875782	3.8	.	.	intergenic	.	rs789631890	2

AT.AT	15	32938036	32938054	9.5	.	.	intergenic	.	rs791873119	2
AAAC.GTTT	15	33061663	33061681	4.75	.	.	intergenic	0.33	rs786707879	2
AT.AT	15	33081552	33081564	6.5	.	.	intergenic	.	328966 rs7901	3
AC.GT	15	33196554	33196568	7.5	.	.	intergenic	-0.221	rs710349359	2
AC.GT	15	33398421	33398435	7.5	.	.	intergenic	-0.145	.	2
AAAAT.ATTTT	15	33521992	33522012	4.2	.	.	intergenic	.	rs790147072	2
AT.AT	15	33528714	33528724	5.5	.	.	intergenic	-0.152	147509 rs7938	2
AT.AT	15	33588829	33588838	5	.	.	intergenic	.	353008 rs7892	2
AAG.CTT	15	33594565	33594585	7	.	.	intergenic	-0.013	976656 rs7882	2
AAC.GTT	15	33600967	33600983	5.66667	.	.	intergenic	-0.186	727670 rs7905	2
AC.GT	15	33638937	33638952	8	.	.	intergenic	0.004	rs788696658	2
ACCC.GGGT	15	34265011	34265022	3	.	.	intergenic	-0.151	rs789193614	2
AG.CT	15	34655965	34655974	5	ENSSSCT00000017129	TSN	intron	-0.344	.	2
AG.CT	15	34723875	34723893	9.5	.	.	intergenic	.	.	5
AAAT.ATTT	15	34750350	34750370	5.25	ENSSSCT00000024092	.	intron	.	.	3
AAC.GTT	15	34985711	34985728	6	.	.	intergenic	.	rs789497981	2
AG.CT	15	35040051	35040065	7.5	ENSSSCT00000033491	TFCP2L1	intron	-0.009	310178 rs7937	2
AG.CT	15	35040051	35040065	7.5	ENSSSCT00000017132	TFCP2L1	intron	-0.009	310178 rs7937	2
AG.CT	15	35040051	35040065	7.5	ENSSSCT00000033811	TFCP2L1	intron	-0.009	310178 rs7937	2
AG.CT	15	35040051	35040065	7.5	ENSSSCT00000033192	TFCP2L1	intron	-0.009	310178 rs7937	2
AAAAT.ATTTT	15	35276732	35276758	5.4	ENSSSCT00000017133	GLI2	intron	-0.143	388237 rs7925	2
AAAC.GTTT	15	35434600	35434623	6	ENSSSCT00000023874	.	intron	.	rs786303132	2
AAAAC.GTTTT	15	35459594	35459614	4.2	ENSSSCT00000023874	.	intron	-0.156	248054 rs7889	2
AAC.GTT	15	35534571	35534583	4.33333	.	.	intergenic	-0.276	rs793312464	2
AAG.CTT	15	35541628	35541642	5	.	.	intergenic	-0.124	526130 rs7903	2
AATG.CATT	15	35627405	35627433	7.25	.	.	intergenic	-0.283	rs788004041	2
AG.CT	15	35767373	35767387	7.5	.	.	intergenic	0.197	rs790729071	2
AC.GT	15	35955958	35955970	6.5	.	.	intergenic	0.117	.	2
AAAC.GTTT	15	36104187	36104211	6.25	.	.	intergenic	0.381	rs788678908	2
AAAAT.ATTTT	15	36128622	36128642	4.2	ENSSSCT00000017141	.	intron	-0.283	rs791953879	2
ACC.GGT	15	36186107	36186120	4.66667	.	.	intergenic	-0.054	265959 rs7930	2
AAAC.GTTT	15	36187265	36187283	4.75	.	.	intergenic	0.159	242946 rs7876	2
AT.AT	15	36262363	36262372	5	.	.	intergenic	.	179010 rs7895	2
AAC.GTT	15	36504033	36504046	4.66667	ENSSSCT00000025582	.	intron	0.126	rs791030721	2
AAAC.GTTT	15	36585619	36585652	8.5	ENSSSCT00000027415	.	intron	.	rs698131914	2
AAAAT.ATTTT	15	36595568	36595582	3	ENSSSCT00000027415	.	intron	.	rs788215203	2
AAAC.GTTT	15	36700160	36700185	6.5	.	.	intergenic	.	550042 rs7904	2
AAAG.CTTT	15	36800772	36800785	3.5	.	.	intergenic	.	rs695180008	2
AT.AT	15	36925683	36925697	7.5	.	.	intergenic	0.199	404464 rs7875	3
AC.GT	15	37435235	37435245	5.5	.	.	intergenic	-0.141	.	2
ACAC.GTGT	15	37531302	37531330	7.25	.	.	intergenic	.	rs792825424	3
AC.GT	15	37569972	37569986	7.5	.	.	intergenic	-0.1	583858 rs7913	4
AAAAT.ATTTT	15	37768573	37768595	4.6	ENSSSCT00000027260	CLN8	promoter	.	rs789223789	2
AC.GT	15	38069662	38069674	6.5	ENSSSCT00000017147	.	promoter	-0.085	rs792367347	3
AC.GT	15	38191070	38191117	24	.	.	intergenic	-0.342	271685 rs7927	2
AAG.CTT	15	38683067	38683082	5.33333	.	.	intergenic	.	107144 rs7880	2
AAC.GTT	15	39273023	39273036	4.66667	ENSSSCT00000027445	.	intron	-0.028	rs792333727	2
AT.AT	15	39298552	39298562	5.5	ENSSSCT00000027445	.	intron	-0.193	rs791391460	3
AAAAG.CTTTT	15	39391651	39391675	5	ENSSSCT00000017148	CSMD1	intron	-0.05	.	2
AT.AT	15	39425276	39425289	7	ENSSSCT00000017148	CSMD1	intron	-0.491	446036 rs7871	3
AGAT.ATCT	15	39699295	39699312	4.5	.	.	intergenic	-0.667	081747 rs7895	2
ACAT.ATGT	15	40123336	40123355	5	.	.	intergenic	.	517965 rs7921	2
AC.GT	15	40147194	40147204	5.5	.	.	intergenic	-0.183	rs789835015	3
AG.CT	15	40159987	40160001	7.5	.	.	intergenic	.	245674 rs7932	2
AT.AT	15	40208836	40208851	8	.	.	intergenic	0.798	.	3
AAT.ATT	15	40379790	40379803	4.66667	.	.	intergenic	-0.219	rs793033774	4
AT.AT	15	40415630	40415640	5.5	.	.	intergenic	-0.324	477111 rs7868	3
AC.GT	15	40613189	40613214	13	.	.	intergenic	-0.063	.	2
AC.GT	15	40642590	40642601	6	.	.	intergenic	0.028	rs694550412	4
AT.AT	15	40999136	40999145	5	.	.	intergenic	-0.228	rs793692307	2
ATC.GAT	15	41065086	41065104	6.33333	.	.	intergenic	-0.309	rs788961115	2
AT.AT	15	41069887	41069900	7	.	.	intergenic	-0.664	rs788759025	4
AT.AT	15	41076224	41076240	8.5	.	.	intergenic	-0.174	rs790995808	4
AC.GT	15	41156904	41156923	10	.	.	intergenic	-0.283	rs793518350	2
AC.GT	15	41219322	41219332	5.5	.	.	intergenic	-0.195	.	3
AT.AT	15	41255147	41255160	7	.	.	intergenic	-0.195	rs787125306	4
AT.AT	15	41531256	41531267	6	.	.	intergenic	-0.221	986251 rs7933	2
AGG.CCT	15	41749311	41749324	4.66667	.	.	intergenic	-0.122	906130 rs7876	2
AG.CT	15	41951219	41951231	6.5	.	.	intergenic	-0.915	.	4
AC.GT	15	42137545	42137554	5	.	.	intergenic	.	754307 rs7883	2
AACAC.GTGTT	15	42268096	42268114	3.8	.	.	intergenic	.	rs793555802	2
AAAG.CTTT	15	42689429	42689452	6	ENSSSCT00000017154	ANGPT2	intron	-0.514	rs790353193	2
AAC.GTT	15	42723940	42723951	4	ENSSSCT00000017154	ANGPT2	intron	-0.102	.	4
AAC.GTT	15	42723940	42723951	4	ENSSSCT00000017155	MCPH1	intron	-0.102	.	4
AAC.GTT	15	43316350	43316374	8.33333	.	.	intergenic	.	416613 rs7912	2
AC.GT	15	43324537	43324546	5	.	.	intergenic	0.292	.	2
AAC.GTT	15	43947562	43947575	4.66667	ENSSSCT00000031639	GPM6A	intron	.	.	2
AC.GT	15	43980139	43980148	5	.	.	intergenic	.	.	3
AAAT.ATTT	15	44386769	44386792	6	ENSSSCT00000017167	.	intron	-0.046	.	2
AG.CT	15	44514749	44514764	8	.	.	intergenic	.	.	2
AAC.GTT	15	44534727	44534756	10	.	.	intergenic	0.235	182480 rs7919	4
AC.GT	15	44704322	44704333	6	.	.	intergenic	-0.015	rs788177221	2
AAT.ATT	15	44989275	44989292	6	.	.	intergenic	.	rs791319922	2
AAAT.ATTT	15	44994312	44994324	3.25	.	.	intergenic	.	rs789801838	2

AC.GT	15	46075247	46075262	8	.	.	intergenic	-0.028	209062 rs7909	2
AT.AT	15	46319525	46319536	6	.	.	intergenic	-0.273	397195 rs7873	2
ACAG.CTGT	15	46455628	46455649	5.5	.	.	intergenic	-0.313	rs792690199	2
AAAC.GTTT	15	46506859	46506878	5	.	.	intergenic	.	.	2
AT.AT	15	46513735	46513745	5.5	.	.	intergenic	.	rs787564632	2
AC.GT	15	46540338	46540347	5	.	.	intergenic	-0.022	.	3
AT.AT	15	46621041	46621056	8	.	.	intergenic	0.045	rs788313054	3
AT.AT	15	46646112	46646129	9	.	.	intergenic	-0.002	rs787969229	2
AAC.GTT	15	46905638	46905651	4.66667	.	.	intergenic	0.069	.	2
AC.GT	15	47336824	47336836	6.5	.	.	intergenic	.	.	3
AAC.GTT	15	47453601	47453613	4.33333	.	.	intergenic	.	rs789415994	2
AAAC.GTTT	15	47681517	47681535	4.75	.	.	intergenic	-0.255	rs708008271	2
AG.CT	15	47739075	47739103	14.5	.	.	intergenic	-0.035	759423 rs7879	2
AT.AT	15	47787898	47787917	10	.	.	intergenic	-0.142	.	2
AAAC.GTTT	15	48745815	48745828	3.5	.	.	intergenic	-0.392	rs791023537	2
AC.GT	15	48811828	48811837	5	.	.	intergenic	0.214	rs786722007	4
AAC.GTT	15	48825234	48825247	4.66667	.	.	intergenic	0.088	406781 rs7885	2
AAAAC.GTTTT	15	48844093	48844115	4.6	.	.	intergenic	0.45	rs790715284	2
AT.AT	15	48888342	48888356	7.5	.	.	intergenic	-0.088	.	2
AC.GT	15	49180908	49180917	5	.	.	intergenic	1.381	rs788081611	2
AG.CT	15	49305928	49305950	11.5	.	.	intergenic	0.109	.	2
AAAG.CTTT	15	49342991	49343020	7.5	.	.	intergenic	0.054	028109 rs7870	2
AAG.CTT	15	49680737	49680751	5	.	.	intergenic	.	235071 rs7868	2
AAAC.GTTT	15	49680817	49680839	5.75	.	.	intergenic	.	rs791498794	2
AG.CT	15	49749522	49749538	8.5	.	.	intergenic	-0.124	rs790090455	4
AT.AT	15	50167170	50167182	6.5	.	.	intergenic	-0.171	rs790540384	2
AAT.ATT	15	50254962	50254973	4	.	.	intergenic	-0.041	rs786737288	2
AAAC.GTTT	15	50277880	50277896	4.25	.	.	intergenic	-0.106	531453 rs7903	2
AAAC.GTTT	15	50343903	50343923	5.25	.	.	intergenic	0.295	158651 rs7899	2
AT.AT	15	50513112	50513130	9.5	.	.	intergenic	0.028	347295 rs7916	2
AC.GT	15	50591680	50591705	13	ENSSSCT00000017174	.	intron	0.321	rs786777276	3
AAAT.ATTT	15	50607098	50607118	5.25	.	.	intergenic	0.198	774165 rs7878	2
AC.GT	15	50660812	50660825	7	.	.	intergenic	0.863	rs791672542	2
AAAAT.ATTTT	15	50817775	50817801	5.4	ENSSSCT00000032149	.	intron	-0.004	rs787383502	2
AC.GT	15	50834695	50834705	5.5	.	.	intergenic	0.066	rs790371129	2
ACAT.ATGT	15	50937601	50937619	4.75	ENSSSCT00000022975	TENM3	intron	0.163	119700 rs7918	2
ACAT.ATGT	15	50937601	50937619	4.75	ENSSSCT00000017175	TENM3	intron	0.163	119700 rs7918	2
ACAT.ATGT	15	50937601	50937619	4.75	ENSSSCT00000026310	TENM3	intron	0.163	119700 rs7918	2
AAG.CTT	15	51002259	51002278	6.66667	.	.	intergenic	.	rs790360611	2
AAAT.ATTT	15	51067510	51067528	4.75	ENSSSCT00000017176	DCTD	intron	-0.368	.	2
AG.CT	15	51124577	51124597	10.5	.	.	intergenic	.	rs791287402	3
AAT.ATT	15	51148290	51148312	7.66667	.	.	intergenic	0.41	rs787691304	2
AAG.CTT	15	51247454	51247467	4.66667	ENSSSCT00000028500	WWC2	intron	.	rs788874186	3
AC.GT	15	51348773	51348785	6.5	ENSSSCT00000028500	WWC2	intron	0.12	rs790751410	3
AT.AT	15	51398378	51398391	7	ENSSSCT00000028500	WWC2	intron	0	251409 rs7937	2
AAAC.GTTT	15	51525206	51525226	5.25	.	.	intergenic	0.06	rs787441060	2
AAAC.GTTT	15	51534993	51535013	5.25	ENSSSCT00000025252	ING2	intron	0.291	rs792390087	2
AC.GT	15	51571826	51571845	10	.	.	intergenic	0.139	rs790779597	2
AAAT.ATTT	15	51621066	51621087	5.5	ENSSSCT00000017178	TRAPPC11	intron	0.177	rs792305290	2
AAAAG.CTTTT	15	51646222	51646244	4.6	ENSSSCT00000017178	TRAPPC11	intron	0.021	rs788521723	2
AC.GT	15	51679030	51679042	6.5	.	.	intergenic	-0.093	rs793710507	2
AT.AT	15	51694104	51694113	5	.	.	intergenic	0.309	rs793064055	2
AGC.GCT	15	51698489	51698501	4.33333	.	.	intergenic	-0.128	rs790533610	2
AAAAC.GTTTT	15	51742848	51742871	4.8	.	.	intergenic	0.071	rs787186952	2
AAC.GTT	15	51909005	51909026	7.33333	ENSSSCT00000017181	STOX2	3'utr	3.499	316552 rs7907	2
AAAC.GTTT	15	51955584	51955598	3.75	.	.	intergenic	-0.148	423905 rs7917	2
AC.GT	15	52210847	52210859	6.5	ENSSSCT00000035483	IRF2	intron	-0.184	rs787649232	2
AC.GT	15	52210847	52210859	6.5	ENSSSCT00000032886	IRF2	intron	-0.184	rs787649232	2
AC.GT	15	52210847	52210859	6.5	ENSSSCT00000017183	IRF2	intron	-0.184	rs787649232	2
AC.GT	15	52434242	52434253	6	ENSSSCT00000017188	.	intron	0.204	rs793451454	3
AC.GT	15	52442644	52442654	5.5	ENSSSCT00000017188	.	intron	-0.263	.	4
AGC.GCT	15	52518116	52518131	5.33333	ENSSSCT00000017189	.	intron	-0.053	.	3
AAAAC.GTTTT	15	53067502	53067522	4.2	.	.	intergenic	-0.131	rs789298160	2
AC.GT	15	53677209	53677219	5.5	.	.	intergenic	0.009	rs787757306	2
AAAC.GTTT	15	53700541	53700561	5.25	.	.	intergenic	0.065	rs705411007	2
AAAAC.GTTTT	15	53767361	53767376	3.2	ENSSSCT00000017210	CYP4V2	intron	.	rs787452369	2
AAAT.ATTT	15	54136895	54136915	5.25	.	.	intergenic	.	rs791955098	2
AAAAG.CTTTT	15	54412472	54412500	5.8	ENSSSCT00000025294	.	intron	.	rs705867287	2
AAAAG.CTTTT	15	54412472	54412500	5.8	ENSSSCT00000017214	.	intron	.	rs705867287	2
AAAAG.CTTTT	15	54412472	54412500	5.8	ENSSSCT00000026211	.	intron	.	rs705867287	2
AAAC.GTTT	15	54503105	54503123	4.75	ENSSSCT00000017215	ADAM32	intron	.	rs790344781	2
AAT.ATT	15	54513547	54513563	5.66667	ENSSSCT00000017215	ADAM32	intron	.	.	2
AT.AT	15	54956145	54956156	6	.	.	intergenic	-0.105	rs789183319	4
AAAG.CTTT	15	54992735	54992752	4.5	.	.	intergenic	0.069	rs789076421	2
AAAAC.GTTTT	15	55165684	55165713	5	.	.	intergenic	-0.116	rs793825362	2
AAC.GTT	15	55780792	55780808	5.66667	.	.	intergenic	0.383	.	4
AACAC.GTGTT	15	55833581	55833598	3.6	.	.	intergenic	-0.419	rs790097439	2
AC.GT	15	56162493	56162506	7	.	.	intergenic	1.102	.	4
AAAT.ATTT	15	56349786	56349800	3.75	.	.	intergenic	0.026	264956 rs7872	2
AAAAC.GTTTT	15	56563329	56563353	5	.	.	intergenic	0.158	.	3
AAAC.GTTT	15	56564119	56564133	3.75	.	.	intergenic	0.086	333640 rs7866	2
AG.CT	15	56668824	56668840	8.5	.	.	intergenic	-0.057	rs790479984	3
AG.CT	15	56814293	56814311	9.5	.	.	intergenic	0.1	.	4
AAAT.ATTT	15	56860666	56860689	6	.	.	intergenic	-0.085	.	2

AAC.GTT	15	56916180	56916192	4.33333	.	.	intergenic	-0.015	rs791207099	4
AAC.GTT	15	56998377	56998397	7	.	.	intergenic	-0.006	rs793437287	2
ACAT.ATGT	15	57492002	57492014	3.25	ENSSSCT00000017238	UNC5D	intron	-0.116	.	2
AAAC.GTTT	15	57639011	57639030	5	.	.	intergenic	-0.329	351688 rs7877	2
AAAT.ATTT	15	57711042	57711061	5	.	.	intergenic	0.41	117028 rs7924	2
AGGG.CCCT	15	57926397	57926408	3	.	.	intergenic	-0.151	rs786831642	2
AAT.ATT	15	57977564	57977593	10	.	.	intergenic	0.482	rs788922088	2
AAAC.GTTT	15	58123420	58123443	6	.	.	intergenic	-0.263	rs787668439	2
AAAC.GTTT	15	58186064	58186079	4	.	.	intergenic	-0.113	rs793370232	2
AG.CT	15	58468578	58468592	7.5	.	.	intergenic	-0.247	rs792062571	3
AAAAT.ATTTT	15	58939710	58939732	4.6	.	.	intergenic	0.107	rs792627637	2
AG.CT	15	59242670	59242700	15.5	.	.	intergenic	-0.04	.	2
AAAT.ATTT	15	59458978	59458995	4.5	.	.	intergenic	.	304173 rs7886	2
AAAT.ATTT	15	59473627	59473646	5	.	.	intergenic	0.525	rs791691049	2
AAAAAT.ATTTTT	15	59502295	59502315	3.5	ENSSSCT00000017242	RNF122	promoter	.	rs793886459	2
AAAG.CTTT	15	59821978	59821992	3.75	.	.	intergenic	-0.09	rs705862048	2
AAAAC.GTTTT	15	59877969	59877985	3.4	.	.	intergenic	0.784	901294 rs7882	2
AAAC.GTTT	15	59913688	59913714	6.75	.	.	intergenic	0.003	rs788502728	2
AT.AT	15	60303081	60303090	5	.	.	intergenic	.	rs788891968	2
AAAAC.GTTTT	15	60959984	60960000	3.4	.	.	intergenic	0.132	002462 rs7888	2
AC.GT	15	61308209	61308236	14	.	.	intergenic	0.236	586778 rs7863	2
AG.CT	15	61665171	61665182	6	.	.	intergenic	0.14	rs786963312	2
AC.GT	15	61896076	61896087	6	ENSSSCT00000017253	GTF2E2	intron	0.161	382065 rs7870	4
AAAT.ATTT	15	62188799	62188814	4	.	.	intergenic	-0.295	018884 rs7888	2
AT.AT	15	62255209	62255227	9.5	ENSSSCT00000017255	.	intron	.	rs792580461	2
AT.AT	15	62255209	62255227	9.5	ENSSSCT00000020631	7SK	promoter	.	rs792580461	2
AAAAC.GTTTT	15	62350130	62350155	5.2	.	.	intergenic	.	180875 rs7919	2
AG.CT	15	62443862	62443878	8.5	.	.	intergenic	0.902	.	2
AAAC.GTTT	15	62602793	62602815	5.75	.	.	intergenic	0.362	rs789197251	2
AT.AT	15	62676209	62676222	7	.	.	intergenic	0.027	rs792910245	2
AT.AT	15	62971065	62971078	7	ENSSSCT00000017259	TNKS	intron	0.173	380939 rs7875	3
AC.GT	15	63003143	63003153	5.5	ENSSSCT00000017259	TNKS	intron	0.145	.	2
AAC.GTT	15	63066186	63066200	5	ENSSSCT00000017259	TNKS	intron	0.271	rs787680408	4
AAC.GTT	15	63093482	63093495	4.66667	ENSSSCT00000017259	TNKS	intron	0.148	rs788724942	2
AT.AT	15	63102747	63102761	7.5	.	.	intergenic	0.007	rs788787126	3
AAAC.GTTT	15	63143378	63143410	8.25	.	.	intergenic	.	.	2
AAAT.ATTT	15	63298328	63298348	5.25	.	.	intergenic	-0.331	rs789503551	2
AC.GT	15	63673913	63673922	5	.	.	intergenic	.	rs786292938	2
AAAC.GTTT	15	63703707	63703725	4.75	.	.	intergenic	-0.061	rs791778627	2
AAC.GTT	15	63790396	63790408	4.33333	ENSSSCT00000017261	.	intron	3.05	rs788533780	2
AGGG.CCCT	15	64030345	64030359	3.75	ENSSSCT00000017263	OCA2	intron	0.089	rs786898531	2
AGGC.GCCT	15	64242795	64242809	3.75	.	.	intergenic	.	.	4
AAGT.ACTT	15	64287295	64287311	4.25	.	.	intergenic	.	963964 rs7879	2
AAAAT.ATTTT	15	64340202	64340228	5.4	.	.	intergenic	-0.207	087618 rs7926	2
AC.GT	15	64547828	64547854	13.5	.	.	intergenic	-0.073	rs792864802	2
AAAT.ATTT	15	64716143	64716160	4.5	.	.	intergenic	.	rs790652479	3
AAAC.GTTT	15	64861066	64861092	6.75	.	.	intergenic	0.119	rs789036949	3
AAAAT.ATTTT	15	64884510	64884531	4.4	.	.	intergenic	0.064	rs793802639	2
AAAAG.CTTTT	15	65114028	65114051	4.8	.	.	intergenic	.	rs788037107	2
AAAT.ATTT	15	65313683	65313697	3.75	.	.	intergenic	.	128435 rs7882	2
AC.GT	15	65472057	65472071	7.5	.	.	intergenic	.	306051 rs7920	2
AAG.CTT	15	65700946	65700957	4	.	.	intergenic	.	rs787772256	2
AAGGG.CCCTT	15	65857161	65857184	4	.	.	intergenic	0.04	368449 rs7924	2
AATG.CATT	15	65984801	65984831	7.75	.	.	intergenic	.	rs791615641	2
AAAC.GTTT	15	66418170	66418194	6.25	.	.	intergenic	-0.2	rs792190163	2
AAAC.GTTT	15	66456762	66456778	4.25	.	.	intergenic	-0.401	rs790952919	2
AC.GT	15	66633760	66633783	12	.	.	intergenic	0.393	rs787836048	2
AT.AT	15	66733922	66733936	7.5	ENSSSCT00000017277	FMNL2	intron	0.063	.	2
AT.AT	15	66733922	66733936	7.5	ENSSSCT00000028547	FMNL2	intron	0.063	.	2
AT.AT	15	66733922	66733936	7.5	ENSSSCT00000023011	FMNL2	intron	0.063	.	2
AAAAG.CTTTT	15	66832833	66832865	6.6	ENSSSCT00000017277	FMNL2	intron	-0.336	rs792895920	2
AAAAG.CTTTT	15	66832833	66832865	6.6	ENSSSCT00000028547	FMNL2	intron	-0.336	rs792895920	2
AAAAG.CTTTT	15	66832833	66832865	6.6	ENSSSCT00000023011	FMNL2	intron	-0.336	rs792895920	2
AG.CT	15	66874335	66874347	6.5	ENSSSCT00000017277	FMNL2	intron	0.018	.	3
AG.CT	15	66874335	66874347	6.5	ENSSSCT00000028547	FMNL2	intron	0.018	.	3
AG.CT	15	66874335	66874347	6.5	ENSSSCT00000023011	FMNL2	intron	0.018	.	3
AAAC.GTTT	15	67542820	67542838	4.75	.	.	intergenic	0.164	rs789077666	2
AAAC.GTTT	15	68091045	68091067	5.75	.	.	intergenic	.	rs793405246	2
AC.GT	15	68182658	68182683	13	.	.	intergenic	0.048	365207 rs7873	2
AC.GT	15	68598419	68598454	18	.	.	intergenic	-0.066	rs792974938	3
AAAT.ATTT	15	68881771	68881783	3.25	ENSSSCT00000024665	.	intron	-0.16	792336 rs7878	2
AAAT.ATTT	15	69299908	69299940	8.25	.	.	intergenic	0.165	rs790492690	2
AAAATC.GATTT	15	69425439	69425466	4.66667	ENSSSCT00000017281	KCNJ3	intron	-0.052	rs791624620	2
AT.AT	15	69659850	69659861	6	.	.	intergenic	0.029	rs786500150	2
AT.AT	15	69699838	69699847	5	.	.	intergenic	0.039	rs786429154	2
AT.AT	15	69886069	69886079	5.5	.	.	intergenic	-0.003	rs790211287	4
AT.AT	15	70383655	70383664	5	.	.	intergenic	-0.011	.	4
AAAAG.CTTTT	15	70618514	70618534	4.2	.	.	intergenic	0.426	rs789196978	2
AAAT.ATTT	15	70955501	70955513	3.25	.	.	intergenic	0.165	rs787115166	2
AAT.ATT	15	70993664	70993686	7.66667	.	.	intergenic	0.179	rs792545337	2
AAC.GTT	15	71027604	71027621	6	.	.	intergenic	0.251	107670 rs7901	2
AT.AT	15	71292172	71292181	5	ENSSSCT00000030069	GALNT5	intron	-0.049	rs791284862	4
AC.GT	15	72235206	72235217	6	.	.	intergenic	0.109	rs787975119	2
AG.CT	15	72379599	72379613	7.5	.	.	intergenic	0.296	.	4

AAAC.GTTT	15	72458615	72458634	5	ENSSSCT00000017287	.	intron	0.208	.	3
AAAC.GTTT	15	72603682	72603700	4.75	.	.	intergenic	0.129	354692 rs7925	2
AAAAT.ATTTT	15	72746081	72746105	5	ENSSSCT00000017290	.	intron	.	rs790942855	2
AT.AT	15	72995134	72995149	8	.	.	intergenic	-0.505	.	2
AC.GT	15	73115258	73115285	14	.	.	intergenic	0.062	rs789963009	2
AAAC.GTTT	15	73522615	73522642	7	ENSSSCT00000017294	BAZ2B	intron	0.348	rs792748417	2
AAAC.GTTT	15	73522615	73522642	7	ENSSSCT00000024565	BAZ2B	intron	0.348	rs792748417	2
AT.AT	15	73524535	73524544	5	ENSSSCT00000017294	BAZ2B	intron	0.393	rs788391162	2
AT.AT	15	73524535	73524544	5	ENSSSCT00000024565	BAZ2B	intron	0.393	rs788391162	2
AT.AT	15	73548841	73548853	6.5	ENSSSCT00000017294	BAZ2B	intron	0.154	rs789836453	3
AT.AT	15	73548841	73548853	6.5	ENSSSCT00000024565	BAZ2B	intron	0.154	rs789836453	3
AT.AT	15	73793986	73793998	6.5	.	.	intergenic	.	150977 rs7862	2
AAAC.GTTT	15	73842117	73842145	7.25	.	.	intergenic	.	.	2
AAAC.GTTT	15	73933343	73933364	5.5	ENSSSCT00000031773	7-Mar	intron	1.29	697980 rs7904	2
AAAC.GTTT	15	73933343	73933364	5.5	ENSSSCT00000017295	7-Mar	intron	1.29	697980 rs7904	2
AAAC.GTTT	15	73977874	73977890	4.25	ENSSSCT00000032142	LY75	intron	0.283	rs787108027	2
AAAC.GTTT	15	73977874	73977890	4.25	ENSSSCT00000034209	LY75	intron	0.283	rs787108027	2
AAC.GTT	15	73996718	73996736	6.33333	ENSSSCT00000032142	LY75	intron	0.374	rs789953761	2
AAC.GTT	15	73996718	73996736	6.33333	ENSSSCT00000035518	LY75	intron	0.374	rs789953761	2
AT.AT	15	74187338	74187355	9	ENSSSCT00000017297	PLA2R1	intron	0.202	.	3
AT.AT	15	74187338	74187355	9	ENSSSCT00000017298	.	intron	0.202	.	3
AG.CT	15	74187754	74187770	8.5	ENSSSCT00000017297	PLA2R1	intron	1.185	.	3
AG.CT	15	74187754	74187770	8.5	ENSSSCT00000017298	.	intron	1.185	.	3
AAAAG.CTTTT	15	74225024	74225043	4	ENSSSCT00000017297	PLA2R1	intron	.	rs791069609	2
AAAAG.CTTTT	15	74225024	74225043	4	ENSSSCT00000017298	.	intron	.	rs791069609	2
AAAC.GTTT	15	74362371	74362389	4.75	.	.	intergenic	0.212	rs790596380	2
AC.GT	15	74402823	74402835	6.5	.	.	intergenic	0.232	.	2
ATCC.GGAT	15	74483032	74483059	7	ENSSSCT00000017300	RBMS1	intron	0.186	rs791872527	2
AAC.GTT	15	74601236	74601258	7.66667	.	.	intergenic	0.24	719290 rs7910	2
AC.GT	15	74797072	74797081	5	.	.	intergenic	-0.017	rs790815387	2
AAAC.GTTT	15	75142603	75142634	8	.	.	intergenic	0.056	rs789670136	2
AT.AT	15	75205413	75205425	6.5	.	.	intergenic	0.395	.	2
AC.GT	15	75325781	75325807	13.5	.	.	intergenic	1.392	rs793032950	3
AT.AT	15	75544741	75544756	8	.	.	intergenic	0.292	rs788671663	2
AAC.GTT	15	75632980	75632991	4	.	.	intergenic	0.592	rs788692030	2
AC.GT	15	75879495	75879508	7	.	.	intergenic	0.297	rs789048407	2
AAAG.CTTT	15	76094741	76094778	9.5	ENSSSCT00000027600	SLC4A10	intron	0.196	.	2
AAAG.CTTT	15	76094741	76094778	9.5	ENSSSCT00000023989	SLC4A10	intron	0.196	.	2
AAAG.CTTT	15	76094741	76094778	9.5	ENSSSCT00000017305	SLC4A10	intron	0.196	.	2
AAAC.GTTT	15	76243187	76243209	5.75	.	.	intergenic	0.262	199002 rs7899	2
AAAT.ATTT	15	76370770	76370792	5.75	ENSSSCT00000017308	FAP	intron	1.153	369708 rs7888	2
AAAT.ATTT	15	76370770	76370792	5.75	ENSSSCT00000036390	FAP	intron	1.153	369708 rs7888	2
AT.AT	15	76401215	76401225	5.5	.	.	intergenic	0.122	rs791991103	2
AC.GT	15	76455829	76455845	8.5	ENSSSCT00000017310	IFIH1	intron	1.335	.	2
AAAT.ATTT	15	76906922	76906933	3	.	.	intergenic	0.088	rs787774824	2
AAAT.ATTT	15	76917099	76917112	3.5	.	.	intergenic	0.085	rs788118856	2
AC.GT	15	77035172	77035199	14	.	.	intergenic	-0.075	rs788829849	3
AGAT.ATCT	15	77061375	77061400	6.5	.	.	intergenic	-0.121	480987 rs7883	2
AT.AT	15	77137064	77137077	7	.	.	intergenic	0.078	rs790479520	4
AAAG.CTTT	15	77696008	77696029	5.5	.	.	intergenic	0.123	rs787452153	2
AT.AT	15	77861867	77861877	5.5	.	.	intergenic	0.619	.	4
AC.GT	15	78005735	78005758	12	.	.	intergenic	0.759	.	2
AAAAAC.GTTTTT	15	78245393	78245411	3.16667	.	.	intergenic	3.479	265482 rs7927	2
AC.GT	15	78269224	78269234	5.5	.	.	intergenic	0.127	.	2
AG.CT	15	78288429	78288438	5	.	.	intergenic	.	.	2
AAAT.ATTT	15	78293773	78293798	6.5	.	.	intergenic	0.21	rs789811758	2
AT.AT	15	78488708	78488730	11.5	.	.	intergenic	-0.051	rs786710084	2
AAAC.GTTT	15	79963856	79963892	9.25	ENSSSCT00000029568	SLC38A11	intron	.	132216 rs7872	2
AAAG.CTTT	15	80265602	80265632	7.75	.	.	intergenic	0.006	rs789941774	2
AG.CT	15	80407277	80407286	5	ENSSSCT00000017318	SCN2A	intron	-0.15	rs792919306	2
AAAT.ATTT	15	80519837	80519859	5.75	.	.	intergenic	-0.047	rs789385303	3
AATG.CATT	15	80753242	80753270	7.25	.	.	intergenic	0.09	rs793039656	2
AT.AT	15	80882873	80882883	5.5	.	.	intergenic	1.661	302423 rs7912	2
AC.GT	15	81231015	81231028	7	.	.	intergenic	.	rs788150270	3
AAAT.ATTT	15	82314956	82314975	5	.	.	intergenic	.	rs787912263	2
AC.GT	15	82441259	82441271	6.5	.	.	intergenic	-0.096	rs793867984	2
AAAC.GTTT	15	82470634	82470654	5.25	.	.	intergenic	.	rs786743650	2
AAAT.ATTT	15	82806875	82806892	4.5	.	.	intergenic	0.022	rs787295052	2
ACAT.ATGT	15	82879341	82879378	9.5	.	.	intergenic	0.167	rs786975114	2
AT.AT	15	82934548	82934559	6	.	.	intergenic	0.129	rs791708809	3
AT.AT	15	83044400	83044411	6	.	.	intergenic	.	rs788922979	2
AAAAC.GTTTT	15	83258950	83258972	4.6	.	.	intergenic	0.181	rs786816449	2
AAAC.GTTT	15	83380323	83380336	3.5	.	.	intergenic	.	rs694912653	2
AATC.GATT	15	83431914	83431938	6.25	ENSSSCT00000030087	.	intron	-0.241	596061 rs7902	2
AAAC.GTTT	15	83477699	83477722	6	ENSSSCT00000030087	.	intron	-0.092	356906 rs7883	2
AC.GT	15	83598049	83598059	5.5	.	.	intergenic	-0.107	.	3
ATCC.GGAT	15	84488147	84488170	6	ENSSSCT00000017341	G6PC2	intron	0.139	rs793016709	2
AAAT.ATTT	15	84551181	84551205	6.25	ENSSSCT00000017343	ABCB11	intron	0.232	rs789979812	2
AAAT.ATTT	15	84551181	84551205	6.25	ENSSSCT00000017342	ABCB11	intron	0.232	rs789979812	2
AC.GT	15	84900144	84900167	12	.	.	intergenic	.	.	2
AC.GT	15	85017895	85017914	10	ENSSSCT00000025574	FASTKD1	intron	0.936	.	2
AC.GT	15	85017895	85017914	10	ENSSSCT00000017349	FASTKD1	intron	0.936	.	2
AAAC.GTTT	15	85076661	85076683	5.75	ENSSSCT00000017350	PPIG	intron	0.102	112844 rs7873	2
AAAAAC.GTTTTT	15	85178218	85178245	4.66667	.	.	intergenic	.	rs791770524	2

AT.AT	15	85479990	85479999	5	ENSSSCT00000017356	.	intron	0.187	254225 rs7910	2
AAAC.GTTT	15	85498638	85498656	4.75	ENSSSCT00000017356	.	intron	0.32	rs786362079	2
AAC.GTT	15	85542144	85542155	4	.	.	intergenic	0.271	rs788611896	2
AC.GT	15	85639400	85639425	13	.	.	intergenic	0.206	140915 rs7930	4
AAAAC.GTTTT	15	85793873	85793900	4.66667	.	.	intergenic	.	rs790913809	2
AATAT.ATATT	15	86281241	86281255	3	.	.	intergenic	0.198	rs792401315	2
AT.AT	15	86429901	86429912	6	.	.	intergenic	.	rs788273603	2
AAAAG.CTTTT	15	86460496	86460517	4.4	.	.	intergenic	0.225	128503 rs7874	2
AAG.CTT	15	86509591	86509604	4.66667	ENSSSCT00000017360	GORASP2	intron	-0.17	rs789299780	2
AAC.GTT	15	86525961	86525974	4.66667	.	.	intergenic	.	rs788656375	2
AAACC.GGTTT	15	86574335	86574360	4.33333	ENSSSCT00000017361	TLK1	intron	0.263	rs788828621	2
AG.CT	15	86886498	86886508	5.5	ENSSSCT00000017362	METTL8	intron	0.308	.	4
AAAC.GTTT	15	86952512	86952530	4.75	ENSSSCT00000023821	DCAF17	intron	.	rs792236291	2
AAAC.GTTT	15	86952512	86952530	4.75	ENSSSCT00000017364	DCAF17	intron	.	rs792236291	2
AAC.GTT	15	87146459	87146475	5.66667	.	.	intergenic	0.111	rs789802622	2
AAAAC.GTTTT	15	87164452	87164473	3.66667	.	.	intergenic	0.194	rs786450414	2
AT.AT	15	87368958	87368971	7	ENSSSCT00000017366	SLC25A12	intron	0.315	rs790816980	2
AT.AT	15	87368958	87368971	7	ENSSSCT00000022813	SLC25A12	intron	0.315	rs790816980	2
AG.CT	15	87649329	87649340	6	ENSSSCT00000017371	DLX2	promoter	0.172	rs793282223	2
AC.GT	15	87757170	87757181	6	.	.	intergenic	.	.	4
AAAAG.CTTTT	15	87966213	87966239	5.4	.	.	intergenic	.	rs787853369	2
AC.GT	15	88130192	88130208	8.5	ENSSSCT00000031710	.	intron	-0.125	463962 rs7863	2
AT.AT	15	88132156	88132166	5.5	ENSSSCT00000031710	.	intron	0.048	rs787175452	2
AAAC.GTTT	15	88169846	88169865	5	ENSSSCT00000031710	.	intron	0.746	797653 rs7872	2
AC.GT	15	88250310	88250342	16.5	ENSSSCT00000031710	.	intron	0.186	409157 rs7932	2
AAAAC.GTTTT	15	88287592	88287605	2.8	.	.	intergenic	0.048	rs788787474	2
AT.AT	15	88307752	88307762	5.5	.	.	intergenic	.	310310 rs7921	2
AAAC.GTTT	15	88472783	88472812	7.5	.	.	intergenic	.	472374 rs7905	3
AAAAC.GTTTT	15	88518928	88518963	7.2	ENSSSCT00000017378	.	intron	0.437	098064 rs7926	2
AAAT.ATTT	15	88537271	88537289	4.75	ENSSSCT00000017378	.	intron	.	rs787668762	2
AAAT.ATTT	15	88554502	88554515	3.5	ENSSSCT00000017378	.	intron	.	rs792615612	2
AAAC.GTTT	15	88573908	88573929	5.5	ENSSSCT00000017378	.	intron	0.158	rs788506805	2
AAG.CTT	15	88602245	88602269	8.33333	ENSSSCT00000017378	.	intron	0.483	.	2
AAAT.ATTT	15	88620245	88620261	4.25	ENSSSCT00000017378	.	intron	0.269	rs788964221	2
AT.AT	15	88786357	88786367	5.5	.	.	intergenic	0.175	rs792295205	2
AC.GT	15	89301581	89301593	6.5	.	.	intergenic	0.273	.	4
AAAC.GTTT	15	89415419	89415455	9.25	ENSSSCT00000017382	OLA1	intron	0.25	rs790473872	2
AT.AT	15	89739679	89739689	5.5	.	.	intergenic	0.568	rs790630708	2
AAACC.GGTTT	15	89826586	89826611	5.2	ENSSSCT00000028507	WIPF1	intron	.	rs789351396	2
AC.GT	15	89887324	89887338	7.5	.	.	intergenic	.	rs792710524	2
AAAC.GTTT	15	89909911	89909931	5.25	.	.	intergenic	0.278	rs791898113	2
ATC.GAT	15	89983432	89983451	6.66667	.	.	intergenic	0.182	.	2
AT.AT	15	90130124	90130134	5.5	.	.	intergenic	.	436367 rs7935	2
AC.GT	15	90133087	90133099	6.5	.	.	intergenic	-0.01	001178 rs7893	3
AAAT.ATTT	15	90289749	90289767	4.75	ENSSSCT00000017391	ATF2	intron	0.251	rs788298288	2
AC.GT	15	90323927	90323941	7.5	.	.	intergenic	0.132	405688 rs7919	3
AT.AT	15	90354287	90354299	6.5	.	.	intergenic	.	080305 rs7870	2
AT.AT	15	90457134	90457145	6	.	.	intergenic	0.061	rs699786243	3
AAAAC.GTTTT	15	90745129	90745147	3.8	.	.	intergenic	-0.089	342070 rs7931	2
AGC.GCT	15	90807655	90807674	6.66667	.	.	intergenic	0.024	rs788109430	2
AGG.CCT	15	91471810	91471829	6.66667	.	.	intergenic	.	391260 rs7878	2
AAAAC.GTTTT	15	91483636	91483662	5.4	.	.	intergenic	0.742	186290 rs7929	2
AAAC.GTTT	15	91520321	91520337	4.25	.	.	intergenic	-0.032	rs789840246	2
ACAG.CTGT	15	91639167	91639189	5.75	.	.	intergenic	0.262	900036 rs7024	4
AAAC.GTTT	15	91750972	91750990	4.75	.	.	intergenic	-0.041	546716 rs7864	2
AC.GT	15	91963515	91963525	5.5	.	.	intergenic	0.025	.	3
AG.CT	15	92019559	92019569	5.5	.	.	intergenic	0.229	.	2
AATC.GATT	15	92072097	92072110	3.5	.	.	intergenic	0.271	rs792746758	2
AAC.GTT	15	92190366	92190384	6.33333	.	.	intergenic	0.284	rs789882563	2
ATC.GAT	15	92225740	92225753	4.66667	.	.	intergenic	0.239	rs789738242	2
AC.GT	15	92274846	92274860	7.5	.	.	intergenic	0.204	rs791845995	3
AAAG.CTTT	15	92344650	92344668	4.75	.	.	intergenic	.	rs786562538	2
AG.CT	15	92901608	92901623	8	ENSSSCT00000017415	PDE11A	intron	0.236	rs788903860	2
AAC.GTT	15	93329415	93329434	6.66667	ENSSSCT00000017417	RBM45	promoter	0.079	117624 rs6904	2
AAAC.GTTT	15	93498834	93498863	7.5	.	.	intergenic	0.202	rs787774825	2
AAAT.ATTT	15	93571469	93571485	4.25	ENSSSCT00000027373	OSBPL6	intron	0.167	397760 rs7892	2
AT.AT	15	93592633	93592656	12	ENSSSCT00000027373	OSBPL6	intron	0.237	358505 rs7886	4
AC.GT	15	93649081	93649092	6	.	.	intergenic	.	rs786400176	2
AATG.CATT	15	93678184	93678210	6.75	ENSSSCT00000017418	PRKRA	intron	0.011	547120 rs7919	2
AC.GT	15	93747239	93747254	8	.	.	intergenic	0.202	.	2
AAAC.GTTT	15	94160431	94160445	3.75	ENSSSCT00000017424	CCDC141	intron	0.547	rs790311326	2
AT.AT	15	94240907	94240917	5.5	.	.	intergenic	.	.	3
AGC.GCT	15	94718562	94718579	6	.	.	intergenic	0	044752 rs7873	2
ACCT.AGGT	15	95047512	95047540	7.25	.	.	intergenic	0.059	.	2
AG.CT	15	95246966	95246989	12	.	.	intergenic	0.103	rs791689812	2
AC.GT	15	96072767	96072785	9.5	.	.	intergenic	-0.014	rs787447913	4
AT.AT	15	96479947	96479959	6.5	.	.	intergenic	-0.124	rs786700754	2
AAAT.ATTT	15	96505476	96505494	4.75	.	.	intergenic	0.056	531364 rs7925	2
ACCC.GGGT	15	96818994	96819008	3.75	.	.	intergenic	-0.049	.	2
AAAAG.CTTTT	15	96996469	96996488	3.33333	.	.	intergenic	-0.05	344344 rs7870	2
AC.GT	15	97018570	97018595	13	ENSSSCT00000024180	PDE1A	intron	0.087	.	4
AAAT.ATTT	15	97021034	97021056	5.75	ENSSSCT00000024180	PDE1A	intron	3.665	rs786510926	2
AAAC.GTTT	15	97227849	97227865	4.25	ENSSSCT00000027867	.	intron	0.085	rs792953840	2
AATT.AATT	15	97392411	97392428	4.5	ENSSSCT00000027867	.	intron	0.085	rs792139599	2

AAATT.AATTT	15	97625750	97625789	8	.	.	intergenic	0.211	438616 rs7933	2
AC.GT	15	97644691	97644702	6.25	.	.	intergenic	0.164	790048 rs7890	2
AAAC.GTTT	15	97752884	97752908	6.25	ENSSSCT00000017440	DNAJC10	intron	.	354464 rs7889	2
AAATT.AATTT	15	98100666	98100690	5	.	.	intergenic	0.166	rs790152023	2
AG.CT	15	98317207	98317217	5.5	.	.	intergenic	0.027	.	2
AAAC.GTTT	15	98380363	98380375	3.25	.	.	intergenic	0.123	rs792282585	2
AAC.GTT	15	98398388	98398424	12.3333	.	.	intergenic	.	rs788991667	2
AAAC.GTTT	15	98844025	98844048	6	.	.	intergenic	-0.276	363203 rs7910	2
AATT.AATT	15	98975325	98975338	3.5	.	.	intergenic	0.256	rs788493740	2
AC.GT	15	99097914	99097928	7.5	.	.	intergenic	.	.	2
AC.GT	15	99298976	99298986	5.5	.	.	intergenic	0.043	rs791669658	2
AC.GT	15	99318946	99318957	6	.	.	intergenic	0.305	.	3
AAAAC.GTTTT	15	99389339	99389366	5.6	.	.	intergenic	.	739777 rs7933	2
AAAAT.ATTTT	15	99457605	99457626	4.4	.	.	intergenic	-0.027	rs789217690	2
AAAC.GTTT	15	99536491	99536517	6.75	.	.	intergenic	0.049	789629 rs7921	2
AC.GT	15	99571919	99571929	5.5	.	.	intergenic	.	.	2
AATT.AATT	15	99651675	99651688	3.5	.	.	intergenic	.	rs792122114	2
AC.GT	15	99657956	99657965	5	.	.	intergenic	.	.	2
AAAG.CTTT	15	99723069	99723100	8	.	.	intergenic	.	rs787607789	2
AAAC.GTTT	15	99731525	99731547	5.75	.	.	intergenic	-0.177	939214 rs7908	2
AT.AT	15	99954837	99954846	5	.	.	intergenic	.	rs787718106	2
AC.GT	15	100369487	100369505	9.5	.	.	intergenic	-0.011	334073 rs7912	2
AAT.ATT	15	100570626	100570640	5	.	.	intergenic	0.083	.	4
AT.AT	15	100574895	100574927	16.5	.	.	intergenic	-0.018	.	2
AC.GT	15	100596749	100596758	5	.	.	intergenic	0.046	rs792597673	2
AC.GT	15	100610758	100610768	5.5	.	.	intergenic	.	.	3
AC.GT	15	101143298	101143318	10.5	.	.	intergenic	0.366	938979 rs7895	2
AAAAT.ATTTT	15	101151340	101151362	4.6	.	.	intergenic	.	514443 rs7938	2
AAAC.GTTT	15	101276771	101276785	3.75	.	.	intergenic	0.144	116265 rs7923	2
AAC.GTT	15	101325135	101325152	6	.	.	intergenic	.	rs787015818	3
AG.CT	15	101494381	101494392	6	.	.	intergenic	0.168	rs789006702	3
AG.CT	15	101538766	101538776	5.5	.	.	intergenic	0.282	.	3
AAAT.ATTT	15	101622450	101622479	7.5	ENSSSCT00000017452	ITGAV	intron	0.426	rs786596228	2
AAAG.CTTT	15	101684900	101684916	4.25	ENSSSCT00000017452	ITGAV	intron	1.055	rs788391626	2
AAAG.CTTT	15	101684900	101684916	4.25	ENSSSCT00000035294	ITGAV	intron	1.055	rs788391626	2
AAAG.CTTT	15	101684900	101684916	4.25	ENSSSCT00000035682	ITGAV	intron	1.055	rs788391626	2
AAAG.CTTT	15	101684900	101684916	4.25	ENSSSCT00000034634	ITGAV	intron	1.055	rs788391626	2
AAC.GTT	15	101712167	101712186	6.66667	ENSSSCT00000017452	ITGAV	intron	0.018	517364 rs7890	2
AAC.GTT	15	101712167	101712186	6.66667	ENSSSCT00000035294	ITGAV	intron	0.018	517364 rs7890	2
AAC.GTT	15	101712167	101712186	6.66667	ENSSSCT00000035682	ITGAV	intron	0.018	517364 rs7890	2
AAC.GTT	15	101712167	101712186	6.66667	ENSSSCT00000034634	ITGAV	intron	0.018	517364 rs7890	2
AC.GT	15	101721653	101721666	7	.	.	intergenic	0.062	rs792945825	4
AC.GT	15	101731230	101731242	6.5	ENSSSCT00000031268	FAM171B	promoter	-0.209	.	2
AAAAC.GTTTT	15	101731688	101731712	4.16667	ENSSSCT00000031268	FAM171B	promoter	0.028	rs788995502	2
AAAAT.ATTTT	15	101798962	101798979	3.6	ENSSSCT00000031268	FAM171B	intron	0.789	rs793368581	2
AAAG.CTTT	15	102048612	102048626	3.75	.	.	intergenic	.	rs789589410	2
AG.CT	15	102219651	102219664	7	.	.	intergenic	0.004	rs791899848	4
AT.AT	15	102232735	102232746	6	.	.	intergenic	.	rs791747265	2
AT.AT	15	102247562	102247574	6.5	.	.	intergenic	.	rs788339154	3
AC.GT	15	102253566	102253576	5.5	.	.	intergenic	0.143	rs790718071	2
AAAAT.ATTTT	15	102263170	102263187	3.6	.	.	intergenic	0.098	.	2
AT.AT	15	102350449	102350463	7.5	.	.	intergenic	.	rs791204366	2
AAAC.GTTT	15	102554773	102554787	3.75	ENSSSCT00000017457	TFPI	intron	0.206	rs786564939	2
ATC.GAT	15	102574553	102574571	6.33333	ENSSSCT00000017457	TFPI	intron	.	rs786917696	2
AAAAG.CTTTT	15	102603782	102603799	3.6	.	.	intergenic	-0.213	rs792673134	2
AAAC.GTTT	15	103075494	103075512	4.75	.	.	intergenic	-0.06	355684 rs7863	2
AAAAAG.CTTTTT	15	103471253	103471272	3.33333	.	.	intergenic	.	.	2
AAAC.GTTT	15	103575418	103575444	6.75	.	.	intergenic	0.117	rs789829095	2
AAAAT.ATTTT	15	103645276	103645298	4.6	ENSSSCT00000017458	.	intron	0.088	rs791146168	2
AAG.CTT	15	104526071	104526086	5.33333	ENSSSCT00000017461	WDR75	intron	.	rs789575893	2
AAAC.GTTT	15	104529407	104529421	3.75	ENSSSCT00000017461	WDR75	intron	.	.	2
AC.GT	15	104683121	104683132	6	.	.	intergenic	.	rs792737346	4
AAC.GTT	15	105463718	105463738	7	ENSSSCT00000017470	PMS1	intron	.	rs789011680	2
AG.CT	15	105474148	105474163	8	ENSSSCT00000017470	PMS1	intron	0.253	rs788857317	4
AAAC.GTTT	15	105721186	105721214	7.25	.	.	intergenic	.	rs793235086	2
AC.GT	15	106372335	106372349	7.5	.	.	intergenic	0.138	.	2
AAAT.ATTT	15	106380143	106380160	4.5	.	.	intergenic	0.447	451923 rs7936	2
AG.CT	15	106799366	106799382	8.5	.	.	intergenic	0.082	rs792941401	4
AGGG.CCCT	15	106885200	106885218	4.75	.	.	intergenic	0.139	rs789960200	2
AC.GT	15	107100988	107101010	11.5	.	.	intergenic	0.033	rs788611174	2
AAAC.GTTT	15	107270705	107270722	4.5	ENSSSCT00000029791	.	intron	.	rs786849617	2
AAAAC.GTTTT	15	107292583	107292607	5	.	.	intergenic	-0.061	286016 rs7911	2
AAAT.ATTT	15	107333159	107333173	3.75	.	.	intergenic	.	968439 rs7903	2
AAG.CTT	15	107463767	107463780	4.66667	.	.	intergenic	-0.092	rs788759473	2
AT.AT	15	107627556	107627591	18	.	.	intergenic	.	rs790116164	2
AC.GT	15	108241436	108241476	20.5	ENSSSCT00000017490	TMEFF2	intron	0.029	rs792369645	4
AT.AT	15	108359000	108359009	5	ENSSSCT00000017490	TMEFF2	intron	0.125	rs791250043	3
AC.GT	15	108503250	108503260	5.5	.	.	intergenic	0.131	.	3
ACAT.ATGT	15	108512707	108512737	7.75	.	.	intergenic	-0.345	rs793428285	2
AC.GT	15	108608676	108608687	6	.	.	intergenic	-0.134	.	3
AT.AT	15	108640333	108640342	5	.	.	intergenic	.	rs789282174	3
AAAAAG.CTTTTT	15	108722656	108722675	3.33333	.	.	intergenic	.	rs790286752	2
AC.GT	15	108814162	108814198	18.5	.	.	intergenic	0.43	rs793817584	2
AT.AT	15	108956914	108956926	6.5	.	.	intergenic	0.091	182863 rs7917	3

AAT.ATT	15	109191497	109191508	4	.	.	intergenic	-0.097	.	2
AAAAT.ATTTT	15	109403485	109403502	3.6	.	.	intergenic	0.091	rs792482748	2
AT.AT	15	109410657	109410667	5.5	.	.	intergenic	0.04	rs792740216	3
AT.AT	15	109549538	109549547	5	.	.	intergenic	.	.	4
AT.AT	15	109573787	109573797	5.5	.	.	intergenic	-0.064	.	2
AAAT.ATTT	15	109841764	109841783	5	.	.	intergenic	.	rs789694109	2
AT.AT	15	109874388	109874400	6.5	.	.	intergenic	-0.284	547461 rs7879	2
AGAT.ATCT	15	110295972	110296008	9.25	.	.	intergenic	0.187	731584 rs7888	2
AT.AT	15	110306837	110306866	15	.	.	intergenic	.	rs789875629	2
AAAT.ATTT	15	110310834	110310860	6.75	.	.	intergenic	.	375930 rs7901	3
AC.GT	15	110347776	110347792	8.5	.	.	intergenic	0.677	rs787963884	2
AAAC.GTTT	15	110781780	110781798	4.75	.	.	intergenic	0.057	rs786740604	2
AC.GT	15	110838111	110838126	8	.	.	intergenic	.	042855 rs7921	2
AAAC.GTTT	15	111051623	111051636	3.5	ENSSSCT00000017493	STK17B	intron	0.285	294316 rs7887	2
AAAC.GTTT	15	111064749	111064763	3.75	ENSSSCT00000028914	.	promoter	0.081	202182 rs7872	2
AC.GT	15	111999995	112000008	7	ENSSSCT00000025335	.	promoter	0.164	rs788572402	2
ACCCT.AGGGT	15	112024852	112024873	3.66667	.	.	intergenic	.	rs793108681	2
AC.GT	15	112089396	112089430	17.5	ENSSSCT00000017501	.	intron	-0.077	688132 rs7874	3
AT.AT	15	112130273	112130285	6.5	ENSSSCT00000017501	.	intron	0.264	rs789383769	3
AAAAT.ATTTT	15	112332226	112332244	3.8	ENSSSCT00000017501	.	intron	0.289	rs790554071	2
AAAC.GTTT	15	112354131	112354150	5	ENSSSCT00000017501	.	intron	0.14	rs789065721	2
AAAT.ATTT	15	112451393	112451416	6	ENSSSCT00000017503	SF3B1	intron	0.316	rs793790133	2
AAAC.GTTT	15	112483180	112483198	4.75	.	.	intergenic	0.238	rs793542383	3
AAC.GTT	15	112506392	112506407	5.33333	.	.	intergenic	.	756404 rs7910	2
AAAAAT.ATTTTT	15	112553678	112553697	3.33333	.	.	intergenic	.	rs788390624	2
AC.GT	15	112868467	112868490	12	.	.	intergenic	0.051	rs787825690	4
AC.GT	15	113022897	113022915	9.5	.	.	intergenic	.	rs787800778	2
AAAC.GTTT	15	113077456	113077476	5.25	.	.	intergenic	-0.093	rs793286634	2
AAC.GTT	15	113195289	113195302	4.66667	.	.	intergenic	-0.313	rs788961736	2
AC.GT	15	113199600	113199618	9.5	.	.	intergenic	0.064	597340 rs7938	3
AATG.CATT	15	113275272	113275289	4.5	.	.	intergenic	0.145	rs792733667	2
AAAG.CTTT	15	113532911	113532930	5	.	.	intergenic	-0.312	.	2
AATG.CATT	15	113593933	113593955	5.75	.	.	intergenic	0.042	rs789183064	2
AAAAC.GTTTT	15	113616171	113616186	3.2	.	.	intergenic	.	rs690870914	2
AAT.ATT	15	113725391	113725403	4.33333	.	.	intergenic	.	491167 rs7932	2
AC.GT	15	113762221	113762235	7.5	.	.	intergenic	0.193	.	4
AC.GT	15	113932454	113932468	7.5	.	.	intergenic	0.056	rs790521332	2
AC.GT	15	114124732	114124748	8.5	.	.	intergenic	0.609	rs789718198	2
AAAC.GTTT	15	114160306	114160338	8.25	.	.	intergenic	0.188	.	4
AAAC.GTTT	15	114602624	114602638	3.75	.	.	intergenic	.	124687 rs7903	2
AAAC.GTTT	15	114810036	114810055	5	.	.	intergenic	.	rs786803074	3
AT.AT	15	114813014	114813023	5	.	.	intergenic	0.101	.	3
AAAG.CTTT	15	114938164	114938185	5.5	.	.	intergenic	0.035	rs792073771	2
AAAC.GTTT	15	114975818	114975845	7	.	.	intergenic	0.094	rs790978672	2
AAAC.GTTT	15	114977058	114977084	6.75	.	.	intergenic	0	rs790228788	2
AAAAG.CTTTT	15	115273433	115273454	4.4	ENSSSCT00000017518	SPATS2L	intron	0.163	.	2
AC.GT	15	115297523	115297534	6	ENSSSCT00000017518	SPATS2L	intron	0.986	556915 rs7889	2
AAAC.GTTT	15	115358312	115358340	7.25	ENSSSCT00000017519	.	intron	.	rs791739267	2
AAAC.GTTT	15	115491394	115491409	4	ENSSSCT00000027335	AOX1	intron	-0.016	rs793097205	2
AT.AT	15	115498717	115498728	6	ENSSSCT00000027335	AOX1	intron	0.035	.	2
AT.AT	15	115589646	115589655	5	.	.	intergenic	.	.	3
AAT.ATT	15	115729485	115729496	4	.	.	intergenic	-0.217	437829 rs7886	2
AAC.GTT	15	115737379	115737397	6.33333	ENSSSCT00000017522	BZW1	intron	.	rs793150996	4
AT.AT	15	115827525	115827534	5	ENSSSCT00000017521	.	intron	0.048	.	2
AAAC.GTTT	15	116098836	116098859	6	.	.	intergenic	.	786469 rs7896	2
AC.GT	15	116118593	116118615	11.5	ENSSSCT00000017531	FLIP-L	intron	.	rs793247754	2
AAAC.GTTT	15	116212187	116212209	5.75	.	.	intergenic	0.022	rs786651658	2
AAAC.GTTT	15	117416845	117416864	5	.	.	intergenic	.	rs793387572	2
AAAT.ATTT	15	117445814	117445826	3.25	ENSSSCT00000017544	BMPR2	intron	.	rs791857228	2
AAAG.CTTT	15	117461648	117461662	3.75	ENSSSCT00000017544	BMPR2	intron	.	767276 rs6962	2
AT.AT	15	117748751	117748763	6.5	.	.	intergenic	.	rs792360520	3
AG.CT	15	117761028	117761037	5	.	.	intergenic	.	.	2
AT.AT	15	117858850	117858859	5	ENSSSCT00000017547	WDR12	promoter	0.147	rs791566384	2
AT.AT	15	117944975	117944988	7	.	.	intergenic	0.212	rs792168015	2
AG.CT	15	118505636	118505653	9	.	.	intergenic	1.948	rs793397474	2
ATCC.GGAT	15	118706823	118706844	5.5	ENSSSCT00000024007	ICOS	intron	0.168	rs786547664	2
AG.CT	15	118816275	118816285	5.5	.	.	intergenic	0.285	rs787317433	3
AATT.AATT	15	118857318	118857337	5	.	.	intergenic	-0.165	464511 rs7867	2
AAT.ATT	15	118881133	118881146	4.66667	.	.	intergenic	0.599	rs789498980	2
AAAC.GTTT	15	118991571	118991585	3.75	.	.	intergenic	.	727904 rs7867	2
AAAC.GTTT	15	119178698	119178713	4	.	.	intergenic	0.146	rs793163140	2
AC.GT	15	119186487	119186503	8.5	.	.	intergenic	0.101	.	2
AAAC.GTTT	15	119249841	119249863	5.75	.	.	intergenic	.	rs792044808	2
AT.AT	15	119274892	119274909	9	.	.	intergenic	-0.005	rs793367622	2
AG.CT	15	119283674	119283689	8	.	.	intergenic	-0.125	rs792856383	2
AC.GT	15	119307092	119307122	15.5	.	.	intergenic	.	rs786680174	2
AAAAC.GTTTT	15	119387730	119387748	3.8	.	.	intergenic	.	rs789695395	2
AT.AT	15	119651208	119651222	7.5	.	.	intergenic	-0.026	rs790052483	3
AAAC.GTTT	15	119807648	119807667	5	.	.	intergenic	0.725	rs790057807	2
AG.CT	15	119823450	119823460	5.5	.	.	intergenic	-0.048	.	2
AC.GT	15	120472082	120472096	7.5	ENSSSCT00000017554	.	intron	-0.066	341928 rs7871	2
AC.GT	15	120666845	120666855	5.5	.	.	intergenic	.	rs788224087	2
AAC.GTT	15	120709771	120709787	5.66667	.	.	intergenic	0.239	rs790458004	2
AAAAAT.ATTTTT	15	120942261	120942279	3.16667	.	.	intergenic	.	rs788870431	2

AAAT.ATTT	15	121002793	121002807	3.75	ENSSSCT00000017560	EEF1B2	intron	4.2	rs791434178	2
AAAT.ATTT	15	121002793	121002807	3.75	ENSSSCT00000020378	snoZ196	promoter	4.2	rs791434178	2
AAAT.ATTT	15	121002793	121002807	3.75	ENSSSCT00000021282	SNORA41	promoter	4.2	rs791434178	2
AAAAT.ATTTT	15	121176010	121176029	4	.	.	intergenic	0.145	rs793494655	2
AAAAG.CTTTT	15	121204412	121204435	4.8	.	.	intergenic	.	rs788674562	2
AAAAAG.CTTTT	15	121448643	121448667	4.16667	ENSSSCT00000017564	ADAM23	intron	-0.002	.	2
AT.AT	15	121525816	121525826	5.5	.	.	intergenic	0.475	.	2
AAAG.CTTT	15	121535733	121535751	4.75	.	.	intergenic	0.103	537956 rs7911	2
AAAC.GTTT	15	121668445	121668459	3.75	.	.	intergenic	0.022	324278 rs7907	2
AAAAC.GTTTT	15	121930930	121930953	4.8	.	.	intergenic	0.183	313851 rs7886	2
AC.GT	15	121945475	121945489	7.5	.	.	intergenic	0.607	.	2
AAAC.GTTT	15	122027580	122027596	4.25	.	.	intergenic	0.416	313866 rs7877	2
AAAT.ATTT	15	122117248	122117266	4.75	.	.	intergenic	-0.016	.	2
AG.CT	15	122150011	122150028	9	.	.	intergenic	1.026	rs788382420	2
AAC.GTT	15	122187663	122187679	5.66667	.	.	intergenic	-0.105	rs790520205	2
AG.CT	15	122197176	122197188	6.5	.	.	intergenic	0.372	rs790368228	2
AAAC.GTTT	15	122290296	122290318	5.75	.	.	intergenic	.	rs793042700	2
AAAAAG.CTTTT	15	122324222	122324245	4	.	.	intergenic	0.534	040437 rs7934	2
AAAAAG.CTTTT	15	122373547	122373572	4.33333	ENSSSCT00000025237	METTL21A	3'utr	0.29	rs789559994	2
AC.GT	15	122414927	122414946	10	.	.	intergenic	0.214	.	4
AT.AT	15	122568454	122568473	10	ENSSSCT00000022797	.	intron	0.109	209632 rs7910	5
AAAT.ATTT	15	122839978	122839990	3.25	.	.	intergenic	0.027	rs789472264	2
AAC.GTT	15	122866014	122866027	4.66667	ENSSSCT00000026412	IDH1	intron	1.615	rs793724428	3
AC.GT	15	122939050	122939070	10.5	ENSSSCT00000017580	PIKFYVE	intron	0.212	rs787951199	4
AC.GT	15	122947648	122947659	6	ENSSSCT00000017580	PIKFYVE	intron	.	.	2
AC.GT	15	122968164	122968174	5.5	ENSSSCT00000017580	PIKFYVE	intron	.	rs787280776	4
AAAC.GTTT	15	123148514	123148530	4.25	ENSSSCT00000017581	PTH2R	intron	0.128	rs790183989	2
AC.GT	15	123206204	123206216	6.5	.	.	intergenic	.	.	2
AAAAC.GTTTT	15	123266809	123266845	7.4	.	.	intergenic	.	rs791613716	2
AT.AT	15	123393198	123393210	6.5	.	.	intergenic	.	rs790719904	2
AT.AT	15	123484761	123484770	5	.	.	intergenic	0.13	rs789113459	2
AAAAC.GTTTT	15	123537363	123537383	4.2	.	.	intergenic	0.197	rs791740796	2
AAT.ATT	15	123638217	123638242	8.66667	.	.	intergenic	-0.03	rs792979141	2
AT.AT	15	123684816	123684835	10	.	.	intergenic	.	rs791016705	2
AC.GT	15	123839468	123839484	8.5	.	.	intergenic	0.007	579550 rs7871	3
AC.GT	15	123903359	123903369	5.5	.	.	intergenic	0.283	rs786410197	2
AAAT.ATTT	15	123920586	123920603	4.5	.	.	intergenic	0.019	355286 rs7921	2
AAT.ATT	15	123953245	123953282	12.6667	.	.	intergenic	0.239	rs787412146	2
AAAT.ATTT	15	124013974	124014002	7.25	.	.	intergenic	.	437597 rs7889	2
AAAC.GTTT	15	124061325	124061344	5	.	.	intergenic	0.507	505097 rs7881	2
AAC.GTT	15	124088928	124088948	7	.	.	intergenic	0.082	234825 rs7914	4
AAC.GTT	15	124220854	124220869	5.33333	ENSSSCT00000017586	UNC80	intron	-0.08	729699 rs7897	2
AAC.GTT	15	124220854	124220869	5.33333	ENSSSCT00000030483	UNC80	intron	-0.08	729699 rs7897	2
AAAC.GTTT	15	124266384	124266408	6.25	ENSSSCT00000017586	UNC80	intron	0.096	rs791516435	2
AAAC.GTTT	15	124266384	124266408	6.25	ENSSSCT00000030483	UNC80	intron	0.096	rs791516435	2
AAAC.GTTT	15	124366837	124366849	3.25	ENSSSCT00000017586	UNC80	intron	0.256	982478 rs7873	2
AAAC.GTTT	15	124366837	124366849	3.25	ENSSSCT00000030483	UNC80	intron	0.256	982478 rs7873	2
AAAAT.ATTTT	15	124384441	124384459	3.8	ENSSSCT00000017586	UNC80	intron	0.044	587053 rs7911	2
AAAAT.ATTTT	15	124384441	124384459	3.8	ENSSSCT00000030483	UNC80	intron	0.044	587053 rs7911	2
AG.CT	15	124425186	124425202	8.5	ENSSSCT00000017586	UNC80	intron	0.328	rs792284114	3
AG.CT	15	124425186	124425202	8.5	ENSSSCT00000030483	UNC80	intron	0.328	rs792284114	3
AC.GT	15	124431066	124431078	6.5	ENSSSCT00000017586	UNC80	intron	0.204	rs786726237	2
AC.GT	15	124431066	124431078	6.5	ENSSSCT00000030483	UNC80	intron	0.204	rs786726237	2
AG.CT	15	124625284	124625300	8.5	ENSSSCT00000029240	KANSL1L	intron	0.217	rs788010873	2
AG.CT	15	124625284	124625300	8.5	ENSSSCT00000025006	KANSL1L	intron	0.217	rs788010873	2
AG.CT	15	124625284	124625300	8.5	ENSSSCT00000031966	KANSL1L	intron	0.217	rs788010873	2
AT.AT	15	124910138	124910149	6	.	.	intergenic	0.219	.	2
AC.GT	15	125140661	125140681	10.5	.	.	intergenic	.	.	2
AAC.GTT	15	125339179	125339194	5.33333	.	.	intergenic	-0.022	585248 rs7908	2
AC.GT	15	125491436	125491448	6.5	.	.	intergenic	0.131	.	4
AAAC.GTTT	15	125551210	125551224	3.75	.	.	intergenic	-0.019	520536 rs7917	2
AG.CT	15	125769237	125769250	7	.	.	intergenic	-0.042	060499 rs7911	3
AC.GT	15	125926827	125926837	5.5	ENSSSCT00000027992	.	intron	-0.068	rs789442837	2
AAC.GTT	15	125940029	125940048	6.66667	ENSSSCT00000027992	.	intron	-0.137	rs789773521	2
AAT.ATT	15	125989313	125989336	8	.	.	intergenic	0.035	rs792938575	2
AAAT.ATTT	15	126072565	126072595	7.75	.	.	intergenic	0.145	rs787276346	2
AC.GT	15	126174829	126174851	11.5	ENSSSCT00000017595	.	intron	-0.106	.	6
AT.AT	15	126307265	126307289	12.5	.	.	intergenic	0.001	391647 rs7914	2
AAT.ATT	15	126391992	126392009	6	.	.	intergenic	-0.142	rs789061113	2
AAAG.CTTT	15	126943131	126943145	3.75	.	.	intergenic	-0.089	rs787713420	2
AC.GT	15	127092595	127092609	7.5	.	.	intergenic	-0.085	rs786515342	2
AAC.GTT	15	127273680	127273691	4	.	.	intergenic	.	rs791642174	2
AAT.ATT	15	127299191	127299209	6.33333	.	.	intergenic	-0.132	rs792230351	2
AT.AT	15	127405836	127405847	6	.	.	intergenic	1.273	572288 rs7928	3
AAAC.GTTT	15	127410267	127410293	6.75	.	.	intergenic	0.207	125571 rs7931	2
AAAT.ATTT	15	127809072	127809098	6.75	.	.	intergenic	0.125	rs793591478	2
AAT.ATT	15	127888251	127888271	7	.	.	intergenic	0.17	314510 rs7931	2
AG.CT	15	127977067	127977078	6	.	.	intergenic	0.876	rs787610732	2
AAC.GTT	15	128088030	128088043	4.66667	.	.	intergenic	.	rs790124933	3
ACCT.AGGT	15	128344102	128344124	5.75	.	.	intergenic	0.237	rs793018247	2
AAC.GTT	15	128386939	128386964	8.66667	.	.	intergenic	.	rs791258576	2
AG.CT	15	128480964	128480978	7.5	.	.	intergenic	0.301	.	2
AT.AT	15	128650188	128650218	15.5	.	.	intergenic	0.054	rs791806284	2
AAAAAC.GTTTT	15	128747966	128747988	3.83333	.	.	intergenic	-0.111	rs786701728	2

AAC.GTT	15	128995624	128995644	7	.	.	intergenic	0.045	41136 rs7932	2
AT.AT	15	129377393	129377403	5.5	.	.	intergenic	.	rs786659537	3
AAC.GTT	15	129544788	129544801	4.66667	.	.	intergenic	-0.001	rs788579348	2
AAC.GTT	15	129546602	129546617	5.33333	.	.	intergenic	.	.	2
AAAAT.ATTTT	15	129566300	129566319	3.33333	.	.	intergenic	0.087	.	2
AT.AT	15	129576559	129576568	5	.	.	intergenic	.	.	2
AAAC.GTTT	15	129577752	129577770	4.75	.	.	intergenic	0.086	70378 rs7865	2
AAAAG.CTTTT	15	129686945	129686970	5.2	.	.	intergenic	0.277	15689 rs7863	2
AT.AT	15	129709107	129709124	9	.	.	intergenic	-0.081	63845 rs7925	4
AG.CT	15	130440174	130440184	5.5	.	.	intergenic	.	rs790056433	3
AC.GT	15	130607462	130607471	5	.	.	intergenic	-0.003	.	2
ATC.GAT	15	130752031	130752057	9	.	.	intergenic	0.018	rs788107186	4
AAC.GTT	15	131052471	131052487	5.66667	ENSSSCT00000017612	MREG	intron	.	170863 rs7923	2
AC.GT	15	131182674	131182690	8.5	ENSSSCT00000028863	PECR	intron	0.144	rs793625575	2
AC.GT	15	131490529	131490557	14.5	.	.	intergenic	0.039	rs791366203	2
AG.CT	15	131652973	131652984	6	.	.	intergenic	.	rs789426217	2
AAAC.GTTT	15	131820398	131820410	3.25	.	.	intergenic	0.074	rs792824034	2
AT.AT	15	131842442	131842457	8	.	.	intergenic	-0.011	.	2
AG.CT	15	132618887	132618908	11	.	.	intergenic	-0.312	92660 rs7903	4
AAAAT.ATTTT	15	132658135	132658154	4	.	.	intergenic	-0.093	rs787778090	2
AG.CT	15	132775836	132775846	5.5	.	.	intergenic	1.068	rs788844613	2
AC.GT	15	133250493	133250509	8.5	ENSSSCT00000017620	RUFY4	intron	-0.13	.	2
AAAC.GTTT	15	133477499	133477514	4	ENSSSCT00000017637	VIL1	promoter	.	rs793873913	2
AAC.GTT	15	133822441	133822455	5	ENSSSCT00000032395	.	intron	.	.	4
AAC.GTT	15	133822441	133822455	5	ENSSSCT00000031662	.	intron	.	.	4
AAAC.GTTT	15	134257965	134257979	3.75	ENSSSCT00000017654	GLB1L	intron	0.065	rs787468597	2
AG.CT	15	134761723	134761739	8.5	.	.	intergenic	-0.053	rs793758891	2
AT.AT	15	134808887	134808897	5.5	.	.	intergenic	0.057	rs790597621	2
AAG.CTT	15	135236177	135236196	6.66667	.	.	intergenic	0.386	rs788642913	2
AAG.CTT	15	135250247	135250261	5	.	.	intergenic	-0.071	24102 rs7916	2
AAAC.GTTT	15	135354595	135354609	3.75	.	.	intergenic	0.129	rs787558168	2
AC.GT	15	135361203	135361215	6.5	.	.	intergenic	0.266	.	2
AG.CT	15	135397119	135397133	7.5	.	.	intergenic	-0.077	rs787396458	2
AAT.ATT	15	135402649	135402670	7.33333	.	.	intergenic	1.518	161516 rs7933	2
AAGG.CCTT	15	135465667	135465693	6.75	.	.	intergenic	0.007	rs786582615	2
AAAAG.CTTTT	15	135898651	135898669	3.8	.	.	intergenic	1.663	rs789090798	2
AC.GT	15	135957380	135957390	5.5	.	.	intergenic	-0.159	.	3
AG.CT	15	135978378	135978394	8.5	.	.	intergenic	.	.	4
ATCC.GGAT	15	136127507	136127528	5.5	.	.	intergenic	-0.158	58927 rs7888	3
AG.CT	15	136136511	136136530	10	.	.	intergenic	-0.009	rs793370809	2
AC.GT	15	136154645	136154657	6.5	.	.	intergenic	-0.013	rs788798023	2
AC.GT	15	136338461	136338493	16.5	.	.	intergenic	-0.123	rs793785114	3
AT.AT	15	136338904	136338913	5	.	.	intergenic	-0.047	.	3
AAAAC.GTTTT	15	136473220	136473248	4.83333	.	.	intergenic	0.117	rs792761591	2
AAAC.GTTT	15	136520783	136520805	5.75	.	.	intergenic	0.003	148725 rs7868	2
AAAC.GTTT	15	136649364	136649380	4.25	.	.	intergenic	-0.039	rs786563860	2
AAAC.GTTT	15	137044900	137044915	4	.	.	intergenic	.	97032 rs7905	2
AT.AT	15	137065803	137065817	7.5	.	.	intergenic	0.152	.	4
AAGG.CCTT	15	137087797	137087823	6.75	.	.	intergenic	0.06	19408 rs7934	3
AC.GT	15	137101018	137101027	5	.	.	intergenic	.	rs692147440	4
AG.CT	15	137132053	137132071	9.5	.	.	intergenic	0.107	.	3
AAAAC.GTTTT	15	137196361	137196378	3.6	.	.	intergenic	-0.225	rs789669619	2
AAAC.GTTT	15	137216831	137216852	5.5	.	.	intergenic	.	rs787717379	2
AG.CT	15	137428177	137428200	12	.	.	intergenic	-0.021	.	2
AAAT.ATTT	15	137438937	137438954	4.5	.	.	intergenic	0.179	66256 rs7874	2
AAAG.CTTT	15	137682720	137682738	4.75	.	.	intergenic	0.455	rs787411539	2
AGG.CCT	15	137701254	137701267	4.66667	.	.	intergenic	-0.105	rs790191279	2
AAT.ATT	15	137855481	137855494	4.66667	.	.	intergenic	0.086	rs789242092	2
AAAG.CTTT	15	137936064	137936093	7.5	.	.	intergenic	0.147	rs790239524	2
AAAG.CTTT	15	137960242	137960255	3.5	.	.	intergenic	-0.188	rs787309802	2
AAAC.GTTT	15	137969686	137969700	3.75	ENSSSCT00000017668	.	intron	0.092	rs788067326	2
AAT.ATT	15	137973918	137973935	6	ENSSSCT00000017668	.	intron	0.228	rs786778572	4
AT.AT	15	138585721	138585731	5.5	.	.	intergenic	-0.234	.	4
AAC.GTT	15	139030955	139030968	4.66667	.	.	intergenic	.	rs791106912	2
AAG.CTT	15	139100890	139100909	6.66667	.	.	intergenic	.	rs791614943	2
AAAAG.CTTTT	15	139204125	139204143	3.8	ENSSSCT00000017676	PN-1	intron	-0.248	rs792865622	2
AAAAG.CTTTT	15	139204125	139204143	3.8	ENSSSCT00000036636	PN-1	intron	-0.248	rs792865622	2
AC.GT	15	139275707	139275718	6	.	.	intergenic	0.151	.	3
AAAT.ATTT	15	139291369	139291391	5.75	.	.	intergenic	0.576	rs788604842	2
AAAT.ATTT	15	139309245	139309262	4.5	.	.	intergenic	.	rs793172179	3
AAAT.ATTT	15	139327230	139327247	4.5	.	.	intergenic	-0.486	rs788996463	2
AC.GT	15	139365057	139365071	7.5	.	.	intergenic	0.025	rs793001972	2
ACAT.ATGT	15	139459242	139459266	6.25	.	.	intergenic	-0.06	.	2
AAAAG.CTTTT	15	139579527	139579549	4.6	.	.	intergenic	-0.103	.	2
AAAC.GTTT	15	139605261	139605275	3.75	.	.	intergenic	-0.012	36829 rs7897	3
AAAAT.ATTTT	15	139793007	139793031	5	.	.	intergenic	0.07	rs790207634	2
AAAC.GTTT	15	139834560	139834582	5.75	.	.	intergenic	0.019	rs787836528	2
AAAAC.GTTTT	15	139977808	139977837	6	ENSSSCT00000017680	.	intron	-0.181	rs790790857	2
AAATG.CATTT	15	139981195	139981213	3.8	ENSSSCT00000017680	.	intron	-0.035	rs791916773	2
AAC.GTT	15	140056559	140056572	4.66667	ENSSSCT00000017680	.	intron	.	19323 rs7890	2
AAAT.ATTT	15	140216073	140216099	6.75	.	.	intergenic	-0.02	rs789477380	3
AAAC.GTTT	15	140222140	140222158	4.75	.	.	intergenic	-0.115	rs786766489	2
AT.AT	15	140549396	140549407	6	ENSSSCT00000017682	NYAP2	intron	.	rs792087984	2
AG.CT	15	140611525	140611534	5	ENSSSCT00000017682	NYAP2	intron	-0.201	rs789082819	2

AT.AT	15	140633616	140633637	11	ENSSSCT00000017682	NYAP2	intron	0.5	rs792742109	2
AC.GT	15	140642691	140642703	6.5	.	.	intergenic	-0.034	rs786654640	6
AAG.CTT	15	140784227	140784241	5	.	.	intergenic	-0.182	rs790075590	2
CACCC.GGGTG	15	141163693	141163723	5.16667	.	.	intergenic	-0.069	rs789539416	2
AT.AT	15	141423858	141423878	10.5	.	.	intergenic	.	.	2
AAT.ATT	15	141514377	141514395	6.33333	.	.	intergenic	-0.09	rs793862751	2
AC.GT	15	141800338	141800352	7.5	.	.	intergenic	0.081	rs788499365	3
AGCT.AGCT	15	141809488	141809507	5	.	.	intergenic	0.065	rs790283056	2
AAAAC.GTTTT	15	141862613	141862634	4.4	.	.	intergenic	0.241	.	2
AAG.CTT	15	142016956	142016969	4.66667	.	.	intergenic	0.045	rs787829493	2
AAAC.GTTT	15	142513121	142513139	4.75	ENSSSCT00000017689	.	intron	0.325	44265 rs7921	2
AAAC.GTTT	15	142513121	142513139	4.75	ENSSSCT00000026444	.	intron	0.325	44265 rs7921	2
AAT.ATT	15	142734438	142734452	5	.	.	intergenic	0.114	rs789639183	2
AAAAG.CTTTT	15	143073126	143073145	4	.	.	intergenic	0.041	rs786799239	2
AAT.ATT	15	143088629	143088652	8	.	.	intergenic	-0.217	.	2
AG.CT	15	143179730	143179742	6.5	.	.	intergenic	.	rs788620477	2
AAAC.GTTT	15	143337154	143337173	5	ENSSSCT00000017701	SPHKAP	intron	0.005	56261 rs7889	2
AAAAC.GTTTT	15	143687576	143687599	4.8	.	.	intergenic	.	rs789578302	3
AT.AT	15	143899014	143899024	5.5	.	.	intergenic	-0.016	rs793294371	2
AAAC.GTTT	15	144095988	144096002	3.75	.	.	intergenic	.	rs787771843	2
AC.GT	15	144292705	144292726	11	.	.	intergenic	0.054	.	3
AG.CT	15	144363289	144363301	6.5	.	.	intergenic	-0.094	.	2
AAAG.CTTT	15	144937897	144937916	5	ENSSSCT00000017707	SP140	intron	-0.911	49267 rs7892	2
AAAAG.CTTTT	15	145199833	145199861	5.8	ENSSSCT00000017712	.	intron	0.161	rs789995387	2
AAAAG.CTTTT	15	145213684	145213706	4.6	ENSSSCT00000017712	.	intron	-0.046	rs793132912	2
AAC.GTT	15	145308449	145308466	6	.	.	intergenic	.	21120 rs7862	2
AC.GT	15	145383217	145383227	5.5	.	.	intergenic	0.112	rs791115370	2
AT.AT	15	145427359	145427374	8	ENSSSCT00000017717	SPATA3	intron	-0.242	.	3
AAAC.GTTT	15	145573620	145573635	4	ENSSSCT00000017721	HTR2B	intron	0.284	59871 rs7879	2
AAAAAT.ATTTTT	15	145962668	145962685	3	.	.	intergenic	0.185	rs788583615	2
AAAC.GTTT	15	145966800	145966815	4	.	.	intergenic	0.083	rs787880291	2
AAAAC.GTTTT	15	145978027	145978042	3.2	.	.	intergenic	0.183	48501 rs7901	2
AAAC.GTTT	15	146079894	146079908	3.75	.	.	intergenic	0.004	rs790378726	2
AAAC.GTTT	15	146537944	146537968	6.25	ENSSSCT00000026575	DIS3L2	intron	0.047	rs792711253	2
ACAG.CTGT	15	146556448	146556470	5.75	ENSSSCT00000026575	DIS3L2	intron	0.206	rs787792615	2
AAAC.GTTT	15	146576038	146576058	5.25	ENSSSCT00000026575	DIS3L2	intron	.	02864 rs7889	2
AC.GT	15	146726837	146726859	11.5	ENSSSCT00000026675	.	intron	-0.173	75005 rs7863	2
AAAT.ATTT	15	147104600	147104622	5.75	ENSSSCT00000017739	EFHD1	intron	-0.211	rs792894506	2
AAAG.CTTT	15	147664543	147664556	3.5	ENSSSCT00000017748	INPP5D	intron	.	rs792433147	2
AAC.GTT	15	148177811	148177823	4.33333	ENSSSCT00000017756	MROH2A	intron	-0.161	rs790820110	2
AATC.GATT	15	148181045	148181063	4.75	ENSSSCT00000017756	MROH2A	intron	-0.179	rs792760060	2
AAAC.GTTT	15	148850029	148850043	3.75	.	.	intergenic	.	04165 rs7920	2
AC.GT	15	149171800	149171814	7.5	.	.	intergenic	-0.34	38402 rs7910	2
AAAC.GTTT	15	149395876	149395891	4	.	.	intergenic	.	rs790915493	2
AC.GT	15	149666631	149666649	9.5	.	.	intergenic	.	rs789866181	2
AAAC.GTTT	15	149699379	149699400	5.5	.	.	intergenic	-0.246	rs791094873	2
AC.GT	15	149933352	149933363	6	ENSSSCT00000022776	.	intron	-0.115	15502 rs7927	2
AC.GT	15	150362355	150362366	6	ENSSSCT00000017770	AGAP1	intron	-0.262	rs788241131	2
AC.GT	15	150439555	150439593	19.5	.	.	intergenic	.	rs792052557	2
ACAG.CTGT	15	150627874	150627895	5.5	.	.	intergenic	0.037	rs790020441	2
AC.GT	15	150738952	150738963	6	ENSSSCT00000017774	IQCA1	intron	-0.033	.	3
AAC.GTT	15	150766627	150766644	6	.	.	intergenic	-0.343	92006 rs7877	2
AAAC.GTTT	15	150935524	150935540	4.25	.	.	intergenic	-0.447	.	3
AAAC.GTTT	15	151054587	151054605	4.75	.	.	intergenic	-0.013	rs694576010	3
AC.GT	15	151238615	151238632	9	.	.	intergenic	-0.063	164749 rs7908	2
AG.CT	15	151252736	151252747	6	.	.	intergenic	0.206	rs788319938	2
AATG.CATT	15	151446281	151446306	6.5	.	.	intergenic	.	rs792541698	2
AAAC.GTTT	15	151490981	151490994	3.5	ENSSSCT00000028799	COL6A3	intron	.	14260 rs7884	2
AAAG.CTTT	15	151557585	151557598	3.5	.	.	intergenic	-0.099	.	2
AC.GT	15	151641080	151641091	6	.	.	intergenic	-0.055	rs789959989	3
AC.GT	15	151680850	151680866	8.5	.	.	intergenic	-0.185	rs788041648	2
AAAAC.GTTTT	15	151700853	151700871	3.8	.	.	intergenic	-0.524	rs787466862	2
AAAAC.GTTTT	15	151765685	151765707	4.6	ENSSSCT00000017783	.	intron	.	66741 rs7920	2
AAC.GTT	15	151965568	151965587	6.66667	.	.	intergenic	.	rs787234052	2
AAAC.GTTT	15	151991076	151991094	4.75	ENSSSCT00000025350	.	intron	0.564	63664 rs7927	2
AC.GT	15	152040314	152040326	6.5	.	.	intergenic	.	rs789970529	3
AAAC.GTTT	15	152305388	152305406	4.75	.	.	intergenic	0.054	rs696463367	2
AACAG.CTGTT	15	152576491	152576510	4	.	.	intergenic	.	rs793421777	2
AC.GT	15	153502186	153502198	6.5	.	.	intergenic	.	rs793007606	2
AG.CT	15	153563071	153563090	10	.	.	intergenic	-0.333	25811 rs7876	5
AT.AT	15	154289335	154289345	5.5	.	.	intergenic	-0.325	23139 rs7920	2
AAAT.ATTT	15	154819785	154819801	4.25	.	.	intergenic	.	.	2
AAT.ATT	15	155885930	155885943	4.66667	.	.	intergenic	.	64731 rs7877	2
AC.GT	15	155911454	155911465	6	.	.	intergenic	.	.	2
AT.AT	15	156592062	156592072	5.5	.	.	intergenic	.	rs792152734	2
AC.GT	15	156599466	156599475	5	.	.	intergenic	.	.	2
AG.CT	15	156614421	156614435	7.5	.	.	intergenic	-0.242	rs792550356	2
AT.AT	15	156616544	156616553	5	.	.	intergenic	-0.092	rs791537309	2
AAAC.GTTT	15	156702066	156702101	9	.	.	intergenic	.	83084 rs7915	2
AC.GT	15	156816275	156816286	6	.	.	intergenic	.	rs791462845	2
AG.CT	15	157103681	157103690	5	.	.	intergenic	.	.	4
AAAAAT.ATTTTT	15	157104262	157104285	4	.	.	intergenic	0.048	.	2
AAAAT.ATTTT	15	157411913	157411930	3.6	ENSSSCT00000017856	STAM2	intron	.	.	3
AAAAC.GTTTT	16	309158	309182	5	ENSSSCT00000018267	CTNND2	intron	-0.193	rs790276146	2

AAAAC.GTTTT	16	473932	473955	4.8	ENSSSCT00000018267	CTNND2	intron	0.003	rs791213137	2
AT.AT	16	521163	521174	6	.	.	intergenic	-0.179	.	2
AAT.ATT	16	536036	536049	4.66667	.	.	intergenic	0.087	rs789600614	3
AC.GT	16	627113	627122	5	.	.	intergenic	-0.385	.	2
AC.GT	16	640395	640407	6.5	.	.	intergenic	-0.166	389603 rs7901	3
AAC.GTT	16	655163	655177	5	.	.	intergenic	.	rs786925278	2
AC.GT	16	907214	907227	7	.	.	intergenic	-0.21	rs790935284	5
AAAT.ATTT	16	973076	973096	5.25	.	.	intergenic	0.121	rs791986786	2
ATC.GAT	16	1017559	1017577	6.33333	.	.	intergenic	.	511125 rs7916	2
AATG.CATT	16	1025409	1025434	6.5	.	.	intergenic	.	rs787894830	2
AAAC.GTTT	16	1128943	1128965	5.75	.	.	intergenic	.	528796 rs7905	2
AAC.GTT	16	1142023	1142036	4.66667	.	.	intergenic	0.491	rs789368904	2
AT.AT	16	1607813	1607832	10	.	.	intergenic	.	.	2
AT.AT	16	1681383	1681397	7.5	.	.	intergenic	-0.105	rs789032240	2
AT.AT	16	1704183	1704193	5.5	.	.	intergenic	.	.	4
AAAT.ATTT	16	2371030	2371043	3.5	.	.	intergenic	0.093	.	2
AAAT.ATTT	16	2375310	2375338	7.25	.	.	intergenic	-0.13	272086 rs7888	2
AC.GT	16	2491482	2491494	6.5	.	.	intergenic	-0.208	916235 rs7922	2
AC.GT	16	2642753	2642763	5.5	.	.	intergenic	.	rs790740427	4
AT.AT	16	2671641	2671650	5	.	.	intergenic	-0.16	.	4
AAAC.GTTT	16	2676988	2677007	5	.	.	intergenic	-0.087	rs792404444	2
AAAAC.GTTTT	16	2690869	2690884	3.2	.	.	intergenic	0.116	rs789481396	2
AAAT.ATTT	16	2717331	2717345	3.75	.	.	intergenic	.	985466 rs7897	2
AAAC.GTTT	16	2764253	2764267	3.75	.	.	intergenic	.	rs788006491	2
AG.CT	16	2960239	2960249	5.5	.	.	intergenic	0.129	.	2
AC.GT	16	3117660	3117672	6.5	.	.	intergenic	-0.232	293736 rs7938	2
AC.GT	16	3228715	3228724	5	.	.	intergenic	0.023	.	2
AC.GT	16	3281457	3281471	7.5	.	.	intergenic	0.21	rs789691695	3
AG.CT	16	3285377	3285390	7	.	.	intergenic	-0.14	rs792332638	4
AT.AT	16	3575752	3575761	5	.	.	intergenic	0.425	.	2
ACAG.CTGT	16	3581051	3581074	6	.	.	intergenic	-0.108	195240 rs7934	2
AC.GT	16	3840208	3840218	5.5	ENSSSCT00000024898	.	intron	1.004	rs792841631	4
AC.GT	16	3956502	3956528	13.5	ENSSSCT00000024898	.	intron	-0.314	rs791404009	2
ACG.CGT	16	4250859	4250873	5	ENSSSCT00000018268	.	intron	-0.169	rs787305763	2
AC.GT	16	4346952	4346962	5.5	.	.	intergenic	0.309	.	2
AAAT.ATTT	16	4382412	4382423	3	.	.	intergenic	.	rs789602007	2
AGC.GCT	16	4419386	4419401	5.33333	ENSSSCT00000018270	FAM105B	intron	1.291	.	2
AC.GT	16	4424116	4424135	10	ENSSSCT00000018270	FAM105B	intron	-0.174	rs788760721	2
AAAC.GTTT	16	4572927	4572946	5	ENSSSCT00000018271	ANKH	intron	0.087	163330 rs7933	2
AG.CT	16	4637163	4637191	14.5	.	.	intergenic	.	725772 rs7903	2
AT.AT	16	4651909	4651919	5.5	.	.	intergenic	.	rs792904217	2
AAAAC.GTTTT	16	4761147	4761168	3.66667	.	.	intergenic	-0.097	rs789603936	2
ATC.GAT	16	4786171	4786199	9.66667	.	.	intergenic	0.101	rs793362052	2
AGAGAT.ATCTC	16	4918486	4918505	3.33333	.	.	intergenic	0.012	570792 rs7928	2
AAAAT.ATTTT	16	4959088	4959114	5.4	.	.	intergenic	0.123	rs787866680	2
ACAT.ATGT	16	5096334	5096354	5.25	.	.	intergenic	.	rs789148362	2
AT.AT	16	5174080	5174089	5	.	.	intergenic	-0.019	.	2
AAAC.GTTT	16	5279899	5279926	7	.	.	intergenic	0.047	rs792878887	2
AT.AT	16	5311119	5311131	6.5	.	.	intergenic	0.431	194423 rs7920	4
AC.GT	16	5487186	5487196	5.5	.	.	intergenic	-0.246	.	3
AC.GT	16	5582903	5582914	6	ENSSSCT00000025512	FBXL7	promoter	0.052	.	2
AAC.GTT	16	5642930	5642941	4	.	.	intergenic	0.641	109891 rs7899	2
AAC.GTT	16	5740511	5740528	6	.	.	intergenic	.	903888 rs7865	2
AC.GT	16	5853796	5853818	11.5	.	.	intergenic	0.042	008461 rs7881	2
AAG.CTT	16	5932047	5932061	5	.	.	intergenic	-0.108	rs789484523	2
AG.CT	16	6176524	6176540	8.5	ENSSSCT00000018280	FAM134B	intron	-0.037	477225 rs7921	3
AAAG.CTTT	16	6287099	6287113	3.75	ENSSSCT00000018280	FAM134B	intron	-0.125	076093 rs7896	2
AAAC.GTTT	16	6346092	6346111	5	.	.	intergenic	0.132	305284 rs7123	2
AAAT.ATTT	16	6448575	6448596	5.5	ENSSSCT00000018282	MYO10	intron	0.088	rs703485085	2
AT.AT	16	6453656	6453665	5	ENSSSCT00000018282	MYO10	intron	-0.252	608113 rs7931	2
AAAC.GTTT	16	6508460	6508482	5.75	.	.	intergenic	.	rs791626591	2
AAAAC.GTTTT	16	6526660	6526675	3.2	.	.	intergenic	-0.314	rs792797476	2
ACC.GGT	16	6623494	6623512	6.33333	.	.	intergenic	.	rs791882449	2
AG.CT	16	6644920	6644931	6	.	.	intergenic	.	rs789904231	2
AAAT.ATTT	16	6691592	6691619	7	.	.	intergenic	.	rs791900336	3
ACC.GGT	16	6827287	6827306	6.66667	.	.	intergenic	-0.061	048379 rs7888	3
AAAAC.GTTTT	16	6886409	6886430	4.4	.	.	intergenic	-0.2	rs793178988	2
AC.GT	16	7030195	7030212	9	.	.	intergenic	0.088	rs787747886	3
AAATT.AATTT	16	7099861	7099881	4.2	.	.	intergenic	.	rs793534604	2
AG.CT	16	7194013	7194023	5.5	.	.	intergenic	0.107	rs792903793	3
AAAAC.GTTTT	16	7580806	7580830	5	.	.	intergenic	0.277	313087 rs7897	2
AAAAAG.CTTTTT	16	7675239	7675263	4.16667	.	.	intergenic	-0.046	654083 rs7864	2
AC.GT	16	7713286	7713309	12	.	.	intergenic	-0.106	.	2
AT.AT	16	7716195	7716214	10	.	.	intergenic	-0.175	rs697011415	2
AAC.GTT	16	8057692	8057711	6.66667	.	.	intergenic	.	721295 rs7899	2
AC.GT	16	8148959	8148982	12	.	.	intergenic	0.245	rs791690521	5
ACAG.CTGT	16	8230603	8230625	5.75	.	.	intergenic	-0.195	rs789692052	2
AAAAAC.GTTTTT	16	8354460	8354489	5	.	.	intergenic	-0.121	rs791919519	2
AG.CT	16	8429112	8429121	5	.	.	intergenic	-0.123	rs786617499	3
AAC.GTT	16	8431359	8431370	4	.	.	intergenic	0.028	rs790770439	2
AG.CT	16	8467095	8467105	5.5	.	.	intergenic	.	.	3
AC.GT	16	8598983	8598995	6.5	.	.	intergenic	-0.203	rs788640291	2
AAAC.GTTT	16	8690535	8690549	3.75	.	.	intergenic	-0.046	rs788507743	2
AC.GT	16	8693728	8693739	6	.	.	intergenic	0.03	.	3

AC.GT	16	9028466	9028476	5.5	.	.	intergenic	-0.326	.	3
AC.GT	16	9042390	9042399	5	.	.	intergenic	-0.123	.	3
ATC.GAT	16	9077186	9077206	7	.	.	intergenic	-0.24	rs787543657	4
AC.GT	16	9114968	9114981	7	.	.	intergenic	.	321395 rs7911	2
AAAT.ATTT	16	9171554	9171575	5.5	.	.	intergenic	.	rs791012944	2
AAAC.GTTT	16	9345692	9345706	3.75	.	.	intergenic	.	481233 rs7876	2
ACAT.ATGT	16	9630025	9630041	4.25	.	.	intergenic	-0.026	rs787672581	2
AT.AT	16	9649892	9649902	5.5	.	.	intergenic	-0.029	315771 rs7922	2
AC.GT	16	9713002	9713014	6.5	.	.	intergenic	.	rs793286454	2
AC.GT	16	9806498	9806510	6.5	.	.	intergenic	0.033	.	4
AT.AT	16	9879197	9879206	5	.	.	intergenic	.	171269 rs7897	2
AAG.CTT	16	9964461	9964474	4.66667	.	.	intergenic	-1.362	rs793567075	2
AAAAT.ATTTT	16	9965769	9965788	4	.	.	intergenic	-0.136	372467 rs7866	2
AAAT.ATTT	16	10193803	10193817	3.75	.	.	intergenic	0.062	rs788168343	2
AAAAAC.GTTTTT	16	10261217	10261252	6	.	.	intergenic	-0.084	rs790717861	2
AG.CT	16	10353184	10353212	14.5	.	.	intergenic	-0.098	.	2
AAAC.GTTT	16	10378230	10378257	7	.	.	intergenic	.	.	2
AC.GT	16	10481635	10481648	7	.	.	intergenic	.	.	3
AAAC.GTTT	16	10485298	10485317	5	.	.	intergenic	-0.338	rs791487507	2
AAAC.GTTT	16	10853196	10853210	3.75	ENSSSCT00000018288	CDH12	intron	.	330015 rs7882	2
AAC.GTT	16	10973179	10973195	5.66667	.	.	intergenic	-0.002	368614 rs7864	2
AAT.ATT	16	11063148	11063176	9.66667	ENSSSCT00000024837	.	intron	-0.101	rs788477452	2
AG.CT	16	11081529	11081539	5.5	ENSSSCT00000024837	.	intron	-0.23	rs789058157	3
AC.GT	16	11253367	11253389	11.5	.	.	intergenic	-0.251	rs789367670	3
AT.AT	16	11258926	11258935	5	.	.	intergenic	.	rs789794638	3
AAT.ATT	16	11342899	11342913	5	.	.	intergenic	-0.133	rs789735987	2
AAAC.GTTT	16	11359455	11359470	4	.	.	intergenic	.	rs791537725	2
AAAC.GTTT	16	11368974	11369009	9	.	.	intergenic	-0.02	rs790020123	2
AC.GT	16	11585318	11585328	5.5	.	.	intergenic	0.153	.	2
AAAT.ATTT	16	11684938	11684952	3.75	.	.	intergenic	0.002	121586 rs7879	2
AC.GT	16	11809894	11809908	7.5	.	.	intergenic	-0.136	rs788930911	2
AC.GT	16	11964585	11964597	6.5	.	.	intergenic	-0.074	.	3
AAC.GTT	16	12057667	12057681	5	.	.	intergenic	0.016	.	2
AT.AT	16	12107893	12107906	7	.	.	intergenic	-0.083	rs699842846	3
AC.GT	16	12321417	12321427	5.5	.	.	intergenic	-0.209	rs692348225	3
AG.CT	16	12384646	12384669	12	.	.	intergenic	-0.245	.	2
AT.AT	16	12478535	12478544	5	.	.	intergenic	.	.	2
AT.AT	16	12497569	12497581	6.5	.	.	intergenic	0.225	rs695844130	2
AAAG.CTTT	16	12564187	12564230	11	.	.	intergenic	-0.346	rs787476019	2
AAAAG.CTTTT	16	12581037	12581067	6.2	.	.	intergenic	-0.37	rs793131971	2
ATC.GAT	16	12619269	12619289	7	.	.	intergenic	-0.076	rs791528234	2
AAG.CTT	16	12639676	12639697	7.33333	ENSSSCT00000018292	CDH10	intron	0.126	.	4
AAAAT.ATTTT	16	12705040	12705062	4.6	ENSSSCT00000018292	CDH10	intron	-0.11	rs793204012	2
AT.AT	16	12793460	12793471	6	.	.	intergenic	.	rs792270042	3
AG.CT	16	12821624	12821640	8.5	.	.	intergenic	0.143	.	4
AATG.CATT	16	12854739	12854758	5	.	.	intergenic	-0.244	rs792750542	2
AC.GT	16	12909375	12909390	8	.	.	intergenic	-0.237	.	2
AC.GT	16	12925377	12925395	9.5	.	.	intergenic	-0.148	350247 rs7872	4
AT.AT	16	12997461	12997476	8	.	.	intergenic	.	rs789800598	2
AAAT.ATTT	16	13106103	13106119	4.25	.	.	intergenic	-0.31	rs787916562	2
AAAAC.GTTTT	16	13180022	13180049	5.6	.	.	intergenic	.	rs788616181	2
AAT.ATT	16	13187780	13187794	5	.	.	intergenic	-0.142	rs787031963	2
AT.AT	16	13202075	13202089	7.5	.	.	intergenic	-0.056	356217 rs7880	3
AG.CT	16	13380232	13380243	6	.	.	intergenic	-0.139	rs789446738	3
AAAAG.CTTTT	16	13383293	13383314	4.4	.	.	intergenic	0.093	rs786574676	2
AAAC.GTTT	16	13555016	13555034	4.75	.	.	intergenic	-0.106	rs791875481	2
AT.AT	16	13584326	13584337	6	.	.	intergenic	0.046	rs793392703	2
AT.AT	16	13813393	13813402	5	.	.	intergenic	.	.	3
AGAT.ATCT	16	13861193	13861209	4.25	.	.	intergenic	-0.076	rs793343251	3
AAAC.GTTT	16	13872006	13872025	5	.	.	intergenic	-0.047	rs792305471	2
AAAC.GTTT	16	14056664	14056686	5.75	.	.	intergenic	.	018693 rs7931	2
AAAC.GTTT	16	14066128	14066142	3.75	.	.	intergenic	0.05	.	2
ATC.GAT	16	14174111	14174136	8.66667	.	.	intergenic	-0.12	441009 rs7878	2
AC.GT	16	14190426	14190440	7.5	.	.	intergenic	-0.185	rs793546604	2
AC.GT	16	14195327	14195356	15	.	.	intergenic	-0.015	.	2
ATC.GAT	16	14224935	14224954	6.66667	.	.	intergenic	0.04	rs787987974	3
AAT.ATT	16	14428706	14428723	6	.	.	intergenic	-0.061	.	2
AC.GT	16	14435043	14435052	5	.	.	intergenic	-0.001	292502 rs7039	2
AAAT.ATTT	16	14448490	14448501	3	.	.	intergenic	.	953455 rs7915	2
AAAG.CTTT	16	14473703	14473734	8	.	.	intergenic	0.108	rs786792168	3
AGAT.ATCT	16	14586825	14586844	5	.	.	intergenic	.	rs791694655	2
AAAC.GTTT	16	14621908	14621928	5.25	.	.	intergenic	0.125	.	2
AAAT.ATTT	16	14895181	14895202	5.5	ENSSSCT00000028848	CDH9	intron	-0.058	rs790854445	2
AAT.ATT	16	14923378	14923396	6.33333	ENSSSCT00000028848	CDH9	intron	-0.399	rs792653896	2
AT.AT	16	15050640	15050649	5	.	.	intergenic	.	.	2
AG.CT	16	15160863	15160873	5.5	.	.	intergenic	-0.277	.	2
AC.GT	16	15381544	15381559	8	.	.	intergenic	-0.01	rs788491442	2
AAAC.GTTT	16	15441632	15441672	10.25	.	.	intergenic	.	rs792839439	3
AAC.GTT	16	15585174	15585193	6.66667	.	.	intergenic	0.087	rs787664091	3
AG.CT	16	15815422	15815436	7.5	.	.	intergenic	0.01	rs788790178	2
AAT.ATT	16	15867892	15867908	5.66667	.	.	intergenic	.	.	3
AC.GT	16	15902892	15902925	17	.	.	intergenic	-0.005	rs788205687	2
AT.AT	16	16075211	16075224	7	.	.	intergenic	.	rs788220698	2
AT.AT	16	16163378	16163389	6	.	.	intergenic	0.176	rs786484447	3

AC.GT	16	16748535	16748545	5.5	.	.	intergenic	.	rs790562349	3
AT.AT	16	17456640	17456651	6	.	.	intergenic	-0.021	rs790894768	2
AAAT.ATTT	16	17678087	17678113	6.75	.	.	intergenic	0.596	508306 rs7929	2
AG.CT	16	17861771	17861798	14	.	.	intergenic	.	rs787061964	2
AAAC.GTTT	16	17878770	17878811	10.5	.	.	intergenic	.	rs792826900	2
AT.AT	16	18027597	18027606	5	.	.	intergenic	0.446	rs791059323	3
AT.AT	16	18068795	18068814	10	.	.	intergenic	0.117	.	3
AAAC.GTTT	16	18069066	18069079	3.5	.	.	intergenic	1.177	.	2
AATT.AATT	16	18144010	18144031	5.5	.	.	intergenic	0.442	279657 rs7863	2
AC.GT	16	18166492	18166503	6	.	.	intergenic	0.456	rs790325528	2
AC.GT	16	18267045	18267061	8.5	.	.	intergenic	-0.127	498395 rs7917	3
AAAC.GTTT	16	18465271	18465289	4.75	ENSSSCT00000018296	CDH6	intron	0.247	rs790093154	2
AT.AT	16	18502731	18502740	5	.	.	intergenic	0.244	rs789561482	2
AC.GT	16	18516696	18516705	5	.	.	intergenic	0.605	.	3
AAT.ATT	16	18517369	18517394	8.66667	.	.	intergenic	.	578406 rs7900	2
AAAACT.AGTTT	16	18603529	18603551	3.83333	ENSSSCT00000018300	DROSHA	intron	0.273	731492 rs7892	2
AAAC.GTTT	16	18612083	18612114	8	ENSSSCT00000018300	DROSHA	intron	-0.027	rs793370252	2
AAAAC.GTTTT	16	18626426	18626449	4.8	ENSSSCT00000018300	DROSHA	intron	0.448	965002 rs7865	2
AAAG.CTTT	16	18629793	18629807	3.75	ENSSSCT00000018300	DROSHA	intron	-0.366	rs691675883	2
AC.GT	16	18821598	18821617	10	.	.	intergenic	0.246	157614 rs7900	3
AAAAT.ATTTT	16	18838325	18838339	3	.	.	intergenic	0.468	rs787727109	2
AC.GT	16	18897410	18897433	12	ENSSSCT00000036642	PDZD2	promoter	-0.043	.	4
AGG.CCT	16	18950686	18950698	4.33333	ENSSSCT00000036642	PDZD2	intron	-0.039	579384 rs7928	2
AAAC.GTTT	16	19614629	19614652	6	.	.	intergenic	0.178	072374 rs7932	2
AG.CT	16	19636178	19636187	5	.	.	intergenic	-0.088	rs791181243	3
AAAAT.ATTTT	16	19660644	19660665	4.4	.	.	intergenic	-0.037	445122 rs7927	2
AG.CT	16	19686652	19686665	7	.	.	intergenic	.	rs707813158	3
AT.AT	16	19780837	19780854	9	.	.	intergenic	0.443	rs787883173	2
AC.GT	16	19859545	19859562	9	.	.	intergenic	.	rs789737210	2
AAAC.GTTT	16	19920601	19920619	4.75	.	.	intergenic	0.13	rs793464995	2
AC.GT	16	20014551	20014564	7	.	.	intergenic	0.135	550242 rs7912	2
AAAT.ATTT	16	20704677	20704691	3.75	.	.	intergenic	-0.016	.	2
AAAC.GTTT	16	20804414	20804434	5.25	ENSSSCT00000018316	C1QTNF3	intron	.	509609 rs7889	3
AAAC.GTTT	16	21084663	21084676	3.5	.	.	intergenic	0.155	rs788469333	2
AC.GT	16	21111919	21111939	10.5	ENSSSCT00000018317	RAI14	intron	0.091	.	3
AAAAC.GTTTT	16	21211220	21211234	3	ENSSSCT00000018317	RAI14	intron	0.328	rs789219534	2
AAGG.CCTT	16	21513251	21513264	3.5	ENSSSCT00000018324	AGXT2	intron	0.185	rs789166224	2
AAC.GTT	16	21877935	21877948	4.66667	.	.	intergenic	.	528267 rs7872	2
AAAAAC.GTTTT	16	21985263	21985283	3.5	.	.	intergenic	0.018	rs793584891	2
ATC.GAT	16	22237873	22237894	7.33333	ENSSSCT00000018326	SPEF2	intron	0.969	rs790589017	2
AAAC.GTTT	16	23150409	23150423	3.75	.	.	intergenic	-0.164	rs793428185	2
AAAT.ATTT	16	23225572	23225591	5	.	.	intergenic	.	rs792799745	2
AT.AT	16	23579597	23579606	5	ENSSSCT00000018340	C5orf42	intron	-0.001	141177 rs7869	2
AAC.GTT	16	23871211	23871227	5.66667	ENSSSCT00000018344	WDR70	intron	.	rs790453810	2
ACAT.ATGT	16	23908694	23908709	4	ENSSSCT00000018344	WDR70	intron	.	.	2
AT.AT	16	23942337	23942352	8	ENSSSCT00000018344	WDR70	intron	1.291	.	2
AGGG.CCCT	16	23976009	23976025	4.25	ENSSSCT00000018344	WDR70	intron	0.054	192236 rs7906	2
AAAC.GTTT	16	24317674	24317692	4.75	.	.	intergenic	0.124	rs792130954	2
AAAAC.GTTTT	16	24331156	24331174	3.8	.	.	intergenic	.	709307 rs7872	2
AG.CT	16	24495957	24495966	5	.	.	intergenic	0.22	311272 rs7904	2
AT.AT	16	24496412	24496424	6.5	.	.	intergenic	.	rs788035572	2
AAATT.AATTT	16	24500647	24500663	3.4	.	.	intergenic	0.048	rs788695615	2
AAT.ATT	16	24529872	24529893	7.33333	.	.	intergenic	0.211	333528 rs7908	2
AC.GT	16	24766881	24766892	6	ENSSSCT00000018347	EGFLAM	intron	.	.	2
AC.GT	16	24766881	24766892	6	ENSSSCT00000025413	EGFLAM	intron	.	.	2
AG.CT	16	24820375	24820384	5	ENSSSCT00000018347	EGFLAM	intron	0.154	rs789135926	2
AG.CT	16	24820375	24820384	5	ENSSSCT00000025413	EGFLAM	intron	0.154	rs789135926	2
AAAC.GTTT	16	24995854	24995868	3.75	ENSSSCT00000018350	LIFR	intron	0.181	rs792126472	2
AAAC.GTTT	16	24995854	24995868	3.75	ENSSSCT00000032662	LIFR	intron	0.181	rs792126472	2
AG.CT	16	25025063	25025079	8.5	.	.	intergenic	-0.007	.	2
AC.GT	16	25053067	25053077	5.5	.	.	intergenic	0.155	rs786916581	2
AAGG.CCTT	16	25120983	25121024	10.5	.	.	intergenic	.	rs789541391	4
AAAAC.GTTTT	16	25152658	25152673	3.2	.	.	intergenic	0.5	rs793590188	2
AAAAAG.CTTTT	16	25213314	25213331	3	.	.	intergenic	0.174	rs789875026	2
AAAG.CTTT	16	25235646	25235660	3.75	.	.	intergenic	.	rs789565836	2
AAAAT.ATTTT	16	25536710	25536727	3.6	ENSSSCT00000035208	FYB	intron	0.109	rs787475423	2
AAAAT.ATTTT	16	25536710	25536727	3.6	ENSSSCT00000034777	FYB	intron	0.109	rs787475423	2
AAAAT.ATTTT	16	25536710	25536727	3.6	ENSSSCT00000018355	FYB	intron	0.109	rs787475423	2
ACC.GGT	16	25574391	25574404	4.66667	ENSSSCT00000035208	FYB	intron	0.046	rs789457418	2
ACC.GGT	16	25574391	25574404	4.66667	ENSSSCT00000034777	FYB	intron	0.046	rs789457418	2
ACC.GGT	16	25574391	25574404	4.66667	ENSSSCT00000018355	FYB	intron	0.046	rs789457418	2
AAAAAC.GTTTT	16	25715388	25715420	5.5	.	.	intergenic	-0.113	rs793488902	2
AAAT.ATTT	16	25806647	25806660	3.5	ENSSSCT00000018358	DAB2	intron	-0.008	rs787833475	2
AAAT.ATTT	16	25806647	25806660	3.5	ENSSSCT00000018356	C9	intron	-0.008	rs787833475	2
AAT.ATT	16	26238611	26238624	4.66667	.	.	intergenic	.	rs790022787	2
AT.AT	16	26252325	26252336	6	.	.	intergenic	0.106	rs790060954	3
AG.CT	16	26352599	26352615	8.5	.	.	intergenic	-0.034	rs791441226	2
AAC.GTT	16	26367838	26367854	5.66667	.	.	intergenic	-0.136	rs793205168	2
AG.CT	16	26443759	26443771	6.5	.	.	intergenic	.	rs789532540	4
AC.GT	16	26445577	26445599	11.5	.	.	intergenic	.	rs788774087	3
AT.AT	16	26445988	26446005	9	.	.	intergenic	.	rs793053962	2
AAAGAG.CTCTT	16	26678259	26678302	7.33333	.	.	intergenic	.	335676 rs7926	2
AAAC.GTTT	16	26788633	26788668	9	.	.	intergenic	0.283	rs790804961	2
AC.GT	16	26849811	26849822	6	.	.	intergenic	0.231	.	5

AAAT.ATTT	16	26859213	26859232	5	ENSSSCT00000030818	.	intron	.	rs791637780	2
AAAT.ATTT	16	26888977	26889002	6.5	.	.	intergenic	.	337112 rs7876	2
AG.CT	16	27033399	27033408	5	ENSSSCT00000023181	MROH2B	intron	0.021	rs790070622	2
AT.AT	16	27035767	27035778	6	ENSSSCT00000023181	MROH2B	intron	-0.229	.	3
AAAC.GTTT	16	27138131	27138153	5.75	ENSSSCT00000018362	.	intron	-0.196	798224 rs7884	2
AAAC.GTTT	16	27228777	27228797	5.25	ENSSSCT00000018364	C6	intron	0.029	rs788644770	2
AT.AT	16	27230782	27230792	5.5	ENSSSCT00000018364	C6	intron	-0.097	rs791565635	2
AC.GT	16	27250252	27250264	6.5	ENSSSCT00000018364	C6	intron	-0.022	.	4
AAAAT.ATTTT	16	27558048	27558067	4	.	.	intergenic	.	rs706610362	2
AG.CT	16	28242644	28242654	5.5	.	.	intergenic	.	.	3
AAAC.GTTT	16	28338782	28338799	4.5	.	.	intergenic	0.022	560701 rs7896	3
AC.GT	16	28548458	28548470	6.5	.	.	intergenic	-0.147	.	2
AAAT.ATTT	16	28571813	28571829	4.25	.	.	intergenic	.	rs793029235	2
AAT.ATT	16	28759127	28759153	9	ENSSSCT00000018369	GHR	intron	0.079	.	2
AG.CT	16	29099330	29099344	7.5	.	.	intergenic	0.134	rs793633251	3
AG.CT	16	29333050	29333059	5	ENSSSCT00000018373	.	promoter	0.081	rs791278182	3
AAAC.GTTT	16	29341318	29341336	4.75	.	.	intergenic	-0.368	rs788706179	2
AAAT.ATTT	16	29451833	29451852	5	ENSSSCT00000018376	HMGCS1	3'utr	1.139	702505 rs7935	2
AAAAC.GTTTT	16	29504440	29504479	8	ENSSSCT00000018377	NIM1K	intron	0.177	323677 rs7102	2
AT.AT	16	29600337	29600347	5.5	ENSSSCT00000018378	PAIP1	intron	1.272	312238 rs7936	2
AC.GT	16	30210703	30210713	5.5	.	.	intergenic	0.262	.	3
AAAC.GTTT	16	30228517	30228529	3.25	.	.	intergenic	0.034	775002 rs7862	2
AC.GT	16	30311679	30311689	5.5	ENSSSCT00000018382	FGF10	intron	0.789	.	3
AAAC.GTTT	16	30435135	30435149	3.75	.	.	intergenic	0.106	rs790430303	2
AAAC.GTTT	16	30437276	30437301	6.5	.	.	intergenic	0.198	.	3
AT.AT	16	30806863	30806873	5.5	.	.	intergenic	.	rs792731234	2
AAAT.ATTT	16	30918190	30918216	6.75	.	.	intergenic	0.023	058818 rs7887	2
AAT.ATT	16	30945206	30945226	7	.	.	intergenic	.	777361 rs7907	2
AC.GT	16	30964932	30964954	11.5	ENSSSCT00000028722	HCN1	intron	0.167	.	2
AC.GT	16	30975055	30975078	12	ENSSSCT00000028722	HCN1	intron	0.316	rs792855461	3
AAC.GTT	16	31083739	31083756	6	ENSSSCT00000028722	HCN1	intron	-0.191	.	2
AG.CT	16	31105392	31105403	6	ENSSSCT00000028722	HCN1	intron	0.068	.	3
AT.AT	16	31129438	31129452	7.5	ENSSSCT00000028722	HCN1	intron	0.124	769755 rs7915	2
AAAT.ATTT	16	31228083	31228106	6	ENSSSCT00000028722	HCN1	intron	0.139	rs788651400	2
AAAC.GTTT	16	31354939	31354950	3	.	.	intergenic	.	rs786327758	2
AG.CT	16	31365002	31365015	7	.	.	intergenic	-0.12	rs791691776	2
AG.CT	16	31389903	31389914	6	.	.	intergenic	0.642	.	2
AACC.GGTT	16	31597888	31597913	6.5	.	.	intergenic	.	.	2
AAAC.GTTT	16	31651389	31651408	5	.	.	intergenic	-0.375	434160 rs7890	2
AG.CT	16	32088067	32088083	8.5	.	.	intergenic	-0.084	.	2
AC.GT	16	32098297	32098308	6	.	.	intergenic	0.111	rs788111302	2
AG.CT	16	32140209	32140223	7.5	.	.	intergenic	-0.137	rs792735470	2
AAAC.GTTT	16	32157384	32157403	5	.	.	intergenic	0.063	227689 rs7933	2
AGAT.ATCT	16	32158737	32158764	7	.	.	intergenic	-0.08	rs788724500	2
AT.AT	16	32421481	32421499	9.5	.	.	intergenic	.	757964 rs7908	2
AT.AT	16	32432158	32432169	6	.	.	intergenic	.	rs695560861	3
AT.AT	16	32436354	32436365	6	.	.	intergenic	-0.039	.	3
AAAG.CTTT	16	32439502	32439516	3.75	.	.	intergenic	0.124	632079 rs7896	2
AC.GT	16	32542631	32542651	10.5	.	.	intergenic	0.151	rs792394207	3
AC.GT	16	32632649	32632658	5	.	.	intergenic	0.312	.	3
AC.GT	16	32652228	32652239	6	.	.	intergenic	-0.04	.	2
AC.GT	16	32678565	32678577	6.5	.	.	intergenic	-0.29	rs792770393	2
AG.CT	16	32705012	32705025	7	.	.	intergenic	-0.025	352236 rs7893	3
AAAC.GTTT	16	32763489	32763515	6.75	.	.	intergenic	.	260079 rs7906	2
AAAAG.CTTTT	16	32996006	32996020	3	.	.	intergenic	-0.017	rs786854793	2
AAAAC.GTTTT	16	33207893	33207909	3.4	.	.	intergenic	-0.094	rs786905410	2
ATC.GAT	16	33307300	33307331	10.6667	.	.	intergenic	-0.29	rs710280074	3
AAAAG.CTTTT	16	33322649	33322675	5.4	.	.	intergenic	-0.042	151070 rs7901	2
AC.GT	16	33411651	33411665	7.5	.	.	intergenic	0.083	rs693440515	4
AAAT.ATTT	16	33661089	33661102	3.5	.	.	intergenic	.	rs790725255	2
AT.AT	16	33662799	33662809	5.5	.	.	intergenic	-0.139	rs788129476	2
AC.GT	16	33768908	33768929	11	.	.	intergenic	0.02	rs789878802	3
AC.GT	16	33779370	33779384	7.5	.	.	intergenic	.	.	2
AGAT.ATCT	16	33903670	33903686	4.25	.	.	intergenic	0.227	rs787961021	2
AAGG.CCTT	16	34023827	34023852	6.5	ENSSSCT00000018389	ITGA1	intron	0.183	530618 rs7905	2
AAGG.CCTT	16	34023827	34023852	6.5	ENSSSCT00000034458	ITGA1	intron	0.183	530618 rs7905	2
ACT.AGT	16	34112120	34112140	7	ENSSSCT00000018389	ITGA1	intron	0.243	rs792793965	2
ACT.AGT	16	34112120	34112140	7	ENSSSCT00000033593	ITGA1	intron	0.243	rs792793965	2
AAC.GTT	16	34152255	34152266	4	.	.	intergenic	.	549487 rs7911	2
AC.GT	16	34197222	34197235	7	ENSSSCT00000032891	ITGA2	intron	0.143	.	3
AC.GT	16	34197222	34197235	7	ENSSSCT00000035637	ITGA2	intron	0.143	.	3
AC.GT	16	34197222	34197235	7	ENSSSCT00000034283	ITGA2	intron	0.143	.	3
AC.GT	16	34197222	34197235	7	ENSSSCT00000018391	ITGA2	intron	0.143	.	3
AC.GT	16	34197222	34197235	7	ENSSSCT00000032880	ITGA2	intron	0.143	.	3
AT.AT	16	34269848	34269860	6.5	ENSSSCT00000018392	MOCS2	3'utr	.	rs791924036	3
AAAAC.GTTTT	16	34476738	34476760	4.6	.	.	intergenic	-0.059	rs790230002	2
AG.CT	16	34493730	34493740	5.5	.	.	intergenic	.	.	2
AT.AT	16	34675497	34675521	12.5	.	.	intergenic	.	rs786429988	2
AC.GT	16	34878262	34878283	11	ENSSSCT00000022508	.	intron	.	rs791833184	2
AAAT.ATTT	16	34907246	34907273	7	.	.	intergenic	0.271	559899 rs7901	2
AAAT.ATTT	16	34931032	34931046	3.75	.	.	intergenic	.	.	2
AAAC.GTTT	16	35016657	35016679	5.75	.	.	intergenic	.	rs786642917	2
AAAT.ATTT	16	35022842	35022861	5	.	.	intergenic	.	rs789688739	2
AC.GT	16	35315651	35315663	6.5	ENSSSCT00000018399	ARL15	intron	-0.079	.	3

AAG.CTT	16	35382833	35382844	4	ENSSSCT00000018399	ARL15	intron	.	rs789556478	2
AG.CT	16	35915730	35915750	10.5	.	.	intergenic	0.02	rs791749427	2
AAAC.GTTT	16	36123484	36123502	4.75	.	.	intergenic	0	rs789688246	2
AAAT.ATTT	16	36206763	36206785	5.75	.	.	intergenic	.	rs789736979	2
AAAC.GTTT	16	36270203	36270239	9.25	.	.	intergenic	0.052	rs793420355	2
AGG.CCT	16	36316766	36316798	11	.	.	intergenic	-0.024	370940 rs7923	2
AGGC.GCCT	16	36318579	36318596	4.5	.	.	intergenic	0.602	rs787936554	2
AT.AT	16	36726073	36726084	6	ENSSSCT00000018418	PPAP2A	intron	0.091	098878 rs7897	2
AAAAC.GTTTT	16	37197569	37197594	4.33333	.	.	intergenic	0.108	745519 rs7927	2
AC.GT	16	37432742	37432756	7.5	.	.	intergenic	0.197	rs793226919	2
AAAC.GTTT	16	37563651	37563674	6	ENSSSCT00000018421	IL31RA	intron	-0.119	722277 rs7889	2
AAAC.GTTT	16	37607016	37607034	4.75	ENSSSCT00000018421	IL31RA	intron	-0.016	rs787361628	2
AAAT.ATTT	16	37681305	37681320	4	.	.	intergenic	.	340741 rs7914	2
AAAAC.GTTTT	16	37681581	37681601	4.2	.	.	intergenic	.	.	2
AAAC.GTTT	16	37789562	37789577	4	ENSSSCT00000020475	U6	promoter	.	749275 rs7900	2
AGG.CCT	16	37911927	37911943	5.66667	.	.	intergenic	.	rs789178893	2
AAAC.GTTT	16	38007221	38007234	3.5	.	.	intergenic	0.103	rs786312620	2
AAAC.GTTT	16	38047317	38047331	3.75	.	.	intergenic	.	rs697025129	2
AAC.GTT	16	38181710	38181726	5.66667	ENSSSCT00000018424	MAP3K1	intron	0.222	.	3
AAC.GTT	16	38185206	38185229	8	ENSSSCT00000018424	MAP3K1	intron	1.659	795942 rs7933	2
AAAAC.GTTTT	16	38230417	38230440	4.8	.	.	intergenic	.	rs787308020	2
AC.GT	16	38285858	38285873	8	ENSSSCT00000018427	MIER3	intron	0.417	554626 rs7871	3
AACT.AGTT	16	38403591	38403608	4.5	.	.	intergenic	-0.157	rs788635949	2
AC.GT	16	38445891	38445901	5.5	.	.	intergenic	.	rs793376879	2
AG.CT	16	38463684	38463693	5	.	.	intergenic	0.134	rs788830227	2
AAT.ATT	16	38755356	38755378	7.66667	.	.	intergenic	0.187	349547 rs7901	2
AG.CT	16	38975037	38975046	5	.	.	intergenic	.	rs788710500	2
AAC.GTT	16	38999929	38999944	5.33333	.	.	intergenic	.	.	2
AG.CT	16	39017580	39017592	6.5	.	.	intergenic	0.157	.	2
AAATT.AATTT	16	39201745	39201763	3.8	.	.	intergenic	.	516544 rs7937	2
AAAAC.GTTTT	16	39761818	39761837	4	.	.	intergenic	0.17	rs793352532	2
AC.GT	16	39811567	39811579	6.5	.	.	intergenic	0.023	906967 rs7934	2
AT.AT	16	40010242	40010251	5	.	.	intergenic	0.089	rs789778091	2
AAAT.ATTT	16	40094741	40094763	5.75	.	.	intergenic	.	rs789102943	2
ACC.GGT	16	40171625	40171645	7	ENSSSCT00000018434	.	promoter	.	014301 rs7904	2
AC.GT	16	40318757	40318769	6.5	ENSSSCT00000018435	RAB3C	intron	0.066	rs790452408	2
AGAT.ATCT	16	40402632	40402651	5	ENSSSCT00000018435	RAB3C	intron	-0.048	925220 rs7908	2
AAAC.GTTT	16	40456442	40456464	5.75	ENSSSCT00000018435	RAB3C	intron	.	546163 rs7868	3
AC.GT	16	40596832	40596842	5.5	.	.	intergenic	0.087	rs788893488	2
AG.CT	16	40639099	40639133	17.5	ENSSSCT00000018436	PDE4D	3'utr	1.597	rs789767444	2
AT.AT	16	40956133	40956142	5	ENSSSCT00000029920	.	intron	.	rs791369265	2
AAAC.GTTT	16	40984210	40984232	5.75	.	.	intergenic	0.002	rs787186622	2
ACAT.ATGT	16	40992875	40992893	4.75	.	.	intergenic	-0.084	.	5
AAAAC.GTTTT	16	41023670	41023698	5.8	.	.	intergenic	.	255236 rs7933	2
AC.GT	16	41084018	41084028	5.5	.	.	intergenic	-0.127	rs789335980	2
ACAT.ATGT	16	41984597	41984615	4.75	.	.	intergenic	.	.	3
AAAT.ATTT	16	42119389	42119412	6	.	.	intergenic	.	223252 rs7902	2
AAAC.GTTT	16	42120343	42120365	5.75	.	.	intergenic	.	rs793146268	3
AAAT.ATTT	16	42223443	42223456	3.5	.	.	intergenic	0.018	.	2
AAAT.ATTT	16	42261897	42261914	4.5	.	.	intergenic	-0.111	rs787201452	2
AAAC.GTTT	16	42480627	42480646	5	.	.	intergenic	0.092	261775 rs7867	2
AAC.GTT	16	42538958	42538974	5.66667	ENSSSCT00000018441	ELOVL7	intron	0.241	264539 rs7910	2
AAC.GTT	16	42538958	42538974	5.66667	ENSSSCT00000018440	ELOVL7	intron	0.241	264539 rs7910	2
AAAG.CTTT	16	42618079	42618113	8.75	ENSSSCT00000018442	ERCC8	intron	.	.	2
AAAAT.ATTTT	16	42871390	42871417	5.6	ENSSSCT00000025662	NDUFAF2	intron	.	rs788783623	2
AT.AT	16	43104787	43104797	5.5	.	.	intergenic	0.384	.	3
AC.GT	16	43317840	43317852	6.5	.	.	intergenic	.	rs789457391	2
AC.GT	16	43356573	43356588	8	.	.	intergenic	-0.033	rs792409759	3
AC.GT	16	43384484	43384494	5.5	.	.	intergenic	.	rs787369148	2
AAC.GTT	16	43480494	43480518	8.33333	.	.	intergenic	0.166	rs789928851	2
AT.AT	16	43506965	43506992	14	.	.	intergenic	0.044	.	2
AGC.GCT	16	43508680	43508693	4.66667	.	.	intergenic	0.014	rs791293949	2
AT.AT	16	43513619	43513640	11	.	.	intergenic	0.449	rs786538720	4
AAAC.GTTT	16	43754923	43754945	5.75	.	.	intergenic	0.182	rs790084382	2
AAAT.ATTT	16	43785052	43785074	5.75	.	.	intergenic	-0.045	.	2
AAAT.ATTT	16	43824890	43824911	5.5	.	.	intergenic	0.13	rs793326782	2
AC.GT	16	44018765	44018774	5	.	.	intergenic	0.171	rs788669519	2
AAAG.CTTT	16	44406519	44406537	4.75	.	.	intergenic	0.458	969099 rs7879	2
AAAC.GTTT	16	44556840	44556863	6	.	.	intergenic	0.074	rs792603949	2
AAAAC.GTTTT	16	45142716	45142738	4.6	.	.	intergenic	0.081	.	2
AC.GT	16	45359435	45359449	7.5	.	.	intergenic	0.069	rs791318897	2
AG.CT	16	45727702	45727714	6.5	.	.	intergenic	-0.01	rs702123902	3
AAC.GTT	16	45737366	45737391	8.66667	.	.	intergenic	.	rs786306000	2
AAAAC.GTTTT	16	45802341	45802364	4	ENSSSCT00000018449	RNF180	intron	.	rs788086072	2
AAAC.GTTT	16	46037801	46037833	8.25	.	.	intergenic	.	rs787148390	2
AG.CT	16	46166740	46166751	6	.	.	intergenic	.	rs793234682	2
AT.AT	16	46326495	46326509	7.5	.	.	intergenic	0.168	.	4
AG.CT	16	46390113	46390131	9.5	.	.	intergenic	.	rs792981997	2
AAAC.GTTT	16	46403295	46403320	6.5	.	.	intergenic	.	rs789658714	2
AAAT.ATTT	16	46592525	46592540	4	ENSSSCT00000018450	CWC27	intron	1.867	.	2
AAAG.CTTT	16	46746600	46746619	5	ENSSSCT00000018450	CWC27	intron	.	rs786832153	2
AAAAG.CTTTT	16	46813099	46813115	3.4	ENSSSCT00000018450	CWC27	intron	-0.474	rs790408024	2
AG.CT	16	46929006	46929021	8	.	.	intergenic	0.116	.	2
AC.GT	16	47153045	47153062	9	ENSSSCT00000018451	ADAMTS6	intron	0.091	rs791325048	2

AC.GT	16	47841009	47841021	6.5	ENSSSCT00000018461	.	intron	0.217	rs787174159	2
AC.GT	16	48013672	48013684	6.5	.	.	intergenic	.	rs790226252	9
AT.AT	16	48117708	48117721	7	.	.	intergenic	0.154	374409 rs7882	4
AAG.CTT	16	48414825	48414845	7	.	.	intergenic	0.148	rs792489384	2
AC.GT	16	49015565	49015577	6.5	.	.	intergenic	0.021	rs788837988	2
AG.CT	16	49191912	49191921	5	.	.	intergenic	-0.036	rs788487689	4
AT.AT	16	49480419	49480431	6.5	.	.	intergenic	0.285	315188 rs7878	2
ACAT.ATGT	16	49544476	49544500	6.25	.	.	intergenic	-0.009	910779 rs7866	2
AC.GT	16	49795505	49795517	6.5	.	.	intergenic	0.054	rs789722624	2
AAT.ATT	16	49821209	49821234	8.66667	.	.	intergenic	0.104	rs791275763	2
ATC.GAT	16	49878566	49878593	9.33333	.	.	intergenic	-0.164	rs788104531	2
AAAAT.ATTTT	16	50380838	50380862	5	ENSSSCT00000035672	PIK3R1	promoter	.	rs791402474	2
AAAAT.ATTTT	16	50380838	50380862	5	ENSSSCT00000036318	PIK3R1	promoter	.	rs791402474	2
AAAAT.ATTTT	16	50380838	50380862	5	ENSSSCT00000033449	PIK3R1	promoter	.	rs791402474	2
AAAAT.ATTTT	16	50380838	50380862	5	ENSSSCT00000035520	PIK3R1	promoter	.	rs791402474	2
ACAGC.GCTGT	16	50671100	50671114	3	.	.	intergenic	0.118	rs787574372	2
AAC.GTT	16	50750983	50751005	7.66667	.	.	intergenic	.	rs787778303	2
AC.GT	16	50799990	50800002	6.5	.	.	intergenic	0.318	rs792910277	2
AG.CT	16	50851774	50851786	6.5	.	.	intergenic	-0.116	.	2
AAAAAC.GTTTTT	16	50940917	50940941	4.16667	.	.	intergenic	0.425	rs694182392	2
AC.GT	16	51246153	51246169	8.5	ENSSSCT00000026613	CENPH	promoter	.	rs786427825	2
AT.AT	16	51395383	51395394	6	ENSSSCT00000018473	.	intron	0.153	rs793654442	2
AT.AT	16	51395383	51395394	6	ENSSSCT00000030315	.	intron	0.153	rs793654442	2
AAAC.GTTT	16	51541514	51541536	5.75	.	.	intergenic	-0.07	.	2
AC.GT	16	51875887	51875899	6.5	ENSSSCT00000018480	.	intron	0.061	.	2
AC.GT	16	51875887	51875899	6.5	ENSSSCT00000026987	.	intron	0.061	.	2
AAAAG.CTTTT	16	52178136	52178153	3.6	.	.	intergenic	.	rs786947480	2
AAAG.CTTT	16	52209258	52209269	3	.	.	intergenic	0.247	rs786627297	2
AATG.CATT	16	52260135	52260161	6.75	.	.	intergenic	-0.251	rs706461025	2
AG.CT	16	52605516	52605527	6	ENSSSCT00000018485	PTCD2	intron	-0.024	.	3
AC.GT	16	52674375	52674393	9.5	.	.	intergenic	-0.043	rs792495326	2
AAC.GTT	16	52852828	52852841	4.66667	.	.	intergenic	0.087	rs789066109	2
AATG.CATT	16	52853914	52853928	3.75	.	.	intergenic	-0.031	.	2
AC.GT	16	52899421	52899432	6	.	.	intergenic	0.769	.	2
AAAT.ATTT	16	52974307	52974332	6.5	.	.	intergenic	0.269	rs789581493	2
AAAAC.GTTTT	16	53285673	53285691	3.8	ENSSSCT00000026046	TNPO1	intron	0.023	rs787348048	2
AG.CT	16	53548780	53548790	5.5	.	.	intergenic	-0.091	rs787397626	2
AACT.AGTT	16	53694555	53694576	5.5	.	.	intergenic	0.193	rs789959912	2
AC.GT	16	54102448	54102458	5.5	.	.	intergenic	.	rs789740992	2
AAAAC.GTTTT	16	54713474	54713503	6	.	.	intergenic	.	rs787237117	2
AAAAT.ATTTT	16	54795857	54795878	4.4	.	.	intergenic	.	rs791038902	2
AG.CT	16	54906245	54906270	13	.	.	intergenic	0.284	rs791926899	2
AAAAC.GTTTT	16	55249413	55249431	3.8	.	.	intergenic	.	rs792896158	2
AAAAT.ATTTT	16	55382996	55383014	3.8	.	.	intergenic	-0.145	rs790463738	2
AAAAC.GTTTT	16	55721037	55721059	4.6	.	.	intergenic	-0.044	326015 rs7909	2
AAT.ATT	16	56114033	56114049	5.66667	.	.	intergenic	-0.153	746318 rs7897	2
AATG.CATT	16	56121572	56121600	7.25	.	.	intergenic	-0.264	rs787813209	2
AT.AT	16	56284299	56284309	5.5	.	.	intergenic	0.222	rs791970621	2
ACC.GGT	16	56423728	56423750	7.66667	.	.	intergenic	0.111	rs787628462	2
AAAT.ATTT	16	56427778	56427808	7.75	ENSSSCT00000018507	.	intron	0.801	762764 rs7889	2
AAAATT.AATTTT	16	56591943	56591965	3.83333	ENSSSCT00000032458	FBXW11	intron	.	rs787021002	2
AC.GT	16	57163973	57163988	8	ENSSSCT00000018512	RANBP17	intron	.	.	4
AAAG.CTTT	16	57166645	57166669	6.25	ENSSSCT00000018512	RANBP17	intron	.	rs790413113	2
AAAAT.ATTTT	16	57543718	57543751	6.8	.	.	intergenic	0.105	346114 rs7029	3
AC.GT	16	57847677	57847695	9.5	.	.	intergenic	-0.044	rs789915821	2
AG.CT	16	57861391	57861418	14	.	.	intergenic	-0.125	788994 rs7905	2
AG.CT	16	57953192	57953214	11.5	.	.	intergenic	-0.242	rs790887361	2
AG.CT	16	58030462	58030481	10	.	.	intergenic	-0.075	rs789804324	2
AAAG.CTTT	16	58031659	58031678	5	.	.	intergenic	.	.	3
AAT.ATT	16	58450072	58450086	5	.	.	intergenic	0.082	rs790212612	3
AG.CT	16	58581663	58581693	15.5	.	.	intergenic	0.618	rs792354367	2
AG.CT	16	58972300	58972316	8.5	.	.	intergenic	0.036	rs791724581	3
AAAAG.CTTTT	16	59203121	59203149	5.8	.	.	intergenic	0.986	.	2
AC.GT	16	59426188	59426200	6.5	.	.	intergenic	-0.049	.	2
AG.CT	16	59525810	59525824	7.5	.	.	intergenic	-0.168	.	4
AGGGG.CCCCT	16	59704195	59704210	3.2	ENSSSCT00000018524	SLIT3	intron	0.051	rs792112862	2
AC.GT	16	59898680	59898692	6.5	.	.	intergenic	0.143	.	4
AAAAT.ATTTT	16	59926308	59926334	5.4	.	.	intergenic	0.141	193959 rs7906	2
ACACT.AGTGT	16	60105986	60106005	4	.	.	intergenic	-0.308	rs793259632	2
AAAC.GTTT	16	60109981	60109992	3	.	.	intergenic	-0.129	rs792479999	2
AAAT.ATTT	16	60120304	60120324	5.25	.	.	intergenic	-0.146	rs786478018	2
AG.CT	16	60574555	60574570	8	.	.	intergenic	0.047	975271 rs7883	2
AAAAAC.GTTTTT	16	60596692	60596715	4	.	.	intergenic	0.015	rs793037577	2
AG.CT	16	60633891	60633905	7.5	.	.	intergenic	0.029	rs791566543	2
AAAT.ATTT	16	60739738	60739753	4	.	.	intergenic	0.005	991546 rs7874	2
AAAAT.ATTTT	16	60758045	60758071	5.4	.	.	intergenic	0.116	rs789512268	2
AAAG.CTTT	16	60930130	60930148	4.75	.	.	intergenic	0.023	rs789687953	2
AG.CT	16	60975992	60976010	9.5	.	.	intergenic	0.35	.	2
AC.GT	16	61000035	61000049	7.5	.	.	intergenic	0.986	rs787090999	3
AC.GT	16	61029740	61029752	6.5	.	.	intergenic	-0.412	.	2
AAAAC.GTTTT	16	61074904	61074927	4.8	.	.	intergenic	1.001	rs787462637	2
AACT.AGTT	16	61112473	61112486	3.5	.	.	intergenic	0.315	rs787728073	2
AAAC.GTTT	16	61134109	61134127	4.75	.	.	intergenic	0.717	rs790045943	3
AG.CT	16	61242758	61242774	8.5	.	.	intergenic	0.353	.	3

AAAAG.CTTTT	16	61566075	61566093	3.8	.	.	intergenic	-0.032	rs700662049	4
AAAAC.GTTTT	16	61904208	61904225	3.6	.	.	intergenic	-0.185	.	2
AAC.GTT	16	61910716	61910729	4.66667	.	.	intergenic	0.694	532251 rs7919	2
AC.GT	16	61973932	61973946	7.5	.	.	intergenic	-0.159	392085 rs7888	3
AAAAC.GTTTT	16	62183786	62183803	3.6	.	.	intergenic	.	184795 rs7870	2
AG.CT	16	62681226	62681241	8	.	.	intergenic	0.37	.	2
AG.CT	16	62686327	62686337	5.5	.	.	intergenic	0.455	rs787288146	2
ACAG.CTGT	16	62813120	62813135	4	.	.	intergenic	0.188	.	2
AC.GT	16	62906703	62906721	9.5	.	.	intergenic	0.081	rs786812457	3
AG.CT	16	62915138	62915147	5	.	.	intergenic	0.086	.	2
AAAT.ATTT	16	63225016	63225052	9.25	.	.	intergenic	-0.169	.	2
AAAT.ATTT	16	63273181	63273200	5	.	.	intergenic	.	rs697865892	2
AAAT.ATTT	16	63292920	63292949	7.5	.	.	intergenic	-0.049	306930 rs7936	2
AC.GT	16	63313183	63313194	6	.	.	intergenic	-0.205	.	3
AAAT.ATTT	16	63314007	63314032	6.5	.	.	intergenic	-0.241	rs791812747	2
AAAAC.GTTTT	16	63357809	63357829	4.2	.	.	intergenic	.	.	2
AAAC.GTTT	16	63585451	63585465	3.75	.	.	intergenic	.	395138 rs7881	2
AAAT.ATTT	16	63605796	63605818	5.75	.	.	intergenic	-0.066	145204 rs7881	2
AAAC.GTTT	16	63660557	63660582	6.5	.	.	intergenic	-0.229	568200 rs7870	2
AAAC.GTTT	16	63805391	63805414	6	.	.	intergenic	-0.092	rs790472393	2
AC.GT	16	63824218	63824240	11.5	.	.	intergenic	0.259	044640 rs7889	4
AAC.GTT	16	64150302	64150319	6	.	.	intergenic	0.122	768292 rs7885	3
AG.CT	16	64386764	64386774	5.5	.	.	intergenic	0.586	204157 rs7924	2
AAC.GTT	16	64407250	64407262	4.33333	.	.	intergenic	.	rs786211821	2
AG.CT	16	64458972	64458981	5	.	.	intergenic	0.018	.	2
AAC.GTT	16	64520285	64520301	5.66667	.	.	intergenic	0.15	rs791495722	2
AAAAC.GTTTT	16	64694487	64694512	5.2	.	.	intergenic	0.269	rs789424372	2
AAAGT.ACTTT	16	64752734	64752748	3	.	.	intergenic	0.117	.	2
AG.CT	16	64778069	64778078	5	.	.	intergenic	-0.095	rs787059129	2
AC.GT	16	64822010	64822020	5.5	.	.	intergenic	0.151	rs791118681	2
AG.CT	16	64992500	64992513	7	.	.	intergenic	0.922	.	3
AC.GT	16	64993805	64993817	6.5	.	.	intergenic	0.234	436015 rs7894	2
AT.AT	16	65053959	65053970	6	.	.	intergenic	0.152	.	2
AAAC.GTTT	16	65145044	65145058	3.75	ENSSSCT00000034673	HMMR	intron	.	rs789677182	2
AAAC.GTTT	16	65145044	65145058	3.75	ENSSSCT00000018534	HMMR	intron	.	rs789677182	2
AAAC.GTTT	16	65145044	65145058	3.75	ENSSSCT00000036191	HMMR	intron	.	rs789677182	2
AAAC.GTTT	16	65145044	65145058	3.75	ENSSSCT00000030611	HMMR	intron	.	rs789677182	2
AAAAC.GTTTT	16	65263601	65263623	4.6	.	.	intergenic	0.127	249353 rs7906	2
AAAC.GTTT	16	65451465	65451483	4.75	.	.	intergenic	0.181	343338 rs7909	2
AC.GT	16	65639527	65639537	5.5	.	.	intergenic	0.047	969737 rs7919	3
AT.AT	16	65956972	65956982	5.5	.	.	intergenic	.	rs793851362	2
AT.AT	16	66020579	66020588	5	.	.	intergenic	-0.12	.	2
AC.GT	16	66083607	66083618	6	.	.	intergenic	0.043	rs703968674	4
AATG.CATT	16	66109737	66109755	4.75	.	.	intergenic	-0.128	771447 rs7872	2
AC.GT	16	66237578	66237593	8	.	.	intergenic	.	rs787202654	2
AT.AT	16	66429147	66429157	5.5	.	.	intergenic	0.163	rs790197522	2
AT.AT	16	66957074	66957086	6.5	.	.	intergenic	-0.07	248801 rs7866	2
AAAT.ATTT	16	67280289	67280305	4.25	.	.	intergenic	0.076	rs792619129	2
AT.AT	16	67344955	67344966	6	.	.	intergenic	0.033	rs793829471	2
AC.GT	16	67368567	67368576	5	.	.	intergenic	-0.248	rs693100085	3
AC.GT	16	67397880	67397895	8	.	.	intergenic	0.037	253017 rs7932	2
AT.AT	16	67766845	67766856	6	.	.	intergenic	0.037	096381 rs7938	2
AAAT.ATTT	16	68339182	68339197	4	.	.	intergenic	0.023	rs789791920	2
AT.AT	16	68517534	68517544	5.5	.	.	intergenic	0.242	.	2
AAAC.GTTT	16	68663138	68663156	4.75	.	.	intergenic	0.146	rs792483906	2
AG.CT	16	68673739	68673755	8.5	.	.	intergenic	0.094	rs788806629	2
ACC.GGT	16	68718068	68718082	5	.	.	intergenic	0.099	.	2
AT.AT	16	68767227	68767238	6	.	.	intergenic	-0.072	.	2
AC.GT	16	68925531	68925545	7.5	.	.	intergenic	1.573	779955 rs7934	2
AC.GT	16	69076070	69076084	7.5	ENSSSCT00000018554	ADRA1B	intron	0.254	rs787729948	3
AT.AT	16	69865985	69866000	8	.	.	intergenic	0.085	.	2
AAT.ATT	16	70127727	70127739	4.33333	.	.	intergenic	0.118	515916 rs7866	2
AAAC.GTTT	16	70299390	70299404	3.75	.	.	intergenic	0.109	071784 rs7913	2
AC.GT	16	70400660	70400675	8	.	.	intergenic	0.559	983736 rs7928	2
AG.CT	16	70606464	70606475	6	.	.	intergenic	-0.027	533159 rs7925	4
AC.GT	16	70618023	70618042	10	.	.	intergenic	-0.013	rs787530618	2
AG.CT	16	70650590	70650600	5.5	.	.	intergenic	0.021	rs788956908	2
AAC.GTT	16	70706779	70706793	5	.	.	intergenic	0.415	rs788387433	2
AAAAC.GTTTT	16	70833339	70833365	5.4	.	.	intergenic	-0.031	142578 rs7921	2
AT.AT	16	70834474	70834483	5	.	.	intergenic	0.029	rs789006462	3
AT.AT	16	70845700	70845713	7	.	.	intergenic	0.079	369714 rs7910	3
AC.GT	16	70910605	70910615	5.5	.	.	intergenic	0.056	rs788819814	3
AAAC.GTTT	16	71044189	71044221	8.25	.	.	intergenic	.	573034 rs7910	2
AATG.CATT	16	71063513	71063531	4.75	.	.	intergenic	0.021	rs786225650	2
AAAT.ATTT	16	71167684	71167706	5.75	.	.	intergenic	0.294	rs791025266	2
AAAT.ATTT	16	71264879	71264919	10.25	.	.	intergenic	.	rs786738689	2
AC.GT	16	71433102	71433112	5.5	.	.	intergenic	0.172	rs791009551	2
AAAC.GTTT	16	71458734	71458756	5.75	ENSSSCT00000018562	THG1L	intron	0.135	969343 rs7896	2
AC.GT	16	71546847	71546859	6.5	.	.	intergenic	0.092	.	2
AC.GT	16	71687548	71687557	5	.	.	intergenic	-0.059	.	4
AG.CT	16	71722889	71722903	7.5	ENSSSCT00000018567	CYFIP2	intron	1.493	rs790650791	2
AAC.GTT	16	71945179	71945200	7.33333	.	.	intergenic	.	rs788568997	2
AT.AT	16	71950738	71950749	6	ENSSSCT00000018576	HAVCR1	intron	-0.13	rs789231859	2
AAAAC.GTTTT	16	72260337	72260361	5	.	.	intergenic	0.044	rs790133190	2

AGAT.ATCT	16	72773644	72773655	3	.	.	intergenic	.	.	2
AT.AT	16	73036396	73036405	5	.	.	intergenic	0.032	.	2
AAC.GTT	16	73333651	73333682	10.6667	.	.	intergenic	.	rs793626055	2
AAG.CTT	16	73366574	73366592	6.33333	.	.	intergenic	0.056	.	2
AT.AT	16	73478640	73478653	7	.	.	intergenic	0.005	87625 rs7933	2
AAAAC.GTTTT	16	73554207	73554237	6.2	.	.	intergenic	0.108	rs789221666	2
ATC.GAT	16	73605230	73605242	4.33333	.	.	intergenic	0.238	.	4
AAAAC.GTTTT	16	73913950	73913971	3.66667	.	.	intergenic	.	52656 rs7898	3
AAAC.GTTT	16	73940066	73940085	5	.	.	intergenic	0.283	09540 rs7905	2
AAAC.GTTT	16	73952698	73952734	9.25	.	.	intergenic	0.005	32834 rs7864	2
AG.CT	16	73962610	73962619	5	.	.	intergenic	-0.118	rs787889595	3
AAT.ATT	16	74062698	74062715	6	.	.	intergenic	0.159	27344 rs7876	2
AAAT.ATTT	16	74165432	74165450	4.75	ENSSSCT00000026183	.	intron	0.18	rs793066133	2
AAAT.ATTT	16	74260656	74260678	5.75	ENSSSCT00000018582	CNOT8	intron	.	rs793762889	2
AAAC.GTTT	16	74296308	74296330	5.75	ENSSSCT00000018583	FAXDC2	intron	.	rs788153875	2
AAAC.GTTT	16	74394116	74394131	4	.	.	intergenic	0.111	rs789208972	2
AAAC.GTTT	16	74711332	74711364	8.25	ENSSSCT00000018588	GALNT10	intron	0.209	48898 rs7913	2
AAAT.ATTT	16	74876438	74876462	6.25	ENSSSCT00000018588	GALNT10	intron	0.014	rs793756355	2
AC.GT	16	74934137	74934157	10.5	.	.	intergenic	0.438	.	3
AG.CT	16	74966939	74966951	6.5	.	.	intergenic	0.238	.	2
AT.AT	16	75309652	75309666	7.5	.	.	intergenic	0.135	rs788723784	2
AT.AT	16	75393558	75393583	13	.	.	intergenic	-0.024	rs690440782	3
AAAAC.GTTTT	16	75507285	75507325	6.83333	.	.	intergenic	0.426	38271 rs7878	3
AAC.GTT	16	75615655	75615671	5.66667	.	.	intergenic	0.176	rs793655493	2
AC.GT	16	75619258	75619268	5.5	.	.	intergenic	0.076	rs788354724	2
AT.AT	16	75692800	75692810	5.5	.	.	intergenic	0.028	47992 rs7910	3
AAAT.ATTT	16	75732417	75732435	4.75	.	.	intergenic	-0.01	41814 rs7932	2
AAT.ATT	16	75804413	75804437	8.33333	.	.	intergenic	-0.028	rs787607577	2
AAC.GTT	16	75828822	75828835	4.66667	ENSSSCT00000021150	SNORA18	promoter	0.146	rs789767581	2
AGAT.ATCT	16	75838282	75838299	4.5	.	.	intergenic	-0.058	76963 rs7870	2
AT.AT	16	75963045	75963063	9.5	.	.	intergenic	-0.105	.	4
AATT.AATT	16	76084440	76084459	5	.	.	intergenic	-0.089	rs791625241	2
AC.GT	16	76096776	76096790	7.5	.	.	intergenic	-0.142	85898 rs7899	4
AAAC.GTTT	16	76188743	76188772	7.5	.	.	intergenic	.	14691 rs7906	2
AC.GT	16	76241774	76241788	7.5	.	.	intergenic	0.006	rs792015599	2
ACAT.ATGT	16	76386781	76386797	4.25	.	.	intergenic	.	rs793864195	2
AAT.ATT	16	76392162	76392175	4.66667	.	.	intergenic	-0.014	rs788860150	2
AAAT.ATTT	16	76396315	76396330	4	.	.	intergenic	-0.061	rs791252130	2
AT.AT	16	76932779	76932789	5.5	.	.	intergenic	.	rs786756811	3
AC.GT	16	77065024	77065039	8	.	.	intergenic	0.205	49560 rs7906	2
AT.AT	16	77262484	77262498	7.5	.	.	intergenic	.	rs791204095	3
AC.GT	16	77324535	77324544	5	.	.	intergenic	0.374	rs786236392	2
AAAAC.GTTTT	16	77371546	77371563	3	ENSSSCT00000018594	GLRA1	intron	.	.	2
AAAC.GTTT	16	77651764	77651782	4.75	.	.	intergenic	-0.326	rs793084259	2
AC.GT	16	77731596	77731626	15.5	ENSSSCT00000018600	FAT2	intron	0.161	.	2
AAAC.GTTT	16	77883067	77883090	6	.	.	intergenic	.	rs792705437	2
AAAC.GTTT	16	78594134	78594148	3.75	.	.	intergenic	.	rs792698270	3
ACAT.ATGT	16	78601934	78601952	4.75	.	.	intergenic	.	rs790360427	2
AC.GT	16	79178910	79178919	5	ENSSSCT00000018613	SEMA5A	intron	-0.27	.	2
AT.AT	16	79445193	79445209	8.5	.	.	intergenic	.	rs786567053	2
AC.GT	16	79609721	79609735	7.5	.	.	intergenic	-0.116	.	2
AC.GT	16	79624728	79624741	7	.	.	intergenic	.	rs792698911	3
AAG.CTT	16	79693488	79693503	5.33333	.	.	intergenic	-0.38	.	2
AAAC.GTTT	16	79749999	79750013	3.75	.	.	intergenic	-0.135	rs792238405	2
AAGAC.GTCTT	16	79888736	79888755	4	.	.	intergenic	-0.09	rs787123363	2
AAC.GTT	16	79897177	79897191	5	.	.	intergenic	.	76360 rs7864	2
AAAG.CTTT	16	79920543	79920557	3.75	.	.	intergenic	.	rs787290795	2
AC.GT	16	80020816	80020827	6	.	.	intergenic	-0.268	rs787175608	2
AT.AT	16	80144211	80144220	5	.	.	intergenic	-0.077	rs786425524	2
AC.GT	16	80173267	80173283	8.5	.	.	intergenic	-0.045	rs787874089	2
AAAT.ATTT	16	80207744	80207763	5	.	.	intergenic	0.065	rs787381802	2
AGC.GCT	16	80493117	80493132	5.33333	ENSSSCT00000018619	ADCY2	intron	.	rs787673946	2
AGG.CCT	16	80696944	80696956	4.33333	.	.	intergenic	-0.245	.	2
AG.CT	16	80946680	80946713	17	.	.	intergenic	.	51788 rs7889	2
AAT.ATT	16	80953271	80953290	6.66667	.	.	intergenic	-0.166	.	4
AG.CT	16	81238760	81238773	7	.	.	intergenic	.	.	3
AAC.GTT	16	81260379	81260401	7.66667	.	.	intergenic	-0.186	rs790707918	2
AAGGG.CCCTT	16	81443850	81443872	4.6	.	.	intergenic	0.233	rs787169989	2
AAAAG.CTTTT	16	81678987	81679007	4.2	ENSSSCT00000018621	SRD5A1	intron	-0.585	28279 rs7869	2
AC.GT	16	81828910	81828925	8	ENSSSCT00000018623	UBE2QL1	intron	-0.421	.	3
AAAC.GTTT	16	81859651	81859677	6.75	.	.	intergenic	-0.51	rs791000701	2
AAT.ATT	16	82034658	82034682	8.33333	.	.	intergenic	.	rs793740434	2
AAAT.ATTT	16	82087345	82087359	3.75	.	.	intergenic	0.093	rs793194155	2
AAAC.GTTT	16	82159887	82159909	5.75	.	.	intergenic	-0.153	rs788768092	2
AAAAC.GTTTT	16	82174059	82174083	5	.	.	intergenic	-0.105	.	2
AG.CT	16	82193716	82193734	9.5	.	.	intergenic	-0.482	34434 rs7885	2
AAACC.GGTTT	16	82691913	82691936	4	ENSSSCT00000018627	ADAMTS1	intron	-0.264	rs787387215	2
AC.GT	16	83291951	83291965	7.5	.	.	intergenic	-0.451	.	2
AG.CT	16	83349160	83349183	12	.	.	intergenic	-0.27	rs792231064	4
AG.CT	16	83699640	83699666	13.5	.	.	intergenic	-0.29	rs793298740	2
AC.GT	16	84227837	84227856	10	.	.	intergenic	.	08503 rs7900	2
AC.GT	16	84734158	84734167	5	.	.	intergenic	-0.09	.	2
AAT.ATT	16	85266104	85266121	6	.	.	intergenic	.	.	2
AAAC.GTTT	16	85788108	85788127	5	.	.	intergenic	-0.257	68603 rs7887	2

AAT.ATT	16	86546615	86546642	9.33333	.	.	intergenic	-0.27	rs791434716	2
AG.CT	16	86853391	86853410	10	.	.	intergenic	-0.186	.	2
AAAAAC.GTTTT	17	410948	410975	4.66667	.	.	intergenic	-0.142	208309 rs6936	2
AAAAAC.GTTTT	17	776357	776374	3	.	.	intergenic	0.68	386453 rs7901	2
AAG.CTT	17	1087904	1087917	4.66667	.	.	intergenic	-0.165	rs693826640	2
AG.CT	17	1148957	1148967	5.5	.	.	intergenic	.	.	2
AT.AT	17	1171631	1171640	5	.	.	intergenic	0.048	rs788697671	4
AAAC.GTTT	17	1614436	1614455	5	.	.	intergenic	-0.013	.	3
AAT.ATT	17	1839436	1839447	4	ENSSSCT00000031192	SGCZ	intron	.	.	2
AAAAC.GTTTT	17	2430649	2430678	6	.	.	intergenic	-0.101	369219 rs7885	2
AAC.GTT	17	2433069	2433082	4.66667	.	.	intergenic	-0.025	375924 rs7929	2
AT.AT	17	2476590	2476606	8.5	.	.	intergenic	.	.	3
ATC.GAT	17	2534112	2534126	5	.	.	intergenic	0.046	rs789138771	2
AAAT.ATTT	17	2572467	2572486	5	.	.	intergenic	-0.007	.	2
AG.CT	17	2622999	2623016	9	.	.	intergenic	-0.161	476980 rs7909	2
AAAAC.GTTTT	17	2857413	2857434	4.4	.	.	intergenic	.	rs787276276	2
AC.GT	17	3117784	3117797	7	.	.	intergenic	0.083	rs790589555	2
AAT.ATT	17	3320889	3320900	4	.	.	intergenic	.	.	2
ACATAT.ATATG	17	3517170	3517187	3	ENSSSCT00000031844	TUSC3	intron	.	rs786368690	2
AAAAT.ATTTT	17	3537020	3537041	4.4	.	.	intergenic	-0.228	564080 rs7915	2
AG.CT	17	3737199	3737211	6.5	.	.	intergenic	0.016	.	4
AAC.GTT	17	3766912	3766928	5.66667	.	.	intergenic	.	.	2
AG.CT	17	4089654	4089664	5.5	.	.	intergenic	-0.204	782682 rs7928	2
AG.CT	17	4275652	4275663	6	.	.	intergenic	.	rs698293279	4
AT.AT	17	4325482	4325495	7	.	.	intergenic	.	381288 rs7931	2
AAC.GTT	17	4395792	4395806	5	.	.	intergenic	-0.166	394112 rs7888	2
AAT.ATT	17	4539656	4539678	7.66667	.	.	intergenic	-0.041	505946 rs7935	3
AAAT.ATTT	17	4588053	4588074	5.5	.	.	intergenic	0.037	237210 rs7868	2
AT.AT	17	4888807	4888816	5	.	.	intergenic	0.031	594941 rs7904	3
AAAC.GTTT	17	5136047	5136061	3.75	ENSSSCT00000007646	MICU3	intron	.	747829 rs7928	2
AT.AT	17	5193862	5193881	10	ENSSSCT00000007646	MICU3	intron	.	.	2
AT.AT	17	5417610	5417621	6	ENSSSCT00000007649	.	intron	1.049	388839 rs7902	2
AT.AT	17	5591003	5591013	5.5	.	.	intergenic	0.248	.	3
AT.AT	17	5711924	5711936	6.5	ENSSSCT00000007652	.	intron	-0.069	rs709001361	3
AC.GT	17	6020488	6020502	7.5	.	.	intergenic	0.023	rs786737891	2
AC.GT	17	6405934	6405948	7.5	ENSSSCT00000007661	.	intron	.	371864 rs7884	2
AAAT.ATTT	17	6580001	6580026	6.5	.	.	intergenic	.	469623 rs7875	2
AAGG.CCTT	17	6991220	6991240	5.25	.	.	intergenic	.	.	2
AAT.ATT	17	7077236	7077262	9	.	.	intergenic	.	513794 rs7924	2
AT.AT	17	7143965	7143976	6	.	.	intergenic	.	.	3
AAC.GTT	17	7159874	7159893	6.66667	.	.	intergenic	.	rs786736780	4
ACAT.ATGT	17	7168722	7168739	4.5	.	.	intergenic	0.237	914324 rs7929	2
AAAC.GTTT	17	7256989	7257005	4.25	.	.	intergenic	.	rs789230809	2
AAGG.CCTT	17	7317535	7317552	4.5	.	.	intergenic	.	120908 rs7923	2
AAAT.ATTT	17	7361464	7361477	3.5	.	.	intergenic	.	197230 rs7933	2
AT.AT	17	7373933	7373943	5.5	.	.	intergenic	.	rs792105506	2
AT.AT	17	7789677	7789689	6.5	.	.	intergenic	.	376900 rs7878	2
AT.AT	17	7894309	7894321	6.5	.	.	intergenic	.	543339 rs7937	2
AC.GT	17	7909068	7909082	7.5	.	.	intergenic	.	.	3
AATG.CATT	17	7910126	7910145	5	.	.	intergenic	.	rs787583452	2
AAAC.GTTT	17	8121401	8121420	5	.	.	intergenic	.	.	2
AAAAC.GTTTT	17	8175766	8175795	6	.	.	intergenic	.	rs789424798	3
AC.GT	17	8250508	8250518	5.5	.	.	intergenic	.	.	3
AAAT.ATTT	17	8349009	8349033	6.25	.	.	intergenic	.	.	2
AC.GT	17	8380256	8380265	5	.	.	intergenic	.	589908 rs7873	3
AG.CT	17	8530334	8530352	9.5	.	.	intergenic	.	313783 rs7916	4
AAT.ATT	17	8598186	8598204	6.33333	.	.	intergenic	.	rs789101481	2
AAC.GTT	17	8656043	8656056	4.66667	.	.	intergenic	.	rs788016126	2
AAT.ATT	17	8723373	8723403	10.3333	.	.	intergenic	.	rs788720855	2
AAC.GTT	17	8801790	8801805	5.33333	.	.	intergenic	.	rs792546362	2
AAC.GTT	17	8912581	8912597	5.66667	.	.	intergenic	.	rs788743123	2
AC.GT	17	9048320	9048331	6	.	.	intergenic	.	rs792827543	2
AAAAT.ATTTT	17	9079126	9079146	4.2	.	.	intergenic	.	.	2
AAAGC.GCTTT	17	9083356	9083384	5.8	.	.	intergenic	.	527828 rs7904	2
AGGG.CCCT	17	9268602	9268621	5	.	.	intergenic	.	rs791088945	2
AC.GT	17	9338689	9338715	13.5	ENSSSCT00000007666	FAT1	promoter	.	rs790655778	3
AAAC.GTTT	17	9414627	9414645	4.75	ENSSSCT00000007666	FAT1	intron	.	543909 rs7862	2
AAAT.ATTT	17	9509278	9509296	4.75	.	.	intergenic	.	.	2
AAAC.GTTT	17	9520237	9520255	4.75	.	.	intergenic	.	767883 rs7875	3
AAAT.ATTT	17	9734117	9734140	6	ENSSSCT00000007672	INTS10	intron	-0.24	rs712559077	2
AT.AT	17	9789948	9789958	5.5	.	.	intergenic	0.029	rs787074125	3
AAAC.GTTT	17	9791530	9791548	4.75	.	.	intergenic	0.179	rs788641660	2
AT.AT	17	9941822	9941833	6	.	.	intergenic	-0.184	947577 rs7887	2
AAAC.GTTT	17	9942079	9942093	3.75	.	.	intergenic	.	rs788111480	2
AC.GT	17	10234842	10234854	6.5	ENSSSCT00000007673	SGALNACT	intron	.	.	2
AC.GT	17	10400218	10400230	6.5	.	.	intergenic	.	rs692722958	3
AAC.GTT	17	10939271	10939292	7.33333	.	.	intergenic	-0.66	rs793594790	2
AAAC.GTTT	17	10989167	10989189	5.75	ENSSSCT00000007676	ADAM18	intron	.	rs792802194	2
AC.GT	17	11041659	11041668	5	.	.	intergenic	.	rs789193889	2
AC.GT	17	11230009	11230031	11.5	.	.	intergenic	-0.008	939977 rs7897	3
AAC.GTT	17	11363824	11363841	6	ENSSSCT00000007678	ZMAT4	intron	-0.088	rs789292238	2
AGAT.ATCT	17	11416126	11416149	6	.	.	intergenic	0.075	rs793052636	2
AG.CT	17	11604167	11604177	5.5	.	.	intergenic	-0.102	.	2
AC.GT	17	11897442	11897453	6	.	.	intergenic	-0.073	rs789100991	2

AAAC.GTTT	17	12035076	12035090	3.75	.	.	intergenic	.	.	2
AAAG.CTTT	17	12075793	12075806	3.5	.	.	intergenic	.	.	2
AAAAG.CTTTT	17	12171833	12171870	6.33333	.	.	intergenic	-0.228	rs787159455	2
AG.CT	17	12249648	12249659	6	ENSSSCT00000007691	ANK1	intron	2.39	348299 rs7908	2
ATCC.GGAT	17	12310121	12310142	5.5	ENSSSCT00000007691	ANK1	intron	0.053	rs790923318	2
AC.GT	17	12732711	12732721	5.5	.	.	intergenic	-0.151	rs790093760	2
AAG.CTT	17	13059549	13059560	4	.	.	intergenic	.	rs792592164	2
AT.AT	17	13434164	13434173	5	.	.	intergenic	-0.004	rs793755734	2
AT.AT	17	13475743	13475756	7	.	.	intergenic	.	377877 rs7899	2
AC.GT	17	13478918	13478939	11	.	.	intergenic	0.398	236450 rs7931	2
AAAG.CTTT	17	13642884	13642895	3	.	.	intergenic	-0.066	.	2
AG.CT	17	13789016	13789025	5	ENSSSCT00000030178	.	intron	-0.04	.	2
AAC.GTT	17	13838268	13838289	7.33333	ENSSSCT00000030178	.	intron	.	223977 rs7877	2
AAAAG.CTTTT	17	13930312	13930334	3.83333	.	.	intergenic	.	761517 rs7878	2
AAAC.GTTT	17	13946979	13946995	4.25	ENSSSCT00000028117	.	intron	-0.1	rs790633316	2
AAAT.ATTT	17	13975457	13975474	4.5	ENSSSCT00000028117	.	intron	.	783734 rs7873	2
AAAAG.CTTTT	17	14146415	14146444	6	.	.	intergenic	.	271028 rs7867	2
AG.CT	17	14148135	14148149	7.5	.	.	intergenic	.	rs788706539	3
AAAG.CTTT	17	14173444	14173461	4.5	.	.	intergenic	.	rs787098090	2
AAAT.ATTT	17	14217017	14217035	4.75	ENSSSCT00000007705	.	promoter	-0.171	rs793177169	2
AAAG.CTTT	17	14252930	14252953	6	.	.	intergenic	.	018169 rs7878	2
AG.CT	17	14525694	14525708	7.5	.	.	intergenic	.	.	3
AC.GT	17	14569184	14569194	5.5	ENSSSCT00000027193	RASSF2	intron	.	rs787735379	2
AAAT.ATTT	17	14625455	14625479	6.25	.	.	intergenic	-0.033	rs789716429	2
AAAAC.GTTTT	17	14647300	14647326	4.5	.	.	intergenic	-0.069	rs786531169	2
AT.AT	17	14656935	14656954	10	ENSSSCT00000007708	PRNP	3'utr	0.675	.	4
AG.CT	17	14796389	14796401	6.5	.	.	intergenic	.	rs792037222	3
AAAC.GTTT	17	15029611	15029629	4.75	.	.	intergenic	0.185	553161 rs7900	2
AAAAC.GTTTT	17	15238718	15238735	3.6	ENSSSCT00000026119	PROKR2	intron	-0.052	rs787692585	3
AG.CT	17	15314597	15314607	5.5	.	.	intergenic	0.136	.	3
AT.AT	17	15411546	15411558	6.5	.	.	intergenic	.	rs793837290	2
AAT.ATT	17	15416523	15416542	6.66667	.	.	intergenic	.	rs791227246	2
AT.AT	17	15420557	15420570	7	.	.	intergenic	.	rs792984559	3
AG.CT	17	15444237	15444249	6.5	.	.	intergenic	0.032	509303 rs7928	2
AAAAT.ATTTT	17	15446286	15446312	5.4	.	.	intergenic	-0.306	rs791882511	2
AT.AT	17	15502460	15502475	8	ENSSSCT00000026307	GPCPD1	intron	0.163	rs787536891	3
AT.AT	17	15502460	15502475	8	ENSSSCT00000007712	GPCPD1	intron	0.163	rs787536891	3
AAC.GTT	17	15550710	15550726	5.66667	.	.	intergenic	.	rs791103244	2
AC.GT	17	15776006	15776016	5.5	.	.	intergenic	.	rs790963178	2
AAAT.ATTT	17	15933048	15933073	6.5	ENSSSCT00000007716	MCM8	intron	0.384	rs793726909	2
AAAT.ATTT	17	16455096	16455111	4	ENSSSCT00000007723	FERMT1	intron	-0.077	rs792786546	2
AC.GT	17	16891362	16891372	5.5	.	.	intergenic	0.222	.	2
AAAAC.GTTTT	17	17436892	17436914	3.83333	.	.	intergenic	.	.	2
AAAC.GTTT	17	17474940	17474955	4	.	.	intergenic	.	363157 rs7909	2
AAAAC.GTTTT	17	17665124	17665154	6.2	.	.	intergenic	0.083	rs700211501	2
AC.GT	17	17784859	17784868	5	.	.	intergenic	0.008	rs790468422	2
AAAC.GTTT	17	18124122	18124148	6.75	.	.	intergenic	0.022	432154 rs7896	2
AAC.GTT	17	18128377	18128395	6.33333	.	.	intergenic	0.415	546384 rs6929	2
AATC.GATT	17	18467110	18467129	5	.	.	intergenic	.	165261 rs7886	2
AAT.ATT	17	18864381	18864395	5	ENSSSCT00000027927	HAO1	intron	0.409	rs700404212	2
AG.CT	17	18882285	18882298	7	.	.	intergenic	-0.08	rs793625297	2
AAAT.ATTT	17	18944256	18944275	5	.	.	intergenic	0.185	rs793888166	2
AAAC.GTTT	17	19009043	19009073	7.75	.	.	intergenic	-0.017	rs788289848	2
AT.AT	17	19021630	19021641	6	.	.	intergenic	.	rs787403886	4
AAC.GTT	17	19055111	19055135	8.33333	.	.	intergenic	.	rs790961227	3
AC.GT	17	19059365	19059377	6.5	.	.	intergenic	0.339	rs792165263	2
AAG.CTT	17	19117289	19117312	8	.	.	intergenic	0.148	rs786255526	2
AAAAG.CTTTT	17	19171154	19171181	4.66667	.	.	intergenic	1.365	rs791930312	3
AAT.ATT	17	19196210	19196225	5.33333	.	.	intergenic	-0.062	571952 rs7882	2
AGAT.ATCT	17	19280717	19280729	3.25	.	.	intergenic	-0.107	970571 rs7931	2
AAAT.ATTT	17	19728305	19728319	3.75	ENSSSCT00000007727	PLCB1	intron	0.022	559490 rs7890	2
AAAT.ATTT	17	19735977	19735990	3.5	ENSSSCT00000007727	PLCB1	intron	0.077	rs786290398	2
AT.AT	17	19843606	19843616	5.5	ENSSSCT00000007727	PLCB1	intron	.	rs786437327	3
AC.GT	17	19907441	19907456	8	.	.	intergenic	0.269	rs789720474	2
AAGG.CCTT	17	20423649	20423663	3.75	.	.	intergenic	-1.678	rs789450849	2
AT.AT	17	20496267	20496276	5	.	.	intergenic	0.148	.	2
AC.GT	17	20708583	20708598	8	ENSSSCT00000007729	PLCB4	intron	3.708	513010 rs7010	5
AC.GT	17	20716005	20716021	8.5	ENSSSCT00000007729	PLCB4	intron	.	.	3
AAAT.ATTT	17	20781268	20781286	4.75	.	.	intergenic	-0.12	rs786610494	2
AAAG.CTTT	17	20824051	20824078	7	.	.	intergenic	0.037	722427 rs7880	3
AT.AT	17	21014731	21014743	6.5	.	.	intergenic	.	rs791698454	2
AAAAT.ATTTT	17	21329780	21329802	3.83333	.	.	intergenic	-0.129	957934 rs7919	2
ACAT.ATGT	17	21487135	21487153	4.75	.	.	intergenic	0.147	.	2
AG.CT	17	21500098	21500122	12.5	.	.	intergenic	0.181	rs792509019	2
AT.AT	17	21502491	21502502	6	.	.	intergenic	0.157	918067 rs7898	2
AAC.GTT	17	21518808	21518827	6.66667	.	.	intergenic	-0.15	398085 rs7918	2
ATC.GAT	17	21519311	21519333	7.66667	.	.	intergenic	0.042	505882 rs7904	2
AC.GT	17	21566526	21566556	15.5	.	.	intergenic	.	472045 rs7876	2
AATC.GATT	17	21592480	21592500	5.25	.	.	intergenic	.	rs793082830	2
AAAC.GTTT	17	21683467	21683491	6.25	ENSSSCT00000030392	.	intron	0.173	390527 rs7898	2
AAC.GTT	17	21694285	21694301	5.66667	ENSSSCT00000030392	.	intron	0.095	383530 rs7868	2
AAAC.GTTT	17	21725943	21725970	7	.	.	intergenic	-0.028	146834 rs7915	2
AAAAC.GTTTT	17	21733326	21733359	6.8	.	.	intergenic	-0.047	523388 rs7905	2
AC.GT	17	21958191	21958200	5	ENSSSCT00000007737	SLX4IP	intron	0.074	026462 rs7915	2

AAC.GTT	17	22076880	22076896	5.66667	ENSSSCT00000007738	JAG1	intron	-0.1	rs789609887	2
AC.GT	17	22364515	22364529	7.5	.	.	intergenic	0.097	.	2
AG.CT	17	22495915	22495924	5	.	.	intergenic	0.067	rs787880805	2
AT.AT	17	22592726	22592739	7	.	.	intergenic	0.082	304262 rs7880	2
AC.GT	17	22754677	22754696	10	.	.	intergenic	.	rs793400492	3
AAAT.ATTT	17	23103541	23103556	4	.	.	intergenic	.	rs789940311	5
AAT.ATT	17	23177053	23177077	8.33333	.	.	intergenic	-0.044	rs792646305	2
AAGG.CCTT	17	23204029	23204040	3	.	.	intergenic	-0.059	.	2
AC.GT	17	23229580	23229594	7.5	.	.	intergenic	0.197	rs790329930	3
AAAC.GTTT	17	23291492	23291508	4.25	.	.	intergenic	0.174	rs788466079	2
AC.GT	17	23506987	23507005	9.5	.	.	intergenic	0.229	728073 rs7921	2
ACC.GGT	17	23648671	23648690	6.66667	.	.	intergenic	0.192	rs793348122	3
AC.GT	17	23679140	23679152	6.5	.	.	intergenic	0.038	.	3
AAT.ATT	17	23755438	23755454	5.66667	.	.	intergenic	.	rs790879274	2
AT.AT	17	23885199	23885218	10	.	.	intergenic	-0.127	375132 rs7884	2
AAAC.GTTT	17	24285509	24285535	6.75	.	.	intergenic	-0.228	rs789245939	2
AAT.ATT	17	24305341	24305357	5.66667	.	.	intergenic	.	rs789420133	2
AG.CT	17	24432542	24432558	8.5	ENSSSCT00000007743	SPTLC3	intron	-0.045	.	4
AAAAC.GTTTT	17	24433395	24433433	7.8	ENSSSCT00000007743	SPTLC3	intron	-0.337	308090 rs7910	4
AAAT.ATTT	17	24440481	24440493	3.25	ENSSSCT00000007743	SPTLC3	intron	0.128	rs793565688	2
AACT.AGTT	17	24632251	24632265	3.75	.	.	intergenic	0.099	452503 rs7888	2
AC.GT	17	24639124	24639136	6.5	.	.	intergenic	0.096	rs788808200	2
AG.CT	17	24690554	24690564	5.5	.	.	intergenic	0.035	rs786692219	3
AG.CT	17	24809670	24809682	6.5	.	.	intergenic	0.186	rs790932374	2
AT.AT	17	25201249	25201261	6.5	ENSSSCT00000007749	SEL1L2	intron	0.037	539279 rs7912	2
AAAAC.GTTTT	17	25242255	25242278	4.8	ENSSSCT00000007749	SEL1L2	intron	0.266	.	2
AT.AT	17	25596123	25596135	6.5	.	.	intergenic	.	223497 rs7909	2
AGC.GCT	17	25614867	25614882	5.33333	.	.	intergenic	0.125	940918 rs7871	2
AAAT.ATTT	17	25723656	25723679	6	.	.	intergenic	0.223	rs789465956	2
AC.GT	17	25992365	25992401	18.5	.	.	intergenic	0.024	.	2
AAAAAC.GTTTT	17	26151466	26151483	3	.	.	intergenic	.	284385 rs7936	2
AT.AT	17	26234030	26234040	5.5	.	.	intergenic	0.044	.	2
AG.CT	17	26541955	26541966	6	.	.	intergenic	0.081	rs789430745	3
AAG.CTT	17	26786043	26786067	8.33333	.	.	intergenic	-0.067	355760 rs7920	3
AAAC.GTTT	17	26815779	26815798	5	.	.	intergenic	0.122	rs789124219	2
AAAAC.GTTTT	17	26893814	26893834	4.2	.	.	intergenic	.	308331 rs7876	2
AC.GT	17	26979556	26979576	10.5	.	.	intergenic	0.055	rs793554991	2
AAAG.CTTT	17	27029140	27029160	5.25	.	.	intergenic	0.751	.	2
AAC.GTT	17	27261267	27261287	7	.	.	intergenic	0.121	rs786838129	2
AAAT.ATTT	17	27341953	27341976	6	.	.	intergenic	0.032	275642 rs7929	3
AAT.ATT	17	27405128	27405152	8.33333	.	.	intergenic	-0.007	162785 rs7908	2
AC.GT	17	27475194	27475203	5	.	.	intergenic	0.303	rs787343614	2
AG.CT	17	27478411	27478426	8	.	.	intergenic	-0.009	rs793832608	3
AAT.ATT	17	27494475	27494488	4.66667	.	.	intergenic	0.027	044342 rs7862	2
AC.GT	17	27500262	27500276	7.5	.	.	intergenic	.	rs788747993	2
AT.AT	17	28008962	28008971	5	.	.	intergenic	-0.036	rs788368965	2
AC.GT	17	28010601	28010611	5.5	.	.	intergenic	.	.	3
AAAAC.GTTTT	17	28034470	28034491	4.4	.	.	intergenic	.	rs787694790	2
AAAC.GTTT	17	28271561	28271575	3.75	ENSSSCT00000030802	KIF16B	intron	.	rs791393125	2
AAAC.GTTT	17	28271561	28271575	3.75	ENSSSCT00000007751	KIF16B	intron	.	rs791393125	2
AAAAAC.GTTTT	17	28354947	28354965	3.16667	ENSSSCT00000030802	KIF16B	intron	-0.063	rs791113750	2
AAAAAC.GTTTT	17	28354947	28354965	3.16667	ENSSSCT00000007751	KIF16B	intron	-0.063	rs791113750	2
AAC.GTT	17	28361349	28361362	4.66667	ENSSSCT00000030802	KIF16B	intron	0.053	rs788279451	2
AAC.GTT	17	28361349	28361362	4.66667	ENSSSCT00000007751	KIF16B	intron	0.053	rs788279451	2
AT.AT	17	28444180	28444201	11	.	.	intergenic	.	rs791523716	2
AAAAC.GTTTT	17	28541590	28541610	4.2	.	.	intergenic	0.58	362898 rs7881	2
AT.AT	17	28669231	28669244	7	.	.	intergenic	.	rs793213335	2
AG.CT	17	28819083	28819097	7.5	.	.	intergenic	0.214	rs792074641	2
AT.AT	17	28906857	28906868	6	.	.	intergenic	.	rs789148591	2
AGG.CCT	17	29086814	29086846	11	.	.	intergenic	0.061	rs788566202	2
AGG.CCT	17	29207583	29207603	7	ENSSSCT00000007755	PCSK2	intron	-0.198	.	2
AATG.CATT	17	29525980	29526016	9.25	.	.	intergenic	0.092	rs789121172	2
AAAG.CTTT	17	29743647	29743658	3	.	.	intergenic	-0.101	rs791238635	2
AAAAAC.GTTTT	17	29810482	29810507	4.33333	.	.	intergenic	0.116	rs792486788	2
AAAAC.GTTTT	17	30029186	30029204	3.8	ENSSSCT00000022008	U6	promoter	.	rs790183053	2
ACT.AGT	17	30185161	30185178	6	ENSSSCT00000007766	.	intron	.	rs788055647	2
AAAG.CTTT	17	30472464	30472486	5.75	ENSSSCT00000007771	SCP2D1	promoter	0.062	rs787897889	2
AC.GT	17	30472879	30472889	5.5	ENSSSCT00000007771	SCP2D1	promoter	0.642	rs788267253	2
AC.GT	17	30885413	30885441	14.5	.	.	intergenic	-0.477	.	3
AAAT.ATTT	17	30970372	30970389	4.5	.	.	intergenic	-0.412	.	2
AAAAC.GTTTT	17	31023536	31023555	4	.	.	intergenic	-0.094	rs790887252	2
AAG.CTT	17	31026511	31026525	5	.	.	intergenic	0.006	rs790979159	2
AAAAC.GTTTT	17	31031568	31031596	5.8	.	.	intergenic	.	rs793677976	2
AC.GT	17	31051526	31051540	7.5	.	.	intergenic	-0.533	rs789325850	3
ACAT.ATGT	17	31166172	31166203	8	.	.	intergenic	.	rs789825753	2
AC.GT	17	31187489	31187499	5.5	.	.	intergenic	-0.101	rs788302377	2
AG.CT	17	31233914	31233924	5.5	.	.	intergenic	-0.022	170604 rs7868	2
AC.GT	17	31412684	31412701	9	ENSSSCT00000028523	.	intron	0.058	.	2
AC.GT	17	31412684	31412701	9	ENSSSCT00000007772	.	intron	0.058	.	2
AC.GT	17	31412684	31412701	9	ENSSSCT00000024048	.	intron	0.058	.	2
AC.GT	17	31451902	31451916	7.5	ENSSSCT00000028523	.	intron	-0.197	rs790325298	4
AC.GT	17	31451902	31451916	7.5	ENSSSCT00000007772	.	intron	-0.197	rs790325298	4
AC.GT	17	31451902	31451916	7.5	ENSSSCT00000024048	.	intron	-0.197	rs790325298	4
AC.GT	17	31466732	31466743	6	ENSSSCT00000028523	.	intron	-0.193	.	2

AC.GT	17	31466732	31466743	6	ENSSSCT00000007772	.	intron	-0.193	.	2
AC.GT	17	31466732	31466743	6	ENSSSCT00000024048	.	intron	-0.193	.	2
AAAC.GTTT	17	31550016	31550036	5.25	.	.	intergenic	-0.039	522613 rs7912	2
AAAC.GTTT	17	31765305	31765328	6	ENSSSCT00000023356	.	intron	0.108	rs786414763	2
AAAC.GTTT	17	31765305	31765328	6	ENSSSCT00000007775	.	intron	0.108	rs786414763	2
AAAAT.ATTTT	17	31774068	31774086	3.8	ENSSSCT00000023356	.	intron	.	rs790111198	2
AT.AT	17	32110314	32110325	6	.	.	intergenic	0.126	270587 rs7877	2
AG.CT	17	32736340	32736349	5	.	.	intergenic	1.084	rs788431717	3
AAAC.GTTT	17	32775463	32775480	4.5	.	.	intergenic	-0.075	rs788061374	2
AAAC.GTTT	17	32886719	32886738	5	.	.	intergenic	0.208	rs788938331	2
AC.GT	17	33029088	33029100	6.5	ENSSSCT00000007780	.	intron	-0.238	.	3
AAAC.GTTT	17	33067336	33067355	5	ENSSSCT00000007781	XRN2	intron	.	.	4
AAC.GTT	17	33123465	33123480	5.33333	ENSSSCT00000007781	XRN2	intron	-0.192	rs695856537	2
AC.GT	17	33259634	33259645	6	.	.	intergenic	-0.102	rs789475638	2
AC.GT	17	33425247	33425281	17.5	.	.	intergenic	-0.169	rs791593152	2
ACAG.CTGT	17	33496456	33496480	6.25	.	.	intergenic	.	rs788073640	2
AAAT.ATTT	17	33639789	33639815	6.75	.	.	intergenic	-0.214	rs787673050	2
AT.AT	17	34213113	34213123	5.5	.	.	intergenic	-0.354	rs791933003	2
ACT.AGT	17	34908194	34908220	9	ENSSSCT00000007803	.	intron	-0.277	390048 rs7892	2
AC.GT	17	34914680	34914691	6	ENSSSCT00000007803	.	intron	-0.728	.	2
AC.GT	17	34942541	34942553	6.5	ENSSSCT00000007804	.	intron	0.052	534822 rs7875	2
AC.GT	17	35215896	35215919	12	ENSSSCT00000007807	NINL	intron	-0.061	.	4
AC.GT	17	35215896	35215919	12	ENSSSCT00000026945	NINL	intron	-0.061	.	4
AT.AT	17	35623738	35623748	5.5	.	.	intergenic	-0.077	rs789988502	2
AAAAC.GTTTT	17	36089940	36089959	4	.	.	intergenic	.	rs792814749	2
AAAT.ATTT	17	36111478	36111496	4.75	.	.	intergenic	0.423	rs790726812	2
AGG.CCT	17	36273861	36273883	7.66667	.	.	intergenic	0.147	.	2
AC.GT	17	36870393	36870405	6.5	.	.	intergenic	0.212	rs791067662	2
AC.GT	17	37020875	37020885	5.5	ENSSSCT00000007839	PTPRA	intron	2.235	rs788479611	2
AAAC.GTTT	17	37054130	37054150	5.25	ENSSSCT00000007839	PTPRA	intron	0.276	rs791046241	2
AC.GT	17	37127854	37127864	5.5	.	.	intergenic	0.116	.	2
AAC.GTT	17	37183875	37183886	4	ENSSSCT00000007840	VPS16	intron	.	rs789244729	2
AC.GT	17	37268979	37268993	7.5	ENSSSCT00000007844	EBF4	intron	0.166	rs793088418	2
AAAAT.ATTTT	17	37425494	37425510	3.4	.	.	intergenic	-0.14	rs790066501	2
AAAC.GTTT	17	38093682	38093699	4.5	.	.	intergenic	0.008	319226 rs7895	2
AC.GT	17	38794016	38794029	7	.	.	intergenic	0.228	rs792557192	2
AG.CT	17	39094704	39094719	8	ENSSSCT00000007877	ANGPT4	intron	-0.015	.	2
AAG.CTT	17	39200287	39200299	4.33333	.	.	intergenic	.	rs790624803	2
AAG.CTT	17	39623004	39623024	7	ENSSSCT00000007890	NRSN2	promoter	0.085	rs787598028	2
AAAT.ATTT	17	39822466	39822483	4.5	.	.	intergenic	.	rs788627349	2
AAAT.ATTT	17	39992547	39992571	6.25	.	.	intergenic	.	rs790674418	2
AAAAT.ATTTT	17	40320809	40320827	3.8	.	.	intergenic	.	rs789424556	2
AAAC.GTTT	17	40715817	40715831	3.75	ENSSSCT00000007928	TM9SF4	intron	0.056	rs792989989	2
AC.GT	17	40952673	40952684	6	ENSSSCT00000007930	ASXL1	intron	.	rs793263044	4
AAAAC.GTTTT	17	40969434	40969455	3.66667	ENSSSCT00000007930	ASXL1	intron	-0.181	rs787561533	2
AT.AT	17	41114297	41114315	9.5	.	.	intergenic	0.1	rs792357156	2
AAAC.GTTT	17	41616021	41616035	3.75	ENSSSCT00000007941	.	intron	0.221	rs786407133	2
ATCC.GGAT	17	41656314	41656346	8.25	.	.	intergenic	-0.182	520702 rs7875	2
AAAT.ATTT	17	42037072	42037087	4	ENSSSCT00000030451	CBFA2T2	intron	.	rs787981246	2
AAAC.GTTT	17	42038183	42038209	6.75	ENSSSCT00000030451	CBFA2T2	intron	.	rs787802748	2
AAAT.ATTT	17	42045443	42045467	6.25	ENSSSCT00000030451	CBFA2T2	intron	-0.091	416702 rs7910	2
AAC.GTT	17	42165140	42165153	4.66667	ENSSSCT00000007955	.	intron	2.857	rs789769921	2
AC.GT	17	42274878	42274888	5.5	.	.	intergenic	0.077	.	3
AAAC.GTTT	17	42437701	42437724	6	.	.	intergenic	.	rs788584551	2
AC.GT	17	42453328	42453338	5.5	.	.	intergenic	0.041	.	4
AAC.GTT	17	42468831	42468855	8.33333	.	.	intergenic	.	rs791629325	3
AAAAC.GTTTT	17	43070855	43070878	4.8	ENSSSCT00000007965	ITCH	intron	.	rs786224516	2
AAAAC.GTTTT	17	43070855	43070878	4.8	ENSSSCT00000036055	ITCH	intron	.	rs786224516	2
AAAAC.GTTTT	17	43070855	43070878	4.8	ENSSSCT00000036444	ITCH	intron	.	rs786224516	2
AAAAC.GTTTT	17	43171147	43171171	5	ENSSSCT00000007967	PIGU	intron	.	287018 rs7872	2
AAAT.ATTT	17	43239906	43239934	7.25	ENSSSCT00000007967	PIGU	intron	.	rs791763004	2
AC.GT	17	43302469	43302483	7.5	ENSSSCT00000025489	TP53INP2	intron	0.381	367589 rs7899	5
AAAT.ATTT	17	43746636	43746653	4.5	.	.	intergenic	.	rs786585630	2
AAAT.ATTT	17	43748657	43748675	4.75	.	.	intergenic	.	rs793353344	2
AAAAC.GTTTT	17	43757898	43757916	3.8	.	.	intergenic	.	rs789277823	2
AG.CT	17	43800079	43800093	7.5	.	.	intergenic	.	rs786912706	2
AAC.GTT	17	44064192	44064208	5.66667	ENSSSCT00000024698	.	intron	.	rs787353273	2
AAAC.GTTT	17	44395668	44395682	3.75	ENSSSCT00000007997	RBM39	intron	.	768622 rs7884	2
AC.GT	17	44540480	44540491	6	.	.	intergenic	0.467	.	2
AATG.CATT	17	44877272	44877287	4	ENSSSCT00000008003	EPB41L1	intron	0.054	455201 rs7934	2
AT.AT	17	45167571	45167581	5.5	.	.	intergenic	.	rs789949945	2
AAAC.GTTT	17	45415812	45415836	6.25	ENSSSCT00000008012	NDRG3	intron	0.203	rs793774537	2
AAAAC.GTTTT	17	45418303	45418325	4.6	ENSSSCT00000008012	NDRG3	intron	0.109	391922 rs7912	2
AAAT.ATTT	17	45489992	45490004	3.25	ENSSSCT00000008014	SOGA1	intron	0.026	rs792517554	2
AT.AT	17	45557309	45557328	10	ENSSSCT00000028674	SAMHD1	3'utr	0.157	rs793198034	2
AT.AT	17	45557309	45557328	10	ENSSSCT00000023002	.	3'utr	0.157	rs793198034	2
AAAC.GTTT	17	45720774	45720785	3	.	.	intergenic	.	.	2
AAAC.GTTT	17	45735846	45735869	6	.	.	intergenic	.	rs788091313	2
AC.GT	17	45878990	45879000	5.5	ENSSSCT00000008023	GHRH	intron	.	.	2
AC.GT	17	46301814	46301843	15	ENSSSCT00000024027	CTNNBL1	intron	0.899	531262 rs7867	3
ACAT.ATGT	17	46921715	46921729	3.75	.	.	intergenic	.	.	2
AAAC.GTTT	17	47126753	47126771	4.75	.	.	intergenic	-0.466	rs789385262	2
AG.CT	17	47283004	47283016	6.5	ENSSSCT00000008042	PPP1R16B	intron	.	.	2
AAC.GTT	17	47542861	47542873	4.33333	.	.	intergenic	.	rs787786366	3

AAAC.GTTT	17	47553168	47553192	6.25	.	.	intergenic	-0.067	rs787770571	2
AAAC.GTTT	17	47747319	47747349	7.75	.	.	intergenic	.	561536 rs7896	2
AAAAAC.GTTTT	17	47829826	47829848	3.83333	.	.	intergenic	-0.061	rs793244534	2
AT.AT	17	47886406	47886419	7	.	.	intergenic	0.178	rs792227898	3
AC.GT	17	48558588	48558598	5.5	.	.	intergenic	-0.137	.	3
AAT.ATT	17	48619690	48619707	6	.	.	intergenic	0.109	rs791840147	2
AAAT.ATTT	17	49069604	49069627	6	.	.	intergenic	.	538568 rs7931	2
AAAAG.CTTTT	17	49121095	49121120	5.2	.	.	intergenic	0.301	rs789510259	2
AAT.ATT	17	49129929	49129944	5.33333	.	.	intergenic	0.236	rs786825039	2
AC.GT	17	49599207	49599219	6.5	.	.	intergenic	0.222	rs786536495	2
AG.CT	17	49626297	49626327	15.5	ENSSSCT00000027570	.	intron	0.311	rs788981323	2
AAAC.GTTT	17	49885384	49885407	6	.	.	intergenic	0.032	996537 rs7906	2
AC.GT	17	49944397	49944410	7	.	.	intergenic	0.129	073244 rs7887	2
AAAC.GTTT	17	50129341	50129355	3.75	.	.	intergenic	.	rs789695156	2
AT.AT	17	50530620	50530631	6	ENSSSCT00000029823	PTPRT	intron	-0.236	rs790101313	4
AAAAT.ATTTT	17	50587024	50587041	3.6	ENSSSCT00000029823	PTPRT	intron	-0.107	rs788705093	2
AG.CT	17	50609798	50609808	5.5	ENSSSCT00000029823	PTPRT	intron	0.411	rs788837009	2
AC.GT	17	50622337	50622350	7	ENSSSCT00000029823	PTPRT	intron	-0.23	rs793106284	2
AC.GT	17	50763597	50763615	9.5	.	.	intergenic	0.037	971062 rs7935	2
AAAACC.GGTTT	17	50879587	50879618	5.33333	.	.	intergenic	-0.083	rs790629617	3
AG.CT	17	50892171	50892181	5.5	.	.	intergenic	-0.073	rs792198746	2
AAAAC.GTTTT	17	51348342	51348358	3.4	.	.	intergenic	0.34	rs787895442	2
AG.CT	17	51359476	51359491	8	.	.	intergenic	-0.247	rs792716598	3
AC.GT	17	51885392	51885402	5.5	ENSSSCT00000008064	.	intron	-0.131	rs789274346	2
AG.CT	17	51990711	51990723	6.5	ENSSSCT00000008064	.	intron	.	.	2
AC.GT	17	52000555	52000569	7.5	ENSSSCT00000008064	.	intron	.	rs791707390	2
AC.GT	17	52000555	52000569	7.5	ENSSSCT00000030459	.	intron	.	rs791707390	2
AAAT.ATTT	17	52568106	52568121	4	.	.	intergenic	.	rs792815840	2
AC.GT	17	52748834	52748844	5.5	.	.	intergenic	.	rs788659010	2
AAAT.ATTT	17	52787253	52787289	9.25	.	.	intergenic	.	.	2
AAAC.GTTT	17	52832761	52832781	5.25	ENSSSCT00000008080	PABPC1L	intron	-0.014	216722 rs7901	2
AAAAC.GTTTT	17	53279348	53279365	3.6	ENSSSCT00000008095	.	3'utr	.	rs792123636	2
AAAAC.GTTTT	17	53279348	53279365	3.6	ENSSSCT00000008102	.	intron	.	rs792123636	2
AAAT.ATTT	17	53519967	53519982	4	.	.	intergenic	0.052	753629 rs7927	2
AAC.GTT	17	53566448	53566463	5.33333	.	.	intergenic	.	rs789781051	4
AAAACC.GGTTT	17	53591632	53591651	3.33333	.	.	intergenic	.	rs792812495	2
AC.GT	17	53615322	53615338	8.5	.	.	intergenic	0.01	.	3
AT.AT	17	53633622	53633636	7.5	ENSSSCT00000008124	WFDC3	intron	0.001	971053 rs7908	2
AT.AT	17	53633622	53633636	7.5	ENSSSCT00000008123	WFDC3	intron	0.001	971053 rs7908	2
AG.CT	17	53848938	53848947	5	ENSSSCT00000008141	SLC12A5	intron	0.138	rs793605231	2
AT.AT	17	54295713	54295732	10	.	.	intergenic	0.024	.	2
AAT.ATT	17	54374402	54374420	6.33333	ENSSSCT00000008150	.	promoter	.	rs786830462	2
AGC.GCT	17	54917245	54917256	4	ENSSSCT00000008157	EYA2	intron	0.089	967974 rs7865	2
AG.CT	17	55023886	55023898	6.5	ENSSSCT00000008157	EYA2	intron	-0.266	592380 rs7903	3
AAGG.CCTT	17	56820557	56820575	4.75	.	.	intergenic	-0.18	rs792826776	2
AC.GT	17	56869715	56869726	6	.	.	intergenic	.	rs704343609	2
AAAAAC.GTTTT	17	57054054	57054079	4.33333	ENSSSCT00000008164	ARFGEF2	intron	-0.07	rs793276396	2
AAAC.GTTT	17	57249955	57249982	7	ENSSSCT00000026255	.	intron	-0.158	rs789630884	2
AG.CT	17	57390910	57390921	6	ENSSSCT00000008166	KCNB1	intron	-0.182	rs790360582	2
ACAG.CTGT	17	57429498	57429516	4.75	ENSSSCT00000008166	KCNB1	intron	-0.534	rs790063358	3
AAAAG.CTTTT	17	57620499	57620513	3	.	.	intergenic	0.221	rs790011982	2
AATG.CATT	17	57655940	57655971	8	.	.	intergenic	0.259	273254 rs7936	2
AAAAC.GTTTT	17	57890490	57890505	3.2	ENSSSCT00000029578	UBE2V1	intron	-0.177	rs790296074	2
ATCC.GGAT	17	58310792	58310810	4.75	.	.	intergenic	-0.334	.	2
AAAAAC.GTTTT	17	58815301	58815326	4.33333	ENSSSCT00000008179	DPM1	intron	0.044	751851 rs7882	2
AAAAAC.GTTTT	17	58815301	58815326	4.33333	ENSSSCT00000033789	DPM1	intron	0.044	751851 rs7882	2
AAAAAC.GTTTT	17	58815301	58815326	4.33333	ENSSSCT00000034996	DPM1	intron	0.044	751851 rs7882	2
AAAAAC.GTTTT	17	58815301	58815326	4.33333	ENSSSCT00000034352	DPM1	promoter	0.044	751851 rs7882	2
AAAG.CTTT	17	58993068	58993081	3.5	.	.	intergenic	-0.008	500567 rs7880	2
AC.GT	17	59057547	59057558	6	.	.	intergenic	0.294	.	2
AG.CT	17	59082876	59082891	8	.	.	intergenic	0.106	rs788656405	3
AAGG.CCTT	17	59235002	59235020	4.75	ENSSSCT00000034393	NFATC2	intron	-0.151	785162 rs7916	2
AAGG.CCTT	17	59235002	59235020	4.75	ENSSSCT00000008184	NFATC2	intron	-0.151	785162 rs7916	2
AAAG.CTTT	17	59677048	59677064	4.25	.	.	intergenic	.	rs786668978	2
ACAT.ATGT	17	59717708	59717721	3.5	.	.	intergenic	.	.	2
AT.AT	17	59853449	59853461	6.5	.	.	intergenic	0.048	rs698645216	2
AAG.CTT	17	60052651	60052673	7.66667	.	.	intergenic	-0.043	.	2
AC.GT	17	60066059	60066069	5.5	.	.	intergenic	0.096	rs793759247	2
AC.GT	17	60183400	60183411	6	.	.	intergenic	-0.316	rs789206155	2
AG.CT	17	60602324	60602350	13.5	.	.	intergenic	0.025	277253 rs7862	2
AC.GT	17	60720865	60720875	5.5	.	.	intergenic	0.339	rs788386399	2
AC.GT	17	61067019	61067031	6.5	ENSSSCT00000034759	TSHZ2	intron	-0.16	924910 rs7908	2
AG.CT	17	61196778	61196792	7.5	ENSSSCT00000034759	TSHZ2	intron	0.184	.	2
AAAAG.CTTTT	17	61221976	61221990	3	ENSSSCT00000034759	TSHZ2	intron	-0.409	rs788945266	2
AAG.CTT	17	61333452	61333464	4.33333	.	.	intergenic	.	774386 rs7900	2
AT.AT	17	61339142	61339160	9.5	.	.	intergenic	0.101	.	2
AAAC.GTTT	17	61376323	61376337	3.75	.	.	intergenic	0.107	385620 rs7881	2
AC.GT	17	61552371	61552392	11	.	.	intergenic	0.297	rs786929085	3
AC.GT	17	61738637	61738646	5	ENSSSCT00000008194	BCAS1	intron	-0.337	542762 rs7910	2
AC.GT	17	61861510	61861519	5	.	.	intergenic	-0.092	rs789471760	3
AG.CT	17	62729115	62729128	7	.	.	intergenic	0.547	rs789714250	2
AC.GT	17	62842535	62842548	7	.	.	intergenic	-0.041	323606 rs7892	4
AG.CT	17	62932099	62932126	14	.	.	intergenic	.	rs786365080	2
AC.GT	17	62995953	62995965	6.5	.	.	intergenic	-0.296	.	4

AATG.CATT	17	63118150	63118172	5.75	.	.	intergenic	-0.17	rs786459929	2
AT.AT	17	63210011	63210020	5	.	.	intergenic	-0.025	rs789618714	4
AAT.ATT	17	63235254	63235268	5	.	.	intergenic	-0.021	.	2
AAAAC.GTTTT	17	63458339	63458358	3.33333	.	.	intergenic	-0.109	rs787152782	2
AAAAG.CTTTT	17	63562812	63562827	3.2	.	.	intergenic	0.007	952926 rs7864	2
AC.GT	17	63631321	63631333	6.5	.	.	intergenic	0.663	573006 rs7910	2
AT.AT	17	63640926	63640939	7	.	.	intergenic	-0.028	.	2
AAAT.ATTT	17	63900424	63900438	3.75	.	.	intergenic	0.49	905874 rs7913	2
AAAG.CTTT	17	64070393	64070407	3.75	ENSSSCT00000036639	AURKA	intron	.	rs793883159	2
AAAG.CTTT	17	64070393	64070407	3.75	ENSSSCT00000008202	AURKA	intron	.	rs793883159	2
AC.GT	17	64123581	64123591	5.5	ENSSSCT00000036338	CASS4	intron	-0.013	rs793612080	2
AC.GT	17	64123581	64123591	5.5	ENSSSCT00000008204	CASS4	intron	-0.013	rs793612080	2
AAC.GTT	17	64183024	64183037	4.66667	ENSSSCT00000033915	RTFDC1	intron	-0.097	.	2
AAC.GTT	17	64183024	64183037	4.66667	ENSSSCT00000008206	RTFDC1	intron	-0.097	.	2
AAC.GTT	17	64183024	64183037	4.66667	ENSSSCT00000036126	RTFDC1	intron	-0.097	.	2
AAAC.GTTT	17	64375556	64375573	4.5	.	.	intergenic	0.042	946317 rs7935	2
AC.GT	17	64504561	64504588	14	.	.	intergenic	-0.443	799626 rs7897	2
AAAATT.AATTTT	17	64644981	64644999	3.16667	.	.	intergenic	-0.258	.	2
AATG.CATT	17	64657987	64658004	4.5	.	.	intergenic	-0.297	rs791688942	2
AGG.CCT	17	64712026	64712040	5	.	.	intergenic	-0.49	rs790085415	2
AC.GT	17	65387499	65387511	6.5	.	.	intergenic	-0.367	rs787555801	2
ATCC.GGAT	17	65477889	65477914	6.5	.	.	intergenic	-0.446	rs788306071	2
AC.GT	17	65535731	65535742	6	.	.	intergenic	-0.185	221018 rs7905	2
AT.AT	17	65555021	65555030	5	.	.	intergenic	.	.	2
AC.GT	17	65591794	65591804	5.5	ENSSSCT00000008225	17H20ORF	intron	-0.173	.	2
AAAAC.GTTTT	17	65717256	65717282	5.4	.	.	intergenic	0.252	431444 rs7867	2
AAGG.CCTT	17	66840156	66840175	5	.	.	intergenic	-0.43	rs786747283	2
AC.GT	17	66913966	66913975	5	.	.	intergenic	-0.464	.	2
AAAAT.ATTTT	17	67153397	67153415	3.8	ENSSSCT00000036671	PHACTR3	intron	-0.376	473426 rs7898	2
AAAAT.ATTTT	17	67153397	67153415	3.8	ENSSSCT00000035421	PHACTR3	intron	-0.376	473426 rs7898	2
AAAAT.ATTTT	17	67446205	67446227	4.6	.	.	intergenic	-0.037	rs698287139	2
AG.CT	17	67447916	67447928	6.5	.	.	intergenic	0.404	rs792013594	2
AGGC.GCCT	17	67539637	67539659	5.75	.	.	intergenic	-0.039	.	2
AAAT.ATTT	17	67720405	67720423	4.75	.	.	intergenic	-0.255	rs787840688	2
AC.GT	17	68087358	68087367	5	.	.	intergenic	0.772	rs791133304	4
AC.GT	17	68370597	68370608	6	.	.	intergenic	-0.195	rs788824286	2
AC.GT	17	68391072	68391091	10	ENSSSCT00000008269	.	intron	-0.511	.	3
AC.GT	17	68456453	68456478	13	.	.	intergenic	-0.279	rs786418583	2
AC.GT	17	68684460	68684484	12.5	.	.	intergenic	.	rs793217486	3
AG.CT	17	69068456	69068474	9.5	.	.	intergenic	.	rs787750384	2
AAAC.GTTT	18	56411	56427	4.25	.	.	intergenic	0.108	390708 rs7909	2
AAAAT.ATTTT	18	2810648	2810663	3.2	.	.	intergenic	.	rs792338680	2
AC.GT	18	3811552	3811569	9	.	.	intergenic	-0.257	rs791615176	2
AAAC.GTTT	18	3862549	3862566	4.5	.	.	intergenic	-0.265	rs789346633	2
AAGC.GCTT	18	4339929	4339943	3.75	.	.	intergenic	-0.477	384215 rs7890	2
AAAC.GTTT	18	4416305	4416317	3.25	.	.	intergenic	-0.16	734693 rs7888	2
AT.AT	18	4531456	4531474	9.5	.	.	intergenic	-0.281	.	3
AAAC.GTTT	18	4689169	4689186	4.5	.	.	intergenic	-0.023	rs791183761	2
AAAAC.GTTTT	18	4888123	4888147	5	.	.	intergenic	0.14	rs788251996	2
AAC.GTT	18	4965017	4965028	4	ENSSSCT00000017888	XRCC2	intron	-0.139	rs787696050	2
AAC.GTT	18	4965017	4965028	4	ENSSSCT00000017887	ACTR3B	intron	-0.139	rs787696050	2
AAC.GTT	18	5309670	5309694	8.33333	.	.	intergenic	.	555472 rs7934	2
AC.GT	18	5444278	5444290	6.5	ENSSSCT00000022851	.	promoter	-0.046	rs787049062	2
AC.GT	18	5618849	5618861	6.5	ENSSSCT00000017891	GALNT11	intron	.	rs792081555	2
AAAAC.GTTTT	18	5663877	5663897	4.2	ENSSSCT00000017892	GALNTL5	intron	-0.107	711972 rs7865	2
AAAC.GTTT	18	5753671	5753689	4.75	.	.	intergenic	.	rs792749474	2
AAAC.GTTT	18	5811201	5811216	4	ENSSSCT00000025093	.	intron	-0.227	rs788391813	2
AAAAC.GTTTT	18	6037277	6037304	5.6	ENSSSCT00000017895	RHEB	intron	.	564163 rs7906	2
ACCT.AGGT	18	6292701	6292714	3.5	ENSSSCT00000017897	ssc-mir-671	promoter	-0.113	.	2
AAAG.CTTT	18	7165057	7165075	4.75	ENSSSCT00000017925	CLCN1	intron	2.402	rs789811323	2
AAAC.GTTT	18	7236549	7236570	5.5	ENSSSCT00000017929	GSTK1	intron	0.02	rs699773451	2
AAAC.GTTT	18	7247813	7247835	5.75	.	.	intergenic	.	326275 rs7923	2
AC.GT	18	7396246	7396258	6.5	.	.	intergenic	.	374058 rs7934	2
AAAAC.GTTTT	18	7409125	7409139	3	.	.	intergenic	.	rs792133428	2
AAT.ATT	18	7556080	7556093	4.66667	ENSSSCT00000017936	KEL	intron	-0.365	.	2
AAC.GTT	18	7619682	7619698	5.66667	.	.	intergenic	-0.102	rs791010246	2
AC.GT	18	7633946	7633959	7	.	.	intergenic	0.062	.	3
AAAAT.ATTTT	18	7828858	7828873	3.2	.	.	intergenic	.	rs786849328	3
AC.GT	18	7963707	7963720	7	ENSSSCT00000017947	.	intron	-0.291	.	3
AAAT.ATTT	18	8058835	8058850	4	ENSSSCT00000017951	.	intron	0.145	rs790760811	2
AC.GT	18	8249421	8249441	10.5	.	.	intergenic	.	rs788874354	2
AT.AT	18	8302072	8302081	5	.	.	intergenic	-0.028	rs786649124	2
AC.GT	18	8323815	8323826	6	.	.	intergenic	-0.106	.	2
AATC.GATT	18	8336022	8336043	5.5	.	.	intergenic	0.23	rs788822990	2
AAAC.GTTT	18	8382738	8382751	3.5	ENSSSCT00000027750	CLEC5A	intron	-0.152	rs788897591	2
AAAC.GTTT	18	8382738	8382751	3.5	ENSSSCT00000031347	CLEC5A	intron	-0.152	rs788897591	2
AGC.GCT	18	8446572	8446590	6.33333	.	.	intergenic	.	119213 rs7868	2
AT.AT	18	8493780	8493798	9.5	.	.	intergenic	.	rs787307952	3
AAAC.GTTT	18	8686988	8687003	4	ENSSSCT00000017956	AGK	intron	-0.07	131785 rs7880	2
AAAAG.CTTTT	18	9034596	9034616	4.2	.	.	intergenic	-0.2	rs790585470	2
AAGAG.CTCTT	18	9192972	9192990	3.8	.	.	intergenic	-0.034	287691 rs7910	2
AG.CT	18	9330637	9330651	7.5	.	.	intergenic	0.162	rs789450604	2
ACGC.GCGT	18	9369605	9369620	4	.	.	intergenic	0.184	rs693217313	2
AAC.GTT	18	9408946	9408968	7.66667	ENSSSCT00000014715	MRPS33	promoter	0.299	rs792001318	2

AC.GT	18	9515536	9515548	6.5	.	.	intergenic	.	457660 rs7899	2
AT.AT	18	9545131	9545140	5	ENSSSCT00000017958	BRAF	intron	0.098	rs789967096	2
AAAAT.ATTTT	18	9651380	9651399	4	ENSSSCT00000017959	NDUFB2	intron	.	497272 rs7886	3
AC.GT	18	9656100	9656112	6.5	ENSSSCT00000017959	NDUFB2	promoter	.	.	2
AAAC.GTTT	18	9672151	9672174	6	ENSSSCT00000017960	ADCK2	intron	0.058	rs788652574	2
AAAC.GTTT	18	9672151	9672174	6	ENSSSCT00000028640	ADCK2	intron	0.058	rs788652574	2
AC.GT	18	9808556	9808567	6	ENSSSCT00000017961	DENND2A	intron	.	rs791493396	2
AC.GT	18	9873945	9873961	8.5	.	.	intergenic	-0.121	rs792750843	5
AT.AT	18	10011874	10011884	5.5	ENSSSCT00000031418	.	intron	.	rs787946397	3
AC.GT	18	10266855	10266868	7	ENSSSCT00000017968	TBXAS1	intron	-0.018	945489 rs7874	3
AAAT.ATTT	18	10764279	10764302	6	ENSSSCT00000017974	.	intron	0.119	rs789533051	2
AAC.GTT	18	10783564	10783584	7	ENSSSCT00000017974	.	intron	.	rs786992638	4
AAAAAC.GTTTTT	18	10833424	10833450	4.5	.	.	intergenic	.	rs789123615	2
AC.GT	18	11040499	11040517	9.5	ENSSSCT00000017979	ZAP	intron	-0.324	.	2
AGG.CCT	18	11356634	11356656	7.66667	.	.	intergenic	.	rs789225819	2
AAAT.ATTT	18	11473681	11473694	3.5	ENSSSCT00000017985	SVOPL	intron	-0.141	.	2
AT.AT	18	11681229	11681244	8	.	.	intergenic	0.14	rs791701801	2
AAC.GTT	18	11695404	11695426	7.66667	.	.	intergenic	.	rs786349627	3
AAAC.GTTT	18	11731638	11731656	4.75	.	.	intergenic	.	rs790886982	3
AC.GT	18	12133802	12133814	6.5	ENSSSCT00000017987	.	intron	0.074	.	2
AAC.GTT	18	12178525	12178539	5	.	.	intergenic	.	729634 rs7862	2
AT.AT	18	12183751	12183764	7	.	.	intergenic	.	rs793593030	2
AG.CT	18	12209006	12209024	9.5	ENSSSCT00000017988	CREB3L2	intron	0.123	721569 rs7129	3
AAT.ATT	18	12316745	12316767	7.66667	ENSSSCT00000017988	CREB3L2	intron	0.047	517536 rs7893	2
ATC.GAT	18	12408708	12408720	4.33333	.	.	intergenic	.	rs790356495	2
AC.GT	18	12430275	12430285	5.5	.	.	intergenic	0.023	.	4
AAAAC.GTTTT	18	12585728	12585759	6.4	.	.	intergenic	-0.392	rs789710996	2
AC.GT	18	13007554	13007566	6.5	.	.	intergenic	.	119184 rs7877	4
AT.AT	18	13088554	13088573	10	.	.	intergenic	-0.102	rs695885752	2
AAC.GTT	18	13191777	13191792	5.33333	.	.	intergenic	-0.096	rs793021436	2
AG.CT	18	13214442	13214451	5	.	.	intergenic	0.007	.	3
AAAAT.ATTTT	18	13585131	13585154	4.8	.	.	intergenic	.	rs789574354	2
AG.CT	18	13713288	13713297	5	.	.	intergenic	-0.002	.	4
AT.AT	18	13786441	13786451	5.5	.	.	intergenic	0.073	286195 rs7936	2
AT.AT	18	13818325	13818335	5.5	.	.	intergenic	0.023	520595 rs7887	2
AAC.GTT	18	14269860	14269874	5	.	.	intergenic	-0.367	rs791469655	2
AT.AT	18	14306414	14306423	5	ENSSSCT00000026748	FAM180A	promoter	-0.088	rs792507346	2
ACAG.CTGT	18	14586922	14586939	4.5	.	.	intergenic	0.124	.	2
AC.GT	18	14801094	14801106	6.5	.	.	intergenic	0.021	rs789085790	2
AC.GT	18	14974833	14974845	6.5	.	.	intergenic	.	rs792223271	2
AC.GT	18	14995444	14995456	6.5	.	.	intergenic	.	rs791594755	5
AC.GT	18	15029288	15029300	6.5	ENSSSCT00000017997	AGBL3	intron	2.151	rs789741943	4
AAAC.GTTT	18	15041100	15041118	4.75	ENSSSCT00000017997	AGBL3	intron	.	316292 rs7867	2
AAAC.GTTT	18	15087385	15087403	4.75	ENSSSCT00000027194	CALD1	intron	1.326	090023 rs7866	2
AAAC.GTTT	18	15087385	15087403	4.75	ENSSSCT00000018005	CALD1	intron	1.326	090023 rs7866	2
AAAC.GTTT	18	15087385	15087403	4.75	ENSSSCT00000031119	CALD1	intron	1.326	090023 rs7866	2
AT.AT	18	15111619	15111632	7	ENSSSCT00000027194	CALD1	intron	0.153	263271 rs7871	2
AT.AT	18	15111619	15111632	7	ENSSSCT00000018005	CALD1	intron	0.153	263271 rs7871	2
AT.AT	18	15111619	15111632	7	ENSSSCT00000031119	CALD1	intron	0.153	263271 rs7871	2
AAAC.GTTT	18	15138225	15138243	4.75	ENSSSCT00000027194	CALD1	intron	0.041	064583 rs7883	2
AAAC.GTTT	18	15138225	15138243	4.75	ENSSSCT00000018005	CALD1	intron	0.041	064583 rs7883	2
AAAC.GTTT	18	15138225	15138243	4.75	ENSSSCT00000031119	CALD1	intron	0.041	064583 rs7883	2
AC.GT	18	15140245	15140255	5.5	ENSSSCT00000027194	CALD1	intron	.	.	2
AC.GT	18	15140245	15140255	5.5	ENSSSCT00000018005	CALD1	intron	.	.	2
AC.GT	18	15140245	15140255	5.5	ENSSSCT00000031119	CALD1	intron	.	.	2
AC.GT	18	15277391	15277403	6.5	.	.	intergenic	0.16	916582 rs7886	2
ACTC.GAGT	18	15342632	15342647	4	.	.	intergenic	-0.021	.	2
AAC.GTT	18	15495330	15495357	9.33333	.	.	intergenic	0.102	684425 rs7914	2
AT.AT	18	15624252	15624261	5	.	.	intergenic	.	.	2
AAAAAC.GTTTTT	18	15826298	15826317	3.33333	.	.	intergenic	.	rs789383834	2
AAAT.ATTT	18	15924495	15924519	6.25	ENSSSCT00000018012	LRGUK	intron	.	rs792788046	2
AAAAC.GTTTT	18	15939248	15939277	6	ENSSSCT00000018012	LRGUK	intron	0.115	.	2
AAAG.CTTT	18	15944905	15944916	3	ENSSSCT00000018012	LRGUK	intron	-0.063	rs787967301	2
AGAT.ATCT	18	15979491	15979529	9.75	ENSSSCT00000018012	LRGUK	intron	-0.217	rs787517879	2
AATG.CATT	18	16100932	16100953	5.5	ENSSSCT00000018013	EXOC4	intron	0.218	rs791781695	3
AG.CT	18	16176162	16176174	6.5	ENSSSCT00000018013	EXOC4	intron	0.035	rs791044847	2
AT.AT	18	16200780	16200789	5	ENSSSCT00000018013	EXOC4	intron	0.009	rs789019683	2
AAC.GTT	18	16268540	16268552	4.33333	ENSSSCT00000018013	EXOC4	intron	0.315	514616 rs7914	3
AG.CT	18	16529059	16529077	9.5	.	.	intergenic	0.009	448311 rs7871	4
AAAC.GTTT	18	16669411	16669432	5.5	.	.	intergenic	.	rs793654091	2
AC.GT	18	16790552	16790568	8.5	.	.	intergenic	.	428508 rs7913	3
AAGG.CCTT	18	16948823	16948836	3.5	.	.	intergenic	0.051	.	2
AAAG.CTTT	18	16987279	16987292	3.5	.	.	intergenic	.	rs786393401	2
AATG.CATT	18	17025897	17025915	4.75	.	.	intergenic	-0.118	rs707096974	2
AAAACC.GGTTTT	18	17034827	17034844	3	.	.	intergenic	0.12	rs788290070	2
AAAC.GTTT	18	17054362	17054376	3.75	.	.	intergenic	0.267	rs792843385	2
AT.AT	18	17067127	17067137	5.5	.	.	intergenic	-0.076	577427 rs7936	2
AC.GT	18	17180585	17180617	16.5	ENSSSCT00000018015	CHCHD3	intron	0.197	rs792959729	3
AAAT.ATTT	18	17227523	17227549	6.75	ENSSSCT00000018015	CHCHD3	intron	0.461	.	2
AAAC.GTTT	18	17237897	17237916	5	ENSSSCT00000018015	CHCHD3	intron	0.03	rs790119189	2
AGGG.CCCT	18	17256814	17256829	4	ENSSSCT00000018015	CHCHD3	intron	-0.156	942932 rs7894	2
ACAT.ATGT	18	17578640	17578656	4.25	.	.	intergenic	0.096	rs787700919	2
AAAT.ATTT	18	17854641	17854658	4.5	.	.	intergenic	.	rs788733363	3
AC.GT	18	18007810	18007822	6.5	.	.	intergenic	0.585	rs787382619	2

AAAT.ATTT	18	18275612	18275625	3.5	.	.	intergenic	.	rs786657237	2
AAAG.CTTT	18	18347520	18347538	4.75	.	.	intergenic	.	512772 rs7864	2
AAC.GTT	18	18484913	18484929	5.66667	.	.	intergenic	-0.337	rs793015349	3
AC.GT	18	18546803	18546825	11.5	ENSSSCT00000018018	PODXL	intron	0.183	rs790815607	2
AAAG.CTTT	18	18769771	18769785	3.75	.	.	intergenic	0.464	985954 rs7929	2
AT.AT	18	19103849	19103858	5	.	.	intergenic	.	rs791559372	3
AGAT.ATCT	18	19206217	19206261	11.25	.	.	intergenic	0.095	rs791223942	2
AAAC.GTTT	18	19256579	19256597	4.75	ENSSSCT00000018023	COPG2	intron	.	rs711299152	2
AAC.GTT	18	19376573	19376592	6.66667	.	.	intergenic	.	595185 rs7910	2
AAAAC.GTTTT	18	19537135	19537151	3.4	.	.	intergenic	0.038	rs790320941	2
AAAT.ATTT	18	19606730	19606750	5.25	.	.	intergenic	0.289	rs788783939	2
ACAT.ATGT	18	19664674	19664696	5.75	ENSSSCT00000018033	.	intron	-0.005	rs793366343	2
AAAC.GTTT	18	19975020	19975039	5	.	.	intergenic	0.9	rs792972156	2
AT.AT	18	20345161	20345172	6	ENSSSCT00000018038	AHCYL2	intron	0.504	rs786729614	2
AT.AT	18	20401378	20401391	7	ENSSSCT00000018038	AHCYL2	intron	0.294	rs788822027	2
AAAAC.GTTTT	18	20457992	20458027	7.2	ENSSSCT00000018038	AHCYL2	intron	0.253	rs791774346	2
AT.AT	18	20724401	20724412	6	ENSSSCT00000018042	TNPO3	intron	1.418	531878 rs7881	2
AAAAT.ATTTT	18	20954632	20954647	3.2	ENSSSCT00000018054	FAM71F1	intron	-0.013	597226 rs7892	2
AAAAAG.CTTTT	18	21010316	21010336	3.5	.	.	intergenic	.	rs791968280	2
AAAG.CTTT	18	21398271	21398284	3.5	ENSSSCT00000018062	SND1	intron	0.043	rs792404525	2
AAAC.GTTT	18	21505735	21505762	7	ENSSSCT00000018062	SND1	intron	0.3	rs790339144	2
AATC.GATT	18	21836773	21836794	5.5	.	.	intergenic	0.094	.	2
AAG.CTT	18	21842932	21842947	5.33333	.	.	intergenic	0.051	rs789598817	2
AAAAC.GTTTT	18	21874892	21874906	3	.	.	intergenic	0.262	.	2
AAAT.ATTT	18	21891012	21891036	6.25	.	.	intergenic	0.052	.	2
AAAC.GTTT	18	22040422	22040441	5	.	.	intergenic	-0.24	rs788575647	2
AAAAT.ATTTT	18	22058846	22058863	3.6	.	.	intergenic	0.154	rs786256687	2
AT.AT	18	22113058	22113071	7	.	.	intergenic	0.294	256502 rs7929	2
AC.GT	18	22347499	22347520	11	.	.	intergenic	0.053	342303 rs7886	2
AAGG.CCTT	18	22522789	22522810	5.5	.	.	intergenic	0.173	rs790621780	2
AAAT.ATTT	18	22592320	22592336	4.25	.	.	intergenic	-0.144	rs789906304	2
AT.AT	18	22593307	22593326	10	.	.	intergenic	-0.21	rs787362769	2
AAAT.ATTT	18	22749350	22749369	5	ENSSSCT00000026245	GRM8	intron	0.036	rs786491091	2
AAT.ATT	18	22803112	22803134	7.66667	ENSSSCT00000026245	GRM8	intron	0.246	rs793249297	2
AAG.CTT	18	22887199	22887211	4.33333	ENSSSCT00000026245	GRM8	intron	.	rs792067490	2
AAAAT.ATTTT	18	22917324	22917343	4	ENSSSCT00000026245	GRM8	intron	.	rs790663652	2
AATC.GATT	18	22985712	22985734	5.75	ENSSSCT00000026245	GRM8	intron	0.185	rs788874775	2
AAAC.GTTT	18	23072009	23072032	6	ENSSSCT00000026245	GRM8	intron	0.142	498947 rs7873	2
AC.GT	18	23149358	23149368	5.5	.	.	intergenic	-0.016	485946 rs7875	2
AAAAC.GTTTT	18	23237316	23237331	3.2	.	.	intergenic	0.565	469267 rs7937	2
AAAC.GTTT	18	23346591	23346610	5	.	.	intergenic	.	rs788445775	2
AAT.ATT	18	23526755	23526777	7.66667	.	.	intergenic	-0.137	rs787857474	2
AAAC.GTTT	18	23601091	23601119	7.25	.	.	intergenic	-0.021	rs787739093	2
AAC.GTT	18	23724311	23724324	4.66667	.	.	intergenic	.	369266 rs7935	2
AG.CT	18	23749024	23749034	5.5	.	.	intergenic	.	rs792108976	2
AT.AT	18	24071976	24071992	8.5	.	.	intergenic	0.255	rs786340368	2
AT.AT	18	24119864	24119883	10	.	.	intergenic	-0.06	.	2
AAAC.GTTT	18	24539082	24539107	6.5	.	.	intergenic	0.134	rs791486857	2
AAAC.GTTT	18	24579230	24579248	4.75	.	.	intergenic	0.239	rs788360059	3
AGGG.CCCT	18	24755641	24755663	5.75	.	.	intergenic	0.467	rs791035565	2
AAAC.GTTT	18	24913534	24913548	3.75	.	.	intergenic	0.074	rs788344979	2
ACAT.ATGT	18	25421890	25421903	3.5	.	.	intergenic	0.168	.	2
AT.AT	18	25453027	25453062	18	.	.	intergenic	0.144	rs790257064	3
AATT.AATT	18	25526529	25526548	5	ENSSSCT00000018081	IQUB	intron	0.045	rs786870143	2
AAAG.CTTT	18	25549423	25549449	6.75	ENSSSCT00000018081	IQUB	intron	.	448296 rs7864	2
AC.GT	18	25567918	25567931	7	.	.	intergenic	.	rs690070626	3
AAAAAC.GTTTTT	18	25727215	25727232	3	.	.	intergenic	0.07	591984 rs7871	2
AAAC.GTTT	18	25760802	25760836	8.75	ENSSSCT00000018083	SLC13A1	intron	0.054	rs787687059	3
AAAAC.GTTTT	18	25777492	25777512	4.2	ENSSSCT00000018083	SLC13A1	intron	-0.099	281221 rs7897	2
AT.AT	18	25933392	25933401	5	.	.	intergenic	.	.	4
AC.GT	18	26462676	26462685	5	ENSSSCT00000018085	CADPS2	intron	0.189	460321 rs7902	2
AG.CT	18	26484090	26484099	5	.	.	intergenic	0.149	.	2
AAAT.ATTT	18	26509898	26509914	4.25	.	.	intergenic	-0.245	rs789325119	2
AATG.CATT	18	26802471	26802487	4.25	.	.	intergenic	0.049	356623 rs7899	2
AAAC.GTTT	18	26976925	26976947	5.75	.	.	intergenic	0.303	rs787177690	2
AC.GT	18	27014466	27014481	8	.	.	intergenic	0.202	rs789337720	2
AAT.ATT	18	27090416	27090435	6.66667	.	.	intergenic	.	.	2
ACAG.CTGT	18	27182800	27182819	5	ENSSSCT00000018090	FAM3C	intron	-0.014	rs788697225	2
AG.CT	18	27261616	27261628	6.5	ENSSSCT00000018092	CPED1	3'utr	0.447	rs787141374	2
AC.GT	18	27460393	27460425	16.5	ENSSSCT00000018092	CPED1	intron	-0.022	.	4
AAAAAC.GTTTTT	18	27518669	27518693	4.16667	ENSSSCT00000018092	CPED1	intron	0.029	rs787130159	2
AG.CT	18	27609201	27609210	5	.	.	intergenic	.	.	2
AGG.CCT	18	27904824	27904835	4	.	.	intergenic	-0.165	rs790873383	2
AAAAAC.GTTTTT	18	27933965	27933988	4	.	.	intergenic	0.632	572307 rs7866	2
AAGG.CCTT	18	28016250	28016268	4.75	.	.	intergenic	0.081	rs789123284	2
AAT.ATT	18	28128662	28128681	6.66667	.	.	intergenic	0.186	346547 rs7882	2
AAC.GTT	18	28189778	28189805	9.33333	.	.	intergenic	0.123	.	2
AAAT.ATTT	18	28601136	28601166	7.75	.	.	intergenic	.	rs793331203	2
AG.CT	18	28995095	28995104	5	.	.	intergenic	.	.	3
AC.GT	18	29227414	29227425	6	.	.	intergenic	0.016	.	2
AAC.GTT	18	29256013	29256033	7	.	.	intergenic	.	781304 rs7876	2
AGATAT.ATATCT	18	29478118	29478146	4.83333	.	.	intergenic	-0.081	rs790656194	2
AGG.CCT	18	29489216	29489232	5.66667	.	.	intergenic	0.003	rs787925493	2
AAAC.GTTT	18	29613398	29613416	4.75	.	.	intergenic	.	rs790396035	2

AAAC.GTTT	18	29872418	29872432	3.75	.	.	intergenic	0.064	rs790719667	2
AAC.GTT	18	29886942	29886958	5.66667	.	.	intergenic	0.101	rs786704097	2
AAGC.GCTT	18	29925496	29925513	4.5	.	.	intergenic	-0.028	rs788076387	2
AC.GT	18	30015962	30015982	10.5	.	.	intergenic	0.128	275816 rs7865	2
AAAC.GTTT	18	30111118	30111137	5	.	.	intergenic	0.02	941162 rs7897	2
AAAC.GTTT	18	30323447	30323465	4.75	ENSSSCT00000018099	CTTNBP2	intron	-0.073	018706 rs7906	2
AAAT.ATTT	18	30630290	30630310	5.25	ENSSSCT00000018103	.	intron	0.177	rs792793783	3
AT.AT	18	30667954	30667968	7.5	.	.	intergenic	0.214	.	3
AATG.CATT	18	30684276	30684293	4.5	.	.	intergenic	0.495	rs790712722	2
AATC.GATT	18	31024968	31024995	7	.	.	intergenic	0.198	646336 rs7880	2
ACAG.CTGT	18	31161136	31161156	5.25	ENSSSCT00000018105	ST7	intron	0.232	.	2
AG.CT	18	31464147	31464159	6.5	.	.	intergenic	-0.044	rs790774994	3
AAC.GTT	18	31812705	31812734	10	.	.	intergenic	.	758282 rs7910	4
AAG.CTT	18	31932602	31932616	5	.	.	intergenic	.	787182 rs7915	2
AAAG.CTTT	18	32030134	32030162	7.25	.	.	intergenic	0.106	rs787749626	2
AAAAT.ATTTT	18	32233824	32233839	3.2	ENSSSCT00000018115	TFEC	intron	0.008	rs790937285	2
AAAAT.ATTTT	18	32233824	32233839	3.2	ENSSSCT00000028328	TFEC	intron	0.008	rs790937285	2
AAAT.ATTT	18	32448761	32448781	5.25	.	.	intergenic	-0.008	rs788461917	2
AAAC.GTTT	18	32802941	32802955	3.75	.	.	intergenic	.	rs790194149	2
AG.CT	18	32859642	32859656	7.5	.	.	intergenic	-0.215	.	2
AAG.CTT	18	32867501	32867520	6.66667	.	.	intergenic	-0.051	rs788322948	2
AT.AT	18	33289759	33289770	6	ENSSSCT00000018116	MDFIC	intron	-0.134	900069 rs7919	2
AT.AT	18	33600130	33600139	5	.	.	intergenic	0.282	rs788370305	2
AC.GT	18	33876928	33876947	10	.	.	intergenic	0.933	.	3
AAAC.GTTT	18	33946419	33946441	5.75	.	.	intergenic	.	rs791230733	2
AAAC.GTTT	18	34276943	34276965	5.75	.	.	intergenic	0.123	rs789370903	2
AAG.CTT	18	34308396	34308410	5	.	.	intergenic	-0.047	.	2
AAC.GTT	18	34391795	34391813	6.33333	.	.	intergenic	-0.2	rs792070720	2
AAAC.GTTT	18	34526024	34526054	7.75	.	.	intergenic	.	.	3
AC.GT	18	34530406	34530417	6	.	.	intergenic	0.013	.	3
AACAG.CTGTT	18	34634680	34634703	4	.	.	intergenic	.	.	2
AC.GT	18	34919230	34919250	10.5	.	.	intergenic	.	rs788388944	2
AAAC.GTTT	18	34920381	34920394	3.5	.	.	intergenic	-0.005	rs791131532	2
AAAAT.ATTTT	18	35003756	35003776	4.2	.	.	intergenic	0.161	333296 rs7867	2
AT.AT	18	35062064	35062077	7	.	.	intergenic	.	rs792542328	2
AAAC.GTTT	18	35182434	35182446	3.25	.	.	intergenic	.	rs792846234	2
AC.GT	18	35432138	35432149	6	.	.	intergenic	0.991	rs786320065	2
AAAC.GTTT	18	35646018	35646047	7.5	.	.	intergenic	.	rs792940498	2
AAAT.ATTT	18	35780645	35780675	7.75	.	.	intergenic	0.228	.	2
AAAC.GTTT	18	35804900	35804918	4.75	.	.	intergenic	.	rs793217187	2
AGC.GCT	18	35896563	35896582	6.66667	.	.	intergenic	-0.071	rs790360418	2
AC.GT	18	35922783	35922792	5	ENSSSCT00000018124	IFRD1	intron	2.145	rs789335076	2
AAC.GTT	18	36246849	36246862	4.66667	.	.	intergenic	0.198	.	4
AAC.GTT	18	36423451	36423465	5	ENSSSCT00000029218	.	intron	-0.09	.	2
AC.GT	18	36587845	36587859	7.5	ENSSSCT00000029218	.	intron	0.03	.	5
AAAAC.GTTTT	18	36605075	36605099	5	ENSSSCT00000029218	.	intron	0.063	rs792521377	2
AC.GT	18	36620069	36620081	6.5	ENSSSCT00000029218	.	intron	.	rs789596267	2
AT.AT	18	36808358	36808368	5.5	ENSSSCT00000018126	.	intron	0.096	034550 rs7917	2
AG.CT	18	36898294	36898303	5	.	.	intergenic	-0.147	.	3
AC.GT	18	36930633	36930642	5	.	.	intergenic	.	.	2
AAAC.GTTT	18	37139015	37139043	7.25	.	.	intergenic	0.006	rs792300511	3
AAAG.CTTT	18	37230221	37230241	5.25	.	.	intergenic	0.024	rs792201652	2
AAC.GTT	18	37279350	37279368	6.33333	.	.	intergenic	-0.035	.	2
AAG.CTT	18	37301080	37301093	4.66667	.	.	intergenic	0.183	183477 rs7899	2
AC.GT	18	37306998	37307007	5	.	.	intergenic	0.041	.	4
AC.GT	18	37501176	37501187	6	.	.	intergenic	.	rs792383278	4
AAC.GTT	18	37504491	37504504	4.66667	.	.	intergenic	0.453	rs792437008	2
AC.GT	18	37521026	37521037	6	.	.	intergenic	0.086	rs788743908	3
AC.GT	18	37543188	37543207	10	.	.	intergenic	0.067	.	2
AT.AT	18	37708105	37708115	5.5	.	.	intergenic	0.015	rs790473291	2
AAAG.CTTT	18	37822343	37822361	4.75	.	.	intergenic	0.151	364674 rs7875	2
AAAAC.GTTTT	18	37894255	37894276	4.4	.	.	intergenic	.	206496 rs7898	2
AG.CT	18	38083039	38083054	8	.	.	intergenic	-0.235	rs791114981	3
AAAC.GTTT	18	38157373	38157391	4.75	.	.	intergenic	.	rs788211767	3
AT.AT	18	38285947	38285960	7	.	.	intergenic	-0.051	718419 rs7863	2
AAAAT.ATTTT	18	38313825	38313851	5.4	.	.	intergenic	0.093	746388 rs7881	2
AAACC.GGTTT	18	39287308	39287332	5	.	.	intergenic	.	rs793242782	2
AAAC.GTTT	18	39288314	39288333	5	.	.	intergenic	.	292128 rs7923	2
AG.CT	18	39551698	39551735	19	.	.	intergenic	0.193	977812 rs7895	2
AAAC.GTTT	18	39557788	39557813	6.5	.	.	intergenic	0.042	043788 rs7864	2
AAC.GTT	18	40254145	40254158	4.66667	ENSSSCT00000018133	GPR141	promoter	.	rs790011778	2
AC.GT	18	40292121	40292143	11.5	.	.	intergenic	-0.143	rs790398189	2
AC.GT	18	40432831	40432844	7	.	.	intergenic	0.406	638015 rs7869	4
AC.GT	18	40521637	40521646	5	.	.	intergenic	0.229	670350 rs7900	3
AT.AT	18	40633517	40633533	8.5	ENSSSCT00000018134	ELMO1	intron	-0.029	rs786973053	4
ACC.GGT	18	40736276	40736291	5.33333	ENSSSCT00000018134	ELMO1	intron	-0.132	rs786508784	2
AAAAC.GTTTT	18	40741342	40741372	6.2	ENSSSCT00000018134	ELMO1	intron	0.074	rs791476967	2
AC.GT	18	40973098	40973108	5.5	ENSSSCT00000018134	ELMO1	intron	-0.014	rs792686180	2
AG.CT	18	40979294	40979304	5.5	ENSSSCT00000018134	ELMO1	intron	-0.005	.	2
AC.GT	18	41120603	41120615	6.5	ENSSSCT00000018135	AOAH	intron	0.16	rs790618579	4
AC.GT	18	41120603	41120615	6.5	ENSSSCT00000034056	AOAH	intron	0.16	rs790618579	4
AC.GT	18	41120603	41120615	6.5	ENSSSCT00000033857	AOAH	intron	0.16	rs790618579	4
AGG.CCT	18	41159443	41159457	5	ENSSSCT00000018135	AOAH	intron	-0.056	903901 rs7901	2
AGG.CCT	18	41159443	41159457	5	ENSSSCT00000034056	AOAH	intron	-0.056	903901 rs7901	2

AGG.CCT	18	41159443	41159457	5	ENSSSCT00000033857	AOAH	intron	-0.056	03901 rs7901	2
AG.CT	18	41309361	41309373	6.5	ENSSSCT00000018136	ANLN	intron	.	41284 rs7901	2
AG.CT	18	41824018	41824027	5	.	.	intergenic	-0.15	.	2
AT.AT	18	42184785	42184795	5.5	.	.	intergenic	-0.04	rs792142099	3
AAAC.GTTT	18	42271215	42271230	4	.	.	intergenic	.	72634 rs7895	2
AAAC.GTTT	18	42280600	42280618	4.75	.	.	intergenic	0.229	rs792411311	2
AG.CT	18	42390871	42390889	9.5	.	.	intergenic	0.11	.	4
AT.AT	18	42752478	42752490	6.5	.	.	intergenic	-0.197	rs790105413	3
AG.CT	18	43044777	43044788	6	.	.	intergenic	-0.183	rs790040353	2
CATAT.ATATG	18	43276654	43276678	4.16667	ENSSSCT00000029081	BMPER	intron	.	73135 rs7924	2
CATAT.ATATG	18	43276654	43276678	4.16667	ENSSSCT00000023205	BMPER	intron	.	73135 rs7924	2
CATAT.ATATG	18	43276654	43276678	4.16667	ENSSSCT00000018144	BMPER	intron	.	73135 rs7924	2
AC.GT	18	43334015	43334030	8	ENSSSCT00000029081	BMPER	intron	-0.096	.	4
AC.GT	18	43334015	43334030	8	ENSSSCT00000018144	BMPER	intron	-0.096	.	4
AAAG.CTTT	18	43390885	43390899	3.75	ENSSSCT00000029081	BMPER	intron	-0.123	rs792186691	2
AAAG.CTTT	18	43390885	43390899	3.75	ENSSSCT00000018144	BMPER	intron	-0.123	rs792186691	2
AG.CT	18	43667395	43667405	5.5	.	.	intergenic	0.036	rs786557169	2
AAAC.GTTT	18	44229762	44229773	3	ENSSSCT00000031056	.	intron	.	rs786441937	2
AAC.GTT	18	44237336	44237351	5.33333	ENSSSCT00000031056	.	intron	0.885	rs790125169	2
AAC.GTT	18	44461584	44461607	8	ENSSSCT00000029905	FKBP9	intron	.	rs789306961	2
AC.GT	18	44663759	44663776	9	.	.	intergenic	.	79460 rs7904	2
ACAG.CTGT	18	44696824	44696840	4.25	.	.	intergenic	0.123	rs786546632	2
AAAC.GTTT	18	44777341	44777357	4.25	ENSSSCT00000029825	AVL9	intron	.	rs793190701	2
AG.CT	18	45102270	45102282	6.5	.	.	intergenic	0.231	.	4
AC.GT	18	45181860	45181869	5	.	.	intergenic	-0.001	52707 rs7902	3
AC.GT	18	45217985	45217995	5.5	.	.	intergenic	.	.	4
ACCT.AGGT	18	45320825	45320843	4.75	ENSSSCT00000018147	PDE1C	intron	0.216	rs792269315	2
AAAC.GTTT	18	45358644	45358670	6.75	ENSSSCT00000018147	PDE1C	intron	.	rs792754433	2
AC.GT	18	45415777	45415789	6.5	ENSSSCT00000018147	PDE1C	intron	0.04	rs786921455	2
AAAG.CTTT	18	45418941	45418958	4.5	ENSSSCT00000018147	PDE1C	intron	0.212	rs789816560	2
AAAAC.GTTTT	18	45751974	45751988	3	ENSSSCT00000018150	CCDC129	intron	-0.022	78414 rs7869	2
AAAAC.GTTTT	18	45751974	45751988	3	ENSSSCT00000023430	CCDC129	intron	-0.022	78414 rs7869	2
AC.GT	18	45792254	45792264	5.5	.	.	intergenic	0.279	.	2
AC.GT	18	46197853	46197878	13	.	.	intergenic	0.018	85611 rs7874	2
AC.GT	18	46218315	46218325	5.5	.	.	intergenic	-0.095	rs792809600	2
AAAAC.GTTTT	18	46683869	46683887	3.8	ENSSSCT00000018157	GARS	intron	0.073	rs792312282	2
AC.GT	18	46718588	46718598	5.5	ENSSSCT00000018157	GARS	intron	.	.	2
AT.AT	18	47217851	47217861	5.5	.	.	intergenic	.	rs790073913	2
AAAC.GTTT	18	47516901	47516927	6.75	.	.	intergenic	.	rs791040824	2
AAAG.CTTT	18	47663217	47663231	3.75	ENSSSCT00000018168	CHN2	intron	-0.049	.	3
AG.CT	18	47672723	47672738	8	ENSSSCT00000018168	CHN2	intron	0.104	.	2
AC.GT	18	47760927	47760936	5	.	.	intergenic	-0.185	rs787229993	2
AAGG.CCTT	18	47951708	47951726	4.75	.	.	intergenic	0.029	rs789639921	2
AG.CT	18	48140879	48140893	7.5	.	.	intergenic	-0.175	rs708248009	4
AAAC.GTTT	18	48141119	48141137	4.75	.	.	intergenic	-0.215	88535 rs7892	3
AAAC.GTTT	18	48264793	48264812	5	ENSSSCT00000018171	.	intron	0.234	rs791037364	2
AG.CT	18	48303959	48303971	6.5	ENSSSCT00000018171	.	intron	0.423	.	3
AAAAG.CTTTT	18	48307049	48307067	3.8	ENSSSCT00000018171	.	intron	0.632	.	2
AAAAC.GTTTT	18	48412067	48412091	5	.	.	intergenic	0.427	rs791409420	2
AG.CT	18	48704531	48704542	6	.	.	intergenic	-0.078	09101 rs7867	2
AAC.GTT	18	48883898	48883914	5.66667	.	.	intergenic	0.149	26902 rs7934	2
AAAG.CTTT	18	48907320	48907336	4.25	.	.	intergenic	0.505	40486 rs7886	2
AATG.CATT	18	49018039	49018061	5.75	.	.	intergenic	.	.	2
AT.AT	18	49177399	49177409	5.5	.	.	intergenic	0.005	rs791422392	2
AATC.GATT	18	49191392	49191406	3.75	.	.	intergenic	-0.29	rs792604424	2
AG.CT	18	49331976	49331985	5	ENSSSCT00000036364	TAX1BP1	intron	1.121	.	2
AAAAC.GTTTT	18	49350303	49350323	4.2	ENSSSCT00000018173	TAX1BP1	intron	.	rs791389669	2
AAAAC.GTTTT	18	49350303	49350323	4.2	ENSSSCT00000036364	TAX1BP1	intron	.	rs791389669	2
AAAC.GTTT	18	49415676	49415694	4.75	.	.	intergenic	0.066	89896 rs7915	2
AAAC.GTTT	18	50240295	50240308	3.5	.	.	intergenic	0.724	rs788834182	2
AC.GT	18	50845451	50845461	5.5	ENSSSCT00000018190	SNX10	intron	0.227	rs788260148	4
AT.AT	18	50848764	50848782	9.5	ENSSSCT00000018190	SNX10	intron	1.09	.	2
AAAG.CTTT	18	51179199	51179211	3.25	.	.	intergenic	0.012	rs791682250	2
AAT.ATT	18	51192432	51192448	5.66667	.	.	intergenic	0.396	rs792335094	2
CACCC.GGGTG	18	51295986	51296009	4	.	.	intergenic	0.706	.	2
AT.AT	18	51357788	51357805	9	.	.	intergenic	.	rs791248633	2
AG.CT	18	51606940	51606956	8.5	.	.	intergenic	-0.055	rs791815901	2
AAGG.CCTT	18	51989080	51989101	5.5	.	.	intergenic	0.215	28562 rs7936	2
AAC.GTT	18	52028181	52028197	5.66667	.	.	intergenic	.	rs713405976	2
AAAAC.GTTTT	18	52249930	52249949	3.33333	.	.	intergenic	-0.038	107354 rs7919	2
AC.GT	18	52342399	52342411	6.5	ENSSSCT00000018196	OSBPL3	intron	.	43916 rs7886	2
AC.GT	18	52342399	52342411	6.5	ENSSSCT00000028478	OSBPL3	intron	.	43916 rs7886	2
AC.GT	18	52342399	52342411	6.5	ENSSSCT00000030453	OSBPL3	intron	.	43916 rs7886	2
AAAAC.GTTTT	18	52438918	52438933	3.2	ENSSSCT00000018197	DFNA5	intron	.	rs786929688	2
AC.GT	18	52576240	52576250	5.5	ENSSSCT00000018198	MPP6	intron	0.39	rs792289562	2
AAT.ATT	18	52814773	52814788	5.33333	.	.	intergenic	0.047	rs789291568	2
AG.CT	18	52927246	52927260	7.5	.	.	intergenic	0.097	rs787722910	4
AAAT.ATTT	18	53069861	53069878	4.5	.	.	intergenic	.	58498 rs7881	2
AG.CT	18	53092269	53092279	5.5	.	.	intergenic	0.334	rs787293934	2
AG.CT	18	53204158	53204167	5	.	.	intergenic	0.187	.	2
AC.GT	18	53307882	53307892	5.5	ENSSSCT00000018200	STK31	intron	0.242	92971 rs7871	2
AAAC.GTTT	18	53310676	53310694	4.75	ENSSSCT00000018200	STK31	intron	.	rs711574465	2
AAAG.CTTT	18	53473330	53473350	5.25	.	.	intergenic	.	.	2
AAAT.ATTT	18	53502872	53502889	4.5	.	.	intergenic	.	rs791410620	2

AC.GT	18	54171718	54171736	9.5	.	.	intergenic	.	rs793722620	6
AAAC.GTTT	18	54177831	54177849	4.75	.	.	intergenic	.	465139 rs7886	3
AC.GT	18	54559085	54559099	7.5	.	.	intergenic	-0.009	rs791995221	4
AAAAT.ATTTT	18	54613180	54613194	3	.	.	intergenic	-0.214	rs791360732	3
AAAC.GTTT	18	54749453	54749475	5.75	.	.	intergenic	.	rs788538873	2
AACC.GGTT	18	54868455	54868476	5.5	ENSSSCT00000018209	IGFBP1	promoter	.	551300 rs7890	2
AGC.GCT	18	55063082	55063094	4.33333	.	.	intergenic	.	rs789731776	2
AC.GT	18	55080555	55080566	6	.	.	intergenic	-0.47	.	2
AC.GT	18	55097381	55097410	15	.	.	intergenic	.	rs788862386	2
AAC.GTT	18	55270490	55270515	8.66667	.	.	intergenic	.	760458 rs7912	3
AAAG.CTTT	18	55300118	55300141	6	.	.	intergenic	-0.244	rs792309624	2
AT.AT	18	55324387	55324396	5	.	.	intergenic	0.197	rs789547807	3
AAAAC.GTTTT	18	55330252	55330270	3.8	.	.	intergenic	0.225	425092 rs7896	2
AAAG.CTTT	18	55655281	55655304	6	ENSSSCT00000018228	NPC1L1	intron	0.04	rs791733617	2
AAC.GTT	18	55862960	55862975	5.33333	ENSSSCT00000018232	YKT6	intron	0.157	.	2
AAAG.CTTT	18	55883948	55883965	4.5	.	.	intergenic	.	rs790607305	2
AT.AT	18	56121758	56121768	5.5	.	.	intergenic	0.155	.	3
AAAT.ATTT	18	56139399	56139414	4	.	.	intergenic	.	rs789429860	2
AAAC.GTTT	18	56338189	56338207	4.75	ENSSSCT00000018242	HECW1	intron	0.047	167144 rs7868	2
AT.AT	18	56344021	56344030	5	ENSSSCT00000018242	HECW1	intron	0.147	.	2
AAAC.GTTT	18	56662106	56662126	5.25	.	.	intergenic	0.135	.	2
AAAAT.ATTTT	18	56746489	56746509	4.2	.	.	intergenic	0.2	rs787812786	2
AC.GT	18	57144526	57144535	5	.	.	intergenic	0.276	rs787740260	3
AAT.ATT	18	57219453	57219471	6.33333	.	.	intergenic	-0.101	rs792919394	2
AAAT.ATTT	18	57238571	57238585	3.75	.	.	intergenic	-0.405	rs791033246	2
AAAC.GTTT	18	57650571	57650593	5.75	ENSSSCT00000018246	.	intron	0.961	rs792864559	2
AG.CT	18	57920861	57920875	7.5	.	.	intergenic	-0.051	rs787116408	4
AAAT.ATTT	18	57931973	57931995	5.75	.	.	intergenic	-0.054	rs788273724	2
AAT.ATT	18	58075328	58075344	5.66667	.	.	intergenic	0.047	rs786345258	3
AC.GT	18	58082080	58082093	7	.	.	intergenic	-0.01	rs789033654	3
AAAC.GTTT	18	58100859	58100875	4.25	.	.	intergenic	0.344	rs792729950	2
AAAG.CTTT	18	58102093	58102110	4.5	.	.	intergenic	0.02	rs792532763	2
AC.GT	18	58168203	58168213	5.5	.	.	intergenic	-0.025	rs791233820	2
AC.GT	18	58352888	58352906	9.5	.	.	intergenic	0.134	.	3
AAT.ATT	18	58764277	58764296	6.66667	.	.	intergenic	.	rs792582410	2
AAAT.ATTT	18	58770041	58770058	4.5	.	.	intergenic	.	.	2
AAAC.GTTT	18	58775118	58775132	3.75	.	.	intergenic	.	150404 rs7912	2
AAGG.CCTT	18	58988461	58988499	9.75	.	.	intergenic	.	rs786808874	2
AC.GT	18	59075823	59075858	18	.	.	intergenic	0.748	rs791622845	2
AC.GT	18	59676748	59676757	5	ENSSSCT00000018255	CDK13	intron	.	rs788510794	2
AAAC.GTTT	18	59684163	59684176	3.5	ENSSSCT00000018255	CDK13	intron	.	rs789784177	2
AC.GT	18	59983698	59983720	11.5	.	.	intergenic	0.132	rs793197482	2
AC.GT	18	60176643	60176662	10	.	.	intergenic	0.17	.	3
AC.GT	18	60225079	60225093	7.5	.	.	intergenic	0.026	560359 rs7894	4
AAAG.CTTT	18	60228659	60228678	5	.	.	intergenic	-0.165	rs791535772	2
AAAC.GTTT	2	278001	278028	7	.	.	intergenic	.	564810 rs7917	2
AAAC.GTTT	2	767768	767787	5	.	.	intergenic	.	103490 rs7899	2
AC.GT	2	840398	840417	10	.	.	intergenic	.	511180 rs7916	2
AAAG.CTTT	2	1993925	1993952	7	.	.	intergenic	.	rs790023774	2
AT.AT	2	2184000	2184009	5	.	.	intergenic	-0.11	.	2
AAAAG.CTTTT	2	2219186	2219208	4.6	.	.	intergenic	.	rs787235985	2
AT.AT	2	2234711	2234721	5.5	.	.	intergenic	-0.454	rs786901894	2
AGGG.CCCT	2	2609038	2609049	3	.	.	intergenic	-0.487	rs787531496	2
AAAT.ATTT	2	2812237	2812267	7.75	.	.	intergenic	-0.16	.	2
AC.GT	2	2999742	2999751	5	ENSSSCT00000014085	PPP6R3	intron	-0.211	.	3
AAAAC.GTTTT	2	3771094	3771117	4.8	.	.	intergenic	.	rs790131631	2
AAAC.GTTT	2	3837290	3837313	6	ENSSSCT00000014115	TBC1D10C	5'utr	0.211	rs790893342	2
AAAC.GTTT	2	3837290	3837313	6	ENSSSCT00000014116	PPP1CA	promoter	0.211	rs790893342	2
AAAC.GTTT	2	3952495	3952512	4.5	.	.	intergenic	0.178	rs788800834	2
AAC.GTT	2	5253017	5253032	5.33333	.	.	intergenic	.	rs791406695	2
AC.GT	2	5530131	5530142	6	.	.	intergenic	.	.	3
AAAC.GTTT	2	6170322	6170337	4	.	.	intergenic	.	322689 rs7895	2
AAC.GTT	2	6392603	6392640	12.6667	ENSSSCT00000026889	ATG2A	promoter	0.036	541086 rs7893	2
AC.GT	2	6592145	6592155	5.5	ENSSSCT00000014237	NRXN2	intron	0.061	rs789225295	4
AAAC.GTTT	2	6839535	6839554	5	.	.	intergenic	.	327488 rs7887	2
AT.AT	2	7997666	7997677	6	.	.	intergenic	.	rs791382855	2
AC.GT	2	8013935	8013946	6	ENSSSCT00000024497	.	intron	-0.445	rs791202983	6
AAG.CTT	2	8016903	8016927	8.33333	ENSSSCT00000024497	.	promoter	.	rs786732840	2
AAC.GTT	2	8207683	8207703	7	.	.	intergenic	-0.088	rs789759580	2
AAAAAC.GTTTTT	2	8404150	8404170	3.5	ENSSSCT00000025488	BSCL2	intron	-0.056	rs789499543	2
AGG.CCT	2	8985691	8985705	5	ENSSSCT00000015890	.	intron	3.292	.	2
AAC.GTT	2	9438228	9438239	4	.	.	intergenic	.	940625 rs7879	2
AGG.CCT	2	9718112	9718128	5.66667	ENSSSCT00000014314	DDB1	promoter	-0.206	rs788596908	2
AGG.CCT	2	9718112	9718128	5.66667	ENSSSCT00000014305	DAK	promoter	-0.206	rs788596908	2
AG.CT	2	9981197	9981206	5	.	.	intergenic	.	rs793875049	2
AAC.GTT	2	11710722	11710742	7	.	.	intergenic	.	118469 rs7928	2
AAAC.GTTT	2	11818832	11818851	5	ENSSSCT00000014365	DTX4	intron	-0.023	rs787285194	2
AAAC.GTTT	2	11931366	11931404	9.75	.	.	intergenic	-0.59	722318 rs7921	2
AAAC.GTTT	2	12176429	12176444	4	.	.	intergenic	.	rs791191782	2
AT.AT	2	12176612	12176638	13.5	.	.	intergenic	0.318	794714 rs7899	2
AAC.GTT	2	12245337	12245369	11	.	.	intergenic	-0.086	576231 rs7897	2
AAT.ATT	2	12617796	12617812	5.66667	ENSSSCT00000014390	OR6Q1	promoter	-0.184	rs789362561	2
AG.CT	2	12654047	12654062	8	.	.	intergenic	.	rs791793108	3
AAAAC.GTTTT	2	12899137	12899155	3.8	ENSSSCT00000014398	ZDHHC5	intron	0.382	526449 rs7938	2

AT.AT	2	12937922	12937931	5	.	.	intergenic	0.194	rs790475174	2
AGCCC.GGGCT	2	12939678	12939692	3	.	.	intergenic	0.15	rs787171033	2
AAAT.ATTT	2	13100893	13100910	4.5	.	.	intergenic	.	rs793849082	2
ACAG.CTGT	2	13633030	13633048	4.75	.	.	intergenic	-0.249	rs793513735	2
AT.AT	2	13639999	13640010	6	.	.	intergenic	-0.181	345041 rs7911	2
AC.GT	2	13807177	13807187	5.5	.	.	intergenic	0.034	rs787183856	3
AAAT.ATTT	2	13826658	13826669	3	.	.	intergenic	0.091	.	2
AG.CT	2	14305857	14305881	12.5	.	.	intergenic	.	.	4
AT.AT	2	14758476	14758487	6	.	.	intergenic	.	rs792448497	2
AATT.AATT	2	15007497	15007511	3.75	.	.	intergenic	.	.	2
AC.GT	2	15178808	15178821	7	.	.	intergenic	.	389546 rs7910	2
AAT.ATT	2	15368445	15368460	5.33333	.	.	intergenic	.	.	2
AAC.GTT	2	15395978	15395994	5.66667	.	.	intergenic	.	300987 rs7875	2
AC.GT	2	16476170	16476181	6	ENSSSCT00000014456	MYBPC3	3'utr	-0.075	rs790142520	2
AAAAAG.CTTTTT	2	16642783	16642806	4	ENSSSCT00000014466	.	intron	0.178	199734 rs7863	2
AAAT.ATTT	2	16711323	16711343	5.25	ENSSSCT00000014466	.	intron	.	rs788694041	2
AT.AT	2	16768766	16768776	5.5	ENSSSCT00000014466	.	intron	.	rs790199661	2
AAG.CTT	2	16808589	16808602	4.66667	ENSSSCT00000014466	.	intron	0.226	.	2
AC.GT	2	17126872	17126881	5	ENSSSCT00000014469	CKAP5	intron	.	rs787588165	2
AG.CT	2	17657161	17657189	14.5	.	.	intergenic	0.085	rs790618398	2
AAAAT.ATTTT	2	17847703	17847725	4.6	.	.	intergenic	0.284	rs792542651	2
AAC.GTT	2	18637232	18637247	5.33333	ENSSSCT00000014499	SYT13	3'utr	-0.151	911148 rs7867	2
AG.CT	2	19058799	19058811	6.5	.	.	intergenic	-0.26	.	2
AC.GT	2	19125574	19125589	8	.	.	intergenic	-0.167	rs792388820	2
AAAAC.GTTTT	2	19431722	19431750	5.8	.	.	intergenic	-0.085	rs791650122	2
AG.CT	2	19489753	19489764	6	.	.	intergenic	-0.011	rs787286974	2
AAAAC.GTTTT	2	19528946	19528965	4	.	.	intergenic	-0.196	rs792382232	2
AG.CT	2	19670762	19670772	5.5	.	.	intergenic	-0.518	rs791048266	2
AAAC.GTTT	2	19768046	19768069	6	ENSSSCT00000014505	EXT2	intron	0.129	rs793521412	2
AAAC.GTTT	2	20145865	20145879	3.75	ENSSSCT00000027104	HSD17B12	intron	-0.128	rs790892673	2
AAAC.GTTT	2	20145865	20145879	3.75	ENSSSCT00000031374	HSD17B12	intron	-0.128	rs790892673	2
AAAC.GTTT	2	20195213	20195229	4.25	ENSSSCT00000027104	HSD17B12	intron	1.138	rs791328046	2
AAAC.GTTT	2	20195213	20195229	4.25	ENSSSCT00000031374	HSD17B12	intron	1.138	rs791328046	2
AAAAC.GTTTT	2	20275035	20275062	5.6	.	.	intergenic	.	598695 rs7865	3
AC.GT	2	20278841	20278853	6.5	.	.	intergenic	.	rs792326767	2
AAAC.GTTT	2	20542414	20542428	3.75	ENSSSCT00000014508	TTC17	intron	0.01	rs789043360	2
AAC.GTT	2	20647405	20647427	7.66667	ENSSSCT00000014508	TTC17	intron	0.191	269465 rs7868	3
AAAC.GTTT	2	20709009	20709035	6.75	.	.	intergenic	-0.13	rs792226030	2
AAAC.GTTT	2	20792860	20792874	3.75	.	.	intergenic	.	064387 rs7864	2
AC.GT	2	20844818	20844838	10.5	.	.	intergenic	0.573	594316 rs7872	4
AAAT.ATTT	2	21402505	21402527	5.75	.	.	intergenic	.	rs786611707	2
AAAG.CTTT	2	21521115	21521127	3.25	.	.	intergenic	-0.149	rs793606615	2
AAAAC.GTTTT	2	21539685	21539718	6.8	.	.	intergenic	0.069	rs786955211	2
AAC.GTT	2	21656216	21656233	6	.	.	intergenic	-0.019	rs791240347	2
ACAGC.GCTGT	2	21728568	21728602	7	.	.	intergenic	-0.298	.	2
ACAT.ATGT	2	21735197	21735216	5	.	.	intergenic	-0.163	rs792038706	2
AG.CT	2	21904530	21904542	6.5	.	.	intergenic	-0.15	308769 rs7880	3
AAAG.CTTT	2	22263244	22263260	4.25	.	.	intergenic	-0.223	781074 rs7912	2
AGC.GCT	2	22323137	22323150	4.66667	.	.	intergenic	.	rs700519962	2
AC.GT	2	22325849	22325861	6.5	.	.	intergenic	.	rs789848479	2
AG.CT	2	22330315	22330326	6	.	.	intergenic	-0.167	rs789212618	3
AC.GT	2	22347604	22347624	10.5	.	.	intergenic	0.336	rs792052065	2
AAAC.GTTT	2	22398311	22398330	5	.	.	intergenic	-0.069	308784 rs7915	3
AAG.CTT	2	22544666	22544680	5	.	.	intergenic	0.141	783026 rs7887	3
AG.CT	2	22565814	22565826	6.5	.	.	intergenic	-0.152	rs788693119	2
AAGG.CCTT	2	22642282	22642300	4.75	.	.	intergenic	0.033	rs793463545	2
AT.AT	2	22784452	22784479	14	.	.	intergenic	.	.	2
AC.GT	2	22966435	22966454	10	.	.	intergenic	-0.074	rs787785885	3
AT.AT	2	22974074	22974085	6	.	.	intergenic	.	.	2
AC.GT	2	23100531	23100541	5.5	.	.	intergenic	0.076	rs696331745	2
AAGG.CCTT	2	23167309	23167331	5.75	.	.	intergenic	-0.109	rs787559129	2
AAAC.GTTT	2	23169608	23169622	3.75	.	.	intergenic	.	rs788529504	2
AC.GT	2	23232549	23232565	8.5	.	.	intergenic	.	970413 rs7907	4
AAAC.GTTT	2	23358454	23358472	4.75	.	.	intergenic	-0.158	372056 rs7864	2
AAAG.CTTT	2	23472939	23472954	4	.	.	intergenic	0.065	054175 rs7900	2
AAAT.ATTT	2	23484991	23485014	6	ENSSSCT00000010323	.	promoter	0.158	rs793219963	2
AGGG.CCCT	2	23487958	23487972	3.75	.	.	intergenic	0.284	rs702363069	2
AG.CT	2	23543154	23543164	5.5	.	.	intergenic	0.635	087726 rs7887	2
AAAC.GTTT	2	23647672	23647693	5.5	.	.	intergenic	.	rs793634634	2
AAAC.GTTT	2	23722325	23722352	7	.	.	intergenic	0.051	998546 rs7930	3
AAC.GTT	2	23811409	23811422	4.66667	.	.	intergenic	-0.069	rs791919475	2
AAAT.ATTT	2	23816030	23816056	6.75	.	.	intergenic	0.001	362475 rs7887	2
AAAC.GTTT	2	23836000	23836030	7.75	.	.	intergenic	0.401	rs793284283	2
AAT.ATT	2	24009611	24009643	11	.	.	intergenic	-0.042	519723 rs7914	2
AC.GT	2	24021898	24021916	9.5	.	.	intergenic	-0.171	.	4
ACAT.ATGT	2	24065004	24065025	5.5	.	.	intergenic	.	575736 rs7899	2
AAC.GTT	2	24065958	24065971	4.66667	.	.	intergenic	.	290484 rs7891	2
AC.GT	2	24111063	24111074	6	.	.	intergenic	.	.	4
AAAT.ATTT	2	24177542	24177564	5.75	.	.	intergenic	0.258	.	3
ACAG.CTGT	2	24203252	24203274	5.75	.	.	intergenic	-0.241	069302 rs7900	2
AT.AT	2	24285149	24285180	16	.	.	intergenic	-0.031	573929 rs7911	2
AAAT.ATTT	2	24410539	24410552	3.5	.	.	intergenic	-0.168	rs699640043	2
AT.AT	2	24525832	24525842	5.5	.	.	intergenic	.	365087 rs7879	2
AT.AT	2	24552785	24552795	5.5	.	.	intergenic	.	.	2

AC.GT	2	24634758	24634768	5.5	.	.	intergenic	.	.	2
AAAC.GTTT	2	24654940	24654967	7	.	.	intergenic	0.152	rs786703297	2
AAC.GTT	2	24663531	24663544	4.66667	.	.	intergenic	0.068	489032 rs7872	2
AT.AT	2	24734911	24734922	6	.	.	intergenic	-0.093	rs788518775	3
AC.GT	2	24906681	24906693	6.5	.	.	intergenic	.	rs704436561	2
AG.CT	2	24936461	24936472	6	.	.	intergenic	-0.077	.	2
AAAC.GTTT	2	25136303	25136319	4.25	.	.	intergenic	-0.016	rs692357142	2
AAAT.ATTT	2	25346831	25346852	5.5	.	.	intergenic	-0.125	555484 rs7885	3
AAAC.GTTT	2	25429863	25429886	6	.	.	intergenic	-0.298	454862 rs7892	2
AAAG.CTTT	2	25660320	25660338	4.75	.	.	intergenic	0.119	rs790127470	2
AT.AT	2	25727327	25727337	5.5	.	.	intergenic	-0.069	022542 rs7871	2
AAAC.GTTT	2	25762968	25762990	5.75	.	.	intergenic	.	.	2
AG.CT	2	25768757	25768774	9	.	.	intergenic	.	rs790042714	2
AG.CT	2	25856645	25856654	5	.	.	intergenic	-0.039	.	3
AAT.ATT	2	25902366	25902382	5.66667	.	.	intergenic	-0.025	.	2
AAC.GTT	2	25907414	25907437	8	.	.	intergenic	-0.006	331780 rs7906	3
AATT.AATT	2	25953715	25953733	4.75	.	.	intergenic	.	rs787936285	2
AAC.GTT	2	26012704	26012720	5.66667	.	.	intergenic	.	579476 rs7931	2
AAAC.GTTT	2	26048168	26048190	5.75	.	.	intergenic	-0.046	017986 rs7902	2
ACAT.ATGT	2	26234987	26235017	7.75	.	.	intergenic	0.069	772865 rs7869	2
AAC.GTT	2	26817143	26817164	7.33333	ENSSSCT00000014517	PRR5L	intron	.	399738 rs7890	3
AAAAC.GTTTT	2	27390492	27390526	7	ENSSSCT00000014519	LDLRAD3	intron	-0.121	428487 rs7881	2
AAT.ATT	2	27434455	27434469	5	ENSSSCT00000014519	LDLRAD3	intron	0.292	.	3
AG.CT	2	27861126	27861138	6.5	.	.	intergenic	-0.061	rs792798150	3
AGAGC.GCTCT	2	27924016	27924041	5.2	ENSSSCT00000029479	PAMR1	intron	-0.154	rs787637939	2
AC.GT	2	27973818	27973830	6.5	.	.	intergenic	0.183	rs694136434	3
AG.CT	2	28166613	28166627	7.5	ENSSSCT00000035454	CD44	intron	.	rs791279472	2
AG.CT	2	28166613	28166627	7.5	ENSSSCT00000034523	CD44	intron	.	rs791279472	2
AG.CT	2	28166613	28166627	7.5	ENSSSCT00000036240	CD44	intron	.	rs791279472	2
AG.CT	2	28166613	28166627	7.5	ENSSSCT00000034695	CD44	intron	.	rs791279472	2
AG.CT	2	28166613	28166627	7.5	ENSSSCT00000033911	CD44	intron	.	rs791279472	2
AG.CT	2	28166613	28166627	7.5	ENSSSCT00000033268	CD44	intron	.	rs791279472	2
AG.CT	2	28166613	28166627	7.5	ENSSSCT00000014523	CD44	intron	.	rs791279472	2
AAAC.GTTT	2	28201884	28201898	3.75	ENSSSCT00000033268	CD44	intron	0.405	rs790193027	2
AAAC.GTTT	2	28201884	28201898	3.75	ENSSSCT00000036145	CD44	intron	0.405	rs790193027	2
AAAC.GTTT	2	28201884	28201898	3.75	ENSSSCT00000035454	CD44	intron	0.405	rs790193027	2
AAAC.GTTT	2	28201884	28201898	3.75	ENSSSCT00000034523	CD44	intron	0.405	rs790193027	2
AAAC.GTTT	2	28201884	28201898	3.75	ENSSSCT00000036240	CD44	intron	0.405	rs790193027	2
AAAC.GTTT	2	28201884	28201898	3.75	ENSSSCT00000034695	CD44	intron	0.405	rs790193027	2
AAAC.GTTT	2	28201884	28201898	3.75	ENSSSCT00000035311	CD44	intron	0.405	rs790193027	2
AAAC.GTTT	2	28201884	28201898	3.75	ENSSSCT00000014523	CD44	intron	0.405	rs790193027	2
AAAC.GTTT	2	28201884	28201898	3.75	ENSSSCT00000033911	CD44	intron	0.405	rs790193027	2
AAAC.GTTT	2	28261521	28261544	6	ENSSSCT00000035311	CD44	intron	-0.042	356874 rs7882	2
AAAC.GTTT	2	28268915	28268938	6	ENSSSCT00000035311	CD44	intron	0.036	rs790444986	2
AAAC.GTTT	2	28432012	28432034	5.75	ENSSSCT00000014524	PDHX	intron	0.298	rs789333835	2
AAT.ATT	2	28506563	28506576	4.66667	.	.	intergenic	0.23	299722 rs7929	2
AAAC.GTTT	2	28590772	28590795	6	.	.	intergenic	0.082	412705 rs7862	4
AAAAT.ATTTT	2	28600559	28600575	3.4	.	.	intergenic	-0.055	rs792984128	2
AAGG.CCTT	2	28605494	28605506	3.25	.	.	intergenic	0.733	.	2
AAAC.GTTT	2	28807996	28808017	5.5	.	.	intergenic	0.046	rs791527196	2
AAAAC.GTTTT	2	28963490	28963509	4	ENSSSCT00000014529	ABTB2	intron	-0.107	325698 rs7917	2
AAACC.GGTTT	2	29059091	29059108	3.6	ENSSSCT00000014529	ABTB2	intron	.	rs710182387	2
AAAC.GTTT	2	29088535	29088576	10.5	ENSSSCT00000014529	ABTB2	intron	0.368	530879 rs7899	2
AG.CT	2	29136023	29136037	7.5	ENSSSCT00000014529	ABTB2	intron	-0.571	rs792778704	2
AG.CT	2	29266329	29266346	9	.	.	intergenic	.	rs788061139	2
AAAAT.ATTTT	2	29429352	29429370	3.8	.	.	intergenic	0.027	581976 rs7903	2
AAG.CTT	2	29623533	29623552	6.66667	ENSSSCT00000014537	KIAA1549L	intron	0.234	rs788784998	2
AAT.ATT	2	29725903	29725921	6.33333	ENSSSCT00000014537	KIAA1549L	intron	0.19	236388 rs7863	2
AG.CT	2	29793891	29793903	6.5	ENSSSCT00000026025	CD59	intron	.	rs790487147	4
AG.CT	2	29892924	29892939	8	.	.	intergenic	0.322	rs792281427	2
ACCCC.GGGGT	2	29966005	29966020	3.2	.	.	intergenic	0.048	rs787748240	2
AAC.GTT	2	29988915	29988942	9.33333	.	.	intergenic	0.136	139091 rs7864	2
AAAC.GTTT	2	30342620	30342634	3.75	ENSSSCT00000029285	DEPDC7	intron	0.865	rs790305084	2
AT.AT	2	30503863	30503874	6	ENSSSCT00000014540	QSER1	intron	0.18	.	4
AT.AT	2	30503863	30503874	6	ENSSSCT00000025799	QSER1	intron	0.18	.	4
AATG.CATT	2	30524868	30524893	6.5	ENSSSCT00000014540	QSER1	intron	0.395	rs788315940	2
AATG.CATT	2	30524868	30524893	6.5	ENSSSCT00000025799	QSER1	intron	0.395	rs788315940	2
AAAC.GTTT	2	30538519	30538541	5.75	.	.	intergenic	0.285	rs791915615	2
AAAAAC.GTTTTT	2	30702670	30702693	4	ENSSSCT00000025907	CCDC73	intron	0.087	rs789310249	2
AG.CT	2	30757108	30757119	6	ENSSSCT00000014541	EIF3M	intron	.	rs791537679	2
AAAT.ATTT	2	30779487	30779509	5.75	.	.	intergenic	-0.099	418464 rs7936	2
AAAC.GTTT	2	30819002	30819025	6	.	.	intergenic	.	.	2
AG.CT	2	30888068	30888084	8.5	.	.	intergenic	0.123	.	3
ATC.GAT	2	31130248	31130272	8.33333	ENSSSCT00000030193	.	intron	0.214	304145 rs7915	2
AG.CT	2	31312275	31312286	6	.	.	intergenic	1.367	rs790625389	2
AAAC.GTTT	2	31717289	31717316	7	ENSSSCT00000031770	ELP4	intron	.	931146 rs7913	2
AAAC.GTTT	2	31717289	31717316	7	ENSSSCT00000014547	ELP4	intron	.	931146 rs7913	2
AAAT.ATTT	2	31955467	31955494	7	.	.	intergenic	-0.024	rs793830208	2
AAAT.ATTT	2	32030446	32030459	3.5	.	.	intergenic	.	rs788668316	3
AAAAAG.CTTTTT	2	32317302	32317327	4.33333	.	.	intergenic	0.229	912825 rs7872	2
AAAAT.ATTTT	2	32420082	32420106	5	.	.	intergenic	0.498	rs786647060	2
AATG.CATT	2	32507862	32507885	6	.	.	intergenic	-0.056	435708 rs7892	2
AAAAC.GTTTT	2	32558602	32558632	6.2	ENSSSCT00000014554	MPPED2	intron	-0.021	rs792325053	2
AAAAC.GTTTT	2	32558602	32558632	6.2	ENSSSCT00000036062	MPPED2	intron	-0.021	rs792325053	2

AAAAC.GTTTT	2	32558602	32558632	6.2	ENSSSCT00000032813	MPPED2	intron	-0.021	rs792325053	2
AAAAC.GTTTT	2	32558602	32558632	6.2	ENSSSCT00000034692	MPPED2	intron	-0.021	rs792325053	2
AAAAC.GTTTT	2	32558602	32558632	6.2	ENSSSCT00000036405	MPPED2	intron	-0.021	rs792325053	2
AAC.GTT	2	32704823	32704836	4.66667	ENSSSCT00000034692	MPPED2	intron	0.168	rs793695934	3
AAC.GTT	2	32704823	32704836	4.66667	ENSSSCT00000014554	MPPED2	intron	0.168	rs793695934	3
AAC.GTT	2	32704823	32704836	4.66667	ENSSSCT00000036405	MPPED2	intron	0.168	rs793695934	3
AAC.GTT	2	32704823	32704836	4.66667	ENSSSCT00000032813	MPPED2	intron	0.168	rs793695934	3
AAC.GTT	2	32727902	32727923	7.33333	.	.	intergenic	-0.225	rs691529408	2
AAG.CTT	2	32838956	32838975	6.66667	.	.	intergenic	0.191	599366 rs7912	2
AG.CT	2	32862319	32862331	6.5	ENSSSCT00000014555	FSHB	intron	1.461	.	2
AATG.CATT	2	32940259	32940295	9.25	.	.	intergenic	0.212	rs790415396	3
AC.GT	2	33131486	33131509	12	.	.	intergenic	0.041	rs786939664	2
AT.AT	2	33155428	33155439	6	.	.	intergenic	0.025	rs792366696	2
AAAG.CTTT	2	33239858	33239893	9	.	.	intergenic	-0.042	)47509 rs7863	3
AAT.ATT	2	33347908	33347928	7	.	.	intergenic	0.008	rs792565862	2
AAAC.GTTT	2	33395755	33395769	3.75	.	.	intergenic	.	rs793333968	2
AAAT.ATTT	2	33405201	33405223	5.75	.	.	intergenic	0.041	.	2
AAAAT.ATTTT	2	33607194	33607210	3.4	.	.	intergenic	-0.024	599628 rs7930	2
AAAT.ATTT	2	33827922	33827952	7.75	.	.	intergenic	.	rs788766892	2
AAAAAC.GTTTT	2	34040144	34040170	4.5	.	.	intergenic	.	rs788831542	2
AG.CT	2	34091900	34091913	7	.	.	intergenic	-0.267	rs793697055	2
ACACAT.ATGTG	2	34140527	34140549	3.83333	.	.	intergenic	0.092	rs791494872	2
AAT.ATT	2	34405037	34405052	5.33333	.	.	intergenic	0.138	rs788557921	2
AAT.ATT	2	34422282	34422304	7.66667	.	.	intergenic	0.041	.	2
AAAAAT.ATTTTT	2	34453428	34453445	3	.	.	intergenic	-0.045	rs789635502	2
AAAC.GTTT	2	34581005	34581023	4.75	.	.	intergenic	.	rs787673377	2
AATC.GATT	2	35109931	35109943	3.25	.	.	intergenic	0.106	rs788359591	2
AC.GT	2	35156922	35156932	5.5	.	.	intergenic	-0.07	.	2
AAAC.GTTT	2	35379982	35379996	3.75	.	.	intergenic	.	rs786377814	2
AAT.ATT	2	35402151	35402163	4.33333	.	.	intergenic	0.209	rs791809524	2
AAAC.GTTT	2	35697375	35697389	3.75	.	.	intergenic	-0.036	rs788836812	2
AAAG.CTTT	2	35704437	35704448	3	.	.	intergenic	0.275	rs787300903	3
AT.AT	2	35737512	35737521	5	.	.	intergenic	-0.012	rs788084764	2
AAAT.ATTT	2	36004194	36004220	6.75	.	.	intergenic	.	rs789836927	3
ACATAT.ATATG	2	36326977	36327000	4	ENSSSCT00000014567	ANO3	intron	-0.12	rs793427155	2
AT.AT	2	36439351	36439362	6	.	.	intergenic	.	.	3
AAAC.GTTT	2	36470508	36470522	3.75	.	.	intergenic	0.034	rs786450669	2
AT.AT	2	36476051	36476063	6.5	.	.	intergenic	-0.064	rs792845746	2
AAAC.GTTT	2	36530541	36530562	5.5	.	.	intergenic	-0.012	710620 rs7868	2
AAAT.ATTT	2	36749498	36749524	6.75	.	.	intergenic	.	rs787612584	2
AT.AT	2	36765193	36765208	8	.	.	intergenic	0.001	rs788153250	2
AAAT.ATTT	2	36783478	36783503	6.5	.	.	intergenic	.	rs790105892	2
AT.AT	2	36784628	36784639	6	.	.	intergenic	-0.087	rs790419897	3
AAAT.ATTT	2	37033928	37033941	3.5	.	.	intergenic	.	)85189 rs7874	2
AT.AT	2	37082758	37082772	7.5	.	.	intergenic	.	rs792761297	4
AAAAAC.GTTTT	2	37096764	37096786	3.83333	.	.	intergenic	0.057	739848 rs7925	2
AAAAC.GTTTT	2	37119786	37119803	3.6	.	.	intergenic	-0.002	rs708161570	2
ACATAT.ATATG	2	37150451	37150496	7.66667	.	.	intergenic	-0.092	)88792 rs7891	2
AC.GT	2	37371445	37371463	9.5	.	.	intergenic	0.051	.	2
ACAT.ATGT	2	37444458	37444470	3.25	.	.	intergenic	.	213434 rs7894	3
AGAT.ATCT	2	37462893	37462904	3	.	.	intergenic	.	383437 rs7906	2
AAAT.ATTT	2	37470219	37470246	7	.	.	intergenic	-0.001	497394 rs7906	2
AC.GT	2	37530338	37530351	7	.	.	intergenic	-0.02	rs792809735	2
AAT.ATT	2	37573681	37573696	5.33333	ENSSSCT00000014569	LUZP2	intron	-0.112	rs787776695	2
AG.CT	2	37665238	37665256	9.5	ENSSSCT00000014569	LUZP2	intron	0.295	528994 rs7890	3
AG.CT	2	37779684	37779693	5	ENSSSCT00000014569	LUZP2	intron	-0.025	rs788346278	2
AAAG.CTTT	2	37892566	37892578	3.25	ENSSSCT00000014569	LUZP2	intron	-0.458	rs788624577	2
AC.GT	2	38136185	38136195	5.5	.	.	intergenic	-0.091	rs792350004	2
AAAC.GTTT	2	38257734	38257750	4.25	.	.	intergenic	-0.125	)28919 rs7882	2
AG.CT	2	38505591	38505608	9	.	.	intergenic	-0.021	769428 rs7925	2
AT.AT	2	38629598	38629607	5	.	.	intergenic	0.111	rs787284534	2
ACAG.CTGT	2	38640877	38640894	4.5	.	.	intergenic	.	.	2
AAGG.CCTT	2	38648511	38648556	11.5	.	.	intergenic	0.3	427019 rs7920	2
AT.AT	2	38654604	38654613	5	.	.	intergenic	-0.123	.	3
AAAC.GTTT	2	38696303	38696325	5.75	.	.	intergenic	0.034	rs793155632	2
AAT.ATT	2	38880844	38880858	5	.	.	intergenic	-0.036	rs789757604	4
AAT.ATT	2	38905770	38905787	6	.	.	intergenic	.	rs786410690	4
AG.CT	2	39213829	39213839	5.5	.	.	intergenic	.	)45122 rs7900	2
AAAAC.GTTTT	2	39567551	39567566	3.2	.	.	intergenic	.	rs787571648	2
AAC.GTT	2	39642026	39642042	5.66667	.	.	intergenic	-0.125	rs787750791	2
AAAAAC.GTTTT	2	39758712	39758730	3.16667	.	.	intergenic	0.089	rs792576376	2
AAAC.GTTT	2	39843336	39843349	3.5	.	.	intergenic	-0.125	rs789904957	2
AC.GT	2	39916704	39916714	5.5	.	.	intergenic	0.709	rs788450267	3
AAAAC.GTTTT	2	40040428	40040470	8.6	.	.	intergenic	-0.067	rs793113403	2
AT.AT	2	40166670	40166680	5.5	.	.	intergenic	-0.157	rs793778328	4
AG.CT	2	40181829	40181843	7.5	.	.	intergenic	-0.118	.	4
AAAT.ATTT	2	40205395	40205416	5.5	.	.	intergenic	-0.09	298686 rs7907	2
AT.AT	2	40259263	40259286	12	.	.	intergenic	0.055	rs788231479	2
AGAT.ATCT	2	40387860	40387890	7.75	ENSSSCT00000014572	ANO5	intron	0.107	rs787298168	2
AGAT.ATCT	2	40387860	40387890	7.75	ENSSSCT00000028769	ANO5	intron	0.107	rs787298168	2
AG.CT	2	40405351	40405381	15.5	ENSSSCT00000014572	ANO5	intron	0.187	rs786325683	2
AG.CT	2	40405351	40405381	15.5	ENSSSCT00000028769	ANO5	intron	0.187	rs786325683	2
AG.CT	2	40486040	40486055	8	.	.	intergenic	-0.191	rs787430733	2
AAT.ATT	2	40572490	40572502	4.33333	.	.	intergenic	0.061	.	2

AC.GT	2	40625753	40625763	5.5	.	.	intergenic	-0.036	.	3
ATC.GAT	2	40674676	40674692	5.66667	.	.	intergenic	-0.028	rs786229736	2
AAAAC.GTTTT	2	40751805	40751823	3.8	.	.	intergenic	.	.	2
AC.GT	2	40811274	40811288	7.5	.	.	intergenic	0.698	rs793061745	3
AG.CT	2	41154823	41154835	6.5	.	.	intergenic	.	rs788289162	2
AAAT.ATTT	2	41195938	41195955	4.5	.	.	intergenic	.	rs786734320	2
AAC.GTT	2	41238094	41238115	7.33333	.	.	intergenic	0.013	rs787827068	2
AAAT.ATTT	2	41301714	41301726	3.25	.	.	intergenic	-0.178	rs786967672	2
AAAAT.ATTTT	2	41378157	41378183	5.4	.	.	intergenic	-0.006	rs790486830	2
AC.GT	2	41599921	41599933	6.5	.	.	intergenic	0.225	rs788934997	3
AAAAC.GTTTT	2	41856117	41856134	3.6	ENSSSCT00000014576	PRMT3	intron	.	)69646 rs7896	2
AAAG.CTTT	2	42032482	42032500	4.75	.	.	intergenic	.	325060 rs7887	2
AC.GT	2	42564049	42564059	5.5	.	.	intergenic	-0.147	rs790698205	2
AC.GT	2	42627269	42627296	14	.	.	intergenic	-0.035	rs787512496	2
\AAACC.GGTTT	2	42804420	42804442	3.83333	.	.	intergenic	-0.024	rs793445815	2
\AAAAC.GTTTT	2	42950058	42950081	4	.	.	intergenic	.	rs789930393	2
AC.GT	2	43048637	43048651	7.5	.	.	intergenic	-0.02	536561 rs7926	2
AATG.CATT	2	43267331	43267360	7.5	ENSSSCT00000014585	CSRP3	intron	0.007	rs790169900	2
AAAC.GTTT	2	43328309	43328323	3.75	.	.	intergenic	.	rs790549467	2
AAC.GTT	2	43328968	43328983	5.33333	.	.	intergenic	.	rs787369898	2
AC.GT	2	43511471	43511481	5.5	ENSSSCT00000014589	PTPN5	intron	-0.058	rs696679403	2
AAC.GTT	2	43646389	43646419	10.3333	ENSSSCT00000014593	UEVLD	intron	0.565	491926 rs7916	2
AATT.AATT	2	43705603	43705616	3.5	ENSSSCT00000014593	UEVLD	intron	.	491773 rs7932	3
AATT.AATT	2	43705603	43705616	3.5	ENSSSCT00000014594	LDHC	intron	.	491773 rs7932	3
AT.AT	2	43909693	43909704	6	.	.	intergenic	0.035	rs791904772	2
AT.AT	2	44031330	44031344	7.5	.	.	intergenic	0.054	rs701407757	2
AACAC.GTGTT	2	44072224	44072240	3.4	.	.	intergenic	.	.	2
AAAC.GTTT	2	44120814	44120843	7.5	.	.	intergenic	0.058	rs787786485	2
AAAC.GTTT	2	45022680	45022702	5.75	ENSSSCT00000024535	PIK3C2A	intron	.	302445 rs7929	2
AG.CT	2	45105155	45105164	5	.	.	intergenic	.	rs789462345	3
AAAC.GTTT	2	45116547	45116563	4.25	.	.	intergenic	.	rs793838474	2
\AAAAG.CTTTT	2	45281467	45281486	3.33333	ENSSSCT00000014616	PLEKHA7	intron	.	522475 rs7935	2
ACAT.ATGT	2	45640309	45640327	4.75	ENSSSCT00000024179	.	intron	0.252	rs788116404	2
AAG.CTT	2	46107305	46107326	7.33333	ENSSSCT00000014618	.	intron	0.768	181596 rs7906	2
AG.CT	2	46456566	46456577	6	.	.	intergenic	-0.015	.	2
AAAT.ATTT	2	46515113	46515133	5.25	.	.	intergenic	.	rs787915949	2
AG.CT	2	46541834	46541845	6	.	.	intergenic	-0.07	128283 rs7878	2
AAAAC.GTTTT	2	46596400	46596419	4	.	.	intergenic	-0.068	.	2
AG.CT	2	46774957	46774967	5.5	.	.	intergenic	0.063	792247 rs7905	3
AG.CT	2	47099299	47099310	6	ENSSSCT00000014620	INSC	intron	0.046	rs786889743	3
AAAC.GTTT	2	47114583	47114605	5.75	ENSSSCT00000014620	INSC	intron	0.254	rs789422308	2
AAAC.GTTT	2	47311358	47311377	5	.	.	intergenic	.	292347 rs7889	2
AAAAC.GTTTT	2	47325177	47325193	3.4	.	.	intergenic	.	rs787021365	2
AAAT.ATTT	2	47419036	47419054	4.75	.	.	intergenic	.	rs792640731	2
AAC.GTT	2	47512879	47512895	5.66667	.	.	intergenic	.	rs786335617	2
AC.GT	2	47545433	47545445	6.5	.	.	intergenic	.	rs786388135	2
AATC.GATT	2	47610137	47610160	6	ENSSSCT00000014624	.	intron	0.033	287644 rs7899	2
AC.GT	2	47776107	47776131	12.5	.	.	intergenic	0.312	rs788035651	2
ATCC.GGAT	2	48428602	48428628	6.75	ENSSSCT00000014630	.	intron	0.203	270787 rs7879	3
AT.AT	2	48574398	48574410	6.5	ENSSSCT00000014630	.	intron	0.257	334126 rs7876	2
AAC.GTT	2	48622204	48622216	4.33333	.	.	intergenic	.	rs788813115	2
AAAC.GTTT	2	49218540	49218562	5.75	.	.	intergenic	0.057	rs787848414	2
AC.GT	2	49584698	49584721	12	.	.	intergenic	0.161	rs790828111	6
AAAC.GTTT	2	50056572	50056587	4	.	.	intergenic	-0.013	223066 rs7890	2
AAC.GTT	2	50222912	50222926	5	.	.	intergenic	-0.106	rs790193322	2
AC.GT	2	50267996	50268006	5.5	.	.	intergenic	1.148	rs787235503	2
AC.GT	2	50330615	50330627	6.5	ENSSSCT00000028332	PARVA	intron	.	rs787080829	2
AAAC.GTTT	2	50979141	50979163	5.75	.	.	intergenic	.	520273 rs7863	2
AAAT.ATTT	2	51474259	51474277	4.75	ENSSSCT00000025073	GALNT18	intron	-0.169	rs787220251	2
AAAT.ATTT	2	51474259	51474277	4.75	ENSSSCT00000014643	GALNT18	intron	-0.169	rs787220251	2
AT.AT	2	51554636	51554649	7	.	.	intergenic	0.624	rs712854257	2
AC.GT	2	51560335	51560346	6	.	.	intergenic	-0.043	566895 rs7905	2
AG.CT	2	51567836	51567846	5.5	.	.	intergenic	-0.003	.	3
AATC.GATT	2	52083250	52083268	4.75	.	.	intergenic	.	128156 rs7865	2
AAGAG.CTCTT	2	52112404	52112425	4.4	.	.	intergenic	0.076	rs786388596	2
AATG.CATT	2	52118452	52118477	6.5	.	.	intergenic	0.263	477519 rs7895	2
ACAT.ATGT	2	52235374	52235385	3	ENSSSCT00000029147	MRVI1	intron	-0.072	rs788682191	2
AAAAC.GTTTT	2	52451644	52451672	5.8	.	.	intergenic	0.169	rs788497566	2
AAAC.GTTT	2	52471314	52471336	5.75	.	.	intergenic	.	rs788678837	2
AAAAT.ATTTT	2	52617510	52617524	3	ENSSSCT00000024151	.	intron	0.42	rs789780879	2
AAC.GTT	2	53145045	53145057	4.33333	ENSSSCT00000014650	SWAP70	intron	0.064	rs787993805	2
AAAC.GTTT	2	53162583	53162602	5	.	.	intergenic	.	rs792264692	4
AAAC.GTTT	2	53386224	53386253	7.5	.	.	intergenic	.	rs791191627	2
AC.GT	2	53427110	53427122	6.5	.	.	intergenic	.	rs790649333	3
AGG.CCT	2	53881510	53881524	5	ENSSSCT00000015283	ARF1	intron	-0.217	rs786621235	2
\AAAAG.CTTTT	2	53912567	53912592	4.33333	ENSSSCT00000031062	WNT3A	intron	0.161	511513 rs7925	2
AAC.GTT	2	54358293	54358330	12.6667	ENSSSCT00000015274	SNAP47	intron	.	rs793263009	2
AAG.CTT	2	55242988	55243001	4.66667	.	.	intergenic	.	rs710751494	3
AG.CT	2	55275921	55275930	5	.	.	intergenic	.	.	3
AAC.GTT	2	55341632	55341651	6.66667	.	.	intergenic	.	rs789257646	2
AC.GT	2	55955790	55955801	6	.	.	intergenic	.	rs791186255	2
AG.CT	2	56014558	56014574	8.5	.	.	intergenic	.	rs789335851	2
AT.AT	2	56019526	56019544	9.5	.	.	intergenic	.	rs789243194	2
AGAT.ATCT	2	56158733	56158770	9.5	.	.	intergenic	.	rs790973178	2

AT.AT	2	56497302	56497312	5.5	.	.	intergenic	.	rs787061884	2
AT.AT	2	56539320	56539332	6.5	.	.	intergenic	.	rs788941583	2
AAT.ATT	2	56593405	56593420	5.33333	.	.	intergenic	.	rs793160310	2
AG.CT	2	56603842	56603855	7	.	.	intergenic	.	.	3
AAT.ATT	2	57242059	57242071	4.33333	.	.	intergenic	.	.	3
AAAT.ATTT	2	57278845	57278858	3.5	.	.	intergenic	.	rs792690849	2
AAC.GTT	2	57837341	57837352	4	.	.	intergenic	.	rs791301945	2
AAAC.GTTT	2	57850914	57850928	3.75	.	.	intergenic	.	rs789979765	2
AAAG.CTTT	2	57946144	57946172	7.25	.	.	intergenic	.	rs793064963	2
AC.GT	2	57961998	57962007	5	ENSSSCT00000015225	ZNF496	intron	.	.	4
AT.AT	2	58137607	58137617	5.5	.	.	intergenic	.	.	2
AC.GT	2	58591674	58591688	7.5	.	.	intergenic	0.228	rs792840493	2
AAAC.GTTT	2	59053444	59053455	3	ENSSSCT00000015192	KXD1	intron	0.005	rs787380602	2
AAAC.GTTT	2	59295349	59295364	4	ENSSSCT00000015180	IL12RB1	intron	.	rs793250804	2
AC.GT	2	59322555	59322565	5.5	.	.	intergenic	-0.07	rs788989673	2
AG.CT	2	59447052	59447070	9.5	ENSSSCT00000015176	SLC5A5	intron	.	rs789009860	2
AAAC.GTTT	2	59799624	59799636	3.25	ENSSSCT00000028010	.	3'utr	1.088	rs788385826	2
AT.AT	2	60114810	60114820	5.5	.	.	intergenic	.	138374 rs7873	4
AAAC.GTTT	2	60118555	60118577	5.75	.	.	intergenic	.	rs792384477	2
AATG.CATT	2	60284809	60284838	7.5	ENSSSCT00000015149	CPAMD8	intron	0.058	376571 rs7899	2
AT.AT	2	60422066	60422083	9	.	.	intergenic	.	753160 rs7872	2
AAAG.CTTT	2	60543235	60543261	6.75	.	.	intergenic	0.241	rs786376311	2
AT.AT	2	60594789	60594799	5.5	.	.	intergenic	.	rs793708030	2
AAAAC.GTTTT	2	60729494	60729510	3.4	ENSSSCT00000015138	C19orf44	intron	.	rs786724761	2
AG.CT	2	60901498	60901512	7.5	.	.	intergenic	.	rs791299181	2
AC.GT	2	61028193	61028211	9.5	ENSSSCT00000015134	FAM32A	promoter	.	rs791655726	3
AAAT.ATTT	2	61028990	61029008	4.75	.	.	intergenic	.	rs791339420	2
AAAAAT.ATTTTT	2	61037610	61037631	3.66667	.	.	intergenic	.	rs793627286	2
AC.GT	2	61126632	61126644	6.5	.	.	intergenic	.	rs787155538	3
ACC.GGT	2	61169672	61169684	4.33333	ENSSSCT00000015127	TPM4	intron	0.051	rs790206471	3
AG.CT	2	61441715	61441725	5.5	ENSSSCT00000022303	.	intron	.	rs786532873	2
ATC.GAT	2	61480316	61480342	9	ENSSSCT00000022303	.	intron	.	rs791914585	3
AATG.CATT	2	61606832	61606853	5.5	ENSSSCT00000024967	CYP4F22	intron	0.075	rs793488279	2
AAAC.GTTT	2	61855007	61855018	3	ENSSSCT00000027972	BRD4	intron	-0.161	rs790672362	2
AC.GT	2	62162129	62162138	5	ENSSSCT00000015111	SLC1A6	intron	0.062	.	3
AAAAC.GTTTT	2	62544014	62544036	4.6	.	.	intergenic	.	032589 rs7915	2
AAAT.ATTT	2	62835454	62835474	5.25	.	.	intergenic	.	rs786491684	2
AG.CT	2	62861442	62861463	11	.	.	intergenic	.	rs787770743	2
AT.AT	2	63268091	63268112	11	.	.	intergenic	.	241598 rs7900	2
AAAAG.CTTTT	2	63929508	63929525	3.6	.	.	intergenic	.	.	2
AT.AT	2	64039361	64039370	5	.	.	intergenic	.	rs791549002	2
AG.CT	2	64123205	64123215	5.5	.	.	intergenic	.	rs786331144	2
AT.AT	2	64331690	64331702	6.5	.	.	intergenic	.	rs789442224	2
AAAAAC.GTTTTT	2	64832114	64832140	4.5	ENSSSCT00000015062	.	intron	.	355377 rs7877	2
AG.CT	2	64834514	64834524	5.5	ENSSSCT00000015062	.	intron	.	183146 rs7916	2
AAAC.GTTT	2	65212869	65212891	5.75	ENSSSCT00000015055	.	promoter	.	rs791440616	2
AC.GT	2	65443727	65443745	9.5	.	.	intergenic	.	rs792160984	2
AAAT.ATTT	2	65496147	65496161	3.75	.	.	intergenic	.	rs786821994	2
AC.GT	2	65875674	65875696	11.5	.	.	intergenic	.	rs789978101	4
AAAC.GTTT	2	66178260	66178281	5.5	.	.	intergenic	.	325560 rs7903	2
AAAC.GTTT	2	66637580	66637593	3.5	ENSSSCT00000015002	ASNA1	intron	.	.	2
AAAG.CTTT	2	66667644	66667663	5	ENSSSCT00000015000	TNPO2	3'utr	1.078	rs787238318	2
AAAC.GTTT	2	66747749	66747764	4	.	.	intergenic	.	rs788584157	2
AC.GT	2	66888190	66888204	7.5	.	.	intergenic	.	rs791266499	3
AAAC.GTTT	2	67231815	67231833	4.75	ENSSSCT00000014979	MUC16	intron	.	rs792249357	2
AAAC.GTTT	2	67231815	67231833	4.75	ENSSSCT00000022428	MUC16	intron	.	rs792249357	2
AT.AT	2	67294389	67294399	5.5	.	.	intergenic	0.088	rs792228653	2
AAAT.ATTT	2	67320131	67320156	6.5	.	.	intergenic	.	708952 rs7935	2
AATG.CATT	2	67448503	67448524	5.5	.	.	intergenic	.	rs792794286	2
AAC.GTT	2	68116064	68116091	9.33333	.	.	intergenic	.	015772 rs7889	2
AAAT.ATTT	2	68366008	68366020	3.25	.	.	intergenic	.	rs790759385	2
AC.GT	2	68412365	68412379	7.5	.	.	intergenic	.	rs790725095	2
AT.AT	2	68442894	68442904	5.5	.	.	intergenic	.	.	2
AAAC.GTTT	2	68496078	68496091	3.5	ENSSSCT00000023717	.	promoter	.	314361 rs7912	2
AAAC.GTTT	2	68496078	68496091	3.5	ENSSSCT00000031182	.	promoter	.	314361 rs7912	2
AATC.GATT	2	69370487	69370521	8.75	.	.	intergenic	.	361812 rs7899	2
AAC.GTT	2	69704861	69704880	6.66667	ENSSSCT00000014905	ATG4D	promoter	.	rs792223697	2
AT.AT	2	70108600	70108609	5	.	.	intergenic	.	rs790254636	2
AAAC.GTTT	2	70179206	70179224	4.75	.	.	intergenic	.	rs791726938	2
AAAAC.GTTTT	2	70554598	70554616	3.8	ENSSSCT00000014868	ELOF1	promoter	.	rs791556973	2
AAAAT.ATTTT	2	70977292	70977318	5.4	.	.	intergenic	.	rs789174305	2
AAAAC.GTTTT	2	71667642	71667672	6.2	.	.	intergenic	.	442309 rs7884	2
AAAC.GTTT	2	71668113	71668135	5.75	.	.	intergenic	.	rs787261715	2
AAG.CTT	2	71873954	71873972	6.33333	.	.	intergenic	.	rs788214667	2
AATT.AATT	2	72172214	72172229	4	ENSSSCT00000014821	PEX11G	intron	-0.266	rs787648135	2
AAAT.ATTT	2	73208255	73208277	5.75	ENSSSCT00000014786	GTF2F1	intron	.	rs790611885	2
AC.GT	2	73330405	73330431	13.5	ENSSSCT00000026229	ACER1	intron	1.208	.	2
AAAT.ATTT	2	73922978	73922992	3.75	ENSSSCT00000014770	SAFB2	intron	-0.257	rs789182128	2
AAAC.GTTT	2	74165839	74165857	4.75	.	.	intergenic	-0.181	rs791105334	2
AAAAC.GTTTT	2	74851196	74851214	3.8	ENSSSCT00000014758	.	promoter	-0.047	095006 rs7894	2
AAAT.ATTT	2	75442059	75442073	3.75	.	.	intergenic	-0.363	705685 rs7894	2
AG.CT	2	75575452	75575475	12	ENSSSCT00000014736	.	intron	-0.273	rs709024084	2
ACC.GGT	2	76097141	76097158	6	.	.	intergenic	0.276	rs791433085	2
AT.AT	2	76154958	76154970	6.5	.	.	intergenic	.	rs790013745	2

AT.AT	2	76214689	76214700	6	.	.	intergenic	.	.	3
AC.GT	2	76438694	76438716	11.5	.	.	intergenic	-0.161	rs788477340	2
AAAC.GTTT	2	76640508	76640526	4.75	.	.	intergenic	-0.394	rs786531180	2
AAT.ATT	2	76726473	76726489	5.66667	ENSSSCT00000031657	TMPRSS9	intron	.	ı69144 rs7870	2
AC.GT	2	77062837	77062848	6	ENSSSCT00000028307	DOT1L	intron	.	rs793213130	2
AC.GT	2	77062837	77062848	6	ENSSSCT00000014701	DOT1L	intron	.	rs793213130	2
AC.GT	2	77301862	77301875	7	.	.	intergenic	.	ɔ37146 rs7921	2
AC.GT	2	78008670	78008683	7	.	.	intergenic	.	rs787562270	2
AT.AT	2	79128389	79128411	11.5	.	.	intergenic	.	rs789808125	2
ACC.GGT	2	79390833	79390854	7.33333	.	.	intergenic	.	ʼ71017 rs7864	2
AAAAC.GTTTT	2	79827599	79827632	6.8	ENSSSCT00000015303	MAPK9	intron	.	.	2
AT.AT	2	80468689	80468702	7	ENSSSCT00000015315	CANX	intron	.	rs789857467	4
ACACC.GGTGT	2	80686084	80686098	3	.	.	intergenic	-0.006	rs792169575	2
AAC.GTT	2	81030289	81030302	4.66667	ENSSSCT00000031469	ZNF354C	intron	.	rs793556170	2
AT.AT	2	81088736	81088752	8.5	.	.	intergenic	.	rs786636418	2
AAAC.GTTT	2	81178626	81178641	4	.	.	intergenic	0.277	rs787730412	2
AAAAT.ATTTT	2	81374445	81374463	3.8	.	.	intergenic	0.259	rs789484955	2
ACAT.ATGT	2	81601829	81601849	5.25	.	.	intergenic	.	rs793072898	2
AAAC.GTTT	2	81626905	81626927	5.75	.	.	intergenic	0.063	rs793437296	2
AATG.CATT	2	81888748	81888764	4.25	ENSSSCT00000015329	RMND5B	intron	0.364	.	2
AATG.CATT	2	81888748	81888764	4.25	ENSSSCT00000015334	FAM193B	intron	0.364	.	2
AAAAAC.GTTTTT	2	81909913	81909939	4.5	ENSSSCT00000015329	RMND5B	intron	.	rs787430004	2
AAAAAC.GTTTTT	2	81909913	81909939	4.5	ENSSSCT00000015333	.	promoter	.	rs787430004	2
AAAT.ATTT	2	81984420	81984433	3.5	ENSSSCT00000015329	RMND5B	intron	.	rs788988498	2
AAAAAC.GTTTTT	2	82143179	82143205	4.5	ENSSSCT00000015341	NSD1	intron	.	rs789750839	2
AC.GT	2	83222756	83222766	5.5	ENSSSCT00000015361	SIMC1	intron	0.221	rs793007928	3
AAAT.ATTT	2	83502688	83502711	6	.	.	intergenic	-0.093	rs792192138	3
AC.GT	2	83701703	83701717	7.5	.	.	intergenic	-0.026	rs792321599	2
AC.GT	2	83918692	83918702	5.5	ENSSSCT00000026067	FCHO2	intron	1.289	ı14250 rs7933	2
AATG.CATT	2	84023259	84023287	7.25	.	.	intergenic	.	rs789452986	2
AAAT.ATTT	2	84046121	84046134	3.5	ENSSSCT00000015367	TMEM174	3'utr	0	rs791594183	2
AAAC.GTTT	2	84089836	84089852	4.25	.	.	intergenic	0.276	ı33570 rs7912	2
AAAAT.ATTTT	2	84253916	84253940	5	.	.	intergenic	-0.173	rs791377758	2
AC.GT	2	84355216	84355226	5.5	.	.	intergenic	-0.155	303880 rs7892	2
AAAT.ATTT	2	84381459	84381480	5.5	.	.	intergenic	0.223	ı38047 rs7917	2
AAAAAT.ATTTTT	2	84932166	84932188	3.83333	.	.	intergenic	0.177	rs793413434	2
AC.GT	2	85161144	85161153	5	.	.	intergenic	0.33	rs690172201	2
AAAC.GTTT	2	85264685	85264704	5	.	.	intergenic	.	rs790850475	2
AG.CT	2	85402032	85402042	5.5	ENSSSCT00000015375	GFM2	intron	.	.	3
AAAC.GTTT	2	85612810	85612829	5	.	.	intergenic	0.024	ı86149 rs7935	2
AG.CT	2	85625340	85625349	5	.	.	intergenic	0.162	rs788862461	2
AAAAC.GTTTT	2	85730335	85730352	3.6	ENSSSCT00000015379	.	promoter	0.216	rs787552926	2
AG.CT	2	85732065	85732079	7.5	ENSSSCT00000015379	.	cds	.	rs792939048	2
AAAC.GTTT	2	85874522	85874545	6	.	.	intergenic	0.366	rs792600814	4
AAAC.GTTT	2	86168138	86168164	6.75	ENSSSCT00000015382	COL4A3BF	intron	0.286	rs789718167	2
AAAC.GTTT	2	86168138	86168164	6.75	ENSSSCT00000026755	COL4A3BF	intron	0.286	rs789718167	2
AC.GT	2	86388142	86388183	21	.	.	intergenic	0.178	rs788988497	3
AG.CT	2	86607526	86607535	5	.	.	intergenic	0.088	.	3
AC.GT	2	86753344	86753354	5.5	.	.	intergenic	0.152	rs788995052	2
AAAAC.GTTTT	2	86766964	86766999	7.2	.	.	intergenic	0.082	ɔ99637 rs7888	2
AT.AT	2	87200724	87200736	6.5	ENSSSCT00000015389	IQGAP2	intron	0.128	rs788381465	2
AAAT.ATTT	2	87225047	87225064	4.5	.	.	intergenic	-0.061	rs788222439	2
AAC.GTT	2	87409879	87409895	5.66667	.	.	intergenic	0.033	ʼ99615 rs7894	2
AAAT.ATTT	2	87421000	87421014	3.75	.	.	intergenic	0.218	rs787795306	2
AC.GT	2	87486794	87486803	5	.	.	intergenic	.	rs792217597	4
AG.CT	2	87790729	87790741	6.5	.	.	intergenic	0.238	rs786452778	2
AC.GT	2	87833451	87833464	7	ENSSSCT00000015399	PDE8B	intron	-0.242	rs788428941	2
AT.AT	2	88186628	88186639	6	.	.	intergenic	0.121	rs791501331	2
AAAT.ATTT	2	88612825	88612840	4	ENSSSCT00000015403	.	intron	0.28	rs788691457	2
AAC.GTT	2	88701669	88701688	6.66667	ENSSSCT00000015403	.	intron	0.131	rs789282523	2
AT.AT	2	89397825	89397839	7.5	.	.	intergenic	.	rs793638485	2
AAAC.GTTT	2	89530464	89530486	5.75	ENSSSCT00000015413	DMGDH	intron	0.239	rs793300985	2
AC.GT	2	89574345	89574357	6.5	ENSSSCT00000015413	DMGDH	intron	0.152	rs789105050	2
AC.GT	2	89574345	89574357	6.5	ENSSSCT00000015408	.	intron	0.152	rs789105050	2
AAC.GTT	2	89584478	89584494	5.66667	ENSSSCT00000015413	DMGDH	intron	0.043	rs787799650	2
AAAC.GTTT	2	89842459	89842492	8.5	.	.	intergenic	0.149	rs789264074	2
AAAT.ATTT	2	90112473	90112491	4.75	.	.	intergenic	0.005	ʼ36607 rs7910	2
AAGG.CCTT	2	90216853	90216869	4.25	ENSSSCT00000024422	.	intron	.	rs787871955	2
AAC.GTT	2	90235938	90235953	5.33333	.	.	intergenic	.	ł25062 rs7878	2
AG.CT	2	90394059	90394074	8	ENSSSCT00000015420	.	intron	0.272	ł24999 rs7930	2
AATG.CATT	2	90413912	90413928	4.25	.	.	intergenic	0.2	rs790820804	2
AG.CT	2	90515084	90515094	5.5	ENSSSCT00000015421	.	intron	0.381	.	4
AAAC.GTTT	2	90599333	90599347	3.75	.	.	intergenic	0.053	ʼ94935 rs7904	2
AT.AT	2	91204065	91204083	9.5	ENSSSCT00000015430	MSH3	intron	-0.22	rs789622819	2
AC.GT	2	91220433	91220459	13.5	ENSSSCT00000015430	MSH3	intron	0.067	rs790805030	3
AAACT.AGTTT	2	91313187	91313202	3.2	.	.	intergenic	0.128	rs792439605	2
AG.CT	2	91719240	91719250	5.5	.	.	intergenic	0.198	.	4
AAC.GTT	2	91849064	91849078	5	.	.	intergenic	.	ɔ20705 rs7910	2
AAAC.GTTT	2	91898225	91898244	5	ENSSSCT00000015435	SSBP2	intron	-0.048	rs789921958	2
AAAC.GTTT	2	91898225	91898244	5	ENSSSCT00000023648	SSBP2	intron	-0.048	rs789921958	2
AG.CT	2	92015956	92015967	6	.	.	intergenic	0.275	ɔ60703 rs7932	2
AAAAC.GTTTT	2	92078654	92078683	6	.	.	intergenic	0.191	rs792344482	2
AAAG.CTTT	2	92099944	92099965	5.5	.	.	intergenic	.	.	2
AAAAAC.GTTTTT	2	92334753	92334771	3.16667	.	.	intergenic	0.076	rs790728489	2

AG.CT	2	92794239	92794251	6.5	.	.	intergenic	0.241	503197 rs7905	2
AC.GT	2	92900293	92900305	6.5	.	.	intergenic	0.009	rs788594790	2
AAT.ATT	2	93391063	93391078	5.33333	.	.	intergenic	.	.	3
AT.AT	2	93589051	93589063	6.5	ENSSSCT00000015439	XRCC4	intron	.	243127 rs7893	2
AC.GT	2	93641300	93641310	5.5	.	.	intergenic	0.015	rs788230938	2
ACAT.ATGT	2	93665541	93665567	6.75	.	.	intergenic	0.367	982639 rs7927	2
AAC.GTT	2	93700511	93700533	7.66667	.	.	intergenic	.	750236 rs7882	3
AAAC.GTTT	2	93774260	93774278	4.75	.	.	intergenic	0.019	.	2
AG.CT	2	93780280	93780290	5.5	.	.	intergenic	0.154	.	4
AT.AT	2	93839807	93839817	5.5	ENSSSCT00000032588	VCAN	intron	-0.051	rs789071097	3
AT.AT	2	93839807	93839817	5.5	ENSSSCT00000028327	VCAN	intron	-0.051	rs789071097	3
AT.AT	2	93839807	93839817	5.5	ENSSSCT00000015440	VCAN	intron	-0.051	rs789071097	3
AT.AT	2	93839807	93839817	5.5	ENSSSCT00000035607	VCAN	intron	-0.051	rs789071097	3
AT.AT	2	93839807	93839817	5.5	ENSSSCT00000033743	VCAN	intron	-0.051	rs789071097	3
AT.AT	2	93839807	93839817	5.5	ENSSSCT00000033299	VCAN	intron	-0.051	rs789071097	3
AT.AT	2	93839807	93839817	5.5	ENSSSCT00000031327	VCAN	intron	-0.051	rs789071097	3
AGAGG.CCTCT	2	93992953	93992972	3.33333	.	.	intergenic	-0.132	rs791199710	2
AT.AT	2	94021708	94021722	7.5	.	.	intergenic	-0.119	.	3
AAAC.GTTT	2	94424080	94424098	4.75	.	.	intergenic	-0.101	550815 rs7927	2
AAAAAC.GTTTTT	2	94428271	94428293	3.83333	.	.	intergenic	-0.016	406278 rs7906	2
AAAC.GTTT	2	94504663	94504683	5.25	.	.	intergenic	0.333	.	2
AAAC.GTTT	2	94609070	94609094	6.25	.	.	intergenic	-0.038	971411 rs7918	2
AC.GT	2	94720997	94721011	7.5	.	.	intergenic	-0.002	rs790242543	4
AATT.AATT	2	94944446	94944467	5.5	.	.	intergenic	0.028	rs787313320	2
AT.AT	2	95124201	95124211	5.5	.	.	intergenic	-0.011	rs790856814	2
AAAT.ATTT	2	95594477	95594499	5.75	ENSSSCT00000024141	.	promoter	0.107	504462 rs7923	2
AAAC.GTTT	2	95822457	95822469	3.25	.	.	intergenic	-0.011	484874 rs7895	2
AT.AT	2	95931742	95931751	5	.	.	intergenic	0.541	rs790042797	2
AAAC.GTTT	2	96125485	96125504	5	.	.	intergenic	-0.043	723095 rs7937	2
AC.GT	2	96210295	96210305	5.5	.	.	intergenic	.	596870 rs7906	2
AC.GT	2	96604909	96604918	5	.	.	intergenic	.	rs792073559	2
AAAT.ATTT	2	96615963	96615976	3.5	.	.	intergenic	.	.	2
AT.AT	2	96910853	96910863	5.5	.	.	intergenic	0.292	371775 rs7893	2
AC.GT	2	96921142	96921151	5	.	.	intergenic	0.14	rs793451378	2
AAAT.ATTT	2	96947457	96947479	5.75	.	.	intergenic	-0.003	rs788701061	2
AC.GT	2	97013529	97013539	5.5	.	.	intergenic	0.274	rs789207491	2
AAAC.GTTT	2	97198791	97198810	5	.	.	intergenic	0.082	224432 rs7868	3
AAAAAC.GTTTTT	2	97298433	97298460	4.66667	.	.	intergenic	-0.241	rs789412756	2
AT.AT	2	97473071	97473082	6	ENSSSCT00000015450	RASA1	intron	0.123	rs792174805	2
AAAT.ATTT	2	97804026	97804043	4.5	.	.	intergenic	-0.001	rs789139482	2
AG.CT	2	98289036	98289046	5.5	ENSSSCT00000015452	TMEM161E	intron	.	.	2
AAAAC.GTTTT	2	98311523	98311538	3.2	ENSSSCT00000015452	TMEM161E	intron	0.178	rs792071667	2
AC.GT	2	98498336	98498346	5.5	.	.	intergenic	0.342	.	3
AAAC.GTTT	2	98570887	98570899	3.25	.	.	intergenic	0.25	214674 rs7927	2
AT.AT	2	98584984	98584993	5	.	.	intergenic	0.021	rs789813112	2
AAAAC.GTTTT	2	98863087	98863110	4.8	ENSSSCT00000015454	MEF2C	intron	0.04	rs792802118	2
AAAAC.GTTTT	2	98863087	98863110	4.8	ENSSSCT00000015453	MEF2C	intron	0.04	rs792802118	2
AC.GT	2	98937453	98937469	8.5	.	.	intergenic	1.648	.	3
AT.AT	2	99683970	99683987	9	.	.	intergenic	-0.101	rs786503094	2
AAAG.CTTT	2	99982389	99982421	8.25	.	.	intergenic	-0.029	239713 rs7880	2
AT.AT	2	100350884	100350894	5.5	.	.	intergenic	.	rs786782754	2
AAAG.CTTT	2	100683540	100683552	3.25	.	.	intergenic	0.252	.	3
AT.AT	2	101240370	101240380	5.5	.	.	intergenic	0.076	729622 rs7915	3
AC.GT	2	101250220	101250231	6	.	.	intergenic	0.053	rs787350983	4
AT.AT	2	101263298	101263307	5	.	.	intergenic	.	rs787642481	3
AAAAC.GTTTT	2	101338989	101339010	4.4	.	.	intergenic	.	401250 rs7925	2
AAAC.GTTT	2	102559677	102559693	4.25	.	.	intergenic	.	rs787459404	2
AAAAT.ATTTT	2	102646860	102646877	3.6	.	.	intergenic	0.456	234453 rs7923	2
AC.GT	2	102738465	102738483	9.5	.	.	intergenic	.	rs788734592	2
AAT.ATT	2	102808990	102809003	4.66667	.	.	intergenic	-0.011	rs788814048	2
AGGG.CCCT	2	102865467	102865481	3.75	.	.	intergenic	0.134	rs786257739	2
AAAT.ATTT	2	102882358	102882372	3.75	.	.	intergenic	0.184	062436 rs7905	2
AAT.ATT	2	103418876	103418890	5	.	.	intergenic	0.412	rs790498589	3
AC.GT	2	103730279	103730289	5.5	.	.	intergenic	0.475	.	4
AT.AT	2	104286012	104286030	9.5	.	.	intergenic	0.82	.	4
AC.GT	2	104476342	104476355	7	ENSSSCT00000015467	.	intron	.	rs791470938	2
AC.GT	2	104479047	104479058	6	ENSSSCT00000015467	.	intron	.	rs791629040	2
AT.AT	2	104518416	104518427	6	ENSSSCT00000015467	.	intron	.	rs793661848	2
AG.CT	2	104780372	104780381	5	ENSSSCT00000015470	KIAA0825	intron	.	.	2
AAC.GTT	2	104812279	104812297	6.33333	ENSSSCT00000015470	KIAA0825	intron	0.189	rs789456230	2
AAC.GTT	2	104851540	104851558	6.33333	ENSSSCT00000015471	.	intron	.	rs786734939	2
AG.CT	2	105072714	105072740	13.5	.	.	intergenic	0.055	rs793481303	3
AT.AT	2	105146841	105146850	5	ENSSSCT00000015473	.	intron	0.199	rs790705430	2
AC.GT	2	105423469	105423478	5	.	.	intergenic	.	.	3
AAT.ATT	2	105429885	105429899	5	.	.	intergenic	.	.	2
AAAAC.GTTTT	2	105447799	105447813	3	.	.	intergenic	.	rs789099024	2
AAAAG.CTTTT	2	105576845	105576866	4.4	.	.	intergenic	.	rs790369614	2
AAAAAC.GTTTTT	2	106289958	106289977	3.33333	.	.	intergenic	.	rs789590506	2
AAAG.CTTT	2	106295940	106295966	6.75	.	.	intergenic	.	rs786528622	2
AC.GT	2	106356978	106356988	5.5	.	.	intergenic	.	.	3
AT.AT	2	106377861	106377871	5.5	.	.	intergenic	.	286416 rs7904	2
AAAT.ATTT	2	106944030	106944050	5.25	ENSSSCT00000015480	CAST	intron	-0.066	rs786874369	2
AAAT.ATTT	2	106944030	106944050	5.25	ENSSSCT00000015481	CAST	intron	-0.066	rs786874369	2
AAAC.GTTT	2	106976745	106976769	6.25	ENSSSCT00000015480	CAST	intron	0.107	rs789069165	2

AAAC.GTTT	2	106976745	106976769	6.25	ENSSSCT00000015481	CAST	intron	0.107	rs789069165	2
AAAAG.CTTTT	2	106998233	106998251	3.8	ENSSSCT00000015480	CAST	intron	0.236	.	2
AAAAG.CTTTT	2	106998233	106998251	3.8	ENSSSCT00000015481	CAST	intron	0.236	.	2
AAC.GTT	2	107410062	107410075	4.66667	ENSSSCT00000015485	LNPEP	intron	0.179	rs789503939	2
AT.AT	2	107448433	107448462	15	ENSSSCT00000015485	LNPEP	intron	0.141	.	2
AC.GT	2	107490885	107490898	7	.	.	intergenic	.	rs793469179	4
AC.GT	2	107767633	107767646	7	.	.	intergenic	0.141	.	3
AAAT.ATTT	2	108047470	108047491	5.5	.	.	intergenic	.	300352 rs7891	2
AT.AT	2	108050579	108050591	6.5	.	.	intergenic	.	417475 rs7863	2
AT.AT	2	108125273	108125291	9.5	.	.	intergenic	-0.009	rs788003837	2
AT.AT	2	108282136	108282149	7	.	.	intergenic	-0.016	541808 rs7933	2
AATT.AATT	2	108398882	108398894	3.25	.	.	intergenic	-0.108	580442 rs7934	2
AC.GT	2	108403971	108403983	6.5	.	.	intergenic	.	.	3
AAAT.ATTT	2	108461233	108461257	6.25	.	.	intergenic	0.045	rs792574788	2
ACGT.ACGT	2	108508748	108508769	5.5	.	.	intergenic	.	rs793871794	2
AGGGG.CCCCT	2	108614513	108614534	3.66667	.	.	intergenic	.	707191 rs7935	2
AAAAC.GTTTT	2	108783180	108783199	4	.	.	intergenic	0.303	rs787242763	2
ACCT.AGGT	2	108794860	108794886	6.75	.	.	intergenic	-0.01	920711 rs7869	2
AGAT.ATCT	2	108963106	108963127	5.5	.	.	intergenic	0.218	rs791727519	2
AC.GT	2	109196852	109196882	15.5	.	.	intergenic	0.138	rs788153958	3
AAAT.ATTT	2	109203830	109203844	3.75	.	.	intergenic	0.204	rs789438488	2
AAC.GTT	2	109261268	109261281	4.66667	.	.	intergenic	0.025	rs790327634	2
AC.GT	2	109320560	109320580	10.5	.	.	intergenic	.	.	4
AG.CT	2	109326863	109326873	5.5	.	.	intergenic	.	.	2
AT.AT	2	109343225	109343236	6	.	.	intergenic	.	.	3
AAT.ATT	2	109465133	109465144	4	.	.	intergenic	.	rs792273709	2
AAAAAT.ATTTTT	2	109666656	109666678	3.83333	.	.	intergenic	-0.019	.	2
AAC.GTT	2	109844582	109844599	6	.	.	intergenic	0.144	501371 rs7909	2
AC.GT	2	109855069	109855084	8	.	.	intergenic	-0.257	rs787768386	2
AT.AT	2	109911257	109911267	5.5	.	.	intergenic	.	rs788327364	2
AC.GT	2	110005926	110005941	8	.	.	intergenic	0.045	956729 rs7907	2
AAAAC.GTTTT	2	110124328	110124352	5	.	.	intergenic	.	rs786738094	2
AAT.ATT	2	110295032	110295046	5	.	.	intergenic	.	rs792729142	2
AAT.ATT	2	110315482	110315493	4	.	.	intergenic	.	.	2
AT.AT	2	110453019	110453035	8.5	.	.	intergenic	.	rs793153185	2
AAT.ATT	2	110502614	110502642	9.66667	.	.	intergenic	0.156	rs788335240	2
AC.GT	2	110653683	110653700	9	.	.	intergenic	0.204	rs788495580	4
AG.CT	2	110672919	110672929	5.5	.	.	intergenic	.	.	4
AAAT.ATTT	2	110732029	110732045	4.25	.	.	intergenic	0.049	727472 rs7885	2
AAAAC.GTTTT	2	110819680	110819694	3	ENSSSCT00000015492	ST8SIA4	intron	0.148	rs789130615	2
AG.CT	2	110834565	110834575	5.5	.	.	intergenic	-0.058	.	4
AT.AT	2	111187011	111187044	17	.	.	intergenic	.	rs791974147	2
AT.AT	2	111400007	111400026	10	.	.	intergenic	.	rs788637213	2
AAAC.GTTT	2	111401995	111402013	4.75	.	.	intergenic	.	126486 rs7895	2
AC.GT	2	111577881	111577895	7.5	.	.	intergenic	0.121	rs709236214	4
AAAAC.GTTTT	2	111633491	111633520	6	.	.	intergenic	.	rs712449974	2
AAAAAC.GTTTTT	2	111710112	111710144	5.5	.	.	intergenic	.	750976 rs7916	2
AAAC.GTTT	2	112210979	112210993	3.75	.	.	intergenic	0.119	rs792861842	2
ACAG.CTGT	2	112278010	112278041	8	.	.	intergenic	0.153	190365 rs7872	2
AT.AT	2	112630885	112630896	6	ENSSSCT00000023670	.	intron	1.687	rs790445899	4
AAAT.ATTT	2	112674119	112674140	5.5	ENSSSCT00000031964	PPIP5K2	intron	0.428	rs788079085	2
AAATC.GATTT	2	112707383	112707397	3	ENSSSCT00000031964	PPIP5K2	intron	0.857	rs791207217	2
AAAC.GTTT	2	113005023	113005037	3.75	.	.	intergenic	.	167889 rs7862	2
AC.GT	2	113390421	113390431	5.5	.	.	intergenic	0.452	.	3
AAGG.CCTT	2	113469173	113469199	6.75	.	.	intergenic	.	366436 rs7867	2
AAAAAT.ATTTTT	2	113505382	113505400	3.16667	.	.	intergenic	.	rs791835419	2
AAAT.ATTT	2	113533672	113533690	4.75	.	.	intergenic	0.225	300061 rs7933	2
AG.CT	2	113543356	113543368	6.5	.	.	intergenic	-0.001	rs706240333	5
AT.AT	2	113647389	113647408	10	.	.	intergenic	-0.177	.	2
AT.AT	2	113843554	113843565	6	.	.	intergenic	.	rs786461627	2
AAAC.GTTT	2	113870947	113870966	5	.	.	intergenic	.	rs788188893	2
AC.GT	2	114458375	114458387	6.5	.	.	intergenic	0.395	398589 rs7934	2
AAAC.GTTT	2	114596454	114596468	3.75	.	.	intergenic	.	rs789199428	2
AT.AT	2	114638600	114638617	9	.	.	intergenic	0.199	.	2
AAG.CTT	2	114935129	114935143	5	.	.	intergenic	-0.064	043353 rs7898	2
AAT.ATT	2	115160021	115160042	7.33333	.	.	intergenic	0.084	419919 rs7866	2
AT.AT	2	115623023	115623033	5.5	.	.	intergenic	-0.121	rs792364414	4
AAAG.CTTT	2	115670186	115670200	3.75	.	.	intergenic	-0.072	.	2
AAAC.GTTT	2	115692090	115692108	4.75	.	.	intergenic	-0.259	rs790793268	2
AAAC.GTTT	2	115777817	115777840	6	.	.	intergenic	.	213259 rs7864	2
AC.GT	2	116107626	116107646	10.5	.	.	intergenic	-0.136	rs788652233	2
AAAT.ATTT	2	116130021	116130038	4.5	.	.	intergenic	.	203877 rs6958	2
AAAAC.GTTTT	2	116180497	116180517	4.2	.	.	intergenic	.	170123 rs7890	2
AT.AT	2	116212592	116212603	6	.	.	intergenic	0.352	rs789209556	2
AT.AT	2	116366924	116366934	5.5	.	.	intergenic	0.051	rs792500992	2
AAAAC.GTTTT	2	116444040	116444063	4.8	.	.	intergenic	-0.029	790338 rs7890	2
AAC.GTT	2	116775772	116775787	5.33333	.	.	intergenic	0.816	rs787324845	2
AAAC.GTTT	2	116819388	116819406	4.75	.	.	intergenic	0.571	rs793193137	2
AT.AT	2	116826835	116826847	6.5	.	.	intergenic	0.229	.	4
AC.GT	2	116843680	116843695	8	.	.	intergenic	-0.16	rs789878250	2
AG.CT	2	116949338	116949361	12	.	.	intergenic	0.7	rs793869426	3
AAAAC.GTTTT	2	117031815	117031837	4.6	.	.	intergenic	.	rs787514192	2
AT.AT	2	117141521	117141532	6	.	.	intergenic	-0.059	.	2
AAAAG.CTTTT	2	117392477	117392497	4.2	.	.	intergenic	0.023	979412 rs6999	2

AT.AT	2	117640441	117640450	5	.	.	intergenic	1.029	rs790091226	2
AAAAC.GTTTT	2	117970155	117970177	3.83333	.	.	intergenic	0.072	.	2
AACAC.GTGTT	2	118035056	118035076	4.2	.	.	intergenic	0.069	.	2
AT.AT	2	118202850	118202867	9	.	.	intergenic	0.118	rs786486354	2
AAC.GTT	2	118224845	118224858	4.66667	.	.	intergenic	-0.049	735519 rs7863	2
AC.GT	2	118469412	118469422	5.5	.	.	intergenic	0.03	rs791496726	3
AC.GT	2	118469546	118469561	8	.	.	intergenic	0.036	rs793556738	2
AAAC.GTTT	2	118672185	118672215	7.75	ENSSSCT00000024375	.	intron	0.056	rs793197428	2
AATGG.CCATT	2	119040191	119040207	3.4	.	.	intergenic	0.177	rs789752835	2
AT.AT	2	119071434	119071443	5	.	.	intergenic	-0.122	rs786960014	2
AAAT.ATTT	2	119098310	119098326	4.25	.	.	intergenic	0.412	rs793393784	2
AC.GT	2	119121377	119121387	5.5	.	.	intergenic	0.18	rs792550630	3
AAAAC.GTTTT	2	119159753	119159767	3	ENSSSCT00000015510	MAN2A1	intron	-0.165	.	2
AAAAC.GTTTT	2	119166131	119166162	6.4	ENSSSCT00000015510	MAN2A1	intron	1.006	086697 rs7936	2
AT.AT	2	119179390	119179400	5.5	ENSSSCT00000015510	MAN2A1	intron	0.041	rs786908687	2
AAT.ATT	2	119317239	119317251	4.33333	.	.	intergenic	0.252	726433 rs7918	2
AAAC.GTTT	2	119387918	119387954	9.25	.	.	intergenic	0.057	954001 rs7864	2
AC.GT	2	119514160	119514172	6.5	.	.	intergenic	0.113	rs793192548	2
AT.AT	2	119566374	119566391	9	.	.	intergenic	0.065	.	2
AAAT.ATTT	2	119699843	119699860	4.5	.	.	intergenic	.	rs789752809	2
AC.GT	2	120182656	120182670	7.5	.	.	intergenic	0.228	rs791183985	3
AT.AT	2	120665090	120665099	5	.	.	intergenic	0.025	.	2
AAAC.GTTT	2	120689640	120689655	4	.	.	intergenic	1.202	070989 rs7892	2
AAAC.GTTT	2	120695062	120695074	3.25	.	.	intergenic	.	rs789230194	2
AAAAG.CTTTT	2	120754653	120754670	3.6	.	.	intergenic	.	927333 rs7911	2
AAAC.GTTT	2	120837268	120837294	6.75	ENSSSCT00000015517	STARD4	intron	0.168	044825 rs7895	2
AC.GT	2	121219879	121219895	8.5	.	.	intergenic	-0.022	rs793759024	2
AAAC.GTTT	2	121308524	121308556	8.25	ENSSSCT00000015519	.	intron	-0.235	rs788608403	2
AC.GT	2	121399949	121399961	6.5	ENSSSCT00000015522	DCP2	intron	.	.	3
AACAC.GTGTT	2	121444651	121444672	3.66667	.	.	intergenic	.	rs792581952	2
AAAC.GTTT	2	121496287	121496307	5.25	ENSSSCT00000024663	REEP5	intron	0.022	754657 rs7917	2
AAAAC.GTTTT	2	121504373	121504392	4	ENSSSCT00000024663	REEP5	intron	0.141	546399 rs7886	2
AAAC.GTTT	2	121715873	121715886	3.5	.	.	intergenic	-0.071	541914 rs7867	2
AAT.ATT	2	121824381	121824401	7	.	.	intergenic	-0.028	330681 rs7910	2
AAAT.ATTT	2	122120626	122120640	3.75	.	.	intergenic	.	rs787224512	2
AT.AT	2	122654697	122654719	11.5	ENSSSCT00000015529	YTHDC2	intron	-0.523	rs786803952	2
AAAAC.GTTTT	2	123057242	123057259	3.6	.	.	intergenic	.	rs793153801	4
AAAC.GTTT	2	123574647	123574665	4.75	ENSSSCT00000015531	KCNN2	intron	0.066	120795 rs7886	2
AC.GT	2	123610233	123610245	6.5	ENSSSCT00000015531	KCNN2	intron	-0.093	rs792098484	2
AC.GT	2	123865264	123865275	6	.	.	intergenic	0.152	rs791076455	2
AAAC.GTTT	2	124082792	124082806	3.75	.	.	intergenic	-0.007	rs786799441	2
AC.GT	2	124148607	124148625	9.5	ENSSSCT00000032987	TRIM36	intron	0.551	.	4
AC.GT	2	124148607	124148625	9.5	ENSSSCT00000015532	TRIM36	intron	0.551	.	4
AC.GT	2	124148607	124148625	9.5	ENSSSCT00000033618	TRIM36	intron	0.551	.	4
AG.CT	2	124196224	124196234	5.5	.	.	intergenic	-0.06	.	4
AC.GT	2	124522674	124522693	10	.	.	intergenic	-0.103	rs705370534	3
AC.GT	2	124608255	124608271	8.5	.	.	intergenic	0.721	rs789375819	3
AG.CT	2	124672349	124672361	6.5	.	.	intergenic	.	396864 rs7884	2
AT.AT	2	125114712	125114722	5.5	.	.	intergenic	.	.	3
AAAC.GTTT	2	125168380	125168400	5.25	ENSSSCT00000015541	COMMD10	intron	.	057169 rs7902	2
AAAT.ATTT	2	125315456	125315472	4.25	ENSSSCT00000015541	COMMD10	intron	0.113	.	2
AG.CT	2	125334672	125334683	6	ENSSSCT00000015541	COMMD10	3'utr	0.277	380906 rs7876	2
AC.GT	2	125535731	125535743	6.5	.	.	intergenic	-0.015	052396 rs7899	2
AT.AT	2	125539062	125539072	5.5	.	.	intergenic	0.027	rs788402560	2
AAAC.GTTT	2	125674898	125674924	6.75	.	.	intergenic	.	349793 rs7874	2
AT.AT	2	126022949	126022973	12.5	.	.	intergenic	-0.032	rs792901839	2
AC.GT	2	126173053	126173065	6.5	.	.	intergenic	0.013	rs788290583	2
AAAAC.GTTTT	2	126173558	126173577	4	.	.	intergenic	0.213	rs787462176	2
AAC.GTT	2	126179786	126179801	5.33333	.	.	intergenic	0.047	rs792654605	2
AT.AT	2	126179853	126179874	11	.	.	intergenic	0.103	rs790898190	4
AAC.GTT	2	126327480	126327491	4	.	.	intergenic	.	.	3
AAAG.CTTT	2	126620593	126620607	3.75	.	.	intergenic	.	rs786673385	2
AGAT.ATCT	2	126876553	126876569	4.25	.	.	intergenic	0.056	344900 rs7869	2
AAAC.GTTT	2	126933047	126933066	5	.	.	intergenic	-0.089	486249 rs7864	2
AC.GT	2	126992872	126992886	7.5	.	.	intergenic	-0.09	.	3
AAC.GTT	2	127041741	127041759	6.33333	.	.	intergenic	-0.066	rs788319300	3
AAAC.GTTT	2	127234226	127234244	4.75	.	.	intergenic	-0.067	rs790690697	2
AAAC.GTTT	2	127354602	127354626	6.25	.	.	intergenic	-0.17	334456 rs7937	3
AAC.GTT	2	127412442	127412454	4.33333	.	.	intergenic	.	.	3
AG.CT	2	127482934	127482948	7.5	.	.	intergenic	-0.121	.	4
AG.CT	2	127597412	127597426	7.5	.	.	intergenic	-0.133	rs793106507	4
AAAAT.ATTTT	2	127665716	127665738	4.6	.	.	intergenic	-0.146	rs793019019	2
AG.CT	2	127865305	127865325	10.5	.	.	intergenic	-0.091	319676 rs7905	2
AAAAC.GTTTT	2	128473037	128473055	3.8	ENSSSCT00000024571	DMXL1	intron	3.213	.	2
AAAAC.GTTTT	2	128473037	128473055	3.8	ENSSSCT00000031873	DMXL1	intron	3.213	.	2
AAC.GTT	2	128478373	128478386	4.66667	ENSSSCT00000024571	DMXL1	intron	0.21	rs793551464	2
AAC.GTT	2	128478373	128478386	4.66667	ENSSSCT00000031873	DMXL1	intron	0.21	rs793551464	2
AAAC.GTTT	2	128573798	128573826	7.25	.	.	intergenic	0.334	rs790281105	3
AAC.GTT	2	128677501	128677514	4.66667	.	.	intergenic	0.084	rs789591912	2
AAAC.GTTT	2	128708245	128708267	5.75	.	.	intergenic	0.032	459991 rs7862	2
AC.GT	2	128753742	128753752	5.5	ENSSSCT00000025027	HSD17B4	intron	0.124	rs790541007	2
AC.GT	2	128755284	128755294	5.5	ENSSSCT00000025027	HSD17B4	intron	0.312	rs788549768	2
AAAAC.GTTTT	2	128845030	128845048	3.8	.	.	intergenic	0.082	rs792727439	2
AGC.GCT	2	128964866	128964882	5.66667	.	.	intergenic	0.219	rs791019146	2

AAAT.ATTT	2	129408735	129408752	4.5	.	.	intergenic	0.065	rs789052992	2
AAC.GTT	2	129556017	129556030	4.66667	.	.	intergenic	.	rs790281040	2
AC.GT	2	129693736	129693754	9.5	.	.	intergenic	-0.043	rs699670189	2
AC.GT	2	129701984	129702000	8.5	.	.	intergenic	.	rs790872116	5
AT.AT	2	129708046	129708057	6	.	.	intergenic	0.059	rs792458306	2
AAC.GTT	2	129980338	129980357	6.66667	.	.	intergenic	0.174	rs787785534	2
AAAC.GTTT	2	130187670	130187684	3.75	.	.	intergenic	0.022	47271 rs7871	2
AAC.GTT	2	130258738	130258761	8	.	.	intergenic	.	20003 rs7894	4
AAAC.GTTT	2	130269063	130269082	5	.	.	intergenic	-0.033	16880 rs7876	2
AAAT.ATTT	2	130375001	130375023	5.75	.	.	intergenic	.	rs788711845	2
AC.GT	2	130579437	130579448	6	.	.	intergenic	.	rs792860786	2
ACAT.ATGT	2	130658533	130658553	5.25	.	.	intergenic	.	18101 rs7914	2
AAAC.GTTT	2	130790724	130790751	7	.	.	intergenic	0.054	rs793190127	2
AG.CT	2	130878828	130878838	5.5	.	.	intergenic	0.097	.	2
AT.AT	2	130968260	130968282	11.5	ENSSSCT00000015551	ZNF474	intron	.	rs793046460	3
AATC.GATT	2	131004834	131004856	5.75	ENSSSCT00000015551	ZNF474	intron	0.193	rs789209563	2
AC.GT	2	131028832	131028844	6.5	.	.	intergenic	0.03	68330 rs7904	5
AAAC.GTTT	2	131238049	131238072	6	.	.	intergenic	-0.011	.	2
AAAC.GTTT	2	131333140	131333154	3.75	.	.	intergenic	0.239	rs787928283	2
AAAT.ATTT	2	131363091	131363105	3.75	.	.	intergenic	-0.001	rs792152902	2
AC.GT	2	131422643	131422653	5.5	.	.	intergenic	0.289	rs793083329	2
ACT.AGT	2	131616834	131616846	4.33333	ENSSSCT00000022941	SNX24	intron	.	.	2
AAC.GTT	2	132021017	132021035	6.33333	.	.	intergenic	0.007	47030 rs7887	2
AAC.GTT	2	132256822	132256842	7	ENSSSCT00000015559	CEP120	intron	.	rs792433916	2
AAAC.GTTT	2	132270130	132270148	4.75	ENSSSCT00000015559	CEP120	promoter	-0.03	rs788946824	2
AC.GT	2	132284239	132284251	6.5	.	.	intergenic	0.154	.	2
AT.AT	2	132429857	132429867	5.5	ENSSSCT00000015560	CSNK1G3	intron	.	rs793095934	2
AT.AT	2	132509099	132509108	5	.	.	intergenic	.	rs786234465	2
AC.GT	2	132731283	132731299	8.5	.	.	intergenic	0.627	rs787127780	2
AAAT.ATTT	2	132834587	132834606	5	.	.	intergenic	.	rs788475441	2
AG.CT	2	132859201	132859213	6.5	.	.	intergenic	0.611	98701 rs7878	2
AAAG.CTTT	2	132893556	132893578	5.75	.	.	intergenic	.	rs788594393	2
AGAT.ATCT	2	133000604	133000618	3.75	.	.	intergenic	-0.062	rs793361947	2
AC.GT	2	133428616	133428632	8.5	.	.	intergenic	0.057	.	2
AAAC.GTTT	2	133439155	133439167	3.25	.	.	intergenic	0.62	rs787653517	2
AAAAC.GTTTT	2	133692948	133692970	4.6	.	.	intergenic	0.3	.	2
AG.CT	2	133799279	133799291	6.5	.	.	intergenic	-0.004	rs791389286	2
AC.GT	2	133953510	133953520	5.5	.	.	intergenic	0.165	rs792346572	2
AAC.GTT	2	134035022	134035044	7.66667	.	.	intergenic	0.206	rs793574441	3
AT.AT	2	134173099	134173108	5	.	.	intergenic	.	.	3
AAAC.GTTT	2	134210094	134210116	5.75	.	.	intergenic	0.138	38492 rs7916	2
AAAAC.GTTTT	2	134360602	134360630	5.8	.	.	intergenic	0.423	rs789286998	3
AT.AT	2	134404454	134404464	5.5	.	.	intergenic	.	rs697954378	2
AG.CT	2	134856073	134856083	5.5	.	.	intergenic	0.074	.	2
AG.CT	2	134876380	134876390	5.5	.	.	intergenic	0.158	rs790946789	2
AAT.ATT	2	134954672	134954684	4.33333	ENSSSCT00000015567	ALDH7A1	intron	-0.206	.	2
AAAT.ATTT	2	135010207	135010221	3.75	ENSSSCT00000027284	.	promoter	.	rs793780625	2
AAAT.ATTT	2	135010207	135010221	3.75	ENSSSCT00000015564	PHAX	promoter	.	rs793780625	2
AAAAC.GTTTT	2	135040272	135040296	4.16667	.	.	intergenic	.	90454 rs7900	2
AAAC.GTTT	2	135051362	135051379	4.5	.	.	intergenic	-0.176	rs792041744	2
AG.CT	2	135280185	135280200	8	ENSSSCT00000015569	3-Mar	intron	0.042	rs790987669	4
AT.AT	2	135503406	135503415	5	.	.	intergenic	0.16	rs787549409	2
AG.CT	2	135525979	135525990	6	ENSSSCT00000015571	MEGF10	intron	0.157	.	2
AAT.ATT	2	135675469	135675494	8.66667	.	.	intergenic	0.163	rs789366242	2
AT.AT	2	135697971	135697980	5	.	.	intergenic	0.633	rs786483060	2
AG.CT	2	135807598	135807608	5.5	ENSSSCT00000015573	.	intron	0.237	.	2
AT.AT	2	135820037	135820048	6	ENSSSCT00000015573	.	intron	0.079	rs789643106	2
AT.AT	2	135862431	135862442	6	.	.	intergenic	.	rs789299756	3
AT.AT	2	135892277	135892288	6	.	.	intergenic	0.127	rs793047990	2
AC.GT	2	135972378	135972388	5.5	.	.	intergenic	0.15	.	5
AAAT.ATTT	2	136043441	136043468	7	.	.	intergenic	0.136	96195 rs7932	2
ACAT.ATGT	2	136129985	136129999	3.75	.	.	intergenic	.	.	2
AAAAC.GTTTT	2	136276192	136276215	4.8	.	.	intergenic	.	71546 rs7876	2
AAAAC.GTTTT	2	136784372	136784388	3.4	.	.	intergenic	.	rs792637807	2
AC.GT	2	137125465	137125477	6.5	ENSSSCT00000015578	SLC27A6	intron	0.155	rs793052762	3
AAAC.GTTT	2	137280821	137280835	3.75	.	.	intergenic	-0.14	31962 rs7929	2
ACAG.CTGT	2	137348183	137348200	4.5	.	.	intergenic	0.034	rs793696240	2
AT.AT	2	137364402	137364412	5.5	.	.	intergenic	-0.033	rs695934974	2
AG.CT	2	137814855	137814865	5.5	.	.	intergenic	-0.035	rs792619456	2
AAAT.ATTT	2	138210174	138210188	3.75	.	.	intergenic	0.075	rs793813022	2
AAAAC.GTTTT	2	138250854	138250881	5.6	.	.	intergenic	0.033	.	2
AAAC.GTTT	2	138451864	138451879	4	.	.	intergenic	.	.	2
AT.AT	2	138455244	138455255	6	.	.	intergenic	0.241	38521 rs7910	2
AG.CT	2	138581383	138581393	5.5	.	.	intergenic	.	.	2
AAAAT.ATTTT	2	138744956	138744976	4.2	.	.	intergenic	.	92927 rs7915	2
AC.GT	2	138766520	138766529	5	.	.	intergenic	0.164	44027 rs7868	2
AC.GT	2	138773416	138773430	7.5	.	.	intergenic	.	rs693953193	2
AC.GT	2	138796719	138796730	6	.	.	intergenic	0.231	rs788595361	2
AAAAC.GTTTT	2	138840338	138840356	3.8	ENSSSCT00000029538	LYRM7	intron	0.008	60213 rs7918	2
AAAAC.GTTTT	2	138840338	138840356	3.8	ENSSSCT00000015586	LYRM7	intron	0.008	60213 rs7918	2
AAC.GTT	2	138841055	138841069	5	ENSSSCT00000029538	LYRM7	intron	.	rs704753358	2
AAC.GTT	2	138841055	138841069	5	ENSSSCT00000015586	LYRM7	intron	.	rs704753358	2
AC.GT	2	138897038	138897048	5.5	ENSSSCT00000029538	LYRM7	intron	-0.124	.	2
AC.GT	2	138897038	138897048	5.5	ENSSSCT00000015586	LYRM7	intron	-0.124	.	2

AC.GT	2	138965710	138965724	7.5	.	.	intergenic	-0.131	rs789032086	2
AC.GT	2	139033382	139033392	5.5	ENSSSCT00000015589	.	intron	0.069	rs789871950	2
AAAAC.GTTTT	2	139122618	139122641	4.8	ENSSSCT00000015589	.	intron	-0.075	rs786355878	3
AC.GT	2	139162213	139162230	9	.	.	intergenic	.	rs791964182	4
AG.CT	2	139255479	139255493	7.5	.	.	intergenic	0.163	rs789293520	2
AC.GT	2	139288429	139288442	7	ENSSSCT00000015591	.	intron	0.018	;73026 rs7882	2
AAAC.GTTT	2	139601896	139601914	4.75	ENSSSCT00000015595	ACSL6	intron	.	rs786903236	2
AT.AT	2	139785510	139785521	6	.	.	intergenic	.	427631 rs7932	3
AAC.GTT	2	139825125	139825145	7	.	.	intergenic	.	rs791546835	2
AAAT.ATTT	2	139837744	139837765	5.5	.	.	intergenic	0.242	rs788323812	2
AAAC.GTTT	2	140525566	140525580	3.75	ENSSSCT00000015606	KIF3A	intron	.	463192 rs7930	2
AT.AT	2	140539653	140539670	9	ENSSSCT00000015606	KIF3A	promoter	0.165	121994 rs7911	2
AT.AT	2	140805863	140805872	5	ENSSSCT00000015614	AFF4	promoter	0.251	rs790238897	3
AT.AT	2	140805863	140805872	5	ENSSSCT00000025744	AFF4	promoter	0.251	rs790238897	3
ACGC.GCGT	2	141353699	141353721	5.75	.	.	intergenic	0.099	rs793265536	2
AG.CT	2	141556785	141556794	5	.	.	intergenic	0.032	rs788849095	3
AC.GT	2	141579260	141579283	12	.	.	intergenic	-0.135	rs789958594	2
AC.GT	2	141608854	141608873	10	.	.	intergenic	-0.308	181841 rs7863	2
AGAGG.CCTCT	2	141792493	141792513	4.2	.	.	intergenic	-0.231	rs708884046	2
AC.GT	2	141876073	141876096	12	.	.	intergenic	-0.333	.	3
AAC.GTT	2	142127357	142127371	5	.	.	intergenic	0.303	rs713701373	2
AAAT.ATTT	2	142538975	142538998	6	ENSSSCT00000015628	SEC24A	intron	.	rs788718625	2
AAAAG.CTTTT	2	142830941	142830964	4.8	.	.	intergenic	-0.035	rs786737717	2
AT.AT	2	143209588	143209598	5.5	.	.	intergenic	0.131	rs792095743	2
AAT.ATT	2	143211310	143211331	7.33333	.	.	intergenic	0.276	rs791277432	2
AT.AT	2	143563405	143563415	5.5	.	.	intergenic	-0.076	.	2
AAAC.GTTT	2	143635743	143635759	4.25	.	.	intergenic	-0.154	rs792468617	2
ACGC.GCGT	2	143995095	143995108	3.5	ENSSSCT00000015644	TRPC7	intron	0.361	;95737 rs7888	2
AC.GT	2	144158639	144158648	5	.	.	intergenic	0.319	153536 rs7936	2
AC.GT	2	144170404	144170418	7.5	.	.	intergenic	.	rs792282596	3
ACCC.GGGT	2	144268253	144268264	3	.	.	intergenic	0.108	rs793429257	2
AG.CT	2	144404527	144404555	14.5	.	.	intergenic	-0.078	174708 rs7123	3
AAAC.GTTT	2	144611061	144611079	4.75	.	.	intergenic	-0.002	rs789268881	2
AGCCC.GGGCT	2	144783280	144783300	4.2	.	.	intergenic	-0.195	rs791339273	2
AAG.CTT	2	145076775	145076789	5	.	.	intergenic	-0.266	;34500 rs7863	2
AG.CT	2	145129821	145129830	5	.	.	intergenic	1.037	rs787978768	2
AT.AT	2	145152866	145152877	6	.	.	intergenic	0.033	rs792918444	2
AAAG.CTTT	2	145488945	145488959	3.75	.	.	intergenic	0.138	105194 rs7901	2
AC.GT	2	145596330	145596339	5	.	.	intergenic	0.269	rs793844919	2
AC.GT	2	145605825	145605834	5	.	.	intergenic	.	rs788318125	2
AAAC.GTTT	2	145799402	145799418	4.25	.	.	intergenic	0.244	rs786362690	2
AT.AT	2	145809839	145809854	8	.	.	intergenic	.	rs790435166	2
AAAT.ATTT	2	146250761	146250775	3.75	.	.	intergenic	0.195	.	2
AAAG.CTTT	2	146263014	146263039	6.5	.	.	intergenic	0.235	377924 rs7886	2
AAAAAG.CTTTTT	2	146469037	146469062	4.33333	ENSSSCT00000015666	CTNNA1	intron	.	rs789720401	2
AAAAAC.GTTTTT	2	146830501	146830529	4.83333	.	.	intergenic	.	462183 rs7920	2
AC.GT	2	147077974	147077983	5	ENSSSCT00000031093	PAIP2	intron	.	487080 rs7878	2
AAG.CTT	2	147158302	147158318	5.66667	ENSSSCT00000015680	ECSCR	intron	0.134	380032 rs7894	2
AAAC.GTTT	2	148012640	148012661	5.5	ENSSSCT00000023543	PFDN1	intron	1.528	282663 rs7899	2
AAAC.GTTT	2	148097331	148097351	5.25	.	.	intergenic	.	rs792803139	2
AAAAC.GTTTT	2	148166564	148166584	4.2	ENSSSCT00000015694	.	intron	0.214	rs790078730	2
AAAAT.ATTTT	2	148183406	148183425	4	ENSSSCT00000015694	.	intron	0.368	rs789728703	2
AC.GT	2	148275084	148275094	5.5	ENSSSCT00000015698	.	intron	0.358	171441 rs7878	2
AT.AT	2	148695005	148695032	14	ENSSSCT00000030809	PCDHAC2	intron	.	339313 rs7885	2
ACAT.ATGT	2	148790790	148790805	4	.	.	intergenic	0.22	.	2
AAAT.ATTT	2	148834243	148834262	5	.	.	intergenic	.	784597 rs7921	2
AC.GT	2	148919522	148919532	5.5	.	.	intergenic	.	rs791860784	3
AG.CT	2	149317620	149317630	5.5	.	.	intergenic	.	rs789972976	2
AGG.CCT	2	149383998	149384010	4.33333	ENSSSCT00000015724	DIAPH1	intron	.	rs792739653	2
AAAAG.CTTTT	2	149630912	149630931	4	.	.	intergenic	0.29	rs788718474	2
AGG.CCT	2	149716474	149716489	5.33333	.	.	intergenic	.	rs790895960	2
AAAC.GTTT	2	149769243	149769258	4	.	.	intergenic	0.228	rs790533865	2
AAAT.ATTT	2	149778772	149778785	3.5	ENSSSCT00000015732	RNF14	intron	0.105	.	2
AAAC.GTTT	2	149855864	149855880	4.25	.	.	intergenic	0.027	rs787246932	2
AAAAT.ATTTT	2	149864930	149864950	4.2	.	.	intergenic	.	rs792540458	2
AC.GT	2	150049738	150049764	13.5	.	.	intergenic	-0.026	309766 rs7932	2
AC.GT	2	150105304	150105316	6.5	.	.	intergenic	0.088	rs787173741	2
AAC.GTT	2	150405336	150405360	8.33333	ENSSSCT00000028925	FGF1	intron	.	rs792715626	2
AG.CT	2	150460739	150460756	9	.	.	intergenic	.	rs791930500	2
AG.CT	2	150673715	150673724	5	ENSSSCT00000015737	ARHGAP26	intron	-0.004	.	2
AC.GT	2	150725722	150725733	6	ENSSSCT00000015737	ARHGAP26	intron	.	rs702976154	2
AAAT.ATTT	2	150891780	150891798	4.75	ENSSSCT00000015737	ARHGAP26	intron	0.049	rs791046372	2
AT.AT	2	151022782	151022806	12.5	.	.	intergenic	-0.132	rs790245049	2
AG.CT	2	151038713	151038725	6.5	.	.	intergenic	.	rs702573682	4
AAAG.CTTT	2	151044387	151044409	5.75	.	.	intergenic	0.071	223544 rs7863	2
AC.GT	2	151088027	151088040	7	ENSSSCT00000015739	NR3C1	intron	-0.001	rs788892891	3
AATG.CATT	2	151214175	151214194	5	.	.	intergenic	0.162	rs788467673	2
AC.GT	2	151368399	151368416	9	.	.	intergenic	.	791554 rs7931	2
AAAC.GTTT	2	151399182	151399203	5.5	.	.	intergenic	0.06	415995 rs7886	2
AGC.GCT	2	151439511	151439524	4.66667	.	.	intergenic	-0.144	.	2
AG.CT	2	151480480	151480492	6.5	.	.	intergenic	0.028	rs791113093	2
AAAAC.GTTTT	2	151645911	151645936	5.2	.	.	intergenic	.	rs792729605	2
AG.CT	2	151855415	151855424	5	.	.	intergenic	0.215	rs787653918	2
AAG.CTT	2	151987242	151987257	5.33333	.	.	intergenic	0.11	rs791961230	2

AAC.GTT	2	152346649	152346662	4.66667	.	.	intergenic	.	371629 rs7892	2
AAAC.GTTT	2	152383413	152383433	5.25	.	.	intergenic	0.071	rs790968962	3
AAC.GTT	2	152584576	152584594	6.33333	.	.	intergenic	0.496	311435 rs7920	2
AAAC.GTTT	2	152662795	152662809	3.75	.	.	intergenic	-0.094	rs791051157	2
AAAC.GTTT	2	152744597	152744617	5.25	.	.	intergenic	0.034	rs787353989	3
AT.AT	2	152814512	152814522	5.5	.	.	intergenic	0.015	rs792944941	3
AAAC.GTTT	2	153038706	153038723	4.5	.	.	intergenic	.	505424 rs7887	2
AC.GT	2	153236002	153236023	11	.	.	intergenic	-0.086	rs787974668	4
AAATT.AATTT	2	153248215	153248231	3.4	.	.	intergenic	0.078	rs793143874	2
AC.GT	2	153267049	153267060	6	.	.	intergenic	0.208	rs787667300	2
AG.CT	2	153310383	153310401	9.5	.	.	intergenic	0.189	317188 rs7867	2
AG.CT	2	153465533	153465543	5.5	.	.	intergenic	.	rs790502902	3
AAAAC.GTTTT	2	153824447	153824469	4.6	ENSSSCT00000015749	.	intron	.	595187 rs7870	2
AAAAG.CTTTT	2	153839670	153839687	3.6	ENSSSCT00000015749	.	intron	0.743	rs793731080	2
AAAAG.CTTTT	2	153839670	153839687	3.6	ENSSSCT00000029042	.	intron	0.743	rs793731080	2
AAAAG.CTTTT	2	153839670	153839687	3.6	ENSSSCT00000015750	.	intron	0.743	rs793731080	2
AT.AT	2	153958554	153958564	5.5	ENSSSCT00000015746	PLAC8L1	intron	0.104	rs793321156	3
AAT.ATT	2	154110133	154110154	7.33333	.	.	intergenic	.	976295 rs7877	2
AAAC.GTTT	2	154167799	154167821	5.75	ENSSSCT00000015753	.	intron	0.26	251836 rs7896	2
AAAC.GTTT	2	154191069	154191092	6	.	.	intergenic	-0.011	rs786911066	2
AAAC.GTTT	2	154256435	154256449	3.75	.	.	intergenic	.	323982 rs7871	2
AG.CT	2	154266253	154266263	5.5	.	.	intergenic	0.273	rs787124101	3
AAAC.GTTT	2	154322690	154322703	3.5	ENSSSCT00000015755	TCERG1	intron	.	374530 rs7899	3
AAAG.CTTT	2	154340833	154340847	3.75	ENSSSCT00000015755	TCERG1	intron	0.302	rs790086387	2
AG.CT	2	154459482	154459491	5	ENSSSCT00000029561	PPP2R2B	intron	0.002	rs791124767	4
AAC.GTT	2	154739588	154739600	4.33333	.	.	intergenic	-0.277	970453 rs7930	3
AG.CT	2	154864453	154864464	6	.	.	intergenic	0.087	rs705091475	2
AC.GT	2	155208697	155208710	7	.	.	intergenic	-0.12	rs786869060	7
AC.GT	2	155452507	155452521	7.5	.	.	intergenic	-0.012	522545 rs7892	4
ACAT.ATGT	2	155472645	155472658	3.5	.	.	intergenic	0.209	000342 rs7904	2
AC.GT	2	155489766	155489776	5.5	.	.	intergenic	0.899	rs787801380	2
AC.GT	2	155575316	155575329	7	.	.	intergenic	-0.023	532968 rs7869	4
AC.GT	2	155588941	155588951	5.5	.	.	intergenic	.	.	3
AACAT.ATGTT	2	155597403	155597434	6.4	.	.	intergenic	0.262	rs788273394	2
AAAC.GTTT	2	155732994	155733008	3.75	.	.	intergenic	0.116	rs787102492	2
AT.AT	2	156075552	156075561	5	.	.	intergenic	.	rs788140328	2
AC.GT	2	156105206	156105220	7.5	.	.	intergenic	.	916009 rs7925	2
AG.CT	2	156531578	156531592	7.5	ENSSSCT00000015770	HTR4	intron	.	rs791793665	2
AAAC.GTTT	2	156628960	156628974	3.75	.	.	intergenic	.	rs788210107	2
AAG.CTT	2	156674485	156674496	4	.	.	intergenic	.	rs788331031	2
AAAC.GTTT	2	157587920	157587943	6	.	.	intergenic	-0.055	575922 rs7893	2
AAAC.GTTT	2	158579464	158579482	4.75	.	.	intergenic	.	rs787404224	2
AGC.GCT	2	158650368	158650385	6	.	.	intergenic	-0.227	rs791733688	2
AT.AT	2	159104897	159104920	12	.	.	intergenic	.	rs793826477	2
AGAT.ATCT	2	159117674	159117688	3.75	.	.	intergenic	.	.	2
AG.CT	2	159126415	159126426	6	.	.	intergenic	.	rs791231361	3
AG.CT	2	159148556	159148568	6.5	.	.	intergenic	.	rs792282056	3
ATC.GAT	2	159152212	159152229	6	.	.	intergenic	.	rs788712173	2
AAAT.ATTT	2	159280686	159280704	4.75	.	.	intergenic	-0.313	rs789576600	2
AAC.GTT	2	159309955	159309971	5.66667	.	.	intergenic	-0.032	rs790244465	2
AT.AT	2	160211596	160211607	6	.	.	intergenic	.	774187 rs7933	4
ACAT.ATGT	2	160428855	160428867	3.25	.	.	intergenic	.	.	2
AG.CT	2	160438561	160438575	7.5	.	.	intergenic	.	.	2
AC.GT	2	160654682	160654697	8	.	.	intergenic	.	rs788953566	3
AAG.CTT	2	160902748	160902789	14	.	.	intergenic	.	.	2
AT.AT	2	161194690	161194704	7.5	.	.	intergenic	.	rs793506435	4
AAAT.ATTT	2	161196644	161196670	6.75	.	.	intergenic	.	rs793602640	2
AT.AT	2	161277953	161277962	5	.	.	intergenic	.	.	2
AG.CT	2	161284968	161284983	8	.	.	intergenic	.	.	2
AAAC.GTTT	2	161319782	161319812	7.75	.	.	intergenic	.	rs697877376	2
AT.AT	2	161507554	161507563	5	.	.	intergenic	.	481225 rs7913	2
AT.AT	2	161621828	161621839	6	.	.	intergenic	.	rs793083876	2
AAC.GTT	3	421468	421481	4.66667	.	.	intergenic	.	rs792696310	2
AC.GT	3	2070057	2070070	7	.	.	intergenic	.	.	2
AG.CT	3	2252647	2252659	6.5	.	.	intergenic	-0.176	422959 rs7903	2
AAAG.CTTT	3	2381908	2381932	6.25	.	.	intergenic	0.145	rs789902690	2
AG.CT	3	2389474	2389499	13	.	.	intergenic	.	563174 rs7883	2
AAAAG.CTTTT	3	3901535	3901552	3.6	.	.	intergenic	.	.	2
AGGG.CCCT	3	4730915	4730929	3.75	ENSSSCT00000008324	ETA-ACTII	intron	3.528	077865 rs7933	2
AC.GT	3	4992914	4992925	6	.	.	intergenic	.	rs787180760	3
AAAC.GTTT	3	5348211	5348228	4.5	.	.	intergenic	.	rs790199766	2
AAAC.GTTT	3	5588626	5588650	6.25	ENSSSCT00000008342	BAIAP2L1	cds	1.291	.	2
AAAC.GTTT	3	5588626	5588650	6.25	ENSSSCT00000008342	BAIAP2L1	intron	1.291	.	2
AGGG.CCCT	3	5799868	5799892	6.25	.	.	intergenic	0.503	327022 rs7926	2
AAAC.GTTT	3	5936249	5936276	7	.	.	intergenic	-0.057	770928 rs7907	3
AAAT.ATTT	3	7120854	7120867	3.5	ENSSSCT00000008382	AZGP1	promoter	.	912620 rs7901	2
AC.GT	3	7269250	7269259	5	.	.	intergenic	.	.	2
AAAAC.GTTTT	3	7415010	7415037	5.6	.	.	intergenic	.	rs787523280	2
AAAC.GTTT	3	7556314	7556328	3.75	.	.	intergenic	.	rs789508326	2
AAAC.GTTT	3	7567839	7567855	4.25	.	.	intergenic	.	rs792469810	2
AAAG.CTTT	3	8026757	8026769	3.25	.	.	intergenic	-0.079	rs786852086	3
AAC.GTT	3	8108749	8108762	4.66667	ENSSSCT00000008416	EPHB4	intron	0.028	rs788231299	2
AG.CT	3	9536768	9536780	6.5	ENSSSCT00000008425	PRKRIP1	intron	.	rs793549770	4
AAGGG.CCCTT	3	9659541	9659563	4.6	.	.	intergenic	.	rs792378961	2

AAAT.ATTT	3	9664587	9664601	3.75	ENSSSCT00000024358	.	promoter	.	072750 rs7898	2
AATG.CATT	3	10000883	10000901	4.75	.	.	intergenic	.	rs786541958	2
AAAG.CTTT	3	10141694	10141713	5	.	.	intergenic	.	rs786767143	3
AAT.ATT	3	10170258	10170273	5.33333	.	.	intergenic	.	rs790090480	3
AC.GT	3	10563415	10563427	6.5	ENSSSCT00000008451	BCL7B	intron	.	.	2
AT.AT	3	10639362	10639372	5.5	.	.	intergenic	.	017783 rs7918	2
AAAC.GTTT	3	10660585	10660607	5.75	ENSSSCT00000008456	WBSCR22	intron	-0.101	rs792096315	2
AAAC.GTTT	3	10919799	10919817	4.75	.	.	intergenic	-0.305	rs793846557	2
AC.GT	3	10934382	10934408	13.5	ENSSSCT00000032354	.	intron	0.335	367184 rs7887	2
AC.GT	3	10934382	10934408	13.5	ENSSSCT00000025955	LIMK1	promoter	0.335	367184 rs7887	2
AAAT.ATTT	3	10972510	10972526	4.25	.	.	intergenic	.	017798 rs7863	2
AAAAC.GTTTT	3	11044661	11044677	3.4	ENSSSCT00000028458	LAT2	intron	.	999638 rs7918	2
AAAC.GTTT	3	11411341	11411355	3.75	.	.	intergenic	.	rs787738819	2
AAAC.GTTT	3	11483100	11483117	4.5	ENSSSCT00000031896	GTF2I	intron	-0.146	190096 rs7901	3
AAAC.GTTT	3	11483100	11483117	4.5	ENSSSCT00000030268	GTF2I	intron	-0.146	190096 rs7901	3
AAAC.GTTT	3	11483100	11483117	4.5	ENSSSCT00000008466	GTF2I	intron	-0.146	190096 rs7901	3
AAAAT.ATTTT	3	11803008	11803027	4	.	.	intergenic	0.094	rs788260334	2
AG.CT	3	11843728	11843739	6	.	.	intergenic	-0.06	rs791683513	2
AC.GT	3	12083600	12083616	8.5	.	.	intergenic	0.005	267192 rs7933	2
AG.CT	3	12123359	12123370	6	.	.	intergenic	.	.	3
AAAC.GTTT	3	12573637	12573655	4.75	.	.	intergenic	0.282	rs792556219	3
AGGG.CCCT	3	13127848	13127862	3.75	.	.	intergenic	-0.097	131810 rs7906	2
AAAC.GTTT	3	13428765	13428776	3	.	.	intergenic	0.365	rs787549328	2
AC.GT	3	13437166	13437177	6	.	.	intergenic	-0.171	rs788567036	2
AC.GT	3	13489359	13489378	10	.	.	intergenic	.	381813 rs7907	3
AAAT.ATTT	3	13545020	13545035	4	.	.	intergenic	.	rs791911327	2
AAAAT.ATTTT	3	13615722	13615737	3.2	.	.	intergenic	0.209	rs791838821	2
AAAAC.GTTTT	3	13802802	13802819	3.6	.	.	intergenic	0.251	722377 rs7901	2
AAC.GTT	3	13846771	13846785	5	.	.	intergenic	0.059	915694 rs7892	2
AAAC.GTTT	3	14145001	14145019	4.75	.	.	intergenic	0.256	596860 rs7927	2
AC.GT	3	14274603	14274613	5.5	.	.	intergenic	-0.017	rs786807599	3
AAT.ATT	3	14302613	14302626	4.66667	.	.	intergenic	.	rs713251305	2
AAAAG.CTTTT	3	14310373	14310399	5.4	.	.	intergenic	-0.216	rs787304370	2
AC.GT	3	14327850	14327879	15	.	.	intergenic	-0.102	rs791399923	5
AGAT.ATCT	3	14967463	14967481	4.75	.	.	intergenic	.	219854 rs7889	2
AAT.ATT	3	15177160	15177180	7	.	.	intergenic	0.081	549306 rs7915	2
AC.GT	3	15192148	15192160	6.5	.	.	intergenic	0.106	rs790747702	8
AAAT.ATTT	3	15210826	15210837	3	.	.	intergenic	0.043	298011 rs7928	2
AT.AT	3	15227122	15227154	16.5	.	.	intergenic	0.036	rs691205448	2
AT.AT	3	15238314	15238326	6.5	.	.	intergenic	0.149	771331 rs7882	2
AGGG.CCCT	3	15247293	15247306	3.5	.	.	intergenic	0.002	334746 rs7884	2
AAAAG.CTTTT	3	15315160	15315180	4.2	.	.	intergenic	0.188	rs786909290	2
AAAC.GTTT	3	15326604	15326625	5.5	.	.	intergenic	0.055	rs792960491	2
AG.CT	3	15366819	15366833	7.5	.	.	intergenic	-0.135	rs788551964	3
AG.CT	3	15374810	15374824	7.5	.	.	intergenic	0.635	rs791028179	2
AC.GT	3	15487909	15487927	9.5	.	.	intergenic	0.591	rs787842560	3
AAT.ATT	3	15851165	15851178	4.66667	.	.	intergenic	.	355772 rs7909	2
AC.GT	3	15855741	15855754	7	.	.	intergenic	0.166	rs786235528	3
AC.GT	3	16023423	16023435	6.5	ENSSSCT00000008475	.	intron	-0.185	.	2
AC.GT	3	16023901	16023918	9	ENSSSCT00000008475	.	intron	-0.026	rs792734795	3
AC.GT	3	16157984	16157993	5	ENSSSCT00000008475	.	intron	0.181	.	2
AT.AT	3	16166135	16166144	5	ENSSSCT00000008475	.	intron	.	rs788679775	2
AC.GT	3	16200725	16200735	5.5	.	.	intergenic	-0.134	.	2
AAAAC.GTTTT	3	16220706	16220729	4.8	.	.	intergenic	0.175	rs792100248	2
ATCC.GGAT	3	16234161	16234185	6.25	.	.	intergenic	.	rs790520415	2
AC.GT	3	16344376	16344386	5.5	ENSSSCT00000008478	.	intron	-0.152	rs786248095	2
AG.CT	3	16626607	16626621	7.5	ENSSSCT00000008480	.	intron	-0.103	.	3
AATC.GATT	3	16653874	16653900	6.75	.	.	intergenic	0.308	175387 rs7876	2
AG.CT	3	16801434	16801446	6.5	.	.	intergenic	.	.	2
AAAC.GTTT	3	16857923	16857942	5	.	.	intergenic	.	rs792463429	3
AC.GT	3	16995043	16995053	5.5	ENSSSCT00000025455	VKORC1L1	intron	-0.029	.	2
AC.GT	3	17000390	17000413	12	ENSSSCT00000025455	VKORC1L1	intron	.	rs790405007	2
AAAG.CTTT	3	17008622	17008636	3.75	ENSSSCT00000025455	VKORC1L1	intron	0.011	rs789078869	2
AAAT.ATTT	3	17252712	17252734	5.75	ENSSSCT00000027523	ZNF713	intron	.	rs793329212	2
AC.GT	3	17258013	17258026	7	ENSSSCT00000027523	ZNF713	intron	.	.	3
AAAC.GTTT	3	17292878	17292892	3.75	ENSSSCT00000008498	14-Sep	intron	1.029	rs790027683	2
AC.GT	3	17334831	17334842	6	.	.	intergenic	.	rs786717932	2
AAAC.GTTT	3	17511891	17511909	4.75	ENSSSCT00000008507	PRSS8	intron	-0.008	rs792308175	2
AG.CT	3	17650992	17651002	5.5	.	.	intergenic	.	222220 rs7865	2
ACAG.CTGT	3	17850137	17850155	4.75	ENSSSCT00000008519	STX1B	intron	0.209	rs791566213	2
AG.CT	3	17911904	17911918	7.5	.	.	intergenic	0.04	rs791765405	2
AAAC.GTTT	3	18627694	18627725	8	ENSSSCT00000028946	KCTD13	promoter	0.058	rs793706001	2
AAAC.GTTT	3	19029919	19029941	5.75	ENSSSCT00000008560	XPO6	intron	0.139	rs793264463	2
AAAT.ATTT	3	19172558	19172580	5.75	ENSSSCT00000026157	GSG1L	intron	0.604	rs790301307	2
ACGC.GCGT	3	19437258	19437269	3	ENSSSCT00000032927	KIAA0556	intron	-0.079	rs790825949	2
ACGC.GCGT	3	19437258	19437269	3	ENSSSCT00000008563	KIAA0556	intron	-0.079	rs790825949	2
ACGC.GCGT	3	19437258	19437269	3	ENSSSCT00000034865	KIAA0556	intron	-0.079	rs790825949	2
ACGC.GCGT	3	19437258	19437269	3	ENSSSCT00000036362	KIAA0556	intron	-0.079	rs790825949	2
AC.GT	3	19675246	19675260	7.5	.	.	intergenic	-0.445	.	2
AG.CT	3	20005785	20005801	8.5	.	.	intergenic	0.22	rs792289629	2
AAAG.CTTT	3	20404346	20404376	7.75	.	.	intergenic	.	.	2
AG.CT	3	20441038	20441051	7	.	.	intergenic	-0.107	rs791525193	2
AT.AT	3	20681897	20681908	6	.	.	intergenic	.	.	2
AC.GT	3	20684382	20684396	7.5	.	.	intergenic	-0.434	rs711639571	2

AG.CT	3	20885265	20885280	8	.	.	intergenic	0.853	.	2
AC.GT	3	21299709	21299720	6	.	.	intergenic	-0.065	rs789187820	2
AAAAG.CTTTT	3	21429644	21429663	4	.	.	intergenic	.	)50629 rs7862	2
AC.GT	3	21471162	21471178	8.5	.	.	intergenic	0.045	532552 rs7922	3
AGAT.ATCT	3	21476940	21476957	4.5	.	.	intergenic	-0.249	384429 rs7932	2
AC.GT	3	21705823	21705837	7.5	.	.	intergenic	0.012	554309 rs7896	4
AT.AT	3	22853768	22853779	6	ENSSSCT00000008581	CACNG3	intron	.	rs789162769	2
ATCC.GGAT	3	23720360	23720397	9.5	ENSSSCT00000008587	HS3ST2	intron	0.084	rs789846499	2
AAAAC.GTTTT	3	23800485	23800501	3.4	ENSSSCT00000008587	HS3ST2	intron	0.153	rs788131476	2
AAG.CTT	3	23873231	23873247	5.66667	.	.	intergenic	-0.149	467760 rs7922	2
AG.CT	3	23881449	23881459	5.5	.	.	intergenic	.	364613 rs7934	3
\AAAAC.GTTTT	3	24150513	24150534	3.66667	ENSSSCT00000030167	METTL9	intron	0.353	rs790661451	2
AATG.CATT	3	24198505	24198533	7.25	.	.	intergenic	-0.07	.	2
AG.CT	3	24289209	24289223	7.5	ENSSSCT00000008589	EEF2K	3'utr	0.004	rs787948656	2
AAAC.GTTT	3	24520932	24520946	3.75	.	.	intergenic	0.416	270773 rs7929	2
AG.CT	3	24719016	24719032	8.5	ENSSSCT00000008595	.	intron	0.083	225340 rs7884	2
ACTC.GAGT	3	25154164	25154182	4.75	ENSSSCT00000008597	.	intron	.	rs787839936	2
AAC.GTT	3	25350401	25350413	4.33333	ENSSSCT00000008598	.	intron	.	751127 rs7863	2
AC.GT	3	25479381	25479394	7	ENSSSCT00000008598	.	intron	.	rs791544974	2
AC.GT	3	25689287	25689300	7	.	.	intergenic	.	.	3
AAAAT.ATTTT	3	25745686	25745703	3.6	.	.	intergenic	0.384	rs792133166	2
AT.AT	3	25749741	25749769	14.5	.	.	intergenic	0.089	rs793766056	2
AAAT.ATTT	3	26072642	26072672	7.75	ENSSSCT00000024527	THUMPD1	intron	0.098	rs791150587	2
AAG.CTT	3	26081905	26081917	4.33333	ENSSSCT00000027442	ACSM4	intron	-0.028	rs793186876	2
AAC.GTT	3	26133293	26133309	5.66667	.	.	intergenic	0.008	rs791972632	2
AAC.GTT	3	26299352	26299370	6.33333	.	.	intergenic	.	151860 rs7866	3
AAAG.CTTT	3	26522382	26522400	4.75	.	.	intergenic	-0.042	572452 rs7888	2
AAAC.GTTT	3	26760369	26760387	4.75	.	.	intergenic	.	507133 rs7916	2
AAAT.ATTT	3	26761175	26761193	4.75	.	.	intergenic	.	.	2
AT.AT	3	26794927	26794936	5	.	.	intergenic	0.07	rs789435071	2
AAC.GTT	3	27072563	27072575	4.33333	ENSSSCT00000020027	U6	promoter	.	355487 rs7926	2
AT.AT	3	27210055	27210064	5	ENSSSCT00000031474	.	intron	-0.174	rs790200791	2
AAAC.GTTT	3	27286736	27286761	6.5	.	.	intergenic	0.158	rs792723982	2
AAAAT.ATTTT	3	27325161	27325183	4.6	.	.	intergenic	.	rs790342853	2
AAC.GTT	3	27334977	27334990	4.66667	.	.	intergenic	.	rs787408074	2
AC.GT	3	27449948	27449961	7	.	.	intergenic	-0.074	.	2
AAAC.GTTT	3	27573131	27573157	6.75	.	.	intergenic	.	rs787659662	2
AAAT.ATTT	3	27769523	27769549	6.75	ENSSSCT00000008627	.	intron	-0.365	284007 rs7933	2
AAAC.GTTT	3	27946124	27946147	6	.	.	intergenic	0.674	938130 rs7929	2
AG.CT	3	28179473	28179502	15	.	.	intergenic	-0.095	312233 rs7878	4
AC.GT	3	28228085	28228095	5.5	.	.	intergenic	.	rs790570479	2
AG.CT	3	28404420	28404432	6.5	.	.	intergenic	-0.145	206218 rs7907	2
AGG.CCT	3	28441500	28441514	5	.	.	intergenic	-0.094	rs787824609	2
AAGG.CCTT	3	28450891	28450940	12.5	.	.	intergenic	.	rs788078843	2
AT.AT	3	28577166	28577178	6.5	.	.	intergenic	0.098	rs787879683	2
AC.GT	3	28934139	28934164	13	.	.	intergenic	.	rs789737939	3
AAAC.GTTT	3	28938708	28938729	5.5	.	.	intergenic	-0.983	334358 rs7916	2
AC.GT	3	29037345	29037359	7.5	ENSSSCT00000008628	.	intron	.	rs790442665	2
AAAC.GTTT	3	29178366	29178390	6.25	.	.	intergenic	.	198267 rs7913	2
AAAC.GTTT	3	29179281	29179299	4.75	.	.	intergenic	.	106248 rs7877	2
AG.CT	3	29290568	29290581	7	.	.	intergenic	0.13	rs787818520	3
AC.GT	3	29366014	29366035	11	.	.	intergenic	.	.	2
AC.GT	3	29514832	29514843	6	ENSSSCT00000028273	CPPED1	intron	-0.345	.	2
AG.CT	3	29638967	29638979	6.5	ENSSSCT00000008633	PARN	intron	-0.071	rs787390643	2
AAAC.GTTT	3	30094814	30094829	4	ENSSSCT00000027014	.	intron	-0.009	rs788019543	2
AT.AT	3	30140590	30140600	5.5	ENSSSCT00000027014	.	intron	0.098	rs791475370	2
AAAAC.GTTTT	3	30386599	30386622	4.8	.	.	intergenic	.	rs790239337	2
AAT.ATT	3	30670742	30670762	7	.	.	intergenic	-0.03	rs791856306	2
AACC.GGTT	3	30771432	30771455	6	.	.	intergenic	-0.141	rs793059327	2
AC.GT	3	30805483	30805502	10	.	.	intergenic	-0.004	.	2
ATCC.GGAT	3	30807557	30807574	4.5	.	.	intergenic	.	369000 rs7899	2
AAAC.GTTT	3	30814789	30814809	5.25	.	.	intergenic	0.045	035118 rs7862	2
AGG.CCT	3	31076364	31076378	5	.	.	intergenic	0.015	467215 rs7930	2
AG.CT	3	31189647	31189659	6.5	.	.	intergenic	-0.098	.	3
\AAAAG.CTTTT	3	31313871	31313894	4	.	.	intergenic	0.06	rs787631771	2
AAACC.GGTTT	3	31336903	31336929	5.4	.	.	intergenic	0.044	)40136 rs7891	2
AC.GT	3	32032502	32032511	5	.	.	intergenic	0.266	rs791211044	4
AC.GT	3	32403270	32403290	10.5	.	.	intergenic	.	.	2
AC.GT	3	32524095	32524105	5.5	ENSSSCT00000008655	.	intron	.	rs787640796	2
AAAT.ATTT	3	32581781	32581803	5.75	.	.	intergenic	.	341057 rs7917	2
AAAG.CTTT	3	32968036	32968051	4	.	.	intergenic	0.027	rs787680043	2
AGC.GCT	3	33183314	33183326	4.33333	ENSSSCT00000008661	.	intron	-0.025	rs790736425	2
AAAC.GTTT	3	33220407	33220430	6	.	.	intergenic	.	)20660 rs7935	2
AG.CT	3	33269693	33269707	7.5	.	.	intergenic	-0.243	.	3
AAAAC.GTTTT	3	33351631	33351652	4.4	ENSSSCT00000032425	ATF7IP2	intron	0.174	rs790991063	2
ACTC.GAGT	3	33611925	33611938	3.5	.	.	intergenic	-0.187	.	2
AAAGC.GCTTT	3	33775902	33775922	4.2	ENSSSCT00000008663	GRIN2A	intron	-0.051	rs790099937	2
AG.CT	3	33880947	33880959	6.5	.	.	intergenic	-0.025	rs790923279	2
AAAAT.ATTTT	3	34438321	34438336	3.2	ENSSSCT00000029316	C16orf72	intron	.	.	2
AG.CT	3	34653544	34653562	9.5	ENSSSCT00000008668	ABAT	intron	0.035	rs788823649	2
AAAC.GTTT	3	34886000	34886018	4.75	.	.	intergenic	.	rs790255667	2
ACAG.CTGT	3	34894962	34894981	5	.	.	intergenic	0.089	rs791418228	2
AC.GT	3	34991972	34991985	7	.	.	intergenic	.	)58010 rs7892	4
AACC.GGTT	3	35206388	35206410	5.75	.	.	intergenic	0.26	rs793015582	2

AAAAC.GTTTT	3	35229566	35229588	4.6	.	.	intergenic	0.062	rs786707448	2
AAAAC.GTTTT	3	35415965	35415990	5.2	.	.	intergenic	0.116	rs789634035	2
AAAAT.ATTTT	3	35657350	35657370	4.2	.	.	intergenic	0.03	738337 rs7924	2
AAC.GTT	3	35861948	35861964	5.66667	.	.	intergenic	.	500461 rs7881	2
AAAAG.CTTTT	3	35976741	35976764	4.8	.	.	intergenic	-0.195	rs792163064	2
AAC.GTT	3	36010165	36010181	5.66667	ENSSSCT00000008677	RBFOX1	intron	0.334	rs790134000	2
AAT.ATT	3	36018170	36018185	5.33333	ENSSSCT00000008677	RBFOX1	intron	0.115	rs791617795	2
AAAAT.ATTTT	3	36305892	36305913	4.4	.	.	intergenic	-0.187	rs698640738	2
AAGG.CCTT	3	36308918	36308941	6	.	.	intergenic	-0.171	rs792092927	3
AAAC.GTTT	3	36313848	36313862	3.75	.	.	intergenic	-0.196	rs789018803	3
ATC.GAT	3	36344317	36344348	10.6667	.	.	intergenic	0.02	rs707885805	2
AG.CT	3	36413067	36413076	5	.	.	intergenic	0.261	.	2
AAT.ATT	3	36418357	36418371	5	.	.	intergenic	-0.245	.	3
AAAC.GTTT	3	36453247	36453273	6.75	.	.	intergenic	0.077	rs789134916	2
AC.GT	3	36518940	36518951	6	.	.	intergenic	-0.051	rs790434773	3
AC.GT	3	36584149	36584183	17.5	.	.	intergenic	-0.48	rs787805291	4
AC.GT	3	36629444	36629456	6.5	.	.	intergenic	.	227677 rs7933	2
AT.AT	3	36901880	36901894	7.5	.	.	intergenic	-0.265	.	2
AAG.CTT	3	37064554	37064573	6.66667	.	.	intergenic	-0.692	rs792070993	3
AC.GT	3	37101013	37101029	8.5	.	.	intergenic	0.102	rs788336016	2
ACATC.GATGT	3	37111945	37111962	3.6	.	.	intergenic	-0.02	rs793607077	2
AC.GT	3	37221424	37221446	11.5	.	.	intergenic	-0.275	.	4
AAAAG.CTTTT	3	37314258	37314275	3	.	.	intergenic	0.115	744785 rs7892	2
AC.GT	3	37508089	37508100	6	.	.	intergenic	-0.736	.	3
AG.CT	3	37680015	37680037	11.5	.	.	intergenic	0.118	rs787100346	4
AT.AT	3	37695929	37695938	5	.	.	intergenic	.	rs793716337	2
AAC.GTT	3	38269718	38269731	4.66667	.	.	intergenic	-0.287	rs788255600	2
AGGG.CCCT	3	39273108	39273120	3.25	.	.	intergenic	0.03	.	2
AC.GT	3	39567975	39567987	6.5	ENSSSCT00000008714	CREBBP	intron	.	.	4
AC.GT	3	39567975	39567987	6.5	ENSSSCT00000034723	CREBBP	intron	.	.	4
AAAT.ATTT	3	40978406	40978422	4.25	ENSSSCT00000008742	LUC7L	intron	.	rs789534264	2
AATT.AATT	3	41139093	41139118	6.5	.	.	intergenic	.	rs790643325	2
AAAC.GTTT	3	41831579	41831595	4.25	ENSSSCT00000008789	IFT140	intron	.	rs792505824	2
AT.AT	3	43124368	43124386	9.5	.	.	intergenic	.	.	3
AC.GT	3	43263445	43263457	6.5	ENSSSCT00000008843	CENPP	intron	0.879	.	3
AC.GT	3	43364100	43364113	7	ENSSSCT00000008843	CENPP	intron	0.175	.	2
AC.GT	3	43364100	43364113	7	ENSSSCT00000008845	ASPN	intron	0.175	.	2
AC.GT	3	43398485	43398494	5	ENSSSCT00000008843	CENPP	intron	0.031	.	2
AC.GT	3	43398485	43398494	5	ENSSSCT00000032452	ECM2	intron	0.031	.	2
AC.GT	3	44380830	44380840	5.5	.	.	intergenic	.	rs793551974	2
AG.CT	3	44619480	44619495	8	.	.	intergenic	-0.095	rs792135547	2
AAAAC.GTTTT	3	44776485	44776523	7.8	.	.	intergenic	.	rs790332319	2
AAAT.ATTT	3	44862459	44862480	5.5	ENSSSCT00000019914	ssc-let-7a-2	promoter	-0.037	rs789989135	2
AAG.CTT	3	45054399	45054410	4	.	.	intergenic	.	rs793647955	2
AAAC.GTTT	3	45200304	45200335	8	.	.	intergenic	.	rs789256224	2
AAG.CTT	3	45376215	45376264	16.6667	.	.	intergenic	.	.	3
AAAG.CTTT	3	45719859	45719885	6.75	.	.	intergenic	0.254	378292 rs7914	2
AAAAC.GTTTT	3	45772649	45772684	6	ENSSSCT00000008872	ZC3H8	intron	.	rs788036463	2
AG.CT	3	45776894	45776904	5.5	ENSSSCT00000008872	ZC3H8	intron	0.401	rs788013580	2
AC.GT	3	46227628	46227641	7	ENSSSCT00000008877	.	intron	.	775253 rs7908	3
AAAC.GTTT	3	46317255	46317273	4.75	ENSSSCT00000008877	.	intron	0.669	rs787334354	2
AAAC.GTTT	3	46317255	46317273	4.75	ENSSSCT00000008879	.	intron	0.669	rs787334354	2
ACC.GGT	3	46343203	46343221	6.33333	ENSSSCT00000008877	.	intron	.	rs786881947	2
CACCC.GGGTG	3	46343356	46343379	4	ENSSSCT00000008877	.	intron	.	rs788904938	2
AT.AT	3	46454856	46454869	7	.	.	intergenic	-0.092	rs789471132	2
AAAAG.CTTTT	3	46466184	46466208	4.16667	.	.	intergenic	-0.097	rs790782182	2
AGG.CCT	3	46543670	46543684	5	.	.	intergenic	.	.	2
AAG.CTT	3	46736828	46736840	4.33333	.	.	intergenic	-0.271	rs788354279	2
AAC.GTT	3	47137836	47137854	6.33333	ENSSSCT00000029914	.	intron	0.15	rs789818528	2
AAAAC.GTTTT	3	47244252	47244273	3.66667	ENSSSCT00000023794	.	intron	.	rs786403932	2
AC.GT	3	47330896	47330908	6.5	ENSSSCT00000008884	.	intron	0.09	.	4
ATCC.GGAT	3	47544227	47544250	6	.	.	intergenic	0.065	rs790772041	2
AAAT.ATTT	3	47753442	47753473	8	.	.	intergenic	.	362933 rs7872	2
AC.GT	3	47839040	47839051	6	.	.	intergenic	.	982401 rs7912	2
AAAC.GTTT	3	47921676	47921695	5	.	.	intergenic	.	055803 rs7908	2
AAAAC.GTTTT	3	48322355	48322384	6	.	.	intergenic	-0.174	449725 rs7893	2
AC.GT	3	48427019	48427031	6.5	ENSSSCT00000008895	KCNIP3	intron	0.149	rs787682158	3
AG.CT	3	48483892	48483901	5	ENSSSCT00000008896	.	intron	-0.226	rs791175603	2
AC.GT	3	49002914	49002932	9.5	ENSSSCT00000008901	.	intron	-0.211	487355 rs7918	2
AAAAAT.ATTTTT	3	49079112	49079132	3.5	ENSSSCT00000008901	.	intron	.	rs787794978	2
AT.AT	3	49137966	49137978	6.5	ENSSSCT00000008909	STARD7	intron	0.282	302349 rs7891	2
AT.AT	3	49291910	49291921	6	.	.	intergenic	.	.	5
AC.GT	3	49323756	49323773	9	.	.	intergenic	.	rs788761719	2
AG.CT	3	49554941	49554952	6	.	.	intergenic	.	rs789609597	2
AAAT.ATTT	3	49637014	49637026	3.25	ENSSSCT00000008912	CCDC138	intron	.	rs790812391	2
AC.GT	3	49658190	49658216	13.5	ENSSSCT00000008912	CCDC138	intron	.	rs790603422	3
AAAC.GTTT	3	49766228	49766246	4.75	.	.	intergenic	.	.	2
AAAAC.GTTTT	3	49894382	49894408	5.4	ENSSSCT00000008915	GCC2	intron	0.558	521655 rs7901	3
ACAT.ATGT	3	50304543	50304565	5.75	.	.	intergenic	0.038	rs787681339	2
AAAG.CTTT	3	50367811	50367828	4.5	.	.	intergenic	-0.426	rs793437954	2
AG.CT	3	50400716	50400731	8	.	.	intergenic	0.199	rs786359354	2
AG.CT	3	50624389	50624406	9	.	.	intergenic	0.068	rs787087650	2
ACAT.ATGT	3	50861754	50861765	3	.	.	intergenic	0.183	rs791489285	2
ACAT.ATGT	3	50967932	50967949	4.5	.	.	intergenic	.	rs791331351	2

AC.GT	3	51157921	51157932	6	.	.	intergenic	.	18597 rs7883	3
AT.AT	3	51289471	51289482	6	.	.	intergenic	-0.04	rs792759189	2
AAAG.CTTT	3	51308956	51308975	5	.	.	intergenic	-0.473	rs789508718	2
AAG.CTT	3	51365551	51365562	4	.	.	intergenic	.	rs793497040	2
AAAAG.CTTTT	3	51417486	51417508	4.6	.	.	intergenic	-0.026	rs791219867	2
AC.GT	3	51616839	51616849	5.5	ENSSSCT00000008924	FHL2	intron	.	rs792211351	2
AAAC.GTTT	3	51778026	51778042	4.25	.	.	intergenic	0.096	rs790676461	2
AAT.ATT	3	52606556	52606567	4	.	.	intergenic	.	.	2
AT.AT	3	52677256	52677272	8.5	.	.	intergenic	.	rs786972748	2
AT.AT	3	52695888	52695900	6.5	.	.	intergenic	-0.243	.	2
AAAC.GTTT	3	52854901	52854919	4.75	.	.	intergenic	0.055	rs793032999	2
AAC.GTT	3	52875396	52875413	6	.	.	intergenic	.	rs790457937	2
AG.CT	3	52907797	52907809	6.5	.	.	intergenic	-0.09	.	3
AT.AT	3	52951117	52951135	9.5	.	.	intergenic	0.057	rs792120594	2
AG.CT	3	53137205	53137217	6.5	.	.	intergenic	-0.033	.	3
AAAAC.GTTTT	3	53182752	53182776	5	.	.	intergenic	-0.067	rs788260723	2
AC.GT	3	53367359	53367370	6	.	.	intergenic	-0.081	rs790354451	4
AAAC.GTTT	3	53373699	53373722	6	.	.	intergenic	0.123	79091 rs7927	2
AAC.GTT	3	53494785	53494797	4.33333	.	.	intergenic	0.357	34074 rs7881	2
AG.CT	3	53728655	53728679	12.5	.	.	intergenic	-0.168	rs788381517	2
AAAC.GTTT	3	53904980	53905002	5.75	ENSSSCT00000008927	TMEM182	intron	.	rs788979679	2
AAC.GTT	3	53944724	53944737	4.66667	ENSSSCT00000008928	MFSD9	intron	-0.084	rs789383230	2
AAT.ATT	3	53955352	53955372	7	.	.	intergenic	.	.	2
AAAC.GTTT	3	54001602	54001620	4.75	ENSSSCT00000008930	NHE-2	intron	0.073	rs788645005	2
AC.GT	3	54255721	54255743	11.5	ENSSSCT00000008934	IL18RAP	intron	-0.365	rs692554221	2
AC.GT	3	54255721	54255743	11.5	ENSSSCT00000034710	IL18RAP	intron	-0.365	rs692554221	2
AC.GT	3	54255721	54255743	11.5	ENSSSCT00000034786	IL18RAP	intron	-0.365	rs692554221	2
AG.CT	3	54276543	54276553	5.5	.	.	intergenic	0.106	.	2
AG.CT	3	54448366	54448375	5	ENSSSCT00000008936	IL1RL1	intron	.	rs786523167	2
AC.GT	3	54627913	54627936	12	.	.	intergenic	.	rs787754077	2
AAAC.GTTT	3	54676165	54676182	4.5	.	.	intergenic	.	41931 rs7933	2
AC.GT	3	54684357	54684368	6	.	.	intergenic	.	.	3
AG.CT	3	54781714	54781725	6	.	.	intergenic	-0.259	16343 rs7870	2
AAGG.CCTT	3	54790454	54790465	3	.	.	intergenic	-0.069	.	2
AAAAT.ATTTT	3	54797534	54797552	3.8	.	.	intergenic	.	77792 rs7886	2
AAAAC.GTTTT	3	54838716	54838741	4.33333	.	.	intergenic	0.111	09547 rs7911	2
AAAC.GTTT	3	54890500	54890511	3	.	.	intergenic	.	.	2
AAC.GTT	3	54972216	54972235	6.66667	.	.	intergenic	-0.021	54675 rs7923	2
AC.GT	3	55396430	55396446	8.5	ENSSSCT00000008942	RFX8	intron	.	rs786278722	5
AG.CT	3	55672673	55672687	7.5	.	.	intergenic	0.208	rs793790178	2
AC.GT	3	55759184	55759206	11.5	ENSSSCT00000008947	RPL31	intron	.	00325 rs7887	5
ACAT.ATGT	3	55871369	55871393	6.25	ENSSSCT00000008948	.	intron	-0.258	rs786951017	3
AGG.CCT	3	56007554	56007565	4	ENSSSCT00000008948	.	intron	0.181	.	2
AC.GT	3	56312439	56312451	6.5	.	.	intergenic	.	rs697944266	4
AC.GT	3	56323814	56323828	7.5	.	.	intergenic	0.11	49971 rs7931	3
AAAAG.CTTTT	3	56446123	56446142	3.33333	.	.	intergenic	.	.	3
AC.GT	3	56684223	56684233	5.5	.	.	intergenic	-0.104	.	3
AT.AT	3	56782197	56782206	5	ENSSSCT00000008955	.	intron	-0.592	.	3
AGG.CCT	3	56850868	56850883	5.33333	ENSSSCT00000008955	.	intron	0.075	20037 rs7900	2
AT.AT	3	56939026	56939036	5.5	ENSSSCT00000008955	.	intron	0.435	94984 rs7897	2
ATC.GAT	3	56966119	56966140	7.33333	ENSSSCT00000008955	.	intron	0.061	rs786283077	2
AAAC.GTTT	3	56966421	56966461	10.25	ENSSSCT00000008955	.	intron	0.331	rs791188348	3
AATG.CATT	3	56993116	56993143	7	ENSSSCT00000008955	.	intron	-0.361	rs786733285	3
AG.CT	3	57188074	57188087	7	ENSSSCT00000008955	.	intron	.	rs793736078	2
AAAC.GTTT	3	57239155	57239183	7.25	.	.	intergenic	-0.03	rs789803412	3
AAAAG.CTTTT	3	57492965	57492998	6.8	ENSSSCT00000008958	REV1	intron	2.45	.	4
AT.AT	3	57655239	57655261	11.5	ENSSSCT00000024738	LYG2	intron	0.091	rs787313547	3
AAAAC.GTTTT	3	57776240	57776267	4.66667	ENSSSCT00000008965	TSGA10	cds	0.376	rs791797830	2
AG.CT	3	57893469	57893479	5.5	.	.	intergenic	0.008	.	2
AAAC.GTTT	3	57944128	57944163	9	.	.	intergenic	.	rs788853730	2
AAAACC.GGTTT	3	57997941	57997960	3.33333	.	.	intergenic	-0.177	.	2
AAAAC.GTTTT	3	58405220	58405261	8.4	.	.	intergenic	.	.	2
AAAAC.GTTTT	3	58828318	58828342	4.16667	.	.	intergenic	0.05	rs790796831	2
AAAT.ATTT	3	59148350	59148366	4.25	ENSSSCT00000035625	ZAP70	intron	.	rs792618811	2
AAAT.ATTT	3	59148350	59148366	4.25	ENSSSCT00000036079	ZAP70	intron	.	rs792618811	2
AAAT.ATTT	3	59148350	59148366	4.25	ENSSSCT00000008972	ZAP70	intron	.	rs792618811	2
AAAT.ATTT	3	59477044	59477056	3.25	ENSSSCT00000008980	.	intron	.	58375 rs7909	2
ACAAT.ATTGT	3	59508393	59508420	4.66667	.	.	intergenic	0.372	rs703238455	2
AAC.GTT	3	60247356	60247382	9	.	.	intergenic	-0.313	19431 rs7882	3
AAC.GTT	3	60394187	60394203	5.66667	.	.	intergenic	.	rs791882021	2
AG.CT	3	60621097	60621108	6	ENSSSCT00000033509	CD8B	intron	-0.079	rs790723495	2
AG.CT	3	60621097	60621108	6	ENSSSCT00000008994	CD8B	intron	-0.079	rs790723495	2
AG.CT	3	60621097	60621108	6	ENSSSCT00000033653	CD8B	intron	-0.079	rs790723495	2
AG.CT	3	60621097	60621108	6	ENSSSCT00000034821	CD8B	intron	-0.079	rs790723495	2
AC.GT	3	60780895	60780907	6.5	.	.	intergenic	.	rs789917204	3
AAAAG.CTTTT	3	60818602	60818626	5	ENSSSCT00000008997	RMND5A	intron	.	rs789515303	2
AT.AT	3	60966749	60966761	6.5	ENSSSCT00000031530	.	intron	0.111	rs788521123	2
AAAT.ATTT	3	61170368	61170394	6.75	.	.	intergenic	.	.	2
AG.CT	3	61192762	61192788	13.5	.	.	intergenic	0.106	rs791475660	2
AT.AT	3	61460580	61460590	5.5	ENSSSCT00000030815	IMMT	intron	.	rs788101578	2
AAT.ATT	3	61719636	61719648	4.33333	.	.	intergenic	.	.	3
AAAC.GTTT	3	61726263	61726277	3.75	ENSSSCT00000025660	.	intron	0.078	41180 rs7871	2
AC.GT	3	61751027	61751039	6.5	.	.	intergenic	0.083	rs792328867	2
AAAT.ATTT	3	62012551	62012567	4.25	.	.	intergenic	.	rs786225079	2

AAAAT.ATTTT	3	62034309	62034332	4.8	ENSSSCT00000009013	ATOH8	intron	-0.211	rs788129432	2
AG.CT	3	62158668	62158692	12.5	.	.	intergenic	-0.014	rs791868086	4
AAAC.GTTT	3	62197861	62197881	5.25	ENSSSCT00000027775	.	intron	.	rs793274522	2
AAAG.CTTT	3	62388137	62388151	3.75	.	.	intergenic	.	750588 rs7063	2
AAAC.GTTT	3	62424214	62424230	4.25	.	.	intergenic	-0.433	rs789249119	2
AC.GT	3	62606985	62606995	5.5	.	.	intergenic	0.115	.	2
AC.GT	3	62708769	62708787	9.5	.	.	intergenic	0.639	rs791220368	2
AC.GT	3	62885802	62885816	7.5	.	.	intergenic	0.029	913649 rs7868	2
ACAT.ATGT	3	62915694	62915707	3.5	.	.	intergenic	-0.205	rs789643936	2
AT.AT	3	63005707	63005716	5	.	.	intergenic	.	rs786279021	2
AAAT.ATTT	3	63033490	63033509	5	.	.	intergenic	0.197	rs790112302	2
AAAC.GTTT	3	63066351	63066367	4.25	.	.	intergenic	0.168	.	2
AATC.GATT	3	63377320	63377339	5	.	.	intergenic	.	969170 rs7896	2
AT.AT	3	63411806	63411820	7.5	.	.	intergenic	0.031	rs790257207	2
AAAC.GTTT	3	63723823	63723837	3.75	.	.	intergenic	.	.	2
AAAAT.ATTTT	3	63801213	63801230	3.6	.	.	intergenic	0.112	275565 rs7929	2
AT.AT	3	63875483	63875495	6.5	.	.	intergenic	0.03	rs792602480	2
AT.AT	3	64003820	64003831	6	.	.	intergenic	0.144	rs787053880	2
AC.GT	3	64049338	64049348	5.5	.	.	intergenic	.	rs791366628	2
AAAC.GTTT	3	64105405	64105423	4.75	.	.	intergenic	.	rs788470526	2
AAAC.GTTT	3	64109806	64109820	3.75	.	.	intergenic	-0.032	rs790924686	2
AAAAC.GTTTT	3	64145656	64145685	6	.	.	intergenic	.	rs790005156	2
AG.CT	3	64314197	64314209	6.5	.	.	intergenic	-0.131	.	3
AC.GT	3	64322258	64322272	7.5	.	.	intergenic	-0.414	480767 rs7891	8
AAAAT.ATTTT	3	64361948	64361969	4.4	.	.	intergenic	.	.	2
AT.AT	3	64437162	64437171	5	.	.	intergenic	0.071	rs793094715	2
AAAC.GTTT	3	64486732	64486762	7.75	.	.	intergenic	-0.146	322037 rs7881	2
AT.AT	3	64604258	64604275	9	.	.	intergenic	-0.159	241871 rs7881	2
AAAC.GTTT	3	64607089	64607113	6.25	.	.	intergenic	-0.081	rs791750228	2
AC.GT	3	64618223	64618232	5	.	.	intergenic	0.095	.	3
AAC.GTT	3	64641821	64641837	5.66667	.	.	intergenic	-0.123	rs790558409	2
AAAC.GTTT	3	64832020	64832047	7	.	.	intergenic	-0.097	701306 rs7866	2
AT.AT	3	64871988	64872010	11.5	.	.	intergenic	-0.234	643966 rs7113	2
AAT.ATT	3	64952556	64952568	4.33333	.	.	intergenic	0.104	.	2
AAAG.CTTT	3	64955818	64955832	3.75	.	.	intergenic	0.157	.	2
AAAT.ATTT	3	65052452	65052466	3.75	.	.	intergenic	-0.063	rs791337103	2
AAGGG.CCCTT	3	65064666	65064683	3.6	.	.	intergenic	-0.108	rs789823809	2
AT.AT	3	65111738	65111747	5	.	.	intergenic	.	.	2
AAT.ATT	3	65140787	65140798	4	.	.	intergenic	.	rs788845407	2
AAC.GTT	3	65271276	65271289	4.66667	.	.	intergenic	0.047	rs788720885	3
AAC.GTT	3	65318156	65318173	6	.	.	intergenic	-0.18	rs791266817	2
AG.CT	3	65372924	65372937	7	.	.	intergenic	.	.	4
AC.GT	3	65545824	65545835	6	.	.	intergenic	.	.	3
AAGC.GCTT	3	65991358	65991374	4.25	.	.	intergenic	.	rs708737788	2
AAAAG.CTTTT	3	66013207	66013224	3.6	.	.	intergenic	-0.044	rs793597171	2
AG.CT	3	66023278	66023311	17	.	.	intergenic	.	180219 rs7896	4
AC.GT	3	66066209	66066222	7	.	.	intergenic	0.375	rs789498643	2
AG.CT	3	66301903	66301912	5	ENSSSCT00000009036	.	intron	-0.109	.	4
AT.AT	3	66345080	66345101	11	ENSSSCT00000009036	.	intron	0.123	141255 rs7892	2
AAG.CTT	3	66479207	66479219	4.33333	ENSSSCT00000009036	.	intron	0.459	rs786301574	2
AAATG.CATTT	3	66538817	66538836	4	ENSSSCT00000009036	.	intron	0.296	486422 rs7921	2
AAAC.GTTT	3	66549757	66549791	8.75	ENSSSCT00000009036	.	intron	.	290029 rs7912	3
AAAC.GTTT	3	66691237	66691261	6.25	.	.	intergenic	0.273	rs791375308	2
AG.CT	3	66703078	66703111	17	.	.	intergenic	1.389	rs786468125	4
AAATT.AATTT	3	66847623	66847637	3	.	.	intergenic	.	.	2
AAAC.GTTT	3	66876555	66876573	4.75	.	.	intergenic	-0.417	rs790869342	2
AG.CT	3	67075708	67075720	6.5	.	.	intergenic	-0.071	571551 rs7892	2
ATC.GAT	3	67109453	67109468	5.33333	.	.	intergenic	-0.197	516488 rs7904	2
AT.AT	3	67293740	67293750	5.5	ENSSSCT00000009040	CTNNA2	intron	-0.051	rs789090427	2
AAAT.ATTT	3	67302455	67302469	3.75	ENSSSCT00000009040	CTNNA2	intron	-0.183	342682 rs7923	2
AAAC.GTTT	3	67802583	67802601	4.75	.	.	intergenic	.	rs787541453	2
AT.AT	3	67827215	67827227	6.5	.	.	intergenic	.	rs792353205	4
AC.GT	3	67870100	67870110	5.5	.	.	intergenic	-0.097	rs788179133	3
AAG.CTT	3	67918573	67918584	4	.	.	intergenic	.	rs787638043	2
AT.AT	3	68046143	68046155	6.5	.	.	intergenic	-0.21	rs791667769	3
AAAT.ATTT	3	68325851	68325876	6.5	.	.	intergenic	0.063	rs788849747	2
AC.GT	3	68512803	68512817	7.5	.	.	intergenic	.	559054 rs7879	2
AT.AT	3	68570459	68570481	11.5	.	.	intergenic	.	rs790775884	2
AGAT.ATCT	3	68877765	68877779	3.75	.	.	intergenic	-0.065	rs791900267	2
AAAAT.ATTTT	3	68968644	68968662	3.8	.	.	intergenic	.	.	2
AAAC.GTTT	3	69020704	69020728	6.25	.	.	intergenic	.	.	4
AC.GT	3	69066934	69066947	7	.	.	intergenic	.	.	3
AAC.GTT	3	69279689	69279712	8	.	.	intergenic	0.043	rs789843558	2
AT.AT	3	69433931	69433950	10	.	.	intergenic	-0.061	rs787157579	2
AAC.GTT	3	69489962	69489978	5.66667	.	.	intergenic	-0.023	rs789741366	3
AAAAC.GTTTT	3	69556808	69556828	4.2	.	.	intergenic	.	rs792117877	2
AAAC.GTTT	3	69656919	69656944	6.5	.	.	intergenic	0.163	234521 rs7878	4
AAAAT.ATTTT	3	69694724	69694746	4.6	.	.	intergenic	.	rs790508552	2
AG.CT	3	69845193	69845211	9.5	.	.	intergenic	-0.001	rs793818419	4
AT.AT	3	69873491	69873506	8	.	.	intergenic	-0.047	rs787000701	2
AC.GT	3	69883953	69883976	12	.	.	intergenic	0.027	rs787973599	2
AAAAAG.CTTTTT	3	69919100	69919121	3.66667	.	.	intergenic	-0.213	388662 rs7926	2
AAAC.GTTT	3	70037428	70037447	5	.	.	intergenic	-0.038	.	3
AAAC.GTTT	3	70044614	70044636	5.75	.	.	intergenic	0.002	rs692417613	2

AAC.GTT	3	70081463	70081476	4.66667	.	.	intergenic	-0.229	rs790375198	2
AAT.ATT	3	70220104	70220120	5.66667	.	.	intergenic	-0.075	.	2
AGAT.ATCT	3	70298457	70298475	4.75	.	.	intergenic	-0.01	rs789715611	2
AAC.GTT	3	70349329	70349348	6.66667	.	.	intergenic	.	rs791721503	2
AAT.ATT	3	70384560	70384571	4	.	.	intergenic	-0.004	354910 rs7926	2
AAAT.ATTT	3	70421368	70421397	7.5	.	.	intergenic	0.134	rs790180251	2
AC.GT	3	70424037	70424064	14	.	.	intergenic	1.091	rs789980403	4
AGG.CCT	3	71074117	71074130	4.66667	.	.	intergenic	0.185	rs791714990	2
AAAC.GTTT	3	71105412	71105423	3	ENSSSCT00000023158	MRPL19	3'utr	.	.	2
AC.GT	3	71453836	71453872	18.5	.	.	intergenic	-0.138	rs791406410	2
AT.AT	3	71519704	71519715	6	.	.	intergenic	0.113	rs793518493	2
AAAG.CTTT	3	71617772	71617783	3	ENSSSCT00000009049	M1AP	intron	.	rs792467269	2
AAAG.CTTT	3	71746418	71746436	4.75	ENSSSCT00000009054	AUP1	intron	0.105	rs793045219	2
AATG.CATT	3	71831003	71831029	6.75	ENSSSCT00000009054	AUP1	intron	-0.11	rs792896544	2
AAG.CTT	3	71863802	71863818	5.66667	ENSSSCT00000009065	.	promoter	0.045	rs793003089	2
AAG.CTT	3	71873861	71873886	8.66667	ENSSSCT00000009066	LBX2	promoter	-0.016	rs792518040	3
AT.AT	3	72381108	72381120	6.5	ENSSSCT00000009091	STAMBP	intron	0.157	rs786939946	2
AT.AT	3	72563191	72563200	5	.	.	intergenic	0.182	.	4
AAC.GTT	3	72626791	72626810	6.66667	.	.	intergenic	0.219	.	4
AC.GT	3	72729683	72729693	5.5	.	.	intergenic	-0.073	rs790803160	2
AAAT.ATTT	3	73443640	73443663	6	.	.	intergenic	.	.	2
AC.GT	3	74059213	74059229	8.5	.	.	intergenic	.	.	2
AAAC.GTTT	3	74229873	74229887	3.75	.	.	intergenic	.	rs789400673	2
AC.GT	3	74252027	74252037	5.5	.	.	intergenic	-0.058	397414 rs7872	2
AG.CT	3	74373332	74373345	7	.	.	intergenic	0.075	rs791470502	2
AC.GT	3	74410699	74410713	7.5	.	.	intergenic	.	.	3
AC.GT	3	74472764	74472779	8	ENSSSCT00000009103	DYSF	intron	-0.136	.	2
AC.GT	3	74559439	74559449	5.5	ENSSSCT00000009103	DYSF	intron	-0.013	.	2
AG.CT	3	74839718	74839732	7.5	.	.	intergenic	.	rs786387139	2
ATC.GAT	3	74863532	74863554	7.66667	.	.	intergenic	0.261	rs788434435	2
AAAC.GTTT	3	74865462	74865482	5.25	.	.	intergenic	0.326	rs792203158	2
AAAAC.GTTTT	3	75015716	75015740	5	.	.	intergenic	-0.123	.	2
AC.GT	3	75125050	75125061	6	.	.	intergenic	.	.	2
AAAG.CTTT	3	75610001	75610019	4.75	.	.	intergenic	-0.168	rs786460534	2
ATC.GAT	3	75827776	75827799	8	.	.	intergenic	0.122	rs790661251	3
AT.AT	3	75877167	75877176	5	.	.	intergenic	-0.269	.	4
.CACAG.CTGTG	3	75931799	75931819	3.5	ENSSSCT00000009126	MXD1	intron	0.153	)52634 rs7900	2
AGG.CCT	3	76003804	76003818	5	ENSSSCT00000009127	.	intron	0.401	.	2
AG.CT	3	76111213	76111223	5.5	ENSSSCT00000009128	ANXA4	intron	0.035	.	3
ACAG.CTGT	3	76147209	76147222	3.5	.	.	intergenic	-0.01	rs790186859	2
AAAC.GTTT	3	76216374	76216388	3.75	.	.	intergenic	.	rs788040403	2
AAAC.GTTT	3	76283156	76283180	6.25	ENSSSCT00000009130	AAK1	intron	0.081	.	2
AAAG.CTTT	3	76520931	76520943	3.25	.	.	intergenic	0.16	rs793814819	2
AAAC.GTTT	3	76554021	76554047	6.75	.	.	intergenic	.	rs786741692	2
AC.GT	3	76621069	76621080	6	ENSSSCT00000009134	ANTXR1	intron	0.06	rs792927723	2
AAAG.CTTT	3	76667819	76667836	4.5	ENSSSCT00000009134	ANTXR1	intron	-0.104	rs793525297	2
AAAC.GTTT	3	76807945	76807963	4.75	ENSSSCT00000009134	ANTXR1	intron	.	rs788577922	2
AAC.GTT	3	77283586	77283605	6.66667	.	.	intergenic	0.204	rs791130116	4
AG.CT	3	77360425	77360436	6	ENSSSCT00000009140	APLF	intron	0.076	.	2
AG.CT	3	77360425	77360436	6	ENSSSCT00000009141	FBXO48	promoter	0.076	.	2
AATT.AATT	3	77643127	77643142	4	.	.	intergenic	0.007	rs791932898	2
AAAT.ATTT	3	77645466	77645488	5.75	.	.	intergenic	-0.037	rs793579987	2
AC.GT	3	77657161	77657171	5.5	.	.	intergenic	0.035	.	2
AAAC.GTTT	3	77721672	77721693	5.5	.	.	intergenic	.	rs793332583	3
AAG.CTT	3	77925315	77925327	4.33333	.	.	intergenic	0.359	rs787199631	2
AC.GT	3	77985710	77985719	5	.	.	intergenic	-0.168	.	2
.CACAG.CTGTG	3	77990885	77990910	4.33333	.	.	intergenic	-0.051	rs789181190	2
AAAC.GTTT	3	78071010	78071036	6.75	.	.	intergenic	0.594	rs792073410	2
AAAC.GTTT	3	78153407	78153429	5.75	.	.	intergenic	0.404	rs793156286	3
AAC.GTT	3	78265147	78265164	6	.	.	intergenic	.	rs793625368	2
AAAT.ATTT	3	78312629	78312652	6	.	.	intergenic	.	rs793110439	2
AT.AT	3	78314264	78314280	8.5	.	.	intergenic	.	rs788975633	2
AAAC.GTTT	3	78317993	78318006	3.5	.	.	intergenic	.	rs792600513	2
AC.GT	3	78332195	78332205	5.5	.	.	intergenic	0.082	rs791711779	2
AAC.GTT	3	78502611	78502625	5	.	.	intergenic	0.212	rs793062317	2
AAAC.GTTT	3	78577938	78577956	4.75	.	.	intergenic	0.179	)70829 rs7881	2
AC.GT	3	78591128	78591137	5	.	.	intergenic	-0.285	.	2
AAAT.ATTT	3	78599544	78599562	4.75	.	.	intergenic	-0.061	rs792644842	2
AAAC.GTTT	3	78961986	78962005	5	.	.	intergenic	0.09	rs789413936	2
AC.GT	3	79030432	79030442	5.5	.	.	intergenic	0.166	479862 rs7935	2
AAAG.CTTT	3	79256888	79256906	4.75	.	.	intergenic	0.976	.	2
AAT.ATT	3	79413749	79413781	11	.	.	intergenic	0.107	rs787650289	2
AG.CT	3	79481548	79481558	5.5	.	.	intergenic	0.077	rs790861052	2
AAAC.GTTT	3	79799653	79799667	3.75	.	.	intergenic	0.076	rs790792578	2
ACTC.GAGT	3	79856508	79856530	5.75	.	.	intergenic	0.08	321105 rs7880	2
AC.GT	3	79900740	79900750	5.5	.	.	intergenic	0.162	rs787108968	4
AAAG.CTTT	3	80084228	80084239	3	.	.	intergenic	.	.	2
AAAC.GTTT	3	80166867	80166887	5.25	.	.	intergenic	0.158	rs788499942	2
AAAAG.CTTTT	3	80294181	80294204	4.8	.	.	intergenic	.	rs786712624	2
AT.AT	3	80670789	80670804	8	.	.	intergenic	.	rs791772707	3
AAAC.GTTT	3	80753419	80753441	5.75	ENSSSCT00000024583	.	intron	-0.012	rs787690095	2
AAAT.ATTT	3	81028486	81028504	4.75	.	.	intergenic	0.193	rs787838005	2
AATG.CATT	3	81166763	81166781	4.75	.	.	intergenic	-0.077	)18907 rs7863	2
AAC.GTT	3	81327236	81327249	4.66667	ENSSSCT00000030136	.	promoter	0.264	rs788481606	2

AT.AT	3	81358479	81358488	5	.	.	intergenic	-0.178	rs791270067	2
AT.AT	3	81557990	81557999	5	.	.	intergenic	.	rs790473996	2
AAC.GTT	3	81604966	81604979	4.66667	ENSSSCT00000028590	.	intron	0.302	rs792812239	2
ACAT.ATGT	3	81796755	81796773	4.75	.	.	intergenic	0.289	556161 rs7895	2
AG.CT	3	82108068	82108079	6	.	.	intergenic	.	.	3
AT.AT	3	82469516	82469525	5	.	.	intergenic	0.523	322515 rs7871	2
AG.CT	3	82670980	82670990	5.5	.	.	intergenic	2.262	rs792856320	2
AC.GT	3	82810840	82810870	15.5	.	.	intergenic	.	rs788907618	2
AAAAC.GTTTT	3	82946284	82946304	4.2	.	.	intergenic	0.152	327597 rs7875	2
AC.GT	3	83261922	83261934	6.5	.	.	intergenic	.	.	2
AAC.GTT	3	83544642	83544658	5.66667	.	.	intergenic	0.176	907934 rs7931	2
AAC.GTT	3	83842812	83842825	4.66667	.	.	intergenic	.	rs789302385	2
AAAT.ATTT	3	84984734	84984752	4.75	ENSSSCT00000009185	REL	intron	0.065	rs790365270	2
AAAC.GTTT	3	84988643	84988661	4.75	ENSSSCT00000009185	REL	intron	0.019	rs788034905	2
AG.CT	3	85157237	85157249	6.5	.	.	intergenic	.	rs787004227	2
AAG.CTT	3	85223587	85223610	8	.	.	intergenic	0.526	rs793010251	2
AC.GT	3	85460461	85460483	11.5	.	.	intergenic	0.161	.	2
AAAC.GTTT	3	85748055	85748090	9	.	.	intergenic	-0.115	586391 rs7901	2
AG.CT	3	85823034	85823061	14	.	.	intergenic	0.003	rs790490510	2
AC.GT	3	86055984	86055998	7.5	.	.	intergenic	0.262	rs787897799	3
AC.GT	3	86114397	86114411	7.5	.	.	intergenic	.	581438 rs7865	5
AC.GT	3	86221712	86221727	8	.	.	intergenic	1.354	.	3
AG.CT	3	86232296	86232310	7.5	.	.	intergenic	0.239	rs791281821	2
AT.AT	3	86246305	86246323	9.5	.	.	intergenic	0.134	.	2
AAAT.ATTT	3	86375061	86375078	4.5	.	.	intergenic	0.01	086902 rs7883	2
AAAC.GTTT	3	86421253	86421279	6.75	.	.	intergenic	0.017	313163 rs7909	2
AC.GT	3	86527505	86527515	5.5	.	.	intergenic	0.294	.	3
AAATT.AATTT	3	86551620	86551638	3.8	.	.	intergenic	0.081	rs793580083	2
AAAT.ATTT	3	86736142	86736164	5.75	.	.	intergenic	0.007	rs793504276	2
AAAAC.GTTTT	3	86823342	86823366	5	.	.	intergenic	-0.058	rs787066139	2
AC.GT	3	86985821	86985835	7.5	.	.	intergenic	.	.	2
AG.CT	3	87042989	87043002	7	.	.	intergenic	0.899	rs788704220	2
AT.AT	3	87182468	87182477	5	.	.	intergenic	1.301	rs789684120	2
ACAG.CTGT	3	87219965	87219991	6.75	.	.	intergenic	-0.064	rs791322659	2
AAAAT.ATTTT	3	87561285	87561304	4	.	.	intergenic	0.563	rs792612432	2
AAAAC.GTTTT	3	87637724	87637748	5	.	.	intergenic	-0.26	.	2
AAAAC.GTTTT	3	88056120	88056134	3	ENSSSCT00000009192	VRK2	intron	.	rs786739719	2
AAAT.ATTT	3	88060017	88060039	5.75	ENSSSCT00000009192	VRK2	intron	0.196	rs786651940	2
AT.AT	3	88247084	88247094	5.5	.	.	intergenic	0.187	rs788302290	2
AT.AT	3	88483069	88483083	7.5	.	.	intergenic	.	rs790980392	2
AAAG.CTTT	3	88513104	88513126	5.75	.	.	intergenic	-0.079	rs787944015	2
AAAC.GTTT	3	88630083	88630099	4.25	.	.	intergenic	0.092	rs788944176	2
AAC.GTT	3	88874567	88874580	4.66667	.	.	intergenic	0.1	rs788045280	2
AT.AT	3	88899445	88899456	6	.	.	intergenic	.	383000 rs7862	2
AGATAT.ATATC	3	88909158	88909186	4.83333	.	.	intergenic	-0.173	353054 rs7905	2
AG.CT	3	88948328	88948338	5.5	.	.	intergenic	.	rs790543712	2
AT.AT	3	88953185	88953194	5	.	.	intergenic	-0.095	178031 rs7882	2
AT.AT	3	88963436	88963445	5	.	.	intergenic	0.119	rs792166769	2
AAC.GTT	3	89070809	89070824	5.33333	.	.	intergenic	.	rs791945823	2
AT.AT	3	89152486	89152507	11	.	.	intergenic	.	.	4
AT.AT	3	89168887	89168898	6	.	.	intergenic	0.125	rs787272654	3
AG.CT	3	89221570	89221589	10	.	.	intergenic	.	.	3
AAAC.GTTT	3	89373092	89373119	7	.	.	intergenic	0.06	rs786358257	2
AAAC.GTTT	3	89764134	89764147	3.5	ENSSSCT00000009193	.	intron	.	rs788940478	2
AAAT.ATTT	3	89838763	89838785	5.75	ENSSSCT00000009193	.	intron	-0.117	372962 rs7905	2
AG.CT	3	89943980	89944000	10.5	.	.	intergenic	0.141	.	2
AC.GT	3	90139903	90139923	10.5	ENSSSCT00000009194	EFEMP1	intron	1.064	.	3
AC.GT	3	90191165	90191199	17.5	ENSSSCT00000009194	EFEMP1	intron	0.271	rs789236620	3
AAAT.ATTT	3	90750562	90750582	5.25	ENSSSCT00000009201	MTIF2	intron	2.367	rs790305446	2
AAAT.ATTT	3	90775286	90775300	3.75	ENSSSCT00000026044	RPS27A	intron	1.64	rs793701981	2
AT.AT	3	90805609	90805621	6.5	ENSSSCT00000009199	CLHC1	intron	.	rs789469141	3
AAAAC.GTTTT	3	90844289	90844325	7.4	.	.	intergenic	-0.009	rs786310564	2
ACC.GGT	3	90884328	90884347	6.66667	.	.	intergenic	.	rs789801513	3
AAT.ATT	3	91147213	91147230	6	ENSSSCT00000009205	RTN4	intron	0.04	rs789224129	2
AAT.ATT	3	91147213	91147230	6	ENSSSCT00000032131	EML6	intron	0.04	rs789224129	2
AAC.GTT	3	91265987	91265999	4.33333	.	.	intergenic	0.119	rs787617788	6
AAAAC.GTTTT	3	91285285	91285303	3.8	.	.	intergenic	.	rs789344168	2
AAT.ATT	3	91331105	91331123	6.33333	.	.	intergenic	0.206	.	2
AT.AT	3	91384670	91384679	5	.	.	intergenic	0.207	rs787288524	2
ATC.GAT	3	91728654	91728671	6	.	.	intergenic	0.282	rs789650928	2
AAAAC.GTTTT	3	91786566	91786583	3.6	.	.	intergenic	.	102647 rs7923	2
AAT.ATT	3	91866725	91866738	4.66667	.	.	intergenic	.	.	2
AATT.AATT	3	92030613	92030631	4.75	.	.	intergenic	-0.154	402176 rs7891	2
AG.CT	3	92058356	92058367	6	.	.	intergenic	-0.097	rs792041475	2
AC.GT	3	92059526	92059538	6.5	.	.	intergenic	0.055	392783 rs7873	2
AG.CT	3	92073844	92073858	7.5	ENSSSCT00000023995	GPR115	intron	0.82	rs792207116	2
AT.AT	3	92239168	92239178	5.5	.	.	intergenic	0.225	rs789633216	4
AT.AT	3	92249093	92249116	12	.	.	intergenic	.	rs793461866	2
AC.GT	3	92297569	92297595	13.5	.	.	intergenic	.	rs786646402	2
AG.CT	3	92822863	92822873	5.5	.	.	intergenic	0.232	.	2
AAAT.ATTT	3	92959695	92959721	6.75	.	.	intergenic	.	rs790475272	2
AAAAC.GTTTT	3	92991737	92991757	4.2	.	.	intergenic	.	rs792517012	2
AAAAT.ATTTT	3	92992482	92992499	3.6	.	.	intergenic	.	rs789309032	2
AAAC.GTTT	3	93009106	93009117	3	.	.	intergenic	-0.048	rs788469028	2

AG.CT	3	93038465	93038475	5.5	.	.	intergenic	.	rs791461811	3
AAT.ATT	3	93169330	93169350	7	.	.	intergenic	.	383556 rs7896	2
AG.CT	3	93301112	93301121	5	.	.	intergenic	-0.08	rs690243232	3
AC.GT	3	93382452	93382466	7.5	.	.	intergenic	0.128	.	4
AAT.ATT	3	93741993	93742010	6	.	.	intergenic	.	.	2
AC.GT	3	93796978	93796988	5.5	.	.	intergenic	.	rs790502528	2
AAATT.AATTT	3	93821052	93821068	3.4	.	.	intergenic	-0.092	rs793434337	2
AT.AT	3	94168685	94168701	8.5	.	.	intergenic	-0.118	.	2
AC.GT	3	94254656	94254667	6	.	.	intergenic	-0.031	.	3
AAAT.ATTT	3	94499870	94499885	4	.	.	intergenic	0.04	rs787542236	2
AAAT.ATTT	3	94738822	94738844	5.75	.	.	intergenic	.	rs789390028	2
AAAC.GTTT	3	94821267	94821285	4.75	.	.	intergenic	0.094	rs788624615	2
AAC.GTT	3	94850873	94850886	4.66667	.	.	intergenic	0.012	rs790005002	2
AAAAC.GTTTT	3	94885149	94885182	6.8	.	.	intergenic	-0.008	rs789599268	2
AAAC.GTTT	3	94908378	94908405	7	.	.	intergenic	.	344109 rs7914	2
AAT.ATT	3	95051133	95051149	5.66667	.	.	intergenic	-0.09	rs792533571	2
AC.GT	3	95062358	95062398	20.5	.	.	intergenic	0.232	rs789248421	4
AATG.CATT	3	95870024	95870046	5.75	.	.	intergenic	0.045	rs789835797	2
ATC.GAT	3	95935225	95935237	4.33333	.	.	intergenic	-0.047	rs790287793	2
AAAAC.GTTTT	3	95949724	95949745	4.4	.	.	intergenic	0.061	rs792066476	2
AAAG.CTTT	3	96056440	96056459	5	.	.	intergenic	-0.083	138697 rs7885	2
AC.GT	3	96061206	96061219	7	.	.	intergenic	-0.047	rs792926568	3
AAAAC.GTTTT	3	96236443	96236463	4.2	.	.	intergenic	.	.	2
AC.GT	3	96382811	96382821	5.5	.	.	intergenic	0.148	rs709029079	4
AAAT.ATTT	3	96707470	96707495	6.5	.	.	intergenic	.	589858 rs7932	2
AAAC.GTTT	3	96873280	96873299	5	.	.	intergenic	.	428124 rs7881	2
AG.CT	3	97202655	97202671	8.5	.	.	intergenic	-0.102	rs789506942	3
AAAC.GTTT	3	97227298	97227314	4.25	.	.	intergenic	0.036	rs703902661	2
AT.AT	3	97284859	97284868	5	.	.	intergenic	.	rs792158256	2
AT.AT	3	97380372	97380381	5	.	.	intergenic	-0.105	rs787714148	3
AAAC.GTTT	3	97392899	97392930	8	.	.	intergenic	.	.	2
AT.AT	3	97477799	97477808	5	.	.	intergenic	-0.627	rs788961049	2
AC.GT	3	97539645	97539655	5.5	ENSSSCT00000024756	FSHR	intron	0.052	rs789055584	2
AAAC.GTTT	3	97595684	97595702	4.75	.	.	intergenic	-0.108	rs789684427	2
AAC.GTT	3	97608006	97608025	6.66667	.	.	intergenic	0.201	rs793335022	2
AC.GT	3	97616356	97616367	6	.	.	intergenic	-0.049	rs786243770	2
AAAT.ATTT	3	97918517	97918545	7.25	.	.	intergenic	0.051	rs786685108	2
AAAG.CTTT	3	98033243	98033265	5.75	ENSSSCT00000009224	PPP1R21	intron	0.179	rs790053748	2
AC.GT	3	98141031	98141043	6.5	ENSSSCT00000025707	FOXN2	intron	0.825	968251 rs7868	2
AAAG.CTTT	3	98473106	98473117	3	.	.	intergenic	-0.024	rs793586692	2
AAC.GTT	3	98509532	98509545	4.66667	.	.	intergenic	0.149	rs692834837	2
AAAC.GTTT	3	98788793	98788816	6	.	.	intergenic	.	rs790207494	2
AAAAAT.ATTTTT	3	98802086	98802108	3.83333	.	.	intergenic	.	.	2
ACTG.CAGT	3	98825336	98825355	5	.	.	intergenic	0.05	.	2
AAAG.CTTT	3	99290273	99290287	3.75	ENSSSCT00000009229	.	intron	.	rs791910003	2
AG.CT	3	99422179	99422191	6.5	.	.	intergenic	0	587720 rs7878	2
AAAC.GTTT	3	99486070	99486089	5	.	.	intergenic	0.047	rs790468781	2
AAAC.GTTT	3	99738233	99738247	3.75	.	.	intergenic	0.027	rs788387164	2
AATG.CATT	3	99869497	99869517	5.25	.	.	intergenic	0.484	rs788166857	2
AT.AT	3	99984366	99984376	5.5	.	.	intergenic	0.03	rs786669131	2
AAAT.ATTT	3	100029898	100029918	5.25	.	.	intergenic	0.055	rs790949526	2
AAAAC.GTTTT	3	100076823	100076846	4.8	ENSSSCT00000009240	RHOQ	intron	-0.065	rs789807451	2
AC.GT	3	100189729	100189747	9.5	ENSSSCT00000009245	EPAS1	intron	0.335	.	2
AAAAG.CTTTT	3	100363722	100363744	4.6	.	.	intergenic	0.302	.	2
AGC.GCT	3	100481749	100481764	5.33333	ENSSSCT00000009246	PRKCE	intron	0.245	rs790495579	2
AAT.ATT	3	100691855	100691869	5	.	.	intergenic	0.056	.	2
AC.GT	3	100775687	100775697	5.5	.	.	intergenic	0.119	.	2
AATC.GATT	3	100901653	100901686	8.5	.	.	intergenic	0.06	rs789667161	2
AAAC.GTTT	3	101066847	101066863	4.25	ENSSSCT00000009247	SRBD1	intron	0.188	rs789899037	2
AAAT.ATTT	3	101200450	101200468	4.75	ENSSSCT00000009247	SRBD1	intron	0.25	rs793445862	2
AAAAG.CTTTT	3	101736530	101736557	5.6	.	.	intergenic	.	rs789132833	2
AT.AT	3	102209333	102209344	6	.	.	intergenic	0.175	)59880 rs7888	2
AAC.GTT	3	102249691	102249708	6	ENSSSCT00000009249	.	promoter	0.233	rs789002244	2
AAC.GTT	3	102249691	102249708	6	ENSSSCT00000009250	PREPL	promoter	0.233	rs789002244	2
AAAT.ATTT	3	102289615	102289650	9	ENSSSCT00000009250	PREPL	intron	.	525016 rs7870	2
AAAAC.GTTTT	3	102337096	102337127	6.4	ENSSSCT00000009251	.	intron	-0.109	727639 rs7915	2
AACTC.GAGTT	3	102386564	102386587	4	.	.	intergenic	0.099	rs791568739	2
AAAAC.GTTTT	3	102480066	102480083	3.6	.	.	intergenic	0.483	rs790396945	2
AAC.GTT	3	102552237	102552251	5	.	.	intergenic	0.135	562562 rs7920	2
ATC.GAT	3	103076951	103076968	6	ENSSSCT00000032410	.	intron	0.53	207850 rs7913	2
AATG.CATT	3	103406215	103406236	5.5	.	.	intergenic	0.049	.	2
AG.CT	3	103493723	103493732	5	.	.	intergenic	0.166	rs788688462	2
AACT.AGTT	3	103511676	103511698	5.75	.	.	intergenic	-0.13	517665 rs7934	2
AAAC.GTTT	3	103869609	103869627	4.75	.	.	intergenic	-0.032	rs790908674	2
AAAC.GTTT	3	104245736	104245758	5.75	.	.	intergenic	0.102	rs789695645	2
AT.AT	3	104294739	104294750	6	.	.	intergenic	.	.	4
AAAAT.ATTTT	3	104472558	104472575	3.6	.	.	intergenic	.	364579 rs7909	2
AAAC.GTTT	3	104568463	104568486	6	.	.	intergenic	0.016	rs789355509	2
AAAC.GTTT	3	104866090	104866108	4.75	.	.	intergenic	0.153	703802 rs7895	2
AC.GT	3	104908825	104908849	12.5	.	.	intergenic	0.151	rs793302767	4
AT.AT	3	104941655	104941664	5	.	.	intergenic	0.121	.	3
AC.GT	3	104990350	104990365	8	.	.	intergenic	.	rs787511400	4
AC.GT	3	105087291	105087301	5.5	.	.	intergenic	.	rs788886211	3
AAAC.GTTT	3	105193695	105193712	4.5	.	.	intergenic	.	rs792704484	3

AAAT.ATTT	3	105411821	105411853	8.25	.	.	intergenic	-0.133	rs792489256	2
AAGC.GCTT	3	105530213	105530245	8.25	.	.	intergenic	0	rs792574275	2
AAAT.ATTT	3	105788919	105788936	4.5	.	.	intergenic	-0.077	rs788244502	3
AAAC.GTTT	3	105856964	105856982	4.75	.	.	intergenic	-0.091	rs791319280	2
AC.GT	3	105864313	105864322	5	.	.	intergenic	.	.	2
AAAC.GTTT	3	105913340	105913366	6.75	.	.	intergenic	.	rs787862840	2
AAAC.GTTT	3	105973682	105973702	5.25	.	.	intergenic	0.107	rs789928947	2
AC.GT	3	105990104	105990121	9	.	.	intergenic	0.028	'28899 rs7936	3
AC.GT	3	105992555	105992590	18	.	.	intergenic	-0.006	.	2
AAAC.GTTT	3	106316384	106316406	5.75	.	.	intergenic	0.187	.	2
AAAAC.GTTTT	3	106352575	106352597	4.6	.	.	intergenic	-0.088	rs786643072	2
AAAAT.ATTTT	3	106358785	106358806	4.4	.	.	intergenic	0.043	rs791687705	2
AAC.GTT	3	106511575	106511589	5	.	.	intergenic	.	.	3
AGAGC.GCTCT	3	106524840	106524859	4	.	.	intergenic	-0.326	rs789906711	2
AC.GT	3	106528156	106528165	5	.	.	intergenic	.	rs787039751	2
AAAAC.GTTTT	3	106574091	106574119	5.8	.	.	intergenic	.	rs702072022	2
AG.CT	3	106853830	106853839	5	.	.	intergenic	0.038	.	2
AAAAC.GTTTT	3	107087927	107087947	4.2	ENSSSCT00000009277	MAP4K3	promoter	0.268	rs788315107	2
AAAG.CTTT	3	107095516	107095534	4.75	ENSSSCT00000009277	MAP4K3	intron	0.272	rs788109267	2
AG.CT	3	107551491	107551500	5	ENSSSCT00000009283	.	promoter	0.859	rs790065722	2
AAC.GTT	3	107739193	107739205	4.33333	.	.	intergenic	.	rs792584983	2
AAC.GTT	3	107834360	107834382	7.66667	ENSSSCT00000009287	.	promoter	.	)71912 rs7898	2
ATAT.ATAT	3	107950288	107950321	8.5	.	.	intergenic	0.161	.	2
AAGG.CCTT	3	108319107	108319137	7.75	.	.	intergenic	.	.	2
AAT.ATT	3	108363079	108363092	4.66667	.	.	intergenic	0.029	.	3
AAAC.GTTT	3	108955258	108955282	6.25	ENSSSCT00000023650	.	intron	0.002	388866 rs7918	2
AC.GT	3	109027289	109027298	5	.	.	intergenic	0.262	rs791273970	3
AAAC.GTTT	3	109476738	109476756	4.75	ENSSSCT00000009297	PRKD3	intron	0.263	'95575 rs7923	2
AAAAT.ATTTT	3	109926962	109926979	3.6	ENSSSCT00000009301	STRN	intron	0.075	rs787165808	2
AAAG.CTTT	3	110179747	110179766	5	.	.	intergenic	0.126	363085 rs7929	2
AT.AT	3	110667331	110667344	7	.	.	intergenic	0.132	rs703982573	3
AAAC.GTTT	3	110825093	110825107	3.75	.	.	intergenic	0.231	307534 rs7877	2
AAAAG.CTTTT	3	110844568	110844585	3.6	.	.	intergenic	.	rs792743928	2
AAAAT.ATTTT	3	111031908	111031922	3	.	.	intergenic	0.476	rs793192195	2
AAT.ATT	3	111157469	111157480	4	.	.	intergenic	0.103	rs792663277	2
AAC.GTT	3	111229064	111229077	4.66667	.	.	intergenic	0.07	rs789993675	2
AAAC.GTTT	3	111369369	111369382	3.5	.	.	intergenic	-0.117	rs792307199	2
AT.AT	3	111497197	111497234	19	.	.	intergenic	0.121	270239 rs7876	2
AT.AT	3	111565594	111565607	7	.	.	intergenic	0.059	rs789837748	2
AAC.GTT	3	111666144	111666160	5.66667	.	.	intergenic	0.047	244353 rs7934	2
AAG.CTT	3	111680705	111680729	8.33333	.	.	intergenic	-0.006	rs792552942	2
AAC.GTT	3	111968292	111968304	4.33333	.	.	intergenic	0.393	rs790477926	2
AG.CT	3	111989953	111989967	7.5	.	.	intergenic	0.021	rs786408214	2
AAAT.ATTT	3	112005531	112005553	5.75	.	.	intergenic	0.027	.	2
AAAC.GTTT	3	112024264	112024282	4.75	.	.	intergenic	-0.049	rs792593944	2
AT.AT	3	112144351	112144361	5.5	.	.	intergenic	0.134	rs792180771	2
AAAAT.ATTTT	3	112228843	112228865	4.6	.	.	intergenic	0.102	rs788206993	2
AAAC.GTTT	3	112523914	112523928	3.75	.	.	intergenic	.	'23510 rs7911	2
AT.AT	3	112550058	112550074	8.5	.	.	intergenic	0.017	rs787589739	3
AG.CT	3	112713735	112713750	8	.	.	intergenic	.	rs790549863	5
AAAAC.GTTTT	3	112920243	112920270	5.6	.	.	intergenic	0.078	413289 rs7893	2
AT.AT	3	113170222	113170231	5	.	.	intergenic	.	rs787135092	2
AG.CT	3	113347669	113347681	6.5	ENSSSCT00000009313	LTBP1	intron	-0.125	.	3
AC.GT	3	113353630	113353643	7	ENSSSCT00000009313	LTBP1	intron	0.508	rs789834603	2
AAG.CTT	3	113362239	113362255	5.66667	ENSSSCT00000009313	LTBP1	intron	0.009	333849 rs7875	2
AAG.CTT	3	113719495	113719511	5.66667	.	.	intergenic	0.001	rs787910259	2
AC.GT	3	113772474	113772489	8	ENSSSCT00000023086	.	intron	-0.01	rs790622178	2
AAAT.ATTT	3	113934562	113934588	6.75	ENSSSCT00000009315	.	intron	0.138	rs790699632	2
AAAG.CTTT	3	113967693	113967706	3.5	ENSSSCT00000009316	BIRC6	intron	2.917	rs789844429	2
AAAG.CTTT	3	114326392	114326409	4.5	ENSSSCT00000009319	MEMO1	intron	0.778	rs790308698	2
AAC.GTT	3	114395179	114395192	4.66667	ENSSSCT00000009319	MEMO1	intron	0.275	rs791115920	2
AAAG.CTTT	3	114448985	114448997	3.25	.	.	intergenic	0.211	rs793763879	2
AAAAT.ATTTT	3	114554112	114554128	3.4	.	.	intergenic	.	rs786495265	2
ACAT.ATGT	3	114580897	114580915	4.75	.	.	intergenic	-0.07	299827 rs7905	2
AG.CT	3	114713275	114713288	7	.	.	intergenic	0.095	rs789128218	2
AAT.ATT	3	114730996	114731007	4	ENSSSCT00000009326	XDH	promoter	-0.008	rs789609981	2
AATG.CATT	3	114778622	114778645	6	ENSSSCT00000009326	XDH	intron	.	rs788772922	2
AG.CT	3	114812835	114812851	8.5	.	.	intergenic	-0.14	rs790327382	2
AG.CT	3	114972396	114972410	7.5	.	.	intergenic	.	.	3
AG.CT	3	114982019	114982030	6	.	.	intergenic	0.058	.	2
ACGC.GCGT	3	115856605	115856620	4	.	.	intergenic	-0.026	)79753 rs7924	2
AG.CT	3	116344049	116344058	5	.	.	intergenic	0.007	)39087 rs7876	3
AAAC.GTTT	3	116398272	116398292	5.25	ENSSSCT00000036644	ALK	intron	-0.023	rs793508544	2
AAGG.CCTT	3	116413659	116413692	8.5	.	.	intergenic	.	289254 rs7899	2
AAAAC.GTTTT	3	116507623	116507663	8.2	.	.	intergenic	-0.03	rs789943936	2
AAC.GTT	3	116536276	116536289	4.66667	.	.	intergenic	0.204	rs788274998	2
AG.CT	3	116861255	116861269	7.5	ENSSSCT00000033256	ALK	intron	-0.017	rs790144974	2
AC.GT	3	117119513	117119525	6.5	.	.	intergenic	-0.051	.	3
AAAAC.GTTTT	3	117195368	117195390	4.6	.	.	intergenic	0.115	rs793679517	2
AAAC.GTTT	3	117601371	117601399	7.25	.	.	intergenic	.	rs788740619	2
AC.GT	3	117952380	117952401	11	.	.	intergenic	-0.2	rs792763065	4
ACG.CGT	3	118077532	118077545	4.66667	ENSSSCT00000023126	BRE	intron	0.246	rs789726253	2
AC.GT	3	118131660	118131669	5	.	.	intergenic	0.159	.	2
AGG.CCT	3	118461440	118461458	6.33333	.	.	intergenic	0.678	171846 rs7885	2

AC.GT	3	119188657	119188675	9.5	ENSSSCT00000009365	DPYSL5	intron	0.207	.	4
AATG.CATT	3	119472662	119472703	10.5	ENSSSCT00000009371	C2orf70	intron	-0.097	229262 rs7885	3
AAAC.GTTT	3	119508390	119508407	4.5	.	.	intergenic	0.051	rs790518947	2
AAAC.GTTT	3	119524595	119524609	3.75	.	.	intergenic	-0.016	rs789521871	2
AAC.GTT	3	120083285	120083304	6.66667	ENSSSCT00000009382	ASXL2	intron	0.159	rs789547809	2
AC.GT	3	120133140	120133153	7	ENSSSCT00000009382	ASXL2	intron	.	rs789944333	2
AG.CT	3	120165616	120165626	5.5	ENSSSCT00000009382	ASXL2	intron	0.218	548950 rs7901	2
AC.GT	3	120261106	120261115	5	ENSSSCT00000009384	DTNB	intron	0.208	rs790822480	6
AAAC.GTTT	3	120837619	120837642	6	ENSSSCT00000031886	.	intron	.	rs791129439	2
AT.AT	3	121038000	121038018	9.5	ENSSSCT00000009386	DNAJC27	intron	1.097	rs790616895	3
AT.AT	3	121134303	121134318	8	ENSSSCT00000009387	ADCY3	intron	.	rs790773869	4
AAC.GTT	3	121387560	121387585	8.66667	.	.	intergenic	0.252	356908 rs7930	2
AAC.GTT	3	121533145	121533159	5	.	.	intergenic	0.163	rs787658730	2
AG.CT	3	121785949	121785963	7.5	ENSSSCT00000009392	FAM228A	intron	.	.	2
AAAAC.GTTTT	3	121872592	121872613	4.4	ENSSSCT00000009397	.	intron	0.194	.	2
AC.GT	3	121901728	121901743	8	ENSSSCT00000009399	C2orf44	intron	0.202	256014 rs7873	4
AC.GT	3	121965790	121965802	6.5	.	.	intergenic	.	.	4
AG.CT	3	122031644	122031658	7.5	ENSSSCT00000009401	.	intron	-0.261	rs792053086	4
AT.AT	3	122047557	122047567	5.5	.	.	intergenic	0.218	rs787552140	2
AAACC.GGTTT	3	122318227	122318249	4.6	.	.	intergenic	0.051	530633 rs7913	3
AG.CT	3	122438123	122438135	6.5	.	.	intergenic	0.912	rs786541883	2
ACAT.ATGT	3	122699663	122699698	9	.	.	intergenic	0.065	rs790388440	2
AAC.GTT	3	122751933	122751958	8.66667	.	.	intergenic	0.078	rs787861805	2
AT.AT	3	122873130	122873143	7	.	.	intergenic	0.462	rs787084816	2
AAG.CTT	3	122926658	122926679	7.33333	.	.	intergenic	0.078	.	2
ACAG.CTGT	3	123121193	123121215	5.75	.	.	intergenic	.	167893 rs7900	2
AAAC.GTTT	3	123300628	123300642	3.75	.	.	intergenic	0.051	rs789029189	2
AC.GT	3	123757249	123757259	5.5	.	.	intergenic	0.04	rs793642645	2
AAAT.ATTT	3	123758705	123758718	3.5	.	.	intergenic	0.243	rs788357131	2
AG.CT	3	123778460	123778469	5	.	.	intergenic	-0.17	.	2
AC.GT	3	124019262	124019274	6.5	.	.	intergenic	.	rs789944526	2
AG.CT	3	124076413	124076422	5	.	.	intergenic	-0.192	345479 rs7937	2
AAT.ATT	3	124127943	124127954	4	.	.	intergenic	0.064	.	2
AAT.ATT	3	124189769	124189785	5.66667	.	.	intergenic	.	.	2
AT.AT	3	124245076	124245091	8	.	.	intergenic	.	.	2
AG.CT	3	124370867	124370878	6	.	.	intergenic	-0.019	.	2
AC.GT	3	124648011	124648022	6	.	.	intergenic	.	rs791596732	2
AAC.GTT	3	124761245	124761256	4	.	.	intergenic	0.268	rs792264894	2
AAT.ATT	3	124876024	124876037	4.66667	.	.	intergenic	.	rs786733326	5
AACCC.GGGTT	3	125263024	125263040	3.4	.	.	intergenic	.	rs787373171	2
AAAC.GTTT	3	125332387	125332401	3.75	.	.	intergenic	.	.	2
AAAC.GTTT	3	125447926	125447944	4.75	ENSSSCT00000025009	C2orf43	intron	.	rs788632839	2
AAAC.GTTT	3	125447926	125447944	4.75	ENSSSCT00000023215	C2orf43	intron	.	rs788632839	2
AT.AT	3	125462389	125462399	5.5	ENSSSCT00000023215	C2orf43	intron	0.271	407566 rs7912	3
AT.AT	3	125462389	125462399	5.5	ENSSSCT00000025009	C2orf43	intron	0.271	407566 rs7912	3
AT.AT	3	125656268	125656278	5.5	.	.	intergenic	0.276	rs792382431	3
AC.GT	3	125744563	125744577	7.5	.	.	intergenic	0.078	rs787649374	3
AG.CT	3	125812973	125812985	6.5	.	.	intergenic	.	rs786508856	2
AAT.ATT	3	126107665	126107680	5.33333	.	.	intergenic	-0.147	rs790888831	2
AC.GT	3	126278559	126278575	8.5	ENSSSCT00000009415	WDR35	intron	.	.	2
AC.GT	3	126278559	126278575	8.5	ENSSSCT00000031965	WDR35	intron	.	.	2
AAC.GTT	3	126281801	126281816	5.33333	ENSSSCT00000009415	WDR35	intron	.	rs793101570	2
AAC.GTT	3	126281801	126281816	5.33333	ENSSSCT00000031965	WDR35	intron	.	rs793101570	2
AAAT.ATTT	3	126436786	126436806	5.25	.	.	intergenic	0.061	rs793381164	2
AC.GT	3	126560242	126560256	7.5	.	.	intergenic	.	.	4
AC.GT	3	126588273	126588297	12.5	.	.	intergenic	1.714	.	2
AAAC.GTTT	3	126751910	126751930	5.25	.	.	intergenic	0.043	rs787295211	3
AAAC.GTTT	3	126784091	126784105	3.75	.	.	intergenic	-0.111	rs791047494	2
AG.CT	3	126785713	126785742	15	.	.	intergenic	0.133	rs790664457	2
AAAT.ATTT	3	126861055	126861082	7	.	.	intergenic	.	rs793139242	2
AAT.ATT	3	126944132	126944155	8	.	.	intergenic	-0.04	.	4
AT.AT	3	127052509	127052518	5	.	.	intergenic	-0.087	.	2
AC.GT	3	127080472	127080482	5.5	.	.	intergenic	-0.187	rs791624296	2
AAAAC.GTTTT	3	127186856	127186876	4.2	.	.	intergenic	-0.264	rs789191765	2
AT.AT	3	127190487	127190496	5	.	.	intergenic	0.122	rs788043692	2
AGAT.ATCT	3	127226787	127226814	7	.	.	intergenic	-0.035	rs790031624	2
ATCC.GGAT	3	127226911	127226925	3.75	.	.	intergenic	0.054	rs787365795	2
AAAG.CTTT	3	127232019	127232032	3.5	.	.	intergenic	0.056	246655 rs7897	2
AAAC.GTTT	3	127310051	127310075	6.25	.	.	intergenic	-0.047	rs788953389	2
AT.AT	3	127715759	127715768	5	.	.	intergenic	.	rs788954530	2
AAAAC.GTTTT	3	127740983	127741005	4.6	.	.	intergenic	.	rs792538814	2
AAAC.GTTT	3	127817520	127817550	7.75	.	.	intergenic	.	rs787100207	2
AT.AT	3	128211693	128211710	9	.	.	intergenic	.	rs793669856	4
AAAC.GTTT	3	128268786	128268800	3.75	ENSSSCT00000009425	SMC6	intron	1.117	rs786957286	2
AC.GT	3	128319815	128319829	7.5	.	.	intergenic	0.117	.	2
AAAACC.GGTTT	3	128439038	128439058	3.5	.	.	intergenic	-0.106	.	2
AG.CT	3	128472156	128472173	9	ENSSSCT00000024717	RAD51AP2	intron	0.141	055842 rs7886	2
AT.AT	3	128561180	128561189	5	.	.	intergenic	.	rs789162099	2
AG.CT	3	128633208	128633219	6	.	.	intergenic	0.375	rs787215421	3
AAAC.GTTT	3	128961407	128961425	4.75	.	.	intergenic	-0.277	rs792732448	2
AC.GT	3	129007138	129007149	6	.	.	intergenic	-0.055	rs709790272	2
AAAAT.ATTTT	3	129037124	129037158	7	.	.	intergenic	0.229	rs792531755	2
AAAAC.GTTTT	3	129208988	129209006	3.8	.	.	intergenic	0.044	rs789206430	2
AAAAAC.GTTTTT	3	129292867	129292891	4.16667	ENSSSCT00000009428	FAM49A	intron	-0.161	267447 rs7885	2

AC.GT	3	129372691	129372703	6.5	.	.	intergenic	-0.086	rs791214230	2
AC.GT	3	129430504	129430518	7.5	.	.	intergenic	-0.142	rs789337606	2
ACTC.GAGT	3	129619945	129619965	5.25	.	.	intergenic	-0.241	278398 rs7933	2
AAAT.ATTT	3	129948506	129948524	4.75	.	.	intergenic	-0.087	rs792856937	2
AAAC.GTTT	3	130026412	130026430	4.75	.	.	intergenic	0.087	229117 rs7914	2
AATG.CATT	3	130067626	130067655	7.5	.	.	intergenic	-0.338	rs792027126	3
AAAAT.ATTTT	3	130122335	130122354	4	.	.	intergenic	-0.29	296634 rs7893	2
AAAATG.CATTT	3	130627568	130627588	3.5	ENSSSCT00000009431	.	intron	0.437	540320 rs7874	2
AAAAAG.CTTTT	3	131204417	131204439	3.83333	.	.	intergenic	.	rs787371550	2
AATC.GATT	3	131333359	131333385	6.75	.	.	intergenic	-0.01	rs788599680	2
AAT.ATT	3	131337772	131337783	4	.	.	intergenic	.	rs786322765	2
AT.AT	3	131351645	131351656	6	.	.	intergenic	0.212	rs793272376	2
AC.GT	3	131464896	131464906	5.5	.	.	intergenic	.	.	3
AT.AT	3	131763273	131763287	7.5	.	.	intergenic	-0.025	rs788840906	2
AT.AT	3	131822391	131822402	6	.	.	intergenic	-0.163	rs789016264	2
AAAC.GTTT	3	131838731	131838750	5	.	.	intergenic	.	rs792408509	2
AAAC.GTTT	3	131975669	131975691	5.75	.	.	intergenic	-0.09	365019 rs7924	2
AG.CT	3	132236959	132236982	12	.	.	intergenic	.	.	4
AC.GT	3	132279645	132279669	12.5	.	.	intergenic	.	rs788854088	2
AAAC.GTTT	3	132333631	132333655	6.25	.	.	intergenic	-0.123	732352 rs7888	2
AC.GT	3	132336638	132336650	6.5	.	.	intergenic	0.13	.	2
AAAAC.GTTTT	3	132441791	132441814	4.8	.	.	intergenic	-0.416	rs789871868	2
AAC.GTT	3	132457949	132457965	5.66667	.	.	intergenic	-0.195	480335 rs7924	2
AT.AT	3	132524354	132524371	9	.	.	intergenic	0.06	rs789988741	2
AT.AT	3	132730905	132730921	8.5	.	.	intergenic	.	rs786593200	2
AC.GT	3	132738006	132738033	14	.	.	intergenic	-0.058	rs788781721	2
AAG.CTT	3	132835008	132835021	4.66667	.	.	intergenic	-0.129	.	2
AC.GT	3	132845324	132845341	9	.	.	intergenic	-0.001	151958 rs7926	3
AAAT.ATTT	3	132865487	132865505	4.75	.	.	intergenic	.	.	2
AG.CT	3	132893408	132893424	8.5	.	.	intergenic	-0.145	rs788400116	2
AAAT.ATTT	3	132904048	132904061	3.5	.	.	intergenic	-0.248	rs788879585	2
AG.CT	3	133509451	133509463	6.5	.	.	intergenic	-0.169	rs787663056	4
ACC.GGT	3	133544992	133545005	4.66667	.	.	intergenic	-0.047	.	2
AAC.GTT	3	134252919	134252933	5	ENSSSCT00000009440	ROCK2	intron	3.313	rs793403816	2
AAAC.GTTT	3	134607641	134607657	4.25	.	.	intergenic	.	rs790213134	3
AG.CT	3	134637254	134637270	8.5	ENSSSCT00000009447	NOL10	intron	1.267	rs787363649	2
AC.GT	3	135499540	135499550	5.5	.	.	intergenic	-0.098	.	2
AC.GT	3	135676870	135676882	6.5	ENSSSCT00000026647	MBOAT2	intron	0.082	rs788289728	2
AAAC.GTTT	3	136064437	136064460	6	.	.	intergenic	-0.02	719610 rs7891	2
AAAAC.GTTTT	3	136719095	136719118	4.8	.	.	intergenic	-0.105	rs788816284	2
AAAC.GTTT	3	136766002	136766018	4.25	.	.	intergenic	-0.366	389492 rs7933	2
AAAAC.GTTTT	3	137761441	137761459	3.8	.	.	intergenic	0.159	rs792594308	2
AG.CT	3	138158994	138159003	5	.	.	intergenic	-0.37	.	2
AC.GT	3	138541310	138541320	5.5	.	.	intergenic	.	273264 rs6996	4
AAT.ATT	3	138760284	138760312	9.66667	.	.	intergenic	-0.172	rs789077091	2
AAAC.GTTT	3	138957218	138957237	5	.	.	intergenic	.	rs793001339	2
AT.AT	3	139002197	139002206	5	.	.	intergenic	.	rs790582314	2
AC.GT	3	139129714	139129724	5.5	.	.	intergenic	.	rs793241759	2
AACT.AGTT	3	139302652	139302673	5.5	.	.	intergenic	-0.098	483905 rs7924	2
AATG.CATT	3	139628663	139628686	6	.	.	intergenic	-0.237	357544 rs7929	2
AC.GT	3	139716374	139716390	8.5	.	.	intergenic	-0.471	.	4
AAT.ATT	3	140069320	140069334	5	.	.	intergenic	-0.124	186471 rs7922	3
AAGG.CCTT	3	140135343	140135365	5.75	.	.	intergenic	-0.223	rs787667650	2
AC.GT	3	140338928	140338937	5	.	.	intergenic	-0.3	rs789614301	3
AAAT.ATTT	3	140365107	140365127	5.25	ENSSSCT00000009464	DCDC2C	intron	0.549	428727 rs7893	2
AAAAT.ATTTT	3	141048960	141048984	5	.	.	intergenic	-0.304	741787 rs7877	2
AAAAT.ATTTT	3	141116010	141116029	4	.	.	intergenic	0.168	136465 rs7876	2
AAAT.ATTT	3	141226210	141226229	5	.	.	intergenic	-0.713	rs790150722	4
AC.GT	3	143735764	143735813	25	.	.	intergenic	0.485	.	5
AAAC.GTTT	3	144162775	144162797	5.75	.	.	intergenic	.	560756 rs7899	2
AAAC.GTTT	4	1119405	1119426	5.5	.	.	intergenic	.	rs786444172	2
AAAC.GTTT	4	1791485	1791503	4.75	.	.	intergenic	-0.135	rs787535543	2
AG.CT	4	2381826	2381838	6.5	ENSSSCT00000006516	AGO2	intron	.	964148 rs7913	2
AT.AT	4	3035800	3035816	8.5	.	.	intergenic	0.007	352362 rs7936	4
AC.GT	4	3433760	3433771	6	.	.	intergenic	.	.	2
AG.CT	4	4275361	4275379	9.5	ENSSSCT00000034533	FAM135B	intron	-0.216	rs702488221	2
AG.CT	4	4275361	4275379	9.5	ENSSSCT00000006522	FAM135B	intron	-0.216	rs702488221	2
AG.CT	4	4529041	4529053	6.5	.	.	intergenic	-0.282	rs789481578	2
ACACC.GGTGT	4	4588547	4588569	4.6	.	.	intergenic	-0.114	546588 rs7901	2
AT.AT	4	4685724	4685742	9.5	.	.	intergenic	-0.282	rs787328281	2
AG.CT	4	4709654	4709664	5.5	.	.	intergenic	-0.443	rs786839524	2
AG.CT	4	4803051	4803065	7.5	.	.	intergenic	-0.256	rs787469393	3
AAAGC.GCTTT	4	5018758	5018774	3.4	.	.	intergenic	-0.207	.	2
AC.GT	4	5061699	5061717	9.5	.	.	intergenic	-0.223	rs789256918	3
AAT.ATT	4	5255251	5255263	4.33333	.	.	intergenic	-0.084	rs793032314	2
AC.GT	4	5336241	5336256	8	.	.	intergenic	0.133	345528 rs7937	2
AG.CT	4	5379583	5379602	10	.	.	intergenic	-0.208	922220 rs7890	2
AC.GT	4	5486359	5486371	6.5	.	.	intergenic	-0.203	775467 rs7872	3
AC.GT	4	5490762	5490774	6.5	.	.	intergenic	-0.062	337345 rs7929	3
AAAAAC.GTTTT	4	5503508	5503529	3.66667	.	.	intergenic	-0.204	rs790701871	2
AT.AT	4	5511670	5511683	7	.	.	intergenic	-0.014	378333 rs7929	2
AAG.CTT	4	5648168	5648181	4.66667	.	.	intergenic	-0.328	rs786969173	6
AAT.ATT	4	5886377	5886392	5.33333	.	.	intergenic	-0.147	rs791844345	2
AC.GT	4	5905391	5905408	9	.	.	intergenic	-0.216	rs787707652	4

AC.GT	4	5936063	5936081	9.5	.	.	intergenic	-0.633	rs790595934	3
AC.GT	4	6025135	6025145	5.5	.	.	intergenic	-0.192	rs789333401	2
AAAAAT.ATTTTT	4	6083094	6083113	3.33333	.	.	intergenic	0.303	rs787268321	2
AAAC.GTTT	4	6180033	6180051	4.75	ENSSSCT00000006524	CU151851.1	intron	.	rs793747849	2
AAAC.GTTT	4	6180033	6180051	4.75	ENSSSCT00000036563	CU151851.1	intron	.	rs793747849	2
AAAC.GTTT	4	6180033	6180051	4.75	ENSSSCT00000035168	CU151851.1	intron	.	rs793747849	2
AC.GT	4	6242958	6242967	5	.	.	intergenic	-0.105	.	2
AAAC.GTTT	4	6324318	6324335	4.5	.	.	intergenic	0.05	rs792477147	2
AC.GT	4	6561317	6561326	5	.	.	intergenic	0.012	rs788433497	2
AC.GT	4	6948626	6948635	5	ENSSSCT00000033897	CU571095.1	intron	.	.	2
AC.GT	4	7077369	7077379	5.5	.	.	intergenic	0.078	.	4
AATG.CATT	4	7081076	7081099	6	.	.	intergenic	0.154	436563 rs7914	2
AGCCC.GGGCT	4	7435013	7435027	3	.	.	intergenic	-0.137	.	2
AGATAT.ATATCT	4	7447065	7447095	5.16667	.	.	intergenic	-0.391	rs793622545	2
AG.CT	4	7492546	7492556	5.5	.	.	intergenic	-0.144	054383 rs7903	3
AG.CT	4	7543767	7543784	9	.	.	intergenic	0.055	.	2
AAC.GTT	4	7812501	7812514	4.66667	.	.	intergenic	-0.124	rs789986688	2
AAAC.GTTT	4	8440418	8440435	4.5	ENSSSCT00000006533	PHF20L1	intron	-0.044	rs789081001	2
AAAC.GTTT	4	8529005	8529029	6.25	ENSSSCT00000006534	.	intron	0.028	409911 rs7892	2
AAAT.ATTT	4	8630675	8630693	4.75	ENSSSCT00000034663	LRRC6	intron	-0.282	rs793661939	2
AAAT.ATTT	4	8630675	8630693	4.75	ENSSSCT00000006535	LRRC6	intron	-0.282	rs793661939	2
AAC.GTT	4	8727804	8727820	5.66667	.	.	intergenic	-0.194	.	2
AAAC.GTTT	4	8810103	8810122	5	.	.	intergenic	-0.183	rs793300960	2
ATCC.GGAT	4	8954675	8954696	5.5	ENSSSCT00000029622	.	promoter	-0.108	rs789261847	2
AC.GT	4	8959633	8959652	10	.	.	intergenic	-0.118	rs786231170	3
AGG.CCT	4	8995151	8995169	6.33333	.	.	intergenic	-0.267	rs793454411	3
AAC.GTT	4	9888015	9888036	7.33333	.	.	intergenic	-0.063	rs793869607	2
AAAAC.GTTTT	4	10687472	10687492	4.2	.	.	intergenic	-0.115	.	2
AATG.CATT	4	10711512	10711528	4.25	.	.	intergenic	-0.264	rs787575676	2
AAAC.GTTT	4	10770069	10770087	4.75	.	.	intergenic	.	044205 rs7887	2
AAAAG.CTTTT	4	11079013	11079031	3.8	.	.	intergenic	-0.188	rs793054851	2
AAAC.GTTT	4	11094146	11094170	6.25	ENSSSCT00000006545	GSDMC	promoter	.	437492 rs7869	2
AGGG.CCCT	4	11264438	11264450	3.25	.	.	intergenic	0.023	rs790569505	2
AAAT.ATTT	4	11522729	11522750	5.5	.	.	intergenic	0.021	rs788927901	2
AAC.GTT	4	11535903	11535918	5.33333	.	.	intergenic	0.172	rs790709093	2
ACAT.ATGT	4	11755897	11755916	5	.	.	intergenic	-0.255	rs787109231	2
ACAT.ATGT	4	11819320	11819334	3.75	.	.	intergenic	-0.327	997543 rs7930	2
AT.AT	4	12073352	12073364	6.5	.	.	intergenic	0.037	rs789883185	3
AG.CT	4	12115242	12115251	5	.	.	intergenic	-0.151	.	2
AAC.GTT	4	12179984	12179996	4.33333	.	.	intergenic	-0.117	rs789006013	2
AG.CT	4	12289638	12289647	5	.	.	intergenic	-0.134	309071 rs7881	2
AC.GT	4	12378700	12378709	5	.	.	intergenic	-0.086	.	2
AAT.ATT	4	12682848	12682866	6.33333	.	.	intergenic	-0.226	rs788653145	2
AAAC.GTTT	4	12743437	12743451	3.75	.	.	intergenic	.	rs786543882	2
AAAT.ATTT	4	12809545	12809567	5.75	.	.	intergenic	-0.175	rs792108620	2
ATC.GAT	4	12820506	12820520	5	.	.	intergenic	-0.28	rs790089840	2
AT.AT	4	12854629	12854646	9	.	.	intergenic	-0.155	468418 rs7933	2
AC.GT	4	13228469	13228480	6	.	.	intergenic	0.133	479763 rs7905	4
AAAAAT.ATTTTT	4	13240829	13240851	3.83333	.	.	intergenic	0.053	rs792057455	2
AT.AT	4	13391073	13391090	9	.	.	intergenic	-0.19	rs787165400	3
AG.CT	4	13392147	13392158	6	.	.	intergenic	1.215	.	3
AAAC.GTTT	4	13430292	13430315	6	.	.	intergenic	-0.11	rs792824417	2
AC.GT	4	13558562	13558576	7.5	.	.	intergenic	-0.037	rs787019350	2
AAAC.GTTT	4	13565672	13565699	7	.	.	intergenic	.	095279 rs7901	2
AAAC.GTTT	4	13645065	13645084	5	.	.	intergenic	0.203	rs789778945	2
AAGAG.CTCTT	4	14443727	14443743	3.4	.	.	intergenic	0.495	rs792049048	2
AAAAAC.GTTTTT	4	14612991	14613015	4.16667	.	.	intergenic	0.444	733021 rs7889	2
AAAC.GTTT	4	14692772	14692803	8	.	.	intergenic	-0.145	967910 rs7882	2
AAAT.ATTT	4	14827673	14827689	4.25	.	.	intergenic	-0.203	rs793855200	2
AAAT.ATTT	4	15101766	15101786	5.25	.	.	intergenic	0.11	rs788516833	2
AC.GT	4	15186550	15186560	5.5	ENSSSCT00000006552	KIAA0196	intron	0.095	.	2
AC.GT	4	15186550	15186560	5.5	ENSSSCT00000026728	.	promoter	0.095	.	2
AT.AT	4	15218845	15218877	16.5	ENSSSCT00000034190	KIAA0196	intron	-0.207	755984 rs7895	2
AT.AT	4	15218845	15218877	16.5	ENSSSCT00000006552	KIAA0196	intron	-0.207	755984 rs7895	2
AAAAT.ATTTT	4	15284545	15284564	4	.	.	intergenic	0.174	rs786493486	2
AAAAT.ATTTT	4	15913328	15913357	6	ENSSSCT00000006560	TMEM65	intron	.	rs790671232	2
AAAAC.GTTTT	4	15949757	15949784	5.6	.	.	intergenic	-0.059	rs790348430	2
AC.GT	4	16162979	16163000	11	.	.	intergenic	0.125	rs709860514	4
AAAC.GTTT	4	16335622	16335635	3.5	.	.	intergenic	-0.18	rs788673593	2
AC.GT	4	16383655	16383666	6	ENSSSCT00000027516	FAM91A1	intron	1.081	rs789870026	2
AGAT.ATCT	4	16441962	16441982	5.25	.	.	intergenic	0.004	rs787170842	2
AAAAT.ATTTT	4	16701839	16701863	5	.	.	intergenic	.	570217 rs7884	2
ACC.GGT	4	16716370	16716383	4.66667	.	.	intergenic	-0.038	rs790856571	4
AT.AT	4	16775843	16775868	13	ENSSSCT00000006566	ATAD2	intron	0.264	rs792103838	4
AATC.GATT	4	16790277	16790298	5.5	ENSSSCT00000006566	ATAD2	intron	.	rs793876993	2
AAAAC.GTTTT	4	16807317	16807336	4	ENSSSCT00000006566	ATAD2	intron	.	329758 rs7909	2
AC.GT	4	16898815	16898827	6.5	ENSSSCT00000024326	.	intron	.	rs694051292	3
AAC.GTT	4	17139111	17139127	5.66667	.	.	intergenic	.	rs790332987	2
AT.AT	4	17184361	17184370	5	.	.	intergenic	.	.	2
AGGG.CCCT	4	17291439	17291450	3	.	.	intergenic	-0.289	.	2
AC.GT	4	17305911	17305925	7.5	.	.	intergenic	-0.09	rs791887360	2
AC.GT	4	17524880	17524894	7.5	.	.	intergenic	0.125	rs699721282	2
AGG.CCT	4	17661863	17661875	4.33333	.	.	intergenic	0.039	rs792035373	2
AC.GT	4	17686018	17686046	14.5	.	.	intergenic	0.207	.	2

AAAC.GTTT	4	17935971	17935988	4.5	.	.	intergenic	.	459050 rs7014	4
AG.CT	4	17994958	17994989	16	.	.	intergenic	-0.215	.	4
AG.CT	4	18137054	18137069	8	.	.	intergenic	-0.022	rs786517363	2
AAAC.GTTT	4	18236573	18236598	6.5	.	.	intergenic	.	.	2
AG.CT	4	18261618	18261632	7.5	.	.	intergenic	0.226	.	3
AAT.ATT	4	18534144	18534158	5	.	.	intergenic	0.379	rs789145513	2
AAAT.ATTT	4	18546564	18546586	5.75	.	.	intergenic	0.049	381433 rs7911	2
AC.GT	4	18749306	18749327	11	.	.	intergenic	0.134	rs788789661	4
AGC.GCT	4	18803246	18803258	4.33333	.	.	intergenic	-0.173	rs788925205	2
AAG.CTT	4	18807366	18807390	8.33333	.	.	intergenic	-0.271	rs787367049	2
AG.CT	4	19011423	19011436	7	.	.	intergenic	0.163	.	3
AC.GT	4	19016979	19016990	6	.	.	intergenic	1.06	rs790883351	2
AAAC.GTTT	4	19225948	19225966	4.75	.	.	intergenic	.	.	2
AAAAC.GTTTT	4	19250745	19250770	5.2	.	.	intergenic	.	172182 rs7904	2
AAAC.GTTT	4	19253068	19253088	5.25	.	.	intergenic	-0.204	323127 rs7863	2
AC.GT	4	19374834	19374852	9.5	.	.	intergenic	0.003	rs712100598	3
AAAAT.ATTTT	4	19774229	19774253	5	ENSSSCT00000035287	COL14A1	intron	0.497	rs788098968	2
AAAAT.ATTTT	4	19774229	19774253	5	ENSSSCT00000006580	COL14A1	intron	0.497	rs788098968	2
AAAC.GTTT	4	19806287	19806309	5.75	ENSSSCT00000035287	COL14A1	intron	-0.172	rs788355159	2
AAAC.GTTT	4	19806287	19806309	5.75	ENSSSCT00000006580	COL14A1	intron	-0.172	rs788355159	2
AAACC.GGTTT	4	19810122	19810142	3.5	ENSSSCT00000035287	COL14A1	intron	1.717	rs793260201	2
AAACC.GGTTT	4	19810122	19810142	3.5	ENSSSCT00000006580	COL14A1	intron	1.717	rs793260201	2
AAAC.GTTT	4	19841774	19841802	7.25	ENSSSCT00000035287	COL14A1	intron	-0.405	rs787937706	3
AAAC.GTTT	4	19841774	19841802	7.25	ENSSSCT00000006580	COL14A1	intron	-0.405	rs787937706	3
AAAC.GTTT	4	20006695	20006718	6	ENSSSCT00000036252	DEPTOR	3'utr	0.218	326352 rs7912	2
AAAC.GTTT	4	20006695	20006718	6	ENSSSCT00000034055	DEPTOR	intron	0.218	326352 rs7912	2
AT.AT	4	20056152	20056162	5.5	ENSSSCT00000034055	DEPTOR	intron	.	rs788345458	3
AT.AT	4	20056152	20056162	5.5	ENSSSCT00000036252	DEPTOR	intron	.	rs788345458	3
AT.AT	4	20056152	20056162	5.5	ENSSSCT00000006581	DEPTOR	intron	.	rs788345458	3
AT.AT	4	20056152	20056162	5.5	ENSSSCT00000034473	DEPTOR	intron	.	rs788345458	3
AAACC.GGTTT	4	20088258	20088276	3.8	ENSSSCT00000006581	DEPTOR	intron	.	rs789452135	2
AAACC.GGTTT	4	20088258	20088276	3.8	ENSSSCT00000034473	DEPTOR	intron	.	rs789452135	2
AAACC.GGTTT	4	20088258	20088276	3.8	ENSSSCT00000036252	DEPTOR	intron	.	rs789452135	2
AAAC.GTTT	4	20103532	20103558	6.75	ENSSSCT00000006581	DEPTOR	intron	0.364	rs788621269	2
AAAC.GTTT	4	20103532	20103558	6.75	ENSSSCT00000034473	DEPTOR	intron	0.364	rs788621269	2
AAAC.GTTT	4	20103532	20103558	6.75	ENSSSCT00000036252	DEPTOR	intron	0.364	rs788621269	2
AT.AT	4	20264682	20264694	6.5	ENSSSCT00000006583	TAF2	intron	0.25	rs793721047	4
AT.AT	4	20264682	20264694	6.5	ENSSSCT00000033583	TAF2	intron	0.25	rs793721047	4
AT.AT	4	20264682	20264694	6.5	ENSSSCT00000034647	TAF2	intron	0.25	rs793721047	4
AAT.ATT	4	20275152	20275164	4.33333	ENSSSCT00000006583	TAF2	intron	0.081	.	2
AAT.ATT	4	20275152	20275164	4.33333	ENSSSCT00000033583	TAF2	intron	0.081	.	2
AAT.ATT	4	20275152	20275164	4.33333	ENSSSCT00000034647	TAF2	intron	0.081	.	2
AAATC.GATTT	4	20378715	20378742	5.6	ENSSSCT00000006585	ENPP2	intron	-0.369	rs793610594	2
AAAC.GTTT	4	20410270	20410292	5.75	ENSSSCT00000006585	ENPP2	intron	0.46	rs788456489	3
AAT.ATT	4	20448994	20449017	8	ENSSSCT00000006585	ENPP2	intron	-0.169	rs792595269	2
AATG.CATT	4	20504033	20504059	6.75	.	.	intergenic	0.08	158900 rs7905	2
AAT.ATT	4	20504839	20504850	4	.	.	intergenic	-0.073	.	2
AAAAG.CTTTT	4	20572605	20572628	4.8	.	.	intergenic	0.05	rs791852858	2
AG.CT	4	20615447	20615461	7.5	ENSSSCT00000006587	MAL2	3'utr	0.395	.	2
AC.GT	4	20648955	20648965	5.5	.	.	intergenic	0.131	.	2
AT.AT	4	20655478	20655490	6.5	.	.	intergenic	0.092	.	3
AAGG.CCTT	4	20674671	20674688	4.5	.	.	intergenic	0.003	rs792477340	2
AT.AT	4	20839501	20839513	6.5	.	.	intergenic	-0.004	rs788243844	2
AC.GT	4	20997483	20997495	6.5	.	.	intergenic	0.166	rs792884111	2
AG.CT	4	21200378	21200390	6.5	.	.	intergenic	-0.05	745662 rs7934	2
AC.GT	4	21247674	21247686	6.5	.	.	intergenic	0.055	459871 rs7883	2
AAAT.ATTT	4	21402576	21402595	5	.	.	intergenic	-0.161	rs787517754	3
AATG.CATT	4	21466125	21466143	4.75	.	.	intergenic	-0.095	rs789626174	2
AG.CT	4	21710514	21710524	5.5	.	.	intergenic	0.066	508321 rs7923	2
AAAAC.GTTTT	4	21738843	21738864	3.66667	.	.	intergenic	0.134	rs791973844	2
AAAG.CTTT	4	21859604	21859623	5	.	.	intergenic	0.363	722409 rs7925	2
AG.CT	4	21864582	21864604	11.5	.	.	intergenic	-0.048	rs793763025	2
AAAAC.GTTTT	4	21872410	21872433	4.8	.	.	intergenic	0.001	rs790036415	3
AC.GT	4	21963529	21963556	14	.	.	intergenic	-0.047	.	3
AC.GT	4	22088178	22088195	9	ENSSSCT00000006593	EXT1	promoter	1.316	.	3
AC.GT	4	22088178	22088195	9	ENSSSCT00000033159	EXT1	promoter	1.316	.	3
AC.GT	4	22281117	22281131	7.5	.	.	intergenic	.	.	2
AAAT.ATTT	4	22313313	22313330	4.5	.	.	intergenic	.	705535 rs7899	2
AG.CT	4	22318563	22318574	6	.	.	intergenic	0.477	rs788091019	2
AC.GT	4	22523383	22523398	8	.	.	intergenic	.	rs789076435	2
AC.GT	4	22606937	22606957	10.5	.	.	intergenic	0.017	.	4
AC.GT	4	22610185	22610199	7.5	.	.	intergenic	-0.036	rs790665028	2
AT.AT	4	22703190	22703199	5	.	.	intergenic	-0.099	.	2
AT.AT	4	22748154	22748165	6	.	.	intergenic	0.202	.	3
AAT.ATT	4	22766210	22766228	6.33333	.	.	intergenic	0.226	.	2
AG.CT	4	22820289	22820300	6	ENSSSCT00000035755	SLC30A8	intron	.	rs787479890	2
AG.CT	4	22820289	22820300	6	ENSSSCT00000006597	SLC30A8	intron	.	rs787479890	2
AAAAC.GTTTT	4	22852602	22852626	5	.	.	intergenic	0.334	rs791498400	2
AC.GT	4	22856504	22856542	19.5	.	.	intergenic	0.041	rs786562340	6
ACTG.CAGT	4	22870012	22870034	5.75	.	.	intergenic	0.164	rs792377824	2
AAAT.ATTT	4	22873035	22873067	8.25	.	.	intergenic	0.097	338782 rs7906	2
AAAC.GTTT	4	22946754	22946776	5.75	.	.	intergenic	0.104	rs789538894	2
AC.GT	4	23065350	23065366	8.5	ENSSSCT00000033947	RAD21	intron	.	.	3
AC.GT	4	23065350	23065366	8.5	ENSSSCT00000006600	RAD21	intron	.	.	3

AAAC.GTTT	4	23125085	23125099	3.75	ENSSSCT00000006600	RAD21	intron	.	723748 rs7935	2
ACC.GGT	4	23152311	23152329	6.33333	ENSSSCT00000006600	RAD21	intron	.	rs788041336	2
AAC.GTT	4	24552019	24552036	6	.	.	intergenic	0.37	rs791493224	4
AAAC.GTTT	4	24554660	24554680	5.25	.	.	intergenic	0.206	rs790027003	2
AC.GT	4	24699536	24699550	7.5	.	.	intergenic	.	rs793033031	2
AC.GT	4	24839841	24839851	5.5	ENSSSCT00000006604	TRPS1	intron	.	rs790021406	2
AAAT.ATTT	4	25094093	25094111	4.75	.	.	intergenic	0.162	.	2
AG.CT	4	25190850	25190859	5	.	.	intergenic	0.013	rs787062006	2
AC.GT	4	25219346	25219358	6.5	.	.	intergenic	-0.124	rs791987235	2
ATC.GAT	4	25309991	25310004	4.66667	.	.	intergenic	-0.115	770108 rs7916	3
AG.CT	4	25317202	25317225	12	.	.	intergenic	0.059	rs793126807	4
ACAT.ATGT	4	25329967	25329990	6	.	.	intergenic	-0.098	rs787376958	2
AT.AT	4	25472143	25472156	7	.	.	intergenic	-0.058	927175 rs7872	4
AC.GT	4	25488800	25488812	6.5	.	.	intergenic	-0.019	rs790900331	2
AT.AT	4	25504607	25504622	8	.	.	intergenic	.	145723 rs7865	3
AT.AT	4	25653752	25653764	6.5	.	.	intergenic	.	.	4
AT.AT	4	25695870	25695881	6	.	.	intergenic	-0.084	.	2
AAAT.ATTT	4	25848227	25848247	5.25	.	.	intergenic	-0.007	rs793596109	2
AAAC.GTTT	4	25951308	25951326	4.75	.	.	intergenic	-0.021	975130 rs7895	2
AAAAAC.GTTTTT	4	26057482	26057499	3	.	.	intergenic	-0.05	rs701864834	2
AAC.GTT	4	26188658	26188680	7.66667	.	.	intergenic	-0.123	500313 rs7935	2
AAC.GTT	4	26194571	26194587	5.66667	.	.	intergenic	.	042062 rs7932	2
AAAAT.ATTTT	4	26259760	26259783	4.8	.	.	intergenic	.	429424 rs7937	2
AC.GT	4	26408372	26408390	9.5	.	.	intergenic	0.115	rs790945249	2
AT.AT	4	26566340	26566349	5	.	.	intergenic	0.077	rs793740238	2
AC.GT	4	26956784	26956805	11	.	.	intergenic	-0.103	rs789666580	2
AAAG.CTTT	4	27337361	27337372	3	.	.	intergenic	-0.11	.	2
AG.CT	4	27403055	27403087	16.5	.	.	intergenic	-0.02	rs792639487	2
AAAAC.GTTTT	4	27733803	27733826	4.8	ENSSSCT00000032782	CSMD3	intron	-0.017	956707 rs7886	2
AAAC.GTTT	4	27745616	27745633	4.5	ENSSSCT00000032782	CSMD3	intron	-0.014	rs790263585	2
AT.AT	4	27787573	27787585	6.5	.	.	intergenic	-0.162	rs788531378	2
AAGT.ACTT	4	27810039	27810050	3	.	.	intergenic	0.301	rs792125711	2
AAAC.GTTT	4	27893021	27893039	4.75	.	.	intergenic	-0.057	rs789434998	2
AT.AT	4	27912038	27912047	5	.	.	intergenic	.	.	2
AG.CT	4	28107775	28107793	9.5	.	.	intergenic	0.089	rs786787905	3
AAAC.GTTT	4	28630313	28630333	5.25	.	.	intergenic	-0.033	rs791608721	2
AAC.GTT	4	28767896	28767912	5.66667	.	.	intergenic	.	.	2
AC.GT	4	28829391	28829404	7	.	.	intergenic	.	rs793159059	4
AAAAC.GTTTT	4	28906858	28906886	5.8	.	.	intergenic	.	rs791989803	2
AT.AT	4	29006083	29006092	5	.	.	intergenic	0.073	rs791149212	2
AAT.ATT	4	29133145	29133160	5.33333	.	.	intergenic	0.128	581093 rs7900	2
AAC.GTT	4	29290618	29290636	6.33333	.	.	intergenic	.	496457 rs7893	2
AT.AT	4	29549568	29549579	6	.	.	intergenic	0.001	rs791375101	4
AAAAC.GTTTT	4	29870449	29870467	3.8	.	.	intergenic	0.245	.	2
AC.GT	4	29879616	29879629	7	.	.	intergenic	0.006	757265 rs7877	2
AAAC.GTTT	4	30252710	30252724	3.75	ENSSSCT00000006611	EBAG9	intron	0.195	rs792582363	2
AG.CT	4	30264673	30264686	7	.	.	intergenic	0.084	rs788079395	2
AAT.ATT	4	30865290	30865303	4.66667	.	.	intergenic	0.108	rs790561795	2
AATG.CATT	4	30970248	30970274	6.75	.	.	intergenic	.	rs791887749	2
AT.AT	4	31018344	31018354	5.5	.	.	intergenic	0.02	966836 rs7886	3
AC.GT	4	31063946	31063961	8	ENSSSCT00000034120	TMEM74	5'utr	0.125	503387 rs7879	2
AC.GT	4	31063946	31063961	8	ENSSSCT00000006620	TMEM74	promoter	0.125	503387 rs7879	2
ACAT.ATGT	4	31605088	31605110	5.75	.	.	intergenic	.	rs702204322	2
AG.CT	4	31614200	31614215	8	.	.	intergenic	.	.	2
AAT.ATT	4	31784048	31784063	5.33333	.	.	intergenic	.	.	2
AC.GT	4	31949510	31949520	5.5	ENSSSCT00000006623	RSPO2	intron	.	rs787653506	2
AAAT.ATTT	4	32142191	32142209	4.75	.	.	intergenic	0.03	rs790625674	2
AC.GT	4	32409386	32409396	5.5	.	.	intergenic	.	.	4
AAC.GTT	4	32839314	32839327	4.66667	.	.	intergenic	.	rs789863448	2
AG.CT	4	32851481	32851490	5	.	.	intergenic	0.02	rs793868839	2
AAC.GTT	4	33051326	33051339	4.66667	.	.	intergenic	.	.	3
ACC.GGT	4	33377944	33377963	6.66667	ENSSSCT00000006626	.	3'utr	0.808	rs793392309	2
AAT.ATT	4	33592029	33592049	7	.	.	intergenic	-0.037	rs787704827	2
AC.GT	4	33668035	33668048	7	.	.	intergenic	-0.006	.	3
AC.GT	4	33781359	33781373	7.5	.	.	intergenic	0.013	990097 rs7898	3
AAG.CTT	4	33815537	33815552	5.33333	.	.	intergenic	-0.176	377920 rs7906	2
AATC.GATT	4	34082211	34082228	4.5	.	.	intergenic	.	rs786401776	2
AC.GT	4	34280725	34280752	14	ENSSSCT00000006627	ZFPM2	intron	0.443	rs792311825	5
AC.GT	4	34331267	34331282	8	ENSSSCT00000006627	ZFPM2	intron	0.222	.	4
AAAG.CTTT	4	34331899	34331912	3.5	ENSSSCT00000006627	ZFPM2	intron	0.046	593505 rs7891	2
AT.AT	4	34333559	34333568	5	ENSSSCT00000006627	ZFPM2	intron	-0.066	rs787578503	2
AAAAT.ATTTT	4	34347204	34347234	6.2	ENSSSCT00000006627	ZFPM2	intron	0.044	rs703940018	2
AAAC.GTTT	4	34549740	34549754	3.75	ENSSSCT00000006627	ZFPM2	intron	-0.211	rs791996694	2
AAAAC.GTTTT	4	34788970	34788998	5.8	.	.	intergenic	.	rs786743055	3
AAAC.GTTT	4	34805281	34805313	8.25	.	.	intergenic	0.086	rs786827137	2
AAC.GTT	4	34950034	34950061	9.33333	.	.	intergenic	.	rs792192542	2
AAT.ATT	4	35066287	35066311	8.33333	.	.	intergenic	0.042	rs791626818	2
AC.GT	4	35083134	35083146	6.5	.	.	intergenic	0.397	794530 rs7892	2
AATG.CATT	4	35252883	35252908	6.5	.	.	intergenic	.	075464 rs7868	2
AAAG.CTTT	4	35365775	35365796	5.5	.	.	intergenic	0.022	112542 rs7937	2
AAAAC.GTTTT	4	35538023	35538041	3.8	ENSSSCT00000006629	DPYS	intron	.	rs704086484	2
AC.GT	4	35810110	35810126	8.5	ENSSSCT00000006634	RIMS2	intron	0.117	143803 rs7884	3
AAT.ATT	4	35881150	35881178	9.66667	ENSSSCT00000006634	RIMS2	intron	0.149	rs696825635	2
AAGG.CCTT	4	35881700	35881712	3.25	ENSSSCT00000006634	RIMS2	intron	0.238	.	2

AAT.ATT	4	35909079	35909097	6.33333	ENSSSCT00000006634	RIMS2	intron	0.121	rs793302596	2
AAGC.GCTT	4	35962821	35962842	5.5	.	.	intergenic	.	I38804 rs7890	2
AAC.GTT	4	36072094	36072109	5.33333	ENSSSCT00000029168	.	intron	0.118	270986 rs7912	2
AAAAC.GTTTT	4	36262406	36262430	5	.	.	intergenic	0.173	rs696189827	2
AAAC.GTTT	4	36331238	36331256	4.75	.	.	intergenic	.	rs788656338	2
AAAAT.ATTTT	4	36468495	36468509	3	ENSSSCT00000006637	DCAF13	intron	-0.022	rs788380830	2
AG.CT	4	36815871	36815880	5	.	.	intergenic	0.092	rs793084015	2
AC.GT	4	36820743	36820758	8	.	.	intergenic	0.083	rs789966357	2
AC.GT	4	36918468	36918477	5	ENSSSCT00000006643	ATP6V1C1	promoter	0.159	.	3
AAAC.GTTT	4	37033825	37033843	4.75	.	.	intergenic	0.345	rs793454367	2
AAAC.GTTT	4	37264898	37264917	5	.	.	intergenic	-0.035	320848 rs7866	2
AAAT.ATTT	4	37296981	37297001	5.25	.	.	intergenic	0.003	rs791217992	2
AAAG.CTTT	4	37306353	37306365	3.25	.	.	intergenic	-0.096	rs791887842	2
AT.AT	4	37675813	37675827	7.5	.	.	intergenic	0.111	rs786883455	4
AAAT.ATTT	4	37781813	37781831	4.75	ENSSSCT00000035758	CU633679.1	intron	.	rs789729881	2
AATT.AATT	4	37855832	37855856	6.25	.	.	intergenic	.	rs790345029	2
AG.CT	4	37858170	37858179	5	.	.	intergenic	-0.074	rs787131631	2
AG.CT	4	38010569	38010582	7	ENSSSCT00000035357	NCALD	intron	-0.073	rs791157844	2
AG.CT	4	38010569	38010582	7	ENSSSCT00000006648	NCALD	intron	-0.073	rs791157844	2
AAAAAC.GTTTTT	4	38174692	38174714	3.83333	ENSSSCT00000036111	NCALD	intron	0.06	rs789261322	2
AAAAAC.GTTTTT	4	38174692	38174714	3.83333	ENSSSCT00000035298	NCALD	intron	0.06	rs789261322	2
AAAAAC.GTTTTT	4	38174692	38174714	3.83333	ENSSSCT00000035679	NCALD	intron	0.06	rs789261322	2
AAAAAC.GTTTTT	4	38174692	38174714	3.83333	ENSSSCT00000006648	NCALD	intron	0.06	rs789261322	2
AAAAAC.GTTTTT	4	38174692	38174714	3.83333	ENSSSCT00000032895	NCALD	intron	0.06	rs789261322	2
AAAAAC.GTTTTT	4	38174692	38174714	3.83333	ENSSSCT00000035469	NCALD	intron	0.06	rs789261322	2
AAAAAC.GTTTTT	4	38174692	38174714	3.83333	ENSSSCT00000033437	NCALD	intron	0.06	rs789261322	2
AAAAAC.GTTTTT	4	38174692	38174714	3.83333	ENSSSCT00000035357	NCALD	intron	0.06	rs789261322	2
AAAAAC.GTTTTT	4	38174692	38174714	3.83333	ENSSSCT00000033719	NCALD	intron	0.06	rs789261322	2
AAAAAC.GTTTTT	4	38174692	38174714	3.83333	ENSSSCT00000034691	NCALD	intron	0.06	rs789261322	2
AAAAAC.GTTTTT	4	38174692	38174714	3.83333	ENSSSCT00000033597	NCALD	intron	0.06	rs789261322	2
AAAAAC.GTTTTT	4	38174692	38174714	3.83333	ENSSSCT00000034309	NCALD	intron	0.06	rs789261322	2
AAC.GTT	4	38180136	38180161	8.66667	ENSSSCT00000036111	NCALD	intron	-0.127	rs793273220	2
AAC.GTT	4	38180136	38180161	8.66667	ENSSSCT00000035298	NCALD	intron	-0.127	rs793273220	2
AAC.GTT	4	38180136	38180161	8.66667	ENSSSCT00000035679	NCALD	intron	-0.127	rs793273220	2
AAC.GTT	4	38180136	38180161	8.66667	ENSSSCT00000006648	NCALD	intron	-0.127	rs793273220	2
AAC.GTT	4	38180136	38180161	8.66667	ENSSSCT00000032895	NCALD	intron	-0.127	rs793273220	2
AAC.GTT	4	38180136	38180161	8.66667	ENSSSCT00000035469	NCALD	intron	-0.127	rs793273220	2
AAC.GTT	4	38180136	38180161	8.66667	ENSSSCT00000033437	NCALD	intron	-0.127	rs793273220	2
AAC.GTT	4	38180136	38180161	8.66667	ENSSSCT00000035357	NCALD	intron	-0.127	rs793273220	2
AAC.GTT	4	38180136	38180161	8.66667	ENSSSCT00000033719	NCALD	intron	-0.127	rs793273220	2
AAC.GTT	4	38180136	38180161	8.66667	ENSSSCT00000034691	NCALD	intron	-0.127	rs793273220	2
AAC.GTT	4	38180136	38180161	8.66667	ENSSSCT00000033597	NCALD	intron	-0.127	rs793273220	2
AAC.GTT	4	38180136	38180161	8.66667	ENSSSCT00000034309	NCALD	intron	-0.127	rs793273220	2
AAAAC.GTTTT	4	38320049	38320069	4.2	ENSSSCT00000006649	GRHL2	intron	-0.119	rs787589205	2
AATG.CATT	4	38329519	38329535	4.25	ENSSSCT00000006649	GRHL2	intron	-0.031	.	2
AG.CT	4	38444191	38444202	6	.	.	intergenic	-0.02	rs791153296	2
AAC.GTT	4	38575770	38575788	6.33333	.	.	intergenic	.	rs793800938	2
AC.GT	4	38629307	38629336	15	.	.	intergenic	-0.002	.	3
AAAC.GTTT	4	38979237	38979253	4.25	.	.	intergenic	-0.047	rs787312116	2
AG.CT	4	39054102	39054111	5	.	.	intergenic	-0.148	rs789371624	2
AAT.ATT	4	39274211	39274228	6	ENSSSCT00000006653	SNX31	intron	0.128	rs792926022	2
AAAG.CTTT	4	39285799	39285813	3.75	ENSSSCT00000006653	SNX31	intron	.	rs792128919	2
AAAT.ATTT	4	39369168	39369193	6.5	.	.	intergenic	-0.187	rs791457235	2
AC.GT	4	39377793	39377809	8.5	.	.	intergenic	0.092	rs793590163	2
AAAC.GTTT	4	39384005	39384026	5.5	.	.	intergenic	0.186	rs791631132	2
AAAG.CTTT	4	39469678	39469696	4.75	.	.	intergenic	0.032	rs787276169	2
AAAT.ATTT	4	39595733	39595755	5.75	ENSSSCT00000006656	RNF19A	intron	0.139	284808 rs7908	2
AC.GT	4	39619636	39619647	6	ENSSSCT00000006656	RNF19A	intron	0.275	rs787441047	4
AG.CT	4	39821996	39822010	7.5	.	.	intergenic	0.458	.	3
AC.GT	4	39936508	39936520	6.5	ENSSSCT00000006660	RGS22	intron	0.098	rs789508917	2
AC.GT	4	39956215	39956228	7	ENSSSCT00000006660	RGS22	intron	.	rs787811735	2
AAAT.ATTT	4	39965055	39965072	4.5	.	.	intergenic	.	.	2
AATC.GATT	4	39979661	39979676	4	.	.	intergenic	0.029	.	2
AAAC.GTTT	4	39982387	39982411	6.25	.	.	intergenic	0.166	rs786476603	2
AG.CT	4	40008999	40009011	6.5	ENSSSCT00000006662	.	intron	0.143	rs790538723	2
AATG.CATT	4	40118990	40119029	10	ENSSSCT00000006662	.	intron	0.648	rs793602475	2
AT.AT	4	40258821	40258832	6	.	.	intergenic	0.109	.	2
AT.AT	4	40322151	40322166	8	.	.	intergenic	0.135	rs786704459	2
AAC.GTT	4	40496113	40496127	5	ENSSSCT00000030508	.	intron	0.529	rs793111008	3
AC.GT	4	40584808	40584818	5.5	ENSSSCT00000030508	.	intron	0.159	.	2
AAAC.GTTT	4	41010776	41010791	4	.	.	intergenic	-0.083	rs788734925	2
AAAT.ATTT	4	41142871	41142888	4.5	ENSSSCT00000006665	STK3	intron	0.293	rs787112513	2
AC.GT	4	41325220	41325229	5	.	.	intergenic	.	rs790943933	2
AAC.GTT	4	41327724	41327737	4.66667	.	.	intergenic	.	326084 rs7895	4
AG.CT	4	41455476	41455490	7.5	.	.	intergenic	-0.09	rs790150311	2
AG.CT	4	41466719	41466732	7	.	.	intergenic	.	rs789916180	2
AAAC.GTTT	4	41522955	41522971	4.25	.	.	intergenic	-0.103	I94394 rs7885	2
AAAAC.GTTTT	4	41628113	41628132	4	ENSSSCT00000026541	.	intron	-0.019	306904 rs7888	2
AAAC.GTTT	4	41633206	41633229	6	ENSSSCT00000026541	.	3'utr	.	rs790229163	2
AAAT.ATTT	4	41643853	41643869	4.25	.	.	intergenic	.	rs787208026	2
AAAC.GTTT	4	41726628	41726646	4.75	ENSSSCT00000023470	.	intron	.	513669 rs7931	4
AAAC.GTTT	4	41726628	41726646	4.75	ENSSSCT00000006669	.	intron	.	513669 rs7931	4
AAAC.GTTT	4	41809188	41809202	3.75	ENSSSCT00000006670	HRSP12	intron	0.103	.	2
AAAC.GTTT	4	41809188	41809202	3.75	ENSSSCT00000023470	.	promoter	0.103	.	2

AAAC.GTTT	4	41809188	41809202	3.75	ENSSSCT00000006669	.	promoter	0.103	.	2
AAT.ATT	4	41947039	41947057	6.33333	.	.	intergenic	0.209	)80968 rs7873	2
AAAC.GTTT	4	42024168	42024183	4	ENSSSCT00000006674	LAPTM4B	intron	0.105	544499 rs7872	2
AAAAC.GTTTT	4	42263507	42263532	5.2	.	.	intergenic	.	I92889 rs7873	2
AAAC.GTTT	4	42473814	42473830	4.25	ENSSSCT00000034269	CU302278.8	promoter	0.175	rs790164653	3
AG.CT	4	42522157	42522177	10.5	ENSSSCT00000006678	.	intron	0.214	rs792916576	4
AG.CT	4	42883848	42883857	5	ENSSSCT00000006678	.	intron	0.567	rs789405615	2
AAAG.CTTT	4	42917013	42917025	3.25	ENSSSCT00000006678	.	intron	.	rs789916649	2
AAAC.GTTT	4	43125816	43125839	6	ENSSSCT00000006679	SDC2	intron	0.511	rs793876679	2
AAT.ATT	4	43174356	43174389	11.3333	.	.	intergenic	-0.06	I20590 rs7922	2
AAAC.GTTT	4	43234734	43234762	7.25	.	.	intergenic	0.236	779661 rs7903	2
AT.AT	4	43306366	43306394	14.5	.	.	intergenic	.	rs787408333	2
AAC.GTT	4	43511356	43511369	4.66667	ENSSSCT00000006680	PTDSS1	intron	-0.19	rs790517324	2
AAT.ATT	4	43578069	43578086	6	ENSSSCT00000006681	MTERFD1	intron	1.029	rs788429201	2
AAAC.GTTT	4	43591461	43591491	7.75	.	.	intergenic	0.229	rs790709224	4
AT.AT	4	43817335	43817350	8	.	.	intergenic	.	.	3
AAAG.CTTT	4	43882653	43882676	6	.	.	intergenic	0.224	345172 rs7909	2
AC.GT	4	43979497	43979525	14.5	.	.	intergenic	0.099	541456 rs7919	2
AAAG.CTTT	4	44015542	44015553	3	.	.	intergenic	0.256	.	2
AAAAT.ATTTT	4	44038683	44038712	6	.	.	intergenic	0.251	)41073 rs7895	2
AAAT.ATTT	4	44242924	44242938	3.75	.	.	intergenic	-0.099	rs790738441	2
AC.GT	4	44271790	44271803	7	.	.	intergenic	0.046	.	2
AT.AT	4	44272326	44272337	6	.	.	intergenic	0.117	rs788693443	2
AAAGAT.ATCTT	4	44336116	44336157	7	.	.	intergenic	.	rs793076761	2
AATG.CATT	4	44532519	44532540	5.5	.	.	intergenic	0.054	rs792228113	2
AAAAAC.GTTTT	4	44870081	44870100	3.33333	.	.	intergenic	-0.009	rs788519154	2
AC.GT	4	44877958	44877978	10.5	ENSSSCT00000006684	NDUFAF6	intron	0.369	383169 rs7876	2
AC.GT	4	45127601	45127611	5.5	ENSSSCT00000006686	CCNE2	intron	0.226	rs790010408	2
AC.GT	4	45176720	45176729	5	ENSSSCT00000006687	INTS8	intron	-0.004	rs788412807	3
AAAC.GTTT	4	45189732	45189758	6.75	ENSSSCT00000006687	INTS8	intron	.	I16691 rs7932	2
AAT.ATT	4	46034038	46034050	4.33333	.	.	intergenic	0.106	rs789521167	2
AT.AT	4	46074249	46074258	5	.	.	intergenic	-0.166	.	2
AG.CT	4	46232453	46232463	5.5	.	.	intergenic	0.088	rs786770046	3
AAT.ATT	4	46331794	46331814	7	ENSSSCT00000006700	.	intron	0.152	rs788997259	2
AAAT.ATTT	4	46362347	46362364	4.5	.	.	intergenic	0.051	rs791219738	2
ACAG.CTGT	4	46505875	46505903	7.25	.	.	intergenic	0.131	580786 rs7901	3
AC.GT	4	46750298	46750314	8.5	.	.	intergenic	0.115	rs786865395	2
AG.CT	4	46939282	46939295	7	.	.	intergenic	.	518938 rs7909	2
AT.AT	4	47104399	47104416	9	.	.	intergenic	-0.075	rs791724381	2
AG.CT	4	47105855	47105867	6.5	.	.	intergenic	-0.155	.	3
AAAT.ATTT	4	47344755	47344773	4.75	.	.	intergenic	0.152	rs793712730	2
AAAC.GTTT	4	47383108	47383136	7.25	.	.	intergenic	-0.042	220084 rs7905	2
AG.CT	4	47433666	47433676	5.5	.	.	intergenic	-0.051	rs791802128	3
AAAAT.ATTTT	4	47460641	47460662	4.4	.	.	intergenic	-0.001	rs792512935	2
AG.CT	4	47964247	47964275	14.5	.	.	intergenic	.	rs789585619	3
AG.CT	4	48138205	48138217	6.5	ENSSSCT00000006707	RUNX1T1	intron	-0.186	rs709744200	4
AC.GT	4	48144578	48144587	5	ENSSSCT00000006707	RUNX1T1	intron	0.013	205980 rs7049	4
AT.AT	4	48166242	48166252	5.5	ENSSSCT00000006707	RUNX1T1	intron	0.056	.	4
AAAT.ATTT	4	48170292	48170321	7.5	ENSSSCT00000006707	RUNX1T1	intron	-0.395	589055 rs7876	2
ACT.AGT	4	48552964	48552975	4	.	.	intergenic	0.029	rs790249038	2
AT.AT	4	48707115	48707125	5.5	.	.	intergenic	0.149	.	4
AT.AT	4	48743372	48743383	6	.	.	intergenic	.	.	2
AC.GT	4	49257055	49257078	12	ENSSSCT00000006708	SLC26A7	intron	0.635	I58802 rs7937	2
AAAAC.GTTTT	4	49559309	49559329	4.2	.	.	intergenic	.	rs790535997	2
AAC.GTT	4	50026945	50026968	8	.	.	intergenic	.	395933 rs7881	2
AT.AT	4	50316884	50316901	9	.	.	intergenic	.	775627 rs7929	2
AAAAC.GTTTT	4	50401989	50402009	4.2	.	.	intergenic	-0.168	rs788453395	2
AG.CT	4	50416666	50416680	7.5	.	.	intergenic	.	.	3
AT.AT	4	50949094	50949115	11	.	.	intergenic	-0.016	rs791752432	2
AT.AT	4	50956826	50956849	12	.	.	intergenic	.	.	2
AG.CT	4	51130755	51130769	7.5	.	.	intergenic	.	I10204 rs7875	3
AC.GT	4	51133369	51133404	18	.	.	intergenic	.	775625 rs7898	3
AG.CT	4	51631785	51631799	7.5	.	.	intergenic	.	.	4
AAAT.ATTT	4	51677915	51677937	5.75	.	.	intergenic	0.223	rs712755880	2
AAAC.GTTT	4	51704605	51704625	5.25	.	.	intergenic	0.209	I67225 rs7896	2
AAAG.CTTT	4	51853617	51853633	4.25	.	.	intergenic	0.19	375373 rs7891	2
AG.CT	4	51948338	51948352	7.5	.	.	intergenic	0.021	rs790058093	3
AT.AT	4	52094379	52094388	5	.	.	intergenic	0.11	rs786260529	2
AG.CT	4	52205038	52205054	8.5	.	.	intergenic	.	rs791297126	2
AAT.ATT	4	52210481	52210501	7	.	.	intergenic	.	.	4
AT.AT	4	52220898	52220908	5.5	.	.	intergenic	-0.166	.	4
AAT.ATT	4	52496082	52496098	5.66667	.	.	intergenic	.	304290 rs7904	2
AG.CT	4	52612166	52612183	9	.	.	intergenic	.	I56301 rs7874	3
AT.AT	4	53401945	53401955	5.5	.	.	intergenic	0.024	560901 rs7898	3
AT.AT	4	53712535	53712550	8	.	.	intergenic	0.114	.	3
AC.GT	4	53738246	53738255	5	.	.	intergenic	0.243	.	4
AC.GT	4	53749049	53749064	8	.	.	intergenic	-0.142	574123 rs7864	2
AAAT.ATTT	4	53969231	53969245	3.75	.	.	intergenic	0.073	.	2
AAAT.ATTT	4	53973927	53973944	4.5	.	.	intergenic	-0.009	384856 rs7930	2
AAC.GTT	4	54044706	54044734	9.66667	.	.	intergenic	.	582072 rs7873	2
AAAAC.GTTTT	4	54114918	54114942	5	.	.	intergenic	.	rs788456743	2
AC.GT	4	54149246	54149260	7.5	.	.	intergenic	0.086	590353 rs7875	3
AAAAC.GTTTT	4	54268685	54268718	6.8	.	.	intergenic	-0.041	523127 rs7871	2
AAC.GTT	4	54426683	54426699	5.66667	.	.	intergenic	.	389672 rs7937	2

AAAC.GTTT	4	55078005	55078018	3.5	.	.	intergenic	.	rs789724700	2
AATG.CATT	4	55155501	55155520	5	ENSSSCT00000006727	CNGB3	intron	0.064	rs790843583	2
AAAT.ATTT	4	55157662	55157688	6.75	ENSSSCT00000006727	CNGB3	intron	.	52749 rs7884	2
AT.AT	4	55185620	55185632	6.5	.	.	intergenic	0.287	189872 rs7873	2
AATG.CATT	4	55860336	55860357	5.5	.	.	intergenic	0.057	78708 rs7897	2
AAAC.GTTT	4	55949519	55949545	6.75	.	.	intergenic	0.122	rs788203272	2
AAAG.CTTT	4	55984161	55984204	11	.	.	intergenic	0.023	rs791136500	2
AG.CT	4	56001084	56001095	6	.	.	intergenic	0.12	73140 rs7916	2
AG.CT	4	56015911	56015931	10.5	.	.	intergenic	0.074	42650 rs7891	4
AC.GT	4	56106935	56106949	7.5	.	.	intergenic	-0.291	rs790911834	2
AC.GT	4	56261816	56261825	5	.	.	intergenic	-0.126	.	2
AT.AT	4	56263245	56263257	6.5	.	.	intergenic	-0.036	76790 rs7901	3
AT.AT	4	56600940	56600950	5.5	.	.	intergenic	.	rs788251980	3
AT.AT	4	56710509	56710523	7.5	.	.	intergenic	.	104605 rs7878	2
AAT.ATT	4	57013447	57013458	4	ENSSSCT00000006742	RALYL	intron	-0.142	rs791625629	2
AT.AT	4	57082420	57082429	5	.	.	intergenic	.	rs790344886	3
AT.AT	4	57511860	57511882	11.5	ENSSSCT00000025498	.	intron	.	.	2
AC.GT	4	57639890	57639907	9	ENSSSCT00000025498	.	intron	0.086	97614 rs7869	5
ACAT.ATGT	4	57656852	57656878	6.75	ENSSSCT00000025498	.	intron	0.001	rs792433616	3
AC.GT	4	57665638	57665656	9.5	ENSSSCT00000025498	.	intron	.	rs787007337	3
AAAC.GTTT	4	57684527	57684549	5.75	ENSSSCT00000025498	.	intron	-0.134	41947 rs7870	4
AAAC.GTTT	4	57724012	57724040	7.25	.	.	intergenic	-0.106	rs786396229	2
AC.GT	4	57732426	57732435	5	.	.	intergenic	0.018	.	2
AAG.CTT	4	57987448	57987463	5.33333	.	.	intergenic	-0.088	31090 rs7895	2
AT.AT	4	58085781	58085793	6.5	.	.	intergenic	.	rs791606032	4
AAAC.GTTT	4	58337761	58337775	3.75	.	.	intergenic	0.025	rs791064930	2
AAC.GTT	4	58355022	58355038	5.66667	.	.	intergenic	-0.058	85054 rs7925	2
AAAC.GTTT	4	58371649	58371665	4.25	.	.	intergenic	0.012	rs793001881	2
AT.AT	4	58462164	58462181	9	.	.	intergenic	.	rs793184789	2
AG.CT	4	58651889	58651911	11.5	.	.	intergenic	.	rs705920180	3
AAC.GTT	4	58750908	58750922	5	.	.	intergenic	0.034	rs793132468	2
AC.GT	4	59412646	59412658	6.5	.	.	intergenic	-0.029	.	3
AG.CT	4	59498453	59498463	5.5	.	.	intergenic	0.11	.	4
AAAC.GTTT	4	59530054	59530070	4.25	.	.	intergenic	0.12	rs793469006	2
AC.GT	4	59585451	59585473	11.5	.	.	intergenic	-0.098	rs787508529	3
AT.AT	4	59832080	59832097	9	.	.	intergenic	.	.	2
AAAC.GTTT	4	59928875	59928889	3.75	.	.	intergenic	0.231	06142 rs7894	2
ATCC.GGAT	4	60024777	60024804	7	.	.	intergenic	0.12	.	4
AAAC.GTTT	4	60195697	60195723	6.75	.	.	intergenic	.	rs790885019	2
AG.CT	4	60468319	60468332	7	.	.	intergenic	-0.154	52769 rs7914	2
AAAG.CTTT	4	60518388	60518409	5.5	.	.	intergenic	-0.152	rs787386957	2
ATC.GAT	4	61011876	61011891	5.33333	.	.	intergenic	-0.085	.	3
AC.GT	4	61019802	61019818	8.5	.	.	intergenic	-0.145	91323 rs7876	2
AAAAT.ATTTT	4	61037904	61037926	4.6	.	.	intergenic	.	rs788797221	2
AAAC.GTTT	4	61247645	61247663	4.75	.	.	intergenic	0.187	87072 rs7864	2
AT.AT	4	61285500	61285511	6	ENSSSCT00000029360	ZBTB10	intron	0.086	rs790892746	2
AT.AT	4	61285500	61285511	6	ENSSSCT00000006749	ZBTB10	intron	0.086	rs790892746	2
AAAAT.ATTTT	4	61423307	61423327	4.2	.	.	intergenic	-0.205	rs793543315	2
AAAT.ATTT	4	61437964	61437978	3.75	.	.	intergenic	0.182	rs791870482	3
AC.GT	4	61513928	61513942	7.5	.	.	intergenic	0.182	rs787090840	3
AAAT.ATTT	4	61588718	61588740	5.75	.	.	intergenic	-0.021	09701 rs7916	2
AAAT.ATTT	4	61735365	61735386	5.5	.	.	intergenic	-0.129	30648 rs7920	2
AT.AT	4	61890316	61890343	14	ENSSSCT00000006752	MRPS28	intron	0.023	.	4
AAAAT.ATTTT	4	62098055	62098077	4.6	.	.	intergenic	0.046	89518 rs7914	2
AC.GT	4	62220995	62221020	13	ENSSSCT00000035189	STMN2	intron	0.592	16993 rs7922	2
AC.GT	4	62220995	62221020	13	ENSSSCT00000006754	STMN2	intron	0.592	16993 rs7922	2
AC.GT	4	62220995	62221020	13	ENSSSCT00000036553	STMN2	intron	0.592	16993 rs7922	2
AC.GT	4	62220995	62221020	13	ENSSSCT00000033041	STMN2	intron	0.592	16993 rs7922	2
AC.GT	4	62220995	62221020	13	ENSSSCT00000032694	STMN2	intron	0.592	16993 rs7922	2
AAC.GTT	4	62262582	62262607	8.66667	.	.	intergenic	.	34921 rs7930	2
AC.GT	4	62399801	62399812	6	.	.	intergenic	-0.085	rs791950094	2
AAAC.GTTT	4	62459737	62459755	4.75	.	.	intergenic	0.008	rs786649829	2
AAT.ATT	4	62875252	62875266	5	.	.	intergenic	0.04	rs792171427	2
AC.GT	4	63184517	63184549	16.5	.	.	intergenic	0.038	10175 rs7889	4
AG.CT	4	63250652	63250687	18	.	.	intergenic	0.02	rs706664001	2
AT.AT	4	63357765	63357779	7.5	.	.	intergenic	0.068	rs788988351	2
AC.GT	4	63401524	63401535	6	.	.	intergenic	-0.013	rs786302516	2
AAC.GTT	4	63412343	63412365	7.66667	.	.	intergenic	-0.121	rs787333275	2
AAAC.GTTT	4	63916707	63916730	6	.	.	intergenic	0.156	187177 rs7921	3
AAG.CTT	4	63955034	63955046	4.33333	.	.	intergenic	-0.179	rs790508067	2
AAC.GTT	4	63967983	63967995	4.33333	.	.	intergenic	.	rs792591159	3
AAAC.GTTT	4	63996959	63996973	3.75	.	.	intergenic	.	rs788650448	2
CATAT.ATATG	4	64205555	64205579	4.16667	.	.	intergenic	1.795	rs792986938	2
AAAT.ATTT	4	64425202	64425221	5	.	.	intergenic	.	rs791741543	2
AAAT.ATTT	4	64435773	64435810	9.5	.	.	intergenic	0.096	rs787185404	2
AC.GT	4	64445823	64445845	11.5	.	.	intergenic	0.36	.	2
ACAT.ATGT	4	64484537	64484548	3	.	.	intergenic	-0.194	.	2
AG.CT	4	64550810	64550820	5.5	.	.	intergenic	-0.143	rs792626519	2
AAAC.GTTT	4	64577372	64577386	3.75	.	.	intergenic	.	rs790975967	2
AAAAT.ATTTT	4	64585784	64585810	5.4	.	.	intergenic	0.081	39955 rs7883	2
AT.AT	4	64633783	64633795	6.5	.	.	intergenic	0.091	rs787397896	3
AAGG.CCTT	4	64641954	64641974	5.25	.	.	intergenic	0.082	119271 rs7915	2
AATT.AATT	4	64806944	64806965	5.5	.	.	intergenic	-0.217	rs792760403	2
AAAT.ATTT	4	64822127	64822151	6.25	.	.	intergenic	0.267	rs791639481	2

AT.AT	4	64937515	64937524	5	ENSSSCT00000006763	ZFHX4	intron	0.074	.	4
AC.GT	4	65258670	65258685	8	.	.	intergenic	0.125	410744 rs7881	2
ACC.GGT	4	65592147	65592159	4.33333	.	.	intergenic	-0.081	rs788446138	2
AC.GT	4	65995479	65995491	6.5	.	.	intergenic	1.024	rs790388685	3
AG.CT	4	66197460	66197472	6.5	.	.	intergenic	-0.025	958355 rs7921	2
AAAT.ATTT	4	66387498	66387511	3.5	.	.	intergenic	0.175	rs786514647	2
AC.GT	4	66517051	66517071	10.5	.	.	intergenic	-0.337	.	3
AC.GT	4	66912417	66912426	5	.	.	intergenic	0.631	rs789713290	2
AT.AT	4	66958113	66958127	7.5	.	.	intergenic	.	rs790060720	2
AC.GT	4	66998827	66998837	5.5	.	.	intergenic	0.06	rs792991092	2
AT.AT	4	67008794	67008804	5.5	.	.	intergenic	.	rs787784436	2
AC.GT	4	67046214	67046230	8.5	.	.	intergenic	0.13	rs791001633	3
AAAC.GTTT	4	67103020	67103035	4	.	.	intergenic	.	rs793363165	2
AC.GT	4	67149108	67149118	5.5	.	.	intergenic	0.16	rs792020106	2
AAAC.GTTT	4	67195843	67195864	5.5	ENSSSCT00000006767	GDAP1	intron	.	rs792952822	2
AC.GT	4	67240957	67240977	10.5	.	.	intergenic	0.114	764370 rs7893	4
AC.GT	4	67512177	67512199	11.5	.	.	intergenic	.	720554 rs7938	5
AAAT.ATTT	4	67596527	67596541	3.75	.	.	intergenic	.	rs790873196	2
AC.GT	4	67597519	67597528	5	.	.	intergenic	-0.038	.	2
AAAAC.GTTTT	4	67630599	67630621	4.6	ENSSSCT00000035167	MD-2	intron	.	rs793448898	2
AAAAC.GTTTT	4	67630599	67630621	4.6	ENSSSCT00000006769	MD-2	intron	.	rs793448898	2
AAAG.CTTT	4	67646001	67646019	4.75	ENSSSCT00000006769	MD-2	intron	0.426	rs792057885	2
AAAG.CTTT	4	67646001	67646019	4.75	ENSSSCT00000035167	MD-2	intron	0.426	rs792057885	2
AAAC.GTTT	4	67686701	67686729	7.25	.	.	intergenic	0.208	.	2
AC.GT	4	67689449	67689458	5	ENSSSCT00000006770	TCEB1	promoter	0.319	rs789254298	2
AAAT.ATTT	4	67765138	67765157	5	ENSSSCT00000006771	.	intron	0.358	rs791973344	2
AAT.ATT	4	67807958	67807970	4.33333	.	.	intergenic	.	rs791378816	2
AAAC.GTTT	4	67814830	67814842	3.25	.	.	intergenic	-0.012	.	2
AAAG.CTTT	4	67819354	67819366	3.25	.	.	intergenic	.	.	3
AC.GT	4	67881440	67881454	7.5	.	.	intergenic	.	rs789318612	6
AAAT.ATTT	4	67918929	67918948	5	.	.	intergenic	0.054	rs790690157	2
AG.CT	4	68106396	68106425	15	.	.	intergenic	0.336	rs787530036	2
AAAAC.GTTTT	4	68126871	68126899	5.8	.	.	intergenic	.	rs787622100	2
AC.GT	4	68565520	68565538	9.5	.	.	intergenic	0.044	891419 rs7882	3
AT.AT	4	68589281	68589291	5.5	.	.	intergenic	.	rs786336742	2
AAGG.CCTT	4	68836041	68836082	10.5	.	.	intergenic	.	rs789480682	2
AT.AT	4	69253653	69253664	6	ENSSSCT00000006780	.	intron	0.049	766862 rs7914	2
AAAAT.ATTTT	4	69358023	69358039	3.4	.	.	intergenic	-0.306	.	2
AC.GT	4	69383931	69383952	11	.	.	intergenic	.	rs788057312	4
AAC.GTT	4	69501220	69501233	4.66667	.	.	intergenic	-0.218	rs790937791	2
AGG.CCT	4	69581229	69581245	5.66667	.	.	intergenic	-0.281	028223 rs7935	2
AT.AT	4	69621226	69621235	5	.	.	intergenic	0.59	.	2
AAAT.ATTT	4	69823345	69823365	5.25	.	.	intergenic	0.077	rs787899194	3
AAAC.GTTT	4	69837097	69837115	4.75	.	.	intergenic	.	rs789719638	2
AG.CT	4	69873988	69874002	7.5	.	.	intergenic	0.452	rs786759634	3
AT.AT	4	69885041	69885059	9.5	.	.	intergenic	0.232	rs786663566	2
AT.AT	4	70172233	70172249	8.5	.	.	intergenic	.	rs788717871	2
AT.AT	4	70189172	70189183	6	.	.	intergenic	0.015	.	3
AC.GT	4	70234140	70234165	13	.	.	intergenic	.	.	4
AAT.ATT	4	70352905	70352916	4	ENSSSCT00000006786	LACTB2	intron	0.327	.	3
AAAG.CTTT	4	70480592	70480609	4.5	.	.	intergenic	0.322	rs790631591	2
AAAG.CTTT	4	70982450	70982478	7.25	.	.	intergenic	0.067	834581 rs7916	2
AAAC.GTTT	4	71012904	71012923	5	.	.	intergenic	.	rs788684339	2
AAAC.GTTT	4	71232755	71232777	5.75	ENSSSCT00000006791	SLCO5A1	intron	0.026	rs790374917	2
AC.GT	4	71368951	71368966	8	ENSSSCT00000006792	SULF1	intron	-0.008	986263 rs7908	2
AC.GT	4	71396331	71396343	6.5	ENSSSCT00000006792	SULF1	intron	0.211	rs790567437	2
AC.GT	4	72033786	72033806	10.5	.	.	intergenic	.	671179 rs7932	2
AT.AT	4	72337357	72337366	5	.	.	intergenic	0.085	rs792302027	2
AT.AT	4	72564757	72564769	6.5	.	.	intergenic	0.331	935116 rs7900	2
AAAC.GTTT	4	72579652	72579669	4.5	.	.	intergenic	0.048	rs789812612	2
AT.AT	4	72587082	72587100	9.5	.	.	intergenic	0.364	653686 rs7866	2
AAAAAC.GTTTTT	4	72625341	72625372	5.33333	.	.	intergenic	0.047	rs788973644	2
AAT.ATT	4	72632382	72632394	4.33333	.	.	intergenic	.	.	2
AG.CT	4	73131717	73131728	6	ENSSSCT00000034412	CPA6	intron	0.009	rs787386044	2
AG.CT	4	73354788	73354797	5	ENSSSCT00000006795	CPA6	intron	0.408	rs705285566	2
AG.CT	4	73354788	73354797	5	ENSSSCT00000034412	CPA6	intron	0.408	rs705285566	2
AATT.AATT	4	73365748	73365761	3.5	ENSSSCT00000006795	CPA6	intron	.	rs786854835	2
AATT.AATT	4	73365748	73365761	3.5	ENSSSCT00000034412	CPA6	intron	.	rs786854835	2
ACAT.ATGT	4	73470743	73470761	4.75	.	.	intergenic	0.01	rs788317004	2
AG.CT	4	73481119	73481133	7.5	.	.	intergenic	-0.298	rs793378025	2
AAAC.GTTT	4	73506788	73506807	5	.	.	intergenic	-0.049	rs792239294	3
AC.GT	4	73652963	73652977	7.5	ENSSSCT00000006796	ARFGEF1	intron	0.044	rs705284403	3
AC.GT	4	73652963	73652977	7.5	ENSSSCT00000035957	ARFGEF1	intron	0.044	rs705284403	3
AT.AT	4	73923573	73923591	9.5	ENSSSCT00000006800	MCMDC2	intron	0.368	rs789966123	2
ACC.GGT	4	74373197	74373210	4.66667	ENSSSCT00000032451	C8orf46	intron	-0.047	.	2
AAT.ATT	4	74493291	74493302	4	.	.	intergenic	.	rs786390115	2
AC.GT	4	74630835	74630854	10	.	.	intergenic	0.041	675808 rs7888	2
AT.AT	4	74961051	74961062	6	.	.	intergenic	0.285	rs792179032	2
AAAT.ATTT	4	75022732	75022748	4.25	.	.	intergenic	.	rs791365378	2
AAAT.ATTT	4	75023101	75023123	5.75	.	.	intergenic	.	rs788072529	2
AAAT.ATTT	4	75155499	75155520	5.5	ENSSSCT00000006816	ARMC1	intron	.	rs790457173	2
AAAT.ATTT	4	75183321	75183350	7.5	.	.	intergenic	0.7	rs789367345	2
AAAG.CTTT	4	75717084	75717098	3.75	.	.	intergenic	2.031	rs788996072	2
AAAT.ATTT	4	75927713	75927738	6.5	ENSSSCT00000031830	.	intron	-0.018	rs786386554	4

AAC.GTT	4	76000829	76000853	8.33333	.	.	intergenic	0.846	396617 rs7867	2
AATT.AATT	4	76031549	76031579	7.75	.	.	intergenic	0.143	rs791943543	2
AAAAC.GTTTT	4	76146136	76146155	4	.	.	intergenic	0.235	.	2
AAAC.GTTT	4	76338094	76338117	6	.	.	intergenic	.	rs790266601	2
AG.CT	4	76542754	76542772	9.5	.	.	intergenic	0.204	300038 rs7924	6
AAAAAC.GTTTTT	4	76776727	76776760	5.66667	.	.	intergenic	0.156	rs793551142	2
AAAT.ATTT	4	76812951	76812965	3.75	.	.	intergenic	.	521914 rs7882	2
AAGAG.CTCTT	4	77185759	77185782	4.8	.	.	intergenic	0.09	rs792044911	2
AG.CT	4	77362501	77362512	6	.	.	intergenic	0.101	rs790235620	3
AG.CT	4	77387152	77387163	6	.	.	intergenic	0.198	771388 rs7926	2
AC.GT	4	77393689	77393705	8.5	.	.	intergenic	0.038	.	4
AC.GT	4	77402946	77402961	8	.	.	intergenic	0.015	.	2
AG.CT	4	77405499	77405515	8.5	.	.	intergenic	.	rs697829498	3
AAAAC.GTTTT	4	77406742	77406767	5.2	.	.	intergenic	.	rs793014921	2
AT.AT	4	77718097	77718108	6	.	.	intergenic	.	.	3
AC.GT	4	77733984	77733994	5.5	.	.	intergenic	.	.	2
AAAG.CTTT	4	77791535	77791572	9.5	ENSSSCT00000020920	U6	exon	-0.006	rs791274123	2
AT.AT	4	78124810	78124819	5	.	.	intergenic	-0.073	rs788986480	2
AAAC.GTTT	4	78132090	78132122	8.25	.	.	intergenic	.	066805 rs7910	2
AC.GT	4	78231685	78231695	5.5	.	.	intergenic	0.096	rs790134924	3
AAT.ATT	4	78480311	78480332	7.33333	ENSSSCT00000028806	.	intron	-0.193	rs790108025	2
ACATAT.ATATGT	4	78486494	78486513	3.33333	ENSSSCT00000028806	.	intron	0.118	rs791719738	2
AAGG.CCTT	4	78498919	78498937	4.75	ENSSSCT00000028806	.	intron	0.052	451822 rs7897	2
AG.CT	4	78574050	78574062	6.5	ENSSSCT00000006827	CLVS1	intron	.	.	2
AAAC.GTTT	4	78590366	78590380	3.75	ENSSSCT00000006827	CLVS1	intron	0.013	rs792363867	2
AAAT.ATTT	4	78630452	78630482	7.75	ENSSSCT00000006827	CLVS1	intron	0.198	.	2
AAC.GTT	4	78662583	78662603	7	.	.	intergenic	0.187	rs787259919	2
AAAT.ATTT	4	78806869	78806883	3.75	.	.	intergenic	0.078	.	2
AGAT.ATCT	4	78853475	78853491	4.25	.	.	intergenic	-0.156	rs791027929	2
AATG.CATT	4	78930287	78930316	7.5	.	.	intergenic	0.067	rs786284797	2
AAAC.GTTT	4	79062249	79062273	6.25	ENSSSCT00000006828	CHD7	intron	0.006	rs791777595	2
AT.AT	4	79411781	79411797	8.5	.	.	intergenic	.	rs793014430	2
AG.CT	4	80254646	80254656	5.5	.	.	intergenic	-0.175	rs787980390	2
AT.AT	4	80257507	80257516	5	.	.	intergenic	0.401	rs792277190	2
ACAGAT.ATCTGT	4	80333863	80333890	4.66667	.	.	intergenic	-0.107	.	2
AAAC.GTTT	4	80756320	80756345	6.5	ENSSSCT00000006832	TOX	promoter	0.448	rs792492372	2
AC.GT	4	81168813	81168825	6.5	.	.	intergenic	.	rs793325325	2
AAT.ATT	4	81244253	81244270	6	.	.	intergenic	0.119	rs788653026	2
AAAAC.GTTTT	4	81263103	81263119	3.4	.	.	intergenic	0.307	rs786865829	2
AC.GT	4	81340467	81340479	6.5	.	.	intergenic	-0.001	rs786375036	2
AAAAC.GTTTT	4	81496043	81496060	3.6	.	.	intergenic	-0.172	rs791710636	2
AAAC.GTTT	4	81568631	81568649	4.75	.	.	intergenic	-0.109	rs792273484	2
AAAAC.GTTTT	4	81574908	81574927	4	.	.	intergenic	-0.22	rs788878242	2
AT.AT	4	81775446	81775455	5	.	.	intergenic	-0.047	rs787849321	2
AAAC.GTTT	4	81788301	81788323	5.75	.	.	intergenic	-0.168	553543 rs7867	2
AAAT.ATTT	4	81925100	81925126	6.75	.	.	intergenic	.	rs792528956	3
AC.GT	4	82058963	82058975	6.5	.	.	intergenic	-0.01	.	3
AAC.GTT	4	82177263	82177282	6.66667	.	.	intergenic	-0.078	199044 rs7884	2
AAT.ATT	4	82245326	82245346	7	.	.	intergenic	0.099	rs709999110	2
AAT.ATT	4	82303988	82304004	5.66667	.	.	intergenic	0.034	rs790849375	4
AAC.GTT	4	82305537	82305550	4.66667	.	.	intergenic	.	rs791917291	2
AAC.GTT	4	82340910	82340929	6.66667	.	.	intergenic	0.114	rs793449986	2
AAAAC.GTTTT	4	82434397	82434432	7.2	.	.	intergenic	0.143	rs792830818	2
AAAC.GTTT	4	82519637	82519670	8.5	.	.	intergenic	0.389	rs787644951	2
AAAC.GTTT	4	82546349	82546361	3.25	.	.	intergenic	-0.092	rs792446379	2
AAC.GTT	4	82708163	82708185	7.66667	.	.	intergenic	0.082	rs788756505	2
AAAC.GTTT	4	82751583	82751596	3.5	ENSSSCT00000006849	LYN	intron	-0.002	rs788920120	2
AC.GT	4	82917915	82917926	6	ENSSSCT00000006851	.	intron	.	rs790038123	3
AAGC.GCTT	4	82928812	82928836	6.25	ENSSSCT00000006851	.	intron	0.019	.	4
AAG.CTT	4	83041881	83041892	4	.	.	intergenic	0.074	928454 rs7887	2
AAAT.ATTT	4	83134114	83134137	6	.	.	intergenic	-0.001	018933 rs7907	4
AT.AT	4	83232668	83232679	6	.	.	intergenic	-0.045	304627 rs7896	2
AG.CT	4	83482960	83482973	7	.	.	intergenic	.	rs793470337	4
ACCT.AGGT	4	83614213	83614228	4	.	.	intergenic	0.301	.	3
AC.GT	4	83774484	83774496	6.5	.	.	intergenic	0.127	.	4
AAAC.GTTT	4	84049000	84049025	6.5	.	.	intergenic	-0.235	rs787168752	2
AAAT.ATTT	4	84101210	84101225	4	.	.	intergenic	-0.019	520020 rs7887	2
AAT.ATT	4	84433036	84433057	7.33333	.	.	intergenic	0.085	.	2
AG.CT	4	84503313	84503324	6	.	.	intergenic	-0.235	rs787908390	2
AAAC.GTTT	4	84633110	84633124	3.75	.	.	intergenic	-0.02	rs788378730	2
AT.AT	4	85011277	85011291	7.5	.	.	intergenic	0.038	.	2
AC.GT	4	85011358	85011372	7.5	.	.	intergenic	0.062	rs792808921	2
AAAG.CTTT	4	85266985	85267013	7.25	.	.	intergenic	.	rs792680446	2
AC.GT	4	85424877	85424888	6	.	.	intergenic	0.067	.	4
AAACT.AGTTT	4	85624382	85624402	4.2	.	.	intergenic	-0.03	255508 rs7880	2
AC.GT	4	85624920	85624929	5	.	.	intergenic	-0.141	rs793710280	2
AT.AT	4	86509775	86509784	5	.	.	intergenic	0.078	rs790849629	2
AG.CT	4	86510924	86510935	6	.	.	intergenic	0.078	rs787543531	2
AC.GT	4	86680384	86680394	5.5	ENSSSCT00000006869	SNAI2	cds	1.388	.	2
AC.GT	4	86768973	86768993	10.5	.	.	intergenic	0.522	.	2
AAATG.CATTT	4	87013290	87013308	3.8	.	.	intergenic	0	rs788681442	2
AAG.CTT	4	87329329	87329340	4	ENSSSCT00000006873	PRKDC	intron	0.077	rs790470818	2
AC.GT	4	87549352	87549371	10	ENSSSCT00000006876	SPIDR	intron	0.297	rs790259410	2
AAAC.GTTT	4	87823194	87823210	4.25	.	.	intergenic	.	.	4

AT.AT	4	88031336	88031346	5.5	.	.	intergenic	-0.033	.	2
AAAC.GTTT	4	88108869	88108889	5.25	.	.	intergenic	0.137	rs787714106	2
AG.CT	4	88417235	88417244	5	.	.	intergenic	-0.072	rs789065087	3
AAAAAT.ATTTTT	4	88468842	88468861	3.33333	.	.	intergenic	.	.	2
AAC.GTT	4	88622432	88622446	5	ENSSSCT00000006882	KIFAP3	intron	0.104	.	2
AAAAC.GTTTT	4	88754552	88754570	3.8	.	.	intergenic	.	rs791632931	2
AT.AT	4	88811832	88811860	14.5	ENSSSCT00000006884	C1orf112	intron	0.092	rs793683184	2
AATG.CATT	4	88811992	88812008	4.25	ENSSSCT00000006884	C1orf112	intron	0.19	.	2
AT.AT	4	88822704	88822728	12.5	ENSSSCT00000006884	C1orf112	intron	0.309	rs791128782	3
AAG.CTT	4	88991797	88991808	4	ENSSSCT00000006891	SELP	intron	0.082	rs789359555	2
AAG.CTT	4	88991797	88991808	4	ENSSSCT00000036036	SELP	intron	0.082	rs789359555	2
AAAT.ATTT	4	88993023	88993041	4.75	ENSSSCT00000006891	SELP	intron	0.119	rs790854616	2
AAAT.ATTT	4	88993023	88993041	4.75	ENSSSCT00000036036	SELP	intron	0.119	rs790854616	2
AAT.ATT	4	89012472	89012483	4	ENSSSCT00000036036	SELP	intron	0.13	.	2
AAT.ATT	4	89012472	89012483	4	ENSSSCT00000006891	SELP	intron	0.13	.	2
AAT.ATT	4	89012472	89012483	4	ENSSSCT00000035164	SELP	intron	0.13	.	2
AAAAAT.ATTTTT	4	89335165	89335192	4.66667	ENSSSCT00000006898	NME7	intron	-0.048	rs790334025	2
AAAC.GTTT	4	89409028	89409051	6	.	.	intergenic	.	345362 rs7868	2
AC.GT	4	89415724	89415746	11.5	.	.	intergenic	.	rs793762164	7
AAAAC.GTTTT	4	89471891	89471912	4.4	.	.	intergenic	.	rs792925704	2
AG.CT	4	89477755	89477771	8.5	.	.	intergenic	.	rs790076869	2
AAAAT.ATTTT	4	89506804	89506821	3.6	.	.	intergenic	.	rs791313268	2
AC.GT	4	89587013	89587033	10.5	.	.	intergenic	.	.	2
AAAAT.ATTTT	4	89738240	89738263	4.8	.	.	intergenic	.	368063 rs7894	2
AAAT.ATTT	4	89738446	89738460	3.75	.	.	intergenic	-0.044	rs792172536	2
AT.AT	4	89945509	89945525	8.5	.	.	intergenic	-0.079	.	2
AAC.GTT	4	90091399	90091413	5	.	.	intergenic	-0.113	rs713488607	3
AAAAG.CTTTT	4	90258733	90258753	4.2	.	.	intergenic	.	rs793090798	2
AAAC.GTTT	4	90376294	90376305	3	.	.	intergenic	.	rs791091989	2
AAC.GTT	4	90487976	90487996	7	.	.	intergenic	0.188	.	2
AAAT.ATTT	4	90768058	90768076	4.75	.	.	intergenic	0.218	rs789927264	2
AAAG.CTTT	4	90796553	90796572	5	ENSSSCT00000006910	ADCY10	intron	0.015	rs793058620	2
AAAAC.GTTTT	4	90864021	90864051	6.2	ENSSSCT00000006910	ADCY10	intron	-0.392	rs788313252	2
AAG.CTT	4	90914520	90914535	5.33333	ENSSSCT00000006911	MPZL1	intron	0.004	rs793008061	2
AATT.AATT	4	90998901	90998926	6.5	ENSSSCT00000006912	RCSD1	3'utr	-0.12	rs792027788	2
AAAT.ATTT	4	91091752	91091772	5.25	.	.	intergenic	.	rs793303659	3
AAG.CTT	4	91265549	91265566	6	.	.	intergenic	0.46	394951 rs7921	2
AAAC.GTTT	4	91517032	91517059	7	.	.	intergenic	-0.037	rs788653903	4
AC.GT	4	91715067	91715091	12.5	ENSSSCT00000006923	.	promoter	.	rs787988874	2
AAAT.ATTT	4	91721044	91721061	4.5	ENSSSCT00000006923	.	intron	.	rs788539225	2
AAC.GTT	4	91737496	91737509	4.66667	.	.	intergenic	.	rs787976200	2
AG.CT	4	91910871	91910882	6	ENSSSCT00000006926	POGK	intron	0.779	rs788601207	3
AT.AT	4	91930446	91930474	14.5	.	.	intergenic	0.164	.	2
AGG.CCT	4	92268259	92268271	4.33333	.	.	intergenic	.	439846 rs7915	2
AAT.ATT	4	92336124	92336144	7	ENSSSCT00000006928	FAM78B	intron	.	rs787539666	2
AAAT.ATTT	4	92628290	92628324	8.75	ENSSSCT00000006931	TMCO1	intron	0.109	rs793081998	2
AT.AT	4	92661199	92661208	5	ENSSSCT00000006931	TMCO1	intron	0.172	.	4
AAC.GTT	4	92664872	92664897	8.66667	ENSSSCT00000006931	TMCO1	intron	-0.029	.	2
AC.GT	4	92857935	92857946	6	ENSSSCT00000027133	LRRC52	intron	0.046	.	3
AAAAT.ATTTT	4	92963272	92963290	3.8	.	.	intergenic	0.107	rs792607926	2
AAAT.ATTT	4	92971174	92971201	7	.	.	intergenic	0.404	159936 rs7913	2
AAT.ATT	4	93035849	93035863	5	ENSSSCT00000006936	RXRG	intron	-0.003	rs786470010	2
AAT.ATT	4	93035849	93035863	5	ENSSSCT00000033501	RXRG	intron	-0.003	rs786470010	2
AT.AT	4	93105835	93105844	5	.	.	intergenic	0	rs791034991	2
AC.GT	4	93175685	93175696	6	.	.	intergenic	0.115	.	2
AC.GT	4	93223485	93223497	6.5	.	.	intergenic	-0.006	rs790582539	2
AAAC.GTTT	4	93321105	93321125	5.25	.	.	intergenic	.	rs787804878	2
AAAAT.ATTTT	4	93353587	93353610	4.8	.	.	intergenic	.	rs793539293	2
AAAT.ATTT	4	93548383	93548406	6	.	.	intergenic	-0.147	039117 rs7901	2
AATC.GATT	4	93960329	93960351	5.75	.	.	intergenic	0.253	rs790060932	2
AT.AT	4	93966186	93966197	6	.	.	intergenic	0.028	rs787565532	2
AG.CT	4	93971442	93971451	5	.	.	intergenic	-0.067	rs790219847	2
AC.GT	4	93982967	93982977	5.5	.	.	intergenic	.	451522 rs7873	2
AT.AT	4	93993864	93993874	5.5	.	.	intergenic	-0.105	rs789464046	2
AC.GT	4	94036235	94036245	5.5	.	.	intergenic	0.617	.	3
AT.AT	4	94374878	94374888	5.5	.	.	intergenic	.	rs793425999	2
AG.CT	4	94415681	94415708	14	.	.	intergenic	1.47	rs788598611	5
AAAT.ATTT	4	94422436	94422454	4.75	.	.	intergenic	-0.068	rs786568166	2
AC.GT	4	94467880	94467894	7.5	.	.	intergenic	-0.04	995133 rs7866	2
AAAC.GTTT	4	94489927	94489946	5	.	.	intergenic	0.002	341726 rs7910	3
AT.AT	4	94671909	94671924	8	.	.	intergenic	.	521804 rs7928	2
AAAC.GTTT	4	94747557	94747568	3	.	.	intergenic	0.009	rs787352350	2
AT.AT	4	94753511	94753520	5	.	.	intergenic	0.027	rs792789597	2
AAAC.GTTT	4	94771725	94771763	9.75	.	.	intergenic	0.211	594735 rs7937	2
AC.GT	4	94896356	94896370	7.5	ENSSSCT00000006941	NUF2	intron	.	rs786726779	2
AAAAG.CTTTT	4	95042931	95042958	5.6	ENSSSCT00000006942	RGS5	intron	0.038	058224 rs7912	2
AC.GT	4	95052713	95052723	5.5	ENSSSCT00000006942	RGS5	3'utr	0.098	182135 rs7917	4
AAAAAG.CTTTTT	4	95093878	95093899	3.66667	.	.	intergenic	.	703994 rs7864	2
AAAC.GTTT	4	95182097	95182129	8.25	.	.	intergenic	-0.114	rs787847532	2
AAAC.GTTT	4	95337025	95337058	8.5	.	.	intergenic	.	rs695474459	2
AC.GT	4	95372204	95372213	5	.	.	intergenic	0.057	.	3
AAC.GTT	4	95457929	95457949	7	.	.	intergenic	-0.031	rs789313766	2
AT.AT	4	95495137	95495156	10	.	.	intergenic	.	rs789035432	2
AC.GT	4	95702637	95702652	8	.	.	intergenic	-0.258	342812 rs7927	2

AC.GT	4	95970588	95970599	6	.	.	intergenic	.	464923 rs7918	2
AT.AT	4	96194910	96194920	5.5	.	.	intergenic	.	580277 rs7926	2
AT.AT	4	96275614	96275623	5	.	.	intergenic	0.006	rs792968449	2
AC.GT	4	96302958	96302968	5.5	.	.	intergenic	-0.135	.	3
AC.GT	4	96522030	96522043	7	.	.	intergenic	-0.239	342024 rs7866	2
AAT.ATT	4	96868587	96868603	5.66667	ENSSSCT00000006957	.	intron	-0.038	957409 rs7882	2
AG.CT	4	96901669	96901713	22.5	ENSSSCT00000033832	CD32	intron	-0.348	428341 rs7897	4
AG.CT	4	96901669	96901713	22.5	ENSSSCT00000033848	CD32	intron	-0.348	428341 rs7897	4
AG.CT	4	96901669	96901713	22.5	ENSSSCT00000006958	CD32	intron	-0.348	428341 rs7897	4
AG.CT	4	96901669	96901713	22.5	ENSSSCT00000033401	CD32	intron	-0.348	428341 rs7897	4
AG.CT	4	96901669	96901713	22.5	ENSSSCT00000006959	CD32	intron	-0.348	428341 rs7897	4
AG.CT	4	96901669	96901713	22.5	ENSSSCT00000031531	CD32	intron	-0.348	428341 rs7897	4
AG.CT	4	96901669	96901713	22.5	ENSSSCT00000035271	CD32	intron	-0.348	428341 rs7897	4
AT.AT	4	97056643	97056656	7	.	.	intergenic	.	.	3
AC.GT	4	97330624	97330638	7.5	ENSSSCT00000006979	F11R	intron	.	rs712139257	2
AC.GT	4	97330624	97330638	7.5	ENSSSCT00000033820	F11R	intron	.	rs712139257	2
AC.GT	4	97355223	97355238	8	.	.	intergenic	.	252415 rs7871	3
AC.GT	4	97370994	97371003	5	.	.	intergenic	-0.019	rs787426485	2
AAAT.ATTT	4	97532675	97532693	4.75	ENSSSCT00000034681	LY9	intron	0.078	rs793218981	2
AAAT.ATTT	4	97532675	97532693	4.75	ENSSSCT00000006986	LY9	intron	0.078	rs793218981	2
AAAT.ATTT	4	97532675	97532693	4.75	ENSSSCT00000036000	LY9	intron	0.078	rs793218981	2
AAGG.CCTT	4	97622363	97622380	4.5	ENSSSCT00000006984	.	promoter	.	100339 rs7091	2
AC.GT	4	97737192	97737205	7	.	.	intergenic	0.223	412844 rs7892	4
AT.AT	4	98734458	98734468	5.5	.	.	intergenic	0.198	rs787290926	2
ACC.GGT	4	98928833	98928852	6.66667	.	.	intergenic	-0.059	rs791774658	2
AAAAC.GTTTT	4	98961477	98961503	5.4	.	.	intergenic	-0.317	rs786360782	2
AC.GT	4	99054937	99054968	16	.	.	intergenic	-0.046	.	2
AAAC.GTTT	4	99064897	99064911	3.75	.	.	intergenic	.	rs791217755	2
AC.GT	4	99134239	99134249	5.5	.	.	intergenic	-0.067	.	3
AG.CT	4	99365376	99365390	7.5	.	.	intergenic	-0.221	rs788673186	3
AAAT.ATTT	4	99529983	99530004	5.5	.	.	intergenic	0.048	rs791486968	2
AT.AT	4	99540169	99540179	5.5	.	.	intergenic	.	rs792580102	2
AAAC.GTTT	4	100458847	100458861	3.75	ENSSSCT00000035228	CD1D	intron	.	rs787616185	2
AC.GT	4	100541776	100541789	7	ENSSSCT00000033538	KIRREL	intron	-0.212	.	2
AC.GT	4	100541776	100541789	7	ENSSSCT00000007068	KIRREL	intron	-0.212	.	2
AC.GT	4	100541776	100541789	7	ENSSSCT00000036287	KIRREL	intron	-0.212	.	2
AAAC.GTTT	4	100711556	100711584	7.25	.	.	intergenic	-0.036	rs694080126	2
AG.CT	4	100825631	100825645	7.5	ENSSSCT00000007074	FCRL3	intron	.	124296 rs7905	2
AAG.CTT	4	101034488	101034501	4.66667	.	.	intergenic	.	238106 rs7876	2
AAAAAT.ATTTTT	4	101518025	101518045	3.5	ENSSSCT00000007078	ARHGEF11	intron	-0.015	rs790652761	2
AC.GT	4	101557538	101557549	6	ENSSSCT00000007078	ARHGEF11	intron	-0.108	rs792463940	2
AAAC.GTTT	4	101788297	101788325	7.25	.	.	intergenic	-0.207	rs789472489	2
AT.AT	4	101871516	101871531	8	ENSSSCT00000007084	PRCC	intron	0.257	rs792310514	3
AAAAC.GTTTT	4	101983084	101983105	4.4	ENSSSCT00000007091	.	promoter	-0.034	573463 rs7887	2
AAAAC.GTTTT	4	101983084	101983105	4.4	ENSSSCT00000027298	.	promoter	-0.034	573463 rs7887	2
AAAAC.GTTTT	4	101983084	101983105	4.4	ENSSSCT00000031509	.	promoter	-0.034	573463 rs7887	2
AAAC.GTTT	4	102189331	102189353	5.75	.	.	intergenic	.	rs792899737	2
AAAAAC.GTTTTT	4	102264266	102264298	5.5	.	.	intergenic	-0.189	rs787642295	2
AAAC.GTTT	4	102424923	102424950	7	.	.	intergenic	.	.	3
AAAAT.ATTTT	4	102441185	102441206	4.4	ENSSSCT00000007106	CCT3	intron	0.086	rs790235469	2
AG.CT	4	102472088	102472098	5.5	ENSSSCT00000007109	SMG5	intron	0.239	764441 rs7937	3
AAC.GTT	4	102908819	102908833	5	ENSSSCT00000028079	.	intron	0.099	rs792003574	4
AC.GT	4	103367631	103367644	7	ENSSSCT00000007143	GBA	intron	-0.215	rs791456973	2
AAC.GTT	4	103368694	103368710	5.66667	ENSSSCT00000007143	GBA	intron	.	rs792493183	4
AAAC.GTTT	4	103719802	103719820	4.75	ENSSSCT00000007166	KCNN3	intron	-0.226	504372 rs7893	2
AAAC.GTTT	4	103867239	103867253	3.75	ENSSSCT00000007166	KCNN3	intron	-0.075	rs788048366	2
AC.GT	4	104029472	104029481	5	ENSSSCT00000007170	CHRNB2	intron	-0.023	rs788715561	3
AAAT.ATTT	4	104524511	104524533	5.75	.	.	intergenic	.	rs789048763	2
AT.AT	4	104552053	104552077	12.5	.	.	intergenic	0.937	rs787940527	3
AAAAT.ATTTT	4	104566884	104566899	3.2	.	.	intergenic	0.604	rs787923303	2
AC.GT	4	104726401	104726417	8.5	ENSSSCT00000007189	GATAD2B	intron	.	rs788246383	2
AG.CT	4	105028813	105028823	5.5	.	.	intergenic	.	rs791150422	2
AAAC.GTTT	4	105204768	105204782	3.75	ENSSSCT00000030953	PGLYRP3	intron	.	999583 rs7903	2
AG.CT	4	105471617	105471627	5.5	.	.	intergenic	.	rs704917131	2
AG.CT	4	105675766	105675777	6	.	.	intergenic	.	rs787287577	2
AAAAC.GTTTT	4	105681763	105681787	5	.	.	intergenic	-0.009	rs786222043	2
AG.CT	4	105705992	105706019	14	.	.	intergenic	0.12	381308 rs7875	2
AT.AT	4	106066459	106066468	5	.	.	intergenic	.	.	4
AG.CT	4	106091412	106091423	6	.	.	intergenic	0.02	rs789665474	2
AG.CT	4	106122980	106122994	7.5	.	.	intergenic	0.094	900876 rs7864	2
AAAC.GTTT	4	106267788	106267802	3.75	.	.	intergenic	0.029	331452 rs7920	2
AAAC.GTTT	4	106586230	106586247	4.5	.	.	intergenic	0.281	rs789902976	2
AT.AT	4	106664459	106664468	5	ENSSSCT00000007253	SNX27	promoter	0.683	.	3
AG.CT	4	106674642	106674652	5.5	ENSSSCT00000007253	SNX27	intron	0.178	rs788490784	3
AAAT.ATTT	4	106703909	106703933	6.25	ENSSSCT00000007253	SNX27	intron	.	039997 rs7907	2
AAAT.ATTT	4	106703909	106703933	6.25	ENSSSCT00000023541	SNORD112	promoter	.	039997 rs7907	2
AAC.GTT	4	107322105	107322129	8.33333	ENSSSCT00000007266	VPS72	intron	0.148	rs789598391	3
AAAG.CTTT	4	107429908	107429928	5.25	ENSSSCT00000007274	CDC42SE1	intron	0.221	rs786233235	2
AAAG.CTTT	4	107667696	107667710	3.75	ENSSSCT00000007284	CTSS	intron	-0.061	rs793065801	2
AAAC.GTTT	4	107699311	107699322	3	ENSSSCT00000007285	HORMAD1	intron	0.389	rs792621973	2
AT.AT	4	109034393	109034408	8	ENSSSCT00000023324	LIX1L	intron	0.093	081791 rs7935	3
AAT.ATT	4	109036290	109036308	6.33333	ENSSSCT00000023324	LIX1L	intron	0.458	306521 rs7911	2
AAAC.GTTT	4	109038160	109038174	3.75	ENSSSCT00000023324	LIX1L	intron	0.429	rs698164247	2
AATT.AATT	4	109040867	109040878	3	ENSSSCT00000023324	LIX1L	intron	0.258	rs790237818	2

AT.AT	4	109604035	109604045	5.5	.	.	intergenic	0.214	.	3
AAAT.ATTT	4	109720078	109720094	4.25	.	.	intergenic	-0.012	rs792604029	2
AG.CT	4	109785395	109785405	5.5	.	.	intergenic	-0.123	.	2
AG.CT	4	110018443	110018453	5.5	.	.	intergenic	0.121	rs791556347	2
ACAT.ATGT	4	110123859	110123877	4.75	ENSSSCT00000007340	CHD1L	intron	-0.025	.	4
AAAC.GTTT	4	110196509	110196529	5.25	ENSSSCT00000007341	FMO5	intron	0.137	352898 rs7869	2
AG.CT	4	110556588	110556598	5.5	ENSSSCT00000007347	PDE4DIP	intron	.	.	3
AC.GT	4	110738323	110738333	5.5	.	.	intergenic	.	.	4
AAAAC.GTTTT	4	110747231	110747249	3.8	.	.	intergenic	0.244	rs787094981	2
AAAG.CTTT	4	111554182	111554206	6.25	.	.	intergenic	-0.544	rs791364294	2
AT.AT	4	111801723	111801736	7	ENSSSCT00000007362	WARS	intron	0.104	.	3
AC.GT	4	111816928	111816940	6.5	ENSSSCT00000007362	WARS	intron	0.05	727858 rs7886	2
AC.GT	4	111936770	111936783	7	.	.	intergenic	.	rs790350667	2
AT.AT	4	112054351	112054361	5.5	.	.	intergenic	0.081	.	3
AAAAC.GTTTT	4	112056862	112056878	3.4	.	.	intergenic	-0.016	rs788354726	2
AG.CT	4	112289429	112289456	14	.	.	intergenic	.	rs791956016	3
AC.GT	4	112383988	112384003	8	.	.	intergenic	0.347	432920 rs7933	5
AC.GT	4	112401210	112401222	6.5	.	.	intergenic	.	rs793429710	2
AG.CT	4	112586713	112586723	5.5	.	.	intergenic	0.151	rs787132408	3
AC.GT	4	112710974	112710985	6	ENSSSCT00000007368	.	intron	0.252	793732 rs7893	2
AC.GT	4	112710974	112710985	6	ENSSSCT00000007367	.	intron	0.252	793732 rs7893	2
AC.GT	4	112981985	112982018	17	.	.	intergenic	0.166	.	2
AC.GT	4	113236104	113236116	6.5	ENSSSCT00000007372	MAN1A2	intron	.	598913 rs7889	3
AAG.CTT	4	113328567	113328581	5	ENSSSCT00000007372	MAN1A2	intron	.	rs790733726	2
AAC.GTT	4	113376136	113376148	4.33333	.	.	intergenic	-0.091	.	2
AG.CT	4	113687693	113687708	8	ENSSSCT00000035345	CD101	intron	0.064	rs793366918	2
AG.CT	4	113687693	113687708	8	ENSSSCT00000033361	CD101	intron	0.064	rs793366918	2
AG.CT	4	113687693	113687708	8	ENSSSCT00000033366	CD101	intron	0.064	rs793366918	2
AG.CT	4	113687693	113687708	8	ENSSSCT00000007377	CD101	intron	0.064	rs793366918	2
AC.GT	4	113693351	113693361	5.5	ENSSSCT00000035345	CD101	intron	-0.19	157095 rs7897	4
AC.GT	4	113693351	113693361	5.5	ENSSSCT00000033361	CD101	intron	-0.19	157095 rs7897	4
AC.GT	4	113693351	113693361	5.5	ENSSSCT00000033366	CD101	intron	-0.19	157095 rs7897	4
AAAT.ATTT	4	113695368	113695391	6	ENSSSCT00000035345	CD101	intron	0.134	290138 rs7864	2
AAAT.ATTT	4	113695368	113695391	6	ENSSSCT00000033361	CD101	intron	0.134	290138 rs7864	2
AAAT.ATTT	4	113695368	113695391	6	ENSSSCT00000033366	CD101	intron	0.134	290138 rs7864	2
AAG.CTT	4	114106232	114106254	7.66667	ENSSSCT00000007380	IGSF3	intron	0.096	rs793392023	2
AC.GT	4	114290959	114290976	9	.	.	intergenic	.	902703 rs7894	3
AT.AT	4	114534681	114534692	6	.	.	intergenic	.	928760 rs7874	2
AT.AT	4	114536321	114536333	6.5	.	.	intergenic	-0.043	273282 rs7923	2
AAAC.GTTT	4	114636022	114636043	5.5	.	.	intergenic	0.219	rs791808624	2
AC.GT	4	114751515	114751535	10.5	.	.	intergenic	0.03	.	2
AGG.CCT	4	114776400	114776413	4.66667	ENSSSCT00000007388	SLC22A15	intron	0.921	rs790261029	2
AC.GT	4	114799616	114799636	10.5	ENSSSCT00000007388	SLC22A15	intron	-0.025	rs792104710	2
ACAT.ATGT	4	114807118	114807135	4.5	ENSSSCT00000007388	SLC22A15	intron	.	rs786717998	2
AT.AT	4	114987885	114987896	6	.	.	intergenic	0.065	rs789969639	3
AC.GT	4	114992113	114992134	11	.	.	intergenic	-0.103	365818 rs7869	2
.GCATG.CATGC	4	114993789	114993811	3.83333	.	.	intergenic	-0.034	rs791516972	2
AC.GT	4	115451806	115451833	14	.	.	intergenic	0.088	624855 rs7884	3
AC.GT	4	115693291	115693306	8	ENSSSCT00000007394	TSHB	promoter	0.229	rs786823137	2
AG.CT	4	115742501	115742515	7.5	ENSSSCT00000007395	SYCP1	intron	.	rs787802898	2
AAAT.ATTT	4	115791225	115791249	6.25	ENSSSCT00000007395	SYCP1	intron	1.372	977704 rs7918	2
AAAT.ATTT	4	116046098	116046124	6.75	.	.	intergenic	0.9	.	2
AC.GT	4	116233201	116233217	8.5	.	.	intergenic	-0.06	.	2
AC.GT	4	116449759	116449768	5	.	.	intergenic	-0.18	.	2
AG.CT	4	116486093	116486105	6.5	.	.	intergenic	0.151	349317 rs7869	3
AGAT.ATCT	4	116504917	116504944	7	.	.	intergenic	.	rs792850401	2
AAC.GTT	4	116535374	116535405	10.6667	.	.	intergenic	-0.229	rs792370991	2
AAGG.CCTT	4	116544878	116544895	4.5	.	.	intergenic	0.463	rs791940826	2
AGAT.ATCT	4	116794646	116794670	6.25	ENSSSCT00000007406	HIPK1	intron	1.015	260982 rs7893	4
AGAT.ATCT	4	116794646	116794670	6.25	ENSSSCT00000007405	HIPK1	intron	1.015	260982 rs7893	4
AAAC.GTTT	4	116881116	116881139	6	ENSSSCT00000025586	BCL2L15	intron	0.258	rs789573201	2
AAAC.GTTT	4	116881116	116881139	6	ENSSSCT00000007409	BCL2L15	intron	0.258	rs789573201	2
AG.CT	4	116980046	116980061	8	ENSSSCT00000007412	PHTF1	intron	.	rs788860196	2
AAAAT.ATTTT	4	116982463	116982481	3.8	ENSSSCT00000007412	PHTF1	intron	0.29	rs791356597	2
AC.GT	4	117020844	117020855	6	ENSSSCT00000007412	PHTF1	intron	0.046	.	3
AT.AT	4	117122962	117122972	5.5	ENSSSCT00000007413	MAGI3	intron	0.167	rs791237278	2
AAT.ATT	4	117130759	117130778	6.66667	ENSSSCT00000007413	MAGI3	intron	0.218	rs790320346	2
AT.AT	4	117352251	117352261	5.5	.	.	intergenic	0.095	rs793354030	2
AAAG.CTTT	4	117357889	117357903	3.75	.	.	intergenic	-0.166	rs786859939	2
AAAC.GTTT	4	117370425	117370451	6.75	.	.	intergenic	0.327	325417 rs7938	2
AAAC.GTTT	4	117590865	117590884	5	.	.	intergenic	0.035	rs792837597	2
AC.GT	4	117597719	117597729	5.5	.	.	intergenic	0.809	rs786238574	2
AG.CT	4	118022451	118022467	8.5	.	.	intergenic	0.663	rs791581826	4
AG.CT	4	118425469	118425500	16	.	.	intergenic	0.145	rs791742631	3
AG.CT	4	118552877	118552890	7	.	.	intergenic	0.226	483575 rs7885	2
AG.CT	4	118662543	118662553	5.5	.	.	intergenic	0.01	rs789573532	2
AATT.AATT	4	119065858	119065873	4	ENSSSCT00000027506	C1orf162	promoter	.	155500 rs7871	2
AAAC.GTTT	4	119418293	119418309	4.25	ENSSSCT00000023214	CEPT1	intron	0.053	395404 rs7913	2
AAAC.GTTT	4	119418293	119418309	4.25	ENSSSCT00000030532	CHIA	intron	0.053	395404 rs7913	2
AT.AT	4	119427784	119427794	5.5	ENSSSCT00000023214	CEPT1	intron	0.056	rs786413265	2
AC.GT	4	119644119	119644132	7	.	.	intergenic	0.203	rs787689226	2
AAAAG.CTTTT	4	119660611	119660629	3.8	.	.	intergenic	0.145	rs790924087	2
AC.GT	4	119735311	119735339	14.5	.	.	intergenic	0.12	.	2
AAAAAT.ATTTTT	4	120198453	120198475	3.83333	.	.	intergenic	0.18	rs790475222	2

AAAC.GTTT	4	120245327	120245345	4.75	.	.	intergenic	.	.	3
ACC.GGT	4	120302917	120302933	5.66667	ENSSSCT00000007459	.	intron	-0.074	rs788288646	2
AAGAG.CTCTT	4	120536659	120536702	8.8	ENSSSCT00000007463	.	intron	-0.128	ɫ18612 rs7877	2
AC.GT	4	120690195	120690209	7.5	.	.	intergenic	.	rs786726796	7
AAAG.CTTT	4	121371120	121371148	7.25	.	.	intergenic	0.103	rs790639693	2
AT.AT	4	121413228	121413237	5	ENSSSCT00000007487	KIAA1324	intron	0.144	rs790815005	2
AC.GT	4	121600850	121600869	10	ENSSSCT00000023502	.	intron	.	.	2
AT.AT	4	121703953	121703964	6	ENSSSCT00000025132	AKNAD1	intron	.	.	4
AT.AT	4	121703953	121703964	6	ENSSSCT00000007493	AKNAD1	intron	.	.	4
AGG.CCT	4	121828012	121828030	6.33333	ENSSSCT00000007495	FNDC7	promoter	.	rs790816646	2
AGG.CCT	4	121828012	121828030	6.33333	ENSSSCT00000007496	FNDC7	promoter	.	rs790816646	2
AT.AT	4	122394762	122394773	6	.	.	intergenic	0.158	rs701312758	2
ATC.GAT	4	122398413	122398426	4.66667	.	.	intergenic	0.17	rs793572984	2
AC.GT	4	122772893	122772909	8.5	ENSSSCT00000007504	.	intron	0.55	.	2
AAT.ATT	4	123129877	123129891	5	ENSSSCT00000035715	NTNG1	intron	0.087	rs791341256	4
AAT.ATT	4	123129877	123129891	5	ENSSSCT00000034022	NTNG1	intron	0.087	rs791341256	4
AAT.ATT	4	123129877	123129891	5	ENSSSCT00000033143	NTNG1	intron	0.087	rs791341256	4
AAT.ATT	4	123129877	123129891	5	ENSSSCT00000022367	NTNG1	intron	0.087	rs791341256	4
AAT.ATT	4	123129877	123129891	5	ENSSSCT00000007505	NTNG1	intron	0.087	rs791341256	4
AAT.ATT	4	123129877	123129891	5	ENSSSCT00000033782	NTNG1	intron	0.087	rs791341256	4
AAT.ATT	4	123129877	123129891	5	ENSSSCT00000035789	NTNG1	intron	0.087	rs791341256	4
ATC.GAT	4	123139761	123139776	5.33333	ENSSSCT00000035789	NTNG1	intron	0.088	ɔ37629 rs7901	2
ATC.GAT	4	123139761	123139776	5.33333	ENSSSCT00000035715	NTNG1	intron	0.088	ɔ37629 rs7901	2
ATC.GAT	4	123139761	123139776	5.33333	ENSSSCT00000033143	NTNG1	intron	0.088	ɔ37629 rs7901	2
ATC.GAT	4	123139761	123139776	5.33333	ENSSSCT00000022367	NTNG1	intron	0.088	ɔ37629 rs7901	2
ATC.GAT	4	123139761	123139776	5.33333	ENSSSCT00000007505	NTNG1	intron	0.088	ɔ37629 rs7901	2
ATC.GAT	4	123139761	123139776	5.33333	ENSSSCT00000033782	NTNG1	intron	0.088	ɔ37629 rs7901	2
ATC.GAT	4	123139761	123139776	5.33333	ENSSSCT00000034022	NTNG1	intron	0.088	ɔ37629 rs7901	2
AC.GT	4	123248841	123248862	11	.	.	intergenic	-0.452	rs696498154	2
AC.GT	4	123285234	123285259	13	.	.	intergenic	0.144	rs791295508	3
AAAT.ATTT	4	123300078	123300096	4.75	.	.	intergenic	0	'78423 rs7876	2
AAAT.ATTT	4	123436784	123436802	4.75	.	.	intergenic	.	rs793588911	2
AG.CT	4	123860254	123860265	6	.	.	intergenic	-0.086	?24749 rs7879	2
AG.CT	4	123922438	123922450	6.5	.	.	intergenic	0.19	.	2
AAT.ATT	4	123931195	123931206	4	.	.	intergenic	0.128	rs787949499	2
AG.CT	4	123982463	123982474	6	.	.	intergenic	-0.204	325621 rs7863	2
AAAAC.GTTTT	4	124386483	124386505	4.6	.	.	intergenic	-0.229	371729 rs7866	2
AC.GT	4	124393654	124393665	6	.	.	intergenic	.	)59380 rs7864	2
ATC.GAT	4	124403401	124403415	5	.	.	intergenic	-0.124	506799 rs7894	2
AT.AT	4	124445590	124445599	5	.	.	intergenic	.	ɫ64434 rs7916	4
AT.AT	4	124453163	124453172	5	.	.	intergenic	.	.	2
ACAT.ATGT	4	124480905	124480933	7.25	.	.	intergenic	0.039	'35249 rs7904	2
AT.AT	4	124608726	124608739	7	.	.	intergenic	.	rs786339736	3
AG.CT	4	124729209	124729219	5.5	.	.	intergenic	-0.176	ɫ51535 rs7080	4
AAAAC.GTTTT	4	125014090	125014108	3.8	.	.	intergenic	0.073	'61045 rs7930	2
AT.AT	4	125124854	125124867	7	.	.	intergenic	.	ɫ94543 rs7892	2
AT.AT	4	125128680	125128689	5	.	.	intergenic	0.098	)90707 rs7912	4
AAC.GTT	4	125682621	125682634	4.66667	.	.	intergenic	-0.02	ɫ69505 rs7916	2
AAAAC.GTTTT	4	125742661	125742677	3.4	.	.	intergenic	0.099	?02608 rs7883	2
AAAG.CTTT	4	125845586	125845608	5.75	.	.	intergenic	.	rs790346002	2
AC.GT	4	125875672	125875689	9	.	.	intergenic	-0.145	513708 rs7936	2
AAG.CTT	4	125887150	125887161	4	.	.	intergenic	.	rs787827179	2
ACAG.CTGT	4	125892356	125892370	3.75	.	.	intergenic	.	.	2
AAAAG.CTTTT	4	125960206	125960229	4.8	.	.	intergenic	.	ɔ37565 rs7876	2
AAAG.CTTT	4	126109574	126109585	3	.	.	intergenic	.	354669 rs7906	2
AAAAG.CTTTT	4	126149369	126149385	3.4	.	.	intergenic	-0.142	.	2
AAAC.GTTT	4	126765524	126765547	6	.	.	intergenic	0.067	ɔ98541 rs7875	2
AAAT.ATTT	4	126919186	126919208	5.75	.	.	intergenic	0.046	340399 rs7932	2
AAC.GTT	4	127064948	127064973	8.66667	.	.	intergenic	0.375	385035 rs7909	3
AC.GT	4	127074650	127074676	13.5	.	.	intergenic	.	rs791419708	4
AAAC.GTTT	4	127301975	127301993	4.75	ENSSSCT00000007510	COL11A1	intron	0.474	)78262 rs7899	2
AAAGC.GCTTT	4	127512443	127512464	4.4	.	.	intergenic	-0.106	rs787128110	2
AT.AT	4	127521827	127521839	6.5	.	.	intergenic	-0.012	rs789407194	4
AG.CT	4	127574506	127574515	5	.	.	intergenic	-0.146	)95846 rs7871	2
AT.AT	4	127593303	127593325	11.5	.	.	intergenic	0.046	.	2
AAAG.CTTT	4	127763731	127763748	4.5	.	.	intergenic	.	rs788081734	2
AATG.CATT	4	127903248	127903263	4	.	.	intergenic	0.023	rs791125815	2
AAAAG.CTTTT	4	128371270	128371291	4.4	.	.	intergenic	.	.	2
AC.GT	4	128570590	128570603	7	.	.	intergenic	.	rs787314544	2
AAAAC.GTTTT	4	128667009	128667031	4.6	.	.	intergenic	-0.098	ɔ50777 rs7884	2
AT.AT	4	128764331	128764354	12	.	.	intergenic	.	rs793469330	2
AG.CT	4	128923517	128923526	5	ENSSSCT00000007513	SLC30A7	intron	-0.456	.	2
AT.AT	4	128998403	128998413	5.5	.	.	intergenic	.	rs787561075	2
AC.GT	4	129044693	129044703	5.5	ENSSSCT00000007515	VCAM1	intron	0.804	rs790763321	3
AC.GT	4	129044693	129044703	5.5	ENSSSCT00000035678	VCAM1	intron	0.804	rs790763321	3
AG.CT	4	129168525	129168534	5	.	.	intergenic	-0.049	.	3
AACC.GGTT	4	129312520	129312537	4.5	ENSSSCT00000007517	CDC14A	intron	0.054	rs791951324	2
AC.GT	4	129410841	129410851	5.5	ENSSSCT00000007517	CDC14A	intron	0.12	rs792765693	2
AT.AT	4	129444234	129444243	5	ENSSSCT00000007517	CDC14A	intron	.	rs786387942	2
AAAAT.ATTTT	4	129604115	129604137	4.6	ENSSSCT00000007520	.	intron	0.042	379447 rs7869	2
AAAAAT.ATTTTT	4	129722191	129722226	6	.	.	intergenic	.	rs793644086	2
AAAAC.GTTTT	4	129725395	129725419	5	ENSSSCT00000007524	SLC35A3	intron	4.582	rs793091476	2
AC.GT	4	129738796	129738806	5.5	ENSSSCT00000007524	SLC35A3	intron	0.143	ɔ93654 rs7936	3
AC.GT	4	129983528	129983537	5	ENSSSCT00000007528	PALMD	intron	0.116	.	2

AT.AT	4	130027608	130027619	6	.	.	intergenic	0.077	.	2
AAAC.GTTT	4	130168903	130168915	3.25	.	.	intergenic	0.185	317376 rs7900	2
AG.CT	4	130183274	130183286	6.5	.	.	intergenic	0.075	rs790163231	3
AAAATT.AATTTT	4	130188619	130188640	3.66667	.	.	intergenic	.	rs790583651	3
AAC.GTT	4	130219709	130219725	5.66667	.	.	intergenic	.	906207 rs7934	4
AC.GT	4	130385433	130385446	7	.	.	intergenic	-0.026	.	4
AT.AT	4	130494681	130494693	6.5	.	.	intergenic	0.075	rs788663421	2
AC.GT	4	130535910	130535922	6.5	.	.	intergenic	0.112	rs790103133	2
AC.GT	4	130754502	130754523	11	.	.	intergenic	-0.028	rs793420662	2
AAT.ATT	4	130760027	130760044	6	.	.	intergenic	.	.	2
AG.CT	4	130835919	130835928	5	ENSSSCT00000007531	.	intron	0.446	rs791168680	2
AT.AT	4	130915187	130915197	5.5	.	.	intergenic	.	902868 rs7879	2
ACAT.ATGT	4	130933174	130933200	6.75	.	.	intergenic	0.036	rs787920757	2
AAAT.ATTT	4	131162248	131162279	8	.	.	intergenic	.	rs793094458	2
AC.GT	4	131522045	131522054	5	.	.	intergenic	0.068	rs793044957	4
AC.GT	4	132012555	132012568	7	ENSSSCT00000007532	DPYD	intron	0.146	rs788560613	2
AAT.ATT	4	132038462	132038481	6.66667	ENSSSCT00000007532	DPYD	intron	-0.067	267249 rs7887	2
AAAC.GTTT	4	132086104	132086122	4.75	ENSSSCT00000007532	DPYD	intron	-0.039	rs793527285	3
AAAG.CTTT	4	132122520	132122533	3.5	ENSSSCT00000007532	DPYD	intron	0.079	.	2
AC.GT	4	132170872	132170897	13	ENSSSCT00000007532	DPYD	intron	-0.036	rs696178186	2
AAAG.CTTT	4	132177930	132177953	6	ENSSSCT00000007532	DPYD	intron	-0.171	rs790768312	2
AT.AT	4	132182652	132182662	5.5	ENSSSCT00000007532	DPYD	intron	3.53	.	3
AAAT.ATTT	4	132269953	132269968	4	ENSSSCT00000007532	DPYD	intron	0.029	523991 rs7900	2
AAAAC.GTTTT	4	132278668	132278696	5.8	ENSSSCT00000007532	DPYD	intron	-0.269	rs791700588	2
AC.GT	4	132434332	132434350	9.5	ENSSSCT00000007532	DPYD	intron	0.101	rs792819384	2
AAAAT.ATTTT	4	132466428	132466456	5.8	ENSSSCT00000007532	DPYD	intron	0.432	rs707676537	2
AG.CT	4	132644460	132644470	5.5	.	.	intergenic	0.348	rs793856650	2
AAAT.ATTT	4	132907639	132907658	5	.	.	intergenic	-0.147	rs792788420	2
AC.GT	4	132962088	132962118	15.5	.	.	intergenic	0.039	rs790861084	3
AAT.ATT	4	132993380	132993403	8	.	.	intergenic	0.131	175644 rs7864	3
AT.AT	4	133051125	133051136	6	.	.	intergenic	0.011	rs791507688	2
AAT.ATT	4	133271245	133271257	4.33333	.	.	intergenic	0.1	315226 rs7909	3
AAC.GTT	4	133380522	133380540	6.33333	.	.	intergenic	-0.051	rs788645404	2
AGGGC.GCCCT	4	133608154	133608174	4.2	.	.	intergenic	0.05	.	2
AC.GT	4	133845545	133845555	5.5	.	.	intergenic	0.136	rs786477494	2
AC.GT	4	133867953	133867976	12	.	.	intergenic	-0.086	rs708535863	2
AAC.GTT	4	133930145	133930159	5	.	.	intergenic	.	rs788471019	2
AATG.CATT	4	134164544	134164559	4	ENSSSCT00000007539	ALG14	intron	0.099	.	3
AG.CT	4	134249868	134249878	5.5	ENSSSCT00000031017	.	intron	1.769	rs786622356	3
AG.CT	4	134249868	134249878	5.5	ENSSSCT00000007541	CNN3	intron	1.769	rs786622356	3
AAAAT.ATTTT	4	134311514	134311528	3	.	.	intergenic	-0.058	rs789238456	2
AAC.GTT	4	134333979	134333994	5.33333	.	.	intergenic	0.052	rs792580625	2
AG.CT	4	134349247	134349256	5	.	.	intergenic	0.056	.	3
AAAC.GTTT	4	134374546	134374559	3.5	ENSSSCT00000007542	.	intron	.	rs789128113	2
AC.GT	4	134402197	134402210	7	ENSSSCT00000028417	.	intron	.	rs786562571	2
AAAAT.ATTTT	4	134593550	134593582	6.6	.	.	intergenic	0.258	rs789551788	2
AAAT.ATTT	4	134625335	134625353	4.75	.	.	intergenic	0.03	354776 rs7901	2
AAAG.CTTT	4	134762571	134762587	4.25	ENSSSCT00000007543	.	intron	1.165	037502 rs7864	2
AC.GT	4	134809903	134809915	6.5	.	.	intergenic	0.153	rs793349084	2
AT.AT	4	134810964	134810974	5.5	.	.	intergenic	0.338	rs789909014	4
AAAC.GTTT	4	134869375	134869395	5.25	ENSSSCT00000007545	ABCA4	intron	0.117	rs788916555	2
AAAAC.GTTTT	4	134965684	134965702	3.8	ENSSSCT00000007545	ABCA4	intron	0.218	rs788276046	2
AAAC.GTTT	4	135022535	135022554	5	ENSSSCT00000007546	GCLM	intron	0.253	rs792689974	2
AAT.ATT	4	135023629	135023642	4.66667	ENSSSCT00000007546	GCLM	intron	0.241	rs793129780	2
AAAAG.CTTTT	4	135579947	135579962	3.2	ENSSSCT00000007551	TMED5	intron	.	rs790765239	2
ACAG.CTGT	4	135587270	135587289	5	.	.	intergenic	.	.	2
AAAC.GTTT	4	135601200	135601214	3.75	ENSSSCT00000007552	.	intron	.	rs792335977	2
AAAT.ATTT	4	135718940	135718965	6.5	ENSSSCT00000007553	MTF2	intron	0.167	rs789674928	2
AAAT.ATTT	4	135718940	135718965	6.5	ENSSSCT00000034081	MTF2	intron	0.167	rs789674928	2
AAT.ATT	4	135759024	135759051	9.33333	.	.	intergenic	0.38	228315 rs7883	2
AT.AT	4	135805841	135805851	5.5	.	.	intergenic	0.614	rs787903374	2
AG.CT	4	136679253	136679265	6.5	ENSSSCT00000033701	TGFBR3	intron	0.251	rs792716239	2
AG.CT	4	136754594	136754606	6.5	ENSSSCT00000033701	TGFBR3	intron	1.392	rs792273460	2
AAAAC.GTTTT	4	136885873	136885892	4	ENSSSCT00000007569	TGFBR3	intron	0.252	rs790274523	2
AAAAC.GTTTT	4	136885873	136885892	4	ENSSSCT00000035989	TGFBR3	intron	0.252	rs790274523	2
AAAAC.GTTTT	4	136885873	136885892	4	ENSSSCT00000036583	TGFBR3	intron	0.252	rs790274523	2
AAAAC.GTTTT	4	136885873	136885892	4	ENSSSCT00000033701	TGFBR3	intron	0.252	rs790274523	2
AAAAC.GTTTT	4	136885873	136885892	4	ENSSSCT00000034411	TGFBR3	intron	0.252	rs790274523	2
AGG.CCT	4	137168365	137168382	6	ENSSSCT00000007570	HFM1	intron	0.126	494219 rs7887	2
AAAC.GTTT	4	137234497	137234523	6.75	.	.	intergenic	0.188	.	2
AAAC.GTTT	4	137273023	137273034	3	.	.	intergenic	0.118	749940 rs7895	2
AT.AT	4	137574337	137574348	6	.	.	intergenic	.	rs789660025	4
AC.GT	4	137691536	137691546	5.5	.	.	intergenic	0.373	.	2
AG.CT	4	137792343	137792375	16.5	.	.	intergenic	0.14	rs789982832	2
AAT.ATT	4	138158472	138158493	7.33333	.	.	intergenic	0.263	rs786704468	2
AAAAC.GTTTT	4	138162885	138162904	4	.	.	intergenic	.	rs786407454	2
AG.CT	4	138315919	138315940	11	.	.	intergenic	.	.	4
AT.AT	4	138418005	138418017	6.5	.	.	intergenic	.	305310 rs7888	3
AC.GT	4	138435749	138435759	5.5	.	.	intergenic	0.314	.	2
AG.CT	4	138647678	138647690	6.5	.	.	intergenic	.	rs788274045	2
AAAC.GTTT	4	139096049	139096072	6	.	.	intergenic	0.028	rs789996241	2
AC.GT	4	139272891	139272901	5.5	.	.	intergenic	-0.21	rs793560151	3
AT.AT	4	139307839	139307849	5.5	.	.	intergenic	.	007328 rs7926	2
AAAT.ATTT	4	139484153	139484171	4.75	.	.	intergenic	.	rs787103557	2

AAAC.GTTT	4	139610163	139610194	8	.	.	intergenic	.	189979 rs7933	4
AAAAC.GTTTT	4	139738145	139738163	3.8	.	.	intergenic	0.777	rs786876175	2
AAG.CTT	4	139793449	139793465	5.66667	.	.	intergenic	.	551456 rs7885	2
AAATT.AATTT	4	140193692	140193714	4.6	.	.	intergenic	.	rs793015156	2
AG.CT	4	141203828	141203837	5	.	.	intergenic	0.046	589896 rs7893	3
AAAAC.GTTTT	4	141298092	141298109	3.6	.	.	intergenic	0.252	297848 rs7874	2
AC.GT	4	141347773	141347790	9	ENSSSCT00000007590	LMO4	intron	3.653	rs786545600	2
AG.CT	4	141406932	141406944	6.5	.	.	intergenic	1.665	rs789009050	2
AAC.GTT	4	141470107	141470123	5.66667	.	.	intergenic	-0.113	366755 rs7917	2
AAAG.CTTT	4	141728671	141728701	7.75	ENSSSCT00000028257	.	intron	0.061	rs786851127	2
AAAC.GTTT	4	141957226	141957244	4.75	.	.	intergenic	0.19	rs793452112	2
AC.GT	4	142145143	142145161	9.5	ENSSSCT00000007595	CLCA1	intron	0.599	430116 rs7885	2
AG.CT	4	142180379	142180389	5.5	.	.	intergenic	0.173	.	2
AAAG.CTTT	4	142418861	142418873	3.25	.	.	intergenic	0.411	.	2
AG.CT	4	142448831	142448846	8	.	.	intergenic	0.026	rs788579170	2
AGG.CCT	4	142643470	142643481	4	.	.	intergenic	0.152	.	2
AT.AT	4	142748854	142748866	6.5	ENSSSCT00000007603	ZNHIT6	intron	1.534	rs709165712	3
AAAAAC.GTTTTT	4	142759611	142759629	3.16667	ENSSSCT00000007603	ZNHIT6	intron	.	295164 rs7914	2
AG.CT	5	1529171	1529182	6	.	.	intergenic	0.062	.	2
AC.GT	5	1852971	1852984	7	.	.	intergenic	-0.039	rs792433403	2
AAAC.GTTT	5	2014771	2014790	5	.	.	intergenic	-0.178	rs792128714	2
AAG.CTT	5	2556673	2556695	7.66667	.	.	intergenic	0.033	465032 rs7883	4
AC.GT	5	2596719	2596731	6.5	ENSSSCT00000000029	EFCAB6	intron	-0.078	387812 rs7884	2
AG.CT	5	2937784	2937794	5.5	.	.	intergenic	-0.119	rs786540436	2
ATC.GAT	5	4161504	4161518	5	.	.	intergenic	-0.03	904185 rs7922	2
AAT.ATT	5	4311716	4311732	5.66667	ENSSSCT00000000066	DESI1	intron	0.184	593571 rs7925	2
AAAC.GTTT	5	4317773	4317791	4.75	ENSSSCT00000000066	DESI1	intron	0.076	rs791267180	2
AAC.GTT	5	4615805	4615824	6.66667	ENSSSCT00000026332	EP300	intron	.	255902 rs7872	2
AAC.GTT	5	4615805	4615824	6.66667	ENSSSCT00000000074	EP300	intron	.	255902 rs7872	2
AAAT.ATTT	5	4683576	4683588	3.25	.	.	intergenic	.	rs792758549	2
AAAC.GTTT	5	4817125	4817143	4.75	ENSSSCT00000000078	SLC25A17	intron	0.175	rs791539431	4
AGG.CCT	5	4851668	4851680	4.33333	.	.	intergenic	0.289	rs791068223	2
AAAC.GTTT	5	5096381	5096406	6.5	.	.	intergenic	.	rs791327969	2
AT.AT	5	5105386	5105397	6	.	.	intergenic	0.673	rs790221783	2
AATG.CATT	5	5153012	5153030	4.75	.	.	intergenic	0.087	rs787709638	2
AAAT.ATTT	5	5229962	5229978	4.25	ENSSSCT00000000082	SGSM3	intron	.	955580 rs7890	2
AAAT.ATTT	5	5229962	5229978	4.25	ENSSSCT00000031184	SGSM3	intron	.	955580 rs7890	2
AGG.CCT	5	5368637	5368651	5	ENSSSCT00000000084	.	intron	0.101	rs695920451	2
AGC.GCT	5	5374983	5375000	6	ENSSSCT00000000084	.	cds	0.281	319057 rs7865	3
AAAC.GTTT	5	5376116	5376142	6.75	ENSSSCT00000000084	.	intron	-0.09	rs792472842	2
ACCCC.GGGGT	5	5380221	5380237	3.4	ENSSSCT00000000084	.	intron	0.21	298535 rs7906	2
AAAT.ATTT	5	5544371	5544387	4.25	.	.	intergenic	.	rs790685073	2
AC.GT	5	5557370	5557381	6	.	.	intergenic	0.193	rs790975584	2
AAAAAC.GTTTTT	5	5557816	5557838	3.83333	.	.	intergenic	0.169	rs789279659	2
AC.GT	5	6020251	6020261	5.5	.	.	intergenic	0.164	rs790179919	2
AG.CT	5	6291504	6291517	7	.	.	intergenic	.	556789 rs7931	2
AAAC.GTTT	5	6310628	6310644	4.25	.	.	intergenic	.	rs790742556	4
AC.GT	5	6504134	6504164	15.5	.	.	intergenic	-0.136	.	2
AAAC.GTTT	5	6678567	6678579	3.25	.	.	intergenic	.	rs788590946	2
AAAT.ATTT	5	6700767	6700778	3	.	.	intergenic	0.144	rs787561508	2
AATC.GATT	5	7017545	7017576	8	ENSSSCT00000022302	PLA2G6	intron	.	rs786564139	3
AATC.GATT	5	7017545	7017576	8	ENSSSCT00000000116	PLA2G6	intron	.	rs786564139	3
AAAC.GTTT	5	7791700	7791718	4.75	.	.	intergenic	.	.	2
AAC.GTT	5	7836333	7836352	6.66667	.	.	intergenic	.	rs786693844	2
AAGG.CCTT	5	8393382	8393395	3.5	.	.	intergenic	-0.091	959885 rs7864	2
AC.GT	5	8505212	8505221	5	.	.	intergenic	-0.285	.	2
AATG.CATT	5	8536292	8536318	6.75	.	.	intergenic	-0.038	908912 rs7915	2
AGAT.ATCT	5	8800627	8800638	3	.	.	intergenic	0.135	rs787425775	2
AAAT.ATTT	5	9056157	9056183	6.75	.	.	intergenic	.	087194 rs7882	2
AAAC.GTTT	5	9354800	9354817	4.5	.	.	intergenic	-0.158	rs789645502	2
AGAT.ATCT	5	9407324	9407338	3.75	ENSSSCT00000024278	.	intron	0.199	rs786553023	2
AAAAC.GTTTT	5	10591248	10591278	6.2	.	.	intergenic	0.15	.	2
AAAAAG.CTTTTT	5	10595119	10595137	3.16667	.	.	intergenic	0.072	rs788666177	2
AAAAC.GTTTT	5	10605387	10605405	3.8	.	.	intergenic	0.041	rs788001243	2
AG.CT	5	10681154	10681165	6	.	.	intergenic	-0.115	rs787493435	2
AT.AT	5	10740662	10740673	6	.	.	intergenic	.	594622 rs7932	3
AAAAC.GTTTT	5	11002822	11002841	4	.	.	intergenic	.	rs793622268	2
AAAAC.GTTTT	5	11595663	11595687	5	.	.	intergenic	-0.242	rs790068249	2
AATG.CATT	5	11665964	11665983	5	ENSSSCT00000028759	.	intron	-0.062	.	2
AG.CT	5	12205469	12205480	6	.	.	intergenic	0.08	rs789112376	2
AG.CT	5	12280669	12280681	6.5	ENSSSCT00000000162	SYN3	intron	-0.065	.	2
AC.GT	5	12555925	12555937	6.5	ENSSSCT00000000171	BTBD11	intron	0.045	230295 rs7882	3
AC.GT	5	12688682	12688694	6.5	ENSSSCT00000000171	BTBD11	intron	-0.058	.	3
AAT.ATT	5	12796399	12796415	5.66667	ENSSSCT00000000171	BTBD11	intron	-0.004	rs789757747	2
AC.GT	5	12863319	12863329	5.5	ENSSSCT00000000171	BTBD11	intron	0.139	rs791039369	2
AAT.ATT	5	12882175	12882216	14	ENSSSCT00000000171	BTBD11	intron	.	rs792254425	2
AAAT.ATTT	5	12927310	12927323	3.5	.	.	intergenic	.	.	2
AC.GT	5	12988389	12988399	5.5	.	.	intergenic	-0.045	.	2
AAC.GTT	5	13174933	13174946	4.66667	ENSSSCT00000000173	CRY1	intron	.	348907 rs7908	2
AG.CT	5	13458979	13458991	6.5	ENSSSCT00000000177	RFX4	intron	0.071	rs790941400	2
ACC.GGT	5	13473533	13473546	4.66667	ENSSSCT00000000177	RFX4	intron	0.215	579284 rs7933	2
AT.AT	5	13491901	13491919	9.5	ENSSSCT00000000177	RFX4	intron	.	rs790435213	2
AAC.GTT	5	13567619	13567630	4	.	.	intergenic	0.063	985995 rs7922	2
AAAC.GTTT	5	13578181	13578199	4.75	.	.	intergenic	0.015	rs789481293	2

AG.CT	5	13581747	13581759	6.5	.	.	intergenic	-0.144	rs699432491	2
AG.CT	5	14116797	14116807	5.5	.	.	intergenic	-0.042	.	2
ACAT.ATGT	5	14141808	14141826	4.75	.	.	intergenic	0.245	rs786742196	2
AAAG.CTTT	5	14211324	14211335	3	.	.	intergenic	0.113	.	2
AC.GT	5	14355221	14355233	6.5	.	.	intergenic	-0.152	rs791814139	2
AG.CT	5	14510301	14510312	6	.	.	intergenic	-0.507	rs788765438	2
AG.CT	5	14828609	14828618	5	.	.	intergenic	-0.225	rs792213669	2
AT.AT	5	14836350	14836359	5	.	.	intergenic	.	rs692763202	2
AC.GT	5	15177959	15177968	5	ENSSSCT00000000185	CACNB3	intron	1.164	rs788831760	2
AAAT.ATTT	5	15363580	15363597	4.5	ENSSSCT00000000188	CCDC65	intron	.	rs791667725	2
AATG.CATT	5	15622236	15622274	9.75	.	.	intergenic	.	rs793599376	2
AC.GT	5	15849304	15849319	8	.	.	intergenic	.	324486 rs7888	3
AC.GT	5	15955788	15955806	9.5	ENSSSCT00000000212	SPATS2	intron	.	.	2
AAAC.GTTT	5	16187602	16187627	6.5	.	.	intergenic	.	.	3
AAAC.GTTT	5	16556625	16556649	6.25	ENSSSCT00000022818	.	intron	0.629	rs790451643	2
AG.CT	5	16580799	16580812	7	.	.	intergenic	0.308	913039 rs7924	2
AAC.GTT	5	16786055	16786069	5	.	.	intergenic	-0.078	959644 rs7893	2
AAAAC.GTTTT	5	17128691	17128707	3.4	ENSSSCT00000028346	DIP2B	intron	-0.019	953759 rs7930	2
AAAAC.GTTTT	5	17163086	17163109	4.8	ENSSSCT00000028346	DIP2B	intron	.	.	2
AAAAAG.CTTTTT	5	17541169	17541190	3.66667	.	.	intergenic	0.195	rs792760808	2
AAAC.GTTT	5	17570945	17570967	5.75	ENSSSCT00000000245	SCN8A	intron	0.109	rs791306152	2
AAT.ATT	5	18445460	18445482	7.66667	ENSSSCT00000000269	KRT2	intron	-0.049	219071 rs7871	2
AC.GT	5	18582059	18582069	5.5	.	.	intergenic	-0.093	.	2
AAAC.GTTT	5	18765703	18765729	6.75	.	.	intergenic	0.108	rs787265951	2
AAAAAG.CTTTTT	5	19073340	19073366	4.5	.	.	intergenic	.	579749 rs7866	2
AAG.CTT	5	19180373	19180397	8.33333	ENSSSCT00000000299	.	intron	-0.054	001145 rs7877	3
AAG.CTT	5	19180373	19180397	8.33333	ENSSSCT00000023450	.	intron	-0.054	001145 rs7877	3
AAT.ATT	5	19302109	19302122	4.66667	.	.	intergenic	0.29	rs787057857	2
AAAC.GTTT	5	19579071	19579089	4.75	.	.	intergenic	.	rs792919399	2
AC.GT	5	20459451	20459463	6.5	ENSSSCT00000035821	ITGA5	intron	-0.092	140258 rs7864	3
AC.GT	5	20459451	20459463	6.5	ENSSSCT00000034427	ITGA5	intron	-0.092	140258 rs7864	3
AC.GT	5	20459451	20459463	6.5	ENSSSCT00000000314	ITGA5	intron	-0.092	140258 rs7864	3
AC.GT	5	20459451	20459463	6.5	ENSSSCT00000033141	ITGA5	promoter	-0.092	140258 rs7864	3
AAAC.GTTT	5	20492473	20492505	8.25	.	.	intergenic	.	rs789830913	2
AAAC.GTTT	5	20706177	20706189	3.25	.	.	intergenic	.	.	2
ACAT.ATGT	5	20807336	20807351	4	.	.	intergenic	-0.147	.	4
AAAG.CTTT	5	21291321	21291340	5	.	.	intergenic	.	357841 rs7891	2
AAAAC.GTTTT	5	22040301	22040315	3	.	.	intergenic	.	.	2
AAAC.GTTT	5	22403097	22403119	5.75	.	.	intergenic	.	587560 rs7901	2
AAAAC.GTTTT	5	22740935	22740954	4	.	.	intergenic	0.372	rs793005507	2
AC.GT	5	22811643	22811661	9.5	ENSSSCT00000000393	DGKA	promoter	0.522	.	2
AAAAAC.GTTTTT	5	23428609	23428638	5	ENSSSCT00000000434	RBMS2	intron	.	rs786286424	2
AC.GT	5	23610219	23610239	10.5	ENSSSCT00000032195	BAZ2A	3'utr	1.282	.	2
AC.GT	5	23610219	23610239	10.5	ENSSSCT00000000436	BAZ2A	3'utr	1.282	.	2
AG.CT	5	24138141	24138153	6.5	ENSSSCT00000028141	.	intron	-0.661	.	2
AC.GT	5	24511712	24511726	7.5	ENSSSCT00000027063	.	intron	0.263	.	3
AT.AT	5	24835363	24835373	5.5	ENSSSCT00000000478	GLI1	3'utr	.	rs788073903	2
AT.AT	5	24835363	24835373	5.5	ENSSSCT00000000472	.	3'utr	.	rs788073903	2
AAAAT.ATTTT	5	24919631	24919646	3.2	.	.	intergenic	0.335	.	3
AAAC.GTTT	5	24920565	24920583	4.75	.	.	intergenic	0.269	rs790219891	2
AAAC.GTTT	5	24974090	24974104	3.75	ENSSSCT00000000489	XRCC6BP1	intron	0.067	.	3
AAT.ATT	5	25098125	25098140	5.33333	.	.	intergenic	-0.041	.	2
AG.CT	5	25152606	25152619	7	.	.	intergenic	0.041	776649 rs7885	2
AAAC.GTTT	5	25194540	25194554	3.75	.	.	intergenic	0.064	.	3
AG.CT	5	25206296	25206308	6.5	.	.	intergenic	0.42	rs787223154	2
AAAAC.GTTTT	5	25216496	25216510	3	.	.	intergenic	.	.	2
AAAAT.ATTTT	5	25608249	25608268	4	.	.	intergenic	0.119	610540 rs7891	2
AC.GT	5	25974711	25974734	12	.	.	intergenic	.	369835 rs7091	4
AAT.ATT	5	26576384	26576402	6.33333	.	.	intergenic	0.162	rs791275580	2
AC.GT	5	26584159	26584180	11	.	.	intergenic	0.11	.	2
AG.CT	5	26649914	26649933	10	.	.	intergenic	.	595878 rs7918	2
AAAT.ATTT	5	26879663	26879680	4.5	.	.	intergenic	.	rs787574936	2
AT.AT	5	26973839	26973848	5	.	.	intergenic	.	458904 rs7880	3
AAAG.CTTT	5	27000988	27001007	5	.	.	intergenic	-0.14	rs787790445	2
AT.AT	5	27056008	27056025	9	.	.	intergenic	0.336	.	4
AAAC.GTTT	5	27172276	27172290	3.75	.	.	intergenic	.	rs789879738	2
AT.AT	5	27279805	27279814	5	.	.	intergenic	.	rs791039313	2
AT.AT	5	27292936	27292945	5	.	.	intergenic	.	rs792362689	2
AAAC.GTTT	5	27303885	27303908	6	.	.	intergenic	0.3	573994 rs7937	2
AAAAC.GTTTT	5	27477660	27477681	4.4	.	.	intergenic	.	991081 rs7916	2
AAAT.ATTT	5	27826224	27826238	3.75	.	.	intergenic	-0.051	rs701609808	3
AAAC.GTTT	5	27894546	27894558	3.25	.	.	intergenic	0.499	rs789225067	2
AAG.CTT	5	27924137	27924152	5.33333	.	.	intergenic	0.125	rs787302028	4
AT.AT	5	28159730	28159743	7	.	.	intergenic	.	952197 rs7912	2
AAAT.ATTT	5	28238832	28238854	5.75	.	.	intergenic	0.056	rs790124948	2
AC.GT	5	28507676	28507688	6.5	.	.	intergenic	.	.	2
AT.AT	5	28537658	28537676	9.5	.	.	intergenic	.	989885 rs7864	3
AAAAG.CTTTT	5	28882558	28882580	4.6	.	.	intergenic	.	rs793420915	2
AGG.CCT	5	29044428	29044446	6.33333	.	.	intergenic	-0.036	rs789989604	3
AAAAC.GTTTT	5	29210231	29210257	5.4	.	.	intergenic	0.037	485295 rs7926	2
AC.GT	5	29853024	29853038	7.5	.	.	intergenic	0.222	rs793567246	2
AC.GT	5	30183337	30183363	13.5	ENSSSCT00000029741	.	intron	1.026	.	2
AG.CT	5	30238992	30239005	7	.	.	intergenic	-0.034	402319 rs7886	2
AT.AT	5	30398998	30399009	6	.	.	intergenic	0.126	586751 rs7931	2

AT.AT	5	31298702	31298720	9.5	.	.	intergenic	0.014	rs790241021	2
AAC.GTT	5	31401697	31401717	7	.	.	intergenic	-0.11	'72776 rs7923	2
AAC.GTT	5	31431410	31431426	5.66667	ENSSSCT00000020867	U6	promoter	.	rs792485779	2
AAAT.ATTT	5	31462571	31462593	5.75	.	.	intergenic	.	rs788870371	2
AAAT.ATTT	5	31561893	31561906	3.5	.	.	intergenic	0.235	.	2
AAAC.GTTT	5	31588670	31588694	6.25	.	.	intergenic	0.068	i99642 rs7902	2
AAAAC.GTTTT	5	31715933	31715958	5.2	.	.	intergenic	0.055	rs787035574	2
AAC.GTT	5	31766269	31766280	4	ENSSSCT00000025861	SRGAP1	intron	0.588	rs789041469	2
AAAC.GTTT	5	32221677	32221691	3.75	ENSSSCT00000028687	TBC1D30	intron	0.147	rs786770777	2
AC.GT	5	32678916	32678930	7.5	.	.	intergenic	0.081	rs791671598	3
AC.GT	5	32786347	32786367	10.5	ENSSSCT00000000507	LEMD3	intron	0.427	.	5
AAC.GTT	5	33009949	33009965	5.66667	.	.	intergenic	0.291	rs789549514	3
AC.GT	5	33036152	33036165	7	ENSSSCT00000028225	MSRB3	intron	0.124	155300 rs7927	2
AAG.CTT	5	33057948	33057962	5	ENSSSCT00000028225	MSRB3	intron	-0.009	.	2
AC.GT	5	33171312	33171326	7.5	.	.	intergenic	0.133	rs790427726	2
AG.CT	5	33228316	33228325	5	.	.	intergenic	0.052	.	4
AAC.GTT	5	33274093	33274106	4.66667	.	.	intergenic	.	rs789529167	2
AAAAC.GTTTT	5	33276883	33276897	3	.	.	intergenic	.	rs787016193	2
AAAC.GTTT	5	33391263	33391279	4.25	ENSSSCT00000000509	HMGA2	intron	.	i87815 rs7902	2
AG.CT	5	33406060	33406070	5.5	ENSSSCT00000000509	HMGA2	intron	0.708	.	2
AAAC.GTTT	5	33936717	33936730	3.5	ENSSSCT00000000515	HELB	intron	-0.066	i19442 rs7867	2
ACTC.GAGT	5	34211027	34211046	5	ENSSSCT00000000516	GRIP1	intron	0.055	rs789141965	2
AAAAC.GTTTT	5	34243841	34243859	3.8	ENSSSCT00000000516	GRIP1	intron	-0.071	rs787356599	2
AG.CT	5	34298935	34298946	6	.	.	intergenic	0.061	125816 rs7903	2
AAT.ATT	5	34389577	34389589	4.33333	.	.	intergenic	-0.281	rs696456646	2
AAAC.GTTT	5	34412269	34412291	5.75	.	.	intergenic	0.051	rs789370231	2
AAAC.GTTT	5	34522475	34522492	4.5	.	.	intergenic	-0.074	rs789436456	2
AAAAC.GTTTT	5	34573928	34573950	4.6	.	.	intergenic	0.208	.	2
AAAAG.CTTTT	5	34601439	34601470	6.4	.	.	intergenic	0.184	)96042 rs7918	2
AAAT.ATTT	5	34833325	34833352	7	.	.	intergenic	0.045	rs711492149	2
AC.GT	5	34862561	34862571	5.5	.	.	intergenic	0.379	366950 rs7890	2
AAAC.GTTT	5	35180908	35180932	6.25	.	.	intergenic	-0.078	.	2
AC.GT	5	35364312	35364322	5.5	.	.	intergenic	0.038	rs786896987	2
.CTCTC.GAGAG	5	35540348	35540370	3.83333	.	.	intergenic	.	.	2
AC.GT	5	35810285	35810302	9	ENSSSCT00000000524	.	intron	0.229	rs793585266	3
AC.GT	5	35962335	35962345	5.5	ENSSSCT00000000526	MDM2	intron	.	rs788012636	2
AT.AT	5	36113275	36113286	6	.	.	intergenic	0.336	rs788075889	3
AC.GT	5	36182189	36182200	6	ENSSSCT00000033585	LYZ	intron	-0.106	.	3
AC.GT	5	36182189	36182200	6	ENSSSCT00000034939	LYZ	intron	-0.106	.	3
AC.GT	5	36182189	36182200	6	ENSSSCT00000000530	LYZ	intron	-0.106	.	3
AC.GT	5	36601044	36601053	5	.	.	intergenic	0.07	i68764 rs7933	3
AAAC.GTTT	5	36686334	36686351	4.5	ENSSSCT00000022623	.	intron	.	rs791374272	2
AT.AT	5	36965115	36965124	5	.	.	intergenic	-0.01	.	2
AAAG.CTTT	5	37057701	37057712	3	.	.	intergenic	0.31	.	2
AAG.CTT	5	37184310	37184323	4.66667	ENSSSCT00000000541	CNOT2	intron	0.204	rs793084428	3
AC.GT	5	37191693	37191704	6	ENSSSCT00000000541	CNOT2	intron	2.23	309398 rs7906	3
\AAAAC.GTTTT	5	37233830	37233853	4	.	.	intergenic	1.936	341253 rs7901	2
AAAAC.GTTTT	5	37275617	37275631	3	ENSSSCT00000000543	PTPRB	intron	0.267	.	2
AC.GT	5	37405659	37405669	5.5	ENSSSCT00000032079	PTPRR	intron	0.08	394799 rs7918	3
AG.CT	5	37493335	37493356	11	ENSSSCT00000032079	PTPRR	intron	.	)11453 rs6989	2
AAAC.GTTT	5	37655578	37655592	3.75	.	.	intergenic	0.149	rs790948225	2
AAC.GTT	5	37747133	37747155	7.66667	.	.	intergenic	-0.064	rs789047498	2
AT.AT	5	37767936	37767946	5.5	.	.	intergenic	-0.279	rs791041653	2
AATG.CATT	5	37852000	37852019	5	.	.	intergenic	0.023	rs787818634	2
AG.CT	5	37901119	37901133	7.5	.	.	intergenic	.	)19093 rs7901	2
AGAT.ATCT	5	38069714	38069750	9.25	.	.	intergenic	.	rs787713533	2
AAAT.ATTT	5	38357105	38357126	5.5	ENSSSCT00000000548	.	intron	-0.01	952081 rs7882	2
ACAT.ATGT	5	38359551	38359566	4	ENSSSCT00000000548	.	intron	0.217	109111 rs7902	2
AAT.ATT	5	38476956	38476972	5.66667	ENSSSCT00000000550	RAB21	intron	0.303	.	2
AC.GT	5	38599574	38599585	6	ENSSSCT00000000551	TBC1D15	intron	0.134	.	3
AT.AT	5	38612650	38612669	10	.	.	intergenic	-0.048	rs790448401	2
AC.GT	5	38695065	38695084	10	ENSSSCT00000000553	TPH2	intron	0.644	rs792164470	2
AT.AT	5	39099742	39099753	6	ENSSSCT00000000555	TRH-DE	intron	0.45	'87906 rs7931	2
AAAAT.ATTTT	5	39147598	39147622	5	ENSSSCT00000000555	TRH-DE	intron	.	i46637 rs7881	2
AAAAT.ATTTT	5	39160393	39160417	5	ENSSSCT00000000555	TRH-DE	intron	0.096	291485 rs7905	2
AATC.GATT	5	39271011	39271029	4.75	ENSSSCT00000000555	TRH-DE	intron	0.28	rs787600447	2
AC.GT	5	39288339	39288350	6	ENSSSCT00000000555	TRH-DE	intron	0.048	rs787442755	2
\AAAAC.GTTTT	5	39333482	39333506	4.16667	ENSSSCT00000000555	TRH-DE	intron	-0.04	rs787954421	4
AAC.GTT	5	39371033	39371052	6.66667	ENSSSCT00000000555	TRH-DE	intron	0.192	.	2
AC.GT	5	39490521	39490537	8.5	ENSSSCT00000000555	TRH-DE	intron	0.142	rs792906584	2
AAC.GTT	5	39506558	39506579	7.33333	ENSSSCT00000000555	TRH-DE	intron	.	rs788671838	2
AC.GT	5	39515548	39515565	9	ENSSSCT00000000555	TRH-DE	intron	.	rs790648064	6
AAT.ATT	5	39603525	39603536	4	.	.	intergenic	0.085	.	2
AAG.CTT	5	39610349	39610361	4.33333	.	.	intergenic	.	rs790195309	2
AAC.GTT	5	39699372	39699391	6.66667	.	.	intergenic	.	'92332 rs7875	3
AAAC.GTTT	5	40139752	40139767	4	.	.	intergenic	-0.189	rs790442389	2
AAAAT.ATTTT	5	40302898	40302923	5.2	.	.	intergenic	.	172994 rs7902	2
AC.GT	5	40321169	40321190	11	.	.	intergenic	-0.009	115590 rs7915	3
AAT.ATT	5	40336325	40336336	4	.	.	intergenic	0.061	rs789673277	2
AT.AT	5	40355074	40355083	5	.	.	intergenic	.	rs787076393	2
AAAG.CTTT	5	40785121	40785135	3.75	.	.	intergenic	0.118	.	2
AT.AT	5	40897041	40897051	5.5	.	.	intergenic	0.096	.	2
AAAC.GTTT	5	41432611	41432629	4.75	.	.	intergenic	-0.041	289135 rs7870	2
AAAGG.CCTTT	5	41433301	41433318	3.6	.	.	intergenic	0.031	319818 rs7924	2

AAAC.GTTT	5	41440160	41440182	5.75	.	.	intergenic	0.093	rs708752907	2
AC.GT	5	41664172	41664198	13.5	.	.	intergenic	-0.096	rs786503046	3
AAAC.GTTT	5	41697137	41697157	5.25	.	.	intergenic	0.175	rs791777335	2
AAT.ATT	5	42317040	42317054	5	.	.	intergenic	0.079	323202 rs7876	2
AT.AT	5	42378896	42378918	11.5	ENSSSCT00000000562	KRR1	intron	0	168676 rs7897	2
AG.CT	5	42450307	42450322	8	.	.	intergenic	0.231	.	2
AT.AT	5	42491107	42491117	5.5	.	.	intergenic	0.137	351470 rs7904	2
AAACT.AGTTT	5	42695366	42695396	6.2	.	.	intergenic	-0.281	rs789911009	2
AT.AT	5	42766377	42766387	5.5	.	.	intergenic	0.583	765195 rs7891	2
AG.CT	5	42964334	42964355	11	.	.	intergenic	0.077	539647 rs7879	2
AAAT.ATTT	5	43032537	43032550	3.5	.	.	intergenic	0.095	rs791403267	2
AAAAC.GTTTT	5	43306894	43306909	3.2	ENSSSCT00000029484	OSBPL8	intron	0.269	.	2
AAAT.ATTT	5	43988807	43988820	3.5	.	.	intergenic	.	.	3
ACAT.ATGT	5	44073284	44073306	5.75	.	.	intergenic	.	281068 rs7868	2
AAAC.GTTT	5	44087608	44087629	5.5	.	.	intergenic	.	rs789357703	2
AAAT.ATTT	5	44101615	44101637	5.75	.	.	intergenic	0.014	rs792393451	2
AAC.GTT	5	44541480	44541491	4	.	.	intergenic	0.18	.	2
AAAC.GTTT	5	44588740	44588756	4.25	ENSSSCT00000000570	PKP2	intron	-0.119	077368 rs7878	2
AT.AT	5	44710359	44710370	6	.	.	intergenic	0.212	361092 rs7921	3
AAAT.ATTT	5	44763382	44763400	4.75	ENSSSCT00000000571	.	intron	.	.	3
AAAT.ATTT	5	44763382	44763400	4.75	ENSSSCT00000030054	.	intron	.	.	3
AACC.GGTT	5	44886260	44886284	6.25	ENSSSCT00000031839	.	intron	-0.214	304868 rs7917	2
AAT.ATT	5	44932344	44932361	6	ENSSSCT00000031839	.	intron	0.051	rs790230710	3
AAT.ATT	5	44932344	44932361	6	ENSSSCT00000000572	FGD4	promoter	0.051	rs790230710	3
AAT.ATT	5	44932344	44932361	6	ENSSSCT00000036458	FGD4	promoter	0.051	rs790230710	3
AAAG.CTTT	5	45536934	45536949	4	.	.	intergenic	.	rs786337738	2
AG.CT	5	46610527	46610539	6.5	.	.	intergenic	.	rs787073657	2
AT.AT	5	46649857	46649867	5.5	.	.	intergenic	.	rs790522238	2
AAAAAC.GTTTTT	5	47129181	47129201	3.5	.	.	intergenic	0.15	554703 rs7883	2
AC.GT	5	47171854	47171872	9.5	.	.	intergenic	0.174	rs791858767	2
AAAT.ATTT	5	47422864	47422887	6	ENSSSCT00000028514	.	promoter	.	168510 rs7903	2
AT.AT	5	47548107	47548119	6.5	ENSSSCT00000000582	OVCH1	intron	.	rs790736162	2
AAAT.ATTT	5	47662507	47662525	4.75	ENSSSCT00000000578	ERGIC2	intron	0.144	rs789788826	2
AC.GT	5	48000562	48000574	6.5	.	.	intergenic	.	565422 rs7923	2
AC.GT	5	48242996	48243008	6.5	.	.	intergenic	1.585	rs786390287	2
AT.AT	5	48244327	48244341	7.5	.	.	intergenic	.	rs791886668	2
AGGG.CCCT	5	48270418	48270445	7	.	.	intergenic	.	.	2
AAC.GTT	5	48538424	48538446	7.66667	.	.	intergenic	.	rs703333250	3
AC.GT	5	48800401	48800413	6.5	.	.	intergenic	0.16	rs789408815	4
AAAT.ATTT	5	49271054	49271075	5.5	.	.	intergenic	.	rs790321352	2
AAC.GTT	5	49444612	49444627	5.33333	.	.	intergenic	.	rs791433919	2
AAAC.GTTT	5	49511773	49511787	3.75	ENSSSCT00000000588	KLHL42	intron	0.038	rs791234072	2
AAAC.GTTT	5	49618912	49618937	6.5	ENSSSCT00000027008	PPFIBP1	intron	0.222	323936 rs7904	2
AAAC.GTTT	5	49618912	49618937	6.5	ENSSSCT00000000592	PPFIBP1	intron	0.222	323936 rs7904	2
AAAC.GTTT	5	49618912	49618937	6.5	ENSSSCT00000031448	PPFIBP1	intron	0.222	323936 rs7904	2
AAC.GTT	5	50138288	50138309	7.33333	.	.	intergenic	.	rs790851123	2
AAC.GTT	5	50384927	50384944	6	ENSSSCT00000031326	ASUN	intron	.	rs791954785	2
AAAC.GTTT	5	50398783	50398796	3.5	ENSSSCT00000031326	ASUN	intron	0.047	rs789249126	2
AAAC.GTTT	5	51525324	51525342	4.75	ENSSSCT00000000602	RASSF8	intron	0.12	rs791460113	3
AAGG.CCTT	5	51631090	51631111	5.5	.	.	intergenic	.	rs696122658	2
AC.GT	5	51998912	51998927	8	.	.	intergenic	.	105604 rs7910	2
AG.CT	5	52107206	52107221	8	.	.	intergenic	-0.594	rs793572838	2
AAAT.ATTT	5	52253996	52254018	5.75	ENSSSCT00000025989	KRAS	intron	0.536	rs793861932	2
AAG.CTT	5	52838182	52838205	8	.	.	intergenic	0.173	rs791553967	2
AG.CT	5	52883414	52883432	9.5	.	.	intergenic	0.101	rs790965057	2
AT.AT	5	53205293	53205304	6	.	.	intergenic	-0.08	rs786936851	2
AAAAC.GTTTT	5	53305701	53305724	4.8	.	.	intergenic	0.158	417186 rs7926	3
AC.GT	5	53375479	53375493	7.5	.	.	intergenic	-0.194	.	3
AC.GT	5	53718466	53718480	7.5	.	.	intergenic	-0.069	.	3
AAC.GTT	5	53787480	53787492	4.33333	.	.	intergenic	-0.081	rs791229785	2
AT.AT	5	53791359	53791368	5	.	.	intergenic	0.3	rs787778496	2
AAAG.CTTT	5	53844213	53844245	8.25	.	.	intergenic	0.437	354317 rs7920	2
AAAT.ATTT	5	53871917	53871936	5	.	.	intergenic	.	rs792563610	2
AAGT.ACTT	5	53910190	53910201	3	.	.	intergenic	0.029	.	2
AC.GT	5	54101821	54101833	6.5	.	.	intergenic	0.075	rs789405302	2
AAAC.GTTT	5	54328697	54328731	8.75	.	.	intergenic	0.979	rs793617609	2
AAAAAC.GTTTTT	5	54660455	54660477	3.83333	.	.	intergenic	-0.167	rs788974012	2
AAAC.GTTT	5	54777123	54777139	4.25	.	.	intergenic	0.217	rs788401105	2
AAAC.GTTT	5	54947959	54947980	5.5	.	.	intergenic	0.005	594962 rs7931	3
AT.AT	5	55084616	55084626	5.5	.	.	intergenic	0.169	rs788722005	2
AAAAC.GTTTT	5	55170394	55170420	5.4	ENSSSCT00000000621	GYS2	intron	.	rs792063776	2
AAAC.GTTT	5	55333293	55333307	3.75	.	.	intergenic	0.02	rs790298794	2
AC.GT	5	55483396	55483406	5.5	.	.	intergenic	.	.	4
AAAAC.GTTTT	5	55695115	55695133	3.8	.	.	intergenic	.	rs787734815	2
AAAAC.GTTTT	5	55809557	55809590	6.8	ENSSSCT00000027940	.	intron	0.103	rs791598239	2
ACAT.ATGT	5	55819066	55819096	7.75	ENSSSCT00000027940	.	intron	0.033	rs790420648	2
AC.GT	5	56114174	56114196	11.5	.	.	intergenic	.	rs790113860	2
AG.CT	5	56161994	56162010	8.5	.	.	intergenic	.	rs787871933	2
AAAC.GTTT	5	56236402	56236417	4	.	.	intergenic	.	rs789188126	2
AT.AT	5	56479762	56479772	5.5	.	.	intergenic	.	rs789535652	4
AC.GT	5	56703873	56703884	6	.	.	intergenic	0.017	rs792346090	2
AAAC.GTTT	5	56831159	56831176	4.5	.	.	intergenic	0.274	104590 rs7936	2
AC.GT	5	56968177	56968195	9.5	ENSSSCT00000000632	AEBP2	intron	0.18	.	3
AAAC.GTTT	5	56977443	56977461	4.75	ENSSSCT00000000632	AEBP2	intron	-0.109	076177 rs7868	2

AAAC.GTTT	5	57002078	57002099	5.5	.	.	intergenic	0.083	rs792524450	2
AAAC.GTTT	5	57075331	57075357	6.75	ENSSSCT00000000633	.	intron	-0.061	rs789182217	2
AG.CT	5	57139998	57140008	5.5	ENSSSCT00000000633	.	intron	0.17	rs792370941	2
AT.AT	5	57207395	57207408	7	.	.	intergenic	0.047	rs789265256	3
AAAT.ATTT	5	57215150	57215173	6	.	.	intergenic	-0.063	rs792187995	2
AAAC.GTTT	5	57218740	57218758	4.75	.	.	intergenic	0.03	461000 rs7931	4
AAAT.ATTT	5	57271827	57271841	3.75	.	.	intergenic	0.016	283973 rs7913	2
AG.CT	5	57539939	57539957	9.5	.	.	intergenic	0.08	rs793216505	2
AT.AT	5	57654693	57654706	7	.	.	intergenic	0.064	517309 rs7926	2
AC.GT	5	57678205	57678221	8.5	.	.	intergenic	-0.031	rs792398517	2
AAG.CTT	5	57705137	57705149	4.33333	ENSSSCT00000000635	PLCZ1	intron	-0.181	rs787313793	2
AAC.GTT	5	57875505	57875524	6.66667	ENSSSCT00000031285	PIK3C2G	intron	0.025	rs786500379	3
AAC.GTT	5	57875505	57875524	6.66667	ENSSSCT00000000636	PIK3C2G	intron	0.025	rs786500379	3
AAC.GTT	5	57875505	57875524	6.66667	ENSSSCT00000027538	PIK3C2G	intron	0.025	rs786500379	3
AAAC.GTTT	5	57880580	57880610	7.75	ENSSSCT00000031285	PIK3C2G	intron	-0.012	rs790745872	2
AAAC.GTTT	5	57880580	57880610	7.75	ENSSSCT00000000636	PIK3C2G	intron	-0.012	rs790745872	2
AAAC.GTTT	5	57880580	57880610	7.75	ENSSSCT00000027538	PIK3C2G	intron	-0.012	rs790745872	2
AC.GT	5	58280881	58280891	5.5	.	.	intergenic	0.061	.	3
AAAC.GTTT	5	58296248	58296275	7	.	.	intergenic	.	rs790214617	2
AG.CT	5	58327749	58327764	8	.	.	intergenic	0.224	rs793271519	5
AG.CT	5	58399288	58399299	6	.	.	intergenic	-0.076	.	4
AATC.GATT	5	58402884	58402907	6	.	.	intergenic	-0.066	rs792850416	2
AC.GT	5	58576414	58576432	9.5	.	.	intergenic	-0.09	rs789976205	2
AT.AT	5	58641927	58641941	7.5	.	.	intergenic	0.058	rs792416349	2
AAC.GTT	5	59027724	59027745	7.33333	.	.	intergenic	.	rs786863185	2
AT.AT	5	59106173	59106183	5.5	.	.	intergenic	0.584	rs787509732	2
AT.AT	5	59298521	59298530	5	.	.	intergenic	0.083	rs792431406	2
AG.CT	5	59321546	59321555	5	.	.	intergenic	1.336	rs789456714	2
AG.CT	5	59433199	59433223	12.5	.	.	intergenic	0.097	rs791481984	2
AAAC.GTTT	5	59615080	59615111	8	.	.	intergenic	0.466	718007 rs7864	2
AC.GT	5	59647563	59647577	7.5	ENSSSCT00000000643	SLC15A5	promoter	.	.	2
AC.GT	5	59649191	59649216	13	ENSSSCT00000000643	SLC15A5	promoter	1.192	rs788035808	2
AG.CT	5	59711975	59711989	7.5	ENSSSCT00000000643	SLC15A5	intron	-0.015	rs789697643	2
AAG.CTT	5	59765979	59766003	8.33333	.	.	intergenic	0.485	rs787690384	3
AC.GT	5	60192297	60192307	5.5	.	.	intergenic	.	.	3
AG.CT	5	60294076	60294086	5.5	.	.	intergenic	-0.353	rs787514915	2
AC.GT	5	60316124	60316141	9	ENSSSCT00000000645	.	intron	0.544	596185 rs7865	2
AAAC.GTTT	5	60365584	60365617	8.5	ENSSSCT00000000645	.	intron	0.029	rs791115998	2
AC.GT	5	60491369	60491381	6.5	ENSSSCT00000027092	.	intron	1.45	.	3
AC.GT	5	60501932	60501960	14.5	ENSSSCT00000027092	.	intron	.	.	3
AAC.GTT	5	60504262	60504275	4.66667	ENSSSCT00000027092	.	intron	0.023	rs791232359	2
AT.AT	5	60582290	60582301	6	.	.	intergenic	0.155	rs790422519	2
AAG.CTT	5	60629468	60629484	5.66667	.	.	intergenic	0.101	501323 rs7869	2
AG.CT	5	60748295	60748322	14	ENSSSCT00000000647	RERG	intron	0.735	rs793211587	3
AC.GT	5	60755669	60755680	6	ENSSSCT00000000647	RERG	intron	0.013	rs790351603	2
AAAAAC.GTTTTT	5	60825047	60825067	3.5	ENSSSCT00000000647	RERG	intron	-0.044	409014 rs7918	2
ATCC.GGAT	5	60827344	60827361	4.5	ENSSSCT00000000647	RERG	intron	-0.003	.	2
AAAG.CTTT	5	60865139	60865165	6.75	.	.	intergenic	0.227	rs790361374	2
AAAC.GTTT	5	60884578	60884601	6	.	.	intergenic	-0.018	rs786397930	2
AG.CT	5	60933457	60933467	5.5	.	.	intergenic	0.069	.	2
AC.GT	5	60975661	60975670	5	ENSSSCT00000000649	ARHGDIB	intron	1.565	rs786598512	2
AC.GT	5	60978296	60978310	7.5	ENSSSCT00000000649	ARHGDIB	intron	0.059	rs787673295	2
ACC.GGT	5	61080613	61080625	4.33333	.	.	intergenic	0.088	492492 rs7911	2
AG.CT	5	61414619	61414634	8	.	.	intergenic	.	.	3
AC.GT	5	61444369	61444383	7.5	ENSSSCT00000000658	ATF7IP	3'utr	1.93	.	3
AC.GT	5	61444369	61444383	7.5	ENSSSCT00000022782	ATF7IP	3'utr	1.93	.	3
AAC.GTT	5	61877438	61877454	5.66667	.	.	intergenic	0.404	rs789418723	2
AC.GT	5	61917578	61917588	5.5	.	.	intergenic	0.317	.	4
AC.GT	5	62351996	62352010	7.5	.	.	intergenic	0.131	.	2
AC.GT	5	62444185	62444197	6.5	.	.	intergenic	0.013	rs786443298	3
AAT.ATT	5	62528315	62528332	6	.	.	intergenic	0.233	rs793780894	2
AAAT.ATTT	5	62638880	62638903	6	.	.	intergenic	-0.089	262287 rs7929	2
AG.CT	5	63075232	63075242	5.5	.	.	intergenic	0.209	rs788737517	5
ATCC.GGAT	5	63185951	63185986	9	ENSSSCT00000031037	ETV6	intron	0.013	515801 rs7885	2
AG.CT	5	63215970	63215979	5	ENSSSCT00000031037	ETV6	intron	-0.074	.	2
AG.CT	5	63234730	63234743	7	ENSSSCT00000031037	ETV6	intron	.	rs793715192	3
AAT.ATT	5	63235580	63235593	4.66667	ENSSSCT00000031037	ETV6	intron	0.186	rs790377810	2
AAAC.GTTT	5	63239469	63239483	3.75	ENSSSCT00000031037	ETV6	intron	-0.145	rs793559511	2
AGGG.CCCT	5	63248284	63248303	5	ENSSSCT00000031037	ETV6	intron	0.777	rs787448705	2
AG.CT	5	63300016	63300027	6	ENSSSCT00000031037	ETV6	intron	-0.057	rs790858751	2
AAAC.GTTT	5	63592694	63592723	7.5	ENSSSCT00000000671	LRP6	intron	.	rs793700854	3
AG.CT	5	63711797	63711815	9.5	ENSSSCT00000000669	BCL2L14	intron	-0.062	rs792818606	2
AAAC.GTTT	5	63971735	63971760	6.5	.	.	intergenic	.	rs786924178	2
AT.AT	5	63976656	63976668	6.5	ENSSSCT00000000678	TAS2R9	promoter	-0.111	532659 rs7914	3
AG.CT	5	63997225	63997237	6.5	.	.	intergenic	.	052750 rs7907	2
AC.GT	5	64132941	64132955	7.5	ENSSSCT00000000681	STYK1	intron	0.125	534258 rs7937	2
AC.GT	5	64159002	64159012	5.5	.	.	intergenic	0.017	rs788817730	2
AG.CT	5	64343133	64343146	7	.	.	intergenic	.	rs787533810	2
AG.CT	5	64414198	64414208	5.5	.	.	intergenic	0.017	rs788257786	2
AG.CT	5	64521553	64521562	5	ENSSSCT00000000703	CLEC1A	intron	0.464	445047 rs7888	4
AG.CT	5	64521553	64521562	5	ENSSSCT00000027764	CLEC1A	intron	0.464	445047 rs7888	4
AT.AT	5	64549810	64549835	13	ENSSSCT00000033773	CLEC7A	intron	0.018	.	4
AT.AT	5	64549810	64549835	13	ENSSSCT00000000700	CLEC7A	intron	0.018	.	4
AT.AT	5	64549810	64549835	13	ENSSSCT00000000699	CLEC7A	intron	0.018	.	4

AT.AT	5	64549810	64549835	13	ENSSSCT00000000701	CLEC7A	intron	0.018	.	4
AT.AT	5	64549810	64549835	13	ENSSSCT00000033700	CLEC7A	intron	0.018	.	4
AG.CT	5	64552169	64552180	6	ENSSSCT00000000699	CLEC7A	intron	0.021	.	3
AG.CT	5	64552169	64552180	6	ENSSSCT00000033700	CLEC7A	intron	0.021	.	3
AG.CT	5	64552169	64552180	6	ENSSSCT00000000700	CLEC7A	intron	0.021	.	3
AG.CT	5	64552169	64552180	6	ENSSSCT00000033773	CLEC7A	intron	0.021	.	3
AG.CT	5	64552169	64552180	6	ENSSSCT00000000701	CLEC7A	intron	0.021	.	3
AT.AT	5	64733633	64733643	5.5	.	.	intergenic	.	rs793167888	3
AAAC.GTTT	5	64759846	64759872	6.75	ENSSSCT00000000707	CLEC12A	intron	.	I75026 rs7936	2
AAAC.GTTT	5	64759846	64759872	6.75	ENSSSCT00000035148	CLEC12A	intron	.	I75026 rs7936	2
AAG.CTT	5	64881915	64881928	4.66667	.	.	intergenic	0.147	rs692962230	2
AAAC.GTTT	5	64892367	64892387	5.25	.	.	intergenic	-0.097	)57897 rs7937	2
AAC.GTT	5	64911233	64911250	6	ENSSSCT00000000709	CD69	intron	-0.071	rs788857275	2
AAC.GTT	5	64911233	64911250	6	ENSSSCT00000000708	CD69	intron	-0.071	rs788857275	2
AAG.CTT	5	65000411	65000434	8	.	.	intergenic	.	rs789668336	2
AAAC.GTTT	5	65007805	65007824	5	.	.	intergenic	.	rs789768111	2
AT.AT	5	65405361	65405374	7	.	.	intergenic	.	.	2
AG.CT	5	65472831	65472846	8	ENSSSCT00000000720	A2ML1	intron	.	;19244 rs7873	2
AAAC.GTTT	5	65602241	65602263	5.75	.	.	intergenic	0.23	rs709732225	2
AT.AT	5	65637818	65637832	7.5	.	.	intergenic	0.13	rs791398221	2
AAT.ATT	5	65940241	65940253	4.33333	ENSSSCT00000027671	PEX5	intron	0.16	rs694101583	4
AC.GT	5	65963672	65963703	16	.	.	intergenic	.	?73754 rs7913	2
AG.CT	5	66310961	66310975	7.5	ENSSSCT00000000742	LEPREL2	intron	.	.	3
AAT.ATT	5	66517352	66517369	6	ENSSSCT00000000754	PIANP	intron	0.137	rs788577014	2
AAAC.GTTT	5	66631900	66631913	3.5	.	.	intergenic	.	rs787391482	2
AC.GT	5	66675234	66675244	5.5	.	.	intergenic	.	rs788931158	2
AC.GT	5	67344826	67344838	6.5	.	.	intergenic	-0.055	.	2
AGC.GCT	5	67348437	67348453	5.66667	.	.	intergenic	-0.208	rs789942600	2
AAAC.GTTT	5	67381639	67381661	5.75	.	.	intergenic	-0.115	rs793366423	2
AC.GT	5	68098717	68098728	6	ENSSSCT00000000785	RAD51AP1	intron	0.626	rs787797665	2
AC.GT	5	68133864	68133874	5.5	ENSSSCT00000000786	C12orf4	intron	-0.037	.	2
AC.GT	5	68241600	68241624	12.5	.	.	intergenic	0.429	rs792077401	2
AC.GT	5	69775864	69775888	12.5	ENSSSCT00000000812	IQSEC3	intron	0.006	rs791057588	4
AAAAT.ATTTT	5	70017275	70017295	4.2	ENSSSCT00000023353	KDM5A	intron	-0.135	rs791439868	2
AAAAC.GTTTT	5	70343594	70343617	4.8	ENSSSCT00000000817	WNK1	intron	0.199	rs788953824	3
AT.AT	5	70563564	70563576	6.5	ENSSSCT00000000819	RAB6IP2	intron	.	rs787191862	2
AG.CT	5	70578218	70578248	15.5	ENSSSCT00000000819	RAB6IP2	intron	0.031	rs789975592	2
ATC.GAT	5	70614382	70614399	6	ENSSSCT00000000819	RAB6IP2	intron	0.163	rs790187760	2
ACAT.ATGT	5	70623677	70623692	4	ENSSSCT00000000819	RAB6IP2	intron	.	.	2
AC.GT	5	70758620	70758631	6	.	.	intergenic	.	.	3
AAC.GTT	5	71309083	71309100	6	.	.	intergenic	0.183	rs788454386	3
AAAAC.GTTTT	5	71361463	71361496	6.8	.	.	intergenic	0.23	rs790004991	2
AAAAC.GTTTT	5	72070900	72070918	3.8	ENSSSCT00000031909	BCL2L13	intron	.	rs793830810	2
AT.AT	5	72089246	72089263	9	ENSSSCT00000031909	BCL2L13	intron	0.212	rs788640242	2
AT.AT	5	72177332	72177344	6.5	ENSSSCT00000000834	.	intron	.	)59999 rs7916	2
AGG.CCT	5	72650256	72650270	5	.	.	intergenic	.	rs789553249	3
AAT.ATT	5	72658577	72658595	6.33333	.	.	intergenic	-0.012	rs788780661	2
ACAT.ATGT	5	72777413	72777426	3.5	.	.	intergenic	0.003	rs793640423	2
AAAT.ATTT	5	72779618	72779633	4	.	.	intergenic	0.257	rs787994679	2
AAT.ATT	5	72804363	72804376	4.66667	ENSSSCT00000028240	CPNE8	intron	0.126	rs788736129	2
AAT.ATT	5	72804363	72804376	4.66667	ENSSSCT00000000842	CPNE8	intron	0.126	rs788736129	2
AAT.ATT	5	72804363	72804376	4.66667	ENSSSCT00000027279	CPNE8	intron	0.126	rs788736129	2
AAAG.CTTT	5	73048700	73048715	4	ENSSSCT00000028240	CPNE8	intron	0.153	rs789089185	2
AT.AT	5	73095654	73095665	6	ENSSSCT00000028240	CPNE8	intron	-0.019	.	2
AT.AT	5	73152609	73152619	5.5	.	.	intergenic	0.218	372384 rs7870	2
AGG.CCT	5	73412917	73412933	5.66667	ENSSSCT00000000843	KIF21A	intron	0.018	.	2
AAAG.CTTT	5	73732161	73732187	6.75	ENSSSCT00000000845	ABCD2	intron	0.133	rs786834847	2
AT.AT	5	73813618	73813632	7.5	ENSSSCT00000000846	C12orf40	intron	.	rs790220822	2
AAAT.ATTT	5	73820653	73820670	4.5	ENSSSCT00000000846	C12orf40	intron	.	rs788968218	2
AG.CT	5	73951966	73951975	5	ENSSSCT00000034370	SLC2A13	intron	0.118	.	3
AT.AT	5	73963953	73963964	6	ENSSSCT00000034370	SLC2A13	intron	0.023	rs792926914	2
ACAT.ATGT	5	73964054	73964072	4.75	ENSSSCT00000034370	SLC2A13	intron	-0.039	.	2
AT.AT	5	73980232	73980249	9	ENSSSCT00000034370	SLC2A13	intron	0.037	.	5
AT.AT	5	73995600	73995614	7.5	ENSSSCT00000034370	SLC2A13	intron	0.172	rs787077874	2
AAT.ATT	5	73997634	73997655	7.33333	ENSSSCT00000034370	SLC2A13	intron	0.175	rs787993260	2
AAG.CTT	5	74134714	74134731	6	.	.	intergenic	0.318	rs789571068	2
AAAC.GTTT	5	74639476	74639498	5.75	ENSSSCT00000000849	LRRK2	intron	2.987	rs788464692	2
AG.CT	5	75026870	75026880	5.5	.	.	intergenic	-0.242	.	2
AC.GT	5	75173842	75173853	6	ENSSSCT00000000855	CNTN1	intron	0.022	rs786239328	2
AT.AT	5	75378144	75378154	5.5	ENSSSCT00000034945	CNTN1	intron	0.259	rs789436177	2
AT.AT	5	75378144	75378154	5.5	ENSSSCT00000036262	CNTN1	intron	0.259	rs789436177	2
AT.AT	5	75460909	75460919	5.5	.	.	intergenic	.	rs787656390	3
AC.GT	5	75548494	75548528	17.5	.	.	intergenic	0.456	;38008 rs7913	4
GAGGG.CCCTC	5	75601711	75601742	5.33333	.	.	intergenic	0.154	.	2
AAAC.GTTT	5	75603867	75603901	8.75	.	.	intergenic	0.072	rs790961447	2
AC.GT	5	75617201	75617214	7	.	.	intergenic	0.047	.	4
AC.GT	5	75624825	75624839	7.5	.	.	intergenic	0.026	rs790767195	2
AC.GT	5	75629390	75629409	10	.	.	intergenic	0.09	?61619 rs7923	4
\AAAAT.ATTTTT	5	75648629	75648651	3.83333	.	.	intergenic	-0.293	rs790946606	2
AAAAG.CTTTT	5	75716649	75716675	5.4	.	.	intergenic	-0.09	rs788869162	2
AG.CT	5	75764940	75764949	5	.	.	intergenic	-0.181	rs792626424	2
AAGG.CCTT	5	75793010	75793035	6.5	.	.	intergenic	-0.068	.	3
AAAC.GTTT	5	75944353	75944368	4	.	.	intergenic	-0.075	rs793046275	2
\AAAAG.CTTTT	5	75985605	75985629	4.16667	.	.	intergenic	0.006	rs787787452	2

AC.GT	5	76212044	76212054	5.5	.	.	intergenic	.	rs786917626	2
AC.GT	5	76325340	76325349	5	ENSSSCT00000000859	PPHLN1	intron	0.131	.	2
AAAT.ATTT	5	76651777	76651788	3	.	.	intergenic	.	rs793759285	2
AC.GT	5	76658063	76658077	7.5	.	.	intergenic	.	rs791570050	4
AAAC.GTTT	5	76771663	76771686	6	.	.	intergenic	-0.22	rs790424050	2
AC.GT	5	76794813	76794823	5.5	.	.	intergenic	0.108	rs789005193	2
AT.AT	5	77057866	77057877	6	.	.	intergenic	.	.	3
AT.AT	5	77214784	77214794	5.5	.	.	intergenic	.	rs786366496	2
AT.AT	5	77446778	77446789	6	ENSSSCT00000000862	ADAMTS2(	intron	0.126	rs793771542	2
AAG.CTT	5	77451208	77451219	4	ENSSSCT00000000862	ADAMTS2(	intron	-0.091	rs786787004	2
AC.GT	5	77480222	77480232	5.5	ENSSSCT00000000862	ADAMTS2(	intron	-0.097	rs786759565	3
AT.AT	5	77489406	77489417	6	.	.	intergenic	0.065	.	2
AAAAT.ATTTT	5	77510071	77510096	5.2	.	.	intergenic	0.03	312515 rs7918	2
AG.CT	5	77517113	77517138	13	.	.	intergenic	0.085	rs792516835	2
AC.GT	5	77526666	77526678	6.5	.	.	intergenic	0.759	rs788107800	2
AAG.CTT	5	77645597	77645608	4	ENSSSCT00000000867	PUS7L	intron	0.106	935086 rs7892	3
AAG.CTT	5	77645597	77645608	4	ENSSSCT00000000866	IRAK4	promoter	0.106	935086 rs7892	3
AAG.CTT	5	77645597	77645608	4	ENSSSCT00000035861	IRAK4	promoter	0.106	935086 rs7892	3
AAAT.ATTT	5	77794536	77794554	4.75	.	.	intergenic	0.073	.	2
ACACAT.ATGTG	5	77966222	77966246	4.16667	.	.	intergenic	0.226	rs792513865	2
AAC.GTT	5	78029012	78029028	5.66667	.	.	intergenic	.	rs791367968	2
AT.AT	5	78086027	78086036	5	.	.	intergenic	-0.085	.	3
AC.GT	5	78120065	78120084	10	.	.	intergenic	0.032	rs793079175	2
AT.AT	5	78125336	78125346	5.5	.	.	intergenic	0.027	rs789274540	3
AAAT.ATTT	5	78134529	78134547	4.75	.	.	intergenic	0.117	rs788974308	2
AAC.GTT	5	78540246	78540262	5.66667	ENSSSCT00000000870	NELL2	intron	0.104	rs789330256	4
AC.GT	5	78565118	78565131	7	ENSSSCT00000000870	NELL2	intron	-0.068	rs789028190	2
AGATAT.ATATC	5	78580040	78580077	6.33333	ENSSSCT00000000870	NELL2	intron	0.004	rs790746379	3
AAAAT.ATTTT	5	78591323	78591341	3.8	ENSSSCT00000000870	NELL2	intron	0.107	rs790749678	2
AAC.GTT	5	78672140	78672159	6.66667	ENSSSCT00000000870	NELL2	intron	0.06	rs786710211	2
AAATC.GATTT	5	78963036	78963064	5.8	.	.	intergenic	.	rs790556993	2
AAAG.CTTT	5	79070827	79070843	4.25	.	.	intergenic	.	538931 rs7937	2
AAAT.ATTT	5	79137934	79137952	4.75	ENSSSCT00000000873	ANO6	intron	.	rs788960988	2
AATAT.ATATT	5	79250357	79250385	5.8	.	.	intergenic	0.196	030703 rs7904	2
AAAAG.CTTTT	5	79362709	79362728	4	.	.	intergenic	0.012	549611 rs7879	3
AAC.GTT	5	79406359	79406384	8.66667	.	.	intergenic	.	538525 rs7925	2
AT.AT	5	79492188	79492198	5.5	.	.	intergenic	.	.	3
AT.AT	5	79739655	79739667	6.5	ENSSSCT00000023077	SCAF11	intron	1.81	328895 rs7882	2
AT.AT	5	79739655	79739667	6.5	ENSSSCT00000031177	SCAF11	intron	1.81	328895 rs7882	2
AT.AT	5	79739655	79739667	6.5	ENSSSCT00000000875	SCAF11	intron	1.81	328895 rs7882	2
AT.AT	5	79908706	79908716	5.5	.	.	intergenic	0.016	.	4
AAAT.ATTT	5	79983942	79983962	5.25	ENSSSCT00000000876	SLC38A1	intron	0.393	378003 rs7936	2
AAAC.GTTT	5	80008702	80008715	3.5	.	.	intergenic	.	rs790227746	2
AAAT.ATTT	5	80016827	80016847	5.25	.	.	intergenic	-0.078	215980 rs7911	2
AC.GT	5	80101801	80101819	9.5	.	.	intergenic	0.049	rs792963302	5
AAAAG.CTTTT	5	80307234	80307260	5.4	.	.	intergenic	.	124519 rs7901	2
AC.GT	5	80410764	80410775	6	ENSSSCT00000029189	SLC38A4	intron	0.105	rs787655268	2
AATG.CATT	5	80642879	80642906	7	.	.	intergenic	-0.091	rs791469144	2
AAACC.GGTTT	5	81082452	81082468	3.4	.	.	intergenic	0.007	rs790882818	2
AG.CT	5	81101898	81101908	5.5	.	.	intergenic	0.366	.	2
AAT.ATT	5	81316067	81316083	5.66667	ENSSSCT00000032274	VDR	intron	0.034	rs792773981	2
AAAC.GTTT	5	81823019	81823033	3.75	.	.	intergenic	.	rs792173823	2
AAAAT.ATTTT	5	81824174	81824195	4.4	.	.	intergenic	.	rs793807236	4
ACC.GGT	5	81897633	81897648	5.33333	.	.	intergenic	-0.286	rs792038136	2
AC.GT	5	82175623	82175633	5.5	.	.	intergenic	0.181	226132 rs7896	2
AT.AT	5	82459452	82459462	5.5	ENSSSCT00000025699	.	intron	.	.	2
AC.GT	5	82662042	82662057	8	ENSSSCT00000000915	APPL2	intron	.	rs791181165	3
AAAT.ATTT	5	82668577	82668591	3.75	ENSSSCT00000000915	APPL2	intron	-0.381	463065 rs7879	2
AAT.ATT	5	83030451	83030476	8.66667	ENSSSCT00000000911	SLC41A2	intron	0.063	rs791433966	2
AC.GT	5	83110990	83111000	5.5	ENSSSCT00000000911	SLC41A2	intron	0.022	rs792503657	2
ATATC.GATAT	5	83233315	83233329	3	ENSSSCT00000000910	CHST11	intron	-0.069	rs786209092	2
AAGG.CCTT	5	83462394	83462412	4.75	ENSSSCT00000000910	CHST11	intron	-0.069	rs787588213	2
AAC.GTT	5	83839954	83839967	4.66667	.	.	intergenic	0.278	070520 rs7938	2
AAAG.CTTT	5	83893623	83893637	3.75	.	.	intergenic	.	rs786711417	2
AAAC.GTTT	5	83984098	83984118	5.25	.	.	intergenic	0.221	rs792320154	2
AC.GT	5	84038931	84038950	10	ENSSSCT00000000926	HSP90B1	intron	-0.256	rs789746840	4
AC.GT	5	84038931	84038950	10	ENSSSCT00000000925	GLT8D2	intron	-0.256	rs789746840	4
AAAG.CTTT	5	84073364	84073382	4.75	ENSSSCT00000000926	HSP90B1	intron	.	rs788422493	2
AC.GT	5	84154401	84154411	5.5	ENSSSCT00000000930	.	intron	-0.021	.	2
AAAC.GTTT	5	84171786	84171804	4.75	ENSSSCT00000000930	.	intron	0.333	169455 rs7910	2
AAAAC.GTTTT	5	84494053	84494077	5	.	.	intergenic	.	266008 rs7871	2
AG.CT	5	84537279	84537295	8.5	.	.	intergenic	-0.2	.	2
AG.CT	5	84597858	84597872	7.5	.	.	intergenic	0.245	.	2
AAAAC.GTTTT	5	84959338	84959359	4.4	.	.	intergenic	0.103	.	2
AT.AT	5	85062200	85062210	5.5	ENSSSCT00000022294	.	promoter	0.002	rs793488247	3
AC.GT	5	85610631	85610644	7	.	.	intergenic	1.757	rs789162522	2
AC.GT	5	85764617	85764627	5.5	ENSSSCT00000034902	IGFI	intron	0.179	.	5
AC.GT	5	85764617	85764627	5.5	ENSSSCT00000034323	IGFI	intron	0.179	.	5
AC.GT	5	85764617	85764627	5.5	ENSSSCT00000034198	IGFI	intron	0.179	.	5
AC.GT	5	85764617	85764627	5.5	ENSSSCT00000035604	IGFI	intron	0.179	.	5
AC.GT	5	85764617	85764627	5.5	ENSSSCT00000000936	IGFI	intron	0.179	.	5
AC.GT	5	85764617	85764627	5.5	ENSSSCT00000035532	IGFI	intron	0.179	.	5
AC.GT	5	85764617	85764627	5.5	ENSSSCT00000035024	IGFI	intron	0.179	.	5
AC.GT	5	85764617	85764627	5.5	ENSSSCT00000034991	IGFI	intron	0.179	.	5

AT.AT	5	85810054	85810070	8.5	.	.	intergenic	0.076	219375 rs7924	2
AC.GT	5	86069438	86069461	12	ENSSSCT00000000939	.	intron	0.082	.	2
AAAC.GTTT	5	86369160	86369177	4.5	ENSSSCT00000028915	.	intron	.	rs790634353	2
AAC.GTT	5	86401970	86401983	4.66667	ENSSSCT00000028915	.	intron	1.907	rs787480497	2
AAC.GTT	5	86401970	86401983	4.66667	ENSSSCT00000000941	.	intron	1.907	rs787480497	2
AAC.GTT	5	86401970	86401983	4.66667	ENSSSCT00000029378	U2	promoter	1.907	rs787480497	2
AG.CT	5	86424190	86424201	6	.	.	intergenic	.	226369 rs7874	4
AAAC.GTTT	5	86472143	86472157	3.75	ENSSSCT00000000943	CHPT1	intron	.	394772 rs7900	2
AAC.GTT	5	86472247	86472258	4	ENSSSCT00000000943	CHPT1	intron	.	rs707653662	2
AAAAC.GTTTT	5	86527346	86527367	4.4	ENSSSCT00000000945	MYBPC1	intron	.	rs789583802	3
AAAAC.GTTTT	5	86527346	86527367	4.4	ENSSSCT00000030865	.	intron	.	rs789583802	3
AAAG.CTTT	5	86570806	86570823	4.5	ENSSSCT00000000945	MYBPC1	intron	1.926	rs790668456	2
AAC.GTT	5	86602807	86602818	4	ENSSSCT00000000945	MYBPC1	intron	0.149	rs790101476	3
AC.GT	5	86688523	86688539	8.5	.	.	intergenic	.	.	4
AAC.GTT	5	86700801	86700813	4.33333	.	.	intergenic	.	rs787814525	3
AAAC.GTTT	5	86763936	86763948	3.25	.	.	intergenic	0.375	rs786247492	2
AATC.GATT	5	86772625	86772644	5	.	.	intergenic	0.162	968426 rs7907	2
AAAT.ATTT	5	86775082	86775110	7.25	.	.	intergenic	.	717248 rs7872	2
AAAC.GTTT	5	86784294	86784312	4.75	.	.	intergenic	0.233	382631 rs7887	2
AAAC.GTTT	5	86824214	86824232	4.75	ENSSSCT00000000949	UTP20	intron	-0.264	rs789181836	3
AAC.GTT	5	86862428	86862443	5.33333	ENSSSCT00000000949	UTP20	intron	-0.064	rs788414750	2
AT.AT	5	86937216	86937226	5.5	.	.	intergenic	.	.	2
AC.GT	5	87212135	87212152	9	ENSSSCT00000000956	ANO4	intron	0.027	rs790804608	2
AC.GT	5	87382682	87382703	11	.	.	intergenic	0.126	rs793787852	2
AAAC.GTTT	5	87434006	87434017	3	.	.	intergenic	0.451	rs787489961	2
AAT.ATT	5	87495988	87496001	4.66667	.	.	intergenic	0.081	rs789824405	2
AAG.CTT	5	87728622	87728638	5.66667	.	.	intergenic	-0.074	rs701341248	2
AT.AT	5	87741299	87741318	10	.	.	intergenic	0.04	790947 rs7912	2
AGATAT.ATATC	5	87743022	87743041	3.33333	.	.	intergenic	0.161	rs793614187	3
ACAT.ATGT	5	87746940	87746966	6.75	.	.	intergenic	.	547165 rs7931	2
AAAAT.ATTTT	5	87955042	87955060	3.8	.	.	intergenic	0.208	rs787671008	2
AT.AT	5	88009909	88009932	12	ENSSSCT00000028017	JHRF1BP1	intron	0.192	221515 rs7891	2
AT.AT	5	88009909	88009932	12	ENSSSCT00000000960	.	intron	0.192	221515 rs7891	2
AT.AT	5	88009909	88009932	12	ENSSSCT00000000963	.	intron	0.192	221515 rs7891	2
AAAT.ATTT	5	88495827	88495843	4.25	.	.	intergenic	0.363	.	2
AATG.CATT	5	88529401	88529419	4.75	ENSSSCT00000000964	.	intron	0.084	rs789115589	2
AAAAC.GTTTT	5	88653480	88653498	3.8	ENSSSCT00000000964	.	intron	-0.036	rs791420906	2
AAAAC.GTTTT	5	88924301	88924324	4.8	ENSSSCT00000000966	.	intron	0.137	295441 rs7938	3
AC.GT	5	89014202	89014212	5.5	ENSSSCT00000000966	.	intron	0.119	525853 rs7894	2
AAAG.CTTT	5	89122176	89122197	5.5	.	.	intergenic	0.23	rs790593233	2
AG.CT	5	89143955	89143970	8	.	.	intergenic	0.108	.	2
AAAC.GTTT	5	89148058	89148080	5.75	.	.	intergenic	.	720081 rs7898	2
AC.GT	5	89223538	89223548	5.5	.	.	intergenic	0.055	rs786450670	2
AT.AT	5	89253502	89253513	6	.	.	intergenic	.	rs793296685	2
AG.CT	5	89639968	89639979	6	.	.	intergenic	0.034	rs791776374	2
AAAC.GTTT	5	89643998	89644009	3	ENSSSCT00000000971	.	intron	1.216	rs786821587	2
AC.GT	5	89666104	89666115	6	ENSSSCT00000000971	.	intron	0.253	rs790384701	2
AC.GT	5	89882086	89882105	10	.	.	intergenic	.	rs790553117	2
AAT.ATT	5	90235614	90235637	8	.	.	intergenic	0.402	500606 rs7921	2
AG.CT	5	90239972	90239982	5.5	.	.	intergenic	-0.014	.	2
AAAG.CTTT	5	90276941	90276967	6.75	.	.	intergenic	0.618	.	2
AT.AT	5	90366564	90366573	5	.	.	intergenic	0.521	rs793425664	2
AC.GT	5	90497295	90497305	5.5	.	.	intergenic	-0.089	rs788127717	2
AAC.GTT	5	90503700	90503713	4.66667	.	.	intergenic	0.517	rs793695733	2
AAAT.ATTT	5	90524433	90524467	8.75	.	.	intergenic	0.191	rs792718994	2
AG.CT	5	90578666	90578675	5	.	.	intergenic	-0.044	rs792615175	2
AAAT.ATTT	5	90617058	90617076	4.75	.	.	intergenic	0.081	rs786429608	2
AC.GT	5	90699270	90699284	7.5	.	.	intergenic	-0.116	rs792364960	3
AAAC.GTTT	5	91044547	91044562	4	.	.	intergenic	0.106	.	2
AAAG.CTTT	5	91073892	91073909	4.5	.	.	intergenic	.	.	2
AAT.ATT	5	91130172	91130185	4.66667	.	.	intergenic	0.005	rs787361439	3
AAAT.ATTT	5	91140280	91140313	8.5	.	.	intergenic	0.087	.	2
AATG.CATT	5	91178665	91178691	6.75	.	.	intergenic	0.042	rs792812403	2
AC.GT	5	91206553	91206569	8.5	.	.	intergenic	.	rs787914436	2
AT.AT	5	91289788	91289809	11	ENSSSCT00000000974	NEDD1	promoter	0.217	.	2
AT.AT	5	91409669	91409679	5.5	.	.	intergenic	.	rs790224224	2
ACAT.ATGT	5	91544997	91545018	5.5	.	.	intergenic	.	rs791163655	2
AAAAC.GTTTT	5	91629314	91629336	4.6	.	.	intergenic	.	rs787188181	2
AAAT.ATTT	5	91833558	91833571	3.5	.	.	intergenic	.	rs786265279	2
AAAC.GTTT	5	91896256	91896288	8.25	ENSSSCT00000000980	NTN4	intron	0.834	754334 rs7868	2
AAAAG.CTTTT	5	91923719	91923739	4.2	ENSSSCT00000000980	NTN4	intron	.	rs786469481	4
AAGC.GCTT	5	91987110	91987137	7	.	.	intergenic	0.316	rs790002367	2
AAC.GTT	5	92133455	92133468	4.66667	.	.	intergenic	.	rs788937456	2
AAAT.ATTT	5	92177803	92177830	7	.	.	intergenic	.	965972 rs7901	2
AAAAC.GTTTT	5	92651889	92651918	6	ENSSSCT00000000989	NDUFA12	intron	0.267	430839 rs7878	2
AAAAC.GTTTT	5	92762822	92762842	4.2	.	.	intergenic	.	rs791602523	2
AT.AT	5	92775891	92775901	5.5	.	.	intergenic	0.388	rs791680818	2
AATG.CATT	5	92993788	92993810	5.75	.	.	intergenic	-0.438	rs791853385	2
AAAAC.GTTTT	5	93470079	93470098	4	ENSSSCT00000032383	CCDC41	intron	.	rs786348283	2
AAAAC.GTTTT	5	93470079	93470098	4	ENSSSCT00000024929	CCDC41	intron	.	rs786348283	2
AC.GT	5	93471864	93471883	10	ENSSSCT00000032383	CCDC41	intron	.	rs793749089	2
AC.GT	5	93471864	93471883	10	ENSSSCT00000024929	CCDC41	intron	.	rs793749089	2
AT.AT	5	93496857	93496867	5.5	ENSSSCT00000032383	CCDC41	intron	.	416225 rs7907	2
AT.AT	5	93496857	93496867	5.5	ENSSSCT00000024929	CCDC41	intron	.	416225 rs7907	2

AC.GT	5	93501766	93501778	6.5	ENSSSCT00000032383	CCDC41	intron	.	rs792519525	2
AC.GT	5	93501766	93501778	6.5	ENSSSCT00000024929	CCDC41	intron	.	rs792519525	2
AAAC.GTTT	5	93516028	93516042	3.75	ENSSSCT00000032383	CCDC41	intron	0.142	rs792607054	2
AAAC.GTTT	5	93516028	93516042	3.75	ENSSSCT00000024929	CCDC41	intron	0.142	rs792607054	2
ATC.GAT	5	93664443	93664464	7.33333	.	.	intergenic	0.289	rs792883074	2
AG.CT	5	93672477	93672487	5.5	.	.	intergenic	0.067	55201 rs7930	2
AAAT.ATTT	5	93724116	93724132	4.25	.	.	intergenic	0.503	518625 rs7938	2
AG.CT	5	93726409	93726418	5	.	.	intergenic	-0.047	484670 rs7922	2
AGATAT.ATATC	5	93825480	93825505	4.33333	.	.	intergenic	.	rs791592558	2
ATC.GAT	5	94027660	94027672	4.33333	.	.	intergenic	0.013	rs787407989	2
AAAAT.ATTTT	5	94331408	94331423	3.2	.	.	intergenic	.	rs787132705	2
AC.GT	5	94379160	94379174	7.5	.	.	intergenic	.	.	3
AG.CT	5	94678481	94678492	6	.	.	intergenic	0.139	rs791125648	2
AAG.CTT	5	94689673	94689689	5.66667	.	.	intergenic	0.761	rs792990085	2
AAAAT.ATTTT	5	94948818	94948834	3.4	.	.	intergenic	0.048	799768 rs7897	2
AGC.GCT	5	95015933	95015951	6.33333	.	.	intergenic	0.177	rs712914937	3
AAAAG.CTTTT	5	95549472	95549495	4.8	.	.	intergenic	0.328	448912 rs7898	2
AT.AT	5	95561483	95561500	9	.	.	intergenic	0.093	rs791093334	2
AAAC.GTTT	5	96153246	96153272	6.75	.	.	intergenic	.	rs791189165	2
AAAAT.ATTTT	5	96376547	96376562	3.2	.	.	intergenic	.	rs787972499	2
AAAC.GTTT	5	96478089	96478107	4.75	.	.	intergenic	.	rs786768232	4
AAAC.GTTT	5	96746480	96746494	3.75	.	.	intergenic	.	rs786628520	2
AC.GT	5	96758071	96758084	7	.	.	intergenic	0.044	.	2
AAT.ATT	5	96924500	96924529	10	.	.	intergenic	-0.093	.	2
AT.AT	5	97232768	97232802	17.5	.	.	intergenic	0.755	rs793437995	2
AG.CT	5	97291369	97291379	5.5	.	.	intergenic	.	.	3
AGC.GCT	5	97486328	97486348	7	.	.	intergenic	0.112	rs789068530	2
AG.CT	5	97849161	97849175	7.5	ENSSSCT00000001004	.	intron	.	rs786742080	2
AAT.ATT	5	97980247	97980258	4	.	.	intergenic	0.068	519913 rs7916	2
AAAAAC.GTTTTT	5	98127633	98127654	3.66667	.	.	intergenic	0.104	rs789887170	2
AAAAC.GTTTT	5	98136774	98136789	3.2	.	.	intergenic	0.414	.	2
ACAG.CTGT	5	98240590	98240612	5.75	.	.	intergenic	-0.089	rs788528592	2
AC.GT	5	98519794	98519808	7.5	.	.	intergenic	.	rs788477120	4
AG.CT	5	98579561	98579583	11.5	.	.	intergenic	0.036	rs788329719	2
AG.CT	5	98740771	98740781	5.5	.	.	intergenic	.	rs789800713	2
AAAC.GTTT	5	98986513	98986531	4.75	.	.	intergenic	0.084	.	4
AT.AT	5	99038056	99038067	6	ENSSSCT00000001012	TMTC3	intron	0.238	rs787082636	2
AAC.GTT	5	99184652	99184668	5.66667	ENSSSCT00000001009	C12orf50	intron	.	rs791776910	2
AAACC.GGTTT	5	99232719	99232742	4.8	.	.	intergenic	0.094	rs792408587	2
AG.CT	5	99252202	99252211	5	.	.	intergenic	.	.	3
AT.AT	5	99448667	99448677	5.5	.	.	intergenic	0.016	rs793140210	3
AAAC.GTTT	5	99543046	99543065	5	.	.	intergenic	.	rs792446462	2
AC.GT	5	99567114	99567124	5.5	.	.	intergenic	0.242	rs789490730	3
AAAC.GTTT	5	99635380	99635394	3.75	.	.	intergenic	0.217	rs791164280	2
AC.GT	5	99655523	99655532	5	.	.	intergenic	0.18	.	2
AAAAAT.ATTTTT	5	99739353	99739374	3.66667	.	.	intergenic	-0.311	156633 rs7898	2
AT.AT	5	99777775	99777785	5.5	.	.	intergenic	0.091	rs786751765	4
AG.CT	5	99781165	99781176	6	.	.	intergenic	.	rs786744983	2
AC.GT	5	99805667	99805682	8	.	.	intergenic	0.025	.	2
AAAC.GTTT	5	99835855	99835873	4.75	.	.	intergenic	0.408	rs788575706	2
AT.AT	5	100171373	100171394	11	.	.	intergenic	0.807	.	2
AG.CT	5	100405143	100405166	12	.	.	intergenic	.	rs790633921	2
AAT.ATT	5	100489003	100489027	8.33333	.	.	intergenic	.	rs791926296	2
AAAC.GTTT	5	100586993	100587011	4.75	.	.	intergenic	0.104	rs789592931	2
ACAT.ATGT	5	100597485	100597503	4.75	.	.	intergenic	.	152025 rs7868	2
AAAC.GTTT	5	100664482	100664518	9.25	.	.	intergenic	0.324	383014 rs7894	2
AAG.CTT	5	100670813	100670826	4.66667	.	.	intergenic	-0.003	rs787028025	2
AAAAC.GTTTT	5	100713210	100713225	3.2	.	.	intergenic	0.264	rs788748061	2
AC.GT	5	100831729	100831739	5.5	ENSSSCT00000001016	MGAT4C	intron	-0.098	.	2
AAT.ATT	5	100924308	100924321	4.66667	ENSSSCT00000001016	MGAT4C	intron	.	rs786986584	2
AAAAC.GTTTT	5	101229552	101229566	3	.	.	intergenic	0.17	rs791538043	2
AG.CT	5	101334939	101334951	6.5	.	.	intergenic	-0.239	368063 rs7908	2
AC.GT	5	101366158	101366178	10.5	.	.	intergenic	0.035	.	2
AAC.GTT	5	101382779	101382792	4.66667	.	.	intergenic	0.539	rs792371523	3
AAC.GTT	5	101581153	101581176	8	.	.	intergenic	0.143	rs792113919	2
AAAG.CTTT	5	101824015	101824026	3	.	.	intergenic	0.025	.	2
AATG.CATT	5	101836650	101836680	7.75	.	.	intergenic	0.017	rs790306807	2
AAAT.ATTT	5	102429164	102429178	3.75	.	.	intergenic	.	rs789910343	2
AAAT.ATTT	5	102500583	102500601	4.75	.	.	intergenic	.	rs788274717	2
AAC.GTT	5	102521978	102521997	6.66667	.	.	intergenic	0.006	rs787471661	2
AC.GT	5	102528401	102528413	6.5	.	.	intergenic	.	rs786707720	2
AAAG.CTTT	5	102760046	102760061	4	.	.	intergenic	0.047	336271 rs7887	2
AT.AT	5	102866686	102866696	5.5	.	.	intergenic	.	.	3
AC.GT	5	103066996	103067007	6	.	.	intergenic	-0.046	rs788166947	2
AC.GT	5	103089638	103089659	11	.	.	intergenic	-0.006	712421 rs7906	2
AAAT.ATTT	5	103102881	103102896	4	.	.	intergenic	.	182147 rs7931	2
AAAT.ATTT	5	103153372	103153391	5	.	.	intergenic	0.178	rs713230482	2
AAAAC.GTTTT	5	103165836	103165854	3.8	.	.	intergenic	0.092	rs787448293	2
AC.GT	5	103238932	103238943	6	.	.	intergenic	0.516	rs789520057	2
ACAT.ATGT	5	103340754	103340788	8.75	.	.	intergenic	.	.	2
AAAAAT.ATTTTT	5	103413916	103413937	3.66667	.	.	intergenic	0.268	rs791353114	2
AC.GT	5	103553044	103553055	6	ENSSSCT00000030219	.	intron	0.006	.	4
AAAAC.GTTTT	5	103633421	103633441	4.2	ENSSSCT00000030219	.	intron	0.395	976939 rs7931	2
AAAC.GTTT	5	103640993	103641012	5	ENSSSCT00000030219	.	intron	0.043	704214 rs7914	3

AC.GT	5	103844708	103844718	5.5	ENSSSCT00000029091	.	intron	0.014	rs792339741	2
AAAT.ATTT	5	103853189	103853203	3.75	ENSSSCT00000029091	.	intron	0.314	538279 rs7914	2
AC.GT	5	104481681	104481690	5	.	.	intergenic	0.041	.	2
AT.AT	5	104651246	104651258	6.5	.	.	intergenic	0.036	081602 rs7873	2
AAAC.GTTT	5	104709317	104709331	3.75	.	.	intergenic	0.007	701516 rs7926	2
AAAC.GTTT	5	104710553	104710578	6.5	.	.	intergenic	-0.043	rs789579235	2
AG.CT	5	105124433	105124443	5.5	ENSSSCT00000001028	PPFIA2	intron	-0.011	rs792273119	2
AGG.CCT	5	105403210	105403236	9	.	.	intergenic	0.062	464875 rs7862	2
AAC.GTT	5	105635855	105635876	7.33333	.	.	intergenic	0.046	rs787854262	2
AT.AT	5	105694646	105694655	5	.	.	intergenic	.	.	3
AAGC.GCTT	5	105817925	105817939	3.75	.	.	intergenic	0.109	.	2
AAAAC.GTTTT	5	105877225	105877247	4.6	ENSSSCT00000001032	OTOGL	intron	.	522441 rs7901	2
AC.GT	5	105934008	105934027	10	ENSSSCT00000001032	OTOGL	intron	1.535	253799 rs7904	2
AATG.CATT	5	106040257	106040272	4	.	.	intergenic	0.151	rs792452297	2
AC.GT	5	106561427	106561441	7.5	.	.	intergenic	-0.146	rs787288510	5
AAC.GTT	5	106608966	106608979	4.66667	ENSSSCT00000028012	PPP1R12A	intron	0.066	rs790441227	2
AGC.GCT	5	106922442	106922461	6.66667	ENSSSCT00000001037	SYT1	intron	0.276	592860 rs7903	2
AAAC.GTTT	5	107240790	107240804	3.75	.	.	intergenic	-0.036	rs792153024	2
AC.GT	5	107321289	107321304	8	.	.	intergenic	-0.034	777334 rs7888	2
AAG.CTT	5	107327869	107327890	7.33333	.	.	intergenic	0.114	rs704144022	2
ACAT.ATGT	5	107332071	107332087	4.25	.	.	intergenic	0.306	rs786450405	2
AAAT.ATTT	5	107332126	107332138	3.25	.	.	intergenic	0.281	.	2
AAAC.GTTT	5	107749297	107749319	5.75	.	.	intergenic	.	925948 rs7930	2
AAC.GTT	5	107752512	107752526	5	.	.	intergenic	.	rs789608839	3
ACATAT.ATATG	5	107789606	107789624	3.16667	.	.	intergenic	0.058	490061 rs7909	2
AC.GT	5	107953362	107953376	7.5	.	.	intergenic	-0.047	rs788680210	2
AAAAC.GTTTT	5	108084154	108084177	4.8	.	.	intergenic	0.025	926866 rs7895	2
AAAT.ATTT	5	108163036	108163051	4	.	.	intergenic	0.37	004751 rs7936	2
AAAC.GTTT	5	108215626	108215643	4.5	ENSSSCT00000029790	.	intron	0.025	rs786429846	2
AAAC.GTTT	5	108215626	108215643	4.5	ENSSSCT00000026141	.	intron	0.025	rs786429846	2
AAT.ATT	5	108228264	108228278	5	ENSSSCT00000029790	.	intron	0.043	.	2
AAT.ATT	5	108228264	108228278	5	ENSSSCT00000026141	.	intron	0.043	.	2
AAAC.GTTT	5	108316282	108316297	4	.	.	intergenic	-0.143	rs786697709	2
AAAC.GTTT	5	108428668	108428685	4.5	.	.	intergenic	-0.18	558384 rs7918	2
AG.CT	5	108551564	108551574	5.5	.	.	intergenic	-0.337	rs787444513	3
AC.GT	5	108700631	108700649	9.5	.	.	intergenic	1.184	.	2
AAAC.GTTT	5	108752416	108752448	8.25	.	.	intergenic	0.47	786650 rs7918	3
AT.AT	5	108839106	108839115	5	.	.	intergenic	.	rs793366402	2
AC.GT	5	108933984	108934003	10	.	.	intergenic	0.117	.	3
AAAAG.CTTTT	5	108953723	108953743	4.2	.	.	intergenic	0.444	rs791147373	2
AAAAC.GTTTT	5	109230938	109230963	5.2	.	.	intergenic	0.146	rs789009525	2
ACAG.CTGT	5	109629922	109629947	6.5	.	.	intergenic	0.149	rs793288320	2
AAT.ATT	5	110140135	110140150	5.33333	.	.	intergenic	0.089	.	2
AT.AT	5	111124216	111124228	6.5	.	.	intergenic	.	rs791670373	2
AAAG.CTTT	5	111267203	111267225	5.75	.	.	intergenic	.	rs790388463	2
AC.GT	5	111336563	111336580	9	.	.	intergenic	-0.335	.	2
AAC.GTT	6	378111	378123	4.33333	ENSSSCT00000031647	FANCA	promoter	.	rs792311531	2
CCCG.CGGG	6	871151	871172	5.5	ENSSSCT00000026558	sc-mir-433	promoter	.	.	2
AAC.GTT	6	950394	950418	8.33333	.	.	intergenic	.	447685 rs7919	2
AC.GT	6	1808171	1808182	6	ENSSSCT00000002944	JPH3	intron	.	rs786537211	4
AC.GT	6	2090337	2090359	11.5	ENSSSCT00000002948	C16orf95	intron	.	.	2
AC.GT	6	2096963	2096972	5	ENSSSCT00000002948	C16orf95	intron	.	.	4
AAAG.CTTT	6	2153902	2153933	8	.	.	intergenic	.	.	2
AAT.ATT	6	2774126	2774139	4.66667	ENSSSCT00000002951	MTHFSD	intron	0.083	rs788889925	2
AAC.GTT	6	5362612	5362634	7.66667	ENSSSCT00000002977	CDH13	intron	0.013	rs789835452	2
AT.AT	6	5365210	5365223	7	ENSSSCT00000002977	CDH13	promoter	-0.167	734637 rs7928	2
AAAT.ATTT	6	5372422	5372434	3.25	.	.	intergenic	0.23	rs786459183	2
AG.CT	6	5478002	5478018	8.5	.	.	intergenic	-0.272	541981 rs7926	2
AGGG.CCCT	6	5516330	5516346	4.25	.	.	intergenic	-0.08	rs788017161	2
AT.AT	6	5609288	5609298	5.5	ENSSSCT00000031996	.	intron	0.483	rs787469867	2
AG.CT	6	5625620	5625631	6	ENSSSCT00000031996	.	intron	-0.005	.	2
AC.GT	6	5723333	5723349	8.5	ENSSSCT00000031996	.	intron	-0.404	rs791377347	4
AC.GT	6	6037174	6037192	9.5	.	.	intergenic	.	rs787796378	2
AAAAC.GTTTT	6	6225985	6226010	5.2	.	.	intergenic	-0.215	.	2
AC.GT	6	6724703	6724719	8.5	.	.	intergenic	0.506	rs787628292	2
AAAC.GTTT	6	6746198	6746215	4.5	.	.	intergenic	0.03	rs788833230	2
ACC.GGT	6	7120597	7120614	6	.	.	intergenic	.	rs792010823	2
AC.GT	6	7254719	7254730	6	ENSSSCT00000002982	CMIP	intron	-0.498	745519 rs7893	3
AC.GT	6	7255807	7255833	13.5	ENSSSCT00000002982	CMIP	intron	-0.065	rs787202640	4
AAT.ATT	6	7423480	7423497	6	.	.	intergenic	.	.	2
AAG.CTT	6	7456080	7456093	4.66667	.	.	intergenic	-0.237	rs788634545	2
AAAT.ATTT	6	7628139	7628156	4.5	.	.	intergenic	-0.089	rs791685293	2
AAC.GTT	6	8224735	8224746	4	.	.	intergenic	-0.18	.	2
AC.GT	6	8238221	8238233	6.5	.	.	intergenic	-0.266	rs793656766	4
AC.GT	6	8303238	8303257	10	.	.	intergenic	-0.099	rs793458584	2
AAAC.GTTT	6	8581625	8581644	5	.	.	intergenic	1.357	rs789251516	2
AAAAC.GTTTT	6	9241129	9241151	4.6	.	.	intergenic	-0.212	rs791592969	2
AAAC.GTTT	6	9265295	9265308	3.5	.	.	intergenic	-0.055	rs793372061	2
AT.AT	6	9335214	9335224	5.5	.	.	intergenic	0.517	.	3
AAAT.ATTT	6	9453824	9453850	6.75	.	.	intergenic	-0.102	130217 rs7938	3
AC.GT	6	9463270	9463294	12.5	.	.	intergenic	-0.04	532942 rs7886	3
AAAG.CTTT	6	9473982	9473996	3.75	.	.	intergenic	0.638	rs788625446	2
ACGG.CCGT	6	9779838	9779854	4.25	.	.	intergenic	0.055	rs789563233	2
AAAC.GTTT	6	10319287	10319301	3.75	.	.	intergenic	-0.166	rs786727791	2

AC.GT	6	10652123	10652139	8.5	.	.	intergenic	-0.179	rs787288479	3
AC.GT	6	10780709	10780727	9.5	.	.	intergenic	0.008	.	5
AAAT.ATTT	6	10799819	10799833	3.75	.	.	intergenic	.	.	2
AAAC.GTTT	6	10932857	10932873	4.25	.	.	intergenic	-0.121	rs786943940	2
AG.CT	6	11175333	11175345	6.5	ENSSSCT00000032493	.	intron	0.218	rs791152904	2
AC.GT	6	11217572	11217598	13.5	ENSSSCT00000032493	.	intron	0.034	rs791636009	2
AAAAAT.ATTTTT	6	11271803	11271832	5	ENSSSCT00000032493	.	intron	.	099013 rs7883	2
AT.AT	6	11277409	11277420	6	ENSSSCT00000032493	.	intron	.	.	3
AAAAC.GTTTT	6	11516966	11516984	3.8	.	.	intergenic	.	rs793193992	2
AAC.GTT	6	11594402	11594418	5.66667	.	.	intergenic	.	993031 rs7870	2
AAAT.ATTT	6	11613189	11613209	5.25	.	.	intergenic	0.339	rs791754170	2
AC.GT	6	11663713	11663733	10.5	.	.	intergenic	.	rs789273733	4
AT.AT	6	11793005	11793035	15.5	.	.	intergenic	0.53	rs789115220	2
AAAC.GTTT	6	11852390	11852412	5.75	.	.	intergenic	.	rs786391027	2
AT.AT	6	11975349	11975358	5	ENSSSCT00000003001	GABARAPL	promoter	0.141	.	2
AAAC.GTTT	6	11988354	11988374	5.25	ENSSSCT00000003002	.	intron	.	.	2
AAAT.ATTT	6	12287445	12287472	7	.	.	intergenic	.	rs793138834	2
AGG.CCT	6	12430547	12430561	5	.	.	intergenic	0.836	rs793718079	2
AC.GT	6	12472449	12472463	7.5	.	.	intergenic	.	rs788706545	3
AAAC.GTTT	6	12603088	12603108	5.25	.	.	intergenic	0.216	rs789505479	2
AAAC.GTTT	6	12654044	12654063	5	ENSSSCT00000028190	.	intron	0.041	rs788701712	2
AG.CT	6	12835665	12835682	9	ENSSSCT00000003012	FA2H	intron	-0.215	519668 rs7901	2
AAAC.GTTT	6	12863975	12863995	5.25	.	.	intergenic	.	rs786733053	2
AAAT.ATTT	6	13005970	13005988	4.75	ENSSSCT00000030720	GLG1	intron	.	rs788610045	2
AAC.GTT	6	13041104	13041120	5.66667	ENSSSCT00000030720	GLG1	intron	.	rs788234911	2
AAAAC.GTTTT	6	13248535	13248558	4.8	ENSSSCT00000003016	.	intron	.	rs787384955	2
AC.GT	6	13369712	13369739	14	ENSSSCT00000003017	AARS	intron	-0.399	414065 rs7868	3
AAG.CTT	6	13626375	13626393	6.33333	.	.	intergenic	.	rs793078644	2
AG.CT	6	13639120	13639131	6	.	.	intergenic	-0.011	rs789296301	2
AC.GT	6	13685755	13685764	5	.	.	intergenic	-0.221	rs791768728	2
AAAC.GTTT	6	13686298	13686312	3.75	.	.	intergenic	.	rs787861254	2
AT.AT	6	13791107	13791119	6.5	ENSSSCT00000003024	.	intron	.	rs790429271	2
AAAT.ATTT	6	13882584	13882601	4.5	.	.	intergenic	0.064	242146 rs7891	2
AGATAT.ATATCT	6	13979674	13979703	5	ENSSSCT00000003034	ZNF23	promoter	.	rs786691656	2
AAGG.CCTT	6	14406477	14406495	4.75	.	.	intergenic	.	163218 rs7900	2
AAAT.ATTT	6	14737527	14737549	5.75	.	.	intergenic	0.147	rs792698507	2
AT.AT	6	14767978	14767992	7.5	.	.	intergenic	.	.	3
AAC.GTT	6	14949942	14949956	5	.	.	intergenic	.	561033 rs7917	2
AT.AT	6	15002446	15002462	8.5	.	.	intergenic	0.022	.	2
AC.GT	6	15183552	15183565	7	.	.	intergenic	-0.008	rs787695299	4
AAAT.ATTT	6	15664397	15664411	3.75	.	.	intergenic	0.228	rs790653835	2
AC.GT	6	15772699	15772723	12.5	.	.	intergenic	0.845	.	3
AG.CT	6	15783251	15783269	9.5	.	.	intergenic	-0.025	rs792829310	2
AT.AT	6	15853618	15853629	6	.	.	intergenic	.	rs786775775	2
AT.AT	6	15866361	15866371	5.5	.	.	intergenic	0.032	rs691475087	2
AG.CT	6	15874807	15874831	12.5	.	.	intergenic	1.078	rs786210449	2
AC.GT	6	15875040	15875057	9	.	.	intergenic	1.534	rs791872566	3
AAAAAG.CTTTTT	6	15931495	15931520	4.33333	.	.	intergenic	0.197	rs790180018	2
AAAAC.GTTTT	6	15991205	15991223	3.8	.	.	intergenic	-0.03	rs789437113	2
AC.GT	6	16006098	16006109	6	.	.	intergenic	0.051	rs786447286	2
AG.CT	6	16021914	16021924	5.5	.	.	intergenic	0.006	.	2
AC.GT	6	16057764	16057773	5	.	.	intergenic	0.026	.	2
AC.GT	6	16063969	16063982	7	.	.	intergenic	0.611	rs786227279	2
AAC.GTT	6	16235596	16235609	4.66667	.	.	intergenic	.	rs791615076	2
AAAAT.ATTTT	6	16541868	16541891	4.8	.	.	intergenic	.	rs787947947	2
AT.AT	6	16618840	16618851	6	.	.	intergenic	.	rs787942805	2
AT.AT	6	17023272	17023287	8	.	.	intergenic	.	.	4
AG.CT	6	17203467	17203477	5.5	ENSSSCT00000003119	ARL2BP	intron	1.921	905991 rs7886	2
AG.CT	6	17235162	17235171	5	.	.	intergenic	0.21	rs790330890	2
AC.GT	6	17301766	17301787	11	.	.	intergenic	-0.084	.	4
AAAC.GTTT	6	17318233	17318257	6.25	.	.	intergenic	-0.275	rs793834986	2
AC.GT	6	17828278	17828287	5	ENSSSCT00000003111	CNGB1	intron	0.982	rs787124819	3
AAAC.GTTT	6	17956378	17956409	8	ENSSSCT00000003106	MMP15	intron	-0.015	rs786570868	2
AC.GT	6	18061804	18061813	5	ENSSSCT00000003104	CSNK2A2	intron	-0.072	.	2
AC.GT	6	18133876	18133895	10	ENSSSCT00000003103	.	intron	.	rs791530019	2
ACTC.GAGT	6	18276711	18276732	5.5	.	.	intergenic	-0.008	503650 rs7887	2
ATCC.GGAT	6	18280224	18280252	7.25	.	.	intergenic	0.093	744926 rs7917	3
AAAT.ATTT	6	18384609	18384622	3.5	.	.	intergenic	.	rs790307644	2
AAAC.GTTT	6	18401161	18401182	5.5	.	.	intergenic	.	rs792345757	2
AAAC.GTTT	6	18418121	18418148	7	ENSSSCT00000023952	CNOT1	intron	0.14	rs787328756	2
AAAC.GTTT	6	18418121	18418148	7	ENSSSCT00000003099	CNOT1	intron	0.14	rs787328756	2
AAAG.CTTT	6	18525393	18525412	5	.	.	intergenic	0.163	343443 rs7865	3
AT.AT	6	18715904	18715916	6.5	.	.	intergenic	.	021876 rs7928	2
AAC.GTT	6	18798448	18798463	5.33333	.	.	intergenic	.	rs789663312	2
AAAT.ATTT	6	18850230	18850261	8	.	.	intergenic	0.162	rs788451449	2
AC.GT	6	19005040	19005061	11	.	.	intergenic	0.059	.	2
AAAC.GTTT	6	19010162	19010176	3.75	.	.	intergenic	0.229	344434 rs7864	2
ACAT.ATGT	6	19189278	19189293	4	.	.	intergenic	0.019	299639 rs7925	2
AAC.GTT	6	19249501	19249519	6.33333	.	.	intergenic	.	380512 rs6947	3
ACC.GGT	6	19541590	19541602	4.33333	.	.	intergenic	-0.09	.	2
AAAAC.GTTTT	6	19636054	19636077	4.8	.	.	intergenic	.	rs789700371	2
AC.GT	6	19783751	19783766	8	.	.	intergenic	-0.079	.	3
AAT.ATT	6	20010193	20010209	5.66667	.	.	intergenic	.	rs696368341	2
AC.GT	6	20544654	20544664	5.5	.	.	intergenic	-0.131	.	4

AGAT.ATCT	6	20566193	20566224	8	.	.	intergenic	.	rs789497309	2
AAAT.ATTT	6	20865349	20865368	5	.	.	intergenic	-0.16	rs790471246	2
AC.GT	6	20973058	20973068	5.5	.	.	intergenic	.	rs786609071	3
AAT.ATT	6	21239921	21239956	12	.	.	intergenic	0.222	.	2
AAC.GTT	6	21366631	21366654	8	.	.	intergenic	-0.249	939505 rs7912	2
ACAG.CTGT	6	21488229	21488246	4.5	.	.	intergenic	.	rs788960983	2
AC.GT	6	21504952	21504966	7.5	.	.	intergenic	-0.279	700475 rs7890	3
AG.CT	6	21594205	21594215	5.5	.	.	intergenic	0.509	353340 rs7934	2
AAC.GTT	6	21618019	21618033	5	.	.	intergenic	0.093	.	2
AT.AT	6	21624146	21624157	6	.	.	intergenic	0.155	rs791411398	2
ACT.AGT	6	21723169	21723187	6.33333	.	.	intergenic	0.047	189309 rs7938	2
AAAC.GTTT	6	21723577	21723597	5.25	.	.	intergenic	.	520342 rs7887	2
AAC.GTT	6	21833661	21833680	6.66667	.	.	intergenic	0.053	.	2
AG.CT	6	21840078	21840094	8.5	.	.	intergenic	.	.	2
AAC.GTT	6	21870140	21870152	4.33333	.	.	intergenic	-0.21	311581 rs7933	2
AAAC.GTTT	6	21901151	21901167	4.25	.	.	intergenic	0.007	rs790516338	2
AAAC.GTTT	6	22054085	22054099	3.75	.	.	intergenic	0.091	rs792824711	2
AG.CT	6	22132905	22132916	6	.	.	intergenic	-0.192	rs793604302	3
AC.GT	6	22556037	22556059	11.5	.	.	intergenic	0.313	rs793820021	2
AAAC.GTTT	6	22592472	22592495	6	.	.	intergenic	-0.002	301225 rs7872	2
AAAAT.ATTTT	6	22621776	22621792	3.4	.	.	intergenic	-0.114	rs790262664	2
AC.GT	6	22755687	22755700	7	.	.	intergenic	-0.092	568226 rs7894	2
AC.GT	6	22821417	22821428	6	.	.	intergenic	0.281	rs789401165	2
AT.AT	6	22905956	22905968	6.5	.	.	intergenic	0.729	595789 rs7927	4
AC.GT	6	23184335	23184344	5	.	.	intergenic	0.513	.	2
AC.GT	6	23478550	23478560	5.5	.	.	intergenic	-0.185	567669 rs7928	2
AAC.GTT	6	23731863	23731885	7.66667	ENSSSCT00000035974	CDH11	intron	-0.033	.	2
AAC.GTT	6	23731863	23731885	7.66667	ENSSSCT00000033754	CDH11	intron	-0.033	.	2
AAC.GTT	6	23731863	23731885	7.66667	ENSSSCT00000003095	CDH11	intron	-0.033	.	2
AAC.GTT	6	23731863	23731885	7.66667	ENSSSCT00000033473	CDH11	intron	-0.033	.	2
AC.GT	6	23747861	23747872	6	ENSSSCT00000035974	CDH11	intron	-0.26	.	2
AC.GT	6	23747861	23747872	6	ENSSSCT00000033754	CDH11	intron	-0.26	.	2
AC.GT	6	23747861	23747872	6	ENSSSCT00000003095	CDH11	intron	-0.26	.	2
AC.GT	6	23747861	23747872	6	ENSSSCT00000033473	CDH11	intron	-0.26	.	2
AC.GT	6	23747861	23747872	6	ENSSSCT00000032635	CDH11	intron	-0.26	.	2
AAAT.ATTT	6	23838643	23838668	6.5	.	.	intergenic	0.097	063017 rs7927	2
AAT.ATT	6	23839088	23839116	9.66667	.	.	intergenic	-0.191	rs790535487	2
AAAC.GTTT	6	23973436	23973456	5.25	.	.	intergenic	-0.06	333689 rs7862	2
ACC.GGT	6	23993779	23993791	4.33333	.	.	intergenic	-0.054	rs792457477	2
AATG.CATT	6	24042330	24042344	3.75	.	.	intergenic	0.246	.	2
AT.AT	6	24180259	24180268	5	.	.	intergenic	0.535	rs792971688	3
AG.CT	6	24232623	24232632	5	.	.	intergenic	-0.095	.	2
AG.CT	6	24406628	24406639	6	.	.	intergenic	0.072	rs791963968	2
AC.GT	6	24413402	24413414	6.5	.	.	intergenic	0.073	rs792178891	3
AAAC.GTTT	6	24731853	24731876	6	.	.	intergenic	0.098	305410 rs7896	2
AGGG.CCCT	6	24928145	24928162	4.5	.	.	intergenic	-0.005	rs790739864	2
AAAC.GTTT	6	25416202	25416213	3	.	.	intergenic	.	rs786443610	2
AAAAAC.GTTTTT	6	26171879	26171900	3.66667	ENSSSCT00000003062	SMPD3	intron	0.169	rs789498524	2
AC.GT	6	26491622	26491631	5	.	.	intergenic	.	.	2
AG.CT	6	26707227	26707237	5.5	.	.	intergenic	0.263	rs787237475	2
AAAT.ATTT	6	27450864	27450882	4.75	.	.	intergenic	-0.071	rs791477237	2
AAAAG.CTTTT	6	27501712	27501732	4.2	ENSSSCT00000003129	LPCAT2	intron	-0.217	rs787050448	2
AGC.GCT	6	27674204	27674219	5.33333	.	.	intergenic	0.503	240471 rs7905	2
AG.CT	6	27676335	27676351	8.5	.	.	intergenic	0.658	403568 rs7921	2
AG.CT	6	27696995	27697015	10.5	.	.	intergenic	0.272	186332 rs7877	2
AAAC.GTTT	6	28028926	28028947	5.5	.	.	intergenic	0.018	rs786872448	2
AAT.ATT	6	28260408	28260424	5.66667	.	.	intergenic	1.366	.	2
AAC.GTT	6	28401362	28401378	5.66667	.	.	intergenic	1.221	rs788920971	2
AAAC.GTTT	6	28525459	28525490	8	.	.	intergenic	-0.121	353611 rs7893	2
AAAAC.GTTTT	6	28775281	28775300	4	.	.	intergenic	0.047	.	2
AT.AT	6	28878339	28878350	6	.	.	intergenic	0.149	rs791418990	2
ATCC.GGAT	6	28903119	28903151	8.25	.	.	intergenic	-0.145	508521 rs7871	2
AAG.CTT	6	29273069	29273080	4	.	.	intergenic	.	726750 rs7916	2
AAAG.CTTT	6	29403535	29403558	6	ENSSSCT00000003136	TOX3	intron	-0.18	.	2
AC.GT	6	29755746	29755758	6.5	.	.	intergenic	-0.189	rs787467010	4
AC.GT	6	29781243	29781256	7	.	.	intergenic	0.113	rs787826106	2
AT.AT	6	29938514	29938524	5.5	.	.	intergenic	-0.25	rs789889385	2
AC.GT	6	30085584	30085594	5.5	.	.	intergenic	.	.	2
AG.CT	6	30202809	30202825	8.5	.	.	intergenic	0.065	rs786701782	2
AAAAC.GTTTT	6	30254966	30254985	4	.	.	intergenic	0.083	rs788829139	4
AC.GT	6	30264054	30264072	9.5	.	.	intergenic	1.231	.	4
AC.GT	6	30272965	30272978	7	.	.	intergenic	0.021	rs789844702	2
AG.CT	6	30331666	30331677	6	.	.	intergenic	0.004	590848 rs7883	3
AAAAT.ATTTT	6	30809995	30810014	4	.	.	intergenic	.	rs793492673	2
AC.GT	6	30964673	30964689	8.5	.	.	intergenic	0.093	335663 rs7888	2
AAAC.GTTT	6	31241391	31241409	4.75	.	.	intergenic	-0.125	rs788439298	2
AAAC.GTTT	6	31372949	31372962	3.5	.	.	intergenic	-0.214	rs788453390	2
AAAC.GTTT	6	31635931	31635945	3.75	.	.	intergenic	.	rs788227585	2
AAAC.GTTT	6	31665852	31665881	7.5	.	.	intergenic	.	198211 rs7888	2
AAAC.GTTT	6	31695255	31695277	5.75	.	.	intergenic	-0.027	445711 rs7883	2
ATC.GAT	6	31987264	31987277	4.66667	.	.	intergenic	-0.022	rs790433018	2
AG.CT	6	32230271	32230281	5.5	ENSSSCT00000003145	PHKB	intron	0.317	rs790108291	4
AAAAG.CTTTT	6	32347599	32347616	3.6	ENSSSCT00000003145	PHKB	intron	.	rs791989465	2
AG.CT	6	32459667	32459678	6	.	.	intergenic	0.277	rs792166104	2

AAAC.GTTT	6	32685355	32685370	4	.	.	intergenic	-0.298	rs788917956	2
AGAGG.CCTCT	6	32727017	32727037	4.2	ENSSSCT00000030872	.	intron	.	rs787509508	2
AAAC.GTTT	6	32816287	32816314	7	.	.	intergenic	.	rs791343785	2
AAC.GTT	6	32899066	32899082	5.66667	ENSSSCT00000003148	GPT2	intron	-0.177	rs793340720	2
AAAT.ATTT	6	32950733	32950751	4.75	.	.	intergenic	0.146	rs786402809	2
AAAC.GTTT	6	33302901	33302923	5.75	.	.	intergenic	.	rs793783039	3
AAAG.CTTT	6	33338749	33338772	6	.	.	intergenic	.	rs792470813	2
AAC.GTT	6	33709343	33709361	6.33333	.	.	intergenic	.	rs787326876	2
AAT.ATT	6	33809784	33809808	8.33333	.	.	intergenic	.	rs791423199	2
AAAC.GTTT	6	34262759	34262775	4.25	.	.	intergenic	-0.296	)63157 rs7905	2
AAAC.GTTT	6	34442993	34443015	5.75	.	.	intergenic	0.138	rs792778222	2
AT.AT	6	34500560	34500574	7.5	.	.	intergenic	.	rs789338429	2
ACAT.ATGT	6	34844453	34844475	5.75	ENSSSCT00000020081	U6	promoter	.	rs790978426	4
AC.GT	6	34934289	34934299	5.5	.	.	intergenic	-0.031	rs786900842	3
\AAAAC.GTTTT	6	35030574	35030596	3.83333	ENSSSCT00000003155	POP4	intron	-0.099	rs793303126	2
AT.AT	6	35273611	35273623	6.5	.	.	intergenic	.	rs792521501	2
AT.AT	6	35274702	35274713	6	.	.	intergenic	.	rs788998404	2
AAAC.GTTT	6	35499672	35499689	4.5	.	.	intergenic	.	rs787905383	2
AC.GT	6	35560955	35560987	16.5	.	.	intergenic	-0.615	.	2
AAT.ATT	6	35597340	35597361	7.33333	.	.	intergenic	-0.153	393751 rs7934	2
AC.GT	6	36034652	36034669	9	ENSSSCT00000026089	ZNF536	intron	.	rs786423523	2
AC.GT	6	36034652	36034669	9	ENSSSCT00000024971	ZNF536	intron	.	rs786423523	2
AT.AT	6	36090836	36090846	5.5	.	.	intergenic	-0.124	289042 rs7931	2
AC.GT	6	36126944	36126968	12.5	.	.	intergenic	-0.231	rs792414955	2
AG.CT	6	36507229	36507256	14	.	.	intergenic	1.227	rs786325930	2
AC.GT	6	36577793	36577821	14.5	.	.	intergenic	-0.341	rs793744013	2
AC.GT	6	36602247	36602266	10	.	.	intergenic	0.006	rs708510675	2
AC.GT	6	36943987	36943998	6	.	.	intergenic	0.286	480484 rs7900	2
AAG.CTT	6	36984349	36984369	7	.	.	intergenic	-0.101	rs712598239	2
AG.CT	6	37173933	37173943	5.5	.	.	intergenic	-0.03	.	2
AAAC.GTTT	6	37328119	37328143	6.25	.	.	intergenic	-0.094	rs791769972	2
ACAT.ATGT	6	37365233	37365244	3	.	.	intergenic	.	590415 rs7876	2
AG.CT	6	37625256	37625266	5.5	.	.	intergenic	0.032	rs790856259	2
AGG.CCT	6	37702857	37702870	4.66667	.	.	intergenic	0.1	.	2
AAAAT.ATTTT	6	37904687	37904707	4.2	ENSSSCT00000025063	DPY19L3	intron	0.108	rs791759180	2
AC.GT	6	37917202	37917226	12.5	ENSSSCT00000025063	DPY19L3	intron	-0.043	rs788596417	2
AC.GT	6	37928379	37928389	5.5	.	.	intergenic	0.351	rs791081247	2
AAC.GTT	6	37981850	37981861	4	.	.	intergenic	.	.	2
AAAC.GTTT	6	38120278	38120290	3.25	ENSSSCT00000003160	ANKRD27	intron	-0.064	rs792893252	2
AAAC.GTTT	6	38120278	38120290	3.25	ENSSSCT00000027536	SLC7A9	intron	-0.064	rs792893252	2
AC.GT	6	38422325	38422336	6	.	.	intergenic	.	rs791224766	2
AAAAC.GTTTT	6	39122749	39122777	5.8	.	.	intergenic	0.298	717506 rs7888	2
AAAC.GTTT	6	39239710	39239722	3.25	ENSSSCT00000021685	U6	promoter	.	rs786655930	2
AAAAT.ATTTT	6	39713751	39713773	4.6	.	.	intergenic	.	rs786721621	2
AT.AT	6	39721897	39721906	5	.	.	intergenic	.	rs788329548	2
AAAC.GTTT	6	40712254	40712266	3.25	ENSSSCT00000003224	TYROBP	intron	0.369	593549 rs7916	2
AC.GT	6	40861068	40861080	6.5	ENSSSCT00000003228	WDR62	intron	0.376	.	3
AAAC.GTTT	6	40978346	40978362	4.25	.	.	intergenic	.	131190 rs7911	2
\AAAAT.ATTTTT	6	41035593	41035615	3.83333	.	.	intergenic	.	rs792075771	2
AC.GT	6	41053266	41053282	8.5	ENSSSCT00000003238	ZNF565	intron	.	rs790278762	4
AC.GT	6	41123837	41123849	6.5	.	.	intergenic	.	rs786837587	2
AAAG.CTTT	6	41133765	41133783	4.75	.	.	intergenic	.	rs788159625	2
AAAT.ATTT	6	41178688	41178711	6	ENSSSCT00000003245	ZNF567	intron	.	.	2
AAC.GTT	6	41199282	41199301	6.66667	ENSSSCT00000003241	ZNF461	intron	0.259	715081 rs7865	2
AAAAT.ATTTT	6	41291053	41291087	7	.	.	intergenic	.	rs793711100	2
AAT.ATT	6	41297238	41297255	6	.	.	intergenic	.	374988 rs7890	2
\AAAAC.GTTTT	6	41310825	41310852	4.66667	.	.	intergenic	0.34	291429 rs7916	2
\AAAAC.GTTTT	6	41336715	41336742	4.66667	.	.	intergenic	.	rs787248368	2
AAAC.GTTT	6	41711908	41711922	3.75	ENSSSCT00000022792	ZNF383	3'utr	0.294	rs789944365	2
AAAT.ATTT	6	41915086	41915106	5.25	ENSSSCT00000003259	.	intron	0.1	rs792679882	2
AAAC.GTTT	6	42070101	42070115	3.75	.	.	intergenic	.	rs792871378	2
AG.CT	6	42941955	42941964	5	ENSSSCT00000003285	RYR1	intron	0.522	rs790396864	3
AC.GT	6	43427740	43427750	5.5	ENSSSCT00000003296	.	intron	.	rs792255101	2
AC.GT	6	43489063	43489073	5.5	.	.	intergenic	.	rs793303830	2
AAAC.GTTT	6	43930510	43930528	4.75	.	.	intergenic	.	369937 rs7865	2
AG.CT	6	44532553	44532569	8.5	.	.	intergenic	.	rs788377739	2
AC.GT	6	45609003	45609016	7	ENSSSCT00000003371	GSK3A	intron	0.045	.	3
AAT.ATT	6	46096117	46096136	6.66667	.	.	intergenic	.	403545 rs7921	2
AAAC.GTTT	6	46301565	46301583	4.75	ENSSSCT00000003398	CADM4	intron	-0.185	)18658 rs7908	2
AC.GT	6	46457368	46457380	6.5	.	.	intergenic	-0.364	986882 rs7921	2
AT.AT	6	46532109	46532118	5	.	.	intergenic	.	.	3
AAAC.GTTT	6	47424545	47424572	7	ENSSSCT00000003432	SYMPK	intron	.	975746 rs7904	2
AAC.GTT	6	47814455	47814466	4	.	.	intergenic	.	rs790740903	2
AAAC.GTTT	6	48085464	48085484	5.25	.	.	intergenic	0.302	792502 rs7893	2
AC.GT	6	48418445	48418472	14	ENSSSCT00000003452	.	intron	0.359	rs788199224	3
AAAC.GTTT	6	48543802	48543823	5.5	.	.	intergenic	-0.072	rs792263379	2
AT.AT	6	48691019	48691029	5.5	.	.	intergenic	0.095	326770 rs7885	2
AAAAC.GTTTT	6	48938652	48938672	4.2	ENSSSCT00000022965	GLTSCR1	intron	-0.127	.	2
AAAAC.GTTTT	6	48938652	48938672	4.2	ENSSSCT00000024758	GLTSCR1	intron	-0.127	.	2
AT.AT	6	49214831	49214842	6	ENSSSCT00000003469	LIG1	intron	-0.593	rs791240257	2
AAT.ATT	6	49392294	49392306	4.33333	ENSSSCT00000024369	EMP3	promoter	0.247	rs790136589	2
AC.GT	6	49881368	49881383	8	ENSSSCT00000003479	HSD17B14	intron	-0.375	.	2
AC.GT	6	50057739	50057749	5.5	.	.	intergenic	-0.317	.	2
AAAAT.ATTTT	6	50910777	50910798	4.4	ENSSSCT00000026507	.	intron	-0.119	rs787935971	2

AAAT.ATTT	6	51342053	51342074	5.5	.	.	intergenic	-0.037	465339 rs7901	2
AAAAT.ATTTT	6	51820343	51820362	4	ENSSSCT00000003595	SIGLEC5	intron	.	rs789240568	2
AAAC.GTTT	6	52341065	52341086	5.5	ENSSSCT00000024905	ZNF615	intron	.	468852 rs7917	3
AT.AT	6	53076559	53076568	5	ENSSSCT00000003618	CACNG6	intron	.	rs786245327	2
AC.GT	6	53444891	53444908	9	ENSSSCT00000025769	TTYH1	promoter	-0.254	rs792523984	2
AC.GT	6	53444891	53444908	9	ENSSSCT00000003632	TTYH1	promoter	-0.254	rs792523984	2
AAC.GTT	6	54275357	54275382	8.66667	ENSSSCT00000027366	PTPRH	3'utr	.	152174 rs7930	2
AAC.GTT	6	54275357	54275382	8.66667	ENSSSCT00000022455	PTPRH	3'utr	.	152174 rs7930	2
AAG.CTT	6	56777388	56777400	4.33333	.	.	intergenic	.	594179 rs7877	2
AAAAC.GTTTT	6	56831282	56831313	6.4	.	.	intergenic	.	454002 rs7887	2
AAAC.GTTT	6	57191369	57191384	4	.	.	intergenic	.	423232 rs7873	2
AAAAAC.GTTTTT	6	57217947	57217974	4.66667	.	.	intergenic	.	rs792988934	2
AAAT.ATTT	6	57687847	57687868	5.5	ENSSSCT00000004425	RPS5	intron	.	rs789579895	2
AAAAAC.GTTTTT	6	58204361	58204387	4.5	.	.	intergenic	.	.	2
AAG.CTT	6	58390673	58390686	4.66667	ENSSSCT00000032230	PRKCZ	intron	0.589	503139 rs7892	2
AG.CT	6	59038847	59038864	9	.	.	intergenic	.	010098 rs7921	2
AAAC.GTTT	6	59527365	59527383	4.75	.	.	intergenic	-0.351	rs792343292	2
AAAAC.GTTTT	6	60378044	60378064	4.2	.	.	intergenic	-0.138	rs786434609	2
AC.GT	6	60438922	60438946	12.5	.	.	intergenic	-0.438	rs789225885	2
AGG.CCT	6	61217006	61217020	5	.	.	intergenic	.	rs789853814	2
AAT.ATT	6	61757978	61757989	4	ENSSSCT00000022446	NPHP4	promoter	0.146	366787 rs7877	2
AAC.GTT	6	62175743	62175755	4.33333	ENSSSCT00000003747	ACOT7	intron	2.137	rs791830740	2
AC.GT	6	62585113	62585127	7.5	.	.	intergenic	-0.228	209403 rs7870	2
AAC.GTT	6	62880331	62880347	5.66667	.	.	intergenic	1.555	750129 rs7882	2
AC.GT	6	63032121	63032134	7	ENSSSCT00000003756	PARK7	intron	-0.097	rs792452892	3
AC.GT	6	63212581	63212595	7.5	.	.	intergenic	.	rs792742785	2
AAAC.GTTT	6	63693957	63693983	6.75	ENSSSCT00000003762	CA6	intron	.	rs787611498	2
AAG.CTT	6	63937289	63937303	5	.	.	intergenic	-0.138	rs786589481	2
AG.CT	6	64237099	64237110	6	.	.	intergenic	0.073	399055 rs7897	2
AAAC.GTTT	6	64261409	64261429	5.25	.	.	intergenic	-0.364	rs693783101	3
AATG.CATT	6	64268130	64268154	6.25	.	.	intergenic	.	rs791020399	2
AAAAC.GTTTT	6	64356773	64356799	5.4	.	.	intergenic	.	rs790040390	2
AAAAG.CTTTT	6	64666429	64666452	4.8	ENSSSCT00000003777	DFFA	intron	0.028	563189 rs7874	2
AAC.GTT	6	64676126	64676145	6.66667	ENSSSCT00000003778	PEX14	intron	.	242410 rs7879	2
AAAC.GTTT	6	64680847	64680865	4.75	ENSSSCT00000003778	PEX14	intron	.	rs792072305	2
AGAT.ATCT	6	65234004	65234022	4.75	ENSSSCT00000003786	FRAP1	intron	0.252	307507 rs7920	2
AAAC.GTTT	6	65302543	65302571	7.25	ENSSSCT00000003786	FRAP1	intron	-0.289	705137 rs7920	2
AT.AT	6	65421909	65421920	6	.	.	intergenic	-0.166	085311 rs7916	2
AC.GT	6	65668318	65668329	6	ENSSSCT00000003795	FBXO44	intron	1.03	rs788152514	2
AAAAAC.GTTTTT	6	65720732	65720753	3.66667	.	.	intergenic	-0.184	347842 rs7885	2
AAAT.ATTT	6	65797477	65797491	3.75	ENSSSCT00000003807	CLCN6	intron	.	574469 rs7871	2
AGG.CCT	6	65915248	65915261	4.66667	ENSSSCT00000003811	PLOD1	intron	0.593	.	2
AAAAAG.CTTTTT	6	67122315	67122338	4	.	.	intergenic	-0.435	rs787235387	2
AAGC.GCTT	6	67189830	67189846	4.25	.	.	intergenic	.	rs791670240	2
AAAAG.CTTTT	6	67205496	67205518	4.6	.	.	intergenic	0.019	104460 rs7863	2
AC.GT	6	67527601	67527613	6.5	.	.	intergenic	-0.043	337990 rs7931	2
AC.GT	6	67720322	67720332	5.5	.	.	intergenic	0.069	rs788410417	3
AG.CT	6	67884087	67884103	8.5	.	.	intergenic	-0.113	rs790737057	2
AGC.GCT	6	67961863	67961875	4.33333	.	.	intergenic	-0.144	rs789737082	2
AAAC.GTTT	6	68161014	68161033	5	.	.	intergenic	-0.243	rs793108665	2
AAAAT.ATTTT	6	68481139	68481157	3.8	.	.	intergenic	-0.189	969310 rs7867	2
AC.GT	6	68662022	68662035	7	ENSSSCT00000003835	KAZN	intron	-0.108	rs793026612	2
AG.CT	6	68937185	68937195	5.5	.	.	intergenic	-0.089	rs787741408	3
AC.GT	6	68947439	68947449	5.5	ENSSSCT00000027709	.	intron	0.144	rs791330954	2
AC.GT	6	68947439	68947449	5.5	ENSSSCT00000003836	.	intron	0.144	rs791330954	2
AAC.GTT	6	69235687	69235699	4.33333	ENSSSCT00000003850	DNAJC16	intron	0.088	rs791116949	2
AAAC.GTTT	6	69249126	69249140	3.75	ENSSSCT00000003850	DNAJC16	intron	0.068	354943 rs7879	2
AAAC.GTTT	6	69355022	69355048	6.75	ENSSSCT00000003851	FBLIM1	intron	0.031	.	2
AAC.GTT	6	69369825	69369840	5.33333	ENSSSCT00000003851	FBLIM1	intron	-0.134	rs793253460	2
AAAC.GTTT	6	69649411	69649437	6.75	.	.	intergenic	-0.202	rs792605751	2
AAAAAC.GTTTTT	6	69745258	69745276	3.16667	ENSSSCT00000032385	FBXO42	intron	.	rs786206765	2
AG.CT	6	70180744	70180758	7.5	ENSSSCT00000033697	PADI1	intron	-0.028	327662 rs7868	2
AAAT.ATTT	6	70225076	70225090	3.75	ENSSSCT00000003868	PADI3	intron	-0.34	rs793205161	2
AAC.GTT	6	70257380	70257402	7.66667	.	.	intergenic	.	rs791376184	2
AAC.GTT	6	70436814	70436833	6.66667	.	.	intergenic	.	rs792305502	2
AAAG.CTTT	6	70468951	70468969	4.75	.	.	intergenic	-0.142	.	2
AAAG.CTTT	6	70562896	70562912	4.25	.	.	intergenic	-0.113	rs788483300	2
ATC.GAT	6	70692860	70692876	5.66667	.	.	intergenic	.	216867 rs7864	2
AC.GT	6	70795566	70795576	5.5	.	.	intergenic	-0.321	355139 rs7918	2
ATCC.GGAT	6	71055621	71055636	4	.	.	intergenic	-0.005	.	2
AC.GT	6	72517021	72517060	20	.	.	intergenic	-0.285	599075 rs7926	4
AC.GT	6	72521624	72521635	6	ENSSSCT00000003882	PLA2G2A	promoter	.	760132 rs7932	2
AC.GT	6	72532189	72532203	7.5	.	.	intergenic	.	rs787663704	2
AAAT.ATTT	6	72616127	72616162	9	.	.	intergenic	.	.	2
AC.GT	6	72891534	72891544	5.5	.	.	intergenic	.	.	2
AC.GT	6	72944253	72944262	5	ENSSSCT00000027632	MUL1	intron	.	.	2
AT.AT	6	73363786	73363799	7	.	.	intergenic	0.139	rs790221559	2
AAAAT.ATTTT	6	73459761	73459782	4.4	.	.	intergenic	0.048	rs791024635	2
AC.GT	6	73564810	73564820	5.5	.	.	intergenic	0.34	.	2
AAAT.ATTT	6	74315245	74315271	6.75	.	.	intergenic	.	396046 rs7908	2
AC.GT	6	74745550	74745563	7	.	.	intergenic	0.038	375504 rs7913	2
AT.AT	6	74769648	74769658	5.5	ENSSSCT00000003918	EPHB2	intron	0.14	.	2
AAAC.GTTT	6	74951866	74951898	8.25	ENSSSCT00000003922	KDM1A	intron	0.19	.	2
AAAC.GTTT	6	74951866	74951898	8.25	ENSSSCT00000003923	KDM1A	intron	0.19	.	2

AT.AT	6	75181348	75181359	6	.	.	intergenic	0.009	47140 rs7922	2
AAAG.CTTT	6	75246258	75246283	6.5	.	.	intergenic	.	10664 rs7897	3
AAAAC.GTTTT	6	76024485	76024504	4	.	.	intergenic	-0.066	rs788885662	2
AAC.GTT	6	76046290	76046303	4.66667	.	.	intergenic	0.148	60569 rs7898	2
AATC.GATT	6	76289081	76289109	7.25	.	.	intergenic	-0.216	.	2
AAAAC.GTTTT	6	76492714	76492748	7	.	.	intergenic	.	rs788056522	2
AT.AT	6	76852789	76852799	5.5	.	.	intergenic	.	rs792296769	2
AC.GT	6	77224880	77224895	8	ENSSSCT00000024815	CATSPER4	intron	0.846	.	2
AAC.GTT	6	78641723	78641737	5	ENSSSCT00000003975	EYA3	intron	-0.02	66971 rs7902	2
AAAT.ATTT	6	79121036	79121053	4.5	.	.	intergenic	0.229	rs786613414	2
AAAC.GTTT	6	79363880	79363901	5.5	ENSSSCT00000028011	RCC1	intron	.	.	2
AAAC.GTTT	6	79405416	79405430	3.75	ENSSSCT00000028704	TRNAU1AP	intron	.	.	3
AAAAC.GTTTT	6	79436575	79436604	6	ENSSSCT00000027861	TAF12	intron	0.456	.	2
AAG.CTT	6	79566125	79566140	5.33333	.	.	intergenic	0.284	rs790470702	2
AAAAT.ATTTT	6	79831189	79831212	4.8	ENSSSCT00000003981	EPB41	intron	0.379	rs789483116	2
AAC.GTT	6	80050145	80050173	9.66667	ENSSSCT00000003985	PTPRU	intron	-0.084	325341 rs7896	2
AG.CT	6	80189534	80189546	6.5	.	.	intergenic	0.281	rs793454489	2
AG.CT	6	80244065	80244076	6	.	.	intergenic	.	.	2
AC.GT	6	80299679	80299691	6.5	.	.	intergenic	0.042	02682 rs7906	2
AG.CT	6	80654265	80654289	12.5	.	.	intergenic	-0.409	.	3
AC.GT	6	80803136	80803147	6	.	.	intergenic	-0.337	rs790491132	2
AAAC.GTTT	6	80851264	80851287	6	.	.	intergenic	.	rs787901763	2
ATC.GAT	6	80868176	80868207	10.6667	.	.	intergenic	-0.101	45427 rs7910	2
ACAG.CTGT	6	81102839	81102856	4.5	.	.	intergenic	-0.167	rs792804670	3
AAAC.GTTT	6	81166754	81166776	5.75	.	.	intergenic	0.115	80692 rs7895	2
AT.AT	6	81410131	81410149	9.5	.	.	intergenic	.	rs792834598	2
AAAT.ATTT	6	81464364	81464377	3.5	.	.	intergenic	.	.	2
AG.CT	6	81765987	81765999	6.5	ENSSSCT00000003994	.	intron	.	rs696422270	3
AC.GT	6	81965995	81966007	6.5	ENSSSCT00000003999	.	intron	-0.001	.	3
AAC.GTT	6	82296547	82296570	8	.	.	intergenic	.	rs787699537	2
AG.CT	6	83398055	83398070	8	.	.	intergenic	0.226	.	4
AAAC.GTTT	6	83914060	83914086	6.75	ENSSSCT00000004018	CSMD2	intron	-0.225	rs789731403	2
AG.CT	6	84041740	84041749	5	ENSSSCT00000004018	CSMD2	intron	-0.39	.	4
AAAC.GTTT	6	84218659	84218675	4.25	.	.	intergenic	-0.02	rs787599556	2
AG.CT	6	84229308	84229319	6	.	.	intergenic	0.114	rs786825269	2
AG.CT	6	84235814	84235843	15	.	.	intergenic	-0.113	.	2
AAC.GTT	6	84520363	84520374	4	.	.	intergenic	0.234	.	2
AT.AT	6	84590312	84590339	14	.	.	intergenic	-0.243	95408 rs7935	2
AC.GT	6	84594503	84594517	7.5	.	.	intergenic	0.259	68243 rs7898	2
AAC.GTT	6	84595567	84595586	6.66667	.	.	intergenic	-0.048	16384 rs7903	2
AC.GT	6	85243563	85243573	5.5	ENSSSCT00000020107	SNORD22	promoter	.	rs790742995	2
AAAT.ATTT	6	85566350	85566376	6.75	ENSSSCT00000004032	.	intron	.	rs793150867	3
AATG.CATT	6	85667662	85667687	6.5	ENSSSCT00000004034	TRAPPC3	intron	.	rs789380643	2
AATG.CATT	6	85790353	85790382	7.5	.	.	intergenic	.	rs788592785	3
AC.GT	6	85916422	85916432	5.5	.	.	intergenic	-0.094	rs793504318	2
AAC.GTT	6	86066088	86066099	4	.	.	intergenic	.	rs709217950	3
AG.CT	6	86482335	86482349	7.5	.	.	intergenic	.	05550 rs7864	2
AAGG.CCTT	6	86493115	86493137	5.75	.	.	intergenic	-0.065	.	4
ACGC.GCGT	6	86880370	86880384	3.75	.	.	intergenic	0.38	rs789731793	2
AAAC.GTTT	6	87705616	87705638	5.75	.	.	intergenic	-0.176	94141 rs7912	2
AAAC.GTTT	6	87833173	87833205	8.25	.	.	intergenic	0.096	11447 rs7063	2
AAAAC.GTTTT	6	88033711	88033737	4.5	ENSSSCT00000004050	MACF1	intron	-0.305	rs787717609	2
AAAC.GTTT	6	88393728	88393739	3	ENSSSCT00000004050	MACF1	intron	-0.021	rs789551531	2
AG.CT	6	88534538	88534563	13	ENSSSCT00000023745	HEYL	intron	-0.1	rs707868325	4
AAC.GTT	6	88578863	88578884	7.33333	ENSSSCT00000004058	NT5C1A	intron	.	rs788938716	2
AAGG.CCTT	6	88733652	88733684	8.25	ENSSSCT00000004061	.	intron	.	rs789241405	2
AAGG.CCTT	6	88733652	88733684	8.25	ENSSSCT00000004052	.	intron	.	rs789241405	2
AC.GT	6	88776350	88776359	5	.	.	intergenic	0.2	.	2
AAAT.ATTT	6	88861388	88861413	6.5	.	.	intergenic	.	.	2
AAAC.GTTT	6	88885661	88885683	5.75	.	.	intergenic	0.042	rs787519975	2
AAT.ATT	6	88992216	88992238	7.66667	.	.	intergenic	.	rs786985793	2
AT.AT	6	89188918	89188928	5.5	ENSSSCT00000004066	RLF	intron	0.405	rs789281296	2
AAG.CTT	6	89248346	89248359	4.66667	.	.	intergenic	.	.	2
AAGG.CCTT	6	89253488	89253499	3	.	.	intergenic	.	rs788450015	2
AC.GT	6	89669154	89669164	5.5	.	.	intergenic	.	rs792851819	2
AT.AT	6	89714997	89715008	6	ENSSSCT00000032434	LDLRAD4	intron	0.164	72173 rs7886	2
ACAT.ATGT	6	89946646	89946665	5	ENSSSCT00000004070	CEP192	intron	0.042	27349 rs7922	2
ACAG.CTGT	6	90053044	90053065	5.5	.	.	intergenic	.	rs791469414	2
AAAC.GTTT	6	90169848	90169862	3.75	ENSSSCT00000025318	PTPN2	intron	.	rs792229356	2
AAT.ATT	6	90336968	90336987	6.66667	ENSSSCT00000031445	CEP76	3'utr	0.324	38459 rs7916	2
AC.GT	6	90369620	90369632	6.5	ENSSSCT00000028047	.	intron	.	.	3
AAC.GTT	6	90449308	90449324	5.66667	.	.	intergenic	-0.3	07338 rs7888	2
AAAT.ATTT	6	90462578	90462595	4.5	.	.	intergenic	.	rs790882559	2
AC.GT	6	90750532	90750546	7.5	ENSSSCT00000022557	IMPA2	intron	.	.	2
ACC.GGT	6	90917060	90917071	4	.	.	intergenic	-0.188	rs789234056	2
AAAT.ATTT	6	90923932	90923943	3	.	.	intergenic	.	rs793781486	2
AAAAC.GTTTT	6	91115487	91115507	4.2	.	.	intergenic	-0.099	rs789889511	2
AAAC.GTTT	6	91123398	91123436	9.75	.	.	intergenic	0.21	54110 rs7899	2
AGC.GCT	6	91161535	91161555	7	.	.	intergenic	0.056	.	2
AT.AT	6	91300157	91300171	7.5	.	.	intergenic	.	rs793292516	2
AC.GT	6	91348549	91348565	8.5	.	.	intergenic	-0.199	rs792634739	2
AT.AT	6	91409909	91409925	8.5	.	.	intergenic	.	rs786349076	2
AG.CT	6	91483293	91483307	7.5	.	.	intergenic	-0.047	rs789877075	2
AT.AT	6	91510196	91510213	9	.	.	intergenic	.	.	2

ATGC.GCAT	6	91824929	91824944	4	ENSSSCT00000004076	.	intron	.	.	2
AAAC.GTTT	6	91866495	91866526	8	ENSSSCT00000004076	.	intron	.	rs790357696	2
AAAC.GTTT	6	91866495	91866526	8	ENSSSCT00000004078	RALBP1	intron	.	rs790357696	2
AT.AT	6	92105019	92105029	5.5	ENSSSCT00000004080	ANKRD12	intron	0.153	rs786819720	4
AAAC.GTTT	6	92346239	92346261	5.75	.	.	intergenic	-0.294	rs789902594	2
AC.GT	6	92409475	92409486	6	ENSSSCT00000024838	SOGA2	intron	0.039	rs793024293	2
AC.GT	6	92409475	92409486	6	ENSSSCT00000004082	SOGA2	intron	0.039	rs793024293	2
AC.GT	6	92545326	92545347	11	ENSSSCT00000032539	PTPRM	intron	.	rs793680660	2
AC.GT	6	92647994	92648004	5.5	.	.	intergenic	-0.236	rs787868324	2
AATT.AATT	6	92726334	92726351	4.5	.	.	intergenic	0.248	rs792453311	2
AC.GT	6	92798062	92798074	6.5	.	.	intergenic	-0.321	rs793607780	2
AAAG.CTTT	6	92936658	92936671	3.5	.	.	intergenic	.	rs789232014	2
AAGG.CCTT	6	93048165	93048184	5	.	.	intergenic	-0.216	rs790632050	2
AAT.ATT	6	93156765	93156782	6	.	.	intergenic	-0.048	.	2
AAG.CTT	6	93233128	93233159	10.6667	.	.	intergenic	0.114	rs791217831	2
AT.AT	6	93338712	93338722	5.5	.	.	intergenic	.	rs793836556	3
AGAGC.GCTCT	6	93442079	93442098	4	.	.	intergenic	.	452437 rs7862	2
AC.GT	6	93686710	93686722	6.5	ENSSSCT00000004084	.	intron	0.161	rs787325777	2
AC.GT	6	93770304	93770327	12	ENSSSCT00000004084	.	intron	.	rs788671695	4
AAAC.GTTT	6	93798120	93798140	5.25	ENSSSCT00000004084	.	intron	0.24	rs789337089	2
AAAG.CTTT	6	93811075	93811120	11.5	ENSSSCT00000004084	.	intron	-0.018	rs786648078	2
AT.AT	6	93960710	93960726	8.5	.	.	intergenic	0.128	rs793283749	2
AAAAG.CTTTT	6	93961047	93961070	4.8	.	.	intergenic	-0.066	rs791636298	2
AC.GT	6	94215508	94215530	11.5	.	.	intergenic	.	379603 rs7931	2
AG.CT	6	94242037	94242047	5.5	.	.	intergenic	.	rs792396324	2
AAAC.GTTT	6	94259653	94259687	8.75	.	.	intergenic	.	rs792230191	2
AAAT.ATTT	6	94752787	94752816	7.5	ENSSSCT00000004085	EPB41L3	intron	0.106	235586 rs7923	2
ACAG.CTGT	6	95003862	95003875	3.5	.	.	intergenic	.	.	2
AAAC.GTTT	6	95027629	95027643	3.75	.	.	intergenic	0.082	rs786343557	4
AC.GT	6	95069327	95069341	7.5	.	.	intergenic	0.123	rs793585110	2
AT.AT	6	95280953	95280966	7	.	.	intergenic	-0.038	rs698121647	2
AAAC.GTTT	6	95330656	95330676	5.25	.	.	intergenic	-0.049	rs791980852	2
AAC.GTT	6	95548825	95548849	8.33333	.	.	intergenic	.	rs791739252	2
AG.CT	6	95581817	95581827	5.5	.	.	intergenic	0.395	rs787984489	4
AAAT.ATTT	6	95590815	95590829	3.75	.	.	intergenic	0.374	rs791136620	2
AAAC.GTTT	6	95703659	95703681	5.75	.	.	intergenic	0.169	rs790017056	2
AG.CT	6	95726530	95726541	6	.	.	intergenic	-0.095	rs793608192	2
AAT.ATT	6	95764845	95764861	5.66667	.	.	intergenic	0.018	rs792608869	2
AC.GT	6	95770294	95770314	10.5	.	.	intergenic	-0.043	014700 rs7884	2
AAAAC.GTTTT	6	95949308	95949330	3.83333	ENSSSCT00000026090	.	intron	.	rs790992061	2
AC.GT	6	95981855	95981865	5.5	.	.	intergenic	-0.046	.	4
AAAT.ATTT	6	96114852	96114872	5.25	.	.	intergenic	-0.05	rs788158908	2
AC.GT	6	96135670	96135682	6.5	.	.	intergenic	0.023	rs790830263	2
AAAAG.CTTTT	6	96205207	96205229	4.6	ENSSSCT00000004088	DLGAP1	intron	-0.004	433453 rs7901	2
AAAT.ATTT	6	96333672	96333695	6	.	.	intergenic	0.115	rs790903171	2
AAAC.GTTT	6	96335091	96335103	3.25	.	.	intergenic	0.036	.	2
AAAG.CTTT	6	96369918	96369941	6	.	.	intergenic	.	rs790012071	2
AAAT.ATTT	6	96763386	96763404	4.75	ENSSSCT00000004093	EMILIN2	intron	0.02	rs790804525	2
AAAAC.GTTTT	6	96951014	96951046	6.6	.	.	intergenic	0.114	rs787885696	2
AG.CT	6	97273108	97273120	6.5	.	.	intergenic	.	rs792814004	3
AAAC.GTTT	6	97279322	97279353	8	.	.	intergenic	0.124	rs790713716	3
AT.AT	6	97456786	97456796	5.5	.	.	intergenic	0.169	rs789605420	2
AAAG.CTTT	6	97459231	97459249	4.75	.	.	intergenic	0.039	rs793229611	2
AT.AT	6	97587678	97587690	6.5	.	.	intergenic	0.392	114893 rs7900	2
AAAT.ATTT	6	97624836	97624853	4.5	.	.	intergenic	.	459270 rs7918	2
AT.AT	6	97917004	97917019	8	.	.	intergenic	0.036	rs787312908	3
AG.CT	6	97963601	97963623	11.5	.	.	intergenic	0.045	328006 rs7908	2
AT.AT	6	98146606	98146620	7.5	.	.	intergenic	.	rs792577476	2
AATCAT.ATGATT	6	98207450	98207478	4.83333	.	.	intergenic	.	.	2
AG.CT	6	98216554	98216570	8.5	.	.	intergenic	.	333408 rs7869	5
AG.CT	6	98407644	98407681	19	.	.	intergenic	0.182	rs790305658	2
AAAC.GTTT	6	98513714	98513736	5.75	.	.	intergenic	0.06	rs791431644	2
AAAT.ATTT	6	98691072	98691084	3.25	.	.	intergenic	0.085	.	3
AAC.GTT	6	98729480	98729492	4.33333	ENSSSCT00000029907	ROCK1	intron	.	rs788861348	3
AAC.GTT	6	98729480	98729492	4.33333	ENSSSCT00000026467	ROCK1	intron	.	rs788861348	3
AG.CT	6	99118258	99118276	9.5	ENSSSCT00000004098	GREB1L	intron	1.322	rs793214520	2
AC.GT	6	99370829	99370838	5	ENSSSCT00000031178	ABHD3	intron	0.12	rs791097121	2
AAAT.ATTT	6	99392333	99392348	4	ENSSSCT00000031178	ABHD3	intron	-0.087	340113 rs7915	2
AC.GT	6	99713900	99713914	7.5	.	.	intergenic	-0.025	953298 rs7930	2
AC.GT	6	99753223	99753237	7.5	.	.	intergenic	0.151	rs790996573	3
AGG.CCT	6	99841236	99841253	6	.	.	intergenic	-0.059	rs793423560	2
AAAAC.GTTTT	6	99892160	99892185	5.2	.	.	intergenic	0.185	278432 rs7892	2
AAAC.GTTT	6	99907720	99907738	4.75	.	.	intergenic	.	320684 rs7901	2
AAAC.GTTT	6	100179386	100179397	3	.	.	intergenic	0.269	.	2
AAG.CTT	6	100330159	100330176	6	.	.	intergenic	.	949485 rs7897	3
AAAAC.GTTTT	6	100572350	100572379	6	.	.	intergenic	0.268	589515 rs7894	2
AAAAC.GTTTT	6	101065449	101065476	5.6	ENSSSCT00000004107	ANKRD29	intron	.	rs793879093	2
AAC.GTT	6	101276400	101276414	5	ENSSSCT00000027989	LAMA5	intron	0.883	rs787272636	2
AATG.CATT	6	101311754	101311771	4.5	ENSSSCT00000027989	LAMA5	intron	-0.047	rs792169969	2
AAAG.CTTT	6	101474831	101474845	3.75	ENSSSCT00000004108	LAMA3	intron	0.296	rs787087609	2
AAAG.CTTT	6	101474831	101474845	3.75	ENSSSCT00000024191	LAMA3	intron	0.296	rs787087609	2
AAAAAT.ATTTTT	6	101761908	101761935	4.66667	ENSSSCT00000004111	OSBPL1A	intron	.	rs787961323	2
AC.GT	6	102448839	102448870	16	.	.	intergenic	-0.247	rs790852455	2
AAC.GTT	6	102688800	102688813	4.66667	.	.	intergenic	0.181	rs792907075	2

AAAC.GTTT	6	102803028	102803048	5.25	.	.	intergenic	-0.203	.	2
AG.CT	6	103107758	103107767	5	.	.	intergenic	.	rs789241293	2
AGAT.ATCT	6	103142644	103142680	9.25	.	.	intergenic	0.07	rs793662530	2
ACAT.ATGT	6	103351557	103351576	5	.	.	intergenic	-0.035	rs789593031	2
AAAC.GTTT	6	103581198	103581218	5.25	.	.	intergenic	0.435	rs793574140	2
AAAG.CTTT	6	103865559	103865571	3.25	ENSSSCT00000004118	TAF4B	intron	.	rs786345540	2
AAG.CTT	6	104041430	104041449	6.66667	ENSSSCT00000031750	KCTD1	intron	0.196	rs788335004	2
AAG.CTT	6	104041430	104041449	6.66667	ENSSSCT00000004119	KCTD1	intron	0.196	rs788335004	2
AAATG.CATTT	6	104060176	104060204	5.8	ENSSSCT00000031750	KCTD1	intron	0.041	rs791388784	2
ACAG.CTGT	6	104085672	104085699	7	ENSSSCT00000031750	KCTD1	intron	0.16	201709 rs7895	2
AC.GT	6	104145577	104145587	5.5	.	.	intergenic	0.279	.	3
AAT.ATT	6	104176575	104176588	4.66667	.	.	intergenic	-0.128	rs792411908	2
AG.CT	6	104229534	104229565	16	.	.	intergenic	-0.026	rs790211676	2
AAC.GTT	6	104286693	104286709	5.66667	.	.	intergenic	0.17	rs789304561	2
ATC.GAT	6	104442774	104442789	5.33333	.	.	intergenic	-0.008	316728 rs7902	4
AAAC.GTTT	6	104573351	104573375	6.25	.	.	intergenic	.	367276 rs7925	2
AAC.GTT	6	104655294	104655310	5.66667	.	.	intergenic	0.099	.	3
AAAC.GTTT	6	104784712	104784737	6.5	.	.	intergenic	0.116	rs789714791	2
AC.GT	6	104914361	104914371	5.5	.	.	intergenic	-0.363	.	2
AC.GT	6	105066561	105066571	5.5	.	.	intergenic	0.397	rs787634463	2
AAAAC.GTTTT	6	105078096	105078111	3.2	.	.	intergenic	-0.008	rs793345289	2
AT.AT	6	105107875	105107885	5.5	.	.	intergenic	-0.041	rs786325269	2
AAC.GTT	6	105395736	105395748	4.33333	.	.	intergenic	0.098	rs786608093	2
AAAAT.ATTTT	6	105549021	105549035	3	.	.	intergenic	-0.128	rs790747339	2
AT.AT	6	105640998	105641008	5.5	.	.	intergenic	0.301	rs787319550	3
AAAC.GTTT	6	105872967	105872989	5.75	.	.	intergenic	-0.061	rs692897045	2
AAAG.CTTT	6	105946705	105946725	5.25	.	.	intergenic	.	rs789392710	2
AAG.CTT	6	105960121	105960151	10.3333	.	.	intergenic	-0.243	rs789439090	2
AAAT.ATTT	6	106104799	106104823	6.25	.	.	intergenic	-0.03	rs792008058	2
AAAAAC.GTTTTT	6	106290475	106290497	3.83333	.	.	intergenic	0.019	rs790070338	2
AAAC.GTTT	6	106588586	106588630	11.25	.	.	intergenic	.	490448 rs7922	2
AAAAAC.GTTTTT	6	107171014	107171038	4.16667	.	.	intergenic	-0.195	rs791817575	2
AT.AT	6	107252641	107252653	6.5	.	.	intergenic	0.007	rs789504308	2
AAAG.CTTT	6	107272613	107272638	6.5	.	.	intergenic	-0.043	rs710553298	2
AT.AT	6	107718266	107718285	10	.	.	intergenic	.	rs789990337	2
AAAC.GTTT	6	107789642	107789660	4.75	.	.	intergenic	.	573796 rs7868	2
AAAAAT.ATTTTT	6	107813933	107813950	3	.	.	intergenic	0.165	rs790107538	2
AG.CT	6	107879950	107879960	5.5	ENSSSCT00000022644	DSG1	intron	0.166	rs792627134	2
ATC.GAT	6	108069907	108069918	4	ENSSSCT00000025628	DSG2	intron	.	rs788631469	2
ATC.GAT	6	108069907	108069918	4	ENSSSCT00000029235	DSG2	intron	.	rs788631469	2
AAAG.CTTT	6	108108674	108108696	5.75	.	.	intergenic	0.119	.	2
AAG.CTT	6	108329397	108329416	6.66667	.	.	intergenic	.	rs791186539	2
AAC.GTT	6	108677144	108677157	4.66667	.	.	intergenic	0.457	rs787290761	2
AAAC.GTTT	6	108719840	108719864	6.25	ENSSSCT00000004132	MEP1B	intron	.	rs789927364	2
AC.GT	6	108780110	108780119	5	ENSSSCT00000004131	GAREM	intron	.	rs792259027	3
AC.GT	6	109271928	109271946	9.5	ENSSSCT00000004133	KLHL14	intron	0.019	.	3
AG.CT	6	109313877	109313902	13	ENSSSCT00000004133	KLHL14	intron	0.227	rs786489059	4
AC.GT	6	109378643	109378653	5.5	.	.	intergenic	0.114	rs792413376	2
AC.GT	6	109404717	109404755	19.5	.	.	intergenic	0.093	762266 rs7862	2
AAAG.CTTT	6	109414711	109414728	4.5	.	.	intergenic	0.037	799994 rs7915	2
AT.AT	6	109490855	109490866	6	.	.	intergenic	0.193	rs788600737	2
AG.CT	6	109869261	109869271	5.5	.	.	intergenic	0.324	rs787489345	2
AAC.GTT	6	109891020	109891039	6.66667	.	.	intergenic	.	rs791529422	2
AATT.AATT	6	109969561	109969574	3.5	.	.	intergenic	.	.	2
AG.CT	6	110069313	110069324	6	ENSSSCT00000004137	NOL4	intron	.	.	2
AAAAG.CTTTT	6	110161544	110161559	3.2	ENSSSCT00000004137	NOL4	intron	.	343877 rs7917	2
AAAT.ATTT	6	110265457	110265471	3.75	.	.	intergenic	.	rs787482435	2
AC.GT	6	110379116	110379129	7	.	.	intergenic	0.068	387969 rs7929	2
AT.AT	6	110429503	110429526	12	.	.	intergenic	.	.	2
AAGAAT.ATTCTT	6	110610900	110610926	4.5	.	.	intergenic	0.078	rs793744491	2
AAAC.GTTT	6	110625449	110625468	5	.	.	intergenic	0.1	rs786973913	2
AATG.CATT	6	110739891	110739912	5.5	ENSSSCT00000024859	.	intron	0.073	rs793687712	2
AAT.ATT	6	110875153	110875164	4	.	.	intergenic	0.156	933615 rs7896	2
AT.AT	6	111343381	111343400	10	ENSSSCT00000004139	MAPRE2	intron	0.017	rs789311392	2
AAAAC.GTTTT	6	111373463	111373484	4.4	ENSSSCT00000004139	MAPRE2	intron	-0.131	rs791926970	2
AAAC.GTTT	6	111669339	111669358	5	.	.	intergenic	-0.011	rs788097862	2
AC.GT	6	111834913	111834928	8	ENSSSCT00000032735	GALNT1	intron	.	310678 rs7878	2
AAC.GTT	6	111926286	111926303	6	ENSSSCT00000004144	GALNT1	intron	0.192	rs788432256	2
AAC.GTT	6	111926286	111926303	6	ENSSSCT00000032735	GALNT1	intron	0.192	rs788432256	2
AG.CT	6	112381167	112381178	6	.	.	intergenic	.	.	3
AC.GT	6	112672333	112672347	7.5	.	.	intergenic	.	275972 rs7876	2
AAG.CTT	6	112680039	112680063	8.33333	.	.	intergenic	.	.	2
AAC.GTT	6	112914578	112914591	4.66667	ENSSSCT00000025775	FHOD3	intron	-0.083	rs791525234	2
AAC.GTT	6	112914578	112914591	4.66667	ENSSSCT00000023176	FHOD3	intron	-0.083	rs791525234	2
AAC.GTT	6	112914578	112914591	4.66667	ENSSSCT00000029253	FHOD3	intron	-0.083	rs791525234	2
AAAC.GTTT	6	112999990	113000008	4.75	ENSSSCT00000025775	FHOD3	intron	0.288	242740 rs7903	2
AAAC.GTTT	6	112999990	113000008	4.75	ENSSSCT00000023176	FHOD3	intron	0.288	242740 rs7903	2
AAAC.GTTT	6	112999990	113000008	4.75	ENSSSCT00000029253	FHOD3	intron	0.288	242740 rs7903	2
AAAAAC.GTTTTT	6	113099188	113099210	3.83333	ENSSSCT00000004148	.	intron	.	491830 rs7863	3
AAAAAC.GTTTTT	6	113099188	113099210	3.83333	ENSSSCT00000029253	FHOD3	intron	.	491830 rs7863	3
AAAAAC.GTTTTT	6	113099188	113099210	3.83333	ENSSSCT00000025775	FHOD3	intron	.	491830 rs7863	3
AAAAAC.GTTTTT	6	113099188	113099210	3.83333	ENSSSCT00000023176	FHOD3	intron	.	491830 rs7863	3
AT.AT	6	113467378	113467393	8	.	.	intergenic	.	rs793355675	2
AAAC.GTTT	6	113827528	113827543	4	.	.	intergenic	0.053	378239 rs7923	2

AAG.CTT	6	114137794	114137817	8	.	.	intergenic	-0.186	.	2
AC.GT	6	114172244	114172262	9.5	.	.	intergenic	-0.509	344421 rs7932	3
AC.GT	6	114286376	114286385	5	.	.	intergenic	.	.	2
AAAAC.GTTTT	6	114469533	114469561	4.83333	.	.	intergenic	.	rs790592644	2
AT.AT	6	114481315	114481325	5.5	.	.	intergenic	0.088	rs792776424	2
AAT.ATT	6	114712169	114712180	4	.	.	intergenic	0.045	.	2
AG.CT	6	114995578	114995600	11.5	.	.	intergenic	-0.081	.	3
AT.AT	6	115250733	115250747	7.5	.	.	intergenic	.	.	2
AT.AT	6	115398630	115398646	8.5	.	.	intergenic	-0.022	rs791048840	2
AC.GT	6	115628804	115628815	6	.	.	intergenic	-0.435	rs790904949	2
AAAT.ATTT	6	115660113	115660146	8.5	.	.	intergenic	.	rs792684981	2
ATC.GAT	6	116028372	116028411	13.3333	.	.	intergenic	0.139	.	2
AT.AT	6	116177083	116177102	10	.	.	intergenic	-0.011	rs788985486	2
AC.GT	6	116248004	116248034	15.5	.	.	intergenic	-0.001	.	2
ATC.GAT	6	116265924	116265937	4.66667	.	.	intergenic	.	rs787392286	2
AT.AT	6	116272303	116272319	8.5	.	.	intergenic	-0.039	rs793493824	2
AC.GT	6	116433204	116433216	6.5	.	.	intergenic	0.306	rs788011604	3
AAT.ATT	6	116561775	116561805	10.3333	.	.	intergenic	-0.066	.	2
AG.CT	6	116668583	116668592	5	.	.	intergenic	-0.126	rs788494159	2
AC.GT	6	116820353	116820365	6.5	.	.	intergenic	-0.016	.	2
AAAT.ATTT	6	116853554	116853566	3.25	.	.	intergenic	.	.	2
AC.GT	6	116963146	116963158	6.5	.	.	intergenic	-0.226	.	3
AAAAT.ATTTT	6	117148363	117148385	4.6	.	.	intergenic	-0.09	rs787214380	2
AG.CT	6	117373637	117373649	6.5	.	.	intergenic	0.004	rs786703456	2
AC.GT	6	117450972	117450985	7	.	.	intergenic	.	rs709573832	4
AG.CT	6	117565898	117565911	7	.	.	intergenic	0.318	rs789172462	2
AC.GT	6	117746379	117746393	7.5	.	.	intergenic	-0.089	905384 rs7931	3
AT.AT	6	117797082	117797093	6	.	.	intergenic	0.887	rs791722278	2
AT.AT	6	118011701	118011712	6	ENSSSCT00000004151	PIK3C3	intron	0.515	.	2
AC.GT	6	118109718	118109730	6.5	.	.	intergenic	.	.	4
AAAC.GTTT	6	118311648	118311666	4.75	.	.	intergenic	.	265494 rs7914	2
AAC.GTT	6	119184353	119184367	5	.	.	intergenic	.	rs787789676	2
AAAC.GTTT	6	119456221	119456254	8.5	.	.	intergenic	0.115	103828 rs7916	4
AAAC.GTTT	6	119689812	119689845	8.5	.	.	intergenic	0.258	rs788122143	2
ATCC.GGAT	6	119697765	119697779	3.75	.	.	intergenic	-0.05	.	2
AAC.GTT	6	119713418	119713431	4.66667	.	.	intergenic	0.153	301154 rs7867	2
AC.GT	6	120307655	120307666	6	.	.	intergenic	0.215	rs788693706	4
AAAT.ATTT	6	120372156	120372177	5.5	.	.	intergenic	0.217	rs791866251	2
AAAT.ATTT	6	120484902	120484920	4.75	.	.	intergenic	0.015	385635 rs7930	2
AAT.ATT	6	120695808	120695823	5.33333	.	.	intergenic	-0.046	775695 rs7870	2
AT.AT	6	120816057	120816081	12.5	.	.	intergenic	1.66	719325 rs7931	2
AT.AT	6	120926659	120926670	6	.	.	intergenic	-0.012	rs787173094	2
AAAC.GTTT	6	121179689	121179704	4	.	.	intergenic	-0.189	rs789649388	2
AG.CT	6	121181736	121181750	7.5	.	.	intergenic	-0.059	.	2
AAAGG.CCTTT	6	121297370	121297392	4.6	.	.	intergenic	-0.222	rs789253107	2
AAAT.ATTT	6	121395690	121395704	3.75	.	.	intergenic	0.197	.	2
AC.GT	6	121460077	121460087	5.5	.	.	intergenic	-0.075	rs196960707	3
AAAAG.CTTTT	6	121630387	121630404	3.6	.	.	intergenic	-0.006	rs790656416	2
AG.CT	6	121936209	121936221	6.5	.	.	intergenic	-0.052	rs786503376	2
AT.AT	6	121968084	121968100	8.5	ENSSSCT00000004163	LPHN2	intron	0.275	rs786596200	2
AC.GT	6	122283987	122283997	5.5	.	.	intergenic	0.595	rs791373771	2
AG.CT	6	122756467	122756477	5.5	.	.	intergenic	0.394	.	3
AAT.ATT	6	122838667	122838680	4.66667	.	.	intergenic	-0.088	rs790214030	2
AAC.GTT	6	122847847	122847860	4.66667	.	.	intergenic	0.53	rs792026947	2
AC.GT	6	122925815	122925833	9.5	.	.	intergenic	-0.071	483029 rs7934	2
AAAC.GTTT	6	122928274	122928294	5.25	.	.	intergenic	-0.029	rs787088260	2
AT.AT	6	122945893	122945928	18	.	.	intergenic	-0.276	rs787173080	2
AT.AT	6	123162216	123162233	9	.	.	intergenic	-0.146	rs789152730	2
AAAC.GTTT	6	123249324	123249347	6	.	.	intergenic	.	rs787244759	2
AAAC.GTTT	6	123293068	123293086	4.75	.	.	intergenic	.	.	2
AG.CT	6	123325638	123325651	7	.	.	intergenic	0.017	rs787698180	2
AACAC.GTGTT	6	123563269	123563284	3.2	.	.	intergenic	.	362557 rs7890	2
AAAG.CTTT	6	123689299	123689321	5.75	.	.	intergenic	-0.09	067552 rs7881	2
ATC.GAT	6	123700242	123700254	4.33333	.	.	intergenic	0.61	rs786848753	2
AAAT.ATTT	6	123902674	123902692	4.75	.	.	intergenic	0.626	.	2
AAAT.ATTT	6	123920457	123920478	5.5	.	.	intergenic	-0.149	rs704502297	2
AAC.GTT	6	123999531	123999547	5.66667	.	.	intergenic	.	371445 rs7869	3
AAAAT.ATTTT	6	124006877	124006900	4.8	.	.	intergenic	-0.072	720572 rs7902	2
AGGG.CCCT	6	124141937	124141948	3	.	.	intergenic	-0.204	.	2
AAAGAG.CTCTT	6	124247551	124247583	5.5	.	.	intergenic	-0.107	rs789311194	2
AT.AT	6	124351908	124351918	5.5	.	.	intergenic	-0.093	.	4
AAAC.GTTT	6	124406662	124406681	5	.	.	intergenic	1.61	098734 rs7863	2
AAAC.GTTT	6	124633551	124633571	5.25	.	.	intergenic	.	rs792153091	2
AAAC.GTTT	6	125136573	125136586	3.5	.	.	intergenic	0.56	541467 rs7873	2
AAAT.ATTT	6	125176360	125176381	5.5	.	.	intergenic	0.015	rs786540515	3
AG.CT	6	125232775	125232789	7.5	.	.	intergenic	.	953163 rs7865	2
AAG.CTT	6	125620651	125620664	4.66667	ENSSSCT00000029114	NEXN	intron	.	911553 rs7894	3
AAG.CTT	6	125620651	125620664	4.66667	ENSSSCT00000004170	NEXN	intron	.	911553 rs7894	3
AAG.CTT	6	125620651	125620664	4.66667	ENSSSCT00000004172	FAM73A	intron	.	911553 rs7894	3
AAG.CTT	6	125620651	125620664	4.66667	ENSSSCT00000025176	FAM73A	intron	.	911553 rs7894	3
AT.AT	6	125648126	125648137	6	ENSSSCT00000029114	NEXN	intron	0.173	rs787010838	2
AT.AT	6	125648126	125648137	6	ENSSSCT00000004170	NEXN	intron	0.173	rs787010838	2
AT.AT	6	125648126	125648137	6	ENSSSCT00000004172	FAM73A	intron	0.173	rs787010838	2
AT.AT	6	125648126	125648137	6	ENSSSCT00000025176	FAM73A	intron	0.173	rs787010838	2

AG.CT	6	125833662	125833676	7.5	.	.	intergenic	.	rs787958920	3
AAG.CTT	6	125854388	125854408	7	.	.	intergenic	0.078	784921 rs7934	2
AC.GT	6	126219697	126219715	9.5	ENSSSCT00000004175	AK5	intron	0.065	rs788921795	4
AATC.GATT	6	126507518	126507543	6.5	.	.	intergenic	0.142	522667 rs7876	2
AAG.CTT	6	126763776	126763790	5	.	.	intergenic	0	.	3
AT.AT	6	126873691	126873701	5.5	.	.	intergenic	-0.016	rs787206463	2
AAAAC.GTTTT	6	127202831	127202861	6.2	.	.	intergenic	0.163	135716 rs7921	2
AC.GT	6	127284905	127284917	6.5	.	.	intergenic	-0.091	.	4
AAAAG.CTTTT	6	127395502	127395525	4.8	ENSSSCT00000023892	MSH4	intron	.	981646 rs7885	2
AAAAG.CTTTT	6	127395502	127395525	4.8	ENSSSCT00000004176	MSH4	intron	.	981646 rs7885	2
AG.CT	6	127452830	127452841	6	ENSSSCT00000023892	MSH4	intron	.	.	3
AG.CT	6	127452830	127452841	6	ENSSSCT00000004176	MSH4	intron	.	.	3
AAAC.GTTT	6	127713981	127713993	3.25	ENSSSCT00000004180	SLC44A5	intron	0.146	rs790709400	2
AT.AT	6	128088640	128088649	5	.	.	intergenic	0.329	.	2
AG.CT	6	128244659	128244671	6.5	.	.	intergenic	0.047	rs789479310	2
AT.AT	6	128306895	128306906	6	ENSSSCT00000004183	CRYZ	intron	0.153	968677 rs7899	2
AC.GT	6	128487208	128487229	11	.	.	intergenic	-0.053	rs790104646	2
AAAC.GTTT	6	128608064	128608095	8	.	.	intergenic	-0.006	rs791889768	2
AT.AT	6	128831613	128831624	6	.	.	intergenic	.	rs791830249	2
AC.GT	6	128902905	128902921	8.5	.	.	intergenic	.	rs791307121	4
AT.AT	6	129130342	129130353	6	.	.	intergenic	.	rs792164700	2
AT.AT	6	129141336	129141347	6	.	.	intergenic	.	504066 rs7919	2
AC.GT	6	129157701	129157715	7.5	.	.	intergenic	0.04	.	2
AT.AT	6	129194506	129194516	5.5	.	.	intergenic	-0.13	rs791577059	2
AC.GT	6	129549587	129549601	7.5	.	.	intergenic	-0.125	rs789954303	2
AT.AT	6	129550461	129550472	6	.	.	intergenic	0.318	.	2
AC.GT	6	129582715	129582727	6.5	.	.	intergenic	-0.059	rs693594986	4
ACAT.ATGT	6	129790977	129791003	6.75	.	.	intergenic	0.27	227482 rs7931	2
AAAC.GTTT	6	129855302	129855324	5.75	.	.	intergenic	-0.157	rs196948674	2
AGAT.ATCT	6	129947662	129947682	5.25	.	.	intergenic	.	472668 rs7931	2
AC.GT	6	130012569	130012580	6	.	.	intergenic	-0.093	rs790799676	2
AG.CT	6	130101661	130101672	6	.	.	intergenic	0.199	494423 rs7919	2
AG.CT	6	130206726	130206747	11	.	.	intergenic	.	526037 rs7925	3
AAAAT.ATTTT	6	130446558	130446577	4	.	.	intergenic	-0.055	rs793147530	2
AAAC.GTTT	6	130451897	130451909	3.25	.	.	intergenic	-0.31	254252 rs7870	2
AAAAC.GTTTT	6	130464637	130464655	3.8	.	.	intergenic	0.112	.	2
AG.CT	6	130545694	130545706	6.5	.	.	intergenic	0.05	rs786335034	2
AC.GT	6	130773803	130773813	5.5	ENSSSCT00000031624	NEGR1	intron	-0.158	rs787914706	2
AC.GT	6	130778001	130778014	7	ENSSSCT00000031624	NEGR1	intron	-0.086	219528 rs7862	2
AC.GT	6	130785012	130785036	12.5	ENSSSCT00000031624	NEGR1	intron	0.069	508948 rs7926	2
AT.AT	6	130811514	130811532	9.5	ENSSSCT00000031624	NEGR1	intron	.	rs792580596	2
AAT.ATT	6	131317625	131317639	5	.	.	intergenic	0.107	574244 rs7908	3
AATG.CATT	6	131345936	131345958	5.75	.	.	intergenic	0.059	rs792508496	2
AAG.CTT	6	131671832	131671857	8.66667	.	.	intergenic	-0.112	rs786671145	2
ATC.GAT	6	131921232	131921266	11.6667	.	.	intergenic	0.132	.	2
AAAC.GTTT	6	131948668	131948688	5.25	.	.	intergenic	.	355485 rs7883	2
AC.GT	6	132032014	132032028	7.5	ENSSSCT00000004193	CTH	intron	-0.018	.	4
AT.AT	6	132086337	132086352	8	ENSSSCT00000004194	ANKRD13C	intron	0.311	436296 rs7911	2
AAAC.GTTT	6	132209283	132209302	5	ENSSSCT00000004195	SFRS11	intron	1.993	446003 rs7906	2
AAC.GTT	6	132368858	132368877	6.66667	ENSSSCT00000004197	LRRC7	intron	.	023086 rs7913	2
AAAG.CTTT	6	132535235	132535257	5.75	.	.	intergenic	0.229	330635 rs7921	2
AATG.CATT	6	132613430	132613452	5.75	ENSSSCT00000027842	.	promoter	-0.053	538771 rs7883	2
AC.GT	6	132619192	132619217	13	.	.	intergenic	-0.155	542440 rs7923	5
AAAC.GTTT	6	132910681	132910705	6.25	.	.	intergenic	.	rs793236703	3
AT.AT	6	133154288	133154299	6	.	.	intergenic	.	053235 rs7880	2
ACAC.GTGT	6	133185266	133185290	6.25	.	.	intergenic	0.149	.	2
AC.GT	6	133200140	133200149	5	.	.	intergenic	0.404	rs786793343	2
AAAAT.ATTTT	6	133224407	133224431	5	.	.	intergenic	.	rs791362212	2
AT.AT	6	133224931	133224940	5	.	.	intergenic	.	rs787928544	2
AC.GT	6	133337118	133337138	10.5	.	.	intergenic	.	rs695544273	3
AAAT.ATTT	6	133351039	133351052	3.5	.	.	intergenic	.	rs793836119	2
AT.AT	6	133486113	133486123	5.5	.	.	intergenic	0.001	374650 rs7891	2
AAAG.CTTT	6	133630589	133630612	6	.	.	intergenic	0.573	.	3
AAAT.ATTT	6	133728862	133728880	4.75	.	.	intergenic	0.052	rs791466090	2
AC.GT	6	133809778	133809791	7	ENSSSCT00000004200	GPR177	intron	0.229	rs788661799	2
AC.GT	6	133824447	133824456	5	.	.	intergenic	-0.072	.	2
AAAT.ATTT	6	133943197	133943212	4	.	.	intergenic	.	rs792150133	2
AC.GT	6	134140234	134140250	8.5	ENSSSCT00000032706	IL12RB2	intron	0.204	rs787793444	5
AC.GT	6	134140234	134140250	8.5	ENSSSCT00000004204	IL12RB2	intron	0.204	rs787793444	5
AC.GT	6	134140234	134140250	8.5	ENSSSCT00000036495	IL12RB2	intron	0.204	rs787793444	5
AC.GT	6	134140234	134140250	8.5	ENSSSCT00000032636	IL12RB2	intron	0.204	rs787793444	5
AC.GT	6	134140234	134140250	8.5	ENSSSCT00000032700	IL12RB2	intron	0.204	rs787793444	5
AG.CT	6	134150180	134150193	7	ENSSSCT00000032706	IL12RB2	intron	1.313	386634 rs7898	2
AG.CT	6	134150180	134150193	7	ENSSSCT00000004204	IL12RB2	intron	1.313	386634 rs7898	2
AG.CT	6	134150180	134150193	7	ENSSSCT00000036495	IL12RB2	intron	1.313	386634 rs7898	2
AG.CT	6	134150180	134150193	7	ENSSSCT00000032636	IL12RB2	intron	1.313	386634 rs7898	2
AG.CT	6	134150180	134150193	7	ENSSSCT00000032700	IL12RB2	intron	1.313	386634 rs7898	2
AG.CT	6	134164945	134164954	5	.	.	intergenic	0.065	.	2
AAAC.GTTT	6	134235667	134235682	4	ENSSSCT00000033328	IL23R	intron	0.07	084541 rs7865	2
AAAC.GTTT	6	134235667	134235682	4	ENSSSCT00000033223	IL23R	intron	0.07	084541 rs7865	2
AAAC.GTTT	6	134235667	134235682	4	ENSSSCT00000034103	IL23R	intron	0.07	084541 rs7865	2
AAAC.GTTT	6	134235667	134235682	4	ENSSSCT00000032983	IL23R	intron	0.07	084541 rs7865	2
AAAC.GTTT	6	134235667	134235682	4	ENSSSCT00000004206	IL23R	intron	0.07	084541 rs7865	2
AAAC.GTTT	6	134235667	134235682	4	ENSSSCT00000032866	IL23R	intron	0.07	084541 rs7865	2

AAAC.GTTT	6	134235667	134235682	4	ENSSSCT00000034401	IL23R	intron	0.07	84541 rs7865	2
AAAC.GTTT	6	134235667	134235682	4	ENSSSCT00000034079	IL23R	intron	0.07	84541 rs7865	2
AAAC.GTTT	6	134235667	134235682	4	ENSSSCT00000033490	IL23R	intron	0.07	84541 rs7865	2
AAAC.GTTT	6	134235667	134235682	4	ENSSSCT00000036227	IL23R	intron	0.07	84541 rs7865	2
AAAC.GTTT	6	134235667	134235682	4	ENSSSCT00000033623	IL23R	intron	0.07	84541 rs7865	2
AAAT.ATTT	6	134306042	134306054	3.25	.	.	intergenic	0.288	.	2
AATG.CATT	6	134310908	134310934	6.75	.	.	intergenic	0.026	13993 rs7908	2
AAAC.GTTT	6	134389459	134389478	5	.	.	intergenic	.	rs788894222	3
AT.AT	6	134435595	134435613	9.5	ENSSSCT00000004208	C1orf141	intron	0.023	.	2
AAAAT.ATTTT	6	134440377	134440391	3	ENSSSCT00000004208	C1orf141	intron	0.455	78815 rs7864	2
AAAAC.GTTTT	6	134766536	134766558	3.83333	.	.	intergenic	.	rs789857878	2
ATC.GAT	6	134842114	134842128	5	.	.	intergenic	-0.026	49583 rs7888	2
AC.GT	6	134850229	134850242	7	.	.	intergenic	-0.016	.	3
AAAAC.GTTTT	6	134861122	134861142	4.2	.	.	intergenic	-0.021	70362 rs7916	2
AAC.GTT	6	134885599	134885617	6.33333	ENSSSCT00000004211	PDE4B	intron	0.126	rs787441868	2
AAAC.GTTT	6	134898718	134898733	4	ENSSSCT00000004211	PDE4B	intron	-0.021	.	2
AC.GT	6	135120122	135120133	6	.	.	intergenic	0.069	95692 rs7886	2
AC.GT	6	135331792	135331803	6	.	.	intergenic	0.244	rs789576686	2
ACC.GGT	6	135393235	135393247	4.33333	.	.	intergenic	0.087	.	2
AG.CT	6	135442826	135442836	5.5	ENSSSCT00000004213	DNAJC6	intron	0.059	rs788745474	2
AAAC.GTTT	6	135675406	135675439	8.5	.	.	intergenic	.	rs791352230	2
AAC.GTT	6	135768458	135768474	5.66667	.	.	intergenic	0.023	rs791536133	2
AAAAC.GTTTT	6	135886391	135886409	3.16667	.	.	intergenic	-0.036	47345 rs7924	2
AATT.AATT	6	136154207	136154219	3.25	ENSSSCT00000005331	CACHD1	intron	0.132	rs791580540	2
AG.CT	6	136195495	136195505	5.5	.	.	intergenic	1.01	rs788096544	4
AC.GT	6	136218927	136218936	5	.	.	intergenic	1.522	rs786924098	2
AGAT.ATCT	6	136233518	136233555	9.5	.	.	intergenic	0.093	rs787076610	2
AAT.ATT	6	136327478	136327493	5.33333	.	.	intergenic	0.006	rs787683874	2
AAAAC.GTTTT	6	136575569	136575589	4.2	.	.	intergenic	.	rs793598614	2
AAT.ATT	6	136753075	136753091	5.66667	ENSSSCT00000004217	UBE2U	intron	.	33671 rs7905	2
AATG.CATT	6	137037447	137037469	5.75	.	.	intergenic	-0.137	rs792540804	2
AAAAC.GTTTT	6	137139578	137139598	4.2	.	.	intergenic	-0.034	rs791166823	2
AAAC.GTTT	6	137140255	137140270	4	.	.	intergenic	-0.097	19172 rs7870	2
AAAAC.GTTTT	6	137183923	137183941	3.8	ENSSSCT00000004219	PGM1	intron	0.409	rs790439394	2
AAAT.ATTT	6	137199645	137199667	5.75	ENSSSCT00000004219	PGM1	intron	0.125	rs786680584	2
AATC.GATT	6	137217631	137217655	6.25	ENSSSCT00000004219	PGM1	intron	0.106	rs789229786	2
AG.CT	6	137562328	137562337	5	.	.	intergenic	-0.018	rs786269567	3
AAAT.ATTT	6	137586950	137586966	4.25	.	.	intergenic	0.27	rs790536339	2
AAAC.GTTT	6	137633844	137633862	4.75	.	.	intergenic	-0.236	68083 rs7903	2
AAT.ATT	6	137637185	137637197	4.33333	.	.	intergenic	0.155	.	3
AC.GT	6	137676351	137676363	6.5	.	.	intergenic	-0.001	rs787281539	3
AC.GT	6	138036359	138036369	5.5	.	.	intergenic	0.284	.	2
AT.AT	6	138390729	138390750	11	.	.	intergenic	0.132	rs692133926	2
AC.GT	6	138653593	138653607	7.5	.	.	intergenic	.	rs788613161	2
AC.GT	6	138702228	138702243	8	.	.	intergenic	.	rs790685471	4
AAAT.ATTT	6	138747991	138748009	4.75	.	.	intergenic	-0.094	rs786232921	2
AAAAT.ATTTT	6	139283186	139283202	3.4	.	.	intergenic	0.445	rs786481231	3
AT.AT	6	139492027	139492036	5	.	.	intergenic	-0.26	.	2
AC.GT	6	139499566	139499576	5.5	.	.	intergenic	-0.006	.	2
AT.AT	6	139532666	139532675	5	.	.	intergenic	0.051	rs787739405	2
AAAG.CTTT	6	139727183	139727195	3.25	ENSSSCT00000004232	C1orf87	intron	0.062	.	2
AG.CT	6	139774942	139774956	7.5	.	.	intergenic	0.21	rs791907947	2
AC.GT	6	140083539	140083548	5	.	.	intergenic	0.166	rs693119419	3
AG.CT	6	140150906	140150934	14.5	ENSSSCT00000004235	HOOK1	intron	0.004	77782 rs7882	2
AT.AT	6	140154043	140154053	5.5	ENSSSCT00000004235	HOOK1	intron	0.049	rs793232280	2
AAAT.ATTT	6	140378413	140378434	5.5	ENSSSCT00000004236	.	intron	-0.109	32703 rs7910	2
AAAC.GTTT	6	140445960	140445978	4.75	ENSSSCT00000004237	.	intron	.	rs786730869	4
AC.GT	6	140465145	140465162	9	ENSSSCT00000004237	.	intron	0.859	04930 rs7880	2
AAT.ATT	6	140581475	140581488	4.66667	ENSSSCT00000004237	.	intron	0.12	rs792122379	2
AAC.GTT	6	140581753	140581770	6	ENSSSCT00000004237	.	intron	0.401	27766 rs7904	2
AC.GT	6	140634331	140634345	7.5	ENSSSCT00000004237	.	intron	0.047	rs786990090	2
AAAT.ATTT	6	140668271	140668293	5.75	ENSSSCT00000004237	.	intron	0.371	23881 rs7899	2
AG.CT	6	140734754	140734764	5.5	.	.	intergenic	0.077	rs791375823	2
AC.GT	6	140767849	140767859	5.5	.	.	intergenic	-0.044	rs790364801	2
AT.AT	6	140784833	140784843	5.5	.	.	intergenic	0.063	rs791400936	3
AG.CT	6	140954432	140954443	6	.	.	intergenic	-0.04	rs789777148	3
AAAT.ATTT	6	140969179	140969201	5.75	.	.	intergenic	0.052	87832 rs7901	2
AAAAC.GTTTT	6	141030491	141030519	5.8	.	.	intergenic	-0.008	rs790987255	2
AC.GT	6	141040781	141040804	12	.	.	intergenic	-0.062	64959 rs7917	3
AT.AT	6	141047426	141047452	13.5	.	.	intergenic	-0.208	77467 rs7898	3
AAAAC.GTTTT	6	141281208	141281233	5.2	.	.	intergenic	.	83807 rs7894	2
AC.GT	6	141402348	141402362	7.5	.	.	intergenic	-0.024	42328 rs7862	2
AT.AT	6	141410080	141410089	5	.	.	intergenic	0.171	.	2
AT.AT	6	141419993	141420010	9	.	.	intergenic	0.015	36173 rs7867	3
AAAT.ATTT	6	141620967	141620978	3	.	.	intergenic	-0.018	.	2
ATCC.GGAT	6	141779841	141779861	5.25	.	.	intergenic	0.069	rs788435093	2
AC.GT	6	141917400	141917424	12.5	.	.	intergenic	-0.13	rs789968649	3
AAAC.GTTT	6	142008740	142008758	4.75	.	.	intergenic	-0.156	84148 rs7870	2
AT.AT	6	142009793	142009802	5	.	.	intergenic	-0.131	rs789996030	3
AAAC.GTTT	6	142035255	142035280	6.5	.	.	intergenic	.	rs694109612	2
AATG.CATT	6	142144489	142144502	3.5	.	.	intergenic	.	.	2
AAAG.CTTT	6	142341162	142341188	6.75	.	.	intergenic	0.862	04514 rs7930	2
AAAC.GTTT	6	142375480	142375498	4.75	.	.	intergenic	0.053	21184 rs7912	2
AAAG.CTTT	6	142472804	142472820	4.25	.	.	intergenic	-0.08	rs786231613	3

AAC.GTT	6	142542993	142543031	13	.	.	intergenic	0.071	rs786924206	2
AAAATT.AATTTT	6	142558671	142558691	3.5	.	.	intergenic	0.055	554949 rs7898	2
AATG.CATT	6	142583058	142583075	4.5	.	.	intergenic	-0.009	.	2
AG.CT	6	142626182	142626193	6	.	.	intergenic	-0.174	552587 rs7892	2
AT.AT	6	142677805	142677825	10.5	.	.	intergenic	-0.098	rs790965648	2
AGC.GCT	6	142945594	142945608	5	.	.	intergenic	0.287	rs791322105	2
AC.GT	6	143743220	143743231	6	.	.	intergenic	0.108	.	2
AAAT.ATTT	6	143916331	143916349	4.75	.	.	intergenic	0.027	rs788199323	2
AG.CT	6	144391436	144391446	5.5	.	.	intergenic	.	.	2
AAAT.ATTT	6	144410114	144410128	3.75	.	.	intergenic	.	438241 rs7902	3
AAT.ATT	6	144807787	144807804	6	.	.	intergenic	0.047	rs706766647	2
AT.AT	6	144823953	144823980	14	.	.	intergenic	.	rs787132390	2
AGGGG.CCCCT	6	144910090	144910106	3.4	.	.	intergenic	-0.09	rs792128324	2
AAAAC.GTTTT	6	144994457	144994485	5.8	.	.	intergenic	0.089	rs788721642	2
AAACC.GGTTT	6	145082498	145082515	3.6	.	.	intergenic	-0.102	rs790222114	2
AT.AT	6	145082619	145082644	13	.	.	intergenic	-0.117	rs789002036	2
AAGGG.CCCTT	6	145507517	145507553	6.16667	ENSSSCT00000027891	TMEM61	intron	0.104	584087 rs7873	3
AG.CT	6	145529899	145529913	7.5	.	.	intergenic	-0.03	.	2
AG.CT	6	145534905	145534917	6.5	.	.	intergenic	-0.111	rs791012087	2
AAAC.GTTT	6	145587237	145587251	3.75	.	.	intergenic	.	334627 rs7865	2
AC.GT	6	145864768	145864780	6.5	ENSSSCT00000031990	LRRC42	intron	0.321	.	2
AG.CT	6	145906813	145906824	6	.	.	intergenic	-0.167	rs791081278	2
AAAC.GTTT	6	145948419	145948433	3.75	ENSSSCT00000029087	TCEANC2	intron	0.12	rs791679163	2
AG.CT	6	145980546	145980558	6.5	.	.	intergenic	0.503	.	4
AAAC.GTTT	6	146486778	146486801	6	.	.	intergenic	.	.	2
AAGT.ACTT	6	146522472	146522486	3.75	.	.	intergenic	1.19	rs786329981	2
AT.AT	6	146561038	146561056	9.5	.	.	intergenic	0.166	027228 rs7868	3
AC.GT	6	146586050	146586064	7.5	ENSSSCT00000004260	APOER2	intron	0.439	rs791589891	2
AAAC.GTTT	6	146847237	146847259	5.75	ENSSSCT00000004264	SLC1A7	intron	-0.179	rs787039018	2
AAAT.ATTT	6	147008791	147008806	4	ENSSSCT00000004265	SCP2	intron	0.214	267042 rs7883	2
AATG.CATT	6	147169333	147169355	5.75	ENSSSCT00000004269	.	intron	0.277	401113 rs7897	2
AAAAG.CTTTT	6	147330356	147330385	6	ENSSSCT00000029203	ZCCHC11	intron	0.394	rs786962198	2
AAAAG.CTTTT	6	147330356	147330385	6	ENSSSCT00000004271	ZCCHC11	intron	0.394	rs786962198	2
AAC.GTT	6	147582148	147582163	5.33333	ENSSSCT00000004275	ZFYVE9	intron	0.832	rs790707238	2
AAAG.CTTT	6	147647936	147647961	6.5	ENSSSCT00000004275	ZFYVE9	intron	0.201	rs792940012	2
AAAAG.CTTTT	6	147766683	147766703	4.2	.	.	intergenic	-0.133	rs790370417	2
AAT.ATT	6	147775653	147775676	8	.	.	intergenic	.	.	2
AAAT.ATTT	6	147923005	147923027	5.75	ENSSSCT00000028423	RAB3B	intron	0.11	258526 rs7880	2
AAAT.ATTT	6	147923005	147923027	5.75	ENSSSCT00000026725	RAB3B	intron	0.11	258526 rs7880	2
AAAT.ATTT	6	147939775	147939793	4.75	ENSSSCT00000028423	RAB3B	intron	1.16	rs789947516	3
AAAT.ATTT	6	147939775	147939793	4.75	ENSSSCT00000026725	RAB3B	intron	1.16	rs789947516	3
AAAT.ATTT	6	147941896	147941910	3.75	ENSSSCT00000028423	RAB3B	intron	0.172	.	2
AAAT.ATTT	6	147941896	147941910	3.75	ENSSSCT00000026725	RAB3B	intron	0.172	.	2
AC.GT	6	147943562	147943571	5	ENSSSCT00000026725	RAB3B	intron	2.521	rs786995489	3
AC.GT	6	147943562	147943571	5	ENSSSCT00000028423	RAB3B	intron	2.521	rs786995489	3
AAC.GTT	6	148033456	148033477	7.33333	ENSSSCT00000004282	NRD1	intron	0.259	101904 rs7880	3
AC.GT	6	148162535	148162565	15.5	ENSSSCT00000028073	OSBPL9	intron	.	712608 rs7925	2
AAAC.GTTT	6	148167922	148167948	6.75	ENSSSCT00000028073	OSBPL9	intron	.	rs792679049	2
AAAAAC.GTTTTT	6	148171730	148171753	4	ENSSSCT00000028073	OSBPL9	intron	0.192	rs791822984	2
AG.CT	6	148236263	148236273	5.5	ENSSSCT00000028073	OSBPL9	intron	0.324	253158 rs7867	2
AAAC.GTTT	6	148383981	148383995	3.75	.	.	intergenic	0.234	.	2
AAAAC.GTTTT	6	148648207	148648221	3	.	.	intergenic	0.296	rs788994166	2
AAC.GTT	6	148873736	148873752	5.66667	.	.	intergenic	.	rs790340445	2
AC.GT	6	149030963	149030975	6.5	ENSSSCT00000004289	FAF1	intron	.	.	4
AC.GT	6	149030963	149030975	6.5	ENSSSCT00000036500	FAF1	intron	.	.	4
AT.AT	6	149222188	149222197	5	ENSSSCT00000004289	FAF1	intron	.	.	2
AT.AT	6	149222188	149222197	5	ENSSSCT00000036500	FAF1	intron	.	.	2
AAAAC.GTTTT	6	149817096	149817120	5	.	.	intergenic	0.288	305106 rs7921	2
AAAC.GTTT	6	149821961	149821979	4.75	.	.	intergenic	0.082	.	2
ACC.GGT	6	150015219	150015235	5.66667	.	.	intergenic	0.206	.	2
GAGGG.CCCTC	6	150275344	150275371	4.66667	.	.	intergenic	0.08	580873 rs7915	2
AC.GT	6	150417271	150417283	6.5	.	.	intergenic	0.199	rs701838177	3
AAAC.GTTT	6	150543481	150543500	5	.	.	intergenic	.	rs788941513	2
AG.CT	6	150794812	150794831	10	.	.	intergenic	.	rs790797808	2
AAT.ATT	6	150969175	150969196	7.33333	.	.	intergenic	0.332	rs793212250	2
AAAT.ATTT	6	151062685	151062704	5	.	.	intergenic	.	461469 rs7875	2
AC.GT	6	151332132	151332147	8	.	.	intergenic	0.356	rs786472882	2
AAC.GTT	6	151402929	151402941	4.33333	.	.	intergenic	.	rs791986828	2
AG.CT	6	151705282	151705291	5	.	.	intergenic	-0.054	rs791021559	2
AC.GT	6	151707249	151707259	5.5	.	.	intergenic	1.257	rs789220647	2
AAAAAC.GTTTTT	6	151760713	151760730	3	ENSSSCT00000004299	.	promoter	0.201	346040 rs7889	2
AAG.CTT	6	151854462	151854476	5	.	.	intergenic	0.183	rs792620983	2
AAAG.CTTT	6	151973399	151973425	6.75	ENSSSCT00000024887	.	intron	.	rs792834602	2
AAAG.CTTT	6	151973399	151973425	6.75	ENSSSCT00000022260	CYP4X1	intron	.	rs792834602	2
AG.CT	6	152794076	152794085	5	.	.	intergenic	0.991	.	2
AAAAAC.GTTTTT	6	152795203	152795232	5	.	.	intergenic	1.046	592279 rs7924	2
AAC.GTT	6	153031251	153031264	4.66667	.	.	intergenic	.	785058 rs7912	2
AAAC.GTTT	6	153164183	153164203	5.25	.	.	intergenic	.	rs786943066	2
AAAAC.GTTTT	6	153362411	153362433	4.6	ENSSSCT00000004332	TESK2	intron	.	061095 rs7876	2
AAG.CTT	6	153532416	153532429	4.66667	.	.	intergenic	0.288	rs788382426	2
AAAC.GTTT	6	153672127	153672145	4.75	.	.	intergenic	.	rs792526266	2
AT.AT	6	153857564	153857574	5.5	ENSSSCT00000022870	EIF2B3	intron	0.249	rs788134555	2
AG.CT	6	153870834	153870850	8.5	ENSSSCT00000022870	EIF2B3	intron	1.387	rs709800826	2
AAAG.CTTT	6	154002722	154002734	3.25	ENSSSCT00000030222	TMEM53	intron	0.168	rs791633225	2

AAAG.CTTT	6	154002722	154002734	3.25	ENSSSCT00000004347	KIF2C	intron	0.168	rs791633225	2
AC.GT	6	154248484	154248494	5.5	.	.	intergenic	0.028	.	2
AT.AT	6	154338369	154338387	9.5	.	.	intergenic	-0.17	256890 rs7910	2
AC.GT	6	154373225	154373238	7	.	.	intergenic	.	rs788735344	4
AAAC.GTTT	6	154824657	154824677	5.25	.	.	intergenic	0.098	rs787980239	2
AG.CT	6	154918448	154918462	7.5	ENSSSCT00000026482	.	intron	0.029	rs791695123	2
AAAC.GTTT	6	155382385	155382408	6	.	.	intergenic	.	rs788529566	3
AAAAG.CTTTT	6	155559175	155559200	5.2	.	.	intergenic	.	rs789946111	3
AAAC.GTTT	6	155891468	155891490	5.75	.	.	intergenic	-0.082	rs793010068	2
AAGG.CCTT	6	156052678	156052696	4.75	ENSSSCT00000023375	ERMAP	intron	.	364712 rs7864	2
AAC.GTT	6	156056255	156056271	5.66667	ENSSSCT00000023375	ERMAP	intron	.	rs791867563	2
ACAG.CTGT	6	156110443	156110461	4.75	ENSSSCT00000023375	ERMAP	intron	0.09	rs786742833	2
AAAAC.GTTTT	6	156142403	156142417	3	.	.	intergenic	0.258	929423 rs7905	2
AC.GT	6	156220284	156220296	6.5	.	.	intergenic	0.198	rs787485613	4
AAAC.GTTT	6	156369860	156369878	4.75	ENSSSCT00000004388	ZMYND12	intron	0.042	940968 rs7920	2
AC.GT	6	157304284	157304295	6	.	.	intergenic	.	.	2
AAAC.GTTT	6	157704796	157704826	7.75	ENSSSCT00000004405	SMAP1L	intron	.	962918 rs7902	2
AC.GT	7	141486	141495	5	ENSSSCT00000001075	EXOC2	intron	.	rs786439632	3
AC.GT	7	1333772	1333787	8	ENSSSCT00000001084	GMDS	intron	0.55	rs793576909	2
AT.AT	7	1406746	1406755	5	.	.	intergenic	0.058	rs786481894	2
AG.CT	7	1669546	1669557	6	.	.	intergenic	0.014	rs789839908	4
AAAAAC.GTTTT	7	2584410	2584439	5	.	.	intergenic	.	rs791686994	2
AAC.GTT	7	2745136	2745149	4.66667	.	.	intergenic	0.59	rs787563117	2
AAAT.ATTT	7	3478394	3478409	4	ENSSSCT00000001108	.	intron	0.043	rs793115973	2
AAC.GTT	7	4031400	4031421	7.33333	ENSSSCT00000035671	F13A1	intron	-0.207	rs790658620	2
AC.GT	7	4048436	4048470	17.5	ENSSSCT00000035671	F13A1	intron	-0.072	rs787446570	4
AAAC.GTTT	7	4091287	4091301	3.75	ENSSSCT00000035671	F13A1	intron	-0.032	.	2
AC.GT	7	4137935	4137956	11	.	.	intergenic	0.616	rs790993589	2
AG.CT	7	4154815	4154838	12	.	.	intergenic	.	543425 rs7874	4
AAAACC.GGTTT	7	4506722	4506740	3.16667	.	.	intergenic	0.39	rs789392018	2
AAAT.ATTT	7	4844338	4844354	4.25	.	.	intergenic	.	.	3
AAAC.GTTT	7	5131967	5131985	4.75	.	.	intergenic	0.463	396336 rs7897	4
AC.GT	7	5410383	5410393	5.5	ENSSSCT00000001123	.	intron	0.124	rs790644361	3
AC.GT	7	5432175	5432185	5.5	ENSSSCT00000027153	.	intron	.	rs793022513	3
AAAAC.GTTTT	7	5452038	5452057	4	ENSSSCT00000027153	.	intron	-0.014	rs787569966	2
AAAT.ATTT	7	5489363	5489379	4.25	.	.	intergenic	0.127	404015 rs7937	2
AAAC.GTTT	7	5808764	5808782	4.75	ENSSSCT00000001126	SLC35B3	intron	0.106	rs792951049	2
AG.CT	7	5835231	5835242	6	.	.	intergenic	-0.113	rs788258949	2
AG.CT	7	5857323	5857333	5.5	.	.	intergenic	-0.098	rs786931576	2
AAAT.ATTT	7	5980520	5980540	5.25	.	.	intergenic	.	.	2
AG.CT	7	6004186	6004198	6.5	.	.	intergenic	-0.218	.	2
AC.GT	7	6090436	6090450	7.5	.	.	intergenic	.	rs790572046	2
AAT.ATT	7	6150514	6150525	4	.	.	intergenic	0.026	rs791444549	2
AC.GT	7	6237781	6237791	5.5	.	.	intergenic	0.022	.	4
AT.AT	7	6343089	6343100	6	.	.	intergenic	0.042	rs788401865	4
AC.GT	7	6497391	6497403	6.5	.	.	intergenic	0.347	rs690642460	4
AC.GT	7	6559133	6559145	6.5	.	.	intergenic	-0.277	.	2
AAAAT.ATTTT	7	6575635	6575660	5.2	.	.	intergenic	-0.003	348879 rs7913	2
AGAT.ATCT	7	6681731	6681748	4.5	.	.	intergenic	.	095198 rs7932	2
AG.CT	7	6689599	6689611	6.5	.	.	intergenic	-0.24	.	2
AC.GT	7	6708791	6708809	9.5	.	.	intergenic	.	084847 rs7897	2
AC.GT	7	6847369	6847403	17.5	.	.	intergenic	0.341	rs792419482	2
AC.GT	7	6901035	6901045	5.5	.	.	intergenic	-0.011	rs789110753	2
AAC.GTT	7	6924080	6924095	5.33333	.	.	intergenic	0.257	965975 rs7867	2
AT.AT	7	7051357	7051366	5	ENSSSCT00000001128	OFCC1	intron	0.194	rs787311678	2
AT.AT	7	7051357	7051366	5	ENSSSCT00000022619	OFCC1	intron	0.194	rs787311678	2
AC.GT	7	7076877	7076887	5.5	.	.	intergenic	-0.165	rs788230583	2
AAAC.GTTT	7	7101060	7101072	3.25	.	.	intergenic	-0.033	rs787209823	2
AAAC.GTTT	7	7115576	7115603	7	.	.	intergenic	0.497	444574 rs7912	2
AAG.CTT	7	7123813	7123834	7.33333	.	.	intergenic	2.922	366051 rs7898	2
AAT.ATT	7	7143716	7143739	8	.	.	intergenic	.	rs790908903	2
AGG.CCT	7	7542088	7542109	7.33333	ENSSSCT00000001129	TFAP2A	intron	0.433	.	3
AT.AT	7	7667644	7667655	6	.	.	intergenic	.	rs789868933	2
AAG.CTT	7	7703272	7703285	4.66667	ENSSSCT00000001133	GCNT2	intron	0.09	rs787400968	2
AAAC.GTTT	7	7745888	7745902	3.75	ENSSSCT00000001134	C6orf52	intron	0.056	rs791917522	2
AGG.CCT	7	8015620	8015639	6.66667	ENSSSCT00000001139	ELOVL2	promoter	0.062	rs787242743	2
AC.GT	7	8336332	8336343	6	.	.	intergenic	-0.022	rs788681979	4
AC.GT	7	8376005	8376018	7	.	.	intergenic	0.508	rs787073395	4
AAAAAC.GTTTT	7	8608864	8608882	3.16667	ENSSSCT00000001142	.	intron	0.27	737443 rs7876	2
AC.GT	7	8622949	8622961	6.5	ENSSSCT00000001142	.	intron	-0.312	.	3
AGC.GCT	7	8720697	8720711	5	ENSSSCT00000001141	.	intron	.	.	3
AAAC.GTTT	7	8940167	8940179	3.25	ENSSSCT00000001143	HIVEP1	intron	-0.169	.	2
AAAAC.GTTTT	7	9043885	9043915	6.2	ENSSSCT00000001143	HIVEP1	intron	0.059	087916 rs7911	2
AAAC.GTTT	7	9318041	9318070	7.5	.	.	intergenic	0.004	726966 rs7919	2
AC.GT	7	9518712	9518725	7	ENSSSCT00000001146	.	intron	0.266	481231 rs7925	2
AAAAC.GTTTT	7	9533420	9533442	4.6	ENSSSCT00000001146	.	intron	0.041	rs786807563	2
AT.AT	7	9778083	9778092	5	ENSSSCT00000001146	.	intron	0.056	rs788338746	2
AC.GT	7	9799685	9799696	6	ENSSSCT00000001146	.	intron	0.189	rs792092349	2
AT.AT	7	10295222	10295232	5.5	ENSSSCT00000001150	RANBP9	intron	0.077	rs788455633	3
AAAAC.GTTTT	7	10508926	10508946	4.2	.	.	intergenic	0.016	446755 rs7893	2
AAAT.ATTT	7	10547710	10547732	5.75	.	.	intergenic	0.33	rs787864141	2
AAAT.ATTT	7	10553874	10553900	6.75	ENSSSCT00000001151	MCUR1	intron	-0.17	927776 rs7909	2
AC.GT	7	10697717	10697728	6	ENSSSCT00000033515	CD83	intron	0.189	.	2
AAAAC.GTTTT	7	10739020	10739054	7	ENSSSCT00000033515	CD83	intron	0.012	rs788033772	2

AC.GT	7	10786245	10786254	5	ENSSSCT00000033515	CD83	intron	-0.099	rs789670384	2
AAC.GTT	7	11346345	11346364	6.66667	.	.	intergenic	0.097	rs790192069	2
AG.CT	7	11490878	11490894	8.5	.	.	intergenic	0.058	rs789136086	2
AAAT.ATTT	7	11611882	11611900	4.75	.	.	intergenic	.	)66358 rs7907	2
\AAAAG.CTTTT'	7	11784381	11784402	3.66667	.	.	intergenic	.	rs792869996	2
AAAC.GTTT	7	11829126	11829140	3.75	.	.	intergenic	-0.02	rs786542311	2
AAC.GTT	7	12204856	12204881	8.66667	ENSSSCT00000001157	DTNBP1	intron	-0.531	rs786478051	2
AAT.ATT	7	12414854	12414875	7.33333	.	.	intergenic	0.29	.	2
AAAC.GTTT	7	12416480	12416502	5.75	.	.	intergenic	0.067	.	2
AAAAC.GTTTT	7	12465578	12465602	5	.	.	intergenic	-0.007	.	2
AAAC.GTTT	7	12591077	12591092	4	.	.	intergenic	-0.101	rs787683444	2
ACC.GGT	7	12637988	12638002	5	.	.	intergenic	-0.07	.	2
AAAG.CTTT	7	12664395	12664411	4.25	.	.	intergenic	-0.046	rs793545452	3
AAAAT.ATTTT	7	12685053	12685081	5.8	.	.	intergenic	-0.138	rs789074809	2
AC.GT	7	12766383	12766395	6.5	.	.	intergenic	-0.024	rs788872084	2
AACC.GGTT	7	12849643	12849663	5.25	ENSSSCT00000001159	GMPR	promoter	0.089	.	2
AG.CT	7	12917192	12917201	5	ENSSSCT00000001160	ATXN1	intron	0.383	rs792510451	2
AT.AT	7	13008037	13008048	6	.	.	intergenic	-0.292	rs790438140	2
AAAT.ATTT	7	13211651	13211664	3.5	.	.	intergenic	-0.048	rs789514160	2
AG.CT	7	13216669	13216679	5.5	.	.	intergenic	0.222	rs791619677	2
AAAAG.CTTTT	7	13413337	13413359	4.6	.	.	intergenic	0.236	rs788567552	2
AAT.ATT	7	13632626	13632637	4	.	.	intergenic	0.048	rs792019751	2
AT.AT	7	13644961	13644973	6.5	.	.	intergenic	.	rs702721440	2
AATC.GATT	7	13713501	13713528	7	.	.	intergenic	0.024	.	2
AC.GT	7	13773624	13773646	11.5	.	.	intergenic	-0.318	rs787259881	4
AAAC.GTTT	7	13795499	13795519	5.25	.	.	intergenic	0.017	rs787137628	2
AT.AT	7	14016199	14016208	5	ENSSSCT00000001163	.	intron	-0.05	.	4
AAAC.GTTT	7	14028128	14028142	3.75	ENSSSCT00000001163	.	intron	-0.168	rs790323091	3
AC.GT	7	14033920	14033931	6	ENSSSCT00000001163	.	intron	0.414	.	2
AAT.ATT	7	14060161	14060185	8.33333	.	.	intergenic	-0.19	222734 rs7921	2
AAAG.CTTT	7	14074326	14074341	4	.	.	intergenic	.	rs786289802	2
AAAC.GTTT	7	14084643	14084661	4.75	ENSSSCT00000001165	FAM8A1	intron	.	286458 rs7883	2
AG.CT	7	14312392	14312403	6	.	.	intergenic	0.158	.	2
AT.AT	7	14312588	14312597	5	.	.	intergenic	0.134	.	3
AAAAG.CTTTT	7	14448421	14448450	6	.	.	intergenic	0.141	rs791901855	2
ACAG.CTGT	7	14567486	14567508	5.75	ENSSSCT00000001168	TPMT	intron	-0.177	'59863 rs7901	2
AAAT.ATTT	7	14725008	14725026	4.75	.	.	intergenic	.	rs786551428	2
AG.CT	7	14798617	14798659	21.5	ENSSSCT00000001171	RNF144B	intron	0.278	.	3
AC.GT	7	14799316	14799325	5	ENSSSCT00000001171	RNF144B	intron	0.514	rs792671403	2
AC.GT	7	14863193	14863207	7.5	.	.	intergenic	0.142	rs790388552	2
AG.CT	7	15138452	15138462	5.5	.	.	intergenic	-0.043	rs691623607	2
\AAAAC.GTTTT'	7	15293372	15293394	3.83333	.	.	intergenic	0.231	rs792378113	2
AAAT.ATTT	7	15357283	15357304	5.5	.	.	intergenic	-0.004	.	4
ACAG.CTGT	7	15466234	15466245	3	.	.	intergenic	-0.114	.	2
AT.AT	7	15524752	15524763	6	.	.	intergenic	0.12	rs690528146	4
AG.CT	7	15613852	15613867	8	.	.	intergenic	.	rs706772144	2
ACAG.CTGT	7	15696501	15696523	5.75	.	.	intergenic	.	rs786741496	2
AAAT.ATTT	7	15727715	15727733	4.75	.	.	intergenic	.	)56415 rs7890	2
AAAC.GTTT	7	15955463	15955480	4.5	.	.	intergenic	0.201	rs791464361	2
AC.GT	7	16078644	16078661	9	.	.	intergenic	.	.	2
AG.CT	7	16172633	16172642	5	.	.	intergenic	0.07	.	2
AAC.GTT	7	16355848	16355870	7.66667	.	.	intergenic	3.405	;81033 rs7906	2
AAAC.GTTT	7	16483057	16483076	5	ENSSSCT00000001173	MBOAT1	intron	.	.	2
ATCC.GGAT	7	16509195	16509213	4.75	ENSSSCT00000001173	MBOAT1	intron	.	rs793468022	2
AAAAT.ATTTT	7	16524476	16524497	4.4	ENSSSCT00000001173	MBOAT1	intron	-0.01	rs789357960	2
AAAT.ATTT	7	16672568	16672583	4	.	.	intergenic	0.161	rs793172800	2
AAAT.ATTT	7	16680507	16680520	3.5	.	.	intergenic	-0.039	rs790209621	2
AC.GT	7	16719914	16719923	5	.	.	intergenic	-0.181	)29182 rs7900	4
AAAT.ATTT	7	16760412	16760448	9.25	.	.	intergenic	0.336	rs793344903	2
AC.GT	7	16830665	16830676	6	ENSSSCT00000001175	CDKAL1	intron	0.184	rs790353822	2
AG.CT	7	17025319	17025331	6.5	ENSSSCT00000001175	CDKAL1	intron	-0.105	rs788267200	2
AAC.GTT	7	17163453	17163469	5.66667	ENSSSCT00000001175	CDKAL1	intron	0.749	;40639 rs7876	3
AAAAT.ATTTT	7	17191305	17191329	5	ENSSSCT00000001175	CDKAL1	intron	.	355331 rs7890	2
AG.CT	7	17530672	17530688	8.5	.	.	intergenic	.	.	4
AGG.CCT	7	17808718	17808736	6.33333	.	.	intergenic	0.037	rs791498373	2
\AAAAG.CTTTT'	7	17837621	17837642	3.66667	.	.	intergenic	-0.024	rs788312506	2
AAGG.CCTT	7	18156300	18156317	4.5	.	.	intergenic	0.095	rs786219103	2
AAAC.GTTT	7	18198629	18198651	5.75	.	.	intergenic	-0.282	rs786871935	2
AC.GT	7	18215943	18215955	6.5	.	.	intergenic	0.242	rs786715761	2
AC.GT	7	18278150	18278174	12.5	.	.	intergenic	-0.175	rs793074234	3
AC.GT	7	18407511	18407525	7.5	.	.	intergenic	0.054	rs791254620	2
AT.AT	7	18548909	18548921	6.5	.	.	intergenic	-0.035	rs695308618	4
AAAC.GTTT	7	18553887	18553904	4.5	.	.	intergenic	0.415	rs789893385	2
AAC.GTT	7	18588213	18588231	6.33333	.	.	intergenic	-0.127	455692 rs7937	2
AT.AT	7	18623423	18623439	8.5	.	.	intergenic	0.076	rs787043206	2
AC.GT	7	18643615	18643624	5	.	.	intergenic	-0.096	382976 rs7862	4
AG.CT	7	18736467	18736492	13	.	.	intergenic	-0.006	441216 rs7921	2
AATG.CATT	7	18814139	18814150	3	.	.	intergenic	0.111	.	3
ACAT.ATGT	7	18858781	18858802	5.5	.	.	intergenic	-0.19	rs786524988	2
AG.CT	7	18934479	18934507	14.5	.	.	intergenic	-0.106	rs792514928	2
AAAC.GTTT	7	19119708	19119723	4	.	.	intergenic	.	rs791086713	2
AG.CT	7	19252430	19252441	6	.	.	intergenic	-0.125	rs787706122	2
AAAC.GTTT	7	19771893	19771905	3.25	.	.	intergenic	0.126	rs791020210	2
AC.GT	7	19963387	19963407	10.5	.	.	intergenic	0.033	rs793491993	2

AAG.CTT	7	20028740	20028754	5	.	.	intergenic	.	rs787559250	2
AG.CT	7	20085383	20085395	6.5	.	.	intergenic	-0.027	.	4
AAAAC.GTTTT	7	20179329	20179348	4	.	.	intergenic	.	rs788934363	2
AC.GT	7	20195229	20195239	5.5	.	.	intergenic	0.072	rs788112684	2
AAAAC.GTTTT	7	20333175	20333190	3.2	ENSSSCT00000033956	DCDC2	intron	-0.278	.	3
AAAAC.GTTTT	7	20333175	20333190	3.2	ENSSSCT00000001180	DCDC2	intron	-0.278	.	3
AAAAC.GTTTT	7	20333175	20333190	3.2	ENSSSCT00000022214	5S_rRNA	promoter	-0.278	.	3
AAAAG.CTTTT	7	20346143	20346158	3.2	ENSSSCT00000033956	DCDC2	intron	0.106	rs792205789	2
AAAAG.CTTTT	7	20346143	20346158	3.2	ENSSSCT00000001180	DCDC2	intron	0.106	rs792205789	2
AC.GT	7	20353021	20353031	5.5	ENSSSCT00000033956	DCDC2	intron	0.104	rs702350524	2
AC.GT	7	20353021	20353031	5.5	ENSSSCT00000001180	DCDC2	intron	0.104	rs702350524	2
AC.GT	7	20398625	20398647	11.5	ENSSSCT00000001183	MRS2	intron	0.034	941571 rs7906	2
AC.GT	7	20398625	20398647	11.5	ENSSSCT00000019917	SNORA11	promoter	0.034	941571 rs7906	2
AC.GT	7	20413967	20413977	5.5	ENSSSCT00000001183	MRS2	intron	.	rs793666164	2
AT.AT	7	20484802	20484814	6.5	ENSSSCT00000001185	GPLD1	intron	-0.087	rs793387443	2
AAAAC.GTTTT	7	20632025	20632049	5	.	.	intergenic	0.089	306321 rs7880	2
AAAC.GTTT	7	20854796	20854809	3.5	ENSSSCT00000001193	FAM65B	intron	0.401	234407 rs7900	2
AT.AT	7	20923331	20923341	5.5	.	.	intergenic	-0.025	rs790602223	3
AAAAC.GTTTT	7	20928477	20928496	4	.	.	intergenic	0.243	rs793858319	3
AAT.ATT	7	20963840	20963853	4.66667	.	.	intergenic	.	.	2
AATC.GATT	7	21135552	21135579	7	.	.	intergenic	.	rs787593181	2
AT.AT	7	21297140	21297153	7	.	.	intergenic	0.068	rs790417878	2
AAC.GTT	7	21529494	21529522	9.66667	ENSSSCT00000001196	LRRC16A	intron	0.131	rs712248884	4
AC.GT	7	21530008	21530019	6	ENSSSCT00000001196	LRRC16A	intron	0.888	754523 rs7901	2
AAAC.GTTT	7	21958348	21958382	8.75	.	.	intergenic	-0.169	468779 rs7920	2
AAC.GTT	7	22076620	22076633	4.66667	.	.	intergenic	-0.024	534780 rs7896	2
AAAAC.GTTTT	7	22406727	22406750	4.8	.	.	intergenic	.	206016 rs7911	2
AAAC.GTTT	7	22496594	22496616	5.75	.	.	intergenic	.	rs792390872	2
AAC.GTT	7	22497519	22497532	4.66667	.	.	intergenic	.	rs786760082	2
AAAC.GTTT	7	22984362	22984383	5.5	.	.	intergenic	0.019	026477 rs7914	2
AAAC.GTTT	7	23201303	23201329	6.75	.	.	intergenic	.	rs786313812	2
AAAG.CTTT	7	23218063	23218096	8.5	.	.	intergenic	.	rs786816087	2
AGG.CCT	7	23292184	23292202	6.33333	.	.	intergenic	.	592268 rs7879	2
AG.CT	7	23328086	23328102	8.5	.	.	intergenic	.	.	2
AAT.ATT	7	24035365	24035376	4	ENSSSCT00000001302	.	promoter	0.338	.	2
AAAG.CTTT	7	24193472	24193509	9.5	.	.	intergenic	.	398431 rs7878	2
AAAT.ATTT	7	24206070	24206093	6	.	.	intergenic	-0.194	395276 rs7935	2
AAAC.GTTT	7	24207644	24207657	3.5	.	.	intergenic	-0.129	rs787823562	2
AT.AT	7	24453527	24453537	5.5	ENSSSCT00000001317	TRIM27	promoter	.	.	4
AAAT.ATTT	7	24824478	24824515	9.5	.	.	intergenic	.	rs704523262	2
AC.GT	7	25386286	25386307	11	ENSSSCT00000030258	U6	promoter	.	rs791148392	4
AT.AT	7	26308177	26308189	6.5	.	.	intergenic	.	509324 rs7915	2
AC.GT	7	26767113	26767125	6.5	.	.	intergenic	.	rs791851405	2
AC.GT	7	26989700	26989716	8.5	ENSSSCT00000034652	IER3	promoter	0.017	rs790175622	2
AC.GT	7	26989700	26989716	8.5	ENSSSCT00000031071	IER3	promoter	0.017	rs790175622	2
AT.AT	7	27324551	27324562	6	.	.	intergenic	.	rs793204991	2
AAACC.GGTTT	7	27330337	27330365	5.8	.	.	intergenic	.	295739 rs7930	2
AC.GT	7	27342765	27342784	10	.	.	intergenic	.	144196 rs7864	2
AAC.GTT	7	27612952	27612975	8	ENSSSCT00000034604	SLA-7	promoter	-0.874	540367 rs7865	2
AAC.GTT	7	27612952	27612975	8	ENSSSCT00000034735	SLA-7	promoter	-0.874	540367 rs7865	2
AAC.GTT	7	27612952	27612975	8	ENSSSCT00000035823	SLA-7	promoter	-0.874	540367 rs7865	2
AAC.GTT	7	27612952	27612975	8	ENSSSCT00000001524	SLA-7	promoter	-0.874	540367 rs7865	2
AAC.GTT	7	27612952	27612975	8	ENSSSCT00000001336	SLA-7	promoter	-0.874	540367 rs7865	2
AAC.GTT	7	27612952	27612975	8	ENSSSCT00000035331	SLA-7	promoter	-0.874	540367 rs7865	2
AAAG.CTTT	7	27731115	27731133	4.75	ENSSSCT00000001570	C4A	intron	-0.296	378311 rs7896	2
AC.GT	7	28033052	28033066	7.5	ENSSSCT00000001583	AGPAT1	intron	-0.001	rs789711840	3
AC.GT	7	28033052	28033066	7.5	ENSSSCT00000035347	AGPAT1	intron	-0.001	rs789711840	3
AC.GT	7	28033052	28033066	7.5	ENSSSCT00000036330	AGPAT1	intron	-0.001	rs789711840	3
AC.GT	7	28033052	28033066	7.5	ENSSSCT00000033826	AGPAT1	intron	-0.001	rs789711840	3
AC.GT	7	28033052	28033066	7.5	ENSSSCT00000035535	AGPAT1	intron	-0.001	rs789711840	3
AC.GT	7	28033052	28033066	7.5	ENSSSCT00000033020	AGPAT1	intron	-0.001	rs789711840	3
AC.GT	7	29180254	29180264	5.5	ENSSSCT00000034363	SLA-DQB2	promoter	-0.079	097584 rs7936	2
AC.GT	7	29918137	29918148	6	ENSSSCT00000001649	BMP5	intron	0.202	581700 rs7877	2
AAG.CTT	7	30021904	30021917	4.66667	.	.	intergenic	0.577	768426 rs7871	2
AG.CT	7	30047304	30047318	7.5	.	.	intergenic	.	rs693921366	4
AAAC.GTTT	7	30107579	30107605	6.75	.	.	intergenic	0.024	774541 rs7893	2
ACT.AGT	7	30496872	30496913	14	.	.	intergenic	-0.041	436151 rs7887	2
AAAAAG.CTTTTT	7	30611200	30611223	4	ENSSSCT00000001655	FAM83B	promoter	0.01	rs790534615	2
AG.CT	7	30667308	30667322	7.5	.	.	intergenic	0.083	.	3
AAAT.ATTT	7	30687473	30687495	5.75	.	.	intergenic	0.025	.	2
AT.AT	7	30729590	30729600	5.5	.	.	intergenic	.	rs788006516	2
ACACAG.CTGTG	7	30815255	30815276	3.66667	.	.	intergenic	0.036	rs793290575	2
AAAC.GTTT	7	30930684	30930712	7.25	.	.	intergenic	0.063	rs788175492	2
AC.GT	7	31035723	31035732	5	.	.	intergenic	.	rs691303695	2
AT.AT	7	31178650	31178661	6	.	.	intergenic	.	rs693563455	2
AC.GT	7	31217113	31217123	5.5	.	.	intergenic	-0.086	rs786307573	3
AAG.CTT	7	31338561	31338574	4.66667	ENSSSCT00000001658	LRRC1	intron	-0.018	rs791129679	2
AAG.CTT	7	31338561	31338574	4.66667	ENSSSCT00000035077	LRRC1	intron	-0.018	rs791129679	2
AAAC.GTTT	7	31345310	31345324	3.75	ENSSSCT00000001658	LRRC1	intron	1.204	591858 rs7863	2
AAAC.GTTT	7	31345310	31345324	3.75	ENSSSCT00000035077	LRRC1	intron	1.204	591858 rs7863	2
AAG.CTT	7	31364950	31364978	9.66667	ENSSSCT00000001658	LRRC1	intron	0.133	rs787503452	2
AT.AT	7	31475386	31475396	5.5	.	.	intergenic	.	rs791843000	2
AAAT.ATTT	7	31672571	31672588	4.5	.	.	intergenic	0.197	.	2
AC.GT	7	31927718	31927729	6	.	.	intergenic	-0.035	124323 rs7908	2

AG.CT	7	32003793	32003804	6	ENSSSCT00000001662	KHDRBS2	intron	0.032	rs708215496	3
AAAAT.ATTTT	7	32251260	32251278	3.8	ENSSSCT00000001662	KHDRBS2	intron	0.307	rs792943635	2
AAAG.CTTT	7	32282712	32282740	7.25	ENSSSCT00000001662	KHDRBS2	intron	0.006	rs793623095	2
AAC.GTT	7	32283886	32283897	4	ENSSSCT00000001662	KHDRBS2	intron	0.074	.	2
AAAAC.GTTTT	7	32287437	32287470	6.8	ENSSSCT00000001662	KHDRBS2	intron	0.024	rs790538024	3
AAAC.GTTT	7	32606104	32606129	6.5	.	.	intergenic	0.036	438071 rs7902	2
AC.GT	7	32956636	32956647	6	ENSSSCT00000001665	.	intron	.	rs790137445	3
AAAC.GTTT	7	33085156	33085170	3.75	.	.	intergenic	0.159	rs789452636	2
AT.AT	7	33152415	33152427	6.5	ENSSSCT00000001669	ZNF451	intron	.	.	2
AAAC.GTTT	7	33296481	33296493	3.25	ENSSSCT00000027614	.	intron	0.042	rs787568404	2
AC.GT	7	33300385	33300397	6.5	ENSSSCT00000027614	.	intron	0.272	rs789053295	3
AAAAT.ATTTT	7	33363364	33363396	6.6	ENSSSCT00000027614	.	intron	-0.218	rs793153182	2
AAAT.ATTT	7	33931712	33931732	5.25	ENSSSCT00000001673	COL21A1	intron	0.508	rs789082287	2
AAAGC.GCTTT	7	33992799	33992820	3.66667	ENSSSCT00000001673	COL21A1	intron	2.507	.	2
ATC.GAT	7	34019373	34019388	5.33333	.	.	intergenic	.	rs788263076	2
AC.GT	7	34162365	34162380	8	ENSSSCT00000001679	RGL2	intron	0.698	rs789752821	2
AC.GT	7	34196564	34196580	8.5	ENSSSCT00000001683	.	promoter	.	411726 rs7921	2
AT.AT	7	34525207	34525219	6.5	ENSSSCT00000030529	UQCC2	intron	.	rs789596352	2
AATG.CATT	7	34875026	34875053	7	ENSSSCT00000001696	GRM4	intron	-0.067	380977 rs7905	2
AT.AT	7	35514892	35514913	11	ENSSSCT00000001707	UHRF1BP1	intron	0.152	563152 rs7903	2
AAAAC.GTTTT	7	36150545	36150568	4.8	ENSSSCT00000001714	PPARD	intron	.	rs792933705	2
AAAC.GTTT	7	36307815	36307827	3.25	.	.	intergenic	.	rs793712938	2
AAAC.GTTT	7	36309169	36309185	4.25	.	.	intergenic	0.042	917874 rs7887	2
AT.AT	7	36332078	36332087	5	.	.	intergenic	0.289	rs789234251	3
AC.GT	7	36501047	36501063	8.5	.	.	intergenic	0.073	rs787270603	2
AC.GT	7	37241843	37241858	8	.	.	intergenic	-0.314	508771 rs7878	2
AG.CT	7	37392954	37392963	5	ENSSSCT00000001746	CPNE5	intron	-0.242	rs791538642	2
AC.GT	7	37450177	37450190	7	ENSSSCT00000028714	PPIL1	promoter	.	rs791513936	2
AC.GT	7	37450177	37450190	7	ENSSSCT00000001748	C6orf89	promoter	.	rs791513936	2
AG.CT	7	37462933	37462942	5	ENSSSCT00000001748	C6orf89	intron	2.163	rs787595822	3
AT.AT	7	37957294	37957303	5	ENSSSCT00000001758	.	intron	.	rs791495289	3
AAC.GTT	7	38019424	38019451	9.33333	.	.	intergenic	-0.073	.	3
AAAC.GTTT	7	38064266	38064280	3.75	.	.	intergenic	.	rs694927431	3
AAC.GTT	7	38212286	38212302	5.66667	.	.	intergenic	0.24	932366 rs7903	2
ACAT.ATGT	7	38850068	38850090	5.75	ENSSSCT00000001764	.	intron	0.046	rs792516526	2
AC.GT	7	38934485	38934498	7	.	.	intergenic	0.075	.	3
AC.GT	7	38982481	38982493	6.5	ENSSSCT00000026749	BTBD9	intron	0.131	349742 rs7882	2
AAAAC.GTTTT	7	39374338	39374362	5	ENSSSCT00000001768	DNAH8	intron	0.056	217261 rs7865	2
AT.AT	7	39448720	39448736	8.5	ENSSSCT00000001768	DNAH8	intron	-0.096	rs792135154	3
AAAC.GTTT	7	39466142	39466172	7.75	ENSSSCT00000001768	DNAH8	intron	2.025	991472 rs7930	2
AAAAC.GTTTT	7	39489154	39489173	4	ENSSSCT00000001768	DNAH8	intron	-0.271	rs788186435	2
AT.AT	7	39603051	39603062	6	ENSSSCT00000001769	GLP1R	intron	-0.157	rs698989796	2
AAAT.ATTT	7	39645056	39645074	4.75	.	.	intergenic	0.129	973396 rs7889	2
AAAC.GTTT	7	39661167	39661181	3.75	.	.	intergenic	.	rs786623952	2
AAAAC.GTTTT	7	39662418	39662432	3	.	.	intergenic	0.148	rs786394230	2
AAT.ATT	7	39766859	39766873	5	.	.	intergenic	.	rs792851490	2
AAAC.GTTT	7	40069540	40069558	4.75	ENSSSCT00000001774	KIF6	intron	2.713	460206 rs7905	2
AC.GT	7	40213384	40213396	6.5	.	.	intergenic	0.042	.	3
AC.GT	7	40428794	40428812	9.5	ENSSSCT00000001776	MOCS1	intron	0.301	441187 rs7936	2
AATT.AATT	7	40529395	40529412	4.5	.	.	intergenic	.	393969 rs7916	2
AAAC.GTTT	7	40532592	40532611	5	.	.	intergenic	.	024363 rs7916	2
AGG.CCT	7	40533291	40533305	5	.	.	intergenic	.	572345 rs7917	2
AAAC.GTTT	7	40552978	40552993	4	.	.	intergenic	.	.	2
AC.GT	7	40909564	40909575	6	ENSSSCT00000001777	LRFN2	intron	-0.334	rs789335130	2
AG.CT	7	40967916	40967929	7	.	.	intergenic	.	rs793741467	2
AAAT.ATTT	7	41524081	41524099	4.75	.	.	intergenic	-0.055	rs788744413	2
AAAG.CTTT	7	41708556	41708568	3.25	ENSSSCT00000001792	.	intron	-0.015	rs793812660	2
AAAG.CTTT	7	41708556	41708568	3.25	ENSSSCT00000030920	.	intron	-0.015	rs793812660	2
AAAG.CTTT	7	41708556	41708568	3.25	ENSSSCT00000027428	.	intron	-0.015	rs793812660	2
AC.GT	7	41717657	41717668	6	ENSSSCT00000001792	.	intron	0.5	351155 rs7914	2
AC.GT	7	41717657	41717668	6	ENSSSCT00000030920	.	intron	0.5	351155 rs7914	2
AC.GT	7	41717657	41717668	6	ENSSSCT00000027428	.	intron	0.5	351155 rs7914	2
AAAGC.GCTTT	7	41760464	41760492	5.8	.	.	intergenic	-0.477	280355 rs7882	2
AAAC.GTTT	7	41827962	41827979	4.5	.	.	intergenic	0.169	.	2
AAAC.GTTT	7	42130525	42130544	5	ENSSSCT00000001801	MDFI	intron	-0.304	.	2
AT.AT	7	42852026	42852035	5	.	.	intergenic	-0.256	rs786454887	2
AC.GT	7	42912451	42912463	6.5	.	.	intergenic	.	rs788967366	2
AAAC.GTTT	7	42916402	42916426	6.25	.	.	intergenic	-0.096	540068 rs7883	2
AAAC.GTTT	7	43150396	43150414	4.75	ENSSSCT00000001825	.	intron	0.151	rs793128882	3
AAAAC.GTTTT	7	43549482	43549500	3.8	ENSSSCT00000001847	CUL7	intron	1.009	rs792043476	2
AAAC.GTTT	7	43629247	43629264	4.5	ENSSSCT00000001850	KLC4	intron	0.122	rs787418887	2
AAAC.GTTT	7	43742558	43742572	3.75	ENSSSCT00000030706	CUL9	intron	0.293	.	2
AAT.ATT	7	43768435	43768451	5.66667	ENSSSCT00000001861	C6ORF108	promoter	.	349946 rs7930	2
AAT.ATT	7	43768435	43768451	5.66667	ENSSSCT00000001854	C6ORF108	promoter	.	349946 rs7930	2
AATG.CATT	7	43797160	43797186	6.75	ENSSSCT00000001855	TTBK1	intron	-0.046	rs790322811	2
AAC.GTT	7	44687221	44687242	7.33333	.	.	intergenic	1.167	rs788002630	2
AAAC.GTTT	7	45072433	45072456	6	.	.	intergenic	0.032	rs791006420	2
AATG.CATT	7	45128625	45128644	5	ENSSSCT00000001903	NFKBIE	intron	0.932	rs792993713	2
AATG.CATT	7	45128625	45128644	5	ENSSSCT00000032591	NFKBIE	intron	0.932	rs792993713	2
AC.GT	7	45740913	45740923	5.5	ENSSSCT00000001909	.	intron	0.814	rs709857311	2
AG.CT	7	45823338	45823354	8.5	ENSSSCT00000001909	.	intron	.	.	4
AAAG.CTTT	7	46086137	46086151	3.75	.	.	intergenic	0.283	rs793115164	2
AAAC.GTTT	7	46186415	46186434	5	.	.	intergenic	.	.	2
AAC.GTT	7	46365927	46365939	4.33333	ENSSSCT00000023043	.	intron	-0.02	318136 rs7934	2

AG.CT	7	46398814	46398830	8.5	ENSSSCT00000023043	.	intron	1.497	rs792944058	3
AAC.GTT	7	46892875	46892888	4.66667	.	.	intergenic	.	134247 rs7925	2
AC.GT	7	47053353	47053369	8.5	ENSSSCT00000001911	CLIC5	intron	-0.037	239149 rs7931	2
AAC.GTT	7	47184859	47184878	6.66667	.	.	intergenic	.	rs790867464	2
AAAAC.GTTTT	7	47205432	47205447	3.2	.	.	intergenic	.	rs792358850	2
AG.CT	7	47408734	47408748	7.5	ENSSSCT00000001918	RCAN2	intron	0.027	rs787496928	2
AATG.CATT	7	47440400	47440433	8.5	ENSSSCT00000001918	RCAN2	intron	0.397	056613 rs7890	2
AAAT.ATTT	7	48187218	48187234	4.25	.	.	intergenic	.	rs792115996	2
AAAAT.ATTTT	7	48194418	48194444	5.4	.	.	intergenic	-0.051	rs787947387	4
AC.GT	7	48383964	48383982	9.5	ENSSSCT00000001929	GPR116	intron	-0.691	74880 rs7934	6
AAAAC.GTTTT	7	48397104	48397134	6.2	ENSSSCT00000001929	GPR116	intron	.	360378 rs6989	3
AAC.GTT	7	48470831	48470851	7	ENSSSCT00000001930	GPR110	intron	.	rs791196014	2
AAC.GTT	7	48521588	48521613	8.66667	.	.	intergenic	0.279	rs790776240	2
AAAC.GTTT	7	48619486	48619504	4.75	.	.	intergenic	0.143	rs788091187	2
AT.AT	7	48665518	48665532	7.5	.	.	intergenic	0.077	.	4
AAAAT.ATTTT	7	48786826	48786848	4.6	.	.	intergenic	0.006	rs791275475	2
AG.CT	7	48828044	48828056	6.5	.	.	intergenic	0.109	366751 rs7907	2
AT.AT	7	48852344	48852354	5.5	.	.	intergenic	0.07	382651 rs7862	2
AAAC.GTTT	7	48873416	48873437	5.5	.	.	intergenic	0.123	560121 rs7926	2
AAAC.GTTT	7	48945324	48945338	3.75	ENSSSCT00000001932	.	intron	0.16	rs786230801	2
AAAAT.ATTTT	7	49091755	49091779	5	ENSSSCT00000001933	.	intron	-0.028	.	2
AAAAT.ATTTT	7	49100709	49100727	3.8	ENSSSCT00000001933	.	intron	-0.035	.	2
AATT.AATT	7	49170585	49170602	4.5	.	.	intergenic	0.044	120140 rs7914	2
AAAT.ATTT	7	49195248	49195270	5.75	.	.	intergenic	.	535268 rs7915	2
AAAAC.GTTTT	7	49220297	49220314	3.6	.	.	intergenic	-0.043	090574 rs7870	2
AC.GT	7	49574606	49574620	7.5	.	.	intergenic	0.033	715745 rs7932	2
AG.CT	7	49625954	49625965	6	.	.	intergenic	.	.	2
AG.CT	7	49676584	49676620	18.5	.	.	intergenic	0.127	352180 rs7895	4
AT.AT	7	49687496	49687506	5.5	.	.	intergenic	.	365293 rs7920	2
AC.GT	7	49785098	49785118	10.5	.	.	intergenic	.	.	2
AAAAG.CTTTT	7	49988076	49988093	3.6	.	.	intergenic	0.043	.	4
AAAAC.GTTTT	7	50014501	50014515	3	.	.	intergenic	0.032	752842 rs7881	2
AT.AT	7	50289991	50290000	5	ENSSSCT00000001944	CRISP1	intron	-0.122	.	2
AAAAT.ATTTT	7	50399073	50399096	4.8	.	.	intergenic	-0.077	.	2
AG.CT	7	50575022	50575034	6.5	.	.	intergenic	.	rs791311865	2
AAAAC.GTTTT	7	50580836	50580857	4.4	.	.	intergenic	-0.134	rs789993159	2
AACC.GGTT	7	50854266	50854297	8	.	.	intergenic	-0.096	rs788052512	2
AAAC.GTTT	7	51011333	51011353	5.25	ENSSSCT00000001950	TFAP2D	intron	0.113	482265 rs7898	2
AG.CT	7	51013146	51013159	7	ENSSSCT00000001950	TFAP2D	intron	-0.037	.	3
AAAT.ATTT	7	51047596	51047614	4.75	ENSSSCT00000001950	TFAP2D	intron	0.077	.	2
AG.CT	7	51078190	51078209	10	.	.	intergenic	0.037	rs711869631	2
AAAAG.CTTTT	7	51788391	51788406	3.2	.	.	intergenic	0.039	rs786562515	2
AAAC.GTTT	7	51795401	51795420	5	.	.	intergenic	.	rs705433247	2
AC.GT	7	52139025	52139049	12.5	ENSSSCT00000001952	.	intron	0.033	rs791005458	4
AT.AT	7	52192035	52192045	5.5	ENSSSCT00000001952	.	intron	.	.	2
ACACAT.ATGTG	7	52226615	52226642	4.66667	ENSSSCT00000001952	.	intron	0.109	rs788239110	2
AT.AT	7	52285592	52285623	16	ENSSSCT00000001952	.	intron	-0.186	rs791216632	2
AG.CT	7	52302478	52302490	6.5	ENSSSCT00000001952	.	intron	0.549	.	2
AAAC.GTTT	7	52471799	52471814	4	.	.	intergenic	0.318	423835 rs7930	4
AAT.ATT	7	52927139	52927163	8.33333	.	.	intergenic	0.287	025269 rs7894	2
AC.GT	7	52950046	52950056	5.5	ENSSSCT00000001961	HYKK	intron	0.075	rs791539519	3
AT.AT	7	53382728	53382737	5	.	.	intergenic	0.204	rs792515055	2
AAAC.GTTT	7	53434484	53434505	5.5	ENSSSCT00000032525	.	intron	-0.048	543859 rs7931	2
AC.GT	7	53777933	53777944	6	ENSSSCT00000001986	RASGRF1	intron	-0.087	954953 rs7079	2
AG.CT	7	53826747	53826757	5.5	.	.	intergenic	-0.072	rs790142400	2
AC.GT	7	53836002	53836012	5.5	.	.	intergenic	-0.025	rs789314176	2
AAAAT.ATTTT	7	53859608	53859632	5	.	.	intergenic	.	rs791153045	2
AT.AT	7	53946103	53946115	6.5	.	.	intergenic	0.06	040957 rs7937	3
AT.AT	7	53994318	53994328	5.5	.	.	intergenic	0.058	rs791321750	2
AG.CT	7	54061586	54061602	8.5	ENSSSCT00000001990	KIAA1024	promoter	1.246	rs788603802	2
AT.AT	7	54076578	54076590	6.5	.	.	intergenic	0.294	rs789626198	3
AC.GT	7	54429804	54429818	7.5	.	.	intergenic	.	rs793787994	2
AAAAAC.GTTTTT	7	54505760	54505781	3.66667	ENSSSCT00000001994	ZFAND6	intron	.	437464 rs7929	2
AAT.ATT	7	54510556	54510572	5.66667	ENSSSCT00000001994	ZFAND6	intron	0.079	573213 rs7864	2
AAAAC.GTTTT	7	54599104	54599124	4.2	.	.	intergenic	-0.019	rs792123937	2
AGGG.CCCT	7	54695801	54695818	4.5	.	.	intergenic	-0.16	008225 rs7921	2
AT.AT	7	55323126	55323137	6	.	.	intergenic	0.091	rs788576756	2
AATG.CATT	7	55361376	55361393	4.5	.	.	intergenic	1.072	.	3
AT.AT	7	55446112	55446127	8	.	.	intergenic	-0.04	rs787131537	2
AC.GT	7	55593319	55593329	5.5	ENSSSCT00000035382	IL-16	intron	-0.15	rs708607449	2
AC.GT	7	55593319	55593329	5.5	ENSSSCT00000025614	IL-16	intron	-0.15	rs708607449	2
AC.GT	7	55593319	55593329	5.5	ENSSSCT00000034701	IL-16	intron	-0.15	rs708607449	2
AAAC.GTTT	7	55683261	55683284	6	ENSSSCT00000035382	IL-16	intron	-0.139	709487 rs7900	2
AAAC.GTTT	7	55683261	55683284	6	ENSSSCT00000034701	IL-16	intron	-0.139	709487 rs7900	2
AAAC.GTTT	7	55683261	55683284	6	ENSSSCT00000002002	IL-16	promoter	-0.139	709487 rs7900	2
AC.GT	7	55712852	55712862	5.5	ENSSSCT00000002003	STARD5	intron	0.018	rs793270793	2
AG.CT	7	55897730	55897748	9.5	.	.	intergenic	-0.221	412877 rs7880	2
AAAC.GTTT	7	56037274	56037294	5.25	.	.	intergenic	.	556325 rs7893	2
AAAG.CTTT	7	56041567	56041578	3	.	.	intergenic	.	.	2
AAG.CTT	7	56260856	56260881	8.66667	.	.	intergenic	-0.29	.	2
AT.AT	7	56282282	56282291	5	.	.	intergenic	-0.011	rs793448581	2
AAAC.GTTT	7	56400532	56400548	4.25	ENSSSCT00000002007	.	intron	.	rs786915599	2
AAAC.GTTT	7	56400532	56400548	4.25	ENSSSCT00000002008	.	promoter	.	rs786915599	2
AAAT.ATTT	7	56496772	56496801	7.5	.	.	intergenic	.	588127 rs7907	2

AAAC.GTTT	7	56510790	56510802	3.25	ENSSSCT00000002009	.	intron	0.198	rs793207873	2
AT.AT	7	56661087	56661097	5.5	ENSSSCT00000002009	.	intron	.	rs790264097	2
AT.AT	7	56816073	56816088	8	.	.	intergenic	0.242	rs706836422	2
AAAC.GTTT	7	56833841	56833859	4.75	.	.	intergenic	0.182	.	2
AC.GT	7	56907248	56907259	6	ENSSSCT00000002011	SH3GL3	intron	-0.001	533064 rs7874	2
AC.GT	7	56907248	56907259	6	ENSSSCT00000029711	SH3GL3	intron	-0.001	533064 rs7874	2
AAAT.ATTT	7	57209890	57209908	4.75	.	.	intergenic	.	rs791877637	2
AG.CT	7	57518253	57518263	5.5	ENSSSCT00000002020	HOMER2	intron	-0.374	545227 rs7896	2
AAAC.GTTT	7	57537546	57537568	5.75	ENSSSCT00000002020	HOMER2	intron	-0.09	196442 rs7869	2
AAAC.GTTT	7	57556317	57556339	5.75	ENSSSCT00000002020	HOMER2	intron	.	rs792240050	2
AAC.GTT	7	57562119	57562146	9.33333	ENSSSCT00000002020	HOMER2	intron	0.099	rs786803206	2
AAAT.ATTT	7	57619571	57619599	7.25	ENSSSCT00000002021	WHAMM	intron	.	rs790409472	2
AAAC.GTTT	7	57624999	57625017	4.75	ENSSSCT00000002021	WHAMM	intron	.	rs789571532	2
AG.CT	7	57639588	57639611	12	.	.	intergenic	0.025	547094 rs7927	2
AAAAAC.GTTTTT	7	57650324	57650354	5.16667	.	.	intergenic	.	704780 rs7935	2
AC.GT	7	57762507	57762520	7	ENSSSCT00000002023	.	intron	1.402	rs788368968	2
AAAAG.CTTTT	7	57884571	57884592	4.4	.	.	intergenic	.	293590 rs7870	2
AAAC.GTTT	7	57971678	57971692	3.75	ENSSSCT00000002032	PDE8A	intron	0.053	rs787387482	2
AT.AT	7	58211090	58211104	7.5	.	.	intergenic	.	rs791849710	2
AC.GT	7	58816274	58816286	6.5	ENSSSCT00000002046	CCDC37	intron	.	rs786387265	2
AAAC.GTTT	7	58900476	58900494	4.75	.	.	intergenic	-0.529	345034 rs7924	2
AAAC.GTTT	7	59510939	59510953	3.75	ENSSSCT00000002056	MFGE8	intron	.	rs789847352	2
ATC.GAT	7	59835081	59835109	9.66667	ENSSSCT00000002059	FANCI	intron	0.976	rs791627407	2
AAC.GTT	7	60154250	60154275	8.66667	ENSSSCT00000022362	PEX11A	promoter	.	rs786957468	2
AC.GT	7	60170573	60170583	5.5	ENSSSCT00000002069	WDR93	intron	.	rs786294466	2
AAC.GTT	7	60313453	60313475	7.66667	ENSSSCT00000025884	AP3S2	intron	0.049	305364 rs7869	2
ACAT.ATGT	7	60374230	60374248	4.75	.	.	intergenic	-0.035	rs790159479	2
AAAT.ATTT	7	60654649	60654676	7	.	.	intergenic	.	471091 rs7874	2
AAGG.CCTT	7	60773497	60773515	4.75	.	.	intergenic	.	.	2
AG.CT	7	61802267	61802281	7.5	.	.	intergenic	0.256	rs791465817	2
ATC.GAT	7	61829299	61829322	8	.	.	intergenic	0.105	.	2
AAGT.ACTT	7	61831977	61831993	4.25	.	.	intergenic	.	460962 rs7890	2
AAAT.ATTT	7	61950143	61950161	4.75	.	.	intergenic	0.187	.	2
AAAC.GTTT	7	62474168	62474200	8.25	.	.	intergenic	-0.112	406511 rs7914	2
AC.GT	7	62680977	62680997	10.5	.	.	intergenic	-0.06	.	2
AAAAG.CTTTT	7	62788599	62788618	4	ENSSSCT00000002103	PTPN9	intron	0.549	rs788332334	2
AAAT.ATTT	7	62985914	62985939	6.5	.	.	intergenic	.	rs791440260	2
AAAAAC.GTTTTT	7	63005980	63006002	3.83333	.	.	intergenic	-0.089	rs791749478	2
AAAC.GTTT	7	63210351	63210374	6	.	.	intergenic	0.333	rs786568239	2
AAG.CTT	7	63752936	63752969	11.3333	.	.	intergenic	0.335	rs791575691	2
AC.GT	7	63934701	63934723	11.5	.	.	intergenic	-0.129	.	4
AAAC.GTTT	7	64054489	64054510	5.5	ENSSSCT00000002141	PML	intron	0.093	rs791847653	2
AAAAC.GTTTT	7	64134096	64134123	5.6	.	.	intergenic	.	rs790274512	2
AAAC.GTTT	7	64135423	64135441	4.75	.	.	intergenic	.	rs790773473	2
AG.CT	7	64159135	64159152	9	ENSSSCT00000002144	.	intron	-0.086	.	3
AGAT.ATCT	7	64202830	64202852	5.75	.	.	intergenic	0.169	rs793063296	2
AAAAG.CTTTT	7	64470478	64470496	3.8	.	.	intergenic	.	rs788425896	2
AAAT.ATTT	7	65191109	65191122	3.5	ENSSSCT00000002153	BBS4	intron	0.131	rs787521357	2
AAAT.ATTT	7	65191109	65191122	3.5	ENSSSCT00000002151	ARIH1	intron	0.131	rs787521357	2
AAAC.GTTT	7	65321659	65321674	4	ENSSSCT00000030450	.	intron	0.033	rs789674067	6
ACCT.AGGT	7	65330258	65330283	6.5	ENSSSCT00000030450	.	intron	.	rs787655448	2
AT.AT	7	65708025	65708035	5.5	ENSSSCT00000002166	.	intron	0.12	rs790436710	2
AT.AT	7	66323236	66323252	8.5	.	.	intergenic	0.446	374460 rs7862	2
AATG.CATT	7	66375733	66375753	5.25	.	.	intergenic	0.165	rs792341705	3
AAAAC.GTTTT	7	66614785	66614808	4.8	.	.	intergenic	0.097	rs791054250	2
AAC.GTT	7	67621606	67621619	4.66667	.	.	intergenic	0.048	rs788636466	2
ATCC.GGAT	7	67724684	67724705	5.5	.	.	intergenic	0.191	.	2
AAAT.ATTT	7	67759605	67759622	4.5	.	.	intergenic	0.024	rs792389911	2
AC.GT	7	67977578	67977593	8	.	.	intergenic	.	rs793253034	6
AC.GT	7	68082885	68082897	6.5	.	.	intergenic	0.205	228187 rs6970	4
AAAAT.ATTTT	7	68187046	68187068	4.6	ENSSSCT00000002174	SLC25A21	intron	0.157	rs792460610	2
AATATC.GATATT	7	68431907	68431937	5.16667	.	.	intergenic	.	524816 rs7909	2
AAAT.ATTT	7	68496545	68496567	5.75	.	.	intergenic	0.169	340708 rs7911	2
AAAT.ATTT	7	68508092	68508112	5.25	.	.	intergenic	0.239	rs787034732	2
AAT.ATT	7	68541439	68541452	4.66667	.	.	intergenic	.	576656 rs7882	2
ACTAT.ATAGT	7	68980793	68980807	3	.	.	intergenic	.	rs789953143	2
AAAC.GTTT	7	69130279	69130302	6	ENSSSCT00000029555	.	intron	.	rs788725404	2
AT.AT	7	69157085	69157097	6.5	ENSSSCT00000029555	.	intron	.	900247 rs7902	2
AT.AT	7	69347307	69347316	5	.	.	intergenic	0.289	.	2
AC.GT	7	69459253	69459268	8	.	.	intergenic	-0.011	rs790538305	2
AATT.AATT	7	69664806	69664828	5.75	ENSSSCT00000002187	SRP54	intron	0.228	rs791380281	2
AAG.CTT	7	70019404	70019426	7.66667	ENSSSCT00000020434	U1	promoter	0.189	rs791439142	2
AAAC.GTTT	7	70024300	70024320	5.25	.	.	intergenic	.	028976 rs7933	2
AAAT.ATTT	7	70049058	70049076	4.75	ENSSSCT00000002193	EAPP	intron	.	rs792805545	2
AAAG.CTTT	7	70078406	70078423	4.5	.	.	intergenic	.	rs789542241	2
AT.AT	7	70189860	70189873	7	.	.	intergenic	-0.066	.	2
AC.GT	7	70200785	70200795	5.5	.	.	intergenic	.	rs791692940	2
AATG.CATT	7	70469199	70469225	6.75	.	.	intergenic	0.01	072634 rs7903	2
AAAC.GTTT	7	70559440	70559462	5.75	.	.	intergenic	0.222	rs786803497	2
AT.AT	7	70695653	70695663	5.5	ENSSSCT00000002196	EGLN3	3'utr	0.801	rs793084999	2
AT.AT	7	70787601	70787613	6.5	.	.	intergenic	5.822	rs788033164	4
AAG.CTT	7	71048902	71048913	4	.	.	intergenic	0.365	074527 rs7905	2
AC.GT	7	71390323	71390333	5.5	ENSSSCT00000022418	.	intron	.	.	3
AG.CT	7	71472848	71472857	5	ENSSSCT00000022418	.	intron	0.085	rs793603118	2

AC.GT	7	71507826	71507837	6	ENSSSCT00000022418	.	intron	-0.077	.	4
AC.GT	7	72213885	72213897	6.5	ENSSSCT00000002199	.	intron	.	rs790782265	2
AT.AT	7	72355835	72355851	8.5	ENSSSCT00000028957	.	3'utr	.	rs793607184	2
AC.GT	7	72369037	72369048	6	ENSSSCT00000028957	.	intron	0.218	.	2
AAAC.GTTT	7	72487426	72487452	6.75	.	.	intergenic	-0.069	rs788033881	3
AAAG.CTTT	7	72704207	72704229	5.75	ENSSSCT00000029319	NUBPL	intron	0.08	rs788407071	2
AAC.GTT	7	72947280	72947296	5.66667	.	.	intergenic	.	02806 rs7881	2
AG.CT	7	72963159	72963173	7.5	ENSSSCT00000023087	GPR33	promoter	0.306	66542 rs7915	3
AG.CT	7	72967826	72967836	5.5	ENSSSCT00000023087	GPR33	intron	0.166	.	2
AAAG.CTTT	7	73030946	73030963	4.5	.	.	intergenic	0.352	rs789752145	3
AAAC.GTTT	7	73906868	73906903	9	.	.	intergenic	.	84575 rs7918	4
AAAC.GTTT	7	74269938	74269960	5.75	.	.	intergenic	0.36	rs792980586	2
AATC.GATT	7	75685810	75685833	6	.	.	intergenic	0.39	rs791010477	2
AAAAC.GTTTT	7	75879025	75879050	5.2	.	.	intergenic	0.227	rs790286712	2
AG.CT	7	75960453	75960462	5	.	.	intergenic	0.101	.	3
AC.GT	7	76222333	76222348	8	.	.	intergenic	.	rs793412251	3
AC.GT	7	76384937	76384947	5.5	.	.	intergenic	.	rs788091845	3
AAAC.GTTT	7	76640506	76640519	3.5	.	.	intergenic	.	rs791067850	2
AT.AT	7	76919177	76919202	13	.	.	intergenic	0.111	rs789175644	2
AAC.GTT	7	77112499	77112511	4.33333	.	.	intergenic	-0.034	rs793540514	2
AAAC.GTTT	7	77248787	77248822	9	.	.	intergenic	0.031	rs790415695	2
AAAG.CTTT	7	77427615	77427636	5.5	.	.	intergenic	0.066	rs786903447	2
AAC.GTT	7	77589115	77589139	8.33333	.	.	intergenic	-0.163	rs793258576	3
AT.AT	7	77734934	77734943	5	.	.	intergenic	.	rs789128625	2
AG.CT	7	77998659	77998682	12	.	.	intergenic	0.145	.	4
AT.AT	7	78096575	78096591	8.5	.	.	intergenic	-0.026	rs788028593	2
AAAC.GTTT	7	78362079	78362093	3.75	.	.	intergenic	0.003	.	2
GATAT.ATATC	7	78409092	78409120	4.83333	.	.	intergenic	-0.108	rs788154095	2
AAAAT.ATTTT	7	78455194	78455214	3.5	.	.	intergenic	.	rs792566919	2
AT.AT	7	78472613	78472627	7.5	.	.	intergenic	0.008	rs790271215	2
AG.CT	7	78817411	78817425	7.5	.	.	intergenic	0.2	rs791770435	2
AAAT.ATTT	7	78976899	78976918	5	.	.	intergenic	0.195	.	2
AAAAC.GTTTT	7	79429993	79430013	4.2	.	.	intergenic	0.044	88751 rs7905	2
AC.GT	7	79660047	79660057	5.5	ENSSSCT00000002211	STXBP6	intron	0.292	rs788733229	2
AATC.GATT	7	79844905	79844924	5	.	.	intergenic	0.045	rs793167922	2
AG.CT	7	79874949	79874959	5.5	.	.	intergenic	0.098	rs707845398	4
AAAT.ATTT	7	79893646	79893670	6.25	.	.	intergenic	-0.107	rs787475155	2
AT.AT	7	79896843	79896857	7.5	.	.	intergenic	-0.22	.	4
AAC.GTT	7	80673143	80673161	6.33333	.	.	intergenic	.	06126 rs7931	2
AAAC.GTTT	7	80705247	80705261	3.75	.	.	intergenic	0.19	rs787298150	2
CCCCC.GGGG	7	80706963	80706981	3.16667	.	.	intergenic	-0.011	rs693200018	2
AG.CT	7	81285343	81285352	5	ENSSSCT00000002277	C14orf119	promoter	0.301	rs789237906	2
AG.CT	7	81285343	81285352	5	ENSSSCT00000002276	ACIN1	promoter	0.301	rs789237906	2
AT.AT	7	81662967	81662976	5	.	.	intergenic	0.211	rs787963477	2
AG.CT	7	81698050	81698062	6.5	.	.	intergenic	-0.017	11181 rs7884	3
AG.CT	7	82023644	82023659	8	.	.	intergenic	.	.	2
AAT.ATT	7	82030117	82030132	5.33333	.	.	intergenic	0.222	53426 rs7925	2
AAAAC.GTTTT	7	82045361	82045385	5	ENSSSCT00000002299	TRAV41	promoter	0.081	74133 rs7899	2
AGAT.ATCT	7	82132773	82132787	3.75	.	.	intergenic	-0.003	72094 rs7899	2
AG.CT	7	82172625	82172634	5	.	.	intergenic	-0.03	.	2
AAT.ATT	7	83262971	83262982	4	.	.	intergenic	.	87422 rs7890	2
AC.GT	7	83423601	83423619	9.5	.	.	intergenic	0.133	.	2
AAAAT.ATTTT	7	83431198	83431215	3.6	.	.	intergenic	.	rs788112670	2
AT.AT	7	83672388	83672403	8	.	.	intergenic	.	rs789241421	2
AG.CT	7	83890276	83890286	5.5	.	.	intergenic	.	rs793236199	2
AC.GT	7	83913149	83913159	5.5	.	.	intergenic	0.483	rs789418865	3
AAC.GTT	7	84272118	84272142	8.33333	.	.	intergenic	.	.	3
AC.GT	7	84733600	84733623	12	.	.	intergenic	.	.	2
AAC.GTT	7	85918256	85918270	5	.	.	intergenic	.	.	2
AT.AT	7	86199707	86199716	5	ENSSSCT00000002498	.	intron	0.249	rs793780149	2
AAAC.GTTT	7	86342388	86342406	4.75	.	.	intergenic	0.473	rs789124154	2
AAT.ATT	7	86365208	86365224	5.66667	.	.	intergenic	.	rs792452402	3
ACAT.ATGT	7	86494759	86494770	3	.	.	intergenic	0.163	rs787244082	2
AAAAT.ATTTT	7	86542973	86542999	4.5	.	.	intergenic	.	rs789685734	2
AAAAC.GTTTT	7	86591289	86591309	4.2	.	.	intergenic	-0.192	rs788287887	2
AAAAC.GTTTT	7	86671304	86671323	4	.	.	intergenic	0.162	28616 rs7928	2
AT.AT	7	86807141	86807152	6	.	.	intergenic	0.201	rs792380757	3
AAAC.GTTT	7	86810536	86810568	8.25	.	.	intergenic	0.178	59672 rs7881	2
AT.AT	7	86881672	86881704	16.5	.	.	intergenic	0.109	rs789125576	2
AG.CT	7	86931053	86931063	5.5	.	.	intergenic	0.248	.	2
AT.AT	7	87505431	87505440	5	.	.	intergenic	0.081	rs790279182	2
AAAT.ATTT	7	87599969	87599992	6	ENSSSCT00000034830	ARRDC4	promoter	-0.036	47131 rs7897	2
AAAT.ATTT	7	87599969	87599992	6	ENSSSCT00000002508	ARRDC4	promoter	-0.036	47131 rs7897	2
AAAT.ATTT	7	87600246	87600257	3	ENSSSCT00000034830	ARRDC4	promoter	-0.209	rs787525351	2
AAAT.ATTT	7	87600246	87600257	3	ENSSSCT00000002508	ARRDC4	promoter	-0.209	rs787525351	2
AAGG.CCTT	7	87605900	87605923	6	.	.	intergenic	-0.229	.	2
AAAC.GTTT	7	87699621	87699643	5.75	.	.	intergenic	0.073	rs791665525	2
AAAC.GTTT	7	87959821	87959835	3.75	.	.	intergenic	.	rs791411544	2
AC.GT	7	87970152	87970165	7	.	.	intergenic	0.023	rs790905034	7
AAAAC.GTTTT	7	87995460	87995479	4	.	.	intergenic	-0.113	rs786330166	2
AATG.CATT	7	88022545	88022563	4.75	.	.	intergenic	0.161	rs786325158	2
AAAT.ATTT	7	88120190	88120204	3.75	.	.	intergenic	-0.033	63195 rs7884	2
AAAC.GTTT	7	88326194	88326208	3.75	.	.	intergenic	-0.045	rs789349595	2
AC.GT	7	88694305	88694318	7	.	.	intergenic	-0.107	79323 rs7885	2

AAC.GTT	7	88869774	88869790	5.66667	.	.	intergenic	-0.272	.	2
AGAT.ATCT	7	89858058	89858073	4	.	.	intergenic	0.133	rs792864102	2
AC.GT	7	89878637	89878649	6.5	.	.	intergenic	-0.176	rs793184046	2
AGG.CCT	7	89903639	89903650	4	.	.	intergenic	2.109	rs790908567	2
AT.AT	7	90079044	90079054	5.5	.	.	intergenic	0.17	rs786473026	2
AC.GT	7	90114647	90114661	7.5	.	.	intergenic	-0.139	;58953 rs7915	2
AT.AT	7	90324501	90324513	6.5	.	.	intergenic	.	'99936 rs7867	2
AT.AT	7	90516163	90516173	5.5	.	.	intergenic	0.263	.	3
AG.CT	7	90565415	90565424	5	.	.	intergenic	-0.05	.	4
AAAT.ATTT	7	90584907	90584929	5.75	.	.	intergenic	-0.089	rs793210470	2
AAAAT.ATTTT	7	90616372	90616390	3.8	.	.	intergenic	0.42	rs791003020	2
AAAC.GTTT	7	90631011	90631022	3	.	.	intergenic	.	rs792471214	2
AAAT.ATTT	7	90662789	90662820	8	ENSSSCT00000026803	.	intron	.	345594 rs7907	3
AC.GT	7	90846684	90846702	9.5	.	.	intergenic	-0.192	rs793679272	4
AC.GT	7	90969175	90969185	5.5	.	.	intergenic	0.188	rs699852960	2
AAAC.GTTT	7	91427875	91427897	5.75	.	.	intergenic	.	;79536 rs7893	2
AG.CT	7	91474386	91474397	6	.	.	intergenic	-0.067	rs790352624	2
AC.GT	7	91667351	91667372	11	.	.	intergenic	-0.132	)89171 rs7886	2
AAAC.GTTT	7	91729432	91729445	3.5	.	.	intergenic	-0.332	379019 rs7900	2
AAAG.CTTT	7	91901853	91901867	3.75	ENSSSCT00000002524	CHD2	intron	.	rs788303780	2
AAAG.CTTT	7	91901853	91901867	3.75	ENSSSCT00000025402	CHD2	intron	.	rs788303780	2
AAAC.GTTT	7	91919654	91919673	5	ENSSSCT00000002524	CHD2	intron	-0.016	;28887 rs7885	2
AAAC.GTTT	7	91919654	91919673	5	ENSSSCT00000027293	CHD2	intron	-0.016	;28887 rs7885	2
AAAC.GTTT	7	91919654	91919673	5	ENSSSCT00000025402	CHD2	intron	-0.016	;28887 rs7885	2
AAAGC.GCTTT	7	92021453	92021470	3.6	.	.	intergenic	0.069	rs789947457	2
AT.AT	7	92362054	92362067	7	.	.	intergenic	0.124	.	2
AC.GT	7	92683096	92683109	7	.	.	intergenic	0.132	381758 rs7899	2
AC.GT	7	92730659	92730670	6	ENSSSCT00000002520	SLCO3A1	intron	-0.267	rs792361778	3
AAAT.ATTT	7	92897324	92897339	4	ENSSSCT00000002520	SLCO3A1	intron	-0.117	rs791671898	2
AAAC.GTTT	7	93051694	93051716	5.75	.	.	intergenic	0.249	'72459 rs7893	2
AAAG.CTTT	7	93233803	93233814	3	.	.	intergenic	0.065	rs707767727	2
AT.AT	7	93400284	93400306	11.5	.	.	intergenic	0.206	;66919 rs7915	2
AT.AT	7	93884707	93884721	7.5	.	.	intergenic	0.017	;40369 rs7921	2
AATT.AATT	7	93947565	93947592	7	.	.	intergenic	0.086	205157 rs7901	2
AG.CT	7	94180897	94180909	6.5	.	.	intergenic	-0.212	242530 rs7899	2
AG.CT	7	94687699	94687709	5.5	.	.	intergenic	.	.	2
AAAAAC.GTTTTT	7	94798221	94798248	4.66667	ENSSSCT00000002528	.	intron	.	rs788867916	2
AGC.GCT	7	94895983	94895994	4	.	.	intergenic	0.034	.	2
AAAG.CTTT	7	95078316	95078361	11.5	.	.	intergenic	0.14	rs786355402	3
AAGG.CCTT	7	95079399	95079423	6.25	.	.	intergenic	0.128	rs786696496	2
AAAAT.ATTTT	7	95082400	95082426	5.4	.	.	intergenic	-0.032	rs787938999	2
AAAAC.GTTTT	7	95264597	95264619	4.6	ENSSSCT00000002535	SPTB	intron	0.168	rs792283089	2
ACAT.ATGT	7	95293953	95293975	5.75	ENSSSCT00000002535	SPTB	intron	-0.144	.	2
AC.GT	7	95502799	95502809	5.5	ENSSSCT00000002539	HURC1-FN	intron	0.423	.	3
AAC.GTT	7	95625127	95625144	6	.	.	intergenic	0.051	)27425 rs7925	2
AAAC.GTTT	7	95916562	95916577	4	ENSSSCT00000002542	FUT8	intron	0.224	rs786505877	2
AG.CT	7	95958167	95958202	18	ENSSSCT00000002542	FUT8	intron	.	rs791724878	2
AG.CT	7	96053100	96053121	11	ENSSSCT00000002542	FUT8	intron	0.13	)96226 rs7911	4
AAAAC.GTTTT	7	96098557	96098582	5.2	ENSSSCT00000002542	FUT8	intron	0.175	442291 rs7886	2
ACC.GGT	7	96206500	96206516	5.66667	.	.	intergenic	0.55	rs792703815	2
AC.GT	7	96248891	96248901	5.5	.	.	intergenic	.	rs792028598	2
AG.CT	7	96256887	96256911	12.5	.	.	intergenic	-0.001	.	2
AAAAT.ATTTT	7	96399563	96399585	4.6	.	.	intergenic	0.071	)68913 rs7864	4
AG.CT	7	96447066	96447078	6.5	.	.	intergenic	0.805	rs786754665	2
AAAT.ATTT	7	96508602	96508623	5.5	.	.	intergenic	-0.309	253148 rs7893	2
AC.GT	7	96726824	96726833	5	.	.	intergenic	0.053	.	2
AAAAAC.GTTTTT	7	97109149	97109178	5	ENSSSCT00000002545	GPHN	intron	-0.078	394595 rs7932	2
AAAT.ATTT	7	97223166	97223189	6	.	.	intergenic	0.292	316828 rs7874	2
AAAC.GTTT	7	97262852	97262872	5.25	.	.	intergenic	0.147	)38583 rs7877	3
AC.GT	7	97355624	97355636	6.5	ENSSSCT00000002548	MPP5	intron	1.804	.	2
AAAC.GTTT	7	97630991	97631016	6.5	.	.	intergenic	.	rs789592157	2
AAAC.GTTT	7	97635996	97636018	5.75	.	.	intergenic	.	rs789097183	2
AAAAT.ATTTT	7	97685269	97685295	5.4	ENSSSCT00000002555	ARG2	intron	0.166	)09773 rs7910	2
AAAAT.ATTTT	7	97685269	97685295	5.4	ENSSSCT00000033367	ARG2	intron	0.166	)09773 rs7910	2
AAAAT.ATTTT	7	97685269	97685295	5.4	ENSSSCT00000033225	ARG2	intron	0.166	)09773 rs7910	2
AAAAT.ATTTT	7	97685269	97685295	5.4	ENSSSCT00000034592	ARG2	intron	0.166	)09773 rs7910	2
AAAT.ATTT	7	97710292	97710314	5.75	ENSSSCT00000002556	VTI1B	intron	0.079	rs787945654	2
AC.GT	7	97790835	97790848	7	ENSSSCT00000002558	RDH12	intron	0.313	.	3
AAAG.CTTT	7	97829623	97829637	3.75	ENSSSCT00000002559	ZFYVE26	intron	0.018	rs793716689	2
AC.GT	7	97834915	97834931	8.5	ENSSSCT00000002559	ZFYVE26	intron	1.948	.	2
AC.GT	7	97889539	97889548	5	ENSSSCT00000002559	ZFYVE26	intron	0.447	.	3
AAAC.GTTT	7	98024458	98024481	6	.	.	intergenic	0.987	rs788389995	2
AAAC.GTTT	7	98072629	98072649	5.25	.	.	intergenic	0.213	rs790646837	2
AT.AT	7	98188931	98188956	13	.	.	intergenic	0.528	353519 rs7878	2
AAC.GTT	7	98231684	98231695	4	.	.	intergenic	.	rs793295010	2
AAC.GTT	7	98342075	98342093	6.33333	ENSSSCT00000023246	.	intron	0.288	rs793095329	2
AAGGG.CCCTT	7	98473315	98473338	4	ENSSSCT00000023246	.	intron	0.203	rs787685449	2
ATC.GAT	7	98631201	98631225	8.33333	.	.	intergenic	0.152	rs788383406	4
AG.CT	7	98733645	98733657	6.5	.	.	intergenic	0.008	rs789208301	2
AAAC.GTTT	7	98970687	98970701	3.75	.	.	intergenic	.	rs787440619	2
AC.GT	7	98989708	98989719	6	.	.	intergenic	0.277	'53526 rs7895	3
AAC.GTT	7	99212175	99212192	6	ENSSSCT00000002568	GALNT16	intron	0.121	319870 rs7898	2
AAAC.GTTT	7	99479000	99479013	3.5	.	.	intergenic	0.009	rs790286551	2
AC.GT	7	99593229	99593240	6	ENSSSCT00000002573	KIAA0247	intron	0.109	.	4

AAAT.ATTT	7	99635434	99635453	5	.	.	intergenic	.	rs786767165	2
AAAAC.GTTTT	7	99747883	99747911	5.8	.	.	intergenic	0.156	)12799 rs7891	2
AAAC.GTTT	7	100191557	100191584	7	ENSSSCT00000002578	COX16	intron	.	rs786746458	2
AAAC.GTTT	7	100581328	100581363	9	.	.	intergenic	0.113	rs787689704	2
AAAAC.GTTTT	7	100630475	100630499	5	.	.	intergenic	0.161	.	2
AAAAG.CTTTT	7	100954863	100954902	8	.	.	intergenic	.	rs790911189	2
AAAC.GTTT	7	100964944	100964958	3.75	.	.	intergenic	.	rs787807528	2
AAAC.GTTT	7	101256704	101256718	3.75	.	.	intergenic	0.046	rs793304430	2
AAG.CTT	7	101565223	101565234	4	.	.	intergenic	-0.034	rs792455858	2
AAG.CTT	7	101614710	101614723	4.66667	.	.	intergenic	0.098	rs786681387	4
AATC.GATT	7	101716793	101716824	8	.	.	intergenic	-0.07	369908 rs7872	2
AAAAC.GTTTT	7	101902719	101902733	3	ENSSSCT00000002596	.	intron	-0.104	rs787560337	2
AC.GT	7	101959191	101959209	9.5	ENSSSCT00000002596	.	intron	1.106	rs788196086	2
AG.CT	7	102006037	102006049	6.5	ENSSSCT00000002596	.	intron	0.759	rs793400304	2
AAC.GTT	7	102151285	102151312	9.33333	ENSSSCT00000002600	ZFYVE1	intron	.	.	2
AC.GT	7	102293591	102293608	9	ENSSSCT00000035550	PSEN1	intron	.	rs792497949	2
AC.GT	7	102293591	102293608	9	ENSSSCT00000002603	PSEN1	intron	.	rs792497949	2
AG.CT	7	102376966	102376979	7	.	.	intergenic	-0.154	.	2
AAAC.GTTT	7	102485566	102485591	6.5	ENSSSCT00000031856	.	intron	.	rs788546391	2
AG.CT	7	102867208	102867228	10.5	.	.	intergenic	0.163	rs792814502	2
AAAC.GTTT	7	102933307	102933324	4.5	ENSSSCT00000002615	.	intron	.	rs789239466	2
AAAAC.GTTTT	7	103039479	103039507	5.8	ENSSSCT00000002619	ENTPD5	intron	0.614	.	2
AAAT.ATTT	7	103059968	103059986	4.75	.	.	intergenic	0.343	rs787052657	2
AC.GT	7	103343974	103343994	10.5	.	.	intergenic	0.119	.	4
AAAT.ATTT	7	103488918	103488941	6	.	.	intergenic	.	rs788321385	2
ACG.CGT	7	103731283	103731297	5	.	.	intergenic	0.412	142377 rs7893	2
AAAG.CTTT	7	103912061	103912081	5.25	ENSSSCT00000002638	PROX2	intron	.	rs789488458	2
AAAG.CTTT	7	103912061	103912081	5.25	ENSSSCT00000002637	PROX2	intron	.	rs789488458	2
AG.CT	7	104105328	104105344	8.5	ENSSSCT00000002646	ACYP1	intron	.	rs793163244	2
AAAC.GTTT	7	104132580	104132607	7	ENSSSCT00000002648	NEK9	intron	0.145	rs790794499	2
AAT.ATT	7	104171241	104171260	6.66667	.	.	intergenic	0.201	rs787518222	2
AG.CT	7	104238182	104238195	7	.	.	intergenic	0.176	.	4
AAC.GTT	7	104351066	104351080	5	.	.	intergenic	.	335464 rs7863	2
AAC.GTT	7	104485589	104485605	5.66667	.	.	intergenic	.	186620 rs7880	2
AG.CT	7	104536763	104536777	7.5	.	.	intergenic	0.336	rs793666281	3
AAAC.GTTT	7	105160150	105160161	3	ENSSSCT00000024345	TTLL5	intron	.	rs788196704	2
AAAC.GTTT	7	105223764	105223795	8	.	.	intergenic	0.24	rs787137915	2
AAAAC.GTTTT	7	105286608	105286628	4.2	ENSSSCT00000002653	IFT43	intron	.	)55095 rs7872	2
AAC.GTT	7	105438456	105438481	8.66667	.	.	intergenic	.	272730 rs7878	3
AT.AT	7	105510467	105510476	5	.	.	intergenic	0.092	'53040 rs7879	2
AG.CT	7	105921091	105921103	6.5	.	.	intergenic	0.123	.	3
AT.AT	7	105927538	105927549	6	.	.	intergenic	0.263	rs789807677	2
AC.GT	7	106061611	106061623	6.5	.	.	intergenic	-0.228	rs790311342	2
AC.GT	7	106128888	106128902	7.5	.	.	intergenic	0.06	181249 rs7918	2
AAAC.GTTT	7	106441808	106441827	5	ENSSSCT00000002675	.	intron	-0.025	.	2
AAAAC.GTTTT	7	106486373	106486400	5.6	ENSSSCT00000023660	NOXRED1	intron	0.343	rs792772094	2
AG.CT	7	106948547	106948556	5	.	.	intergenic	0.096	.	2
AT.AT	7	106966854	106966863	5	.	.	intergenic	.	rs787796168	2
AC.GT	7	107264924	107264935	6	.	.	intergenic	0.061	rs790224522	3
AGGG.CCCT	7	107335217	107335235	4.75	.	.	intergenic	0.575	rs792404294	2
ACAG.CTGT	7	107491456	107491472	4.25	.	.	intergenic	0.044	rs788871347	2
ATC.GAT	7	107497310	107497329	6.66667	.	.	intergenic	0.14	351029 rs7867	2
AG.CT	7	107524165	107524178	7	.	.	intergenic	0.091	rs791814613	2
AATT.AATT	7	107648226	107648241	4	.	.	intergenic	.	rs788839664	2
AAC.GTT	7	107687053	107687074	7.33333	.	.	intergenic	0.021	520170 rs7873	2
AAAAC.GTTTT	7	107761411	107761425	3	.	.	intergenic	0.825	rs787396856	2
AG.CT	7	107953293	107953305	6.5	ENSSSCT00000025256	NRXN3	intron	-0.027	rs786391036	2
AAAT.ATTT	7	107982121	107982141	5.25	.	.	intergenic	0.304	rs788851024	2
AC.GT	7	108444932	108444951	10	.	.	intergenic	1.251	.	2
AC.GT	7	108512335	108512354	10	ENSSSCT00000031767	.	intron	0.354	531932 rs7937	3
AG.CT	7	108570840	108570851	6	ENSSSCT00000031767	.	intron	0.043	rs788032377	3
AC.GT	7	108648435	108648446	6	ENSSSCT00000031767	.	intron	0.051	rs792963703	2
AC.GT	7	109085659	109085671	6.5	.	.	intergenic	-0.097	322303 rs7878	2
AAAT.ATTT	7	109101711	109101726	4	.	.	intergenic	0.077	rs790636503	2
AAAAT.ATTTT	7	109126716	109126734	3.8	.	.	intergenic	.	462148 rs7931	2
AAAC.GTTT	7	109196283	109196305	5.75	.	.	intergenic	.	rs793538058	2
AC.GT	7	109239454	109239466	6.5	.	.	intergenic	-0.033	rs793578924	3
AAAAC.GTTTT	7	109262633	109262654	4.4	.	.	intergenic	0.138	180063 rs7868	2
ATC.GAT	7	109271904	109271915	4	.	.	intergenic	0.299	517632 rs7867	3
AAAT.ATTT	7	109485184	109485202	4.75	.	.	intergenic	0.231	rs793658461	2
AAAAC.GTTTT	7	109648044	109648069	5.2	.	.	intergenic	0.061	494039 rs7915	2
AAAAT.ATTTT	7	109827715	109827738	4.8	ENSSSCT00000028622	.	intron	.	rs792905672	2
AG.CT	7	110421806	110421822	8.5	ENSSSCT00000002683	SEL1L	intron	0.243	.	4
AAAG.CTTT	7	110492286	110492314	7.25	.	.	intergenic	0.208	rs792577578	2
AT.AT	7	110506324	110506333	5	.	.	intergenic	0.34	rs787825149	2
ATCC.GGAT	7	110527683	110527696	3.5	.	.	intergenic	0.239	rs789607714	2
AAAT.ATTT	7	110552596	110552614	4.75	.	.	intergenic	0.093	rs789541216	2
AT.AT	7	110670792	110670803	6	.	.	intergenic	0.298	rs793701691	2
AAACC.GGTTT	7	110980357	110980389	6.6	.	.	intergenic	0.241	rs791420423	2
AG.CT	7	111398541	111398553	6.5	.	.	intergenic	-0.089	.	3
ATC.GAT	7	111413385	111413397	4.33333	.	.	intergenic	-0.053	.	2
AT.AT	7	111635306	111635318	6.5	.	.	intergenic	-0.168	372690 rs7905	2
AC.GT	7	111885752	111885761	5	.	.	intergenic	.	.	2
AG.CT	7	111983812	111983825	7	.	.	intergenic	-0.011	.	3

AAAAG.CTTTT	7	112392545	112392563	3.8	.	.	intergenic	-0.102	536768 rs7877	2
AAAC.GTTT	7	112414758	112414784	6.75	.	.	intergenic	.	rs791879394	2
AAAT.ATTT	7	112490695	112490711	4.25	.	.	intergenic	-0.008	987364 rs7924	2
AC.GT	7	112779082	112779091	5	.	.	intergenic	0.046	.	3
AAC.GTT	7	112810611	112810624	4.66667	.	.	intergenic	0.427	rs792157222	4
AGG.CCT	7	112896847	112896858	4	.	.	intergenic	-0.126	032066 rs7938	2
AT.AT	7	112999679	112999688	5	.	.	intergenic	0.022	rs786480695	2
AGAGAT.ATCTC	7	113009721	113009758	6.33333	.	.	intergenic	.	rs792753953	2
AAAG.CTTT	7	113058920	113058943	6	.	.	intergenic	-0.105	rs788154398	2
AC.GT	7	113169654	113169665	6	.	.	intergenic	-0.119	rs786503151	2
AT.AT	7	113205023	113205033	5.5	.	.	intergenic	-0.21	853156 rs7910	2
ACAT.ATGT	7	113276446	113276476	7.75	.	.	intergenic	0.216	114678 rs7889	2
AC.GT	7	113376520	113376534	7.5	.	.	intergenic	-0.217	873812 rs7900	2
AC.GT	7	113382670	113382698	14.5	.	.	intergenic	-0.006	502407 rs7869	2
AG.CT	7	113400021	113400032	6	.	.	intergenic	-0.171	rs789204045	2
AT.AT	7	113514496	113514505	5	.	.	intergenic	0.382	rs793804263	2
AC.GT	7	113560755	113560765	5.5	.	.	intergenic	-0.194	rs792277110	2
AC.GT	7	113593443	113593455	6.5	.	.	intergenic	-0.124	.	5
AAAC.GTTT	7	113632201	113632215	3.75	.	.	intergenic	0.116	rs786869494	2
AAC.GTT	7	113695692	113695713	7.33333	.	.	intergenic	-0.122	rs791099855	3
ATCC.GGAT	7	113748721	113748741	5.25	.	.	intergenic	0.299	490964 rs7871	2
AC.GT	7	113778208	113778230	11.5	.	.	intergenic	0.012	.	4
AT.AT	7	113786683	113786697	7.5	.	.	intergenic	0.016	621451 rs7903	2
AAAC.GTTT	7	113979561	113979580	5	.	.	intergenic	0.719	rs792907056	2
AAAC.GTTT	7	114001440	114001458	4.75	.	.	intergenic	0.198	rs791030091	2
ATC.GAT	7	114062616	114062634	6.33333	.	.	intergenic	0.071	014053 rs7875	2
AAAC.GTTT	7	114144290	114144312	5.75	.	.	intergenic	-0.258	rs790293758	2
ACT.AGT	7	114390552	114390563	4	.	.	intergenic	0.308	rs791730855	2
AAT.ATT	7	114461852	114461869	6	.	.	intergenic	-0.162	rs792111292	2
AAAC.GTTT	7	114516062	114516092	7.75	.	.	intergenic	.	rs787469654	2
AAT.ATT	7	114516217	114516228	4	.	.	intergenic	.	.	2
AT.AT	7	114626474	114626486	6.5	.	.	intergenic	0.01	rs792054778	2
AC.GT	7	114711531	114711555	12.5	.	.	intergenic	-0.043	.	2
AT.AT	7	114822722	114822733	6	.	.	intergenic	0.029	rs698526009	4
AG.CT	7	114863853	114863873	10.5	.	.	intergenic	-0.25	rs709745074	2
AC.GT	7	114966101	114966110	5	.	.	intergenic	-0.121	rs787754338	2
AATT.AATT	7	115123899	115123920	5.5	.	.	intergenic	-0.124	783162 rs7889	2
AC.GT	7	115142849	115142859	5.5	.	.	intergenic	-0.233	rs792222358	2
AAAC.GTTT	7	115152852	115152866	3.75	.	.	intergenic	0.19	rs787201727	2
AC.GT	7	115263773	115263782	5	.	.	intergenic	-0.028	.	2
AAC.GTT	7	115438971	115438983	4.33333	.	.	intergenic	-0.052	861830 rs7864	2
AC.GT	7	115529049	115529064	8	.	.	intergenic	-0.189	rs791098103	2
AC.GT	7	115623626	115623653	14	.	.	intergenic	-0.004	504897 rs7877	2
AT.AT	7	115743575	115743586	6	.	.	intergenic	.	700884 rs7873	2
AAAC.GTTT	7	115791411	115791422	3	.	.	intergenic	-0.029	.	2
AAAC.GTTT	7	115833559	115833573	3.75	.	.	intergenic	0.134	173780 rs7901	2
AG.CT	7	115878903	115878915	6.5	.	.	intergenic	-0.047	139396 rs7914	2
AG.CT	7	115993296	115993308	6.5	.	.	intergenic	1.763	rs793450837	2
AAAC.GTTT	7	116048588	116048607	5	.	.	intergenic	0.07	rs786949644	2
AC.GT	7	116175804	116175813	5	.	.	intergenic	-0.18	rs787436239	3
AC.GT	7	116222792	116222807	8	.	.	intergenic	0.82	rs791571865	2
AGG.CCT	7	116226125	116226141	5.66667	.	.	intergenic	.	.	2
AAACC.GGTTT	7	116282641	116282664	4.8	.	.	intergenic	0.413	709802 rs7884	2
AAAAC.GTTTT	7	116571023	116571055	6.6	.	.	intergenic	0.237	418379 rs7862	2
AG.CT	7	116667021	116667031	5.5	ENSSSCT00000002692	KCNK10	intron	0.044	372887 rs7890	2
AC.GT	7	116668501	116668514	7	ENSSSCT00000002692	KCNK10	intron	0.158	.	4
AATG.CATT	7	116685255	116685279	6.25	ENSSSCT00000002692	KCNK10	intron	0.096	.	2
AATC.GATT	7	116915865	116915877	3.25	ENSSSCT00000002694	PTPN21	intron	.	rs789633884	2
AAAG.CTTT	7	117244598	117244615	4.5	ENSSSCT00000002697	TTC8	intron	0.117	rs787902370	2
AGG.CCT	7	117287343	117287356	4.66667	.	.	intergenic	-0.081	.	2
AAT.ATT	7	117336684	117336704	7	ENSSSCT00000022230	U6	promoter	.	471442 rs7873	2
AC.GT	7	117338904	117338920	8.5	.	.	intergenic	.	rs791206783	2
AG.CT	7	117719086	117719107	11	ENSSSCT00000002698	FOXN3	intron	0.015	rs793640726	2
AAAG.CTTT	7	117755683	117755697	3.75	ENSSSCT00000002698	FOXN3	intron	.	rs791884789	2
AAAC.GTTT	7	117773459	117773473	3.75	ENSSSCT00000002698	FOXN3	intron	-0.076	533941 rs7897	2
AAC.GTT	7	117857363	117857384	7.33333	ENSSSCT00000002698	FOXN3	intron	0.28	rs789227671	2
AC.GT	7	118201819	118201829	5.5	ENSSSCT00000002700	EFCAB11	intron	-0.184	rs786623831	2
AAAT.ATTT	7	118217558	118217574	4.25	ENSSSCT00000002700	EFCAB11	intron	.	rs791390270	2
AAAACT.AGTTTT	7	118325546	118325566	3.5	ENSSSCT00000002700	EFCAB11	intron	0.566	rs787517863	2
AAAAT.ATTTT	7	118357534	118357557	4.8	ENSSSCT00000002701	.	intron	-0.134	rs788311059	2
AG.CT	7	118470902	118470911	5	ENSSSCT00000002702	KCNK13	intron	.	.	2
AAAC.GTTT	7	118508614	118508636	5.75	ENSSSCT00000002702	KCNK13	intron	.	rs792697160	2
AAT.ATT	7	118515285	118515302	6	ENSSSCT00000002702	KCNK13	intron	-0.089	rs792715431	2
AAT.ATT	7	118777397	118777416	6.66667	.	.	intergenic	-0.031	rs789508161	2
AAT.ATT	7	118816640	118816654	5	.	.	intergenic	-0.017	rs788860747	2
AG.CT	7	118929457	118929471	7.5	ENSSSCT00000002706	.	intron	0.256	rs790085272	4
ATCC.GGAT	7	118934781	118934819	9.75	ENSSSCT00000002706	.	intron	-0.017	rs793377269	2
AG.CT	7	119282846	119282860	7.5	.	.	intergenic	0.212	rs786430076	4
AC.GT	7	119338904	119338915	6	.	.	intergenic	0.038	rs791587315	3
AG.CT	7	119350502	119350511	5	.	.	intergenic	-0.163	rs789806469	2
AAAAAC.GTTTTT	7	119386867	119386887	3.5	.	.	intergenic	.	971690 rs7863	2
AAAAT.ATTTT	7	119631316	119631343	5.6	.	.	intergenic	.	rs789839651	2
AAAGC.GCTTT	7	119858385	119858415	6.2	.	.	intergenic	.	182649 rs7937	2
AAAAC.GTTTT	7	119936516	119936550	7	.	.	intergenic	0.194	134658 rs7924	2

AG.CT	7	120112399	120112408	5	ENSSSCT00000002714	FBLN5	intron	0.745	.	2
AAAT.ATTT	7	120194805	120194821	4.25	ENSSSCT00000002715	TRIP11	intron	0.148	rs786789505	2
AAAC.GTTT	7	120209400	120209422	5.75	ENSSSCT00000002715	TRIP11	promoter	0.126	rs792173378	2
AGC.GCT	7	120224820	120224840	7	ENSSSCT00000002716	ATXN3	cds	0.269	rs790699493	2
AT.AT	7	120225142	120225158	8.5	ENSSSCT00000002716	ATXN3	intron	0.191	rs789531793	3
AAAAT.ATTTT	7	120284062	120284078	3.4	.	.	intergenic	0.05	rs792247595	2
AAAC.GTTT	7	120381144	120381161	4.5	.	.	intergenic	.	354653 rs7872	2
AC.GT	7	120490204	120490214	5.5	ENSSSCT00000002720	SLC24A4	intron	.	rs788896225	2
ATCC.GGAT	7	120649680	120649697	4.5	.	.	intergenic	-0.046	551673 rs7885	2
AC.GT	7	120778886	120778903	9	.	.	intergenic	-0.24	rs790480972	3
AC.GT	7	120781867	120781878	6	ENSSSCT00000002722	.	intron	0.389	rs792259932	2
AAAG.CTTT	7	120861596	120861614	4.75	ENSSSCT00000002725	GOLGA5	intron	0.548	777680 rs7867	2
AG.CT	7	120938954	120938976	11.5	.	.	intergenic	-0.174	rs787053616	3
AAAG.CTTT	7	121445941	121445953	3.25	.	.	intergenic	.	524888 rs7925	2
AC.GT	7	121508309	121508319	5.5	.	.	intergenic	.	rs707111398	2
AG.CT	7	121602806	121602821	8	.	.	intergenic	.	rs788305062	2
AAAG.CTTT	7	121643942	121643956	3.75	.	.	intergenic	.	rs792292088	2
AATG.CATT	7	122917333	122917349	4.25	ENSSSCT00000002757	SERPINA5	intron	-0.354	rs793655442	2
AAAGC.GCTTT	7	123009632	123009661	6	.	.	intergenic	.	rs791438123	2
AC.GT	7	123202464	123202482	9.5	.	.	intergenic	-0.035	.	2
AC.GT	7	123615942	123615952	5.5	ENSSSCT00000022914	DICER1	intron	-0.022	.	2
AAC.GTT	7	123686055	123686071	5.66667	.	.	intergenic	0.116	rs791349693	2
AC.GT	7	123748341	123748353	6.5	ENSSSCT00000002772	CLMN	intron	-0.361	rs790275812	3
AC.GT	7	123993719	123993734	8	.	.	intergenic	-0.423	rs793351392	2
AAAG.CTTT	7	124052611	124052634	6	.	.	intergenic	.	rs791943235	3
AC.GT	7	124053705	124053716	6	.	.	intergenic	.	rs788566625	5
AT.AT	7	124063996	124064005	5	.	.	intergenic	.	.	2
AT.AT	7	124385943	124385952	5	.	.	intergenic	0.002	.	2
AAC.GTT	7	124488720	124488731	4	.	.	intergenic	.	rs789950489	2
AAAC.GTTT	7	124796207	124796231	6.25	ENSSSCT00000002780	ATG2B	intron	1.67	538726 rs7913	2
AAC.GTT	7	124814030	124814049	6.66667	ENSSSCT00000002780	ATG2B	intron	0.311	rs792491171	2
AAT.ATT	7	124938215	124938227	4.33333	ENSSSCT00000002783	PAPOLA	intron	-0.461	rs792292858	2
AAAAC.GTTTT	7	125013031	125013051	4.2	.	.	intergenic	-0.176	433566 rs7932	2
AAAAT.ATTTT	7	125071859	125071877	3.8	.	.	intergenic	.	rs786290760	2
AC.GT	7	125130651	125130660	5	.	.	intergenic	-0.145	rs789095348	2
AC.GT	7	125172820	125172830	5.5	.	.	intergenic	-0.45	rs788082700	2
AC.GT	7	125647395	125647406	6	.	.	intergenic	-0.633	.	2
AC.GT	7	125670677	125670691	7.5	.	.	intergenic	.	195450 rs7901	2
ATCC.GGAT	7	125677186	125677199	3.5	.	.	intergenic	.	.	2
AG.CT	7	126139060	126139084	12.5	.	.	intergenic	-0.277	.	3
AGAT.ATCT	7	126208243	126208259	4.25	.	.	intergenic	.	rs792044989	2
AAAT.ATTT	7	126544521	126544542	5.5	.	.	intergenic	-0.086	094609 rs7908	3
AC.GT	7	126774510	126774526	8.5	.	.	intergenic	0.078	rs786974274	2
AAAC.GTTT	7	127299998	127300012	3.75	.	.	intergenic	0.072	rs789334067	2
AC.GT	7	127559636	127559649	7	.	.	intergenic	1.584	799250 rs7893	2
AAC.GTT	7	127741811	127741830	6.66667	.	.	intergenic	-0.215	rs786571678	2
AAGG.CCTT	7	127929222	127929237	4	.	.	intergenic	-0.188	.	2
AAAT.ATTT	7	128030543	128030556	3.5	ENSSSCT00000002786	SETD3	intron	.	.	2
AAGG.CCTT	7	128052763	128052782	5	ENSSSCT00000002787	CCNK	intron	0.065	rs789299568	2
AG.CT	7	128272133	128272145	6.5	ENSSSCT00000002788	CYP46A1	intron	-0.237	rs790335550	3
AAAC.GTTT	7	128447835	128447849	3.75	.	.	intergenic	0.217	rs786889822	2
AAAC.GTTT	7	128888878	128888900	5.75	ENSSSCT00000002802	CDC42BPE	promoter	-0.581	rs791110180	2
AC.GT	7	129025743	129025752	5	.	.	intergenic	-0.094	rs790845321	2
AT.AT	7	129066331	129066341	5.5	.	.	intergenic	2.489	.	3
AC.GT	7	129072132	129072142	5.5	ENSSSCT00000002805	RCOR1	intron	2.111	rs792203310	2
AC.GT	7	129118119	129118133	7.5	ENSSSCT00000002805	RCOR1	intron	.	.	4
AAAAT.ATTTT	7	129178625	129178654	6	ENSSSCT00000002805	RCOR1	intron	.	rs788332471	2
AAAAAC.GTTTTT	7	129368763	129368785	3.83333	.	.	intergenic	.	rs792026412	2
AAC.GTT	7	129992918	129992933	5.33333	ENSSSCT00000002821	PPP2R5C	intron	0	127888 rs7873	2
AC.GT	7	130495363	130495372	5	.	.	intergenic	.	rs789054591	2
AGC.GCT	7	130796553	130796565	4.33333	.	.	intergenic	.	rs786330619	2
AAAC.GTTT	7	131042022	131042048	6.75	ENSSSCT00000002836	TDRD9	intron	-0.123	rs697285782	2
AAT.ATT	7	133000423	133000443	7	.	.	intergenic	.	rs788679408	2
AG.CT	7	133514794	133514827	17	.	.	intergenic	.	586838 rs7937	2
AAAAT.ATTTT	7	133564107	133564132	5.2	.	.	intergenic	-0.003	rs791577872	2
AAAT.ATTT	7	133969000	133969017	4.5	ENSSSCT00000002907	EFHC1	intron	.	355506 rs7906	2
AG.CT	7	134204752	134204766	7.5	.	.	intergenic	0.165	320582 rs7883	2
AAGG.CCTT	7	134364074	134364091	4.5	ENSSSCT00000024099	.	intron	0.146	.	2
AC.GT	7	134431791	134431813	11.5	ENSSSCT00000002916	ICK	intron	0.1	rs792987054	2
AAAAAC.GTTTTT	8	103716	103741	4.33333	ENSSSCT00000030851	PCGF3	intron	.	301085 rs7879	2
AC.GT	8	162382	162404	11.5	ENSSSCT00000026976	MFSD7	intron	0.623	956902 rs7885	3
AAAAT.ATTTT	8	573418	573437	4	ENSSSCT00000009498	NELFA	intron	-0.126	978410 rs7915	2
AAAAT.ATTTT	8	573418	573437	4	ENSSSCT00000009501	POLN	intron	-0.126	978410 rs7915	2
AAAC.GTTT	8	611273	611297	6.25	ENSSSCT00000009498	NELFA	intron	.	538194 rs7918	2
AAAC.GTTT	8	611273	611297	6.25	ENSSSCT00000009501	POLN	intron	.	538194 rs7918	2
AAAC.GTTT	8	957403	957423	5.25	.	.	intergenic	-0.047	rs788408460	2
AAAC.GTTT	8	1213801	1213820	5	ENSSSCT00000009510	ADD1	intron	.	rs793745503	2
AC.GT	8	1504872	1504886	7.5	ENSSSCT00000009515	HUNTINGTI	intron	0.937	525245 rs7881	2
AAAG.CTTT	8	2281887	2281902	4	.	.	intergenic	-0.254	.	2
AAC.GTT	8	2552390	2552407	6	ENSSSCT00000009528	C4orf50	intron	-0.034	rs789321351	2
AAAAC.GTTTT	8	2724824	2724847	4.8	.	.	intergenic	-0.21	235476 rs7883	4
AAAC.GTTT	8	2743168	2743184	4.25	.	.	intergenic	.	rs792868142	3
AAAC.GTTT	8	4022811	4022829	4.75	.	.	intergenic	.	.	2
AAAC.GTTT	8	4097828	4097839	3	.	.	intergenic	-0.354	rs792038696	2

AC.GT	8	4258208	4258217	5	ENSSSCT00000009541	ABLIM2	intron	-1.261	.	2
AAAG.CTTT	8	4727635	4727670	9	.	.	intergenic	.	rs792746792	3
ACAT.ATGT	8	4821957	4821971	3.75	.	.	intergenic	0.022	.	2
AATG.CATT	8	4899252	4899281	7.5	ENSSSCT00000023202	STK32B	intron	-0.107	770211 rs7936	2
AG.CT	8	5168439	5168449	5.5	.	.	intergenic	-0.377	144858 rs7878	2
AC.GT	8	5366570	5366580	5.5	.	.	intergenic	-0.123	.	2
AAAC.GTTT	8	5392620	5392641	5.5	.	.	intergenic	.	731574 rs7900	2
AAAC.GTTT	8	5458392	5458414	5.75	.	.	intergenic	-0.24	rs793644552	2
AC.GT	8	5566139	5566153	7.5	ENSSSCT00000031128	.	intron	.	.	3
AAC.GTT	8	5566921	5566937	5.66667	ENSSSCT00000031128	.	intron	.	.	4
AT.AT	8	6000740	6000756	8.5	.	.	intergenic	.	.	2
AAAT.ATTT	8	6011302	6011320	4.75	.	.	intergenic	.	rs788046244	2
AG.CT	8	6080014	6080023	5	.	.	intergenic	-0.33	rs792649347	2
AAAC.GTTT	8	6227302	6227331	7.5	.	.	intergenic	.	585884 rs7863	2
AC.GT	8	6560000	6560015	8	.	.	intergenic	-0.012	rs788437266	2
AAACC.GGTTT	8	6605551	6605581	6.2	.	.	intergenic	0.148	rs786246493	2
AAAAT.ATTTT	8	6627481	6627499	3.8	.	.	intergenic	-0.169	rs790674358	2
AC.GT	8	7073483	7073498	8	.	.	intergenic	-0.384	rs789145751	5
AAC.GTT	8	7330998	7331009	4	.	.	intergenic	-0.268	.	2
AT.AT	8	7520519	7520537	9.5	.	.	intergenic	.	762279 rs7890	2
AC.GT	8	7635693	7635705	6.5	.	.	intergenic	0.047	rs793072992	2
AC.GT	8	7783158	7783168	5.5	.	.	intergenic	0.022	.	2
AT.AT	8	7897135	7897144	5	.	.	intergenic	.	rs793297160	2
AAAAC.GTTTT	8	7910390	7910408	3.8	.	.	intergenic	.	933686 rs7868	2
ATC.GAT	8	7978829	7978843	5	.	.	intergenic	.	.	2
AAAAAT.ATTTTT	8	7984825	7984842	3	.	.	intergenic	-0.139	284011 rs7890	2
AATG.CATT	8	8077223	8077245	5.75	.	.	intergenic	-0.06	rs790678444	2
ATC.GAT	8	8125259	8125271	4.33333	.	.	intergenic	-0.236	.	2
AC.GT	8	8131414	8131430	8.5	.	.	intergenic	0.446	.	5
AATG.CATT	8	8356983	8357009	6.75	.	.	intergenic	-0.129	100683 rs7904	2
AT.AT	8	8809172	8809184	6.5	.	.	intergenic	-0.001	.	2
AAAAT.ATTTT	8	8852903	8852920	3.6	.	.	intergenic	-0.338	rs790981685	2
AC.GT	8	8857301	8857315	7.5	.	.	intergenic	-0.3	rs791776419	2
AT.AT	8	8861207	8861217	5.5	.	.	intergenic	.	.	2
AC.GT	8	8901442	8901451	5	.	.	intergenic	0.015	rs788436326	2
AAAC.GTTT	8	8920975	8920994	5	.	.	intergenic	.	.	2
AAAG.CTTT	8	8935103	8935120	4.5	.	.	intergenic	-0.085	312308 rs7936	2
ATC.GAT	8	8962885	8962896	4	.	.	intergenic	-0.067	.	3
AAAC.GTTT	8	9257368	9257389	5.5	.	.	intergenic	-0.187	rs786243889	2
AG.CT	8	9278730	9278751	11	.	.	intergenic	-0.121	rs790807686	2
AAAT.ATTT	8	9285613	9285632	5	.	.	intergenic	0.128	rs790897552	2
AT.AT	8	9469125	9469138	7	.	.	intergenic	0.081	rs786481795	2
AC.GT	8	9640439	9640448	5	.	.	intergenic	-0.155	rs790195973	2
ATCC.GGAT	8	9786566	9786583	4.5	.	.	intergenic	-0.021	.	2
AT.AT	8	9867748	9867757	5	ENSSSCT00000009556	CPEB2	intron	-0.04	rs789586651	2
AAC.GTT	8	10538117	10538141	8.33333	ENSSSCT00000009559	CC2D2A	intron	-0.038	376686 rs7908	2
AAAC.GTTT	8	10548554	10548576	5.75	.	.	intergenic	.	rs789722395	2
AG.CT	8	10574320	10574333	7	ENSSSCT00000009562	FBXL5	intron	.	rs788624803	2
AAAC.GTTT	8	10616020	10616033	3.5	.	.	intergenic	0.352	rs791014019	2
AG.CT	8	10657081	10657095	7.5	.	.	intergenic	.	rs789562259	3
AGAGG.CCTCT	8	10739734	10739760	5.4	ENSSSCT00000009565	CD38	intron	-0.115	rs787578552	2
AC.GT	8	10959894	10959910	8.5	ENSSSCT00000009568	PROM1	intron	-0.069	rs791637446	3
AAAT.ATTT	8	11142113	11142131	4.75	.	.	intergenic	.	rs788538426	2
AAAAT.ATTTT	8	11242101	11242125	5	.	.	intergenic	-0.403	rs793725577	2
AAAC.GTTT	8	11279596	11279610	3.75	.	.	intergenic	.	rs793720867	2
AAAC.GTTT	8	11477368	11477386	4.75	ENSSSCT00000025105	LDB2	intron	-0.16	462292 rs7869	3
AATG.CATT	8	11519134	11519151	4.5	ENSSSCT00000025105	LDB2	intron	-0.038	.	4
AAAC.GTTT	8	11520864	11520886	5.75	ENSSSCT00000025105	LDB2	intron	0.155	rs792508393	2
AAG.CTT	8	11819573	11819584	4	.	.	intergenic	-0.24	377247 rs7913	2
AT.AT	8	12278627	12278636	5	.	.	intergenic	.	.	2
AG.CT	8	12365825	12365856	16	ENSSSCT00000024457	CLRN2	intron	0.062	rs788705339	2
AC.GT	8	12548122	12548133	6	.	.	intergenic	0.794	.	3
AT.AT	8	12568096	12568106	5.5	ENSSSCT00000009570	NCAPG	intron	0.083	rs791056354	3
AAAG.CTTT	8	12777855	12777879	6.25	.	.	intergenic	-0.14	594587 rs7926	2
AG.CT	8	13073070	13073082	6.5	.	.	intergenic	-0.033	rs792158817	3
AG.CT	8	13136208	13136234	13.5	.	.	intergenic	0.223	rs792267914	2
AT.AT	8	13300914	13300924	5.5	.	.	intergenic	-0.084	rs790869032	2
AC.GT	8	13418911	13418923	6.5	.	.	intergenic	0.096	rs790111261	2
AAAG.CTTT	8	13435501	13435517	4.25	.	.	intergenic	.	290605 rs7901	2
AG.CT	8	13559638	13559673	18	.	.	intergenic	0.304	rs791942511	2
AAG.CTT	8	14112921	14112935	5	.	.	intergenic	-0.27	.	2
AAAAC.GTTTT	8	14125969	14125999	6.2	.	.	intergenic	.	rs792783936	2
AATG.CATT	8	14286018	14286044	6.75	.	.	intergenic	-0.281	rs788597653	2
AT.AT	8	14332046	14332055	5	.	.	intergenic	-0.153	rs788325070	3
AT.AT	8	14412006	14412022	8.5	.	.	intergenic	-0.002	.	2
AAT.ATT	8	14549699	14549717	6.33333	.	.	intergenic	-0.049	rs791147045	2
AC.GT	8	14662630	14662640	5.5	.	.	intergenic	0.035	570377 rs7932	2
AC.GT	8	14792846	14792862	8.5	.	.	intergenic	0.215	.	2
AAC.GTT	8	14894805	14894818	4.66667	.	.	intergenic	-0.008	rs787181271	3
AAAT.ATTT	8	14980246	14980260	3.75	.	.	intergenic	.	rs787451274	2
AAAG.CTTT	8	15018840	15018863	6	.	.	intergenic	1.185	rs789204400	2
AAAC.GTTT	8	15194638	15194655	4.5	.	.	intergenic	0.861	336823 rs7925	2
AG.CT	8	15407248	15407260	6.5	ENSSSCT00000009572	SLIT2	intron	-0.212	286562 rs7867	2
AG.CT	8	15481343	15481352	5	ENSSSCT00000030135	KCNIP4	intron	.	.	2

AG.CT	8	15481343	15481352	5	ENSSSCT00000031079	KCNIP4	intron	.	.	2
AAAC.GTTT	8	15495103	15495117	3.75	ENSSSCT00000030135	KCNIP4	intron	.	rs787261368	2
AAAC.GTTT	8	15495103	15495117	3.75	ENSSSCT00000031079	KCNIP4	intron	.	rs787261368	2
AG.CT	8	15512533	15512544	6	ENSSSCT00000030135	KCNIP4	intron	0.015	rs793019907	3
AAAAC.GTTTT	8	15515348	15515366	3.8	ENSSSCT00000030135	KCNIP4	intron	-0.17	rs786981797	2
AAAT.ATTT	8	15569492	15569510	4.75	ENSSSCT00000030135	KCNIP4	intron	.	.	2
AAAAAC.GTTTT	8	15744933	15744954	3.66667	.	.	intergenic	-0.045	.	2
AAC.GTT	8	16007359	16007373	5	.	.	intergenic	.	.	4
AT.AT	8	16009913	16009926	7	.	.	intergenic	-0.191	rs791720466	2
AAT.ATT	8	16152778	16152790	4.33333	.	.	intergenic	-0.149	960357 rs7872	2
AAAAC.GTTTT	8	16448889	16448914	5.2	.	.	intergenic	0.044	rs788953206	2
AAAAAC.GTTTT	8	16486685	16486707	3.83333	.	.	intergenic	-0.255	rs790223651	2
AC.GT	8	16576985	16576999	7.5	.	.	intergenic	.	.	3
AC.GT	8	16664700	16664715	8	.	.	intergenic	-0.165	.	3
AAAT.ATTT	8	16751030	16751045	4	.	.	intergenic	-0.009	rs791634499	3
AAAT.ATTT	8	16817119	16817139	5.25	.	.	intergenic	0.274	rs789639713	2
AT.AT	8	17014834	17014845	6	.	.	intergenic	.	rs792813563	2
AC.GT	8	17068274	17068300	13.5	.	.	intergenic	-0.064	rs691364496	3
AG.CT	8	17324914	17324928	7.5	.	.	intergenic	1.491	rs793174631	3
AC.GT	8	17364014	17364028	7.5	.	.	intergenic	0.148	996288 rs7869	2
AC.GT	8	17390595	17390613	9.5	.	.	intergenic	-0.012	.	4
AAC.GTT	8	17391399	17391414	5.33333	.	.	intergenic	0.53	941426 rs7907	2
AT.AT	8	17481785	17481800	8	.	.	intergenic	0.107	rs788694877	2
AGATAT.ATATC	8	17505744	17505776	5.5	.	.	intergenic	-0.08	.	2
ATC.GAT	8	17578811	17578825	5	.	.	intergenic	-0.071	.	2
AAT.ATT	8	17578906	17578918	4.33333	.	.	intergenic	-0.165	.	2
AAC.GTT	8	17626645	17626658	4.66667	.	.	intergenic	-0.243	250918 rs7866	2
AT.AT	8	17664634	17664643	5	.	.	intergenic	-0.072	.	2
ATC.GAT	8	17806984	17807009	8.66667	.	.	intergenic	-0.046	.	2
AAAC.GTTT	8	17817029	17817043	3.75	.	.	intergenic	-0.148	509814 rs7912	2
AAAT.ATTT	8	17832309	17832323	3.75	.	.	intergenic	0.134	241872 rs7886	2
AC.GT	8	17959664	17959676	6.5	.	.	intergenic	0.674	116669 rs7898	3
AC.GT	8	18160846	18160864	9.5	.	.	intergenic	0.729	rs789859480	2
AC.GT	8	18237884	18237895	6	.	.	intergenic	1.872	rs792177477	2
AAC.GTT	8	18258098	18258110	4.33333	.	.	intergenic	0.045	rs787417637	2
AT.AT	8	18307235	18307250	8	.	.	intergenic	-0.184	.	3
ACC.GGT	8	18396171	18396186	5.33333	.	.	intergenic	-0.087	rs702842129	2
AAT.ATT	8	18580959	18580983	8.33333	.	.	intergenic	0.091	rs790156901	2
AT.AT	8	18732503	18732519	8.5	.	.	intergenic	-0.043	.	2
AAAC.GTTT	8	18901808	18901822	3.75	ENSSSCT00000009580	DHX15	intron	-0.271	rs793479569	2
AGG.CCT	8	18978151	18978165	5	.	.	intergenic	1.254	.	2
AAG.CTT	8	19008672	19008696	8.33333	.	.	intergenic	0.805	rs791012265	2
AG.CT	8	19323602	19323614	6.5	.	.	intergenic	-0.269	rs791129488	3
AC.GT	8	19349382	19349396	7.5	ENSSSCT00000029731	.	intron	0.095	rs786377930	2
AAAAAC.GTTTT	8	19392159	19392196	6.33333	.	.	intergenic	.	rs792756504	3
AG.CT	8	19513361	19513370	5	.	.	intergenic	.	rs790471182	2
AAAAC.GTTTT	8	20004343	20004366	4.8	.	.	intergenic	.	rs788829420	2
AAT.ATT	8	20237194	20237210	5.66667	.	.	intergenic	.	rs790676517	2
AT.AT	8	20296437	20296448	6	.	.	intergenic	-0.024	rs786713885	2
AAGG.CCTT	8	20321129	20321154	6.5	.	.	intergenic	.	.	2
ACC.GGT	8	20347052	20347065	4.66667	.	.	intergenic	0.046	rs792307824	2
AAAC.GTTT	8	20429002	20429020	4.75	.	.	intergenic	0.171	098077 rs7889	2
AAT.ATT	8	20469968	20469980	4.33333	ENSSSCT00000023054	RBPJ	intron	0.129	150810 rs6904	3
AAC.GTT	8	20761461	20761474	4.66667	.	.	intergenic	0.077	rs791846051	2
AT.AT	8	20780429	20780440	6	.	.	intergenic	0.107	rs787684610	2
AAAC.GTTT	8	20813320	20813342	5.75	.	.	intergenic	0.219	rs791568598	2
AT.AT	8	20905891	20905901	5.5	.	.	intergenic	.	.	4
AT.AT	8	21071268	21071278	5.5	.	.	intergenic	-0.058	rs789821039	2
AGGG.CCCT	8	21189686	21189711	6.5	.	.	intergenic	-0.155	353653 rs7881	2
AAC.GTT	8	21539138	21539156	6.33333	.	.	intergenic	-0.097	540445 rs7870	2
AAAAC.GTTTT	8	21656833	21656854	4.4	.	.	intergenic	-0.228	rs790917211	2
ACAT.ATGT	8	21754505	21754517	3.25	.	.	intergenic	0.099	rs789773229	2
AAAC.GTTT	8	21894215	21894227	3.25	.	.	intergenic	-0.114	rs786890984	4
AC.GT	8	22079162	22079203	21	.	.	intergenic	-0.091	970618 rs7927	3
AT.AT	8	22133785	22133794	5	.	.	intergenic	.	.	3
AAT.ATT	8	22188500	22188515	5.33333	.	.	intergenic	0.068	.	2
AT.AT	8	22337656	22337665	5	.	.	intergenic	0.015	.	2
AC.GT	8	22379794	22379803	5	.	.	intergenic	0.043	rs793753928	2
AAAC.GTTT	8	22399964	22399985	5.5	.	.	intergenic	-0.31	rs792277616	3
AAAT.ATTT	8	22674705	22674727	5.75	.	.	intergenic	-0.139	rs713493605	4
AAAC.GTTT	8	22955070	22955094	6.25	.	.	intergenic	.	143056 rs7883	2
AAAC.GTTT	8	23063662	23063678	4.25	.	.	intergenic	0.074	093149 rs7916	2
AC.GT	8	23082991	23083007	8.5	.	.	intergenic	0.301	.	3
AG.CT	8	23083688	23083698	5.5	.	.	intergenic	-0.199	rs708205747	2
AAAT.ATTT	8	23104894	23104916	5.75	.	.	intergenic	-0.066	396527 rs7882	2
AAG.CTT	8	23161014	23161030	5.66667	.	.	intergenic	.	055530 rs7927	2
AC.GT	8	23270478	23270488	5.5	.	.	intergenic	0.02	.	2
AG.CT	8	23272861	23272873	6.5	.	.	intergenic	.	159799 rs7893	3
AGAT.ATCT	8	23301252	23301278	6.75	.	.	intergenic	-0.223	509091 rs7899	2
AG.CT	8	23303803	23303817	7.5	.	.	intergenic	-0.004	rs793085390	2
AAAC.GTTT	8	23311129	23311152	6	.	.	intergenic	0.301	095233 rs7909	2
AAC.GTT	8	23338330	23338346	5.66667	.	.	intergenic	-0.212	353831 rs7930	2
AAAAC.GTTTT	8	23342321	23342339	3.8	.	.	intergenic	0.025	527873 rs7926	2
AC.GT	8	23485109	23485125	8.5	.	.	intergenic	.	150187 rs7891	2

AAAC.GTTT	8	23530389	23530419	7.75	.	.	intergenic	-0.262	rs788232629	5
AAAT.ATTT	8	23531315	23531330	4	.	.	intergenic	0.729	157759 rs7900	2
AC.GT	8	23533563	23533575	6.5	.	.	intergenic	.	rs790756805	2
AC.GT	8	23721038	23721051	7	.	.	intergenic	-0.22	rs786453055	2
AAAAC.GTTTT	8	23728779	23728816	7.6	.	.	intergenic	-0.002	rs788537010	2
AAAT.ATTT	8	23836080	23836098	4.75	.	.	intergenic	0.026	382235 rs7881	2
AT.AT	8	24284282	24284292	5.5	.	.	intergenic	-0.141	456301 rs7893	3
AAAT.ATTT	8	24285174	24285198	6.25	.	.	intergenic	0.045	200550 rs7876	2
AG.CT	8	24309288	24309297	5	.	.	intergenic	-0.053	.	2
AG.CT	8	24397961	24397973	6.5	.	.	intergenic	-0.057	546995 rs7886	4
AAAC.GTTT	8	24423738	24423760	5.75	.	.	intergenic	0.472	rs792785548	2
AAT.ATT	8	24434067	24434084	6	.	.	intergenic	-0.106	547135 rs7932	2
AT.AT	8	24458867	24458876	5	.	.	intergenic	0.064	.	2
AAAC.GTTT	8	24460720	24460737	4.5	.	.	intergenic	-0.088	531541 rs7936	2
AC.GT	8	24614928	24614959	16	.	.	intergenic	0.281	914137 rs7870	2
AC.GT	8	24660213	24660222	5	.	.	intergenic	0.72	rs787476598	2
AC.GT	8	25037044	25037058	7.5	.	.	intergenic	0.557	556209 rs7872	2
AT.AT	8	25201314	25201331	9	.	.	intergenic	-0.08	rs790811561	2
AC.GT	8	25226314	25226323	5	.	.	intergenic	-0.07	rs788683554	2
AAT.ATT	8	25284207	25284235	9.66667	.	.	intergenic	0.036	585941 rs7881	2
AAG.CTT	8	25308246	25308262	5.66667	.	.	intergenic	0.426	rs788333189	2
AT.AT	8	25377397	25377406	5	.	.	intergenic	0.161	rs793755109	4
AC.GT	8	26081711	26081723	6.5	.	.	intergenic	.	rs792789401	2
AAAG.CTTT	8	26252523	26252536	3.5	.	.	intergenic	0.035	754489 rs7894	2
AC.GT	8	26283491	26283505	7.5	.	.	intergenic	-0.27	.	3
AT.AT	8	26512327	26512336	5	.	.	intergenic	-0.072	rs701813559	2
AAAC.GTTT	8	26516911	26516942	8	.	.	intergenic	.	rs793586506	2
AG.CT	8	26532775	26532789	7.5	.	.	intergenic	-0.016	rs789979221	2
AC.GT	8	26579419	26579435	8.5	.	.	intergenic	-0.156	.	2
AAAC.GTTT	8	26617082	26617111	7.5	.	.	intergenic	-0.126	470801 rs7906	3
AAAC.GTTT	8	26630224	26630250	6.75	.	.	intergenic	.	rs786725408	2
AAC.GTT	8	26631069	26631082	4.66667	.	.	intergenic	.	rs792214389	2
AATG.CATT	8	26819972	26819983	3	.	.	intergenic	.	rs793597849	3
AAAAC.GTTTT	8	26982051	26982074	4.8	.	.	intergenic	-0.054	503331 rs7875	2
AG.CT	8	26987922	26987932	5.5	.	.	intergenic	-0.035	.	3
AAAAC.GTTTT	8	26991507	26991535	5.8	.	.	intergenic	-0.051	rs786595901	2
AAT.ATT	8	27059365	27059378	4.66667	.	.	intergenic	-0.124	rs792864442	3
AT.AT	8	27086197	27086206	5	.	.	intergenic	-0.162	999843 rs7887	2
AAC.GTT	8	27101102	27101124	7.66667	.	.	intergenic	-0.039	395958 rs7894	4
AAAT.ATTT	8	27140914	27140927	3.5	.	.	intergenic	0.047	rs789346556	2
AAAC.GTTT	8	27305404	27305423	5	.	.	intergenic	.	rs790881240	2
AAAT.ATTT	8	27421998	27422011	3.5	.	.	intergenic	-0.219	rs788753815	2
AG.CT	8	27425782	27425792	5.5	.	.	intergenic	.	106438 rs7921	2
AAAT.ATTT	8	27497564	27497577	3.5	.	.	intergenic	-0.012	724855 rs7864	2
AAC.GTT	8	27714066	27714083	6	.	.	intergenic	.	rs786228671	2
AC.GT	8	27897672	27897684	6.5	.	.	intergenic	.	589677 rs7921	2
AAAT.ATTT	8	28030790	28030811	5.5	.	.	intergenic	.	rs791065062	2
AC.GT	8	28032165	28032174	5	.	.	intergenic	.	rs790670413	4
AT.AT	8	28074785	28074800	8	.	.	intergenic	.	rs789546675	2
AAC.GTT	8	28104872	28104895	8	.	.	intergenic	.	368925 rs7903	2
AC.GT	8	28189373	28189387	7.5	.	.	intergenic	0.045	571928 rs7903	2
AT.AT	8	28220015	28220026	6	.	.	intergenic	.	rs790355837	2
AAAC.GTTT	8	28287514	28287531	4.5	.	.	intergenic	0.176	rs792119380	2
AC.GT	8	28670727	28670741	7.5	.	.	intergenic	-0.098	.	2
AT.AT	8	28735653	28735662	5	.	.	intergenic	.	317601 rs7886	2
AAAAC.GTTTT	8	28815887	28815910	4.8	.	.	intergenic	-0.006	rs790311431	2
AC.GT	8	28989998	28990009	6	.	.	intergenic	.	rs787911091	2
AG.CT	8	29262424	29262433	5	ENSSSCT00000009593	ARAP2	intron	-0.026	rs790734834	2
AATG.CATT	8	29458152	29458169	4.5	.	.	intergenic	0.05	.	2
AC.GT	8	29670551	29670565	7.5	ENSSSCT00000009594	1242-305H!	promoter	-0.058	914255 rs7930	2
AC.GT	8	29670551	29670565	7.5	ENSSSCT00000035368	1242-305H!	promoter	-0.058	914255 rs7930	2
AC.GT	8	29670551	29670565	7.5	ENSSSCT00000034516	1242-305H!	promoter	-0.058	914255 rs7930	2
AC.GT	8	29670551	29670565	7.5	ENSSSCT00000034680	1242-305H!	promoter	-0.058	914255 rs7930	2
AAAC.GTTT	8	29859855	29859875	5.25	.	.	intergenic	0.077	105527 rs7896	2
AAG.CTT	8	29904521	29904534	4.66667	.	.	intergenic	0.133	rs792788226	2
AC.GT	8	30137629	30137642	7	.	.	intergenic	0.213	rs787466548	3
AG.CT	8	30404161	30404171	5.5	ENSSSCT00000009595	KIAA1239	intron	3.067	391296 rs7906	2
AAT.ATT	8	30425136	30425170	11.6667	.	.	intergenic	1.816	rs787320774	2
AC.GT	8	30513717	30513730	7	ENSSSCT00000009596	C4orf19	intron	-0.036	rs788478452	3
AATG.CATT	8	30616182	30616193	3	.	.	intergenic	.	.	2
AAC.GTT	8	30621743	30621756	4.66667	.	.	intergenic	0.165	228804 rs7912	2
AT.AT	8	30645871	30645882	6	.	.	intergenic	0.066	rs787801308	2
AC.GT	8	30690255	30690265	5.5	.	.	intergenic	0.228	rs791042953	2
AG.CT	8	30719326	30719338	6.5	ENSSSCT00000009598	PGM2	intron	-0.232	rs789935046	3
AAC.GTT	8	30797202	30797219	6	.	.	intergenic	0.214	584179 rs7907	2
AC.GT	8	31048391	31048404	7	ENSSSCT00000009599	TBC1D1	intron	-0.01	.	3
AGAGG.CCTCT	8	31099761	31099778	3	.	.	intergenic	-0.335	rs787542011	2
AG.CT	8	31348231	31348243	6.5	.	.	intergenic	0.117	148382 rs7900	3
AAGG.CCTT	8	31370576	31370610	8.75	.	.	intergenic	0.499	563162 rs7879	2
ACAT.ATGT	8	31426974	31427002	7.25	.	.	intergenic	0.235	011836 rs7868	2
AAG.CTT	8	31437919	31437938	6.66667	.	.	intergenic	0.035	rs791713743	2
AC.GT	8	31477779	31477794	8	.	.	intergenic	-0.007	rs788426590	4
AAC.GTT	8	31477904	31477917	4.66667	.	.	intergenic	-0.125	rs793507078	2
AAC.GTT	8	31487971	31487984	4.66667	.	.	intergenic	0.037	rs692826570	4

AGG.CCT	8	31554934	31554946	4.33333	.	.	intergenic	0.102	rs791090893	2
AT.AT	8	31715709	31715734	13	ENSSSCT00000009611	FAM114A1	intron	.	rs793154833	4
AC.GT	8	31870106	31870118	6.5	ENSSSCT00000009613	.	intron	0.174	942117 rs7932	2
AATAT.ATATT	8	31995419	31995443	5	.	.	intergenic	.	rs793298239	2
AC.GT	8	32082099	32082110	6	ENSSSCT00000009615	WDR19	intron	0.189	rs792250336	2
AC.GT	8	32082099	32082110	6	ENSSSCT00000027462	WDR19	intron	0.189	rs792250336	2
AC.GT	8	32212752	32212761	5	ENSSSCT00000009614	.	intron	0.255	.	2
AAT.ATT	8	32320975	32320991	5.66667	ENSSSCT00000022874	LIAS	intron	0.125	336555 rs7908	2
AAAC.GTTT	8	32526231	32526245	3.75	.	.	intergenic	.	rs787952444	2
AT.AT	8	32638552	32638569	9	ENSSSCT00000030457	UBE2K	intron	0.15	rs788084415	4
AC.GT	8	32646037	32646051	7.5	ENSSSCT00000030457	UBE2K	intron	0.159	724990 rs7891	2
AAAC.GTTT	8	33002359	33002379	5.25	ENSSSCT00000009621	RHOH	intron	.	291455 rs7862	2
AC.GT	8	33046700	33046717	9	.	.	intergenic	0.113	176750 rs7894	3
AG.CT	8	33101632	33101641	5	.	.	intergenic	-0.12	rs786651508	3
AGG.CCT	8	33129610	33129629	6.66667	.	.	intergenic	0.05	rs787275632	2
AC.GT	8	33165232	33165270	19.5	.	.	intergenic	-0.005	rs791542617	4
AAAC.GTTT	8	33209987	33210016	7.5	.	.	intergenic	.	.	2
AC.GT	8	33247471	33247482	6	ENSSSCT00000029046	APBB2	intron	.	.	4
AG.CT	8	33291939	33291951	6.5	ENSSSCT00000029046	APBB2	intron	0.128	151716 rs7907	2
AG.CT	8	33291939	33291951	6.5	ENSSSCT00000021654	SNORD113	promoter	0.128	151716 rs7907	2
AT.AT	8	33454763	33454782	10	ENSSSCT00000027797	NSUN7	intron	.	rs790423517	2
AT.AT	8	33717305	33717314	5	.	.	intergenic	-0.176	.	2
AAAC.GTTT	8	33743781	33743808	7	.	.	intergenic	-0.106	rs790306378	2
AAAC.GTTT	8	33824272	33824291	5	.	.	intergenic	0.086	rs792663288	2
AAAAG.CTTTT	8	33966283	33966303	4.2	.	.	intergenic	0.085	.	2
AG.CT	8	33967611	33967622	6	.	.	intergenic	0.118	rs790574690	2
AT.AT	8	34043070	34043094	12.5	.	.	intergenic	0.2	rs788111804	3
AAG.CTT	8	34136062	34136075	4.66667	ENSSSCT00000009626	.	intron	-0.092	rs791656724	2
AC.GT	8	34166939	34166951	6.5	ENSSSCT00000009626	.	intron	0.105	728622 rs7877	2
ACAT.ATGT	8	34198554	34198575	5.5	ENSSSCT00000009626	.	intron	-0.156	rs788626397	2
AG.CT	8	34262260	34262273	7	ENSSSCT00000009626	.	intron	0.017	rs791132607	2
AAC.GTT	8	34263337	34263351	5	ENSSSCT00000009626	.	intron	-0.141	269980 rs7875	2
AAAC.GTTT	8	34352923	34352946	6	ENSSSCT00000009626	.	intron	-0.065	566624 rs7928	2
AT.AT	8	34647687	34647696	5	.	.	intergenic	.	rs792295059	2
AAAC.GTTT	8	34731390	34731416	6.75	ENSSSCT00000009628	SLC30A9	intron	.	rs787596028	2
AG.CT	8	34908915	34908924	5	.	.	intergenic	0.067	992400 rs7935	4
AAAAC.GTTTT	8	34966860	34966881	4.4	.	.	intergenic	0.214	rs792201830	2
AC.GT	8	35394263	35394275	6.5	.	.	intergenic	-0.107	774794 rs7909	2
AAAAC.GTTTT	8	35533861	35533876	3.2	.	.	intergenic	0.451	rs695568172	2
AAGG.CCTT	8	35778458	35778482	6.25	.	.	intergenic	0.077	rs786693670	2
AAAAT.ATTTT	8	36297265	36297294	6	.	.	intergenic	-0.191	rs786491275	2
AT.AT	8	36833344	36833370	13.5	.	.	intergenic	0.004	rs788479877	2
AT.AT	8	36993685	36993700	8	.	.	intergenic	.	.	2
AG.CT	8	37010054	37010066	6.5	.	.	intergenic	.	rs792293909	2
AAAC.GTTT	8	37187549	37187560	3	.	.	intergenic	.	rs786590782	2
AC.GT	8	37318846	37318857	6	.	.	intergenic	-0.363	.	3
AC.GT	8	37358312	37358324	6.5	.	.	intergenic	0.095	rs787948698	2
AG.CT	8	37405184	37405194	5.5	.	.	intergenic	0.034	rs792420495	2
AT.AT	8	37422209	37422220	6	.	.	intergenic	0.356	rs786874686	2
AAAAC.GTTTT	8	37569781	37569800	4	.	.	intergenic	.	rs786645821	3
AAAC.GTTT	8	37727424	37727450	6.75	.	.	intergenic	-0.074	373158 rs7862	3
AG.CT	8	37757196	37757205	5	.	.	intergenic	-0.066	rs792407059	4
AAAAT.ATTTT	8	37844429	37844452	4.8	.	.	intergenic	-0.131	345338 rs7897	2
AC.GT	8	37874496	37874518	11.5	.	.	intergenic	-0.097	rs788459224	5
AT.AT	8	37875857	37875868	6	.	.	intergenic	0.13	.	2
AT.AT	8	37999636	37999647	6	.	.	intergenic	-0.262	rs788905739	2
AC.GT	8	38003387	38003405	9.5	.	.	intergenic	0.078	rs791120518	2
AAC.GTT	8	38025001	38025020	6.66667	ENSSSCT00000009639	GABRG1	intron	0.053	rs790037569	2
AG.CT	8	38259958	38259969	6	ENSSSCT00000023000	GABRA2	intron	-0.161	.	2
AT.AT	8	38274854	38274877	12	ENSSSCT00000023000	GABRA2	intron	0.062	.	2
ATC.GAT	8	38313655	38313673	6.33333	ENSSSCT00000023000	GABRA2	intron	0.058	.	2
AATC.GATT	8	38325328	38325358	7.75	ENSSSCT00000023000	GABRA2	intron	-0.017	140557 rs7927	2
AAAAAC.GTTTTT	8	38432853	38432882	5	.	.	intergenic	.	787864 rs7924	2
AT.AT	8	38440884	38440894	5.5	.	.	intergenic	0.415	rs790913325	4
AT.AT	8	38545155	38545171	8.5	.	.	intergenic	-0.135	rs786790150	2
AC.GT	8	38635356	38635383	14	.	.	intergenic	0.098	rs786450046	2
AAAC.GTTT	8	38659226	38659245	5	.	.	intergenic	.	247513 rs7901	2
AT.AT	8	38801343	38801357	7.5	ENSSSCT00000031430	GABRA4	intron	0.179	981030 rs7927	2
AC.GT	8	38941150	38941160	5.5	.	.	intergenic	0.055	416983 rs7867	2
AAAG.CTTT	8	38953467	38953483	4.25	.	.	intergenic	0.226	rs793401290	2
AAAG.CTTT	8	39007346	39007360	3.75	.	.	intergenic	.	rs788869750	2
AAC.GTT	8	39213325	39213342	6	.	.	intergenic	0.092	392486 rs7919	4
AAAT.ATTT	8	39331878	39331895	4.5	.	.	intergenic	0.123	rs787926822	4
AT.AT	8	39522402	39522413	6	ENSSSCT00000009643	CORIN	intron	.	578038 rs7929	2
AAG.CTT	8	39566798	39566817	6.66667	ENSSSCT00000009643	CORIN	intron	.	368262 rs7928	2
AC.GT	8	39571500	39571538	19.5	ENSSSCT00000009643	CORIN	intron	0.564	107595 rs7912	3
AAAC.GTTT	8	39650077	39650091	3.75	ENSSSCT00000009643	CORIN	intron	.	.	2
AG.CT	8	39667037	39667047	5.5	ENSSSCT00000009643	CORIN	intron	0.215	rs793042598	2
AAAAAT.ATTTTT	8	39711032	39711050	3.16667	ENSSSCT00000009643	CORIN	intron	.	rs788764519	2
AC.GT	8	40004049	40004063	7.5	ENSSSCT00000009647	.	5'utr	.	rs787891231	2
AAAC.GTTT	8	40113837	40113851	3.75	ENSSSCT00000009650	TEC	intron	.	rs787738707	2
AAAC.GTTT	8	40113837	40113851	3.75	ENSSSCT00000034638	TEC	intron	.	rs787738707	2
AAAC.GTTT	8	40113837	40113851	3.75	ENSSSCT00000032911	TEC	intron	.	rs787738707	2
AC.GT	8	40299614	40299630	8.5	ENSSSCT00000009651	SLAIN2	intron	0.169	313417 rs7898	4

AAAG.CTTT	8	40304801	40304821	5.25	ENSSSCT00000009651	SLAIN2	intron	0.22	rs789645700	2
AAAG.CTTT	8	40377412	40377434	5.75	.	.	intergenic	0.284	rs792034078	2
AATT.AATT	8	40664143	40664166	6	ENSSSCT00000009661	.	intron	0.073	;53409 rs7862	2
AATT.AATT	8	40664143	40664166	6	ENSSSCT00000031947	.	intron	0.073	;53409 rs7862	2
AATT.AATT	8	40664143	40664166	6	ENSSSCT00000009658	.	intron	0.073	;53409 rs7862	2
AAAAT.ATTTT	8	40817679	40817698	4	.	.	intergenic	.	rs793779590	2
AC.GT	8	41078009	41078021	6.5	ENSSSCT00000009664	CWH43	intron	0.293	.	3
ACCT.AGGT	8	41142287	41142317	7.75	.	.	intergenic	.	rs793454760	2
AAT.ATT	8	41209492	41209505	4.66667	ENSSSCT00000009666	DCUN1D4	intron	0.035	rs786252079	3
AAAC.GTTT	8	41313461	41313477	4.25	ENSSSCT00000009669	SGCB	intron	.	rs792319300	2
AG.CT	8	41478949	41478965	8.5	.	.	intergenic	-0.086	.	2
AC.GT	8	41479686	41479707	11	.	.	intergenic	0.31	rs788884059	3
AAAC.GTTT	8	41507953	41507979	6.75	.	.	intergenic	.	422525 rs7913	2
AAAAC.GTTTT	8	41571645	41571672	5.6	.	.	intergenic	.	rs786425298	2
AC.GT	8	41711513	41711526	7	.	.	intergenic	0.119	.	4
AAAC.GTTT	8	41779688	41779703	4	.	.	intergenic	.	rs787332826	2
AGAT.ATCT	8	41997816	41997830	3.75	.	.	intergenic	0.397	rs786238981	2
AAAC.GTTT	8	42005708	42005726	4.75	.	.	intergenic	0.151	rs791676588	2
AG.CT	8	42254432	42254442	5.5	ENSSSCT00000009672	.	intron	.	rs787270967	3
AG.CT	8	42283657	42283673	8.5	.	.	intergenic	0.513	rs790591198	2
AC.GT	8	42347517	42347527	5.5	.	.	intergenic	0.141	.	3
AAAAG.CTTTT	8	42567825	42567849	4.16667	.	.	intergenic	.	rs791931395	2
AC.GT	8	42606499	42606508	5	.	.	intergenic	.	rs788590117	2
AAC.GTT	8	42758195	42758211	5.66667	.	.	intergenic	0.112	rs786339186	3
AT.AT	8	42797523	42797546	12	.	.	intergenic	.	rs791913163	2
AAAT.ATTT	8	42821463	42821477	3.75	.	.	intergenic	0.38	.	2
AC.GT	8	42936250	42936263	7	.	.	intergenic	0.215	rs789387043	2
AGC.GCT	8	43184403	43184419	5.66667	.	.	intergenic	0.004	325193 rs7911	2
AGAT.ATCT	8	43359612	43359627	4	.	.	intergenic	0.124	rs786297559	2
AC.GT	8	43456336	43456350	7.5	.	.	intergenic	0.157	.	4
AC.GT	8	43571669	43571683	7.5	ENSSSCT00000009679	KIT	intron	0.311	291962 rs7925	2
ATC.GAT	8	43600998	43601015	6	ENSSSCT00000009679	KIT	3'utr	0.284	rs788115809	2
ACAT.ATGT	8	43751297	43751319	5.75	.	.	intergenic	-0.037	rs790076446	2
AAAT.ATTT	8	43756893	43756913	5.25	.	.	intergenic	-0.071	361952 rs7870	2
AG.CT	8	43861747	43861776	15	.	.	intergenic	-0.077	.	2
AC.GT	8	44173301	44173315	7.5	.	.	intergenic	.	106291 rs7885	2
ACAG.CTGT	8	44415305	44415319	3.75	.	.	intergenic	.	493438 rs7897	2
AT.AT	8	44583718	44583728	5.5	.	.	intergenic	.	rs786674947	2
AAAG.CTTT	8	44711347	44711365	4.75	.	.	intergenic	.	rs787124809	2
AC.GT	8	44790654	44790673	10	.	.	intergenic	.	rs791837194	2
AG.CT	8	44810410	44810420	5.5	.	.	intergenic	.	.	2
AC.GT	8	45195809	45195823	7.5	.	.	intergenic	.	900950 rs7921	4
AAAAAT.ATTTTT	8	45233188	45233206	3.16667	.	.	intergenic	-0.003	.	2
AAAC.GTTT	8	45245155	45245171	4.25	.	.	intergenic	-0.062	347394 rs7898	2
AT.AT	8	45323120	45323147	14	.	.	intergenic	0.094	rs793874905	2
AAAAC.GTTTT	8	45879863	45879892	6	.	.	intergenic	.	rs791407686	2
AAAAG.CTTTT	8	45879966	45879984	3.8	.	.	intergenic	.	rs789636985	2
AAAG.CTTT	8	46033898	46033925	7	.	.	intergenic	.	595770 rs7923	2
AAG.CTT	8	46185199	46185218	6.66667	.	.	intergenic	.	.	4
AT.AT	8	46326414	46326423	5	.	.	intergenic	-0.274	.	3
AAT.ATT	8	46679337	46679355	6.33333	.	.	intergenic	.	rs792287461	3
AT.AT	8	46698160	46698172	6.5	.	.	intergenic	.	578636 rs7888	2
AT.AT	8	46714320	46714344	12.5	ENSSSCT00000009708	TDO2	intron	-0.068	.	2
AT.AT	8	47108919	47108930	6	.	.	intergenic	.	168555 rs7891	2
AG.CT	8	47607888	47607912	12.5	.	.	intergenic	-0.308	rs791882825	2
AAAC.GTTT	8	47696141	47696168	7	ENSSSCT00000024403	PDGFC	intron	0.032	rs788914386	2
AG.CT	8	47747886	47747922	18.5	ENSSSCT00000024403	PDGFC	intron	0.044	rs793636139	2
ACAG.CTGT	8	47990166	47990184	4.75	.	.	intergenic	-0.052	rs786950441	2
AG.CT	8	48036276	48036285	5	.	.	intergenic	0.064	092168 rs7914	2
AC.GT	8	48269967	48269976	5	ENSSSCT00000022356	GRIA2	intron	.	.	4
AAC.GTT	8	48283207	48283223	5.66667	.	.	intergenic	0.064	179032 rs7931	2
AT.AT	8	48324057	48324067	5.5	.	.	intergenic	.	418410 rs7921	4
AG.CT	8	48410232	48410248	8.5	.	.	intergenic	.	983417 rs7912	2
AC.GT	8	48793442	48793461	10	.	.	intergenic	0.138	rs791550964	3
AC.GT	8	48802149	48802159	5.5	.	.	intergenic	-0.149	rs786395102	2
AAC.GTT	8	48978726	48978741	5.33333	.	.	intergenic	-0.186	rs786282356	3
AAAC.GTTT	8	49038798	49038813	4	.	.	intergenic	.	rs786835263	2
AAAC.GTTT	8	49199019	49199043	6.25	.	.	intergenic	0.211	rs790737468	2
AAAG.CTTT	8	49201555	49201567	3.25	.	.	intergenic	0.191	rs790049598	2
AAAC.GTTT	8	49289902	49289925	6	ENSSSCT00000009714	FAM198B	intron	.	rs791258912	2
AAAC.GTTT	8	49296999	49297037	9.75	ENSSSCT00000009714	FAM198B	intron	0.07	rs793214506	2
AC.GT	8	49368631	49368647	8.5	ENSSSCT00000009715	TMEM144	intron	.	719365 rs7870	2
AAAC.GTTT	8	49405200	49405225	6.5	ENSSSCT00000009715	TMEM144	intron	0.268	480486 rs7874	2
AC.GT	8	49491226	49491240	7.5	.	.	intergenic	-0.08	517473 rs7932	5
AG.CT	8	49940941	49940952	6	ENSSSCT00000009716	RXFP1	intron	-0.008	.	2
AATG.CATT	8	49942920	49942949	7.5	ENSSSCT00000009716	RXFP1	intron	0.159	384637 rs7910	2
AAAC.GTTT	8	50415079	50415103	6.25	ENSSSCT00000009724	RAPGEF2	intron	0.211	.	2
AAC.GTT	8	50433122	50433147	8.66667	ENSSSCT00000009724	RAPGEF2	intron	0.244	rs789906347	2
AC.GT	8	50441924	50441933	5	ENSSSCT00000009724	RAPGEF2	intron	-0.021	rs791499977	2
AAG.CTT	8	50448077	50448094	6	ENSSSCT00000009724	RAPGEF2	intron	0.109	rs787601230	2
AT.AT	8	50497456	50497466	5.5	ENSSSCT00000009724	RAPGEF2	intron	-0.145	.	2
AG.CT	8	50602985	50602999	7.5	ENSSSCT00000009724	RAPGEF2	intron	0.052	928640 rs7884	2
AAAC.GTTT	8	50776954	50776968	3.75	.	.	intergenic	0.108	rs791148525	2
AAG.CTT	8	50846585	50846604	6.66667	.	.	intergenic	-0.035	rs692361014	2

AAAG.CTTT	8	51045015	51045033	4.75	.	.	intergenic	.	316166 rs7934	3
AT.AT	8	51476416	51476426	5.5	.	.	intergenic	-0.255	.	2
AT.AT	8	51582470	51582479	5	.	.	intergenic	.	rs786400394	2
AT.AT	8	51754664	51754674	5.5	.	.	intergenic	.	rs789838316	2
AC.GT	8	52270189	52270199	5.5	.	.	intergenic	.	.	3
ACAT.ATGT	8	52663055	52663091	9.25	.	.	intergenic	-0.196	rs787339990	2
AAAAAT.ATTTTT	8	52911237	52911259	3.83333	.	.	intergenic	.	rs791299875	2
AG.CT	8	53036524	53036535	6	.	.	intergenic	-0.007	rs791123957	2
AAAC.GTTT	8	53471457	53471488	8	.	.	intergenic	-0.16	765810 rs7900	2
AAAC.GTTT	8	53592692	53592711	5	.	.	intergenic	-0.059	rs788981500	2
AAAT.ATTT	8	53854051	53854069	4.75	ENSSSCT00000031578	.	intron	-0.008	rs789656641	2
AT.AT	8	53905830	53905843	7	ENSSSCT00000031578	.	intron	-0.067	772649 rs7929	3
AT.AT	8	54107095	54107118	12	.	.	intergenic	-0.021	.	2
AT.AT	8	54196180	54196189	5	.	.	intergenic	.	rs793236790	2
AT.AT	8	54239430	54239450	10.5	.	.	intergenic	.	.	3
AT.AT	8	54253165	54253174	5	.	.	intergenic	.	rs793389893	2
AT.AT	8	54445307	54445320	7	.	.	intergenic	.	913267 rs7878	2
AAG.CTT	8	54494987	54495001	5	.	.	intergenic	.	rs712339377	3
AG.CT	8	54751514	54751530	8.5	.	.	intergenic	-0.017	.	3
AAAC.GTTT	8	55129746	55129772	6.75	ENSSSCT00000009730	NAF1	intron	.	597464 rs7887	2
AC.GT	8	55443037	55443049	6.5	.	.	intergenic	0.331	rs791863805	3
AAAG.CTTT	8	55487644	55487662	4.75	ENSSSCT00000009733	TMA16	intron	0.057	594924 rs7863	2
AAT.ATT	8	55707762	55707784	7.66667	.	.	intergenic	.	749853 rs7888	2
AC.GT	8	55806884	55806894	5.5	.	.	intergenic	-0.025	rs789365908	2
AAT.ATT	8	55938873	55938884	4	.	.	intergenic	.	rs790993968	2
AAAC.GTTT	8	57478310	57478324	3.75	.	.	intergenic	.	rs791713968	2
AAAAT.ATTTT	8	58250231	58250248	3.6	ENSSSCT00000031478	CEP135	intron	0.213	.	2
AAAC.GTTT	8	58364598	58364618	5.25	ENSSSCT00000024124	EXOC1	intron	.	rs789916947	2
AAAC.GTTT	8	58364598	58364618	5.25	ENSSSCT00000031053	EXOC1	intron	.	rs789916947	2
AG.CT	8	58384838	58384848	5.5	.	.	intergenic	.	.	3
AAAAC.GTTTT	8	59417862	59417882	4.2	.	.	intergenic	.	395923 rs7895	2
AAAT.ATTT	8	59554623	59554643	5.25	.	.	intergenic	-0.146	rs790788215	2
AAT.ATT	8	59557202	59557218	5.66667	.	.	intergenic	0.046	rs790304180	2
AGG.CCT	8	59827401	59827417	5.66667	.	.	intergenic	.	rs793360760	2
AG.CT	8	60008113	60008123	5.5	.	.	intergenic	-0.155	rs788836008	2
AAAAC.GTTTT	8	60040514	60040545	6.4	.	.	intergenic	-0.062	341506 rs7893	2
AC.GT	8	60041947	60041957	5.5	.	.	intergenic	.	rs790964506	2
AG.CT	8	60280893	60280903	5.5	.	.	intergenic	0.061	rs786739170	2
AT.AT	8	60447576	60447586	5.5	.	.	intergenic	0.046	rs786908465	2
AC.GT	8	61028293	61028314	11	.	.	intergenic	-0.024	rs792438350	2
AATAT.ATATT	8	61036649	61036668	4	.	.	intergenic	-0.073	533785 rs7910	2
AC.GT	8	61078556	61078567	6	.	.	intergenic	.	.	2
AG.CT	8	61154656	61154678	11.5	.	.	intergenic	-0.03	176603 rs7915	2
AC.GT	8	61180483	61180504	11	.	.	intergenic	-0.053	331109 rs7887	3
AC.GT	8	61224449	61224459	5.5	.	.	intergenic	0.076	.	2
AAAAG.CTTTT	8	61267183	61267217	7	.	.	intergenic	.	210466 rs7865	2
AC.GT	8	61804676	61804688	6.5	.	.	intergenic	-0.021	rs789556391	2
AAG.CTT	8	62910737	62910752	5.33333	.	.	intergenic	.	549029 rs7869	4
AT.AT	8	62915264	62915278	7.5	.	.	intergenic	-0.062	325458 rs7877	2
AC.GT	8	63452870	63452882	6.5	.	.	intergenic	0.023	rs693017382	3
AC.GT	8	63569659	63569675	8.5	.	.	intergenic	.	rs791043259	4
AAAC.GTTT	8	63945963	63945993	7.75	.	.	intergenic	.	rs789917065	2
AAT.ATT	8	64732492	64732505	4.66667	.	.	intergenic	.	rs792571248	2
AT.AT	8	64790943	64790953	5.5	.	.	intergenic	.	.	2
AT.AT	8	65113003	65113014	6	.	.	intergenic	-0.008	rs792448791	2
AC.GT	8	65575336	65575345	5	.	.	intergenic	0.042	.	4
AC.GT	8	66068232	66068250	9.5	.	.	intergenic	.	rs789776985	2
AAAAC.GTTTT	8	66128879	66128899	4.2	.	.	intergenic	.	rs792031846	2
AAC.GTT	8	66689283	66689294	4	.	.	intergenic	0.105	084391 rs7885	3
AT.AT	8	66695811	66695822	6	.	.	intergenic	-0.251	rs793584436	2
AT.AT	8	66894734	66894744	5.5	.	.	intergenic	-0.08	.	2
AAAT.ATTT	8	67082956	67082976	5.25	.	.	intergenic	0.032	rs793809298	2
AAAC.GTTT	8	67308353	67308366	3.5	ENSSSCT00000009766	EPHA5	intron	-0.058	303250 rs7925	2
AC.GT	8	67570966	67570979	7	.	.	intergenic	0.009	.	4
AAG.CTT	8	68164132	68164147	5.33333	.	.	intergenic	0.352	356026 rs7885	2
AG.CT	8	68635294	68635314	10.5	.	.	intergenic	-0.2	.	3
AC.GT	8	68691734	68691744	5.5	.	.	intergenic	.	229607 rs7885	2
AAAT.ATTT	8	68726332	68726346	3.75	.	.	intergenic	.	967193 rs7897	2
AT.AT	8	68729987	68729998	6	.	.	intergenic	.	rs787458737	3
AAAAG.CTTTT	8	68731269	68731286	3.6	.	.	intergenic	.	rs787254727	2
AT.AT	8	68751812	68751826	7.5	.	.	intergenic	-0.042	rs793659346	2
AT.AT	8	68797185	68797198	7	.	.	intergenic	0.167	064349 rs7898	2
AAAC.GTTT	8	68878094	68878124	7.75	.	.	intergenic	0.071	260381 rs7890	2
AAAC.GTTT	8	68879923	68879938	4	.	.	intergenic	0.164	rs789471982	2
AAGAG.CTCTT	8	68906245	68906270	5.2	.	.	intergenic	0.054	rs787470268	2
AAAC.GTTT	8	69050660	69050683	6	.	.	intergenic	0.243	384085 rs7938	2
AT.AT	8	69110618	69110628	5.5	.	.	intergenic	0.339	rs791033739	2
AAAT.ATTT	8	69171697	69171715	4.75	.	.	intergenic	0.041	558726 rs7881	2
AATAT.ATATT	8	69176466	69176480	3	.	.	intergenic	0.125	rs787802345	2
AAAT.ATTT	8	69570670	69570685	4	.	.	intergenic	.	rs786259550	2
AAAC.GTTT	8	69608389	69608418	7.5	.	.	intergenic	-0.142	117922 rs7897	3
AAAC.GTTT	8	69826818	69826840	5.75	ENSSSCT00000009772	UBA6	intron	.	.	2
AC.GT	8	70011717	70011728	6	ENSSSCT00000009774	MPRSS11I	intron	0.123	336963 rs7907	2
AC.GT	8	70042508	70042523	8	.	.	intergenic	0.073	.	4

AT.AT	8	70082277	70082287	5.5	ENSSSCT00000009775	TMPRSS11/	intron	-0.073	rs793760924	2
AATG.CATT	8	70217402	70217426	6.25	ENSSSCT00000009778	TMPRSS11I	intron	-0.106	570820 rs7916	3
AAAC.GTTT	8	70333636	70333650	3.75	.	.	intergenic	.	rs789811992	2
AAAT.ATTT	8	71010004	71010030	6.75	.	.	intergenic	-0.202	rs792021702	2
AC.GT	8	71066821	71066834	7	ENSSSCT00000022622	SULT1B1	intron	-0.033	.	4
AAAC.GTTT	8	71435419	71435452	8.5	ENSSSCT00000028087	RUFY3	intron	0.11	003582 rs7868	2
AC.GT	8	71465489	71465507	9.5	ENSSSCT00000028087	RUFY3	intron	0.209	rs790160040	2
AT.AT	8	71479301	71479312	6	.	.	intergenic	0.472	970210 rs7879	2
AAAC.GTTT	8	71626546	71626576	7.75	ENSSSCT00000009795	DCK	intron	.	rs697598100	2
AG.CT	8	71675210	71675226	8.5	.	.	intergenic	0.005	rs709910177	4
ACC.GGT	8	71756942	71756960	6.33333	.	.	intergenic	0.006	.	2
AGG.CCT	8	71932406	71932421	5.33333	ENSSSCT00000009797	SLC4A4	intron	-0.094	376467 rs7928	2
AAC.GTT	8	72175901	72175912	4	.	.	intergenic	0.303	rs791723288	2
AAAAC.GTTTT	8	72198053	72198087	7	.	.	intergenic	-0.011	783658 rs7915	2
AAAC.GTTT	8	72547496	72547516	5.25	ENSSSCT00000009798	NPFFR2	intron	-0.05	rs792840995	2
AAAC.GTTT	8	72615482	72615501	5	.	.	intergenic	0.001	rs790118981	2
AG.CT	8	73349289	73349301	6.5	ENSSSCT00000009801	COX18	intron	.	.	2
AT.AT	8	73401800	73401810	5.5	ENSSSCT00000028188	ANKRD17	intron	0.089	417081 rs7916	2
AAAC.GTTT	8	73645333	73645350	4.5	.	.	intergenic	.	rs789950904	2
AT.AT	8	73665856	73665866	5.5	.	.	intergenic	0.232	rs786622707	2
AAC.GTT	8	73856440	73856469	10	ENSSSCT00000026155	.	intron	.	rs789418853	2
AAAC.GTTT	8	74085318	74085332	3.75	ENSSSCT00000034805	IL8	exon	0.196	rs787169444	2
AAAC.GTTT	8	74085318	74085332	3.75	ENSSSCT00000009807	IL8	intron	0.196	rs787169444	2
AC.GT	8	74287530	74287549	10	ENSSSCT00000009813	CXCL2	intron	.	rs788330415	2
AC.GT	8	74345732	74345744	6.5	ENSSSCT00000009815	MTHFD2L	intron	0.069	346913 rs7869	3
AG.CT	8	74669315	74669325	5.5	.	.	intergenic	.	.	4
AATG.CATT	8	75003925	75003953	7.25	.	.	intergenic	0.07	rs787853987	2
AATG.CATT	8	75067531	75067552	5.5	.	.	intergenic	-0.058	rs787880101	2
AT.AT	8	75117399	75117414	8	.	.	intergenic	0.173	rs790883650	2
AC.GT	8	75176789	75176818	15	.	.	intergenic	0.112	388673 rs7869	4
AT.AT	8	75402189	75402206	9	.	.	intergenic	0.141	341467 rs7931	2
AAAT.ATTT	8	75633243	75633264	5.5	.	.	intergenic	.	rs703889887	2
AC.GT	8	75685581	75685595	7.5	ENSSSCT00000009827	PPEF2	intron	-0.141	273105 rs7937	3
AAC.GTT	8	75807173	75807186	4.66667	ENSSSCT00000023437	CXCL9	promoter	0.252	274640 rs7898	2
AT.AT	8	75999719	75999730	6	ENSSSCT00000009834	.	intron	0.277	rs792507106	2
AAG.CTT	8	76038457	76038469	4.33333	ENSSSCT00000009835	SCARB2	intron	0.148	rs791490063	2
AAAC.GTTT	8	76271101	76271115	3.75	ENSSSCT00000009840	CCDC158	intron	0.178	354887 rs7882	2
AAC.GTT	8	76283154	76283175	7.33333	ENSSSCT00000009840	CCDC158	intron	0.433	rs789528174	2
AC.GT	8	76552878	76552889	6	ENSSSCT00000009841	SHROOM3	intron	0.235	rs786629207	4
AAAT.ATTT	8	76622407	76622424	4.5	ENSSSCT00000009841	SHROOM3	intron	-0.158	rs790495872	2
AAAC.GTTT	8	76760036	76760055	5	.	.	intergenic	0.108	.	2
AGC.GCT	8	76760479	76760497	6.33333	.	.	intergenic	0.141	rs787744147	2
AAC.GTT	8	76799178	76799189	4	.	.	intergenic	0.208	344578 rs7898	2
AAAC.GTTT	8	76841025	76841042	4.5	ENSSSCT00000009843	11-Sep	intron	0.034	351911 rs7878	2
AAAAC.GTTTT	8	76886631	76886649	3.8	.	.	intergenic	-0.13	rs789016495	2
AAAAC.GTTTT	8	76940293	76940325	6.6	.	.	intergenic	0.207	376518 rs7872	2
AC.GT	8	76976357	76976367	5.5	.	.	intergenic	.	.	2
AAAC.GTTT	8	76978499	76978522	6	.	.	intergenic	0.355	509497 rs7867	2
AAAC.GTTT	8	77208423	77208460	9.5	.	.	intergenic	0.11	rs792264292	2
AT.AT	8	77281229	77281239	5.5	.	.	intergenic	.	.	2
AT.AT	8	77475392	77475403	6	.	.	intergenic	.	rs792092433	2
AAT.ATT	8	77575350	77575362	4.33333	.	.	intergenic	0.237	.	2
AGAT.ATCT	8	77936537	77936553	4.25	ENSSSCT00000009848	FRAS1	intron	0.102	rs792177883	2
AATG.CATT	8	77945365	77945379	3.75	ENSSSCT00000009848	FRAS1	intron	0.155	rs793504321	2
AC.GT	8	78013339	78013355	8.5	ENSSSCT00000009848	FRAS1	intron	0.274	rs793672627	2
AG.CT	8	78177671	78177680	5	ENSSSCT00000009848	FRAS1	intron	0.158	.	2
AG.CT	8	78177671	78177680	5	ENSSSCT00000033980	FRAS1	intron	0.158	.	2
AGC.GCT	8	78192532	78192546	5	ENSSSCT00000009848	FRAS1	intron	-0.104	rs793025269	2
AGC.GCT	8	78192532	78192546	5	ENSSSCT00000033980	FRAS1	intron	-0.104	rs793025269	2
AAAT.ATTT	8	78417267	78417288	5.5	.	.	intergenic	-0.149	rs791711702	2
AAAC.GTTT	8	78675449	78675471	5.75	.	.	intergenic	0.266	rs789644404	2
AAAT.ATTT	8	78744053	78744088	9	.	.	intergenic	.	091749 rs7880	2
AAAAC.GTTTT	8	78786674	78786703	6	.	.	intergenic	-0.085	rs790191916	2
AAAAG.CTTTT	8	78817776	78817793	3.6	.	.	intergenic	0.038	rs787947846	2
AC.GT	8	78823662	78823672	5.5	.	.	intergenic	.	167672 rs7928	4
ACAT.ATGT	8	79250477	79250492	4	.	.	intergenic	.	rs792215963	2
AC.GT	8	79294724	79294744	10.5	ENSSSCT00000009857	DCHS2	intron	.	.	2
AT.AT	8	79500546	79500567	11	.	.	intergenic	0.107	.	2
AAC.GTT	8	79570134	79570150	5.66667	.	.	intergenic	0.215	rs791836760	2
AC.GT	8	79869007	79869019	6.5	.	.	intergenic	0.086	350484 rs7929	2
AC.GT	8	80272492	80272507	8	ENSSSCT00000026657	.	intron	0.266	rs787826674	2
AC.GT	8	80595649	80595666	9	.	.	intergenic	-0.235	rs792390679	2
AAAC.GTTT	8	80937423	80937441	4.75	.	.	intergenic	.	rs789524571	4
AC.GT	8	80993290	80993300	5.5	.	.	intergenic	0.199	rs789829086	3
AAAC.GTTT	8	81072583	81072601	4.75	.	.	intergenic	0.25	.	2
AAAAC.GTTTT	8	81429119	81429138	4	.	.	intergenic	-0.299	rs792236132	3
AAAC.GTTT	8	81582691	81582708	4.5	.	.	intergenic	-0.038	rs788773572	2
AAAAT.ATTTT	8	81730123	81730149	5.4	.	.	intergenic	0.18	rs788081839	2
AC.GT	8	81864449	81864461	6.5	ENSSSCT00000009874	.	intron	-0.238	rs786425197	2
AT.AT	8	81885890	81885903	7	ENSSSCT00000009874	.	intron	.	527299 rs7869	2
AG.CT	8	81968730	81968740	5.5	ENSSSCT00000025424	.	intron	0.004	197022 rs7862	3
AG.CT	8	81968730	81968740	5.5	ENSSSCT00000026948	.	intron	0.004	197022 rs7862	3
AAAAC.GTTTT	8	82013506	82013524	3.8	.	.	intergenic	0.165	rs790437713	2
AG.CT	8	82631867	82631882	8	.	.	intergenic	.	.	4

AG.CT	8	83276796	83276818	11.5	ENSSSCT00000009880	.	intron	0.592	)70724 rs7912	2
AC.GT	8	83310175	83310189	7.5	ENSSSCT00000009880	.	intron	0.103	rs793851378	2
AC.GT	8	83716839	83716853	7.5	ENSSSCT00000009882	DCLK2	intron	-0.035	rs787433440	2
AAC.GTT	8	83779582	83779594	4.33333	.	.	intergenic	0.131	)26075 rs7931	2
AAAAC.GTTTT	8	83842194	83842213	4	.	.	intergenic	0.094	?77513 rs7931	2
AC.GT	8	83934926	83934936	5.5	.	.	intergenic	.	.	2
AT.AT	8	84187809	84187819	5.5	.	.	intergenic	0.056	rs793253003	2
AAG.CTT	8	84277153	84277167	5	.	.	intergenic	0.208	)31580 rs7885	2
AAAT.ATTT	8	84305658	84305676	4.75	.	.	intergenic	0.129	327282 rs7920	2
AAAT.ATTT	8	84527843	84527865	5.75	.	.	intergenic	0.068	rs793842754	2
AT.AT	8	84722179	84722193	7.5	.	.	intergenic	.	rs793070837	2
AC.GT	8	84918852	84918864	6.5	.	.	intergenic	-0.021	.	5
AAAC.GTTT	8	84945004	84945018	3.75	.	.	intergenic	.	rs788246631	2
AAC.GTT	8	85269813	85269839	9	.	.	intergenic	0.199	rs791800333	2
AAC.GTT	8	85414360	85414391	10.6667	.	.	intergenic	0.153	'89549 rs7895	2
AAAG.CTTT	8	85711930	85711954	6.25	ENSSSCT00000009887	.	intron	0.045	.	2
AAAAG.CTTTT	8	85865503	85865520	3.6	.	.	intergenic	-0.086	rs788365714	2
AAC.GTT	8	85950160	85950173	4.66667	ENSSSCT00000009888	ARHGAP1(	intron	-0.246	rs791978842	2
ATC.GAT	8	86234778	86234805	9.33333	ENSSSCT00000024567	TMEM184C	intron	0.348	rs788579003	2
AC.GT	8	86390457	86390469	6.5	.	.	intergenic	.	rs789663181	2
AAG.CTT	8	86510398	86510411	4.66667	.	.	intergenic	-0.119	325773 rs7876	2
AAAAT.ATTTT	8	86541751	86541765	3	.	.	intergenic	0.048	391942 rs7887	2
AAGG.CCTT	8	86846923	86846941	4.75	.	.	intergenic	0.111	rs793300148	2
AG.CT	8	87116246	87116256	5.5	.	.	intergenic	.	481012 rs7896	2
AAAC.GTTT	8	87291578	87291599	5.5	ENSSSCT00000009895	SLC10A7	intron	.	rs691279881	3
AAAG.CTTT	8	87522887	87522928	10.5	ENSSSCT00000009895	SLC10A7	intron	0.138	)52842 rs7935	2
AG.CT	8	87720708	87720718	5.5	ENSSSCT00000009896	LSM6	intron	0.096	rs792747840	2
AC.GT	8	87939074	87939086	6.5	ENSSSCT00000009897	ZNF827	intron	0.236	rs711022675	2
AC.GT	8	88065599	88065612	7	ENSSSCT00000009897	ZNF827	intron	-0.126	rs789759401	2
ACACT.AGTGT	8	88165874	88165904	6.2	ENSSSCT00000009899	MMAA	intron	-0.095	rs786278734	2
AAT.ATT	8	88184186	88184200	5	.	.	intergenic	0.031	rs791654811	2
ATC.GAT	8	88322671	88322691	7	.	.	intergenic	0.148	.	2
AT.AT	8	88546363	88546375	6.5	ENSSSCT00000009902	OTUD4	intron	0.019	.	4
AT.AT	8	88621518	88621534	8.5	ENSSSCT00000009903	ABCE1	intron	0.196	rs790966375	2
AAAAC.GTTTT	8	88739397	88739417	4.2	.	.	intergenic	.	rs793582133	2
AT.AT	8	88996470	88996489	10	.	.	intergenic	0.328	)27356 rs7869	3
AAAAT.ATTTT	8	89125837	89125854	3.6	.	.	intergenic	0.134	rs792161080	2
AG.CT	8	89284309	89284319	5.5	.	.	intergenic	0.059	rs706713104	2
AC.GT	8	89296098	89296120	11.5	.	.	intergenic	-0.048	rs698405364	2
AAC.GTT	8	89415374	89415390	5.66667	ENSSSCT00000035764	GAB1	intron	-0.011	rs788670556	2
AAC.GTT	8	89415374	89415390	5.66667	ENSSSCT00000032897	GAB1	intron	-0.011	rs788670556	2
AAC.GTT	8	89415374	89415390	5.66667	ENSSSCT00000009909	GAB1	intron	-0.011	rs788670556	2
AAC.GTT	8	89415374	89415390	5.66667	ENSSSCT00000034785	GAB1	intron	-0.011	rs788670556	2
ACAT.ATGT	8	89442988	89443003	4	ENSSSCT00000035764	GAB1	intron	-0.058	rs792609848	2
ACAT.ATGT	8	89442988	89443003	4	ENSSSCT00000032897	GAB1	intron	-0.058	rs792609848	2
ACAT.ATGT	8	89442988	89443003	4	ENSSSCT00000009909	GAB1	intron	-0.058	rs792609848	2
ACAT.ATGT	8	89442988	89443003	4	ENSSSCT00000034785	GAB1	intron	-0.058	rs792609848	2
AC.GT	8	89698438	89698455	9	.	.	intergenic	.	.	4
\AAAAC.GTTTT'	8	89809327	89809350	4	.	.	intergenic	.	rs786532742	2
AT.AT	8	89828034	89828043	5	.	.	intergenic	.	rs791236800	2
AT.AT	8	89970000	89970011	6	.	.	intergenic	.	rs791283052	2
AC.GT	8	90061360	90061372	6.5	.	.	intergenic	.	.	3
AAAAG.CTTTT	8	90163843	90163870	5.6	.	.	intergenic	-0.319	.	2
ACAT.ATGT	8	90200161	90200184	6	.	.	intergenic	0.086	rs701251270	2
AAAC.GTTT	8	90475084	90475104	5.25	.	.	intergenic	0.02	rs789986005	2
\AAAAG.CTTTT'	8	90568094	90568121	4.66667	.	.	intergenic	-0.027	rs788664084	2
AT.AT	8	91041190	91041200	5.5	.	.	intergenic	-0.21	.	3
AT.AT	8	91047381	91047390	5	.	.	intergenic	0.162	.	2
AAAG.CTTT	8	91159653	91159676	6	.	.	intergenic	0.223	582107 rs7935	2
AC.GT	8	91406975	91406985	5.5	.	.	intergenic	.	rs704900651	2
AAT.ATT	8	91429833	91429859	9	.	.	intergenic	.	.	2
AT.AT	8	91673450	91673461	6	ENSSSCT00000009917	RNF150	intron	0.082	rs793444097	2
AAAC.GTTT	8	91820233	91820254	5.5	.	.	intergenic	0.005	I33014 rs7881	2
AG.CT	8	91929226	91929239	7	.	.	intergenic	0.909	rs788170884	2
\AAAAC.GTTTT'	8	91942921	91942946	4.33333	.	.	intergenic	4.676	394968 rs7932	2
AT.AT	8	92117882	92117898	8.5	ENSSSCT00000025519	CLGN	intron	.	I45227 rs7912	2
AAAC.GTTT	8	92349182	92349209	7	.	.	intergenic	.	rs692904740	3
AC.GT	8	92360455	92360469	7.5	.	.	intergenic	-0.108	rs792902566	2
AAAG.CTTT	8	92412029	92412060	8	.	.	intergenic	0.13	510659 rs7913	2
AAAAG.CTTTT	8	92413018	92413036	3.8	.	.	intergenic	-0.118	303559 rs7921	2
AG.CT	8	92443487	92443503	8.5	.	.	intergenic	1.363	.	2
AAAC.GTTT	8	92471131	92471154	6	.	.	intergenic	0.05	rs793575196	2
AG.CT	8	92776289	92776313	12.5	ENSSSCT00000009924	MAML3	intron	0.234	)24362 rs7894	2
AAAT.ATTT	8	92794722	92794742	5.25	ENSSSCT00000009924	MAML3	intron	0.014	rs789976924	2
AC.GT	8	92844092	92844103	6	ENSSSCT00000009924	MAML3	intron	-0.016	rs790696088	2
AAAT.ATTT	8	93171338	93171359	5.5	ENSSSCT00000023019	SETD7	promoter	-0.012	rs793676997	2
AAAC.GTTT	8	93273732	93273743	3	ENSSSCT00000009925	NAA15	intron	0.302	.	2
AGG.CCT	8	93637680	93637711	10.6667	.	.	intergenic	-0.015	rs789146764	4
GAGGG.CCCTC	8	93655286	93655304	3.16667	.	.	intergenic	0.312	502878 rs7891	2
AC.GT	8	93683940	93683958	9.5	.	.	intergenic	0.125	?44728 rs7921	2
AAT.ATT	8	93700622	93700641	6.66667	.	.	intergenic	-0.114	rs793011633	2
AAAC.GTTT	8	93789494	93789509	4	.	.	intergenic	0.89	rs788256051	4
AG.CT	8	93832230	93832246	8.5	.	.	intergenic	0.32	rs792370359	2
\AAAAC.GTTTT'	8	93915894	93915921	4.66667	.	.	intergenic	.	)20826 rs7882	2

AAAAT.ATTTT	8	93933067	93933093	5.4	.	.	intergenic	0.031	rs791942175	2
AC.GT	8	94000650	94000673	12	.	.	intergenic	0.199	006451 rs7917	2
AT.AT	8	94721307	94721322	8	.	.	intergenic	.	rs788303461	2
AAAAT.ATTTT	8	94742901	94742934	6.8	.	.	intergenic	0.072	571436 rs7895	2
AC.GT	8	94977359	94977369	5.5	.	.	intergenic	-0.1	.	4
AT.AT	8	95017884	95017894	5.5	.	.	intergenic	0.068	.	3
AC.GT	8	95067400	95067413	7	.	.	intergenic	-0.069	.	3
AAAT.ATTT	8	95614715	95614727	3.25	.	.	intergenic	-0.37	rs792132135	2
AT.AT	8	95621757	95621768	6	.	.	intergenic	.	rs789346333	2
AAAT.ATTT	8	95952526	95952544	4.75	.	.	intergenic	-0.218	rs793514596	2
AC.GT	8	96173489	96173508	10	.	.	intergenic	0.026	.	4
AC.GT	8	96650931	96650956	13	.	.	intergenic	-0.14	rs702409172	2
AC.GT	8	96744815	96744827	6.5	.	.	intergenic	.	.	3
AT.AT	8	96756116	96756127	6	.	.	intergenic	.	rs793734143	2
AAC.GTT	8	96864549	96864575	9	.	.	intergenic	0.11	376630 rs7880	2
AAT.ATT	8	97207244	97207266	7.66667	.	.	intergenic	.	.	2
AAT.ATT	8	97450882	97450893	4	.	.	intergenic	-0.077	922870 rs7910	2
AT.AT	8	97593048	97593059	6	.	.	intergenic	-0.039	rs793203853	2
AAT.ATT	8	97594584	97594596	4.33333	.	.	intergenic	0.034	512499 rs7871	2
ACAT.ATGT	8	97960232	97960256	6.25	.	.	intergenic	-0.136	rs793563877	2
AAAAC.GTTTT	8	98473310	98473330	4.2	.	.	intergenic	0.136	762525 rs7928	2
AT.AT	8	98667735	98667744	5	.	.	intergenic	-0.073	rs790893134	2
AAAC.GTTT	8	99048097	99048122	6.5	.	.	intergenic	-0.133	rs789978003	2
AC.GT	8	99729898	99729910	6.5	.	.	intergenic	-0.11	rs792405928	2
AC.GT	8	99817925	99817949	12.5	.	.	intergenic	.	.	2
AAG.CTT	8	99846058	99846075	6	.	.	intergenic	0.356	951730 rs6964	2
AAT.ATT	8	99918683	99918695	4.33333	.	.	intergenic	-0.101	267361 rs7894	2
AAAATT.AATTTT	8	100234007	100234034	4.66667	.	.	intergenic	.	.	2
AT.AT	8	100275194	100275204	5.5	.	.	intergenic	0.443	.	2
AAC.GTT	8	100401074	100401086	4.33333	.	.	intergenic	0.057	.	2
AC.GT	8	100444141	100444167	13.5	.	.	intergenic	0.094	949730 rs7866	3
AAAC.GTTT	8	100645317	100645344	7	.	.	intergenic	.	rs791913849	2
AT.AT	8	100836210	100836220	5.5	.	.	intergenic	0.095	148702 rs7932	2
AAAC.GTTT	8	100837552	100837569	4.5	.	.	intergenic	.	rs787056481	2
AT.AT	8	100856552	100856579	14	.	.	intergenic	-0.158	.	2
AG.CT	8	101080528	101080540	6.5	.	.	intergenic	0.347	rs789979081	2
AC.GT	8	101333963	101333978	8	.	.	intergenic	-0.051	168895 rs7881	2
AAG.CTT	8	101415998	101416010	4.33333	.	.	intergenic	-0.271	524760 rs7938	2
AG.CT	8	101495129	101495141	6.5	.	.	intergenic	0.143	542192 rs7873	2
AAG.CTT	8	101700130	101700145	5.33333	.	.	intergenic	.	rs786424775	2
AAT.ATT	8	101701559	101701573	5	.	.	intergenic	-0.044	347959 rs7911	2
AAC.GTT	8	101746947	101746958	4	.	.	intergenic	0.09	rs791926486	4
AAATT.AATTT	8	101834134	101834148	3	.	.	intergenic	-0.089	rs791137508	2
AAAG.CTTT	8	102181908	102181932	6.25	.	.	intergenic	0.631	.	2
AT.AT	8	102185659	102185674	8	.	.	intergenic	-0.024	rs710100018	4
AT.AT	8	102285986	102285995	5	.	.	intergenic	.	rs787640464	3
AG.CT	8	102347106	102347122	8.5	.	.	intergenic	0.86	969401 rs7863	2
AT.AT	8	102521872	102521881	5	.	.	intergenic	.	rs790207742	2
AT.AT	8	102970805	102970821	8.5	.	.	intergenic	0.31	rs790056631	2
AG.CT	8	103259266	103259282	8.5	.	.	intergenic	-0.072	rs786630660	4
AT.AT	8	103326644	103326657	7	.	.	intergenic	.	547645 rs7926	4
ACAT.ATGT	8	103452423	103452442	5	.	.	intergenic	0.08	rs787222517	2
AAAT.ATTT	8	103551882	103551908	6.75	.	.	intergenic	.	.	3
AAC.GTT	8	103588600	103588613	4.66667	.	.	intergenic	0.267	rs786235878	2
AG.CT	8	104156558	104156570	6.5	.	.	intergenic	0.117	rs791212598	2
AC.GT	8	104174261	104174273	6.5	.	.	intergenic	0.267	459430 rs7864	2
AT.AT	8	104487782	104487812	15.5	.	.	intergenic	0.117	rs791965747	2
AC.GT	8	104614747	104614760	7	.	.	intergenic	0.173	338966 rs7870	4
AAAT.ATTT	8	104830684	104830700	4.25	.	.	intergenic	.	rs789251433	2
AT.AT	8	104873226	104873236	5.5	.	.	intergenic	0.031	rs787418768	3
AT.AT	8	104892053	104892067	7.5	.	.	intergenic	0.075	148484 rs7890	2
AT.AT	8	104982112	104982122	5.5	.	.	intergenic	0.115	rs792059120	4
AC.GT	8	104995039	104995059	10.5	.	.	intergenic	-0.088	rs792114878	4
AAT.ATT	8	105201215	105201228	4.66667	.	.	intergenic	0.06	rs787373892	2
AT.AT	8	105208019	105208030	6	.	.	intergenic	0.173	rs786486203	2
AAAC.GTTT	8	105296521	105296535	3.75	.	.	intergenic	0.164	rs793715082	2
AAAC.GTTT	8	105302566	105302594	7.25	.	.	intergenic	0.138	954657 rs7863	2
AAT.ATT	8	105382277	105382302	8.66667	.	.	intergenic	0.09	rs789047280	2
AC.GT	8	105801999	105802016	9	.	.	intergenic	-0.097	594229 rs7912	2
AAC.GTT	8	106293540	106293555	5.33333	.	.	intergenic	0.145	rs788424993	2
AAC.GTT	8	106575308	106575320	4.33333	.	.	intergenic	.	376083 rs7928	2
AAAC.GTTT	8	106678236	106678262	6.75	.	.	intergenic	0.064	rs793088360	2
AT.AT	8	106853438	106853457	10	.	.	intergenic	0.118	rs789312532	2
AG.CT	8	107110857	107110869	6.5	.	.	intergenic	-0.051	306728 rs7938	2
AC.GT	8	107256051	107256071	10.5	.	.	intergenic	.	.	2
AAC.GTT	8	107263218	107263232	5	.	.	intergenic	.	.	2
AAC.GTT	8	107439524	107439537	4.66667	.	.	intergenic	0.834	272483 rs7932	2
CACAG.CTGTG	8	107541120	107541143	4	.	.	intergenic	0.705	rs702978370	2
AAAC.GTTT	8	107545062	107545084	5.75	.	.	intergenic	0.252	rs793206206	2
AAAAC.GTTTT	8	107760093	107760111	3.8	.	.	intergenic	0.026	rs789072172	2
AAT.ATT	8	107951135	107951156	7.33333	.	.	intergenic	0.287	.	2
AC.GT	8	108092922	108092945	12	ENSSSCT00000009949	SPRY1	intron	.	.	4
AAAC.GTTT	8	108143946	108143968	5.75	ENSSSCT00000009949	SPRY1	intron	0.061	rs791728360	2
AT.AT	8	108262947	108262956	5	.	.	intergenic	0.312	rs788404786	2

AAAG.CTTT	8	108381713	108381753	10.25	ENSSSCT00000009950	SPATA5	intron	0.232	210723 rs7903	3
AG.CT	8	108388784	108388798	7.5	ENSSSCT00000009950	SPATA5	intron	.	214349 rs7880	2
AT.AT	8	108485432	108485444	6.5	ENSSSCT00000009951	NUDT6	intron	0.167	.	2
AAAT.ATTT	8	108495015	108495027	3.25	ENSSSCT00000009952	FGF2	intron	0.157	.	2
AG.CT	8	108609204	108609214	5.5	.	.	intergenic	0.972	.	4
AAAC.GTTT	8	108995826	108995840	3.75	ENSSSCT00000009956	KIAA1109	intron	.	rs792396051	2
AT.AT	8	108999661	108999678	9	ENSSSCT00000009956	KIAA1109	intron	.	.	2
AAG.CTT	8	109681454	109681466	4.33333	.	.	intergenic	.	rs788384789	2
AC.GT	8	110097270	110097284	7.5	.	.	intergenic	0.121	.	2
GAGAT.ATCTC	8	110513523	110513541	3.16667	ENSSSCT00000030696	.	intron	-0.002	rs792516854	2
AAAC.GTTT	8	110558082	110558104	5.75	.	.	intergenic	0.14	rs786901201	2
AT.AT	8	110765689	110765698	5	ENSSSCT00000009967	PRDM5	intron	-0.002	578097 rs7929	2
AT.AT	8	110765689	110765698	5	ENSSSCT00000021244	U6	promoter	-0.002	578097 rs7929	2
AAAC.GTTT	8	111071878	111071904	6.75	.	.	intergenic	0.084	111202 rs7902	2
AC.GT	8	111106857	111106868	6	.	.	intergenic	.	508491 rs7884	2
AAT.ATT	8	111195550	111195563	4.66667	.	.	intergenic	0.128	rs786430177	2
AG.CT	8	111288955	111288967	6.5	.	.	intergenic	-0.146	527598 rs7881	2
AC.GT	8	111303333	111303345	6.5	.	.	intergenic	-0.098	rs790480222	2
AAAT.ATTT	8	111378902	111378919	4.5	.	.	intergenic	.	015065 rs7885	2
AAAG.CTTT	8	111403123	111403141	4.75	.	.	intergenic	0.166	169556 rs7907	2
AC.GT	8	111495352	111495363	6	ENSSSCT00000009971	MAD2L1	promoter	0.018	.	2
AG.CT	8	111591447	111591456	5	.	.	intergenic	.	rs690841189	4
AAAG.CTTT	8	111636268	111636291	6	.	.	intergenic	0.144	089676 rs7863	2
AAAT.ATTT	8	111994856	111994871	4	ENSSSCT00000009973	PDE5A	intron	.	.	2
AAAAAG.CTTTTT	8	112041190	112041211	3.66667	ENSSSCT00000009973	PDE5A	intron	0.095	rs788113733	2
AAAAG.CTTTT	8	112122113	112122129	3.4	ENSSSCT00000035856	FABP2	intron	0.128	394632 rs7876	2
AAAAG.CTTTT	8	112122113	112122129	3.4	ENSSSCT00000030658	FABP2	intron	0.128	394632 rs7876	2
AAAC.GTTT	8	112135087	112135100	3.5	.	.	intergenic	.	rs790237937	2
AAAC.GTTT	8	112603697	112603719	5.75	ENSSSCT00000009979	.	intron	-0.041	rs789549504	2
AAAC.GTTT	8	112809590	112809617	7	.	.	intergenic	.	rs791407909	2
AT.AT	8	112820185	112820199	7.5	.	.	intergenic	0.047	.	2
ACACT.AGTGT	8	112900431	112900445	3	ENSSSCT00000009981	PRSS12	intron	0.108	rs786771795	2
AAAC.GTTT	8	112903285	112903310	6.5	ENSSSCT00000009981	PRSS12	intron	0.087	573533 rs7880	2
AT.AT	8	112996784	112996794	5.5	ENSSSCT00000009982	NDST3	intron	.	.	4
AC.GT	8	113257079	113257094	8	.	.	intergenic	0.042	rs791835984	2
AAAAT.ATTTT	8	113270875	113270892	3.6	.	.	intergenic	0.38	rs787778535	2
AGC.GCT	8	113289327	113289342	5.33333	.	.	intergenic	0.163	rs787003031	2
AAC.GTT	8	113318763	113318776	4.66667	.	.	intergenic	-0.019	rs786861597	2
AAAT.ATTT	8	113606573	113606597	6.25	.	.	intergenic	.	rs788957871	2
AC.GT	8	113688705	113688720	8	.	.	intergenic	-0.015	373231 rs7925	2
AAGG.CCTT	8	113699388	113699407	5	.	.	intergenic	.	.	2
AC.GT	8	114045852	114045862	5.5	.	.	intergenic	.	rs792796016	3
AT.AT	8	114292780	114292789	5	.	.	intergenic	-0.158	rs786224148	2
AC.GT	8	114553226	114553237	6	.	.	intergenic	-0.077	rs786765022	2
AC.GT	8	114973363	114973377	7.5	.	.	intergenic	.	rs793548375	2
AC.GT	8	115438765	115438776	6	.	.	intergenic	.	.	2
AC.GT	8	115442798	115442824	13.5	.	.	intergenic	.	rs788483292	3
AG.CT	8	115597542	115597552	5.5	.	.	intergenic	0.033	rs790638724	2
AG.CT	8	115645949	115645959	5.5	.	.	intergenic	-0.225	.	4
AG.CT	8	115845728	115845748	10.5	.	.	intergenic	-0.037	483552 rs7874	2
AG.CT	8	115880801	115880815	7.5	.	.	intergenic	.	343722 rs7920	2
AC.GT	8	115972875	115972921	23.5	ENSSSCT00000029398	UGT8	3'utr	1.228	589545 rs7882	2
AG.CT	8	116233051	116233064	7	ENSSSCT00000024625	.	intron	-0.172	.	2
AG.CT	8	116368299	116368312	7	.	.	intergenic	0.14	.	3
AATG.CATT	8	116830221	116830234	3.5	.	.	intergenic	0.048	.	2
AAAAC.GTTTT	8	116948960	116948979	4	.	.	intergenic	0.145	rs787665149	2
AC.GT	8	116956673	116956688	8	ENSSSCT00000009990	CAMK2D	intron	0.854	355099 rs7891	2
AAAC.GTTT	8	116976907	116976924	4.5	ENSSSCT00000009990	CAMK2D	intron	0.134	rs789776342	2
AAAAC.GTTTT	8	117095484	117095514	6.2	ENSSSCT00000009990	CAMK2D	intron	0.022	rs788878118	2
AAG.CTT	8	117167096	117167107	4	ENSSSCT00000009990	CAMK2D	intron	.	589042 rs7923	2
AAAAT.ATTTT	8	117451972	117451994	4.6	ENSSSCT00000009992	ANK2	intron	0.1	rs791707787	2
AAAG.CTTT	8	117541785	117541803	4.75	ENSSSCT00000009992	ANK2	intron	0.095	480799 rs7928	2
AAC.GTT	8	117778506	117778517	4	.	.	intergenic	.	rs791705206	2
AAAT.ATTT	8	117911169	117911196	7	.	.	intergenic	.	.	2
AG.CT	8	117969106	117969120	7.5	.	.	intergenic	0.135	019882 rs7923	2
AAAT.ATTT	8	118281050	118281071	5.5	.	.	intergenic	0.015	438271 rs7932	3
AAT.ATT	8	118376886	118376906	7	ENSSSCT00000009996	AP1AR	intron	0.268	940800 rs7862	4
AC.GT	8	118414878	118414894	8.5	.	.	intergenic	.	.	2
AT.AT	8	118517659	118517684	13	.	.	intergenic	0.328	rs701802493	2
AAAT.ATTT	8	118709493	118709510	4.5	.	.	intergenic	0.019	.	2
AAAC.GTTT	8	118846705	118846723	4.75	.	.	intergenic	.	311913 rs7928	2
AG.CT	8	118914274	118914289	8	.	.	intergenic	0.07	940658 rs7071	2
AAAAAC.GTTTTT	8	118937719	118937758	6.66667	.	.	intergenic	-0.065	rs792488552	3
ATC.GAT	8	119088909	119088933	8.33333	.	.	intergenic	.	rs793541332	3
AAAC.GTTT	8	119274291	119274322	8	.	.	intergenic	0.049	363414 rs7918	2
AT.AT	8	119277574	119277608	17.5	.	.	intergenic	.	rs786908678	2
AAAG.CTTT	8	119376537	119376561	6.25	.	.	intergenic	0.585	151872 rs7919	2
AAC.GTT	8	119497860	119497873	4.66667	.	.	intergenic	1.4	451216 rs7887	2
AC.GT	8	119715124	119715145	11	.	.	intergenic	0.444	378112 rs7919	3
AAT.ATT	8	119753001	119753017	5.66667	.	.	intergenic	0.046	rs793214643	2
AAAT.ATTT	8	119841495	119841526	8	.	.	intergenic	0.113	567433 rs7922	2
AAAG.CTTT	8	119842718	119842735	4.5	.	.	intergenic	0.076	rs792461767	2
AAAC.GTTT	8	119939656	119939667	3	.	.	intergenic	-0.247	rs788918506	2
ACT.AGT	8	119981230	119981241	4	ENSSSCT00000036057	ENPEP	intron	0.154	308348 rs7935	2

ACT.AGT	8	119981230	119981241	4	ENSSSCT00000010000	ENPEP	intron	0.154	308348 rs7935	2
AAAAG.CTTTT	8	119992546	119992562	3.4	ENSSSCT00000010000	ENPEP	intron	1.055	rs786399137	2
AGGG.CCCT	8	120160210	120160221	3	ENSSSCT00000024189	ELOVL6	intron	0.023	.	2
ACATAT.ATATG	8	120174113	120174131	3.16667	ENSSSCT00000024189	ELOVL6	intron	.	.	2
AT.AT	8	120202080	120202090	5.5	ENSSSCT00000024189	ELOVL6	intron	-0.058	.	4
AAAAC.GTTTT	8	120227275	120227303	5.8	.	.	intergenic	.	rs792340338	2
AAAG.CTTT	8	120483186	120483200	3.75	ENSSSCT00000010004	RRH	promoter	0.124	rs792431228	2
AT.AT	8	120607456	120607466	5.5	.	.	intergenic	.	.	4
AAAAT.ATTTT	8	121247229	121247245	3.4	.	.	intergenic	0.127	rs790804683	2
AGG.CCT	8	121335063	121335080	6	.	.	intergenic	.	rs787964174	2
AC.GT	8	121411454	121411466	6.5	ENSSSCT00000010011	.	intron	0.031	599918 rs7914	2
AAAC.GTTT	8	121434172	121434200	7.25	ENSSSCT00000010011	.	intron	.	444518 rs7881	2
AAC.GTT	8	121637989	121638011	7.66667	.	.	intergenic	0.002	rs787441065	3
AAAAC.GTTTT	8	121782039	121782057	3.8	.	.	intergenic	0.012	502571 rs7920	2
AAAC.GTTT	8	121957122	121957150	7.25	.	.	intergenic	.	rs789408800	2
AC.GT	8	121988548	121988558	5.5	.	.	intergenic	-0.032	rs793128255	2
AAAAC.GTTTT	8	122207416	122207443	5.6	.	.	intergenic	0.128	rs791222352	2
AAC.GTT	8	122312364	122312376	4.33333	.	.	intergenic	0.202	rs787719870	2
AC.GT	8	122318003	122318014	6	ENSSSCT00000010022	SGMS2	3'utr	-0.038	.	4
AT.AT	8	122552445	122552454	5	.	.	intergenic	-0.043	.	3
ACAT.ATGT	8	122560314	122560335	5.5	.	.	intergenic	-0.107	.	2
AC.GT	8	122570514	122570540	13.5	.	.	intergenic	.	rs690951549	2
AG.CT	8	122665874	122665885	6	.	.	intergenic	0.02	.	4
AAT.ATT	8	122872638	122872651	4.66667	.	.	intergenic	.	rs788426830	2
AAAGG.CCTTT	8	122876328	122876352	5	.	.	intergenic	.	907228 rs7897	2
AT.AT	8	122950347	122950358	6	.	.	intergenic	.	rs789594708	2
AG.CT	8	123029424	123029433	5	.	.	intergenic	-0.007	323753 rs7928	2
AC.GT	8	123219759	123219783	12.5	.	.	intergenic	0.449	rs791708142	2
AAAC.GTTT	8	123263287	123263305	4.75	.	.	intergenic	-0.091	rs786213351	2
AAAC.GTTT	8	123291652	123291670	4.75	.	.	intergenic	0.152	397161 rs7881	2
AAAAC.GTTTT	8	123343616	123343634	3.8	.	.	intergenic	.	rs790067518	3
AAAAT.ATTTT	8	123347577	123347595	3.8	.	.	intergenic	1.074	rs792545743	2
AAAC.GTTT	8	123475093	123475111	4.75	.	.	intergenic	0.141	rs787481693	2
AT.AT	8	123528400	123528442	21.5	.	.	intergenic	0.102	.	4
AAAC.GTTT	8	123551452	123551474	5.75	.	.	intergenic	-0.258	rs788928491	2
AG.CT	8	124052203	124052223	10.5	ENSSSCT00000031175	.	intron	.	rs789490245	4
AAAT.ATTT	8	124070011	124070022	3	.	.	intergenic	0.266	577350 rs7884	2
ATC.GAT	8	124120722	124120740	6.33333	.	.	intergenic	-0.28	rs787939190	3
AAAAT.ATTTT	8	124124156	124124172	3.4	.	.	intergenic	0.225	rs792455631	2
AATG.CATT	8	124217764	124217782	4.75	ENSSSCT00000030690	.	intron	.	rs791886914	2
AAAC.GTTT	8	124345399	124345413	3.75	ENSSSCT00000010025	.	intron	0.148	554906 rs7896	2
AG.CT	8	124666422	124666434	6.5	ENSSSCT00000010027	.	intron	-0.1	921245 rs7907	2
AG.CT	8	124730194	124730208	7.5	.	.	intergenic	0.38	077409 rs7921	3
AAAC.GTTT	8	124837581	124837604	6	ENSSSCT00000030394	PPA2	intron	-0.139	rs791613162	2
AC.GT	8	124903322	124903335	7	ENSSSCT00000030394	PPA2	intron	0.167	.	4
AACCC.GGGTT	8	125134892	125134911	4	ENSSSCT00000010028	TET2	intron	0.643	rs792362005	2
AAAC.GTTT	8	125280352	125280372	5.25	.	.	intergenic	-0.096	rs791018284	3
AAC.GTT	8	125503668	125503681	4.66667	.	.	intergenic	0.016	770982 rs7902	4
AAAT.ATTT	8	125846327	125846349	5.75	.	.	intergenic	.	rs702742457	2
AG.CT	8	126121715	126121725	5.5	.	.	intergenic	.	rs787024596	2
AT.AT	8	126147658	126147667	5	.	.	intergenic	.	746590 rs7882	2
AAAT.ATTT	8	126212862	126212885	6	.	.	intergenic	.	183644 rs7900	2
AC.GT	8	126246707	126246717	5.5	.	.	intergenic	-0.11	.	2
AC.GT	8	126310070	126310083	7	.	.	intergenic	0.251	560950 rs7905	2
AT.AT	8	126348520	126348531	6	.	.	intergenic	-0.062	rs793680475	2
AAT.ATT	8	126570966	126570979	4.66667	.	.	intergenic	0.068	rs792888887	2
AAAT.ATTT	8	126681476	126681492	4.25	.	.	intergenic	.	732695 rs7905	2
AAC.GTT	8	126705162	126705180	6.33333	.	.	intergenic	-0.09	rs788751601	2
AC.GT	8	126715239	126715264	13	.	.	intergenic	0.245	rs789896356	4
AC.GT	8	126718451	126718462	6	.	.	intergenic	.	rs790338014	4
AAAC.GTTT	8	126731489	126731508	5	.	.	intergenic	0.075	rs789518099	2
AAAC.GTTT	8	126734829	126734849	5.25	.	.	intergenic	0.197	702238 rs7869	2
AAC.GTT	8	127083514	127083525	4	ENSSSCT00000010034	.	intron	.	.	2
AG.CT	8	127366041	127366051	5.5	ENSSSCT00000033438	NFKB1	intron	.	rs788752996	2
AC.GT	8	127810579	127810595	8.5	ENSSSCT00000010041	SLC39A8	intron	-0.005	rs792371269	2
AAAG.CTTT	8	127815781	127815798	4.5	ENSSSCT00000010041	SLC39A8	intron	0.35	507703 rs7911	2
AC.GT	8	127838830	127838841	6	.	.	intergenic	0.027	rs697337693	2
AG.CT	8	127843084	127843100	8.5	.	.	intergenic	0.053	rs788271575	2
AC.GT	8	127897823	127897835	6.5	.	.	intergenic	.	rs786365754	3
AAAC.GTTT	8	128259871	128259900	7.5	ENSSSCT00000010043	.	intron	0.129	788974 rs7924	2
AAAAG.CTTTT	8	128275866	128275887	4.4	ENSSSCT00000010043	.	intron	0.331	rs789185882	2
AC.GT	8	128313523	128313533	5.5	ENSSSCT00000010043	.	intron	.	rs786460499	2
AG.CT	8	128524306	128524321	8	ENSSSCT00000010044	PPP3CA	intron	0.051	.	4
AAAAT.ATTTT	8	128598311	128598325	3	ENSSSCT00000010044	PPP3CA	intron	0.2	rs792197841	2
AG.CT	8	128607977	128607987	5.5	ENSSSCT00000010044	PPP3CA	intron	-0.083	.	4
AAAAAC.GTTTT	8	128638575	128638597	3.83333	ENSSSCT00000010044	PPP3CA	intron	-0.275	rs789124095	2
AAAAC.GTTTT	8	128842147	128842172	5.2	.	.	intergenic	.	rs793412063	2
AC.GT	8	128982957	128982969	6.5	.	.	intergenic	-0.044	rs787556310	4
AG.CT	8	129054173	129054185	6.5	.	.	intergenic	.	318737 rs7910	4
AAAAC.GTTTT	8	129138121	129138139	3.8	.	.	intergenic	.	rs792745686	2
AT.AT	8	129213263	129213274	6	.	.	intergenic	-0.186	rs792896547	3
AG.CT	8	129223724	129223736	6.5	.	.	intergenic	-0.069	509494 rs7911	4
AT.AT	8	129300405	129300414	5	.	.	intergenic	.	rs791111340	2
AAAC.GTTT	8	129501186	129501204	4.75	.	.	intergenic	0.098	rs791977224	2

AAAAT.ATTTT	8	129573492	129573514	4.6	.	.	intergenic	0.243	784192 rs7903	2
AAAAG.CTTTT	8	129728627	129728647	4.2	.	.	intergenic	.	rs786226896	3
AATC.GATT	8	129742305	129742325	5.25	ENSSSCT00000010051	H2AFZ	promoter	0.163	rs786565737	2
AAAG.CTTT	8	130071388	130071400	3.25	ENSSSCT00000010052	.	intron	0.128	.	2
AAAG.CTTT	8	130122592	130122618	6.75	ENSSSCT00000010053	.	intron	.	590964 rs7911	2
AG.CT	8	130425448	130425467	10	ENSSSCT00000027188	ADH4	intron	.	rs792145086	2
AG.CT	8	130425448	130425467	10	ENSSSCT00000010057	ADH4	intron	.	rs792145086	2
AAAG.CTTT	8	130481122	130481144	5.75	ENSSSCT00000010059	METAP1	intron	2.43	rs791881328	2
AC.GT	8	130598786	130598798	6.5	ENSSSCT00000032198	EIF4E	intron	0.241	rs790624688	2
AAAC.GTTT	8	130661440	130661481	10.5	.	.	intergenic	0.111	539036 rs7869	2
AAAC.GTTT	8	130713399	130713417	4.75	.	.	intergenic	.	.	2
AAAC.GTTT	8	130796855	130796875	5.25	.	.	intergenic	.	200065 rs7882	2
AT.AT	8	130811814	130811831	9	.	.	intergenic	-0.056	522110 rs7887	2
AAC.GTT	8	131119582	131119598	5.66667	.	.	intergenic	.	rs786900454	3
AC.GT	8	131229768	131229793	13	.	.	intergenic	.	rs693393275	2
AAAC.GTTT	8	131246100	131246127	7	.	.	intergenic	0.234	rs793021392	2
AAAAAC.GTTTTT	8	131319057	131319080	4	.	.	intergenic	0.396	rs789776801	2
AC.GT	8	131453819	131453829	5.5	.	.	intergenic	0.205	.	2
AAAC.GTTT	8	131545816	131545834	4.75	.	.	intergenic	0.125	357029 rs7876	2
AC.GT	8	131632835	131632847	6.5	.	.	intergenic	.	rs791754492	2
AAAAC.GTTTT	8	131677428	131677448	4.2	.	.	intergenic	0.345	562512 rs7894	2
AT.AT	8	131782234	131782243	5	.	.	intergenic	0.073	rs791079084	2
AT.AT	8	131868194	131868217	12	.	.	intergenic	0.388	008596 rs7879	2
AAAAAC.GTTTTT	8	131874434	131874459	4.33333	.	.	intergenic	-0.214	rs788152396	2
AAAT.ATTT	8	132070588	132070602	3.75	.	.	intergenic	.	952424 rs7879	2
AG.CT	8	132110619	132110641	11.5	.	.	intergenic	-0.045	353624 rs7043	2
AT.AT	8	132432111	132432121	5.5	.	.	intergenic	0.004	.	3
AG.CT	8	132446599	132446609	5.5	.	.	intergenic	-0.023	.	3
AC.GT	8	132450737	132450756	10	.	.	intergenic	0.074	rs792078813	2
AC.GT	8	132456993	132457031	19.5	.	.	intergenic	-0.11	rs789089957	4
AG.CT	8	132470931	132470944	7	.	.	intergenic	0.039	rs792143943	2
AG.CT	8	132500016	132500025	5	.	.	intergenic	-0.045	.	2
AAAAAG.CTTTTT	8	132617127	132617155	4.83333	.	.	intergenic	-0.131	rs791688841	2
AAAAAT.ATTTTT	8	132688251	132688279	4.83333	.	.	intergenic	-0.128	rs789382846	2
AT.AT	8	132714990	132714999	5	.	.	intergenic	0.281	rs791751981	2
AAAAC.GTTTT	8	132760578	132760601	4.8	.	.	intergenic	0.149	rs791804922	2
AAAAAC.GTTTTT	8	132764073	132764100	4.66667	.	.	intergenic	0.091	172418 rs7869	2
AC.GT	8	132792621	132792634	7	.	.	intergenic	-0.064	rs787898179	2
AAC.GTT	8	132962596	132962619	8	.	.	intergenic	0.167	rs792150334	2
AG.CT	8	133076000	133076009	5	.	.	intergenic	-0.235	rs787275305	2
AC.GT	8	133162502	133162525	12	.	.	intergenic	-0.1	rs790660216	2
AAAAC.GTTTT	8	133354566	133354584	3.8	.	.	intergenic	0.753	rs788876985	2
AG.CT	8	133391378	133391388	5.5	.	.	intergenic	0.156	rs786740838	3
AAAT.ATTT	8	133441078	133441100	5.75	.	.	intergenic	0.02	rs786254344	2
AC.GT	8	133584492	133584504	6.5	.	.	intergenic	-0.034	rs789863045	3
AAATT.AATTT	8	133691017	133691031	3	.	.	intergenic	0.054	rs790101000	2
AAAC.GTTT	8	133758675	133758693	4.75	ENSSSCT00000028459	BMPR1B	intron	.	rs793860508	2
AAC.GTT	8	133903189	133903205	5.66667	ENSSSCT00000034827	BMPR1B	intron	0.145	310831 rs7884	2
AC.GT	8	133931299	133931311	6.5	ENSSSCT00000034827	BMPR1B	intron	0.213	rs788421242	2
AAAC.GTTT	8	133939986	133940000	3.75	ENSSSCT00000034827	BMPR1B	intron	0.044	101528 rs7871	2
AC.GT	8	133954187	133954196	5	ENSSSCT00000034827	BMPR1B	intron	.	rs792437659	3
AAAAC.GTTTT	8	134018015	134018034	4	ENSSSCT00000034827	BMPR1B	intron	.	426428 rs7913	2
AT.AT	8	134067146	134067157	6	.	.	intergenic	.	rs790106764	2
AT.AT	8	134207714	134207728	7.5	.	.	intergenic	-0.092	.	2
AATC.GATT	8	134217312	134217336	6.25	.	.	intergenic	.	rs790442598	2
AC.GT	8	134247603	134247621	9.5	.	.	intergenic	0.085	.	2
AAAG.CTTT	8	134276075	134276094	5	.	.	intergenic	0.227	rs790543115	2
AAT.ATT	8	134292660	134292676	5.66667	.	.	intergenic	0.133	rs787929999	2
AG.CT	8	134303322	134303334	6.5	.	.	intergenic	0.224	.	2
AT.AT	8	134538343	134538357	7.5	ENSSSCT00000010065	PDLIM5	intron	0.239	.	2
AG.CT	8	134620761	134620773	6.5	.	.	intergenic	0.111	582613 rs7917	2
AAAAT.ATTTT	8	134630687	134630716	6	.	.	intergenic	-0.002	379802 rs7875	2
AAAAC.GTTTT	8	134818232	134818288	11.4	.	.	intergenic	.	rs790587863	2
AT.AT	8	134890000	134890019	10	.	.	intergenic	.	rs792751099	2
AAAC.GTTT	8	134947581	134947600	5	.	.	intergenic	.	321052 rs7872	2
AAACC.GGTTT	8	134973938	134973966	5.8	.	.	intergenic	.	rs789009715	2
AGATG.CATCT	8	135091352	135091373	4.4	.	.	intergenic	0.101	710285 rs7870	2
AAAT.ATTT	8	135117191	135117212	5.5	.	.	intergenic	.	551254 rs7924	2
AAC.GTT	8	135335551	135335564	4.66667	.	.	intergenic	-0.288	rs789754043	2
AAC.GTT	8	135360267	135360282	5.33333	.	.	intergenic	0.157	rs790472910	2
AAC.GTT	8	135448936	135448955	6.66667	ENSSSCT00000026735	.	intron	0.152	rs790778411	2
AAAC.GTTT	8	135494828	135494842	3.75	ENSSSCT00000026735	.	intron	0.13	rs791663669	2
ACC.GGT	8	135506899	135506912	4.66667	ENSSSCT00000026735	.	intron	-0.158	rs789642818	2
AAAC.GTTT	8	135523424	135523449	6.5	ENSSSCT00000026735	.	intron	.	rs792473506	2
AAAAC.GTTTT	8	135526749	135526768	4	ENSSSCT00000026735	.	intron	0.452	318035 rs7890	2
AAAT.ATTT	8	135620968	135620984	4.25	.	.	intergenic	0.25	rs787594597	3
AAAT.ATTT	8	135639602	135639623	5.5	.	.	intergenic	-0.113	rs788492847	2
AAAT.ATTT	8	135732106	135732119	3.5	.	.	intergenic	-0.005	rs787347740	2
AAAT.ATTT	8	136000258	136000271	3.5	.	.	intergenic	-0.062	327060 rs7867	2
AAAC.GTTT	8	136079092	136079107	4	.	.	intergenic	.	rs790191226	2
ACAT.ATGT	8	136143443	136143461	4.75	.	.	intergenic	-0.128	rs788406326	2
AC.GT	8	136145749	136145761	6.5	.	.	intergenic	.	.	2
AAAT.ATTT	8	136232640	136232663	6	.	.	intergenic	.	.	2
AT.AT	8	136247433	136247443	5.5	.	.	intergenic	-0.02	.	2

AAG.CTT	8	136326450	136326464	5	.	.	intergenic	0.013	736773 rs7867	2
AAAC.GTTT	8	136401759	136401779	5.25	.	.	intergenic	0.389	rs789874095	2
AT.AT	8	136880133	136880143	5.5	.	.	intergenic	.	761360 rs7886	2
AAAG.CTTT	8	136987542	136987555	3.5	.	.	intergenic	.	530422 rs7059	2
AAAT.ATTT	8	137040327	137040341	3.75	.	.	intergenic	-0.052	272497 rs7890	2
AAAC.GTTT	8	137278190	137278208	4.75	.	.	intergenic	0.447	519299 rs7899	2
AC.GT	8	137450795	137450811	8.5	.	.	intergenic	-0.107	.	2
ATC.GAT	8	137468176	137468193	6	.	.	intergenic	.	rs787774716	2
AAAAC.GTTTT	8	137479958	137479980	4.6	.	.	intergenic	-0.017	515499 rs7899	2
AC.GT	8	137480758	137480768	5.5	.	.	intergenic	0.027	.	2
AAAT.ATTT	8	137666567	137666593	6.75	ENSSSCT00000031781	.	intron	-0.111	rs790156220	2
AC.GT	8	137667262	137667272	5.5	ENSSSCT00000031781	.	intron	0.6	rs788353302	3
AAAC.GTTT	8	137723194	137723212	4.75	ENSSSCT00000031781	.	intron	0.075	rs696068555	2
AG.CT	8	137759491	137759504	7	.	.	intergenic	0.218	190472 rs7927	2
AC.GT	8	137840938	137840949	6	.	.	intergenic	0.106	rs792867535	4
AAAC.GTTT	8	137856724	137856748	6.25	.	.	intergenic	0.448	700994 rs7886	2
AAAT.ATTT	8	138227573	138227594	5.5	.	.	intergenic	.	rs791692403	2
AAAC.GTTT	8	138263949	138263966	4.5	.	.	intergenic	-0.129	584602 rs7893	2
ACAG.CTGT	8	138528956	138528970	3.75	ENSSSCT00000010075	MMRN1	intron	-0.011	302477 rs7897	2
AAT.ATT	8	138688062	138688077	5.33333	ENSSSCT00000010077	SNCA	intron	.	511642 rs7926	2
AG.CT	8	138862754	138862765	6	.	.	intergenic	0.046	.	4
AAAAT.ATTTT	8	138926985	138927001	3.4	.	.	intergenic	0.032	339728 rs7869	2
AAAGG.CCTTT	8	139039117	139039133	3.4	.	.	intergenic	0.084	rs790292379	3
AAAAC.GTTTT	8	139070763	139070797	7	ENSSSCT00000010079	.	promoter	0.209	rs786915821	2
AAAAC.GTTTT	8	139091756	139091795	8	.	.	intergenic	.	rs787720917	2
AAAC.GTTT	8	139097517	139097538	5.5	.	.	intergenic	0.083	rs787445639	2
AG.CT	8	139299019	139299028	5	.	.	intergenic	0.115	.	2
AC.GT	8	139322497	139322508	6	.	.	intergenic	0.024	101563 rs7904	3
AC.GT	8	139488712	139488724	6.5	.	.	intergenic	-0.002	226237 rs7896	2
AC.GT	8	139494901	139494919	9.5	.	.	intergenic	0.169	rs793765403	3
AAT.ATT	8	139551433	139551446	4.66667	.	.	intergenic	0.054	541429 rs7864	2
AC.GT	8	139590204	139590215	6	.	.	intergenic	.	904352 rs7923	2
AAAAC.GTTTT	8	139591565	139591592	5.6	.	.	intergenic	0.05	929806 rs7862	3
AAT.ATT	8	139701408	139701430	7.66667	ENSSSCT00000022397	.	intron	0.075	557547 rs7904	2
AAAC.GTTT	8	139851574	139851586	3.25	ENSSSCT00000010082	HERC3	intron	.	932493 rs7892	2
AAC.GTT	8	139900485	139900498	4.66667	.	.	intergenic	0.212	rs790109048	2
AAC.GTT	8	140100877	140100903	9	ENSSSCT00000036615	ABCG2	promoter	.	575612 rs7865	2
AAC.GTT	8	140100877	140100903	9	ENSSSCT00000036669	ABCG2	promoter	.	575612 rs7865	2
AAG.CTT	8	140305320	140305333	4.66667	.	.	intergenic	0.129	rs786771185	2
AACC.GGTT	8	140377642	140377653	3	.	.	intergenic	0.324	rs791114600	2
AG.CT	8	140537628	140537639	6	ENSSSCT00000010099	DSPP	intron	-0.024	rs790583342	2
AG.CT	8	140547591	140547602	6	.	.	intergenic	.	.	2
AAAC.GTTT	8	140607149	140607170	5.5	ENSSSCT00000010101	SPARCL1	intron	0.231	.	2
AAC.GTT	8	140768798	140768825	9.33333	ENSSSCT00000010104	HSD17B13	intron	0.491	rs792080054	2
AAAT.ATTT	8	141247647	141247665	4.75	.	.	intergenic	.	.	2
AAAT.ATTT	8	141461072	141461097	6.5	ENSSSCT00000029989	PTPN13	intron	0.221	rs793781640	2
AC.GT	8	141942458	141942467	5	.	.	intergenic	-0.014	rs788147165	3
AAC.GTT	8	142076573	142076597	8.33333	ENSSSCT00000036195	MAPK10	intron	0.037	rs789549521	4
AAC.GTT	8	142076573	142076597	8.33333	ENSSSCT00000033080	MAPK10	intron	0.037	rs789549521	4
AAC.GTT	8	142076573	142076597	8.33333	ENSSSCT00000010107	MAPK10	intron	0.037	rs789549521	4
AAGG.CCTT	8	142167628	142167644	4.25	ENSSSCT00000010108	ARHGAP24	intron	-0.068	rs787763054	2
AC.GT	8	142235119	142235131	6.5	ENSSSCT00000010108	ARHGAP24	intron	-0.009	575468 rs7925	2
AAAT.ATTT	8	142476329	142476346	4.5	ENSSSCT00000010108	ARHGAP24	intron	.	rs787028153	3
AT.AT	8	142836952	142836961	5	.	.	intergenic	.	rs791855715	2
AAAAT.ATTTT	8	142846620	142846638	3.8	.	.	intergenic	0.158	rs786365970	2
AAG.CTT	8	143117909	143117924	5.33333	.	.	intergenic	.	.	3
AAAT.ATTT	8	143173278	143173298	5.25	.	.	intergenic	0.286	.	2
AAC.GTT	8	143428066	143428083	6	.	.	intergenic	.	rs788495553	2
AC.GT	8	143498788	143498806	9.5	ENSSSCT00000010110	CDS1	intron	0.059	rs789107945	2
AATG.CATT	8	143723466	143723491	6.5	.	.	intergenic	0.25	rs790159325	2
AAAC.GTTT	8	143856601	143856619	4.75	.	.	intergenic	0.555	922500 rs7885	2
AAAAC.GTTTT	8	144045929	144045952	4.8	.	.	intergenic	0.094	rs793297366	2
AAT.ATT	8	144051603	144051618	5.33333	.	.	intergenic	0.015	rs790785691	2
AC.GT	8	144098427	144098437	5.5	.	.	intergenic	0.647	.	2
AAC.GTT	8	144144579	144144592	4.66667	.	.	intergenic	.	541935 rs7889	2
AAAT.ATTT	8	144182011	144182024	3.5	.	.	intergenic	0.111	rs704868975	2
AT.AT	8	144591216	144591227	6	ENSSSCT00000010121	COPS4	intron	0.321	774307 rs7908	2
ACAT.ATGT	8	144777080	144777093	3.5	ENSSSCT00000010124	SEC31A	intron	0.25	rs793165680	2
AG.CT	8	145011346	145011358	6.5	.	.	intergenic	0.065	rs791082126	2
AAAC.GTTT	8	145099808	145099830	5.75	.	.	intergenic	.	903142 rs7883	2
AAT.ATT	8	145142060	145142073	4.66667	.	.	intergenic	0.057	.	2
AC.GT	8	145182291	145182303	6.5	.	.	intergenic	.	781747 rs7899	2
AAAAT.ATTTT	8	145482655	145482675	4.2	.	.	intergenic	0.088	rs788453808	2
AAAC.GTTT	8	145484539	145484562	6	.	.	intergenic	0.531	949092 rs7891	2
AT.AT	8	145794180	145794199	10	.	.	intergenic	0.189	361014 rs7926	2
AG.CT	8	145928478	145928491	7	.	.	intergenic	1.837	.	2
AAAC.GTTT	8	145990266	145990284	4.75	.	.	intergenic	0.13	rs787390905	2
AC.GT	8	146396283	146396299	8.5	.	.	intergenic	.	rs786988531	4
AAC.GTT	8	146426484	146426496	4.33333	.	.	intergenic	-0.16	.	2
AAT.ATT	8	146732490	146732510	7	.	.	intergenic	0.002	rs789683448	3
AT.AT	8	146817392	146817406	7.5	ENSSSCT00000022547	PRDM8	intron	.	324627 rs7896	2
AT.AT	8	146817392	146817406	7.5	ENSSSCT00000010135	PRDM8	intron	.	324627 rs7896	2
AAAT.ATTT	8	147023103	147023133	7.75	.	.	intergenic	0.113	074334 rs7906	2
AC.GT	8	147306470	147306486	8.5	.	.	intergenic	0.153	rs787242962	2

AC.GT	8	147371100	147371113	7	.	.	intergenic	0.034	rs791028159	5
AT.AT	8	147395068	147395078	5.5	.	.	intergenic	.	642026 rs7878	2
AAC.GTT	8	147486895	147486914	6.66667	.	.	intergenic	.	465691 rs7870	2
AAAC.GTTT	8	147682821	147682845	6.25	.	.	intergenic	0.216	917046 rs7870	2
AT.AT	8	147787496	147787514	9.5	.	.	intergenic	0.138	.	4
AAAC.GTTT	8	147957689	147957704	4	.	.	intergenic	0.266	rs788337022	2
AAAC.GTTT	8	148213382	148213400	4.75	ENSSSCT00000010143	CSN1S1	intron	-0.07	rs790219149	2
AAAAC.GTTTT	9	300698	300716	3.8	.	.	intergenic	0.32	rs791291749	2
AAAAG.CTTTT	9	2317987	2318002	3.2	ENSSSCT00000032189	OVCH2	intron	0.135	rs791651133	2
AC.GT	9	2547460	2547473	7	.	.	intergenic	-0.059	458489 rs7866	2
AAAC.GTTT	9	2740055	2740073	4.75	ENSSSCT00000015949	SYT9	intron	.	rs791337645	2
AC.GT	9	2809285	2809301	8.5	ENSSSCT00000015949	SYT9	intron	0.282	620079 rs6961	7
AAAT.ATTT	9	2914966	2914980	3.75	.	.	intergenic	0.13	rs791215351	2
AT.AT	9	2923026	2923041	8	.	.	intergenic	.	rs789350881	3
AC.GT	9	2933906	2933932	13.5	.	.	intergenic	0.312	033295 rs7871	2
AAAT.ATTT	9	2954688	2954701	3.5	.	.	intergenic	.	.	2
AAAC.GTTT	9	3043984	3044001	4.5	.	.	intergenic	0.376	286201 rs7866	2
AAC.GTT	9	3348271	3348282	4	.	.	intergenic	.	592543 rs7922	2
AC.GT	9	3453243	3453253	5.5	.	.	intergenic	.	.	2
AC.GT	9	4286814	4286823	5	.	.	intergenic	.	.	3
AAAC.GTTT	9	5344642	5344656	3.75	.	.	intergenic	.	rs790277652	4
AT.AT	9	5657795	5657805	5.5	.	.	intergenic	0.007	rs789974413	3
AAAAC.GTTTT	9	5674091	5674113	4.6	.	.	intergenic	-0.099	517684 rs7892	2
AATT.AATT	9	5921161	5921179	4.75	.	.	intergenic	.	.	2
AT.AT	9	5972417	5972427	5.5	.	.	intergenic	-0.009	.	5
ACAT.ATGT	9	6042573	6042595	5.75	.	.	intergenic	-0.074	767949 rs7904	2
AC.GT	9	6141694	6141706	6.5	ENSSSCT00000016119	TRIM68	intron	0.391	331730 rs7876	2
AAAC.GTTT	9	6249758	6249774	4.25	.	.	intergenic	0.049	rs791993539	2
AT.AT	9	6561161	6561174	7	.	.	intergenic	-0.519	rs786726445	2
AC.GT	9	6575896	6575914	9.5	.	.	intergenic	.	.	2
AG.CT	9	6795076	6795089	7	.	.	intergenic	.	rs694452350	2
AC.GT	9	6818212	6818221	5	ENSSSCT00000016142	.	3'utr	0.207	rs788449837	3
AAAAT.ATTTT	9	6915060	6915081	4.4	ENSSSCT00000016143	STIM1	intron	0.481	rs787365479	2
AC.GT	9	7053879	7053890	6	ENSSSCT00000016143	STIM1	intron	0.243	rs793816607	2
AC.GT	9	7065708	7065722	7.5	.	.	intergenic	0.284	rs793148982	2
AC.GT	9	7124760	7124770	5.5	ENSSSCT00000016145	NUP98	intron	.	rs791067197	2
AT.AT	9	7144142	7144152	5.5	ENSSSCT00000016145	NUP98	intron	.	rs788731116	3
AAAGC.GCTTT	9	7153308	7153323	3.2	ENSSSCT00000016145	NUP98	intron	-0.047	rs789190428	2
AAAT.ATTT	9	7157918	7157938	5.25	ENSSSCT00000016145	NUP98	intron	0.3	rs791994375	2
AAAAC.GTTTT	9	7269515	7269540	5.2	.	.	intergenic	.	rs792111651	3
AAGG.CCTT	9	8085892	8085922	7.75	ENSSSCT00000016169	STARD10	intron	0.164	rs791761170	2
AAAC.GTTT	9	8264503	8264514	3	ENSSSCT00000016171	FCHSD2	intron	0.345	rs787321263	2
AAAC.GTTT	9	8264503	8264514	3	ENSSSCT00000016172	FCHSD2	intron	0.345	rs787321263	2
ACC.GGT	9	8308020	8308036	5.66667	ENSSSCT00000016171	FCHSD2	intron	.	rs790120607	2
ACC.GGT	9	8308020	8308036	5.66667	ENSSSCT00000016172	FCHSD2	intron	.	rs790120607	2
ACC.GGT	9	8308020	8308036	5.66667	ENSSSCT00000020569	U6	promoter	.	rs790120607	2
AT.AT	9	8655157	8655168	6	ENSSSCT00000016174	.	intron	0.196	rs789342938	2
AC.GT	9	8743881	8743891	5.5	.	.	intergenic	0.708	.	2
AAAC.GTTT	9	8774688	8774710	5.75	.	.	intergenic	0.382	rs787227863	2
AAAAC.GTTTT	9	8813701	8813716	3.2	.	.	intergenic	0.262	rs787154286	2
AC.GT	9	9129489	9129505	8.5	.	.	intergenic	0.129	rs710761134	4
AC.GT	9	9156131	9156143	6.5	ENSSSCT00000016185	UCP2	promoter	.	580769 rs7912	4
AAC.GTT	9	9514098	9514109	4	.	.	intergenic	.	rs787153031	2
AAAC.GTTT	9	9607579	9607597	4.75	.	.	intergenic	.	rs787677659	2
AAAAT.ATTTT	9	9641780	9641803	4.8	.	.	intergenic	.	234679 rs7891	2
AC.GT	9	10777516	10777525	5	.	.	intergenic	-0.358	rs789445648	2
AC.GT	9	11086909	11086922	7	ENSSSCT00000016213	MAP6	intron	0.097	.	4
AAC.GTT	9	11379554	11379567	4.66667	ENSSSCT00000025652	UVRAG	intron	-0.104	rs789732243	4
AAAGC.GCTTT	9	11411967	11411992	5.2	ENSSSCT00000025652	UVRAG	intron	.	rs788546334	2
AG.CT	9	11656368	11656382	7.5	ENSSSCT00000016218	PRKRIR	intron	0.142	rs786947500	2
AAAAAC.GTTTTT	9	11845595	11845613	3.16667	.	.	intergenic	.	393696 rs7925	2
AC.GT	9	12248858	12248872	7.5	ENSSSCT00000023312	.	intron	.	.	2
AGAT.ATCT	9	12382919	12382951	8.25	ENSSSCT00000016229	MYO7A	intron	0.046	rs791257542	2
AAAAAT.ATTTTT	9	12624520	12624543	4	ENSSSCT00000016230	.	intron	.	rs787307793	2
AAT.ATT	9	12731506	12731533	9.33333	ENSSSCT00000016231	.	intron	.	rs787882344	2
AT.AT	9	12848247	12848256	5	.	.	intergenic	.	rs691967153	2
AAAC.GTTT	9	12963737	12963762	6.5	.	.	intergenic	.	rs792029980	2
AAC.GTT	9	13394131	13394145	5	.	.	intergenic	0.127	.	2
ACT.AGT	9	13846859	13846872	4.66667	.	.	intergenic	0.217	.	2
AAAG.CTTT	9	14039798	14039824	6.75	ENSSSCT00000016246	GAB2	intron	.	rs787535301	2
AC.GT	9	14074989	14075000	6	ENSSSCT00000016246	GAB2	intron	0.14	rs792638897	3
AAC.GTT	9	14106368	14106381	4.66667	ENSSSCT00000016246	GAB2	intron	.	975109 rs7880	2
AT.AT	9	14109433	14109461	14.5	ENSSSCT00000016246	GAB2	intron	.	503613 rs7892	2
AT.AT	9	14167652	14167662	5.5	ENSSSCT00000016248	NARS2	intron	.	rs792842559	2
AC.GT	9	14462973	14462986	7	ENSSSCT00000016249	.	intron	0.093	rs790457535	2
AAAC.GTTT	9	14476013	14476027	3.75	ENSSSCT00000016249	.	intron	.	738849 rs7908	2
AC.GT	9	14910807	14910817	5.5	.	.	intergenic	0.094	rs708830826	2
AG.CT	9	15054050	15054063	7	.	.	intergenic	0.347	137703 rs7900	2
AG.CT	9	15367511	15367524	7	.	.	intergenic	-0.001	562535 rs7903	2
AAAC.GTTT	9	15372465	15372479	3.75	.	.	intergenic	-0.097	.	2
AC.GT	9	15645702	15645722	10.5	.	.	intergenic	-0.1	rs793264184	2
AAAG.CTTT	9	15679706	15679719	3.5	.	.	intergenic	-0.104	.	2
AAC.GTT	9	15897197	15897213	5.66667	.	.	intergenic	.	526474 rs7877	4
AC.GT	9	15942921	15942941	10.5	.	.	intergenic	-0.046	rs789973187	3

AAC.GTT	9	16100769	16100790	7.33333	.	.	intergenic	-0.145	rs791597400	2
AAAAC.GTTTT	9	16179071	16179094	4.8	.	.	intergenic	0.574	rs788872213	2
AAAAT.ATTTT	9	16414481	16414500	4	.	.	intergenic	.	rs791366873	2
AAAC.GTTT	9	16519206	16519233	7	.	.	intergenic	0.323	560124 rs6907	2
AAAC.GTTT	9	16581117	16581140	6	.	.	intergenic	0.514	rs793321828	2
AAAC.GTTT	9	16774642	16774654	3.25	.	.	intergenic	-0.005	553973 rs7927	2
AAAC.GTTT	9	16841095	16841121	6.75	.	.	intergenic	0.031	rs790897883	2
AATT.AATT	9	16871993	16872009	4.25	.	.	intergenic	-0.114	rs791299309	2
AT.AT	9	16891732	16891743	6	.	.	intergenic	-0.148	rs793745863	3
AG.CT	9	16965862	16965887	13	.	.	intergenic	-0.343	rs789033905	4
AAAT.ATTT	9	16977593	16977611	4.75	.	.	intergenic	.	.	3
AAAAT.ATTTT	9	17041154	17041184	6.2	.	.	intergenic	.	526955 rs7922	2
AC.GT	9	17197722	17197740	9.5	.	.	intergenic	-0.078	130228 rs7936	2
AAAC.GTTT	9	17224074	17224097	6	.	.	intergenic	0.137	rs788934179	2
AC.GT	9	17233545	17233572	14	.	.	intergenic	-0.077	rs789321240	2
AT.AT	9	17254194	17254204	5.5	.	.	intergenic	-0.105	968790 rs7892	2
AAAAC.GTTTT	9	17317134	17317151	3.6	.	.	intergenic	.	rs788861149	2
AC.GT	9	17345180	17345191	6	.	.	intergenic	.	066574 rs7937	2
AAAAAC.GTTTT	9	17420122	17420143	3.66667	.	.	intergenic	.	rs791810169	2
AAG.CTT	9	17442721	17442748	9.33333	.	.	intergenic	1.001	rs791360309	2
AAT.ATT	9	17453106	17453125	6.66667	.	.	intergenic	0.731	rs792876857	2
AT.AT	9	17760784	17760794	5.5	.	.	intergenic	0.058	rs787117180	3
AC.GT	9	17800933	17800964	16	.	.	intergenic	0.085	rs789412245	2
AT.AT	9	17912458	17912471	7	.	.	intergenic	.	rs792196535	2
AAT.ATT	9	17974204	17974215	4	.	.	intergenic	0.106	.	2
AT.AT	9	18053116	18053131	8	.	.	intergenic	0.013	.	2
AGAT.ATCT	9	18071795	18071806	3	.	.	intergenic	0.08	rs787075615	2
AAT.ATT	9	18086626	18086655	10	.	.	intergenic	0.101	774988 rs7902	2
ACAG.CTGT	9	18535514	18535527	3.5	.	.	intergenic	0.078	rs786603253	3
AC.GT	9	18652858	18652867	5	.	.	intergenic	0.081	.	2
AAAT.ATTT	9	18794021	18794043	5.75	.	.	intergenic	.	rs793788192	2
AG.CT	9	18891430	18891440	5.5	ENSSSCT00000016253	C11orf82	intron	0.223	513607 rs7900	2
AC.GT	9	19055311	19055325	7.5	.	.	intergenic	0.202	130434 rs7902	2
AAATAT.ATATT	9	19173757	19173775	3.16667	.	.	intergenic	0.446	rs789925290	2
AC.GT	9	19352645	19352655	5.5	ENSSSCT00000016258	CCDC90B	intron	.	.	2
AAT.ATT	9	19353649	19353663	5	ENSSSCT00000016258	CCDC90B	intron	.	rs787176367	2
AAAT.ATTT	9	19987927	19987947	5.25	.	.	intergenic	.	rs792095226	2
AC.GT	9	20004158	20004177	10	.	.	intergenic	0.083	rs788213430	3
AC.GT	9	20385667	20385676	5	.	.	intergenic	0.056	.	3
AAT.ATT	9	20497885	20497896	4	.	.	intergenic	0.014	rs787111691	2
AT.AT	9	20578802	20578814	6.5	.	.	intergenic	.	369630 rs7885	2
AC.GT	9	20598449	20598464	8	.	.	intergenic	.	.	3
AGG.CCT	9	20616601	20616619	6.33333	.	.	intergenic	0.041	rs791435816	2
AT.AT	9	20790986	20790995	5	.	.	intergenic	.	rs787576344	2
AAAG.CTTT	9	20794357	20794370	3.5	.	.	intergenic	-0.053	.	2
AG.CT	9	20957929	20957940	6	.	.	intergenic	-0.013	.	2
AAAC.GTTT	9	20972178	20972196	4.75	.	.	intergenic	0.304	rs789229351	2
AG.CT	9	21062424	21062434	5.5	.	.	intergenic	0.627	.	4
AC.GT	9	21311578	21311588	5.5	.	.	intergenic	0.272	rs786683405	2
AG.CT	9	21554830	21554839	5	.	.	intergenic	.	.	2
AAAC.GTTT	9	21811940	21811953	3.5	.	.	intergenic	0.387	098396 rs7875	2
AAC.GTT	9	21879793	21879807	5	ENSSSCT00000016264	CCDC89	promoter	0.192	rs792012926	2
AAAC.GTTT	9	21975380	21975406	6.75	ENSSSCT00000016265	SYTL2	intron	-0.01	rs787418326	2
AAC.GTT	9	22117538	22117554	5.66667	ENSSSCT00000016269	.	intron	0.6	.	2
AT.AT	9	22204434	22204443	5	.	.	intergenic	0.027	.	2
AAAC.GTTT	9	22314175	22314200	6.5	.	.	intergenic	0.114	rs786210390	2
AAAT.ATTT	9	22358142	22358163	5.5	ENSSSCT00000030954	.	intron	0.369	rs788355596	2
AATG.CATT	9	22708034	22708054	5.25	.	.	intergenic	0.071	rs791267428	2
AC.GT	9	23121517	23121529	6.5	ENSSSCT00000028393	.	intron	.	rs789296000	2
AC.GT	9	23315753	23315773	10.5	.	.	intergenic	0.336	090084 rs7898	2
AAAT.ATTT	9	23584999	23585025	6.75	.	.	intergenic	.	rs789112954	2
AAAAG.CTTTT	9	23829317	23829341	5	ENSSSCT00000016279	RAB38	intron	0.072	334696 rs7863	2
AGC.GCT	9	23924122	23924138	5.66667	.	.	intergenic	-0.009	303036 rs7902	2
AAAC.GTTT	9	24194356	24194378	5.75	.	.	intergenic	-0.002	rs793871109	2
AG.CT	9	24332048	24332062	7.5	ENSSSCT00000027396	.	intron	0.049	rs793727508	2
AAAAC.GTTTT	9	24470844	24470873	6	ENSSSCT00000016281	.	intron	.	994195 rs7889	2
AAAT.ATTT	9	24494387	24494402	4	ENSSSCT00000016281	.	intron	.	112578 rs7937	2
AT.AT	9	24856785	24856806	11	.	.	intergenic	0.97	988239 rs7868	2
AAT.ATT	9	25242353	25242367	5	ENSSSCT00000016284	NOX4	intron	.	rs789364117	2
AT.AT	9	25508087	25508098	6	.	.	intergenic	0.139	rs788043567	3
AAT.ATT	9	25684852	25684871	6.66667	ENSSSCT00000024828	FOLH1	intron	-0.178	rs792162580	2
AAC.GTT	9	25967453	25967469	5.66667	.	.	intergenic	1.391	rs787361056	2
AAAAAC.GTTTT	9	26013910	26013932	3.83333	.	.	intergenic	.	rs793144136	2
AAAC.GTTT	9	26037758	26037781	6	.	.	intergenic	.	381703 rs7920	3
AAAC.GTTT	9	26074035	26074056	5.5	.	.	intergenic	0.243	573725 rs7922	2
AAG.CTT	9	26139168	26139190	7.66667	.	.	intergenic	0.142	rs791226822	2
AG.CT	9	26144544	26144555	6	.	.	intergenic	.	rs791723340	2
AAAC.GTTT	9	26179547	26179563	4.25	.	.	intergenic	0.14	160744 rs7874	2
AAGAG.CTCTT	9	26210481	26210510	6	.	.	intergenic	0.021	300759 rs7879	2
AC.GT	9	26449223	26449257	17.5	.	.	intergenic	-0.071	411179 rs7005	2
AAT.ATT	9	26917237	26917250	4.66667	.	.	intergenic	0.033	710599 rs7924	2
AC.GT	9	26954556	26954566	5.5	.	.	intergenic	.	.	2
AT.AT	9	26960612	26960623	6	.	.	intergenic	-0.172	rs793287660	3
AT.AT	9	27446775	27446784	5	.	.	intergenic	0.127	rs787330328	2

AAAT.ATTT	9	27600543	27600564	5.5	.	.	intergenic	0.348	rs788232719	4
AAAC.GTTT	9	28081753	28081777	6.25	.	.	intergenic	0.194	387032 rs7876	2
AAAG.CTTT	9	28126273	28126289	4.25	.	.	intergenic	-0.037	rs791504563	2
AAAT.ATTT	9	28173043	28173059	4.25	ENSSSCT00000016292	FAT3	intron	.	.	2
AT.AT	9	28231737	28231769	16.5	ENSSSCT00000016292	FAT3	intron	0.47	rs697186427	2
AG.CT	9	28287939	28287948	5	ENSSSCT00000016292	FAT3	intron	0.265	rs787201920	2
AAAG.CTTT	9	28543496	28543511	4	ENSSSCT00000016292	FAT3	intron	0.07	rs791046313	2
AC.GT	9	28589697	28589726	15	ENSSSCT00000016292	FAT3	intron	0.037	.	2
AT.AT	9	28621062	28621073	6	ENSSSCT00000016292	FAT3	intron	0.375	rs787508368	4
AAT.ATT	9	28691376	28691395	6.66667	ENSSSCT00000016292	FAT3	intron	-0.185	150614 rs7903	2
AAC.GTT	9	28835976	28835999	8	.	.	intergenic	-0.2	356697 rs7900	2
AC.GT	9	29516294	29516306	6.5	.	.	intergenic	0.281	061977 rs7934	4
AT.AT	9	29748599	29748609	5.5	ENSSSCT00000016304	KIAA1731	intron	.	rs791659121	2
AAT.ATT	9	29814619	29814642	8	ENSSSCT00000016307	C11orf54	intron	.	rs787040952	2
AG.CT	9	30573920	30573929	5	.	.	intergenic	0.139	095434 rs7932	2
AG.CT	9	30578801	30578812	6	.	.	intergenic	-0.125	447264 rs7921	2
AAAC.GTTT	9	30788859	30788880	5.5	ENSSSCT00000016322	CWC15	3'utr	-0.053	rs792682550	2
AT.AT	9	31132775	31132789	7.5	.	.	intergenic	0.064	593613 rs7864	2
AAAT.ATTT	9	31315984	31316007	6	.	.	intergenic	0.621	rs793308749	4
AAAG.CTTT	9	31326747	31326758	3	.	.	intergenic	-0.056	rs789915844	2
AAAAAT.ATTTTT	9	31418484	31418501	3	.	.	intergenic	0.11	.	2
AAAC.GTTT	9	31464012	31464030	4.75	.	.	intergenic	0.267	578310 rs7938	2
AT.AT	9	31684352	31684362	5.5	.	.	intergenic	-0.024	.	3
AAACC.GGTTT	9	31713641	31713671	6.2	ENSSSCT00000016328	FAM76B	intron	0.046	rs793452394	2
AC.GT	9	31733711	31733725	7.5	.	.	intergenic	0.152	.	2
AC.GT	9	31832001	31832011	5.5	.	.	intergenic	0.111	.	2
AC.GT	9	31897034	31897044	5.5	ENSSSCT00000016332	MTMR2	intron	.	.	3
AGC.GCT	9	31932707	31932723	5.66667	.	.	intergenic	0.067	.	2
AG.CT	9	32110505	32110517	6.5	.	.	intergenic	0.311	rs786831315	2
AG.CT	9	32204333	32204342	5	.	.	intergenic	0.745	rs793874719	2
AAAC.GTTT	9	32304442	32304465	6	.	.	intergenic	0.123	rs790671634	2
AAAAC.GTTTT	9	32415772	32415794	4.6	ENSSSCT00000016334	CCDC82	intron	0.077	rs789526368	2
AAC.GTT	9	32784395	32784411	5.66667	.	.	intergenic	.	rs786381086	4
AAGG.CCTT	9	32812607	32812630	6	.	.	intergenic	-0.159	097729 rs6973	2
AC.GT	9	32893375	32893388	7	.	.	intergenic	-0.108	rs791212794	2
AC.GT	9	32918657	32918669	6.5	.	.	intergenic	-0.215	166524 rs7906	2
AAAC.GTTT	9	32932318	32932339	5.5	.	.	intergenic	0.119	rs790349412	4
AAAC.GTTT	9	32934107	32934126	5	.	.	intergenic	-0.042	359049 rs7133	4
AT.AT	9	32982753	32982775	11.5	.	.	intergenic	.	.	3
AT.AT	9	32986429	32986439	5.5	.	.	intergenic	.	.	3
AAAC.GTTT	9	32990760	32990787	7	.	.	intergenic	-0.089	381455 rs7876	2
AAAT.ATTT	9	33127317	33127332	4	.	.	intergenic	-0.039	rs788109172	2
AAAC.GTTT	9	33268160	33268171	3	.	.	intergenic	.	rs792710642	2
AAC.GTT	9	33711766	33711779	4.66667	.	.	intergenic	0.376	081099 rs7879	2
AAAC.GTTT	9	33765602	33765616	3.75	.	.	intergenic	-0.121	rs786390217	2
ACAT.ATGT	9	33851682	33851711	7.5	.	.	intergenic	.	rs787797767	2
AAAAG.CTTTT	9	33914344	33914360	3.4	.	.	intergenic	.	.	2
AC.GT	9	33946095	33946107	6.5	.	.	intergenic	.	rs789751856	2
AAAC.GTTT	9	33996883	33996905	5.75	.	.	intergenic	.	rs787562307	2
AAAAG.CTTTT	9	34206128	34206147	4	.	.	intergenic	.	773905 rs7872	2
AAC.GTT	9	34364052	34364074	7.66667	.	.	intergenic	-0.008	354386 rs7919	2
AC.GT	9	34448076	34448096	10.5	.	.	intergenic	-0.165	.	3
AAAC.GTTT	9	34691063	34691089	6.75	.	.	intergenic	0.098	rs787825136	2
AT.AT	9	34863998	34864011	7	.	.	intergenic	-0.048	rs789885388	2
AT.AT	9	34941134	34941147	7	.	.	intergenic	-0.135	923452 rs7894	2
AC.GT	9	34973412	34973455	22	.	.	intergenic	-0.008	rs699194832	2
AAAT.ATTT	9	35042103	35042124	5.5	.	.	intergenic	0.067	rs789567435	2
AT.AT	9	35188415	35188426	6	ENSSSCT00000016337	CNTN5	intron	0.076	rs793477795	2
AAAG.CTTT	9	35232074	35232093	5	ENSSSCT00000016337	CNTN5	intron	0.075	rs793121449	3
AAT.ATT	9	35395557	35395581	8.33333	ENSSSCT00000016337	CNTN5	intron	-0.212	rs791596313	2
AC.GT	9	35686227	35686243	8.5	.	.	intergenic	-0.035	.	2
AGAT.ATCT	9	35833309	35833334	6.5	.	.	intergenic	0.021	rs789023534	2
AAC.GTT	9	35948596	35948613	6	ENSSSCT00000016338	ARHGAP42	intron	1.886	175691 rs7930	2
AAC.GTT	9	36191021	36191056	12	.	.	intergenic	0.101	139444 rs7899	4
AAT.ATT	9	36335030	36335054	8.33333	ENSSSCT00000024893	TRPC6	intron	0.286	rs788330488	2
AAAC.GTTT	9	36364895	36364909	3.75	ENSSSCT00000024893	TRPC6	promoter	0.077	705019 rs7883	2
AAT.ATT	9	37063588	37063610	7.66667	.	.	intergenic	.	rs787411411	2
AG.CT	9	37313644	37313654	5.5	ENSSSCT00000022911	MMP27	intron	0.874	rs791186095	3
AG.CT	9	37313644	37313654	5.5	ENSSSCT00000016346	MMP27	intron	0.874	rs791186095	3
AAAT.ATTT	9	37348659	37348675	4.25	.	.	intergenic	-0.095	rs786279170	2
ATCC.GGAT	9	37587476	37587498	5.75	.	.	intergenic	0.186	048471 rs7901	3
AAG.CTT	9	37946907	37946920	4.66667	ENSSSCT00000016356	.	intron	0.118	rs789903919	2
AAAG.CTTT	9	38007898	38007910	3.25	.	.	intergenic	.	079518 rs7889	2
AATT.AATT	9	38165449	38165473	6.25	.	.	intergenic	0.381	rs791868284	2
AT.AT	9	38171419	38171428	5	.	.	intergenic	.	rs790725554	2
AATG.CATT	9	38237962	38237983	5.5	.	.	intergenic	-0.103	rs786465387	3
AC.GT	9	38334488	38334497	5	.	.	intergenic	.	rs788778050	2
AC.GT	9	38439746	38439755	5	.	.	intergenic	-0.006	274802 rs7898	2
AT.AT	9	38470468	38470481	7	ENSSSCT00000016357	PDGFD	intron	0.151	rs788353894	2
AG.CT	9	38525151	38525188	19	ENSSSCT00000016357	PDGFD	intron	-0.123	338827 rs7864	2
AAAC.GTTT	9	38645506	38645521	4	.	.	intergenic	0.25	rs790709616	2
AAAAC.GTTTT	9	38675975	38676008	6.8	.	.	intergenic	0.3	.	2
AAAAAT.ATTTTT	9	38678323	38678344	3.66667	.	.	intergenic	-0.13	rs789112714	2
AT.AT	9	38696614	38696623	5	.	.	intergenic	.	rs786900713	2

AC.GT	9	38716101	38716111	5.5	.	.	intergenic	.	rs791478261	2
AG.CT	9	38830290	38830299	5	.	.	intergenic	0.141	394301 rs7904	4
AC.GT	9	38835362	38835397	18	.	.	intergenic	-0.212	955807 rs7878	4
AT.AT	9	39103255	39103265	5.5	.	.	intergenic	.	rs789143987	2
AAC.GTT	9	39518012	39518025	4.66667	ENSSSCT00000027751	GRIA4	intron	.	283823 rs7915	2
AAAATT.AATTTT	9	39596985	39597017	5.5	ENSSSCT00000027751	GRIA4	intron	.	rs789205783	2
AC.GT	9	39677192	39677227	18	ENSSSCT00000027751	GRIA4	intron	.	rs791829801	3
AT.AT	9	39792654	39792664	5.5	ENSSSCT00000027751	GRIA4	intron	0.012	455645 rs7936	2
AAAC.GTTT	9	40206974	40207000	6.75	ENSSSCT00000016366	ELMOD1	intron	.	213442 rs7876	2
AAAG.CTTT	9	40999085	40999111	6.75	.	.	intergenic	.	rs707461527	3
AT.AT	9	41060062	41060078	8.5	ENSSSCT00000016373	KDELC2	intron	0.511	rs790261646	4
AGAT.ATCT	9	41092555	41092582	7	ENSSSCT00000016374	EXPH5	intron	-0.095	rs789193247	2
AAAAC.GTTTT	9	41259476	41259492	3.4	.	.	intergenic	0.31	rs791931794	2
AT.AT	9	41264211	41264222	6	.	.	intergenic	0.396	.	2
AAAAG.CTTTT	9	41345409	41345427	3.8	.	.	intergenic	-0.036	rs787204774	2
AAAAAC.GTTTTT	9	41889937	41889963	4.5	.	.	intergenic	0.131	rs791388179	2
AAC.GTT	9	41939585	41939606	7.33333	.	.	intergenic	.	201437 rs7897	2
AAAC.GTTT	9	42020323	42020345	5.75	.	.	intergenic	0.346	945121 rs7873	2
AT.AT	9	42065186	42065195	5	.	.	intergenic	.	rs790334887	2
AG.CT	9	42068985	42068998	7	.	.	intergenic	0.098	.	2
AG.CT	9	42278783	42278794	6	.	.	intergenic	.	rs792992348	2
AT.AT	9	42720761	42720793	16.5	.	.	intergenic	0.039	rs786873057	2
AAAG.CTTT	9	42931395	42931413	4.75	.	.	intergenic	0.14	498833 rs7929	2
AG.CT	9	43201317	43201328	6	ENSSSCT00000029186	.	intron	.	.	2
AG.CT	9	43201317	43201328	6	ENSSSCT00000016379	.	intron	.	.	2
AAAAC.GTTTT	9	43419295	43419313	3.8	.	.	intergenic	0.213	rs790498450	2
AC.GT	9	43513844	43513879	18	.	.	intergenic	.	rs787061253	4
AG.CT	9	43701735	43701744	5	.	.	intergenic	-0.01	.	2
AT.AT	9	43719487	43719497	5.5	.	.	intergenic	0.204	rs693095242	4
AG.CT	9	43739553	43739575	11.5	.	.	intergenic	-0.022	311601 rs7897	2
AAAAT.ATTTT	9	43897016	43897042	5.4	.	.	intergenic	.	rs704986473	2
AC.GT	9	44722375	44722384	5	.	.	intergenic	0.261	rs791106788	4
AT.AT	9	44850667	44850695	14.5	.	.	intergenic	.	.	2
AAATT.AATTT	9	45059706	45059723	3.6	.	.	intergenic	0.274	rs790464637	2
AAG.CTT	9	45181283	45181309	9	.	.	intergenic	0.029	531386 rs7870	2
AC.GT	9	45496104	45496122	9.5	.	.	intergenic	.	rs708122071	3
AG.CT	9	45558262	45558273	6	.	.	intergenic	0.152	rs786589383	2
AC.GT	9	45582812	45582823	6	.	.	intergenic	0.671	rs786746114	2
AAAC.GTTT	9	45634803	45634823	5.25	.	.	intergenic	.	598560 rs7909	2
AAC.GTT	9	45660504	45660521	6	.	.	intergenic	0.25	rs790449678	2
AC.GT	9	45661402	45661415	7	.	.	intergenic	0.165	rs791450812	4
AT.AT	9	45711050	45711060	5.5	.	.	intergenic	-0.145	rs789990948	2
AATC.GATT	9	45737446	45737462	4.25	ENSSSCT00000034031	NCAM1	intron	0.861	rs788095779	2
AATC.GATT	9	45737446	45737462	4.25	ENSSSCT00000036483	NCAM1	intron	0.861	rs788095779	2
AATC.GATT	9	45737446	45737462	4.25	ENSSSCT00000032935	NCAM1	intron	0.861	rs788095779	2
AATC.GATT	9	45737446	45737462	4.25	ENSSSCT00000016411	NCAM1	intron	0.861	rs788095779	2
AAAC.GTTT	9	45762119	45762133	3.75	ENSSSCT00000016411	NCAM1	intron	0.201	962320 rs7904	2
AAAC.GTTT	9	45762119	45762133	3.75	ENSSSCT00000034031	NCAM1	intron	0.201	962320 rs7904	2
AAAC.GTTT	9	45762119	45762133	3.75	ENSSSCT00000036483	NCAM1	intron	0.201	962320 rs7904	2
AAAC.GTTT	9	45762119	45762133	3.75	ENSSSCT00000032935	NCAM1	intron	0.201	962320 rs7904	2
AC.GT	9	45835273	45835283	5.5	ENSSSCT00000016413	TTC12	intron	0.202	.	2
AT.AT	9	45844491	45844505	7.5	ENSSSCT00000016413	TTC12	intron	-0.044	730478 rs7917	3
AAG.CTT	9	45927228	45927240	4.33333	.	.	intergenic	.	rs786990267	2
AAG.CTT	9	45948403	45948420	6	.	.	intergenic	-0.104	rs789892565	2
AGGG.CCCT	9	45962279	45962297	4.75	.	.	intergenic	-0.058	rs790440019	2
AC.GT	9	46096770	46096782	6.5	.	.	intergenic	0.183	.	5
AT.AT	9	46162039	46162050	6	.	.	intergenic	0.042	704739 rs7893	2
AAAAC.GTTTT	9	46221331	46221351	4.2	.	.	intergenic	.	rs703922132	2
AAAAAC.GTTTTT	9	46222566	46222588	3.83333	.	.	intergenic	0.238	rs786886021	2
AG.CT	9	46326058	46326068	5.5	.	.	intergenic	2.652	.	3
AAAC.GTTT	9	46661067	46661086	5	.	.	intergenic	-0.234	rs791491049	2
AAG.CTT	9	46663174	46663185	4	.	.	intergenic	-0.099	.	2
AAAGC.GCTTT	9	46703314	46703331	3.6	.	.	intergenic	0.135	rs793194223	2
AAC.GTT	9	46847196	46847218	7.66667	.	.	intergenic	0.157	705520 rs7903	2
AC.GT	9	46851575	46851587	6.5	.	.	intergenic	.	.	4
AAC.GTT	9	46912979	46912997	6.33333	.	.	intergenic	0.006	rs787721099	2
AG.CT	9	46958237	46958246	5	.	.	intergenic	0.139	.	4
AAAAAC.GTTTTT	9	47825145	47825164	3.33333	.	.	intergenic	0.594	rs788848389	2
AAAAG.CTTTT	9	48336468	48336497	6	.	.	intergenic	-0.022	rs791392401	2
AAC.GTT	9	48479279	48479292	4.66667	.	.	intergenic	0.355	137452 rs7890	2
AAAT.ATTT	9	48482670	48482686	4.25	.	.	intergenic	-0.383	056211 rs7920	2
AC.GT	9	48750827	48750847	10.5	.	.	intergenic	0.008	.	3
AG.CT	9	48773253	48773263	5.5	.	.	intergenic	-0.123	rs789192530	2
AAAC.GTTT	9	48864350	48864388	9.75	.	.	intergenic	0.087	rs789547256	4
AAG.CTT	9	48880189	48880200	4	.	.	intergenic	.	rs786878253	2
AC.GT	9	49103047	49103059	6.5	.	.	intergenic	0.267	.	2
AAAAC.GTTTT	9	49360027	49360065	7.8	ENSSSCT00000016437	SIK3	intron	.	rs789012235	2
AAAAC.GTTTT	9	49360027	49360065	7.8	ENSSSCT00000025641	SIK3	intron	.	rs789012235	2
AAAAT.ATTTT	9	49362663	49362700	7.6	ENSSSCT00000016437	SIK3	intron	.	rs790846894	2
AAAAT.ATTTT	9	49362663	49362700	7.6	ENSSSCT00000025641	SIK3	intron	.	rs790846894	2
AAAAAT.ATTTTT	9	49438394	49438421	4.66667	.	.	intergenic	0.254	208691 rs7889	2
AAAC.GTTT	9	49464458	49464481	6	.	.	intergenic	0.12	909462 rs7932	2
AAAC.GTTT	9	49690922	49690944	5.75	ENSSSCT00000016443	RNF214	intron	0.282	rs791410222	2
AAAT.ATTT	9	49696807	49696822	4	ENSSSCT00000016443	RNF214	intron	.	rs791005933	2

AAAT.ATTT	9	49766942	49766964	5.75	ENSSSCT00000016446	CEP164	intron	.	rs789563997	2
AAC.GTT	9	49784683	49784708	8.66667	ENSSSCT00000016446	CEP164	intron	.	313853 rs7882	2
ATC.GAT	9	49833527	49833546	6.66667	.	.	intergenic	.	990937 rs7912	2
AG.CT	9	50169268	50169278	5.5	ENSSSCT00000016448	.	intron	-0.009	390363 rs7125	3
AG.CT	9	50200979	50200989	5.5	ENSSSCT00000016448	.	intron	-0.113	.	4
AC.GT	9	50238178	50238188	5.5	.	.	intergenic	-0.161	.	2
AAG.CTT	9	50282548	50282559	4	.	.	intergenic	0.129	rs786614785	2
AC.GT	9	50448221	50448245	12.5	ENSSSCT00000016452	TMPRSS4	intron	0.006	.	2
AC.GT	9	50579539	50579552	7	ENSSSCT00000016456	MPZL3	3'utr	0.333	770427 rs7875	3
AAAAC.GTTTT	9	50582976	50582994	3.8	ENSSSCT00000016456	MPZL3	intron	-0.053	rs787700216	2
AT.AT	9	50613624	50613635	6	ENSSSCT00000016457	MPZL2	intron	0.247	rs788906408	2
AAAT.ATTT	9	50843964	50843981	4.5	ENSSSCT00000027542	IFT46	5'utr	0.519	rs787906375	2
AAAT.ATTT	9	50843964	50843981	4.5	ENSSSCT00000028599	ARCN1	promoter	0.519	rs787906375	2
AAAAT.ATTTT	9	51148386	51148402	3.4	.	.	intergenic	.	rs791592504	2
AG.CT	9	51220153	51220163	5.5	.	.	intergenic	-0.189	rs792336978	2
AT.AT	9	51293974	51293986	6.5	.	.	intergenic	.	rs788704293	2
AAAC.GTTT	9	51348882	51348899	4.5	ENSSSCT00000016476	DPAGT1	intron	-0.044	rs787777958	2
AG.CT	9	51381860	51381871	6	ENSSSCT00000016478	HINFP	intron	-0.084	.	4
AC.GT	9	51476962	51476974	6.5	ENSSSCT00000016481	CBL	intron	0.197	rs789765203	2
AAAC.GTTT	9	51564569	51564587	4.75	.	.	intergenic	.	rs789157038	2
AT.AT	9	52944347	52944359	6.5	.	.	intergenic	-0.532	.	2
AC.GT	9	53117633	53117644	6	ENSSSCT00000016497	GRIK4	intron	0.59	rs788885120	2
AG.CT	9	53218433	53218444	6	ENSSSCT00000016497	GRIK4	intron	-0.156	rs789939211	2
AG.CT	9	53222065	53222078	7	ENSSSCT00000016497	GRIK4	intron	1.747	rs786290893	2
AAAC.GTTT	9	53421117	53421132	4	ENSSSCT00000032235	TECTA	intron	0.048	rs789070806	4
ACAG.CTGT	9	53500489	53500508	5	.	.	intergenic	0.157	011506 rs7902	2
AACC.GGTT	9	53566884	53566905	5.5	ENSSSCT00000026020	SC5D	intron	0.025	734646 rs7923	4
AAAAT.ATTTT	9	53807280	53807294	3	ENSSSCT00000031058	SORL1	intron	.	rs790693585	2
AT.AT	9	53821920	53821929	5	ENSSSCT00000031058	SORL1	intron	-0.434	rs787021042	2
AAGC.GCTT	9	53975964	53975976	3.25	.	.	intergenic	0.558	105074 rs7892	2
ACAT.ATGT	9	54147210	54147229	5	.	.	intergenic	.	045688 rs7864	2
AC.GT	9	54174818	54174830	6.5	.	.	intergenic	-0.04	.	3
AAAC.GTTT	9	54218127	54218143	4.25	.	.	intergenic	.	rs788743789	2
AC.GT	9	54222989	54223003	7.5	.	.	intergenic	.	rs793223508	2
AAAC.GTTT	9	54366033	54366056	6	.	.	intergenic	.	rs788379187	2
AG.CT	9	54507041	54507052	6	.	.	intergenic	0.142	rs791322766	2
AC.GT	9	54775255	54775264	5	.	.	intergenic	-0.051	066350 rs7876	3
AAAT.ATTT	9	54812485	54812500	4	.	.	intergenic	0.253	rs792949313	2
AT.AT	9	55146003	55146016	7	ENSSSCT00000016503	CRTAM	intron	0.023	958272 rs7935	2
AC.GT	9	55187281	55187291	5.5	ENSSSCT00000016504	C11orf63	intron	0.128	rs787511183	2
AC.GT	9	55187281	55187291	5.5	ENSSSCT00000025784	C11orf63	intron	0.128	rs787511183	2
AAAT.ATTT	9	55229087	55229104	4.5	ENSSSCT00000016504	C11orf63	intron	0.6	rs788704935	2
AC.GT	9	55251648	55251659	6	ENSSSCT00000016505	BSX	intron	-0.286	rs793258178	3
AAAAC.GTTTT	9	55608923	55608940	3.6	.	.	intergenic	0.132	rs787703101	2
AAG.CTT	9	55720796	55720818	7.66667	.	.	intergenic	-0.177	312635 rs7891	2
AAAAT.ATTTT	9	56000211	56000228	3.6	.	.	intergenic	.	rs791019650	2
AAAG.CTTT	9	56541567	56541581	3.75	ENSSSCT00000016541	VWA5A	intron	0.098	rs793408245	2
AAAG.CTTT	9	56541567	56541581	3.75	ENSSSCT00000026868	VWA5A	intron	0.098	rs793408245	2
AAAT.ATTT	9	56595214	56595234	5.25	.	.	intergenic	0.136	546402 rs7871	2
AAAAC.GTTTT	9	56725368	56725391	4.8	.	.	intergenic	.	rs787448823	2
AAAC.GTTT	9	56746945	56746967	5.75	.	.	intergenic	.	rs787917629	2
AAAAC.GTTTT	9	56780575	56780596	4.4	.	.	intergenic	.	764760 rs7936	2
AT.AT	9	57340640	57340650	5.5	.	.	intergenic	.	.	3
AT.AT	9	57418027	57418036	5	ENSSSCT00000016562	.	intron	.	rs786429265	2
AGG.CCT	9	57422502	57422520	6.33333	ENSSSCT00000016562	.	intron	1.439	395489 rs7886	2
AC.GT	9	57582193	57582208	8	ENSSSCT00000016568	ROBO3	intron	0.435	rs787830558	2
AG.CT	9	58171085	58171099	7.5	ENSSSCT00000016577	.	intron	0.054	.	2
AT.AT	9	58400778	58400790	6.5	ENSSSCT00000016580	STT3A	intron	.	428896 rs7864	2
AAAAG.CTTTT	9	58445916	58445938	4.6	.	.	intergenic	.	369280 rs7911	2
AC.GT	9	59043411	59043425	7.5	.	.	intergenic	.	307064 rs7877	2
AC.GT	9	59348239	59348249	5.5	ENSSSCT00000016600	KIRREL3	intron	.	rs790437107	2
AC.GT	9	59389040	59389056	8.5	ENSSSCT00000016600	KIRREL3	intron	.	rs792300133	3
AG.CT	9	59489872	59489891	10	.	.	intergenic	.	rs792969792	2
AAAAAC.GTTTTT	9	59537127	59537155	4.83333	.	.	intergenic	.	534788 rs7930	2
AG.CT	9	60281212	60281229	9	.	.	intergenic	.	307621 rs7876	2
AAAC.GTTT	9	60368557	60368572	4	.	.	intergenic	.	rs791817934	2
AT.AT	9	60463731	60463743	6.5	.	.	intergenic	.	159688 rs7868	2
AC.GT	9	60760909	60760950	21	.	.	intergenic	.	.	2
AC.GT	9	60846338	60846348	5.5	.	.	intergenic	.	075319 rs7905	3
AC.GT	9	60894424	60894435	6	.	.	intergenic	.	rs697083775	2
AGAT.ATCT	9	61067534	61067557	6	.	.	intergenic	.	rs793482509	2
AC.GT	9	61104905	61104916	6	.	.	intergenic	.	.	4
ACC.GGT	9	61399865	61399883	6.33333	.	.	intergenic	.	552817 rs7926	2
AC.GT	9	61401601	61401618	9	.	.	intergenic	.	549152 rs7867	2
AC.GT	9	61656518	61656532	7.5	.	.	intergenic	.	.	2
AC.GT	9	61983824	61983836	6.5	.	.	intergenic	.	.	2
AC.GT	9	62800493	62800520	14	ENSSSCT00000016610	TMEM45B	intron	.	901449 rs7935	2
AAAAC.GTTTT	9	62981165	62981182	3.6	ENSSSCT00000016613	APLP2	intron	.	557305 rs7928	2
AAAG.CTTT	9	63122406	63122438	8.25	ENSSSCT00000016616	ZBTB44	intron	.	531602 rs7865	2
AAAG.CTTT	9	63122406	63122438	8.25	ENSSSCT00000032122	ZBTB44	intron	.	531602 rs7865	2
AC.GT	9	63403179	63403190	6	ENSSSCT00000016619	ADAMTS15	intron	.	.	3
AC.GT	9	63907896	63907908	6.5	.	.	intergenic	.	rs788166839	2
AAAAT.ATTTT	9	63920770	63920789	4	.	.	intergenic	.	rs787244717	2
AAAC.GTTT	9	64121538	64121565	7	.	.	intergenic	.	rs789888398	2

AAAC.GTTT	9	64580888	64580902	3.75	.	.	intergenic	.	rs789974133	2
AC.GT	9	64637071	64637099	14.5	.	.	intergenic	.	rs791013889	2
AGAT.ATCT	9	64782457	64782488	8	.	.	intergenic	.	248176 rs7919	3
AAAAC.GTTTT	9	64843884	64843906	4.6	.	.	intergenic	.	187378 rs7872	2
AG.CT	9	64987068	64987080	6.5	.	.	intergenic	.	rs793509422	2
AAAAAC.GTTTT	9	65027858	65027883	4.33333	.	.	intergenic	.	536364 rs7894	2
AAAT.ATTT	9	65034095	65034108	3.5	.	.	intergenic	.	rs792129112	2
AC.GT	9	65111126	65111137	6	.	.	intergenic	.	.	2
AT.AT	9	65237942	65237958	8.5	ENSSSCT00000016622	NTM	intron	.	.	2
AGAGG.CCTCT	9	65263431	65263473	7.16667	ENSSSCT00000016622	NTM	intron	.	rs698251682	2
AAAC.GTTT	9	65278476	65278500	6.25	ENSSSCT00000016622	NTM	intron	.	rs787033686	2
AC.GT	9	65743975	65743988	7	.	.	intergenic	.	rs789651972	2
AATC.GATT	9	65822788	65822811	6	.	.	intergenic	.	rs789367313	3
AAAAT.ATTTT	9	66213047	66213067	4.2	.	.	intergenic	.	.	2
AAAT.ATTT	9	66328561	66328582	5.5	.	.	intergenic	.	264193 rs7908	3
AAAT.ATTT	9	66552413	66552425	3.25	.	.	intergenic	.	rs793546199	2
AAAT.ATTT	9	66584420	66584434	3.75	.	.	intergenic	.	rs792632602	2
AC.GT	9	66654231	66654243	6.5	.	.	intergenic	.	.	4
AC.GT	9	66925457	66925469	6.5	.	.	intergenic	.	rs706312379	2
AATG.CATT	9	67139833	67139858	6.5	.	.	intergenic	.	rs792083896	2
AG.CT	9	67457220	67457230	5.5	ENSSSCT00000022758	.	intron	.	.	2
AAAAT.ATTTT	9	67607549	67607566	3.6	ENSSSCT00000016627	GLB1L2	intron	.	rs793804745	2
AC.GT	9	67826857	67826872	8	.	.	intergenic	.	.	2
AAAC.GTTT	9	67982422	67982438	4.25	.	.	intergenic	.	rs787747200	2
AAAC.GTTT	9	68038737	68038757	5.25	.	.	intergenic	.	rs790975004	2
AAAAC.GTTTT	9	68765147	68765162	3.2	.	.	intergenic	.	rs788994551	2
AAAT.ATTT	9	69093670	69093692	5.75	ENSSSCT00000024361	GUCY1A2	intron	0.279	901254 rs7901	2
AAAT.ATTT	9	69093670	69093692	5.75	ENSSSCT00000025938	GUCY1A2	intron	0.279	901254 rs7901	2
AC.GT	9	69969041	69969076	18	.	.	intergenic	.	.	3
AT.AT	9	70405802	70405815	7	.	.	intergenic	0.196	rs791525943	2
AAC.GTT	9	70436021	70436036	5.33333	.	.	intergenic	.	rs788953329	2
ATC.GAT	9	70470600	70470630	10.3333	.	.	intergenic	0.164	rs790068581	2
AAAT.ATTT	9	70908143	70908161	4.75	ENSSSCT00000023057	SNRPE	promoter	0.178	rs789287067	2
AG.CT	9	70966784	70966797	7	.	.	intergenic	.	rs789012955	2
AG.CT	9	70993879	70993889	5.5	.	.	intergenic	.	.	3
AAAC.GTTT	9	71149242	71149260	4.75	.	.	intergenic	.	rs793212952	2
AC.GT	9	71472095	71472107	6.5	ENSSSCT00000016654	PIK3C2B	intron	0.031	rs788259913	2
AC.GT	9	71489065	71489100	18	ENSSSCT00000016654	PIK3C2B	intron	0.615	rs786754553	3
AAAAC.GTTTT	9	71709416	71709443	5.6	.	.	intergenic	-0.01	.	2
AAAAAC.GTTTT	9	71778670	71778693	4	.	.	intergenic	-0.427	243548 rs7873	2
AC.GT	9	72281071	72281084	7	ENSSSCT00000016662	LEMD1	intron	-0.067	rs793853417	2
AG.CT	9	72410270	72410282	6.5	.	.	intergenic	-0.28	rs692243873	3
AAAAT.ATTTT	9	72574540	72574567	5.6	ENSSSCT00000028838	ELK4	intron	0.187	rs792854051	2
AC.GT	9	72665304	72665317	7	.	.	intergenic	0.266	.	3
AC.GT	9	72875995	72876012	9	.	.	intergenic	-0.093	rs790371111	2
AT.AT	9	72902796	72902805	5	.	.	intergenic	.	.	2
AC.GT	9	73000639	73000661	11.5	ENSSSCT00000017034	C1orf186	intron	.	rs791086610	2
AAAC.GTTT	9	73005367	73005389	5.75	ENSSSCT00000017034	C1orf186	intron	-0.076	rs791251087	2
AAC.GTT	9	73068705	73068737	11	.	.	intergenic	.	411236 rs7867	2
AT.AT	9	73073030	73073039	5	.	.	intergenic	-0.393	rs793592742	2
AAAT.ATTT	9	73078420	73078434	3.75	.	.	intergenic	0.488	rs793260342	2
AC.GT	9	73228050	73228078	14.5	ENSSSCT00000017041	SRGAP2	intron	0.076	290770 rs7890	2
AAG.CTT	9	73288306	73288325	6.66667	ENSSSCT00000017041	SRGAP2	intron	-0.192	rs790275853	2
AAAC.GTTT	9	73305861	73305879	4.75	ENSSSCT00000017041	SRGAP2	intron	0.16	rs788241238	2
AAAC.GTTT	9	73637225	73637246	5.5	.	.	intergenic	-0.053	rs788550820	2
AATC.GATT	9	74112158	74112188	7.75	ENSSSCT00000017060	C4BPA	intron	0.115	442188 rs7901	2
AC.GT	9	74464037	74464047	5.5	ENSSSCT00000028859	CR2	intron	0.156	rs787583275	3
AAAC.GTTT	9	74488139	74488161	5.75	ENSSSCT00000028859	CR2	intron	0.326	037833 rs7896	3
AC.GT	9	74523899	74523913	7.5	ENSSSCT00000033340	CR1	intron	-0.14	rs786767276	4
AG.CT	9	75038982	75038998	8.5	.	.	intergenic	0.048	rs791050818	2
AT.AT	9	75051972	75051984	6.5	.	.	intergenic	0.624	rs788021079	3
AC.GT	9	75230830	75230849	10	.	.	intergenic	0.556	.	2
AT.AT	9	75258997	75259006	5	.	.	intergenic	-0.042	.	2
AC.GT	9	75274162	75274179	9	.	.	intergenic	.	rs708587834	5
AAAC.GTTT	9	75310954	75310968	3.75	.	.	intergenic	0.016	rs793052814	2
AAC.GTT	9	75484992	75485005	4.66667	.	.	intergenic	-0.092	rs789661865	2
AAAC.GTTT	9	75546068	75546092	6.25	.	.	intergenic	-0.045	rs791048013	2
AC.GT	9	75607624	75607639	8	.	.	intergenic	.	.	2
ATC.GAT	9	75639123	75639139	5.66667	.	.	intergenic	0.023	rs793517484	2
AC.GT	9	75710416	75710426	5.5	.	.	intergenic	-0.13	rs787156089	2
AAAAG.CTTTT	9	75853493	75853512	4	.	.	intergenic	0.004	rs790689585	2
AG.CT	9	75978205	75978219	7.5	.	.	intergenic	.	rs792697806	2
AAAC.GTTT	9	76008247	76008267	5.25	.	.	intergenic	-0.067	245423 rs7892	3
AG.CT	9	76091413	76091435	11.5	.	.	intergenic	-0.347	rs789926364	2
AAAT.ATTT	9	76170537	76170560	6	.	.	intergenic	.	564816 rs7864	2
AAAACT.AGTTT	9	76261173	76261190	3	.	.	intergenic	.	rs787310633	2
AAC.GTT	9	76464348	76464359	4	.	.	intergenic	0.371	rs694693675	3
AAAG.CTTT	9	76574170	76574184	3.75	.	.	intergenic	.	rs690021944	2
AC.GT	9	76682149	76682163	7.5	.	.	intergenic	0.298	rs790581964	2
AAAC.GTTT	9	76742405	76742429	6.25	.	.	intergenic	0.215	rs793315469	2
AAT.ATT	9	77273241	77273257	5.66667	.	.	intergenic	0.04	rs787119386	2
AAT.ATT	9	77341068	77341097	10	ENSSSCT00000016680	CDK14	intron	0.246	717069 rs7868	2
AG.CT	9	77368798	77368807	5	ENSSSCT00000016680	CDK14	intron	.	.	4
AC.GT	9	77771683	77771692	5	ENSSSCT00000016680	CDK14	intron	.	rs791743949	3

AAAC.GTTT	9	77867483	77867501	4.75	.	.	intergenic	0.216	rs789469730	2
AT.AT	9	77931326	77931349	12	.	.	intergenic	0.575	rs789188889	2
AAGG.CCTT	9	77944518	77944539	5.5	.	.	intergenic	0.212	745637 rs7938	2
AT.AT	9	78374389	78374399	5.5	.	.	intergenic	.	.	2
AAG.CTT	9	78443272	78443284	4.33333	.	.	intergenic	0.249	rs788404199	2
AT.AT	9	78539522	78539532	5.5	.	.	intergenic	0.316	055769 rs7868	2
AT.AT	9	78559071	78559085	7.5	.	.	intergenic	0.256	236153 rs7909	4
AG.CT	9	78863569	78863579	5.5	.	.	intergenic	.	rs787751365	2
AAGG.CCTT	9	79211519	79211537	4.75	ENSSSCT00000016690	FAM133B	intron	.	rs789513491	2
AC.GT	9	79297795	79297822	14	ENSSSCT00000036613	CDK6	intron	0.054	202735 rs7863	3
AAAAG.CTTTT	9	79300105	79300133	5.8	ENSSSCT00000036613	CDK6	intron	0.908	rs792838967	2
AAAAC.GTTTT	9	79303425	79303452	5.6	ENSSSCT00000036613	CDK6	intron	0.181	rs789694558	2
AAAC.GTTT	9	79702735	79702757	5.75	.	.	intergenic	.	202827 rs7919	2
AGAT.ATCT	9	79813542	79813560	4.75	ENSSSCT00000024549	HEPACAM2	intron	0.303	rs791948749	2
AGAT.ATCT	9	79813542	79813560	4.75	ENSSSCT00000028375	HEPACAM2	intron	0.303	rs791948749	2
AAAAT.ATTTT	9	80079885	80079903	3.8	.	.	intergenic	.	rs790622197	2
AAAT.ATTT	9	80084488	80084508	5.25	ENSSSCT00000032573	.	promoter	2.021	.	2
AAT.ATT	9	80084747	80084766	6.66667	ENSSSCT00000032573	.	intron	2.077	.	3
AG.CT	9	80532456	80532470	7.5	.	.	intergenic	0.298	rs789878003	2
AAC.GTT	9	81514023	81514042	6.66667	ENSSSCT00000016700	CASD1	intron	.	rs789961539	2
AAAC.GTTT	9	81537783	81537801	4.75	ENSSSCT00000016700	CASD1	intron	0.267	rs790141777	2
AAAAC.GTTTT	9	81719290	81719317	5.6	.	.	intergenic	.	377614 rs7868	3
AAC.GTT	9	82277488	82277502	5	.	.	intergenic	0.211	rs791170207	2
ATC.GAT	9	82558243	82558262	6.66667	ENSSSCT00000016706	ASB4	intron	0.345	rs702251457	2
ACAT.ATGT	9	82588465	82588485	5.25	.	.	intergenic	0.029	rs710374313	2
AAAAC.GTTTT	9	82752115	82752145	6.2	.	.	intergenic	.	rs789624486	2
AAC.GTT	9	83113286	83113299	4.66667	ENSSSCT00000016708	DYNC1I1	intron	.	189078 rs7912	2
AG.CT	9	83139318	83139330	6.5	ENSSSCT00000016708	DYNC1I1	intron	0.19	.	4
AT.AT	9	83367427	83367443	8.5	.	.	intergenic	0.238	rs790572509	2
AC.GT	9	84022824	84022839	8	.	.	intergenic	-0.003	.	2
AC.GT	9	84133248	84133258	5.5	.	.	intergenic	1.385	.	2
AAC.GTT	9	84317213	84317229	5.66667	.	.	intergenic	.	rs792677837	2
ACAT.ATGT	9	84387259	84387277	4.75	.	.	intergenic	0.06	rs790464505	2
AC.GT	9	84671054	84671069	8	.	.	intergenic	0.05	.	2
AAAAC.GTTTT	9	84787117	84787140	4.8	.	.	intergenic	0.287	734990 rs7933	2
AT.AT	9	84822643	84822653	5.5	.	.	intergenic	.	rs789026999	2
AAAC.GTTT	9	84872138	84872157	5	.	.	intergenic	.	523984 rs7937	2
AAC.GTT	9	84879293	84879304	4	.	.	intergenic	-0.501	.	2
AAAC.GTTT	9	85053689	85053708	5	.	.	intergenic	0.28	rs789673825	2
AAC.GTT	9	85269497	85269515	6.33333	ENSSSCT00000016715	COL28A1	intron	0.059	.	2
ACAT.ATGT	9	85471167	85471181	3.75	ENSSSCT00000016715	COL28A1	intron	.	rs791493941	2
AAC.GTT	9	85513076	85513091	5.33333	.	.	intergenic	0.186	rs694846967	2
AGAT.ATCT	9	86176104	86176136	8.25	.	.	intergenic	0.14	353238 rs7930	2
AAC.GTT	9	86178709	86178722	4.66667	.	.	intergenic	0.069	rs792571638	2
AC.GT	9	86454420	86454432	6.5	.	.	intergenic	0.032	rs788667026	2
AT.AT	9	86672864	86672874	5.5	.	.	intergenic	0.02	rs789178435	2
AC.GT	9	86674713	86674722	5	.	.	intergenic	.	rs790870834	3
AGAT.ATCT	9	86974699	86974713	3.75	.	.	intergenic	.	363992 rs7938	2
AC.GT	9	87184700	87184710	5.5	.	.	intergenic	.	rs786706727	2
AAAAC.GTTTT	9	87228031	87228053	4.6	.	.	intergenic	0.379	rs790154592	2
AT.AT	9	87257167	87257177	5.5	.	.	intergenic	0.185	762759 rs7883	2
AG.CT	9	87763573	87763587	7.5	.	.	intergenic	0.742	rs791066314	2
AC.GT	9	87825501	87825515	7.5	.	.	intergenic	-0.079	.	2
AAAC.GTTT	9	87855027	87855046	5	.	.	intergenic	.	rs790894964	2
AAAG.CTTT	9	87974855	87974866	3	.	.	intergenic	.	rs787297553	2
AT.AT	9	87979180	87979189	5	.	.	intergenic	0.092	.	2
AC.GT	9	88159941	88159958	9	.	.	intergenic	0.101	275569 rs7929	2
AAC.GTT	9	88479607	88479626	6.66667	.	.	intergenic	.	rs789119057	2
AG.CT	9	88479869	88479880	6	.	.	intergenic	.	rs788446265	2
AT.AT	9	88544553	88544563	5.5	.	.	intergenic	0.227	rs787581329	2
AG.CT	9	88580265	88580277	6.5	.	.	intergenic	.	.	2
AG.CT	9	88642602	88642612	5.5	ENSSSCT00000030889	PHF14	cds	2.309	.	2
AG.CT	9	88642602	88642612	5.5	ENSSSCT00000031171	PHF14	cds	2.309	.	2
AG.CT	9	88642602	88642612	5.5	ENSSSCT00000032105	PHF14	cds	2.309	.	2
AAG.CTT	9	88843124	88843139	5.33333	.	.	intergenic	.	rs791915238	2
ATCC.GGAT	9	88940294	88940335	10.5	ENSSSCT00000024928	THSD7A	intron	0.121	.	2
ATCC.GGAT	9	88940294	88940335	10.5	ENSSSCT00000025355	THSD7A	intron	0.121	.	2
ATCC.GGAT	9	88940294	88940335	10.5	ENSSSCT00000016724	THSD7A	intron	0.121	.	2
AAAT.ATTT	9	89107787	89107804	4.5	ENSSSCT00000024928	THSD7A	intron	0.008	rs786284705	2
AAAT.ATTT	9	89107787	89107804	4.5	ENSSSCT00000025355	THSD7A	intron	0.008	rs786284705	2
AAAT.ATTT	9	89107787	89107804	4.5	ENSSSCT00000016724	THSD7A	intron	0.008	rs786284705	2
AC.GT	9	89579605	89579615	5.5	.	.	intergenic	0.076	.	2
AAT.ATT	9	90032373	90032387	5	.	.	intergenic	0.006	rs792405219	2
AAAC.GTTT	9	90197333	90197365	8.25	.	.	intergenic	0.146	rs787398444	3
AAAAC.GTTTT	9	90333750	90333771	4.4	ENSSSCT00000016727	SCIN	intron	.	rs791288304	3
AT.AT	9	90408763	90408774	6	.	.	intergenic	0.267	rs786498153	2
AG.CT	9	90471190	90471201	6	.	.	intergenic	0.406	rs790059667	2
AAC.GTT	9	91103378	91103397	6.66667	.	.	intergenic	-0.094	rs789121382	2
AAAAT.ATTTT	9	91174317	91174336	4	.	.	intergenic	0.096	rs790488362	2
AC.GT	9	91418828	91418839	6	.	.	intergenic	.	rs791779119	2
AC.GT	9	91492431	91492441	5.5	.	.	intergenic	-0.225	rs790945136	2
AAAC.GTTT	9	91539836	91539862	6.75	ENSSSCT00000029225	ETV1	intron	0.087	240149 rs7927	2
AAAAAC.GTTTTT	9	92220533	92220566	5.66667	.	.	intergenic	.	469294 rs7916	2
AATT.AATT	9	92297509	92297526	4.5	.	.	intergenic	-0.121	799856 rs7905	2

AAAC.GTTT	9	92491725	92491745	5.25	.	.	intergenic	.	rs787689991	2
AT.AT	9	92551911	92551920	5	.	.	intergenic	-0.172	rs789683011	3
AAT.ATT	9	93223373	93223386	4.66667	.	.	intergenic	-0.019	rs788235429	2
ATC.GAT	9	93249427	93249438	4	ENSSSCT00000016731	.	intron	1.27	.	2
AAAC.GTTT	9	93376045	93376065	5.25	.	.	intergenic	.	386044 rs7922	2
AAAG.CTTT	9	93868217	93868243	6.75	.	.	intergenic	-0.017	503720 rs7930	2
AAAC.GTTT	9	94127974	94127992	4.75	.	.	intergenic	-0.04	rs789282185	2
AT.AT	9	94153285	94153294	5	.	.	intergenic	0.059	rs790581864	3
AT.AT	9	94238610	94238622	6.5	.	.	intergenic	-0.04	.	2
AT.AT	9	94239421	94239445	12.5	.	.	intergenic	0.217	rs790377964	5
AAAAC.GTTTT	9	94334745	94334767	4.6	ENSSSCT00000029064	.	intron	.	rs789611584	2
AAAT.ATTT	9	94389448	94389469	5.5	ENSSSCT00000029064	.	intron	0.026	555767 rs7873	2
AAAT.ATTT	9	94576270	94576296	6.75	.	.	intergenic	.	.	2
AAAT.ATTT	9	94858252	94858265	3.5	.	.	intergenic	-0.191	rs792051717	2
AAAAC.GTTTT	9	95141258	95141277	4	.	.	intergenic	.	rs793549523	2
AT.AT	9	95172700	95172709	5	.	.	intergenic	0.086	rs790689400	2
AT.AT	9	95311912	95311926	7.5	.	.	intergenic	.	rs792993351	2
AG.CT	9	95392308	95392318	5.5	ENSSSCT00000027011	.	promoter	.	.	2
AG.CT	9	95392308	95392318	5.5	ENSSSCT00000016739	.	promoter	.	.	2
AT.AT	9	95744616	95744626	5.5	.	.	intergenic	0.02	733443 rs7899	3
AAAAG.CTTTT	9	95807836	95807878	7.16667	.	.	intergenic	0.01	145759 rs7934	2
AT.AT	9	95876529	95876544	8	.	.	intergenic	0.052	rs790042417	2
AC.GT	9	95939519	95939532	7	.	.	intergenic	.	.	2
AAT.ATT	9	95972995	95973007	4.33333	.	.	intergenic	0.029	rs704451919	2
AG.CT	9	96012796	96012806	5.5	ENSSSCT00000029645	SNX13	intron	.	.	2
AAAC.GTTT	9	96018738	96018765	7	ENSSSCT00000029645	SNX13	intron	0.32	004535 rs7899	2
AAAAC.GTTTT	9	96100711	96100733	3.83333	ENSSSCT00000029645	SNX13	intron	.	346110 rs7920	2
AC.GT	9	96202468	96202478	5.5	.	.	intergenic	0.426	rs793395249	2
AC.GT	9	96236296	96236310	7.5	.	.	intergenic	.	371429 rs7916	3
AG.CT	9	96520749	96520763	7.5	.	.	intergenic	0.136	rs788396659	2
AAAC.GTTT	9	96561370	96561384	3.75	.	.	intergenic	0.373	748442 rs7887	2
AG.CT	9	96584640	96584657	9	.	.	intergenic	-0.113	rs788747541	2
AAT.ATT	9	96616191	96616214	8	.	.	intergenic	.	099828 rs7936	2
AGG.CCT	9	97075027	97075042	5.33333	.	.	intergenic	1.745	rs792427080	2
AT.AT	9	97093126	97093137	6	.	.	intergenic	.	.	2
AT.AT	9	97152962	97152973	6	.	.	intergenic	.	rs789288586	2
AAAT.ATTT	9	97292876	97292894	4.75	.	.	intergenic	0.066	.	2
AT.AT	9	97471253	97471265	6.5	.	.	intergenic	0.185	rs790788601	2
AAAG.CTTT	9	97564524	97564543	5	.	.	intergenic	-0.273	rs787339683	2
AC.GT	9	97798367	97798379	6.5	.	.	intergenic	.	rs786771774	4
AC.GT	9	98069557	98069571	7.5	.	.	intergenic	0.26	rs790106814	2
AAT.ATT	9	98116403	98116419	5.66667	.	.	intergenic	.	rs793044333	2
AAAT.ATTT	9	98177440	98177455	4	.	.	intergenic	0.059	rs787218709	2
AAAAT.ATTTT	9	98190141	98190158	3.6	.	.	intergenic	-0.037	rs788688215	2
AG.CT	9	98229597	98229633	18.5	.	.	intergenic	0.031	rs789373164	2
AC.GT	9	98314832	98314845	7	.	.	intergenic	0.051	rs787733015	2
AT.AT	9	98328636	98328646	5.5	ENSSSCT00000025990	MACC1	intron	0.01	rs789932055	2
AT.AT	9	98335899	98335915	8.5	ENSSSCT00000025990	MACC1	intron	0.252	rs788330170	3
AAAC.GTTT	9	98456486	98456505	5	.	.	intergenic	0.16	114895 rs7873	2
AAAC.GTTT	9	98925980	98925999	5	ENSSSCT00000016752	ABCB5	intron	0.123	rs789121637	2
AC.GT	9	99190973	99191006	17	.	.	intergenic	0.183	303552 rs7915	2
AAAAT.ATTTT	9	99234924	99234948	5	.	.	intergenic	0.274	766312 rs7863	2
AAAC.GTTT	9	99625954	99625975	5.5	.	.	intergenic	0.026	rs787704710	2
AAAC.GTTT	9	99843986	99844002	4.25	ENSSSCT00000016754	DNAH11	intron	.	rs790662068	2
AT.AT	9	99880678	99880692	7.5	ENSSSCT00000016754	DNAH11	intron	.	rs788166128	2
AC.GT	9	99905290	99905301	6	ENSSSCT00000016754	DNAH11	intron	.	rs707623446	3
AAAC.GTTT	9	99915095	99915106	3	ENSSSCT00000016754	DNAH11	intron	1.63	rs787981669	2
AAT.ATT	9	100003894	100003916	7.66667	ENSSSCT00000016754	DNAH11	intron	-0.024	rs787827182	2
AC.GT	9	100052895	100052906	6	ENSSSCT00000016754	DNAH11	intron	.	rs788598533	2
AAAAC.GTTTT	9	100058648	100058666	3.8	ENSSSCT00000016754	DNAH11	intron	-0.321	rs787320055	2
AAAAG.CTTTT	9	100059656	100059671	3.2	ENSSSCT00000016754	DNAH11	intron	-0.149	rs790415367	2
AAAAC.GTTTT	9	100154382	100154397	3.2	ENSSSCT00000016754	DNAH11	intron	0.163	rs791760435	2
AAAAG.CTTTT	9	100238989	100239015	5.4	.	.	intergenic	.	rs792009992	2
AAC.GTT	9	100280230	100280244	5	.	.	intergenic	0.121	rs793683589	4
AAC.GTT	9	100399291	100399307	5.66667	.	.	intergenic	-0.221	247264 rs7923	2
AAAC.GTTT	9	100419459	100419486	7	ENSSSCT00000016758	RAPGEF5	intron	0.571	rs789154067	2
AC.GT	9	100434024	100434042	9.5	ENSSSCT00000016758	RAPGEF5	intron	-0.003	464050 rs7926	2
AAAC.GTTT	9	100461838	100461862	6.25	ENSSSCT00000016758	RAPGEF5	intron	-0.044	rs788295783	2
AAAAC.GTTTT	9	100651812	100651840	5.8	.	.	intergenic	0.079	rs704208738	2
AC.GT	9	100859740	100859771	16	.	.	intergenic	.	rs792884417	2
ATC.GAT	9	101192322	101192339	6	.	.	intergenic	0.241	rs712173891	2
AAC.GTT	9	101254353	101254366	4.66667	ENSSSCT00000022993	FAM126A	intron	0.253	rs788169672	2
AAC.GTT	9	101254353	101254366	4.66667	ENSSSCT00000016761	FAM126A	intron	0.253	rs788169672	2
AAAAT.ATTTT	9	101500607	101500628	4.4	.	.	intergenic	-0.039	rs793106616	2
AC.GT	9	101555684	101555698	7.5	ENSSSCT00000030773	.	intron	1.359	rs789712567	2
AC.GT	9	101717328	101717340	6.5	ENSSSCT00000029993	IGF2BP3	intron	.	rs788299579	2
AT.AT	9	102088039	102088050	6	ENSSSCT00000026738	RUNDC3B	intron	.	rs788349748	2
AT.AT	9	102088039	102088050	6	ENSSSCT00000030941	RUNDC3B	intron	.	rs788349748	2
AAAC.GTTT	9	102170202	102170216	3.75	.	.	intergenic	.	rs788164439	2
AAAAC.GTTTT	9	102405959	102405986	5.6	.	.	intergenic	.	rs786208115	2
AAAG.CTTT	9	102513674	102513697	6	.	.	intergenic	0.053	rs791834419	2
AAC.GTT	9	102618958	102618977	6.66667	ENSSSCT00000016765	PGP1A	intron	0.153	533088 rs7872	2
AAC.GTT	9	102618958	102618977	6.66667	ENSSSCT00000034567	PGP1A	intron	0.153	533088 rs7872	2
AAAAG.CTTTT	9	102749507	102749526	4	ENSSSCT00000016766	CROT	intron	0.001	rs787977186	2

AG.CT	9	102776582	102776596	7.5	ENSSSCT00000016766	CROT	intron	.	rs787370890	2
AAC.GTT	9	102807311	102807328	6	.	.	intergenic	.	500988 rs7898	3
AG.CT	9	102840401	102840410	5	.	.	intergenic	.	rs793603397	2
AAAT.ATTT	9	102872469	102872493	6.25	ENSSSCT00000026505	TMEM243	promoter	-0.005	rs787331612	2
AAAC.GTTT	9	103136055	103136078	6	ENSSSCT00000016769	KIAA1324L	intron	0.044	rs790783040	2
AT.AT	9	103162818	103162827	5	ENSSSCT00000016769	KIAA1324L	intron	0.042	.	3
AAAC.GTTT	9	103240726	103240744	4.75	.	.	intergenic	.	rs789227469	2
AT.AT	9	103322866	103322884	9.5	.	.	intergenic	0.163	948654 rs7890	2
AAAT.ATTT	9	103339650	103339665	4	.	.	intergenic	0.147	.	2
AC.GT	9	104239688	104239707	10	.	.	intergenic	.	377315 rs7883	4
AAAC.GTTT	9	104270351	104270370	5	.	.	intergenic	-0.079	rs792914742	2
AAAATT.AATTTT	9	104724340	104724366	4.5	.	.	intergenic	.	rs789990591	2
ACAGC.GCTGT	9	104776819	104776836	3.6	.	.	intergenic	0.613	rs791546139	2
AT.AT	9	104787195	104787204	5	.	.	intergenic	.	.	2
AAAC.GTTT	9	104973039	104973061	5.75	.	.	intergenic	-0.118	rs788568658	2
AAAAT.ATTTT	9	105174814	105174831	3.6	.	.	intergenic	-0.1	rs793687633	2
AT.AT	9	105249180	105249190	5.5	.	.	intergenic	0.046	rs793270941	2
AAAC.GTTT	9	105414842	105414865	6	.	.	intergenic	.	rs792718110	2
AAAC.GTTT	9	105415109	105415129	5.25	.	.	intergenic	.	060358 rs7907	2
AAAT.ATTT	9	105476909	105476930	5.5	ENSSSCT00000016771	SEMA3D	intron	0.071	rs792718382	2
AC.GT	9	105519023	105519041	9.5	ENSSSCT00000016771	SEMA3D	intron	0.556	rs786705328	2
AAAT.ATTT	9	105948548	105948562	3.75	.	.	intergenic	.	.	2
AAAG.CTTT	9	105961919	105961936	4.5	.	.	intergenic	-0.198	rs789802130	2
AAAT.ATTT	9	105964122	105964154	8.25	.	.	intergenic	-0.01	949601 rs7882	2
AAAAC.GTTTT	9	106104161	106104198	7.6	.	.	intergenic	0.183	rs789247435	2
AAAAC.GTTTT	9	106345615	106345630	3.2	.	.	intergenic	0.051	rs788809051	2
AC.GT	9	106787296	106787310	7.5	.	.	intergenic	0.103	.	3
AAAT.ATTT	9	106848109	106848128	5	.	.	intergenic	0.22	rs787448756	2
AAAAC.GTTTT	9	106862128	106862145	3.6	.	.	intergenic	-0.16	253291 rs7884	2
AAAAC.GTTTT	9	107026829	107026854	5.2	ENSSSCT00000016774	SEMA3E	intron	0.161	rs792290526	2
AT.AT	9	107105502	107105528	13.5	ENSSSCT00000016774	SEMA3E	intron	-0.049	rs789085396	2
AGAT.ATCT	9	107149659	107149690	8	.	.	intergenic	.	551065 rs7880	2
AT.AT	9	107214626	107214635	5	.	.	intergenic	-0.078	rs790558264	4
AC.GT	9	107295342	107295351	5	.	.	intergenic	-0.061	rs712127363	3
AAAC.GTTT	9	107378876	107378893	4.5	ENSSSCT00000016776	PCLO	intron	-0.026	rs787365383	2
AAAC.GTTT	9	107378876	107378893	4.5	ENSSSCT00000025400	PCLO	intron	-0.026	rs787365383	2
AAAC.GTTT	9	107378876	107378893	4.5	ENSSSCT00000027728	PCLO	intron	-0.026	rs787365383	2
AATG.CATT	9	107466687	107466727	10.25	ENSSSCT00000016776	PCLO	intron	0.112	775943 rs7907	3
AATG.CATT	9	107466687	107466727	10.25	ENSSSCT00000025400	PCLO	intron	0.112	775943 rs7907	3
AATG.CATT	9	107466687	107466727	10.25	ENSSSCT00000027728	PCLO	intron	0.112	775943 rs7907	3
AATC.GATT	9	107478072	107478090	4.75	ENSSSCT00000025400	PCLO	intron	-0.077	rs791461415	2
AATC.GATT	9	107478072	107478090	4.75	ENSSSCT00000027728	PCLO	intron	-0.077	rs791461415	2
AATC.GATT	9	107478072	107478090	4.75	ENSSSCT00000016776	PCLO	intron	-0.077	rs791461415	2
AAAT.ATTT	9	107638010	107638024	3.75	ENSSSCT00000016776	PCLO	intron	0.984	rs790945519	2
AAAT.ATTT	9	107638010	107638024	3.75	ENSSSCT00000025400	PCLO	intron	0.984	rs790945519	2
AAAT.ATTT	9	107638010	107638024	3.75	ENSSSCT00000027728	PCLO	intron	0.984	rs790945519	2
AG.CT	9	107724555	107724565	5.5	ENSSSCT00000016776	PCLO	intron	-0.189	.	2
AG.CT	9	107724555	107724565	5.5	ENSSSCT00000025400	PCLO	intron	-0.189	.	2
AG.CT	9	107724555	107724565	5.5	ENSSSCT00000027728	PCLO	intron	-0.189	.	2
AAAC.GTTT	9	108001608	108001626	4.75	.	.	intergenic	.	182214 rs7898	2
AC.GT	9	108121058	108121070	6.5	ENSSSCT00000016777	CACNA2D1	intron	-0.136	rs789478293	2
AT.AT	9	108348579	108348602	12	ENSSSCT00000016777	CACNA2D1	intron	0.178	371146 rs7889	2
AG.CT	9	108355561	108355577	8.5	ENSSSCT00000016777	CACNA2D1	intron	-0.117	011204 rs7906	2
AC.GT	9	108461001	108461017	8.5	ENSSSCT00000016777	CACNA2D1	intron	-0.057	rs793693753	2
AG.CT	9	108465182	108465203	11	ENSSSCT00000016777	CACNA2D1	intron	0.031	.	5
AAAC.GTTT	9	108634993	108635012	5	.	.	intergenic	-0.12	rs786668922	2
AAT.ATT	9	108671795	108671811	5.66667	.	.	intergenic	0.036	rs792301533	2
AAC.GTT	9	108675004	108675029	8.66667	.	.	intergenic	0.062	488294 rs7877	2
AG.CT	9	108753304	108753317	7	.	.	intergenic	-0.249	rs793533320	2
AAC.GTT	9	109037436	109037447	4	.	.	intergenic	0.089	rs792993297	2
AAAG.CTTT	9	109244937	109244949	3.25	.	.	intergenic	.	.	2
AC.GT	9	109248571	109248582	6	.	.	intergenic	0.081	.	2
AAC.GTT	9	109320519	109320532	4.66667	.	.	intergenic	.	rs793511566	3
ACAT.ATGT	9	109496277	109496316	10	.	.	intergenic	-0.141	.	2
AAAT.ATTT	9	109554243	109554259	4.25	.	.	intergenic	.	rs792335298	2
AAT.ATT	9	109556989	109557004	5.33333	.	.	intergenic	0.266	rs787494556	2
AT.AT	9	109574148	109574157	5	.	.	intergenic	0.046	.	2
AAAAT.ATTTT	9	109606739	109606768	6	ENSSSCT00000016779	.	intron	.	.	2
AAAC.GTTT	9	109674557	109674583	6.75	ENSSSCT00000016779	.	intron	-0.304	rs787188557	2
AAATC.GATTT	9	109768642	109768664	4.6	.	.	intergenic	-0.107	rs792113163	2
AT.AT	9	109808279	109808291	6.5	.	.	intergenic	0.021	rs791161182	2
AC.GT	9	109872889	109872907	9.5	.	.	intergenic	.	rs788057266	3
AT.AT	9	110022962	110022979	9	.	.	intergenic	.	rs789036241	2
AGAT.ATCT	9	110280360	110280383	6	ENSSSCT00000016781	GNAT3	promoter	0.178	594677 rs7872	5
AAC.GTT	9	110317908	110317924	5.66667	ENSSSCT00000016781	GNAT3	intron	1.482	rs790361978	4
AAAT.ATTT	9	110391209	110391228	5	.	.	intergenic	0.141	751955 rs7913	2
AC.GT	9	110461441	110461461	10.5	.	.	intergenic	0.034	.	2
AAAG.CTTT	9	110805712	110805732	5.25	.	.	intergenic	.	rs789836680	2
AG.CT	9	110829433	110829454	11	.	.	intergenic	.	rs791054338	2
AC.GT	9	111029645	111029676	16	.	.	intergenic	.	rs695270297	4
AT.AT	9	111056016	111056026	5.5	.	.	intergenic	-0.129	rs788158050	4
AAC.GTT	9	111081517	111081535	6.33333	.	.	intergenic	.	rs792045822	2
AC.GT	9	111196147	111196157	5.5	.	.	intergenic	0.116	rs790128306	2
AAAT.ATTT	9	111366217	111366240	6	.	.	intergenic	-0.057	.	2

AT.AT	9	111551267	111551284	9	.	.	intergenic	0.029	rs788081815	2
AAAAC.GTTTT	9	111603527	111603543	3.4	.	.	intergenic	0.134	rs789760632	2
AT.AT	9	111852487	111852502	8	.	.	intergenic	0.037	.	3
AC.GT	9	111993712	111993737	13	.	.	intergenic	0.012	rs790843149	3
AT.AT	9	112139058	112139069	6	.	.	intergenic	.	rs789095120	2
AC.GT	9	112331898	112331910	6.5	.	.	intergenic	0.035	970691 rs7880	2
AAAC.GTTT	9	112527926	112527949	6	.	.	intergenic	0.33	974366 rs7930	2
AT.AT	9	112618603	112618625	11.5	ENSSSCT00000023465	.	intron	-0.048	rs789069308	2
ATCC.GGAT	9	112629570	112629587	4.5	ENSSSCT00000023465	.	intron	0.026	591722 rs7913	2
AT.AT	9	112800453	112800472	10	ENSSSCT00000016785	PHTF2	intron	0.232	rs787593832	2
AT.AT	9	112934143	112934158	8	ENSSSCT00000016786	PTPN12	intron	.	.	2
AC.GT	9	112948564	112948578	7.5	ENSSSCT00000016786	PTPN12	intron	0.259	rs788364518	2
AAAAC.GTTTT	9	112984556	112984584	5.8	.	.	intergenic	.	rs793686386	2
ATC.GAT	9	113254398	113254409	4	ENSSSCT00000021410	U6	promoter	0.168	rs789410460	2
AAAAT.ATTTT	9	113279212	113279230	3.8	ENSSSCT00000016789	FAM185A	intron	0.796	rs790606571	2
AGG.CCT	9	113963908	113963919	4	ENSSSCT00000016801	DNAJC2	intron	-0.024	.	3
AAAAAC.GTTTTT	9	114042552	114042579	4.66667	.	.	intergenic	0.145	rs790447274	2
AT.AT	9	114139764	114139774	5.5	ENSSSCT00000016805	RELN	intron	0.55	.	3
AAAT.ATTT	9	114475763	114475777	3.75	.	.	intergenic	0.213	.	4
AAAAT.ATTTT	9	114484669	114484684	3.2	.	.	intergenic	0.201	rs786256585	2
AC.GT	9	114652719	114652731	6.5	.	.	intergenic	-0.022	rs709426894	2
AAC.GTT	9	114737886	114737919	11.3333	.	.	intergenic	.	rs788333118	2
AATG.CATT	9	114793222	114793251	7.5	.	.	intergenic	0.177	.	2
AACAC.GTGTT	9	114870383	114870398	3.2	ENSSSCT00000016806	ORC5	intron	-0.124	.	2
AT.AT	9	115084766	115084775	5	.	.	intergenic	0.234	482433 rs7887	3
AAAAAC.GTTTTT	9	115130564	115130592	4.83333	.	.	intergenic	0.261	rs788514809	2
AAC.GTT	9	115213371	115213386	5.33333	.	.	intergenic	0.18	rs788985894	2
AAAAT.ATTTT	9	115360703	115360720	3.6	.	.	intergenic	-0.009	507382 rs7931	2
AAC.GTT	9	115738369	115738396	9.33333	ENSSSCT00000032237	SRPK2	intron	.	rs788424450	2
AC.GT	9	115939535	115939548	7	.	.	intergenic	0.146	rs787128875	4
AT.AT	9	115954396	115954407	6	.	.	intergenic	.	.	4
AAAT.ATTT	9	116382157	116382174	4.5	ENSSSCT00000016812	ATXN7L1	intron	.	780155 rs7907	2
AGGG.CCCT	9	116611000	116611014	3.75	.	.	intergenic	-0.206	rs792500736	2
AC.GT	9	116627938	116627949	6	ENSSSCT00000022799	.	intron	-0.298	160218 rs7935	2
AC.GT	9	116731437	116731446	5	ENSSSCT00000016813	.	intron	-0.053	rs788102530	2
AAAAC.GTTTT	9	116761275	116761300	5.2	.	.	intergenic	-0.175	179530 rs7911	2
AAAT.ATTT	9	116785251	116785286	9	.	.	intergenic	.	rs788385082	2
AATCTC.GAGAT	9	116966742	116966763	3.66667	.	.	intergenic	.	023735 rs7894	2
AATT.AATT	9	117049078	117049101	6	.	.	intergenic	0.03	787444 rs7927	2
AAC.GTT	9	117065911	117065933	7.66667	.	.	intergenic	0.034	rs793702489	3
AC.GT	9	117075630	117075639	5	.	.	intergenic	-0.064	.	4
AAC.GTT	9	117280958	117280971	4.66667	.	.	intergenic	0.202	rs790192950	2
AG.CT	9	117293469	117293484	8	.	.	intergenic	-0.056	rs787374223	2
AC.GT	9	117361566	117361578	6.5	.	.	intergenic	0.156	rs793589464	4
AC.GT	9	117446409	117446419	5.5	.	.	intergenic	0.174	.	4
AAG.CTT	9	117761212	117761225	4.66667	.	.	intergenic	-0.077	.	3
AAG.CTT	9	118184124	118184135	4	.	.	intergenic	0.186	700367 rs7887	2
AC.GT	9	118273475	118273489	7.5	ENSSSCT00000031097	.	intron	0.116	rs790559662	2
ACAT.ATGT	9	118587904	118587918	3.75	.	.	intergenic	0.197	.	2
AAAAAG.CTTTTT	9	118639713	118639736	4	.	.	intergenic	0.039	rs791529141	2
AAAT.ATTT	9	118671864	118671881	4.5	.	.	intergenic	0.046	rs790752147	2
AG.CT	9	118672390	118672404	7.5	.	.	intergenic	0.07	.	2
AAC.GTT	9	118698801	118698824	8	.	.	intergenic	-0.114	rs790392335	5
AC.GT	9	118711950	118711963	7	.	.	intergenic	0.012	974415 rs7920	3
AAC.GTT	9	119125717	119125730	4.66667	ENSSSCT00000016830	NME8	intron	.	rs789641623	2
AC.GT	9	119399924	119399940	8.5	.	.	intergenic	0.123	.	3
AG.CT	9	119560200	119560217	9	ENSSSCT00000029694	.	intron	0.687	363699 rs7919	2
AG.CT	9	119560200	119560217	9	ENSSSCT00000029130	STARD3NL	intron	0.687	363699 rs7919	2
AAAC.GTTT	9	119756493	119756504	3	.	.	intergenic	-0.007	rs789934354	2
AAAG.CTTT	9	119931116	119931128	3.25	.	.	intergenic	.	rs789802949	2
AAAAAC.GTTTTT	9	120148551	120148573	3.83333	.	.	intergenic	.	448403 rs7919	2
AAAT.ATTT	9	120161309	120161325	4.25	ENSSSCT00000031790	ZNF425	intron	0.207	rs792769207	2
AAC.GTT	9	120188788	120188801	4.66667	.	.	intergenic	.	574570 rs7877	2
ACGC.GCGT	9	120210117	120210134	4.5	ENSSSCT00000016835	PDIA4	intron	-0.307	rs792631106	2
AT.AT	9	120362600	120362611	6	ENSSSCT00000023706	EZH2	intron	0.175	319465 rs7893	2
AAAAC.GTTTT	9	120428972	120429004	6.6	ENSSSCT00000016837	CUL1	intron	0.009	rs790429663	2
AT.AT	9	120482483	120482495	6.5	ENSSSCT00000031913	.	promoter	-0.073	rs788074391	3
AT.AT	9	120891798	120891808	5.5	ENSSSCT00000026150	CNTNAP2	intron	-0.124	rs793714400	2
AC.GT	9	120915591	120915615	12.5	.	.	intergenic	-0.087	rs793388199	2
AT.AT	9	120925061	120925085	12.5	.	.	intergenic	-0.031	.	2
AG.CT	9	120975017	120975030	7	.	.	intergenic	0.214	.	4
AC.GT	9	121114399	121114411	6.5	ENSSSCT00000016840	.	intron	0.019	rs788682422	2
AAAC.GTTT	9	121317206	121317226	5.25	.	.	intergenic	.	087137 rs7909	2
AG.CT	9	121337217	121337229	6.5	.	.	intergenic	0.611	926691 rs7894	2
AAAC.GTTT	9	121369068	121369095	7	.	.	intergenic	-0.068	rs793876919	2
AC.GT	9	121499774	121499792	9.5	.	.	intergenic	0.061	rs791322188	6
AAAT.ATTT	9	121519107	121519130	6	.	.	intergenic	-0.136	420364 rs7930	2
AC.GT	9	121533703	121533715	6.5	.	.	intergenic	-0.15	.	4
AAAC.GTTT	9	121713227	121713245	4.75	.	.	intergenic	-0.126	rs792803802	2
AAAG.CTTT	9	121756397	121756420	6	.	.	intergenic	-0.162	rs791985956	2
AT.AT	9	121785918	121785929	6	ENSSSCT00000024740	.	promoter	-0.102	rs788530476	2
AG.CT	9	121828819	121828837	9.5	.	.	intergenic	-0.131	rs787115525	3
AAG.CTT	9	121879046	121879077	10.6667	.	.	intergenic	.	rs788480384	2
AAAG.CTTT	9	121894402	121894425	6	.	.	intergenic	.	rs789279615	2

AC.GT	9	121898766	121898777	6	.	.	intergenic	0.015	906042 rs7899	4
AC.GT	9	121954245	121954261	8.5	ENSSSCT00000016843	.	promoter	-0.062	rs790176232	2
AAAC.GTTT	9	122169500	122169535	9	.	.	intergenic	-0.327	rs705930507	3
AG.CT	9	122191020	122191044	12.5	.	.	intergenic	-0.141	403164 rs7868	2
AG.CT	9	122282415	122282425	5.5	.	.	intergenic	0.119	.	3
AAAC.GTTT	9	122373987	122374006	5	.	.	intergenic	-0.293	rs789505205	2
AT.AT	9	122552454	122552467	7	.	.	intergenic	-0.403	555443 rs7911	3
AAAC.GTTT	9	122652997	122653018	5.5	.	.	intergenic	.	rs790526818	4
AG.CT	9	122676302	122676312	5.5	.	.	intergenic	-0.103	rs787609783	2
AG.CT	9	122834528	122834550	11.5	.	.	intergenic	-0.122	.	4
AG.CT	9	123050339	123050349	5.5	.	.	intergenic	-0.046	rs790663179	2
AAAG.CTTT	9	123073209	123073227	4.75	.	.	intergenic	.	rs788642985	2
AAAC.GTTT	9	123117940	123117982	10.75	.	.	intergenic	0	rs792722862	2
AG.CT	9	123279709	123279728	10	.	.	intergenic	-0.287	589647 rs7917	2
AAGG.CCTT	9	123298824	123298868	11.25	.	.	intergenic	-0.265	rs789178775	2
AAAC.GTTT	9	123517703	123517724	5.5	.	.	intergenic	0.291	rs789920018	2
AAAT.ATTT	9	123542879	123542904	6.5	.	.	intergenic	0.189	rs791610430	2
AT.AT	9	123553718	123553730	6.5	.	.	intergenic	0.174	537701 rs7928	2
AC.GT	9	123602640	123602650	5.5	.	.	intergenic	.	rs792840363	2
AAAC.GTTT	9	123695790	123695816	6.75	.	.	intergenic	-0.185	rs788386582	2
AT.AT	9	123746826	123746836	5.5	.	.	intergenic	-0.051	.	4
AC.GT	9	123971705	123971715	5.5	.	.	intergenic	-0.085	.	2
AAAC.GTTT	9	124222416	124222432	4.25	.	.	intergenic	0.285	rs790764761	2
AAAG.CTTT	9	124229324	124229340	4.25	.	.	intergenic	0.247	.	2
AT.AT	9	124248102	124248119	9	.	.	intergenic	0.1	rs790253409	2
AAC.GTT	9	124594839	124594852	4.66667	ENSSSCT00000016844	TPK1	intron	-0.119	783643 rs7922	2
AGG.CCT	9	124640323	124640334	4	.	.	intergenic	.	rs792939018	2
AC.GT	9	124823055	124823071	8.5	ENSSSCT00000022548	.	promoter	.	.	3
AAAAAC.GTTTTT	9	124872607	124872635	4.83333	.	.	intergenic	.	.	3
AAG.CTT	9	124876347	124876360	4.66667	.	.	intergenic	-0.564	rs789303023	2
AT.AT	9	124922610	124922619	5	.	.	intergenic	.	rs787799576	2
AAAG.CTTT	9	125043128	125043147	5	.	.	intergenic	.	rs790042659	2
AAAAC.GTTTT	9	125059094	125059118	5	.	.	intergenic	.	rs791523956	2
AG.CT	9	125100348	125100357	5	ENSSSCT00000016855	.	intron	.	rs792708238	2
AG.CT	9	125100348	125100357	5	ENSSSCT00000016854	.	intron	.	rs792708238	2
AAAC.GTTT	9	125346699	125346725	6.75	ENSSSCT00000016859	CHI3L1	intron	.	020699 rs7896	2
AAAC.GTTT	9	125346699	125346725	6.75	ENSSSCT00000036518	CHI3L1	intron	.	020699 rs7896	2
ATC.GAT	9	125365020	125365035	5.33333	.	.	intergenic	-0.003	rs791324870	2
AAAC.GTTT	9	125594569	125594585	4.25	.	.	intergenic	.	rs789946925	2
AAG.CTT	9	125623365	125623381	5.66667	ENSSSCT00000016865	VAMP4	intron	0.5	722354 rs7865	2
AAAC.GTTT	9	125688493	125688515	5.75	.	.	intergenic	-0.143	rs787056589	3
AG.CT	9	125849176	125849188	6.5	.	.	intergenic	-0.206	rs790623506	4
AC.GT	9	126082907	126082919	6.5	.	.	intergenic	.	rs791095952	2
AAAC.GTTT	9	126088146	126088167	5.5	.	.	intergenic	0.17	rs791585137	2
AAC.GTT	9	126209748	126209765	6	.	.	intergenic	0.885	rs694781134	3
AAAAC.GTTTT	9	126270451	126270479	5.8	ENSSSCT00000028491	.	intron	-0.028	rs788801237	2
AATG.CATT	9	126455471	126455489	4.75	.	.	intergenic	0.116	.	2
AAAC.GTTT	9	126581533	126581561	7.25	ENSSSCT00000016869	.	intron	0.153	987172 rs7907	2
AC.GT	9	126672946	126672956	5.5	.	.	intergenic	0.196	.	2
AAAT.ATTT	9	127104925	127104943	4.75	.	.	intergenic	.	rs792316973	2
AAAT.ATTT	9	127136038	127136052	3.75	.	.	intergenic	-0.23	rs787048313	2
AC.GT	9	127426556	127426581	13	.	.	intergenic	0.008	343482 rs7880	4
AAATT.AATTT	9	127480995	127481010	3.2	.	.	intergenic	.	rs789967989	3
AAAG.CTTT	9	127495618	127495629	3	ENSSSCT00000028213	PRDX6	intron	1.855	rs793088164	2
AAAC.GTTT	9	127517766	127517780	3.75	ENSSSCT00000016873	SLC9C2	intron	0.176	rs787564387	2
AG.CT	9	127624077	127624086	5	.	.	intergenic	0.18	.	2
AAAT.ATTT	9	127700478	127700501	6	ENSSSCT00000016882	KLHL20	intron	0.294	rs788240629	2
AG.CT	9	127757181	127757190	5	ENSSSCT00000016876	DARS2	intron	0.049	.	4
AAGT.ACTT	9	127895282	127895312	7.75	ENSSSCT00000016884	RC3H1	intron	.	939617 rs7922	2
AAAAG.CTTTT	9	127965187	127965210	4.8	.	.	intergenic	0.193	rs793540457	2
ACAG.CTGT	9	128032636	128032666	7.75	.	.	intergenic	0.055	.	4
ATCG.CGAT	9	128128125	128128145	5.25	ENSSSCT00000016886	.	intron	0.196	rs792938907	2
AAAAC.GTTTT	9	128406912	128406938	5.4	.	.	intergenic	0.095	rs790212073	2
AC.GT	9	128413324	128413342	9.5	.	.	intergenic	.	rs786432582	3
ACAT.ATGT	9	129172131	129172151	5.25	ENSSSCT00000016895	TNR	intron	0.059	.	2
AATAT.ATATT	9	129263767	129263783	3.4	.	.	intergenic	0.113	rs787490616	2
AAAC.GTTT	9	129406015	129406033	4.75	.	.	intergenic	-0.303	rs793558438	2
AAAAAC.GTTTTT	9	129533020	129533041	3.66667	.	.	intergenic	.	382912 rs7916	2
AATG.CATT	9	129589500	129589524	6.25	.	.	intergenic	0.08	338454 rs7912	3
AC.GT	9	129692484	129692508	12.5	ENSSSCT00000016896	.	intron	0.129	327965 rs7906	4
AAAC.GTTT	9	129761018	129761038	5.25	ENSSSCT00000016896	.	intron	0.252	rs788606098	2
AAAAG.CTTTT	9	129971946	129971969	4.8	.	.	intergenic	.	rs793507808	2
AC.GT	9	130141729	130141744	8	ENSSSCT00000016899	PAPPA2	intron	0.101	rs789643509	2
AT.AT	9	130214418	130214430	6.5	ENSSSCT00000016899	PAPPA2	intron	0.09	rs710085081	4
AT.AT	9	130569982	130570001	10	ENSSSCT00000026733	ASTN1	intron	-0.031	rs792936491	2
AT.AT	9	130594623	130594645	11.5	ENSSSCT00000026733	ASTN1	intron	-0.044	rs790363249	2
ATC.GAT	9	130618018	130618035	6	ENSSSCT00000026733	ASTN1	intron	0.01	rs791320565	2
AGG.CCT	9	130721585	130721601	5.66667	.	.	intergenic	-0.18	.	2
AG.CT	9	130787839	130787848	5	ENSSSCT00000016902	BRINP2	intron	.	rs788410792	2
AAAAT.ATTTT	9	130892330	130892346	3.4	.	.	intergenic	0.379	rs791896726	2
AAC.GTT	9	131092030	131092051	7.33333	.	.	intergenic	.	rs791290753	4
AAC.GTT	9	131390568	131390583	5.33333	.	.	intergenic	0.569	317671 rs7912	2
AGG.CCT	9	131503482	131503495	4.66667	ENSSSCT00000031105	.	intron	0.273	rs790986375	2
AC.GT	9	131662068	131662082	7.5	.	.	intergenic	.	rs793777266	2

AC.GT	9	131710606	131710617	6	ENSSSCT00000016906	RASAL2	intron	.	rs792433811	3
AC.GT	9	131710606	131710617	6	ENSSSCT00000032575	RASAL2	intron	.	rs792433811	3
AGGG.CCCT	9	131888400	131888413	3.5	ENSSSCT00000016906	RASAL2	intron	.	.	2
AGGG.CCCT	9	131888400	131888413	3.5	ENSSSCT00000032575	RASAL2	intron	.	.	2
AAAAT.ATTTT	9	131993180	131993200	4.2	ENSSSCT00000016906	RASAL2	intron	0.293	rs792281163	2
AAAAT.ATTTT	9	131993180	131993200	4.2	ENSSSCT00000032575	RASAL2	intron	0.293	rs792281163	2
AG.CT	9	132269341	132269357	8.5	ENSSSCT00000026955	RALGPS2	intron	.	rs694828370	5
AAAAT.ATTTT	9	132341503	132341528	5.2	ENSSSCT00000016910	.	intron	.	.	2
AAAC.GTTT	9	132376655	132376692	9.5	ENSSSCT00000016910	.	intron	.	rs787546904	2
AC.GT	9	132655792	132655804	6.5	ENSSSCT00000016913	TOR3A	intron	-0.296	rs793602355	2
AAAC.GTTT	9	132742961	132742972	3	ENSSSCT00000027500	ABL2	intron	0.064	rs788220721	2
AAAAC.GTTTT	9	132860030	132860049	4	ENSSSCT00000016916	AXDND1	intron	0.178	542617 rs7877	2
AG.CT	9	132950975	132950984	5	ENSSSCT00000023854	SOAT1	intron	0.248	.	2
AAG.CTT	9	133134405	133134417	4.33333	ENSSSCT00000016915	TDRD5	intron	.	381760 rs7928	3
AAG.CTT	9	133134405	133134417	4.33333	ENSSSCT00000028228	TDRD5	intron	.	381760 rs7928	3
AC.GT	9	133171397	133171410	7	.	.	intergenic	0.508	.	2
AAAAC.GTTTT	9	133311571	133311591	3.5	ENSSSCT00000030836	.	intron	0.412	rs788544348	2
AC.GT	9	133388792	133388801	5	ENSSSCT00000023749	.	intron	0.259	rs790727157	2
AAAC.GTTT	9	133493299	133493321	5.75	.	.	intergenic	.	132276 rs7903	2
AAAC.GTTT	9	133894693	133894716	6	ENSSSCT00000016923	ACBD6	intron	0.103	528200 rs7927	2
AC.GT	9	133911722	133911733	6	ENSSSCT00000016923	ACBD6	intron	0.445	rs791759072	4
AT.AT	9	134030527	134030538	6	.	.	intergenic	.	.	3
AAAT.ATTT	9	134062558	134062578	5.25	.	.	intergenic	0.095	rs789011461	2
AC.GT	9	134122745	134122757	6.5	.	.	intergenic	.	.	3
AAAAC.GTTTT	9	134321229	134321252	4.8	ENSSSCT00000016926	XPR1	intron	.	480824 rs7938	2
AAAC.GTTT	9	134667117	134667135	4.75	.	.	intergenic	0.128	rs791720802	2
AAAC.GTTT	9	134809426	134809440	3.75	.	.	intergenic	0.034	530741 rs7873	2
AC.GT	9	134826512	134826522	5.5	.	.	intergenic	0.131	rs693101499	2
AAC.GTT	9	134918748	134918763	5.33333	.	.	intergenic	0.159	rs791230030	2
AG.CT	9	135129001	135129014	7	ENSSSCT00000016932	CACNA1E	intron	-0.06	rs789282233	2
AG.CT	9	135157218	135157233	8	ENSSSCT00000016932	CACNA1E	intron	0.06	rs704600815	2
AAAAC.GTTTT	9	135168156	135168180	5	ENSSSCT00000016932	CACNA1E	intron	.	rs705395785	2
AC.GT	9	135202849	135202885	18.5	ENSSSCT00000016932	CACNA1E	intron	0.274	rs788260281	3
AC.GT	9	135301342	135301354	6.5	ENSSSCT00000016932	CACNA1E	intron	0.004	rs789862207	2
AC.GT	9	135325888	135325900	6.5	ENSSSCT00000016932	CACNA1E	intron	0.074	969386 rs7937	3
AAAAC.GTTTT	9	135467507	135467524	3.6	ENSSSCT00000016932	CACNA1E	intron	-0.199	rs790689267	2
ACACT.AGTGT	9	135590696	135590713	3.6	.	.	intergenic	-0.095	912195 rs7073	2
AAAT.ATTT	9	135650736	135650758	5.75	.	.	intergenic	-0.155	rs789863966	2
AAAT.ATTT	9	135662500	135662522	5.75	.	.	intergenic	0.225	232079 rs7874	2
AAG.CTT	9	135727976	135727989	4.66667	.	.	intergenic	0.181	rs790799410	2
AT.AT	9	135744386	135744400	7.5	.	.	intergenic	0.024	rs792866142	2
AG.CT	9	135789979	135789990	6	.	.	intergenic	-0.016	rs787274031	3
AAAAC.GTTTT	9	136644996	136645022	4.5	ENSSSCT00000016945	LAMC1	intron	0.449	rs790902219	2
AG.CT	9	136761963	136761978	8	.	.	intergenic	0.025	.	2
AC.GT	9	136868007	136868017	5.5	ENSSSCT00000016947	NMNAT2	intron	0.013	.	5
AAC.GTT	9	136876862	136876874	4.33333	ENSSSCT00000016947	NMNAT2	intron	-0.104	rs793473525	2
AG.CT	9	136928987	136928998	6	ENSSSCT00000016947	NMNAT2	intron	0.06	.	3
AC.GT	9	136970300	136970312	6.5	.	.	intergenic	0.439	rs787185843	3
AAAC.GTTT	9	137390700	137390714	3.75	ENSSSCT00000016953	RGL1	intron	.	436445 rs7915	2
AG.CT	9	137545115	137545127	6.5	ENSSSCT00000030440	COLGALT2	intron	-0.006	750203 rs7917	2
AT.AT	9	137591967	137591978	6	ENSSSCT00000030440	COLGALT2	intron	0.193	rs786864030	2
ATCC.GGAT	9	137665189	137665204	4	.	.	intergenic	0.045	rs787112246	2
AG.CT	9	137709043	137709057	7.5	.	.	intergenic	-0.056	387804 rs7920	2
AG.CT	9	137796877	137796887	5.5	.	.	intergenic	0.074	rs793692290	2
AAC.GTT	9	138086258	138086272	5	ENSSSCT00000016955	C1orf21	intron	0.062	rs791442412	3
AGGGC.GCCCT	9	138150918	138150932	3	ENSSSCT00000016955	C1orf21	intron	0.133	.	2
AC.GT	9	138178014	138178024	5.5	ENSSSCT00000016955	C1orf21	intron	0.036	rs790664818	2
AC.GT	9	138433510	138433531	11	.	.	intergenic	0.077	.	2
AAAC.GTTT	9	138594675	138594694	5	ENSSSCT00000035845	FAM129A	intron	0.045	rs789732288	2
AAAC.GTTT	9	138594675	138594694	5	ENSSSCT00000032989	FAM129A	intron	0.045	rs789732288	2
AAAC.GTTT	9	138594675	138594694	5	ENSSSCT00000016958	FAM129A	intron	0.045	rs789732288	2
AAAC.GTTT	9	138594675	138594694	5	ENSSSCT00000036674	FAM129A	intron	0.045	rs789732288	2
AAAC.GTTT	9	138594675	138594694	5	ENSSSCT00000034931	FAM129A	intron	0.045	rs789732288	2
AT.AT	9	138598829	138598840	6	ENSSSCT00000035845	FAM129A	intron	0.407	.	2
AT.AT	9	138598829	138598840	6	ENSSSCT00000032989	FAM129A	intron	0.407	.	2
AT.AT	9	138598829	138598840	6	ENSSSCT00000016958	FAM129A	intron	0.407	.	2
AT.AT	9	138598829	138598840	6	ENSSSCT00000036674	FAM129A	intron	0.407	.	2
AT.AT	9	138598829	138598840	6	ENSSSCT00000034931	FAM129A	intron	0.407	.	2
AAT.ATT	9	138606455	138606471	5.66667	ENSSSCT00000035845	FAM129A	intron	0.346	.	2
AAT.ATT	9	138606455	138606471	5.66667	ENSSSCT00000032989	FAM129A	intron	0.346	.	2
AAT.ATT	9	138606455	138606471	5.66667	ENSSSCT00000016958	FAM129A	intron	0.346	.	2
AAT.ATT	9	138606455	138606471	5.66667	ENSSSCT00000036674	FAM129A	intron	0.346	.	2
AAT.ATT	9	138606455	138606471	5.66667	ENSSSCT00000034931	FAM129A	intron	0.346	.	2
AAAC.GTTT	9	138623533	138623556	6	.	.	intergenic	.	rs786768326	2
AAT.ATT	9	139089627	139089638	4	.	.	intergenic	.	.	2
AG.CT	9	139139633	139139653	10.5	.	.	intergenic	0.022	541030 rs7887	2
AG.CT	9	139186960	139186977	9	.	.	intergenic	.	rs791180569	2
AAAT.ATTT	9	139304512	139304534	5.75	.	.	intergenic	0.041	363086 rs7894	2
AAAT.ATTT	9	139379329	139379343	3.75	.	.	intergenic	0.091	rs788526101	2
AT.AT	9	139738315	139738334	10	.	.	intergenic	0.032	.	2
AAC.GTT	9	139834025	139834041	5.66667	.	.	intergenic	0.022	rs792592227	3
AAT.ATT	9	139964785	139964800	5.33333	ENSSSCT00000016969	TPR	intron	-0.003	rs791991735	2
AAG.CTT	9	139975191	139975203	4.33333	ENSSSCT00000016969	TPR	intron	-0.128	977169 rs7892	2
AC.GT	9	139977728	139977741	7	ENSSSCT00000016969	TPR	intron	-0.293	.	2

AT.AT	9	140078471	140078483	6.5	.	.	intergenic	.	rs792594960	4
AAAT.ATTT	9	140136456	140136475	5	.	.	intergenic	.	rs791443033	2
AAAAG.CTTTT	9	140416032	140416050	3.8	.	.	intergenic	-0.029	942485 rs7907	2
AC.GT	9	140484248	140484257	5	ENSSSCT00000022784	PLA2G4A	intron	.	rs789244680	3
AAAT.ATTT	9	140487104	140487119	4	ENSSSCT00000022784	PLA2G4A	intron	-0.082	rs788301503	2
AAAAC.GTTTT	9	140551232	140551255	4.8	ENSSSCT00000022784	PLA2G4A	intron	-0.342	rs786208974	2
AAT.ATT	9	140559997	140560010	4.66667	ENSSSCT00000022784	PLA2G4A	intron	0.003	rs792570717	2
AAAAAC.GTTTTT	9	140642771	140642793	3.83333	.	.	intergenic	-0.178	.	2
AAAC.GTTT	9	140786929	140786951	5.75	.	.	intergenic	.	rs787325721	3
AC.GT	9	141140559	141140585	13.5	ENSSSCT00000031583	KCNK2	intron	-0.015	rs705423429	5
AAAC.GTTT	9	141140706	141140719	3.5	ENSSSCT00000031583	KCNK2	intron	-0.024	rs787446109	2
AT.AT	9	141434236	141434245	5	.	.	intergenic	.	rs786589715	2
AAAAAT.ATTTTT	9	141478335	141478356	3.66667	.	.	intergenic	-0.12	rs788929234	2
AAAT.ATTT	9	141497858	141497870	3.25	.	.	intergenic	0.159	.	2
AAAT.ATTT	9	141607835	141607852	4.5	.	.	intergenic	-0.098	rs793537795	2
AAAT.ATTT	9	141614355	141614377	5.75	.	.	intergenic	.	116220 rs7898	2
AAG.CTT	9	141626848	141626862	5	.	.	intergenic	-0.016	.	2
AAGAG.CTCTT	9	141761125	141761141	3.4	.	.	intergenic	0.033	.	2
AC.GT	9	141788155	141788175	10.5	.	.	intergenic	-0.022	rs790578766	2
AAC.GTT	9	141869029	141869044	5.33333	.	.	intergenic	-0.075	rs791060592	2
AAC.GTT	9	142185454	142185477	8	ENSSSCT00000016976	SMYD2	intron	-0.14	709583 rs7900	2
AAC.GTT	9	142292276	142292294	6.33333	.	.	intergenic	-0.008	rs787171927	2
GAGGG.CCCTC	9	142539244	142539268	4.16667	.	.	intergenic	.	rs791639471	2
AC.GT	9	143080322	143080338	8.5	ENSSSCT00000016978	.	promoter	0.385	.	2
AAC.GTT	9	143144279	143144293	5	.	.	intergenic	0.445	rs787383243	2
AT.AT	9	143327715	143327728	7	ENSSSCT00000016980	.	intron	0.401	rs792811327	2
AAAT.ATTT	9	143396161	143396174	3.5	ENSSSCT00000016981	ANGEL2	intron	1.513	rs786882826	2
AG.CT	9	143551153	143551165	6.5	ENSSSCT00000016986	NSL1	intron	0.163	.	3
AG.CT	9	143551153	143551165	6.5	ENSSSCT00000016985	TATDN3	promoter	0.163	.	3
AAAC.GTTT	9	143566189	143566220	8	ENSSSCT00000016986	NSL1	intron	0.275	979706 rs7925	2
AAAAAC.GTTTTT	9	143756750	143756772	3.83333	.	.	intergenic	-0.182	rs788744350	2
AC.GT	9	143776206	143776215	5	.	.	intergenic	.	rs786796886	2
AC.GT	9	143881885	143881903	9.5	.	.	intergenic	-0.012	.	2
AG.CT	9	143991835	143991849	7.5	.	.	intergenic	-0.07	392084 rs7901	2
AAC.GTT	9	144008082	144008094	4.33333	ENSSSCT00000016990	NENF	promoter	-0.033	rs791697404	2
AAC.GTT	9	144008082	144008094	4.33333	ENSSSCT00000024915	NENF	promoter	-0.033	rs791697404	2
AC.GT	9	144019920	144019935	8	ENSSSCT00000016991	TMEM206	intron	0.443	rs786631961	4
AAAC.GTTT	9	144074551	144074574	6	ENSSSCT00000016992	PPP2R5A	intron	0.254	094943 rs7889	2
AAT.ATT	9	144101349	144101362	4.66667	ENSSSCT00000016992	PPP2R5A	intron	.	rs793203983	2
AAGG.CCTT	9	144243921	144243935	3.75	.	.	intergenic	-0.072	rs792749633	2
AC.GT	9	144300481	144300490	5	ENSSSCT00000016993	DTL	intron	0.732	rs793467476	3
AC.GT	9	144436451	144436465	7.5	.	.	intergenic	0.129	rs791491585	6
AAAG.CTTT	9	144454541	144454552	3	.	.	intergenic	0.047	.	2
AAAC.GTTT	9	144479645	144479659	3.75	ENSSSCT00000016996	LPGAT1	intron	.	235677 rs7877	2
AT.AT	9	144496102	144496113	6	ENSSSCT00000016996	LPGAT1	intron	0.315	112013 rs7912	2
AAAC.GTTT	9	144596844	144596858	3.75	ENSSSCT00000016997	NEK2	intron	-0.097	037492 rs7905	2
AAAC.GTTT	9	144723285	144723309	6.25	.	.	intergenic	0.138	rs791698863	2
AAAG.CTTT	9	144795588	144795607	5	.	.	intergenic	0.184	rs792512992	2
AAAAC.GTTTT	9	144993132	144993146	3	.	.	intergenic	0.21	386668 rs7885	2
AAC.GTT	9	145018231	145018246	5.33333	.	.	intergenic	0.042	943808 rs7871	2
AAAC.GTTT	9	145188651	145188672	5.5	.	.	intergenic	.	rs791276127	2
AAGG.CCTT	9	145210878	145210893	4	.	.	intergenic	0.082	067600 rs7937	2
AG.CT	9	145313395	145313407	6.5	.	.	intergenic	0.248	300509 rs7920	2
AATT.AATT	9	145350562	145350578	4.25	.	.	intergenic	.	029704 rs7912	2
AG.CT	9	145389748	145389758	5.5	ENSSSCT00000017001	HHAT	intron	-0.355	.	2
AC.GT	9	145456134	145456148	7.5	ENSSSCT00000017001	HHAT	intron	0.066	539024 rs7910	4
AT.AT	9	145584547	145584560	7	ENSSSCT00000017001	HHAT	intron	-0.322	rs789032404	2
AAAT.ATTT	9	145728870	145728884	3.75	.	.	intergenic	.	017791 rs7935	2
AAC.GTT	9	145820187	145820203	5.66667	.	.	intergenic	.	rs788209539	3
AAC.GTT	9	145836630	145836642	4.33333	.	.	intergenic	.	rs789585165	4
AAT.ATT	9	145858452	145858466	5	ENSSSCT00000017004	SYT14	intron	1.179	rs793383000	2
AAAAC.GTTTT	9	145982081	145982098	3.6	ENSSSCT00000017004	SYT14	intron	.	rs791800001	2
AACT.AGTT	9	146224869	146224886	4.5	.	.	intergenic	0.057	014875 rs7918	2
AAGG.CCTT	9	146230832	146230857	6.5	.	.	intergenic	0.102	rs788503885	2
AC.GT	9	146287564	146287583	10	.	.	intergenic	.	rs792309976	2
AAC.GTT	9	146496495	146496513	6.33333	.	.	intergenic	0.04	rs786521685	2
AC.GT	9	146496901	146496913	6.5	.	.	intergenic	0.014	rs791432331	2
AAAT.ATTT	9	146743122	146743136	3.75	.	.	intergenic	-0.011	.	3
AC.GT	9	146947497	146947515	9.5	.	.	intergenic	0.023	722556 rs7890	2
AC.GT	9	147020795	147020807	6.5	.	.	intergenic	.	rs792578160	2
AAAAAC.GTTTTT	9	147076990	147077018	4.83333	.	.	intergenic	-0.058	511048 rs7878	2
AG.CT	9	147093646	147093673	14	.	.	intergenic	.	.	2
AT.AT	9	147123143	147123154	6	.	.	intergenic	.	396089 rs7885	2
AAAC.GTTT	9	147132552	147132584	8.25	.	.	intergenic	-0.075	573832 rs7893	2
AC.GT	9	147142866	147142880	7.5	.	.	intergenic	-0.022	rs787458208	2
AC.GT	9	147407154	147407163	5	.	.	intergenic	.	rs791367130	2
AAAAC.GTTTT	9	147428765	147428782	3.6	.	.	intergenic	.	448635 rs7893	2
AC.GT	9	147581472	147581483	6	.	.	intergenic	-0.072	719520 rs7906	2
AT.AT	9	148364489	148364499	5.5	.	.	intergenic	-0.218	rs792443462	2
AG.CT	9	148412162	148412172	5.5	.	.	intergenic	.	rs787523203	2
AC.GT	9	148551534	148551548	7.5	ENSSSCT00000020629	sc-mir-29b-	promoter	0.01	.	2
AC.GT	9	148551534	148551548	7.5	ENSSSCT00000021258	ssc-mir-29c	promoter	0.01	.	2
AAAC.GTTT	9	148858681	148858703	5.75	ENSSSCT00000031404	ABCA13	intron	.	rs793245285	2
AAAAAT.ATTTTT	9	148863486	148863507	3.66667	ENSSSCT00000031404	ABCA13	intron	-0.318	.	2

AC.GT	9	148897728	148897738	5.5	ENSSSCT00000031404	ABCA13	intron	-0.303	rs787117636	2
AT.AT	9	148900571	148900580	5	ENSSSCT00000031404	ABCA13	intron	0.419	rs788650583	2
AAAAC.GTTTT	9	148967931	148967954	4.8	.	.	intergenic	0.035	rs792411458	2
AG.CT	9	148992024	148992050	13.5	.	.	intergenic	-0.305	rs786535082	2
AAC.GTT	9	149022438	149022454	5.66667	.	.	intergenic	-0.244	rs788033088	3
AT.AT	9	149062857	149062866	5	.	.	intergenic	0.022	.	2
AT.AT	9	149216873	149216884	6	.	.	intergenic	.	.	3
AAC.GTT	9	149313360	149313371	4	.	.	intergenic	.	rs789046986	2
AAAC.GTTT	9	149480497	149480516	5	.	.	intergenic	.	rs787686379	2
AAAT.ATTT	9	149879385	149879402	4.5	.	.	intergenic	.	rs788095348	2
AC.GT	9	150286532	150286541	5	ENSSSCT00000031836	GRB10	intron	-0.026	rs791775782	2
AC.GT	9	150286532	150286541	5	ENSSSCT00000017025	GRB10	intron	-0.026	rs791775782	2
AC.GT	9	150766463	150766473	5.5	.	.	intergenic	.	rs786567765	2
ATAT.ATAT	9	151014667	151014697	7.75	.	.	intergenic	.	24701 rs7884	4
AAAC.GTTT	9	151367447	151367467	5.25	.	.	intergenic	.	.	2
AC.GT	9	151418328	151418344	8.5	.	.	intergenic	-0.178	rs788934233	3
AT.AT	9	151428155	151428166	6	.	.	intergenic	-0.283	14226 rs7872	2
AAAG.CTTT	9	151699754	151699768	3.75	.	.	intergenic	-0.295	92608 rs7881	2
AC.GT	9	151756977	151756992	8	.	.	intergenic	.	.	2
AG.CT	9	151886869	151886878	5	.	.	intergenic	-0.144	93196 rs7064	3
AC.GT	9	151914967	151914978	6	.	.	intergenic	.	23057 rs7015	2
AAAC.GTTT	9	151926566	151926583	4.5	.	.	intergenic	-0.113	rs788495217	2
AC.GT	9	152161133	152161157	12.5	.	.	intergenic	.	rs786980826	2
AAAC.GTTT	9	152161969	152161984	4	.	.	intergenic	.	.	3
AC.GT	9	152235226	152235238	6.5	.	.	intergenic	.	.	2
AAAAG.CTTTT	9	152281102	152281123	4.4	.	.	intergenic	-0.066	08330 rs7937	2
AAAC.GTTT	9	152429412	152429426	3.75	.	.	intergenic	.	98566 rs7906	4
AG.CT	9	152434883	152434906	12	.	.	intergenic	-0.307	84027 rs7936	2
AAT.ATT	9	152561961	152561986	8.66667	.	.	intergenic	-0.434	rs789942763	2
AT.AT	9	152614199	152614210	6	.	.	intergenic	.	.	2
AAAC.GTTT	9	152834598	152834616	4.75	.	.	intergenic	-0.057	rs789968655	2
AC.GT	9	152990072	152990084	6.5	.	.	intergenic	0.183	33918 rs7896	2
AC.GT	9	152997985	152997997	6.5	.	.	intergenic	0.108	rs793708017	2
AG.CT	9	153063490	153063505	8	.	.	intergenic	.	rs789055778	2
AC.GT	X	444037	444070	17	.	.	intergenic	-0.46	29075 rs7914	4
AC.GT	X	819613	819625	6.5	.	.	intergenic	-0.066	108129 rs7917	2
AC.GT	X	1129513	1129525	6.5	.	.	intergenic	.	rs789524002	3
AAAC.GTTT	X	1289334	1289350	4.25	.	.	intergenic	-0.169	41192 rs7936	2
AAG.CTT	X	1467641	1467652	4	.	.	intergenic	.	rs787427320	3
AAAAT.ATTTT	X	1522406	1522424	3.8	.	.	intergenic	.	rs787500245	2
AAT.ATT	X	1541856	1541879	8	.	.	intergenic	-0.421	rs793083766	2
ACCT.AGGT	X	1639259	1639281	5.75	.	.	intergenic	.	rs786272548	2
AAAGG.CCTTT	X	1685405	1685428	4.8	.	.	intergenic	-0.534	rs786842340	3
AT.AT	X	1704171	1704180	5	.	.	intergenic	.	rs709120291	2
AAAT.ATTT	X	1876058	1876086	7.25	.	.	intergenic	.	rs790687663	2
AAAG.CTTT	X	2210963	2210983	5.25	.	.	intergenic	0.09	rs791672665	2
AC.GT	X	2257515	2257525	5.5	.	.	intergenic	-0.002	rs786587327	2
AG.CT	X	2351534	2351546	6.5	.	.	intergenic	.	rs786812803	2
AC.GT	X	2362792	2362801	5	.	.	intergenic	.	rs790758536	2
AAAC.GTTT	X	2736242	2736269	7	.	.	intergenic	.	.	2
AT.AT	X	2796922	2796942	10.5	ENSSSCT00000013231	NLGN4X	intron	-0.292	rs788940848	2
AACCC.GGGTT	X	2802844	2802859	3.2	ENSSSCT00000013231	NLGN4X	intron	.	rs790303695	2
AT.AT	X	2826775	2826784	5	ENSSSCT00000013231	NLGN4X	intron	-0.282	rs786925596	3
AAC.GTT	X	2912015	2912027	4.33333	ENSSSCT00000013231	NLGN4X	intron	.	rs791744762	2
AAAAC.GTTTT	X	3058293	3058315	3.83333	ENSSSCT00000013231	NLGN4X	intron	.	rs790392878	2
AAAC.GTTT	X	3164803	3164821	4.75	ENSSSCT00000013231	NLGN4X	intron	.	rs791208215	2
AT.AT	X	3386575	3386585	5.5	.	.	intergenic	-0.113	rs703409209	3
AC.GT	X	3463312	3463326	7.5	.	.	intergenic	.	rs790478183	7
AAAT.ATTT	X	3512867	3512881	3.75	.	.	intergenic	.	27427 rs7896	2
AC.GT	X	3747742	3747752	5.5	.	.	intergenic	0.07	.	3
AACC.GGTT	X	4116492	4116506	3.75	ENSSSCT00000032160	STS	intron	.	rs792136715	2
AC.GT	X	4202205	4202219	7.5	ENSSSCT00000035627	STS	intron	.	rs786933849	2
AC.GT	X	4202205	4202219	7.5	ENSSSCT00000032160	STS	intron	.	rs786933849	2
AT.AT	X	4231696	4231705	5	ENSSSCT00000035627	STS	intron	.	66110 rs7898	2
AT.AT	X	4231696	4231705	5	ENSSSCT00000032160	STS	intron	.	66110 rs7898	2
AAAT.ATTT	X	4445274	4445288	3.75	.	.	intergenic	.	rs789908377	2
AAC.GTT	X	4447363	4447385	7.66667	.	.	intergenic	-0.291	rs788770945	2
AAAC.GTTT	X	4487649	4487664	4	.	.	intergenic	-0.526	40671 rs7933	2
AC.GT	X	4704811	4704822	6	.	.	intergenic	-0.256	rs789499434	2
AG.CT	X	4770462	4770478	8.5	.	.	intergenic	-0.214	rs709407472	2
AC.GT	X	4929889	4929900	6	ENSSSCT00000034295	242-236E1	intron	-0.111	rs789409316	3
AC.GT	X	4929889	4929900	6	ENSSSCT00000035815	242-236E1	intron	-0.111	rs789409316	3
AT.AT	X	5244346	5244361	8	ENSSSCT00000035815	242-236E1	intron	0.084	90917 rs6974	4
AC.GT	X	5470234	5470248	7.5	ENSSSCT00000035619	KAL1	intron	0.052	rs792609471	3
AC.GT	X	5470234	5470248	7.5	ENSSSCT00000013235	KAL1	intron	0.052	rs792609471	3
AG.CT	X	5534751	5534762	6	ENSSSCT00000035619	KAL1	intron	.	36011 rs7882	2
AG.CT	X	5534751	5534762	6	ENSSSCT00000013235	KAL1	intron	.	36011 rs7882	2
ACAG.CTGT	X	5552046	5552061	4	ENSSSCT00000035619	KAL1	intron	-0.256	rs791173408	2
ACAG.CTGT	X	5552046	5552061	4	ENSSSCT00000013235	KAL1	intron	-0.256	rs791173408	2
AAAC.GTTT	X	5624170	5624191	5.5	.	.	intergenic	-0.117	rs786376842	2
AC.GT	X	5716801	5716815	7.5	.	.	intergenic	-0.018	.	2
AAAT.ATTT	X	5839225	5839247	5.75	.	.	intergenic	.	rs789577959	4
AAAAC.GTTTT	X	5868146	5868174	5.8	.	.	intergenic	0.065	13969 rs7922	2
AAAC.GTTT	X	5873883	5873905	5.75	.	.	intergenic	0.095	84605 rs7921	2

AC.GT	X	6413272	6413283	6	.	.	intergenic	-0.135	rs786732548	2
AC.GT	X	7054349	7054359	5.5	.	.	intergenic	.	rs787964763	2
AC.GT	X	7351388	7351402	7.5	ENSSSCT00000033365	CLCN4	intron	-0.058	rs791752577	2
AC.GT	X	7351388	7351402	7.5	ENSSSCT00000035935	CLCN4	intron	-0.058	rs791752577	2
AAC.GTT	X	7413661	7413676	5.33333	ENSSSCT00000022263	CLCN4	intron	-0.229	rs787765686	2
AAC.GTT	X	7413661	7413676	5.33333	ENSSSCT00000035935	CLCN4	intron	-0.229	rs787765686	2
AAAC.GTTT	X	8312285	8312312	7	.	.	intergenic	0.07	rs789020935	2
ACAT.ATGT	X	8434275	8434291	4.25	.	.	intergenic	.	.	2
AAG.CTT	X	8473400	8473411	4	ENSSSCT00000035835	ARHGAP6	3'utr	1.552	rs791195052	2
AAG.CTT	X	8473400	8473411	4	ENSSSCT00000013247	ARHGAP6	3'utr	1.552	rs791195052	2
AAAAT.ATTTT	X	8867390	8867409	4	ENSSSCT00000033647	ARHGAP6	intron	.	rs786820460	2
AT.AT	X	8882856	8882867	6	ENSSSCT00000033647	ARHGAP6	intron	0.19	.	2
AC.GT	X	9272068	9272080	6.5	.	.	intergenic	.	rs786647653	2
AAC.GTT	X	9470380	9470396	5.66667	.	.	intergenic	0.243	643119 rs7865	2
AAAG.CTTT	X	9996528	9996544	4.25	.	.	intergenic	-0.349	rs786301018	2
AAAC.GTTT	X	10064586	10064600	3.75	.	.	intergenic	0.174	rs793795236	2
AG.CT	X	10158556	10158577	11	.	.	intergenic	.	732419 rs7922	3
AAC.GTT	X	10219237	10219259	7.66667	.	.	intergenic	0.086	rs789657942	2
AT.AT	X	10331375	10331392	9	ENSSSCT00000034697	FRMPD4	intron	0.076	rs791476539	2
AAAAC.GTTTT	X	10538650	10538668	3.8	.	.	intergenic	0.094	rs789328627	2
AAAAAC.GTTTTT	X	10718558	10718581	4	.	.	intergenic	-0.021	rs786546504	2
AAT.ATT	X	10998450	10998469	6.66667	ENSSSCT00000035913	EGFL6	intron	0.117	rs791838738	2
AAAAC.GTTTT	X	11168688	11168717	6	ENSSSCT00000035913	EGFL6	intron	0.129	rs788768559	2
AAAC.GTTT	X	11609926	11609949	6	ENSSSCT00000013266	GEMIN8	intron	0.199	390522 rs7893	2
AAAC.GTTT	X	11609926	11609949	6	ENSSSCT00000033507	GEMIN8	intron	0.199	390522 rs7893	2
AG.CT	X	11635521	11635533	6.5	.	.	intergenic	0.486	rs788300475	2
AT.AT	X	11715821	11715831	5.5	.	.	intergenic	-0.021	rs793590804	2
AAAAAC.GTTTTT	X	11803988	11804013	4.33333	.	.	intergenic	0.4	974458 rs7935	2
AAAC.GTTT	X	11826470	11826489	5	.	.	intergenic	0.421	rs786596315	2
AG.CT	X	11888005	11888015	5.5	.	.	intergenic	0.14	.	3
AAAC.GTTT	X	12069720	12069740	5.25	.	.	intergenic	.	rs786507618	2
AAAC.GTTT	X	12154582	12154594	3.25	ENSSSCT00000033189	GLRA2	intron	0.134	rs788252665	2
AAAC.GTTT	X	12154582	12154594	3.25	ENSSSCT00000013268	GLRA2	intron	0.134	rs788252665	2
AAAT.ATTT	X	12294886	12294905	5	.	.	intergenic	0.236	646600 rs7888	2
AAAAT.ATTTT	X	12332588	12332610	4.6	.	.	intergenic	.	195322 rs7893	2
AAAC.GTTT	X	12369150	12369165	4	ENSSSCT00000036081	MOSPD2	intron	0.317	963822 rs7909	2
AAAC.GTTT	X	12369150	12369165	4	ENSSSCT00000013270	MOSPD2	intron	0.317	963822 rs7909	2
AAAC.GTTT	X	12369150	12369165	4	ENSSSCT00000033995	MOSPD2	intron	0.317	963822 rs7909	2
AAAC.GTTT	X	13009154	13009174	5.25	ENSSSCT00000032788	BMX	intron	0.152	rs787247646	2
AAAC.GTTT	X	13009154	13009174	5.25	ENSSSCT00000013276	BMX	intron	0.152	rs787247646	2
AAAC.GTTT	X	13009154	13009174	5.25	ENSSSCT00000032899	BMX	promoter	0.152	rs787247646	2
AC.GT	X	13067229	13067239	5.5	ENSSSCT00000013277	ACE2	intron	0.13	rs788810847	2
AC.GT	X	13067229	13067239	5.5	ENSSSCT00000034032	ACE2	intron	0.13	rs788810847	2
AC.GT	X	13067229	13067239	5.5	ENSSSCT00000034745	ACE2	promoter	0.13	rs788810847	2
AC.GT	X	13088154	13088169	8	ENSSSCT00000013277	ACE2	intron	.	rs792419619	3
AAAAG.CTTTT	X	13705813	13705833	4.2	.	.	intergenic	0.369	rs788335952	2
AC.GT	X	13847579	13847598	10	.	.	intergenic	.	rs789287321	2
AAG.CTT	X	14019281	14019294	4.66667	ENSSSCT00000013285	CTPS2	intron	0.21	rs790732654	2
AG.CT	X	14391053	14391067	7.5	ENSSSCT00000033396	REPS2	intron	.	.	4
AG.CT	X	14391053	14391067	7.5	ENSSSCT00000026314	REPS2	intron	.	.	4
AG.CT	X	14391053	14391067	7.5	ENSSSCT00000034223	REPS2	intron	.	.	4
AG.CT	X	14391053	14391067	7.5	ENSSSCT00000013289	REPS2	intron	.	.	4
AG.CT	X	14765960	14765971	6	.	.	intergenic	0.503	rs793769259	2
AC.GT	X	14873910	14873929	10	.	.	intergenic	0.269	rs792226537	5
AATG.CATT	X	15225740	15225764	6.25	ENSSSCT00000033095	NHS	intron	0.195	rs788102735	2
AATG.CATT	X	15225740	15225764	6.25	ENSSSCT00000032786	NHS	intron	0.195	rs788102735	2
AAAAAC.GTTTTT	X	15438238	15438260	3.83333	.	.	intergenic	.	rs791443818	2
AAT.ATT	X	16000228	16000258	10.3333	.	.	intergenic	.	rs791824693	3
AAAC.GTTT	X	16231586	16231606	5.25	ENSSSCT00000032659	CDKL5	intron	0.358	rs792529452	2
AAAC.GTTT	X	16231586	16231606	5.25	ENSSSCT00000033642	CDKL5	intron	0.358	rs792529452	2
AAAC.GTTT	X	16231586	16231606	5.25	ENSSSCT00000013297	CDKL5	intron	0.358	rs792529452	2
AAAC.GTTT	X	16350441	16350459	4.75	ENSSSCT00000032792	PPEF1	intron	0.39	rs791763448	2
AAAC.GTTT	X	16350441	16350459	4.75	ENSSSCT00000035020	PPEF1	intron	0.39	rs791763448	2
AAAC.GTTT	X	16350441	16350459	4.75	ENSSSCT00000034554	PPEF1	intron	0.39	rs791763448	2
AAG.CTT	X	16400734	16400750	5.66667	ENSSSCT00000031758	PPEF1	intron	0.31	rs792324460	2
AAG.CTT	X	16400734	16400750	5.66667	ENSSSCT00000035020	PPEF1	intron	0.31	rs792324460	2
AAG.CTT	X	16400734	16400750	5.66667	ENSSSCT00000034554	PPEF1	intron	0.31	rs792324460	2
AC.GT	X	16692006	16692016	5.5	.	.	intergenic	.	.	2
AAAT.ATTT	X	17038275	17038292	4.5	ENSSSCT00000036543	MAP3K15	intron	.	rs790081070	2
AAAT.ATTT	X	17038275	17038292	4.5	ENSSSCT00000024706	MAP3K15	intron	.	rs790081070	2
ACC.GGT	X	17100361	17100380	6.66667	ENSSSCT00000036543	MAP3K15	intron	0.548	rs793334333	2
ACC.GGT	X	17100361	17100380	6.66667	ENSSSCT00000024706	MAP3K15	intron	0.548	rs793334333	2
AT.AT	X	17911140	17911149	5	.	.	intergenic	.	rs793560852	2
AC.GT	X	17962087	17962099	6.5	.	.	intergenic	0.324	.	2
AAAC.GTTT	X	19408511	19408531	5.25	ENSSSCT00000034419	PHEX	intron	0.23	rs793838836	2
AAAC.GTTT	X	19408511	19408531	5.25	ENSSSCT00000013308	PHEX	intron	0.23	rs793838836	2
AG.CT	X	19546508	19546519	6	ENSSSCT00000034419	PHEX	intron	0.17	rs787540011	2
AG.CT	X	19546508	19546519	6	ENSSSCT00000013308	PHEX	intron	0.17	rs787540011	2
AC.GT	X	20130106	20130120	7.5	.	.	intergenic	.	347187 rs7909	2
AT.AT	X	20447048	20447068	10.5	.	.	intergenic	-0.031	rs792383454	2
AAAC.GTTT	X	20769242	20769263	5.5	.	.	intergenic	0.228	rs789589154	2
AAAAG.CTTTT	X	20891858	20891881	4.8	ENSSSCT00000034582	PTCHD1	intron	0.517	rs791382418	2
AAAAG.CTTTT	X	20891858	20891881	4.8	ENSSSCT00000013312	PTCHD1	intron	0.517	rs791382418	2
AT.AT	X	20915904	20915915	6	.	.	intergenic	1.015	rs789568474	2

AG.CT	X	21157234	21157250	8.5	.	.	intergenic	-0.23	rs789902622	2
AAAC.GTTT	X	22331548	22331574	6.75	.	.	intergenic	0.596	rs788801306	4
AAT.ATT	X	22351133	22351144	4	.	.	intergenic	0.2	rs789490613	2
AAAG.CTTT	X	22395699	22395718	5	ENSSSCT00000033790	POLA1	intron	0.518	rs786684734	2
AAACC.GGTTT	X	22426208	22426235	5.6	ENSSSCT00000033790	POLA1	intron	0.438	.	2
AAAT.ATTT	X	22488261	22488279	4.75	.	.	intergenic	.	.	2
AAAT.ATTT	X	22539939	22539966	7	.	.	intergenic	.	rs793757045	2
AC.GT	X	22677879	22677889	5.5	.	.	intergenic	.	.	2
AAAC.GTTT	X	22811269	22811283	3.75	.	.	intergenic	0.32	rs793339842	2
AAAG.CTTT	X	22895788	22895812	6.25	.	.	intergenic	0.202	rs790168826	2
AAAT.ATTT	X	23009506	23009518	3.25	.	.	intergenic	.	rs791935652	2
AAATT.AATTT	X	23234475	23234500	5.2	.	.	intergenic	0.234	rs788739561	2
AAAAC.GTTTT	X	23299237	23299255	3.8	.	.	intergenic	.	.	3
AAT.ATT	X	23303109	23303135	9	.	.	intergenic	0.165	rs788784682	2
AAT.ATT	X	23401877	23401890	4.66667	.	.	intergenic	0.265	rs791622522	2
AAAAG.CTTTT	X	23822154	23822184	6.2	.	.	intergenic	.	rs793482822	2
AAAT.ATTT	X	24305338	24305355	4.5	.	.	intergenic	0.145	rs787083544	2
AT.AT	X	24487702	24487712	5.5	.	.	intergenic	0.53	rs789895739	3
AAAAC.GTTTT	X	24563571	24563589	3.8	.	.	intergenic	0.137	rs790636137	2
AG.CT	X	24571632	24571643	6	.	.	intergenic	0.225	.	2
AC.GT	X	24664275	24664284	5	.	.	intergenic	.	.	2
AT.AT	X	24768644	24768661	9	.	.	intergenic	.	rs788583599	2
AT.AT	X	25151129	25151156	14	.	.	intergenic	0.247	)58160 rs7937	2
AAAC.GTTT	X	25269977	25270002	6.5	.	.	intergenic	.	rs792342108	2
ATATG.CATAT	X	25592448	25592469	3.66667	.	.	intergenic	0.256	rs791283087	2
AC.GT	X	26001291	26001303	6.5	.	.	intergenic	.	rs792970151	2
AAAC.GTTT	X	26148597	26148612	4	.	.	intergenic	0.298	rs791909843	2
AAAC.GTTT	X	26221486	26221506	5.25	.	.	intergenic	.	rs791306507	2
AC.GT	X	26295726	26295750	12.5	.	.	intergenic	0.267	)54083 rs7869	2
AC.GT	X	26300140	26300150	5.5	.	.	intergenic	0.386	.	2
AAAT.ATTT	X	26319267	26319287	5.25	.	.	intergenic	0.599	281781 rs7874	2
AAC.GTT	X	26726654	26726680	9	.	.	intergenic	0.021	rs792264873	3
AATC.GATT	X	26754543	26754570	7	.	.	intergenic	.	rs790036968	2
AT.AT	X	26831808	26831826	9.5	.	.	intergenic	0.206	rs789252749	2
AC.GT	X	26957814	26957829	8	.	.	intergenic	-0.045	.	2
AC.GT	X	27672549	27672570	11	.	.	intergenic	-0.054	rs788284359	3
ACAT.ATGT	X	27677955	27677970	4	.	.	intergenic	0.024	rs787777193	2
AATG.CATT	X	27874529	27874557	7.25	.	.	intergenic	0.196	rs789921955	2
AAAT.ATTT	X	27932768	27932792	6.25	.	.	intergenic	0.012	rs788999321	2
AATAC.GTATT	X	27962946	27962966	3.5	.	.	intergenic	0.501	rs788096655	2
AG.CT	X	27969973	27969983	5.5	.	.	intergenic	0.03	rs791206836	2
AAAC.GTTT	X	27980554	27980568	3.75	.	.	intergenic	0.473	rs792488458	2
AC.GT	X	28193683	28193705	11.5	ENSSSCT00000026433	.	exon	0.729	.	2
AC.GT	X	28668684	28668694	5.5	.	.	intergenic	.	.	4
AAAT.ATTT	X	28931920	28931935	4	.	.	intergenic	.	rs786311944	2
AC.GT	X	28938922	28938932	5.5	.	.	intergenic	.	.	2
AC.GT	X	29484329	29484340	6	ENSSSCT00000029079	DMD	intron	-0.027	rs786867395	2
AC.GT	X	29484329	29484340	6	ENSSSCT00000013350	DMD	intron	-0.027	rs786867395	2
AC.GT	X	29505452	29505462	5.5	ENSSSCT00000029079	DMD	intron	.	rs786592457	2
AC.GT	X	29505452	29505462	5.5	ENSSSCT00000013350	DMD	intron	.	rs786592457	2
AAAC.GTTT	X	29594018	29594053	9	.	.	intergenic	0.092	.	2
AC.GT	X	29683392	29683413	11	.	.	intergenic	0.264	.	2
AAAT.ATTT	X	29783843	29783862	5	.	.	intergenic	0.389	rs792058914	2
AAAAC.GTTTT	X	31002914	31002943	5	.	.	intergenic	0.107	rs793277652	2
AAAC.GTTT	X	31322893	31322904	3	ENSSSCT00000034304	DMD	intron	0.016	.	2
AAAC.GTTT	X	31322893	31322904	3	ENSSSCT00000026041	DMD	intron	0.016	.	2
AG.CT	X	31362918	31362929	6	ENSSSCT00000034304	DMD	intron	-0.183	.	3
AG.CT	X	31362918	31362929	6	ENSSSCT00000026041	DMD	intron	-0.183	.	3
AT.AT	X	31764118	31764127	5	ENSSSCT00000034304	DMD	intron	0.067	rs790032476	2
AT.AT	X	31764118	31764127	5	ENSSSCT00000026041	DMD	intron	0.067	rs790032476	2
AAAC.GTTT	X	31809224	31809267	11	ENSSSCT00000026041	DMD	intron	.	rs786660655	2
AAAC.GTTT	X	31809224	31809267	11	ENSSSCT00000034304	DMD	intron	.	rs786660655	2
AT.AT	X	31825580	31825589	5	ENSSSCT00000026041	DMD	intron	0.089	rs792469622	2
AT.AT	X	31825580	31825589	5	ENSSSCT00000034304	DMD	intron	0.089	rs792469622	2
AAAC.GTTT	X	31875864	31875888	6.25	ENSSSCT00000034304	DMD	intron	0.773	.	2
AAAC.GTTT	X	31875864	31875888	6.25	ENSSSCT00000026041	DMD	promoter	0.773	.	2
AC.GT	X	32057928	32057968	20.5	.	.	intergenic	0.127	461238 rs7933	3
AAT.ATT	X	32321319	32321336	6	.	.	intergenic	0.074	rs790635253	2
AAT.ATT	X	32686731	32686744	4.66667	.	.	intergenic	.	rs787979627	2
AC.GT	X	34092317	34092346	15	.	.	intergenic	0.131	rs790831853	3
AT.AT	X	34146088	34146098	5.5	.	.	intergenic	.	.	2
ACAT.ATGT	X	34582621	34582646	6.5	.	.	intergenic	0.319	rs787650765	3
AC.GT	X	34748953	34748977	12.5	.	.	intergenic	.	.	4
AAAC.GTTT	X	34973092	34973119	7	.	.	intergenic	.	rs793339151	3
AAAT.ATTT	X	34980131	34980151	5.25	.	.	intergenic	0.072	.	2
AAT.ATT	X	35210614	35210635	7.33333	.	.	intergenic	.	rs788500941	2
AAC.GTT	X	35563234	35563251	6	.	.	intergenic	.	334297 rs7876	2
AT.AT	X	35639983	35639995	6.5	ENSSSCT00000034859	CXorf30	intron	0.258	402498 rs7862	3
AG.CT	X	35701844	35701858	7.5	ENSSSCT00000023277	CXorf30	intron	.	.	2
AAAC.GTTT	X	35821033	35821047	3.75	.	.	intergenic	.	rs790780328	2
AC.GT	X	35833975	35833990	8	.	.	intergenic	0.055	.	3
AC.GT	X	36411268	36411299	16	.	.	intergenic	0.144	rs786376478	3
AC.GT	X	36842334	36842345	6	ENSSSCT00000028670	PRRG1	intron	.	307532 rs7916	3
AC.GT	X	36842334	36842345	6	ENSSSCT00000036005	PRRG1	intron	.	307532 rs7916	3

AC.GT	X	37274150	37274159	5	.	.	intergenic	-0.037	.	2
AC.GT	X	37882455	37882470	8	.	.	intergenic	0.123	.	4
AAC.GTT	X	37905681	37905692	4	ENSSSCT00000034622	SRPX	intron	.	rs792105344	2
AAC.GTT	X	37905681	37905692	4	ENSSSCT00000013380	SRPX	intron	.	rs792105344	2
AAC.GTT	X	37905681	37905692	4	ENSSSCT00000036512	SRPX	intron	.	rs792105344	2
AAC.GTT	X	37905681	37905692	4	ENSSSCT00000034428	SRPX	intron	.	rs792105344	2
AT.AT	X	38273741	38273750	5	.	.	intergenic	0.361	964543 rs7917	2
AGC.GCT	X	38280232	38280245	4.66667	.	.	intergenic	0.236	.	2
AAAC.GTTT	X	39415385	39415399	3.75	.	.	intergenic	-0.134	rs788832010	2
AAAT.ATTT	X	39541295	39541309	3.75	.	.	intergenic	0.544	rs788877296	2
ACG.CGT	X	39904595	39904611	5.66667	ENSSSCT00000035677	BCOR	intron	0.146	rs790956970	2
ACG.CGT	X	39904595	39904611	5.66667	ENSSSCT00000034818	BCOR	intron	0.146	rs790956970	2
ACG.CGT	X	39904595	39904611	5.66667	ENSSSCT00000035361	BCOR	intron	0.146	rs790956970	2
AG.CT	X	40059612	40059625	7	.	.	intergenic	0.021	.	2
AT.AT	X	40362325	40362336	6	.	.	intergenic	0.252	rs791381713	2
AT.AT	X	40433357	40433367	5.5	ENSSSCT00000013395	CXorf38	intron	-0.156	.	3
AT.AT	X	40433357	40433367	5.5	ENSSSCT00000035325	CXorf38	intron	-0.156	.	3
AT.AT	X	40433357	40433367	5.5	ENSSSCT00000036189	CXorf38	intron	-0.156	.	3
AAC.GTT	X	40662973	40662998	8.66667	.	.	intergenic	.	rs790460649	2
AAAC.GTTT	X	41061754	41061766	3.25	ENSSSCT00000033052	H242-15C8	exon	1.399	339780 rs7869	2
AAG.CTT	X	41097333	41097348	5.33333	.	.	intergenic	.	rs789109384	2
AG.CT	X	41263607	41263620	7	.	.	intergenic	0.177	rs793312528	2
AAAAC.GTTTT	X	41267907	41267924	3.6	.	.	intergenic	-0.115	rs790424528	2
AAAC.GTTT	X	41628962	41628981	5	ENSSSCT00000035253	CASK	intron	0.235	rs789268222	2
AAAC.GTTT	X	41628962	41628981	5	ENSSSCT00000013399	CASK	intron	0.235	rs789268222	2
AC.GT	X	42212688	42212698	5.5	.	.	intergenic	0.134	rs793542398	2
AAC.GTT	X	42339329	42339340	4	.	.	intergenic	0.217	rs788173813	2
AAT.ATT	X	42672018	42672038	7	.	.	intergenic	0.284	rs788902643	2
AT.AT	X	42817787	42817800	7	.	.	intergenic	0.25	476322 rs7866	2
AC.GT	X	43105036	43105049	7	.	.	intergenic	0.112	rs787821952	2
AC.GT	X	43180788	43180801	7	ENSSSCT00000033354	MAOA	intron	0.306	rs789525502	2
AC.GT	X	43180788	43180801	7	ENSSSCT00000013404	MAOA	intron	0.306	rs789525502	2
AC.GT	X	43180788	43180801	7	ENSSSCT00000032764	MAOA	intron	0.306	rs789525502	2
AAAG.CTTT	X	43182887	43182900	3.5	ENSSSCT00000033354	MAOA	intron	.	rs788131584	2
AAAG.CTTT	X	43182887	43182900	3.5	ENSSSCT00000013404	MAOA	intron	.	rs788131584	2
AAAG.CTTT	X	43182887	43182900	3.5	ENSSSCT00000032764	MAOA	intron	.	rs788131584	2
AAAC.GTTT	X	43688332	43688354	5.75	.	.	intergenic	0.166	rs787330094	2
AT.AT	X	43794506	43794517	6	ENSSSCT00000029739	EFHC2	intron	.	rs790624497	2
AT.AT	X	43794506	43794517	6	ENSSSCT00000035249	EFHC2	intron	.	rs790624497	2
AATT.AATT	X	44651634	44651651	4.5	ENSSSCT00000034832	KDM6A	intron	0.305	rs791262832	2
AATT.AATT	X	44651634	44651651	4.5	ENSSSCT00000034861	KDM6A	intron	0.305	rs791262832	2
AATT.AATT	X	44651634	44651651	4.5	ENSSSCT00000035071	KDM6A	intron	0.305	rs791262832	2
AATT.AATT	X	44651634	44651651	4.5	ENSSSCT00000035936	KDM6A	intron	0.305	rs791262832	2
AATT.AATT	X	44651634	44651651	4.5	ENSSSCT00000013409	KDM6A	intron	0.305	rs791262832	2
AATT.AATT	X	44651634	44651651	4.5	ENSSSCT00000034919	KDM6A	intron	0.305	rs791262832	2
AATT.AATT	X	44651634	44651651	4.5	ENSSSCT00000033770	KDM6A	intron	0.305	rs791262832	2
AATT.AATT	X	44651634	44651651	4.5	ENSSSCT00000033265	KDM6A	intron	0.305	rs791262832	2
AATT.AATT	X	44651634	44651651	4.5	ENSSSCT00000035863	KDM6A	intron	0.305	rs791262832	2
AATT.AATT	X	44651634	44651651	4.5	ENSSSCT00000036305	KDM6A	intron	0.305	rs791262832	2
AAC.GTT	X	44822239	44822250	4	.	.	intergenic	.	.	2
AC.GT	X	44958444	44958456	6.5	.	.	intergenic	0.19	514497 rs7930	2
AC.GT	X	45156176	45156194	9.5	.	.	intergenic	0.202	rs792973283	2
AT.AT	X	45168144	45168154	5.5	.	.	intergenic	-0.028	.	3
AAAT.ATTT	X	45272186	45272208	5.75	.	.	intergenic	0.071	rs793305311	2
AAAG.CTTT	X	45342534	45342548	3.75	.	.	intergenic	0.458	rs790881103	2
AG.CT	X	45393260	45393270	5.5	.	.	intergenic	0.281	rs787783842	2
AAAAC.GTTTT	X	45542374	45542392	3.8	.	.	intergenic	.	204260 rs7871	2
AC.GT	X	46315059	46315069	5.5	.	.	intergenic	0.357	rs792373853	4
AAAC.GTTT	X	46427287	46427314	7	ENSSSCT00000013414	JADE3	intron	.	129070 rs7921	2
AAAC.GTTT	X	46427287	46427314	7	ENSSSCT00000033102	JADE3	intron	.	129070 rs7921	2
AC.GT	X	47111007	47111016	5	.	.	intergenic	.	383659 rs7937	4
AAAC.GTTT	X	47345819	47345834	4	ENSSSCT00000033024	SYN1	intron	0.182	rs793225989	2
AAAC.GTTT	X	47345819	47345834	4	ENSSSCT00000013425	SYN1	intron	0.182	rs793225989	2
AAAT.ATTT	X	48214404	48214418	3.75	ENSSSCT00000029010	.	intron	-0.672	rs791215178	2
AC.GT	X	48460740	48460756	8.5	.	.	intergenic	0.207	rs792956381	2
AAAC.GTTT	X	48517075	48517098	6	.	.	intergenic	.	rs792616012	2
AC.GT	X	48537959	48537971	6.5	.	.	intergenic	.	rs787260020	2
AAAAT.ATTTT	X	48624892	48624910	3.8	.	.	intergenic	0.385	rs789937779	2
AAT.ATT	X	48740717	48740737	7	.	.	intergenic	0.165	rs786988680	2
AAAAT.ATTTT	X	48799129	48799145	3.4	.	.	intergenic	0.16	rs792340013	2
AG.CT	X	48801987	48802000	7	.	.	intergenic	0.184	rs786224782	2
AAAAC.GTTTT	X	48889720	48889743	4	ENSSSCT00000013457	CCNB3	intron	.	rs786973839	2
AAAAC.GTTTT	X	48889720	48889743	4	ENSSSCT00000035730	CCNB3	intron	.	rs786973839	2
AAAAG.CTTTT	X	48920252	48920270	3.8	.	.	intergenic	.	rs788661283	2
AAC.GTT	X	48957362	48957375	4.66667	ENSSSCT00000013458	.	intron	.	rs786784976	4
AT.AT	X	48994774	48994786	6.5	ENSSSCT00000013458	.	intron	.	282497 rs7872	2
AT.AT	X	49054855	49054874	10	ENSSSCT00000013458	.	intron	0.978	rs788960456	3
AG.CT	X	49127104	49127128	12.5	.	.	intergenic	0.134	305087 rs7921	4
AATG.CATT	X	49214980	49215000	5.25	.	.	intergenic	0.183	rs790786205	2
AT.AT	X	49353169	49353198	15	.	.	intergenic	0.239	rs791524335	2
AAT.ATT	X	49597802	49597820	6.33333	.	.	intergenic	0.161	rs792412282	2
AAG.CTT	X	49653988	49654003	5.33333	.	.	intergenic	.	rs789461783	2
AC.GT	X	50006282	50006294	6.5	.	.	intergenic	0.296	rs786760175	3
AG.CT	X	50389150	50389170	10.5	.	.	intergenic	.	rs788426983	2

AAAAT.ATTTT	X	50515676	50515697	4.4	.	.	intergenic	.	rs790591914	2
AT.AT	X	50550137	50550152	8	.	.	intergenic	.	rs792178346	2
AT.AT	X	50555730	50555739	5	.	.	intergenic	0.094	rs786766926	3
AAAAC.GTTTT	X	50709174	50709203	6	.	.	intergenic	0.176	rs787692147	2
AT.AT	X	50723851	50723869	9.5	.	.	intergenic	.	.	2
ACC.GGT	X	50894362	50894375	4.66667	.	.	intergenic	0.057	rs788184220	2
AT.AT	X	50980195	50980210	8	.	.	intergenic	.	rs786964156	2
AT.AT	X	51001066	51001075	5	.	.	intergenic	.	rs793641251	2
AAAC.GTTT	X	51218405	51218431	6.75	.	.	intergenic	0.088	rs787635637	3
AC.GT	X	51340559	51340574	8	.	.	intergenic	0.337	rs788498102	6
AC.GT	X	51390452	51390465	7	ENSSSCT00000033938	GPR173	intron	0.184	rs791598326	2
AAAAT.ATTTT	X	51703606	51703637	6.4	ENSSSCT00000034912	SMC1A	intron	0.415	rs792206716	2
AAAAT.ATTTT	X	51703606	51703637	6.4	ENSSSCT00000033805	RIBC1	intron	0.415	rs792206716	2
AAAAT.ATTTT	X	51703606	51703637	6.4	ENSSSCT00000033717	.	promoter	0.415	rs792206716	2
AAAAT.ATTTT	X	51703606	51703637	6.4	ENSSSCT00000033262	.	promoter	0.415	rs792206716	2
AAAAT.ATTTT	X	51703606	51703637	6.4	ENSSSCT00000035966	.	promoter	0.415	rs792206716	2
AAAAT.ATTTT	X	51703606	51703637	6.4	ENSSSCT00000028659	.	promoter	0.415	rs792206716	2
AAAAT.ATTTT	X	51703606	51703637	6.4	ENSSSCT00000013478	.	promoter	0.415	rs792206716	2
AAC.GTT	X	51885038	51885060	7.66667	ENSSSCT00000034630	HUWE1	intron	0.782	rs790406966	2
AAC.GTT	X	51885038	51885060	7.66667	ENSSSCT00000035055	HUWE1	intron	0.782	rs790406966	2
AAC.GTT	X	51885038	51885060	7.66667	ENSSSCT00000013481	HUWE1	intron	0.782	rs790406966	2
AAC.GTT	X	51885038	51885060	7.66667	ENSSSCT00000032330	HUWE1	intron	0.782	rs790406966	2
ACC.GGT	X	51992675	51992686	4	.	.	intergenic	.	rs792431099	2
AC.GT	X	52896611	52896626	8	ENSSSCT00000013486	WNK3	intron	.	rs790756884	2
AC.GT	X	52896611	52896626	8	ENSSSCT00000034044	WNK3	intron	.	rs790756884	2
AG.CT	X	52981915	52981933	9.5	.	.	intergenic	0.236	rs786958891	4
AT.AT	X	53110969	53110980	6	.	.	intergenic	.	rs790055442	2
AC.GT	X	53243802	53243816	7.5	.	.	intergenic	.	rs788642381	2
AAAT.ATTT	X	54077231	54077251	5.25	.	.	intergenic	.	rs787588125	2
AG.CT	X	55283934	55283948	7.5	.	.	intergenic	.	rs793506657	2
AAAAC.GTTTT	X	55912666	55912687	4.4	.	.	intergenic	.	rs788733873	2
AAC.GTT	X	56012734	56012753	6.66667	.	.	intergenic	.	rs787687596	2
AAAC.GTTT	X	56160831	56160853	5.75	.	.	intergenic	.	rs791048923	2
AAC.GTT	X	56270907	56270934	9.33333	.	.	intergenic	.	rs789499714	2
AAC.GTT	X	56617365	56617387	7.66667	ENSSSCT00000033812	ARHGEF9	intron	.	rs789732194	2
AAC.GTT	X	56617365	56617387	7.66667	ENSSSCT00000033580	ARHGEF9	intron	.	rs789732194	2
AAC.GTT	X	56617365	56617387	7.66667	ENSSSCT00000013519	ARHGEF9	intron	.	rs789732194	2
AAC.GTT	X	56617365	56617387	7.66667	ENSSSCT00000033226	ARHGEF9	intron	.	rs789732194	2
AAC.GTT	X	56617365	56617387	7.66667	ENSSSCT00000035007	ARHGEF9	intron	.	rs789732194	2
AG.CT	X	56825471	56825511	20.5	.	.	intergenic	0.212	rs787666160	3
AAT.ATT	X	56852785	56852802	6	.	.	intergenic	.	rs791749371	2
AT.AT	X	57006481	57006494	7	.	.	intergenic	.	ı82233 rs7911	2
AG.CT	X	57183539	57183551	6.5	.	.	intergenic	.	.	2
AC.GT	X	57683687	57683705	9.5	ENSSSCT00000032193	ZC4H2	intron	0.214	rs790965704	2
AC.GT	X	57989487	57989499	6.5	.	.	intergenic	.	rs787253601	2
AT.AT	X	58180293	58180321	14.5	ENSSSCT00000013524	ZC3H12B	intron	0.192	rs789544291	2
AG.CT	X	58198170	58198184	7.5	ENSSSCT00000013524	ZC3H12B	intron	.	rs793608651	2
AC.GT	X	58409470	58409483	7	.	.	intergenic	.	.	2
AAAAAT.ATTTTT	X	58441694	58441715	3.66667	.	.	intergenic	0.193	rs790820786	2
AC.GT	X	58455381	58455403	11.5	.	.	intergenic	.	rs792384781	2
AC.GT	X	58486018	58486051	17	ENSSSCT00000034603	MSN	intron	0.355	rs792100215	2
AAGG.CCTT	X	58743506	58743524	4.75	.	.	intergenic	0.321	rs787944163	2
AG.CT	X	58783062	58783075	7	.	.	intergenic	0.059	rs788616054	3
AAAT.ATTT	X	58893252	58893263	3	ENSSSCT00000013520	HEPH	promoter	-0.042	)86603 rs7870	2
AAAT.ATTT	X	58893252	58893263	3	ENSSSCT00000034250	HEPH	promoter	-0.042	)86603 rs7870	2
AAAAT.ATTTT	X	59060033	59060052	4	.	.	intergenic	.	.	2
AT.AT	X	59113771	59113783	6.5	.	.	intergenic	.	rs789239446	2
AAC.GTT	X	59412727	59412743	5.66667	.	.	intergenic	.	.	2
AAG.CTT	X	60399370	60399384	5	ENSSSCT00000013528	AR	intron	.	rs793429697	2
AG.CT	X	60630427	60630443	8.5	.	.	intergenic	.	rs788226555	2
AAT.ATT	X	60931187	60931200	4.66667	.	.	intergenic	.	rs791884275	2
AAAC.GTTT	X	61376249	61376264	4	.	.	intergenic	.	rs787399676	2
ACC.GGT	X	61431865	61431878	4.66667	.	.	intergenic	.	rs790126169	2
AG.CT	X	61446124	61446135	6	.	.	intergenic	0.16	rs791300667	3
AAAC.GTTT	X	62094883	62094902	5	.	.	intergenic	0.105	rs787242792	2
AG.CT	X	62436155	62436184	15	.	.	intergenic	.	rs787968075	2
AAAC.GTTT	X	62470960	62470996	9.25	.	.	intergenic	0.158	ı95378 rs7916	3
AG.CT	X	62614845	62614855	5.5	.	.	intergenic	0.095	rs787319455	2
AATG.CATT	X	63127059	63127076	4.5	ENSSSCT00000034708	EDA	intron	0.091	rs788582524	2
AATG.CATT	X	63127059	63127076	4.5	ENSSSCT00000033545	EDA	intron	0.091	rs788582524	2
AG.CT	X	63485430	63485444	7.5	.	.	intergenic	0.167	.	2
AT.AT	X	63682597	63682608	6	ENSSSCT00000013532	DLG3	intron	0.365	rs791792379	2
AC.GT	X	63686551	63686564	7	ENSSSCT00000013532	DLG3	intron	0.48	rs792217786	3
AAAC.GTTT	X	64462753	64462772	5	.	.	intergenic	0.332	rs791644106	2
AAAAC.GTTTT	X	64484995	64485014	4	.	.	intergenic	.	rs789214604	2
AC.GT	X	64629900	64629910	5.5	.	.	intergenic	0.539	rs790505038	2
AAAG.CTTT	X	64836696	64836709	3.5	.	.	intergenic	0.139	.	2
AAAC.GTTT	X	64955365	64955386	5.5	.	.	intergenic	0.432	;40622 rs7888	2
AAAC.GTTT	X	64982299	64982317	4.75	ENSSSCT00000013561	OGT	intron	3.039	rs792757274	2
AAAC.GTTT	X	64982299	64982317	4.75	ENSSSCT00000035659	OGT	intron	3.039	rs792757274	2
AAAC.GTTT	X	64982299	64982317	4.75	ENSSSCT00000032783	OGT	intron	3.039	rs792757274	2
ATC.GAT	X	65020663	65020681	6.33333	ENSSSCT00000034006	ACRC	promoter	-0.072	?14314 rs7910	2
AAC.GTT	X	65025130	65025142	4.33333	ENSSSCT00000034006	ACRC	intron	0.266	rs789634854	3
AAC.GTT	X	65025130	65025142	4.33333	ENSSSCT00000013559	ACRC	promoter	0.266	rs789634854	3

ACAT.ATGT	X	65389689	65389704	4	.	.	intergenic	0.208	.	2
AAAC.GTTT	X	65400053	65400074	5.5	.	.	intergenic	.	rs791620242	2
ATCC.GGAT	X	65566986	65567021	9	.	.	intergenic	0.207	rs793148681	2
AAAAC.GTTTT	X	65941713	65941736	4.8	.	.	intergenic	0.431	rs791773164	2
AATG.CATT	X	66091912	66091933	5.5	ENSSSCT00000013570	.	intron	0.988	rs788916292	2
AAAAC.GTTTT	X	66445214	66445232	3.8	.	.	intergenic	0.274	rs792978486	2
AG.CT	X	66541199	66541214	8	.	.	intergenic	.	rs787998638	2
AAC.GTT	X	66562845	66562858	4.66667	.	.	intergenic	.	rs787662462	3
AC.GT	X	66699179	66699191	6.5	.	.	intergenic	.	rs787300540	2
AAAC.GTTT	X	66952756	66952777	5.5	.	.	intergenic	.	rs793349905	2
AT.AT	X	66972899	66972909	5.5	ENSSSCT00000030959	CDX4	intron	0.243	rs789920426	2
AT.AT	X	66972899	66972909	5.5	ENSSSCT00000034899	CDX4	intron	0.243	rs789920426	2
AT.AT	X	67054811	67054824	7	ENSSSCT00000013576	CHIC1	intron	0.388	rs791738866	3
AT.AT	X	67054811	67054824	7	ENSSSCT00000034312	CHIC1	intron	0.388	rs791738866	3
AT.AT	X	67054811	67054824	7	ENSSSCT00000033759	CHIC1	intron	0.388	rs791738866	3
AAAAC.GTTTT	X	67370573	67370591	3.8	.	.	intergenic	.	rs787565489	2
AAAT.ATTT	X	67391494	67391508	3.75	ENSSSCT00000020222	.	promoter	.	rs788278582	2
AAAT.ATTT	X	67545239	67545253	3.75	.	.	intergenic	.	rs793600060	2
AT.AT	X	67639602	67639629	14	.	.	intergenic	.	rs792412868	5
AAT.ATT	X	67655991	67656017	9	.	.	intergenic	.	rs787611239	3
AAAT.ATTT	X	68280378	68280407	7.5	.	.	intergenic	0.278	rs790307588	2
AAAAC.GTTTT	X	68384436	68384452	3.4	.	.	intergenic	0.335	.	2
ACC.GGT	X	68392676	68392691	5.33333	.	.	intergenic	0.098	.	2
AAAT.ATTT	X	68445285	68445300	4	ENSSSCT00000036440	ABCB7	intron	0.475	rs786326208	2
AAAT.ATTT	X	68445285	68445300	4	ENSSSCT00000036358	ABCB7	intron	0.475	rs786326208	2
AAAT.ATTT	X	68445285	68445300	4	ENSSSCT00000032985	ABCB7	intron	0.475	rs786326208	2
AAAT.ATTT	X	68445285	68445300	4	ENSSSCT00000013585	ABCB7	intron	0.475	rs786326208	2
AAAT.ATTT	X	68445285	68445300	4	ENSSSCT00000033898	ABCB7	intron	0.475	rs786326208	2
AAAAC.GTTTT	X	68687380	68687397	3.6	ENSSSCT00000013586	UPRT	promoter	0.236	rs789434264	2
AAAAC.GTTTT	X	68687380	68687397	3.6	ENSSSCT00000035125	UPRT	promoter	0.236	rs789434264	2
AAAAC.GTTTT	X	68687380	68687397	3.6	ENSSSCT00000033503	UPRT	promoter	0.236	rs789434264	2
AT.AT	X	69256911	69256929	9.5	.	.	intergenic	-0.044	rs788079280	2
AAAT.ATTT	X	70246369	70246387	4.75	.	.	intergenic	.	223895 rs7901	2
ACAT.ATGT	X	70430505	70430521	4.25	ENSSSCT00000035999	H242-77E21	intron	0.275	rs788575186	2
ACAT.ATGT	X	70430505	70430521	4.25	ENSSSCT00000036604	ATRX	intron	0.275	rs788575186	2
ACAT.ATGT	X	70430505	70430521	4.25	ENSSSCT00000013595	ATRX	intron	0.275	rs788575186	2
AC.GT	X	70665103	70665119	8.5	ENSSSCT00000036604	ATRX	intron	0.261	rs788883380	2
AC.GT	X	70665103	70665119	8.5	ENSSSCT00000034791	ATRX	intron	0.261	rs788883380	2
AC.GT	X	70665103	70665119	8.5	ENSSSCT00000013595	ATRX	intron	0.261	rs788883380	2
AC.GT	X	70681287	70681296	5	ENSSSCT00000034747	ATRX	intron	0.454	.	2
AC.GT	X	70681287	70681296	5	ENSSSCT00000036604	ATRX	intron	0.454	.	2
AC.GT	X	70681287	70681296	5	ENSSSCT00000036526	ATRX	intron	0.454	.	2
AC.GT	X	70681287	70681296	5	ENSSSCT00000013596	ATRX	intron	0.454	.	2
AC.GT	X	70681287	70681296	5	ENSSSCT00000013595	ATRX	promoter	0.454	.	2
AAAAAC.GTTTTT	X	70813006	70813031	4.33333	ENSSSCT00000035022	MAGT1	3'utr	0.564	rs791347800	2
AG.CT	X	71079040	71079064	12.5	.	.	intergenic	.	rs792850813	4
AC.GT	X	71252168	71252177	5	.	.	intergenic	.	.	2
AAAT.ATTT	X	71327075	71327103	7.25	ENSSSCT00000029310	TAF9B	intron	.	347660 rs7886	2
AAAT.ATTT	X	71327075	71327103	7.25	ENSSSCT00000033907	TAF9B	intron	.	347660 rs7886	2
AAAT.ATTT	X	71327075	71327103	7.25	ENSSSCT00000023290	TAF9B	intron	.	347660 rs7886	2
AC.GT	X	71734736	71734753	9	.	.	intergenic	.	rs791218449	2
AAT.ATT	X	71746209	71746223	5	.	.	intergenic	0.17	rs792701856	2
AT.AT	X	71769157	71769168	6	.	.	intergenic	.	rs788532882	2
AT.AT	X	71842458	71842469	6	.	.	intergenic	.	rs792985978	2
AAC.GTT	X	72337387	72337399	4.33333	.	.	intergenic	.	rs787111826	2
AAAC.GTTT	X	72338356	72338370	3.75	.	.	intergenic	.	rs790706996	2
AAAAT.ATTTT	X	72376884	72376898	3	.	.	intergenic	.	rs789557316	2
AAT.ATT	X	72387554	72387566	4.33333	.	.	intergenic	.	rs791364033	2
AAATG.CATTT	X	72433095	72433122	5.6	.	.	intergenic	.	rs787617817	2
AAAC.GTTT	X	72451133	72451147	3.75	.	.	intergenic	.	.	2
AAT.ATT	X	72477193	72477211	6.33333	.	.	intergenic	0.025	rs790657483	2
AAAC.GTTT	X	72611180	72611204	6.25	.	.	intergenic	.	rs787830843	2
AT.AT	X	72729237	72729246	5	.	.	intergenic	.	rs790516349	2
AC.GT	X	72809015	72809024	5	.	.	intergenic	-0.229	rs786603809	2
AAC.GTT	X	72827991	72828011	7	.	.	intergenic	.	rs789357093	2
AC.GT	X	73528807	73528825	9.5	.	.	intergenic	.	.	4
AC.GT	X	73723153	73723163	5.5	.	.	intergenic	.	.	2
AG.CT	X	73770849	73770865	8.5	.	.	intergenic	.	rs789773029	2
AT.AT	X	73886236	73886245	5	ENSSSCT00000033871	I242-123K1	intron	0.138	.	2
AT.AT	X	73886236	73886245	5	ENSSSCT00000033174	I242-123K1	intron	0.138	.	2
AT.AT	X	73886236	73886245	5	ENSSSCT00000031239	I242-123K1	intron	0.138	.	2
AAT.ATT	X	73972617	73972630	4.66667	ENSSSCT00000033174	I242-123K1	intron	0.142	.	2
AAT.ATT	X	73972617	73972630	4.66667	ENSSSCT00000031239	I242-123K1	intron	0.142	.	2
AAAC.GTTT	X	74247486	74247500	3.75	.	.	intergenic	0.27	602431 rs7932	2
AAAAT.ATTTT	X	74377712	74377728	3.4	.	.	intergenic	.	rs793428251	2
AAT.ATT	X	74423790	74423809	6.66667	.	.	intergenic	.	rs793010287	2
AC.GT	X	74797907	74797923	8.5	.	.	intergenic	.	rs787158234	4
AG.CT	X	74810517	74810529	6.5	.	.	intergenic	.	.	2
AAC.GTT	X	75033179	75033196	6	.	.	intergenic	.	rs789327056	2
AAC.GTT	X	75048010	75048040	10.3333	.	.	intergenic	.	rs792288922	2
AAAAC.GTTTT	X	75110632	75110660	5.8	.	.	intergenic	.	rs789323168	2
AT.AT	X	75541614	75541627	7	.	.	intergenic	.	rs792090473	2
AT.AT	X	75551020	75551031	6	.	.	intergenic	.	rs790752513	2
AAAT.ATTT	X	75575793	75575811	4.75	.	.	intergenic	.	.	2

AAT.ATT	X	76258124	76258141	6	.	.	intergenic	.	rs788566218	4
AAAAT.ATTTT	X	76453880	76453903	4.8	.	.	intergenic	.	rs793462338	2
AG.CT	X	76578223	76578243	10.5	.	.	intergenic	.	rs793429248	3
AAAC.GTTT	X	76721441	76721471	7.75	.	.	intergenic	-0.036	;73506 rs7898	2
AG.CT	X	76778848	76778858	5.5	.	.	intergenic	.	rs792010964	2
AT.AT	X	77182594	77182605	6	ENSSSCT00000032934	RPS6KA6	intron	.	rs793318647	2
AT.AT	X	77182594	77182605	6	ENSSSCT00000013622	RPS6KA6	intron	.	rs793318647	2
AAAC.GTTT	X	77252488	77252514	6.75	ENSSSCT00000013622	RPS6KA6	intron	.	rs791505897	2
AAAC.GTTT	X	77252488	77252514	6.75	ENSSSCT00000032934	RPS6KA6	intron	.	rs791505897	2
AG.CT	X	77302879	77302899	10.5	.	.	intergenic	.	.	2
AT.AT	X	77409737	77409752	8	.	.	intergenic	0.15	rs793559558	2
AAAAC.GTTTT	X	77426728	77426760	6.6	.	.	intergenic	0.876	.	2
AAAG.CTTT	X	77469995	77470021	6.75	.	.	intergenic	-0.029	.	2
AT.AT	X	77674789	77674808	10	.	.	intergenic	.	rs791966059	2
AGAT.ATCT	X	77850649	77850668	5	.	.	intergenic	.	rs789515026	2
AG.CT	X	77883085	77883104	10	.	.	intergenic	.	rs786742640	2
AT.AT	X	77886868	77886877	5	.	.	intergenic	.	rs789033272	2
AAAT.ATTT	X	77917804	77917819	4	.	.	intergenic	0.373	rs791299010	2
AG.CT	X	77970396	77970408	6.5	.	.	intergenic	.	rs791279021	2
AAAT.ATTT	X	78198920	78198937	4.5	ENSSSCT00000029661	.	intron	.	.	2
AT.AT	X	78271280	78271301	11	.	.	intergenic	.	rs786582178	2
AT.AT	X	78417726	78417737	6	.	.	intergenic	.	rs790941312	2
AG.CT	X	78471671	78471693	11.5	.	.	intergenic	0.24	rs692863645	2
AC.GT	X	78613889	78613898	5	.	.	intergenic	.	rs791690546	2
AAG.CTT	X	78640813	78640824	4	.	.	intergenic	-0.031	.	2
AT.AT	X	78714289	78714301	6.5	.	.	intergenic	0.259	rs787555762	2
AAT.ATT	X	79790981	79791006	8.66667	.	.	intergenic	-0.012	rs793417337	2
AG.CT	X	80433727	80433741	7.5	.	.	intergenic	.	.	3
AGAT.ATCT	X	80752189	80752222	8.5	.	.	intergenic	.	'35620 rs7937	2
AATG.CATT	X	80770513	80770539	6.75	.	.	intergenic	.	rs787993169	2
AT.AT	X	80871968	80871978	5.5	.	.	intergenic	.	.	2
AAAT.ATTT	X	80877211	80877232	5.5	.	.	intergenic	.	rs789982113	2
AAC.GTT	X	80945255	80945270	5.33333	ENSSSCT00000013634	KLHL4	intron	.	rs792596337	2
AC.GT	X	80971422	80971432	5.5	ENSSSCT00000013634	KLHL4	intron	3.287	rs793375645	2
AAC.GTT	X	81037779	81037790	4	.	.	intergenic	.	.	2
AT.AT	X	81075568	81075578	5.5	.	.	intergenic	.	rs789055204	4
AAAT.ATTT	X	81219804	81219824	5.25	.	.	intergenic	.	rs791822041	2
AAC.GTT	X	81297953	81297969	5.66667	.	.	intergenic	.	rs791214461	2
AC.GT	X	81423436	81423457	11	.	.	intergenic	.	.	5
AT.AT	X	81640264	81640284	10.5	.	.	intergenic	.	rs791138283	2
AC.GT	X	82201715	82201725	5.5	.	.	intergenic	.	rs789087231	2
AC.GT	X	82646963	82646977	7.5	.	.	intergenic	.	rs786596897	2
AT.AT	X	82661958	82661968	5.5	.	.	intergenic	.	rs792221130	2
AT.AT	X	82701367	82701376	5	.	.	intergenic	.	rs788521438	2
AAAT.ATTT	X	83109358	83109377	5	.	.	intergenic	.	rs788760320	2
AAAT.ATTT	X	84691923	84691938	4	.	.	intergenic	.	rs792454875	2
AAAT.ATTT	X	84751574	84751597	6	.	.	intergenic	0.217	rs792293298	2
AAAC.GTTT	X	84890326	84890358	8.25	.	.	intergenic	0.152	rs791625988	2
AC.GT	X	84962729	84962741	6.5	.	.	intergenic	0.179	.	2
AC.GT	X	85196155	85196168	7	.	.	intergenic	0.361	.	2
\AAAAC.GTTTT	X	85232036	85232060	4.16667	.	.	intergenic	.	rs788091451	2
AAAAC.GTTTT	X	85251358	85251375	3.6	.	.	intergenic	.	rs788832750	2
AC.GT	X	85263535	85263561	13.5	.	.	intergenic	.	rs788312270	4
AATC.GATT	X	85587348	85587371	6	.	.	intergenic	0.052	rs787067504	2
AG.CT	X	85638738	85638749	6	.	.	intergenic	0.388	rs787667382	3
AAAT.ATTT	X	85681234	85681248	3.75	.	.	intergenic	0.271	rs793746266	2
AAAC.GTTT	X	85767148	85767179	8	.	.	intergenic	0.239	rs788303683	2
AC.GT	X	85785075	85785084	5	.	.	intergenic	0.005	.	2
AT.AT	X	85884987	85884998	6	.	.	intergenic	.	rs787903219	2
AG.CT	X	85996454	85996463	5	ENSSSCT00000013641	DIAPH2	intron	0.045	rs788735007	2
AC.GT	X	86072902	86072923	11	ENSSSCT00000013641	DIAPH2	intron	0.103	rs791976114	2
AAAC.GTTT	X	86084158	86084178	5.25	ENSSSCT00000013641	DIAPH2	intron	0.065	.	2
AC.GT	X	86424582	86424594	6.5	ENSSSCT00000028335	.	intron	0.176	rs793010480	2
ACT.AGT	X	86462304	86462325	7.33333	ENSSSCT00000028335	.	intron	0.313	.	2
AGAT.ATCT	X	86497780	86497806	6.75	ENSSSCT00000028335	.	intron	0.356	.	3
AC.GT	X	86678395	86678409	7.5	ENSSSCT00000028335	.	intron	0.267	rs792746525	2
AC.GT	X	86914676	86914688	6.5	.	.	intergenic	0.189	rs790822403	2
AAAC.GTTT	X	86994934	86994948	3.75	.	.	intergenic	0.082	.	2
AT.AT	X	87171089	87171102	7	.	.	intergenic	0.112	.	2
AC.GT	X	87346134	87346145	6	.	.	intergenic	.	rs787904480	3
AAAAC.GTTTT	X	87704040	87704073	6.8	.	.	intergenic	0.046	'58841 rs7902	2
AT.AT	X	87763440	87763458	9.5	.	.	intergenic	0.051	rs789563160	2
AAAC.GTTT	X	87804721	87804734	3.5	.	.	intergenic	-0.081	)05683 rs7935	2
AAAT.ATTT	X	87827744	87827759	4	.	.	intergenic	.	rs793566937	2
AGAT.ATCT	X	87849896	87849914	4.75	.	.	intergenic	.	rs791133929	2
AAAG.CTTT	X	87924537	87924549	3.25	.	.	intergenic	0.212	.	2
AAAT.ATTT	X	88091977	88091997	5.25	.	.	intergenic	0.152	.	3
AAAT.ATTT	X	88178346	88178360	3.75	.	.	intergenic	-0.256	rs786433735	2
AAAC.GTTT	X	88357937	88357952	4	.	.	intergenic	0.197	rs792416875	2
AT.AT	X	88366850	88366860	5.5	.	.	intergenic	.	rs789203542	2
AAG.CTT	X	88417897	88417912	5.33333	.	.	intergenic	0.059	.	2
AT.AT	X	88537354	88537365	6	.	.	intergenic	-0.071	rs791451474	2
AT.AT	X	88546575	88546587	6.5	.	.	intergenic	0.068	'58104 rs7872	2
AT.AT	X	88591540	88591552	6.5	.	.	intergenic	0.057	.	2

AAAC.GTTT	X	88621593	88621608	4	.	.	intergenic	0.12	rs787942322	2
AAT.ATT	X	88626441	88626462	7.33333	.	.	intergenic	0.192	.	2
AC.GT	X	88944350	88944366	8.5	.	.	intergenic	.	rs789789067	2
AAAAT.ATTTT	X	88968854	88968871	3.6	.	.	intergenic	0.365	rs791565972	2
AT.AT	X	89040476	89040487	6	.	.	intergenic	0.221	rs793399678	2
AG.CT	X	89083959	89083979	10.5	.	.	intergenic	-0.04	rs792963307	2
AAC.GTT	X	89159435	89159463	9.66667	.	.	intergenic	-0.062	rs787206193	4
AAAT.ATTT	X	89273313	89273331	4.75	.	.	intergenic	0.044	rs791160654	2
AGAT.ATCT	X	89286220	89286243	6	.	.	intergenic	0.074	rs788742084	2
ATC.GAT	X	89941478	89941494	5.66667	ENSSSCT00000013646	.	intron	0.37	rs792077542	2
AAAC.GTTT	X	90140448	90140474	6.75	.	.	intergenic	0.458	rs787833304	4
AC.GT	X	90210338	90210367	15	.	.	intergenic	1.668	rs792946364	4
AC.GT	X	90321507	90321518	6	.	.	intergenic	0.465	;66178 rs7913	2
AAAT.ATTT	X	90351663	90351683	5.25	.	.	intergenic	.	rs786779991	2
AAAT.ATTT	X	90370413	90370431	4.75	.	.	intergenic	.	rs792093689	2
AAACC.GGTTT	X	90399818	90399837	4	ENSSSCT00000034722	CSTF2	intron	0.238	rs789305427	2
AAACC.GGTTT	X	90399818	90399837	4	ENSSSCT00000034276	CSTF2	intron	0.238	rs789305427	2
AAACC.GGTTT	X	90399818	90399837	4	ENSSSCT00000034376	CSTF2	intron	0.238	rs789305427	2
AAACC.GGTTT	X	90399818	90399837	4	ENSSSCT00000027214	CSTF2	intron	0.238	rs789305427	2
AAACC.GGTTT	X	90399818	90399837	4	ENSSSCT00000013653	CSTF2	intron	0.238	rs789305427	2
AAACC.GGTTT	X	90399818	90399837	4	ENSSSCT00000033718	CSTF2	intron	0.238	rs789305427	2
AAACC.GGTTT	X	90399818	90399837	4	ENSSSCT00000035264	CSTF2	intron	0.238	rs789305427	2
AAAC.GTTT	X	90705802	90705825	6	ENSSSCT00000013658	CENPI	intron	.	.	2
AAAC.GTTT	X	90705802	90705825	6	ENSSSCT00000032805	CENPI	intron	.	.	2
AAAC.GTTT	X	90705802	90705825	6	ENSSSCT00000035785	CENPI	promoter	.	.	2
AAT.ATT	X	90771894	90771912	6.33333	.	.	intergenic	.	rs791382239	2
AAAT.ATTT	X	90774615	90774638	6	.	.	intergenic	0.205	rs789060664	2
AAC.GTT	X	90903174	90903188	5	ENSSSCT00000032924	DRP2	promoter	0.341	rs786762987	2
AAC.GTT	X	90903174	90903188	5	ENSSSCT00000033286	DRP2	promoter	0.341	rs786762987	2
AAC.GTT	X	90903174	90903188	5	ENSSSCT00000034812	DRP2	promoter	0.341	rs786762987	2
AAC.GTT	X	90903174	90903188	5	ENSSSCT00000033320	DRP2	promoter	0.341	rs786762987	2
AAC.GTT	X	90903174	90903188	5	ENSSSCT00000036590	DRP2	promoter	0.341	rs786762987	2
\AAAAC.GTTTT	X	90978438	90978459	3.66667	ENSSSCT00000013660	TAF7L	intron	0.341	rs790382962	2
\AAAAC.GTTTT	X	90978438	90978459	3.66667	ENSSSCT00000032946	TAF7L	intron	0.341	rs790382962	2
AC.GT	X	91039664	91039696	16.5	.	.	intergenic	0.406	rs786583234	2
AATT.AATT	X	91104403	91104428	6.5	.	.	intergenic	.	rs792729921	2
AT.AT	X	91166172	91166183	6	.	.	intergenic	0.146	rs789592035	2
AC.GT	X	91361386	91361408	11.5	.	.	intergenic	.	rs789888369	3
AC.GT	X	91452428	91452447	10	.	.	intergenic	.	.	2
ACAT.ATGT	X	91468249	91468274	6.5	.	.	intergenic	.	351302 rs7921	2
AT.AT	X	91540863	91540874	6	.	.	intergenic	0.217	rs792753400	3
AAT.ATT	X	91670013	91670027	5	.	.	intergenic	0.123	rs792253691	2
AT.AT	X	91690894	91690909	8	.	.	intergenic	.	.	2
AAAAG.CTTTT	X	91820080	91820097	3.6	.	.	intergenic	.	rs790651578	2
AG.CT	X	91834159	91834173	7.5	.	.	intergenic	.	rs789875055	2
AC.GT	X	91842449	91842458	5	.	.	intergenic	.	rs786681627	3
AT.AT	X	91893787	91893798	6	.	.	intergenic	0.087	rs793104776	2
AT.AT	X	91899312	91899322	5.5	.	.	intergenic	-0.024	rs792358222	3
AC.GT	X	91904327	91904337	5.5	.	.	intergenic	.	.	4
AT.AT	X	92163159	92163180	11	.	.	intergenic	0.086	rs787267750	2
AAATT.AATTT	X	92380684	92380700	3.4	.	.	intergenic	0.253	rs787459905	2
AT.AT	X	92691872	92691881	5	.	.	intergenic	0.067	.	3
AT.AT	X	93038534	93038544	5.5	.	.	intergenic	0.119	.	2
AT.AT	X	93138674	93138690	8.5	.	.	intergenic	.	rs788205007	2
AT.AT	X	93307451	93307461	5.5	.	.	intergenic	0.927	rs789926969	3
AT.AT	X	93447199	93447222	12	.	.	intergenic	.	rs792490935	2
AAAT.ATTT	X	93588385	93588399	3.75	.	.	intergenic	0.034	.	2
AC.GT	X	93650191	93650204	7	.	.	intergenic	.	rs791536584	2
AAAAC.GTTTT	X	93665578	93665596	3.8	.	.	intergenic	.	rs792177801	2
AAAAT.ATTTT	X	93746242	93746261	4	.	.	intergenic	0.761	rs787602762	2
AGAGG.CCTCT	X	93862216	93862230	3	.	.	intergenic	.	rs793057812	2
\AAAAG.CTTTT	X	93930709	93930737	4.83333	.	.	intergenic	.	rs787574849	2
AT.AT	X	93973448	93973461	7	.	.	intergenic	.	rs790490206	2
AAAT.ATTT	X	93982840	93982861	5.5	.	.	intergenic	0.021	rs793794887	2
AC.GT	X	94014315	94014327	6.5	.	.	intergenic	-0.132	.	2
AAAAT.ATTTT	X	94174496	94174518	4.6	.	.	intergenic	0.294	rs790791827	2
AAC.GTT	X	94283897	94283910	4.66667	.	.	intergenic	0.09	.	2
AT.AT	X	94285592	94285602	5.5	.	.	intergenic	-1.291	rs788547646	2
AAG.CTT	X	94394594	94394621	9.33333	ENSSSCT00000023372	.	cds	.	rs788365930	2
AAAC.GTTT	X	95122360	95122387	7	.	.	intergenic	.	rs790934583	2
AATT.AATT	X	95123003	95123024	5.5	.	.	intergenic	.	rs793100888	2
AAAAC.GTTTT	X	95209585	95209603	3.8	.	.	intergenic	0.188	rs787109511	2
AT.AT	X	95367674	95367687	7	.	.	intergenic	-0.223	.	2
AGAT.ATCT	X	95669006	95669044	9.75	.	.	intergenic	.	rs788569040	2
AC.GT	X	96397935	96397947	6.5	.	.	intergenic	.	rs790419754	2
AT.AT	X	96478413	96478436	12	.	.	intergenic	.	rs793256035	2
AT.AT	X	96506326	96506336	5.5	.	.	intergenic	-0.226	I65407 rs7927	2
AG.CT	X	96517114	96517138	12.5	.	.	intergenic	.	rs787902160	2
AG.CT	X	96531396	96531407	6	.	.	intergenic	.	rs790360831	2
AAAT.ATTT	X	96562034	96562052	4.75	.	.	intergenic	0.169	rs788339648	2
AC.GT	X	96633657	96633689	16.5	.	.	intergenic	.	rs787575456	3
AT.AT	X	96637405	96637416	6	.	.	intergenic	-0.018	rs788056355	2
AAAC.GTTT	X	96665561	96665584	6	.	.	intergenic	.	.	2
ACT.AGT	X	96722582	96722594	4.33333	.	.	intergenic	.	rs790308767	2

AAAC.GTTT	X	96794662	96794680	4.75	.	.	intergenic	.	rs789225210	2
AC.GT	X	96823495	96823507	6.5	.	.	intergenic	.	rs789560253	2
AT.AT	X	97011874	97011885	6	.	.	intergenic	.	rs793772649	2
AT.AT	X	97131339	97131351	6.5	.	.	intergenic	.	rs786571001	2
AC.GT	X	97383980	97383989	5	.	.	intergenic	.	rs790892118	2
AATC.GATT	X	97437677	97437703	6.75	.	.	intergenic	.	356813 rs7885	2
AAAAC.GTTTT	X	97483740	97483765	5.2	.	.	intergenic	.	rs788715285	2
ACC.GGT	X	97660007	97660020	4.66667	.	.	intergenic	-0.148	.	2
AAAAC.GTTTT	X	97702243	97702262	4	.	.	intergenic	.	.	2
AT.AT	X	97734981	97734992	6	.	.	intergenic	.	rs793022127	2
AATT.AATT	X	97782979	97783000	5.5	.	.	intergenic	.	rs786422323	2
AC.GT	X	98013674	98013689	8	ENSSSCT00000013694	.	promoter	0.185	rs791511199	2
AAAC.GTTT	X	98116852	98116876	6.25	.	.	intergenic	0.139	rs789459856	2
AAAAC.GTTTT	X	98201560	98201585	5.2	.	.	intergenic	-0.145	rs791101592	2
AAC.GTT	X	98217160	98217175	5.33333	.	.	intergenic	-0.081	rs793797775	2
AC.GT	X	98236628	98236638	5.5	.	.	intergenic	0.111	rs789663279	2
AG.CT	X	98319098	98319108	5.5	.	.	intergenic	.	rs789652629	3
AG.CT	X	98373789	98373803	7.5	.	.	intergenic	0.461	rs786459861	2
AAC.GTT	X	98485944	98485957	4.66667	.	.	intergenic	0.229	rs791036096	2
AC.GT	X	98653083	98653111	14.5	ENSSSCT00000033539	PLP1	intron	1.589	.	3
AC.GT	X	98653083	98653111	14.5	ENSSSCT00000035182	PLP1	intron	1.589	.	3
AC.GT	X	98653083	98653111	14.5	ENSSSCT00000035493	PLP1	intron	1.589	.	3
AC.GT	X	98653083	98653111	14.5	ENSSSCT00000034814	PLP1	intron	1.589	.	3
AC.GT	X	98653083	98653111	14.5	ENSSSCT00000013705	PLP1	intron	1.589	.	3
AC.GT	X	98653083	98653111	14.5	ENSSSCT00000033433	PLP1	intron	1.589	.	3
AC.GT	X	99003665	99003676	6	.	.	intergenic	0.189	rs792976538	2
AG.CT	X	99278225	99278237	6.5	.	.	intergenic	0.327	rs786525179	2
AT.AT	X	99554770	99554779	5	ENSSSCT00000034930	IL1RAPL2	intron	.	.	4
AAAAT.ATTTT	X	99620011	99620032	4.4	ENSSSCT00000034930	IL1RAPL2	intron	0.025	rs790231431	2
AC.GT	X	99666562	99666573	6	ENSSSCT00000034930	IL1RAPL2	intron	0.103	rs789982958	2
AC.GT	X	99835071	99835081	5.5	ENSSSCT00000034930	IL1RAPL2	intron	0.215	rs788891399	3
AT.AT	X	99840623	99840645	11.5	ENSSSCT00000034930	IL1RAPL2	intron	.	rs787663403	2
AAATT.AATTT	X	99859168	99859188	4.2	ENSSSCT00000034930	IL1RAPL2	intron	0.071	.	2
AT.AT	X	99883292	99883305	7	ENSSSCT00000034930	IL1RAPL2	intron	0.214	rs790555507	2
AT.AT	X	99900764	99900775	6	ENSSSCT00000034930	IL1RAPL2	intron	0.393	rs791052868	2
AAC.GTT	X	100093204	100093224	7	ENSSSCT00000034930	IL1RAPL2	intron	.	rs793662628	2
AT.AT	X	100493244	100493265	11	ENSSSCT00000034930	IL1RAPL2	intron	0.18	rs789628846	2
AAAAAC.GTTTTT	X	100502265	100502296	5.33333	ENSSSCT00000034930	IL1RAPL2	intron	0.232	.	2
AC.GT	X	100510477	100510487	5.5	ENSSSCT00000034930	IL1RAPL2	intron	0.358	.	4
AG.CT	X	100519919	100519937	9.5	ENSSSCT00000034930	IL1RAPL2	intron	0.18	.	2
AAAAC.GTTTT	X	100753257	100753276	4	ENSSSCT00000034930	IL1RAPL2	intron	0.618	rs786915026	2
AC.GT	X	100885724	100885734	5.5	.	.	intergenic	.	rs787786823	3
AT.AT	X	100908660	100908671	6	ENSSSCT00000035651	NRK	intron	0.19	rs788173567	3
AAAAT.ATTTT	X	100982772	100982789	3.6	ENSSSCT00000013716	.	intron	0.299	rs790355688	2
AC.GT	X	101041997	101042006	5	.	.	intergenic	0.148	rs788474466	2
AT.AT	X	101124118	101124129	6	.	.	intergenic	.	rs789218015	2
AG.CT	X	101191660	101191669	5	.	.	intergenic	0.186	rs793413130	2
AAAC.GTTT	X	101397263	101397281	4.75	ENSSSCT00000013721	.	intron	0.329	rs786890164	2
AAC.GTT	X	101664158	101664171	4.66667	ENSSSCT00000013724	TBC1D8B	intron	.	rs789047898	2
AAC.GTT	X	101664158	101664171	4.66667	ENSSSCT00000035728	TBC1D8B	intron	.	rs789047898	2
AC.GT	X	101933014	101933027	7	ENSSSCT00000013728	RBM41	intron	.	rs787824541	3
AAAC.GTTT	X	102222083	102222105	5.75	.	.	intergenic	0.308	rs789788039	2
AAAC.GTTT	X	102497447	102497484	9.5	ENSSSCT00000013734	FRMPD3	intron	0.28	302214 rs7922	4
AAAG.CTTT	X	102545747	102545767	5.25	.	.	intergenic	0.214	rs792386451	2
AG.CT	X	102766750	102766764	7.5	ENSSSCT00000013739	MID2	intron	0.219	rs789004582	2
AAAC.GTTT	X	102900122	102900139	4.5	.	.	intergenic	0.231	rs789469796	2
AC.GT	X	103654797	103654815	9.5	.	.	intergenic	0.086	.	2
AC.GT	X	103808191	103808205	7.5	.	.	intergenic	0.283	389872 rs7897	2
AT.AT	X	103961009	103961024	8	ENSSSCT00000013749	AMMECR1	intron	0.468	rs791113642	2
AT.AT	X	104660299	104660309	5.5	.	.	intergenic	.	rs787730772	2
AAAC.GTTT	X	104718083	104718106	6	.	.	intergenic	0.282	rs791114678	2
AG.CT	X	105890024	105890035	6	.	.	intergenic	0.155	.	2
AT.AT	X	106594064	106594074	5.5	.	.	intergenic	0.235	rs787139034	2
AG.CT	X	106833430	106833442	6.5	ENSSSCT00000033227	LHFPL1	intron	-0.041	.	3
AG.CT	X	106833430	106833442	6.5	ENSSSCT00000036569	LHFPL1	intron	-0.041	.	3
AG.CT	X	106833430	106833442	6.5	ENSSSCT00000013769	LHFPL1	intron	-0.041	.	3
AG.CT	X	106882727	106882738	6	.	.	intergenic	.	rs790053740	2
AC.GT	X	106894298	106894309	6	.	.	intergenic	.	rs789236363	2
AAC.GTT	X	107146200	107146213	4.66667	.	.	intergenic	.	rs791741145	2
AT.AT	X	107222785	107222796	6	.	.	intergenic	.	rs787145282	2
AG.CT	X	107289908	107289920	6.5	.	.	intergenic	.	.	2
CCAGG.CCTGG	X	107433427	107433446	3.33333	.	.	intergenic	0.023	.	2
AGG.CCT	X	107482105	107482119	5	.	.	intergenic	0.559	rs793428613	2
AT.AT	X	107889912	107889931	10	.	.	intergenic	.	rs790872554	2
AG.CT	X	108735351	108735364	7	ENSSSCT00000035084	HTR2C	intron	0.107	.	4
AG.CT	X	109462755	109462765	5.5	ENSSSCT00000013776	PLS3	intron	0.088	rs790817181	2
GGGGC.GCCCC	X	109464104	109464123	3.33333	ENSSSCT00000013776	PLS3	intron	0.273	rs793030958	2
AC.GT	X	110035250	110035262	6.5	.	.	intergenic	0.038	rs789990293	2
AAAC.GTTT	X	110480278	110480299	5.5	.	.	intergenic	0.192	rs788368961	2
AAT.ATT	X	110551295	110551313	6.33333	.	.	intergenic	0.157	rs792732897	2
AC.GT	X	110555969	110555989	10.5	.	.	intergenic	-0.053	rs788988815	2
AGATAT.ATATCT	X	110826918	110826948	5.16667	.	.	intergenic	0.337	rs793494037	2
AT.AT	X	110878079	110878089	5.5	.	.	intergenic	0.245	.	2
AC.GT	X	111083579	111083588	5	.	.	intergenic	.	.	4

AT.AT	X	111157127	111157139	6.5	.	.	intergenic	0.132	38402 rs7873	2
AT.AT	X	111217765	111217779	7.5	.	.	intergenic	0.137	rs788589024	2
AT.AT	X	111388646	111388664	9.5	.	.	intergenic	.	rs788868843	2
AAAAC.GTTTT	X	111816511	111816529	3.8	.	.	intergenic	0.05	03347 rs7862	3
AAAT.ATTT	X	113122832	113122845	3.5	.	.	intergenic	-0.546	rs792888886	2
AAAT.ATTT	X	113610965	113610981	4.25	ENSSSCT00000013798	6-Sep	intron	-0.335	12620 rs7881	2
AAAT.ATTT	X	113610965	113610981	4.25	ENSSSCT00000035968	6-Sep	intron	-0.335	12620 rs7881	2
AAAT.ATTT	X	113610965	113610981	4.25	ENSSSCT00000035573	6-Sep	intron	-0.335	12620 rs7881	2
AAAT.ATTT	X	113610965	113610981	4.25	ENSSSCT00000035644	6-Sep	intron	-0.335	12620 rs7881	2
AAAT.ATTT	X	113610965	113610981	4.25	ENSSSCT00000034429	6-Sep	intron	-0.335	12620 rs7881	2
AAAT.ATTT	X	113610965	113610981	4.25	ENSSSCT00000034508	6-Sep	intron	-0.335	12620 rs7881	2
AAAT.ATTT	X	113610965	113610981	4.25	ENSSSCT00000033202	6-Sep	intron	-0.335	12620 rs7881	2
AAAT.ATTT	X	113610965	113610981	4.25	ENSSSCT00000036221	6-Sep	intron	-0.335	12620 rs7881	2
AAAT.ATTT	X	113610965	113610981	4.25	ENSSSCT00000034740	6-Sep	intron	-0.335	12620 rs7881	2
AAAC.GTTT	X	113648716	113648745	7.5	ENSSSCT00000013799	NKRF	intron	.	.	2
AAAC.GTTT	X	113648716	113648745	7.5	ENSSSCT00000036344	NKRF	intron	.	.	2
AAAC.GTTT	X	113648716	113648745	7.5	ENSSSCT00000034398	NKRF	promoter	.	.	2
AAAAC.GTTTT	X	113720695	113720713	3.8	.	.	intergenic	.	rs792382169	2
AAAT.ATTT	X	114461047	114461062	4	ENSSSCT00000013815	.	intron	-0.063	28734 rs7887	2
AAAT.ATTT	X	114461047	114461062	4	ENSSSCT00000035722	DOCK11	intron	-0.063	28734 rs7887	2
AAAAC.GTTTT	X	114485716	114485738	3.83333	ENSSSCT00000013815	.	cds	0.434	rs792712529	2
AAAAC.GTTTT	X	114485716	114485738	3.83333	ENSSSCT00000013815	.	intron	0.434	rs792712529	2
AAAAC.GTTTT	X	114485716	114485738	3.83333	ENSSSCT00000035005	DOCK11	intron	0.434	rs792712529	2
AAAAC.GTTTT	X	114485716	114485738	3.83333	ENSSSCT00000035722	DOCK11	intron	0.434	rs792712529	2
AT.AT	X	114498608	114498618	5.5	ENSSSCT00000013815	.	intron	0.145	.	2
AT.AT	X	114498608	114498618	5.5	ENSSSCT00000035722	DOCK11	intron	0.145	.	2
AAAC.GTTT	X	114547891	114547918	7	ENSSSCT00000013815	.	intron	0.297	rs792302123	2
AAAC.GTTT	X	114547891	114547918	7	ENSSSCT00000035722	DOCK11	intron	0.297	rs792302123	2
ATCC.GGAT	X	115024835	115024864	7.5	.	.	intergenic	0.068	rs787283372	2
AT.AT	X	115718167	115718177	5.5	ENSSSCT00000013819	GRIA3	intron	.	rs789414883	2
AT.AT	X	115718167	115718177	5.5	ENSSSCT00000032933	GRIA3	intron	.	rs789414883	2
AT.AT	X	115718167	115718177	5.5	ENSSSCT00000036560	GRIA3	intron	.	rs789414883	2
AAAC.GTTT	X	116374435	116374455	5.25	ENSSSCT00000013823	STAG2	intron	0.229	rs792581610	2
AAG.CTT	X	116489113	116489129	5.66667	.	.	intergenic	0.359	rs787664252	2
AG.CT	X	116534044	116534056	6.5	.	.	intergenic	0.161	rs790791408	2
AAC.GTT	X	116723274	116723291	6	.	.	intergenic	1.23	rs786593162	2
AG.CT	X	117092101	117092117	8.5	ENSSSCT00000033370	TENM1	intron	0.084	.	4
AC.GT	X	117116792	117116809	9	ENSSSCT00000033370	TENM1	intron	1.655	.	3
AAAC.GTTT	X	117258195	117258222	7	ENSSSCT00000033370	TENM1	intron	.	.	2
AAAC.GTTT	X	117258195	117258222	7	ENSSSCT00000030109	U2	promoter	.	.	2
AT.AT	X	117579380	117579389	5	.	.	intergenic	0.219	rs792056007	2
AT.AT	X	117581945	117581957	6.5	.	.	intergenic	0.282	.	2
AAT.ATT	X	117611033	117611054	7.33333	.	.	intergenic	0.513	rs791836397	2
AAAAC.GTTTT	X	117687477	117687505	5.8	.	.	intergenic	0.437	rs789538764	2
AAT.ATT	X	117719241	117719255	5	.	.	intergenic	0.496	rs789519529	2
AC.GT	X	118109367	118109378	6	.	.	intergenic	0.063	75609 rs6920	4
AAAAC.GTTTT	X	118163715	118163743	5.8	.	.	intergenic	0.477	rs792046128	2
AT.AT	X	118662866	118662878	6.5	.	.	intergenic	0.218	rs788554439	2
AC.GT	X	118702361	118702371	5.5	.	.	intergenic	0.006	.	4
AG.CT	X	118911571	118911603	16.5	.	.	intergenic	0.083	rs789546047	2
AAC.GTT	X	118914116	118914135	6.66667	.	.	intergenic	0.171	rs793621895	2
AC.GT	X	118968737	118968748	6	.	.	intergenic	-0.067	rs792468071	2
AG.CT	X	119390409	119390419	5.5	.	.	intergenic	-0.144	.	2
AT.AT	X	119473622	119473637	8	.	.	intergenic	.	rs792127392	2
AT.AT	X	119652451	119652462	6	.	.	intergenic	.	rs791754764	2
AG.CT	X	120886661	120886670	5	.	.	intergenic	0.469	rs790407818	2
AAC.GTT	X	120939955	120939970	5.33333	.	.	intergenic	.	rs793605184	2
AAAAC.GTTTT	X	121311469	121311483	3	.	.	intergenic	0.353	rs793469258	2
AG.CT	X	121324586	121324596	5.5	.	.	intergenic	0.582	rs786227941	3
AAAT.ATTT	X	121935312	121935325	3.5	ENSSSCT00000013831	OCRL	intron	0.171	75467 rs7905	2
AAAT.ATTT	X	121935312	121935325	3.5	ENSSSCT00000035468	OCRL	intron	0.171	75467 rs7905	2
AAAT.ATTT	X	121935312	121935325	3.5	ENSSSCT00000036143	OCRL	intron	0.171	75467 rs7905	2
AAAC.GTTT	X	121998310	121998328	4.75	.	.	intergenic	0.303	rs792123574	2
AAAC.GTTT	X	122133590	122133606	4.25	.	.	intergenic	0.087	rs790202669	2
AAAC.GTTT	X	122217874	122217892	4.75	.	.	intergenic	0.097	rs786317082	2
AAAT.ATTT	X	122286230	122286252	5.75	.	.	intergenic	.	rs787625612	2
AAAC.GTTT	X	122561557	122561573	4.25	ENSSSCT00000013840	ZNF280C	intron	0.18	rs790173553	2
AAAC.GTTT	X	122561557	122561573	4.25	ENSSSCT00000033263	ZNF280C	intron	0.18	rs790173553	2
AG.CT	X	122940691	122940705	7.5	ENSSSCT00000034092	ENOX2	intron	-1.025	.	2
AG.CT	X	122940691	122940705	7.5	ENSSSCT00000026646	ENOX2	intron	-1.025	.	2
AG.CT	X	122940691	122940705	7.5	ENSSSCT00000035630	ENOX2	intron	-1.025	.	2
AG.CT	X	123652038	123652048	5.5	.	.	intergenic	0.262	rs790137626	2
AC.GT	X	123868435	123868445	5.5	.	.	intergenic	0.108	rs698983496	3
AAC.GTT	X	124018096	124018111	5.33333	.	.	intergenic	.	23309 rs7926	2
AG.CT	X	124033403	124033413	5.5	.	.	intergenic	.	.	4
AAAC.GTTT	X	124033820	124033834	3.75	.	.	intergenic	.	rs786808447	2
AAC.GTT	X	124134751	124134763	4.33333	.	.	intergenic	0.056	rs792426782	3
AGGGC.GCCCT	X	124407155	124407178	4.8	.	.	intergenic	0.159	rs793177907	2
AC.GT	X	124413324	124413341	9	.	.	intergenic	.	.	2
AAC.GTT	X	124466449	124466469	7	.	.	intergenic	.	26606 rs7896	2
AT.AT	X	124474936	124474945	5	.	.	intergenic	0.237	.	2
AC.GT	X	124558135	124558145	5.5	.	.	intergenic	.	rs789920078	2
AAC.GTT	X	124998276	124998289	4.66667	.	.	intergenic	0.01	rs787409280	2
ATTAG.CTAAT	X	125201122	125201143	3.66667	.	.	intergenic	0.034	rs792079171	2

AC.GT	X	125308237	125308249	6.5	.	.	intergenic	0.031	rs789089510	2
AC.GT	X	125404334	125404346	6.5	.	.	intergenic	1.11	rs792394828	2
AG.CT	X	125429690	125429700	5.5	.	.	intergenic	0.05	rs792101626	2
AC.GT	X	125829394	125829403	5	ENSSSCT00000013862	GPC3	intron	0.746	rs787985736	2
AAAT.ATTT	X	126099425	126099440	4	.	.	intergenic	0.5	rs790147175	2
AC.GT	X	126130088	126130106	9.5	.	.	intergenic	0.244	.	2
AAAC.GTTT	X	126302697	126302714	4.5	.	.	intergenic	.	rs792630423	2
AAAC.GTTT	X	126508867	126508895	7.25	ENSSSCT00000034119	FAM122C	intron	0.056	rs793343825	2
AAAC.GTTT	X	126508867	126508895	7.25	ENSSSCT00000033658	FAM122C	intron	0.056	rs793343825	2
AAAC.GTTT	X	126508867	126508895	7.25	ENSSSCT00000034247	FAM122C	intron	0.056	rs793343825	2
AAAC.GTTT	X	126508867	126508895	7.25	ENSSSCT00000035919	FAM122C	intron	0.056	rs793343825	2
AAAC.GTTT	X	126508867	126508895	7.25	ENSSSCT00000034857	FAM122C	intron	0.056	rs793343825	2
AAAC.GTTT	X	126508867	126508895	7.25	ENSSSCT00000034621	FAM122C	intron	0.056	rs793343825	2
AG.CT	X	126634361	126634382	11	.	.	intergenic	0.032	346866 rs7929	4
AC.GT	X	126988477	126988486	5	ENSSSCT00000033983	DDX26B	intron	0.022	rs786669414	3
AC.GT	X	126988477	126988486	5	ENSSSCT00000033063	DDX26B	intron	0.022	rs786669414	3
AC.GT	X	126988477	126988486	5	ENSSSCT00000013878	DDX26B	intron	0.022	rs786669414	3
AC.GT	X	126988477	126988486	5	ENSSSCT00000033505	DDX26B	promoter	0.022	rs786669414	3
AG.CT	X	127621880	127621890	5.5	.	.	intergenic	0.939	rs701993104	2
AAC.GTT	X	128277283	128277295	4.33333	.	.	intergenic	.	555725 rs7891	2
AG.CT	X	128342746	128342763	9	.	.	intergenic	.	rs789573567	3
AT.AT	X	128408989	128408999	5.5	.	.	intergenic	.	rs789833648	2
AAAC.GTTT	X	128476471	128476489	4.75	.	.	intergenic	0.279	rs790344260	2
AAAAC.GTTTT	X	128591545	128591568	4.8	.	.	intergenic	.	rs790788706	2
AC.GT	X	128665953	128665987	17.5	.	.	intergenic	0.255	.	2
AT.AT	X	128717118	128717129	6	.	.	intergenic	.	rs788806337	2
AC.GT	X	129507081	129507107	13.5	.	.	intergenic	.	rs788354141	5
AG.CT	X	129652604	129652614	5.5	ENSSSCT00000029012	FGF13	intron	0.195	rs788796963	2
AG.CT	X	129652604	129652614	5.5	ENSSSCT00000021518	ssc-mir-504	promoter	0.195	rs788796963	2
AAC.GTT	X	129762391	129762409	6.33333	.	.	intergenic	-0.004	rs792550389	2
AAAC.GTTT	X	129863552	129863578	6.75	.	.	intergenic	-0.314	rs789351317	2
AT.AT	X	129975811	129975822	6	.	.	intergenic	0.134	596972 rs7868	2
ATCC.GGAT	X	130056644	130056665	5.5	.	.	intergenic	.	rs790413356	2
AT.AT	X	130472870	130472879	5	ENSSSCT00000013898	MCF2	intron	0.207	rs792392851	2
AG.CT	X	130713960	130713971	6	ENSSSCT00000013899	ATP11C	intron	0.184	rs791997431	2
AAAAC.GTTTT	X	130738543	130738562	4	ENSSSCT00000013899	ATP11C	intron	0.191	307460 rs7923	2
ACAT.ATGT	X	130912916	130912934	4.75	.	.	intergenic	0.392	rs789742404	2
AAAAT.ATTTT	X	130924703	130924719	3.4	.	.	intergenic	0.167	rs792711403	2
AAAT.ATTT	X	131047687	131047709	5.75	.	.	intergenic	0.547	rs790750720	2
AAC.GTT	X	131276456	131276471	5.33333	.	.	intergenic	.	rs788948555	2
AT.AT	X	131347239	131347276	19	.	.	intergenic	0.19	362047 rs7870	2
AC.GT	X	131483414	131483425	6	ENSSSCT00000034891	1242-176I1!	promoter	0.115	480564 rs7927	2
AAT.ATT	X	131541219	131541232	4.66667	.	.	intergenic	0.623	rs788520075	3
AAAC.GTTT	X	132049655	132049679	6.25	.	.	intergenic	0.083	.	2
AT.AT	X	132323268	132323280	6.5	.	.	intergenic	0.01	rs788774980	2
ACC.GGT	X	132407414	132407428	5	.	.	intergenic	.	rs792678511	2
AAAC.GTTT	X	132431782	132431797	4	.	.	intergenic	0.056	300381 rs7929	4
AC.GT	X	132537838	132537855	9	.	.	intergenic	0.088	.	2
AAC.GTT	X	132808238	132808255	6	.	.	intergenic	.	.	2
AC.GT	X	132885164	132885176	6.5	.	.	intergenic	0.009	rs790421315	3
AG.CT	X	132982207	132982217	5.5	.	.	intergenic	.	.	2
AC.GT	X	133160879	133160898	10	.	.	intergenic	.	.	2
AAGG.CCTT	X	133750323	133750344	5.5	ENSSSCT00000036202	1242-522I2	intron	-0.179	rs792572226	2
AAAG.CTTT	X	133903733	133903746	3.5	ENSSSCT00000036202	1242-522I2	intron	0.005	rs787221756	2
AC.GT	X	134242320	134242330	5.5	ENSSSCT00000036202	1242-522I2	intron	0.003	rs789134361	3
AT.AT	X	134244773	134244782	5	ENSSSCT00000036202	1242-522I2	intron	0.275	rs786890758	3
AC.GT	X	134476434	134476446	6.5	.	.	intergenic	-0.413	.	2
AAC.GTT	X	134883691	134883704	4.66667	.	.	intergenic	-0.151	.	2
AC.GT	X	135281010	135281024	7.5	.	.	intergenic	.	rs706115978	2
AAAC.GTTT	X	135410416	135410434	4.75	.	.	intergenic	.	rs790308569	2
AT.AT	X	135461249	135461260	6	.	.	intergenic	.	rs788699685	2
AAT.ATT	X	135461707	135461723	5.66667	.	.	intergenic	.	rs786560260	2
AAAT.ATTT	X	135686088	135686101	3.5	.	.	intergenic	.	rs787431963	2
AG.CT	X	135867673	135867691	9.5	.	.	intergenic	-0.004	rs786728112	2
AC.GT	X	136010747	136010773	13.5	.	.	intergenic	.	rs790614937	3
AT.AT	X	136027366	136027379	7	.	.	intergenic	.	.	2
AG.CT	X	136045151	136045162	6	.	.	intergenic	0.042	.	2
AC.GT	X	136196560	136196587	14	.	.	intergenic	-0.195	rs792657051	2
AG.CT	X	136210862	136210871	5	.	.	intergenic	0.148	.	2
AAAG.CTTT	X	136387615	136387645	7.75	.	.	intergenic	0.076	rs787025725	2
AC.GT	X	136421407	136421419	6.5	.	.	intergenic	0.101	rs790521711	2
AT.AT	X	136631517	136631534	9	.	.	intergenic	.	rs792199874	2
AAAT.ATTT	X	136841904	136841925	5.5	.	.	intergenic	0.274	rs792133606	2
AAAG.CTTT	X	136856894	136856913	5	.	.	intergenic	.	rs786662800	2
AT.AT	X	137102925	137102936	6	.	.	intergenic	.	rs786215988	3
AAAT.ATTT	X	137484926	137484938	3.25	.	.	intergenic	0.098	rs793724611	2
AGGG.CCCT	X	137719051	137719063	3.25	.	.	intergenic	-0.013	.	2
AAAAC.GTTTT	X	137842009	137842032	4.8	.	.	intergenic	0.23	rs786841233	3
AACC.GGTT	X	137994785	137994813	7.25	.	.	intergenic	0.084	rs792402999	3
AG.CT	X	138148198	138148213	8	.	.	intergenic	0.083	rs787828470	2
AAC.GTT	X	138236855	138236883	9.66667	.	.	intergenic	0.309	rs790347507	2
AAG.CTT	X	138442987	138443001	5	ENSSSCT00000013917	AFF2	intron	0.462	rs786421766	2
AC.GT	X	138474113	138474123	5.5	ENSSSCT00000013917	AFF2	intron	0.197	rs790316203	2
AG.CT	X	138576943	138576952	5	ENSSSCT00000013917	AFF2	intron	0.31	rs790137645	2

AATG.CATT	X	138785931	138785962	8	.	.	intergenic	0.047	376563 rs7930	2
AC.GT	X	139627871	139627881	5.5	.	.	intergenic	-0.185	rs788333674	2
AGAT.ATCT	X	140346886	140346901	4	.	.	intergenic	0.184	rs787934905	2
ACAT.ATGT	X	140382144	140382166	5.75	.	.	intergenic	0.219	905994 rs7870	2
AAC.GTT	X	140543095	140543114	6.66667	.	.	intergenic	.	rs788245836	2
AT.AT	X	140595675	140595685	5.5	.	.	intergenic	0.099	rs793819929	2
AAAT.ATTT	X	140808537	140808563	6.75	.	.	intergenic	0.278	rs790901175	2
AC.GT	X	141373574	141373588	7.5	ENSSSCT00000034124	CETN2	intron	1.202	rs788472291	2
AC.GT	X	141373574	141373588	7.5	ENSSSCT00000032639	CETN2	intron	1.202	rs788472291	2
AC.GT	X	141373574	141373588	7.5	ENSSSCT00000013947	CETN2	intron	1.202	rs788472291	2
AC.GT	X	141373574	141373588	7.5	ENSSSCT00000033471	CETN2	intron	1.202	rs788472291	2
AT.AT	X	141407326	141407342	8.5	.	.	intergenic	-0.039	rs793491580	4
AC.GT	X	142191018	142191030	6.5	.	.	intergenic	.	rs791729025	3
AAAAC.GTTTT	X	143222896	143222916	4.2	.	.	intergenic	-0.098	rs790268045	2
AATT.AATT	X	143853116	143853129	3.5	.	.	intergenic	.	.	2
AC.GT	X	143857650	143857662	6.5	.	.	intergenic	.	383018 rs7911	2
AAG.CTT	X	144153854	144153865	4	ENSSSCT00000014028	PRKX	intron	.	.	5
ATCC.GGAT	X	144159488	144159506	4.75	ENSSSCT00000014028	PRKX	intron	.	.	2

Supplementary Figure S1.

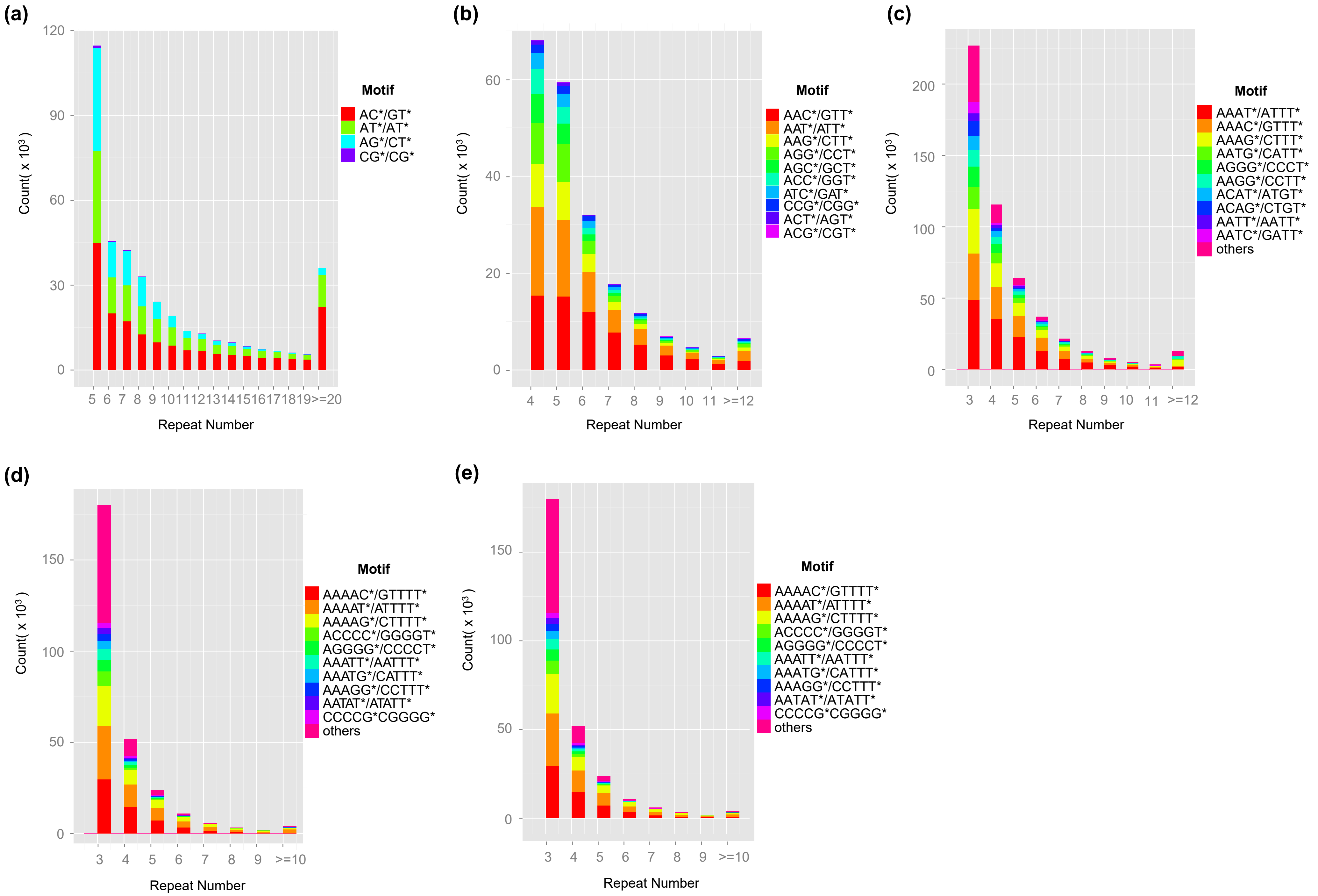

Frequency distribution of overall SSRs in reference genome. (a) Dinucleotide, (b) Trinucleotide, (c) Tetranucleotide, (d) Pentanucleotide, (e) Hexanucleotide. The count distribution of different SSRs is classified according to the repeat number of the motif.

Supplementary Figure S2.

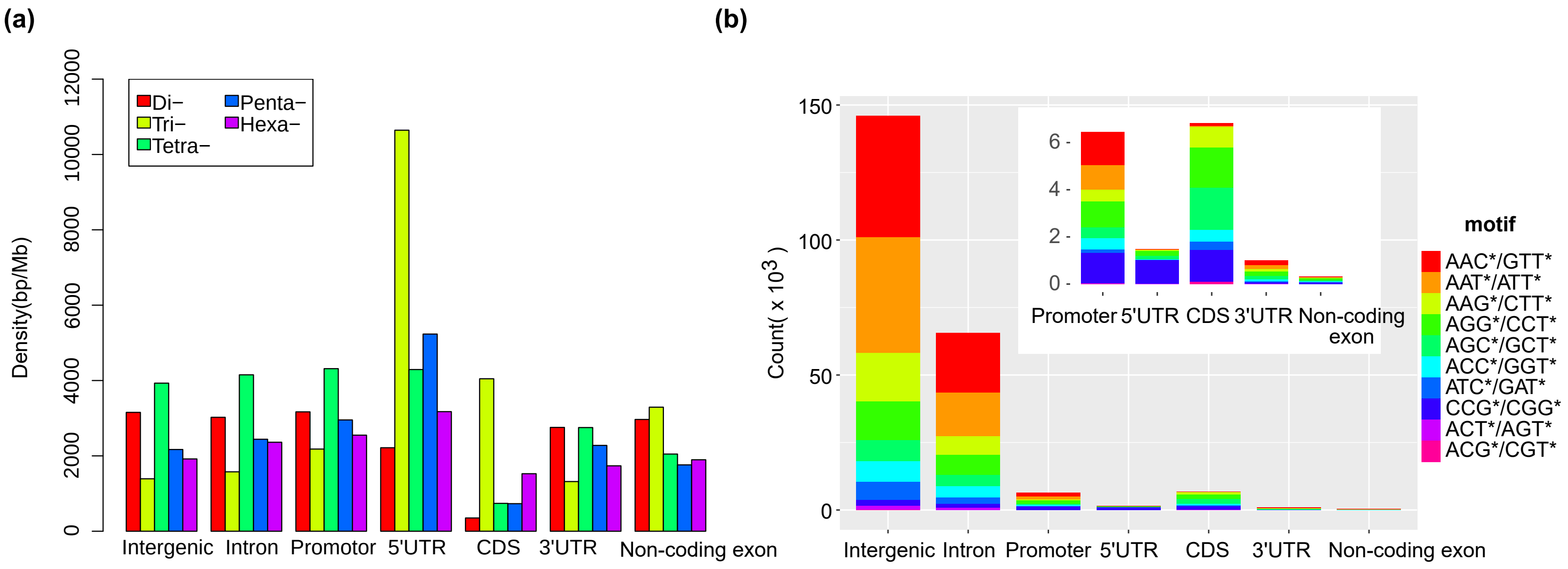

Overall SSRs distribution in different genomic region. (a) The distribution of each kind of SSR in different genomic regions. (b) The distribution of trinucleotide SSRs in different genomic regions.

Supplementary Figure S3.

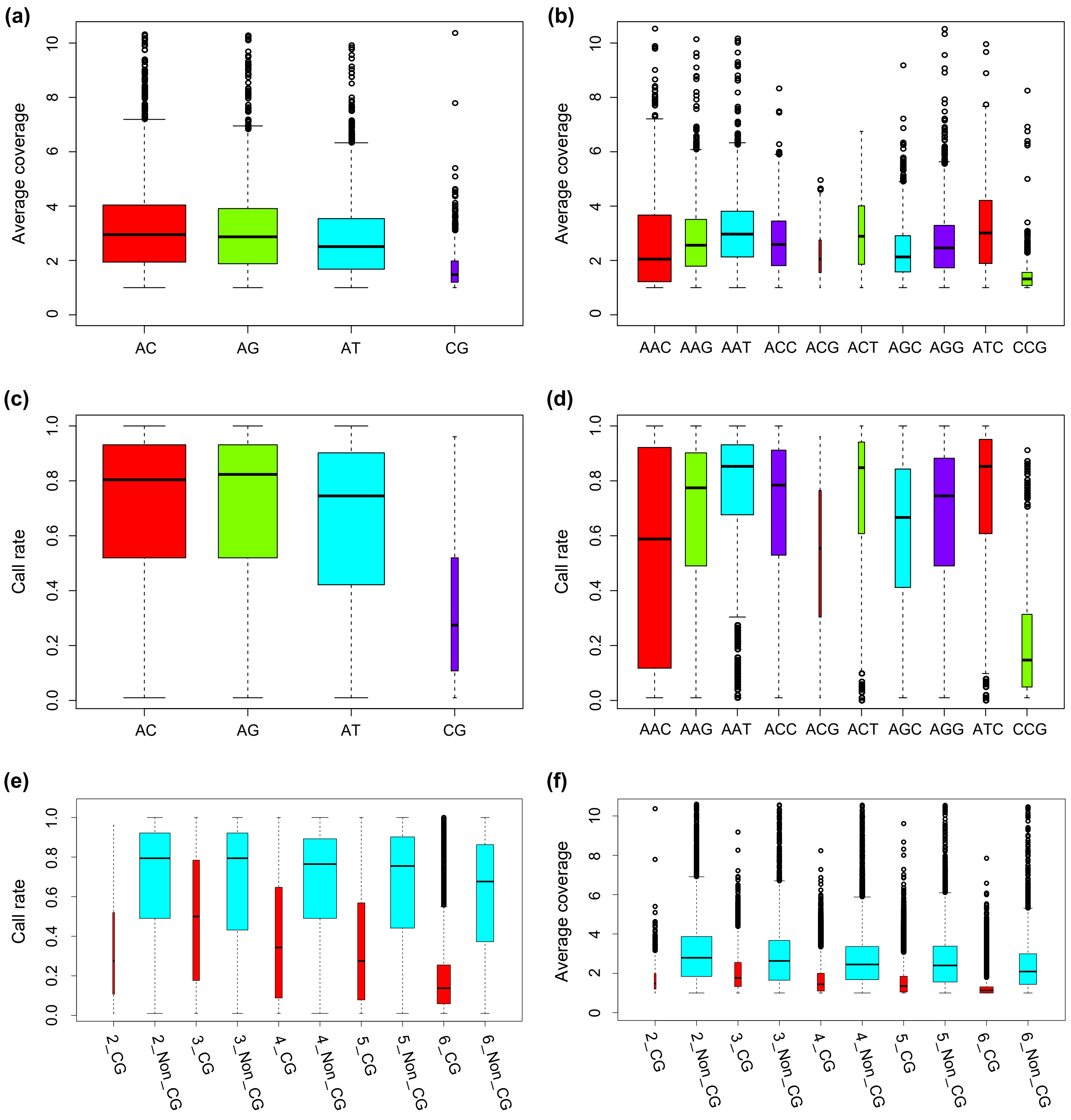

Motif type effect to Call rate and sequence coverage. (a) The average coverage of different motifs in dinucleotide SSRs. (b) The average coverage of different motifs in trinucleotide SSRs. (c) The call rate of different motifs in dinucleotide SSRs. (d) The call rate of different motifs in trinucleotide SSRs. (e) Comparison of the call rate between CG-containing motifs and non-CG containing motifs. (f) Comparison of the average coverage between CG-containing motifs and non-CG containing motifs. The numbers in (e) and (f) represent different SSR type.

Supplementary Figure S4.

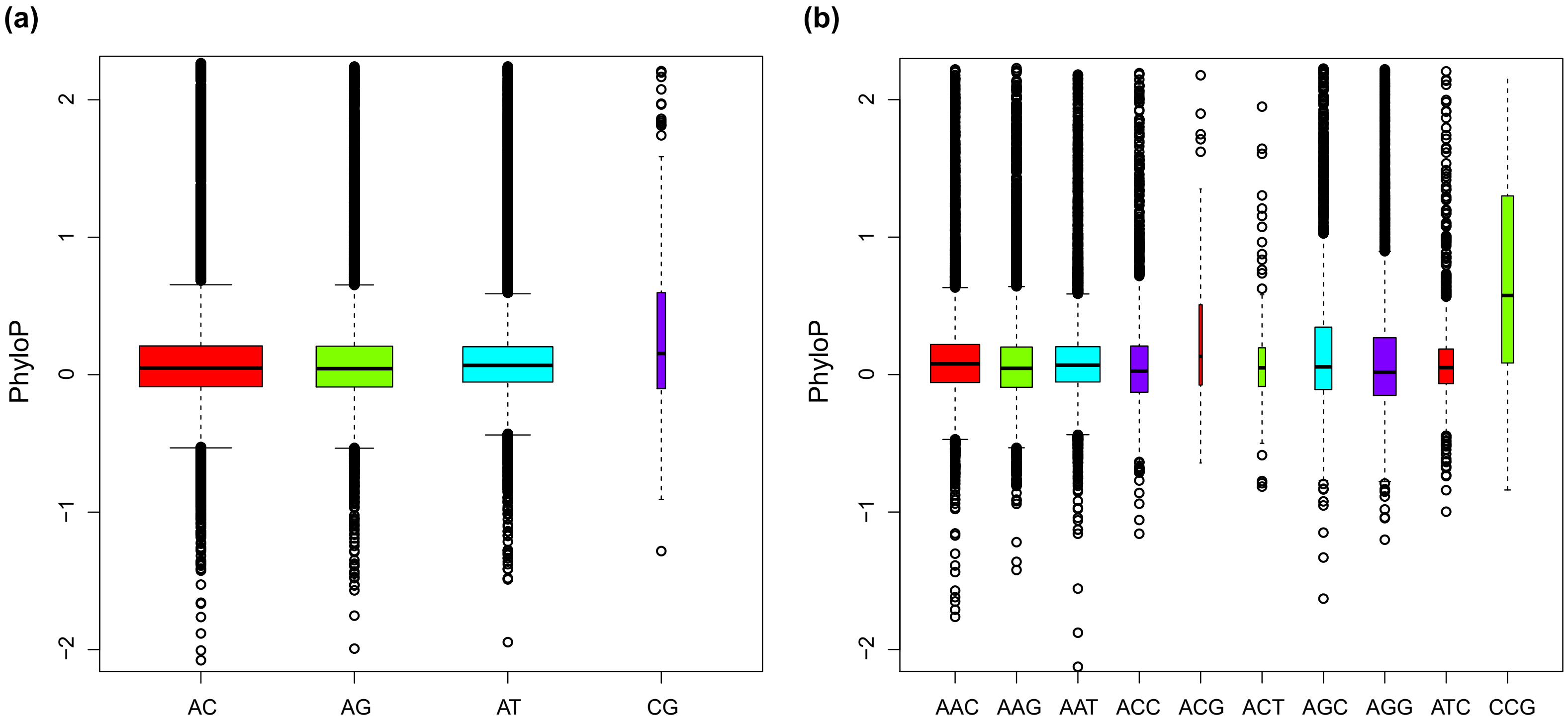

The PhyloP score comparisons among different motifs. (a) Comparison of the PhyloP score in all identified dinucleotide polymorphic SSRs. (b) Comparison of the PhyloP score in all identified trinucleotide polymorphic SSRs.
